# Cobaltaphotocatalysed intramolecular hydroarylation of alkenes for the construction of tetrahydroquinolines

**DOI:** 10.1039/d6sc05922h

**Published:** 2026-08-20

**Authors:** Balakumar Emayavaramban, David G. Groves, Reece H. Hoogesteger, Tatjana Jerkovic, Loïc R. E. Pantaine, Craig P. Johnston

**Affiliations:** a EaStCHEM, School of Chemistry, University of St Andrews St Andrews, Fife Scotland KY16 9ST UK cpj3@st-andrews.ac.uk; b Process and Analytical Chemistry, Pharmaron UK Ltd Hoddesdon EN11 9FH UK

## Abstract

1,2,3,4-Tetrahydroquinolines are a class of heterocycles that commonly feature in both biologically active natural products and pharmaceuticals. However, their synthesis is often dominated by either high-pressure hydrogenation reactions or acid-mediated Friedel–Crafts-like hydroarylations. Recently, cobalt-salen catalysed cycloisomerisation reactions have been reported as a promising method to construct heterocycles, but there are limited examples of tetrahydroquinolines. Moreover, these protocols often rely on stoichiometric additives and proceed through radical pathways in which catalyst control is difficult to exert. Herein, we report a photochemical cobalt-salen catalysed intramolecular hydroarylation for the synthesis of C4-functionalised tetrahydroquinolines. The mild reaction conditions tolerate a wide array of functional groups, enabling the preparation of a diverse catalogue of tetrahydroquinoline derivatives. This protocol was readily adapted for scale-up by employing a photoflow system, a first for a cobalt-salen metal-hydride hydrogen atom transfer process. Collectively, experimental observations are consistent with a radical-polar crossover pathway involving formation of a free carbocation or a putative alkylcobalt(iv) carbocation surrogate.

## Abstract 2

Hydroarylation of cinnamylanilines under cobalt and photoredox catalysis provides diverse C4-functionalised tetrahydroquinolines in batch and flow. Mechanistic studies are consistent with a radical-polar crossover event before cyclisation.

## Graphical abstract

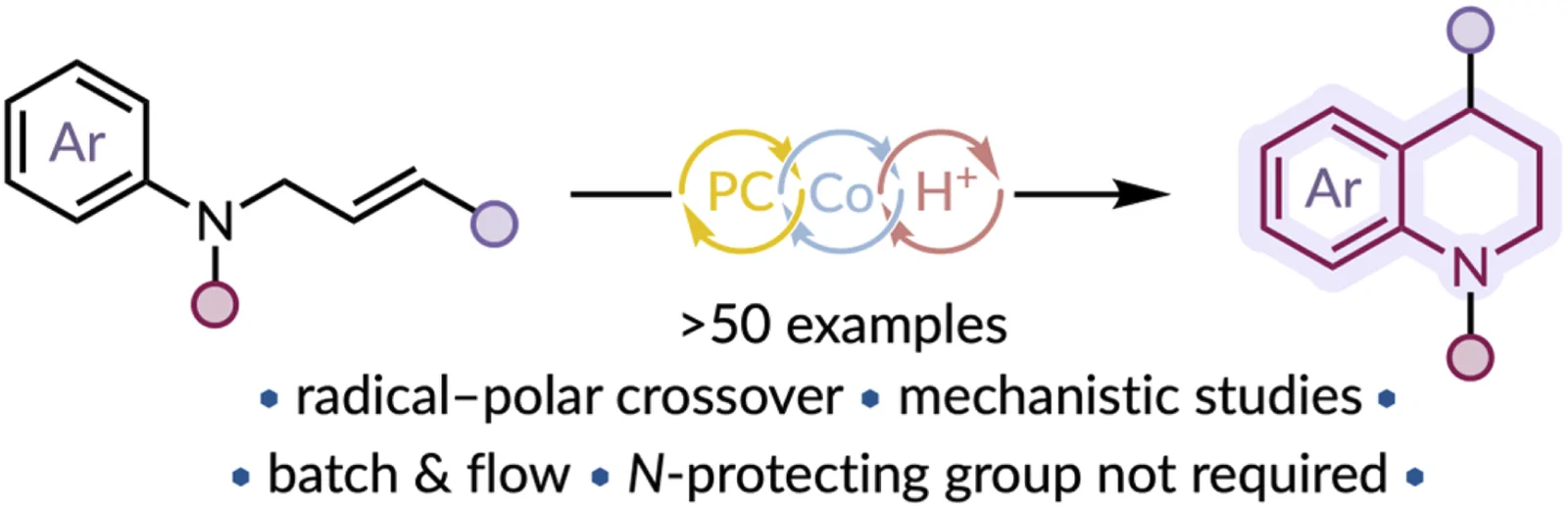

## Introduction

The 1,2,3,4-tetrahydroquinoline (THQ) architecture is a privileged structural motif found in numerous natural products, synthetic dyes, agrochemicals, and pharmaceutically relevant compounds (Fig. 1).^1–3^ These structures have attracted considerable interest due to their wide-ranging therapeutic potential, including treatments for cancer, HIV, and Parkinson's disease.^4,5^ Therefore, new synthetic strategies that enable tetrahydroquinoline formation from readily accessible starting materials, while controlling chemoselectivity and regioselectivity, remain highly desirable.

**Fig. 1:**
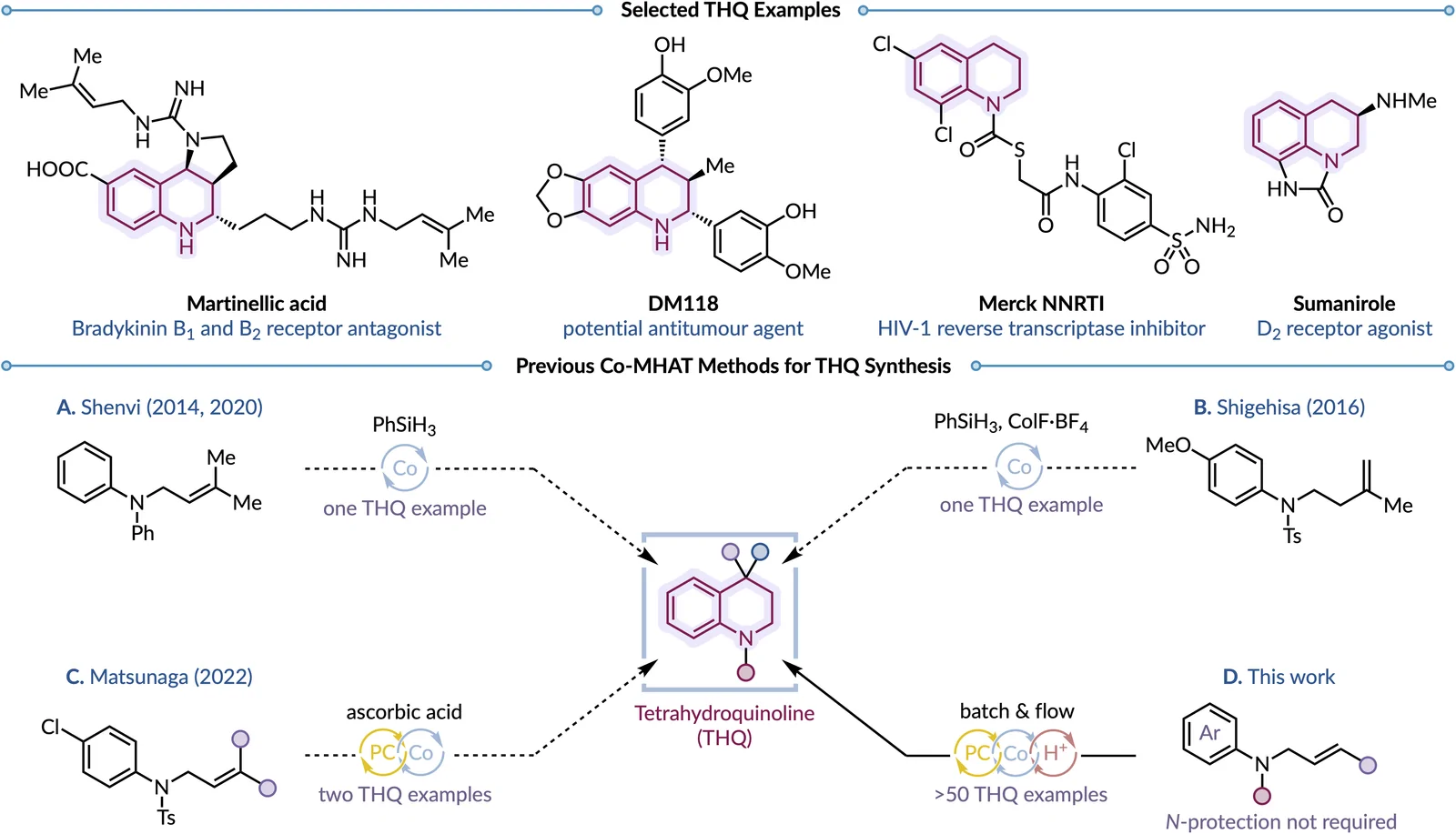
Bioactive tetrahydroquinoline-containing small molecules (top) and cobalt-catalysed approaches to THQ synthesis (bottom).

The intramolecular cyclisation of appropriately functionalised arylamines is a common approach used for the synthesis of THQs. Such strategies invoke a multitude of distinct bond-forming processes to construct these heterocyclic systems, including the intramolecular hydroarylation of pendant alkenes tethered to anilines.^3^ This cycloisomerisation disconnection represents a particularly efficient approach to THQ scaffolds. However, classical implementations of this strategy typically rely on Friedel–Crafts chemistry, which uses strong Brønsted acids and elevated temperatures, resulting in poor functional group tolerance. In recent years, milder cycloisomerisation strategies catalysed by earth-abundant transition metals, such as iron and manganese, have been developed more broadly.^6^ In particular, cobalt catalysis has emerged as a powerful platform for the hydrofunctionalisation of alkenes, including hydroarylations.^7–15^

Seminal studies by Drago and Mukaiyama established that cobalt complexes could mediate the aerobic oxidation of alkenes under comparatively mild conditions, providing important early examples of cobalt-catalysed alkene hydrofunctionalisation.^16–18^ Although these reactions were not originally formulated in terms of metal-hydride hydrogen atom transfer (MHAT), they have since been reinterpreted within an MHAT framework, wherein carbon-centred radical intermediates are generated with Markovnikov selectivity. Later, Shenvi and co-workers exploited this mechanistic understanding for the cobalt-salen catalysed isomerisation and cycloisomerisation of alkenes to construct heterocycles (Fig. 1A).^19,20^

An alternative intramolecular hydroarylation protocol was developed by the Shigehisa group, using a Co(ii)-salen complex and a stoichiometric quantity of both an oxidant (*N*-fluoropyridinium salt) and an organosilane (Fig. 1B).^8^ This transformation begins in a similar manner to Shenvi's protocol: the generation of an alkyl radical *via* MHAT. However, it was proposed that under these oxidative conditions, a carbocation-like intermediate was produced, resulting in Friedel–Crafts-like reactivity. This strategy has been exploited for the construction of carbocycles,^8,21^ N-,^22–26^ O-,^26–30^ and S-containing heterocycles *via* cycloisomerisation,^31,32^ as well as a range of intermolecular alkene hydrofunctionalisations.^33–45^ Despite the success of these oxidative cobalt-catalysed alkene functionalisations, their use of stoichiometric quantities of costly oxidants (*N*-fluoropyridinium salts)^46^ or potentially hazardous peroxides raises concerns over the sustainability and safety of this approach.^47^

In 2021, Matsunaga and co-workers developed a photochemical procedure to access a transient cobalt(iii) hydride without the addition of a silane or a stoichiometric oxidant. Instead, excess ascorbic acid serves as a terminal reductant and a proton source. Initially, this protocol was applied to the hydrogenation of alkenes, but the methodology was subsequently extended to cycloisomerisation for the synthesis of heterocycles, with a particular focus on 3,4-dihydroisoquinolinones (Fig. 1C).^10,48^ In this manifold, C–C bond formation is proposed to proceed through intramolecular radical addition to the arene, followed by rearomatisation *via* Co(ii)-promoted hydrogen atom transfer.

In a related advance, Ohmiya and Nagao developed a triple catalysis system in which photoredox catalysis and a substoichiometric Brønsted acid modulate the oxidation state of cobalt.^49^ This strategy enabled Markovnikov hydroalkoxylation of unactivated alkenes *via* an MHAT-initiated radical-polar crossover pathway, indicating that organocobalt(iv) carbocation-like reactivity could be translated into a photochemical manifold without silanes or stoichiometric oxidants.^28,50^ Soon after, the Carreira group broadened this concept to construct assorted saturated heterocycles predominantly through intramolecular trapping with heteroatom nucleophiles.^11,51^ More recently, Zhang and co-workers developed a cascade process for the construction of chromanes *via* hydroalkoxylation of a 1,3-diene with a phenol, which subsequently undergoes hydroarylation.^52^ Despite these advancements and several other cobalt-photochemical approaches,^53–61^ there have been no efficient methods developed for the synthesis of tetrahydroquinoline moieties.

Herein, we report the first dedicated cobaltaphotocatalysed protocol for the synthesis of tetrahydroquinolines (Fig. 1D). The economical dye *peri*-xanthenoxanthene (PXX) was used as an organophotocatalyst given its desirable redox properties. Despite being commercially available and conveniently synthesised from BINOL, PXX is comparatively underexplored within photoredox catalysis.^62^ This is, to the best of our knowledge, the first report demonstrating its viability for cobalt-based metallaphotoredox catalysis. Furthermore, we demonstrate the first continuous-flow photochemical scale-up of a cobalt-salen MHAT-type transformation, highlighting the potential of this platform for large-scale synthesis.

## Results and discussion

### Reaction development

The reaction optimisation was performed with *N*-benzyl-*N*-cinnamylaniline 1aa as the model substrate to investigate the formation of tetrahydroquinolines *via* a photocatalysed intramolecular hydroarylation. Employing Co(ii)-salophen [Co-1] (1 mol%), organic photocatalyst PC-1 PXX (2 mol%), with lutidine-based Brønsted acid Lut·HOTf (10 mol%) in PhCl (0.20 M) under blue light (450 nm) irradiation for 16 h, with the internal reaction temperature reaching 32 °C, produced the desired tetrahydroquinoline 2aa in near quantitative yield (Table 1, entry 1). To determine the robustness of the developed protocol, several reaction parameters and reagents were varied. Alternative solvents such as dichloromethane and acetone gave minor reductions in yield in contrast to tetrahydrofuran, which performed poorly (entries 2–4). Evaluating other highly reducing photocatalysts confirmed PXX (*E*_1/2_(PXX^·+^/PXX*) = −2.00 V *vs.* SCE)^62,63^ to be optimal compared to other literature-reported organophotocatalysts PC-2 (*E*_1/2_(PC^·+^/PC*) = −2.08 V *vs.* SCE)^64^ and PC-3 (*E*_1/2_ (PC^·+^/PC*) = −1.43 V *vs.* SCE)^56^ (entries 5 and 6). Although *fac*-Ir(ppy)_3_ also furnished 2aa in excellent yield, PXX was selected for further studies because it provided comparable reactivity while avoiding the use of a precious-metal photocatalyst (entry 7). It was found that salophen-ligated catalyst [Co-1] was superior to [Co-2] or [Co-3], as a minor drop in yield was observed (entries 8 and 9). Replacement of the Brønsted acid, Lut·HOTf, with its collidinium analogue, Col·HOTf, had minimal impact on the formation of 2aa, yet the pyridinium equivalent Py·HOTf failed to generate any product (entries 10 and 11). Performing the reaction without prior degassing led to a significant reduction in yield, affording 2aa in only 15% yield (entry 12). Control experiments confirmed that PXX, [Co-1], Lut·HOTf, and LED irradiation were all required for reactivity, as tetrahydroquinoline 2aa was not observed when any of these components were omitted (entry 13).

**Table 1:** Hydroarylation reaction optimisation^a^

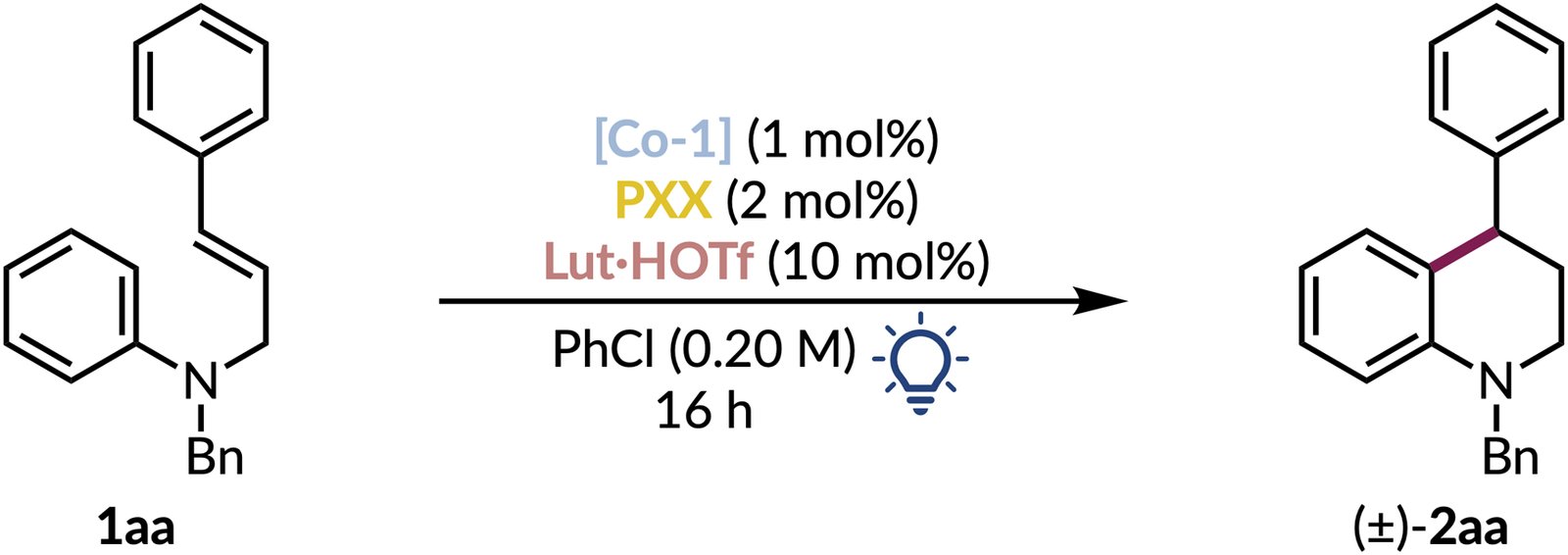		
Entry	Deviation	Yield of (±)-2aa
1	—	99 (92)
2	CH_2_Cl_2_	90
3	Acetone	84
4	THF	32
5	PC-2	42
6	PC-3	58
7	*fac*-Ir(ppy)_3_	99
8	[Co-2]	89
9	[Co-3]	83
10	Col·HOTf	90
11	**Py·HOTf**	0
12	No solvent degassing	15
13	No PC or Co or Lut·HOTf or *hv*	0
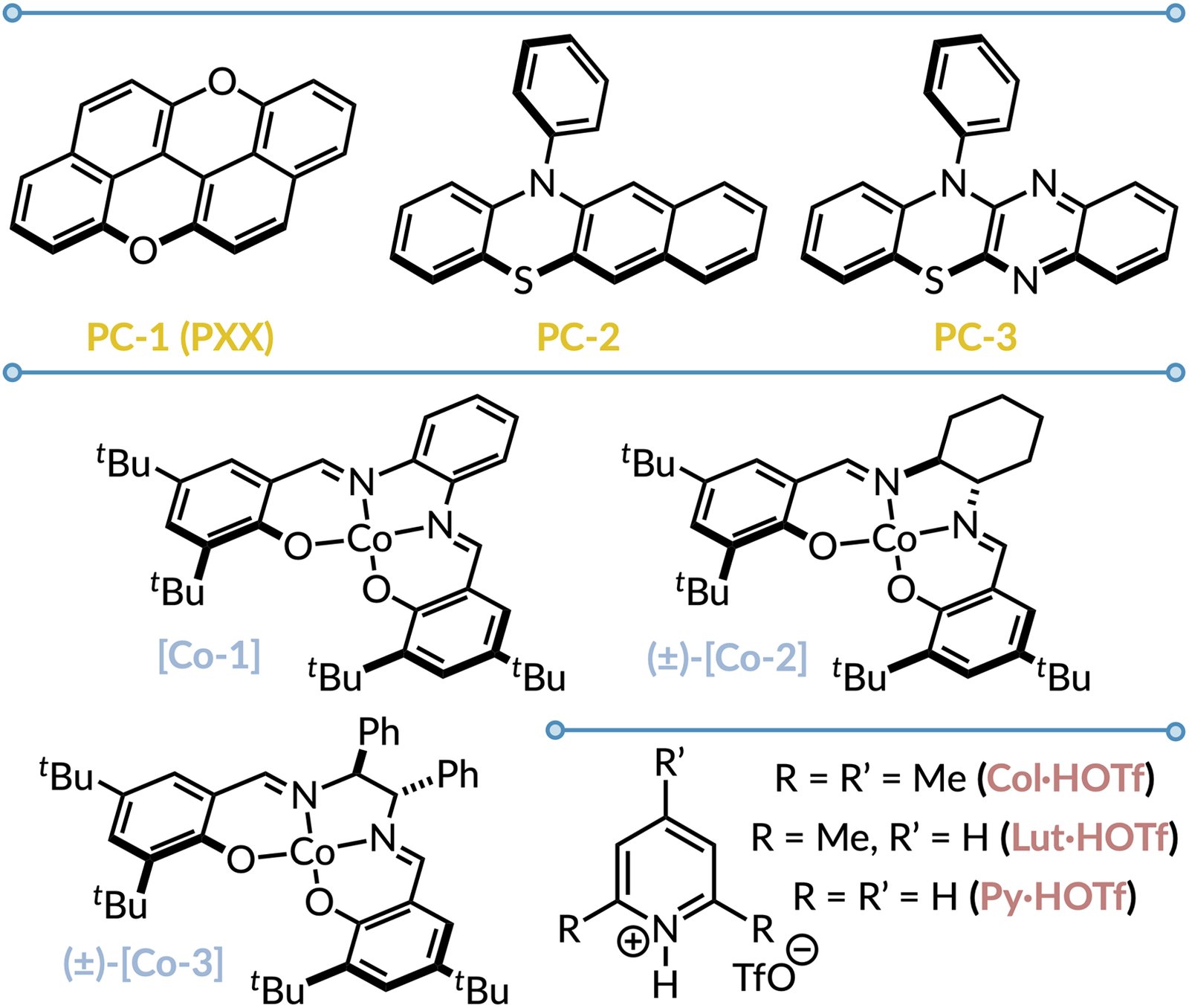		

a Reactions were performed on laa (0.20 mmol) with the yield of 2aa determined by ^1^H NMR analysis of the crude reaction with 1,2-dibromoethane (see SI for details). Isolated yields given in brackets.

### Reaction scope

With the optimised conditions in hand, the generality of the reaction was then assessed by synthesising a wide variety of THQs (Fig. 2). Varying the electronics of the *N*-benzyl substituent had a negligible effect, with substrates bearing either electron-donating or electron-withdrawing groups leading to high yields of 2aa–2ad. The reaction also proceeded smoothly with alternative aryl and heteroaryl groups at this site, forming the corresponding THQs 2ae and 2af in excellent yields. Additionally, it was found that the *N*-benzyl substituent was not mandatory, as the simple *N*-alkyl 2ag–2ah and *N*-aryl 2ai products were obtained with high efficiency. A substrate containing two distinct alkenes was hydroarylated regioselectively, as the *N*-cinnamyl olefin was activated over the *N*-allyl alkene under the standard reaction conditions, enabling the synthesis of the *N*-allyl THQ 2aj in very good yield. Gratifyingly, *N*,*N*-bis(cinnamyl)aniline 1ak underwent sequential hydroarylation, enabling rapid access to the corresponding julolidine 2ak as a 1.0 : 1.0 mixture of diastereomers. In contrast, the use of standard nitrogen-deactivating protecting groups such as Boc or tosyl resulted in no conversion to the desired product (see SI for details). These electron-withdrawing groups suppress lone pair delocalisation from nitrogen into the arene, which we hypothesise reduces its nucleophilicity below the threshold required for the C–C bond-forming step. Fortunately, the inclusion of methoxy groups on the *N*-aryl ring was sufficient to restore reactivity, allowing the tosyl-protected THQ 2al and the 3,4-dihydroquinolone 2am to be prepared in superb yields.

**Fig. 2:**
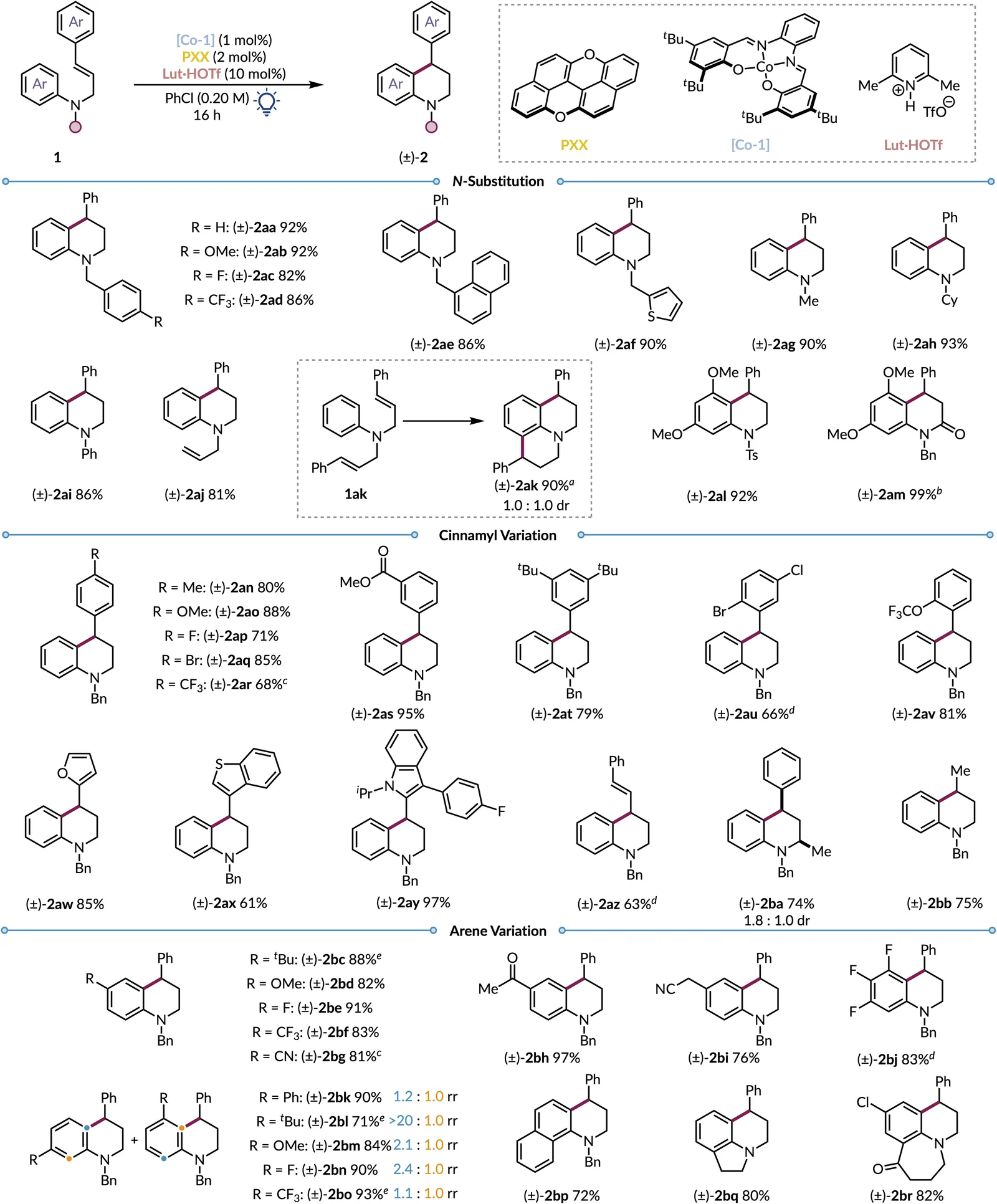
Scope of *N*-substituted tetrahydroquinolines *via* intramolecular hydroarylation. Reactions performed on 0.20 mmol of alkene, [Co-1] (1 mol%), PXX (2 mol%), Lut·HOTf (10 mol%) in PhCl (0.20 M) under N_2_ for 16 h at RT irradiated with a single 450 nm lamp. Yields refer to isolated products. ^*a*^ Lut·HOTf (20 mol%). *^b^* Two 450 nm lamps. *^c^* 64 h. *^d^* 40 h. ^*e*^[Co-1] (2 mol%), PXX (4 mol%).

We next examined substitution on the aryl ring of the *N*-cinnamyl fragment, with a wide range of *p*-substituents being well tolerated, forming the analogous THQs in good to excellent yields 2an–2ar. However, the highly electron-deficient *p*-CF_3_-substituted substrate reacted sluggishly, requiring 64 h for complete conversion to THQ 2ar. Substrates bearing *meta*- or *ortho*-substituted arenes on the cinnamyl fragment were also found to be amenable, giving moderate to excellent yields of products 2as–2av. Alternative heteroaryl cinnamyl derivatives, including 2-furan 2aw, 3-benzothiophene 2ax and a fluvastatin-inspired indole derivative 2ay, also demonstrated efficient conversion to the corresponding THQs. Interestingly, when a styrenyldiene was subjected to the reaction conditions, THQ 2az was formed preferentially over the possible tetrahydrobenzo[*b*]azocine core, leaving the styrenyl olefin intact as a useful chemical handle. Variation of the aliphatic portion of the THQ scaffold was also explored. Substitution at the C2 position was well tolerated with the incorporation of a methyl group, providing 2ba in good yield. Notably, styrenyl alkenes were not essential, with C4-methyl THQ 2bb being formed by replacing the cinnamyl fragment with a homoallylic chain. Unfortunately, attempts to form THQs bearing quaternary centres at C4 either resulted in recovery of starting material or gave complex mixtures from which the desired products could not be isolated. Similarly, cinnamyl substrates that would introduce a C3 methyl or *n*-hexyl group onto the THQ core predominantly returned starting materials (see SI for details).

Next, modification of the *N*-aryl ring was also surveyed, with a wide range of electron-donating and electron-withdrawing substituents demonstrating high tolerance at the *para-*position to give products 2bc–2bj. A comparable assortment of *meta*-substituents (2bk–2bo) displayed similar levels of efficiency. However, due to the unsymmetrical nature of the aniline ring in these substrates, mixtures of regioisomers were formed, except for the *t*-Bu-substituted example 2bl, where steric bias led to enhanced regioselectivity. The presence of an *ortho*-substituent was only tolerated when it was part of a cyclic framework, either through arene annulation (2bp) or by tethering to the nitrogen atom (2bq and 2br). In the absence of this conformational lock, no desired product was formed under the reaction conditions; instead, slow degradation of the starting material was observed, leading to the accumulation of styrene. We postulate that, in examples lacking this conformational lock, significant A^1,3^-strain between the *ortho-*substituent and the *N*-benzyl group disfavours adoption of the conformations that permit facile delocalisation of the nitrogen lone pair into the arene. This is consistent with the sensitivity of this transformation to arene electronics; the resulting attenuation of arene nucleophilicity is likely detrimental, as observed with the use of Boc or Tosyl *N*-protecting groups. The formation of styrene was observed with several substrates bearing different *ortho*-substituents, suggesting that fragmentation of the cinnamyl moiety is responsible for its generation. It was hypothesised that by minimising benzylic strain, substrates with acyclic *ortho*-groups would become productive. However, during the reaction development, it was observed that unprotected THQ 4a was formed relatively slowly, resulting in less than 50% conversion of alkene 3a even after 48 h (see SI for details). Therefore, to overcome this, the photon flux was approximately doubled by irradiating the reaction with two LED lamps, which fully consumed 3a after 24 h and afforded the corresponding THQ 4a in 67% yield (Fig. 3). Pleasingly, a variety of *ortho*-substituents were now well tolerated, allowing 8-substituted THQs 4b–4f to be accessed.

**Fig. 3:**
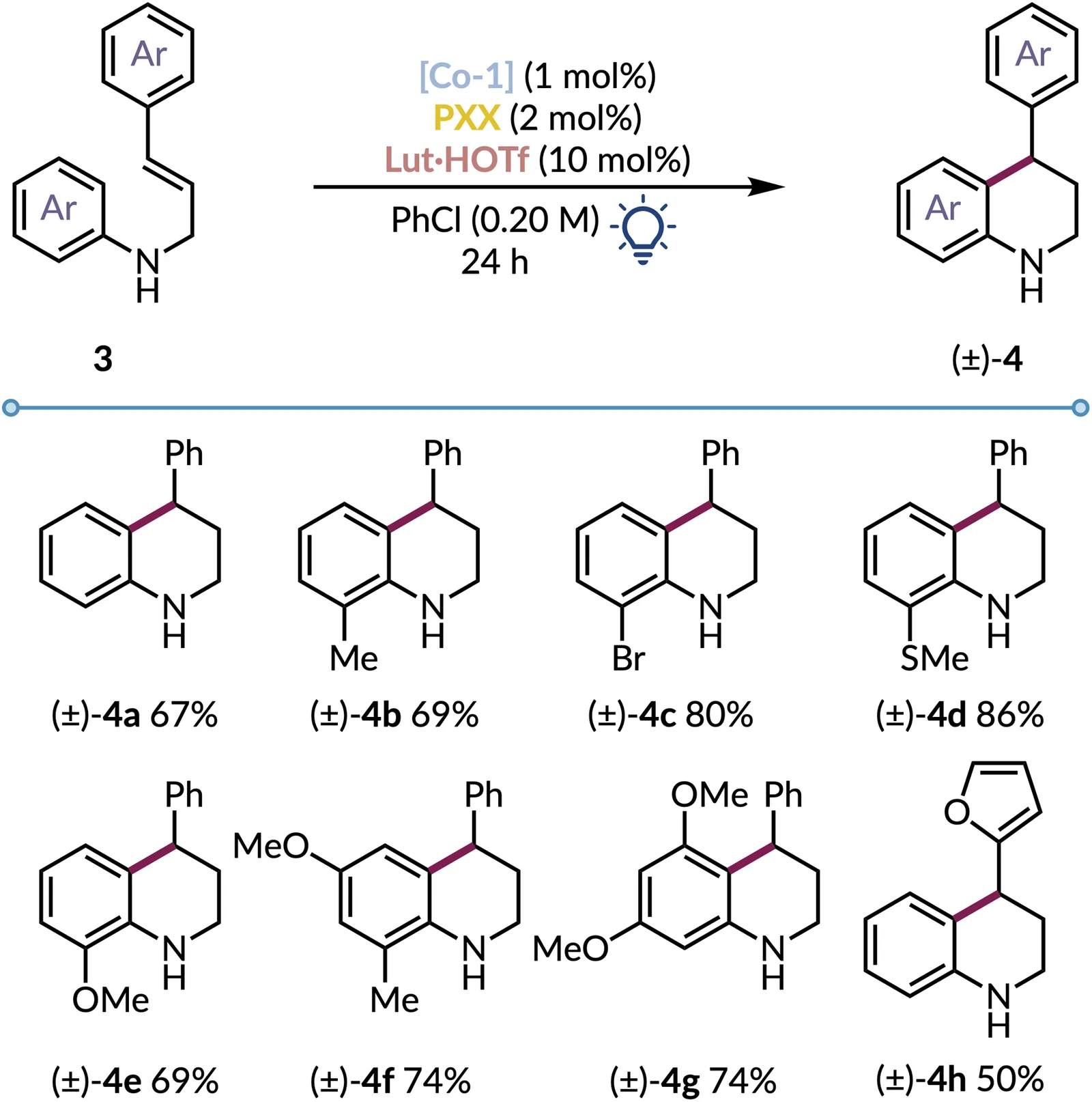
Scope of *N*-unsubstituted tetrahydroquinolines. Reactions performed on 0.20 mmol of alkene, [Co-1] (1 mol%), PXX (2 mol%), Lut·HOTf (10 mol%) in PhCl (0.20 M) under N_2_ for 24 h, irradiated with two 450 nm lamps.

To illustrate the scalability of this light-driven MHAT protocol, we explored its translation into a photoflow reactor. Initial studies emulating the batch conditions resulted in only moderate conversion, which we attributed primarily to the limited solubility of Lut·HOTf in chlorobenzene. Consequently, the reaction conditions underwent a partial re-optimisation for the flow system (see SI for details). Accordingly, the photocatalyst and solvent were replaced with Ir(ppy)_3_ and dichloromethane, respectively, leading to significantly improved conversion of alkene 1aa within a considerably reduced timeframe (30 min in flow *vs.* 16 h in batch). The optimised process was operated using a single-feed configuration, delivering THQ 2aa at a productivity rate of 0.69 g h^−1^ (Fig. 4).

**Fig. 4:**
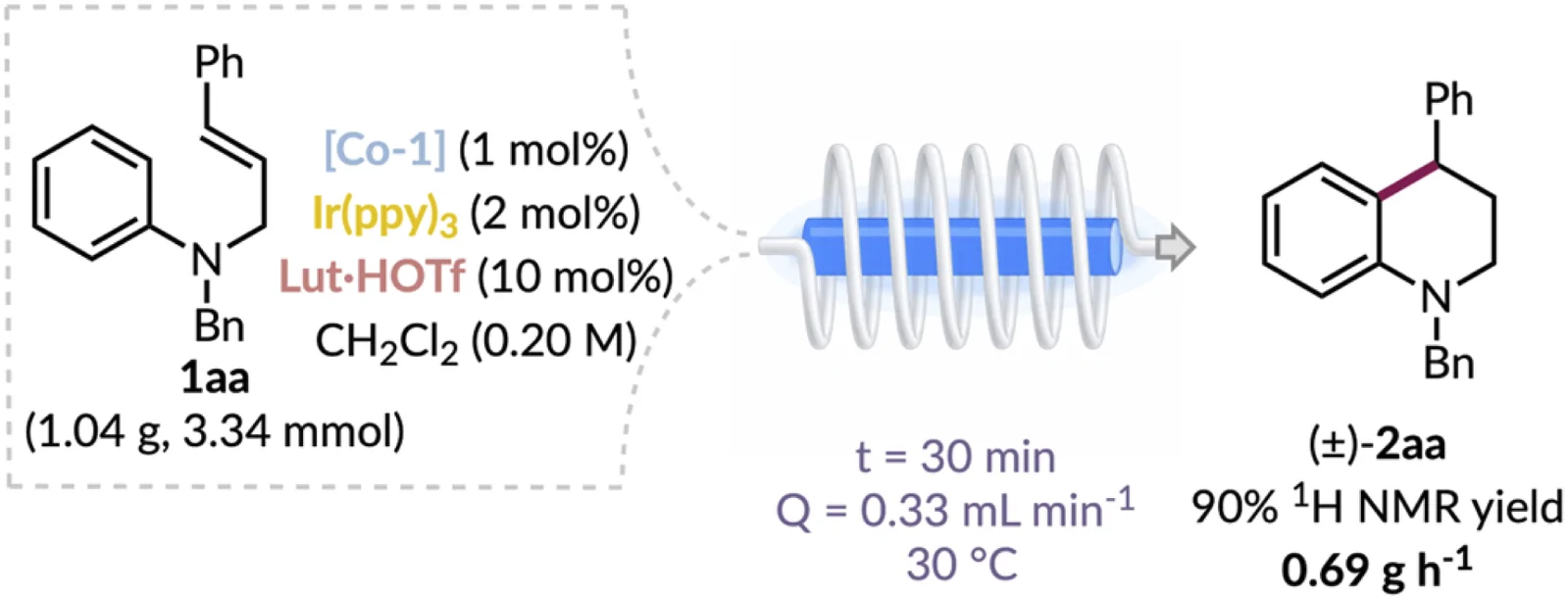
Photoflow scale up of the cobalt-salen intramolecular hydroarylation.

### Mechanistic investigation

To gain a deeper insight into this hydroarylation reaction, we performed a series of mechanistic experiments. When the *N*-aryl ring was fully deuterated (1aa-d_5_), it was transformed into THQ 2aa-d_5_ in 90% yield with 80% deuterium incorporation at the C3 position (Fig. 5A). The high levels of deuteration at C3 indicate that Co(iii)–D is formed during the reaction. This is consistent with deprotonation of the Wheland intermediate by 2,6-lutidine, which regenerates the lutidinium cation and restores aromaticity. The slight erosion in deuterium incorporation is expected, as the unlabelled Lut·HOTf provides a competing protium source. A light/dark experiment demonstrated that the reaction proceeded only during periods of constant irradiation, with no measurable increase in the yield of product 2aa observed during the dark intervals (Fig. 5B).

**Fig. 5:**
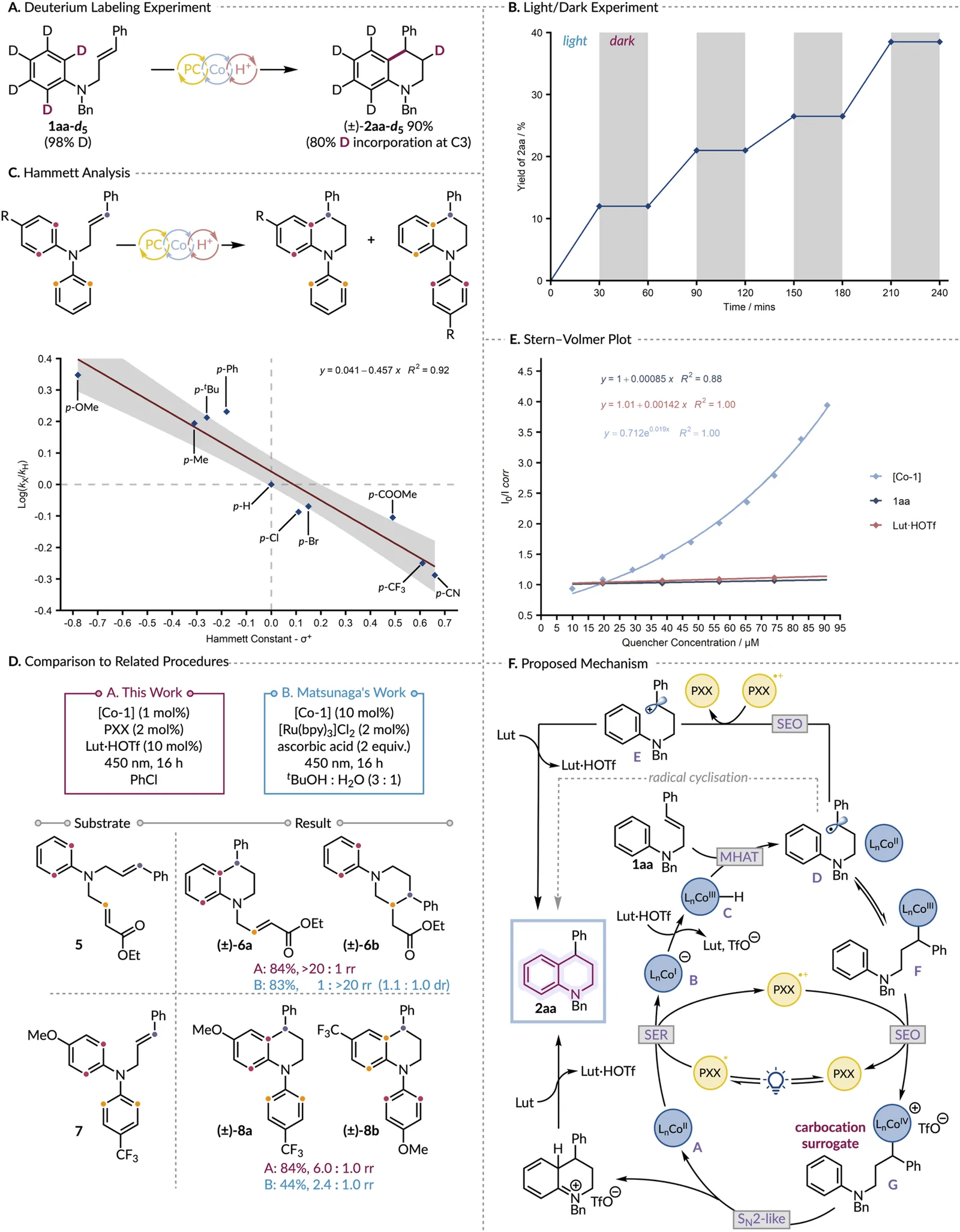
Mechanistic investigation and proposed mechanism: [A] deuterium labelling experiment; [B] light/dark experiment; [C] Hammett analysis; [D] comparison with Matsunaga’s protocol; [E] Stern–Volmer quenching experiments; and [F] proposed mechanism.

Next, to probe the nature of the C–C bond-forming step, we conducted intramolecular competition experiments to determine whether cyclisation was favoured with the more electron-rich or electron-deficient arene (Fig. 5C). The substrates selected for this study paired an unsubstituted (4-H) aryl ring with a 4-substituted (4-X) arene to simplify post-reaction analysis by producing only two regioisomers.^65^ The logarithm of the regioisomer ratio, log_10_(*k*_X_/*k*_H_), was plotted against a series of Hammett substituent constants (*σ*_p_, *σ*_m_, *σ*^+^, and *σ*^−^; see SI for details). The best linear correlations were obtained using *σ*_p_ (*ρ* = −0.63, *R*^2^ = 0.90) and *σ*^+^ (*ρ* = −0.46, *R*^2^ = 0.92) values. This analysis confirmed that electron-rich aryl rings undergo cyclisation preferentially over their electron-deficient counterparts. However, the modestly negative *ρ* values are not conclusive to distinguish between the aryl ring attacking a radical or cationic species to forge the new ring system.

Consequently, to obtain evidence to support the cationic nature of the carbon–carbon bond-forming step, our methodology was compared with Matsunaga's hydroarylation protocol, which is proposed to proceed *via* radical addition to the aryl system (Fig. 5D).^10^ Under our developed conditions, substrate 5 underwent selective arene cyclisation to afford THQ 6a in 84% yield, with no Giese-type product 6b observed. Conversely, under Matsunaga's conditions, the selectivity was completely reversed: THQ 6a was not detected, and *N*-phenyl piperidine 6b was obtained as the sole product. Furthermore, substrate 7, bearing two electronically differentiated *N*-aryl rings bearing *p*-methoxy and *p-*trifluoromethyl substituents, was examined. In this case, both sets of conditions favoured the formation of THQ 8a, which cyclises through the more electron-rich anisidine ring. However, our protocol displayed markedly enhanced regioselectivity relative to Matsunaga's conditions, consistent with a greater dependence on arene nucleophilicity during cyclisation. Overall, these results, together with the poor reactivity of substrates in which nitrogen lone pair donation is attenuated, as discussed above, are consistent with at least partial involvement of a cation-like intermediate during C–C bond formation.

As PXX had not been previously explored in combination with cobalt catalysis, Stern–Volmer quenching experiments were conducted to determine that [Co-1] was the primary quencher of the excited-state photocatalyst, whereas both alkene 1aa and Lut·HOTf show negligible quenching (Fig. 5E). Notably, the Stern–Volmer plot for [Co-1] displayed a positive deviation from linearity over the concentration range examined, even after correction for competitive absorption at the excitation wavelength. Nevertheless, residual inner-filter effects may persist, and contributions from both static and dynamic quenching cannot be excluded (see SI for details).^66^

Based on the experimental evidence gathered and literature precedent,^49,67^ we propose the following mechanistic scenario (Fig. 5F). The excited state photocatalyst (PXX*) performs a single electron reduction (SER) on cobalt(ii)salen A to afford the Brønsted basic cobalt(i) species B and the PXX radical cation. Following this, protonation of Co(i) complex B with Lut·HOTf generates a cobalt(iii) hydride species C. Subsequently, this catalytic intermediate delivers a hydrogen atom regioselectively in a Markovnikov fashion to alkene 1aa, forming secondary benzylic radical D. The requirement for an electron-rich arene, together with the contrasting selectivity observed under literature conditions that invoke radical cyclisation (Fig. 5D), argues against direct radical cyclisation of D as a major product-forming pathway. Two radical-polar crossover pathways from radical D are instead feasible. Direct single-electron oxidation (SEO) of the benzylic radical D (*E*^ox^_1/2_ = +0.37 V *vs.* SCE, for the 1-phenylethyl radical)^68^ by the PXX radical cation (*E*_1/2_[PXX^·+^/PXX] = +0.77 V *vs.* SCE)^62^ would furnish the benzylic carbocation E, which can undergo a Friedel–Crafts cyclisation to form THQ 2aa. Alternatively, secondary benzylic radicals are known to be reversibly captured by cobalt(ii) complexes to form organocobalt(iii) complexes,^69^ and benzylic alkylCo(iv) intermediates have been invoked in related photochemical transformations.^59,70^ Therefore, it is viable that alkylCo(iii) species F (*E*^ox^_1/2_ = +0.39 V *vs.* SCE, when alkyl is *i*-Pr)^71^ could undergo SEO mediated by the PXX radical cation to generate a putative organocobalt(iv) complex G,^67,71^ and return the photocatalyst to its ground state. The carbocation surrogate G can cyclise, followed by deprotonation with lutidine, to form the desired THQ 2aa, thereby restoring the cobalt catalyst to its +2 oxidation state and the Brønsted acid Lut·HOTf. Attempts to distinguish between these two polar pathways using chiral cobalt-salen complexes were unsuccessful (see SI for details), so we cannot exclude the possibility that product formation occurs through multiple competing pathways.

## Conclusions

In summary, we have developed an efficient cobalt-salen catalysed photochemical cycloisomerisation of olefins for the construction of tetrahydroquinoline derivatives. The protocol is highly atom-economical, circumventing the requirement for stoichiometric oxidants or reductants. A commercial organic dye, PXX, was employed as the photoredox catalyst, demonstrating its utility for modulating the oxidation state of cobalt complexes. For operational reasons, *fac*-Ir(ppy)_3_ was used in place of PXX to scale up the transformation using a commercial photoflow system to illustrate that light-driven cobalt MHAT-type transformations can be conducted in continuous flow. Preliminary mechanistic investigations are consistent with the cobalt catalyst trapping a radical intermediate and redirecting it towards a polar, cation-like cyclisation pathway. Future work will focus on expanding the role of PXX-based dyes in cobaltaphotoredox catalysis, while addressing the practical limitations that currently restrict their application in continuous flow systems.

## Author contributions

BE and CPJ conceived the project. BE, DGG, and RHH performed the batch experiments and analysed the data. TJ carried out and analysed the continuous-flow photochemical experiments with supervision from LREP. BE, DGG, RHH, and CPJ drafted the manuscript with input from all other authors. CPJ directed the project and acquired funding. All authors reviewed and approved the final manuscript.

## Conflicts of interest

There are no conflicts to declare.

## Supplementary Material

SC-017-D6SC05922H-s001

## Data Availability

The underlying data that support the findings of this study are openly available in PURE at https://doi.org/10.17630/1bff24be-9184-4ab7-92fa-b0f230941158, reference number 336359794. The data supporting this article are included in the supplementary information (SI). Supplementary information: comprising experimental procedures, characterisation data, and mechanistic studies. See DOI: https://doi.org/10.1039/d6sc05922h.
