# Supplementary material for: Cobaltaphotocatalysed intramolecular hydroarylation of alkenes for the construction of tetrahydroquinolines
Source: Chem Sci. 2026 Aug 20;17(40):19304–12. doi: 10.1039/d6sc05922h (PMC13519910; doi:10.1039/d6sc05922h)

Supplementary Information (SI) for Chemical Science.
This journal is © The Royal Society of Chemistry 2026

Supplementary Information for

Cobaltaphotocatalysed Intramolecular Hydroarylation of alkenes for the Construction of Tetrahydroquinolines

Balakumar Emayavaramban[1#], Reece H. Hoogesteger[1#], David G. Groves[1#], Tatjana Jerkovic[2], Loïc R. E. Pantaine[2], Craig P. Johnston[1*]

[#]Authors contributed equally

[1] EaStCHEM, School of Chemistry, University of St Andrews St Andrews, Fife, Scotland, KY16 9ST, UK

[2] Process and Analytical Chemistry, Pharmaron UK Ltd., Hoddesdon, UK

*Correspondence to: cpj3@st-andrews.ac.uk

Table of Contents

1. General Information 1
1.1 General Experimental....1
1.2 Analysis of products....3
2. Reaction Development 5
2.1 Catalyst and Reagent Syntheses 5
2.2 Substrate Synthesis 8
2.2.1 General Procedures....8
2.2.2 Substrate Synthesis 10
2.3 Reaction Optimisation and Deviations....63
2.4 Reaction Scope....65
2.5 Scope Limitation 102
3. Photo-flow Experiments....103
3.1 General Procedures for Photoflow reactions 103
3.2 Optimisation of Batch Conditions to Continuous Flow Setup....105
3.3 Optimised procedure for 1 gram lab-scale continuous photoflow setup 110
3.4 Quantitative ^{1}H NMR analysis and Assay Yield calculation....111
4. Mechanistic Investigation 114
4.1 Literature Condition Comparisons 114
4.1.1 Radical Cyclisation Experiments 114
4.1.2 Differing Arene Cyclisation Experiments....116
4.2 Isotopic Labelling Experiments 119
4.3 On-Off Experiments....121
4.4 Hammett Analysis 122
4.4.1 Hammett Substrate Synthesis 122
4.4.2 Competition Reactions 129
4.5 Stern-Volmer Quenching....133
4.5.1 UV-Vis Measurements....133

4.5.2 Quenching Experiments 134
4.5.3 Inner-Filter-Effect 136
4.5.4 Low concentration Stern-Volmer for [Co-1] 138
4.6 Cobalt-Catalyst Control Experiments 139
4.6.1 Diastereocontrol of 2ba 139
4.6.2 Enantioselective Example of 2aa 140
5. References 141
6. Spectra 142

1. General Information

1.1 General Experimental

Reactions involving moisture sensitive reagents were carried out in flame-dried glassware under an inert atmosphere (N_2) using standard vacuum line techniques. Anhydrous solvents (Et_2O, CH_2Cl_2, THF, PhMe, DMF) were obtained after passing through an alumina column (Mbraun SPS-800). Anhydrous solvents (MeCN) were purchased as Sure/Seal™ bottles from Sigma-Aldrich or as AcroSeal™ bottles from Thermo Scientific Chemicals (formerly Acros Organics) and used without further purification. All other solvents and commercial reagents were used as received without further purification unless otherwise stated. Hexane is defined as ^{n}hexane.

^{n}Butyllithium (^{n}BuLi) solutions were titrated before use in THF using diphenyl acetic acid as the indicator. Grignard reagents were titrated before use in THF using 2-hydroxybenzaldehyde phenylhydrazone as the indicator.

Room temperature (RT) refers to 20 – 25 °C. A reaction temperature of 0 °C was obtained using ice/water bath. Reaction temperatures varying from 0 °C to -40 °C were obtained by using an acetone bath cooled by an immersion chiller (Huber TC45E-F). Reaction temperatures varying from -40 °C to -70 °C were obtained by using an acetone bath cooled by an immersion chiller (Huber TC100E-F). A reaction temperature of -78 °C was obtained using CO_2(s)/acetone bath or N_2(l)/acetone bath. Reactions involving heating were performed using DrySyn blocks and a contact thermocouple.

Under reduced pressure refers to the use of an IKA RV8 with an IKA HB digital heating bath and Vacuubrand MD1C vacuum controller. Rotary evaporator condensers were fitted to a recirculating chiller (Julabo F500) filled with either a water/ethylene glycol mixture (1:1) or Julabo Thermal G cooling fluid set to 0 °C. "Dried in vacuum" refers to the use of a Schlenk line and high vacuum pump (Edwards RV8).

Analytical thin layer chromatography (TLC) was performed using pre-coated aluminum backed plates (Merck TLC Silica 60 F254). Visualisation was achieved under ultraviolet light (short wavelength: λ = 254 nm or long wavelength: λ = 365 nm) and/or with staining with aqueous $KMnO_4$ solution or ethanolic phosphomolybdic acid (PMA) solution followed by gentle heating. Flash chromatography was performed using compressed air on glass columns containing porosity 2/3 sintered disks over silica gel (60 Å pore size, 40 – 63 μm particle size), using the solvent system stated.

Photochemical reactions were irradiated with the commercially available sources of light as stated. The sources of light are characterised as follows: λ of light source in nm, (part number, electric power (W), relative irradiance at 2 cm (mW/cm^2). Most light sources are EvoluChem

type LEDs. 425 nm (HCK1012-01-012, 18 W, 166 mW/cm^2), 450 nm (HCK1012-01-002, 18 W, 328 mW/cm^2), 475 nm (HCK1012-01-003, 18 W, 206 mW/cm^2). Kessil 456 nm (PR160L Gen 2, 50 W, 352 mW/cm^2 at 1 cm). Reaction temperatures were maintained with the use of a commercially available household fans or compressed air lines.

1.2 Analysis of products

Compound names have been generated using ChemDraw® Professional (PerkinElmer) software.

Melting Points (mp) were measured on a Büchi M-565 melting point apparatus and are uncorrected. Solvents are reported in brackets when the solid was recrystallised and *dec* refers to decomposition. Temperatures are reported in °C.

High Performance Liquid Chromatography (HPLC) was performed on an Agilent 1260 Infinity II LC system consisting of a 1260 Infinity II Quaternary Pump, 1260 Infinity II Vial Sampler, 1260 Infinity II Multicolumn Thermostat, and a 1260 Infinity II Diode Array Detector WR. Separation was achieved using either Daicel CHIRALPAK® AD-H, AS-H, IA, IB, IC, or IG columns or Daicel CHIRALCEL® OJ-H or OD-H columns using the method stated. All columns (4.6 mm ø x 250 mm, 5 μm particle size) were used with corresponding guard column (4 mm ø x 10 mm, 5 μm particle size). HPLC traces of enantiomerically enriched compounds were compared with authentic racemic samples. Wavelengths (λ) are reported in nm, retention times (tR) are reported in minutes, temperatures are reported in °C, and solvent flow rates are reported in mL/min.

Infrared spectra (ν_{max}) were recorded on a Shimadzu IRAffinity-1 Fourier transform IR spectrophotometer fitted with a Specac Quest ATR accessory (diamond puck). Spectra were recorded neat with characteristic absorption wavenumbers (ν_{max}) reported in cm^{-1}.

Nuclear Magnetic Resonance (NMR) spectra (^{1}H, $^{13}C\{^{1}H\}$, and ^{19}F) were acquired on either a Bruker AVII 400 with a BBFO probe (^{1}H 400 MHz; $^{13}C\{^{1}H\}$ 101 MHz; ^{19}F 377 MHz), a Bruker AVIII-HD 400 with a BBFO probe (^{1}H 400 MHz; $^{13}C\{^{1}H\}$ 101 MHz; ^{19}F 376 MHz), a Bruker Neo 400 with a BBF-H-D-iprobe (^{1}H 400 MHz; $^{13}C\{^{1}H\}$ 101 MHz; ^{19}F 377 MHz)), a Bruker AVIII-HD 500 with a SmartProbe BBFO+ probe (^{1}H 500 MHz, $^{13}C\{^{1}H\}$ 126 MHz, ^{19}F 470 MHz), a Bruker AVIII 500 with a CryoProbe Prodigy BBO probe (^{1}H 500 MHz, $^{13}C\{^{1}H\}$ 126 MHz, ^{19}F 471 MHz), or a Bruker AVIII-HD 700 with CryoProbe Prodigy TCI probe (^{1}H 700 MHz, $^{13}C\{^{1}H\}$ 176 MHz). All NMR spectra were recorded at 25 °C unless otherwise stated with the stated deuterated solvent used as a lock for spectra and internal reference ($CDCl_3$: ^{1}H, 7.26 ppm; ^{13}C, 77.2 ppm). All chemical shifts are quoted in parts per million (ppm) relative to the residual solvent peak. All coupling constants, *J*, are quoted in Hz. Multiplicities are indicated as s (singlet), d (doublet), t (triplet), q (quartet), m (multiplet), and multiples thereof. 2D ^{1}H correlated spectroscopy (COSY), 2D ^{1}H–^{13}C heteronuclear multiple-bond correlation spectroscopy (HMBC), and 2D ^{1}H–^{13}C heteronuclear single quantum coherence (HSQC) were used when necessary for full structural determination.

High resolution mass spectrometry (HRMS) was acquired by either electrospray ionisation (ESI), electron impact (EI), or atmospheric-pressure chemical ionisation (APCI) at the University of St

Andrews Mass Spectrometry Facility and the University of Edinburgh Mass Spectrometry Facility. Values are reported as a ratio of mass to charge in Daltons. Under ESI^+ conditions intermediate and substrate compounds can form positively charged iminium fragments giving rise to the $[M-H]^+$ notation. Additionally, products can form positively charged quinolinium fragments giving rise to the $[M-3H]^+$ notation.

2. Reaction Development

2.1 Catalyst and Reagent Syntheses

Salen-ligands and cobalt-catalysts were prepared according to literature procedures from Jacobsen and co-workers.[1]

6,6'-((1*E*,1'*E*)-(1,2-Phenylenebis(azaneylylidene))bis(methaneylylidene))bis(2,4-di-*tert*-butylphenol) (L1)

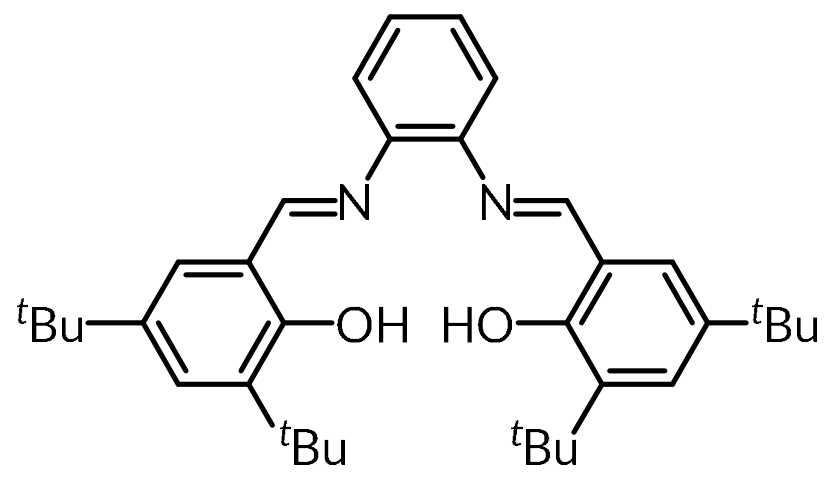

A solution of benzene-1,2-diamine (368 mg, 3.40 mmol) and 3,5-di-*tert*-butyl-2-hydroxybenzaldehyde (1.59 g, 6.80 mmol) in EtOH (20 mL) was refluxed at 80 °C for 2 h. The flask was then sonicated for 5 mins (to aid precipitation) before being cooled in the freezer for 30 mins. The solid was filtered under reduced pressure, washed with MeOH (× 3), and then dried in vacuo to yield **L1** (1.18 g, 64%) as a yellow solid which was used without further purification.

mp 197 – 198 °C

ν_{max}(film) / cm^{-1}: 2954, 1614 (C=N), 1574, 1437, 1362, 1273, 1250, 1200, 1171, 752

^{1}H NMR (500 MHz, $CDCl_3$) δ_H: 13.53 (2H, s), 8.66 (2H, s), 7.44 (2H, d, *J* 2.0), 7.34 – 7.29 (2H, m), 7.25 – 7.22 (2H, m), 7.22 (2H, d, *J* 2.2), 1.44 (18H, s), 1.32 (18H, s)

^{13}C{^{1}H} NMR (126 MHz, $CDCl_3$) δ_C: 164.9, 158.7, 142.9, 140.5, 137.3, 128.3, 127.4, 126.9, 120.0, 118.5, 35.3, 34.3, 31.6, 29.6

HRMS: (ESI$^+$) $C_{36}H_{49}N_2O_2$ [M+H]$^+$ requires 541.3789, found 541.3778 (-2.0 ppm)

Co(II) [6,6'-((1*E*,1'*E*)-(1,2-Phenylenebis(azaneylylidene))bis(methaneylylidene))bis(2,4-di-*tert*-butylphenol)] ([Co-1])

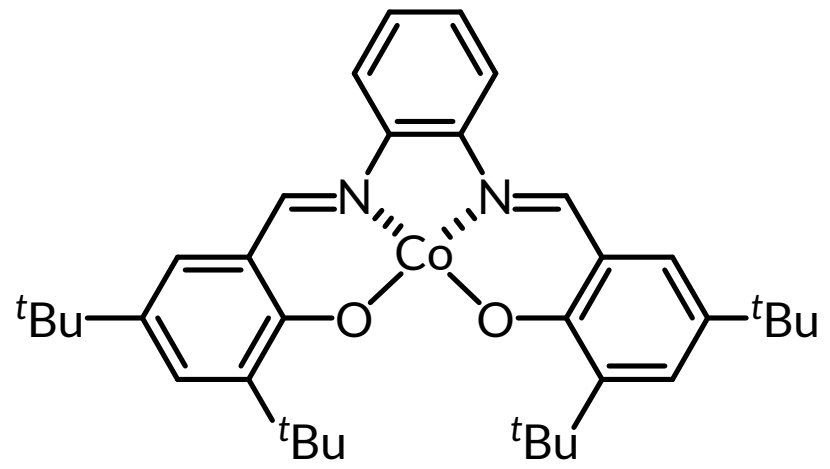

A solution of ligand **L1** (1.18 g, 2.18 mmol) and Co(acac)$_2$·4H$_2$O (600 mg, 2.40 mmol) in EtOH (20 mL) was refluxed at 80 °C for 2 h. The flask was then sonicated for 5 mins (to aid precipitation) before being cooled in the freezer for 30 mins. The solid was filtered under reduced pressure, washed with MeOH (× 3), and then dried in vacuo to yield **[Co-1]** (1.15 g, 88%) as a dark-purple solid.

mp >250 °C

ν_{max}(film) / cm^{-1}: 2935, 1520 (C=N), 1354, 1198, 785

HRMS: (ESI$^+$) $C_{36}H_{46}CoN_2O_2$ [M]$^+$ requires 597.2886, found 597.2874 (-2.0 ppm)

***peri*-Xanthenoxanthene (PXX)**

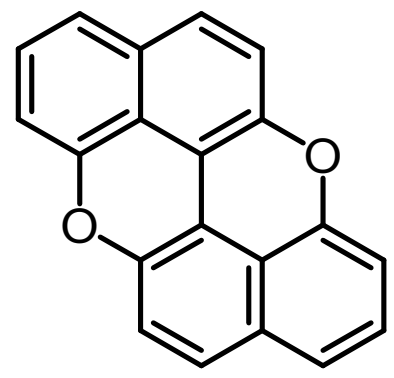

Prepared according to literature.[2] To [1,1′-binaphthalene]-2,2′-diol (BINOL) (573 mg, 2.00 mmol), CuI (1.14 g, 6.00 mmol) and pivalic acid (0.45 mL, 4.00 mmol) was added DMSO (25 mL). The reaction mixture was stirred open to the air at 140 °C for 8 h. The reaction mixture was cooled to RT then diluted with CH_2Cl_2. The organic layer was washed with 30% aq. NH_4OH solution (× 3). The combined aqueous layers were then extracted with CH_2Cl_2 (× 3). The combined organics layers were washed with brine, dried over Na_2SO_4, filtered, and concentrated under reduced pressure. The crude product was purified by silica-gel column chromatography (hexane/CH_2Cl_2, 100:0 → 95:5) to give **PXX** (311 mg, 55%) as a yellow solid with data in accordance with the literature.[2]

mp 238 – 240 °C

ν_{max}(film) / cm^{-1}: 2359, 1603, 1504, 1425, 1331, 1254, 1074, 816

^{1}H NMR (700 MHz, $CDCl_3$) δ_H: 7.30 (2H, d, *J* 8.9), 7.12 – 7.07 (4H, m), 6.91 (2H, d, *J* 9.0), 6.69 – 6.59 (2H, m)

^{13}C{^{1}H} NMR (176 MHz, $CDCl_3$) δ_C: 152.9, 144.4, 131.5, 127.3, 126.5, 121.8, 120.2, 117.5, 111.7, 108.8

2,6-Dimethylpyridin-1-ium trifluoromethanesulfonate (Lut·HOTf)

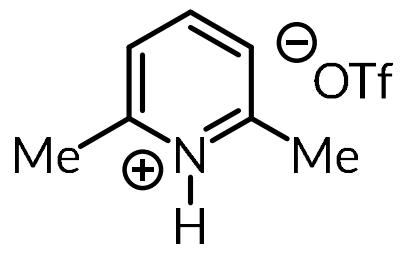

Prepared according to literature.[3] To solution of lutidine (1.1 mL, 9.33 mmol) in ^{n}pentane (10 mL) at RT was added triflic acid (0.83 mL, 9.33 mmol) dropwise over 5 minutes. The reaction was stirred for 15 minutes at RT, and the resulting white solid was filtered and washed with pentane (× 3) to give **Lut·HOTf** (1.51 g, 63%) as a white solid with data in accordance with the literature.[3]

mp 86 – 88 °C

ν_{max}(film) / cm^{-1}: 3503, 2909, 1467, 1277, 1238, 1153, 1026, 792, 635

^{1}H NMR (500 MHz, $CDCl_3$) δ_H: 8.22 (1H, t, *J* 7.9), 7.55 (2H, d, *J* 7.9), 2.86 (6H, s)

^{13}C{^{1}H} NMR (126 MHz, $CDCl_3$) δ_C: 154.2, 145.9, 125.0, 120.4 (q, *J* 319.0), 19.8

^{19}F NMR (471 MHz, $CDCl_3$) δ_F: -78.5 (s)

2.2 Substrate Synthesis

2.2.1 General Procedures

General procedure A: Reductive amination

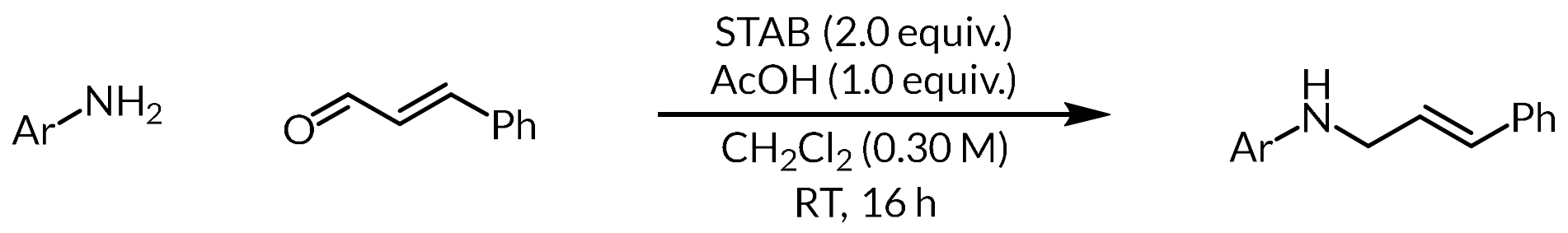

To a solution of aniline (1.0 equiv.), *trans*-cinnamaldehyde (1.0 equiv.), and acetic acid (1.0 equiv.) in CH_2Cl_2 (0.30 M) was added sodium triacetoxyborohydride (STAB) (2.0 equiv.) and the mixture was stirred at room temperature for 16 h. The reaction mixture was quenched with sat. aq. $NaHCO_3$ and stirred for 30 minutes. The layers were separated and the aqueous layer was extracted with CH_2Cl_2 (× 3). The combined organic layers were washed with brine, dried over Na_2SO_4, filtered, and concentrated under reduced pressure. The crude product was purified by silica-gel column chromatography.

General procedure B: Alkylation

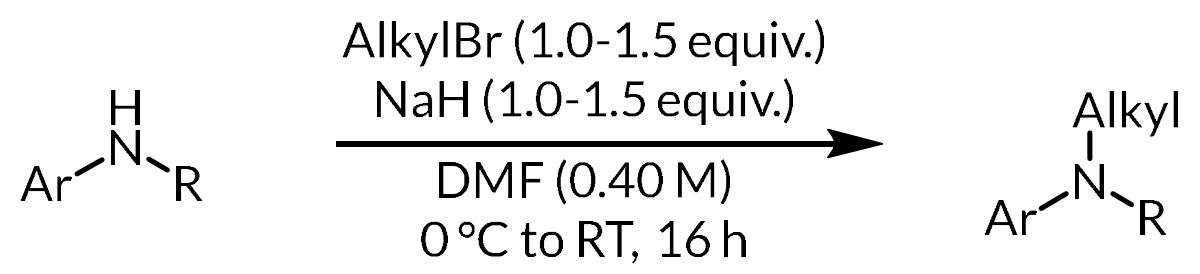

To a solution of secondary amine (1.0 equiv.) in dry DMF (0.40 M) at 0 °C was added sodium hydride (60% in mineral oil, 1.0-1.5 equiv.) and stirred for 15 minutes. Alkyl bromide (1.0-1.5 equiv.) was then added, warmed to RT, and stirred for 16 h. The reaction mixture was quenched with water, and the aqueous layer was extracted with EtOAc (× 3). The combined organic layers were washed with water (× 3), dried over Na_2SO_4, filtered, and concentrated under reduced pressure. The crude product was purified by silica-gel column chromatography.

General procedure C: Benzylation

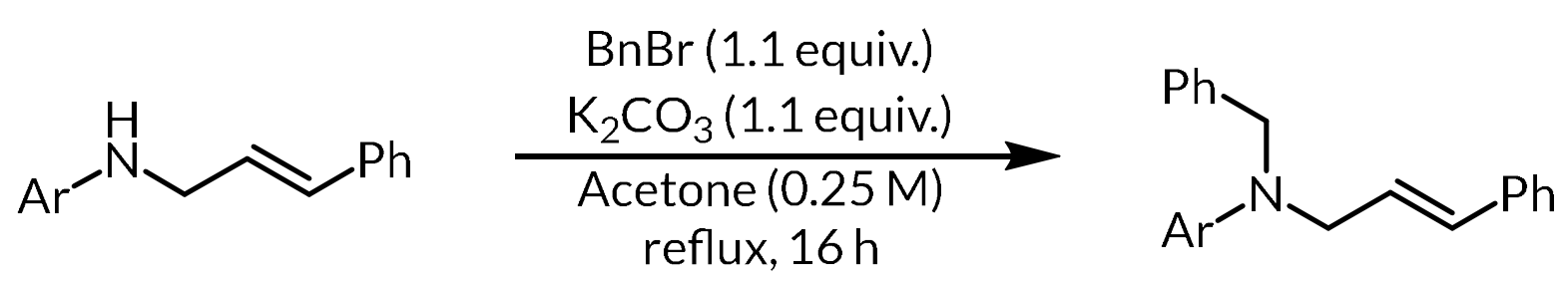

To a solution of secondary amine (1.0 equiv.) and K_2CO_3 (1.1 equiv.) in acetone (0.25 M) was added benzyl bromide (1.1 equiv.) and stirred at reflux for 16 h. The reaction mixture was cooled to RT, filtered, and the filter cake was washed with acetone (× 3). The solvent was removed under reduced pressure, and the crude product was purified by silica-gel column chromatography.

General procedure D: Wittig olefination

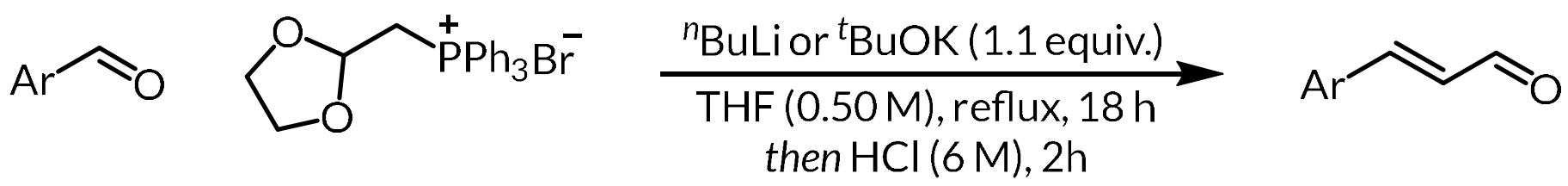

To a solution of (1,3-diozolan-2-ylmethyl)triphenylphosphanium bromide (1.1 equiv.) in dry THF (0.50 M) was added ^{n}BuLi (2.5 M in hexane, 1.1 equiv.) or ^{t}BuOK (1.1 equiv.) and stirred at 0 °C for 1 h. Benzaldehyde was added (1.0 equiv.) and stirred at reflux for 16 h. The reaction mixture was cooled to RT and added 6.0 M aq. HCl. The solution stirred for a further 2 h then the aqueous layer was extracted with CH_2Cl_2 (× 3). The combined organic layers were neutralized with sat. aq. $NaHCO_3$, dried over Na_2SO_4, filtered, and concentrated under reduced pressure. The crude product was purified by silica-gel column chromatography.

2.2.2 Substrate Synthesis

_N_-Cinnamylaniline (3a)

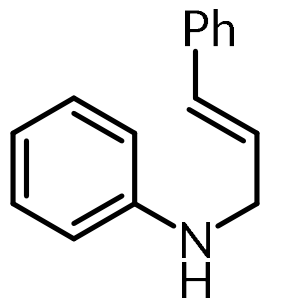

Following general procedure **A**, aniline (0.49 mL, 5.37 mmol), *trans*-cinnamaldehyde (0.68 mL, 5.37 mmol), acetic acid (0.31 mL, 5.37 mmol), sodium triacetoxyborohydride (2.28 g, 10.74 mmol) and CH_2Cl_2 (17.9 mL), after purification by column chromatography (hexane/CH_2Cl_2, 80:20 → 70:30 → 60:40), gave the title compound **3a** (1.12 g, 99%) as a brown oil.

ν_{max} (film) / cm^{-1}: 3410 (N–H), 3022, 1601 (C=C), 1504, 1321, 1250, 966, 746, 691

^{1}H NMR (500 MHz, $CDCl_3$) δ_H: 7.42 – 7.38 (2H, m), 7.36 – 7.31 (2H, m), 7.29 – 7.19 (3H, m), 6.81 – 6.73 (1H, m), 6.70 (2H, d, *J* 8.3), 6.65 (1H, d, *J* 15.9), 6.36 (1H, dt, *J* 16.0, 5.7), 3.96 (2H, d, *J* 5.8), 3.86 (1H, br s)

^{13}C{^{1}H} NMR (126 MHz, $CDCl_3$) δ_C: 148.2, 137.0, 131.6, 129.4, 128.7, 127.7, 127.2, 126.5, 117.8, 113.2, 46.3

HRMS: (ESI$^+$) $C_{15}H_{14}N$ [M-H]$^+$ requires 208.1121, found 208.1122 (+0.5 ppm)

_N_-Benzyl-_N_-cinnamylaniline (1aa)

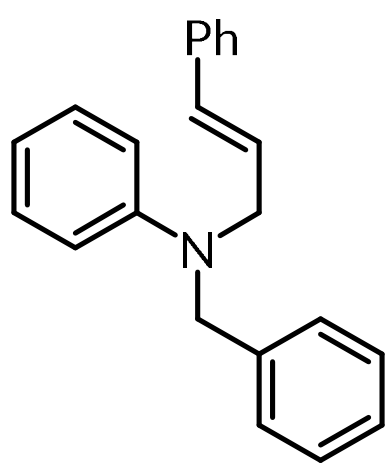

Following general procedure **B**, *N*-benzylaniline (500 mg, 2.72 mmol), cinnamyl bromide (644 mg, 3.27 mmol), NaH (131 mg, 3.27 mmol), and DMF (10 mL), after purification by column chromatography (hexane/CH_2Cl_2, 80:20 → 70:30), gave the title compound **1aa** (743 mg, 91%) as a pale-yellow solid.

mp 57 – 59 °C

ν_{max}(film) / cm^{-1}: 3024, 2860, 2359, 1597, 1504, 1354, 1223, 966, 746, 727, 691

^{1}H NMR (500 MHz, $CDCl_3$) δ_H: 7.40 – 7.26 (9H, m), 7.26 – 7.19 (3H, m), 6.80 (2H, d, *J* 8.6), 6.73 (1H, td, *J* 7.3, 1.4), 6.53 (1H, d, *J* 15.8), 6.34 – 6.25 (1H, m), 4.62 (2H, s), 4.19 (2H, d, *J* 4.6)

$^{13}C\{^1H\}$ NMR (126 MHz, $CDCl_3$) δ_C: 149.1, 139.0, 137.0, 131.5, 129.4, 128.8, 128.7, 127.6, 127.0, 126.8, 126.5, 125.6, 116.8, 112.6, 54.0, 52.7

HRMS: (ESI$^+$) $C_{22}H_{22}N$ $[M+H]^+$ requires 300.1747, found 300.1744 (-1.0 ppm)

_N_-(4-Methoxybenzyl)aniline (SI-01)

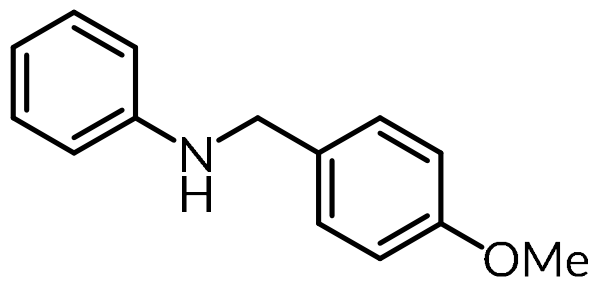

Following general procedure **A**, aniline (0.49 mL, 5.37 mmol), 4-methoxybenzaldehyde (0.65 mL, 5.37 mmol), acetic acid (0.31 mL, 5.36 mmol), sodium triacetoxyborohydride (2.28 g, 10.74 mmol) and CH_2Cl_2 (17.9 mL), after purification by column chromatography (hexane/CH_2Cl_2, 80:20 → 70:30 → 60:40), gave the title compound **SI-01** (965 mg, 84%) as a white solid.

mp 64 – 66 °C

ν_{max}(film) / cm^{-1}: 3418 (N–H), 3049, 2835, 1603, 1508, 1246, 1177, 1034, 824, 750, 692

^1H NMR (500 MHz, $CDCl_3$) δ_H: 7.30 (2H, d, *J* 8.6), 7.22 – 7.16 (2H, m), 6.89 (2H, d, *J* 8.6), 6.75 – 6.70 (1H, m), 6.65 (2H, d, *J* 7.5), 4.26 (2H, s), 3.99 (1H, br s), 3.81 (3H, s)

$^{13}C\{^1H\}$ NMR (126 MHz, $CDCl_3$) δ_C: 159.0, 148.3, 131.5, 129.4, 129.0, 117.7, 114.2, 113.0, 55.4, 48.0

HRMS: (ESI$^+$) $C_{14}H_{16}NO$ $[M+H]^+$ requires 214.1226, found 214.1228 (+0.9 ppm)

_N_-Cinnamyl-_N_-(4-methoxybenzyl)aniline (1ab)

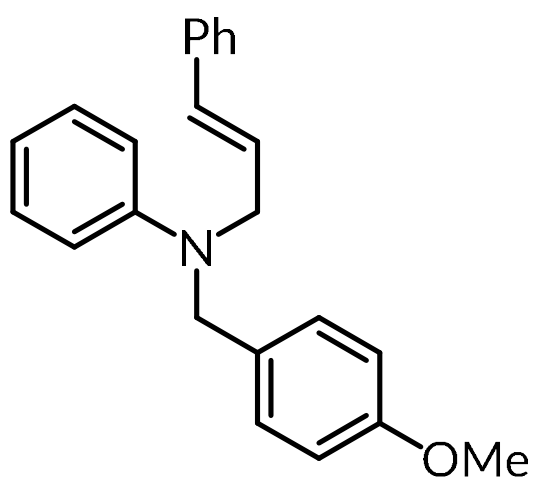

Following general procedure **B**, **SI-01** (500 mg, 2.34 mmol), cinnamyl bromide (553 mg, 2.80 mmol), NaH (112 mg, 2.80 mmol), and DMF (10 mL), after purification by column chromatography (hexane/CH_2Cl_2, 90:10 → 80:20 → 70:30), gave the title compound **1ab** (521 mg, 68%) as a colourless oil.

ν_{max} (film) / cm^{-1}: 3024, 2833, 1590, 1506, 1356, 1246, 1171, 1036, 746

^{1}H NMR (400 MHz, CDCl$_3$) δ_H: 7.37 – 7.28 (4H, m), 7.25 – 7.17 (5H, m), 6.91 – 6.83 (2H, m), 6.86 – 6.76 (2H, m), 6.75 – 6.69 (1H, m), 6.51 (1H, dt, *J* 15.9, 1.7), 6.27 (1H, dt, *J* 15.9, 5.3), 4.54 (2H, s), 4.15 (2H, dd, *J* 5.3, 1.7), 3.80 (3H, s)

^{13}C{^{1}H} NMR (101 MHz, CDCl$_3$) δ_C: 158.7, 149.2, 137.0, 131.5, 130.9, 129.4, 128.7, 128.0, 127.6, 126.5, 125.8, 116.7, 114.2, 112.7, 55.4, 53.4, 52.5

HRMS: (ESI$^+$) $C_{23}H_{23}NONa$ [M+Na]$^+$ requires 352.1672, found 352.1678 (+1.7 ppm)

***N*-(4-Fluorobenzyl)aniline (SI-02)**

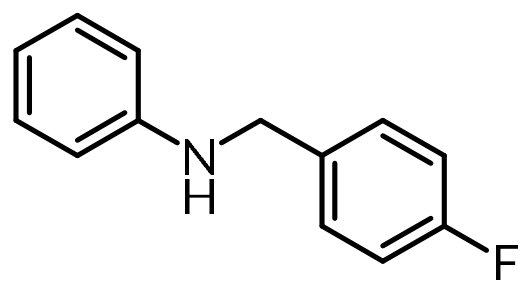

Following general procedure **A**, aniline (0.49 mL, 5.37 mmol), 4-fluorobenzaldehyde (0.58 mL, 5.37 mmol), acetic acid (0.31 mL, 5.36 mmol), sodium triacetoxyborohydride (2.28 g, 10.74 mmol) and CH_2Cl_2 (17.9 mL), after purification by column chromatography (hexane/CH_2Cl_2, 80:20 → 70:30 → 60:40), gave the title compound **SI-02** (969 mg, 90%) as a colourless oil.

ν_{max} (film) / cm^{-1}: 3418 (N–H), 3051, 2849, 1603, 1506, 1221, 825, 750, 692

^{1}H NMR (500 MHz, CDCl$_3$) δ_H: 7.38 – 7.31 (2H, m), 7.22 – 7.16 (2H, m), 7.09 – 7.00 (2H, m), 6.80 – 6.71 (1H, m), 6.64 (2H, d, *J* 7.8), 4.31 (2H, s), 4.02 (1H, br s)

^{13}C{^{1}H} NMR (126 MHz, CDCl$_3$) δ_C: 162.2 (d, *J* 245.0), 148.1, 135.2 (d, *J* 2.9), 129.4, 129.1 (d, *J* 8.0), 117.9, 115.6 (d, *J* 21.4), 113.0, 47.7

^{19}F{^{1}H} NMR (377 MHz, CDCl$_3$) δ_F: -115.6 (s)

HRMS: (ESI$^+$) $C_{13}H_{13}FN$ [M+H]$^+$ requires 202.1027, found 202.1028 (+0.5 ppm)

***N*-Cinnamyl-*N*-(4-fluorobenzyl)aniline (1ac)**

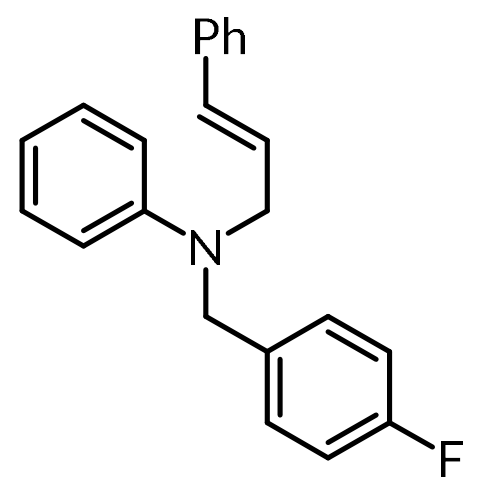

Following general procedure **B**, **SI-02** (500 mg, 2.48 mmol), cinnamyl bromide (587 mg, 2.97 mmol), NaH (119 mg, 2.97 mmol), and DMF (10 mL), after purification by column chromatography (hexane/CH_2Cl_2, 90:10 → 80:20 → 70:30), gave the title compound **1ac** (633 mg, 80%) as a colourless oil.

ν_{max} (film) / cm^{-1}: 3026, 2862, 1597, 1504, 1219, 1153, 746, 692

^{1}H NMR (400 MHz, $CDCl_3$) δ_H: 7.41 – 7.31 (4H, m), 7.29 – 7.21 (5H, m), 7.08 – 7.01 (2H, m), 6.90 – 6.73 (3H, m), 6.55 (1H, dt, *J* 16.0, 1.7), 6.31 (1H, dt, *J* 15.9, 5.3), 4.59 (2H, s), 4.19 (2H, d, *J* 5.4)

^{13}C{^{1}H} NMR (101 MHz, $CDCl_3$) δ_C: 162.0 (d, *J* 244.5), 149.0, 136.9, 134.6 (d, *J* 2.9), 131.7, 129.4, 128.7, 128.3 (d, *J* 7.9), 127.7, 126.5, 125.5, 117.0, 115.6 (d, *J* 21.4), 112.8, 53.4, 52.7

^{19}F{^{1}H} NMR (377 MHz, $CDCl_3$) δ_F: -116.2 (s)

HRMS: (ESI$^+$) $C_{22}H_{21}FN$ $[M+H]^+$ requires 318.1653, found 318.1650 (-0.9 ppm)

***N*-(4-(Trifluoromethyl)benzyl)aniline (SI-03)**

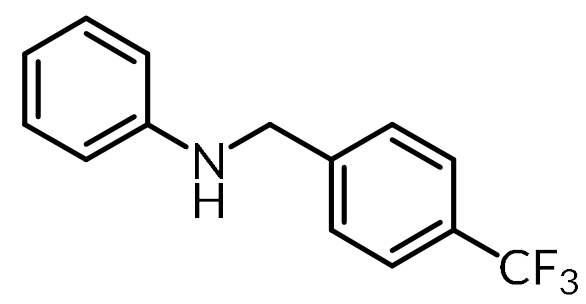

Following general procedure **A**, aniline (0.49 mL, 5.37 mmol), 4-(trifluoromethyl)benzaldehyde (0.73 mL, 5.37 mmol), acetic acid (0.31 mL, 5.36 mmol), sodium triacetoxyborohydride (2.28 g, 10.74 mmol) and CH_2Cl_2 (17.9 mL), after purification by column chromatography (hexane/CH_2Cl_2, 80:20 → 70:30 → 60:40), gave the title compound **SI-03** (1.21 g, 90%) as a colourless oil.

ν_{max}(film) / cm^{-1}: 3420 (N–H), 3053, 1603, 1506, 1323, 1161, 1119, 1065, 1018, 829, 750

^{1}H NMR (500 MHz, $CDCl_3$) δ_H: 7.60 (2H, d, *J* 8.0), 7.49 (2H, d, *J* 8.0), 7.21 – 7.15 (2H, m), 6.75 (1H, t, *J* 7.3), 6.62 (2H, d, *J* 7.6), 4.42 (2H, s), 4.15 (1H, br s)

$^{13}C\{^1H\}$ NMR (126 MHz, $CDCl_3$) δ_C: 147.8, 143.9, 129.6 (q, *J* 32.6), 129.5, 127.6, 125.7 (q, *J* 3.8), 124.3 (q, *J* 272.0), 118.1, 113.0, 47.9

$^{19}F\{^1H\}$ NMR (377 MHz, $CDCl_3$) δ_F: -62.4 (s)

HRMS: (ESI$^+$) $C_{14}H_{13}F_3N$ $[M+H]^+$ requires 252.0995, found 252.0995 (0.0 ppm)

***N*-Cinnamyl-*N*-(4-(trifluoromethyl)benzyl)aniline (1ad)**

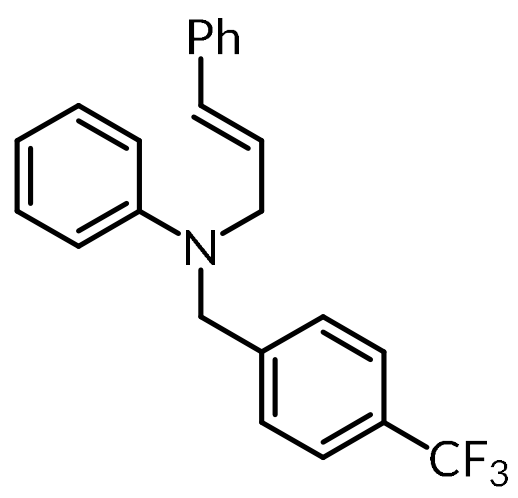

Following general procedure **B**, **SI-03** (600 mg, 2.38 mmol), cinnamyl bromide (565 mg, 2.86 mmol), NaH (114 mg, 2.86 mmol), and DMF (10 mL), after purification by column chromatography (hexane/CH_2Cl_2, 90:10 → 80:20 → 70:30), gave the title compound **1ad** (624 mg, 71%) as a pale-yellow solid.

mp 103 – 105 °C

ν_{max} (film) / cm^{-1}: 3026, 1599, 1504, 1323, 1161, 1121, 1067, 1016, 746, 692

^1H NMR (400 MHz, $CDCl_3$) δ_H: 7.59 (2H, d, *J* 7.9), 7.44 – 7.28 (6H, m), 7.27 – 7.19 (4H, m), 6.77 (2H, d, *J* 8.0), 6.54 (1H, d, *J* 15.9), 6.29 (1H, dt, *J* 15.9, 5.4), 4.65 (2H, s), 4.19 (2H, d, *J* 5.0)

$^{13}C\{^1H\}$ NMR (126 MHz, $CDCl_3$) δ_C: 148.8, 143.4, 136.8, 131.9, 129.5, 129.4 (q, *J* 32.6), 128.7, 127.8, 127.0, 126.5, 125.7 (q, *J* 3.8), 125.2, 124.4 (q, *J* 271.9), 117.3, 112.8, 53.9, 52.9

$^{19}F\{^1H\}$ NMR (377 MHz, $CDCl_3$) δ_F: -62.3 (s)

HRMS: (ESI$^+$) $C_{23}H_{19}F_3N$ $[M-H]^+$ requires 366.1464, found 366.1465 (+0.3 ppm)

N-Cinnamyl-N-(naphthalen-1-ylmethyl)aniline (1ae)

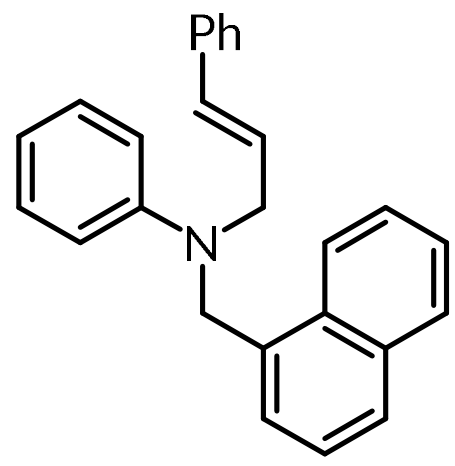

Following general procedure **C**, **3a** (500 mg, 2.39 mmol), 1-(bromomethyl)naphthalene (581 mg, 2.63 mmol), K_2CO_3 (363 mg, 2.63 mmol), and acetone (9.6 mL), after purification by column chromatography (hexane/CH_2Cl_2, 80:20 → 70:30), gave the title compound **1ae** (593 mg, 71%) as a pale-yellow solid.

mp 85 – 87 °C

ν_{max}(film) / cm^{-1}: 3059, 1597 (C=C), 1504, 1381, 1225, 966, 908, 795, 733, 692

^{1}H NMR (500 MHz, $CDCl_3$) δ_H: 8.12 – 8.06 (1H, m), 8.04 – 7.99 (1H, m), 7.92 – 7.86 (1H, m), 7.67 – 7.60 (2H, m), 7.54 – 7.45 (4H, m), 7.44 – 7.38 (2H, m), 7.36 – 7.30 (3H, m), 6.91 (2H, d, *J* 8.3), 6.86 (1H, t, *J* 7.3), 6.66 (1H, dd, *J* 15.9, 1.8), 6.44 (1H, dt, *J* 15.9, 5.2), 5.15 (2H, s), 4.33 (2H, dd, *J* 5.3, 1.8)

^{13}C{^{1}H} NMR (126 MHz, $CDCl_3$) δ_C: 149.1, 136.9, 134.0, 132.9, 131.5, 131.3, 129.4, 129.0, 128.7, 127.6 (2C), 126.5, 126.2, 125.83, 125.78, 125.6, 123.8, 122.8, 116.7, 112.5, 52.5, 51.9

Note: 127.6 ppm peak contains two carbon peaks.

HRMS: (ESI$^+$) $C_{26}H_{23}NNa$ [M+Na]$^+$ requires 372.1723, found 372.1725 (+0.5 ppm)

N-(Thiophen-2-ylmethyl)aniline (SI-04)

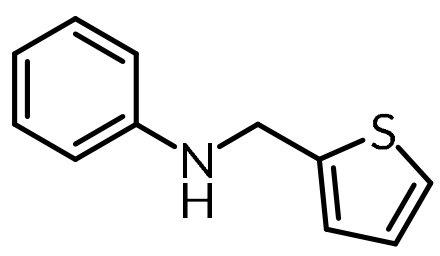

Following general procedure **A**, aniline (0.49 mL, 5.37 mmol), 2-thiophencarboxaldehyde (0.50 mL, 5.37 mmol), acetic acid (0.31 mL, 5.36 mmol), sodium triacetoxyborohydride (2.28 g, 10.74 mmol) and CH_2Cl_2 (17.9 mL), after purification by column chromatography (hexane/CH_2Cl_2, 80:20 → 70:30 → 60:40), gave the title compound **SI-04** (898 mg, 88%) as a colourless oil.

ν_{max}(film) / cm^{-1}: 3410 (N–H), 3049, 1601, 1504, 1312, 1256, 750, 692

^{1}H NMR (500 MHz, $CDCl_3$) δ_H: 7.24 – 7.17 (3H, m), 7.07 – 7.01 (1H, m), 7.00 – 6.96 (1H, m), 6.80 – 6.73 (1H, m), 6.71 – 6.68 (2H, m), 4.53 (2H, s), 4.05 (1H, br s)

$^{13}C\{^1H\}$ NMR (126 MHz, $CDCl_3$) δ_C: 147.7, 143.1, 129.4, 127.0, 125.2, 124.7, 118.2, 113.3, 43.6

HRMS: (ESI^+) $C_{11}H_{11}NSNa$ $[M+Na]^+$ requires 212.0504, found 212.0505 (+0.5 ppm)

_N_-Cinnamyl-_N_-(thiophen-2-ylmethyl)aniline (1af)

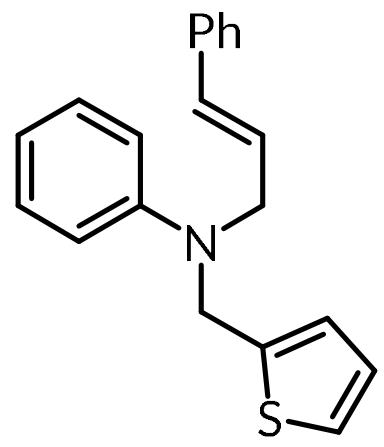

Following general procedure **B**, **SI-04** (500 mg, 2.64 mmol), cinnamyl bromide (624 mg, 3.17 mmol), NaH (127 mg, 3.17 mmol,), and DMF (10 mL), after purification by column chromatography (hexane/CH_2Cl_2, 90:10 → 80:20 → 70:30), gave the title compound **1af** (790 mg, 98%) as a pale-yellow solid.

mp 71 – 73 °C

ν_{max} (film) / cm^{-1}: 3024, 1597, 1504, 1367, 1219, 968, 746, 691

^1H NMR (400 MHz, $CDCl_3$) δ_H: 7.41 – 7.30 (4H, m), 7.30 – 7.18 (4H, m), 7.02 – 6.94 (2H, m), 6.92 – 6.86 (2H, m), 6.81 – 6.74 (1H, m), 6.56 (1H, dt, *J* 15.9, 1.7), 6.30 (1H, dt, *J* 15.9, 5.4), 4.74 (2H, s), 4.17 (2H, dd, *J* 5.4, 1.7)

$^{13}C\{^1H\}$ NMR (101 MHz, $CDCl_3$) δ_C: 148.7, 142.8, 137.0, 131.8, 129.4, 128.7, 127.6, 126.9, 126.5, 125.8, 124.9, 124.4, 117.5, 113.4, 52.5, 49.7

HRMS: (ESI^+) $C_{20}H_{20}NS$ $[M+H]^+$ requires 306.1311, found 306.1308 (-1.0 ppm)

_N_-Cinnamyl-_N_-methylaniline (1ag)

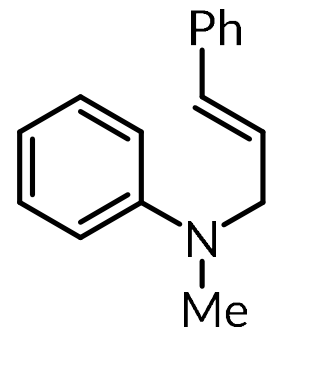

Following general procedure **C**, *N*-methylaniline (0.51 mL, 4.67 mmol), cinnamyl bromide (1.01 g, 5.13 mmol), K_2CO_3 (709 mg, 5.13 mmol), and acetone (18.7 mL), after purification by column chromatography (hexane/CH_2Cl_2, 80:20 → 65:35), gave the title compound **1ag** (951 mg, 91%) as a brown oil.

ν_{max}(film) / cm^{-1}: 3024, 2891, 1597, 1504, 1354, 1119, 964, 748, 691

^{1}H NMR (500 MHz, $CDCl_3$) δ_H: 7.41 (2H, d, *J* 7.2), 7.37 – 7.24 (5H, m), 6.85 (2H, d, *J* 8.2), 6.79 (1H, t, *J* 7.3), 6.57 (1H, dt, *J* 15.7, 1.8), 6.30 (1H, dt, *J* 15.9, 5.5), 4.13 (2H, dd, *J* 5.5, 1.7), 3.03 (3H, s)

^{13}C{^{1}H} NMR (126 MHz, $CDCl_3$) δ_C: 149.7, 137.0, 131.3, 129.3, 128.6, 127.5, 126.4, 125.8, 116.7, 112.7, 55.0, 38.1

HRMS: (ESI$^+$) $C_{16}H_{18}N$ [M+H]$^+$ requires 224.1434, found 224.1433 (-0.4 ppm)

***N*-Cinnamyl-*N*-cyclohexylaniline (1ah)**

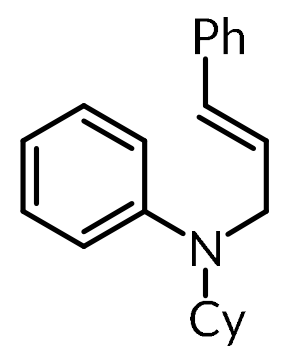

Following general procedure **B**, *N*-cyclohexylaniline (500 mg, 2.85 mmol), cinnamyl bromide (674 mg, 3.42 mmol), NaH (137 mg, 3.42 mmol), and DMF (10 mL), after purification by column chromatography (hexane/EtOAc, 90:10 → 80:20 → 70:30), gave the title compound **1ah** (640 mg, 77%) as a pale-yellow solid.

mp 69 – 71 °C

ν_{max}(film) / cm^{-1}: 2927, 2853, 1593, 1501, 1233, 1163, 962, 745, 729, 688

^{1}H NMR (500 MHz, $CDCl_3$) δ_H: 7.37 – 7.33 (2H, m), 7.32 – 7.27 (2H, m), 7.24 – 7.18 (3H, m), 6.78 (2H, d, *J* 8.0), 6.71 – 6.65 (1H, m), 6.54 (1H, dt, *J* 15.9, 2.0), 6.27 (1H, dt, *J* 16.0, 4.6), 4.01 (2H, dd, *J* 4.7, 2.0), 3.73 – 3.63 (1H, m), 1.99 – 1.89 (2H, m), 1.88 – 1.79 (2H, m), 1.74 – 1.66 (1H, m), 1.48 – 1.32 (4H, m), 1.21 – 1.09 (1H, m)

^{13}C{^{1}H} NMR (126 MHz, $CDCl_3$) δ_C: 149.2, 137.3, 130.2, 129.3, 129.1, 128.6, 127.3, 126.3, 116.1, 112.8, 57.1, 47.5, 30.7, 26.3, 26.1

HRMS: (ESI$^+$) $C_{21}H_{26}N$ [M+H]$^+$ requires 292.2060, found 292.2058 (-0.7 ppm)

***N*-Cinnamyl-*N*-phenylaniline (1ai)**

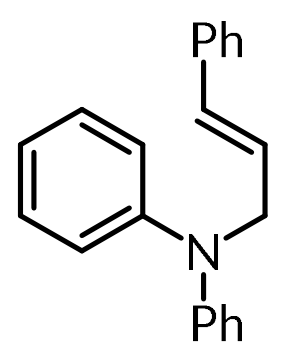

Following general procedure **B**, diphenyl amine (500 mg, 2.96 mmol), cinnamyl bromide (700 mg, 3.55 mmol), NaH (142 mg, 3.55 mmol), and DMF (10 mL), after purification by column

chromatography (hexane/CH_2Cl_2, 80:20 → 70:30), gave the title compound **1ai** (788 mg, 93%) as a yellow oil.

ν_{max}(film) / cm^{-1}: 3057, 3024, 1587, 1493, 1366, 964, 746, 692

^{1}H NMR (500 MHz, $CDCl_3$) δ_H: 7.37 – 7.34 (2H, m), 7.32 – 7.25 (6H, m), 7.25 – 7.19 (1H, m), 7.12 – 7.07 (4H, m), 6.97 (2H, td, *J* 7.3, 1.2), 6.60 (1H, dt, *J* 16.0, 1.9), 6.35 (1H, dt, *J* 15.9, 5.2), 4.54 (2H, dd, *J* 5.2, 1.8)

^{13}C{^{1}H} NMR (126 MHz, $CDCl_3$) δ_C: 148.0, 137.0, 131.4, 129.4, 128.6, 127.5, 126.4, 126.3, 121.5, 120.9, 54.5

HRMS: (ESI$^+$) $C_{21}H_{19}NNa$ [M+Na]$^+$ requires 308.1410, found 308.1408 (-0.6 ppm)

_N_-Allyl-_N_-cinnamylaniline (1aj)

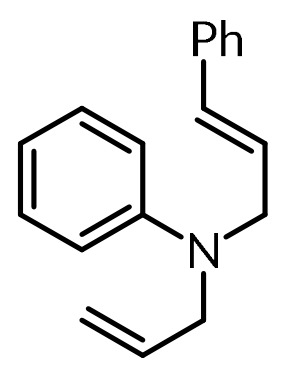

Following general procedure **B**, *N*-allylaniline (500 mg, 3.75 mmol), cinnamyl bromide (888 mg, 4.50 mmol), NaH (180 mg, 4.50 mmol), and DMF (10 mL), after purification by column chromatography (hexane/CH_2Cl_2, 90:10 → 80:20 → 70:30), gave the title compound **1aj** (803 mg, 86%) as a colourless oil.

ν_{max}(film) / cm^{-1}: 3059, 3024, 2909, 1597, 1504, 1356, 1231, 966, 756, 733, 691

^{1}H NMR (500 MHz, $CDCl_3$) δ_H: 7.41 – 7.36 (2H, m), 7.35 – 7.30 (2H, m), 7.27 – 7.20 (3H, m), 6.79 (2H, d, *J* 7.9), 6.73 (1H, t, *J* 7.2), 6.54 (1H, dt, *J* 15.9, 1.8), 6.28 (1H, dt, *J* 15.9, 5.3), 5.91 (1H, ddt, *J* 17.2, 10.0, 4.8), 5.27 – 5.18 (2H, m), 4.11 (2H, dd, *J* 5.3, 1.7), 4.00 (2H, d, *J* 4.9)

^{13}C{^{1}H} NMR (126 MHz, $CDCl_3$) δ_C: 148.9, 137.0, 134.1, 131.1, 129.3, 128.7, 127.5, 126.4, 126.1, 116.5, 116.2, 112.5, 52.8, 52.4

HRMS: (ESI$^+$) $C_{18}H_{20}N$ [M+H]$^+$ requires 250.1590, found 250.1589 (-0.4 ppm)

***N,N*-Dicinnamylaniline (1ak)**

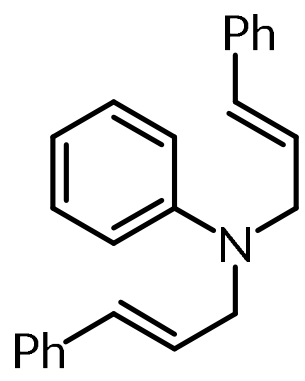

Following general procedure **B**, aniline (0.49 mL, 5.37 mmol), cinnamyl bromide (2.54 g, 12.89 mmol), NaH (515 mg, 12.89 mmol), and DMF (20 mL), after purification by column chromatography (hexane/CH_2Cl_2, 90:10 → 80:20 → 70:30), gave the title compound **1ak** (568 mg, 33%) as a pale-yellow solid.

mp 73 – 75 °C

ν_{max}(film) / cm^{-1}: 3024, 1676, 1597, 1504, 1356, 966, 746, 731, 691

^{1}H NMR (500 MHz, $CDCl_3$) δ_H: 7.38 – 7.35 (4H, m), 7.33 – 7.29 (4H, m), 7.27 – 7.20 (4H, m), 6.85 – 6.82 (2H, m), 6.76 – 6.71 (1H, m), 6.55 (2H, dd, *J* 15.9, 1.7), 6.29 (2H, dt, *J* 15.9, 5.2), 4.15 (4H, dd, *J* 5.2, 1.7)

^{13}C{^{1}H} NMR (126 MHz, $CDCl_3$) δ_C: 149.0, 137.0, 131.3, 129.4, 128.7, 127.6, 126.5, 126.0, 116.7, 112.7, 52.3

HRMS: (ESI$^+$) $C_{24}H_{24}N$ [M+H]$^+$ requires 326.1903, found 326.1902 (-0.3 ppm)

***N*-Cinnamyl-3,5-dimethoxyaniline (3g)**

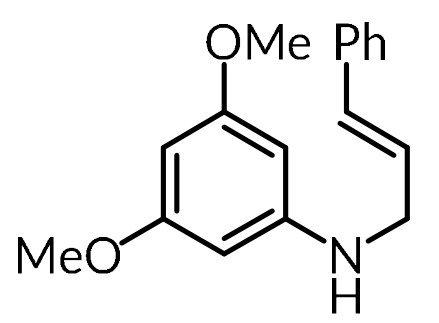

Following general procedure **A**, 3,5-dimethoxy aniline (500 mg, 3.26 mmol), *trans*-cinnamaldehyde (0.41 mL, 3.26 mmol), acetic acid (0.19 mL, 3.26 mmol), sodium triacetoxyborohydride (1.38 g, 6.53 mmol) and CH_2Cl_2 (10.9 mL), after purification by column chromatography (hexane/CH_2Cl_2, 80:20 → 70:30 → 60:40), gave the title compound **3g** (772 mg, 88%) as a white solid.

mp 68 – 70 °C

ν_{max}(film) / cm^{-1}: 3406, 2936, 2839, 1614, 1593, 1202, 1175, 1150, 1069, 810

^{1}H NMR (500 MHz, CDCl$_3$) δ$_H$: 7.40 – 7.35 (2H, m), 7.34 – 7.29 (2H, m), 7.26 – 7.21 (1H, m), 6.62 (1H, dt, *J* 15.8, 1.6), 6.32 (1H, dt, *J* 15.9, 5.7), 5.91 (1H, t, *J* 2.2), 5.87 (2H, d, *J* 2.1), 3.94 – 3.85 (3H, m), 3.76 (6H, s)

^{13}C{^{1}H} NMR (126 MHz, CDCl$_3$) δ$_C$: 161.9, 150.1, 137.0, 131.8, 128.7, 127.7, 127.0, 126.5, 92.0, 90.1, 55.3, 46.4

HRMS: (ESI$^+$) $C_{17}H_{19}NO_2Na$ [M+Na]$^+$ requires 292.1308, found 292.1306 (-0.7 ppm)

***N*-Cinnamyl-*N*-(3,5-dimethoxyphenyl)-4-methylbenzenesulfonamide (1al)**

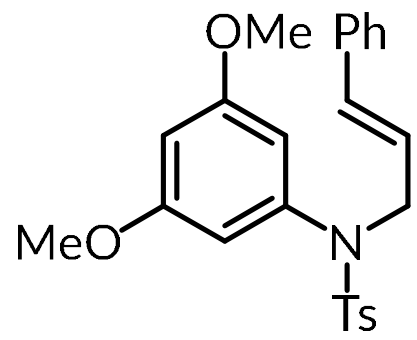

To a solution of **3g** (500 mg, 1.85 mmol) in CH_2Cl_2 (20 mL) was added tosyl chloride (423 mg, 2.22 mmol) and triethylamine (0.31 mL, 2.22 mmol) and stirred at RT for 16 h. The reaction mixture was quenched with H_2O (20 mL). The layers were separated and the aqueous layer was extracted with CH_2Cl_2 (× 3). The combined organic layers were washed with brine, dried over Na_2SO_4, filtered, and concentrated under reduced pressure. Purification by column chromatography (hexane/EtOAc, 80:20 → 70:30 → 60:40) gave the title compound **1al** (375 mg, 48%) as a white solid.

mp 128 – 130 °C

ν_{max}(film) / cm^{-1}: 2959, 2839, 1593, 1344, 1206, 1155, 1067, 692, 663, 548

^{1}H NMR (500 MHz, CDCl$_3$) δ$_H$: 7.61 – 7.54 (2H, m), 7.30 – 7.18 (7H, m), 6.40 (1H, dt, *J* 15.8, 1.4), 6.36 (1H, t, *J* 2.3), 6.25 (2H, d, *J* 2.3), 6.11 (1H, dt, *J* 15.8, 6.6), 4.29 (2H, dd, *J* 6.6, 1.4), 3.69 (6H, s), 2.43 (3H, s)

^{13}C{^{1}H} NMR (126 MHz, CDCl$_3$) δ$_C$: 160.7, 143.6, 141.2, 136.5, 135.9, 133.8, 129.6, 128.7, 128.0, 127.9, 126.6, 124.3, 107.2, 100.3, 55.5, 53.5, 21.7

HRMS: (ESI$^+$) $C_{24}H_{25}NO_4SNa$ [M+Na]$^+$ requires 446.1397, found 446.1395 (-0.4 ppm)

_N_-(3,5-Dimethoxyphenyl)cinnamamide (SI-05)

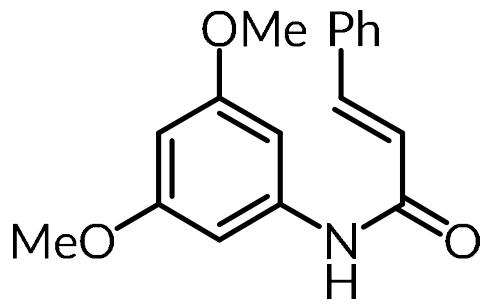

Following a procedure by Zhao and coworkers.[4] To a solution of 3,5-dimethoxyaniline (0.77 g, 5.00 mmol) and K_2CO_3 (1.04 g, 7.5 mmol) in a mixture of acetone (5 mL) and water (10 mL) was added cinnamoyl chloride (1.00 g, 6.00 mmol) at 0 °C. After stirring at 0 °C for 1.5 h, water was added and the mixture was extracted into EtOAc, dried over Na_2SO_4 and concentrated under reduced pressure to give the title compound **SI-05** (1.32 g, 93%) as a white solid.

mp 142 – 145 °C

ν_{max}(film) / cm^{-1}: 3277 (N–H), 1661 (C=O), 1612 (C=C), 1601, 1553, 1450, 1420, 1337, 1206, 1153, 1069

^{1}H NMR (500 MHz, CDCl$_3$) δ_H: 7.75 (1H, d, *J* 15.5), 7.55 – 7.46 (3H, m), 7.41 – 7.33 (3H, m), 6.90 (2H, s), 6.55 (1H, d, *J* 15.5), 6.26 (1H, t, *J* 2.2), 3.78 (6H, s)

^{13}C{^{1}H} NMR (126 MHz, CDCl$_3$) δ_C: 164.2, 161.2, 142.8, 139.9, 134.7, 130.2, 129.0, 128.1, 120.9, 98.3, 97.1, 55.5

HRMS: (ESI$^+$) $C_{17}H_{17}NO_3Na$ [M+Na]$^+$ requires 306.1101, found 306.1099 (-0.7 ppm)

_N_-Benzyl-_N_-(3,5-dimethoxyphenyl)cinnamamide (1am)

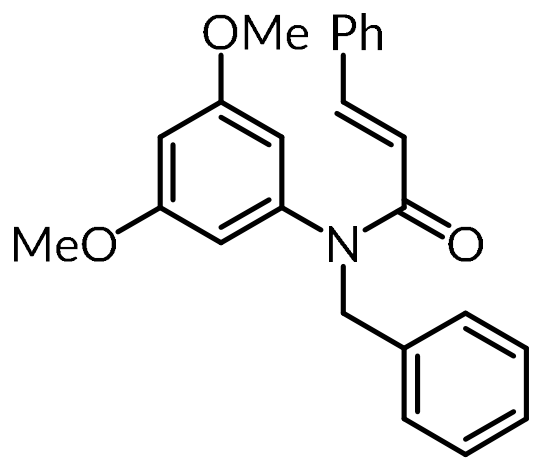

Following a procedure by Zhao and coworkers.[5] To a solution of **SI-05** (0.70 g, 2.50 mmol) in dry THF (8 mL) at 0 °C was added NaH (0.13 g, 3.25 mmol, 60% in mineral oil). After stirring for 1 h at RT, benzyl bromide (0.33 mL, 2.75 mmol) was added dropwise and the mixture stirred at RT for 16 hours. The reaction was quenched with water extracted with EtOAc (× 3), washed with brine, dried over Na_2SO_4, filtered and concentrated under reduced pressure. Purification via column chromatography (hexane/EtOAc, 90:10 → 80:20 → 70:30 → 50:50) gave the title compound **1am** (0.77 g, 82%) as a yellow solid.

mp 160 – 163 °C

ν_{max}(film) / cm^{-1}: 1653 (C=O), 1591, 1454, 1427, 1389, 1339, 1206, 1155, 1064, 1032, 763, 702

^{1}H NMR (500 MHz, CDCl$_3$) δ_H: 7.75 (1H, d, *J* 15.5), 7.37 – 7.32 (2H, m), 7.32 – 7.27 (7H, m), 7.26 – 7.22 (1H, m), 6.43 (1H, t, *J* 2.3), 6.43 (1H, d, *J* 15.6), 6.20 (2H, d, *J* 2.2), 5.00 (2H, s), 3.70 (6H, s)

^{13}C{^{1}H} NMR (126 MHz, CDCl$_3$) δ_C: 166.0, 161.2, 143.8, 142.4, 137.8, 135.3, 129.7, 128.9, 128.8, 128.5, 128.1, 127.5, 119.0, 106.8, 100.2, 55.6, 53.3

HRMS: (ESI$^+$) $C_{24}H_{23}NO_3Na$ [M+Na]$^+$ requires 396.1570, found 396.1569 (-0.3 ppm)

(*E*)-*N*-Benzyl-*N*-(3-(*p*-tolyl)allyl)aniline (1an)

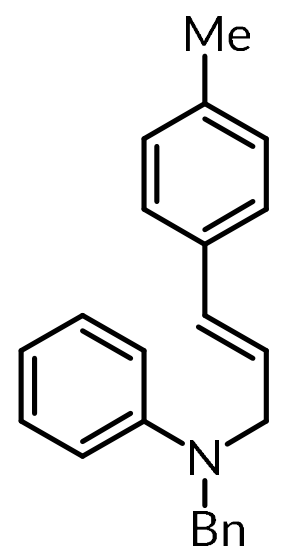

Following general procedure **A**, *N*-benzylaniline (0.92 g, 5.00 mmol), (*E*)-3-(p-tolyl)acrylaldehyde (0.73 g, 5.00 mmol), acetic acid (0.29 mL, 5.00 mmol), sodium triacetoxyborohydride (2.12 g, 10.00 mmol) and CH_2Cl_2 (16.7 mL), after purification by column chromatography (hexane/EtOAc, 100:0 → 99:1 → 98:2), gave the title compound **1an** (0.95 g, 60%) as a colourless oil.

ν_{max}(film) / cm^{-1}: 3024 (C–H), 1597 (C=C), 1504, 1354, 1225, 968

^{1}H NMR (500 MHz, CDCl$_3$) δ_H: 7.38 – 7.32 (2H, m), 7.31 – 7.25 (5H, m), 7.25 – 7.20 (2H, m), 7.13 (2H, d, *J* 7.9), 6.80 (2H, d, *J* 8.0), 6.73 (1H, t, *J* 7.3), 6.51 (1H, d, *J* 15.9), 6.25 (1H, dt, *J* 15.9, 5.4), 4.62 (2H, s), 4.18 (2H, d, *J* 4.5), 2.35 (3H, s)

^{13}C{^{1}H} NMR (126 MHz, CDCl$_3$) δ_C: 149.2, 139.1, 137.4, 134.2, 131.4, 129.4 (2C), 128.7, 127.0, 126.8, 126.4, 124.5, 116.7, 112.6, 53.9, 52.7, 21.3

Note: 129.4 ppm peak contains two carbon peaks.

HRMS: (ESI$^+$) $C_{23}H_{24}N$ [M+H]$^+$ requires 314.1903, found 314.1901 (+0.6 ppm)

(*E*)-*N*-Benzyl-*N*-(3-(4-methoxyphenyl)allyl)aniline (1ao)

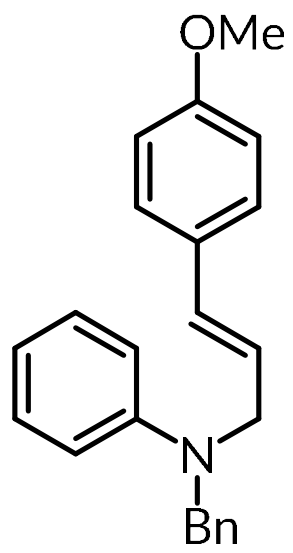

Following general procedure **A**, *N*-benzylaniline (0.92 g, 5.00 mmol), (*E*)-3-(4-methoxyphenyl)acrylaldehyde (0.81 g, 5.00 mmol), acetic acid (0.29 mL, 5.00 mmol), sodium triacetoxyborohydride (2.12 g, 10.00 mmol) and CH_2Cl_2 (16.7 mL), after purification by column chromatography (hexane/CH_2Cl_2/Et_2O, 90:10:0 → 80:20:0 → 99:0:1 → 98:0:2 → 95:0:5), gave the title compound **1ao** (0.51 g, 31%) as an off white solid.

mp 87 – 89 °C

ν_{max}(film) / cm^{-1}: 1595 (C=C), 1491, 1450, 1358, 1244, 1175, 1022

^{1}H NMR (400 MHz, CDCl3) δ_H: 7.42 – 7.11 (9H, m), 6.82 (2H, d, *J* 8.7), 6.77 (2H, d, *J* 8.2), 6.69 (1H, t, *J* 7.3), 6.45 (1H, d, *J* 15.8), 6.12 (1H, dt, *J* 15.9, 5.4), 4.58 (2H, s), 4.14 (2H, d, *J* 5.3), 3.78 (3H, s)

^{13}C{^{1}H} NMR (101 MHz, $CDCl_3$) δ_C: 159.3, 149.2, 139.1, 131.0, 129.8, 129.4, 128.7, 127.6, 127.0, 126.8, 123.3, 116.7, 114.1, 112.6, 55.4, 53.9, 52.7

HRMS: (ESI$^+$) $C_{23}H_{24}NO$ [M+H]$^+$ requires 330.1852, found 330.1850 (-0.6 ppm)

(*E*)-*N*-Benzyl-*N*-(3-(4-fluorophenyl)allyl)aniline (1ap)

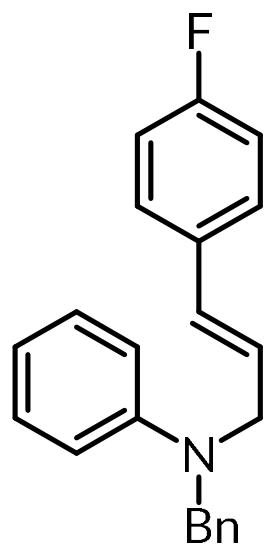

Following general procedure **A**, *N*-benzylaniline (0.92 g, 5.00 mmol), (*E*)-3-(4-fluorophenyl)acrylaldehyde (0.75 g, 5.00 mmol), acetic acid (0.29 mL, 5.00 mmol), sodium triacetoxyborohydride (2.12 g, 10.00 mmol) and CH_2Cl_2 (16.7 mL), after purification by column chromatography (hexane/Et_2O, 100:0 → 99:1 → 98:2), gave the title compound **1ap** (0.51 g, 32%) as white solid.

mp 72 – 75 °C

ν_{max}(film) / cm^{-1}: 3024, 1595, 1504, 1356, 1233, 1217, 1198, 1171, 1153

^{1}H NMR (500 MHz, CDCl$_3$) δ_H: 7.39 – 7.26 (7H, m), 7.24 – 7.19 (2H, m), 7.05 – 6.94 (2H, m), 6.78 (2H, d, *J* 7.8), 6.76 – 6.70 (1H, m), 6.48 (1H, d, *J* 15.9), 6.20 (1H, dt, *J* 15.9, 5.3), 4.61 (2H, s), 4.17 (2H, dd, *J* 5.3, 0.9)

^{13}C{^{1}H} NMR (126 MHz, CDCl$_3$) δ_C: 162.4 (d, *J* 246.6), 149.1, 139.0, 133.1 (d, *J* 3.3), 130.3, 129.4, 128.8, 128.0 (d, *J* 8.0), 127.0, 126.8, 125.4 (d, *J* 2.2), 116.8, 115.6 (d, *J* 21.6), 112.6, 54.0, 52.6

^{19}F{^{1}H} NMR (377 MHz, CDCl$_3$) δ_F: -114.7 (s)

HRMS: (ESI$^+$) $C_{22}H_{21}FN$ [M+H]$^+$ requires 318.1653, found 318.1650 (-0.9 ppm)

(*E*)-*N*-(3-(4-Bromophenyl)allyl)aniline (SI-06)

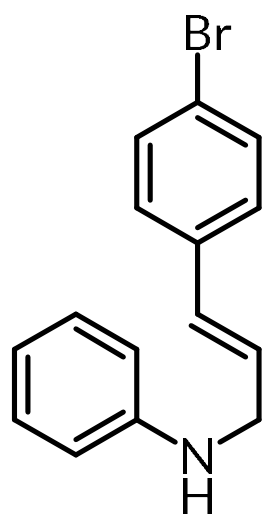

Following general procedure **A**, aniline (0.91 mL, 10.00 mmol), 3-(4-bromophenyl)acrylaldehyde (2.11 g, 10.00 mmol), acetic acid (0.58 mL, 10.00 mmol), sodium triacetoxyborohydride (4.24 g, 20.00 mmol) and CH_2Cl_2 (34.4 mL), after purification by column chromatography (hexane/Et_2O, 100:0 → 99:1 → 98:2 → 95:5), gave the title compound **SI-06** (2.10 g, 73%) as white solid.

mp 67 – 69 °C

ν_{max}(film) / cm^{-1}: 3391 (N–H), 1601 (C=C), 1504, 1314, 1069, 966

^{1}H NMR (700 MHz, CDCl$_3$) δ_H: 7.44 (2H, d, *J* 8.1), 7.25 – 7.17 (4H, m), 6.75 (1H, t, *J* 7.3), 6.68 (2H, d, *J* 7.9), 6.57 (1H, d, *J* 15.9), 6.33 (1H, dt, *J* 15.9, 5.6), 3.94 (2H, d, *J* 5.7), 3.86 (1H, br s)

^{13}C{^{1}H} NMR (176 MHz, CDCl$_3$) δ_C: 148.0, 135.9, 131.8, 130.3, 129.4, 128.1, 128.0, 121.4, 117.9, 113.1, 46.2

HRMS: (ESI$^+$) $C_{15}H_{14}{}^{79}BrN$ [M]$^+$ requires 287.0304, found 287.0304 (0.0 ppm)

(*E*)-*N*-Benzyl-*N*-(3-(4-bromophenyl)allyl)aniline (1aq)

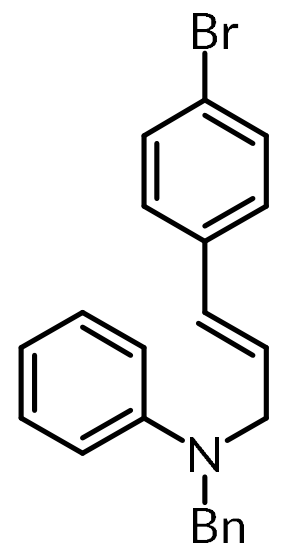

Following general procedure **C**, **SI-06** (0.86 g, 3.00 mmol), benzyl bromide (0.40 mL, 3.30 mmol), K_2CO_3 (0.46 g, 3.30 mmol), and acetone (12.0 mL), after purification by column chromatography (hexane/Et_2O, 100:0 → 99:1 → 98:2), gave the title compound **1aq** (1.10 g, 97%) as a white solid.

mp 62 – 65 °C

ν_{max}(film) / cm^{-1}: 1595, 1503, 1485, 1360, 1219, 1163, 1070, 968

^{1}H NMR (500 MHz, $CDCl_3$) δ_H: 7.43 (2H, d, *J* 8.4), 7.39 – 7.32 (2H, m), 7.30 – 7.26 (3H, m), 7.26 – 7.17 (4H, m), 6.79 (2H, d, *J* 8.2), 6.74 (1H, t, *J* 7.3), 6.47 (1H, d, *J* 15.9), 6.29 (1H, dt, *J* 15.9, 5.2), 4.62 (2H, s), 4.18 (2H, d, *J* 5.0)

^{13}C{^{1}H} NMR (126 MHz, $CDCl_3$) δ_C: 149.0, 138.9, 135.9, 131.8, 130.2, 129.4, 128.8, 128.0, 127.0, 126.8, 126.6, 121.3, 116.9, 112.6, 54.0, 52.6

HRMS: (ESI$^+$) $C_{22}H_{21}{}^{79}BrN$ [M+H]$^+$ requires 378.0852, found 378.0854 (+0.5 ppm)

(*E*)-*N*-Benzyl-*N*-(3-(4-(trifluoromethyl)phenyl)allyl)aniline (1ar)

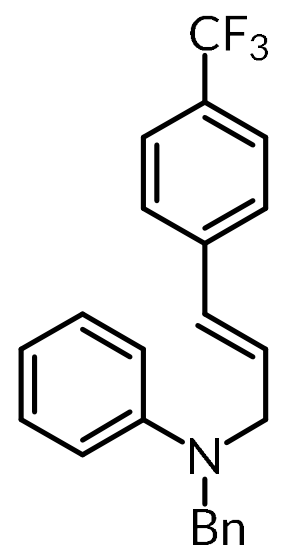

Following general procedure **A**, *N*-benzylaniline (0.18 g, 1.00 mmol), (*E*)-3-(4-(trifluoromethyl)phenyl)acrylaldehyde (0.20 g, 1.00 mmol), acetic acid (0.06 mL, 1.00 mmol), sodium triacetoxyborohydride (0.42 g, 2.00 mmol) and CH_2Cl_2 (3.3 mL), after purification by column chromatography (hexane/Et_2O, 100:0 → 99:1 → 98:2), gave the title compound **1ar** (0.19 g, 51%) as pale yellow oil.

ν_{max}(film) / cm^{-1}: 1597 (C=C), 1504, 1321, 1161, 1117, 1065,

^{1}H NMR (500 MHz, CDCl$_3$) δ_H: 7.54 (2H, d, *J* 8.1), 7.43 (2H, d, *J* 8.1), 7.36 – 7.31 (2H, m), 7.29 – 7.23 (3H, m), 7.24 – 7.19 (2H, m), 6.78 (2H, d, *J* 7.8), 6.73 (1H, t, *J* 7.2), 6.54 (1H, d, *J* 16.1), 6.38 (1H, dt, *J* 16.0, 5.1), 4.61 (2H, s), 4.20 (2H, d, *J* 4.2)

^{13}C{^{1}H} NMR (176 MHz, CDCl$_3$) δ_C: 149.0, 140.4, 138.8, 130.1, 129.5, 129.4 (q, *J* 32.2), 128.8, 128.7, 127.1, 126.8, 126.6, 125.6 (q, *J* 3.8), 124.3 (q, *J* 271.9), 117.0, 112.6, 54.2, 52.6

^{19}F NMR (471 MHz, CDCl$_3$) δ_F: -62.5 (s)

HRMS: (ESI$^+$) $C_{23}H_{21}F_3N$ [M+H]$^+$ requires 368.1621, found 368.1618 (-0.8 ppm)

Methyl (*E*)-3-(3-oxoprop-1-en-1-yl)benzoate (SI-07)

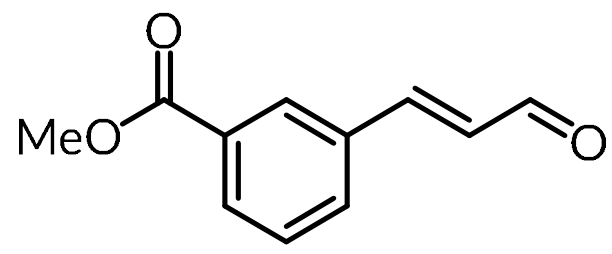

Following general procedure **D**, methyl 3-formylbenzoate (0.82 g, 5.00 mmol), ^{t}BuOK (0.62 g, 5.50 mmol), and (1,3-dioxolan-2-ylmethyl)triphenylphosphanium bromide (2.36 g, 5.50 mmol) in THF (10 mL), after purification by column chromatography (hexane/Et$_2$O, 98:2 → 96:4 → 94:6) gave the title compound **SI-07** (0.36 g, 38%) as a white solid.

mp 90 – 92 °C

ν_{max}(film) / cm^{-1}: 1722 (C=O), 1682 (C=O), 1631 (C=C), 1443, 1319, 1298, 1246, 1202, 1125, 1105

^{1}H NMR (500 MHz, CDCl$_3$) δ_H: 9.74 (1H, d, *J* 7.6), 8.24 (1H, s), 8.10 (1H, dt, *J* 7.8, 1.4), 7.76 (1H, dt, *J* 7.7, 1.5), 7.53 (1H, t, J 7.8), 7.51 (1H, d, *J* 15.9), 6.79 (1H, dd, *J* 16.0, 7.6), 3.95 (3H, s)

^{13}C{^{1}H} NMR (126 MHz, CDCl$_3$) δ_C: 193.5, 166.4, 151.4, 134.5, 132.4, 132.1, 131.3, 129.8, 129.7, 129.4, 52.6

HRMS: (ESI$^+$) $C_{11}H_{10}O_3Na$ [M+Na]$^+$ requires 213.0522, found 213.0522 (0.0 ppm)

Methyl (*E*)-3-(3-(phenylamino)prop-1-en-1-yl)benzoate (SI-08)

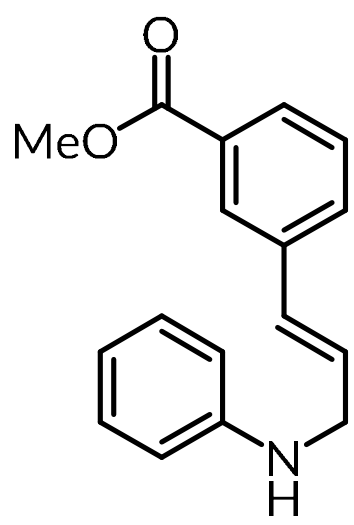

Following general procedure **A**, aniline (0.17 mL, 1.84 mmol), **SI-07** (0.35 g, 1.84 mmol), acetic acid (0.11 mL, 1.84 mmol), sodium triacetoxyborohydride (0.78 g, 3.68 mmol) and CH_2Cl_2 (6.1 mL), after purification by column chromatography (hexane/EtOAc, 98:2 → 95:5 → 92:8 → 90:10), gave the title compound **SI-08** (0.35 g, 72%) as a colourless oil.

ν_{max}(film) / cm^{-1}: 3397 (N–H), 1713 (C=O), 1601 (C=C), 1504, 1431, 1288, 1200, 1105, 964

^{1}H NMR (500 MHz, $CDCl_3$) δ_H: 8.05 (1H, t, *J* 1.8), 7.90 (1H, dt, *J* 7.7, 1.4), 7.55 (1H, dt, *J* 7.7, 1.5), 7.38 (1H, t, *J* 7.7), 7.24 – 7.16 (2H, m), 6.74 (1H, tt, *J* 7.4, 1.1), 6.70 – 6.62 (3H, m), 6.42 (1H, dt, *J* 15.9, 5.6), 3.97 (2H, dd, *J* 5.6, 1.7), 3.92 (3H, s), 3.90 (1H, br. s)

^{13}C{^{1}H} NMR (126 MHz, $CDCl_3$) δ_C: 167.1, 148.0, 137.3, 130.8, 130.6, 130.5, 129.4, 128.8, 128.63, 128.61, 127.5, 117.8, 113.1, 52.3, 46.2

HRMS: (ESI$^+$) $C_{17}H_{17}NO_2Na$ [M+Na]$^+$ requires 290.1151, found 290.1150 (-0.3 ppm)

Methyl (*E*)-3-(3-(benzyl(phenyl)amino)prop-1-en-1-yl)benzoate (1as)

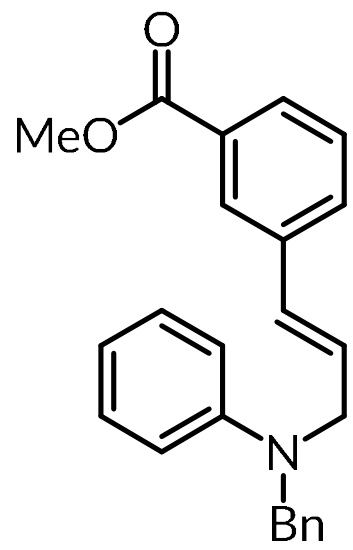

Following general procedure **C**, **SI-08** (0.27 g, 1.00 mmol), benzyl bromide (0.13 mL, 1.10 mmol), K_2CO_3 (0.15 g, 1.10 mmol), and acetone (4.0 mL), after purification by column chromatography (hexane/CH_2Cl_2, 95:5 → 90:10 → 80:20), gave the title compound **1as** (0.32 g, 97%) as a white solid.

mp 76 – 79 °C

ν_{max}(film) / cm^{-1}: 1719 (C=O), 1595 (C=C), 1504, 1429, 1271, 1221, 1194, 1169, 966

^{1}H NMR (500 MHz, $CDCl_3$) δ_H: 8.03 (1H, s), 7.90 (1H, dt, *J* 7.7, 1.4), 7.53 (1H, d, *J* 7.8), 7.40 – 7.32 (3H, m), 7.30 – 7.24 (3H, m), 7.24 – 7.19 (2H, m), 6.79 (2H, d, *J* 7.8), 6.73 (1H, tt, *J* 7.3, 1.0), 6.55 (1H, d, *J* 16.0), 6.37 (1H, dt, *J* 16.0, 5.1), 4.62 (2H, s), 4.20 (2H, dd, *J* 5.2, 1.7), 3.92 (3H, s)

^{13}C{^{1}H} NMR (126 MHz, $CDCl_3$) δ_C: 167.1, 149.0, 138.9, 137.3, 130.8, 130.6, 130.4, 129.4, 128.78, 128.75, 128.6, 127.6, 127.1, 127.0, 126.8, 116.9, 112.6, 54.1, 52.5, 52.3

HRMS: (ESI$^+$) $C_{24}H_{23}NO_2Na$ [M+Na]$^+$ requires 380.1621, found 380.1619 (-0.5 ppm)

(*E*)-3-(3,5-Di-*tert*-butylphenyl)acrylaldehyde (SI-09)

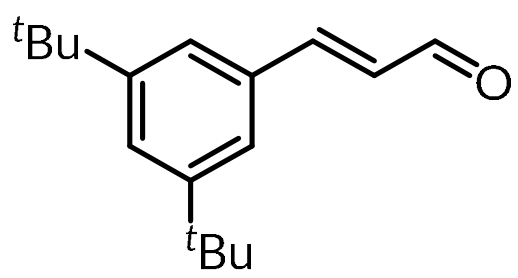

Following general procedure **D**, 3,5-bis(*tert*-butyl)benzaldehyde (1.09 g, 5.00 mmol), ^{n}BuLi (2.2 mL, 5.50 mmol, 2.5 M in hexanes), and (1,3-dioxolan-2-ylmethyl)triphenylphosphanium bromide (2.36 g, 5.50 mmol) in THF (10 mL), after purification by column chromatography (hexane/EtOAc, 99:1 → 98:2) gave the title compound **SI-09** (0.32 g, 26%) as a yellow oil.

ν_{max}(film) / cm^{-1}: 2961 (C–H), 1678 (C=O), 1626 (C=C), 1593, 1476, 1364, 1248, 1121, 970, 700

^{1}H NMR (500 MHz, CDCl$_3$) δ_H: 9.71 (1H, d, *J* 7.7), 7.53 (1H, d, *J* 1.8), 7.52 (1H, d, *J* 16.0), 7.41 (2H, d, *J* 1.8), 6.75 (1H, dd, *J* 15.9, 7.7), 1.35 (18H, s)

^{13}C{^{1}H} NMR (126 MHz, CDCl$_3$) δ_C: 194.0, 154.3, 151.9, 133.5, 128.3, 126.0, 123.0, 35.0, 31.5

HRMS: (ESI$^+$) $C_{17}H_{24}ONa$ [M+Na]$^+$ requires 267.1719, found 267.1716 (-1.1 ppm)

(*E*)-*N*-(3-(3,5-Di-*tert*-butylphenyl)allyl)aniline (SI-10)

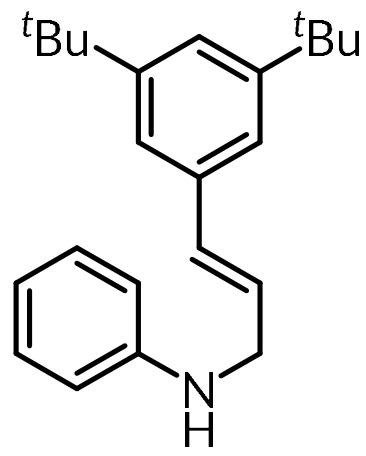

Following general procedure **A**, aniline (0.12 mL, 1.30 mmol), **SI-09** (0.32 g, 1.30 mmol), acetic acid (0.08 mL, 1.30 mmol), sodium triacetoxyborohydride (0.55 g, 2.60 mmol) and CH_2Cl_2 (4.3 mL), after purification by column chromatography (hexane/Et_2O, 100:0 → 99:1 → 98:2), gave the title compound **SI-10** (0.28 g, 57%) as a colourless oil.

ν_{max}(film) / cm^{-1}: 3420 (N–H), 2961, 1601 (C=C), 1504, 1362, 1248, 964, 876, 746, 691

^{1}H NMR (500 MHz, CDCl$_3$) δ_H: 7.33 (1H, t, *J* 1.8), 7.24 (2H, d, *J* 1.8), 7.23 – 7.18 (2H, m), 6.74 (1H, tt, *J* 7.4, 1.1), 6.71 – 6.64 (3H, m), 6.34 (1H, dt, *J* 15.8, 5.8), 3.95 (2H, d, *J* 5.6), 3.85 (1H, br. s), 1.34 (18H, s)

^{13}C{^{1}H} NMR (126 MHz, CDCl$_3$) δ_C: 151.1, 148.3, 136.1, 132.6, 129.4, 126.4, 122.1, 120.8, 117.7, 113.2, 46.5, 35.0, 31.6

HRMS: (ESI$^+$) $C_{23}H_{30}N$ [M-H]$^+$ requires 320.2373, found 320.2372 (-0.3 ppm)

(*E*)-N-Benzyl-*N*-(3-(3,5-di-*tert*-butylphenyl)allyl)aniline (1at)

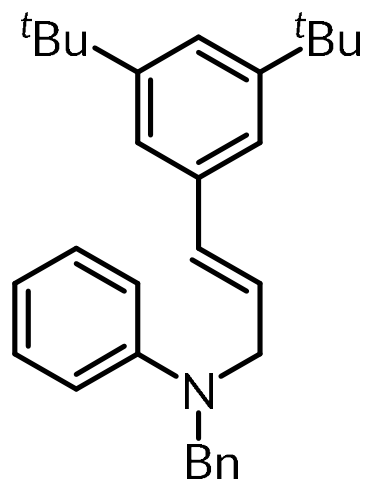

Following general procedure **C**, **SI-10** (0.28 g, 0.86 mmol), benzyl bromide (0.11 mL, 0.95 mmol), K_2CO_3 (0.13 g, 0.95 mmol), and acetone (3.4 mL), after purification by column chromatography (hexane/CH_2Cl_2, 99:1 → 98:2 → 95:5 → 90:10), gave the title compound **1at** (0.30 g, 91%) as a colourless oil.

ν_{max}(film) / cm^{-1}: 2963, 1597, 1506, 1452, 1362, 1248, 964, 746

^{1}H NMR (500 MHz, $CDCl_3$) δ_H: 7.37 – 7.31 (3H, m), 7.31 – 7.24 (3H, m), 7.24 – 7.19 (4H, m), 6.85 – 6.78 (2H, m), 6.72 (1H, tt, *J* 7.3, 1.1), 6.55 (1H, dt, *J* 15.8, 1.7), 6.27 (1H, dt, *J* 15.9, 5.3), 4.63 (2H, s), 4.19 (2H, dd, *J* 5.4, 1.7), 1.33 (18H, s)

^{13}C{^{1}H} NMR (126 MHz, $CDCl_3$) δ_C: 151.1, 149.3, 139.1, 136.1, 132.4, 129.4, 128.8, 127.0, 126.8, 124.8, 122.0, 120.8, 116.6, 112.5, 53.8, 52.7, 35.0, 31.6

HRMS: (ESI$^+$) $C_{30}H_{37}NNa$ [M+Na]$^+$ requires 434.2818, found 434.2815 (-0.7 ppm)

(*E*)-3-(2-Bromo-5-chlorophenyl)acrylaldehyde (SI-11)

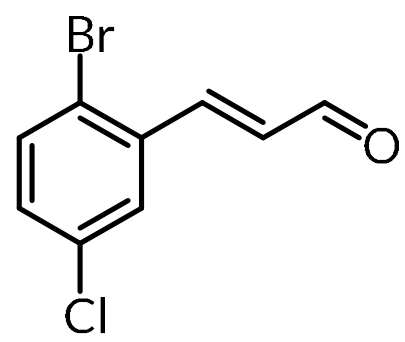

Following general procedure **D**, 2-bromo-5-chlorobenzaldehyde (1.10 g, 5.00 mmol), ^{n}BuLi (2.2 mL, 5.50 mmol, 2.5 M in hexanes), and (1,3-dioxolan-2-ylmethyl)triphenylphosphanium bromide (2.36 g, 5.50 mmol) in THF (10 mL), after purification by column chromatography (hexane/EtOAc, 99:1 → 98:2 → 95:5) gave the title compound **SI-11** (0.41 g, 33%) as a yellow solid.

mp 97 – 100 °C

ν_{max}(film) / cm^{-1}: 1684 (C=O), 1667 (C=C), 1458, 1132, 1109, 1099, 1028, 972

^{1}H NMR (500 MHz, $CDCl_3$) δ_H: 9.78 (1H, d, *J* 7.5), 7.80 (1H, d, *J* 15.9), 7.61 (1H, d, *J* 2.5), 7.59 (1H, d, *J* 8.6), 7.26 (1H, dd, *J* 8.6, 2.5), 6.65 (1H, dd, *J* 16.0, 7.6)

$^{13}C\{^1H\}$ NMR (126 MHz, $CDCl_3$) δ_C: 193.1, 149.1, 135.5, 134.8, 134.3, 132.1, 131.7, 128.0, 123.4

HRMS: (ESI$^+$) $C_9H_7{}^{79}Br^{35}ClO$ [M+H]$^+$ requires 244.9363, found 244.9364 (+0.4 ppm)

(*E*)-*N*-(3-(2-Bromo-5-chlorophenyl)allyl)aniline (SI-12)

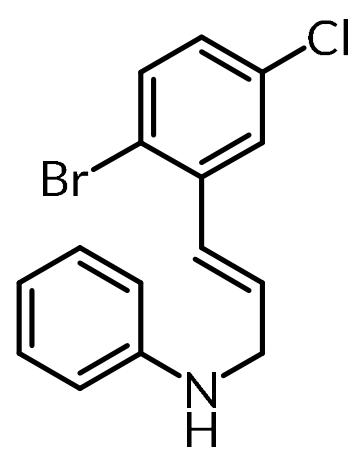

Following general procedure **A**, aniline (0.14 mL, 1.50 mmol), **SI-11** (0.37 g, 1.50 mmol), acetic acid (0.09 mL, 1.50 mmol), sodium triacetoxyborohydride (0.64 g, 3.00 mmol) and CH_2Cl_2 (5.0 mL), after purification by column chromatography (hexane/Et_2O, 100:0 → 99:1 → 98:2), gave the title compound **SI-12** (0.43 g, 88%) as a yellow oil.

ν_{max}(film) / cm^{-1}: 3410 (N–H), 1601 (C=C), 1504, 1456, 1323, 1258, 1094, 1026, 962, 808, 748

^1H NMR (500 MHz, $CDCl_3$) δ_H: 7.47 (1H, d, *J* 2.5), 7.46 (1H, d, *J* 8.5), 7.24 – 7.16 (2H, m), 7.07 (1H, dd, *J* 8.5, 2.5), 6.89 (1H, dt, *J* 15.7, 1.7), 6.75 (1H, tt, *J* 7.3, 1.1), 6.71 – 6.66 (2H, m), 6.28 (1H, dt, *J* 15.8, 5.6), 3.99 (2H, dd, *J* 5.7, 1.7), 3.90 (1H, br. s)

$^{13}C\{^1H\}$ NMR (126 MHz, $CDCl_3$) δ_C: 147.9, 138.5, 134.0, 133.7, 131.9, 129.54, 129.46, 128.8, 127.1, 121.3, 118.0, 113.2, 46.1

HRMS: (ESI$^+$) $C_{15}H_{12}{}^{79}Br^{35}ClN$ [M-H]$^+$ requires 319.9836, found 319.9837 (+0.3 ppm)

(*E*)-*N*-Benzyl-*N*-(3-(2-bromo-5-chlorophenyl)allyl)aniline (1au)

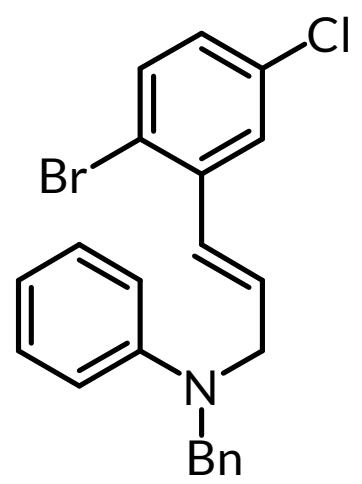

Following general procedure **C**, **SI-12** (0.43 g, 1.32 mmol), benzyl bromide (0.17 mL, 1.45 mmol), K_2CO_3 (0.20 g, 1.45 mmol), and acetone (5.3 mL), after purification by column chromatography (hexane/CH_2Cl_2, 99:1 → 95:5 → 90:10), gave the title compound **1au** (0.43 g, 84%) as a white solid.

mp 104 – 107 °C

ν_{max}(film) / cm^{-1}: 1597 (C=C), 1504, 1452, 1354, 1229, 1094, 1026, 748, 692

^{1}H NMR (500 MHz, CDCl$_3$) δ_H: 7.45 (1H, d, *J* 8.5), 7.41 (1H, d, *J* 2.5), 7.37 – 7.32 (2H, m), 7.31 – 7.25 (3H, m), 7.24 – 7.19 (2H, m), 7.06 (1H, dd, *J* 8.5, 2.5), 6.83 – 6.77 (3H, m), 6.74 (1H, t, *J* 7.3), 6.20 (1H, dt, *J* 15.8, 5.4), 4.61 (2H, s), 4.19 (2H, dd, *J* 5.5, 1.8)

^{13}C{^{1}H} NMR (126 MHz, CDCl$_3$) δ_C: 149.0, 138.9, 138.6, 134.0, 133.6, 130.4, 129.9, 129.4, 128.81, 128.76, 127.2, 127.1, 126.9, 121.3, 117.2, 112.9, 54.3, 52.6

HRMS: (ESI$^+$) $C_{22}H_{20}{}^{81}Br^{35}ClN$ [M+H]$^+$ requires 414.0442, found 414.0440 (0.5 ppm)

(*E*)-3-(2-(Trifluoromethoxy)phenyl)acrylaldehyde (SI-13)

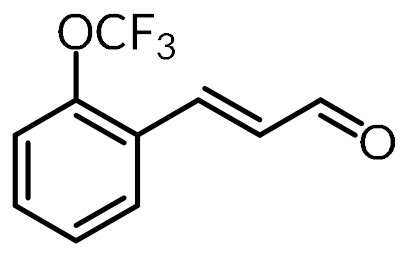

Following general procedure **D**, 2-(trifluoromethoxy)benzaldehyde (0.71 mL, 5.00 mmol), ^{n}BuLi (2.2 mL, 5.50 mmol, 2.5 M in hexanes), and (1,3-dioxolan-2-ylmethyl)triphenylphosphanium bromide (2.36 g, 5.50 mmol) in THF (10 mL), after purification by column chromatography (hexane/EtOAc, 99:1 → 98:2) gave the title compound **SI-13** (0.63 g, 58%) as an orange oil.

ν_{max}(film) / cm^{-1}: 1680 (C=O), 1626 (C=C), 1487, 1244, 1206, 1155, 1119, 1090, 974, 924, 758

^{1}H NMR (500 MHz, CDCl$_3$) δ_H: 9.75 (1H, d, *J* 7.6), 7.75 (1H, d, *J* 16.1), 7.71 (1H, dd, *J* 7.8, 1.7), 7.52 – 7.45 (1H, m), 7.37 (1H, t, *J* 7.8), 7.34 (1H, dt, *J* 8.5, 1.5), 6.75 (1H, dd, *J* 16.1, 7.6)

^{13}C{^{1}H} NMR (126 MHz, CDCl$_3$) δ_C: 193.7, 147.6, 144.9, 132.5, 131.0, 128.2, 127.5, 127.4, 121.6, 120.6 (q, *J* 258.9)

^{19}F NMR (471 MHz, CDCl$_3$) δ_F: -57.3 (s)

HRMS: (ESI$^+$) $C_{10}H_7F_3O_2Na$ [M+Na]$^+$ requires 239.0290, found 239.0289 (-0.4 ppm)

(*E*)-*N*-(3-(2-(trifluoromethoxy)phenyl)allyl)aniline (SI-14)

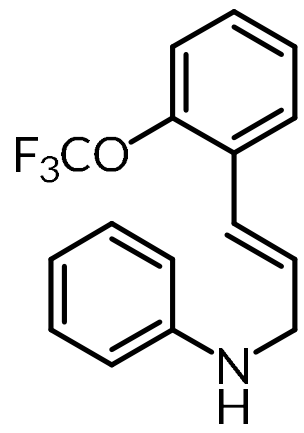

Following general procedure **A**, aniline (0.14 mL, 1.50 mmol), **SI-13** (0.32 g, 1.50 mmol), acetic acid (0.09 mL, 1.50 mmol), sodium triacetoxyborohydride (0.64 g, 3.00 mmol) and CH_2Cl_2 (5.0 mL), after purification by column chromatography (hexane/Et_2O, 100:0 → 99:1 → 98:2), gave the title compound **SI-14** (0.40 g, 92%) as a yellow oil.

ν_{max}(film) / cm^{-1}: 3414 (N–H), 1601 (C=C), 1504, 1246, 1209, 1153, 748, 692

^{1}H NMR (500 MHz, $CDCl_3$) δ_H: 7.56 (1H, dd, *J* 7.2, 2.3), 7.30 – 7.18 (5H, m), 6.88 (1H, d, *J* 16.0), 6.77 – 6.71 (1H, m), 6.71 – 6.66 (2H, m), 6.38 (1H, dt, *J* 16.0, 5.8), 3.99 (2H, dd, *J* 5.9, 1.7), 3.87 (1H, br. s)

^{13}C{^{1}H} NMR (126 MHz, $CDCl_3$) δ_C: 148.0, 146.4, 130.6, 130.5, 129.4, 128.7, 127.14, 127.11, 124.7, 121.5, 120.8 (q, *J* 257.6), 117.9, 113.2, 46.5

^{19}F NMR (471 MHz, $CDCl_3$) δ_F: -57.3 (s)

HRMS: (ESI$^+$) $C_{16}H_{13}F_3NO$ [M-H]$^+$ requires 292.0944, found 292.0941 (-1.0 ppm)

(*E*)-*N*-Benzyl-*N*-(3-(2-(trifluoromethoxy)phenyl)allyl)aniline (1av)

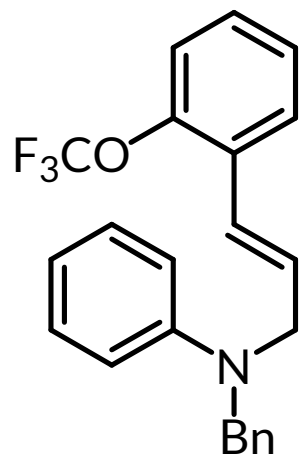

Following general procedure **C**, **SI-14** (0.40 g, 1.38 mmol), benzyl bromide (0.18 mL, 1.51 mmol), K_2CO_3 (0.21 g, 1.51 mmol), and acetone (5.5 mL), after purification by column chromatography (hexane/CH_2Cl_2, 98:2 → 95:5 → 90:10), gave the title compound **1av** (0.44 g, 91%) as a colourless oil.

ν_{max}(film) / cm^{-1}: 1597, 1504, 1452, 1356, 1248, 1213, 1163, 746, 727, 692

^{1}H NMR (500 MHz, $CDCl_3$) δ_H: 7.52 (1H, dd, *J* 7.2, 2.2), 7.38 – 7.32 (2H, m), 7.31 – 7.19 (8H, m), 6.80 (2H, dd, *J* 8.8, 1.0), 6.79 (1H, dt, *J* 16.1, 1.8), 6.73 (1H, tt, *J* 7.3, 1.1), 6.32 (1H, dt, *J* 16.0, 5.5), 4.60 (2H, s), 4.20 (2H, dd, *J* 5.6, 1.7)

$^{13}C\{^1H\}$ NMR (126 MHz, $CDCl_3$) δ_C: 149.1, 146.3, 139.0, 130.6, 129.4, 129.1, 128.8, 128.6, 127.2, 127.12, 127.06, 126.9, 125.0, 121.6, 120.6 (q, *J* 257.5), 117.0, 112.8, 54.2, 52.8

^{19}F NMR (471 MHz, $CDCl_3$) δ_F: -57.3 (s)

HRMS: (ESI$^+$) $C_{23}H_{21}F_3NO$ $[M+H]^+$ requires 384.1570, found 384.1569 (-0.3 ppm)

(*E*)-*N*-(3-(Furan-2-yl)allyl)aniline (3h)

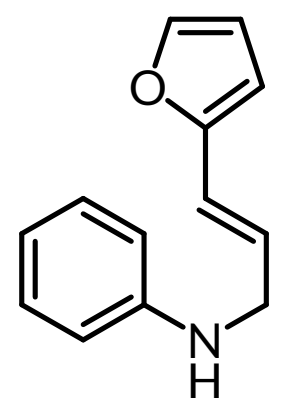

Following general procedure **A**, aniline (0.91 mL, 10.00 mmol), (3-(2-furyl)acrolein (1.22 g, 10.00 mmol), acetic acid (0.58 mL, 10.00 mmol), sodium triacetoxyborohydride (4.24 g, 20.00 mmol) and CH_2Cl_2 (34.4 mL), after purification by column chromatography (hexane/Et_2O, 100:0 → 98:2 → 95:5), gave the title compound **3h** (1.40 g, 70%) as pale-green oil.

ν_{max}(film) / cm^{-1}: 3414 (N–H), 3021, 1601 (C=C), 1504, 1323, 1250, 1013, 961

^1H NMR (500 MHz, $CDCl_3$) δ_H: 7.34 (1H, d, *J* 1.8), 7.23 – 7.16 (2H, m), 6.73 (1H, t, *J* 7.3), 6.66 (2H, d, *J* 7.6), 6.45 (1H, d, *J* 15.9), 6.36 (1H, dd, *J* 3.3, 1.9), 6.28 (1H, dt, *J* 15.8, 5.6), 6.21 (1H, d, *J* 3.3), 3.93 (2H, dd, *J* 5.6, 1.7), 3.86 (1H, br s)

$^{13}C\{^1H\}$ NMR (126 MHz, $CDCl_3$) δ_C: 152.5, 148.0, 141.8, 129.3, 125.8, 119.6, 117.5, 113.0, 111.3, 107.6, 45.7

HRMS: (ESI$^+$) $C_{13}H_{14}NO$ $[M+H]^+$ requires 200.1070, found 200.1070 (0 ppm)

(*E*)-*N*-Benzyl-*N*-(3-(furan-2-yl)allyl)aniline (1aw)

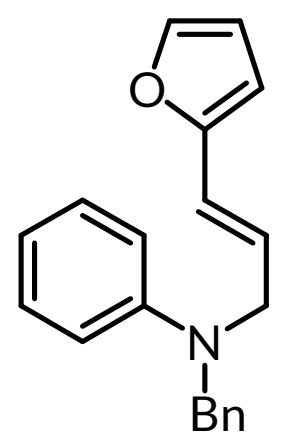

Following general procedure **C**, **3h** (0.60 g, 3.00 mmol), benzyl bromide (0.40 mL, 3.30 mmol), K_2CO_3 (0.46 g, 3.30 mmol), and acetone (12.0 mL), after purification by column chromatography (hexane/Et_2O, 100:0 → 99:1 → 98:2), gave the title compound **1aw** (0.87 g, 100%) as a yellow oil.

ν_{max}(film) / cm^{-1}: 1595, 1504, 1389, 1356, 1233, 930

^{1}H NMR (500 MHz, CDCl$_3$) δ_H: 7.37 – 7.31 (3H, m), 7.28 – 7.25 (3H, m), 7.23 – 7.18 (2H, m), 6.76 (2H, d, *J* 7.9), 6.72 (1H, td, *J* 7.3, 1.1), 6.38 – 6.31 (2H, m), 6.23 (1H, dt, *J* 15.9, 5.0), 6.19 (1H, d, *J* 3.3), 4.61 (2H, s), 4.16 (2H, d, *J* 4.8)

^{13}C{^{1}H} NMR (126 MHz, CDCl$_3$) δ_C: 152.7, 149.0, 141.9, 138.9, 129.4, 128.8, 127.0, 126.7, 124.3, 119.7, 116.8, 112.5, 111.4, 107.6, 54.0, 52.3

HRMS: (ESI$^+$) $C_{20}H_{19}NO$ [M]$^+$ requires 289.1461, found 289.1460 (-0.3 ppm)

(*E*)-3-(Benzo[*b*]thiophen-3-yl)acrylaldehyde (SI-15)

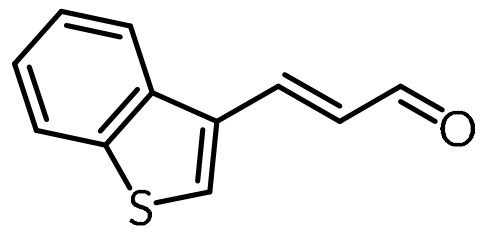

Following general procedure **D**, benzothiophene-3-carboxaldehyde (811 mg, 5.00 mmol), ^{n}BuLi (2.2 mL, 5.50 mmol, 2.5 M in hexanes), and (1,3-dioxolan-2-ylmethyl)triphenylphosphanium bromide (2.36 g, 5.50 mmol) in THF (10 mL), after purification by column chromatography (hexane/EtOAc, 99:1 → 98:2) gave the title compound **SI-15** (0.21 g, 22%) as a white solid.

mp 84 – 87 °C

ν_{max}(film) / cm^{-1}: 1672 (C=O), 1616 (C=C), 1499, 1423, 1126, 964, 756

^{1}H NMR (500 MHz, CDCl$_3$) δ_H: 9.75 (1H, d, *J* 7.6), 8.02 (1H, d, *J* 8.1), 7.92 (1H, d, *J* 8.0), 7.89 (1H, s), 7.75 (1H, d, *J* 16.0), 7.51 (1H, ddd, *J* 8.2, 7.1, 1.2), 7.45 (1H, ddd, *J* 8.2, 7.1, 1.2), 6.84 (1H, dd, *J* 16.0, 7.6)

^{13}C{^{1}H} NMR (126 MHz, CDCl$_3$) δ_C: 193.9, 144.0, 140.8, 136.9, 131.5, 130.3, 129.0, 125.5, 125.4, 123.3, 122.0

HRMS: (ESI$^+$) $C_{11}H_9OS$ [M+H]$^+$ requires 189.0369, found 189.0368 (-0.5 ppm)

(*E*)-*N*-(3-(Benzo[*b*]thiophen-3-yl)allyl)aniline (SI-16)

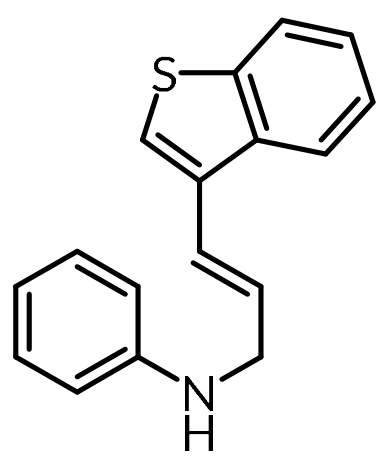

Following general procedure **A**, aniline (0.10 mL, 1.09 mmol), **SI-15** (0.21 g, 1.09 mmol), acetic acid (0.06 mL, 1.09 mmol), sodium triacetoxyborohydride (0.46 g, 2.18 mmol) and CH_2Cl_2 (3.6

mL), after purification by column chromatography (hexane/Et_2O, 99:1 → 98:2 → 97:3 → 96:4 → 95:5), gave the title compound **SI-16** (0.29 g, 99%) as a pale yellow solid.

mp 63 – 66 °C

ν_{max}(film) / cm^{-1}: 3406 (N–H), 3049, 1601 (C=C) 1504, 1425, 1319, 1250, 960. 748, 729, 692

^{1}H NMR (500 MHz, $CDCl_3$) δ_H: 7.93 – 7.81 (2H, m), 7.44 – 7.39 (2H, m), 7.37 (1H, td, *J* 7.5, 1.5), 7.25 – 7.19 (2H, m), 6.91 (1H, dq, *J* 15.8, 1.4), 6.76 (1H, tt, *J* 7.4, 1.1), 6.73 – 6.69 (2H, m), 6.41 (1H, dt, *J* 15.8, 5.7), 4.01 (2H, dd, *J* 5.8, 1.7), 3.91 (1H, br. s)

^{13}C{^{1}H} NMR (126 MHz, $CDCl_3$) δ_C: 148.1, 140.6, 137.8, 133.7, 129.4, 129.0, 124.6, 124.4, 124.0, 123.0, 122.1, 122.0, 117.9, 113.2, 46.6

HRMS: (ESI$^+$) $C_{17}H_{14}NS$ [M-H]$^+$ requires 264.0841, found 264.0840 (-0.4 ppm)

(*E*)-*N*-(3-(Benzo[*b*]thiophen-3-yl)allyl)-*N*-benzylaniline (1ax)

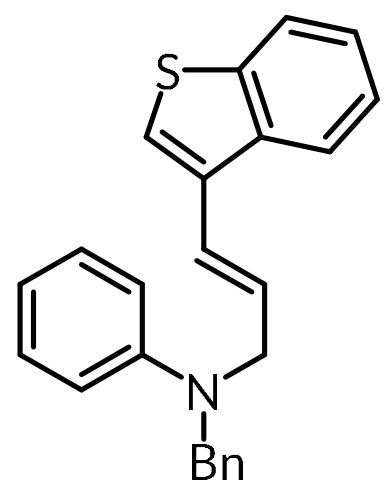

Following general procedure **C**, **SI-16** (0.27 g, 1.00 mmol), benzyl bromide (0.13 mL, 1.10 mmol), K_2CO_3 (0.15 g, 1.10 mmol), and acetone (4.0 mL), after purification by column chromatography (hexane/CH_2Cl_2, 98:2 → 95:5 → 90:10 → 80:20), gave the title compound **1ax** (0.32 g, 89%) as a colourless oil.

ν_{max}(film) / cm^{-1}: 1597 (C=C), 1504, 1450, 1425, 1356, 1219, 961, 746, 727, 692

^{1}H NMR (500 MHz, $CDCl_3$) δ_H: 7.89 – 7.84 (1H, m), 7.84 – 7.79 (1H, m), 7.42 – 7.34 (5H, m), 7.34 – 7.27 (3H, m), 7.27 – 7.22 (2H, m), 6.85 (2H, d, *J* 7.8), 6.81 (1H, d, *J* 15.9), 6.76 (1H, t, *J* 7.3), 6.35 (1H, dt, *J* 15.9, 5.3), 4.67 (2H, s), 4.25 (2H, dd, *J* 5.4, 1.7)

^{13}C{^{1}H} NMR (126 MHz, $CDCl_3$) δ_C: 149.2, 140.6, 139.0, 137.8, 133.7, 129.4, 128.8, 127.5, 127.0, 126.9, 124.6, 124.4, 124.0, 123.0, 122.1, 122.0, 116.9, 112.7, 54.1, 52.9

HRMS: (ESI$^+$) $C_{24}H_{20}NS$ [M-H]$^+$ requires 354.1311, found 354.1309 (-0.6 ppm)

(*E*)-*N*-Benzyl-*N*-(3-(3-(4-fluorophenyl)-1-isopropyl-1*H*-indol-2-yl)allyl)aniline (1ay)

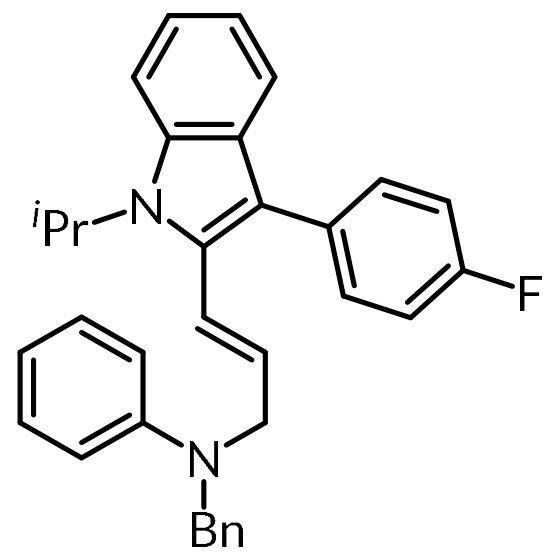

Following general procedure **A**, *N*-benzylaniline (0.46 g, 2.50 mmol), (*E*)-3-(3-(4-fluorophenyl)-1-isopropyl-1*H*-indol-2-yl)acrylaldehyde (0.77 g, 2.50 mmol), acetic acid (0.14 mL, 2.50 mmol), sodium triacetoxyborohydride (1.06 g, 5.00 mmol) and CH_2Cl_2 (8.4 mL), after purification by column chromatography (hexane/CH_2Cl_2, 90:10 → 85:15 → 80:20), gave the title compound **1ay** (0.73 g, 61%) as pale-orange solid.

mp 115 – 118 °C

ν_{max}(film) / cm^{-1}: 1595 (C=C), 1545, 1501, 1454, 1335, 1217, 837

^{1}H NMR (500 MHz, $CDCl_3$) δ_H: 7.54 (1H, d, *J* 7.9), 7.52 (1H, d, *J* 8.5), 7.39 – 7.29 (4H, m), 7.29 – 7.15 (6H, m), 7.08 (1H, t, *J* 7.5), 7.03 (2H, t, *J* 8.7), 6.78 – 6.71 (3H, m), 6.53 (1H, dt, *J* 16.1, 1.9), 5.78 (1H, dt, *J* 16.1, 4.8), 4.72 (1H, hept, *J* 7.0), 4.56 (2H, s), 4.09 (2H, dd, *J* 4.9, 1.9), 1.59 (6H, d, *J* 7.0)

^{13}C{^{1}H} NMR (126 MHz, $CDCl_3$) δ_C: 161.4 (d, *J* 244.5), 148.8, 138.9, 135.2, 133.9, 133.2, 131.9 (d, *J* 7.7), 131.6 (d, *J* 3.4), 129.3, 128.8, 128.3, 127.1, 126.8, 121.8, 120.7, 119.7, 119.5, 117.1, 115.3 (d, *J* 21.2), 114.7, 112.9, 111.8, 54.8, 53.0, 47.8, 21.8

^{19}F NMR (471 MHz, $CDCl_3$) δ_F: -116.9 – -117.0 (m)

HRMS: (ESI$^+$) $C_{33}H_{32}FN_2$ $[M+H]^+$ requires 475.2544, found 475.2549 (+1.1 ppm)

(2*E*,4*E*)-5-Phenylpenta-2,4-dienal (SI-17)

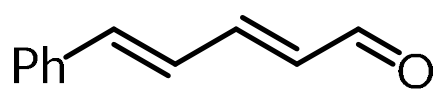

Following general procedure **D**, *trans*-cinnamaldehyde (0.63 mL, 5.00 mmol), ^{n}BuLi (2.2 mL, 5.50 mmol, 2.5 M in hexanes), and (1,3-dioxolan-2-ylmethyl)triphenylphosphanium bromide (2.36 g, 5.50 mmol) in THF (10 mL), after purification by column chromatography (hexane/EtOAc, 98:2 → 95:5 → 90:10) gave the title compound **SI-17** (0.53 g, 67%) as a yellow oil.

ν_{max}(film) / cm^{-1}: 1672 (C=O), 1616 (C=C), 1499, 1423, 1126, 964, 756

^{1}H NMR (500 MHz, $CDCl_3$) δ_H: 9.62 (1H, d, *J* 7.9), 7.54 – 7.48 (2H, m), 7.41 – 7.37 (2H, m), 7.37 – 7.33 (1H, m), 7.27 (1H, ddd, *J* 15.3, 7.7, 2.4), 7.06 – 6.96 (2H, m), 6.27 (1H, dd, *J* 15.2, 7.9)

$^{13}C\{^1H\}$ NMR (126 MHz, $CDCl_3$) δ_C: 193.7, 152.1, 142.6, 135.7, 131.8, 129.8, 129.1, 127.7, 126.3

HRMS: (ESI^+) $C_{11}H_{11}O$ $[M+H]^+$ requires 159.0804, found 159.0804 (0.0 ppm)

***N*-((2*E*,4*E*)-5-Phenylpenta-2,4-dien-1-yl)aniline (SI-18)**

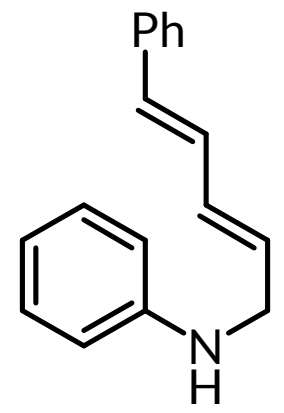

Following general procedure **A**, aniline (0.28 mL, 3.00 mmol), **SI-17** (0.47 g, 3.00 mmol), acetic acid (0.17 mL, 3.00 mmol), sodium triacetoxyborohydride (1.27 g, 6.00 mmol) and CH_2Cl_2 (10 mL), after purification by column chromatography (hexane/Et_2O, 99:1 → 98:2 → 97:3 → 96:4 → 95:5), gave the title compound **SI-18** (0.30 g, 44%, as a 94:6 (2*E*, 4*E*):(2*Z*, 4*E*) mixture of stereoisomer) as a pale-yellow oil.

ν_{max}(film) / cm^{-1}: 3404 (N–H), 3021, 1601, 1503, 1317, 1254, 986, 746

^1H NMR (500 MHz, $CDCl_3$) δ_H: 7.41 – 7.36 (2H, m), 7.31 (2H, t, *J* 7.7), 7.25 – 7.17 (3H, m), 6.79 (1H, dd, *J* 15.7, 10.5), 6.73 (1H, tt, *J* 7.3, 1.1), 6.65 (2H, dd, *J* 8.7, 1.1), 6.53 (1H, d, *J* 15.7), 6.44 (1H, dd, *J* 15.2, 10.5), 5.94 (1H, dt, *J* 15.2, 5.8), 3.88 (2H, d, *J* 5.8), 3.81 (1H, br. s)

$^{13}C\{^1H\}$ NMR (126 MHz, $CDCl_3$) δ_C: 148.1, 137.4, 132.3, 132.1, 131.3, 129.4, 128.8, 128.4, 127.7, 126.5, 117.8, 113.2, 46.1

HRMS: (ESI^+) $C_{17}H_{16}N$ $[M-H]^+$ requires 234.1277, found 234.1276 (-0.4 ppm)

***N*-Benzyl-*N*-((2*E*,4*E*)-5-phenylpenta-2,4-dien-1-yl)aniline (1az)**

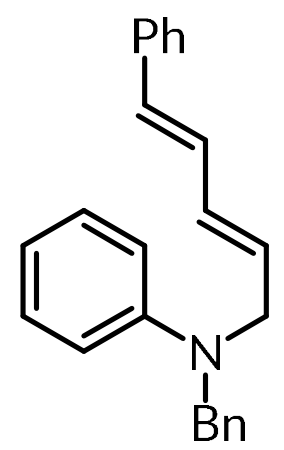

Following general procedure **C**, **SI-18** (0.24 g, 1.00 mmol), benzyl bromide (0.13 mL, 1.10 mmol), K_2CO_3 (0.15 g, 1.10 mmol), and acetone (4.0 mL), after purification by column chromatography (hexane/CH_2Cl_2, 98:2 → 95:5 → 90:10 → 80:20), gave the title compound **1az** (0.26 g, 81%, as a 93:7 (2E,4E):(2Z,4E) mixture of stereoisomers) as a yellow solid.

mp 69 – 72 °C

ν_{max}(film) / cm^{-1}: 1597 (C=C), 1504, 1450, 1356, 1233, 989, 746, 691

^{1}H NMR (500 MHz, CDCl$_3$) δ_H: 7.40 – 7.36 (2H, m), 7.36 – 7.27 (6H, m), 7.26 – 7.18 (4H, m), 6.83 – 6.74 (3H, m), 6.72 (1H, tt, *J* 7.3, 1.1), 6.50 (1H, d, *J* 15.7), 6.35 (1H, dd, *J* 15.1, 10.5), 5.89 (1H, dt, *J* 15.2, 5.3), 4.59 (2H, s), 4.12 (2H, d, *J* 4.6)

^{13}C{^{1}H} NMR (126 MHz, CDCl$_3$) δ_C: 149.1, 139.0, 137.4, 132.1, 132.0, 130.0, 129.4, 128.8, 128.7, 128.4, 127.6, 127.0, 126.8, 126.4, 116.8, 112.6, 54.1, 52.4

HRMS: (ESI$^+$) $C_{24}H_{22}N$ [M-H]$^+$ requires 324.1747, found 324.1745 (-0.6 ppm)

(*E*)-*N*-(4-Phenylbut-3-en-2-yl)aniline (SI-19)

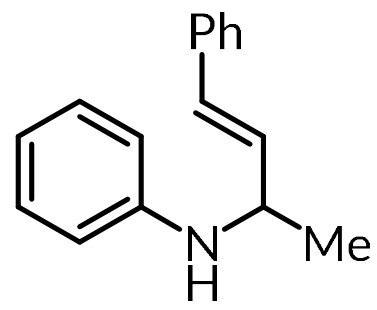

Following general procedure **A**, aniline (0.46 mL, 5.00 mmol), 4-phenylbut-3-en-2-one (0.73 g, 5.00 mmol), acetic acid (0.29 mL, 2.00 mmol), sodium triacetoxyborohydride (2.12 g, 10.00 mmol) and CH_2Cl_2 (16.7 mL), after purification by column chromatography (hexane/Et_2O, 99:1 → 97.5:2.5 → 95:5), gave the title compound **SI-19** (0.61 g, 55%) as a pale-yellow oil.

ν_{max}(film) / cm^{-1}: 3408 (N–H), 2970, 1601 (C=C), 1504, 1504, 1319, 1257, 1179, 966, 746, 692

^{1}H NMR (500 MHz, CDCl$_3$) δ_H: 7.39 – 7.34 (2H, m), 7.34 – 7.27 (2H, m), 7.24 – 7.20 (1H, m), 7.19 – 7.14 (2H, m), 6.70 (1H, tt, *J* 7.4, 1.1), 6.68 – 6.64 (2H, m), 6.59 (1H, dd, *J* 16.0, 1.4), 6.23 (1H, dd, *J* 15.9, 5.8), 4.16 (1H, p, *J* 6.3), 3.71 (1H, s), 1.42 (3H, d, *J* 6.6)

^{13}C{^{1}H} NMR (126 MHz, CDCl$_3$) δ_C: 147.6, 137.1, 133.4, 129.4, 129.3, 128.7, 127.5, 126.5, 117.5, 113.5, 51.0, 22.2

HRMS: (ESI$^+$) $C_{16}H_{16}N$ [M-H]$^+$ requires 222.1277, found 222.1276 (-0.5 ppm)

(*E*)-*N*-Benzyl-*N*-(4-phenylbut-3-en-2-yl)aniline (1ba)

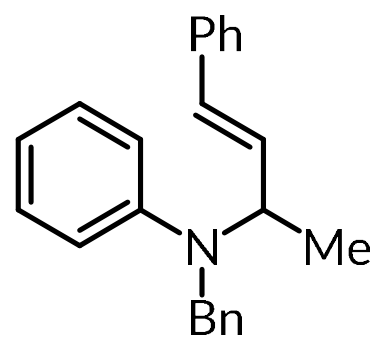

Following general procedure **C**, **SI-19** (0.60 g, 2.69 mmol), benzyl bromide (0.35 mL, 2.96 mmol), K_2CO_3 (0.41 g, 2.95 mmol), and acetone (11 mL), after purification by column chromatography

(hexane/CH_2Cl_2, 95:5 → 90:10 → 80:20), gave the title compound **1ba** (0.44 g, 53%) as a pale-yellow oil.

ν_{max}(film) / cm^{-1}: 3024 (N–H), 1595, 1503, 1450, 1381, 1248, 1161, 968, 746, 727, 692

^{1}H NMR (500 MHz, $CDCl_3$) δ_H: 7.35 – 7.26 (8H, m), 7.25 – 7.16 (4H, m), 6.80 (2H, d, *J* 7.9), 6.72 (1H, tt, *J* 7.2, 1.0), 6.50 (1H, dd, *J* 16.2, 1.8), 6.34 (1H, dd, *J* 16.2, 4.5), 4.83 (1H, qdd, *J* 6.7, 4.4, 1.8), 4.49 (2H, s), 1.43 (3H, d, *J* 6.8)

^{13}C{^{1}H} NMR (126 MHz, $CDCl_3$) δ_C: 149.3, 140.7, 137.1, 131.1, 130.3, 129.3, 128.7, 128.6, 127.6, 126.6, 126.5 (2C), 117.1, 113.9, 54.7, 50.4, 17.6

Note: 126.5 ppm peak contains two carbon peaks.

HRMS: (ESI$^+$) $C_{23}H_{24}N$ $[M+H]^+$ requires 314.1903, found 314.1901 (-0.6 ppm)

_N_-(But-3-en-1-yl)aniline (SI-20)

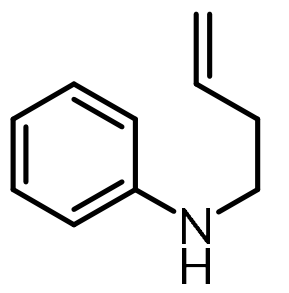

Prepared according to literature.[6] To a solution of aniline (2.3 mL, 25.00 mmol) and NaI (74.9 mg, 0.50 mmol) in EtOH (10 mL) was added 4-bromobut-1-ene (0.53 mL, 5.00 mmol) and the resulting mixture stirred at reflux for 18 hours. The mixture was concentrated under reduced pressure, diluted with CH_2Cl_2, and quenched with 1.0 M aq. NaOH (10 mL). The organic layer was separated, washed with brine, dried over Na_2SO_4, filtered, and concentrated under reduced pressure. Purification by column chromatography (hexane/EtOAc, 100:0 → 98:2 → 95:5) gave the title compound **SI-20** (0.70 g, 95 %) as a yellow oil.

ν_{max}(film) / cm^{-1}: 3406 (N–H), 1601, 1504, 1474, 1431, 1315, 1263, 1179, 991, 914, 746, 691

^{1}H NMR (500 MHz, $CDCl_3$) δ_H: 7.22 – 7.16 (2H, m), 6.72 (1H, tt, *J* 7.4, 1.1), 6.63 (2H, dd, *J* 8.6, 1.1), 5.85 (1H, ddt, *J* 17.1, 10.1, 6.8), 5.17 (1H, dq, *J* 17.1, 1.6), 5.14 – 5.11 (1H, m), 3.67 (1H, s), 3.20 (2H, t, *J* 6.7), 2.40 (2H, qt, *J* 6.8, 1.4)

^{13}C{^{1}H} NMR (126 MHz, $CDCl_3$) δ_C: 148.4, 135.9, 129.4, 117.5, 117.2, 113.0, 42.9, 33.8

HRMS: (ESI$^+$) $C_{10}H_{14}N$ $[M+H]^+$ requires 148.1121, found 148.1121 (0.0 ppm)

N-Benzyl-N-(but-3-en-1-yl)aniline (1bb)

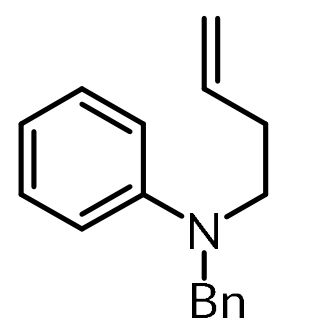

Following general procedure **C**, **SI-20** (0.37 g, 2.50 mmol), benzyl bromide (0.33 mL, 2.75 mmol), K_2CO_3 (0.38 g, 2.75 mmol), and acetone (10 mL), after purification by column chromatography (hexane/Et_2O, 100:0 → 99:1 → 98:2), gave the title compound **1bb** (0.46 g, 77%) as a colourless oil.

ν_{max}(film) / cm^{-1}: 1597, 1504, 1450, 1354, 1213, 1193, 987, 912, 745, 725, 691

^{1}H NMR (500 MHz, $CDCl_3$) δ_H: 7.35 – 7.29 (2H, m), 7.26 – 7.22 (3H, m), 7.22 – 7.17 (2H, m), 6.76 – 6.64 (3H, m), 5.84 (1H, ddt, *J* 17.1, 10.2, 6.9), 5.12 (1H, dq, *J* 17.1, 1.7), 5.06 (1H, ddt, *J* 10.2, 2.2, 1.2), 4.57 (2H, s), 3.52 – 3.45 (2H, m), 2.47 – 2.39 (2H, m)

^{13}C{^{1}H} NMR (126 MHz, $CDCl_3$) δ_C: 148.5, 139.1, 135.8, 129.4, 128.7, 126.9, 126.7, 116.7, 116.3, 112.3, 54.6, 50.9, 31.7

HRMS: (ESI$^+$) $C_{17}H_{19}NNa$ [M+Na]$^+$ requires 260.1410, found 260.1408 (-0.8 ppm)

4-(*Tert*-butyl)-N-cinnamylaniline (SI-21)

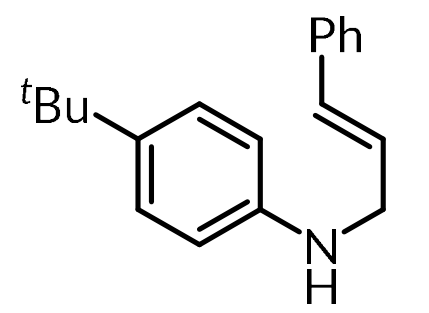

Following general procedure **A**, 4-(tert-butyl)aniline (0.80 mL, 5.00 mmol), *trans*-cinnamaldehyde (0.63 mL, 5.00 mmol), acetic acid (0.29 mL, 5.00 mmol), sodium triacetoxyborohydride (2.12 g, 10.00 mmol) and CH_2Cl_2 (16.7 mL), after purification by column chromatography (hexane/Et_2O, 100:0 → 98:2), gave the title compound **SI-21** (913 mg, 69%) as a pink solid.

mp 37 – 39 °C

ν_{max} (film) / cm^{-1}: 3387 (N–H), 3019, 2949, 2889, 2864, 1611 (C=C), 1516, 956, 817

^{1}H NMR (400 MHz, $CDCl_3$) δ_H: 7.42 – 7.38 (2H, m), 7.36 – 7.31 (2H, m), 7.28 – 7.22 (3H, m), 6.70 – 6.61 (3H, m), 6.37 (1H, dt, *J* 15.9, 5.8), 3.95 (2H, dd, *J* 5.8, 1.6), 1.31 (9H, s)

^{13}C{^{1}H} NMR (101 MHz, $CDCl_3$) δ_C: 145.9, 140.6, 137.1, 131.5, 128.7, 127.62, 127.56, 126.5, 126.2, 113.0, 46.7, 34.0, 31.7

HRMS: (ESI+) $C_{19}H_{24}N$ $[M+H]^+$ requires 266.1903, found 266.1902 (-0.4 ppm)

N-Benzyl-4-(tert-butyl)-N-cinnamylaniline (1bc)

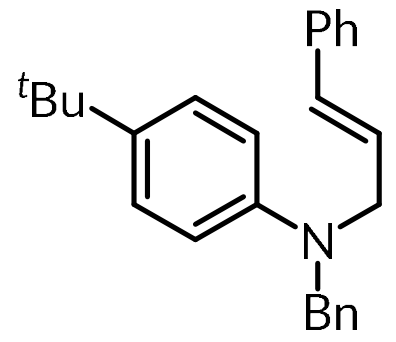

Following general procedure **C**, **SI-21** (531 mg, 2.00 mmol), benzyl bromide (0.26 mL, 2.20 mmol), K_2CO_3 (304 mg, 2.20 mmol), and acetone (8.0 mL), after purification by column chromatography (hexane/Et_2O, 100:0 → 98:2), gave the title compound **1bc** (695 mg, 98%) as a pale red oil.

ν_{max} (film) / cm^{-1}: 2959, 1612, 1518, 1362, 1204, 964, 812, 727

^{1}H NMR (400 MHz, $CDCl_3$) δ_H: 7.41 – 7.21 (12H, m), 6.77 (2H, d, *J* 8.9), 6.55 (1H, dt, *J* 15.9, 1.7), 6.32 (1H, dt, *J* 15.9, 5.4), 4.60 (2H, s), 4.18 (2H, dd, *J* 5.4, 1.7), 1.31 (9H, s)

^{13}C{^{1}H} NMR (101 MHz, $CDCl_3$) δ_C: 147.0, 139.4 (2C), 137.1, 131.5, 128.72, 128.68, 127.5, 126.94, 126.89, 126.5, 126.2, 126.0, 112.4, 54.2, 52.7, 33.9, 31.7

Note: 139.4 ppm peak contains two carbon peaks.

HRMS: (ESI+) $C_{26}H_{30}N$ $[M+H]^+$ requires 356.2373, found 356.2369 (-1.1 ppm)

N-Benzyl-4-methoxyaniline (SI-22)

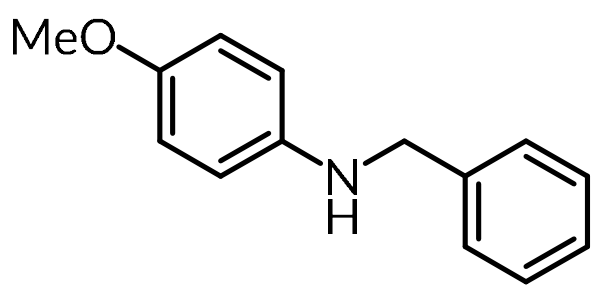

Following modified general procedure **C**, *p*-anisidine (1.00 g, 8.12 mmol), benzyl bromide (0.48 mL, 4.06 mmol), Cs_2CO_3 (2.65 g, 8.12 mmol), and DMF (10 mL), room temperature stirring for 16 h, after purification by column chromatography (hexane/CH_2Cl_2, 90:10 → 80:20 → 60:40), gave the title compound **SI-22** (623 mg, 72%) as a colourless oil.

ν_{max} (film) / cm^{-1}: 3399 (N–H), 2830, 2361, 1508, 1231, 1034, 818, 741

^{1}H NMR (500 MHz, $CDCl_3$) δ_H: 7.45 – 7.35 (4H, m), 7.31 (1H, t, *J* 6.9), 6.82 (2H, d, *J* 8.9), 6.64 (2H, d, *J* 8.9), 4.32 (2H, s), 3.84 – 3.76 (4H, m)

^{13}C{^{1}H} NMR (126 MHz, $CDCl_3$) δ_C: 152.2, 142.5, 139.8, 128.7, 127.6, 127.2, 115.0, 114.2, 55.9, 49.3

HRMS: (ESI$^+$) $C_{14}H_{16}NO$ $[M+H]^+$ requires 214.1226, found 214.1225 (-0.5 ppm)

_N_-Benzyl-_N_-cinnamyl-4-methoxyaniline (1bd)

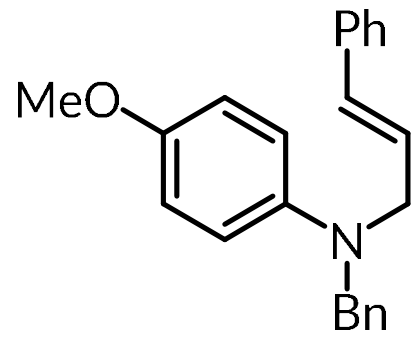

Following general procedure **B**, **SI-22** (500 mg, 2.34 mmol), cinnamyl bromide (553 mg, 2.81 mmol), NaH (112 mg, 2.81 mmol), and DMF (10 mL), after purification by column chromatography (hexane/CH_2Cl_2, 90:10 → 80:20 → 70:30), gave the title compound **1bd** (756 mg, 98%) as a colourless oil.

ν_{max} (film) / cm^{-1}: 3026, 2831, 1510, 1450, 1240, 1040, 966, 814, 733, 692

^{1}H NMR (500 MHz, CDCl$_3$) δ_H: 7.38 – 7.21 (10H, m), 6.87 – 6.80 (2H, m), 6.80 – 6.76 (2H, m), 6.53 (1H, dt, *J* 16.0, 1.7), 6.29 (1H, dt, *J* 15.9, 5.5), 4.54 (2H, s), 4.12 (2H, dd, *J* 5.5, 1.7), 3.76 (3H, s)

^{13}C{^{1}H} NMR (126 MHz, CDCl$_3$) δ_C: 151.8, 143.9, 139.4, 137.1, 131.6, 128.7 (2C), 127.5, 127.1, 126.9, 126.4, 126.2, 114.9, 114.7, 55.9, 55.0, 53.5

<u>Note: 128.7 ppm peak contains two carbon peaks.</u>

HRMS: (ESI$^+$) $C_{23}H_{24}NO$ $[M+H]^+$ requires 330.1852, found 330.1849 (-0.9 ppm)

_N_-Cinnamyl-4-fluoroaniline (SI-23)

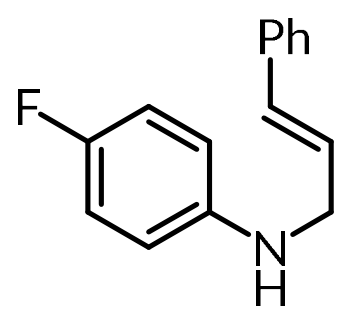

Following general procedure **A**, 4-fluoroaniline (0.47 mL, 5.00 mmol), *trans*-cinnamaldehyde (0.63 mL, 5.00 mmol), acetic acid (0.29 mL, 5.00 mmol), sodium triacetoxyborohydride (2.12 g, 10.00 mmol) and CH_2Cl_2 (16.7 mL), after purification by column chromatography (hexane/Et_2O, 100:0 → 98:2 → 95:5), gave the title compound **SI-23** (756 mg, 67%) as a white solid.

mp 58 – 60 °C

ν_{max} (film) / cm^{-1}: 3420 (N–H), 2357, 2322, 1506 (C=C), 1207, 968, 817, 741

^{1}H NMR (500 MHz, CDCl$_3$) δ_H: 7.40 – 7.36 (2H, m), 7.35 – 7.30 (2H, m), 7.28 – 7.22 (1H, m), 6.95 – 6.87 (2H, m), 6.67 – 6.58 (3H, m), 6.32 (1H, dt, *J* 15.9, 5.8), 3.91 (2H, dd, *J* 5.8, 1.7), 3.75 (1H, br s)

$^{13}C\{^1H\}$ NMR (126 MHz, $CDCl_3$) δ_C: 156.1 (d, *J* 235.0), 144.5, 136.9, 131.8, 128.7, 127.7, 127.0, 126.5, 115.8 (d, *J* 22.3), 114.0 (d, *J* 7.5), 47.0

^{19}F NMR (471 MHz, $CDCl_3$) δ_F: -127.8 – -127.9 (m)

HRMS: (ESI$^+$) $C_{15}H_{14}FNNa$ $[M+Na]^+$ requires 250.1002, found 250.1001 (-0.4 ppm)

***N*-Benzyl-*N*-cinnamyl-4-fluoroaniline (1be)**

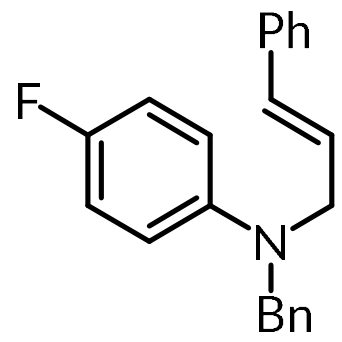

Following general procedure **C**, **SI-23** (568 mg, 2.50 mmol), benzyl bromide (0.33 mL, 2.75 mmol), K_2CO_3 (380 mg, 2.75 mmol), and acetone (10.0 mL), after purification by column chromatography (hexane/EtOAc, 100:0 → 98:2 → 95:5), gave the title compound **1be** (689 mg, 87%) as a white solid.

mp 55 – 57 °C

ν_{max} (film) / cm^{-1}: 3021, 2357, 1506, 1225, 812, 729, 690

^1H NMR (500 MHz, $CDCl_3$) δ_H: 7.44 – 7.26 (10H, m), 7.01 – 6.92 (2H, m), 6.81 – 6.72 (2H, m), 6.57 (1H, dt, *J* 15.9, 1.7), 6.31 (1H, dt, *J* 15.9, 5.3), 4.61 (2H, s), 4.18 (2H, dd, *J* 5.4, 1.7)

$^{13}C\{^1H\}$ NMR (126 MHz, $CDCl_3$) δ_C: 155.6 (d, *J* 235.2), 145.7 (d, *J* 1.8), 138.8, 136.9, 131.6, 128.8, 128.7, 127.6, 127.1, 126.8, 126.4, 125.5, 115.7 (d, *J* 22.0), 113.9 (d, *J* 7.2), 54.7, 53.3

^{19}F NMR (471 MHz, $CDCl_3$) δ_F: -128.8 – -128.9 (m)

HRMS: (ESI$^+$) $C_{22}H_{21}FN$ $[M+H]^+$ requires 318.1653, found 318.1652 (-0.3 ppm)

***N*-Cinnamyl-4-(trifluoromethyl)aniline (SI-24)**

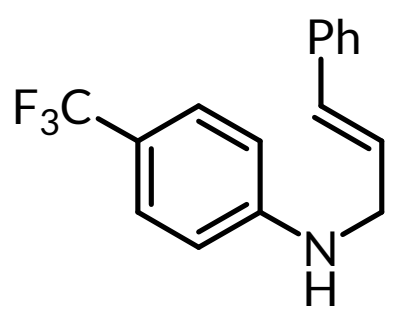

Following general procedure **A**, 4-(trifluoromethyl)aniline (0.63 mL, 5.00 mmol), *trans*-cinnamaldehyde (0.63 mL, 5.00 mmol), acetic acid (0.29 mL, 5.00 mmol), sodium triacetoxyborohydride (2.12 g, 10.00 mmol) and CH_2Cl_2 (16.7 mL), after purification by column

chromatography (hexane/EtOAc, 100:0 → 98:2 → 95:5), gave the title compound **SI-24** (1.21 g, 88%) as a white solid.

mp 72 – 74 °C

ν_{max} (film) / cm^{-1}: 3418 (N–H), 2353, 2322, 1616 (C=C), 1319, 1092, 1055, 964, 823

^{1}H NMR (500 MHz, CDCl$_3$) δ_H: 7.42 (2H, d, *J* 8.7), 7.39 – 7.36 (2H, m), 7.35 – 7.30 (2H, m), 7.28 – 7.23 (1H, m), 6.66 (2H, d, *J* 8.6), 6.62 (1H, dt, *J* 15.9, 1.7), 6.29 (1H, dt, *J* 15.8, 5.7), 4.22 (1H, br s), 4.03 – 3.92 (2H, m)

^{13}C{^{1}H} NMR (126 MHz, CDCl$_3$) δ_C: 150.5, 136.7, 132.1, 128.8, 127.9, 126.8 (q, *J* 3.8), 126.5, 126.0, 125.1 (q, *J* 270.3), 119.1 (q, *J* 32.7), 112.2, 45.8

^{19}F NMR (471 MHz, CDCl$_3$) δ_F: -61.0 (s)

HRMS: (ESI$^-$) $C_{16}H_{13}F_3N$ [M-H]$^-$ requires 276.1006, found 276.1003 (-1.1 ppm)

***N*-Benzyl-*N*-cinnamyl-4-(trifluoromethyl)aniline (1bf)**

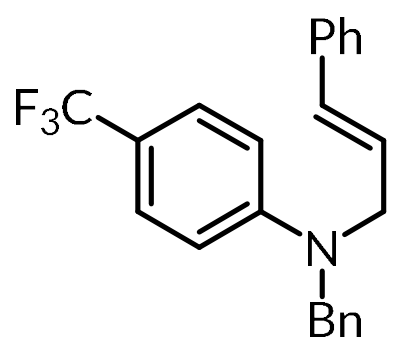

Following general procedure **B**, **SI-24** (693 mg, 2.50 mmol), benzyl bromide (0.33 mL, 2.75 mmol), NaH (150 mg, 3.75 mmol), and DMF (6.4 mL), after purification by column chromatography (hexane/CH_2Cl_2, 100:0 → 95:5 → 90:10), gave the title compound **1bf** (373 mg, 41%) as a pale-yellow solid.

mp 103 – 105 °C

ν_{max} (film) / cm^{-1}: 2918, 2351, 1612, 1327, 1098, 1070, 822, 733

^{1}H NMR (500 MHz, CDCl$_3$) δ_H: 7.43 (2H, d, *J* 8.8), 7.38 – 7.20 (10H, m), 6.78 (2H, d, *J* 8.7), 6.52 (1H, dt, *J* 15.9, 1.8), 6.26 (1H, dt, *J* 15.9, 5.2), 4.66 (2H, s), 4.23 (2H, dd, *J* 5.3, 1.8)

^{13}C{^{1}H} NMR (126 MHz, CDCl$_3$) δ_C: 151.2, 137.9, 136.6, 131.9, 129.0, 128.8, 127.9, 127.3, 126.7 (q, *J* 3.8), 126.53, 126.51, 125.2 (q, *J* 270.3), 124.3, 118.2 (q, *J* 32.8), 111.6, 53.9, 52.7

^{19}F NMR (471 MHz, CDCl$_3$) δ_F: -60.9 (s)

HRMS: (ESI$^+$) $C_{23}H_{20}F_3NNa$ [M+Na]$^+$ requires 390.1440, found 390.1438 (-0.5 ppm)

4-(Cinnamylamino)benzonitrile (SI-25)

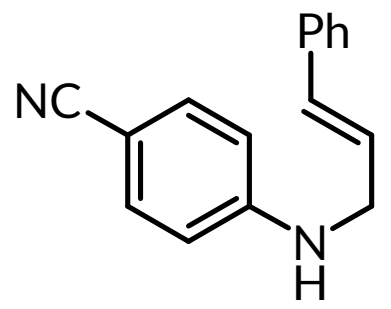

Following general procedure **A**, 4-aminobenzonitrile (591 mg, 5.00 mmol), *trans*-cinnamaldehyde (0.63 mL, 5.00 mmol), acetic acid (0.29 mL, 5.00 mmol), sodium triacetoxyborohydride (2.12 g, 10.00 mmol) and CH_2Cl_2 (16.7 mL), after purification by column chromatography (hexane/EtOAc, 90:10 → 80:20), gave the title compound **SI-25** (1.13 g, 96%) as a white solid.

mp 90 – 92 °C

ν_{max} (film) / cm^{-1}: 3372 (N–H), 2207 (C≡N), 1603 (C=C), 1520, 1171, 960, 829

^{1}H NMR (500 MHz, CDCl$_3$) δ_H: 7.42 (2H, d, *J* 8.5), 7.39 – 7.35 (2H, m), 7.35 – 7.31 (2H, m), 7.26 (1H, t, *J* 7.2), 6.66 – 6.53 (3H, m), 6.26 (1H, dt, *J* 15.9, 5.7), 4.54 (1H, t, *J* 5.8), 3.98 (2H, td, *J* 5.7, 1.6)

^{13}C{^{1}H} NMR (126 MHz, CDCl$_3$) δ_C: 151.2, 136.4, 133.8, 132.3, 128.7, 127.9, 126.4, 125.3, 120.6, 112.5, 98.9, 45.3

HRMS: (ESI$^+$) $C_{16}H_{14}N_2Na$ [M+Na]$^+$ requires 257.1049, found 257.1049 (0.0 ppm)

4-(Benzyl(cinnamyl)amino)benzonitrile (1bg)

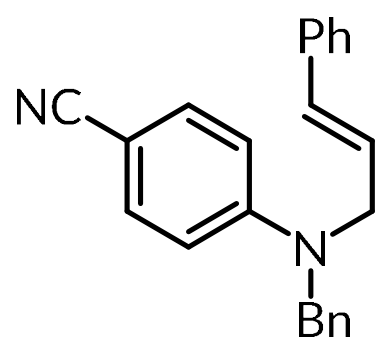

Following general procedure **B**, **SI-25** (820 mg, 3.50 mmol), benzyl bromide (0.46 mL, 3.85 mmol), NaH (210 mg, 5.25 mmol), and DMF (8.8 mL), after purification by column chromatography (hexane/EtOAc, 97:3 → 95:5 → 90:10), gave the title compound **1bg** (617 mg, 54%) as a yellow solid.

mp 85 – 87 °C

ν_{max} (film) / cm^{-1}: 2212 (C≡N), 1603, 1522, 1396, 1354, 1180, 968, 814, 690

^{1}H NMR (500 MHz, CDCl$_3$) δ_H: 7.45 (2H, d, *J* 8.9), 7.40 – 7.18 (10H, m), 6.75 (2H, d, *J* 8.9), 6.51 (1H, dt, *J* 15.9, 1.8), 6.24 (1H, dt, *J* 15.9, 5.2), 4.68 (2H, s), 4.24 (2H, dd, *J* 5.3, 1.8)

$^{13}C\{^1H\}$ NMR (126 MHz, CDCl$_3$) δ_C: 151.7, 137.1, 136.4, 133.7, 132.2, 129.0, 128.8, 128.0, 127.5, 126.5, 126.4, 123.6, 120.5, 112.1, 98.4, 53.8, 52.7

HRMS: (ESI$^+$) $C_{23}H_{20}N_2Na$ [M+Na]$^+$ requires 347.1519, found 347.1516 (-0.9 ppm)

1-(4-(Cinnamylamino)phenyl)ethan-1-one (SI-26)

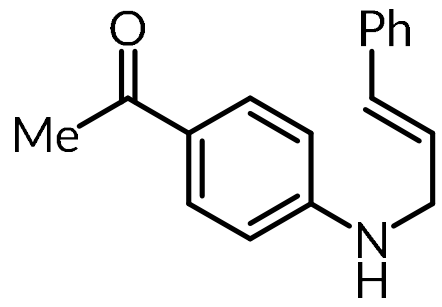

Following general procedure **A**, 1-(4-aminophenyl)ethan-1-one (676 mg, 5.00 mmol), *trans*-cinnamaldehyde (0.63 mL, 5.00 mmol), acetic acid (0.29 mL, 5.00 mmol), sodium triacetoxyborohydride (2.12 g, 10.00 mmol) and CH_2Cl_2 (16.7 mL), after purification by column chromatography (hexane/EtOAc, 80:20 → 70:30 → 60:40), gave the title compound **SI-26** (1.05 g, 84%) as a white solid.

mp 134 – 136 °C

ν_{max} (film) / cm^{-1}: 3340 (N–H), 1647 (C=O), 1585, 1358, 1277, 1179, 968, 820

^1H NMR (500 MHz, CDCl$_3$) δ_H: 7.84 (2H, d, *J* 8.8), 7.38 – 7.35 (2H, m), 7.34 – 7.29 (2H, m), 7.27 – 7.23 (1H, m), 6.67 – 6.58 (3H, m), 6.29 (1H, dt, *J* 15.9, 5.7), 4.44 (1H, t, *J* 5.9), 4.01 (2H, td, *J* 5.8, 1.6), 2.50 (3H, s)

$^{13}C\{^1H\}$ NMR (126 MHz, CDCl$_3$) δ_C: 196.5, 152.0, 136.6, 132.3, 130.9, 128.8, 127.9, 127.1, 126.5, 125.7, 111.8, 45.6, 26.2

HRMS: (ESI$^+$) $C_{17}H_{17}NONa$ [M+Na]$^+$ requires 274.1202, found 274.1201 (-0.4 ppm)

1-(4-(Benzyl(cinnamyl)amino)phenyl)ethan-1-one (1bh)

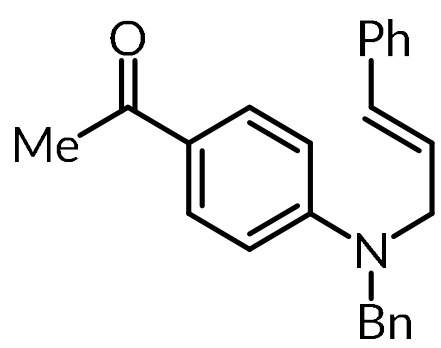

Following general procedure **C**, **SI-26** (880 mg, 3.50 mmol), benzyl bromide (0.46 mL, 3.85 mmol), K_2CO_3 (532 mg, 3.85 mmol), and acetone (14.0 mL), after purification by column chromatography (hexane/Et_2O, 75:25), gave the title compound **1bh** (433 mg, 36%) as a white solid.

mp 80 – 82 °C

ν_{max} (film) / cm^{-1}: 3026, 2862, 1653 (C=O), 1591, 1356, 1281, 1188, 816, 735

^{1}H NMR (500 MHz, CDCl$_3$) δ_H: 7.85 (2H, d, *J* 9.0), 7.41 – 7.18 (10H, m), 6.76 (2H, d, *J* 9.1), 6.51 (1H, dt, *J* 16.0, 1.7), 6.25 (1H, dt, *J* 15.9, 5.2), 4.69 (2H, s), 4.25 (2H, dd, *J* 5.2, 1.7), 2.50 (3H, s)

^{13}C{^{1}H} NMR (126 MHz, CDCl$_3$) δ_C: 196.4, 152.6, 137.6, 136.6, 132.1, 130.9, 129.0, 128.8, 127.9, 127.4, 126.53, 126.51, 126.2, 124.1, 111.3, 53.8, 52.6, 26.1

HRMS: (ESI$^+$) $C_{24}H_{24}NO$ [M+H]$^+$ requires 342.1852, found 342.1851 (-0.3 ppm)

2-(4-(Cinnamylamino)phenyl)acetonitrile (SI-27)

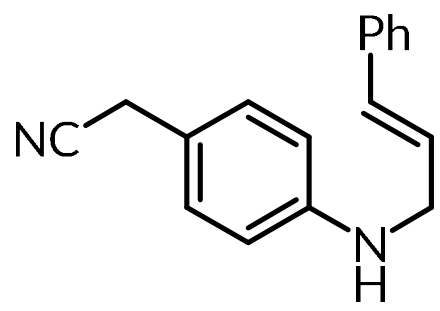

Following general procedure **A**, 2-(4-aminophenyl)acetonitrile (661 mg, 5.00 mmol), *trans*-cinnamaldehyde (0.63 mL, 5.00 mmol), acetic acid (0.29 mL, 5.00 mmol), sodium triacetoxyborohydride (2.12 g, 10.00 mmol) and CH_2Cl_2 (16.7 mL), after purification by column chromatography (hexane/EtOAc, 90:10 → 85:15 → 80:20), gave the title compound **SI-27** (958 mg, 77%) as a beige solid.

mp 91 – 93 °C

ν_{max} (film) / cm^{-1}: 3393 (N–H), 3024, 2247 (C≡N), 1614 (C=C), 1518, 1325, 966, 806, 692

^{1}H NMR (500 MHz, CDCl$_3$) δ_H: 7.40 – 7.34 (2H, m), 7.34 – 7.29 (2H, m), 7.26 – 7.21 (1H, m), 7.15 – 7.09 (2H, m), 6.71 – 6.57 (3H, m), 6.31 (1H, dt, *J* 16.1, 4.9), 3.97 – 3.91 (3H, m), 3.63 (2H, s)

^{13}C{^{1}H} NMR (126 MHz, CDCl$_3$) δ_C: 147.9, 136.8, 131.9, 129.1, 128.7, 127.8, 126.7, 126.5, 118.7, 118.4, 113.5, 46.2, 22.9

HRMS: (ESI$^+$) $C_{17}H_{15}N_2$ [M-H]$^+$ requires 247.1230, found 247.1228 (-0.8 ppm)

2-(4-(Benzyl(cinnamyl)amino)phenyl)acetonitrile (1bi)

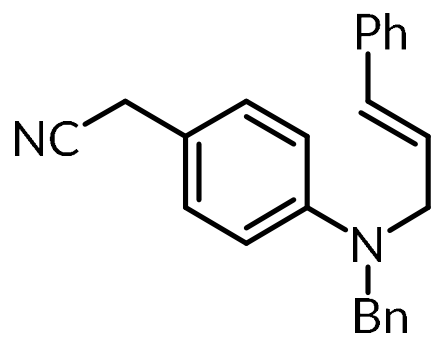

Following general procedure **A**, **SI-27** (621 mg, 2.50 mmol), benzaldehyde (0.26 mL, 2.50 mmol), acetic acid (0.14 mL, 2.50 mmol), sodium triacetoxyborohydride (1.06 g, 5.00 mmol) and CH_2Cl_2 (8.3 mL), after purification by column chromatography (hexane/EtOAc, 95:5 → 90:10), gave the title compound **1bi** (642 mg, 76%) as a yellow solid.

mp 72 – 74 °C

ν_{max} (film) / cm^{-1}: 3026, 2247 (C≡N), 1614 (C=C), 1518, 1354, 1229, 966, 799, 729, 692

^{1}H NMR (500 MHz, CDCl$_3$) δ_H: 7.40 – 7.22 (10H, m), 7.14 (2H, d, *J* 8.7), 6.77 (2H, d, *J* 8.8), 6.53 (1H, dt, *J* 16.0, 1.8), 6.28 (1H, dt, *J* 15.9, 5.3), 4.63 (2H, s), 4.20 (2H, dd, *J* 5.3, 1.7), 3.63 (2H, s)

^{13}C{^{1}H} NMR (126 MHz, CDCl$_3$) δ_C: 148.7, 138.5, 136.8, 131.7, 129.0, 128.8, 128.7, 127.7, 127.1, 126.6, 126.5, 125.1, 118.7, 117.4, 112.9, 54.1, 52.8, 22.7

HRMS: (ESI$^+$) $C_{24}H_{22}N_2Na$ [M+Na]$^+$ requires 361.1675, found 361.1672 (-0.8 ppm)

***N*-Cinnamyl-3,4,5-trifluoroaniline (SI-28)**

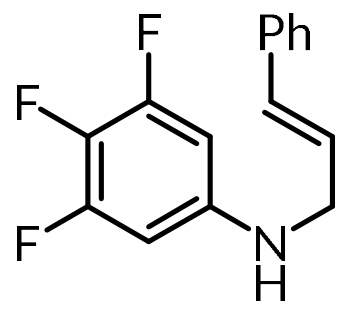

Following general procedure **A**, 3,4,5-trifluoroaniline (735 mg, 5.00 mmol), *trans*-cinnamaldehyde (0.63 mL, 5.00 mmol), acetic acid (0.29 mL, 5.00 mmol), sodium triacetoxyborohydride (2.12 g, 10.00 mmol) and CH_2Cl_2 (16.7 mL), after purification by column chromatography (hexane/Et_2O, 99:1 → 95:5), gave the title compound **SI-28** (923 mg, 70%) as a yellow oil.

ν_{max} (film) / cm^{-1}: 3026 (N–H), 1645 (C=C), 1526, 1231, 1179, 1036, 964, 735, 691

^{1}H NMR (500 MHz, CDCl$_3$) δ_H: 7.41 – 7.37 (2H, m), 7.37 – 7.32 (2H, m), 7.29 – 7.25 (1H, m), 6.61 (1H, dt, *J* 15.9, 1.7), 6.30 – 6.16 (3H, m), 3.94 – 3.89 (1H, m), 3.86 (2H, td, *J* 5.7, 1.6)

^{13}C{^{1}H} NMR (126 MHz, CDCl$_3$) δ_C: 152.1 (ddd, *J* 245.3, 10.3, 6.1), 144.0 (td, *J* 11.3, 2.5), 136.6, 132.5 (dt, *J* 239.3, 15.7), 132.3, 128.8, 128.0, 126.5, 125.6, 97.1 – 96.0 (m), 46.3

^{19}F NMR (471 MHz, CDCl$_3$) δ_F: -134.8 – -134.9 (m), -176.0 (tt, *J* 21.6, 5.7)

HRMS: (ESI$^-$) $C_{15}H_{11}F_3N$ [M-H]$^-$ requires 262.0849, found 262.0847 (-0.8 ppm)

***N*-Benzyl-*N*-cinnamyl-3,4,5-trifluoroaniline (1bj)**

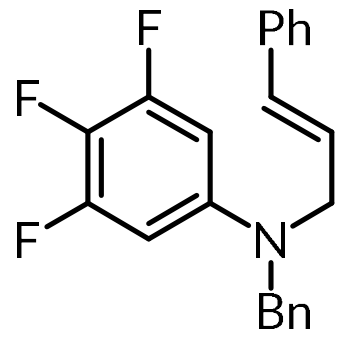

Following general procedure **C**, **SI-28** (895 mg, 3.40 mmol), benzyl bromide (0.45 mL, 3.74 mmol), K_2CO_3 (517 mg, 3.74 mmol), and acetone (13.6 mL) for 72 h, after purification by column chromatography (hexane/Et_2O, 98:2), gave the title compound **1bj** (1.155 g, 96%) as a colourless oil.

ν_{max} (film) / cm^{-1}: 3028, 1645 (C=C), 1526, 1234, 1167, 1038, 964, 806, 773, 729, 692

^{1}H NMR (500 MHz, CDCl$_3$) δ_H: 7.43 – 7.20 (10H, m), 6.52 (1H, dt, *J* 15.9, 1.8), 6.36 – 6.28 (2H, m), 6.22 (1H, dt, *J* 15.9, 5.3), 4.56 (2H, s), 4.14 (2H, dd, *J* 5.4, 1.7)

^{13}C{^{1}H} NMR (126 MHz, CDCl$_3$) δ_C: 152.0 (ddd, *J* 244.3, 10.1, 6.4), 145.0 (td, *J* 11.2, 1.8), 137.6, 136.5, 132.1, 132.0 (dt, *J* 239.4, 15.8), 129.0, 128.8, 127.9, 127.4, 126.5 (2C), 124.1, 96.6 – 96.0 (m), 54.3, 53.1

Note: 126.5 ppm peak contains two carbon peaks.

^{19}F NMR (471 MHz, CDCl$_3$) δ_F: -134.5 – -134.6 (m), -176.8 (tt, *J* 21.9, 5.7)

HRMS: (ESI$^+$) $C_{22}H_{17}F_3N$ [M-H]$^+$ requires 352.1308, found 352.1307 (-0.3 ppm)

***N*-Cinnamyl-[1,1'-biphenyl]-3-amine (SI-29)**

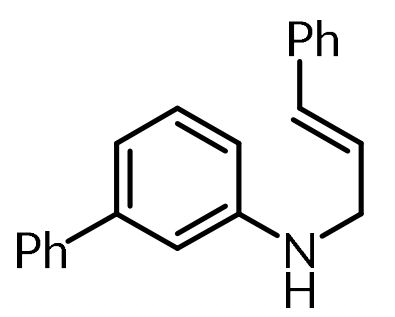

Following general procedure **A**, [1,1'-biphenyl]-3-amine (846 mg, 5.00 mmol), *trans*-cinnamaldehyde (0.63 mL, 5.00 mmol), acetic acid (0.29 mL, 5.00 mmol), sodium triacetoxyborohydride (2.12 g, 10.00 mmol) and CH_2Cl_2 (16.7 mL), after purification by column chromatography (hexane/Et_2O, 98:2 → 95:5), gave the title compound **SI-29** (1.21 g, 85%) as a yellow oil.

ν_{max} (film) / cm^{-1}: 3410 (N–H), 3024, 1597, 1481, 964, 752, 691

^{1}H NMR (500 MHz, $CDCl_3$) δ_H: 7.62 – 7.58 (2H, m), 7.47 – 7.39 (4H, m), 7.37 – 7.32 (3H, m), 7.31 – 7.24 (2H, m), 6.99 (1H, ddd, *J* 7.6, 1.7, 1.0), 6.91 (1H, t, *J* 2.1), 6.73 – 6.64 (2H, m), 6.38 (1H, dt, *J* 15.8, 5.8), 4.08 – 3.96 (3H, m)

^{13}C{^{1}H} NMR (126 MHz, $CDCl_3$) δ_C: 148.4, 142.6, 141.8, 136.9, 131.8, 129.8, 128.75, 128.72, 127.7, 127.3 (2C), 127.0, 126.5, 117.0, 112.2, 112.1, 46.4

Note: 127.3 ppm peak contains two carbon peaks.

HRMS: (ESI$^+$) $C_{21}H_{20}N$ [M+H]$^+$ requires 286.1590, found 286.1588 (-0.7 ppm)

_N_-Benzyl-_N_-cinnamyl-[1,1'-biphenyl]-3-amine (1bk)

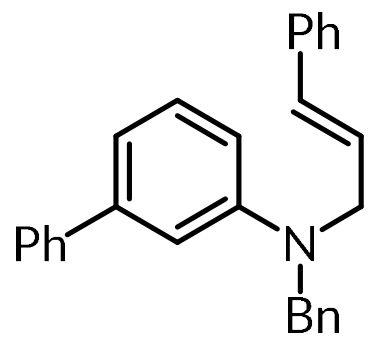

Following general procedure **C**, **SI-29** (1.17 g, 4.10 mmol), benzyl bromide (0.54 mL, 4.51 mmol), K_2CO_3 (623 mg, 4.51 mmol), and acetone (16.4 mL) for 48 h, after purification by column chromatography (hexane/Et_2O, 97:3), gave the title compound **1bk** (1.28 g, 83%) as a colourless oil.

ν_{max} (film) / cm^{-1}: 3026, 1595, 1568, 1485, 1352, 1213, 959, 752

^{1}H NMR (500 MHz, $CDCl_3$) δ_H: 7.59 – 7.54 (2H, m), 7.46 – 7.24 (14H, m), 7.04 (1H, t, *J* 2.1), 6.98 (1H, dt, *J* 7.5, 1.1), 6.82 (1H, dd, *J* 8.3, 2.6), 6.64 – 6.54 (1H, m), 6.35 (1H, dt, *J* 15.9, 5.3), 4.70 (2H, s), 4.26 (2H, dd, *J* 5.4, 1.7)

^{13}C{^{1}H} NMR (126 MHz, $CDCl_3$) δ_C: 149.5, 142.5, 142.2, 138.9, 136.9, 131.7, 129.7, 128.8, 128.72, 128.69, 127.6, 127.4, 127.2, 127.1, 126.8, 126.5, 125.5, 116.0, 111.7, 111.5, 54.1, 52.8

HRMS: (ESI$^+$) $C_{28}H_{26}N$ [M+H]$^+$ requires 376.2060, found 376.2057 (+0.8 ppm)

3-(*Tert*-butyl)-*N*-cinnamylaniline (SI-30)

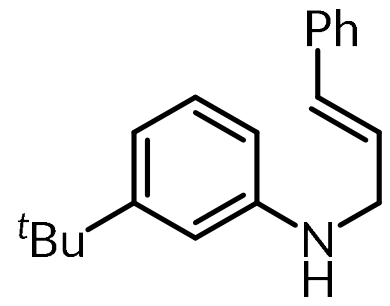

Following general procedure **A**, 3-(tert-butyl)aniline (0.79 mL, 5.00 mmol), *trans*-cinnamaldehyde (0.63 mL, 5.00 mmol), acetic acid (0.29 mL, 5.00 mmol), sodium triacetoxyborohydride (2.12 g, 10.00 mmol) and CH_2Cl_2 (16.7 mL), after purification by column chromatography (hexane/Et_2O, 98:2 → 95:5), gave the title compound **SI-30** (1.06 g, 80%) as an orange oil.

ν_{max} (film) / cm^{-1}: 3410 (N–H), 2961, 1603, 1585, 1481, 964, 775, 735

^{1}H NMR (500 MHz, $CDCl_3$) δ_H: 7.43 – 7.38 (2H, m), 7.36 – 7.31 (2H, m), 7.28 – 7.23 (1H, m), 7.17 (1H, t, *J* 7.9), 6.81 (1H, ddd, *J* 7.8, 1.8, 0.9), 6.74 (1H, t, *J* 2.1), 6.67 (1H, dt, *J* 15.9, 1.7), 6.56 – 6.51 (1H, m), 6.37 (1H, dt, *J* 15.9, 5.9), 3.97 (2H, dd, *J* 6.0, 1.6), 3.83 (1H, br s), 1.33 (9H, s)

^{13}C{^{1}H} NMR (126 MHz, $CDCl_3$) δ_C: 152.4, 148.0, 137.0, 131.7, 129.1, 128.7, 127.6, 127.4, 126.5, 115.2, 110.9, 110.1, 46.6, 34.8, 31.5

HRMS: (ESI$^+$) $C_{19}H_{24}N$ [M+H]$^+$ requires 266.1903, found 266.1900 (-1.1 ppm)

***N*-Benzyl-3-(*tert*-butyl)-N-cinnamylaniline (1bl)**

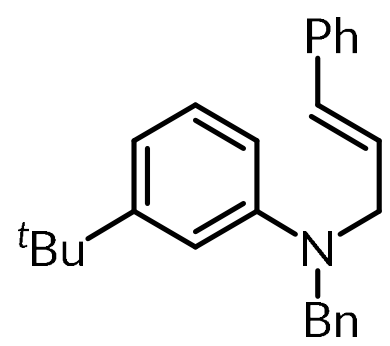

Following general procedure **C**, **SI-30** (1.06 g, 4.00 mmol), benzyl bromide (0.52 mL, 4.40 mmol), K_2CO_3 (608 mg, 4.40 mmol), and acetone (16.0 mL) for 48 h, after purification by column chromatography (hexane/Et_2O, 97:3), gave the title compound **1bl** (1.38 g, 97%) as a yellow oil.

ν_{max} (film) / cm^{-1}: 2961, 1599, 1495, 1449, 1362, 959, 729

^{1}H NMR (400 MHz, $CDCl_3$) δ_H: 7.45 – 7.26 (10H, m), 7.22 (1H, t, *J* 8.0), 6.92 (1H, dd, *J* 2.6, 1.8), 6.85 (1H, ddd, *J* 7.8, 1.8, 0.9), 6.71 (1H, ddd, *J* 8.2, 2.6, 0.9), 6.62 (1H, dt, *J* 15.8, 1.7), 6.36 (1H, dt, *J* 15.9, 5.5), 4.67 (2H, s), 4.25 (2H, dd, *J* 5.5, 1.7), 1.34 (9H, s)

^{13}C{^{1}H} NMR (101 MHz, $CDCl_3$) δ_C: 152.2, 149.0, 139.4, 137.1, 131.7, 128.9, 128.69, 128.66, 127.5, 126.94, 126.92, 126.5, 126.1, 114.2, 110.3, 110.2, 54.4, 53.0, 34.9, 31.5

HRMS: (ESI$^+$) $C_{26}H_{30}N$ [M+H]$^+$ requires 356.2373, found 356.2368 (-1.4 ppm)

***N*-Cinnamyl-3-methoxyaniline (SI-31)**

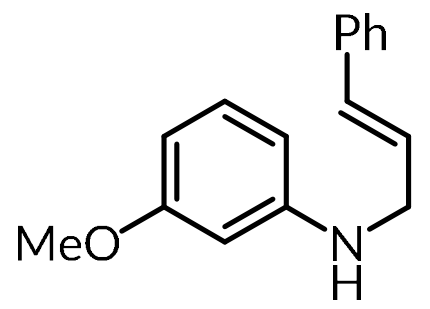

Following general procedure **A**, 3-methoxyaniline (0.56 mL, 5.00 mmol), *trans*-cinnamaldehyde (0.63 mL, 5.00 mmol), acetic acid (0.29 mL, 5.00 mmol), sodium triacetoxyborohydride (2.12 g, 10.00 mmol) and CH_2Cl_2 (16.7 mL), after purification by column chromatography (hexane/Et_2O, 95:5 → 90:10), gave the title compound **SI-31** (1.14 g, 95%) as a yellow oil.

ν_{max} (film) / cm^{-1}: 3404 (N–H), 3024, 2833, 1612 (C=C), 1495, 1207, 1159, 964, 741

^{1}H NMR (500 MHz, $CDCl_3$) δ_H: 7.41 – 7.36 (2H, m), 7.35 – 7.30 (2H, m), 7.28 – 7.22 (1H, m), 7.11 (1H, t, *J* 8.1), 6.63 (1H, dt, *J* 15.9, 1.6), 6.38 – 6.29 (3H, m), 6.24 (1H, t, *J* 2.3), 3.94 (2H, dd, *J* 5.9, 1.6), 3.88 (1H, br s), 3.79 (3H, s)

^{13}C{^{1}H} NMR (126 MHz, $CDCl_3$) δ_C: 161.0, 149.6, 137.0, 131.7, 130.1, 128.7, 127.7, 127.0, 126.5, 106.3, 102.8, 99.2, 55.2, 46.3

HRMS: (ESI$^+$) $C_{16}H_{16}NO$ [M-H]$^+$ requires 238.1226, found 238.1228 (+0.8 ppm)

***N*-Benzyl-*N*-cinnamyl-3-methoxyaniline (1bm)**

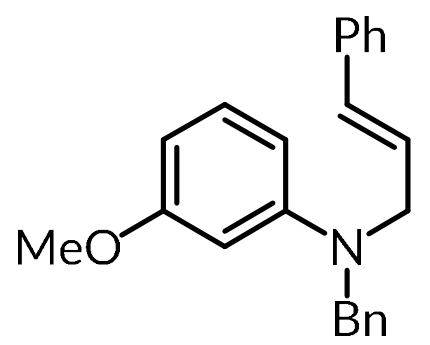

Following general procedure **C**, **SI-31** (1.10 g, 4.60 mmol), benzyl bromide (0.60 mL, 5.06 mmol), K_2CO_3 (699 mg, 5.06 mmol), and acetone (18.4 mL) for 24 h, after purification by column chromatography (hexane/Et_2O, 99:1 → 98:2), gave the title compound **1bm** (1.17 g, 77%) as a white solid.

mp 97 – 99 °C

ν_{max} (film) / cm^{-1}: 3024, 2833, 1609, 1495, 1450, 1200, 1163, 959, 729

^{1}H NMR (500 MHz, $CDCl_3$) δ_H: 7.44 – 7.22 (10H, m), 7.15 (1H, t, *J* 8.2), 6.55 (1H, d, *J* 15.9), 6.45 (1H, dd, *J* 8.2, 2.5), 6.39 (1H, t, *J* 2.5), 6.36 – 6.26 (2H, m), 4.63 (2H, s), 4.19 (2H, d, *J* 5.6), 3.78 (3H, s)

^{13}C{^{1}H} NMR (126 MHz, $CDCl_3$) δ_C: 160.9, 150.5, 138.9, 136.9, 131.5, 130.1, 128.74, 128.66, 127.6, 127.0, 126.7, 126.5, 125.5, 105.8, 101.5, 99.2, 55.2, 54.0, 52.7

HRMS: (ESI$^+$) $C_{23}H_{22}NO$ [M-H]$^+$ requires 328.1696, found 328.1699 (+0.9 ppm)

_N_-Cinnamyl-3-fluoroaniline (SI-32)

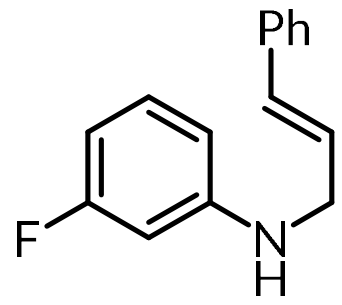

Following general procedure **A**, 3-fluoroaniline (0.48 mL, 5.00 mmol), *trans*-cinnamaldehyde (0.63 mL, 5.00 mmol), acetic acid (0.29 mL, 5.00 mmol), sodium triacetoxyborohydride (2.12 g, 10.00 mmol) and CH_2Cl_2 (16.7 mL), after purification by column chromatography (hexane/Et_2O, 98:2 → 95:5), gave the title compound **SI-32** (830 mg, 73%) as a colourless oil.

ν_{max} (film) / cm^{-1}: 3417 (N–H), 3026, 1618 (C=C), 1585, 1493, 1148, 962, 827, 741

^{1}H NMR (500 MHz, CDCl$_3$) δ_H: 7.40 – 7.37 (2H, m), 7.33 (2H, dd, *J* 8.5, 6.8), 7.28 – 7.23 (1H, m), 7.11 (1H, td, *J* 8.2, 6.7), 6.63 (1H, dt, *J* 15.9, 1.7), 6.44 – 6.39 (2H, m), 6.36 (1H, dt, *J* 11.6, 2.3), 6.31 (1H, dt, *J* 15.9, 5.8), 3.99 (1H, br s), 3.94 – 3.92 (2H, m)

^{13}C{^{1}H} NMR (126 MHz, CDCl$_3$) δ_C: 164.3 (d, *J* 242.7), 149.9 (d, *J* 10.8), 136.8, 132.0, 130.4 (d, *J* 10.3), 128.7, 127.8, 126.5, 126.5, 109.0 (d, *J* 2.4), 104.1 (d, *J* 21.5), 99.8 (d, *J* 25.3), 46.2

^{19}F NMR (470 MHz, CDCl$_3$) δ_F: -112.7 – -112.8 (m)

HRMS: (ESI$^+$) $C_{15}H_{15}FN$ [M+H]$^+$ requires 228.1183, found 228.1182 (-0.4 ppm)

_N_-Benzyl-_N_-cinnamyl-3-fluoroaniline (1bn)

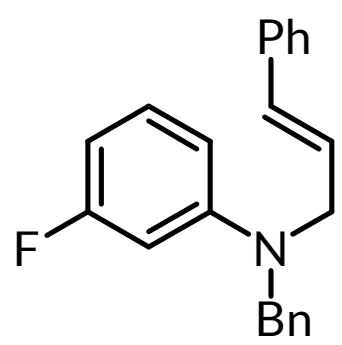

Following general procedure **C**, **SI-32** (750 mg, 3.30 mmol), benzyl bromide (0.43 mL, 3.63 mmol), K_2CO_3 (502 mg, 3.63 mmol), and acetone (13.2 mL) for <u>48 h</u>, after purification by column chromatography (hexane/Et_2O, 99:1), gave the title compound **1bn** (978 mg, 93%) as a white solid.

mp 39 – 41 °C

ν_{max} (film) / cm^{-1}: 3026, 1618, 1495, 1354, 1155, 961, 820, 729

^{1}H NMR (500 MHz, CDCl$_3$) δ$_H$: 7.40 – 7.23 (10H, m), 7.17 – 7.10 (1H, m), 6.56 – 6.50 (2H, m), 6.48 (1H, dt, *J* 12.8, 2.4), 6.42 (1H, td, *J* 8.2, 2.4), 6.27 (1H, dt, *J* 15.9, 5.3), 4.61 (2H, s), 4.18 (2H, dd, *J* 5.4, 1.7)

^{13}C{^{1}H} NMR (126 MHz, CDCl$_3$) δ$_C$: 164.3 (d, *J* 242.0), 150.8 (d, *J* 10.6), 138.3, 136.8, 131.8, 130.4 (d, *J* 10.3), 128.9, 128.7, 127.7, 127.2, 126.6, 126.5, 124.9, 108.1 (d, *J* 2.2), 103.2 (d, *J* 21.5), 99.6 (d, *J* 26.2), 54.0, 52.8

^{19}F NMR (471 MHz, CDCl$_3$) δ$_F$: -112.3 – -112.4 (m)

HRMS: (ESI$^+$) $C_{22}H_{21}FN$ [M+H]$^+$ requires 318.1653, found 318.1650 (-0.9 ppm)

***N*-Cinnamyl-3-(trifluoromethyl)aniline (SI-33)**

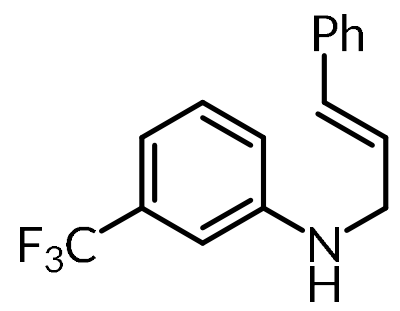

Following general procedure **A**, 3-(trifluoromethyl)aniline (0.62 mL, 5.00 mmol), *trans*-cinnamaldehyde (0.63 mL, 5.00 mmol), acetic acid (0.29 mL, 5.00 mmol), sodium triacetoxyborohydride (2.12 g, 10.00 mmol) and CH_2Cl_2 (16.7 mL), after purification by column chromatography (hexane/Et_2O, 98:2 → 95:5), gave the title compound **SI-33** (826 mg, 60%) as a colourless oil.

ν$_{max}$ (film) / cm^{-1}: 3419 (N–H), 3026, 1614 (C=C), 1495, 1339, 1161, 1113, 1067, 964, 718

^{1}H NMR (500 MHz, CDCl$_3$) δ$_H$: 7.42 – 7.37 (2H, m), 7.36 – 7.30 (2H, m), 7.30 – 7.23 (2H, m), 6.97 (1H, d, *J* 7.6), 6.87 (1H, t, *J* 2.1), 6.80 (1H, dd, *J* 8.1, 2.4), 6.64 (1H, dt, *J* 15.8, 1.7), 6.31 (1H, dt, *J* 15.9, 5.7), 4.06 (1H, br s), 3.97 (2H, d, *J* 5.8)

^{13}C{^{1}H} NMR (126 MHz, CDCl$_3$) δ$_C$: 148.3, 136.7, 132.2, 131.7 (q, *J* 31.7), 129.8, 128.8, 127.9, 126.5, 126.2, 124.5 (q, *J* 272.3), 116.0, 114.1 (q, *J* 3.9), 109.3 (q, *J* 3.9), 46.1

^{19}F NMR (471 MHz, CDCl$_3$) δ$_F$: -62.8 (s)

HRMS: (ESI$^+$) $C_{16}H_{13}F_3N$ [M-H]$^+$ requires 276.0995, found 276.0996 (+0.4 ppm)

N-Benzyl-N-cinnamyl-3-(trifluoromethyl)aniline (1bo)

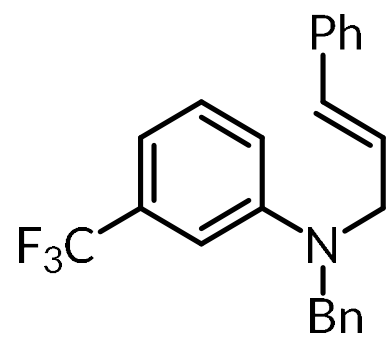

Following general procedure **B**, **SI-33** (776 mg, 2.80 mmol), benzyl bromide (0.37 mL, 3.08 mmol), NaH (168 mg, 4.20 mmol), and DMF (7.0 mL), after purification by column chromatography (hexane/CH_2Cl_2, 100:0 → 95:5 → 92:8), gave the title compound **1bo** (471 mg, 56%) as a white solid.

mp 64 – 66 °C

ν_{max} (film) / cm^{-1}: 3028, 1608 (C=C), 1495, 1450, 1317, 1161, 1115, 1074, 727, 691

^{1}H NMR (500 MHz, $CDCl_3$) δ_H: 7.39 – 7.21 (11H, m), 7.01 (1H, t, *J* 2.2), 6.96 (1H, d, *J* 7.6), 6.90 (1H, dd, *J* 8.4, 2.7), 6.53 (1H, dt, *J* 15.9, 1.8), 6.26 (1H, dt, *J* 15.9, 5.3), 4.65 (2H, s), 4.22 (2H, dd, *J* 5.4, 1.7)

^{13}C{^{1}H} NMR (126 MHz, $CDCl_3$) δ_C: 149.2, 138.1, 136.7, 132.0, 131.7 (d, *J* 31.4), 129.8, 128.9, 128.7, 127.8, 127.3, 126.7, 126.5, 124.59, 124.56 (d, *J* 272.5), 115.5, 113.2 (q, *J* 3.9), 108.7 (q, *J* 4.0), 54.0, 52.7

^{19}F NMR (471 MHz, $CDCl_3$) δ_F: -62.7 (s)

HRMS: (ESI$^+$) $C_{23}H_{19}F_3N$ [M-H]$^+$ requires 366.1464, found 366.1462 (-0.5 ppm)

N-Cinnamylnaphthalen-1-amine (SI-34)

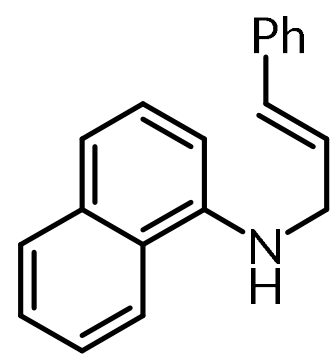

Following general procedure **A**, 1-naphthylamine (500 mg, 3.49 mmol), *trans*-cinnamaldehyde (0.44 mL, 3.49 mmol), acetic acid (0.20 mL, 3.49 mmol), sodium triacetoxyborohydride (1.48 g, 6.98 mmol) and CH_2Cl_2 (11.6 mL), after purification by column chromatography (hexane/CH_2Cl_2, 80:20 → 70:30 → 60:40), gave the title compound **SI-34** (615 mg, 68%) as a brown solid.

mp 82 – 84 °C

ν_{max} (film) / cm^{-1}: 3443 (N–H), 3055, 1582, 1526, 1408, 966, 770

^{1}H NMR (400 MHz, $CDCl_3$) δ_H: 7.91 – 7.79 (2H, m), 7.57 – 7.21 (9H, m), 6.82 – 6.66 (2H, m), 6.48 (1H, dt, *J* 15.9, 5.8), 4.55 (1H, br s), 4.13 (2H, d, *J* 5.7)

$^{13}C\{^{1}H\}$ NMR (101 MHz, $CDCl_3$) δ_C: 143.3, 136.9, 134.4, 132.1, 128.8, 128.7, 127.7, 126.8 (2C), 126.5, 125.9, 124.9, 123.6, 120.0, 117.8, 105.0, 46.5

Note: 127.8 ppm peak contains two carbon peaks.

HRMS: (ESI$^+$) $C_{19}H_{16}N$ $[M-H]^+$ requires 258.1277, found 258.1281 (+1.5 ppm)

N-Benzyl-*N*-cinnamylnaphthalen-1-amine (1bp)

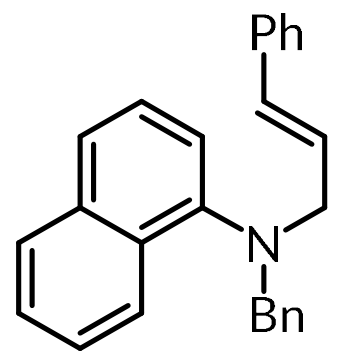

Following general procedure **C**, **SI-34** (500 mg, 1.93 mmol), benzyl bromide (0.25 mL, 2.12 mmol), K_2CO_3 (293 mg, 2.12 mmol), and acetone (7.7 mL), after purification by column chromatography (hexane/CH_2Cl_2, 80:20 → 70:30), gave the title compound **1bp** (323 mg, 48%) as a brown oil.

ν_{max} (film) / cm^{-1}: 3026, 2812, 1576, 1495, 1298, 966, 907, 775, 735, 692

^{1}H NMR (700 MHz, $CDCl_3$) δ_H: 8.59 – 8.47 (1H, m), 7.91 (1H, d, *J* 7.8), 7.63 – 7.52 (3H, m), 7.48 – 7.31 (9H, m), 7.31 – 7.24 (2H, m), 7.18 – 7.13 (1H, m), 6.60 – 6.51 (1H, m), 6.41 – 6.28 (1H, m), 4.48 (2H, s), 3.96 (2H, d, *J* 6.1)

$^{13}C\{^{1}H\}$ NMR (126 MHz, $CDCl_3$) δ_C: 147.8, 138.8, 137.2, 135.1, 132.8, 130.0, 128.63, 128.62, 128.5, 128.4, 127.5, 127.0, 126.48, 126.45, 125.9, 125.63, 125.58, 124.0, 123.6, 118.2, 56.9, 56.0

HRMS: (ESI$^+$) $C_{26}H_{24}N$ $[M+H]^+$ requires 350.1903, found 350.1901 (-0.6 ppm)

1-Cinnamylindoline (1bq)

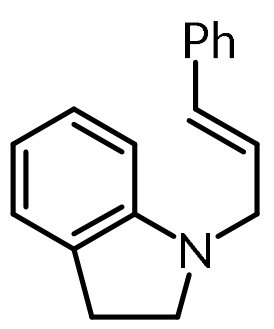

Following modified general procedure **B**, indoline (500 mg, 4.20 mmol), cinnamyl bromide (992 mg, 5.03 mmol), K_2CO_3 (871 mg, 6.30 mmol), NaI (6.3 mg, 0.042 mmol, 1 mol%), and DMF (10 mL) at RT, after purification by column chromatography (hexane/CH_2Cl_2, 90:10 → 80:20), gave the title compound **1bq** (575 mg, 58%) as a pale-yellow oil.

ν_{max} (film) / cm^{-1}: 3024, 2843, 1605, 1487, 1267, 966, 743, 692

^{1}H NMR (500 MHz, $CDCl_3$) δ_H: 7.43 – 7.37 (2H, m), 7.36 – 7.30 (2H, m), 7.28 – 7.23 (1H, m), 7.14 – 7.07 (2H, m), 6.70 (1H, td, *J* 7.4, 1.0), 6.67 – 6.62 (1H, m), 6.59 (1H, d, *J* 7.8), 6.33 (1H, dt, *J* 15.8, 6.2), 3.89 (2H, dd, *J* 6.2, 1.6), 3.41 (2H, t, *J* 8.3), 3.00 (2H, t, *J* 8.3)

^{13}C{^{1}H} NMR (126 MHz, $CDCl_3$) δ_C: 152.3, 137.0, 132.4, 130.4, 128.7, 127.6, 127.4, 126.5, 126.0, 124.6, 117.9, 107.5, 53.5, 51.7, 28.7

HRMS: (ESI$^+$) $C_{17}H_{16}N$ [M-H]$^+$ requires 234.1277, found 234.1279 (+0.9 ppm)

7-Chloro-1-cinnamyl-1,2,3,4-tetrahydro-5*H*-benzo[*b*]azepin-5-one (1br)

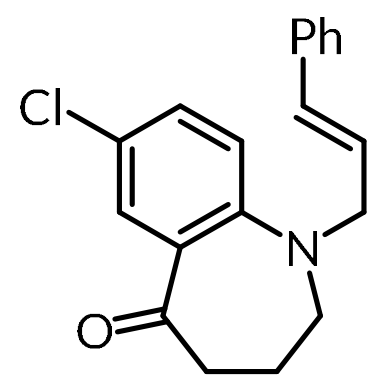

Following general procedure **A**, 7-chloro-1,2,3,4-tetrahydro-5H-benzo[b]azepin-5-one (978 mg, 5.00 mmol), *trans*-cinnamaldehyde (0.63 mL, 5.00 mmol), acetic acid (0.29 mL, 5.00 mmol), sodium triacetoxyborohydride (2.12 g, 10.00 mmol) and CH_2Cl_2 (16.7 mL), after purification by column chromatography (hexane/Et_2O, 80:20), gave the title compound **1br** (608 mg, 65%) as a yellow oil.

ν_{max} (film) / cm^{-1}: 2928, 2860, 1664 (C=O), 1593, 1551, 1485, 1206, 1161, 1125, 964, 806

^{1}H NMR (500 MHz, $CDCl_3$) δ_H: 7.72 (1H, d, *J* 2.7), 7.39 – 7.35 (2H, m), 7.35 – 7.30 (2H, m), 7.28 – 7.19 (2H, m), 6.88 (1H, d, *J* 8.9), 6.51 (1H, dt, *J* 16.0, 1.7), 6.26 (1H, dt, *J* 15.9, 5.5), 4.24 (2H, dd, *J* 5.6, 1.7), 3.33 (2H, t, *J* 6.6), 2.81 (2H, t, *J* 7.2), 2.26 (2H, p, *J* 7.0)

^{13}C{^{1}H} NMR (126 MHz, $CDCl_3$) δ_C: 201.9, 152.2, 136.4, 132.8, 132.2, 129.2, 128.8, 128.1, 127.7, 126.5, 124.6, 123.5, 116.4, 54.1, 53.8, 41.1, 31.8

HRMS: (ESI$^+$) $C_{19}H_{18}{}^{35}ClNONa$ [M+Na]$^+$ requires 334.0969, found 334.0968 (-0.3 ppm)

***N*-Cinnamyl-2-methylaniline (3b)**

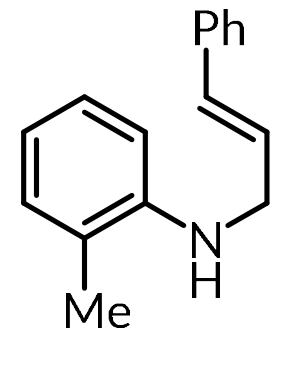

Following general procedure **A**, *o*-toluidine (0.51 mL, 5.00 mmol), *trans*-cinnamaldehyde (0.63 mL, 5.00 mmol), acetic acid (0.29 mL, 5.00 mmol), sodium triacetoxyborohydride (2.12 g, 10.00

mmol) and CH_2Cl_2 (16.7 mL), after purification by column chromatography (hexane/Et_2O, 100:0 → 99:1 → 98:2), gave the title compound **3b** (793 mg, 71%) as a white solid.

mp 36 – 38 °C

ν_{max} (film) / cm^{-1}: 3435 (N–H), 3024, 1605 (C=C), 1508, 1447, 1314, 1252, 964, 742

^{1}H NMR (500 MHz, $CDCl_3$) δ_H: 7.43 – 7.39 (2H, m), 7.36 – 7.31 (2H, m), 7.29 – 7.23 (1H, m), 7.15 (1H, td, *J* 7.7, 1.6), 7.11 – 7.08 (1H, m), 6.73 – 6.68 (2H, m), 6.66 (1H, dt, *J* 15.8, 1.6), 6.39 (1H, dt, *J* 15.8, 5.8), 4.01 (2H, dd, *J* 5.9, 1.6), 3.70 (1H, br s), 2.19 (3H, s)

^{13}C{^{1}H} NMR (126 MHz, $CDCl_3$) δ_C: 146.1, 137.0, 131.7, 130.2, 128.7, 127.7, 127.3, 127.2, 126.5, 122.2, 117.3, 110.2, 46.3, 17.7

HRMS: (ESI$^-$) $C_{16}H_{16}N$ $[M-H]^-$ requires 222.1288, found 222.1291 (+1.4 ppm)

2-Bromo-*N*-cinnamylaniline (3c)

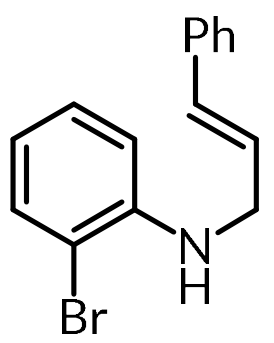

Following general procedure **A**, 2-bromoaniline (860 mg, 5.00 mmol), *trans*-cinnamaldehyde (0.63 mL, 5.00 mmol), acetic acid (0.29 mL, 5.00 mmol), sodium triacetoxyborohydride (2.12 g, 10.00 mmol) and CH_2Cl_2 (16.7 mL), after purification by column chromatography (hexane/Et_2O, 98:2), gave the title compound **3c** (743 mg, 52%) as a colourless oil.

ν_{max} (film) / cm^{-1}: 3410 (N–H), 3024, 1595, 1495, 1317, 1016, 962, 733

^{1}H NMR (500 MHz, $CDCl_3$) δ_H: 7.46 (1H, dd, *J* 7.9, 1.5), 7.42 – 7.37 (2H, m), 7.36 – 7.31 (2H, m), 7.28 – 7.23 (1H, m), 7.22 – 7.17 (1H, m), 6.71 (1H, dd, *J* 8.2, 1.5), 6.67 – 6.58 (2H, m), 6.34 (1H, dt, *J* 15.9, 5.7), 4.57 (1H, br s), 4.01 (2H, td, *J* 5.5, 1.7)

^{13}C{^{1}H} NMR (126 MHz, $CDCl_3$) δ_C: 144.9, 136.8, 132.5, 131.9, 128.7, 128.6, 127.8, 126.5, 126.4, 118.1, 111.8, 109.9, 46.1

HRMS: (ESI$^+$) $C_{15}H_{13}{}^{79}BrN$ $[M-H]^+$ require 286.0226, found 286.0226 (0.0 ppm)

N-Cinnamyl-2-(methylthio)aniline (3d)

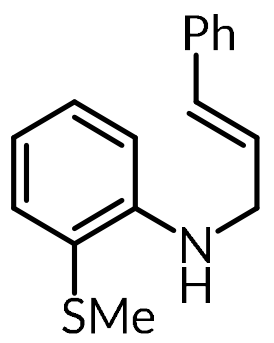

Following general procedure **A**, 2-(methylthio)aniline (0.63 mL, 5.00 mmol), *trans*-cinnamaldehyde (0.63 mL, 5.00 mmol), acetic acid (0.29 mL, 5.00 mmol), sodium triacetoxyborohydride (2.12 g, 10.00 mmol) and CH_2Cl_2 (16.7 mL), after purification by column chromatography (hexane/Et_2O, 98:2 → 95:5), gave the title compound **3d** (1.06 g, 83%) as a yellow oil.

ν_{max} (film) / cm^{-1}: 3368 (N–H), 3024, 2918, 1585, 1295, 1314, 964, 735, 691

^{1}H NMR (500 MHz, $CDCl_3$) δ_H: 7.45 – 7.37 (3H, m), 7.35 – 7.31 (2H, m), 7.27 – 7.23 (1H, m), 7.22 – 7.18 (1H, m), 6.71 – 6.67 (2H, m), 6.64 (1H, dt, *J* 15.9, 1.8), 6.36 (1H, dt, *J* 15.9, 5.7), 5.16 (1H, br s), 4.02 (2H, dd, *J* 5.7, 1.7), 2.37 (3H, s)

^{13}C{^{1}H} NMR (126 MHz, $CDCl_3$) δ_C: 148.1, 136.9, 134.1, 131.6, 129.5, 128.7, 127.7, 126.9, 126.5, 120.0, 117.3, 110.6, 46.1, 18.2

HRMS: (ESI$^+$) $C_{16}H_{18}NS$ [M+H]$^+$ requires 256.1154, found 256.1155 (+0.4 ppm)

N-Cinnamyl-2-methoxyaniline (3e)

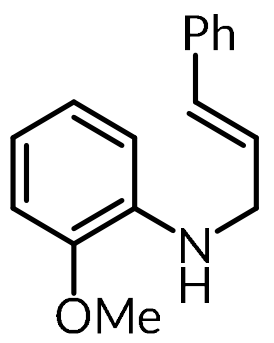

Following general procedure **A**, 2-methoxyaniline (500 mg, 4.06 mmol), *trans*-cinnamaldehyde (0.51 mL, 4.06 mmol), acetic acid (0.23 mL, 4.06 mmol), sodium triacetoxyborohydride (1.72 g, 8.12 mmol) and CH_2Cl_2 (13.5 mL), after purification by column chromatography (hexane/CH_2Cl_2, 80:20 → 70:30 → 60:40), gave the title compound **3e** (795 mg, 82%) as a white solid.

mp 57 – 59 °C

ν_{max} (film) / cm^{-1}: 3422 (N–H), 3024, 1601, 1512, 1246, 1221, 1030, 733

^{1}H NMR (500 MHz, $CDCl_3$) δ_H: 7.41 – 7.37 (2H, m), 7.34 – 7.29 (2H, m), 7.26 – 7.20 (1H, m), 6.94 – 6.84 (1H, m), 6.82 – 6.78 (1H, m), 6.74 – 6.66 (2H, m), 6.64 (1H, dd, *J* 15.9, 1.6), 6.64 (1H, dt, *J* 15.9, 5.8), 4.44 (1H, br s), 3.97 (2H, dd, *J* 5.8, 1.6), 3.87 (3H, s)

$^{13}C\{^1H\}$ NMR (126 MHz, $CDCl_3$) δ_C: 147.0, 138.1, 137.1, 131.5, 128.7, 127.6, 127.4, 126.5, 121.4, 116.8, 110.4, 109.5, 55.5, 46.1

HRMS: (ESI^+) $C_{16}H_{18}NO$ $[M+H]^+$ requires 240.1383, found 240.1382 (-0.4 ppm)

N-Cinnamyl-4-methoxy-2-methylaniline (3f)

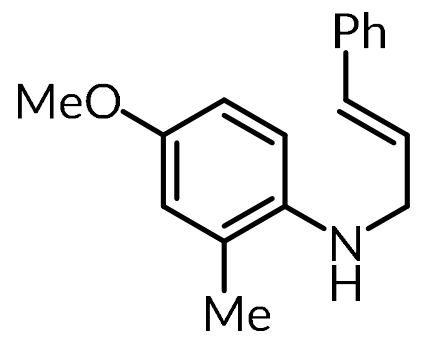

Following general procedure **A**, 4-methoxy-2-methylaniline (0.64 mL, 5.00 mmol), *trans*-cinnamaldehyde (0.63 mL, 5.00 mmol), acetic acid (0.29 mL, 5.00 mmol), sodium triacetoxyborohydride (2.12 g, 10.00 mmol) and CH_2Cl_2 (16.7 mL), after purification by column chromatography (hexane/Et_2O, 99:2 → 90:10), gave the title compound **3f** (993 mg, 78%) as a yellow solid.

mp 53 – 55 °C

ν_{max} (film) / cm^{-1}: 3404 (N–H), 3024, 2830, 2359, 1506, 1287, 1221, 1047, 964, 745

^1H NMR (500 MHz, $CDCl_3$) δ_H: 7.43 – 7.39 (2H, m), 7.37 – 7.32 (2H, m), 7.28 – 7.24 (1H, m), 6.77 – 6.71 (2H, m), 6.69 – 6.63 (2H, m), 6.41 (1H, dt, *J* 15.9, 5.8), 3.96 (2H, dd, *J* 6.0, 1.6), 3.77 (3H, s), 3.41 (1H, br s), 2.20 (3H, s)

$^{13}C\{^1H\}$ NMR (126 MHz, $CDCl_3$) δ_C: 151.9, 140.4, 137.0, 131.6, 128.7, 127.64, 127.55, 126.4, 124.1, 117.1, 111.7, 111.4, 55.9, 47.1, 17.9

HRMS: (ESI^+) $C_{17}H_{18}NO$ $[M-H]^+$ requires 252.1383, found 252.1384 (+0.4 ppm)

Ethyl (*E*)-4-(cinnamyl(phenyl)amino)but-2-enoate (5)

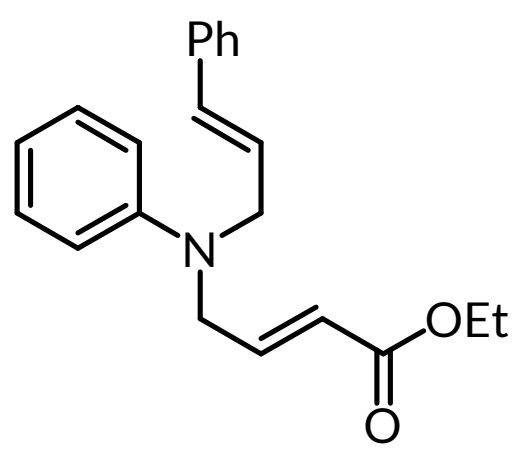

Following general procedure **C**, **3a** (0.52 g, 2.50 mmol), ethyl (*E*)-4-bromobut-2-enoate (0.33 mL, 2.75 mmol), K_2CO_3 (0.38 g, 2.75 mmol), and acetone (10 mL) for 16 h, after purification by column

chromatography (hexane/CH_2Cl_2, 100:0 → 99:1 → 98:2 → 97:3 → 96:4 → 95:5), gave the title compound **5** (0.75 g, 90%) as a pale-yellow solid.

mp 60 – 63 °C

ν_{max}(film) / cm^{-1}: 1715 (C=O), 1659 (C=C), 1599, 1504, 1368, 1294, 1273, 1173, 1038, 966, 746, 692

^{1}H NMR (500 MHz, $CDCl_3$) δ_H: 7.39 – 7.33 (2H, m), 7.33 – 7.28 (2H, m), 7.25 – 7.19 (3H, m), 7.00 (1H, dt, *J* 15.7, 4.3), 6.78 – 6.70 (3H, m), 6.52 (1H, dt, *J* 15.9, 1.7), 6.25 (1H, dt, *J* 15.9, 5.3), 5.98 (1H, dt, *J* 15.6, 1.9), 4.19 (2H, q, *J* 7.1), 4.12 (2H, dd, *J* 4.4, 2.0), 4.10 (2H, dd, *J* 5.4, 1.7), 1.28 (3H, t, *J* 7.1)

^{13}C{^{1}H} NMR (126 MHz, $CDCl_3$) δ_C: 166.4, 148.3, 144.7, 136.8, 131.7, 129.5, 128.7, 127.7, 126.5, 125.4, 122.1, 117.2, 112.6, 60.6, 52.7, 51.3, 14.4

HRMS: (ESI$^+$) $C_{21}H_{23}NO_2Na$ [M+Na]$^+$ requires 344.1621, found 344.1620 (-0.3 ppm)

***N*-Cinnamyl-4-methoxy-*N*-(4-(trifluoromethyl)phenyl)aniline (7)**

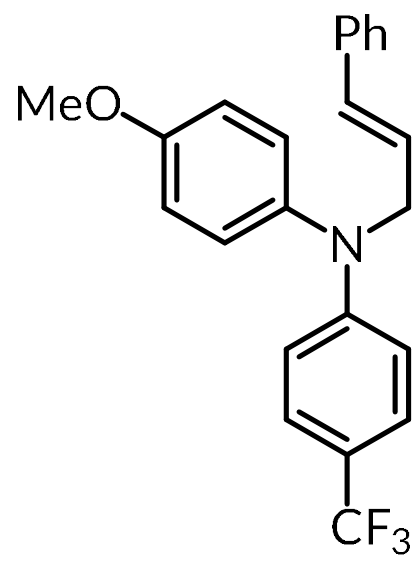

Prepared according to literature.[7] A 2-neck RBF was charged with **SI-24** (1.11 g, 4.00 mmol), ^{t}BuOK (583 mg, 5.20 mmol), $Pd(dba)_2$ (115 mg, 0.20 mmol, 5 mol%), and [(^{t}Bu$_3$)PH]BF_4 (116 mg, 0.40 mmol, 10 mol%) and back re-filled with N2 (× 3). De-gassed anhydrous PhMe (8.0 mL, 0.5 M) was added followed by de-gassed 4-bromoanisole (0.75 mL, 6.00 mmol). The reaction was stirred at reflux for 24 h. The reaction mixture was cooled to RT and water (25 mL) was added. The layers were separated and the aqueous layer was extracted with EtOAc (× 3). The combined organic layers were washed with brine, dried over Na_2SO_4, filtered, and concentrated under reduced pressure. Purification via column chromatography (hexane/CH_2Cl_2, 95:5 → 90:10 → 85:15 → 80:20) gave the title compound **7** (900 mg, 59%) as a beige solid.

mp 87 – 89 °C

ν_{max}(film) / cm^{-1}: 1614 (C=C), 1508, 1323, 1244, 1103, 1065, 824

^{1}H NMR (500 MHz, $CDCl_3$) δ_H: 7.39 – 7.27 (6H, m), 7.25 – 7.21 (1H, m), 7.19 (2H, d, *J* 8.8), 6.95 (2H, d, *J* 8.8), 6.73 (2H, d, *J* 8.6), 6.57 (1H, d, *J* 15.9), 6.31 (1H, dt, *J* 16.0, 5.4), 4.46 (2H, dd, *J* 5.5, 1.7), 3.84 (3H, s)

$^{13}C\{^1H\}$ NMR (126 MHz, $CDCl_3$) δ_C: 157.9, 151.3, 139.3, 136.8, 132.0, 128.9, 128.7, 127.8, 126.5, 126.4 (q, *J* 3.7), 125.1 (q, *J* 270.3) 125.0, 118.9 (q, *J* 32.7), 115.4, 113.3, 55.6, 54.9

^{19}F NMR (471 MHz, $CDCl_3$) δ_F: -61.0 (s)

HRMS: (ESI^-) $C_{14}H_{11}F_3NO$ $[M-C_9H_9]^-$ requires 266.0798, found 266.0798 (0.0 ppm)

2.3 Reaction Optimisation and Deviations

Table S1: Reaction Deviations

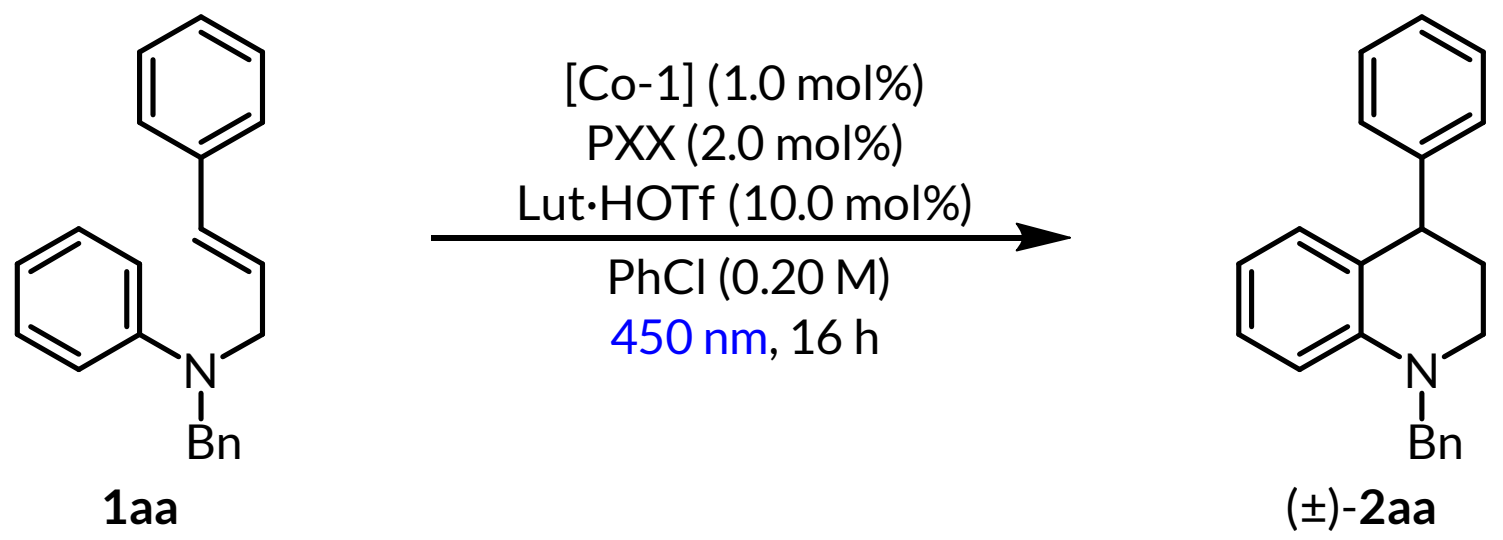

Entry	Deviations	2aa / %
**1**	-	99
**2**	CH_2Cl_2	90
**3**	$PhCF_3$	58
**4**	PhF	46
**5**	THF	32
**6**	Acetone	84
**7**	EtOAc	63
**8**	$Ir(ppy)_3$	99
**9**	$Ru(bpy)_3Cl_2$	0
**10**	PC-2	42
**11**	PC-3	58
**12**	4CzIPN	0
**13**	(±)-[Co-2]	89
**14**	(±)-[Co-3]	83
**17**	Col·HOTf	90
**18**	Py·HOTf	0
**19**	PTSA	0
**20**	Ascorbic acid	0
**21**	5 mol% Lut·HOTf	30
**22**	0.5 mol% [Co-1] and 1.0 mol% PXX	95
**23**	2.0 mL PhCl (0.10 M)	99
**24**	0.5 mL PhCl (0.40 M)	99
**25**	No degassing	15
**26**	456 nm Kessil	99
**27**	425 nm	92
**28**	475 nm	34
**29**	No PXX	0
**30**	No [Co-1]	0
**31**	No Lut·HOTf	0
**32**	No light	0

Table S2: Reaction Optimisation for NH Substrates

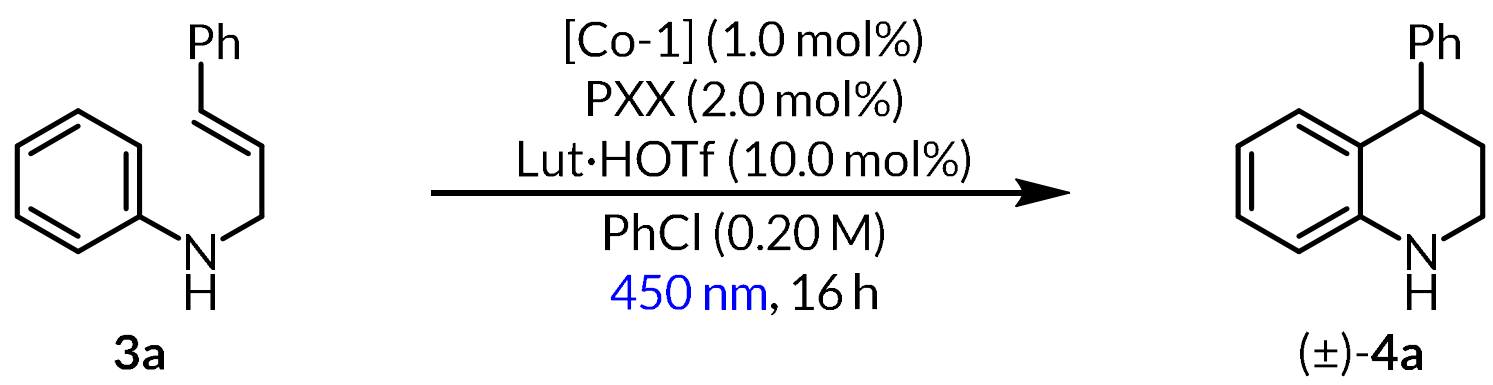

Entry	Deviations	3a / %	4a / %
**1**	-	46	53
**2**	PXX 3 mol%	46	52
**3**	PXX 5 mol%	12	50
**4**	2 mol% Co-1 and 4 mol% PXX	27	61
**5**	20 mol% Lut·HOTf	60	40
**6**	48 h	50	49
**7**	CH_2Cl_2	55	35
**8**	Acetone	83	8
**9**	Col·HOTf	11	60
**10**	Two 450 nm lamps	11	60
**11**	Two 450 nm lamps for 24 h	0	75

2.4 Reaction Scope

General procedure E: Hydroarylation of *N*-substituted substrates

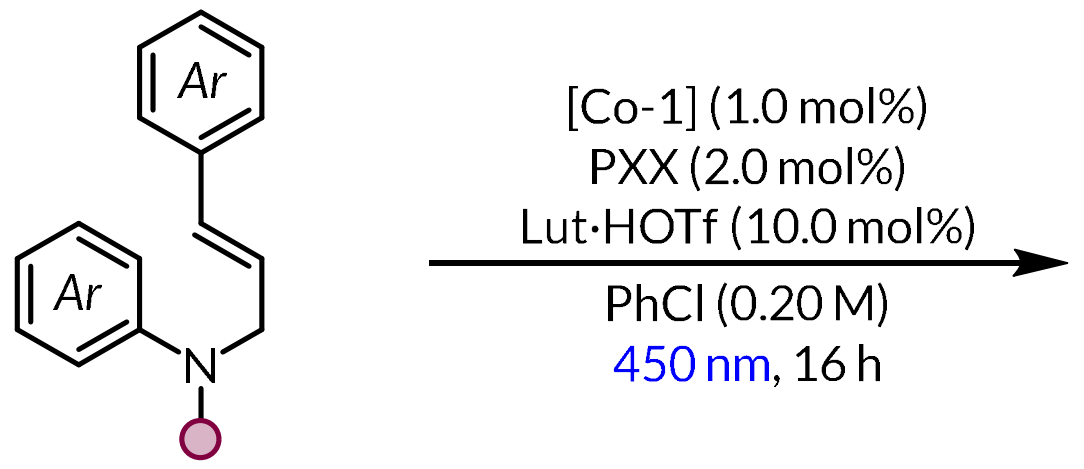

A 4-mL vial was charged with [Co-1] (1.2 mg, 0.002 mmol, 1.0 mol%), PXX (1.1 mg, 0.004 mmol, 2.0 mol%), Lut·HOTf (5.1 mg, 0.02 mmol, 10 mol%), and appropriate alkene (0.20 mmol). The vial was capped with a rubber septum and evacuated/backfilled with nitrogen (× 3). PhCl (1.0 mL, 0.20 M) was then added, and the reaction mixture was degassed by bubbling nitrogen through the solution for 15 mins. The reaction was irradiated by one 450 nm lamp (HCK1012-01-002 at a 10 mm distance, see below) for 16 h. 1,2-Dibromoethane (17.2 µL, 0.20 mmol) was added as the internal standard to the crude reaction mixture to determine the yield via ^{1}H-NMR. The crude product was purified by silica-gel column chromatography.

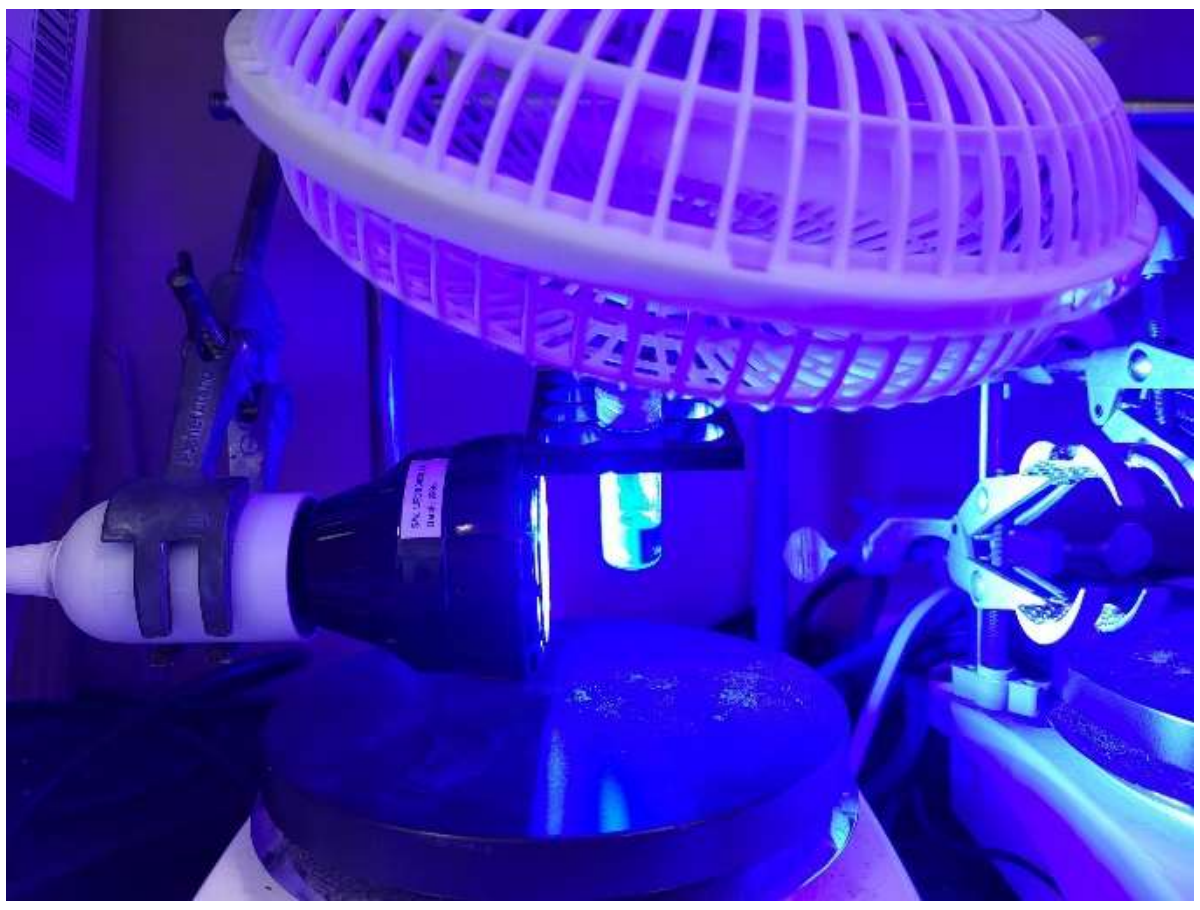

General procedure F: Hydroarylation of *N*-unsubstituted substrates

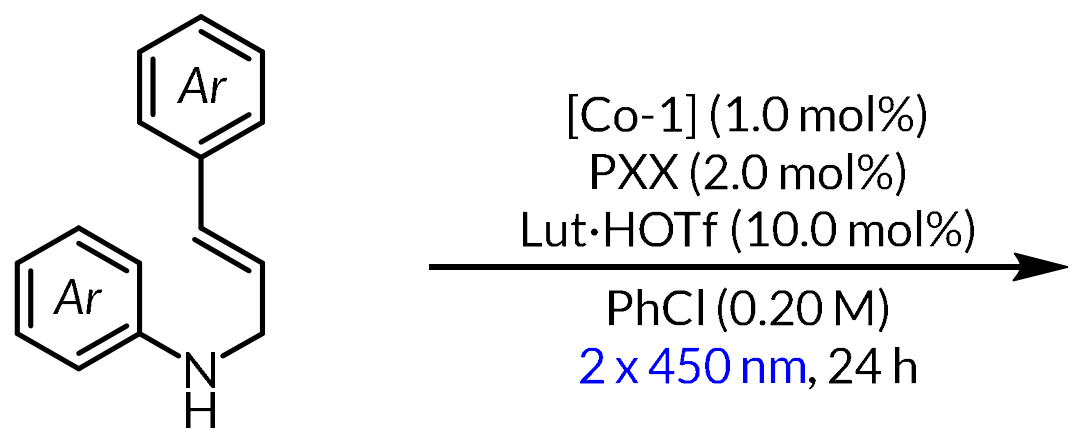

A 4-mL vial was charged with [Co-1] (1.2 mg, 0.002 mmol, 1.0 mol%), PXX (1.1 mg, 0.004 mmol, 2.0 mol%), Lut·HOTf (5.1 mg, 0.02 mmol, 10 mol%), and appropriate alkene (0.20 mmol). The vial was capped with a rubber septum and evacuated/backfilled with nitrogen (× 3). PhCl (1.0 mL, 0.20 M) was then added, and the reaction mixture was degassed by bubbling nitrogen through the solution for 15 mins. The reaction was irradiated by two 450 nm lamps (HCK1012-

01-002 at a 10 mm distance either side of the reaction vial, see below) for 24 h. 1,2-Dibromoethane (17.2 µL, 0.20 mmol) was added as the internal standard to the crude reaction mixture to determine the yield via ^{1}H-NMR. The crude product was purified by silica-gel column chromatography.

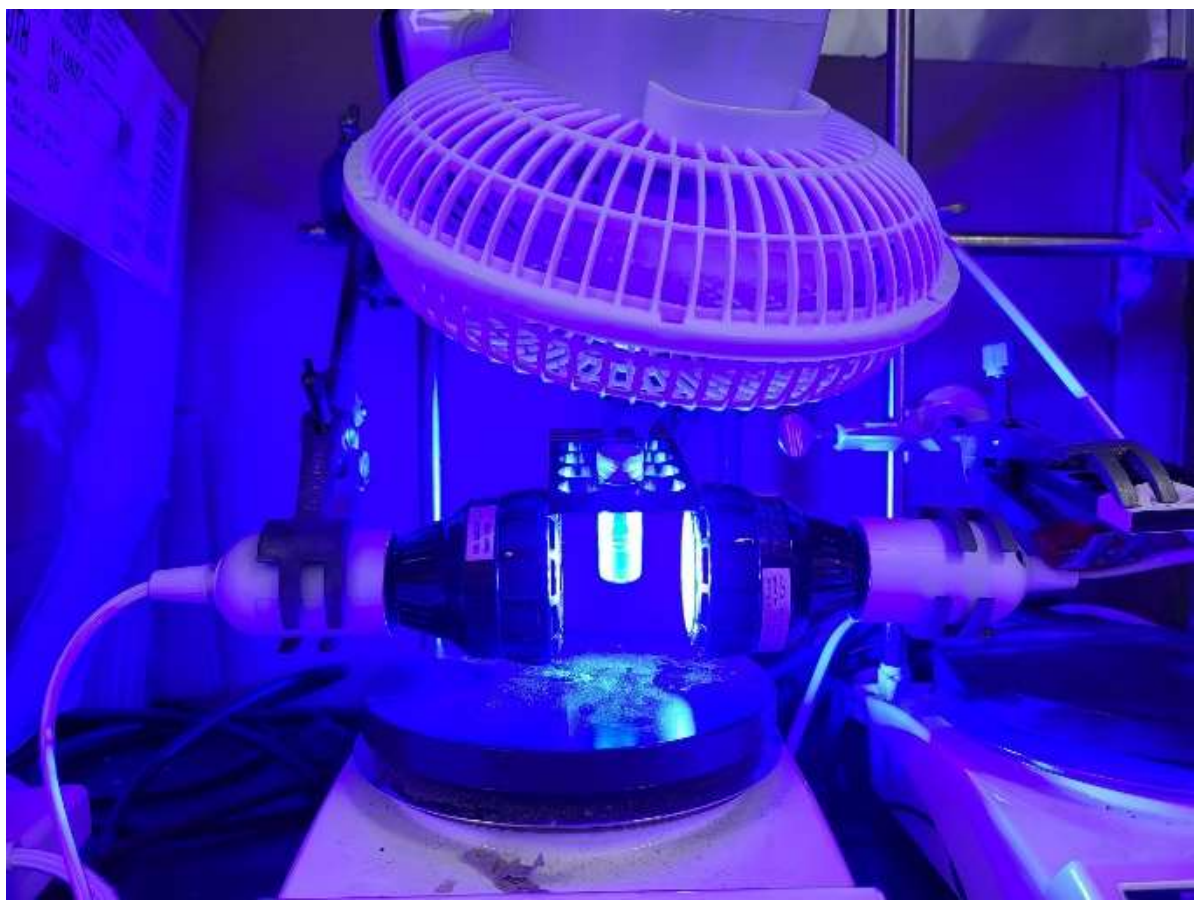

(±)-1-Benzyl-4-phenyl-1,2,3,4-tetrahydroquinoline (2aa)

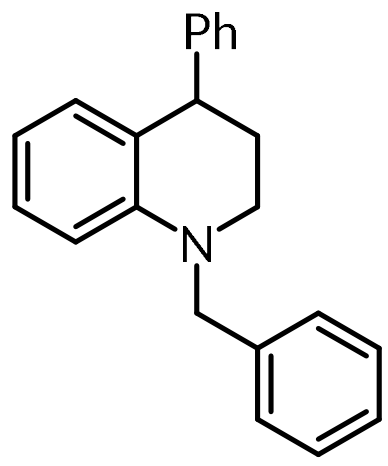

Following general procedure **E**, [Co-1] (1.2 mg, 0.002 mmol, 1.0 mol%), PXX (1.1 mg, 0.004 mmol, 2.0 mol%), Lut·HOTf (5.1 mg, 0.02 mmol, 10 mol%), **1aa** (59.9 mg, 0.20 mmol), and PhCl (1.0 mL) for 16 h at 450 nm irradiation gave a ^{1}H-NMR yield of 99%. Purification by column chromatography (hexane/CH_2Cl_2, 95:5 → 90:10 → 80:20) gave the title compound **2aa** (55.2 mg, 92%) as a yellow oil.

ν_{max}(film) / cm^{-1}: 3024 (C–H), 2854, 1590, 1503, 1492, 1450, 1337, 1250, 700

^{1}H NMR (500 MHz, CDCl$_3$) δ_H: 7.43 – 7.35 (6H, m), 7.34 – 7.27 (2H, m), 7.24 – 7.19 (2H, m), 7.14 – 7.08 (1H, m), 6.88 (1H, d, *J* 7.4), 6.69 (1H, d, *J* 8.3), 6.63 (1H, t, *J* 7.3), 4.61 (2H, s), 4.27 (1H, t, *J* 5.7), 3.44 – 3.32 (2H, m), 2.41 – 2.33 (1H, m), 2.27 – 2.18 (1H, m)

^{13}C{^{1}H} NMR (126 MHz, CDCl$_3$) δ_C: 146.2, 145.8, 138.9, 130.1, 128.8, 128.7, 128.4, 127.8, 126.9, 126.7, 126.2, 124.1, 116.0, 111.2, 55.4, 46.8, 43.5, 30.7

HRMS: (ESI$^+$) $C_{22}H_{18}N$ [M-3H]$^+$ requires 296.1434, found 296.1431 (-1.0 ppm)

(±)-1-(4-Methoxybenzyl)-4-phenyl-1,2,3,4-tetrahydroquinoline (2ab)

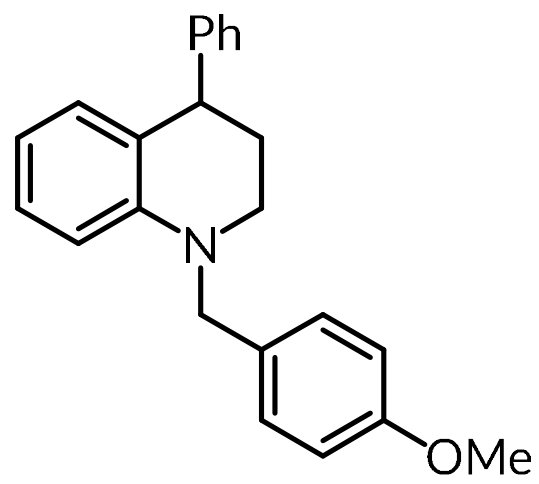

Following general procedure **E**, [Co-1] (1.2 mg, 0.002 mmol, 1.0 mol%), PXX (1.1 mg, 0.004 mmol, 2.0 mol%), Lut·HOTf (5.1 mg, 0.02 mmol, 10 mol%), **1ab** (65.9 mg, 0.20 mmol), and PhCl (1.0 mL) for 16 h at 450 nm irradiation gave a ^{1}H-NMR yield of 92%. Purification by column chromatography (hexane/CH_2Cl_2, 95:5 → 90:10 → 80:20→ 70:30) gave the title compound **2ab** (60.6 mg, 92%) as a yellow oil.

ν_{max}(film) / cm^{-1}: 3024, 2833, 1601, 1503, 1450, 1244, 1171, 1032, 745, 700

^{1}H NMR (500 MHz, CDCl$_3$) δ$_H$: 7.37 – 7.30 (2H, m), 7.27 – 7.22 (3H, m), 7.16 (2H, d, *J* 7.4), 7.11 – 7.04 (1H, m), 6.90 (2H, d, *J* 8.6), 6.82 (1H, d, *J* 7.6), 6.68 (1H, d, *J* 8.3), 6.61 – 6.55 (1H, m), 4.50 (2H, s), 4.21 (1H, t, *J* 5.7), 3.82 (3H, s), 3.38 – 3.23 (2H, m), 2.37 – 2.25 (1H, m), 2.22 – 2.11 (1H, m)

^{13}C{^{1}H} NMR (126 MHz, CDCl$_3$) δ$_C$: 158.7, 146.3, 145.9, 130.8, 130.2, 128.8, 128.4, 127.9, 127.8, 126.2, 124.2, 116.0, 114.1, 111.3, 55.4, 54.7, 46.6, 43.5, 30.7

HRMS: (ESI$^+$) $C_{23}H_{20}NO$ [M-3H]$^+$ requires 326.1539, found 326.1538 (-0.3 ppm)

(±)-1-(4-Fluorobenzyl)-4-phenyl-1,2,3,4-tetrahydroquinoline (2ac)

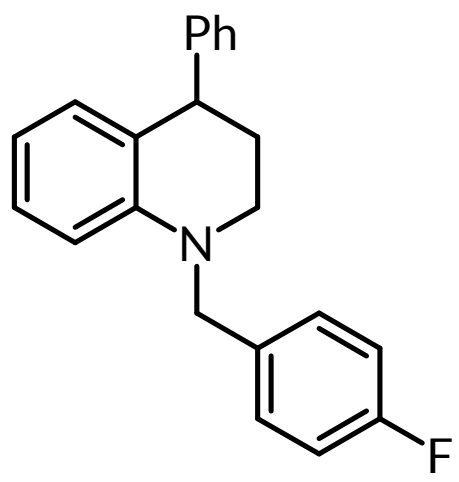

Following general procedure **E**, [Co-1] (1.2 mg, 0.002 mmol, 1.0 mol%), PXX (1.1 mg, 0.004 mmol, 2.0 mol%), Lut·HOTf (5.1 mg, 0.02 mmol, 10 mol%), **1ac** (63.5 mg, 0.20 mmol), and PhCl (1.0 mL) for 16 h at 450 nm irradiation gave a ^{1}H-NMR yield of 88%. Purification by column chromatography (hexane/CH_2Cl_2, 95:5 → 80:20 → 70:30) gave the title compound **2ac** (52.1 mg, 82%) as a yellow oil.

ν_{max} (film) / cm^{-1}: 3024, 2854, 1598, 1502, 1450, 1337, 1219, 1153, 816, 746

^{1}H NMR (500 MHz, CDCl$_3$) δ$_H$: 7.36 – 7.30 (2H, m), 7.30 – 7.21 (3H, m), 7.15 (2H, d, *J* 7.5), 7.09 – 7.01 (3H, m), 6.83 (1H, d, *J* 7.5), 6.65 – 6.56 (2H, m), 4.52 (2H, s), 4.22 (1H, t, *J* 5.7), 3.38 – 3.24 (2H, m), 2.37 – 2.28 (1H, m), 2.22 – 2.11 (1H, m)

^{13}C{^{1}H} NMR (126 MHz, CDCl$_3$) δ$_C$: 162.0 (d, *J* 244.5), 146.2, 145.7, 134.5 (d, *J* 3.0), 130.3, 128.7, 128.4, 128.2 (d, *J* 7.9), 127.9, 126.3, 124.3, 116.3, 115.6 (d, *J* 21.3), 111.2, 54.8, 46.7, 43.4, 30.7

^{19}F{^{1}H} NMR (471 MHz, CDCl$_3$) δ$_F$: -116.2 (s)

HRMS: (ESI$^+$) $C_{22}H_{17}FN$ [M-3H]$^+$ requires 314.1340, found 314.1345 (+1.6 ppm)

(±)-4-Phenyl-1-(4-(trifluoromethyl)benzyl)-1,2,3,4-tetrahydroquinoline (2ad)

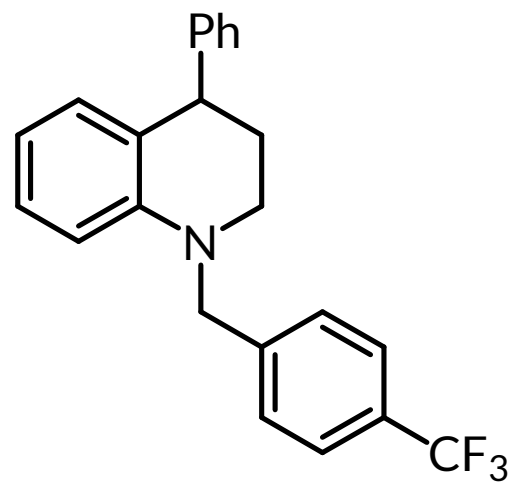

Following general procedure **E**, [Co-1] (1.2 mg, 0.002 mmol, 1.0 mol%), PXX (1.1 mg, 0.004 mmol, 2.0 mol%), Lut·HOTf (5.1 mg, 0.02 mmol, 10 mol%), **1ad** (73.5 mg, 0.20 mmol), and PhCl (1.0 mL) for 16 h at 450 nm irradiation gave a ^{1}H-NMR yield of 95%. Purification by column chromatography (hexane/CH_2Cl_2, 95:5 → 80:20 → 70:30) gave the title compound **2ad** (63.3 mg, 86%) as a yellow oil.

ν_{max} (film) / cm^{-1}: 3026, 2924, 1600, 1504, 1323, 1162, 1121, 1067, 746

^{1}H NMR (500 MHz, CDCl$_3$) δ_H: 7.64 – 7.58 (2H, m), 7.44 (2H, d, *J* 7.9), 7.37 – 7.32 (2H, m), 7.29 – 7.22 (1H, m), 7.17 (2H, d, *J* 6.8), 7.07 (1H, t, *J* 7.7), 6.86 (1H, d, *J* 6.9), 6.62 (1H, t, *J* 7.3), 6.56 (1H, d, *J* 8.2), 4.60 (2H, s), 4.29 – 4.19 (1H, m), 3.42 – 3.23 (2H, m), 2.43 – 2.28 (1H, m), 2.26 – 2.14 (1H, m)

^{13}C{^{1}H} NMR (126 MHz, CDCl$_3$) δ_C: 146.1, 145.5, 143.3, 130.3, 129.4 (q, *J* 32.3), 128.7, 128.5, 127.9, 127.0, 126.4, 125.7 (q, *J* 3.8), 124.4 (q, *J* 271.9), 124.3, 116.5, 111.1, 55.2, 47.0, 43.4, 30.7

^{19}F{^{1}H} NMR (471 MHz, CDCl$_3$) δ_F: -62.4 (s)

HRMS: (ESI$^+$) $C_{23}H_{17}F_3N$ [M-3H]$^+$ requires 364.1308, found 364.1317 (+2.5 ppm)

(±)-1-(Naphthalen-1-ylmethyl)-4-phenyl-1,2,3,4-tetrahydroquinoline (2ae)

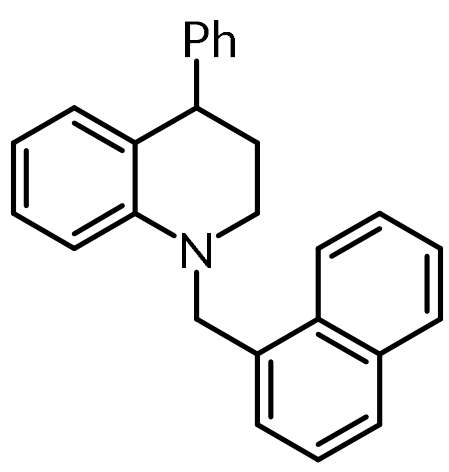

Following general procedure **E**, [Co-1] (1.2 mg, 0.002 mmol, 1.0 mol%), PXX (1.1 mg, 0.004 mmol, 2.0 mol%), Lut·HOTf (5.1 mg, 0.02 mmol, 10 mol%), **1ae** (69.9 mg, 0.20 mmol), and PhCl (1.0 mL) for 16 h at 450 nm irradiation gave a ^{1}H-NMR yield of 90%. Purification by column chromatography (hexane/CH_2Cl_2, 95:5 → 90:10 → 80:20) gave the title compound **2ae** (60.1 mg, 86%) as a yellow oil.

ν_{max} (film) / cm^{-1}: 3059, 2854, 1597, 1503, 1319, 1256, 1175, 907, 791, 729

^{1}H NMR (500 MHz, CDCl$_3$) δ_H: 8.05 (1H, d, *J* 7.9), 7.94 (1H, d, *J* 7.7), 7.81 (1H, d, *J* 7.6), 7.65 – 7.52 (2H, m), 7.50 – 7.41 (2H, m), 7.39 – 7.33 (2H, m), 7.31 – 7.19 (3H, m), 7.09 – 7.01 (1H, m), 6.88 (1H, d, *J* 7.4), 6.67 – 6.54 (2H, m), 5.03 (1H, d, *J* 17.0), 4.98 (1H, d, *J* 17.0), 4.28 (1H, t, *J* 11.7), 3.44 – 3.27 (2H, m), 2.44 – 2.31 (1H, m), 2.29 – 2.17 (1H, m)

^{13}C{^{1}H} NMR (126 MHz, CDCl$_3$) δ_C: 146.2, 145.9, 134.0, 132.8, 131.4, 130.1, 129.0, 128.8, 128.5, 127.9, 127.6, 126.3, 126.2, 125.82, 125.80, 124.3, 123.7, 122.8, 116.1, 111.2, 53.4, 46.6, 43.6, 30.8

HRMS: (ESI$^+$) $C_{26}H_{20}N$ [M-3H]$^+$ requires 346.1590, found 346.1589 (-0.3 ppm)

(±)-4-Phenyl-1-(thiophen-2-ylmethyl)-1,2,3,4-tetrahydroquinoline (2af)

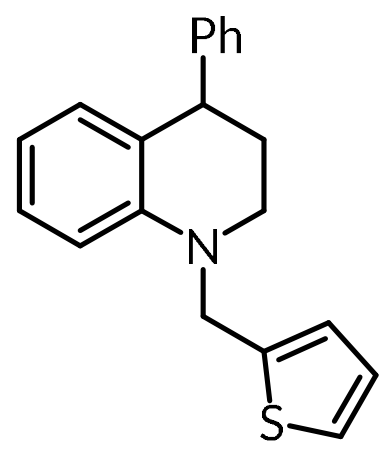

Following general procedure **E**, [Co-1] (1.2 mg, 0.002 mmol, 1.0 mol%), PXX (1.1 mg, 0.004 mmol, 2.0 mol%), Lut·HOTf (5.1 mg, 0.02 mmol, 10 mol%), **1af** (61.1 mg, 0.20 mmol), and PhCl (1.0 mL) for 16 h at 450 nm irradiation gave a ^{1}H-NMR yield of 99%. Purification by column chromatography (hexane/CH_2Cl_2, 95:5 → 80:20 → 70:30) gave the title compound **2af** (55.1 mg, 90%) as a yellow oil.

ν_{max} (film) / cm^{-1}: 3022, 2922, 1599, 1499, 1489, 1335, 746, 698

^{1}H NMR (500 MHz, CDCl$_3$) δ_H: 7.33 – 7.27 (2H, m), 7.25 – 7.19 (2H, m), 7.15 – 7.09 (3H, m), 7.01 – 6.95 (2H, m), 6.87 – 6.79 (2H, m), 6.65 – 6.58 (1H, m), 4.75 (1H, d, *J* 16.6), 4.67 (1H, d, *J* 16.6), 4.18 (1H, t, *J* 5.7), 3.43 – 3.20 (2H, m), 2.37 – 2.24 (1H, m), 2.18 – 2.07 (1H, m)

^{13}C{^{1}H} NMR (126 MHz, CDCl$_3$) δ_C: 146.4, 145.1, 142.1, 130.4, 128.8, 128.4, 127.7, 126.9, 126.2, 125.0, 124.7, 124.4, 116.7, 111.7, 50.6, 46.3, 43.4, 30.7

HRMS: (ESI$^+$) $C_{20}H_{16}NS$ [M-3H]$^+$ requires 302.0998, found 302.0995 (-1.0 ppm)

(±)-1-Methyl-4-phenyl-1,2,3,4-tetrahydroquinoline (2ag)

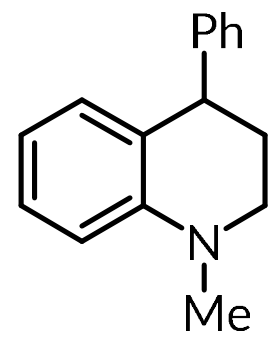

Following general procedure **E**, [Co-1] (1.2 mg, 0.002 mmol, 1.0 mol%), PXX (1.1 mg, 0.004 mmol, 2.0 mol%), Lut·HOTf (5.1 mg, 0.02 mmol, 10 mol%), **1ag** (44.7 mg, 0.20 mmol), and PhCl (1.0 mL) for 16 h at 450 nm irradiation gave a ^{1}H-NMR yield of 95%. Purification by column chromatography (hexane/CH_2Cl_2, 95:5 → 80:20 → 70:30) gave the title compound **2ag** (38.4 mg, 86%) as a yellow oil.

ν_{max}(film) / cm^{-1}: 2941, 2818, 1601, 1501, 1325, 1207, 744, 700

^{1}H NMR (500 MHz, $CDCl_3$) δ_H: 7.33 – 7.28 (2H, m), 7.25 – 7.19 (1H, m), 7.17 – 7.10 (3H, m), 6.76 (1H, d, *J* 7.4), 6.69 (1H, d, *J* 8.2), 6.58 (1H, td, *J* 7.3, 1.2), 4.15 (1H, t, *J* 6.2), 3.28 – 3.14 (2H, m), 2.96 (3H, s), 2.32 – 2.23 (1H, m), 2.16 – 2.07 (1H, m)

^{13}C{^{1}H} NMR (126 MHz, $CDCl_3$) δ_C: 147.0, 146.7, 130.0, 128.8, 128.4, 127.7, 126.2, 124.9, 116.3, 111.1, 48.6, 43.5, 39.4, 31.2

HRMS: (ESI$^+$) $C_{16}H_{14}N$ [M-3H]$^+$ requires 220.1121, found 220.1119 (-0.9 ppm)

(±)-1-Cyclohexyl-4-phenyl-1,2,3,4-tetrahydroquinoline (2ah)

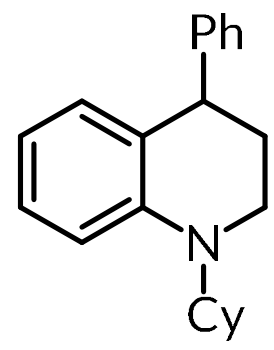

Following general procedure **E**, [Co-1] (1.2 mg, 0.002 mmol, 1.0 mol%), PXX (1.1 mg, 0.004 mmol, 2.0 mol%), Lut·HOTf (5.1 mg, 0.02 mmol, 10 mol%), **1ah** (58.3 mg, 0.20 mmol), and PhCl (1.0 mL) for 16 h at 450 nm irradiation gave a ^{1}H-NMR yield of 99%. Purification by column chromatography (hexane/CH_2Cl_2, 95:5 → 80:20 → 70:30) gave the title compound **2ah** (54.3 mg, 93%) as a yellow oil.

ν_{max}(film) / cm^{-1}: 2928, 2853, 1599, 1493, 1449, 1310, 1171, 740, 700

^{1}H NMR (500 MHz, $CDCl_3$) δ_H: 7.34 – 7.28 (2H, m), 7.24 – 7.18 (1H, m), 7.14 – 7.08 (3H, m), 6.81 – 6.73 (2H, m), 6.56 – 6.47 (1H, m), 4.11 (1H, t, *J* 5.6), 3.74 – 3.64 (1H, m), 3.25 – 3.18 (1H, m), 3.12 – 3.03 (1H, m), 2.23 – 2.13 (1H, m), 2.09 – 2.00 (1H, m), 1.92 – 1.80 (4H, m), 1.77 – 1.68 (1H, m), 1.58 – 1.35 (4H, m), 1.21 – 1.07 (1H, m)

$^{13}C\{^1H\}$ NMR (126 MHz, $CDCl_3$) δ_C: 146.5, 145.7, 130.4, 128.7, 128.3, 127.7, 126.1, 124.9, 115.2, 110.9, 56.6, 43.7, 38.6, 30.8, 29.7, 29.6, 26.40, 26.37, 26.2

HRMS: (ESI$^+$) $C_{21}H_{22}N$ $[M-3H]^+$ requires 288.1747, found 288.1743 (-1.4 ppm)

(±)-1,4-Diphenyl-1,2,3,4-tetrahydroquinoline (2ai)

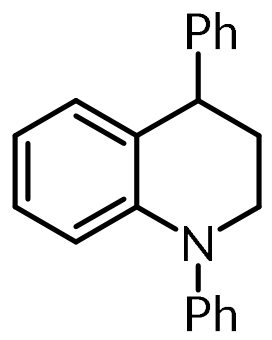

Following general procedure **E**, [Co-1] (1.2 mg, 0.002 mmol, 1.0 mol%), PXX (1.1 mg, 0.004 mmol, 2.0 mol%), Lut·HOTf (5.1 mg, 0.02 mmol, 10 mol%), **1ai** (57.1 mg, 0.20 mmol), and PhCl (1.0 mL) for 16 h at 450 nm irradiation gave a ^{1}H-NMR yield of 99%. Purification by column chromatography (hexane/CH_2Cl_2, 95:5 → 80:20 → 70:30) gave the title compound **2ai** (49.2 mg, 86%) as a yellow oil.

ν_{max}(film) / cm^{-1}: 3024, 2855, 1591, 1491, 1445, 1312, 748, 698

^1H NMR (500 MHz, $CDCl_3$) δ_H: 7.42 – 7.29 (6H, m), 7.28 – 7.20 (3H, m), 7.14 (1H, t, *J* 7.3), 7.04 – 6.96 (1H, m), 6.87 (1H, d, *J* 7.5), 6.83 (1H, d, *J* 8.3), 6.68 (1H, t, *J* 7.4), 4.25 (1H, t, *J* 6.1), 3.71 – 3.52 (2H, m), 2.46 – 2.32 (1H, m), 2.26 – 2.13 (1H, m)

$^{13}C\{^1H\}$ NMR (126 MHz, $CDCl_3$) δ_C: 148.5, 146.3, 144.8, 130.5, 129.6, 128.8, 128.5, 127.0, 126.5, 126.4, 125.1, 124.0, 118.4, 115.9, 48.3, 43.5, 31.4

HRMS: (ESI$^+$) $C_{21}H_{16}N$ $[M-3H]^+$ requires 282.1277, found 282.1275 (-0.7 ppm)

(±)-1-Allyl-4-phenyl-1,2,3,4-tetrahydroquinoline (2aj)

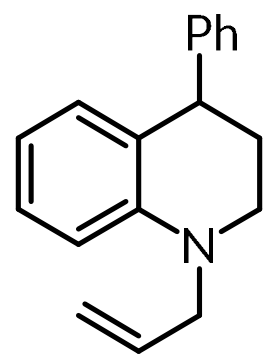

Following general procedure **E**, [Co-1] (1.2 mg, 0.002 mmol, 1.0 mol%), PXX (1.1 mg, 0.004 mmol, 2.0 mol%), Lut·HOTf (5.1 mg, 0.02 mmol, 10 mol%), **1aj** (49.9 mg, 0.20 mmol), and PhCl (1.0 mL) for 16 h at 450 nm irradiation gave a ^{1}H-NMR yield of 82%. Purification by column chromatography (hexane/CH_2Cl_2, 95:5 → 90:10 → 80:20 → 70:30) gave the title compound **2aj** (40.4 mg, 81%) as a yellow oil.

ν_{max}(film) / cm^{-1}: 3024, 2855, 1601 (C=C), 1503, 1337, 1248, 918, 746, 700

^{1}H NMR (500 MHz, $CDCl_3$) δ_H: 7.34 – 7.29 (2H, m), 7.24 – 7.20 (1H, m), 7.17 – 7.06 (3H, m), 6.78 (1H, d, *J* 7.3), 6.66 (1H, d, *J* 8.3), 6.59 – 6.52 (1H, m), 5.89 (1H, ddt, *J* 17.2, 10.1, 5.0), 5.24 (1H, dq, *J* 17.2, 1.8), 5.18 (1H, dt, *J* 10.3, 1.7), 4.15 (1H, t, *J* 5.8), 3.96 – 3.91 (2H, m), 3.32 – 3.15 (2H, m), 2.30 – 2.21 (1H, m), 2.16 – 2.03 (1H, m)

^{13}C{^{1}H} NMR (126 MHz, $CDCl_3$) δ_C: 146.5, 145.6, 133.5, 130.2, 128.8, 128.4, 127.7, 126.2, 124.3, 116.1, 115.9, 111.2, 54.0, 46.1, 43.5, 30.7

HRMS: (ESI$^+$) $C_{18}H_{16}N$ [M-3H]$^+$ requires 246.1277, found 246.1275 (-0.8 ppm)

(±)-1,7-Diphenyl-2,3,6,7-tetrahydro-1*H*,5*H*-pyrido[3,2,1-*ij*]quinoline (2ak)

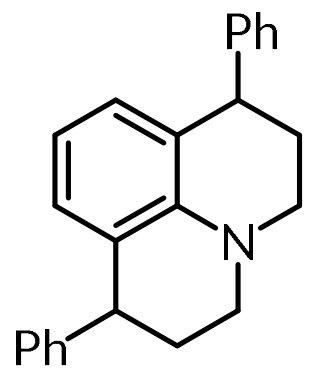

Following modified general procedure **E**, [Co-1] (1.2 mg, 0.002 mmol, 1.0 mol%), PXX (1.1 mg, 0.004 mmol, 2.0 mol%), Lut·HOTf (10.2 mg, 0.04 mmol, 20 mol%), **1ak** (65.1 mg, 0.20 mmol), and PhCl (1.0 mL) for 16 h at 450 nm irradiation gave a ^{1}H-NMR yield of 84%. Purification by column chromatography (hexane/CH_2Cl_2, 95:5 → 80:20→ 60:40) gave the title compound **2ak** (58.6 mg, 90%, *dr* = 1.0 : 1.0) as a yellow oil.

ν_{max}(film) / cm^{-1}: 3024, 2940, 1651, 1597, 1491, 1449, 1314, 908, 729, 700

^{1}H NMR (500 MHz, $CDCl_3$) δ_H: 7.38 – 7.30 (4H, m), 7.27 – 7.12 (6H, m), 6.69 – 6.60 (2H, m), 6.47 – 6.38 (1H, m), 4.27 – 4.13 (2H, m), 3.25 – 3.08 (4H, m), 2.39 – 2.25 (2H, m), 2.21 – 2.09 (2H, m)

^{13}C{^{1}H} NMR (126 MHz, $CDCl_3$) δ_C: 147.1, 147.0, 143.40, 143.36, 128.8, 128.72, 128.69, 128.42, 128.41, 126.2, 123.61, 123.58, 115.8, 115.7, 47.4 (2C), 43.7, 43.6, 30.85, 30.80

Note: Multiple peaks overlapping in the 128-ppm region.

HRMS: (ESI$^+$) $C_{24}H_{20}N$ [M-3H]$^+$ requires 322.1590, found 322.1597 (+2.2 ppm)

(±)-5,7-Dimethoxy-4-phenyl-1-tosyl-1,2,3,4-tetrahydroquinoline (2al)

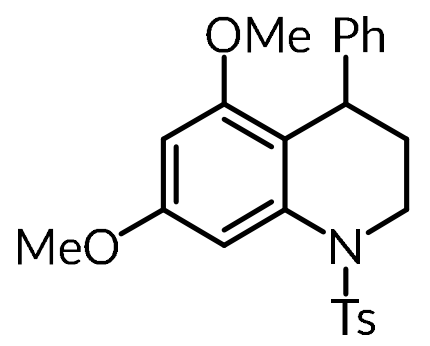

Following general procedure **E**, [Co-1] (1.2 mg, 0.002 mmol, 1.0 mol%), PXX (1.1 mg, 0.004 mmol, 2.0 mol%), Lut·HOTf (5.1 mg, 0.02 mmol, 10 mol%), **1al** (84.7 mg, 0.20 mmol), and PhCl (1.0 mL) for 16 h at 450 nm irradiation gave a ^{1}H-NMR yield of 99%. Purification by column chromatography (hexane/CH_2Cl_2, 95:5 → 90:10 → 80:20 → 60:40) gave the title compound **2al** (78.1 mg, 92%) as a yellow oil.

ν_{max}(film) / cm^{-1}: 2938, 1607, 1585, 1350, 1161, 1125, 1049, 908, 727, 702, 699

^{1}H NMR (500 MHz, $CDCl_3$) δ_H: 7.59 (2H, d, *J* 8.3), 7.32 (1H, d, *J* 2.3), 7.21 (2H, d, *J* 8.0), 7.12 – 7.01 (3H, m), 6.67 – 6.61 (2H, m), 6.22 (1H, d, *J* 2.4), 4.17 – 4.12 (1H, m), 3.98 – 3.92 (1H, m), 3.86 (3H, s), 3.57 – 3.49 (1H, m), 3.47 (3H, s), 2.41 (3H, s), 1.95 – 1.84 (1H, m), 1.81 – 1.73 (1H, m)

^{13}C{^{1}H} NMR (126 MHz, $CDCl_3$) δ_C: 159.2, 158.4, 146.1, 143.8, 138.8, 136.6, 129.7, 128.0, 127.5, 127.3, 125.6, 112.1, 99.3, 95.8, 55.57, 55.56, 43.4, 36.7, 29.8, 21.7

HRMS: (ESI$^+$) $C_{24}H_{26}NO_4S$ $[M+H]^+$ requires 424.1577, found 424.1574 (-0.7 ppm)

(±)-1-Benzyl-5,7-dimethoxy-4-phenyl-3,4-dihydroquinolin-2(1*H*)-one (2am)

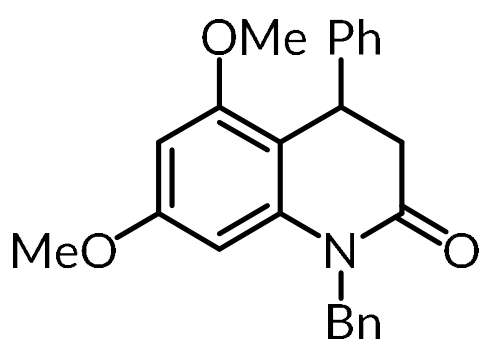

Following modified general procedure **E**, [Co-1] (1.2 mg, 0.002 mmol, 1.0 mol%), PXX (1.1 mg, 0.004 mmol, 2.0 mol%), Lut·HOTf (5.1 mg, 0.02 mmol, 10 mol%), **1am** (74.7 mg, 0.20 mmol), and PhCl (1.0 mL) for 16 h at 450 nm irradiation (two lamps) gave a ^{1}H-NMR yield of 100%. Purification by column chromatography (hexane/EtOAc, 95:5 → 90:10 → 80:20) gave the title compound **2am** (74.6 mg, 99%) as a yellow solid

mp 118 – 121 °C

ν_{max}(film) / cm^{-1}: 1674 (C=O), 1597, 1495, 1456, 1369, 1341, 1200, 1153, 1138, 1036, 735, 698

^{1}H NMR (700 MHz, $CDCl_3$) δ_H: 7.25 – 7.16 (6H, m), 7.11 (2H, d, *J* 7.4), 7.07 (2H, d, *J* 7.5), 6.24 – 6.20 (2H, m), 5.16 (1H, d, *J* 16.0), 5.12 (1H, d, *J* 16.1), 4.61 (1H, dd, *J* 6.7, 2.2), 3.75 (3H, s), 3.69 (3H, s), 3.11 (1H, dd, *J* 15.9, 2.2), 3.06 (1H, dd, *J* 15.9, 6.7)

$^{13}C\{^1H\}$ NMR (176 MHz, $CDCl_3$) δ_C: 169.7, 160.2, 157.6, 142.0, 141.2, 137.3, 128.7, 128.6, 127.23, 127.21, 127.1, 126.7, 109.2, 95.1, 93.2, 55.9, 55.4, 46.1, 38.8, 33.6

HRMS: (ESI$^+$) $C_{24}H_{23}NO_3Na$ [M+Na]$^+$ requires 396.1570, found 396.1567 (-0.8 ppm)

(±)-1-Benzyl-4-(*p*-tolyl)-1,2,3,4-tetrahydroquinoline (2an)

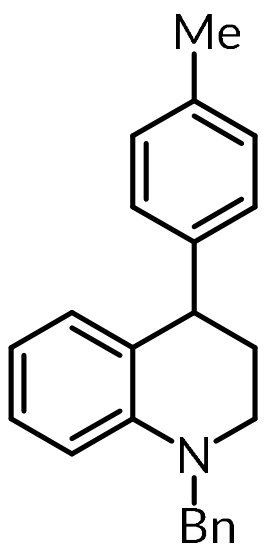

Following general procedure **E**, [Co-1] (1.2 mg, 0.002 mmol, 1.0 mol%), PXX (1.1 mg, 0.004 mmol, 2.0 mol%), Lut·HOTf (5.1 mg, 0.02 mmol, 10 mol%), **1an** (62.7 mg, 0.20 mmol), and PhCl (1.0 mL) for 16 h at 450 nm irradiation gave a ^{1}H-NMR yield of 90%. Purification by column chromatography (hexane/CH_2Cl_2, 95:5 → 90:10 → 80:20) gave the title compound **2an** (50.3 mg, 80%) as a yellow oil.

ν_{max}(film) / cm^{-1}: 2920 (C–H), 1601, 1501, 1450, 907, 725

^1H NMR (500 MHz, $CDCl_3$) δ_H: 7.37 – 7.28 (4H, m), 7.26 (1H, t, *J* 6.9), 7.12 (2H, d, *J* 7.8), 7.06 – 7.00 (3H, m), 6.80 (1H, d, *J* 7.3), 6.60 (1H, d, *J* 8.3), 6.55 (1H, t, *J* 7.3), 4.54 (2H, s), 4.16 (1H, t, *J* 5.6), 3.40 – 3.23 (2H, m), 2.34 (3H, s), 2.33 – 2.24 (1H, m), 2.18 – 2.07 (1H, m)

$^{13}C\{^1H\}$ NMR (126 MHz, $CDCl_3$) δ_C: 145.8, 143.3, 139.0, 135.8, 130.1, 129.1, 128.74, 128.66, 127.8, 127.0, 126.7, 124.4, 116.0, 111.2, 55.4, 46.9, 43.1, 30.8, 21.2

HRMS: (ESI$^+$) $C_{23}H_{20}N$ [M-3H]$^+$ requires 310.1590, found 310.1596 (+1.9 ppm)

(±)-1-Benzyl-4-(4-methoxyphenyl)-1,2,3,4-tetrahydroquinoline (2ao)

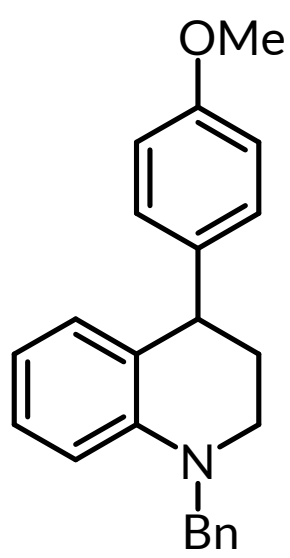

Following general procedure **E**, [Co-1] (1.2 mg, 0.002 mmol, 1.0 mol%), PXX (1.1 mg, 0.004 mmol, 2.0 mol%), Lut·HOTf (5.1 mg, 0.02 mmol, 10 mol%), **1ao** (65.9 mg, 0.20 mmol), and PhCl (1.0 mL)

for 16 h at 450 nm irradiation gave a ^{1}H-NMR yield of 96%. Purification by column chromatography (hexane/CH_2Cl_2, 95:5 → 90:10 → 80:20 → 70:30) gave the title compound **2ao** (57.8 mg, 88%) as an orange oil.

ν_{max}(film) / cm^{-1}: 1599, 1503, 1450, 1337, 1300, 1244, 1175, 1028, 829

^{1}H NMR (500 MHz, $CDCl_3$) δ_H: 7.39 – 7.28 (4H, m), 7.28 – 7.22 (1H, m), 7.10 – 7.00 (3H, m), 6.89 – 6.83 (2H, m), 6.80 (1H, d, *J* 7.2), 6.60 (1H, d, *J* 8.4), 6.56 (1H, t, *J* 7.3), 4.54 (2H, s), 4.15 (1H, t, *J* 5.7), 3.81 (3H, s), 3.37 – 3.24 (2H, m), 2.32 – 2.22 (1H, m), 2.16 – 2.06 (1H, m)

^{13}C{^{1}H} NMR (126 MHz, $CDCl_3$) δ_C: 158.1, 145.8, 139.0, 138.4, 130.1, 129.7, 128.7, 127.8, 127.0, 126.7, 124.5, 116.0, 113.8, 111.2, 55.39, 55.36, 46.9, 42.7, 30.8

HRMS: (ESI$^+$) $C_{23}H_{24}NO$ [M+H]$^+$ requires 330.1852, found 330.1849 (-0.9 ppm)

(±)-1-Benzyl-4-(4-fluorophenyl)-1,2,3,4-tetrahydroquinoline (2ap)

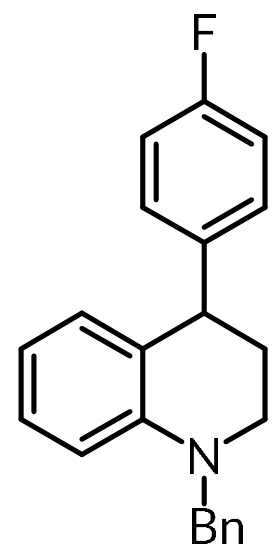

Following general procedure **E**, [Co-1] (1.2 mg, 0.002 mmol, 1.0 mol%), PXX (1.1 mg, 0.004 mmol, 2.0 mol%), Lut·HOTf (5.1 mg, 0.02 mmol, 10 mol%), **1ap** (63.5 mg, 0.20 mmol), and PhCl (1.0 mL) for 16 h at 450 nm irradiation gave a ^{1}H-NMR yield of 80%. Purification by column chromatography (hexane/CH_2Cl_2, 95:5 → 90:10 → 80:20) gave the title compound **2ap** (44.8 mg, 71%) as an orange oil.

ν_{max}(film) / cm^{-1}: 2922 (C–H), 1599, 1501, 1450, 1337, 1219, 1155, 833

^{1}H NMR (500 MHz, $CDCl_3$) δ_H: 7.39 – 7.31 (2H, m), 7.31 – 7.23 (3H, m), 7.13 – 7.07 (2H, m), 7.07 – 7.02 (1H, m), 6.99 (2H, t, *J* 8.7), 6.78 (1H, d, *J* 7.6), 6.62 (1H, d, *J* 8.3), 6.57 (1H, t, *J* 7.3), 4.54 (2H, s), 4.18 (1H, t, *J* 5.6), 3.39 – 3.31 (1H, m), 3.31 – 3.21 (1H, m), 2.36 – 2.25 (1H, m), 2.16 – 2.06 (1H, m)

^{13}C{^{1}H} NMR (126 MHz, $CDCl_3$) δ_C: 161.5 (d, *J* 244.1), 145.1, 141.9 (d, *J* 3.1), 138.8, 130.10 (d, *J* 7.7), 130.07 (2C), 128.8, 128.0, 127.0, 126.7, 123.6, 116.1, 115.2 (d, *J* 21.0), 111.3, 55.3, 46.6, 42.8, 30.8

<u>Note: 130.07 ppm peak contains two carbon peaks.</u>

^{19}F NMR (471 MHz, $CDCl_3$) δ_F: -117.2 – -117.3 (m)

HRMS: (ESI^+) $C_{22}H_{20}FN$ $[M]^+$ requires 317.1574, found 317.1574 (0.0 ppm)

(±)-1-Benzyl-4-(4-bromophenyl)-1,2,3,4-tetrahydroquinoline (2aq)

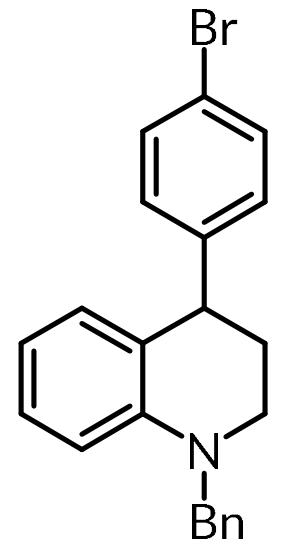

Following general procedure **E**, [Co-1] (1.2 mg, 0.002 mmol, 1.0 mol%), PXX (1.1 mg, 0.004 mmol, 2.0 mol%), Lut·HOTf (5.1 mg, 0.02 mmol, 10 mol%), **1aq** (75.7 mg, 0.20 mmol), and PhCl (1.0 mL) for 16 h at 450 nm irradiation gave a ^{1}H-NMR yield of 88%. Purification by column chromatography (hexane/CH_2Cl_2, 95:5 → 90:10 → 80:20) gave the title compound **2aq** (64.3 mg, 85%) as an orange oil.

ν_{max}(film) / cm^{-1}: 2922, 1599, 1501, 1485, 1450, 1335, 1070, 1009, 820

^{1}H NMR (500 MHz, $CDCl_3$) δ_H: 7.46 – 7.39 (2H, m), 7.37 – 7.31 (2H, m), 7.31 – 7.24 (3H, m), 7.07 – 7.03 (1H, m), 7.01 (2H, d, *J* 8.4), 6.77 (1H, d, *J* 7.4), 6.62 (1H, d, *J* 8.5), 6.56 (1H, t, *J* 7.2), 4.53 (2H, s), 4.16 (1H, t, *J* 5.6), 3.38 – 3.29 (1H, m), 3.28 – 3.20 (1H, m), 2.35 – 2.22 (1H, m), 2.13 – 2.05 (1H, m)

^{13}C{^{1}H} NMR (126 MHz, $CDCl_3$) δ_C: 145.7, 145.3, 138.8, 131.5, 130.5, 130.1, 128.8, 128.1, 127.0, 126.7, 123.5, 120.1, 116.1, 111.4, 55.3, 46.6, 43.0, 30.6

HRMS: (ESI^+) $C_{22}H_{21}{}^{79}BrN$ $[M+H]^+$ requires 378.0852, found 378.0850 (-0.5 ppm)

(±)-1-Benzyl-4-(4-trifluoromethylphenyl)-1,2,3,4-tetrahydroquinoline (2ar)

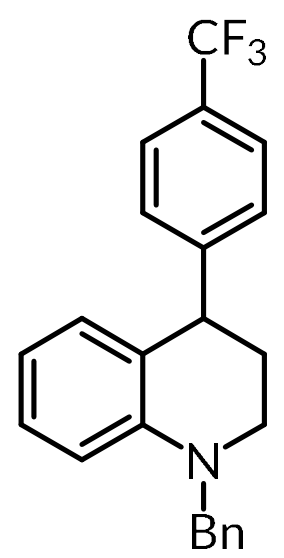

Following modified general procedure **E**, [Co-1] (1.2 mg, 0.002 mmol, 1.0 mol%), PXX (1.1 mg, 0.004 mmol, 2.0 mol%), Lut·HOTf (5.1 mg, 0.02 mmol, 10 mol%), **1ar** (73.5 mg, 0.20 mmol), and PhCl (1.0 mL) for 64 h at 450 nm irradiation gave a ^{1}H-NMR yield of 80%. Purification by column

chromatography (hexane/CH_2Cl_2, 95:5 → 90:10 → 80:20) gave the title compound **2ar** (50.0 mg, 68%) as an orange oil.

ν_{max}(film) / cm^{-1}: 1601, 1504, 1450, 1321, 1159, 1107, 1065, 1016, 833

^{1}H NMR (500 MHz, $CDCl_3$) δ_H: 7.56 (2H, d, *J* 8.0), 7.34 (2H, t, *J* 7.4), 7.31 – 7.24 (5H, m), 7.10 – 7.03 (1H, m), 6.76 (1H, d, *J* 7.4), 6.64 (1H, d, *J* 8.3), 6.57 (1H, t, *J* 7.3), 4.55 (2H, s), 4.26 (1H, t, *J* 5.7), 3.40 – 3.30 (1H, m), 3.28 – 3.19 (1H, m), 2.39 – 2.27 (1H, m), 2.19 – 2.08 (1H, m)

^{13}C{^{1}H} NMR (126 MHz, $CDCl_3$) δ_C: 150.4, 145.8, 138.7, 130.1, 129.1, 128.8, 128.6 (q, *J* 32.9), 128.2, 127.1, 126.7, 125.4 (q, *J* 3.9), 124.4 (q, *J* 271.6), 123.1, 116.2, 111.5, 55.3, 46.5, 43.4, 30.6

^{19}F NMR (471 MHz, $CDCl_3$) δ_F: -62.3 (s)

HRMS: (ESI$^+$) $C_{23}H_{20}F_3NNa$ $[M+Na]^+$ requires 390.1440, found 390.1445 (+1.3 ppm)

(±)-Methyl 3-(1-benzyl-1,2,3,4-tetrahydroquinolin-4-yl)benzoate (2as)

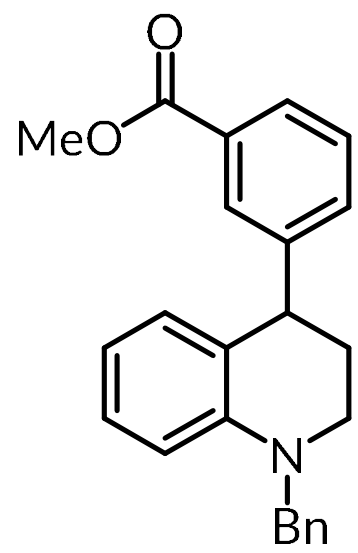

Following general procedure **E**, [Co-1] (1.2 mg, 0.002 mmol, 1.0 mol%), PXX (1.1 mg, 0.004 mmol, 2.0 mol%), Lut·HOTf (5.1 mg, 0.02 mmol, 10 mol%), **1as** (71.5 mg, 0.20 mmol), and PhCl (1.0 mL) for 16 h at 450 nm irradiation gave a ^{1}H-NMR yield of 100%. Purification by column chromatography (hexane/CH_2Cl_2 80:20 then hexane/EtOAc, 95:5 → 90:10 → 80:20) gave the title compound **2as** (68.0 mg, 95%) as a yellow oil.

ν_{max}(film) / cm^{-1}: 1716 (C=O), 1601, 1504, 1445, 1285, 907

^{1}H NMR (500 MHz, $CDCl_3$) δ_H: 7.97 – 7.86 (2H, m), 7.42 – 7.22 (7H, m), 7.05 (1H, t, *J* 7.7), 6.75 (1H, d, *J* 7.5), 6.62 (1H, d, *J* 8.3), 6.55 (1H, t, *J* 7.3), 4.55 (2H, s), 4.26 (1H, t, *J* 5.9), 3.92 (3H, s), 3.41 – 3.33 (1H, m), 3.31 – 3.24 (1H, m), 2.37 – 2.25 (1H, m), 2.22 – 2.13 (1H, m)

^{13}C{^{1}H} NMR (126 MHz, $CDCl_3$) δ_C: 167.4, 146.6, 145.8, 138.8, 133.5, 130.4, 130.0, 129.7, 128.8, 128.5, 128.1, 127.7, 127.0, 126.7, 123.6, 116.2, 111.4, 55.3, 52.3, 46.8, 43.5, 30.7

HRMS: (ESI$^+$) $C_{24}H_{23}NO_2Na$ $[M+Na]^+$ requires 380.1621, found 380.1620 (-0.3 ppm)

(±)-1-Benzyl-4-(3,5-di-*tert*-butylphenyl)-1,2,3,4-tetrahydroquinoline (2at)

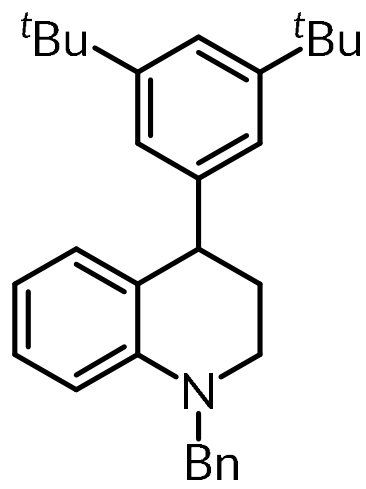

Following general procedure **E**, [Co-1] (1.2 mg, 0.002 mmol, 1.0 mol%), PXX (1.1 mg, 0.004 mmol, 2.0 mol%), Lut·HOTf (5.1 mg, 0.02 mmol, 10 mol%), **1at** (82.3 mg, 0.20 mmol), and PhCl (1.0 mL) for 16 h at 450 nm irradiation gave a ^{1}H-NMR yield of 80%. Purification by column chromatography (hexane/CH_2Cl_2, 97:3 → 95:5 → 90:10) gave the title compound **2at** (64.6 mg, 79%) as a yellow oil.

ν_{max} (film) / cm^{-1}: 2951, 2359, 1599, 1504, 1451, 1362, 1248, 870, 743, 729, 694

^{1}H NMR (500 MHz, $CDCl_3$) δ_H: 7.39 – 7.31 (5H, m), 7.30 – 7.25 (1H, m), 7.10 – 7.00 (3H, m), 6.84 (1H, d, *J* 7.3), 6.64 (1H, d, *J* 8.2), 6.57 (1H, t, *J* 7.3), 4.63 (1H, d, *J* 17.0), 4.56 (1H, d, *J* 16.9), 4.21 (1H, t, *J* 5.8), 3.42 – 3.35 (2H, m), 2.40 – 2.27 (1H, m), 2.23 – 2.14 (1H, m), 1.34 (18H, s)

$^{13}C\{^1H\}$ NMR (126 MHz, $CDCl_3$) δ_C: 150.6, 145.7, 145.2, 139.1, 130.1, 128.7, 127.7, 126.9, 126.8, 124.7, 123.1, 120.1, 116.0, 111.0, 55.4, 47.3, 44.1, 35.0, 31.7, 30.7

HRMS: (ESI$^+$) $C_{30}H_{34}N$ [M-3H]$^+$ requires 408.2686, found 408.2684 (-0.5 ppm)

(±)-1-Benzyl-4-(2-bromo-5-chlorophenyl)-1,2,3,4-tetrahydroquinoline (2au)

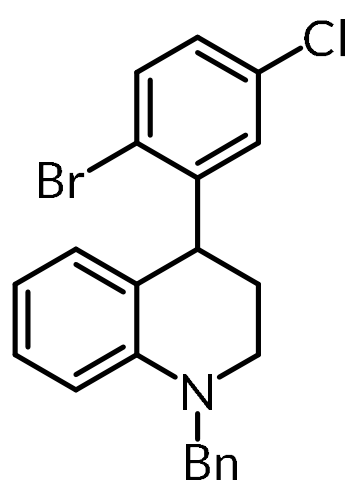

Following <u>modified</u> general procedure **E**, [Co-1] (1. 2 mg, 0.002 mmol, 1.0 mol%), PXX (1.1 mg, 0.004 mmol, 2.0 mol%), Lut·HOTf (5.1 mg, 0.02 mmol, 10 mol%), **1au** (82.6 mg, 0.20 mmol), and PhCl (1.0 mL) for <u>40 h</u> at 450 nm irradiation gave a ^{1}H-NMR yield of 69%. Purification by column chromatography (hexane/CH_2Cl_2, 97:3 → 95:5 → 90:10) gave the title compound **2au** (54.6 mg, 66%) as a yellow oil.

ν_{max} (film) / cm^{-1}: 2922, 2359, 1601, 1504, 1450, 1384, 1335, 1096, 1020, 808, 727, 694

^{1}H NMR (500 MHz, CDCl$_3$) δ$_H$: 7.52 (1H, d, *J* 8.4), 7.42 – 7.20 (5H, m), 7.13 – 7.02 (2H, m), 6.90 (1H, d, *J* 2.6), 6.80 (1H, d, *J* 7.4), 6.67 (1H, d, *J* 8.4), 6.60 (1H, t, *J* 7.4), 4.64 – 4.59 (2H, m), 4.54 (1H, d, *J* 16.9), 3.36 – 3.19 (2H, m), 2.34 – 2.22 (1H, m), 2.20 – 2.08 (1H, m)

^{13}C{^{1}H} NMR (126 MHz, CDCl$_3$) δ$_C$: 146.9, 145.9, 138.7, 134.0, 133.5, 131.1, 130.2, 128.8, 128.4, 128.1, 127.1, 126.7, 122.3, 122.1, 116.4, 111.6, 55.3, 46.2, 42.9, 28.1

HRMS: (ESI$^+$) $C_{22}H_{16}{}^{79}Br^{35}ClN$ [M-3H]$^+$ requires 408.0149, found 408.0148 (-0.2 ppm)

(±)-1-Benzyl-4-(2-(trifluoromethoxy)phenyl)-1,2,3,4-tetrahydroquinoline (2av)

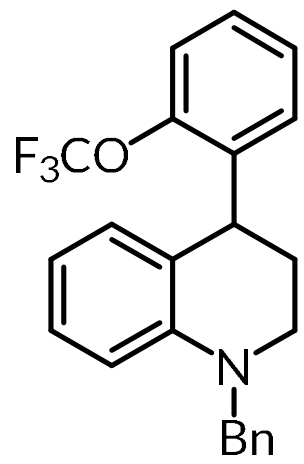

Following general procedure **E**, [Co-1] (1.2 mg, 0.002 mmol, 1.0 mol%), PXX (1.1 mg, 0.004 mmol, 2.0 mol%), Lut·HOTf (5.1 mg, 0.02 mmol, 10 mol%), **1av** (76.7 mg, 0.20 mmol), and PhCl (1.0 mL) for 16 h at 450 nm irradiation gave a ^{1}H-NMR yield of 90%. Purification by column chromatography (hexane/CH$_2$Cl$_2$, 97:3 → 95:5 → 90:10) gave the title compound **2av** (61.9 mg, 81%) as a yellow oil.

ν$_{max}$ (film) / cm^{-1}: 2926, 2357, 1601, 1504, 1450, 1246, 1204, 1150, 743, 725, 694

^{1}H NMR (500 MHz, CDCl$_3$) δ$_H$: 7.42 – 7.24 (7H, m), 7.20 (1H, td, *J* 7.5, 1.4), 7.08 (1H, ddd, *J* 8.6, 7.2, 1.7), 7.01 (1H, dd, *J* 7.7, 1.7), 6.78 (1H, d, *J* 7.2), 6.67 (1H, d, *J* 8.3), 6.59 (1H, t, *J* 7.3), 4.63 (1H, t, *J* 5.7), 4.58 (2H, s), 3.43 – 3.35 (1H, m), 3.34 – 3.24 (1H, m), 2.38 – 2.25 (1H, m), 2.19 – 2.08 (1H, m)

^{13}C{^{1}H} NMR (126 MHz, CDCl$_3$) δ$_C$: 147.2, 146.2, 138.9, 138.5, 131.3, 130.1, 128.8, 128.1, 127.6, 127.1, 126.8, 126.7, 123.2, 120.9 (q, *J* 257.3), 120.3, 116.3, 111.4, 55.4, 46.7, 36.8, 29.1

^{19}F NMR (471 MHz, CDCl$_3$) δ$_F$: -56.7 (s)

HRMS: (ESI$^+$) $C_{23}H_{17}F_3NO$ [M-3H]$^+$ requires 380.1257, found 380.1255 (-0.5 ppm)

(±)-1-Benzyl-4-(4-furan-2-yl)-1,2,3,4-tetrahydroquinoline (2aw)

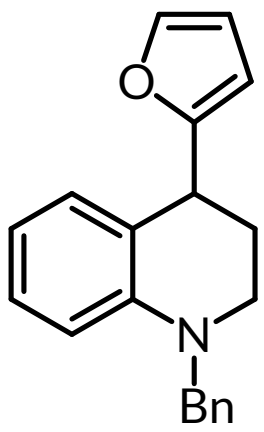

Following general procedure **E**, [Co-1] (1.2 mg, 0.002 mmol, 1.0 mol%), PXX (1.1 mg, 0.004 mmol, 2.0 mol%), Lut·HOTf (5.1 mg, 0.02 mmol, 10 mol%), **1aw** (57.9 mg, 0.20 mmol), and PhCl (1.0 mL) for 16 h at 450 nm irradiation gave a ^{1}H-NMR yield of 91%. Purification by column chromatography (hexane/CH_2Cl_2, 95:5 → 90:10 → 80:20) gave the title compound **2aw** (49.0 mg, 85%) as an orange oil.

ν_{max}(film) / cm^{-1}: 1601, 1501, 1450, 1350, 1337, 1007, 974, 727

^{1}H NMR (500 MHz, CDCl$_3$) δ_H: 7.39 – 7.35 (1H, m), 7.35 – 7.29 (2H, m), 7.28 – 7.22 (3H, m), 7.08 – 7.01 (1H, m), 6.98 (1H, d, *J* 7.3), 6.63 – 6.53 (2H, m), 6.33 – 6.27 (1H, m), 5.88 – 5.84 (1H, m), 4.51 (2H, s), 4.22 (1H, t, *J* 4.9), 3.36 – 3.26 (2H, m), 2.40 – 2.30 (1H, m), 2.27 – 2.17 (1H, m)

^{13}C{^{1}H} NMR (126 MHz, CDCl$_3$) δ_C: 158.5, 145.4, 141.5, 138.8, 130.0, 128.7, 128.2, 126.9, 126.7, 121.4, 116.0, 111.5, 110.2, 107.4, 55.3, 46.7, 37.2, 27.0

HRMS: (ESI$^+$) $C_{20}H_{19}NONa$ [M+Na]$^+$ requires 312.1359, found 312.1363 (+1.3 ppm)

(±)-4-(Benzo[*b*]thiophen-3-yl)-1-benzyl-1,2,3,4-tetrahydroquinoline (2ax)

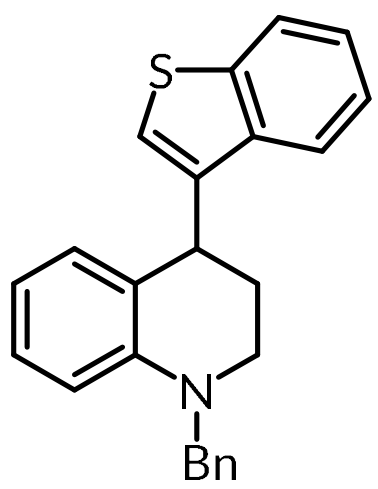

Following general procedure **E**, [Co-1] (1.2 mg, 0.002 mmol, 1.0 mol%), PXX (1.1 mg, 0.004 mmol, 2.0 mol%), Lut·HOTf (5.1 mg, 0.02 mmol, 10 mol%), **1ax** (71.1 mg, 0.20 mmol), and PhCl (1.0 mL) for 16 h at 450 nm irradiation gave a ^{1}H-NMR yield of 75%. Purification by column chromatography (hexane/CH_2Cl_2, 95:5 → 90:10 → 80:20) gave the title compound **2ax** (43.5 mg, 61%) as a yellow oil.

ν_{max}(film) / cm^{-1}: 1601, 1503, 1493, 1450, 1348, 1335, 1250, 907, 762, 727, 694

^{1}H NMR (500 MHz, $CDCl_3$) δ_H: 7.89 (1H, d, *J* 7.9), 7.85 (1H, d, *J* 7.8), 7.44 – 7.30 (6H, m), 7.27 (1H, t, *J* 6.8), 7.11 – 7.04 (1H, m), 6.94 (1H, dd, *J* 7.5, 1.7), 6.75 (1H, s), 6.67 (1H, d, *J* 8.3), 6.59 (1H, t, *J* 7.3), 4.60 (1H, t, *J* 4.9), 4.56 (2H, s), 3.35 – 3.24 (2H, m), 2.37 – 2.27 (2H, m)

^{13}C{^{1}H} NMR (126 MHz, $CDCl_3$) δ_C: 145.7, 141.1, 140.9, 138.9, 137.9, 130.1, 128.8, 128.1, 127.0, 126.8, 124.8, 124.3, 124.1, 123.3 (2C), 122.0, 116.3, 111.5, 55.4, 46.5, 37.8, 27.8

Note: 123.3 ppm peak contains two carbon peaks.

HRMS: (ESI$^+$) $C_{24}H_{22}NS$ [M+H]$^+$ requires 356.1467, found 356.1466 (-0.3 ppm)

(±)-1-Benzyl-4-(3-(4-fluorophenyl)-1-isopropyl-1*H*-indol-2-yl)-1,2,3,4-tetrahydroquinoline (2ay)

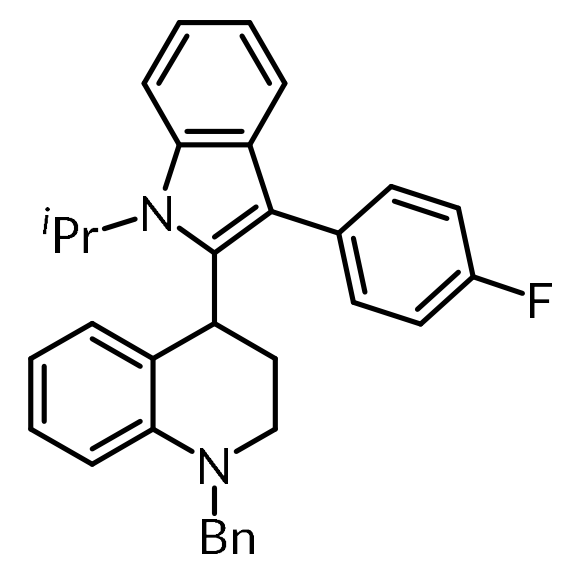

Following general procedure **E**, [Co-1] (1.2 mg, 0.002 mmol, 1.0 mol%), PXX (1.1 mg, 0.004 mmol, 2.0 mol%), Lut·HOTf (5.1 mg, 0.02 mmol, 10 mol%), **1ay** (94.9 mg, 0.20 mmol), and PhCl (1.0 mL) for 16 h at 450 nm irradiation gave a ^{1}H-NMR yield of 99%. Purification by column chromatography (hexane/CH_2Cl_2, 90:10 → 80:20 → 70:30) gave the title compound **2ay** (92.1 mg, 97%) as a beige solid.

mp 189 – 191 °C

ν_{max}(film) / cm^{-1}: 1599, 1495, 1454, 1350, 1337, 1301, 1217, 1153, 841

^{1}H NMR (500 MHz, $CDCl_3$) δ_H: 7.66 – 7.54 (2H, m), 7.53 – 7.41 (2H, m), 7.41 – 7.28 (5H, m), 7.22 (1H, t, *J* 7.7), 7.19 – 7.10 (3H, m), 7.03 (1H, t, *J* 7.8), 6.86 (1H, d, *J* 7.4), 6.64 (1H, d, *J* 8.4), 6.57 (1H, t, *J* 7.4), 4.77 (1H, dd, *J* 12.7, 5.8), 4.62 – 4.37 (3H, m), 3.58 – 3.49 (1H, m), 3.44 – 3.33 (1H, m), 2.58 – 2.41 (1H, m), 2.24 – 2.13 (1H, m), 1.72 (3H, d, *J* 7.0), 1.32 – 1.13 (3H, m)

^{13}C{^{1}H} NMR (126 MHz, $CDCl_3$) δ_C: 161.8 (d, *J* 245.1), 145.6, 138.2, 134.6, 131.8 (d, *J* 3.3), 131.7 (d, *J* 7.8), 128.9, 128.8, 128.6, 127.7, 127.1, 126.9, 123.7, 121.2, 116.9, 115.6 (d, *J* 21.8), 112.4, 111.9, 55.7, 50.0, 48.5, 35.6, 29.4, 21.4, 19.7

^{19}F NMR (471 MHz, $CDCl_3$) δ_F: -116.9 – -117.0 (m)

HRMS: (ESI$^+$) $C_{33}H_{31}FN_2Na$ [M+Na]$^+$ requires 497.2363, found 497.2361 (-0.4 ppm)

(±)-(*E*)-1-Benzyl-4-styryl-1,2,3,4-tetrahydroquinoline (2az)

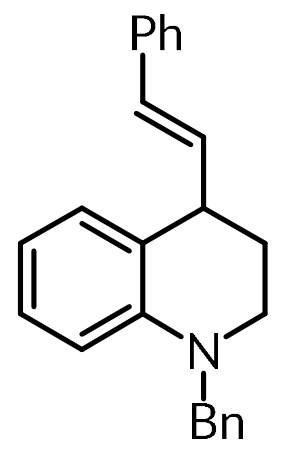

Following modified general procedure **E**, [Co-1] (1.2 mg, 0.002 mmol, 1.0 mol%), PXX (1.1 mg, 0.004 mmol, 2.0 mol%), Lut·HOTf (5.1 mg, 0.02 mmol, 10 mol%), **1az** (65.1 mg, 0.20 mmol), and PhCl (1.0 mL) for 40 h at 450 nm irradiation gave a ^{1}H-NMR yield of 60%. Purification by column chromatography (hexane/CH_2Cl_2, 95:5 → 90:10 → 80:20) gave the title compound **2az** (40.8 mg, 63%) as a pale-yellow oil.

ν_{max}(film) / cm^{-1}: 3022, 1599, 1495, 1449, 1339, 1296, 1250, 1169, 966, 743, 727, 691

^{1}H NMR (500 MHz, CDCl$_3$) δ_H: 7.44 – 7.39 (2H, m), 7.38 – 7.29 (6H, m), 7.29 – 7.22 (2H, m), 7.11 (1H, d, *J* 7.5), 7.09 – 7.01 (1H, m), 6.64 (1H, td, *J* 7.4, 1.1), 6.59 (1H, d, *J* 8.3), 6.41 (1H, d, *J* 15.8), 6.36 (1H, dd, *J* 15.8, 6.9), 4.56 (1H, d, *J* 17.0), 4.52 (1H, d, *J* 17.0), 3.73 (1H, q, *J* 6.4), 3.51 – 3.44 (1H, m), 3.44 – 3.38 (1H, m), 2.24 – 2.15 (1H, m), 2.09 – 2.01 (1H, m)

^{13}C{^{1}H} NMR (126 MHz, CDCl$_3$) δ_C: 145.3, 138.9, 137.6, 134.2, 131.3, 129.7, 128.74, 128.66, 127.9, 127.3, 126.9, 126.7, 126.3, 123.5, 116.0, 111.3, 55.3, 47.3, 41.2, 28.8

HRMS: (ESI$^+$) $C_{24}H_{24}N$ [M+H]$^+$ requires 326.1903, found 326.1901 (-0.6 ppm)

(±)-1-Benzyl-2-methyl-4-phenyl-1,2,3,4-tetrahydroquinoline (2ba)

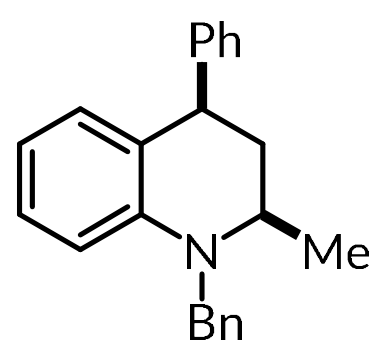

Following general procedure **E**, [Co-1] (1.2 mg, 0.002 mmol, 1.0 mol%), PXX (1.1 mg, 0.004 mmol, 2.0 mol%), Lut·HOTf (5.1 mg, 0.02 mmol, 10 mol%), **1ba** (62.7 mg, 0.20 mmol), and PhCl (1.0 mL) for 16 h at 450 nm irradiation gave a ^{1}H-NMR yield of 81%. Purification by column chromatography (hexane/CH_2Cl_2, 95:5 → 90:10 → 80:20) gave the title compound **2ba** (46.3 mg, 74%, inseparable diastereomers, *dr* = 1.8 : 1.0 (syn : anti)) as a pale-yellow oil.

ν_{max}(film) / cm^{-1}: 3026, 1597, 1493, 1450, 1331, 1244, 1028, 907, 725, 696

NMR data for *syn*-**2ba**

^{1}H NMR (500 MHz, CDCl$_3$) δ$_H$: 7.42 – 7.32 (6H, m), 7.31 – 7.21 (4H, m), 7.02 – 6.96 (1H, m), 6.58 (1H, d, *J* 7.1), 6.55 – 6.45 (2H, m), 4.66 (1H, d, *J* 17.5), 4.49 (1H, d, *J* 17.5), 4.22 – 4.11 (1H, m), 3.70 (1H, dqd, *J* 10.3, 6.2, 4.0), 2.32 – 2.16 (2H, m), 1.18 (3H, d, *J* 6.2)

^{13}C{^{1}H} NMR (126 MHz, CDCl$_3$) δ$_C$: 147.3, 145.1, 140.6, 128.9, 128.7, 128.61, 128.57, 127.6, 127.2, 126.7, 126.62, 126.56, 116.5, 112.9, 54.4, 54.3, 44.0, 41.3, 22.0

NMR data for *anti*-**2ba**

^{1}H NMR (500 MHz, CDCl$_3$) δ$_H$: 7.41 – 7.31 (6H, m), 7.31 – 7.20 (4H, m), 7.02 – 6.96 (1H, m), 6.71 (1H, d, *J* 7.1), 6.56 – 6.47 (2H, m), 4.61 (1H, d, *J* 17.3), 4.53 (1H, d, *J* 17.4), 4.22 – 4.11 (1H, m), 3.56 (1H, qt, *J* 6.4, 4.6), 2.31 – 2.16 (1H, m), 2.09 (1H, dt, *J* 13.0, 5.0), 1.27 (3H, d, *J* 6.5)

^{13}C{^{1}H} NMR (126 MHz, CDCl$_3$) δ$_C$: 146.0, 145.4, 139.7, 129.6, 128.9, 128.7, 128.6, 127.6, 126.8, 126.5, 126.4, 125.0, 115.9, 111.9, 53.8, 52.0, 40.5, 38.1, 19.8

HRMS: (ESI$^+$) $C_{23}H_{24}N$ [M+H]$^+$ requires 314.1903, found 314.1902 (-0.3 ppm)

(±)-1-Benzyl-4-methyl-1,2,3,4-tetrahydroquinoline (2bb)

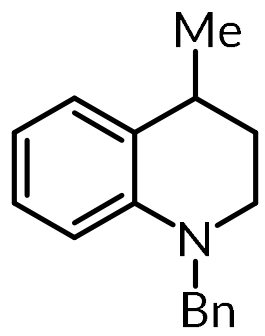

Following general procedure **E**, [Co-1] (1.2 mg, 0.002 mmol, 1.0 mol%), PXX (1.1 mg, 0.004 mmol, 2.0 mol%), Lut·HOTf (5.1 mg, 0.02 mmol, 10 mol%), **1bb** (47.5 mg, 0.20 mmol), and PhCl (1.0 mL) for 16 h at 450 nm irradiation gave a ^{1}H-NMR yield of 75%. Purification by column chromatography (hexane/CH_2Cl_2, 95:5 → 90:10 → 80:20) gave the title compound **2bb** (33.0 mg, 70%) as a pale-yellow oil.

ν_{max}(film) / cm^{-1}: 2922, 1601, 1501, 1450, 1339, 1304, 1252, 1204, 743, 729, 696

^{1}H NMR (500 MHz, CDCl$_3$) δ$_H$: 7.36 – 7.30 (2H, m), 7.30 – 7.23 (3H, m), 7.10 (1H, d, *J* 7.5), 7.05 – 6.95 (1H, m), 6.63 (1H, td, *J* 7.3, 1.1), 6.53 (1H, d, *J* 8.3), 4.56 – 4.47 (2H, m), 3.44 (1H, ddd, *J* 11.4, 9.0, 3.9), 3.34 (1H, ddd, *J* 11.2, 6.4, 4.3), 2.98 (1H, hex, *J* 6.7), 2.08 (1H, ddt, *J* 13.4, 9.1, 4.6), 1.77 (1H, dtd, *J* 12.7, 6.2, 3.9), 1.34 (3H, d, *J* 7.0)

^{13}C{^{1}H} NMR (126 MHz, CDCl$_3$) δ$_C$: 145.1, 139.0, 128.7, 128.0, 127.4, 127.3, 126.9, 126.7, 115.9, 111.1, 55.3, 46.8, 31.2, 29.8, 22.4

HRMS: (ESI$^+$) $C_{17}H_{20}N$ [M+H]$^+$ requires 238.1590, found 238.1590 (0.0 ppm)

(±)-1-Benzyl-6-(*tert*-butyl)-4-phenyl-1,2,3,4-tetrahydroquinoline (2bc)

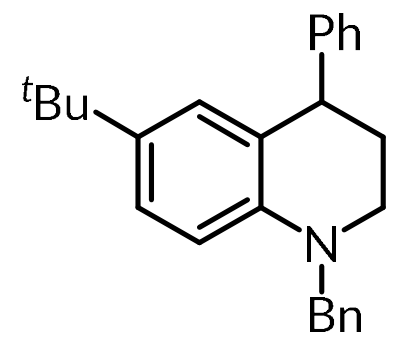

Following modified general procedure **E**, [Co-1] (2.4 mg, 0.004 mmol, 2.0 mol%), PXX (2.3 mg, 0.008 mmol, 4.0 mol%), Lut·HOTf (5.1 mg, 0.02 mmol, 10 mol%), **1bc** (71.1 mg, 0.20 mmol), and PhCl (1.0 mL) for 16 h at 450 nm irradiation gave a ^{1}H-NMR yield of 90%. Purification by column chromatography (hexane/CH_2Cl_2, 95:5 → 90:10 → 80:20) gave the title compound **2bc** (57.1 mg, 88%) as a beige solid.

mp 106 – 108 °C

ν_{max} (film) / cm^{-1}: 2951, 2903, 1510, 1248, 810, 732, 700

^{1}H NMR (500 MHz, CDCl$_3$) δ_H: 7.39 – 7.32 (6H, m), 7.30 – 7.23 (2H, m), 7.21 – 7.16 (2H, m), 7.10 (1H, dd, *J* 8.6, 2.5), 6.88 (1H, d, *J* 2.4), 6.61 (1H, d, *J* 8.6), 4.53 (2H, s), 4.25 (1H, t, *J* 5.5), 3.37 – 3.20 (2H, m), 2.39 – 2.31 (1H, m), 2.20 – 2.12 (1H, m), 1.22 (9H, s)

^{13}C{^{1}H} NMR (126 MHz, CDCl$_3$) δ_C: 146.6, 143.8, 139.4, 138.6, 128.8, 128.7, 128.3, 127.3, 126.9, 126.8, 126.1, 124.5, 123.4, 110.9, 55.6, 46.6, 43.7, 33.8, 31.6, 31.0

HRMS: (ESI$^+$) $C_{26}H_{26}N$ [M-3H]$^+$ requires 352.2060, found 352.2056 (-1.1 ppm)

(±)-1-Benzyl-6-methoxy-4-phenyl-1,2,3,4-tetrahydroquinoline (2bd)

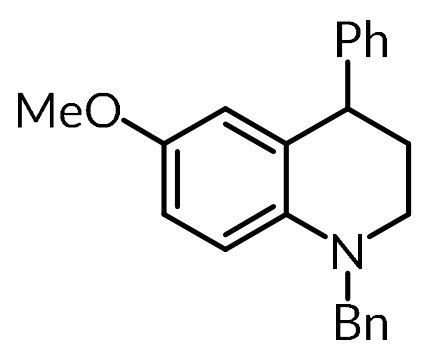

Following general procedure **E**, [Co-1] (1.2 mg, 0.002 mmol, 1.0 mol%), PXX (1.1 mg, 0.004 mmol, 2.0 mol%), Lut·HOTf (5.1 mg, 0.02 mmol, 10 mol%), **1bd** (65.9 mg, 0.20 mmol), and PhCl (1.0 mL) for 16 h at 450 nm irradiation gave a ^{1}H-NMR yield of 86%. Purification by column chromatography (hexane/CH_2Cl_2, 95:5 → 90:10 → 80:20 → 70:30) gave the title compound **2bd** (54.1 mg, 82%) as a yellow oil.

ν_{max} (film) / cm^{-1}: 3024, 2943, 1600, 1504, 1250, 1252, 1211, 1053, 729, 700

^{1}H NMR (500 MHz, CDCl$_3$) δ_H: 7.36 – 7.22 (8H, m), 7.15 (2H, d, *J* 7.7), 6.67 – 6.66 (1H, m), 6.59 – 6.57 (1H, m), 6.44 (1H, s), 4.49 (2H, s), 4.18 (1H, t, *J* 6.0), 3.64 (3H, s), 3.31 – 3.22 (2H, m), 2.34 – 2.28 (1H, m), 2.19 – 2.11 (1H, m)

$^{13}C\{^1H\}$ NMR (126 MHz, $CDCl_3$) δ_C: 151.0, 146.2, 140.6, 139.3, 128.8, 128.7, 128.4, 126.9 (2C), 126.3, 125.7, 116.2, 113.3, 112.5, 56.1, 55.8, 46.9, 43.9, 31.1

Note: 126.9 ppm peak contains two carbon peaks.

HRMS: (ESI$^+$) $C_{23}H_{23}NO$ [M]$^+$ requires 329.1774, found 329.1773 (-0.3 ppm)

(±)-1-Benzyl-6-fluoro-4-phenyl-1,2,3,4-tetrahydroquinoline (2be)

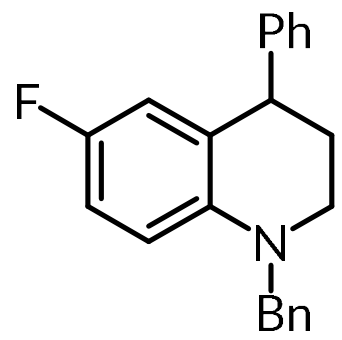

Following general procedure **E**, [Co-1] (1.2 mg, 0.002 mmol, 1.0 mol%), PXX (1.1 mg, 0.004 mmol, 2.0 mol%), Lut·HOTf (5.1 mg, 0.02 mmol, 10 mol%), **1be** (63.5 mg, 0.20 mmol), and PhCl (1.0 mL) for 16 h at 450 nm irradiation gave a ^{1}H-NMR yield of 92%. Purification by column chromatography (hexane/CH_2Cl_2, 95:5 → 90:10 → 80:20) gave the title compound **2be** (57.5 mg, 91%) as a yellow oil.

ν_{max} (film) / cm^{-1}: 3026, 2918, 1503, 1450, 1254, 1204, 797, 729, 698

^1H NMR (500 MHz, $CDCl_3$) δ_H: 7.42 – 7.25 (8H, m), 7.21 – 7.15 (2H, m), 6.77 (1H, td, *J* 8.6, 3.1), 6.59 – 6.51 (2H, m), 4.54 (2H, s), 4.19 (1H, t, *J* 5.9), 3.43 – 3.26 (2H, m), 2.38 – 2.28 (1H, m), 2.25 – 2.12 (1H, m)

$^{13}C\{^1H\}$ NMR (126 MHz, $CDCl_3$) δ_C: 154.9 (d, *J* 234.3), 145.6, 142.3 (d, *J* 1.6), 138.8, 128.8, 128.7, 128.6, 127.1, 126.7, 126.5, 125.7 (d, *J* 6.2), 116.4 (d, *J* 22.2), 114.0 (d, *J* 21.9), 112.0 (d, *J* 7.3), 55.9, 47.1, 43.7, 30.8

^{19}F NMR (471 MHz, $CDCl_3$) δ_F: -129.5 – -129.6 (m)

HRMS: (ESI$^+$) $C_{22}H_{17}FN$ [M-3H]$^+$ requires 314.1340, found 314.1338 (-0.6 ppm)

(±)-1-Benzyl-4-phenyl-6-(trifluoromethyl)-1,2,3,4-tetrahydroquinoline (2bf)

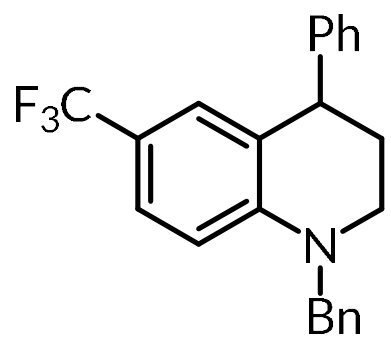

Following general procedure **E**, [Co-1] (1.2 mg, 0.002 mmol, 1.0 mol%), PXX (1.1 mg, 0.004 mmol, 2.0 mol%), Lut·HOTf (5.1 mg, 0.02 mmol, 10 mol%), **1bf** (73.5 mg, 0.20 mmol), and PhCl (1.0 mL)

for 16 h at 450 nm irradiation gave a ^{1}H-NMR yield of 95%. Purification by column chromatography (hexane/CH_2Cl_2, 97:3 → 95:5 → 90:10) gave the title compound **2bf** (61.1 mg, 83%) as a yellow oil.

ν_{max} (film) / cm^{-1}: 3030, 2945, 1616, 1524, 1323, 1097, 1086, 729

^{1}H NMR (500 MHz, $CDCl_3$) δ_H: 7.44 – 7.23 (9H, m), 7.17 – 7.09 (3H, m), 6.64 (1H, d, *J* 8.7), 4.63 (1H, d, *J* 17.2), 4.59 (1H, d, *J* 17.1), 4.25 (1H, t, *J* 5.3), 3.44 – 3.31 (2H, m), 2.38 – 2.28 (1H, m), 2.25 – 2.15 (1H, m)

^{13}C{^{1}H} NMR (126 MHz, $CDCl_3$) δ_C: 148.0, 145.1, 137.8, 128.9, 128.63, 128.56, 127.3, 127.1 (q, *J* 3.8), 126.6, 126.5, 125.22 (q, *J* 270.3), 125.19 (q, *J* 3.8), 123.5, 117.4 (q, *J* 32.5), 110.4, 55.1, 46.6, 43.2, 30.0

^{19}F NMR (471 MHz, $CDCl_3$) δ_F: -60.7 (s)

HRMS: (ESI$^+$) $C_{23}H_{17}F_3N$ [M-3H]$^+$ requires 364.1308, found 364.1303 (-1.4 ppm)

(±)-1-Benzyl-4-phenyl-1,2,3,4-tetrahydroquinoline-6-carbonitrile (2bg)

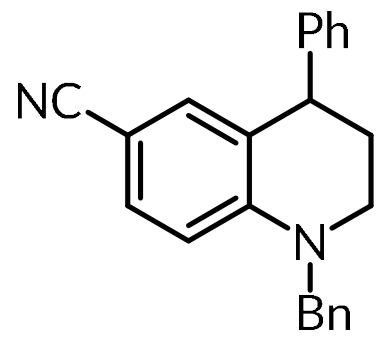

Following modified general procedure **E**, [Co-1] (1.2 mg, 0.002 mmol, 1.0 mol%), PXX (1.1 mg, 0.004 mmol, 2.0 mol%), Lut·HOTf (5.1 mg, 0.02 mmol, 10 mol%), **1bg** (64.9 mg, 0.20 mmol), and PhCl (1.0 mL) for 64 h at 450 nm irradiation gave a ^{1}H-NMR yield of 98%. Purification by column chromatography (hexane/CH_2Cl_2, 90:10 → 75:25 → 50:50) gave the title compound **2bg** (52.3 mg, 81%) as a yellow oil.

ν_{max} (film) / cm^{-1}: 3026, 2922, 2210 (C≡N), 1601, 1514, 1493, 1339, 1196, 731, 698

^{1}H NMR (500 MHz, $CDCl_3$) δ_H: 7.40 – 7.33 (4H, m), 7.32 – 7.22 (5H, m), 7.14 – 7.09 (2H, m), 7.07 – 7.02 (1H, m), 6.57 (1H, d, *J* 8.7), 4.66 – 4.56 (2H, m), 4.16 (1H, dd, *J* 6.8, 4.7), 3.46 (1H, ddd, *J* 11.3, 6.6, 4.3), 3.38 (1H, ddd, *J* 12.3, 8.4, 4.1), 2.35 – 2.25 (1H, m), 2.23 – 2.14 (1H, m)

^{13}C{^{1}H} NMR (126 MHz, $CDCl_3$) δ_C: 148.7, 144.3, 137.0, 133.6, 132.3, 129.0, 128.8, 128.4, 127.5, 126.9, 126.3, 124.5, 120.8, 110.8, 97.4, 54.9, 47.1, 43.0, 29.6

HRMS: (ESI$^+$) $C_{23}H_{21}N_2$ [M+H]$^+$ requires 325.1699, found 325.1703 (+1.2 ppm)

(±)-1-(1-Benzyl-4-phenyl-1,2,3,4-tetrahydroquinolin-6-yl)ethan-1-one (2bh)

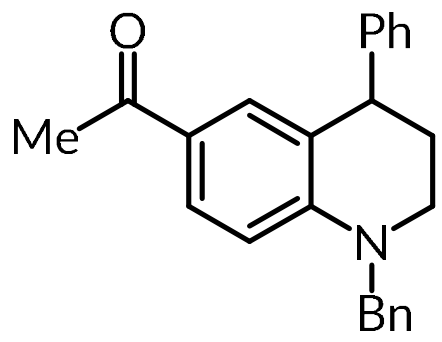

Following general procedure **E**, [Co-1] (1.2 mg, 0.002 mmol, 1.0 mol%), PXX (1.1 mg, 0.004 mmol, 2.0 mol%), Lut·HOTf (5.1 mg, 0.02 mmol, 10 mol%), **1bh** (68.3 mg, 0.20 mmol), and PhCl (1.0 mL) for 16 h at 450 nm irradiation gave a ^{1}H-NMR yield of 99%. Purification by column chromatography (hexane/CH_2Cl_2, 90:10 then hexane/EtOAc, 97:3 → 90:10 → 80:20) gave the title compound **2bh** (66.0 mg, 97%) as a yellow solid.

mp 107 – 109 °C

ν_{max} (film) / cm^{-1}: 2951, 1655, 1589, 1520, 1292, 1244, 795, 733

^{1}H NMR (500 MHz, $CDCl_3$) δ_H: 7.72 (1H, dd, *J* 8.8, 2.3), 7.55 (1H, d, *J* 2.2), 7.39 – 7.21 (8H, m), 7.13 – 7.08 (2H, m), 6.63 (1H, d, *J* 8.8), 4.66 (1H, d, *J* 17.0), 4.61 (1H, d, *J* 17.0), 4.26 (1H, t, *J* 5.0), 3.47 – 3.28 (2H, m), 2.41 (3H, s), 2.35 – 2.28 (1H, m), 2.22 – 2.11 (1H, m)

^{13}C{^{1}H} NMR (126 MHz, $CDCl_3$) δ_C: 196.3, 149.6, 145.1, 137.4, 131.2, 129.6, 128.9, 128.54, 128.47, 127.3, 126.5, 126.4, 125.5, 122.8, 110.1, 54.9, 46.4, 43.0, 29.8, 26.0

HRMS: (ESI$^+$) $C_{24}H_{23}NONa$ [M+Na]$^+$ requires 364.1672, found 364.1667 (-1.4 ppm)

(±)-2-(1-Benzyl-4-phenyl-1,2,3,4-tetrahydroquinolin-6-yl)acetonitrile (2bi)

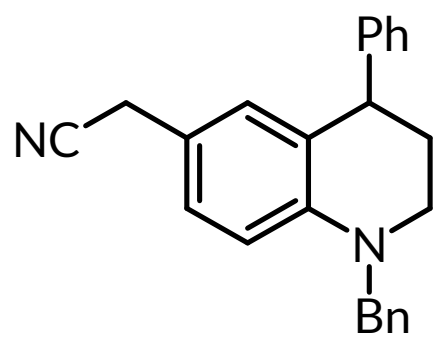

Following general procedure **E**, [Co-1] (1.2 mg, 0.002 mmol, 1.0 mol%), PXX (1.1 mg, 0.004 mmol, 2.0 mol%), Lut·HOTf (5.1 mg, 0.02 mmol, 10 mol%), **1bi** (67.7 mg, 0.20 mmol), and PhCl (1.0 mL) for 16 h at 450 nm irradiation gave a ^{1}H-NMR yield of 82%. Purification by column chromatography (hexane/CH_2Cl_2, 90:10 then hexane/EtOAc, 95:5 → 90:10) gave the title compound **2bi** (51.4 mg, 76%) as a yellow oil.

ν_{max} (film) / cm^{-1}: 2947, 2247 (C≡N), 1510, 1492, 1196, 802, 731, 696

^{1}H NMR (500 MHz, $CDCl_3$) δ_H: 7.40 – 7.22 (8H, m), 7.13 (2H, d, *J* 7.0), 6.98 (1H, dd, *J* 8.5, 2.4), 6.74 (1H, d, *J* 2.3), 6.60 (1H, d, *J* 8.5), 4.55 (2H, s), 4.20 (1H, t, *J* 5.5), 3.56 – 3.49 (2H, m), 3.40 – 3.25 (2H, m), 2.36 – 2.26 (1H, m), 2.21 – 2.09 (1H, m)

$^{13}C\{^1H\}$ NMR (126 MHz, $CDCl_3$) δ_C: 145.7, 145.4, 138.4, 129.7, 128.8, 128.6, 128.5, 127.5, 127.1, 126.6, 126.5, 124.6, 118.8, 116.6, 111.6, 55.3, 46.6, 43.3, 30.5, 22.7

HRMS: (ESI$^+$) $C_{24}H_{22}N_2Na$ $[M+Na]^+$ requires 361.1675, found 361.1679 (+1.1 ppm)

(±)-1-Benzyl-5,6,7-trifluoro-4-phenyl-1,2,3,4-tetrahydroquinoline (2bj)

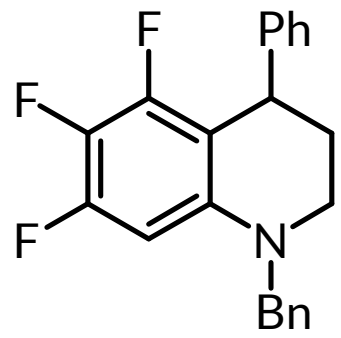

Following modified general procedure **E**, [Co-1] (1.2 mg, 0.002 mmol, 1.0 mol%), PXX (1.1 mg, 0.004 mmol, 2.0 mol%), Lut·HOTf (5.1 mg, 0.02 mmol, 10 mol%), **1bj** (70.7 mg, 0.20 mmol), and PhCl (1.0 mL) for 40 h at 450 nm irradiation gave a ^{1}H-NMR yield of 85%. Purification by column chromatography (hexane/CH_2Cl_2, 97:3 → 95:5 → 90:10) gave the title compound **2bj** (58.5 mg, 83%) as a yellow oil.

ν_{max} (film) / cm^{-1}: 3026, 2926, 1645, 1514, 1450, 1192, 1053, 953, 754, 729, 696

^1H NMR (500 MHz, $CDCl_3$) δ_H: 7.41 – 7.19 (8H, m), 7.08 (2H, d, *J* 7.2), 6.20 (1H, ddd, *J* 13.6, 6.1, 2.1), 4.48 (2H, s), 4.47 – 4.44 (1H, m), 3.22 (1H, td, *J* 12.2, 3.1), 3.19 – 3.14 (1H, m), 2.26 (1H, tt, *J* 12.8, 4.9), 2.06 (1H, dq, *J* 13.2, 2.9)

$^{13}C\{^1H\}$ NMR (126 MHz, $CDCl_3$) δ_C: 150.8 (ddd, *J* 242.4, 10.1, 6.5), 149.6 (ddd, *J* 243.1, 10.0, 6.1), 144.6, 141.5 (t, *J* 9.6), 137.6, 131.7 (dt, *J* 238.6, 16.1), 129.0, 128.5, 127.9, 127.4, 126.6, 126.5, 106.9 (d, *J* 17.5), 94.3 (d, *J* 22.2), 55.6, 44.7, 35.5 (d, *J* 3.9), 29.3

^{19}F NMR (471 MHz, $CDCl_3$) δ_F: -137.2 (ddd, *J* 21.8, 13.7, 6.9), -138.2 (dd, *J* 22.2, 6.8), -177.6 (td, *J* 22.5, 6.1)

HRMS: (ESI$^+$) $C_{22}H_{18}F_3NNa$ $[M+Na]^+$ requires 376.1284, found 376.1284 (0.0 ppm)

(±)-1-Benzyl-4,7-diphenyl-1,2,3,4-tetrahydroquinoline (2bk-a) and (±)-1-Benzyl-4,5-diphenyl-1,2,3,4-tetrahydroquinoline (2bk-b)

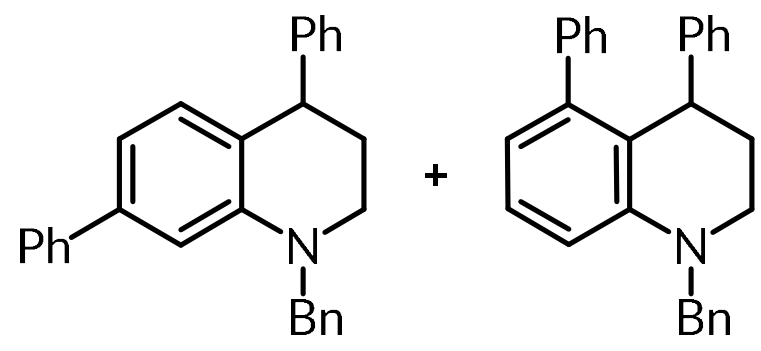

Following general procedure **E**, [Co-1] (1.2 mg, 0.002 mmol, 1.0 mol%), PXX (1.1 mg, 0.004 mmol, 2.0 mol%), Lut·HOTf (5.1 mg, 0.02 mmol, 10 mol%), **1bk** (75.1 mg, 0.20 mmol), and PhCl (1.0 mL) for 16 h at 450 nm irradiation gave a ^{1}H-NMR yield of 98% with a r.r. of 1.2 to 1.0. Purification by column chromatography (hexane/CH_2Cl_2, 97:3 → 90:10) gave the title compounds **2bk-a** + **2bk-b** (67.6 mg, 90%, mostly inseparable mix of regioisomers 1.2 to 1.0) as an orange oil. Small amounts of pure regioisomers were obtained for characterisation.

Data for major regioisomer **2bk-a** (Orange oil)

ν_{max} (film) / cm^{-1}: 3024, 2920, 1599, 1558, 1489, 758, 727, 694

^{1}H NMR (500 MHz, $CDCl_3$) δ_H: 7.48 – 7.45 (2H, m), 7.38 – 7.31 (8H, m), 7.30 – 7.22 (3H, m), 7.22 – 7.17 (2H, m), 6.87 (1H, d, *J* 7.8), 6.85 (1H, d, *J* 1.7), 6.79 (1H, dd, *J* 7.7, 1.7), 4.62 (2H, s), 4.24 (1H, t, *J* 5.7), 3.43 – 3.27 (2H, m), 2.39 – 2.28 (1H, m), 2.23 – 2.14 (1H, m)

^{13}C{^{1}H} NMR (126 MHz, $CDCl_3$) δ_C: 146.14, 146.06, 142.1, 140.9, 138.9, 130.5, 128.81, 128.79, 128.6, 128.5, 127.2, 127.1, 127.0, 126.9, 126.4, 123.5, 115.1, 110.0, 55.4, 46.9, 43.4, 30.8

HRMS: (ESI$^+$) $C_{28}H_{22}N$ [M-3H]$^+$ requires 372.1747, found 372.1742 (-1.3 ppm)

Data for minor regioisomer B-**2bk-b** (Orange oil)

ν_{max} (film) / cm^{-1}: 3057, 2918, 1570, 1489, 1449, 756, 729, 694

^{1}H NMR (500 MHz, $CDCl_3$) δ_H: 7.37 – 7.14 (11H, m), 7.12 (1H, dd, *J* 8.4, 7.4), 7.00 – 6.87 (4H, m), 6.67 (1H, d, *J* 8.3), 6.54 (1H, dd, *J* 7.5, 1.2), 4.65 (1H, d, *J* 17.0), 4.53 (1H, d, *J* 16.9), 4.13 (1H, t, *J* 3.6), 3.23 – 3.10 (2H, m), 2.27 – 2.14 (1H, m), 1.98 – 1.86 (1H, m)

^{13}C{^{1}H} NMR (126 MHz, $CDCl_3$) δ_C: 146.3, 145.7, 142.8, 142.1, 138.9, 129.2, 128.8, 128.5, 128.2, 127.5, 127.4, 127.0, 126.7, 126.6, 125.9, 121.0, 118.2, 110.4, 55.4, 44.9, 40.2, 29.7

HRMS: (ESI$^+$) $C_{28}H_{22}N$ [M-3H]$^+$ requires 372.1747, found 372.1744 (-0.8 ppm)

(±)-1-Benzyl-7-(*tert*-butyl)-4-phenyl-1,2,3,4-tetrahydroquinoline (2bl)

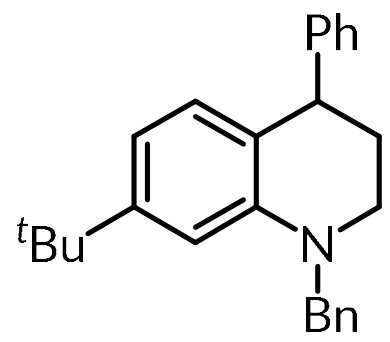

Following modified general procedure **E**, [Co-1] (2.4 mg, 0.004 mmol, 2.0 mol%), PXX (2.3 mg, 0.008 mmol, 4.0 mol%), Lut·HOTf (5.1 mg, 0.02 mmol, 10 mol%), **1bl** (71.1 mg, 0.20 mmol), and PhCl (1.0 mL) for 16 h at 450 nm irradiation gave a ^{1}H-NMR yield of 75% with a r.r. of 13.0 to 1.0. Purification by column chromatography (hexane/CH_2Cl_2, 95:5 → 90:10 → 80:20) gave the title compound **2bl** (50.6 mg, 71%, single regioisomer) as a yellow oil.

ν_{max} (film) / cm^{-1}: 2951, 2862, 1603, 1491, 1451, 1327, 731, 698

^{1}H NMR (500 MHz, CDCl$_3$) δ_H: 7.40 – 7.23 (8H, m), 7.22 – 7.19 (2H, m), 6.74 (1H, d, *J* 7.9), 6.70 (1H, d, *J* 1.9), 6.62 (1H, dd, *J* 7.9, 1.9), 4.58 (2H, s), 4.19 (1H, t, *J* 6.0), 3.43 – 3.29 (2H, m), 2.38 – 2.28 (1H, m), 2.22 – 2.14 (1H, m), 1.24 (9H, s)

^{13}C{^{1}H} NMR (126 MHz, CDCl$_3$) δ_C: 150.5, 146.4, 145.4, 139.4, 129.6, 128.8, 128.7, 128.4, 127.0, 126.9, 126.2, 121.7, 113.3, 108.9, 55.9, 47.4, 43.3, 34.7, 31.5, 31.0

HRMS: (ESI$^+$) $C_{26}H_{26}N$ [M-3H]$^+$ requires 352.2060, found 352.2057 (-0.9 ppm)

(±)-1-Benzyl-7-methoxy-4-phenyl-1,2,3,4-tetrahydroquinoline (2bm-a) and (±)-1-Benzyl-5-methoxy-4-phenyl-1,2,3,4-tetrahydroquinoline (2bm-b)

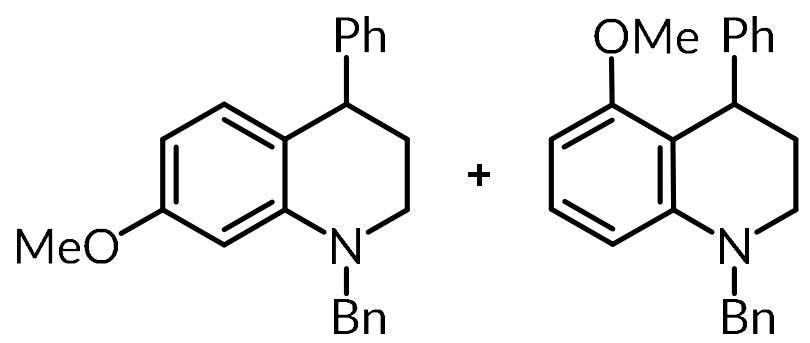

(±)-2bm-a *(major)* **(±)-2bm-b** *(minor)*

Following general procedure **E**, [Co-1] (1.2 mg, 0.002 mmol, 1.0 mol%), PXX (1.1 mg, 0.004 mmol, 2.0 mol%), Lut·HOTf (5.1 mg, 0.02 mmol, 10 mol%), **1bm** (65.9 mg, 0.20 mmol), and PhCl (1.0 mL) for 16 h at 450 nm irradiation gave a ^{1}H-NMR yield of 93% with a r.r. of 2.1 to 1.0. Purification by column chromatography (hexane/CH_2Cl_2, 95:5 → 85:15 → 75:25) gave **2bm-a** (37.3 mg, 57%) as a beige solid and **2bm-b** (18.0 mg, 27%) as a beige solid.

Data for major regioisomer **2bm-a**

mp 105 – 107 °C

ν_{max} (film) / cm^{-1}: 3024, 2922, 2359, 1611, 1506, 1450, 1207, 1163, 1049, 821, 731, 700

^{1}H NMR (500 MHz, CDCl$_3$) δ_H: 7.38 – 7.30 (6H, m), 7.29 – 7.21 (2H, m), 7.19 – 7.15 (2H, m), 6.73 (1H, d, *J* 8.3), 6.21 (1H, d, *J* 2.4), 6.16 (1H, dd, *J* 8.3, 2.4), 4.54 (2H, s), 4.16 (1H, t, *J* 5.7), 3.70 (3H, s), 3.41 – 3.21 (2H, m), 2.38 – 2.24 (1H, m), 2.19 – 2.08 (1H, m)

^{13}C{^{1}H} NMR (126 MHz, CDCl$_3$) δ_C: 159.6, 146.8, 146.5, 138.8, 130.7, 128.75, 128.70, 128.4, 127.0, 126.7, 126.2, 117.2, 100.6, 97.7, 55.4, 55.1, 46.8, 42.8, 30.9

HRMS: (ESI$^+$) $C_{23}H_{23}NONa$ [M+Na]$^+$ requires 352.1672, found 352.1672 (0.0 ppm)

Data for minor regioisomer **2bm-b**

mp 99 – 101 °C

ν_{max} (film) / cm^{-1}: 3022, 2920, 2359, 1714, 1601, 1576, 1492, 1450, 1246, 1124, 721, 698

^{1}H NMR (500 MHz, CDCl$_3$) δ_H: 7.37 – 7.22 (7H, m), 7.21 – 7.16 (1H, m), 7.12 – 7.09 (2H, m), 7.06 (1H, t, *J* 8.2), 6.36 (1H, d, *J* 8.4), 6.26 (1H, dd, *J* 8.1, 0.9), 4.59 (1H, d, *J* 17.0), 4.56 (1H, d, *J* 5.1), 4.52 (1H, d, *J* 17.1), 3.64 (3H, s), 3.18 (1H, ddd, *J* 12.7, 11.6, 3.2), 3.15 – 3.09 (1H, m), 2.27 (1H, tt, *J* 12.9, 4.9), 2.02 (1H, dq, *J* 13.1, 2.8)

^{13}C{^{1}H} NMR (126 MHz, CDCl$_3$) δ_C: 157.8, 146.7, 146.6, 139.1, 128.7, 128.2, 128.1, 127.9, 126.9, 126.7, 125.7, 110.9, 104.7, 98.9, 55.7, 55.5, 44.6, 36.0, 29.5

HRMS: (ESI$^+$) $C_{23}H_{23}NONa$ [M+Na]$^+$ requires 352.1672, found 352.1669 (-0.9 ppm)

(±)-1-Benzyl-7-fluoro-4-phenyl-1,2,3,4-tetrahydroquinoline (2bn-a) and (±)-1-Benzyl-5-fluoro-4-phenyl-1,2,3,4-tetrahydroquinoline (2bn-b)

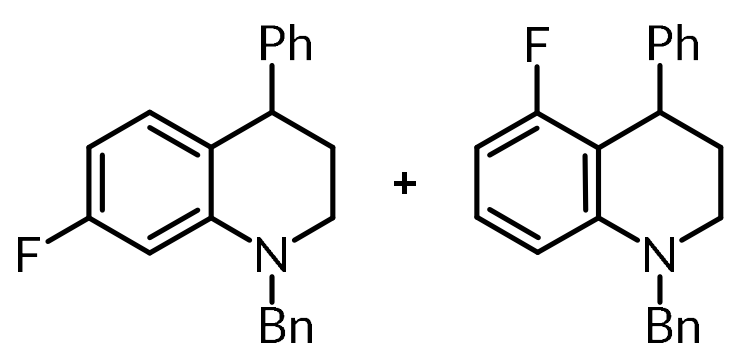

(±)-2bn-a *(major)* **(±)-2bn-b** *(minor)*

Following general procedure **E**, [Co-1] (1.2 mg, 0.002 mmol, 1.0 mol%), PXX (1.1 mg, 0.004 mmol, 2.0 mol%), Lut·HOTf (5.1 mg, 0.02 mmol, 10 mol%), **1bn** (63.5 mg, 0.20 mmol), and PhCl (1.0 mL) for 16 h at 450 nm irradiation gave a ^{1}H-NMR yield of 95% with a r.r. of 2.4 to 1.0. Purification by column chromatography (hexane/CH_2Cl_2, 97:3 → 90:10 → 85:15) gave the title compounds **2bn-a** + **2bn-b** (57.1 mg, 90%, inseparable mix of regioisomers 2.4 to 1.0) as a yellow oil.

ν_{max} (film) / cm^{-1}: 3026, 2922, 2853, 1616, 1504, 1493, 1450, 1161, 1109, 813, 713, 698

NMR data for major regioisomer **2bn-a**

^{1}H NMR (500 MHz, CDCl$_3$) δ_H: 7.41 – 7.20 (8H, m), 7.18 – 7.11 (2H, m), 6.78 – 6.73 (1H, m), 6.33 (1H, dd, *J* 12.5, 2.5), 6.27 (1H, td, *J* 8.3, 2.5), 4.55 – 4.51 (2H, m), 4.18 (1H, t, *J* 5.6), 3.39 – 3.27 (2H, m), 2.36 – 2.26 (1H, m), 2.20 – 2.12 (1H, m)

^{13}C{^{1}H} NMR (126 MHz, CDCl$_3$) δ_C: 163.1 (d, *J* 240.7), 147.0 (d, *J* 10.6), 145.9, 138.1, 131.0 (d, *J* 10.1), 128.9, 128.6, 128.5, 127.2, 126.6, 126.4, 119.7 (d, *J* 2.6), 102.2 (d, *J* 21.4), 98.0 (d, *J* 26.5), 55.3, 46.5, 42.8, 30.5

^{19}F NMR (471 MHz, CDCl$_3$) δ_F: -114.64 – -114.82 (m)

NMR data for minor regioisomer **2bn-b**

^{1}H NMR (500 MHz, CDCl$_3$) δ_H: 7.41 – 7.20 (8H, m), 7.18 – 7.11 (2H, m), 7.07 – 7.01 (1H, m), 6.46 (1H, d, *J* 8.4), 6.37 (1H, t, *J* 8.8), 4.58 (2H, d, *J* 8.0), 4.55 – 4.51 (1H, m), 3.26 – 3.22 (1H, m), 3.21 – 3.15 (1H, m), 2.36 – 2.26 (1H, m), 2.07 (1H, dq, *J* 13.2, 2.9)

^{13}C{^{1}H} NMR (126 MHz, CDCl$_3$) δ_C: 161.4 (d, *J* 242.1), 147.1 (d, *J* 8.2), 145.5, 138.5, 128.8, 128.37 (d, *J* 11.1), 128.36, 128.1, 127.1, 126.6, 126.3, 110.1 (d, *J* 20.3), 106.8 (d, *J* 2.4), 102.6 (d, *J* 22.7), 55.5, 44.6, 35.6 (d, *J* 4.9), 29.3

^{19}F NMR (471 MHz, CDCl$_3$) δ_F: -117.05 – -117.21 (m).

HRMS: (ESI$^+$) $C_{22}H_{17}FN$ [M-3H]$^+$ requires 314.1340, found 314.1338 (-0.6 ppm)

(±)-1-Benzyl-4-phenyl-7-(trifluoromethyl)-1,2,3,4-tetrahydroquinoline (2bo-a) and (±)-1-Benzyl-4-phenyl-5-(trifluoromethyl)-1,2,3,4-tetrahydroquinoline (2bo-b)

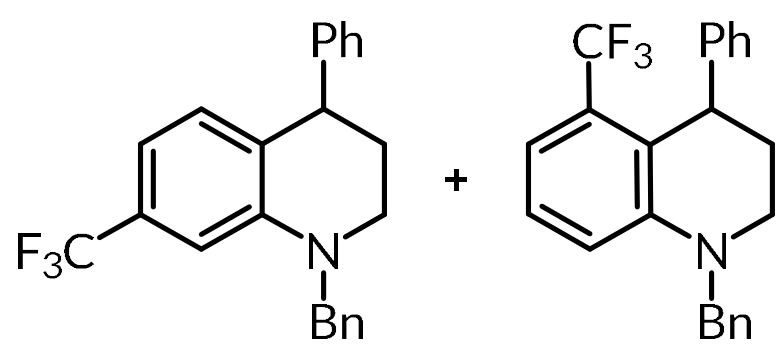

(±)-2bo-a *(major)* **(±)-2bo-b** *(minor)*

Following modified general procedure **E**, [Co-1] (2.4 mg, 0.004 mmol, 2.0 mol%), PXX (2.3 mg, 0.008 mmol, 4.0 mol%), Lut·HOTf (5.1 mg, 0.02 mmol, 10 mol%), **1bo** (73.5 mg, 0.20 mmol), and PhCl (1.0 mL) for 16 h at 450 nm irradiation gave a ^{1}H-NMR yield of 99% with a r.r. of 1.1 to 1.0. Purification by column chromatography (hexane/CH_2Cl_2, 97:3 → 95:5→ 90:10) gave the title compounds **2bo-a** + **2bo-b** (68.2 mg, 93%, inseparable mix of regioisomers 1.1 to 1.0) as a yellow oil.

ν_{max} (film) / cm^{-1}: 3026, 2924, 2854, 1593, 1492, 1450, 1308, 1248, 1157, 1111, 1053, 1028, 845, 727, 696, 667

^{1}H-NMR data for major regioisomer **2bo-a**

^{1}H NMR (500 MHz, CDCl$_3$) δ_H: 7.42 – 7.11 (10H, m), 6.91 (1H, d, *J* 7.8), 6.88 (1H, d, *J* 1.7), 6.84 – 6.79 (1H, m), 4.59 (2H, s), 4.23 (1H, t, *J* 5.7), 3.38 (1H, ddd, *J* 11.0, 6.3, 4.2), 3.32 (1H, ddd, *J* 12.0, 8.7, 3.9), 2.35 – 2.26 (1H, m), 2.23 – 2.14 (1H, m)

^{1}H-NMR data for minor regioisomer **2bo-b**

^{1}H NMR (500 MHz, CDCl$_3$) δ_H: 7.42 – 7.11 (9H, m), 7.02 (2H, d, *J* 7.6), 6.99 (1H, d, *J* 7.7), 6.84 – 6.79 (1H, m), 4.70 (1H, t, *J* 3.6), 4.65 (1H, d, *J* 17.2), 4.52 (1H, d, *J* 17.2), 3.26 (1H, dd, *J* 11.5, 5.1), 3.16 (1H, ddd, *J* 13.0, 11.4, 4.2), 2.35 – 2.26 (1H, m), 2.23 – 2.14 (1H, m)

^{13}C{^{1}H} NMR (126 MHz, CDCl$_3$) δ_C: 146.4, 145.9, 145.3, 144.7, 137.94, 137.92, 130.4, 130.1 (q, *J* 31.5), 129.0 (d, *J* 29.5), 128.9 (2C), 128.64, 128.61, 128.3, 127.9, 127.8, 127.7, 127.3, 127.2, 126.8, 126.6, 126.4, 126.1, 124.8 (d, *J* 274.7), 124.6 (d, *J* 272.3), 120.8, 114.8, 113.4 (q, *J* 6.2), 112.4 (q, *J* 3.8), 107.4 (q, *J* 4.0), 55.3, 55.1, 46.5, 44.6, 43.4, 38.8 – 38.4 (m), 30.2, 28.9

Note: 128.9 ppm peak contains two carbon peaks.

^{19}F NMR (471 MHz, CDCl$_3$) δ_F: -59.1 (s), -62.5 (s)

HRMS: (ESI$^+$) $C_{23}H_{19}F_3N$ [M-H]$^+$ requires 366.1464, found 366.1470 (+1.6 ppm)

(±)-1-Benzyl-4-phenyl-1,2,3,4-tetrahydrobenzo[*h*]quinoline (2bp)

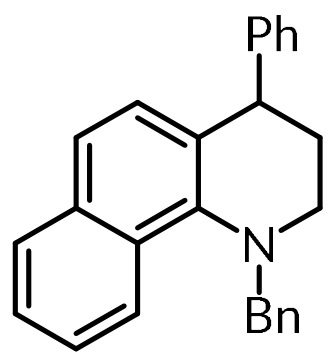

Following general procedure **E**, [Co-1] (1.2 mg, 0.002 mmol, 1.0 mol%), PXX (1.1 mg, 0.004 mmol, 2.0 mol%), Lut·HOTf (5.1 mg, 0.02 mmol, 10 mol%), **1bp** (69.9 mg, 0.20 mmol), and PhCl (1.0 mL) for 16 h at 450 nm irradiation gave a ^{1}H-NMR yield of 72%. Purification by column chromatography (hexane/CH_2Cl_2, 95:5 → 80:20 → 70:30) gave the title compound **2bp** (50.3 mg, 72%) as a brown oil.

ν_{max} (film) / cm^{-1}: 3059, 2937, 1600, 1493, 1450, 1398, 1362, 908, 731, 700

^{1}H NMR (500 MHz, CDCl$_3$) δ_H: 8.29 – 8.21 (1H, m), 7.83 – 7.76 (1H, m), 7.67 (2H, dd, *J* 8.1, 1.3), 7.50 – 7.35 (6H, m), 7.34 – 7.29 (2H, m), 7.26 – 7.21 (1H, m), 7.21 – 7.14 (2H, m), 7.01 (1H, d, *J* 8.5),

4.51 (1H, d, *J* 16.3), 4.47 (1H, d, *J* 16.3), 4.34 (1H, t, *J* 6.6), 3.30 – 3.15 (2H, m), 2.31 – 2.21 (1H, m), 2.03 – 1.93 (1H, m)

$^{13}C\{^1H\}$ NMR (126 MHz, $CDCl_3$) δ_C: 147.4, 145.1, 139.6, 133.9, 128.9, 128.8 (2C), 128.5 (2C), 127.5, 127.4, 127.2, 126.3, 125.61, 125.59, 123.4, 122.5, 59.3, 44.6, 44.1, 26.8

Note: Both 128.8 ppm and 128.5 ppm peaks contain two carbon peaks.

HRMS: (ESI^+) $C_{26}H_{23}N$ $[M]^+$ requires 349.1825, found 349.1830 (+1.4 ppm)

(±)-6-Phenyl-1,2,5,6-tetrahydro-4*H*-pyrrolo[3,2,1-*ij*]quinoline (2bq)

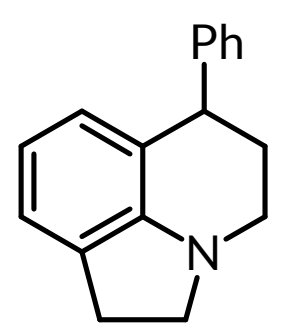

Following general procedure **E**, [Co-1] (1.2 mg, 0.002 mmol, 1.0 mol%), PXX (1.1 mg, 0.004 mmol, 2.0 mol%), Lut·HOTf (5.1 mg, 0.02 mmol, 10 mol%), **1bq** (47.1 mg, 0.20 mmol), and PhCl (1.0 mL) for 16 h at 450 nm irradiation gave a ^{1}H-NMR yield of 82%. Purification by column chromatography (hexane/CH_2Cl_2, 95:5 → 90:10 → 80:20 → 70:30) gave the title compound **2bq** (37.7 mg, 80%) as a yellow oil.

ν_{max} (film) / cm^{-1}: 3024, 2920, 1601, 1491, 1452, 1337, 1273, 750, 700

^1H NMR (500 MHz, $CDCl_3$) δ_H: 7.34 – 7.28 (2H, m), 7.25 – 7.20 (1H, m), 7.20 – 7.14 (2H, m), 7.01 – 6.96 (1H, m), 6.61 – 6.55 (2H, m), 4.10 (1H, dd, *J* 8.3, 6.1), 3.41 (1H, td, *J* 8.3, 5.5), 3.23 (1H, q, *J* 8.6), 3.14 – 3.07 (1H, m), 3.06 – 2.92 (3H, m), 2.42 – 2.32 (1H, m), 2.25 – 2.13 (1H, m)

$^{13}C\{^1H\}$ NMR (126 MHz, $CDCl_3$) δ_C: 150.3, 146.6, 128.9, 128.8, 128.5, 127.1, 126.4, 122.5, 122.2, 118.7, 55.3, 46.3, 41.7, 33.6, 29.1

HRMS: (ESI^+) $C_{17}H_{14}N$ $[M-3H]^+$ requires 232.1121, found 232.1118 (-1.3 ppm)

(±)-10-Chloro-1-phenyl-2,3,6,7-tetrahydro-1*H*-azepino[3,2,1-*ij*]quinolin-8(5*H*)-one (2br)

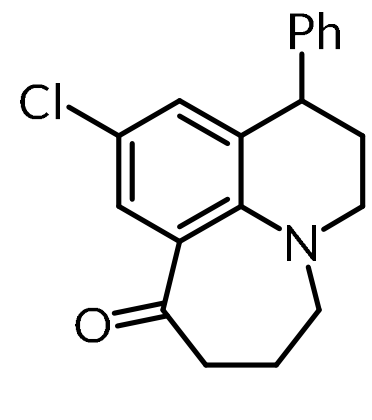

Following general procedure **E**, [Co-1] (1.2 mg, 0.002 mmol, 1.0 mol%), PXX (1.1 mg, 0.004 mmol, 2.0 mol%), Lut·HOTf (5.1 mg, 0.02 mmol, 10 mol%), **1br** (62.4 mg, 0.20 mmol), and PhCl (1.0 mL)

for 16 h at 450 nm irradiation gave a ^{1}H-NMR yield of 84%. Purification by column chromatography (hexane/CH_2Cl_2, 95:5 then hexane/Et_2O, 85:15 → 75:25) gave the title compound **2br** (51.2 mg, 82%) as a yellow solid.

mp 131 – 133 °C

ν_{max} (film) / cm^{-1}: 2926, 2860, 2359, 2341, 1653 (C=O), 1491, 1437, 1229, 831, 702

^{1}H NMR (500 MHz, $CDCl_3$) δ_H: 7.59 (1H, d, *J* 2.6), 7.32 (2H, dd, *J* 8.3, 6.8), 7.28 – 7.23 (1H, m), 7.09 – 7.03 (2H, m), 6.77 (1H, dd, *J* 2.7, 1.0), 4.09 (1H, dd, *J* 7.1, 4.6), 3.48 – 3.40 (1H, m), 3.37 – 3.21 (3H, m), 2.78 (2H, t, *J* 7.3), 2.35 – 2.26 (3H, m), 2.21 – 2.12 (1H, m)

^{13}C{^{1}H} NMR (126 MHz, $CDCl_3$) δ_C: 201.8, 149.7, 144.2, 132.2, 129.5, 128.8, 128.5, 127.7, 126.9, 126.4, 121.9, 56.1, 48.5, 43.3, 41.0, 31.9, 30.9

HRMS: (ESI$^+$) $C_{19}H_{18}{}^{35}ClNONa$ [M+Na]$^+$ requires 334.0969, found 334.0970 (+0.3 ppm)

(±)-4-Phenyl-1,2,3,4-tetrahydroquinoline (4a)

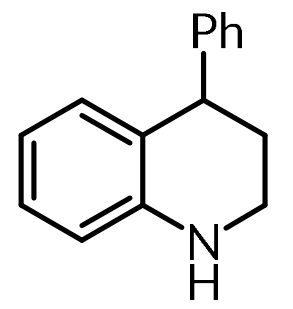

Following general procedure **F**, [Co-1] (1.2 mg, 0.002 mmol, 1.0 mol%), PXX (1.1 mg, 0.004 mmol, 2.0 mol%), Lut·HOTf (5.1 mg, 0.02 mmol, 10 mol%), **3a** (41.9 mg, 0.20 mmol), and PhCl (1.0 mL) for 24 h at 450 nm irradiation gave a ^{1}H-NMR yield of 75%. Purification by column chromatography (hexane/CH_2Cl_2, 97:3 → 95:5 → 90:10 → 85:15) gave the title compound **4a** (28.2 mg, 67%) as a brown solid.

mp 69 – 71 °C

ν_{max} (film) / cm^{-1}: 3408 (N–H), 3020, 2920, 1605, 1491, 1314, 747, 700

^{1}H NMR (500 MHz, $CDCl_3$) δ_H: 7.33 – 7.28 (2H, m), 7.24 – 7.19 (1H, m), 7.18 – 7.13 (2H, m), 7.05 – 7.00 (1H, m), 6.78 – 6.74 (1H, m), 6.62 – 6.54 (2H, m), 4.16 (1H, t, *J* 6.1), 3.90 (1H, br s), 3.34 – 3.28 (1H, m), 3.28 – 3.22 (1H, m), 2.27 – 2.19 (1H, m), 2.11 – 2.03 (1H, m)

^{13}C{^{1}H} NMR (126 MHz, $CDCl_3$) δ_C: 146.8, 145.1, 130.6, 128.8, 128.4, 127.4, 126.2, 123.5, 117.2, 114.3, 42.9, 39.3, 31.2

HRMS: (ESI$^+$) $C_{15}H_{16}N$ [M+H]$^+$ requires 210.1277, found 210.1275 (-1.0 ppm)

(±)-8-Methyl-4-phenyl-1,2,3,4-tetrahydroquinoline (4b)

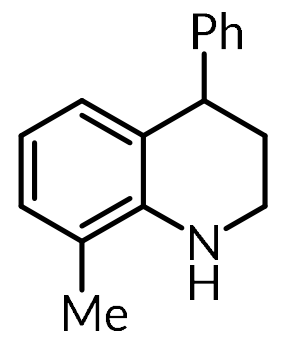

Following general procedure **F**, [Co-1] (1.2 mg, 0.002 mmol, 1.0 mol%), PXX (1.1 mg, 0.004 mmol, 2.0 mol%), Lut·HOTf (5.1 mg, 0.02 mmol, 10 mol%), **3b** (44.7 mg, 0.20 mmol), and PhCl (1.0 mL) for 24 h at 450 nm irradiation gave a ^{1}H-NMR yield of 80%. Purification by column chromatography (hexane/CH_2Cl_2, 97:3 → 95:5 → 90:10) gave the title compound **4b** (30.8 mg, 69%) as a brown oil.

ν_{max} (film) / cm^{-1}: 3427 (N–H), 2920, 2853, 1597, 1491, 1462, 1307, 1263, 742, 700

^{1}H NMR (500 MHz, $CDCl_3$) δ_H: 7.35 – 7.28 (2H, m), 7.25 – 7.20 (1H, m), 7.18 – 7.13 (2H, m), 6.96 (1H, d, *J* 6.7), 6.68 (1H, d, *J* 7.6), 6.54 (1H, t, *J* 7.5), 4.20 (1H, t, *J* 6.0), 3.80 (1H, br s), 3.43 – 3.37 (1H, m), 3.37 – 3.28 (1H, m), 2.28 – 2.20 (1H, m), 2.17 (3H, s), 2.13 – 2.05 (1H, m)

^{13}C{^{1}H} NMR (126 MHz, $CDCl_3$) δ_C: 147.0, 143.1, 128.8, 128.52, 128.47, 128.4, 126.2, 122.9, 121.2, 116.5, 43.1, 39.5, 31.1, 17.5

HRMS: (ESI$^+$) $C_{16}H_{18}N$ [M+H]$^+$ requires 224.1434, found 224.1433 (-0.4 ppm)

(±)-8-Bromo-4-phenyl-1,2,3,4-tetrahydroquinoline (4c)

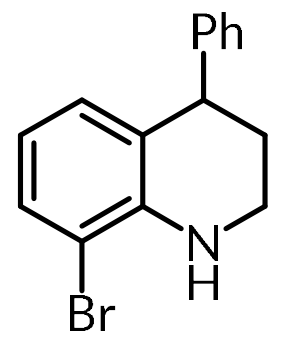

Following general procedure **F**, [Co-1] (1.2 mg, 0.002 mmol, 1.0 mol%), PXX (1.1 mg, 0.004 mmol, 2.0 mol%), Lut·HOTf (5.1 mg, 0.02 mmol, 10 mol%), **3c** (57.6 mg, 0.20 mmol), and PhCl (1.0 mL) for 24 h at 450 nm irradiation gave a ^{1}H-NMR yield of 88%. Purification by column chromatography (hexane/CH_2Cl_2, 95:5 → 90:10 → 85:15) gave the title compound **4c** (45.8 mg, 80%) as a yellow oil.

ν_{max} (film) / cm^{-1}: 3412 (N–H), 2922, 1597, 1491, 1458, 1354, 1294, 1130, 843, 750, 729, 698

^{1}H NMR (500 MHz, $CDCl_3$) δ_H: 7.36 – 7.29 (3H, m), 7.25 – 7.19 (1H, m), 7.17 – 7.09 (2H, m), 6.73 (1H, d, *J* 7.2), 6.44 (1H, t, *J* 7.7), 4.59 (1H, br s), 4.17 (1H, t, *J* 5.9), 3.50 – 3.36 (1H, m), 3.36 – 3.27 (1H, m), 2.26 – 2.15 (1H, m), 2.13 – 2.02 (1H, m)

$^{13}C\{^1H\}$ NMR (126 MHz, $CDCl_3$) δ_C: 146.1, 142.0, 130.7, 129.6, 128.7, 128.5, 126.4, 124.9, 117.1, 108.9, 43.2, 39.1, 30.5

HRMS: (ESI^+) $C_{15}H_{15}{}^{79}BrN$ $[M+H]^+$ requires 288.0382, found 288.0382 (0.0 ppm)

(±)-8-(Methylthio)-4-phenyl-1,2,3,4-tetrahydroquinoline (4d)

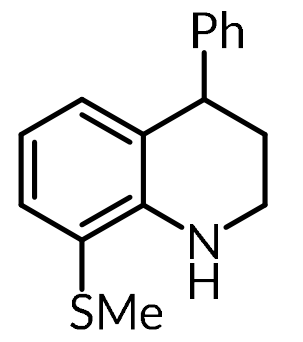

Following general procedure **F**, [Co-1] (1.2 mg, 0.002 mmol, 1.0 mol%), PXX (1.1 mg, 0.004 mmol, 2.0 mol%), Lut·HOTf (5.1 mg, 0.02 mmol, 10 mol%), **3d** (51.1 mg, 0.20 mmol), and PhCl (1.0 mL) for 24 h at 450 nm irradiation gave a ^{1}H-NMR yield of 90%. Purification by column chromatography (hexane/CH_2Cl_2, 90:10 → 85:15 → 80:20) gave the title compound **4d** (43.9 mg, 86%) as a beige solid.

mp 92 – 94 °C

ν_{max} (film) / cm^{-1}: 3374 (N–H), 2920, 2359, 1589, 1491, 1456, 1352, 1290, 741, 702

^1H NMR (500 MHz, $CDCl_3$) δ_H: 7.32 (2H, t, *J* 7.5), 7.28 – 7.21 (2H, m), 7.15 (2H, d, *J* 7.7), 6.73 (1H, d, *J* 7.5), 6.53 (1H, t, *J* 7.6), 5.09 (1H, br s), 4.18 (1H, t, *J* 5.9), 3.47 – 3.37 (1H, m), 3.36 – 3.29 (1H, m), 2.39 (3H, s), 2.28 – 2.17 (1H, m), 2.13 – 2.04 (1H, m)

$^{13}C\{^1H\}$ NMR (126 MHz, $CDCl_3$) δ_C: 146.5, 145.0, 131.8, 130.3, 128.7, 128.4, 126.3, 123.3, 118.7, 116.3, 43.2, 39.2, 30.7, 17.9

HRMS: (ESI^+) $C_{16}H_{18}NS$ $[M+H]^+$ requires 256.1154, found 256.1154 (0.0 ppm)

(±)-8-Methoxy-4-phenyl-1,2,3,4-tetrahydroquinoline (4e)

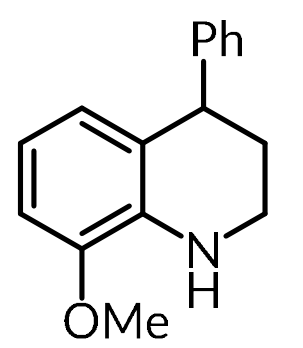

Following general procedure **F**, [Co-1] (1.2 mg, 0.002 mmol, 1.0 mol%), PXX (1.1 mg, 0.004 mmol, 2.0 mol%), Lut·HOTf (5.1 mg, 0.02 mmol, 10 mol%), **3e** (47.9 mg, 0.20 mmol), and PhCl (1.0 mL) for 24 h at 450 nm irradiation gave a ^{1}H-NMR yield of 74%. Purification by column

chromatography (hexane/CH_2Cl_2, 90:10 then hexane/EtOAc, 98:2 → 96:4) gave the title compound **4e** (33.0 mg, 69%) as an orange solid.

mp 54 – 56 °C

ν_{max} (film) / cm^{-1}: 3421 (N–H), 2933, 1585, 1493, 1450, 1333, 1246, 1105, 1055, 1018, 765, 731, 700

^{1}H NMR (500 MHz, $CDCl_3$) δ_H: 7.34 – 7.28 (2H, m), 7.25 – 7.19 (1H, m), 7.18 – 7.13 (2H, m), 6.68 (1H, d, *J* 8.1), 6.55 (1H, t, *J* 7.8), 6.45 (1H, d, *J* 7.8), 4.41 (1H, br s), 4.20 (1H, t, *J* 6.0), 3.89 (3H, s), 3.38 – 3.33 (1H, m), 3.32 – 3.26 (1H, m), 2.30 – 2.20 (1H, m), 2.14 – 2.04 (1H, m)

^{13}C{^{1}H} NMR (126 MHz, $CDCl_3$) δ_C: 146.9, 146.3, 135.0, 128.8, 128.3, 126.2, 123.3, 122.6, 115.7, 107.7, 55.5, 42.7, 38.7, 31.2

HRMS: (ESI$^+$) $C_{16}H_{18}NO$ [M+H]$^+$ requires 240.1383, found 240.1381 (-0.8 ppm)

(±)-6-Methoxy-8-methyl-4-phenyl-1,2,3,4-tetrahydroquinoline (4f)

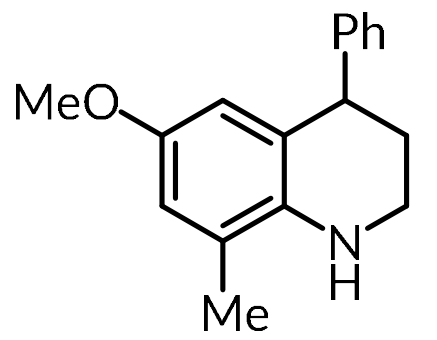

Following general procedure **F**, [Co-1] (1.2 mg, 0.002 mmol, 1.0 mol%), PXX (1.1 mg, 0.004 mmol, 2.0 mol%), Lut·HOTf (5.1 mg, 0.02 mmol, 10 mol%), **3f** (50.7 mg, 0.20 mmol), and PhCl (1.0 mL) for 24 h at 450 nm irradiation gave a ^{1}H-NMR yield of 87%. Purification by column chromatography (hexane/CH_2Cl_2, 95:5 → 90:10 then hexane/EtOAc, 97:3) gave the title compound **4f** (37.4 mg, 74%) as a yellow oil.

ν_{max} (film) / cm^{-1}: 3393 (N–H), 2922, 2357, 1489, 1464, 1252, 1148, 1051, 849, 700

^{1}H NMR (500 MHz, $CDCl_3$) δ_H: 7.33 – 7.27 (2H, m), 7.23 – 7.19 (1H, m), 7.17 – 7.12 (2H, m), 6.60 (1H, d, *J* 2.9), 6.27 (1H, d, *J* 2.9), 4.17 (1H, t, *J* 6.1), 3.63 (3H, s), 3.50 (1H, br s), 3.40 – 3.20 (2H, m), 2.27 – 2.19 (1H, m), 2.16 (3H, s), 2.09 – 2.02 (1H, m)

^{13}C{^{1}H} NMR (126 MHz, $CDCl_3$) δ_C: 151.3, 146.9, 137.4, 128.9, 128.4, 126.2, 124.2, 123.2, 115.4, 113.4, 55.8, 43.5, 39.7, 31.6, 17.8

HRMS: (ESI$^+$) $C_{17}H_{19}NONa$ [M+Na]$^+$ requires 276.1359, found 276.1356 (-1.1 ppm)

(±)-5,7-Dimethoxy-4-phenyl-1,2,3,4-tetrahydroquinoline (4g)

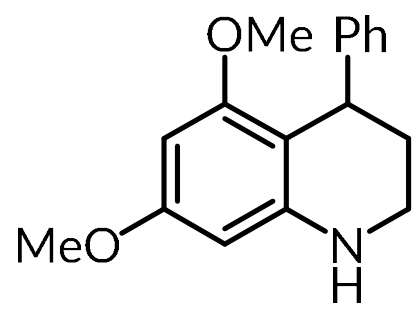

Following general procedure **F**, [Co-1] (1.2 mg, 0.002 mmol, 1.0 mol%), PXX (1.1 mg, 0.004 mmol, 2.0 mol%), Lut·HOTf (5.1 mg, 0.02 mmol, 10 mol%), **3g** (53.9 mg, 0.20 mmol), and PhCl (1.0 mL) for 24 h at 450 nm irradiation gave a ^{1}H-NMR yield of 87%. Purification by column chromatography (hexane/CH_2Cl_2, 95:5 → 80:20 → 50:50) gave the title compound **4g** (39.9 mg, 74%) as a brown solid.

mp 75 – 77 °C

ν_{max} (film) / cm^{-1}: 3410 (N–H), 2934, 2837, 1601, 1201, 1134, 908, 727, 700

^{1}H NMR (500 MHz, $CDCl_3$) δ_H: 7.28 – 7.22 (2H, m), 7.18 – 7.13 (1H, m), 7.12 – 7.06 (2H, m), 5.86 (1H, d, *J* 2.3), 5.80 (1H, d, *J* 2.4), 4.33 (1H, d, *J* 3.3), 3.97 (1H, br s), 3.78 (3H, s), 3.58 (3H, s), 3.16 – 2.98 (2H, m), 2.18 – 2.08 (1H, m), 1.93 (1H, dq, *J* 12.9, 2.7)

^{13}C{^{1}H} NMR (126 MHz, $CDCl_3$) δ_C: 160.1, 159.2, 147.5, 146.2, 128.1, 128.0, 125.6, 103.4, 91.3, 88.2, 55.5, 55.2, 36.9, 35.6, 30.1

HRMS: (ESI^{+}) $C_{17}H_{20}NO_2$ $[M+H]^{+}$ requires 270.1489, found 270.1488 (-0.4 ppm)

(±)-4-(Furan-2-yl)-1,2,3,4-tetrahydroquinoline (4h)

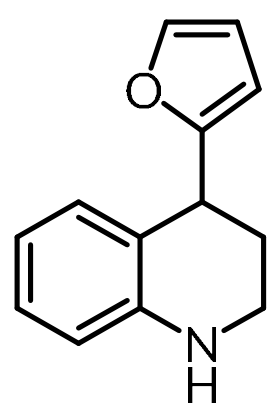

Following general procedure **F**, [Co-1] (1.2 mg, 0.002 mmol, 1.0 mol%), PXX (1.1 mg, 0.004 mmol, 2.0 mol%), Lut·HOTf (5.1 mg, 0.02 mmol, 10 mol%), **3h** (39.9 mg, 0.20 mmol), and PhCl (1.0 mL) for 24 h at 450 nm irradiation gave a ^{1}H-NMR yield of 60%. Purification by column chromatography (hexane/CH_2Cl_2, 90:10 then hexane/EtOAc, 98:2 → 95:5) gave the title compound **4h** (22.3 mg, 56%) as an orange oil.

ν_{max} (film) / cm^{-1}: 3410 (N–H), 2926, 1607, 1584, 1497, 1356, 1315, 1007, 745, 733

^{1}H NMR (500 MHz, CDCl$_3$) δ$_H$: 7.37 – 7.33 (1H, m), 7.03 (1H, td, *J* 7.7, 1.6), 6.96 (1H, d, *J* 7.6), 6.62 (1H, td, *J* 7.5, 1.1), 6.53 (1H, d, *J* 8.2), 6.28 (1H, dd, *J* 3.2, 1.9), 5.86 (1H, d, *J* 3.1), 4.18 (1H, t, *J* 5.1), 3.91 (1H, br s), 3.31 – 3.16 (2H, m), 2.31 – 2.23 (1H, m), 2.17 – 2.07 (1H, m)

^{13}C{^{1}H} NMR (126 MHz, CDCl$_3$) δ$_C$: 158.8, 144.7, 141.4, 130.4, 127.8, 120.7, 117.1, 114.6, 110.1, 107.2, 39.0, 36.3, 27.2

HRMS: (ESI$^+$) $C_{13}H_{10}NO$ [M-3H]$^+$ requires 196.0757, found 196.0757 (0.0 ppm)

2.5 Scope Limitation

Degradation of Starting Material

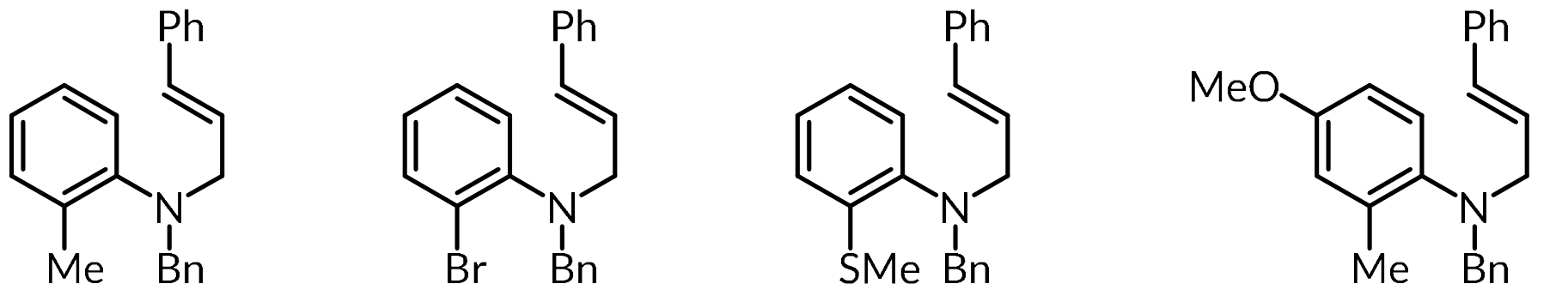

Return of Starting Material

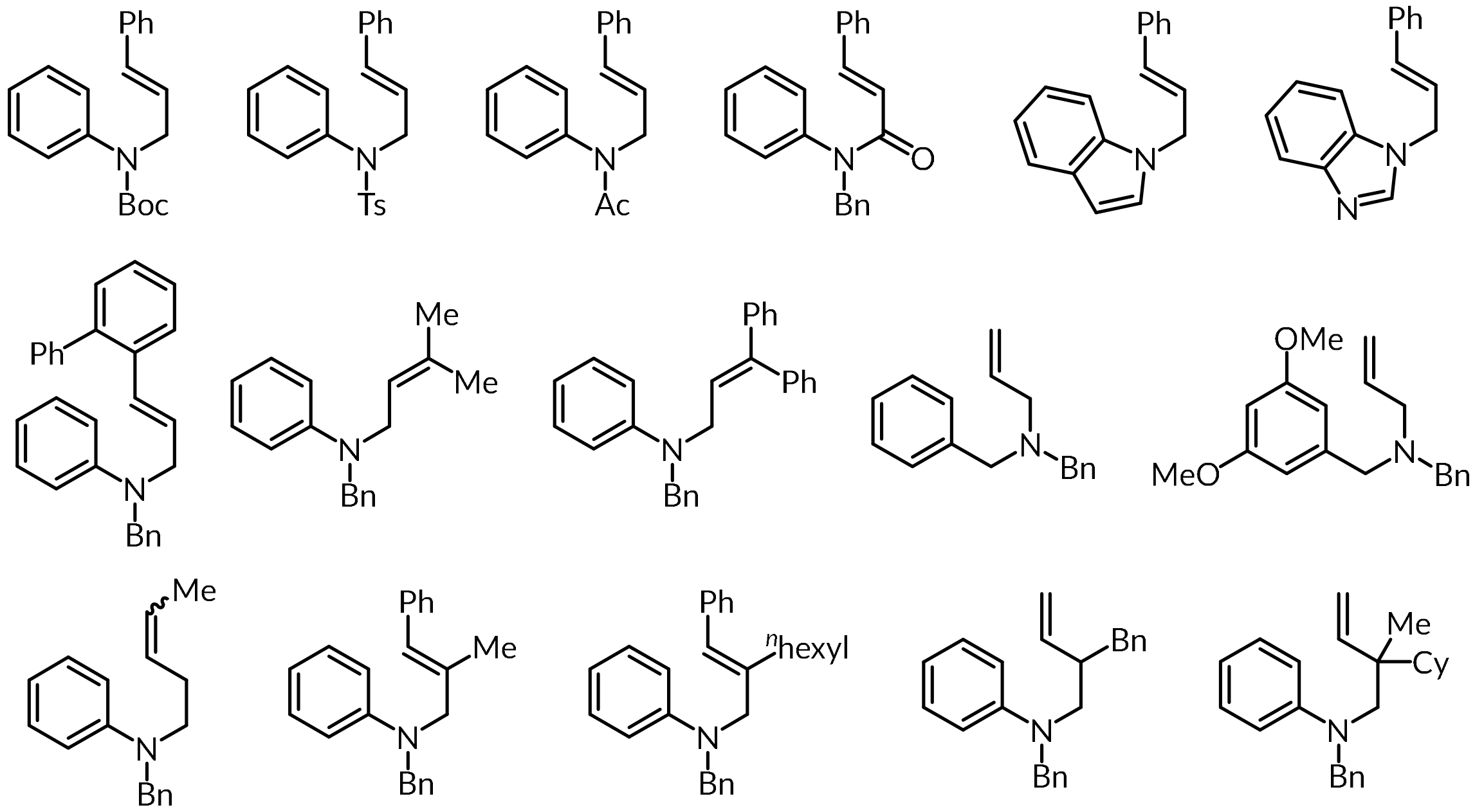

Produced a Complex Reaction Mixture

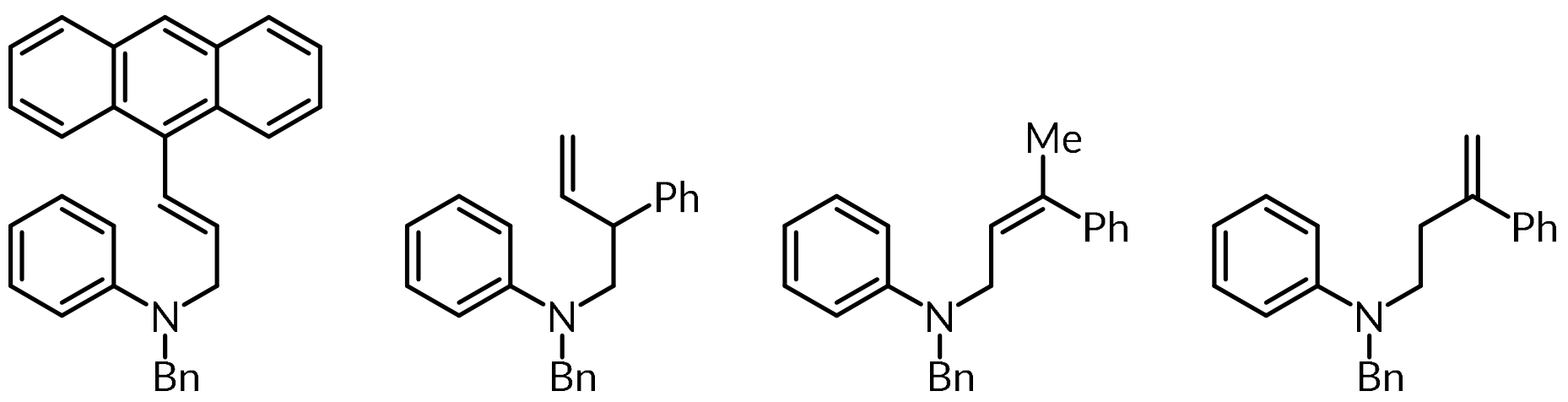

3. Photo-flow Experiments

3.1 General Procedures for Photoflow reactions

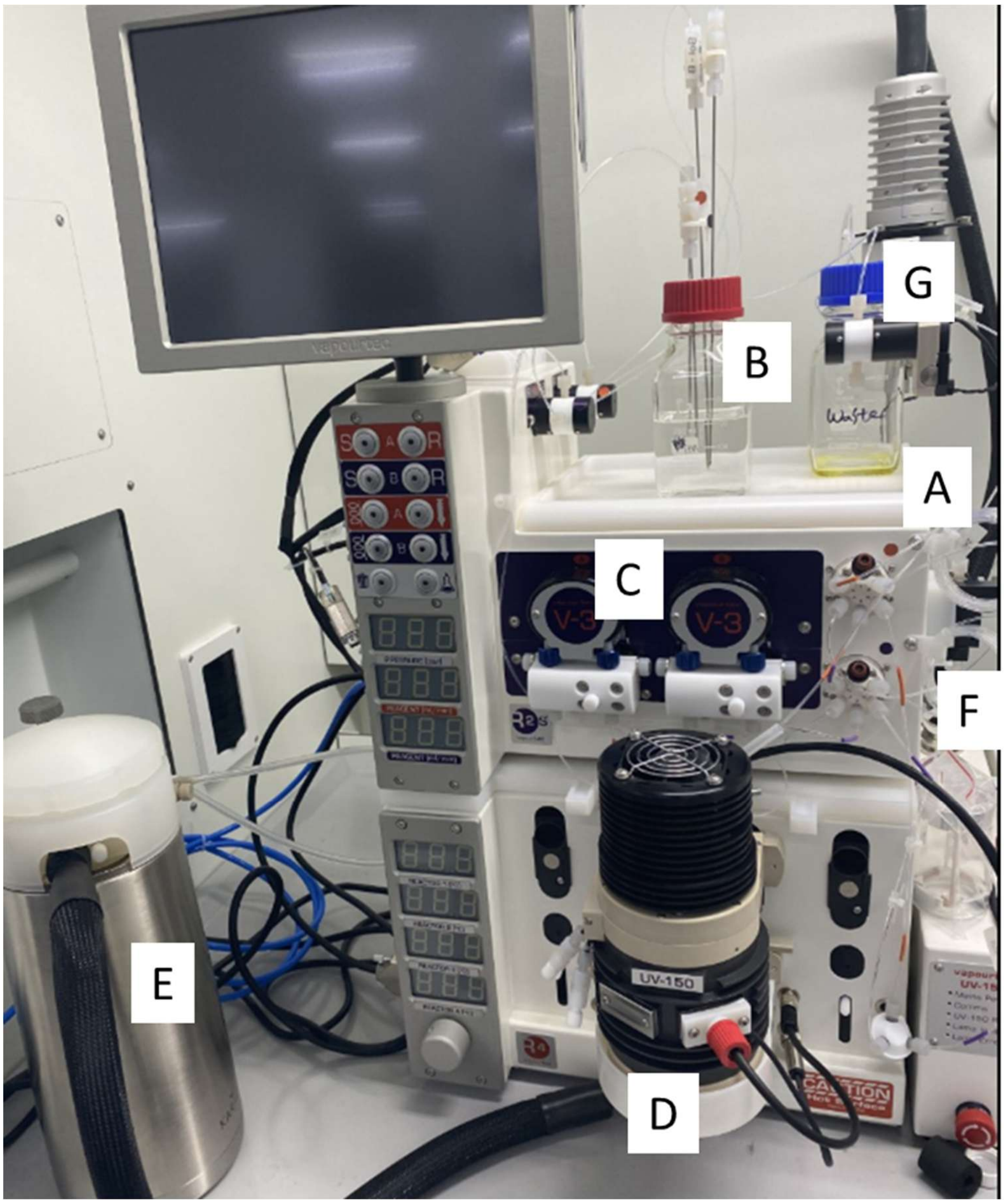

Figure S1: Photo of the flow system (Vapourtec® R2/R4 series connected to a UV-150 photoreactor). **A**: 2 mL loading coil; **B**: Inlet for reagent and solvent feed bottles; **C**: peristaltic pump; **D**: UV-150 photoreactor; **E**: cooling tank; **F**: backpressure regulator; **G**: collection port.

General Procedure for lab-scale continuous flow setup optimisation

Cobalt catalyst, photocatalyst, lutidinium triflate, and *N*-benzyl *N*-cinnamyl aniline were charged to a vial with a magnetic stirrer bar. The vial was capped and evacuated/backfilled with nitrogen three times using a needle (No stirring during degassing). The solvent was added via syringe, and the cap was sealed using parafilm. In cases using PhCl, solvent sparging was then performed directly in the reaction mixture over 10 min; for examples using CH_2Cl_2, sparging was performed prior to introduction to the vial, to avoid solvent losses through evaporation. The mixture was stirred to full dissolution. The Vapourtec® R2/R4 system was primed with

previously degassed solvent before and after addition of reaction mixture. The reaction mixture was loaded to the system via the reagent addition line. The mixture was pumped under N_2 with a peristaltic pump into the UV-150 photoreactor (10 mL reactor volume, thin-wall FEP tubing, 1 hour residence time maximum) and irradiated by 450 nm LED lamp (60 W output power, 24 W radiant power). The reactor temperature was kept at 30 °C (unless otherwise noted) and 1-4 bar pressure. The resulting mixture was collected as fractions of the end of reaction stream; to each fraction was added a known amount of 1,2-dibromoethane as internal standard and a sample of each fraction was diluted in $CDCl_3$ and subjected to ^1H qNMR analysis to obtain the end of reaction assay yield. The spectra were obtained using a Bruker 400 Ultrashield Spectrometer at 25 °C, with the acquisition parameters set to 8 scans and 40 s d1 delay.

3.2 Optimisation of Batch Conditions to Continuous Flow Setup

Continuous flow conditions were examined, with the optimised batch conditions as a starting point. The key modifications from batch to flow were replacing PhCl with CH_2Cl_2 as solvent (mostly due to solubility issues) and PXX with $Ir(ppy)_3$ as photocatalyst.

Table S3: Stability test in batch

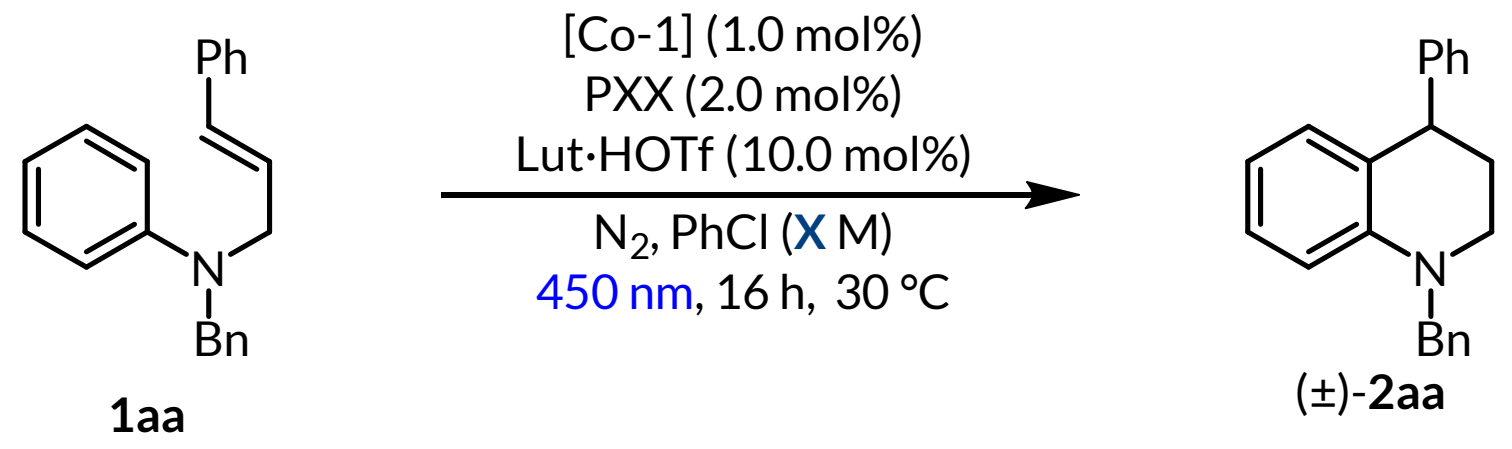

Entry	Concentration (mol/L)	Aging time (in the dark) at rt of reaction stream before irradiation	^{1}H qNMR yield of 2aa (%)
1[a]	0.4	*none*	95
2	0.1	*none*	90
3	0.4	1 h	94
4	0.4	4 h	92
5	0.4	24 h	>99

[a]Previously optimised batch conditions

Compared to the standard batch conditions (Table S3, entry 1), dilution (entry 2) did not impact the excellent yield of **2aa** significantly. The pre-irradiation reaction stream showed good stability over time (entries 3-5), with no impact on yield even after significant aging. This is a major criterion for transferring reactions from batch to flow, especially on larger scale, as the stream will be stable while waiting to be pumped into the reactor.

Table S4: PhCl as solvent & PXX as photocatalyst

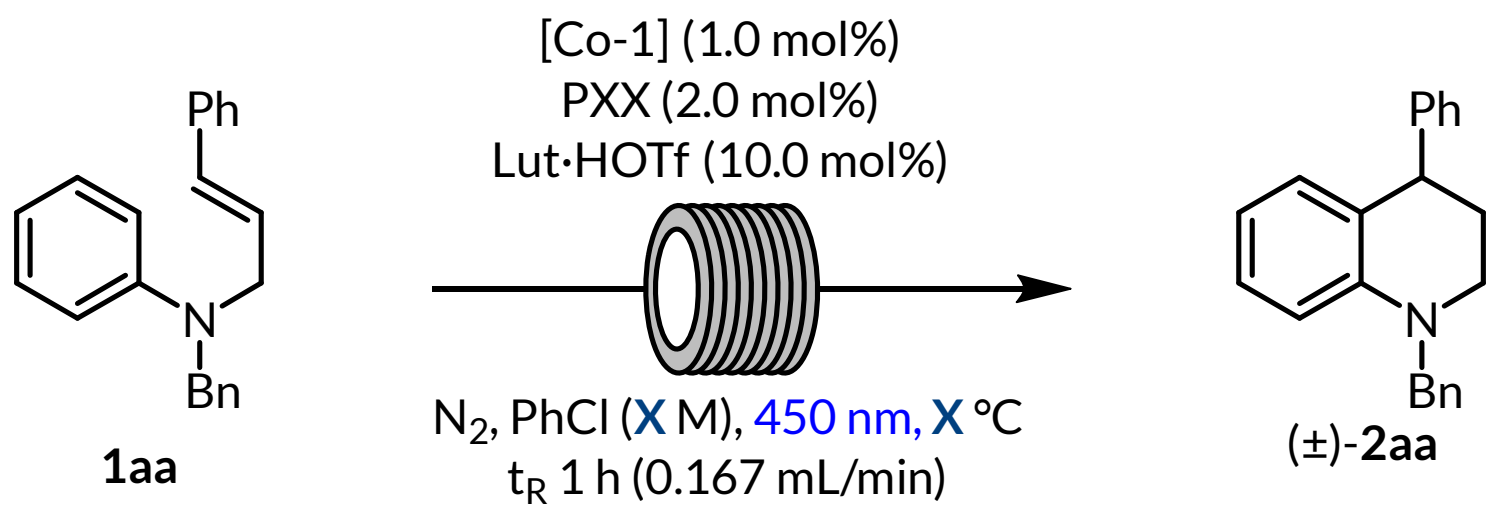

Entry	Temperature (°C)	Concentration (mol/L)	^{1}H qNMR yield (%)		
			2aa	1aa	Mass balance
1[a]	30	0.4	18	71	89
2[a]	45	0.4	20	68	88
3[b]	30	1.0	9	77	86
4	30	0.1	12	67	79
5[c]	30	0.4	15	77	92

[a]Lut·HOTf not fully dissolved. [b]Significant amounts of all reagents not dissolved. [c]Reaction stream heated to 30 °C for 1 h before introduction into the photoflow reactor.

The first attempt in photoflow (Table S4, entry 1) was based on the batch conditions but gave poor yield of **2aa** (18%). The mass balance (**1aa** + **2aa**) was high, indicating no degradation of **1aa**, but rather a slow or stalling reaction. As the residence time was already at its maximum (1 h), the temperature in the photoflow reactor was increased in the hope of accelerating the reaction (entry 2), however this did not improve the yield.

Concentrating the reaction mixture to 1.0 M (entry 3) led to even lower yields, likely due to the significant amounts of undissolved material that did not get introduced into the photoflow reactor. Upon inspection, it was observed that the Lut·HOTf had not fully dissolved in entries 1-2 either, remaining at the bottom of the vial and not introduced into the photoreactor. This had remained unnoticed until then due to the dark black colour of the reaction mixture and the translucent nature of the lutidinium crystals.

Dilution to 0.1 M led to a fully homogenous reaction stream (entry 4) but did not improve the yield. Alternatively, warming the reaction mixture slightly prior to irradiation also did not fully dissolve the Lut·HOTf and led to similar yields as previously observed (entry 5).

The Lut·HOTf salt showed better solubility in CH_2Cl_2, and all further optimisation was performed with CH_2Cl_2 as the solvent of choice.

Table S5: Equivalents & loadings

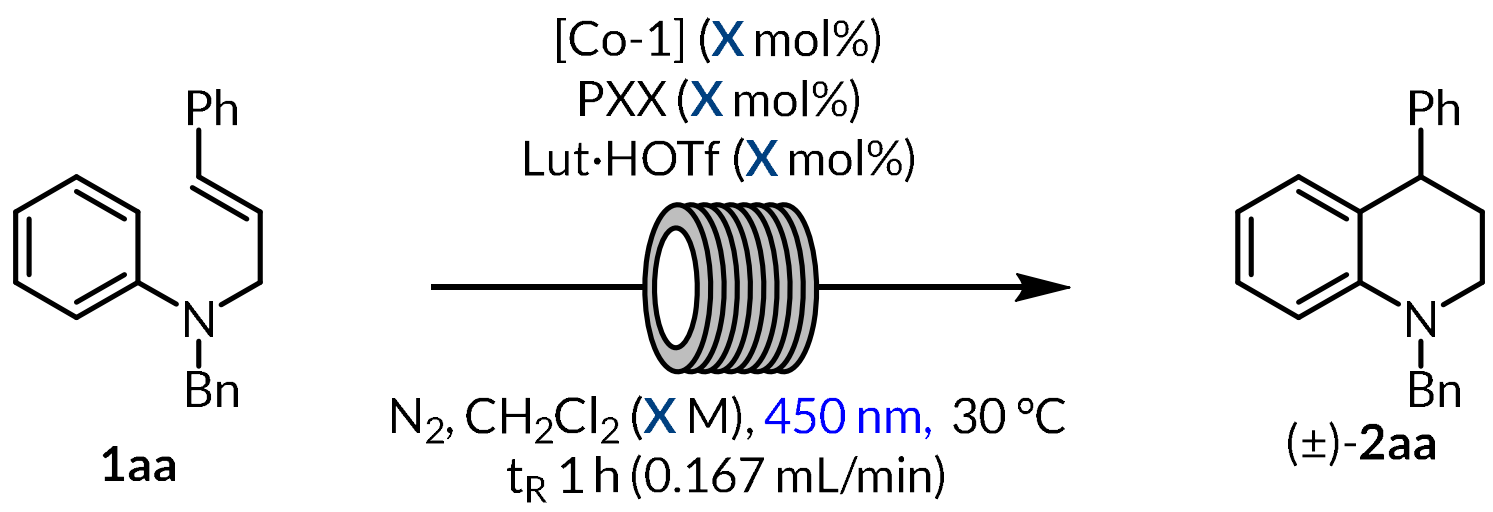

Entry	Concentration (mol/L)	Equivalents (mol%)			¹H qNMR yield (%)		
		[Co]	PXX	Lut·HOTf	2aa	1aa	Mass balance
1	0.4	1	2	10	25	57	82
2 [a]	0.4	5	10	50	21	69	90
3	0.2	5	10	50	54	41	95
4 [b]	0.2	5	10	50	0.9	90	91
5	0.2	5	10	100	45	40	85
6	0.2	10	20	100	58	38	96

[a]PXX not fully dissolved. [b]Water (1 equiv.) added

Switching to CH_2Cl_2 (with no other changes compared to the batch conditions) led to full dissolution of all reagents yet only gave 25% yield of **2aa** (Table S5, entry 1). However, mass balance was still very high, indicating no degradation of **1aa** occurring. Increasing all loadings ([Co], PXX, and Lut·HOTf loadings) 5-fold did not improve yield (entry 2), due to most of the PXX not being solubilised, leaving it at the bottom of the vial when the stream was introduced into the photoreactor. Repeating the reaction in more dilute conditions (0.2 M instead of 0.4 M) dissolved the PXX and gave a more promising yield of 54% (entry 3). Adding 1 equivalent of water (entry 4) led to almost full shutdown of the reactivity. As 50 mol% Lut·HOTf gave 54% yield, the next step was to try using the salt stoichiometrically (entry 5), though this led to a slight decrease in yield. A 10-fold increase of all loadings compared to the batch conditions lead to a slightly higher yield still, though not significant enough to justify such an increase in loadings. In summary, increasing catalyst loadings led to increasing yields, albeit with diminishing returns.

Table S6: Miscellaneous

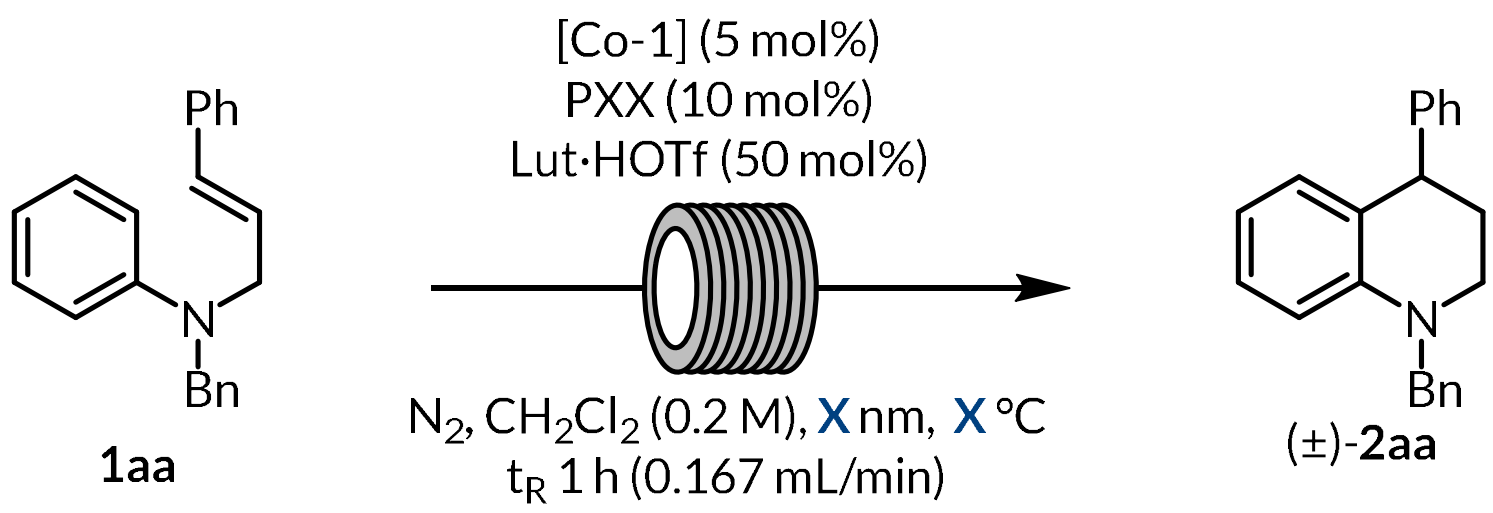

Entry	Wavelength (nm)	Temperature (°C)	^{1}H qNMR yield (%)		
			2aa	1aa	Mass balance
1	450	45	41	36	77
2	420	30	16	23	39
3[a]	450	30	51	26	77

[a]The end of reaction stream was resubmitted to the photoflow reactor for a second loop

Repeating the best conditions so far (Table S5, entry 3) but at higher temperature led to a slight decrease in yield of **2aa** (Table S6, entry 1). Increasing the energy provided to the system by switching the light wavelength from 450 nm to 420 nm led to a drop in yield and significant degradation (entry 2). Resubmitting the end stream back into the photoflow reactor, performing an overall 2 h long double loop, did not improve the yield. It appears the reaction stalls part way through, likely due to one of the catalytic species degrading.

Table S7: CH_2Cl_2 as solvent & $Ir(ppy)_3$ as photocatalyst

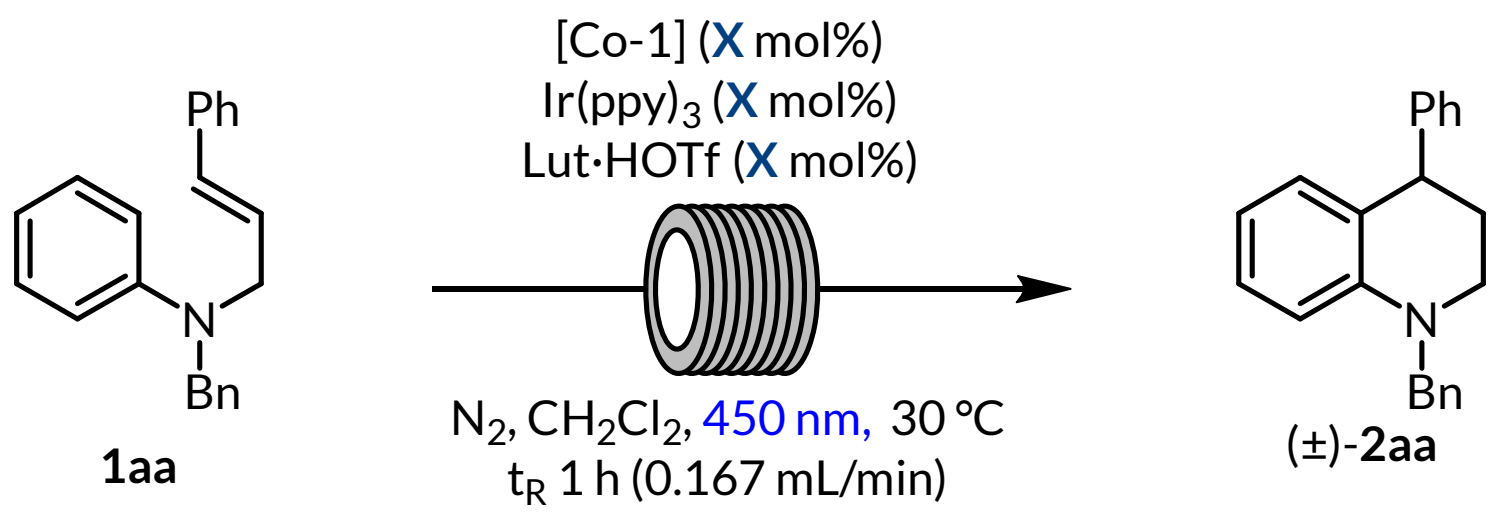

Entry	Equivalents (mol%)			^{1}H qNMR yield (%)		
	[Co]	$Ir(ppy)_3$	Lut·HOTf	2aa	1aa	Mass balance
1	5	5	50	89	-	89
2	1	2	10	80	-	80

Switching from PXX to $Ir(ppy)_3$ as photocatalyst, while maintaining loadings as previously optimised (except for the $Ir(ppy)_3$ levels which were halved compared to PXX) gave a significant boost to the yield of **2aa**, increasing from the previous maximum of 54% to 89% (Table S7, entry 1) and leading to complete consumption of the **1aa**. This appears to show the PXX as the main

reason for the lower yields in flow so far. Returning to the initial low loadings optimised for the batch conditions (entry 2) led to similarly high yield and will significantly decrease costs on a larger scale.

Table S8: Concentration & residence time t_R

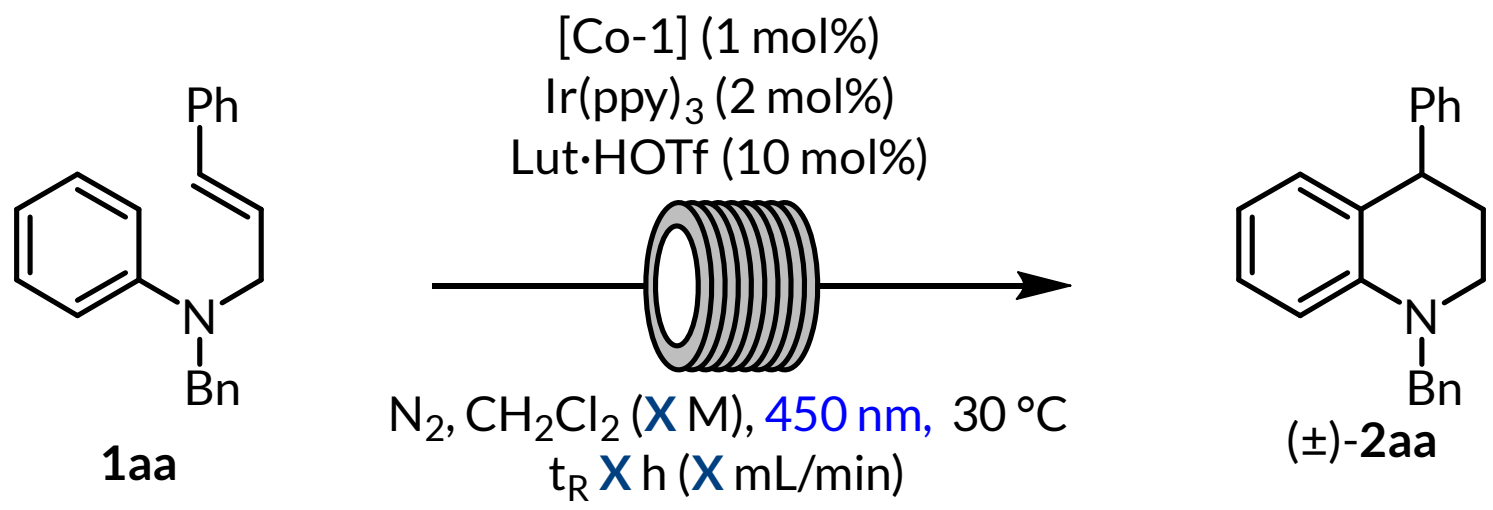

Entry	t_R (flow speed)	Concentration (mol/L)	^{1}H qNMR yield (%)		
			2aa	1aa	Mass balance
1	1 h (0.167 mL/min)	0.2	80	-	80
2	30 min (0.33 mL/min)	0.2	81	-	81
3	10 min (1.00 mL/min)	0.2	57	39	96
4[a]	30 min (0.33 mL/min)	0.2	61	3.2	64
5	30 min (0.33 mL/min)	0.3	83	1.7	85
6	30 min (0.33 mL/min)	0.4	78	3.6	82

[a]1 mol% of Ir(ppy)3 used (instead of 2 mol%).

With a high yield in hand (Table S8, entry 1), the reaction was then optimised for efficiency. Firstly, halving the residence time (doubling the flow speed) did not impact the reaction (entry 2) and gave similarly high yield of **2aa**. However, lowering it further (entry 3) did not allow enough time for the reaction to reach completion. Lowering the $Ir(ppy)_3$ loading down to 1 mol% also led to lower yield and lower mass balance overall. Finally, increasing the concentration (entries 5-6) still gave high yields. A higher concentration is a desirable trait within a flow process, as this increases its productivity. However, high concentrations also led to higher levels of leftover **1aa**, which, as an isomer of the product, has been difficult to remove through purification. Due to this, the final best conditions, to be performed on a 1-gram scale, were deemed to be entry 2.

3.3 Optimised procedure for 1 gram lab-scale continuous photoflow setup

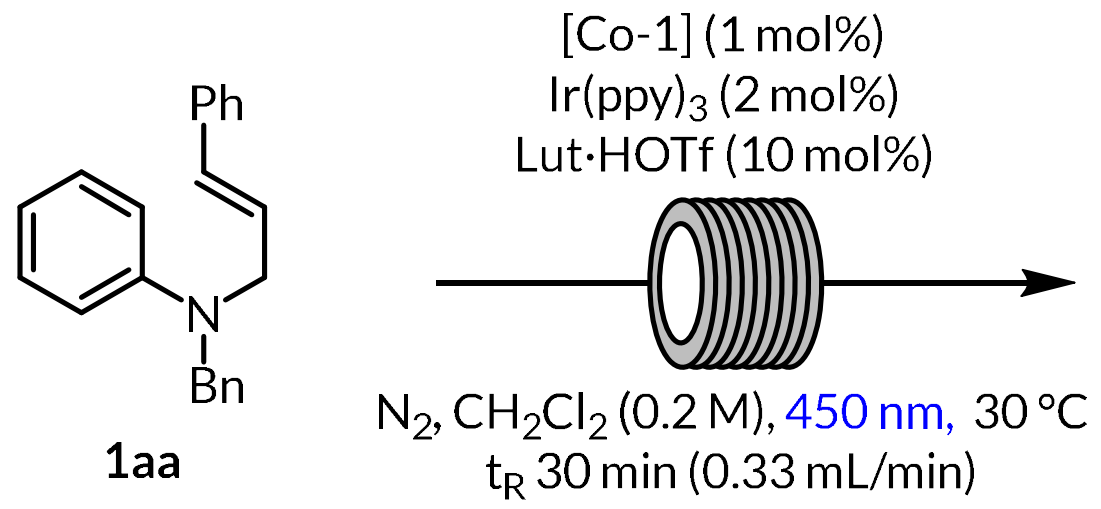

A 30 ml glass vial was charged with *N*-benzyl *N*-cinnamyl aniline **1aa** (1.04 g, 96 wt%, 3.34 mmol, 1.0 equiv.), [Co-1] (19.96 mg, 0.0334 mmol, 0.01 equiv.), Ir(ppy)$_3$ (43.74 mg, 0.0668 mmol, 0.02 equiv.) and Lut·HOTf (85.91 mg, 0.3334 mmol, 0.10 equiv.). The vial was capped and sealed with parafilm, then evacuated/backfilled with N_2 three times using a needle. CH_2Cl_2 (16 mL, 0.2 M), which had been previously degassed for 1 hour, was added via a syringe.

The Vapourtec R2/R4 system and UV-150 photoreactor were primed with degassed CH_2Cl_2, which was also used as the carrier solvent throughout the reaction. The reaction mixture was loaded via the reagent feed line with flow rate at 0.333 mL/min and irradiated by 450 nm LED lamp. The reactor temperature was kept at 30 °C and 1-2 bar pressure. After approx. 78 min of continuous flow, the output stream was collected in 7 fractions.

Each fraction was added 1,2-dibromoethane (78.5 mg) as internal standard and diluted with $CDCl_3$. The assay yield of the product was determined by quantitative ^{1}H NMR analysis. The product was obtained with a 90% yield of **2aa**, yielding 896 mg over 78 min, or approximately 11.5 mg/min (0.69 g/h), with 0.9% leftover **1aa**.

3.4 Quantitative ^{1}H NMR analysis and Assay Yield calculation

Chemical shifts are reported in parts per million (ppm) and referenced against the solvent signal of CH_2Cl_2 (5.30 ppm in $CDCl_3$), since the residual solvent peak of $CDCl_3$ was obscured by other signals from the reaction mixture. The peaks chosen for analysis of the product (**2aa**) and starting material (**1aa**) had the chemical shift of 2.28 ppm and 6.30 ppm respectively. The chemical shift of the internal standard peak was 3.66 ppm.

The peaks were integrated (I_{int} and $I_{fraction}$) and used, along with their corresponding numbers of nuclei (N_{int} and $N_{fraction}$), to calculate the masses of the product (**2aa**) and starting material (**1aa**) in each fraction ($m_{fraction\ n}$, where n is the number of the fraction). The qNMR assay yield was determined by adding up the masses in each fraction (**Error! Reference source not found.**S9) and calculating it against the theoretical mass output of the product as follows:

$$m_{fraction\ n} = \frac{I_{fraction\ n}}{I_{int}} \times \frac{N_{int}}{N_{fraction}} \times \frac{M_{fraction}}{M_{int}} \times m_{int} \times P_{int}$$

$$Assay\ Yield = \frac{\sum_1^n m_{fraction\ n}}{m_{theory}} \times 100\%$$

Table S9: Product and starting material assay calculation for 1 gram scale experiment.

Fraction	Product (2aa)		Starting material (1aa)	
	$I_{fraction}$	$m_{fraction}$ (mg)	$I_{fraction}$	$m_{fraction}$ (mg)
1	6.84	87.84	0.10	1.28
2	13.79	177.11	0.11	1.41
3	12.18	156.43	0.09	1.21
4	9.05	116.23	0.04	0.47
5	10.57	135.75	0.11	1.41
6	8.00	102.74	0.06	0.72
7	9.32	119.70	0.19	2.44
Total		895.80		8.94
**Assay yield (%)**	**89.58**		**0.894**	

Overall, 90% assay yield of **2aa** was achieved, with 0.9% starting material **1aa** remaining. The full calculation for the masses in each fraction is shown below, along with a representative qNMR spectrum of one of the fractions (Figure 2).

qNMR assay calculation for the 1-gram demonstration

Table S10: Assay yield calculation information for 1 gram scale experiment.

**1 gram experiment – qNMR assay**	
Internal standard peak integration I_{int}	40.0
Internal standard peak nuclei N_{int}	4
Target product and starting material peak nuclei $N_{fraction}$	1
Product and starting material molecular weight $M_{fraction}$ (g/mol)	299.42
Internal standard molecular weight M_{int}	187.86
Internal standard weight m_{int} (mg)	80.66
Internal standard purity P_{int} (%)	99.9
Theoretical output m_{theory} (mg)	1000

$$m_{fraction\ 1} = \frac{6.84}{40.0} \times \frac{4}{1} \times \frac{299.42}{187.86} \times 80.66 \times 0.999 = 87.84\ mg$$

$$m_{fraction\ 2} = \frac{13.79}{40.0} \times \frac{4}{1} \times \frac{299.42}{187.86} \times 80.66 \times 0.999 = 177.11\ mg$$

$$m_{fraction\ 3} = \frac{12.18}{40.0} \times \frac{4}{1} \times \frac{299.42}{187.86} \times 80.66 \times 0.999 = 156.43\ mg$$

$$m_{fraction\ 4} = \frac{9.05}{40.0} \times \frac{4}{1} \times \frac{299.42}{187.86} \times 80.66 \times 0.999 = 116.23\ mg$$

$$m_{fraction\ 5} = \frac{10.57}{40.0} \times \frac{4}{1} \times \frac{299.42}{187.86} \times 80.66 \times 0.999 = 135.75\ mg$$

$$m_{fraction\ 6} = \frac{8.00}{40.0} \times \frac{4}{1} \times \frac{299.42}{187.86} \times 80.66 \times 0.999 = 102.74\ mg$$

$$m_{fraction\ 7} = \frac{9.32}{40.0} \times \frac{4}{1} \times \frac{299.42}{187.86} \times 80.66 \times 0.999 = 119.70\ mg$$

$$Assay\ Yield = \frac{87.84 + 177.11 + 156.43 + 116.23 + 135.75 + 102.74 + 119.70}{1000} \times 100\%$$
$$= 89.58\%$$

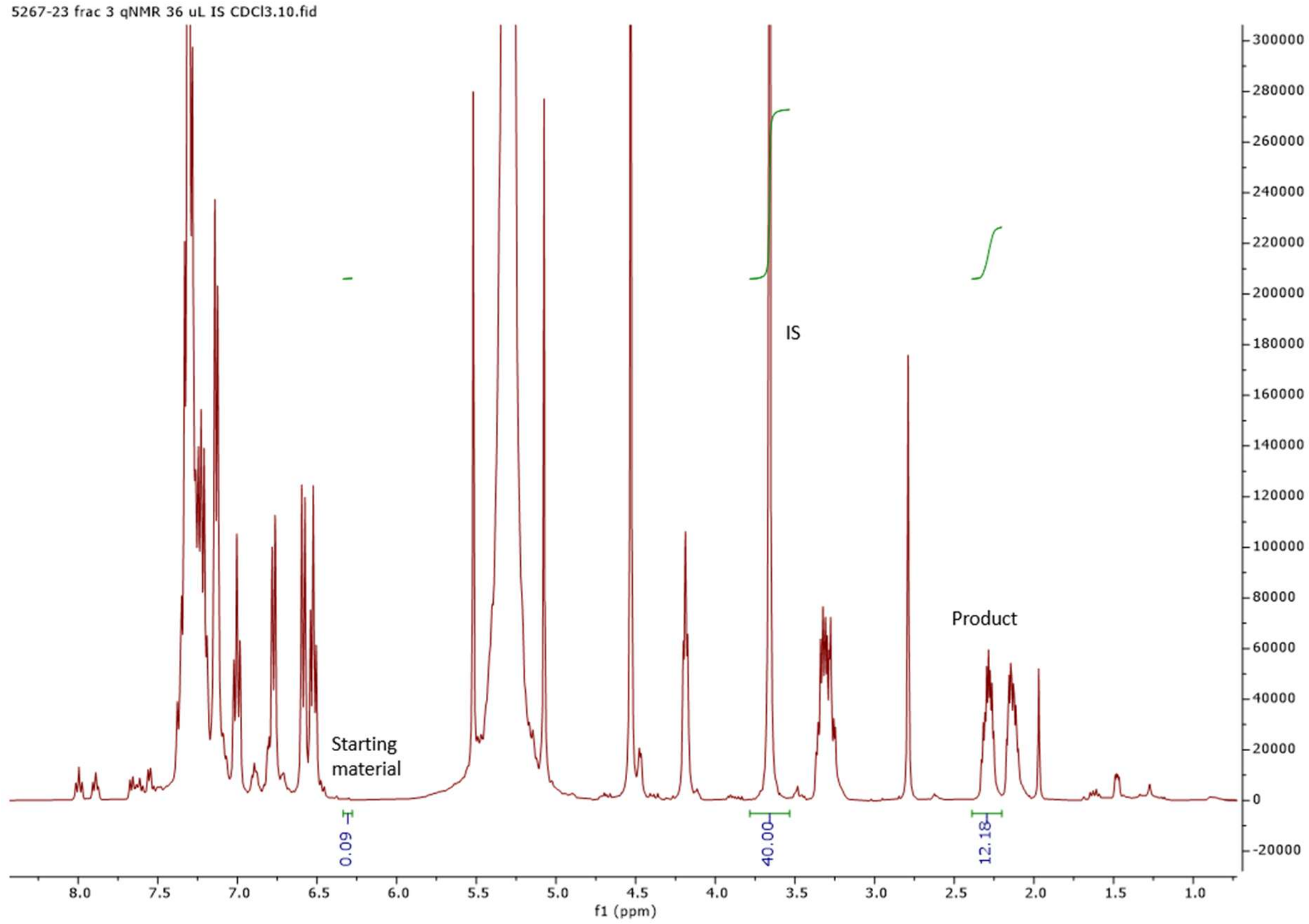

Figure S2: ^{1}H qNMR spectrum of a sample of fraction 3 of the 1-gram photoflow experiment.

4. Mechanistic Investigation

4.1 Literature Condition Comparisons

4.1.1 Radical Cyclisation Experiments

(±)-Ethyl-(*E*)-4-(4-phenyl-3,4-dihydroquinolin-1(2*H*)-yl)but-2-enoate (6a)

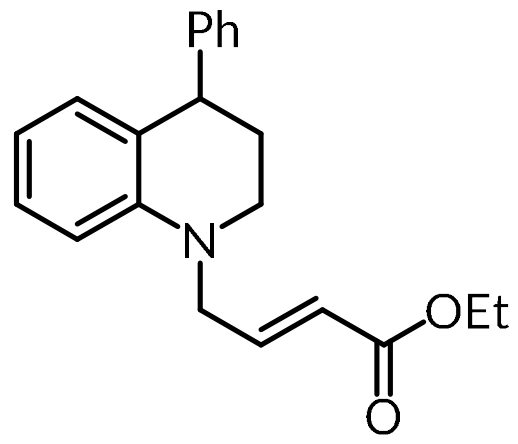

Following modified general procedure **E**, [Co-1] (2.4 mg, 0.004 mmol, 2.0 mol%), PXX (2.3 mg, 0.008 mmol, 4.0 mol%), Lut·HOTf (5.1 mg, 0.02 mmol, 10 mol%), **5** (64.3 mg, 0.20 mmol), and PhCl (1.0 mL) for 16 h at 450 nm irradiation gave a ^{1}H-NMR yield of 85%. Purification by column chromatography (hexane/EtOAc, 98:2 → 95:5 → 05:10 → 85:15) gave the title compound **6a** (54.1 mg, 84%) as a brown oil.

ν_{max} (film) / cm^{-1}: 2924, 1717 (C=O), 1600, 1502, 1273, 1173, 1036, 746

^{1}H NMR (500 MHz, CDCl$_3$) δ_H: 7.35 – 7.28 (2H, m), 7.24 – 7.20 (1H, m), 7.14 – 7.06 (3H, m), 7.00 (1H, dt, *J* 15.7, 4.4), 6.79 (1H, d, *J* 7.4), 6.59 (1H, t, *J* 7.4), 6.54 (1H, d, *J* 8.3), 6.01 (1H, dt, *J* 15.7, 2.0), 4.23 – 4.15 (3H, m), 4.07 (2H, d, *J* 2.6), 3.31 – 3.17 (2H, m), 2.34 – 2.24 (1H, m), 2.18 – 2.08 (1H, m), 1.30 (3H, t, *J* 7.1)

^{13}C{^{1}H} NMR (126 MHz, CDCl$_3$) δ_C: 166.4, 146.1, 145.0, 144.2, 130.3, 128.7, 128.5, 127.9, 126.3, 124.4, 122.0, 116.5, 111.1, 60.5, 52.7, 46.6, 43.4, 30.7, 14.4

HRMS: (ESI$^+$) $C_{21}H_{23}NO_2Na$ [M+Na]$^+$ requires 344.1621, found 344.1619 (-0.6 ppm)

(±)-Ethyl 2-(1,4-diphenylpiperidin-3-yl)acetate (6b)

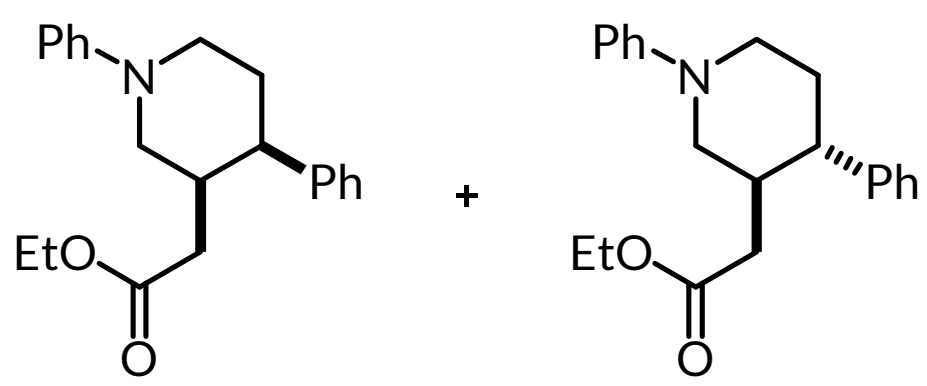

(±)-6b
(syn-major)

(±)-6b
(anti-minor)

Following hydroarylation procedure by Matsunaga and coworkers.[8] A solution of Ru(bpy)$_3$Cl$_2$ (2.6 mg, 2.0 mol%), [Co-1] (12.0 mg, 10 mol%), ascorbic acid (70.4 mg, 0.40 mmol), and **5** (64.3 mg, 0.20 mmol) in ^{t}BuOH:H$_2$O (3:1, 3.0 mL) was irradiated (2 x 450 nm lamps) for 16 hours. The

mixture was quenched with $NaHCO_3$, extracted with CH_2Cl_2, washed with brine, dried over Na_2SO_4 and concentrated under reduced pressure. Purification via column chromatography (hexane/Et_2O, 99:1 → 98:2 → 97:3 → 96:4 → 95:5 → 94:6) gave the title compounds *syn*-**6b** (25.5 mg, 39%) as a white solid and *anti*-**6b** (28.7 mg, 44%) as a colourless oil.

Data for major *syn*-**6b** diastereomer

mp 84 – 87 °C

ν_{max}(film) / cm^{-1}: 2928, 1728 (C=O), 1599, 1495, 1382, 1227, 1169, 1034, 926, 756, 696

^{1}H NMR (700 MHz, $CDCl_3$) δ_H: 7.36 (2H, t, *J* 7.6), 7.31 – 7.23 (5H, m), 6.96 (2H, d, *J* 8.0), 6.87 (1H, t, *J* 7.3), 4.06 (1H, dq, *J* 10.8, 7.1), 4.02 (1H, dq, *J* 10.8, 7.1), 3.87 (1H, dt, *J* 12.3, 2.4), 3.81 (1H, ddt, *J* 11.7, 4.6, 2.3), 3.05 (1H, dt, *J* 13.0, 3.6), 3.02 (1H, dt, *J* 12.3, 2.1), 2.82 (1H, td, *J* 11.9, 2.9), 2.74 (1H, dd, *J* 16.3, 10.9), 2.61 (1H, dt, *J* 11.1, 3.4), 2.15 (1H, qd, *J* 12.8, 4.4), 2.00 (1H, dt, *J* 16.1, 2.3), 1.89 (1H, dq, *J* 12.9, 3.1), 1.19 (3H, t, *J* 7.1)

^{13}C{^{1}H} NMR (126 MHz, $CDCl_3$) δ_C: 173.7, 152.5, 143.2, 129.2, 128.6, 127.5, 126.5, 119.7, 117.1, 60.3, 55.3, 49.9, 44.0, 37.4, 30.6, 25.2, 14.3

HRMS: (ESI$^+$) $C_{21}H_{25}NO_2Na$ [M+Na]$^+$ requires 346.1777, found 346.1775 (-0.6 ppm)

Data for minor *anti*-**6b** diastereomer

ν_{max}(film) / cm^{-1}: 2916, 1730, 1597, 1495, 1389, 1375, 1213, 1155, 1030, 756, 700

^{1}H NMR (500 MHz, $CDCl_3$) δ_H: 7.36 – 7.27 (4H, m), 7.27 – 7.20 (3H, m), 7.02 (2H, d, *J* 8.1), 6.87 (1H, t, *J* 7.3), 4.06 (2H, q, *J* 7.1), 3.98 (1H, d, *J* 11.2), 3.83 (1H, d, *J* 12.3), 2.87 (1H, td, *J* 12.2, 2.9), 2.58 (1H, t, *J* 11.5, 11.1), 2.50 (1H, qt, *J* 11.1, 10.6, 3.4), 2.39 (1H, td, *J* 11.5, 4.1), 2.25 (1H, dd, *J* 16.0, 3.5), 2.00 (1H, dd, *J* 16.0, 9.5), 1.98 (1H, qd, *J* 12.0, 4.1), 1.90 (1H, dq, *J* 13.4, 3.3), 1.22 (3H, t, *J* 7.1)

^{13}C{^{1}H} NMR (126 MHz, $CDCl_3$) δ_C: 172.5, 151.3, 143.8, 129.3, 128.8, 127.9, 126.8, 119.6, 116.7, 60.4, 55.6, 50.5, 48.8, 38.0, 36.8, 34.2, 14.3

HRMS: (ESI$^+$) $C_{21}H_{25}NO_2Na$ [M+Na]$^+$ requires 346.1777, found 346.1775 (-0.6 ppm)

4.1.2 Differing Arene Cyclisation Experiments

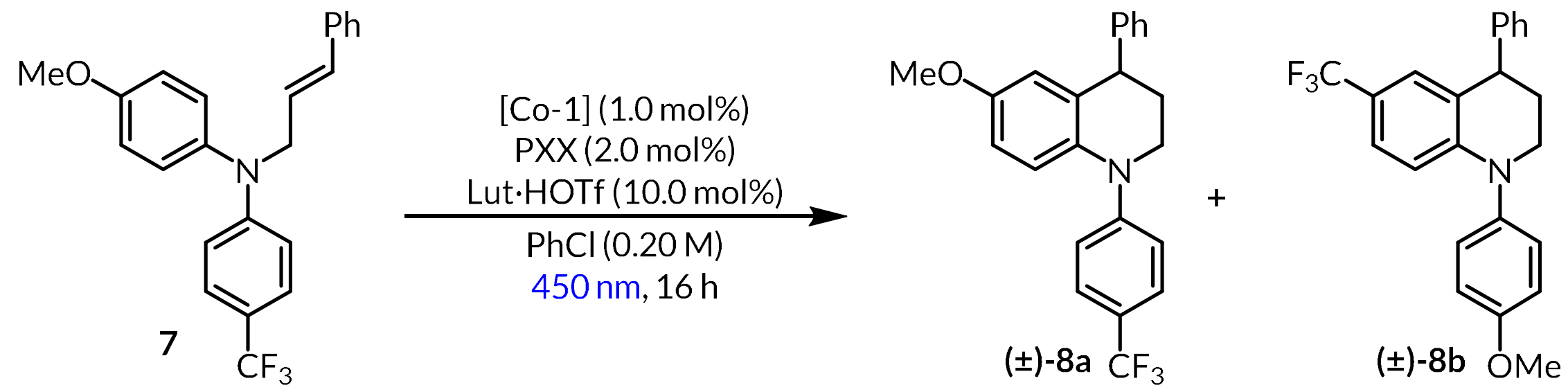

Following general procedure **E**, [Co-1] (1.2 mg, 0.002 mmol, 1.0 mol%), PXX (1.1 mg, 0.004 mmol, 2.0 mol%), Lut·HOTf (5.1 mg, 0.02 mmol, 10 mol%), **7** (76.7 mg, 0.20 mmol), and PhCl (1.0 mL) for 16 h at 450 nm irradiation. Purification by column chromatography (hexane/CH_2Cl_2, 95:5 → 90:10 → 98:20) gave **8a** (54.1 mg, 84%) as a brown oil.

(±)-6-Methoxy-4-phenyl-1-(4-(trifluoromethyl)phenyl)-1,2,3,4-tetrahydroquinoline (8a)

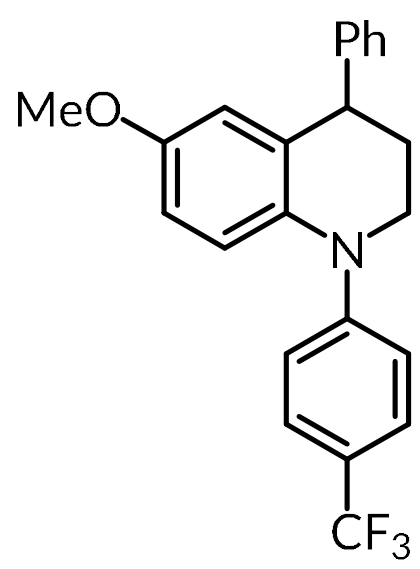

ν_{max}(film) / cm^{-1}: 2937, 1614, 1520, 1497, 1323, 1275, 1163, 1113, 1068

^{1}H NMR (500 MHz, $CDCl_3$) δ_H: 7.49 (2H, d, *J* 8.6), 7.34 – 7.28 (2H, m), 7.25 – 7.20 (3H, m), 7.19 – 7.15 (2H, m), 7.10 (1H, d, *J* 8.9), 6.69 (1H, dd, *J* 8.9, 3.0), 6.45 (1H, dd, *J* 2.9, 0.8), 4.12 (1H, dd, *J* 7.8, 5.3), 3.69 – 3.61 (5H, m), 2.37 – 2.24 (1H, m), 2.19 – 2.07 (1H, m)

^{13}C{^{1}H} NMR (126 MHz, $CDCl_3$) δ_C: 154.5, 151.8, 144.5, 135.8, 132.8, 128.7, 128.6, 126.7, 126.4 (q, *J* 3.7), 124.7 (q, *J* 271.0), 122.2 (q, *J* 32.5), 121.1, 119.4, 114.9, 113.0, 55.6, 47.6, 43.7, 32.0

^{19}F NMR (471 MHz, $CDCl_3$) δ_F: -61.6 (s)

HRMS: (ESI$^+$) $C_{23}H_{17}F_3NO$ [M-3H]$^+$ requires 380.1257, found 380.1251 (-1.6 ppm)

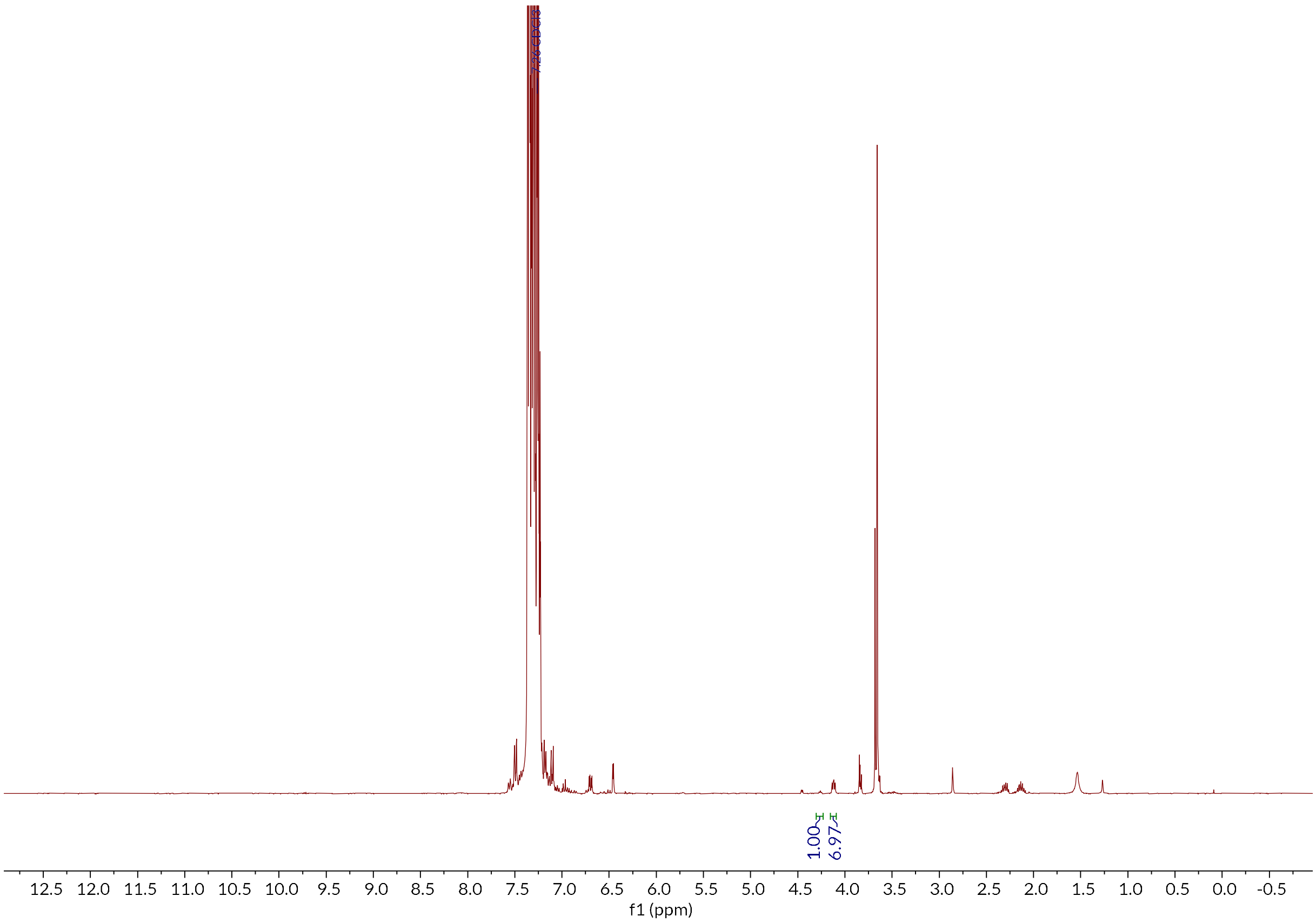

Figure S3: ^{1}H NMR spectra of crude reaction of **7**.

Following hydroarylation procedure by Matsunaga and coworkers.[8] A solution of $Ru(bpy)_3Cl_2$ (2.6 mg, 2.0 mol%), [Co-1] (12.0 mg, 10 mol%), ascorbic acid (70.4 mg, 0.40 mmol), and **7** (76.7 mg, 0.20 mmol) in ^{t}BuOH:H_2O (3:1, 3.0 mL) was irradiated (2 x 450 nm lamps) for 16 hours. The mixture was quenched with $NaHCO_3$, extracted with CH_2Cl_2, washed with brine, dried over Na_2SO_4 and concentrated under reduced pressure.

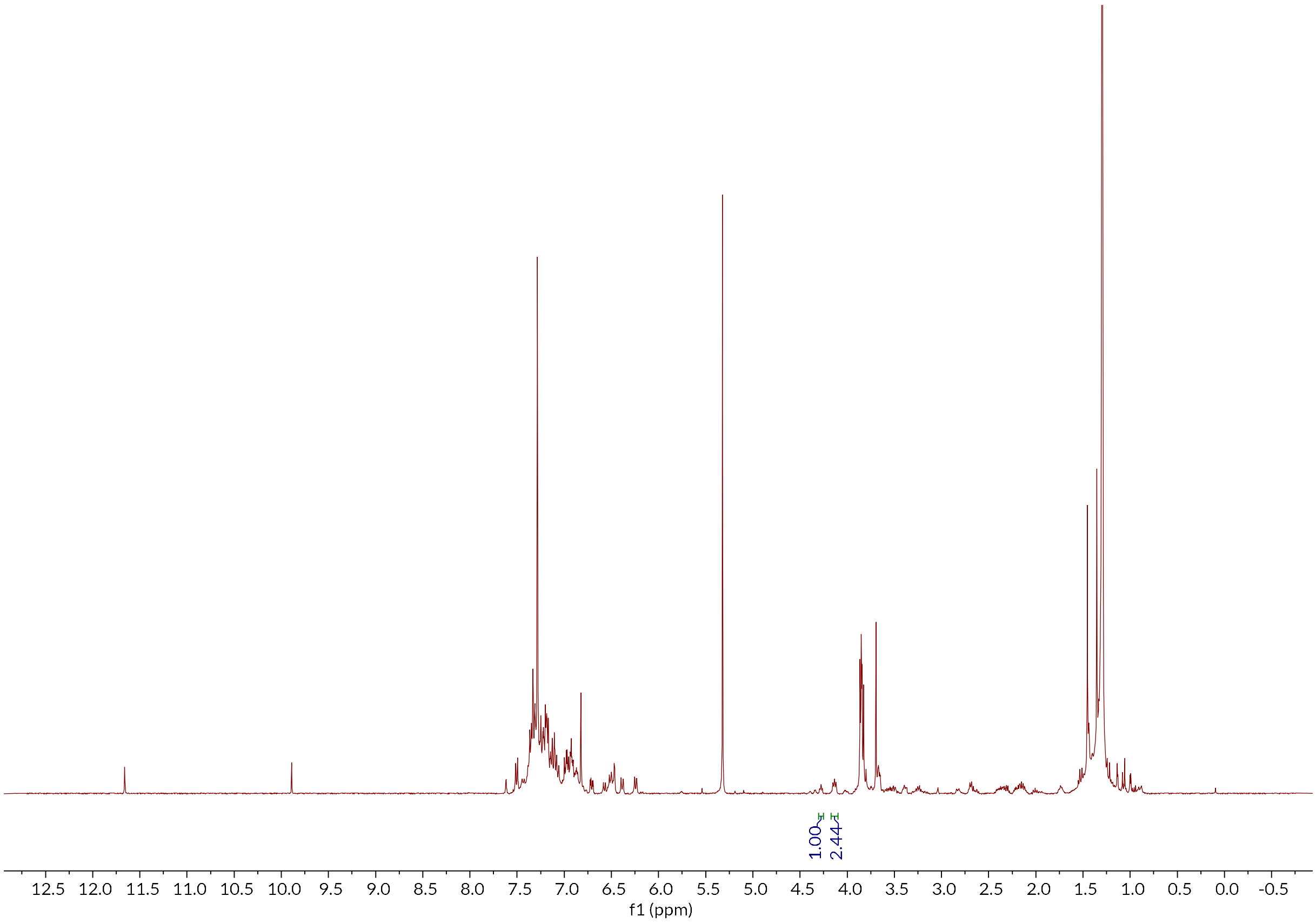

Figure S4: ^{1}H NMR spectra of crude reaction of **7** under Matsunaga's conditions.

4.2 Isotopic Labelling Experiments

_N_-Cinnamylbenzen-_d_$_5$-amine (3a-_d_$_5$)

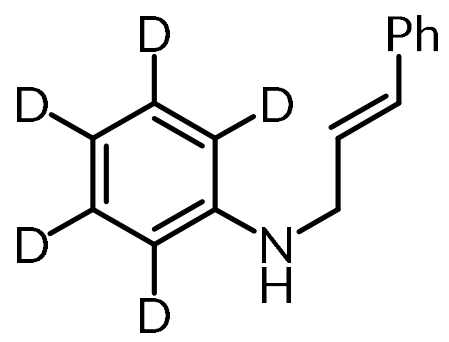

Following general procedure **A**, aniline-_d_$_5$ (1.00 mL, 10.96 mmol), _trans_-cinnamaldehyde (1.38 mL, 10.96 mmol), acetic acid (0.63 mL, 10.96 mmol), sodium triacetoxyborohydride (4.64 g, 21.92 mmol) and CH_2Cl_2 (36.5 mL), after purification by column chromatography (hexane/CH_2Cl_2, 80:20 → 70:30 → 60:40), gave the title compound **3a-_d_$_5$** (1.26 g, 54%) as a brown oil.

ν_{max} (film) / cm^{-1}: 3412 (N–H), 3024, 1572, 1474, 1400, 1360, 1213, 966, 746, 692

^{1}H NMR (500 MHz, $CDCl_3$) δ_H: 7.43 – 7.38 (2H, m), 7.36 – 7.31 (2H, m), 7.28 – 7.23 (1H, m), 6.65 (1H, d, _J_ 16.0), 6.36 (1H, dt, _J_ 15.5, 5.5), 3.96 (2H, dd, _J_ 5.8, 1.7), 3.86 (1H, br s)

^{13}C{^{1}H} NMR (126 MHz, $CDCl_3$) δ_C: 148.0, 137.0, 131.6, 129.2 – 128.8 (m), 128.7, 127.7, 127.2, 126.5, 117.6 – 116.9 (m), 113.2 – 112.3 (m), 46.3

HRMS: (ESI$^+$) $C_{15}H_9D_5N$ [M-H]$^+$ requires 213.1435, found 213.1434 (-0.5 ppm)

_N_-Benzyl-_N_-cinnamylaniline-_d_$_5$ (1aa-_d_$_5$)

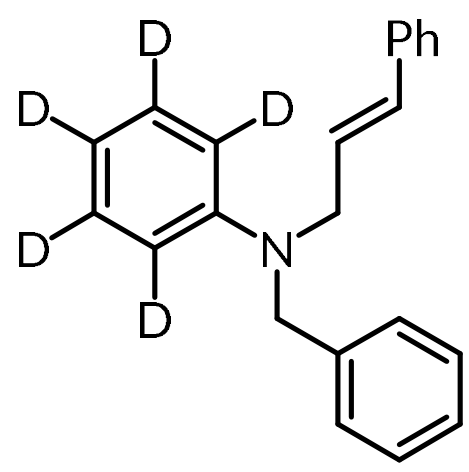

Following general procedure **C**, **3a-_d_$_5$** (700 mg, 3.26 mmol), benzyl bromide (0.42 mL, 3.59 mmol), K_2CO_3 (497 mg, 3.59 mmol), and acetone (16.3 mL), after purification by column chromatography (hexane/CH_2Cl_2, 80:20 → 70:30), gave the title compound **1aa-_d_$_5$** (765 mg, 77%, 98% deuterium) as a white solid.

mp 57 – 59 °C

ν_{max} (film) / cm^{-1}: 3026, 1564, 1425, 1360, 1194, 959, 731, 694

^{1}H NMR (500 MHz, $CDCl_3$) δ_H: 7.50 – 7.29 (10H, m), 6.62 (1H, d, _J_ 15.9), 6.38 (1H, dt, _J_ 15.9, 5.3), 4.70 (2H, s), 4.27 (2H, dd, _J_ 5.3, 1.7)

$^{13}C\{^1H\}$ NMR (126 MHz, $CDCl_3$) δ_C: 149.0, 139.0, 136.9, 131.5, 129.1 – 128.8 (m), 128.72, 128.65, 127.6, 127.0, 126.8, 126.4, 125.6, 116.5 – 116.0 (m), 112.6 – 111.9 (m), 54.0, 52.6

HRMS: (ESI$^+$) $C_{22}H_{15}D_5N$ [M-H]$^+$ requires 303.1904, found 303.1903 (-0.3 ppm)

(±)-1-Benzyl-4-phenyl-1,2,3,4-tetrahydroquinoline-3,5,6,7,8-d$_5$ (2aa-*d*$_5$)

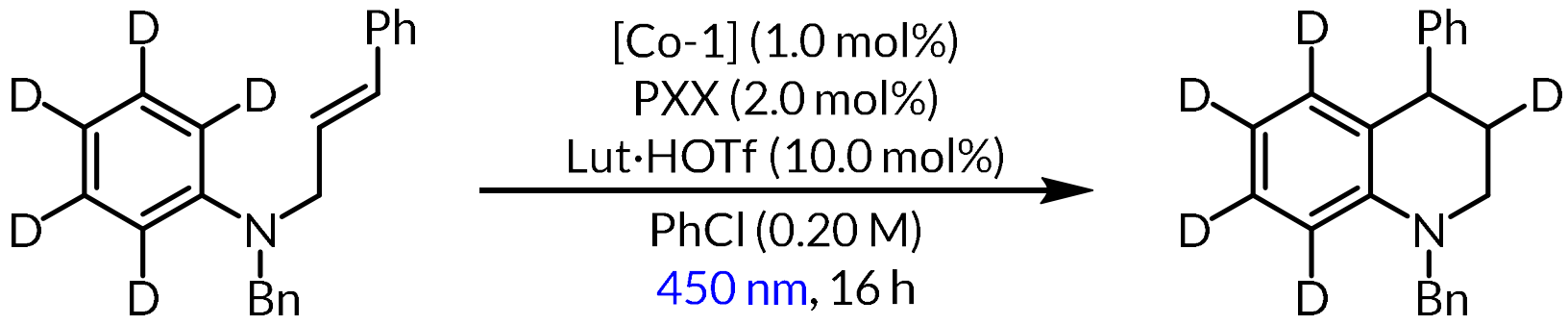

Following general procedure **E**, [Co-1] (1.2 mg, 0.002 mmol, 1.0 mol%), PXX (1.1 mg, 0.004 mmol, 2.0 mol%), Lut·HOTf (5.1 mg, 0.02 mmol, 10 mol%), **1aa-*d*$_5$** (60.8 mg, 0.20 mmol), and PhCl (1.0 mL) for 16 h at 450 nm irradiation gave a ^{1}H-NMR yield of 99%. Purification by column chromatography (hexane/CH_2Cl_2, 95:5 → 80:20 → 70:30) gave the title compound **2aa-*d*$_5$** (54.6 mg, 90%) as a yellow oil.

ν_{max} (film) / cm^{-1}: 3024, 2920, 1574, 1429, 1354, 729, 698

^1H NMR (500 MHz, $CDCl_3$) δ_H: 7.40 – 7.31 (6H, m), 7.30 – 7.23 (2H, m), 7.17 (2H, d, *J* 7.4), 4.56 (2H, s), 4.22 (1H, d, *J* 5.8), 3.40 – 3.26 (2H, m), 2.39 – 2.25 (0.54H, m), 2.22 – 2.11 (0.66H, m)

Note: 80% deuterium incorporation at the C3 position.

$^{13}C\{^1H\}$ NMR (126 MHz, $CDCl_3$) δ_C: 146.3, 145.8, 138.9, 130.0 – 129.5 (m), 128.8, 128.7, 128.4, 127.6 – 127.1 (m), 127.0, 126.7, 126.3, 124.0, 115.9 – 115.1 (m), 111.1 – 110.5 (m), 55.4, 46.7, 43.4, 30.5 – 30.1 (m)

HRMS: (ESI$^+$) $C_{22}H_{13}D_5N$ [M-3H]$^+$ requires 301.1748, found 301.1746 (-0.7 ppm)

4.3 On-Off Experiments

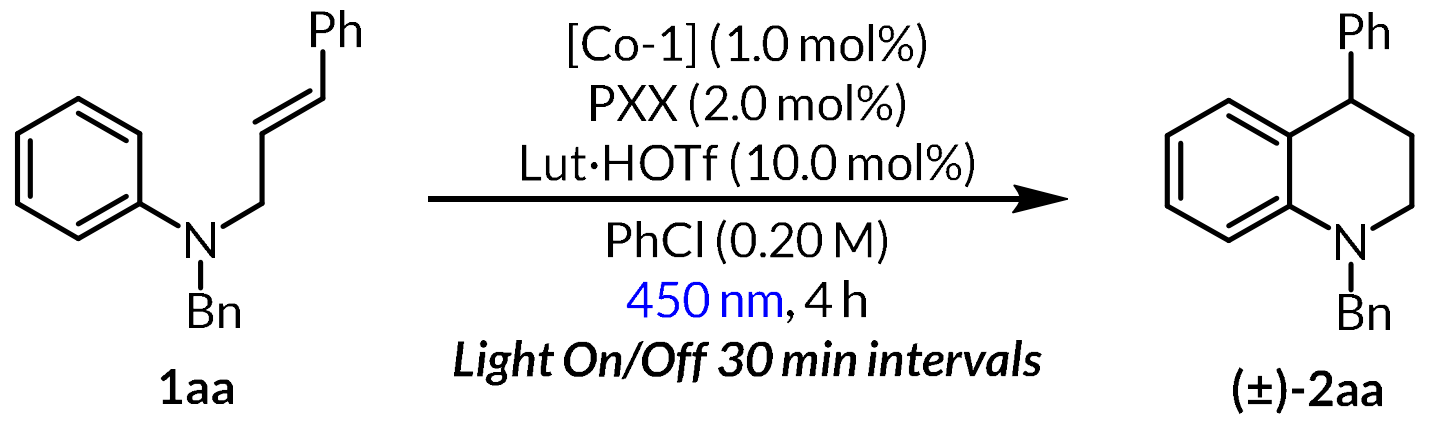

Reaction set up following general procedure **E** of 0.20 mmol of **1aa**. The reaction solution was then irradiated for 30 minutes then placed in the dark for 30 minutes and this continued over a period of 4 hours. A stock solution of 1,2-dibromoethane was prepared (17.2 µL (0.20 mmol)) in 1.0 mL of $CDCl_3$) as the internal standard. At each-time point (30 min): 10 µL of the reaction solution was taken from the vial, concentrated under reduced pressure, dissolved in $CDCl_3$ and 10 µL of internal standard stock solution was added for ^{1}H NMR analysis. When in a light-off phase, the seal of the vial was changed, and the reaction was back refilled with N_2 (×3) and sparged for 5 mins prior to being re-illuminated.

Table 11: On/Off experiment results

Entry	Time / min	Yield of (±)-2aa / %	Yield of 1aa / %	Mass Balance
**1**	0	0.0	100.0	100.0
**2**	30	12.0	83.5	95.5
**3**	60	12.0	87.0	99.0
**4**	90	21.0	78.5	99.5
**5**	120	21.0	78.0	99.0
**6**	150	26.5	65.5	92.0
**7**	180	26.5	68.0	94.5
**8**	210	38.5	62.5	101.0
**9**	240	38.5	61.0	99.5

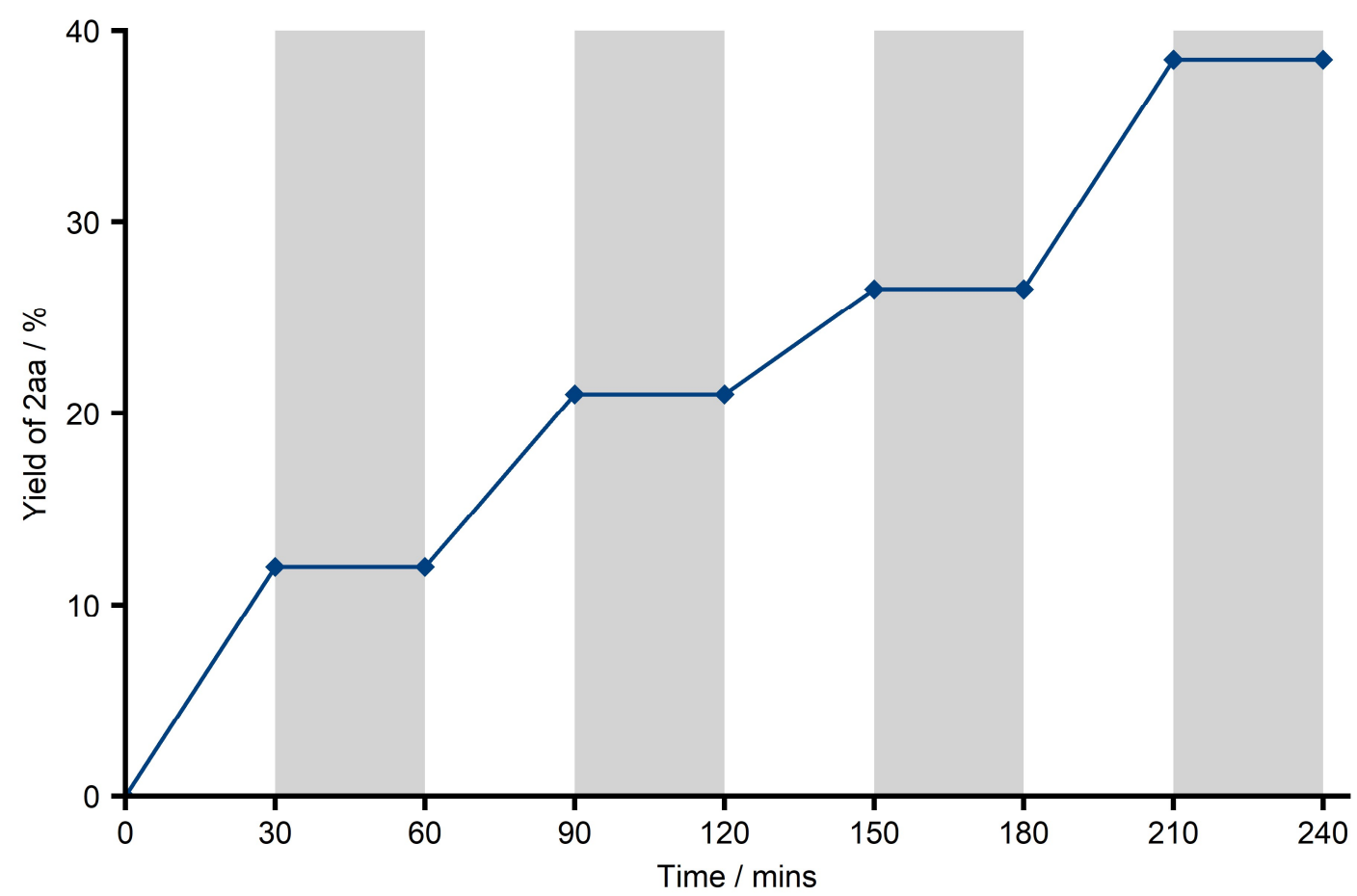

Figure S5: On/Off experiment data

4.4 Hammett Analysis

4.4.1 Hammett Substrate Synthesis

_N_-Cinnamyl-4-methoxy-_N_-phenylaniline (SI-35)

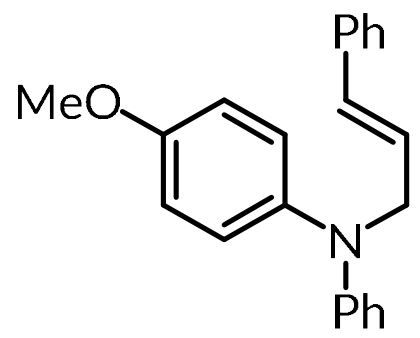

Following general procedure **B**, 4-methoxy-*N*-phenylaniline (500 mg, 2.51 mmol), cinnamyl bromide (593 mg, 3.01 mmol), NaH (121 mg, 3.01 mmol), and DMF (10 mL), after purification by column chromatography (hexane/Et_2O, 99:1 → 98:2), gave the title compound **SI-35** (423 mg, 53%) as a colourless oil.

ν_{max} (film) / cm^{-1}: 3024, 2833, 1593, 1495, 1240, 1217, 1032, 962, 829, 745, 731, 691

^{1}H NMR (500 MHz, $CDCl_3$) δ_H: 7.37 – 7.33 (2H, m), 7.33 – 7.28 (2H, m), 7.24 – 7.15 (5H, m), 6.95 – 6.89 (2H, m), 6.85 – 6.81 (2H, m), 6.78 (1H, tt, *J* 7.3, 1.1), 6.58 (1H, dt, *J* 15.9, 1.7), 6.35 (1H, dt, *J* 15.9, 5.3), 4.46 (2H, dd, *J* 5.4, 1.7), 3.82 (3H, s)

^{13}C{^{1}H} NMR (126 MHz, $CDCl_3$) δ_C: 156.6, 148.9, 140.7, 137.1, 131.4, 129.1, 128.6, 127.5, 127.3, 126.4, 126.3, 118.3, 115.7, 115.0, 55.6, 54.8

HRMS: (ESI$^+$) $C_{22}H_{21}NONa$ [M+Na]$^+$ requires 338.1515, found 338.1515 (0.0 ppm)

_N_-Cinnamyl-4-methyl-_N_-phenylaniline (SI-36)

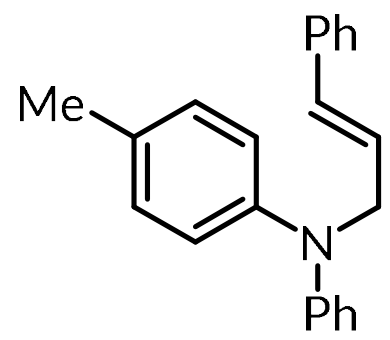

Following general procedure **B**, 4-methyl-*N*-phenylaniline (500 mg, 2.73 mmol), cinnamyl bromide (643 mg, 3.26 mmol), NaH (130 mg, 3.26 mmol), and DMF (10 mL), after purification by column chromatography (hexane/CH_2Cl_2, 95:5 → 80:20), gave the title compound **SI-36** (665 mg, 81%) as a pale-yellow solid.

mp 54 – 56 °C

ν_{max} (film) / cm^{-1}: 3024, 2981, 1678, 1595, 1508, 1495, 1352, 964, 746, 692

^{1}H NMR (500 MHz, $CDCl_3$) δ_H: 7.35 (2H, d, *J* 7.6), 7.32 – 7.27 (2H, m), 7.27 – 7.20 (3H, m), 7.14 (2H, d, *J* 8.0), 7.08 (2H, d, *J* 8.2), 6.98 (2H, d, *J* 8.2), 6.88 (1H, t, *J* 7.4), 6.60 (1H, d, *J* 16.0), 6.35 (1H, dt, *J* 15.9, 5.2), 4.51 (2H, d, *J* 5.2), 2.34 (3H, s)

^{13}C{^{1}H} NMR (126 MHz, $CDCl_3$) δ_C: 148.4, 145.4, 137.1, 132.4, 131.4, 130.2, 129.2, 128.6, 127.5, 126.5, 126.4, 123.3, 119.9, 118.5, 54.6, 20.9

HRMS: (ESI$^+$) $C_{22}H_{20}N$ [M-H]$^+$ requires 298.1590, found 298.1589 (-0.3 ppm)

4-(*Tert*-butyl)-*N*-cinnamyl-*N*-phenylaniline (SI-37)

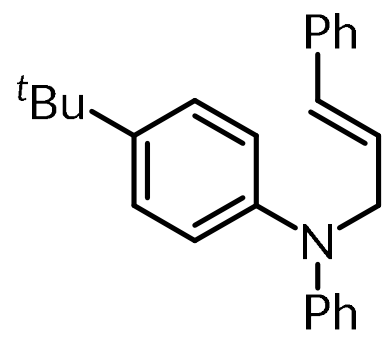

Following general procedure **A**, 4-(*tert*-butyl)-*N*-phenylaniline (1.01 g, 4.50 mmol), *trans*-cinnamaldehyde (0.57 mL, 4.50 mmol), acetic acid (0.26 mL, 4.50 mmol), sodium triacetoxyborohydride (1.91 g, 9.00 mmol) and CH_2Cl_2 (15.0 mL), after purification by column chromatography (hexane/CH_2Cl_2, 95:5), gave the title compound **SI-37** (368 mg, 22%) as a white solid.

mp 93 – 95 °C

ν_{max} (film) / cm^{-1}: 2959, 1593, 1512, 1495, 1264, 964, 746, 731

^{1}H NMR (500 MHz, $CDCl_3$) δ_H: 7.43 – 7.21 (9H, m), 7.11 – 7.06 (2H, m), 7.07 – 7.03 (2H, m), 6.95 – 6.89 (1H, m), 6.63 (1H, dt, *J* 16.0, 1.9), 6.37 (1H, dt, *J* 16.0, 5.2), 4.54 (2H, dd, *J* 5.3, 1.8), 1.36 (9H, s)

^{13}C{^{1}H} NMR (126 MHz, $CDCl_3$) δ_C: 148.2, 145.3, 145.1, 137.1, 131.3, 129.3, 128.6, 127.5, 126.6, 126.5, 126.3, 121.8, 120.4, 119.4, 54.6, 34.3, 31.6

HRMS: (ESI$^+$) $C_{25}H_{27}NNa$ [M+Na]$^+$ requires 364.2036, found 364.2037 (+0.3 ppm)

N-Cinnamyl-N-phenyl-[1,1'-biphenyl]-4-amine (SI-38)

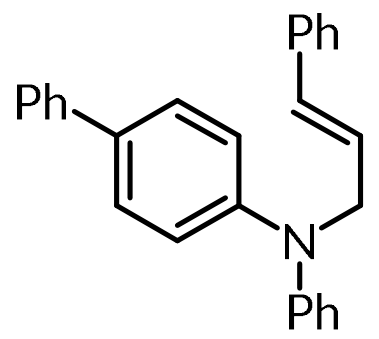

Following general procedure **A**, *N*-phenyl-[1,1'-biphenyl]-4-amine (1.23 g, 5.00 mmol), *trans*-cinnamaldehyde (0.63 mL, 5.00 mmol), acetic acid (0.29 mL, 5.00 mmol), sodium triacetoxyborohydride (2.12 g, 10.00 mmol) and CH_2Cl_2 (16.7 mL), after purification by column chromatography (hexane/Et_2O, 99:1), gave the title compound **SI-38** (566 mg, 31%) as a brown oil.

ν_{max} (film) / cm^{-1}: 3026, 1591, 1520, 1438, 962, 826, 760, 690

^{1}H NMR (500 MHz, $CDCl_3$) δ_H: 7.62 – 7.58 (2H, m), 7.55 – 7.51 (2H, m), 7.46 – 7.41 (2H, m), 7.40 – 7.29 (7H, m), 7.26 – 7.22 (1H, m), 7.22 – 7.18 (2H, m), 7.16 – 7.12 (2H, m), 7.07 – 7.02 (1H, m), 6.64 (1H, dt, *J* 15.9, 1.8), 6.39 (1H, dt, *J* 15.9, 5.2), 4.59 (2H, dd, *J* 5.2, 1.8)

^{13}C{^{1}H} NMR (126 MHz, $CDCl_3$) δ_C: 147.8, 147.4, 141.0, 137.0, 133.6, 131.6, 129.5, 128.8, 128.7, 128.0, 127.6, 126.7 (2C), 126.5, 126.1, 122.3, 122.1, 120.0, 54.6

<u>Note: 126.7 ppm peak contains two carbon peaks.</u>

HRMS: (ESI$^+$) $C_{27}H_{23}NNa$ [M+Na]$^+$ requires 384.1723, found 384.1721 (-0.5 ppm)

4-Chloro-N-cinnamyl-N-phenylaniline (SI-39)

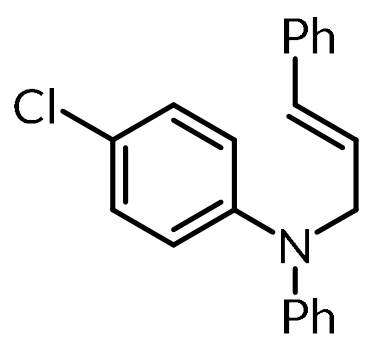

Prepared using the same synthetic procedure as substrate **7**. 4-Chloro-*N*-cinnamylaniline (487 mg, 2.00 mmol), ^{t}BuOK (292 mg, 2.60 mmol), Pd(dba)$_2$ (57 mg, 0.10 mmol, 5 mol%), and [(^{t}Bu$_3$)PH]BF_4 (58 mg, 0.20 mmol, 10 mol%), and bromobenzene (0.32 mL, 3.00 mmol) and PhMe (4.0 mL) after purification by column chromatography (hexane/Et_2O, 99:1), gave the title compound **SI-39** (543 mg, 85%) as a yellow solid.

mp 74 – 76 °C

ν_{max} (film) / cm^{-1}: 3024, 1585, 1487, 1364, 1219, 1088, 964, 816, 748, 731, 692

^{1}H NMR (500 MHz, CDCl$_3$) δ_H: 7.36 – 7.27 (6H, m), 7.25 – 7.19 (3H, m), 7.13 – 7.09 (2H, m), 7.03 (1H, tt, *J* 7.2, 1.1), 6.99 – 6.94 (2H, m), 6.58 (1H, dt, *J* 15.9, 1.8), 6.31 (1H, dt, *J* 16.0, 5.2), 4.50 (2H, dd, *J* 5.2, 1.7)

^{13}C{^{1}H} NMR (126 MHz, CDCl$_3$) δ_C: 147.7, 146.6, 136.8, 131.7, 129.6, 129.3, 128.7, 127.7, 126.5, 125.8, 125.7, 122.5, 122.0, 121.1, 54.6

HRMS: (ESI$^+$) $C_{21}H_{17}{}^{35}ClN$ [M-H]$^+$ requires 318.1044, found 318.1043 (-0.3 ppm)

4-Bromo-*N*-cinnamyl-*N*-phenylaniline (SI-40)

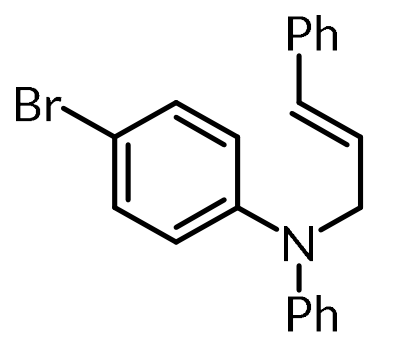

Following general procedure **B**, 4-bromo-*N*-phenylaniline (500 mg, 2.02 mmol), cinnamyl bromide (477 mg, 2.42 mmol), NaH (97 mg, 2.42 mmol), and DMF (10 mL), after purification by column chromatography (hexane/Et$_2$O, 99:1), gave the title compound **SI-40** (564 mg, 77%) as a white solid.

mp 105 – 107 °C

ν_{max} (film) / cm^{-1}: 3024, 2359, 1582, 1485, 1350, 1217, 1070, 962, 812, 746, 729, 691

^{1}H NMR (500 MHz, CDCl$_3$) δ_H: 7.37 – 7.27 (8H, m), 7.25 – 7.20 (1H, m), 7.17 – 7.09 (2H, m), 7.04 (1H, t, *J* 7.4), 6.89 (2H, d, *J* 8.9), 6.57 (1H, dt, *J* 16.0, 1.8), 6.30 (1H, dt, *J* 16.0, 5.2), 4.49 (2H, dd, *J* 5.3, 1.8)

^{13}C{^{1}H} NMR (126 MHz, CDCl$_3$) δ_C: 147.5, 147.1, 136.8, 132.2, 131.7, 129.6, 128.7, 127.7, 126.5, 125.6, 122.9, 122.5, 121.0, 112.8, 54.5

HRMS: (ESI$^+$) $C_{21}H_{17}{}^{79}BrN$ [M-H]$^+$ requires 362.0539, found 362.0541 (+0.6 ppm)

Methyl 4-(cinnamylamino)benzoate (SI-41)

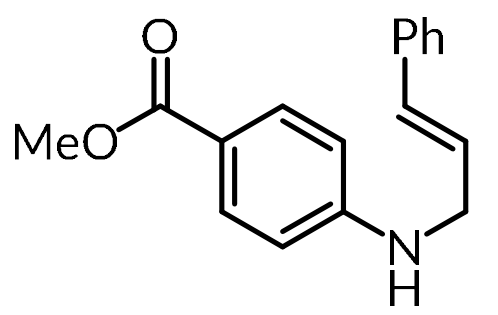

Following general procedure **A**, methyl 4-aminobenzoate (756 mg, 5.00 mmol), *trans*-cinnamaldehyde (0.63 mL, 5.00 mmol), acetic acid (0.29 mL, 5.00 mmol), sodium

triacetoxyborohydride (2.12 g, 10.00 mmol) and CH_2Cl_2 (16.7 mL), after purification by column chromatography (hexane/EtOAc, 95:5 → 90:10 → 85:15), gave the title compound **SI-41** (805 mg, 60%) as a white solid.

mp 133 – 135 °C

ν_{max} (film) / cm^{-1}: 3362 (N–H), 2947, 1684, 1601 (C=O), 1526, 1433, 1277, 1175, 1111, 986, 771

^{1}H NMR (500 MHz, $CDCl_3$) δ_H: 7.91 – 7.84 (2H, m), 7.38 – 7.35 (2H, m), 7.34 – 7.29 (2H, m), 7.28 – 7.21 (1H, m), 6.65 – 6.57 (3H, m), 6.29 (1H, dt, *J* 15.8, 5.8), 4.33 (1H, t, *J* 5.9), 4.00 (2H, td, *J* 5.7, 1.6), 3.85 (3H, s)

^{13}C{^{1}H} NMR (126 MHz, $CDCl_3$) δ_C: 167.4, 151.8, 136.7, 132.3, 131.7, 128.8, 127.9, 126.5, 125.9, 118.8, 111.9, 51.7, 45.7

HRMS: (ESI$^+$) $C_{17}H_{18}NO_2$ $[M+H]^+$ requires 268.1332, found 268.1330 (-0.7 ppm)

Methyl 4-(cinnamyl(phenyl)amino)benzoate (SI-42)

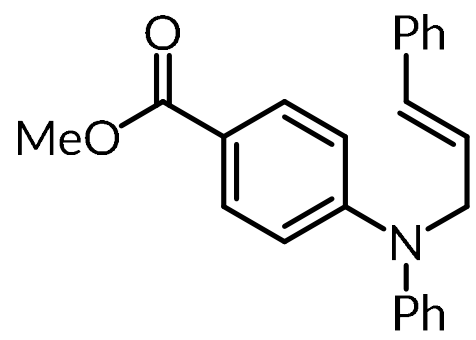

Prepared using the same synthetic procedure as substrate **7**. **SI-41** (535 mg, 2.00 mmol), ^{t}BuOK (292 mg, 2.60 mmol), $Pd(dba)_2$ (57 mg, 0.10 mmol, 5 mol%), and $[(^tBu_3)PH]BF_4$ (58 mg, 0.20 mmol, 10 mol%), and bromobenzene (0.32 mL, 3.00 mmol) and PhMe (4.0 mL) after purification by column chromatography (hexane/Et_2O, 90:10), gave the title compound **SI-42** (345 mg, 50%) as a yellow oil.

ν_{max} (film) / cm^{-1}: 3024, 2947, 1703 (C=O), 1605, 1587, 1512, 1491, 1433, 1273, 1179, 1107, 765, 692

^{1}H NMR (500 MHz, $CDCl_3$) δ_H: 7.89 – 7.84 (2H, m), 7.43 – 7.38 (2H, m), 7.35 – 7.27 (6H, m), 7.25 – 7.20 (2H, m), 6.84 – 6.79 (2H, m), 6.57 (1H, dt, *J* 15.8, 1.8), 6.31 (1H, dt, *J* 16.0, 5.3), 4.55 (2H, dd, *J* 5.3, 1.7), 3.86 (3H, s)

^{13}C{^{1}H} NMR (126 MHz, $CDCl_3$) δ_C: 167.3, 151.9, 146.5, 136.7, 132.1, 131.2, 130.0, 128.7, 127.8, 126.6, 126.5, 125.7, 124.9, 119.4, 114.3, 54.6, 51.7

HRMS: (ESI$^+$) $C_{23}H_{20}NO_2$ $[M-H]^+$ requires 342.1489, found 342.1488 (-0.3 ppm)

N-Cinnamyl-N-phenyl-4-(trifluoromethyl)aniline (SI-43)

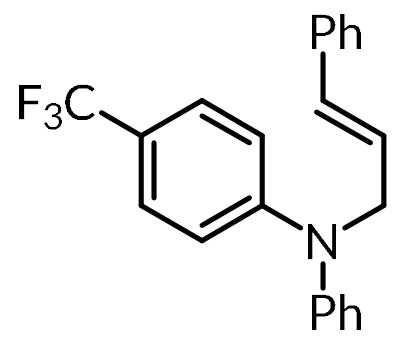

Prepared using the same synthetic procedure as substrate **7**. **SI-24** (555 mg, 2.00 mmol), ^{t}BuOK (292 mg, 2.60 mmol), Pd(dba)$_2$ (57 mg, 0.10 mmol, 5 mol%), and [(^{t}Bu$_3$)PH]BF$_4$ (58 mg, 0.20 mmol, 10 mol%), and bromobenzene (0.32 mL, 3.00 mmol) and PhMe (4.0 mL) after purification by column chromatography (hexane/Et$_2$O, 99:1), gave the title compound **SI-43** (438 mg, 62%) as a yellow solid.

mp 89 – 91 °C

ν_{max} (film) / cm^{-1}: 3028, 2351, 1614, 1593, 1520, 1493, 1323, 1107, 1067, 825, 698

^{1}H NMR (500 MHz, CDCl$_3$) δ_H: 7.45 – 7.17 (12H, m), 6.89 (2H, d, *J* 8.6), 6.62 – 6.55 (1H, m), 6.31 (1H, dt, *J* 16.0, 5.3), 4.54 (2H, dd, *J* 5.3, 1.8)

^{13}C{^{1}H} NMR (126 MHz, CDCl$_3$) δ_C: 150.7, 146.7, 136.7, 132.0, 130.0, 128.7, 127.8, 126.5, 126.4 (q, *J* 3.8), 126.0, 125.4, 125.1, 125.0 (q, *J* 270.6), 120.1 (q, *J* 32.6), 115.2, 54.6

^{19}F NMR (471 MHz, CDCl$_3$) δ_F: -61.2 (s)

HRMS: (ESI$^+$) $C_{22}H_{17}F_3N$ [M-H]$^+$ requires 352.1308, found 352.1305 (-0.9 ppm)

4-(Cinnamyl(phenyl)amino)benzonitrile (SI-44)

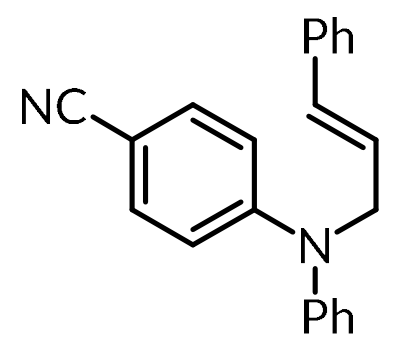

Following general procedure **B**, 4-(phenylamino)benzonitrile (300 mg, 1.54 mmol), cinnamyl bromide (365 mg, 1.85 mmol), NaH (74 mg, 1.85 mmol), and DMF (6 mL), after purification by column chromatography (hexane/EtOAc, 90:10 → 80:20), gave the title compound **SI-43** (302 mg, 63%) as a pale-yellow solid.

mp 91 – 93 °C

ν_{max} (film) / cm^{-1}: 3028, 2214 (C≡N), 1607, 1589, 1510, 1493, 1175, 824

^{1}H (500 MHz, CDCl$_3$) δ_H: 7.46 – 7.39 (4H, m), 7.36 – 7.22 (8H, m), 6.76 (2H, d, *J* 9.0), 6.57 (1H, dt, *J* 15.9, 1.7), 6.28 (1H, dt, *J* 16.0, 5.3), 4.52 (2H, dd, *J* 5.4, 1.8)

$^{13}C\{^1H\}$ NMR (126 MHz, $CDCl_3$) δ_C: 151.4, 145.8, 136.5, 133.5, 132.4, 130.3, 128.8, 128.0, 127.2, 126.6, 126.5, 124.2, 120.4, 114.3, 99.7, 54.7

HRMS: (ESI^+) $C_{22}H_{18}N_2Na$ $[M+Na]^+$ requires 333.1362, found 333.1361 (-0.3 ppm)

4.4.2 Competition Reactions

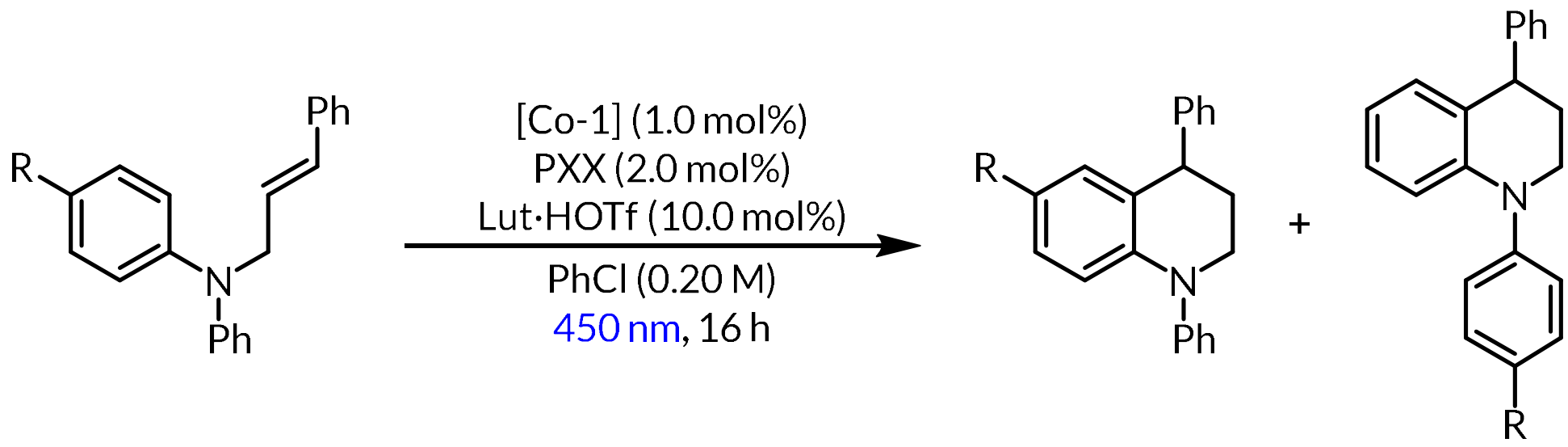

Hammett experiments were performed according to general procedure **E**. 1,2-Dibromoethane was added as the internal standard to the crude reaction mixture to determine the regioselectivity of the reaction via ^{1}H-NMR. If the regiomeric ratio could not be reliably calculated from the crude ^{1}H-NMR spectrum, isolation via column chromatography of the regioisomeric mix of products was necessary (Figure S10). Regiomeric ratio was determined via the tetrahydroquinoline aryl ring due to the characteristic differences observed depending on which aryl ring underwent cyclisation (Figure S11). Hammett parameters were taken from literature.[9]

Table 12: Hammett competition results

R Group	σ_p^+	σ_p	σ_p^-	σ_m	% Cyclised through Ph ring (K_H)	% Cyclised through R containing ring (K_X)
**OMe**	-0.78	-0.27	-0.26	0.12	31	69
**Me**	-0.31	-0.17	-0.17	-0.07	39	61
**^{t}Bu**	-0.26	-0.20	-0.13	-0.10	38	62
**Ph**	-0.18	-0.01	0.02	0.06	37	63
**H**	0	0	0	0	50	50
**Cl**	0.11	0.23	0.19	0.37	55	45
**Br**	0.15	0.23	0.25	0.39	54	46
**COOMe**	0.49	0.45	0.75	0.37	56	44
**CF_3**	0.61	0.54	0.65	0.43	64	36
**CN**	0.66	0.66	1.00	0.56	66	34

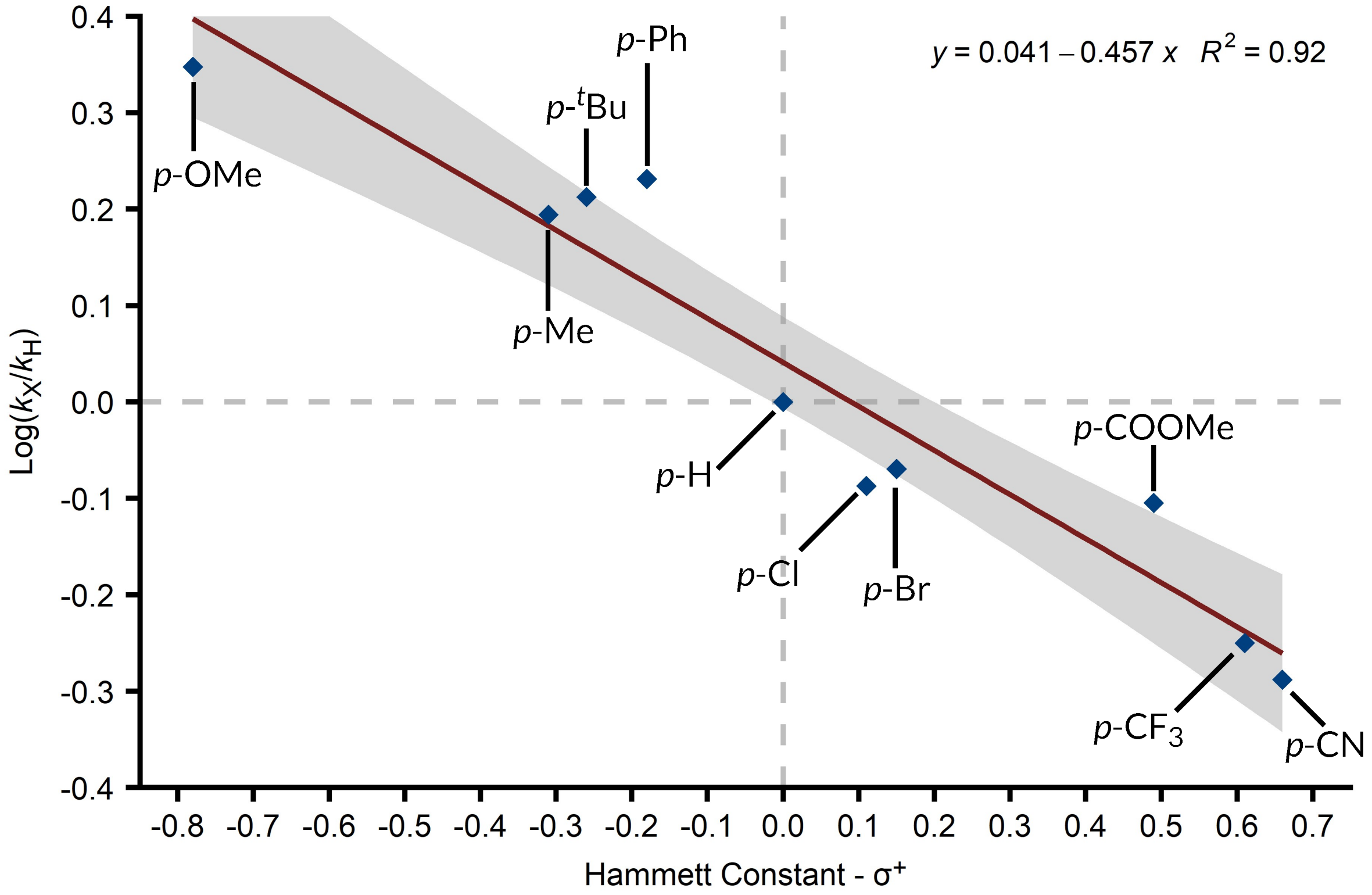

Figure S6: Hammett Constant - σ_p^+

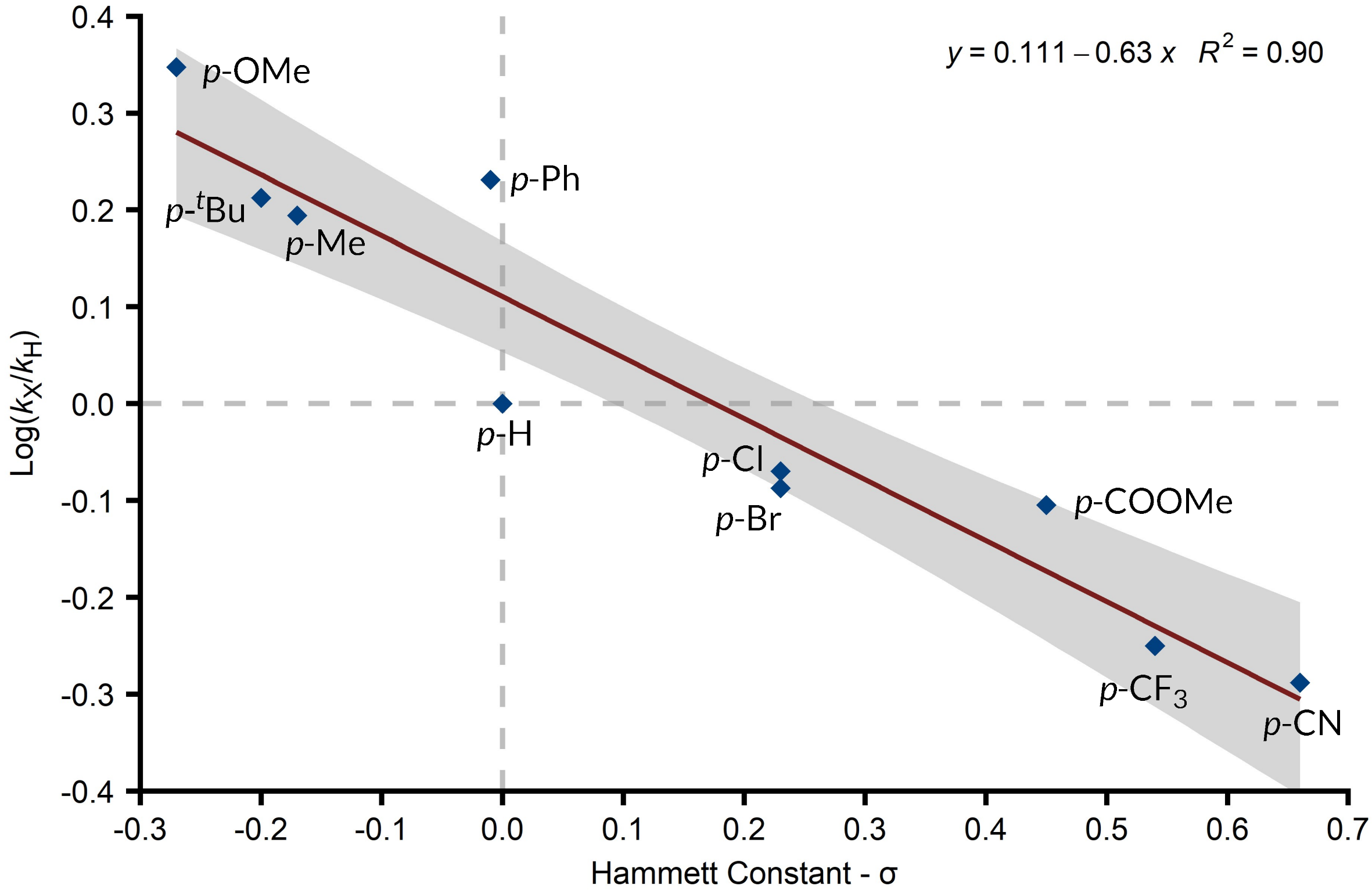

Figure S7: Hammett Constant - σ_p

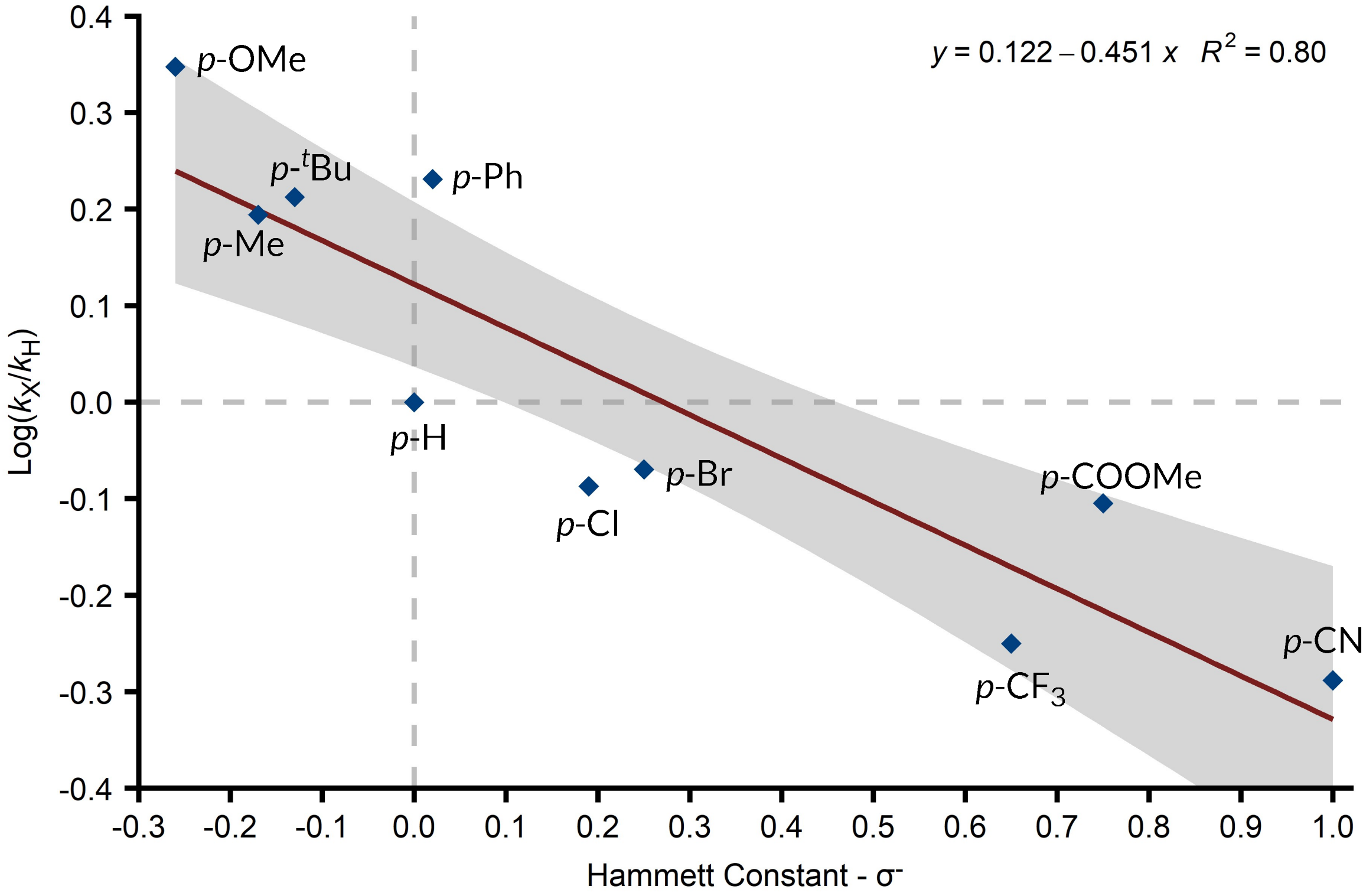

Figure S8: Hammett Constant – σ_p^-

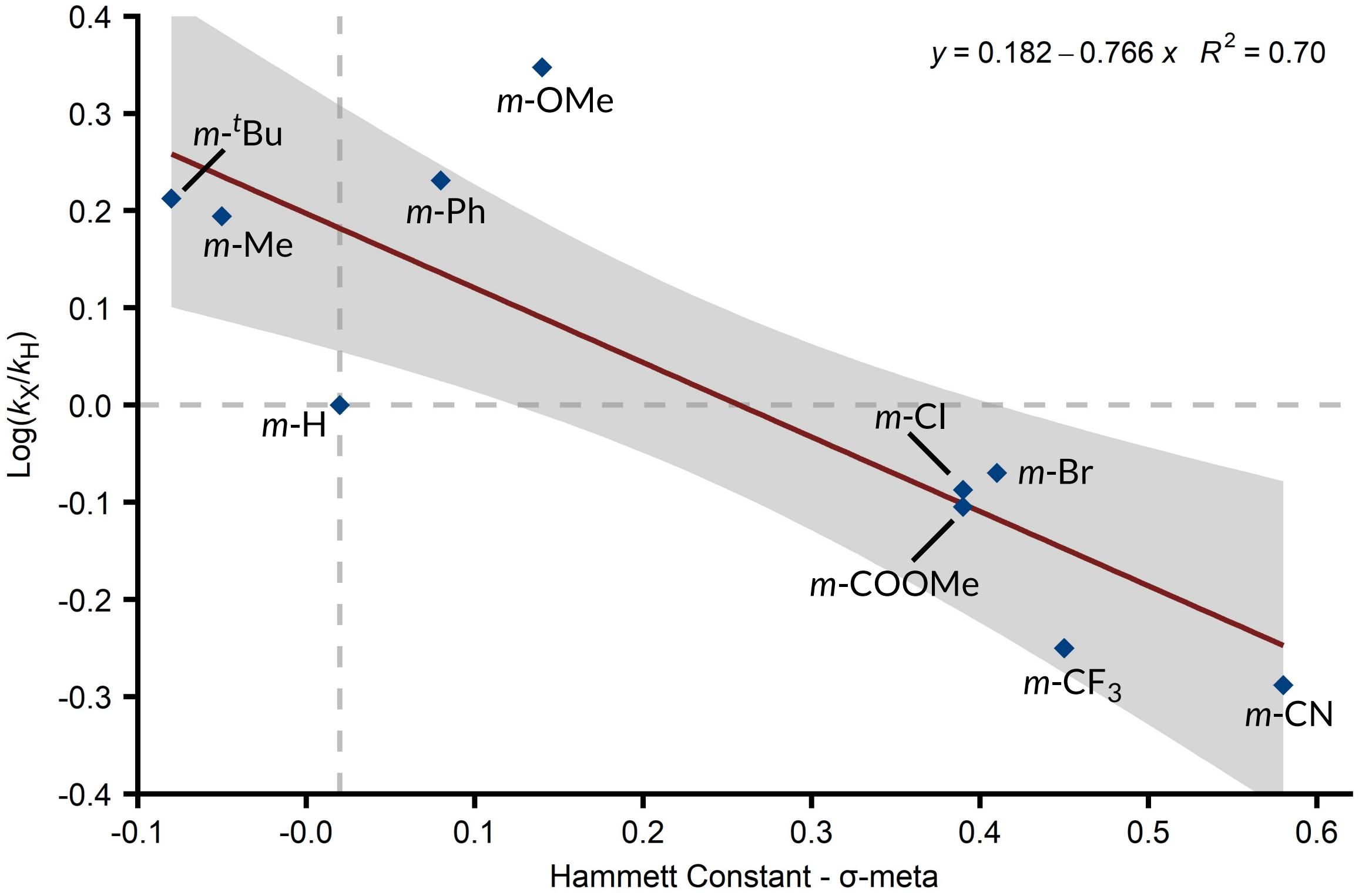

Figure S9: Hammett Constant – σ-meta

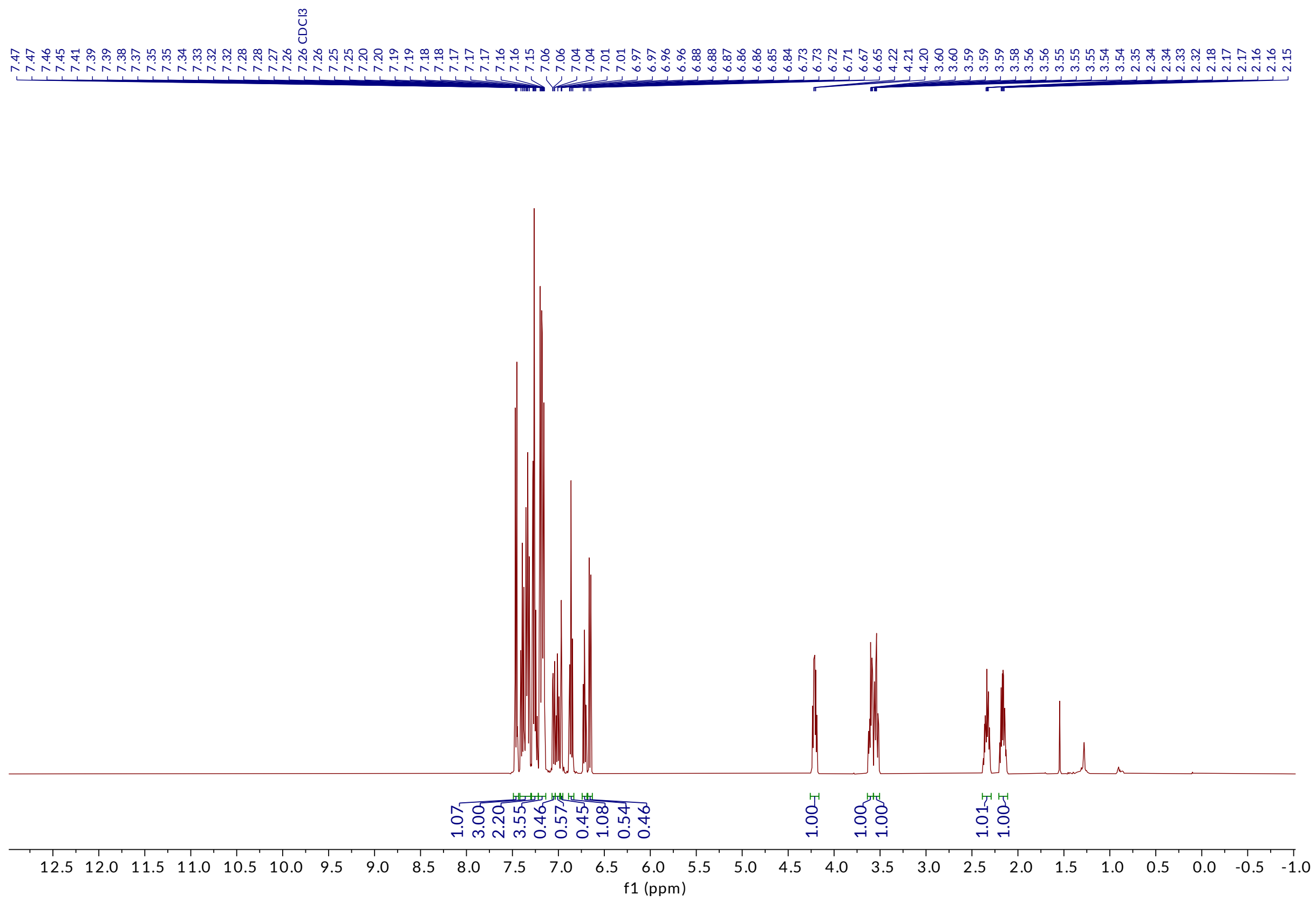

Figure S10: ^{1}H NMR spectrum of isolated regioisomers from 4-Br (**SI-40**) example.

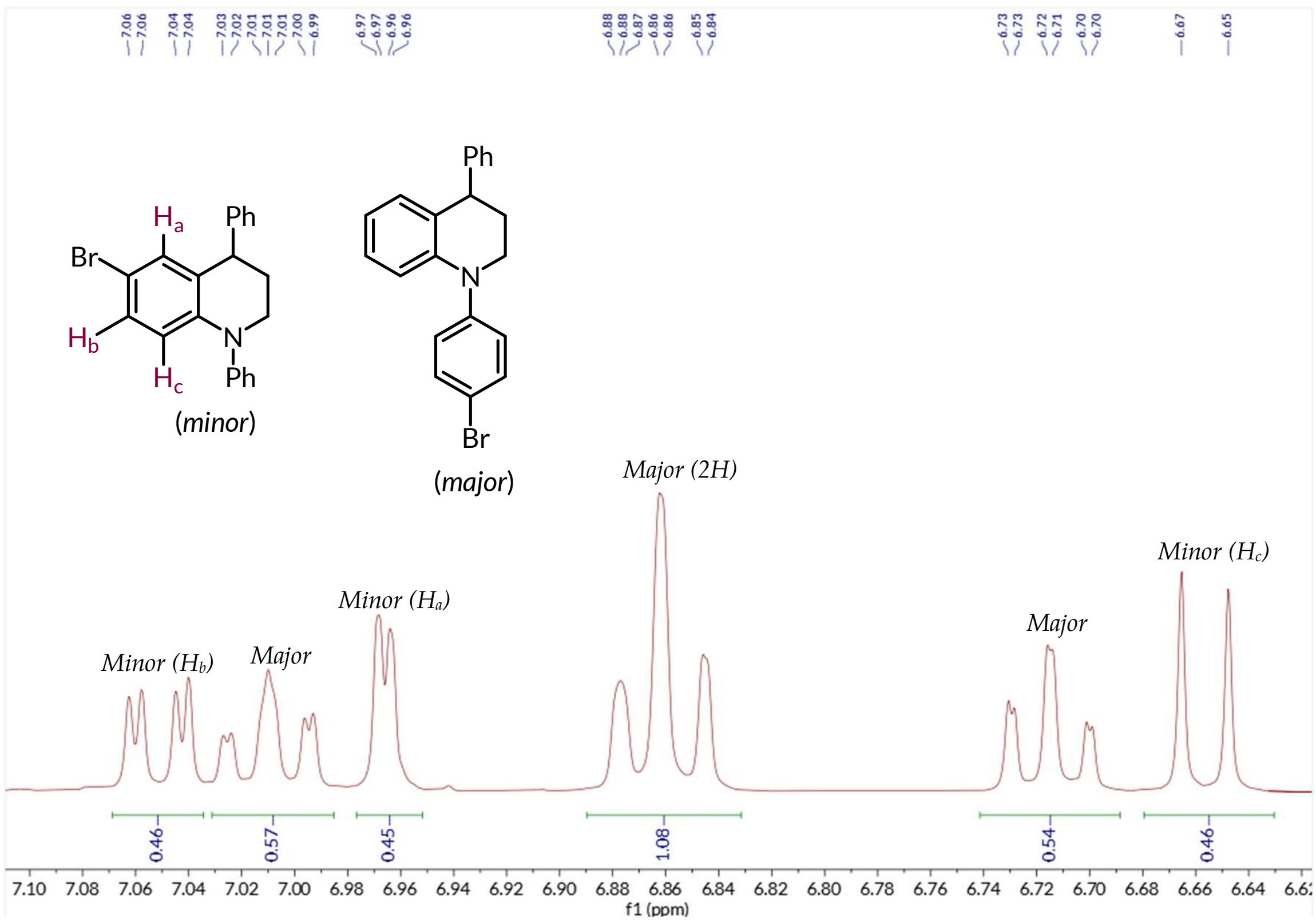

Figure S11: ^{1}H NMR spectrum of isolated regioisomers for 4-Br example, aromatic region zoomed in.

4.5 Stern-Volmer Quenching

4.5.1 UV-Vis Measurements

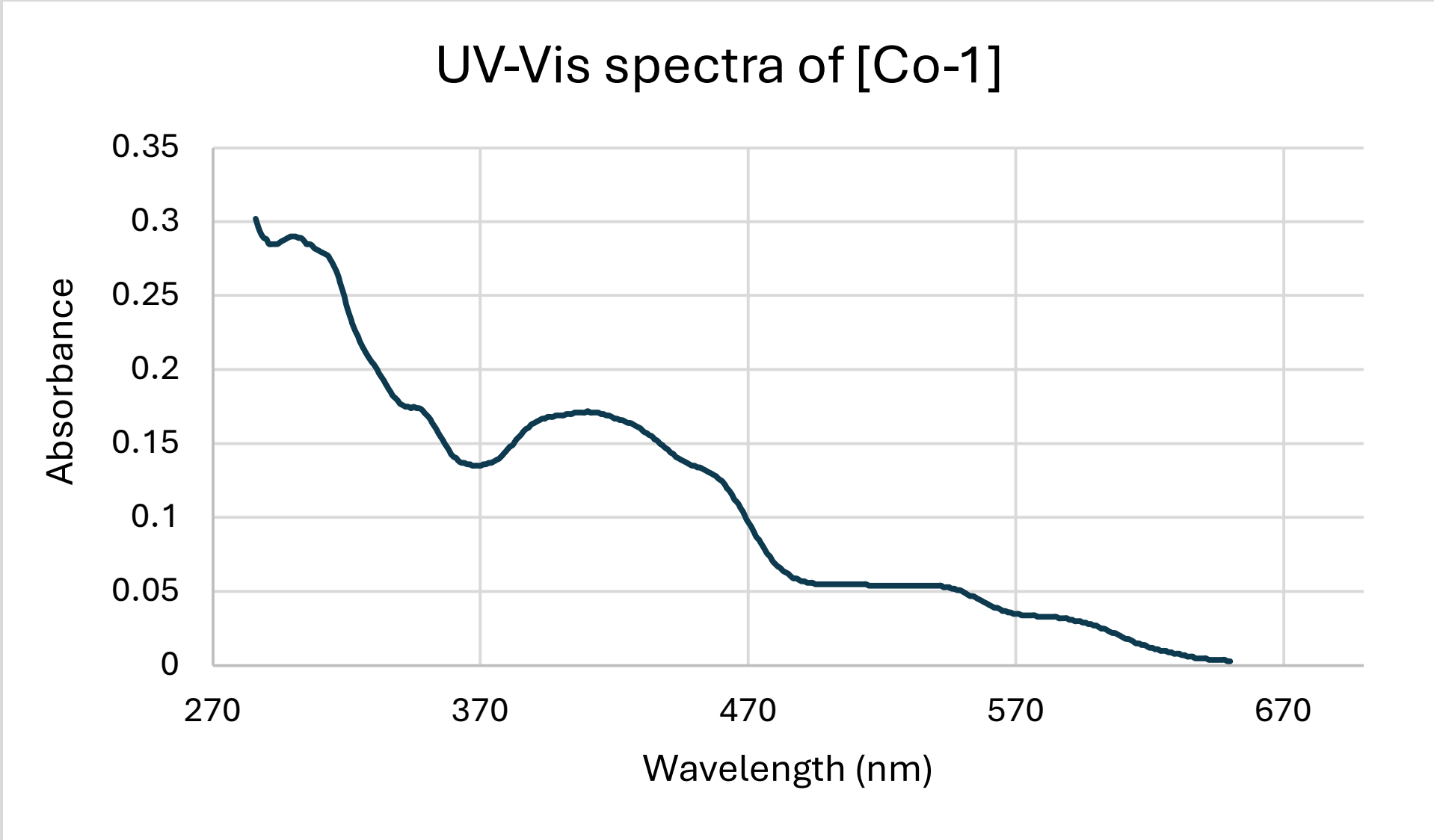

Figure S11: UV-Spectra of cobalt-salen **[Co-1]** in PhCl at 0.01 mM.

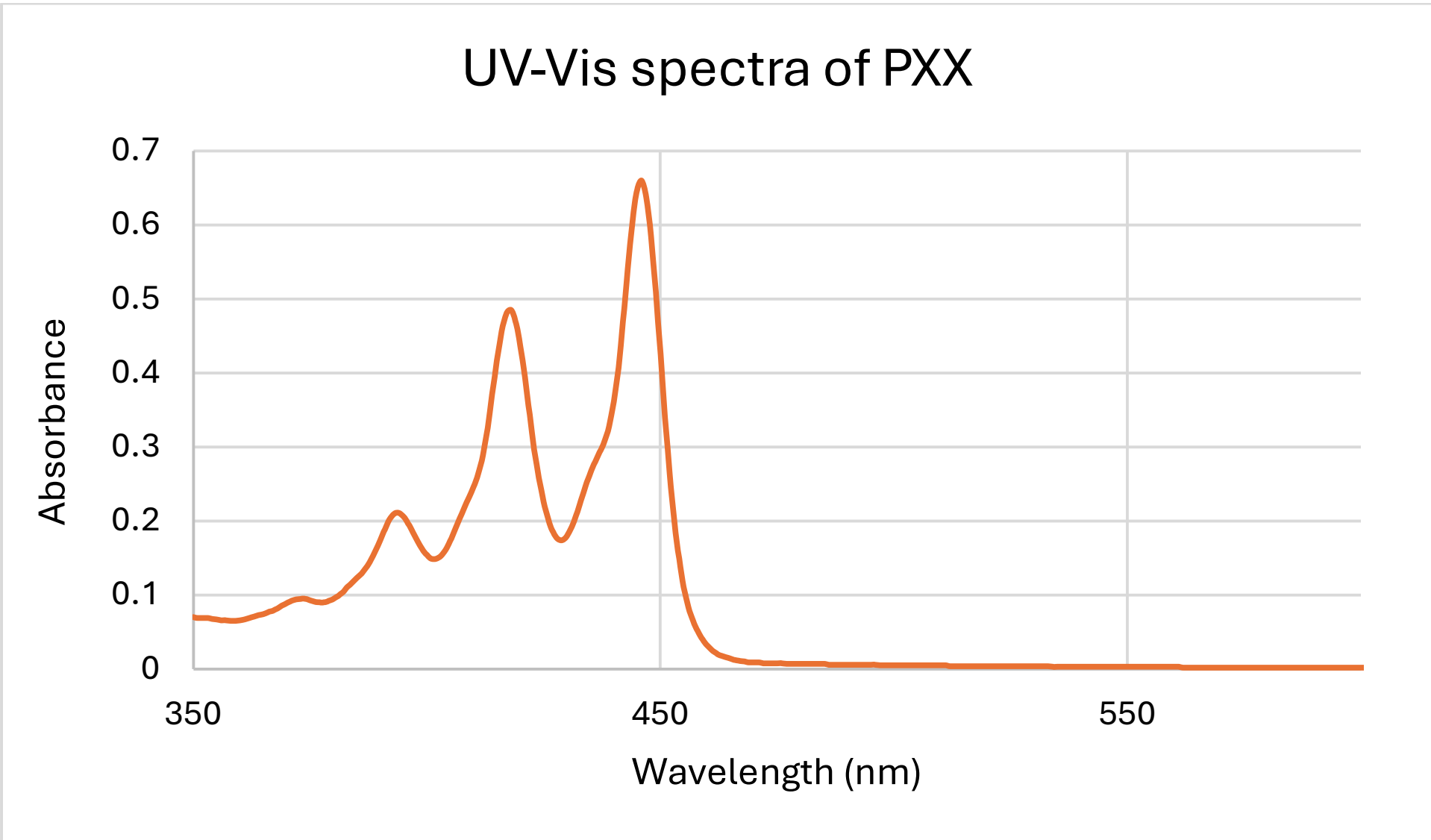

Figure S12: UV-Spectra of photocatalyst **PXX** in PhCl at 0.04 mM.

4.5.2 Quenching Experiments

Measurements recorded on an Edinburgh FS5 spectrofluorometer. A 1.0 cm (diameter) quartz cuvette (volume capacity ~3.50 mL) was added a 40 µM solution of **PXX** (3.0 mL) in degassed PhCl. Sequential addition (30 µL) of the appropriate quencher (1000 µM) (**[Co-1]**, **1aa**, **Lut·HOTf**) in degassed PhCl was added. The fluorescence intensity (I) was then measured using the fluorescence max of **PXX** (λ = 482 nm, Scheme S13) after excitation at λ = 410 nm.

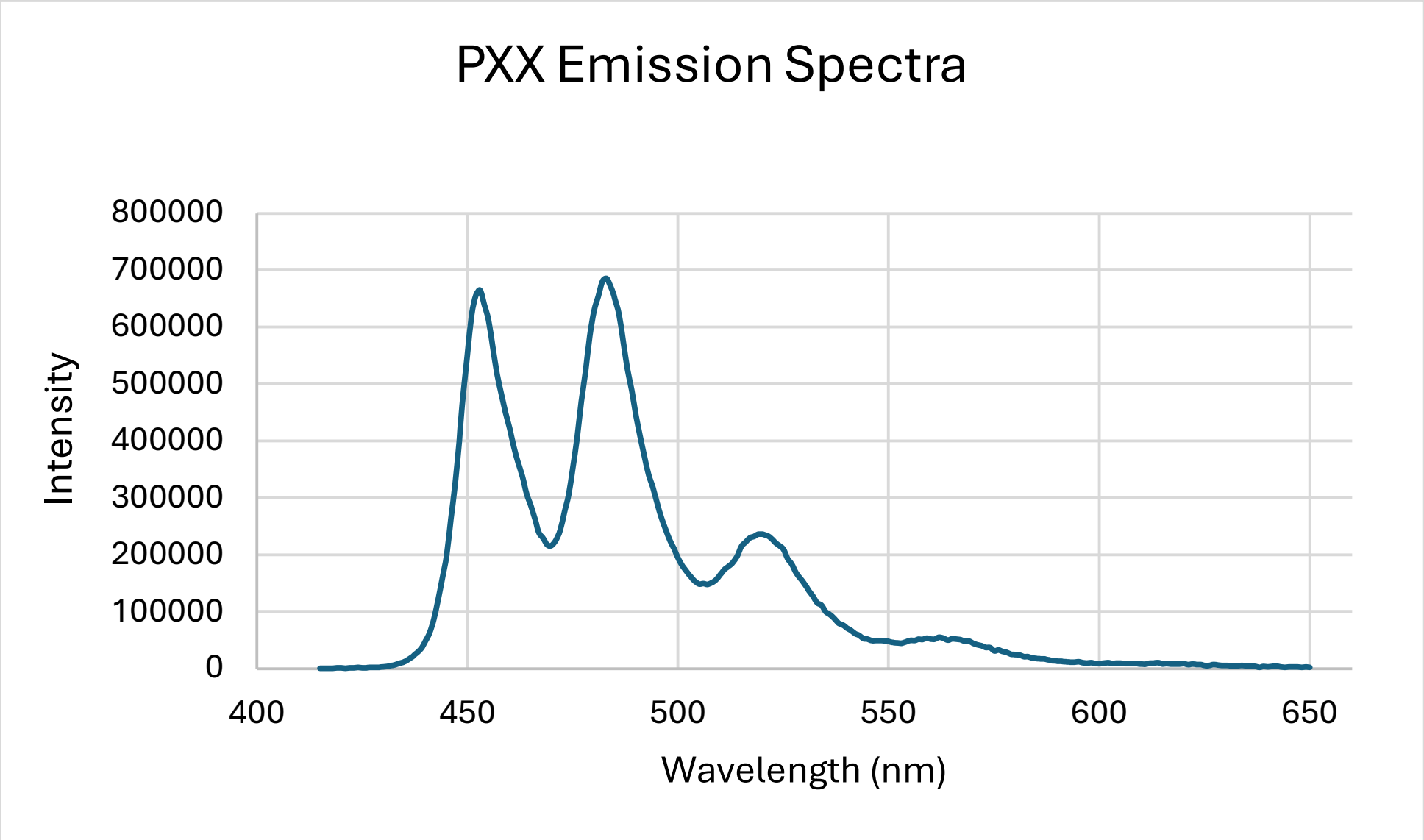

Figure S13: Emission spectra of **PXX** in PhCl.

Table 13: Quencher intensity

Quencher Concentration / µM	I_0/I		
	[Co-1]	1aa	Lut·HOTf
9.9	1.106	/	/
19.6	1.504	1.026	1.038
29.1	2.003	/	/
38.5	2.673	1.025	1.067
47.6	3.508	/	/
56.6	4.648	1.054	1.091
65.4	6.011	/	/
74.1	7.860	1.068	1.116
82.6	10.332	/	/
90.9	13.029	/	/

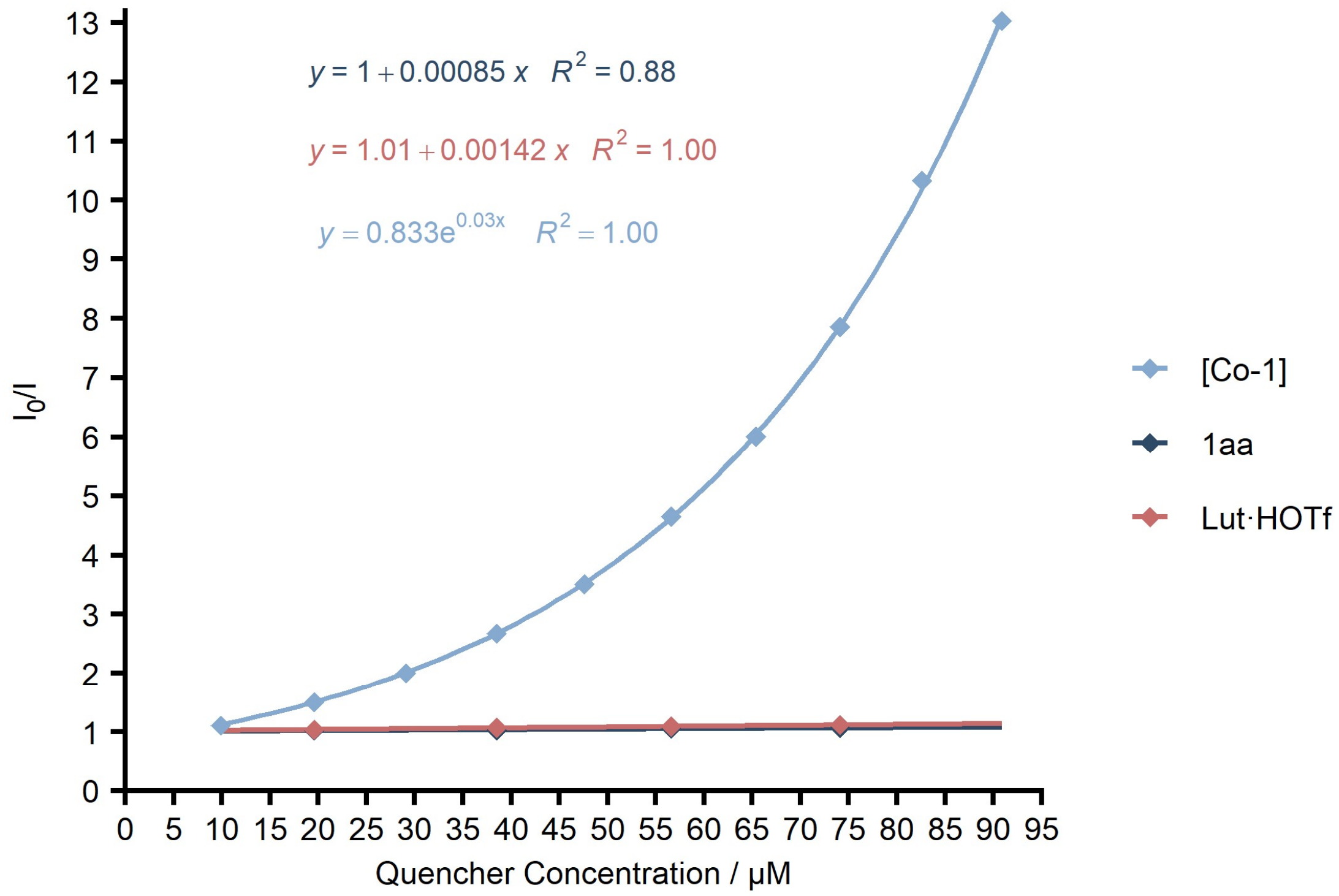

Figure S14: Stern-Volmer plot of **[Co-1]**, **1aa**, and **Lut·HOTf**.

Stern-Volmer experiments indicate the quencher of the excited state photocatalysts (**PXX***) to be the cobalt-catalyst (**[Co-1]**) and not the alkene substrate (**1aa**) or Brønsted acid (**Lut·HOTf**).

4.5.3 Inner-Filter-Effect

[Co-1] has broad absorption in the visible light region (Figure S11), including at the excitation wavelength (λ = 410 nm) used for the fluorescence measurements. To account for competitive absorption of the excitation light by **[Co-1]**, a correction for the primary inner-filter effect was applied to I_0/I using the following formula.[10–12] The absorbance values used in the correction were measured at 410 nm.

$$\frac{I_0}{I}corr = \frac{I_0}{I} * \frac{Abs(PC)}{Abs(PC) + Abs\ ([Co])} * \frac{1 - 10^{-(Abs(PC)+Abs([Co]))}}{1 - 10^{-Abs(PC)}}$$

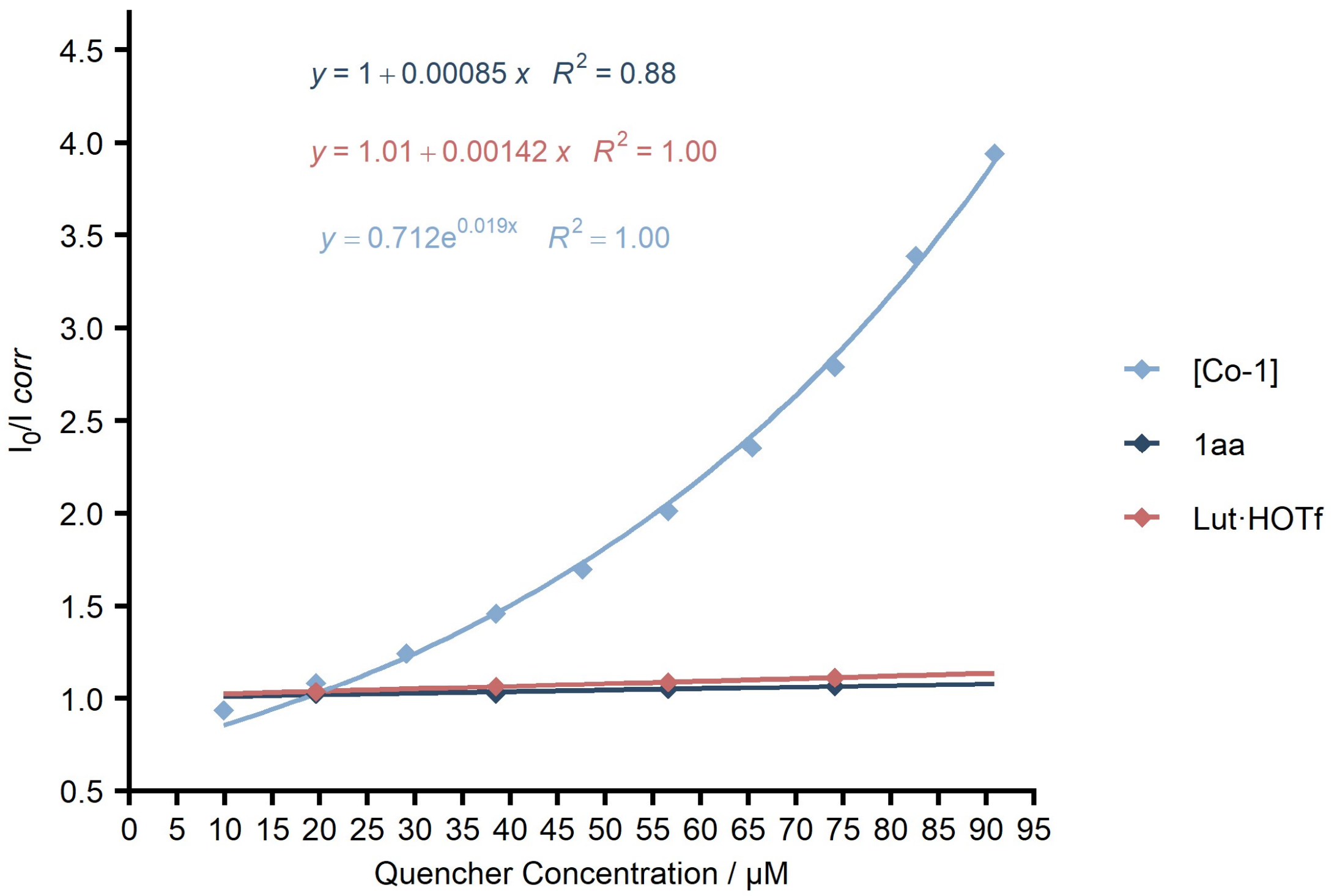

Figure S15: Stern-Volmer plot after correcting for inner-filter effects of **[Co-1]**.

Application of this correction did not remove the positive curvature of the Stern-Volmer plot. However, **[Co-1]** also absorbs at the PXX emission wavelength (λ = 482 nm), introducing a secondary inner-filter effect that is not accounted for by this correction.

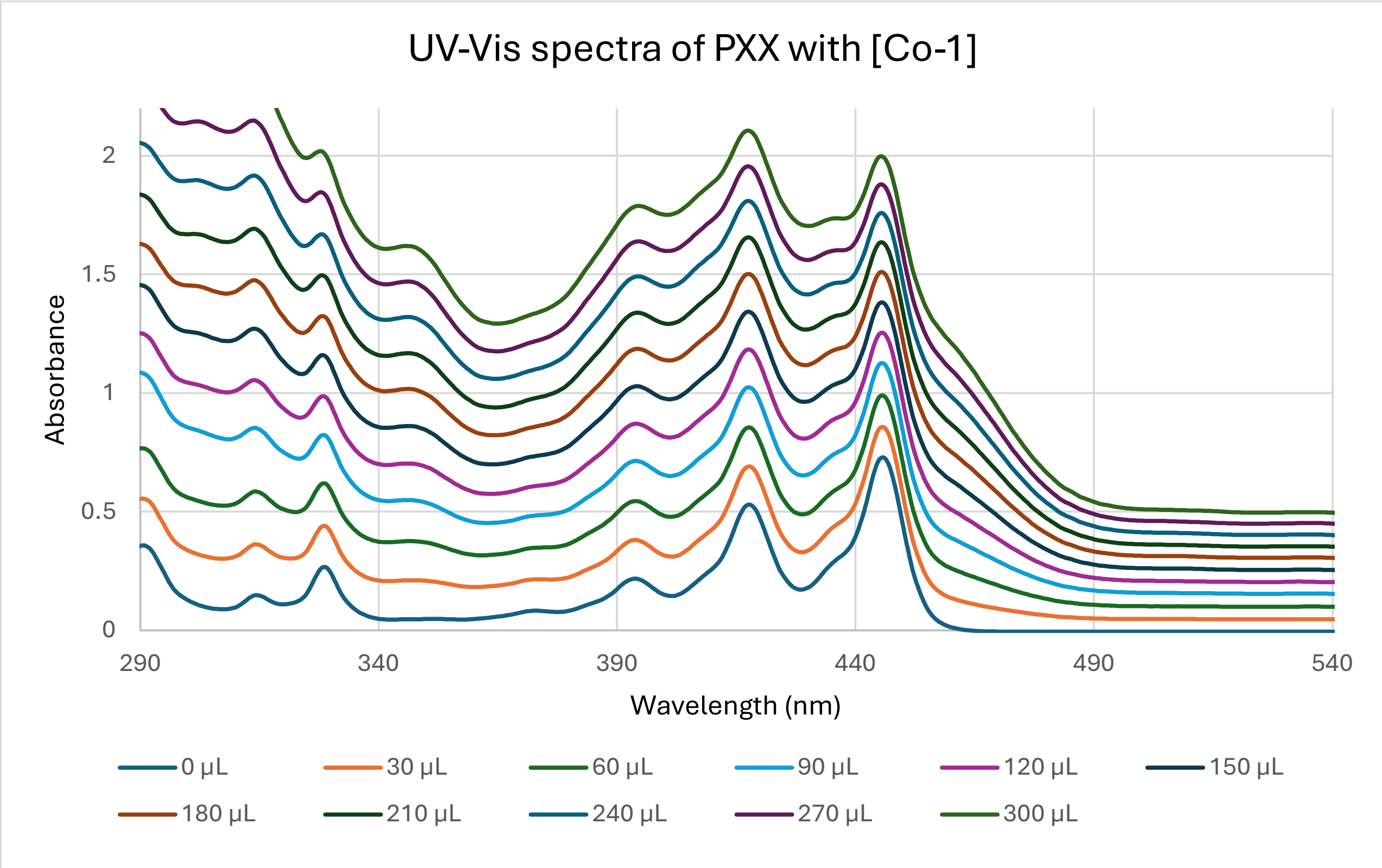

Figure S16: UV-Spectra of **PXX** (40 µM, in PhCl) with sequential additions (30 µL) of a **[Co-1]** (1000 µM, in PhCl) solution.

The UV–Vis spectra of **PXX**/**[Co-1]** mixtures showed no new absorption features beyond the superposition of the individual spectra, providing no indication of a ground-state complex, although a static contribution cannot be excluded based on this alone.

4.5.4 Low concentration Stern-Volmer for [Co-1]

Stern-Volmer measurements were repeated for **[Co-1]** at lower quencher concentrations to observe if the positive deviation from linearity still occurs. Measurements recorded on an Edinburgh FS5 spectrofluorometer. A 1.0 cm (diameter) quartz cuvette (volume capacity ~3.50 mL) was added a 40 µM solution of **PXX** (3.0 mL) in degassed PhCl. Sequential addition (10 µL) of **[Co-1]** (1000 µM) in degassed PhCl was added. The fluorescence intensity (I) was then measured using the fluorescence max of **PXX** (λ = 482 nm, Scheme S13) after excitation at λ = 410 nm.

Table 14: Quencher intensity

Quencher Concentration / µM	I_0/I
	[Co-1]
3.32	1.130
6.62	1.242
13.16	1.498
16.39	1.681
22.80	2.038
25.97	2.264

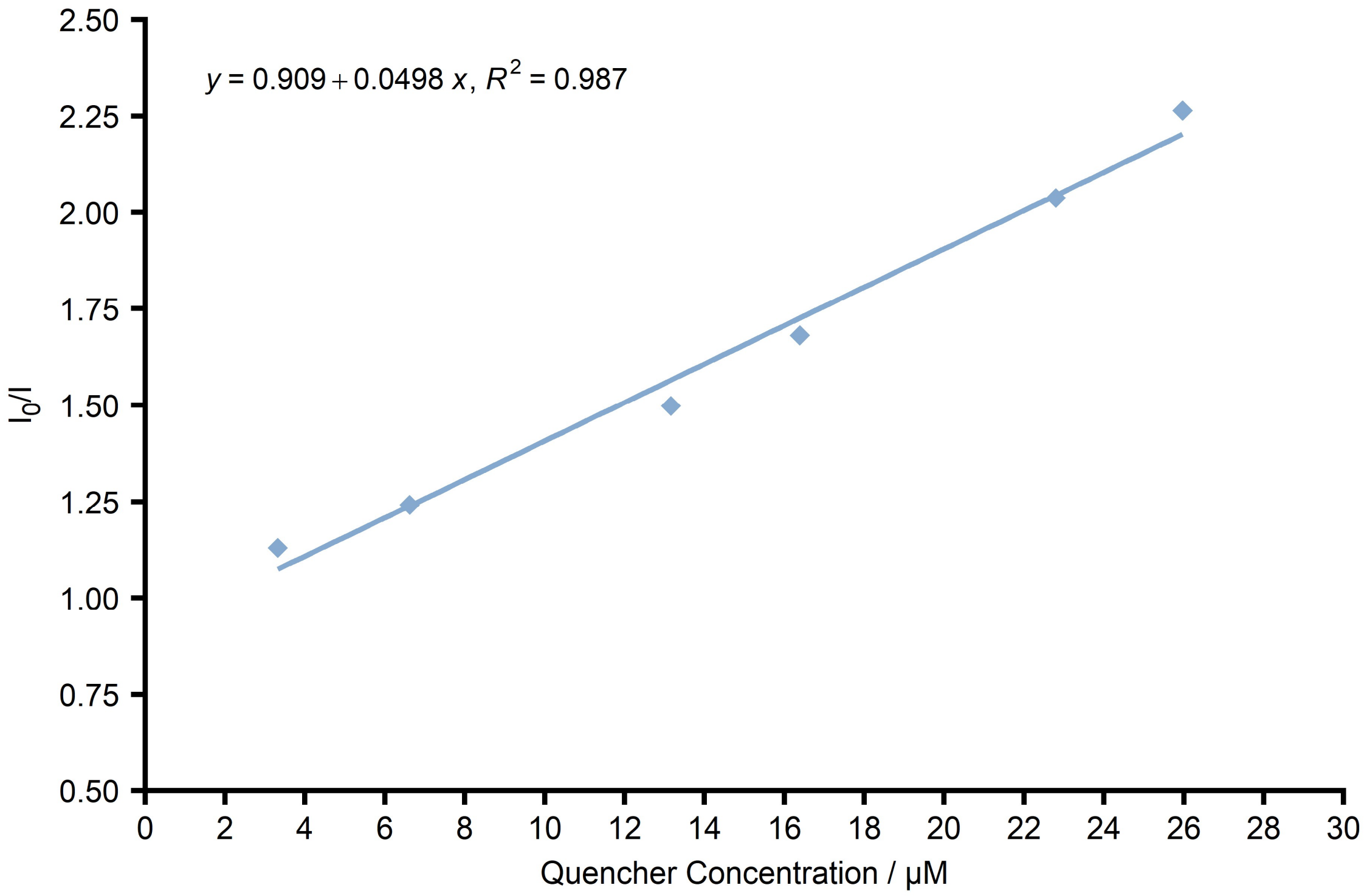

Figure S17: Stern-Volmer plot at lower concentrations of **[Co-1]** with a linear fit.

At lower [**Co-1**] concentrations, the data is reasonably approximated by a linear fit; however, this may simply reflect examination of the approximately linear region of a broader nonlinear response.

4.6 Cobalt-Catalyst Control Experiments

4.6.1 Diastereocontrol of 2ba

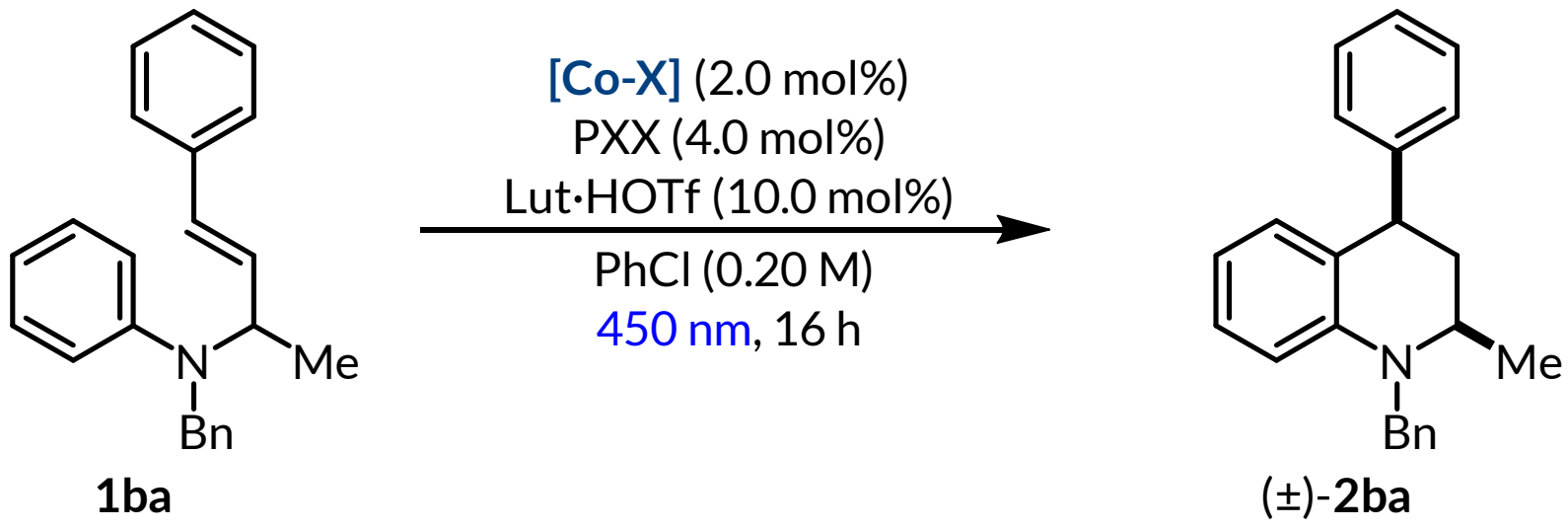

Table S15: Catalyst Dependent Diastereocontrol

Entry	[Co]-catalyst	^{1}H NMR Yield of (±)-2ba / %	dr (*syn major*)
1	[Co-1]	81	1.8:1.0
2	(±)-[Co-2]	84	1.1:1.0
3	(±)-[Co-3]	61	1.7:1.0

4.6.2 Enantioselective Example of 2aa

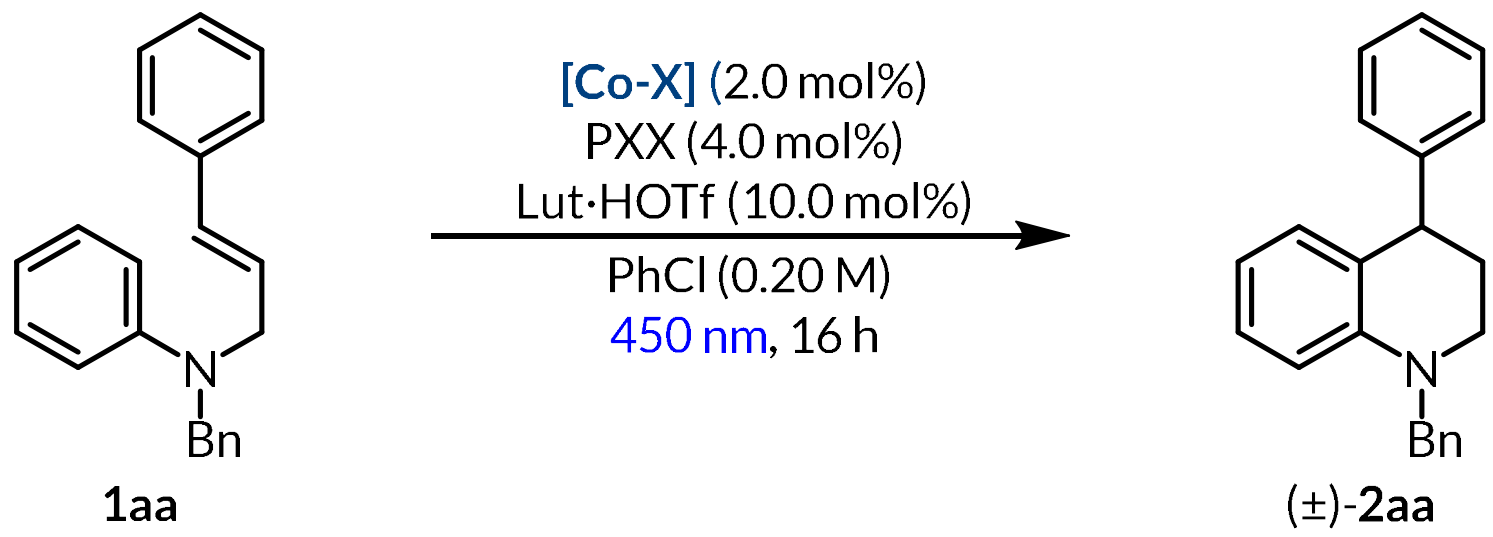

Table S16: Enantioselective attempts

Entry	[Co]-catalyst	^{1}H NMR Yield of 2aa / %	er of 2aa
1	(*R*,*R*)-[Co-2]	91	53:47
2	(*R*,*R*)-[Co-3]	82	49:51
3	(*S*,*S*)-[Co-4]	57	45:55
4	(*S*,*S*)-[Co-5]	65	44:56
5	(*S*,*S*)-[Co-6]	63	46:54

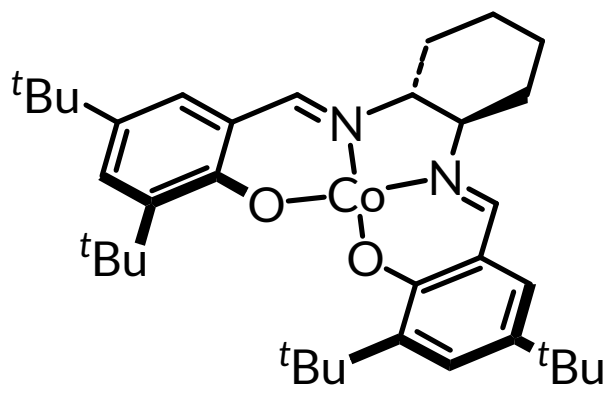

(*R*,*R*)-[Co-2]

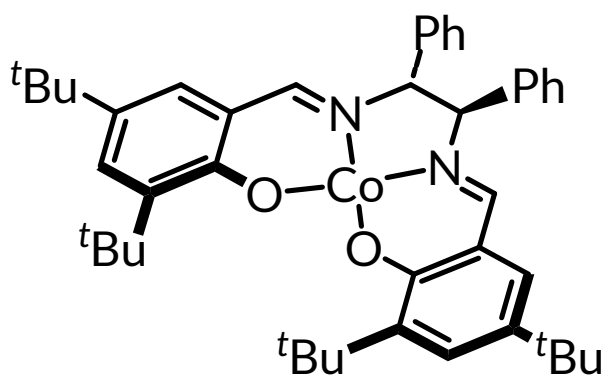

(*R*,*R*)-[Co-3]

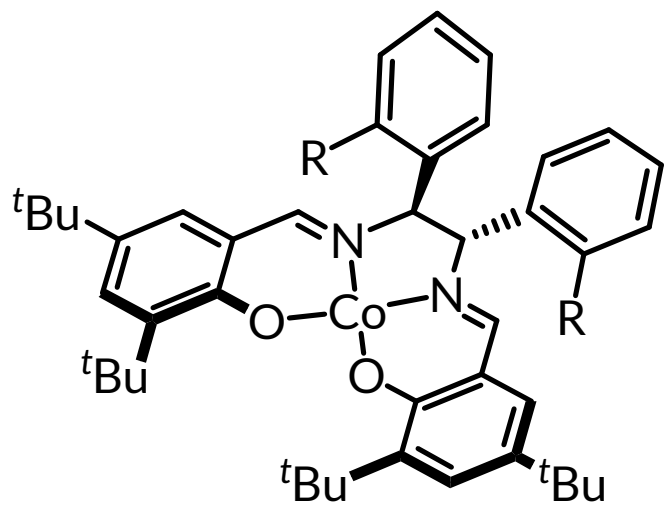

(*S*,*S*)-[Co-4], R = Phenyl
(*S*,*S*)-[Co-5], R = 2-Naphthyl
(*S*,*S*)-[Co-6], R = 4-Dibenzofuran

5. References

[1] L. P. C. Nielsen, C. P. Stevenson, D. G. Blackmond, E. N. Jacobsen, "Mechanistic Investigation Leads to a Synthetic Improvement in the Hydrolytic Kinetic Resolution of Terminal Epoxides" *J. Am. Chem. Soc.* **2004**, *126*, 1360–1362.

[2] C. Pezzetta, A. Folli, O. Matuszewska, D. Murphy, R. W. M. Davidson, D. Bonifazi, "peri-Xanthenoxanthene (PXX): a Versatile Organic Photocatalyst in Organic Synthesis" *Adv. Synth. Catal.* **2021**, *363*, 4740–4753.

[3] J. J. Curley, R. G. Bergman, T. D. Tilley, "Preparation and physical properties of early-late heterobimetallic compounds featuring Ir–M bonds (M = Ti, Zr, Hf)" *Dalton Trans.* **2011**, *41*, 192–200.

[4] Y. Chen, L. Yang, H. Qiao, Z. Cheng, J. Xie, W. Zhou, X. Huang, Y. Jiang, B. Yu, W. Zhao, "Discovery of new thieno[3,2-*d*]pyrimidine derivatives targeting EGFRL858R/T790M NSCLCs by the conformation constrained strategy" *Eur. J. Med. Chem.* **2020**, *199*, 112388.

[5] L. Liu, H. Lu, H. Wang, C. Yang, X. Zhang, D. Zhang-Negrerie, Y. Du, K. Zhao, "PhI(OCOCF3)2-Mediated C–C Bond Formation Concomitant with a 1,2-Aryl Shift in a Metal-Free Synthesis of 3-Arylquinolin-2-ones" *Org. Lett.* **2013**, *15*, 2906–2909.

[6] X. Wu, J. Qu, Y. Chen, "Quinim: A New Ligand Scaffold Enables Nickel-Catalyzed Enantioselective Synthesis of α-Alkylated γ-Lactam" *J. Am. Chem. Soc.* **2020**, *142*, 15654–15660.

[7] K. Targos, O. P. Williams, Z. K. Wickens, "Unveiling Potent Photooxidation Behavior of Catalytic Photoreductants" *J. Am. Chem. Soc.* **2021**, *143*, 4125–4132.

[8] Y. Yamaguchi, Y. Seino, A. Suzuki, Y. Kamei, T. Yoshino, M. Kojima, S. Matsunaga, "Intramolecular Hydrogen Atom Transfer Hydroarylation of Alkenes toward δ-Lactams Using Cobalt-Photoredox Dual Catalysis" *Org. Lett.* **2022**, *24*, 2441–2445.

[9] Corwin. Hansch, A. Leo, R. W. Taft, "A survey of Hammett substituent constants and resonance and field parameters" *Chem. Rev.* **1991**, *91*, 165–195.

[10] H. Zhou, D. Lunic, N. Sanosa, D. Sampedro, I. Funes-Ardoiz, C. J. Teskey, "Merging Hydrogen-Atom-Transfer and the Truce-Smiles Rearrangement for Synthesis of β-Arylethylamines from Unactivated Allylsulfonamides" *Angew. Chem. Int. Ed.* **2025**, *64*, e202418869.

[11] D. Lunic, N. Vystavkin, J. Qin, C. J. Teskey, "Dual-Catalytic Structural Isomerisation as a Route to α-Arylated Ketones" *Angew. Chem. Int. Ed.* **2024**, *63*, e202409388.

[12] M. Nakagawa, Y. Matsuki, K. Nagao, H. Ohmiya, "A Triple Photoredox/Cobalt/Brønsted Acid Catalysis Enabling Markovnikov Hydroalkoxylation of Unactivated Alkenes" *J. Am. Chem. Soc.* **2022**, *144*, 7953–7959.

6. Spectra

6,6'-((1*E*,1'*E*)-(1,2-Phenylenebis(azaneylylidene))bis(methaneylylidene))bis(2,4-di-*tert*-butylphenol) (L1)

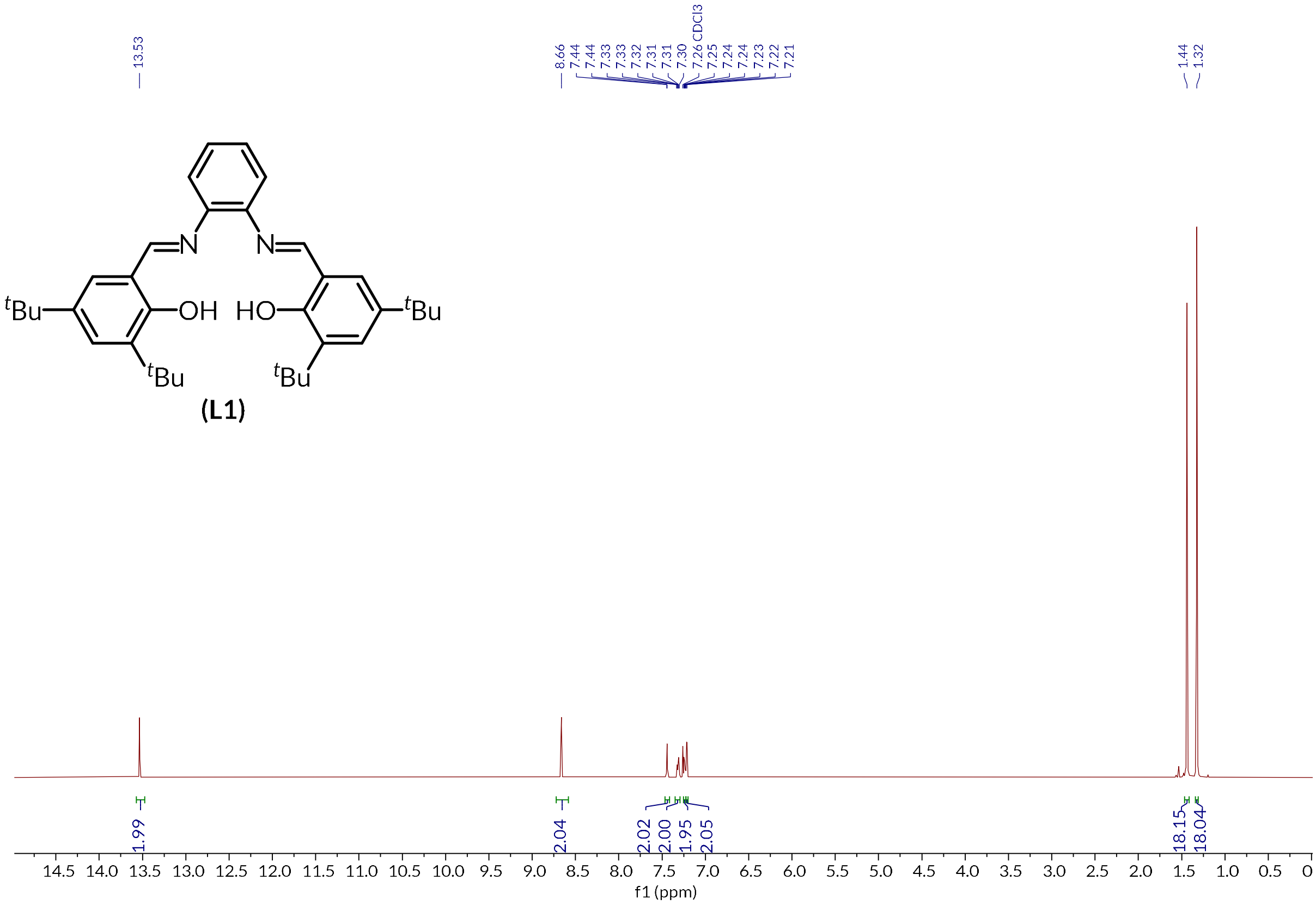

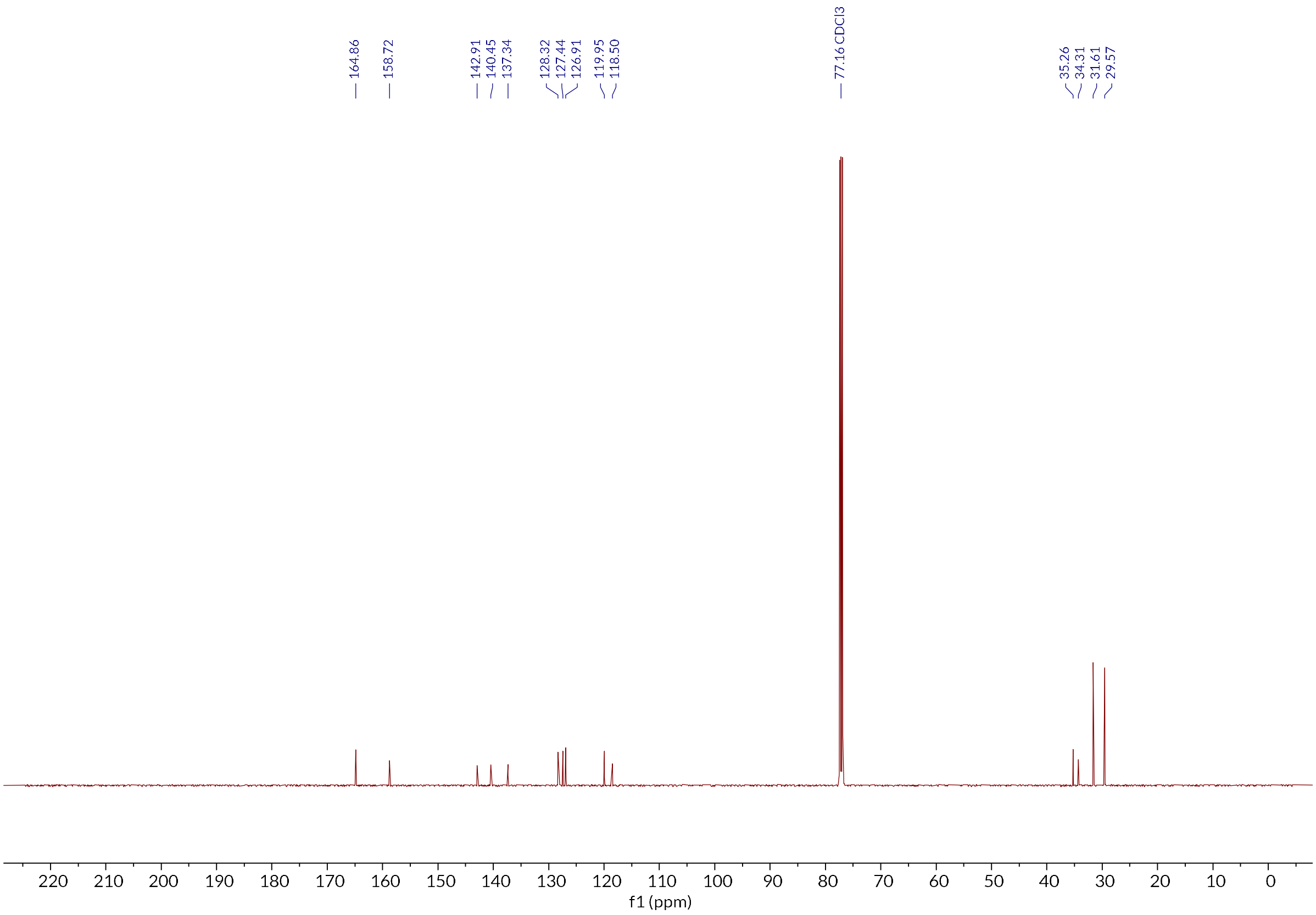

peri-Xanthenoxanthene (PXX)

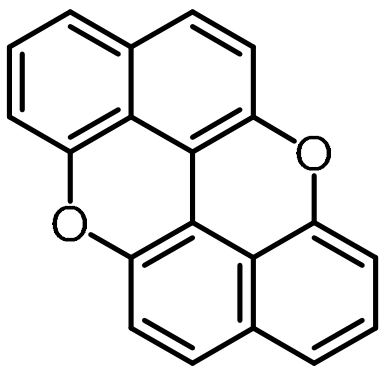

(PXX)

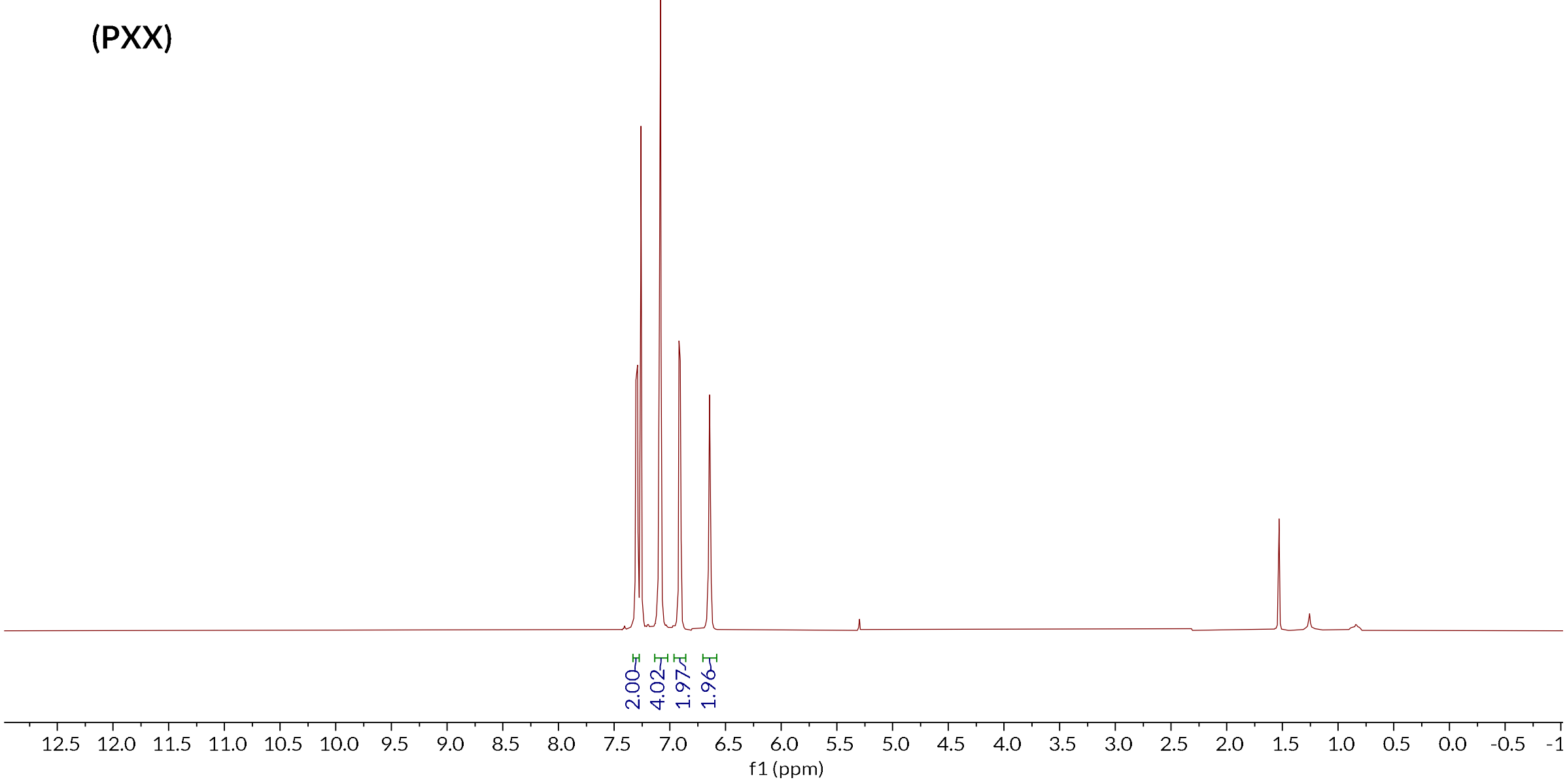

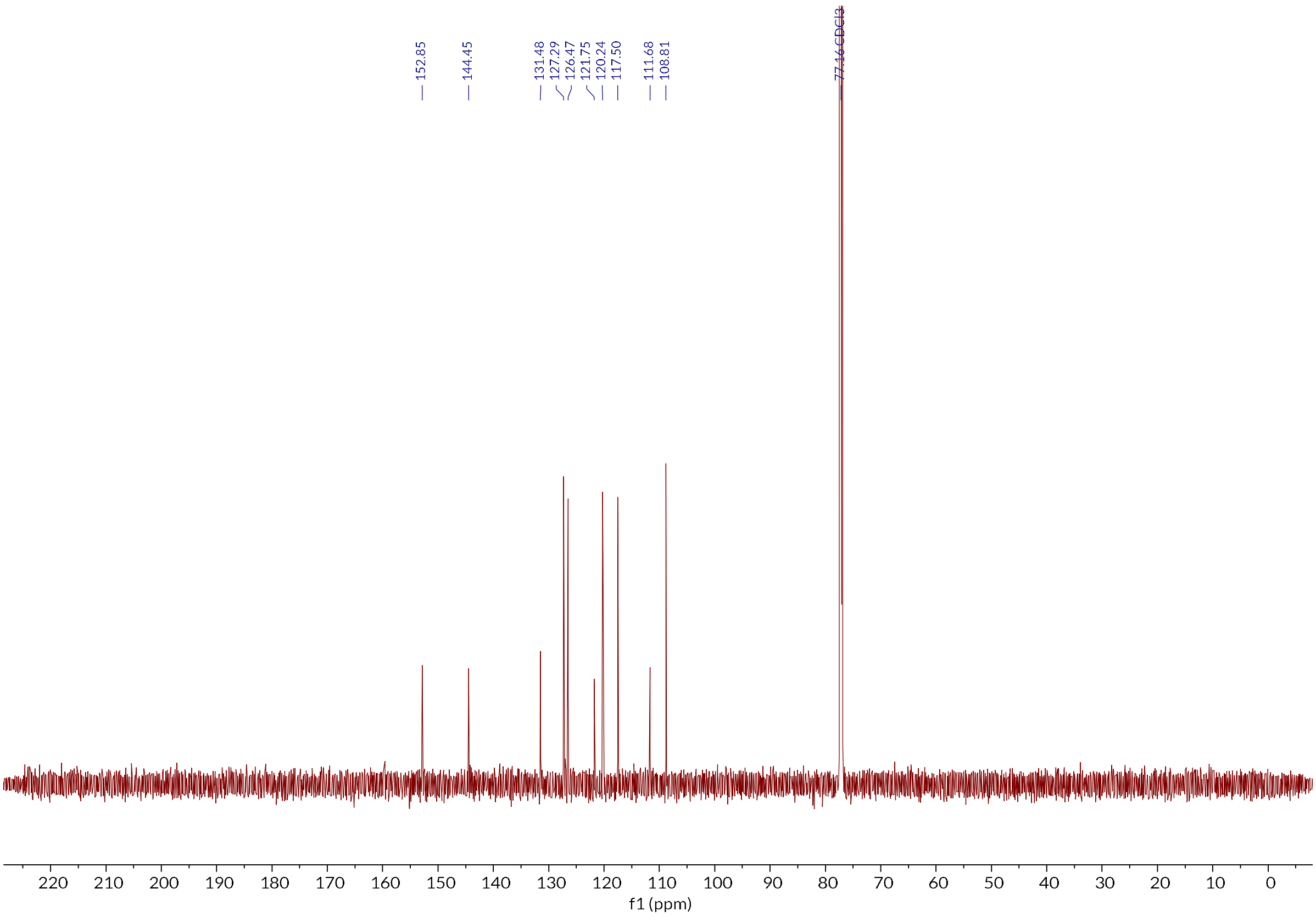

2,6-Dimethylpyridin-1-ium trifluoromethanesulfonate (Lut·HOTf)

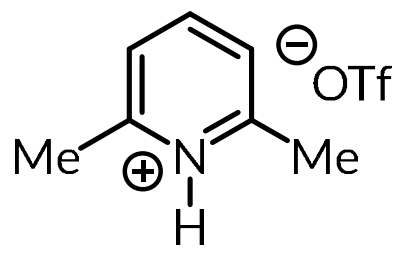

(Lut·HOTf)

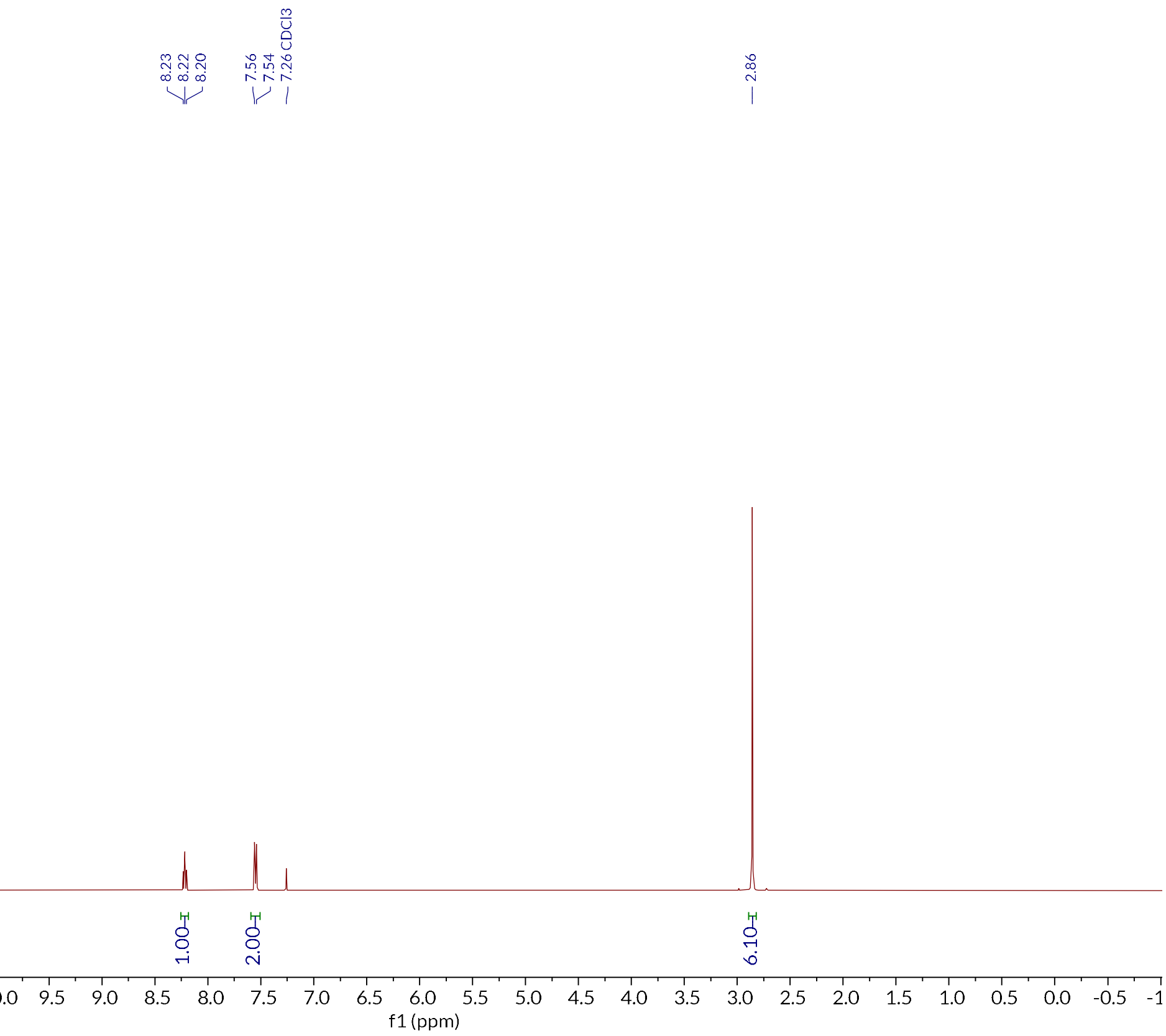

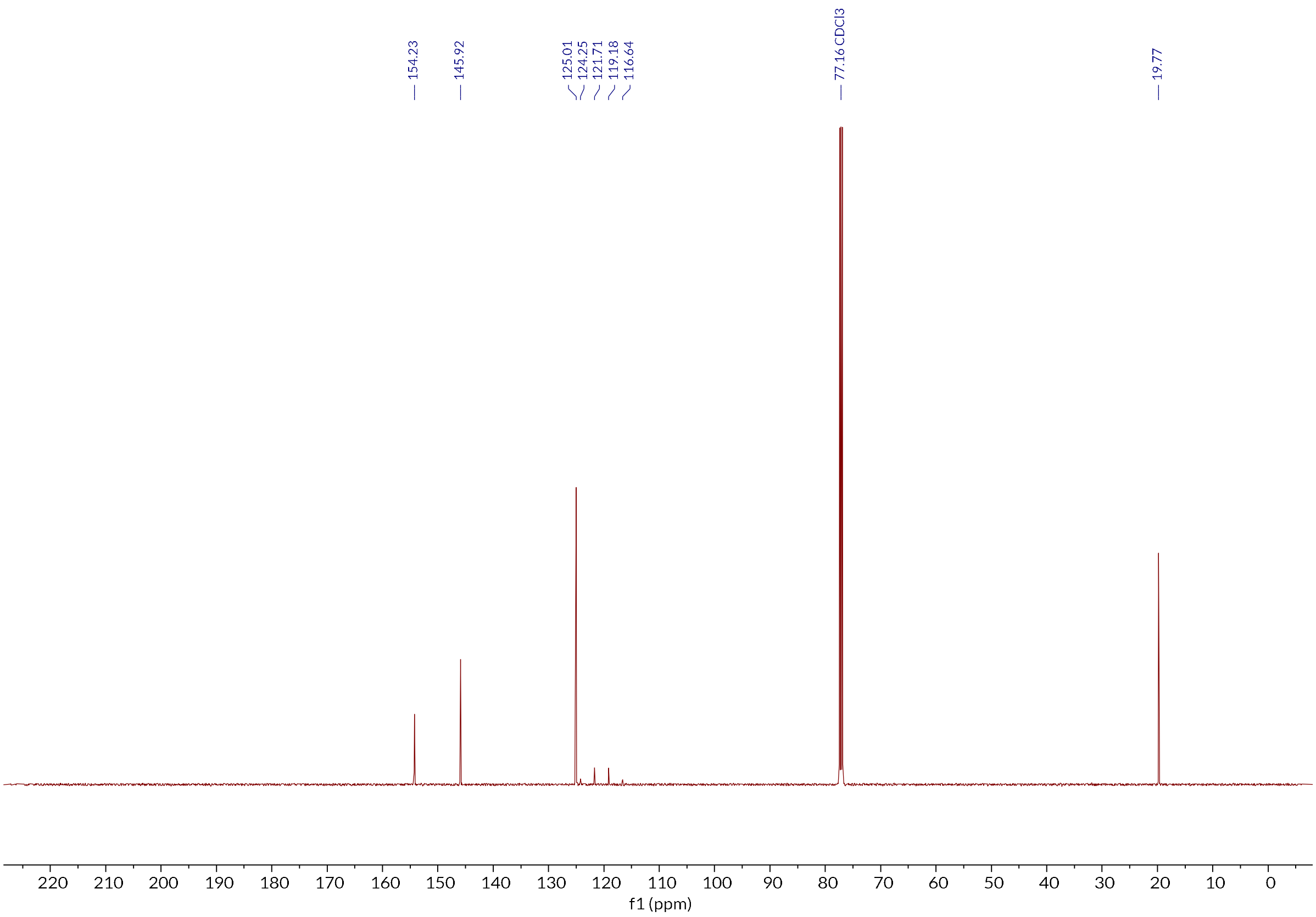

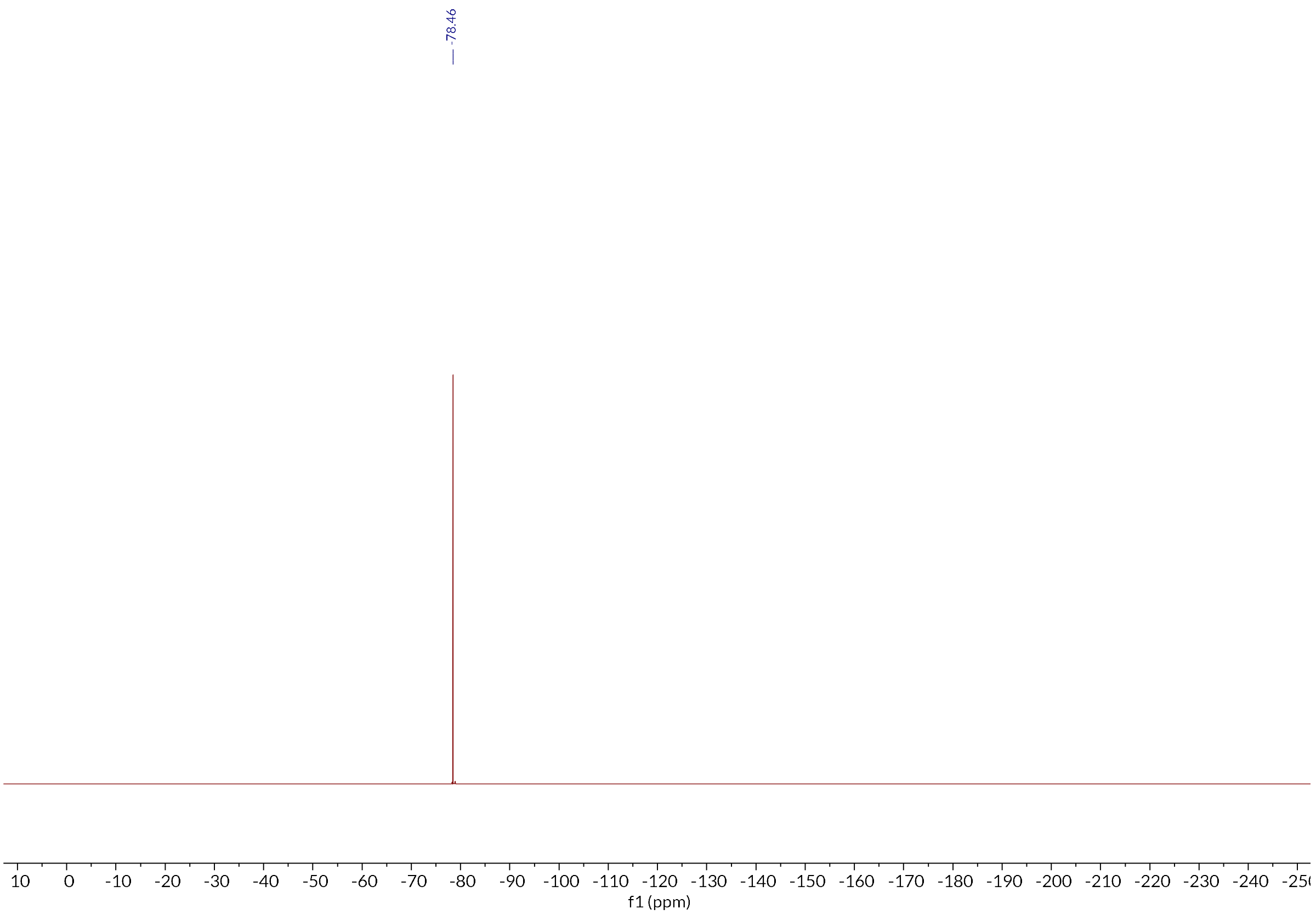
-78.46
10 0 -10 -20 -30 -40 -50 -60 -70 -80 -90 -100 -110 -120 -130 -140 -150 -160 -170 -180 -190 -200 -210 -220 -230 -240 -250
f1 (ppm)

N-Cinnamylaniline (3a)

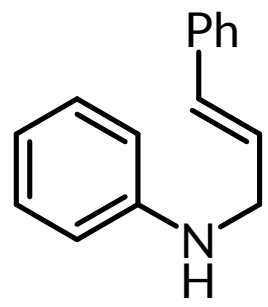

(3a)

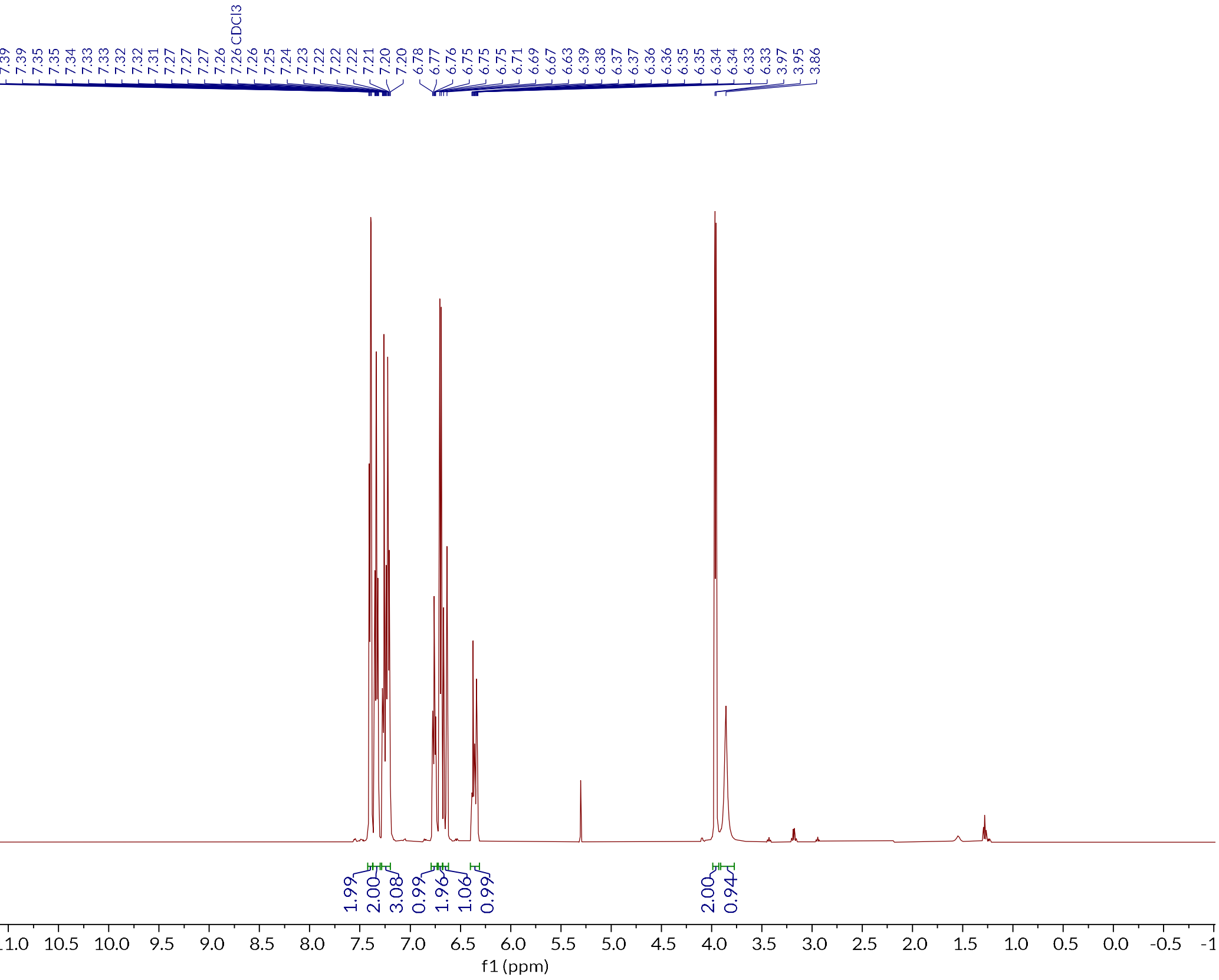

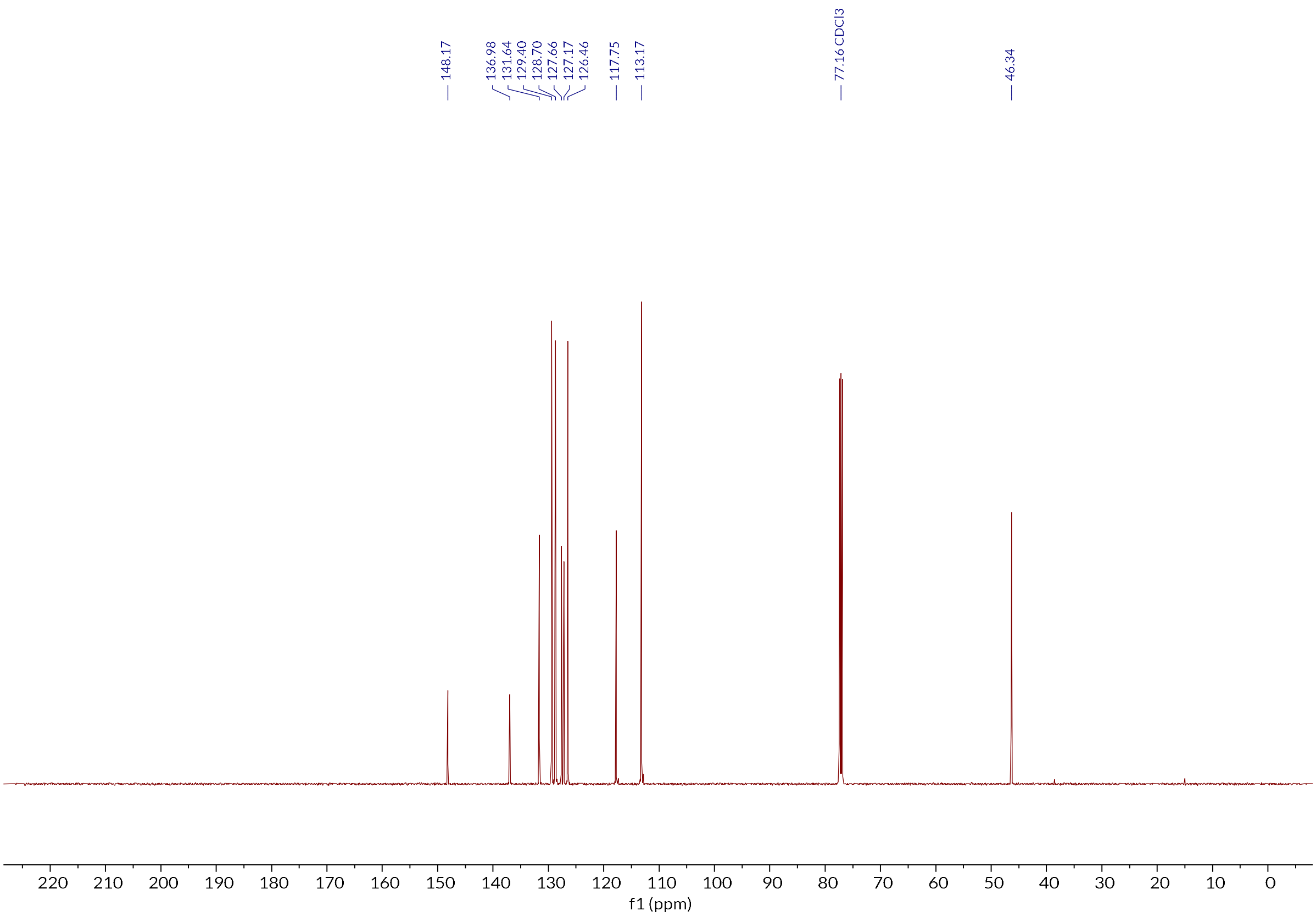

N-Benzyl-*N*-cinnamylaniline (1aa)

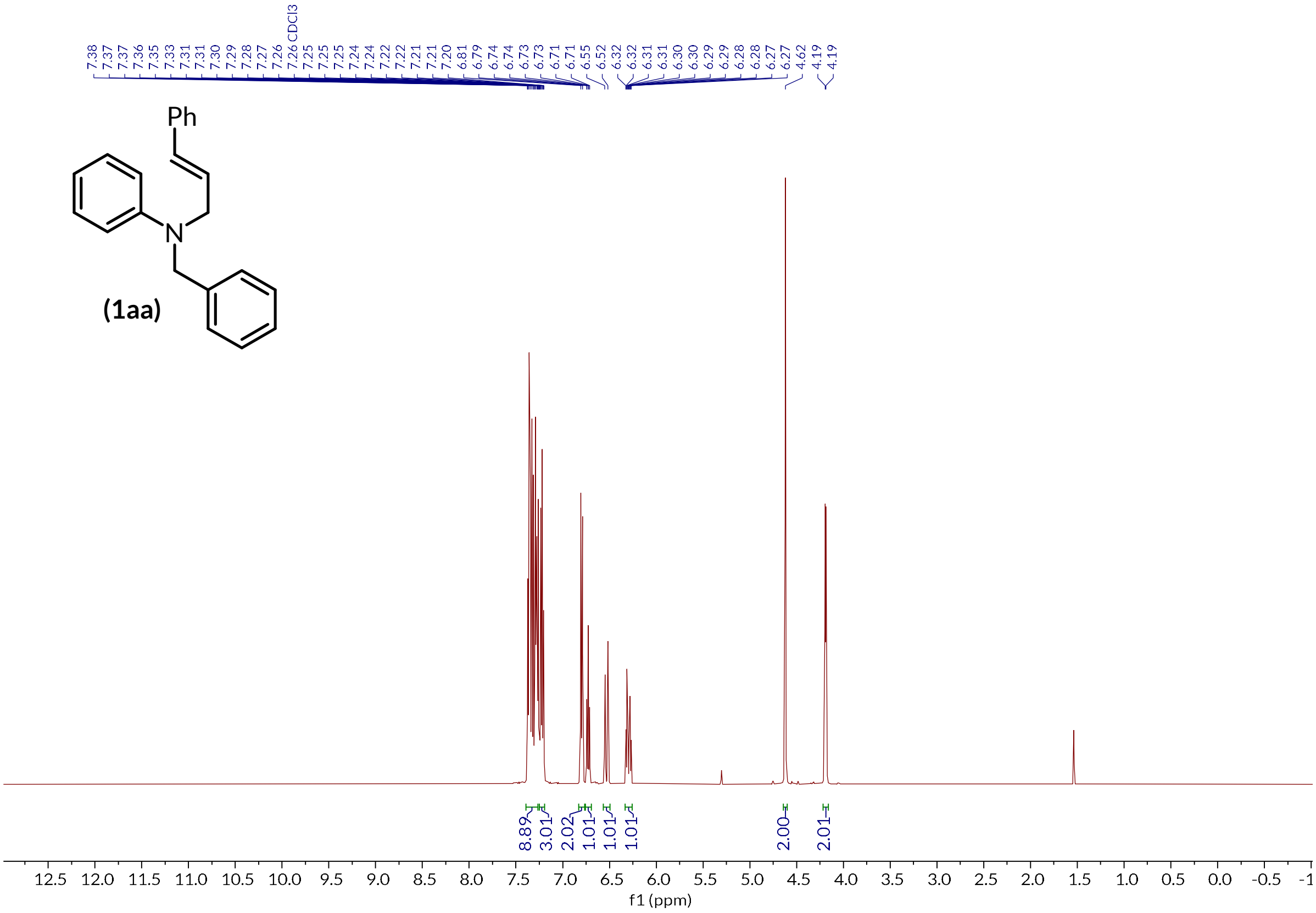

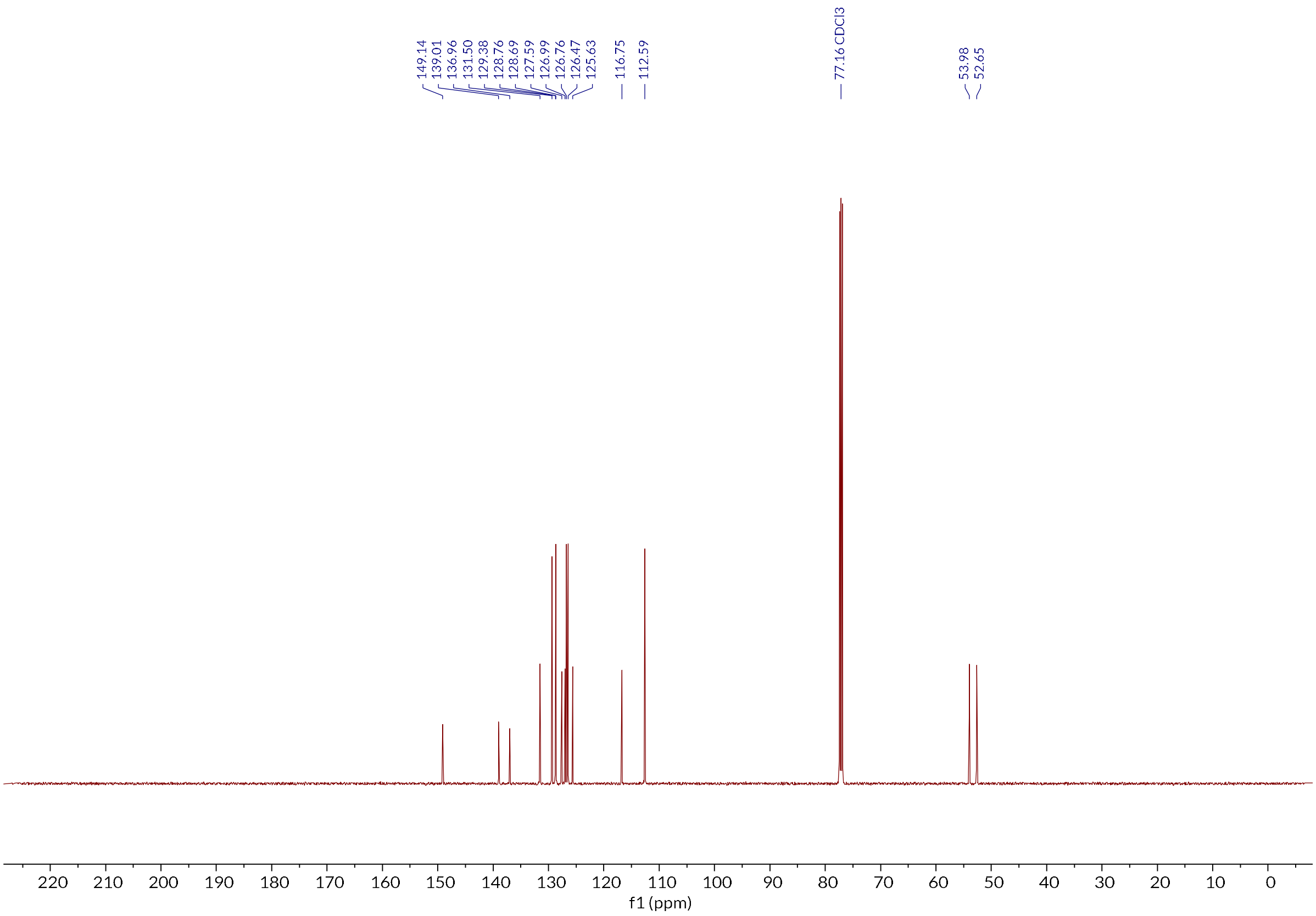

N-(4-Methoxybenzyl)aniline (SI-01)

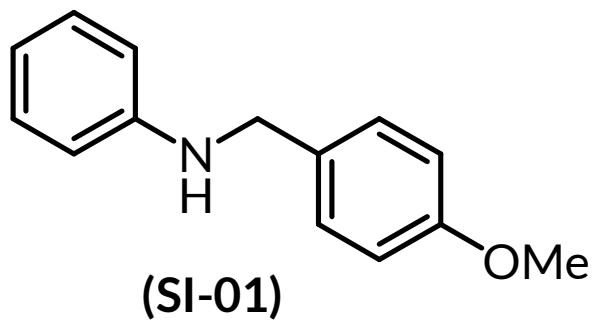

(SI-01)

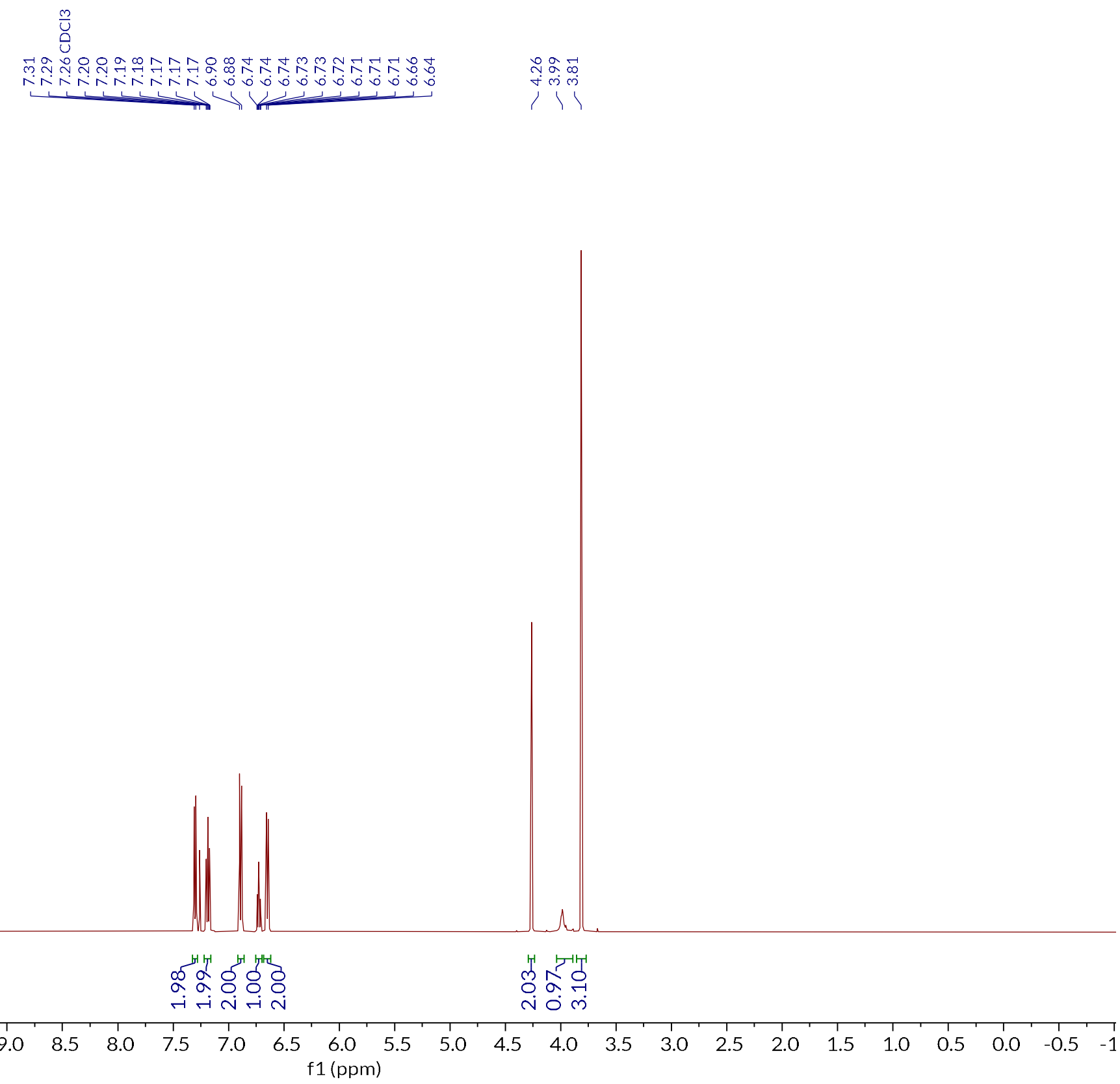

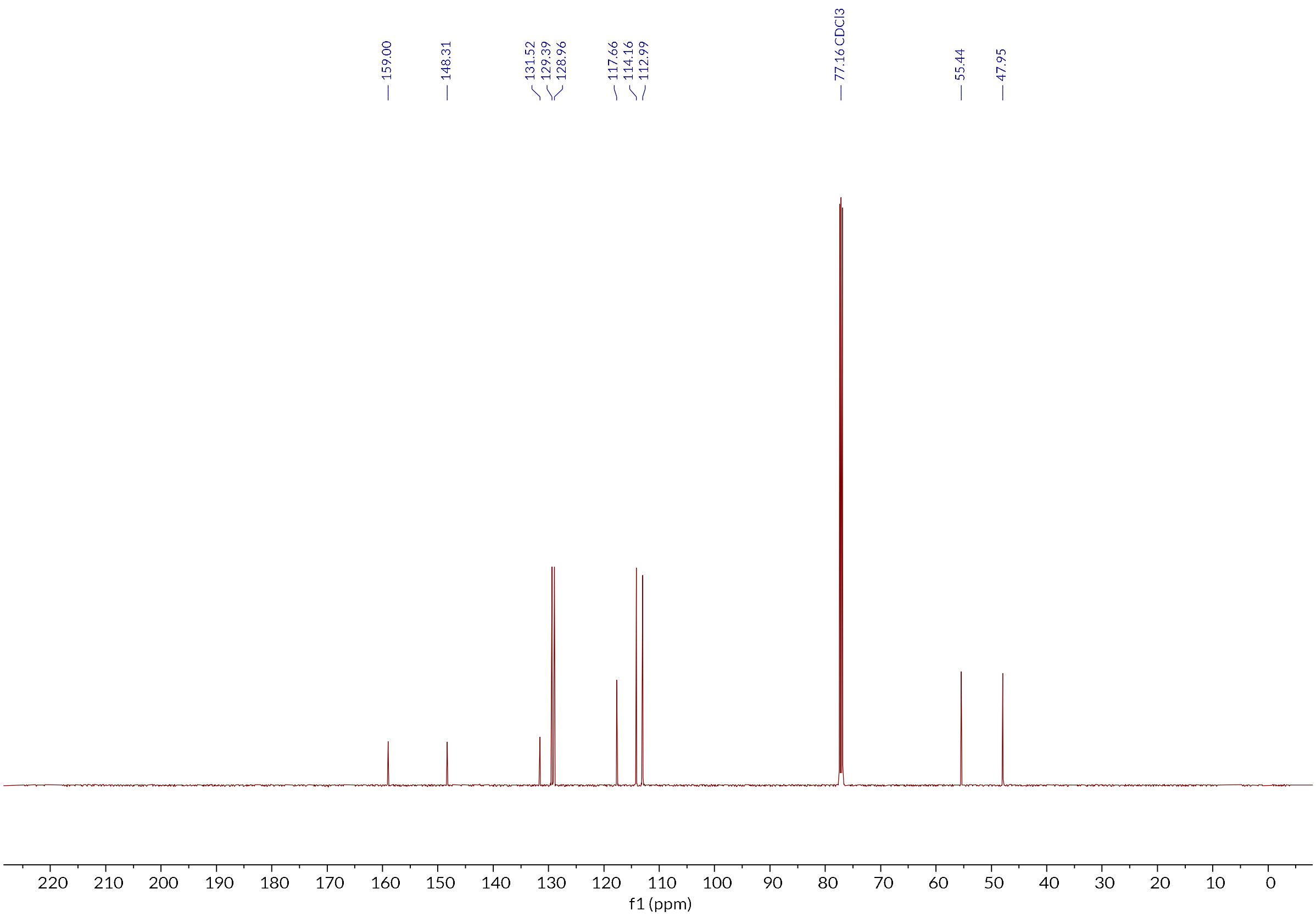

N-Cinnamyl-*N*-(4-methoxybenzyl)aniline (1ab)

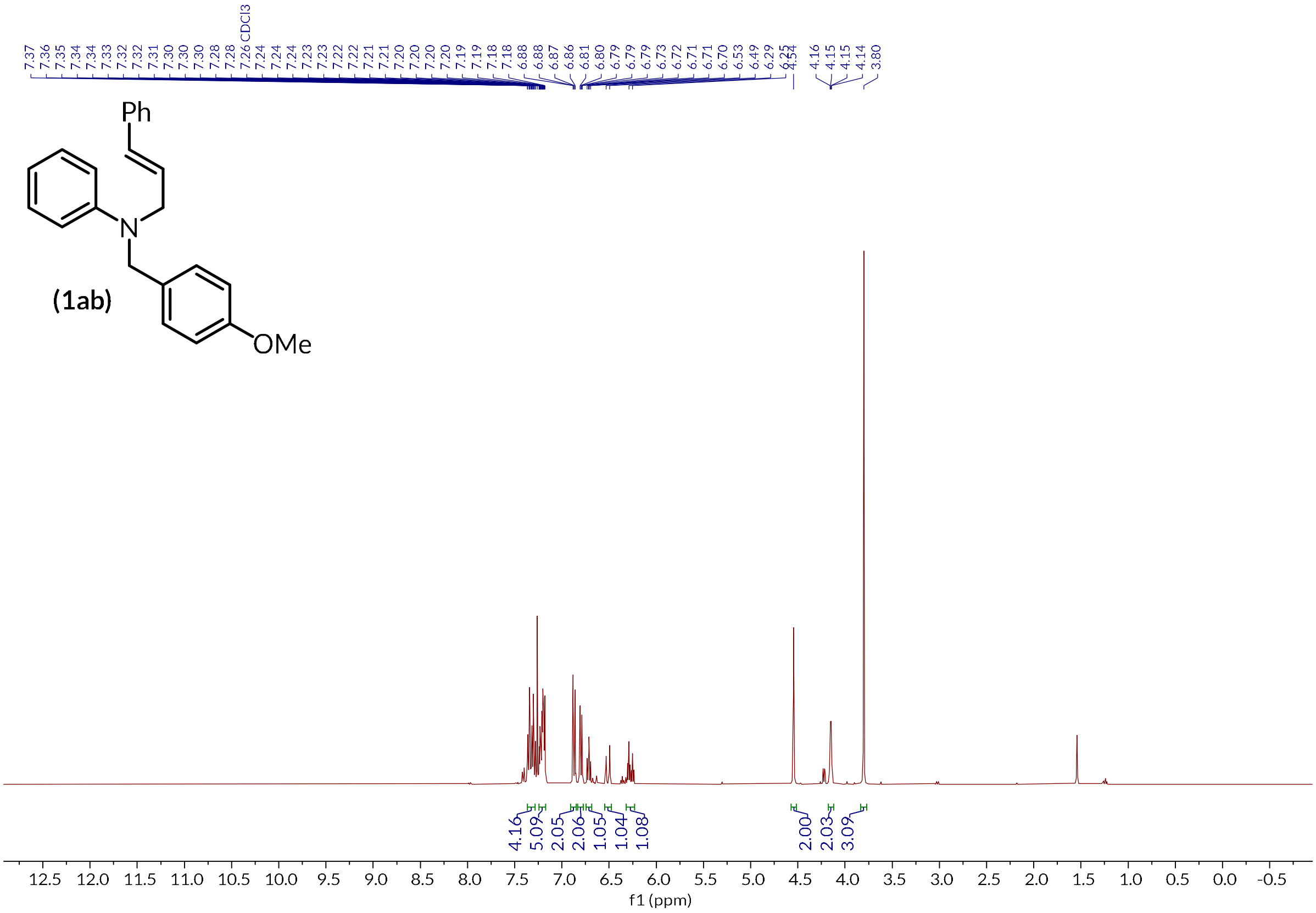

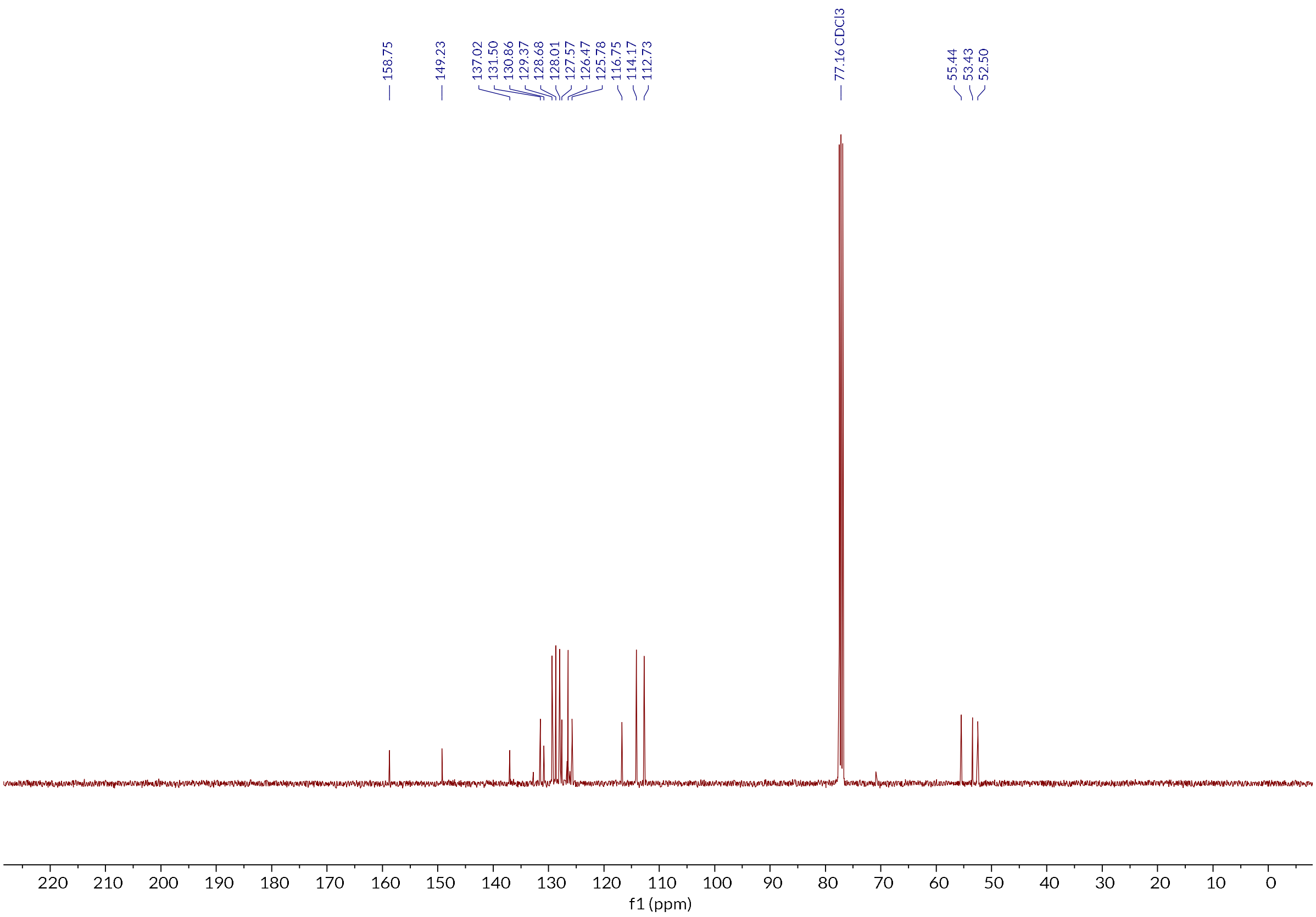

N-(4-Fluorobenzyl)aniline (SI-02)

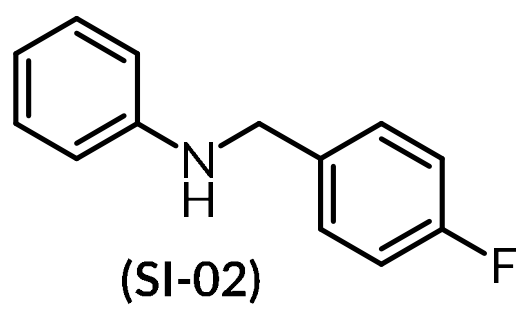

(SI-02)

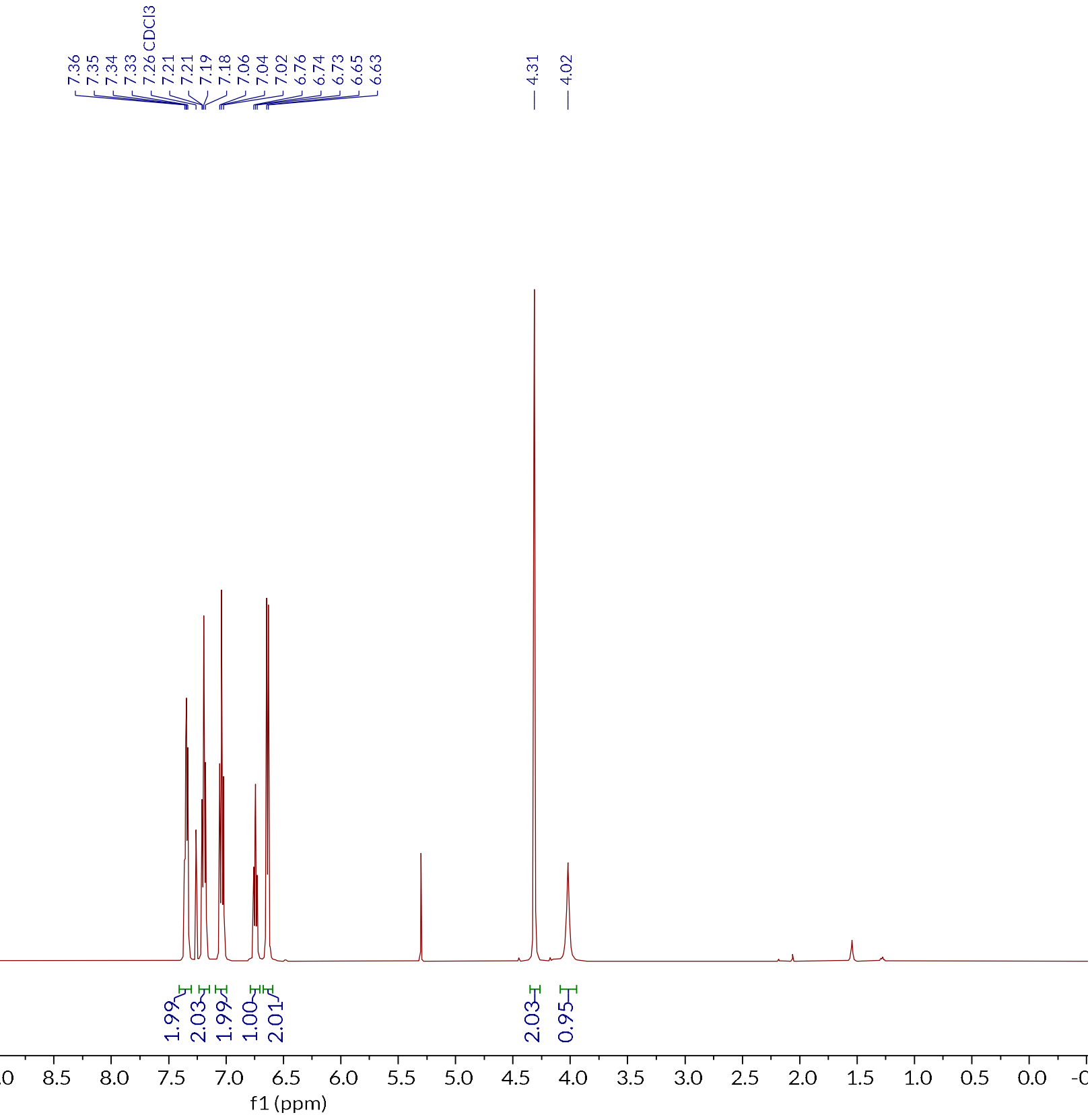

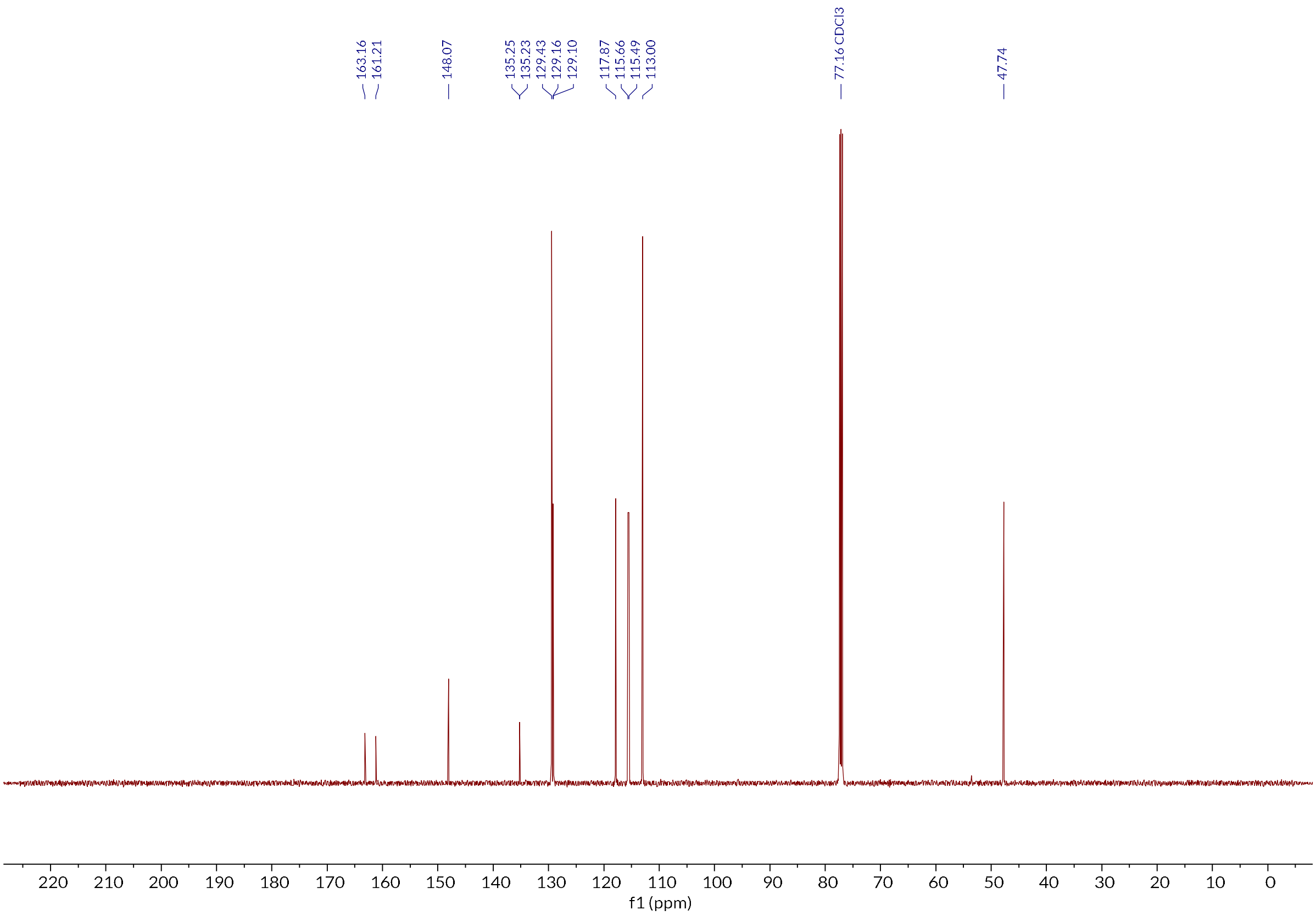

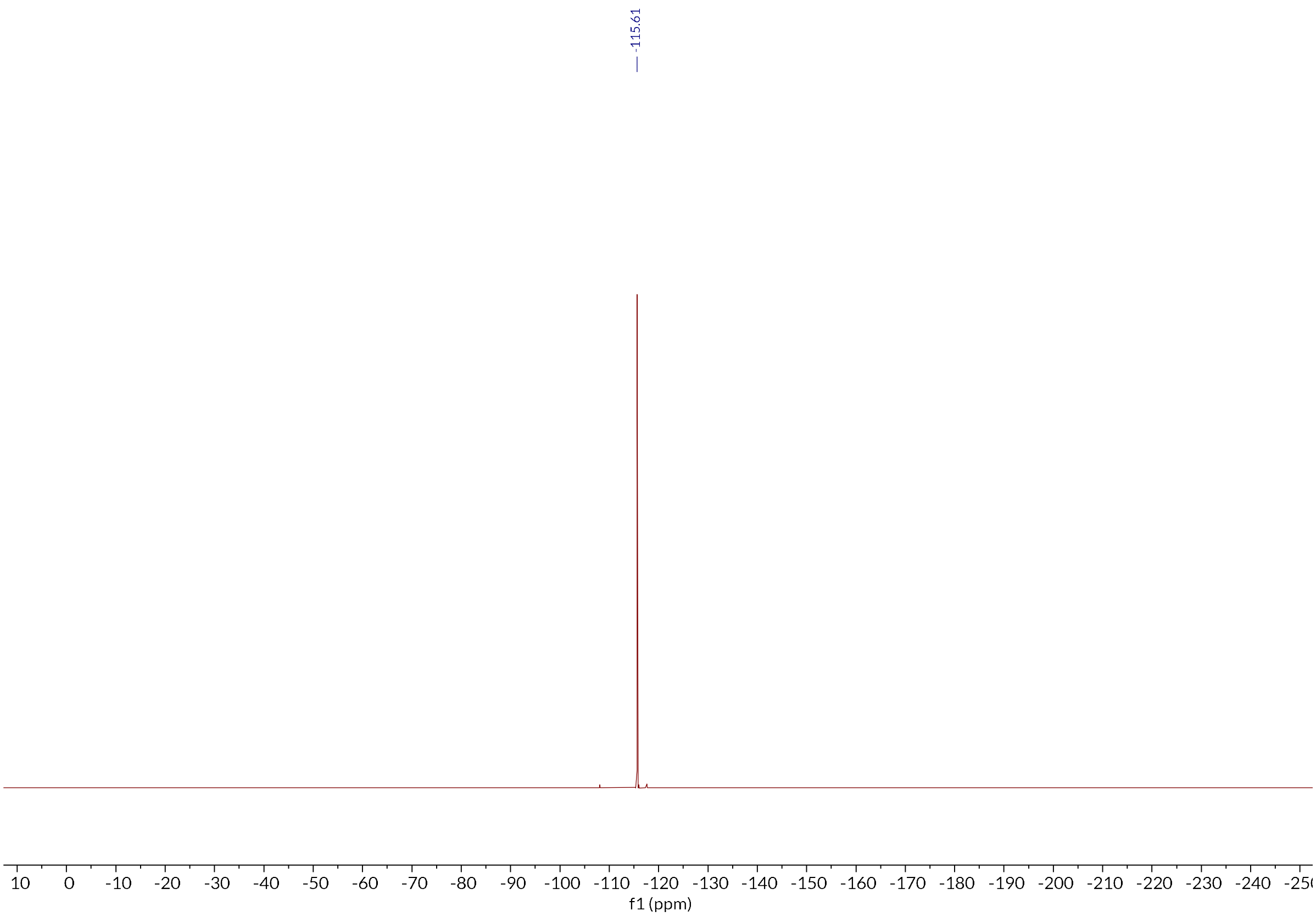
-115.61
10
0
-10
-20
-30
-40
-50
-60
-70
-80
-90
-100
-110
-120
-130
-140
-150
-160
-170
-180
-190
-200
-210
-220
-230
-240
f1 (ppm)

N-Cinnamyl-*N*-(4-fluorobenzyl)aniline (1ac)

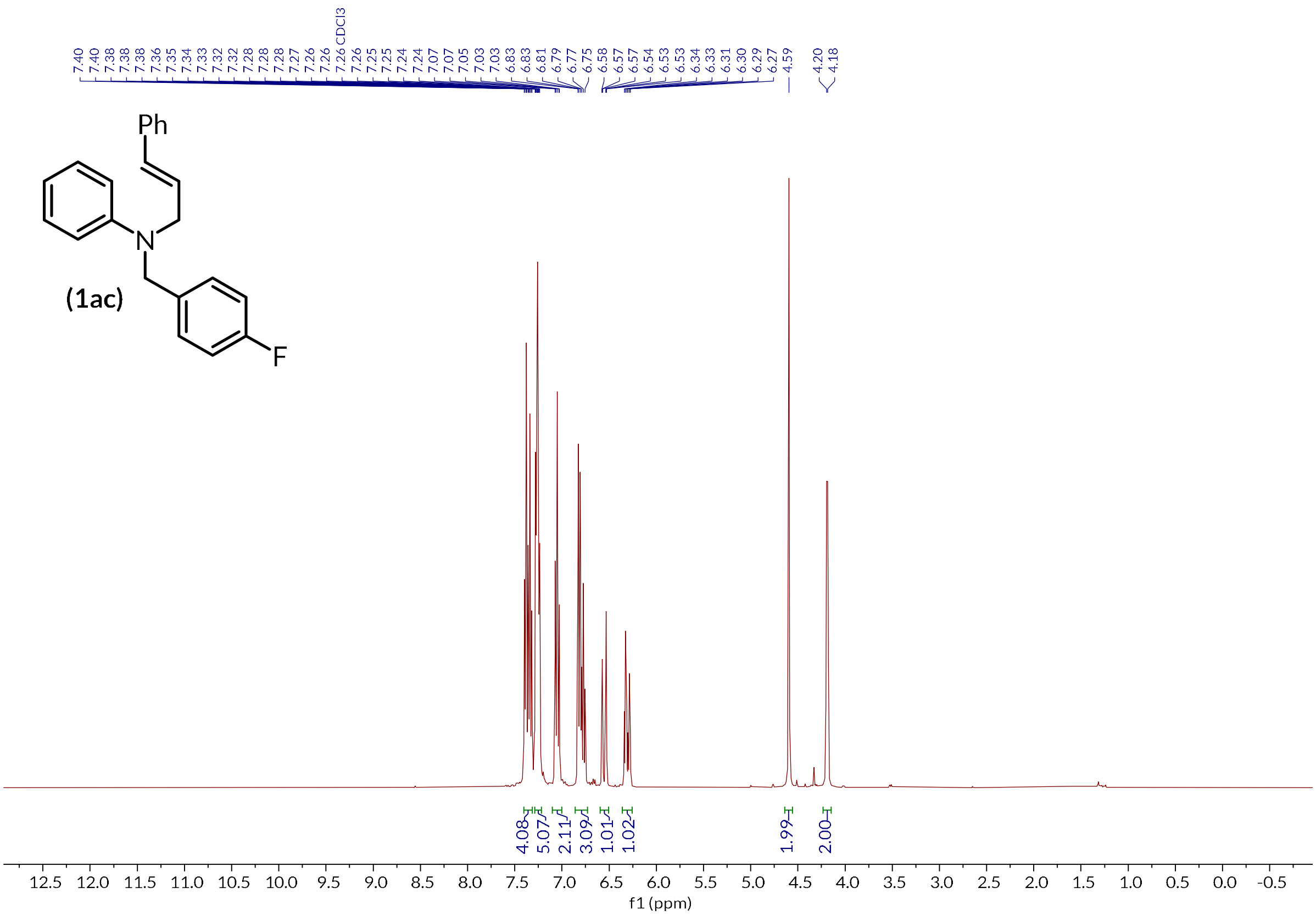

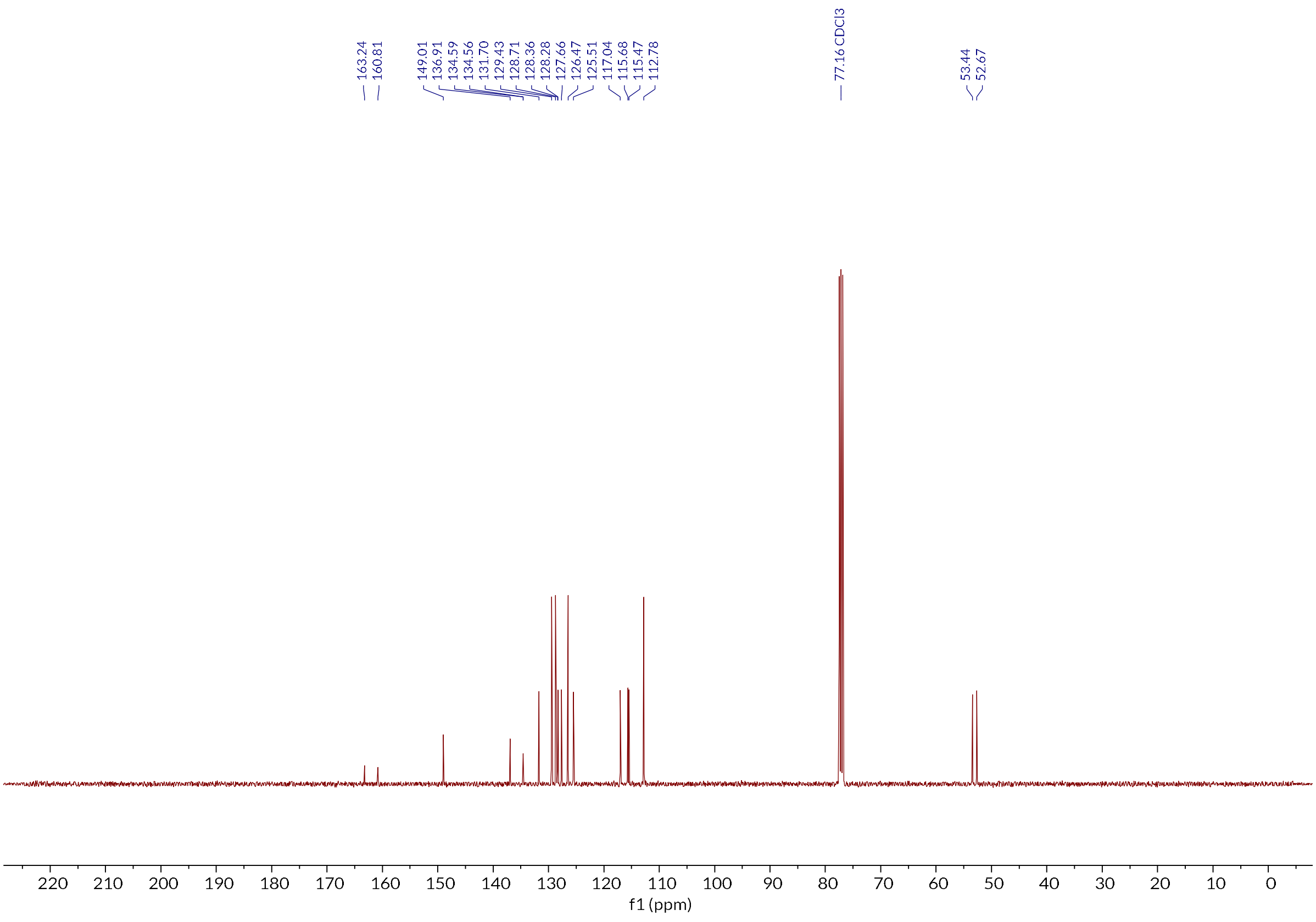

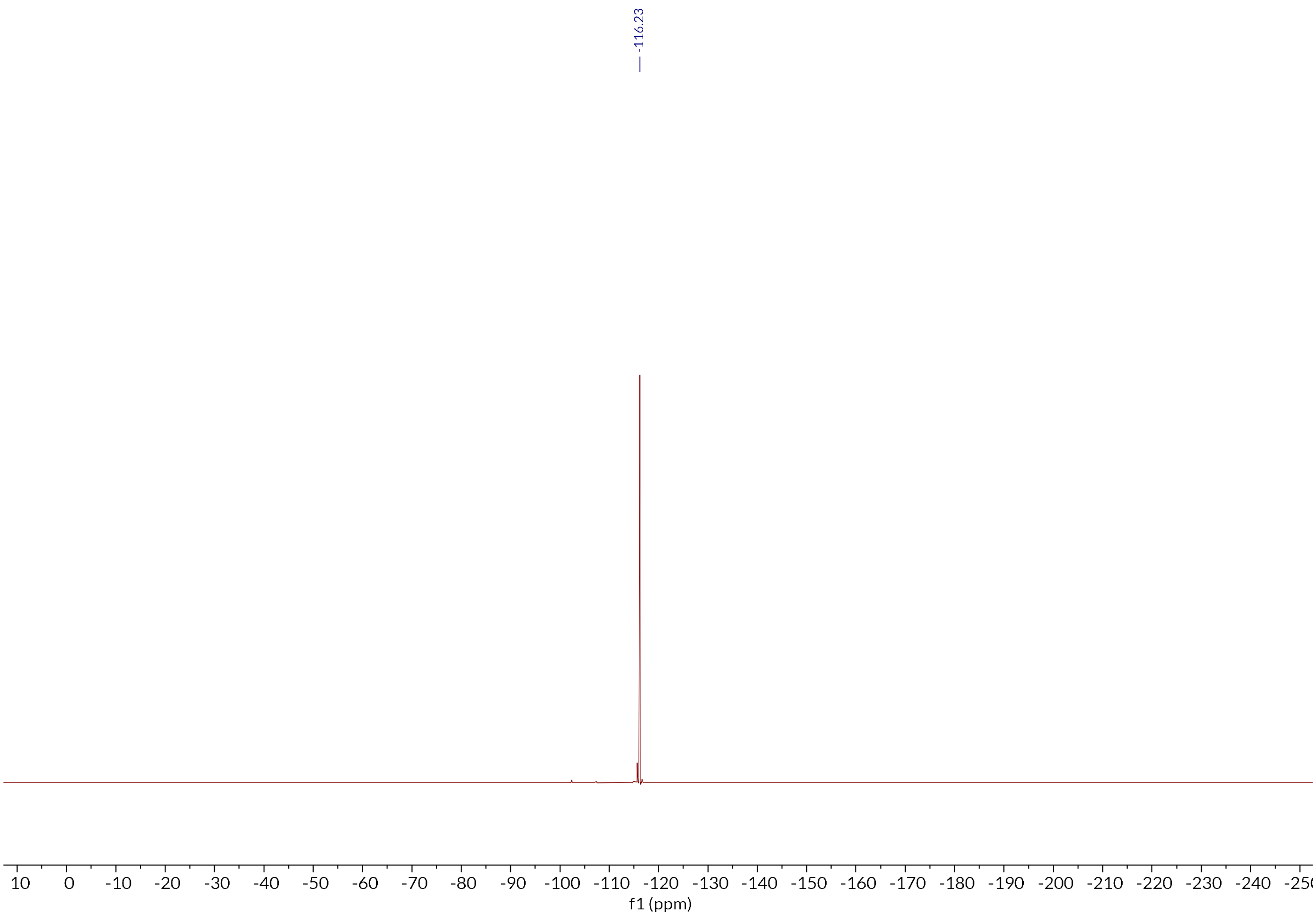
-116.23
10 0 -10 -20 -30 -40 -50 -60 -70 -80 -90 -100 -110 -120 -130 -140 -150 -160 -170 -180 -190 -200 -210 -220 -230 -240
f1 (ppm)

N-(4-(Trifluoromethyl)benzyl)aniline (SI-03)

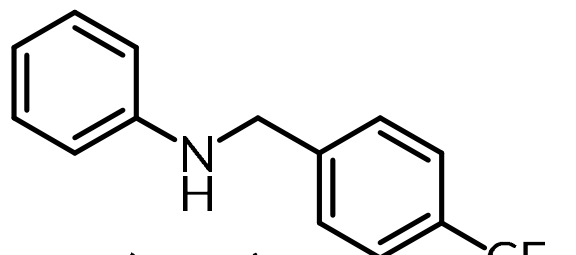

(SI-03)

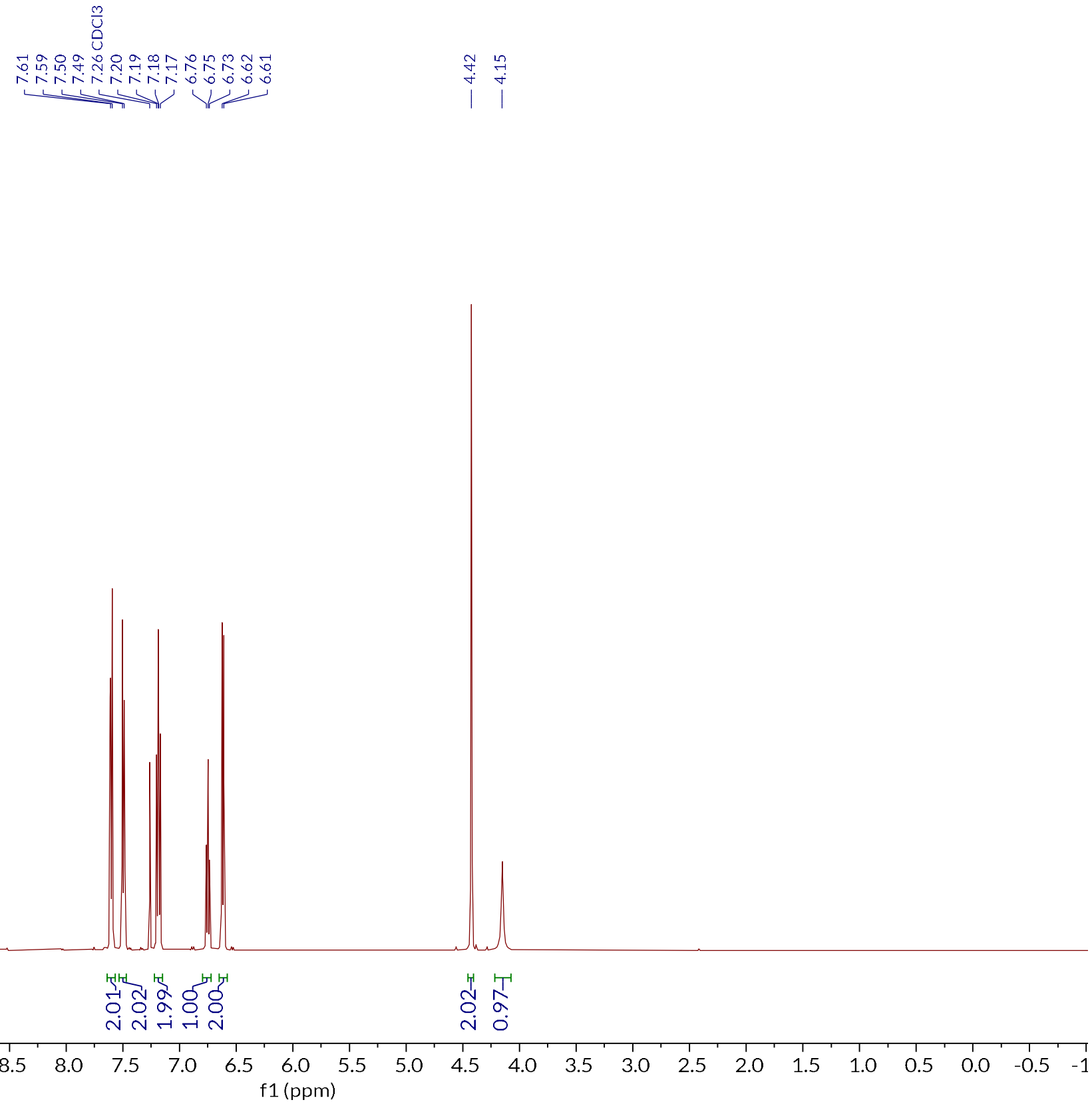

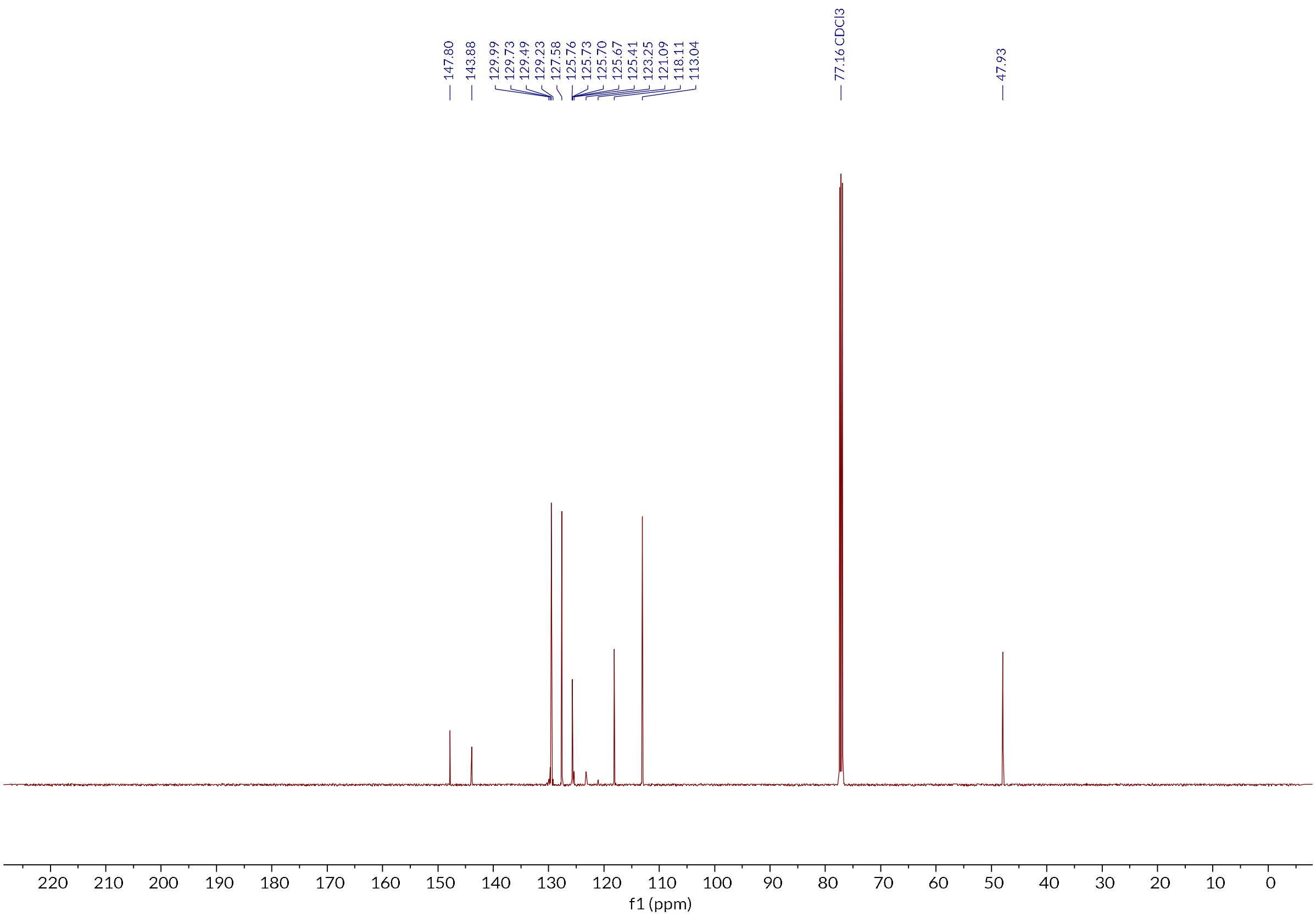

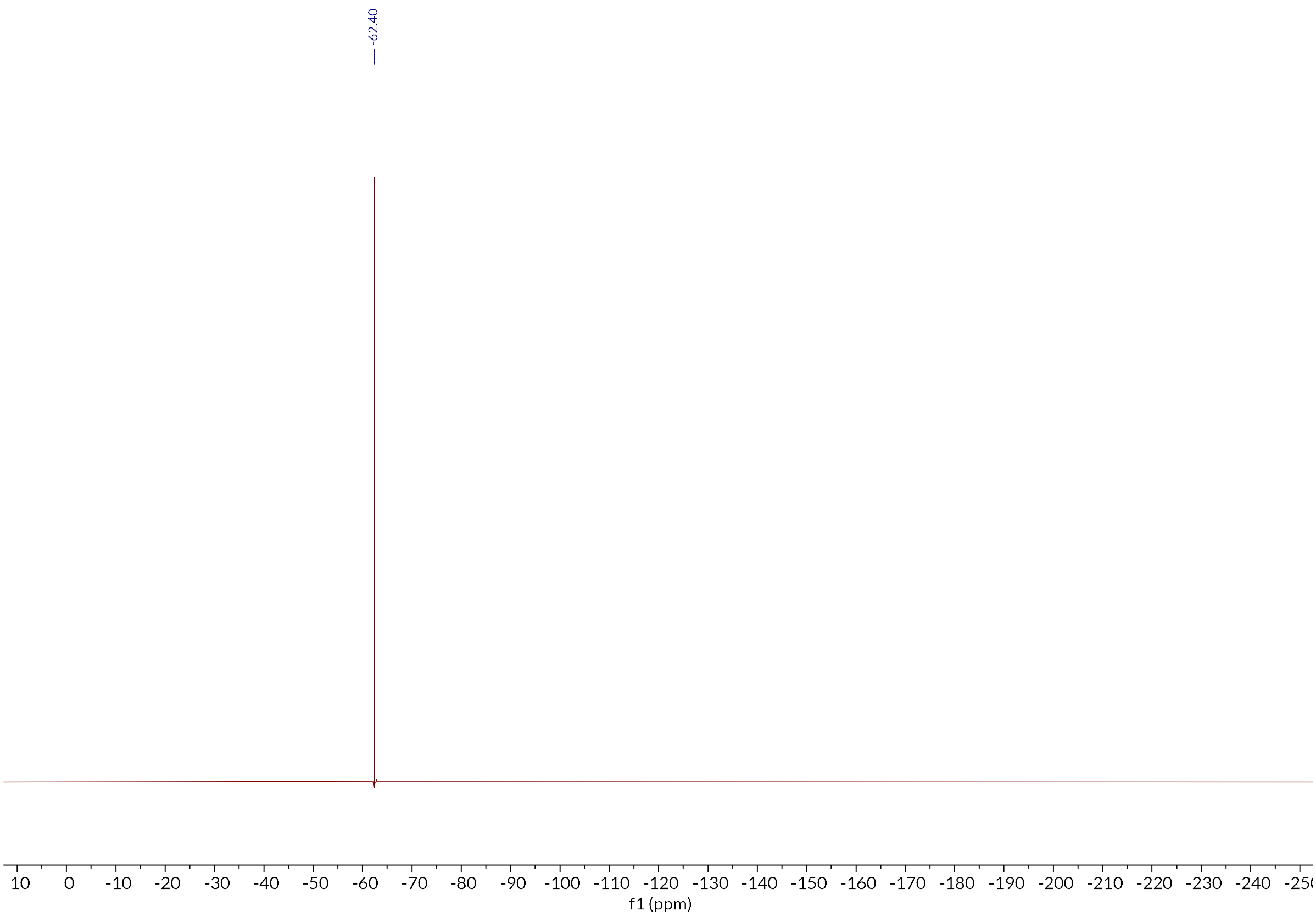
-62.40
10 0 -10 -20 -30 -40 -50 -60 -70 -80 -90 -100 -110 -120 -130 -140 -150 -160 -170 -180 -190 -200 -210 -220 -230 -240
f1 (ppm)

N-Cinnamyl-*N*-(4-(trifluoromethyl)benzyl)aniline (1ad)

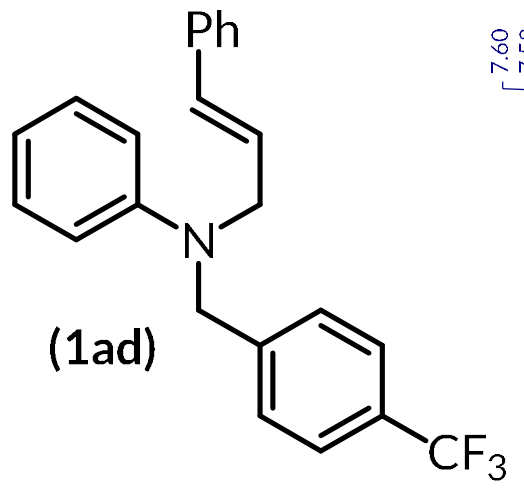

(1ad)

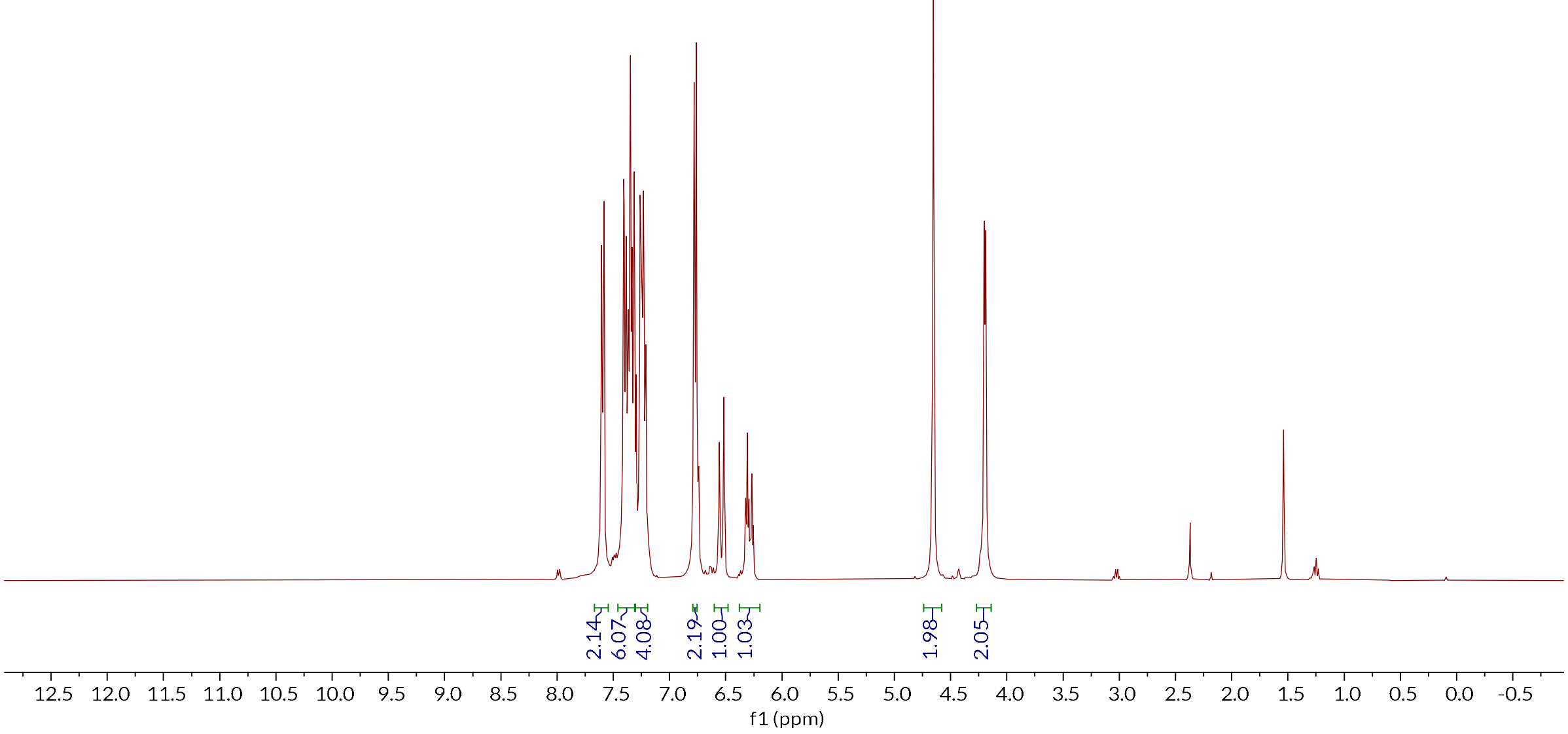

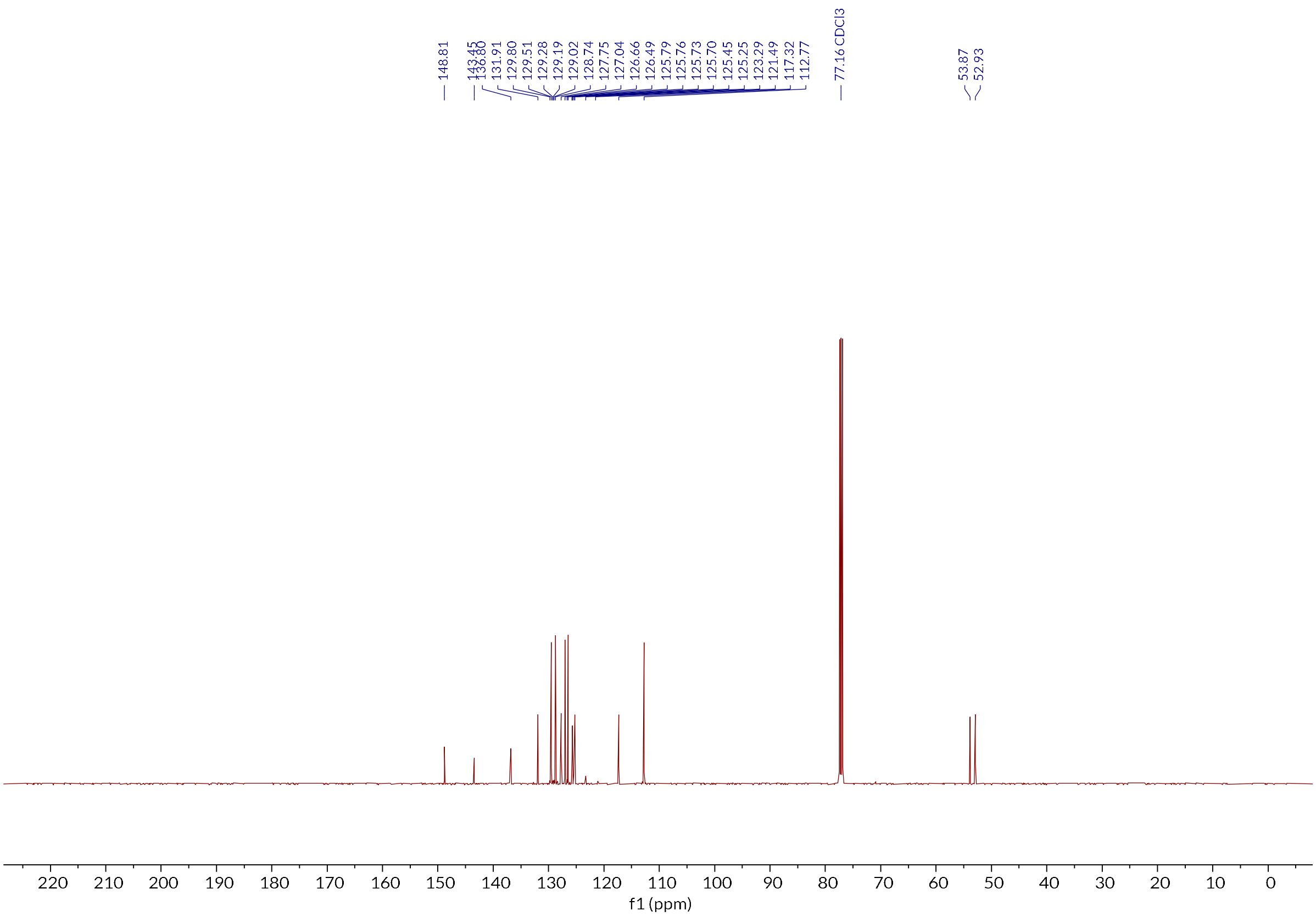

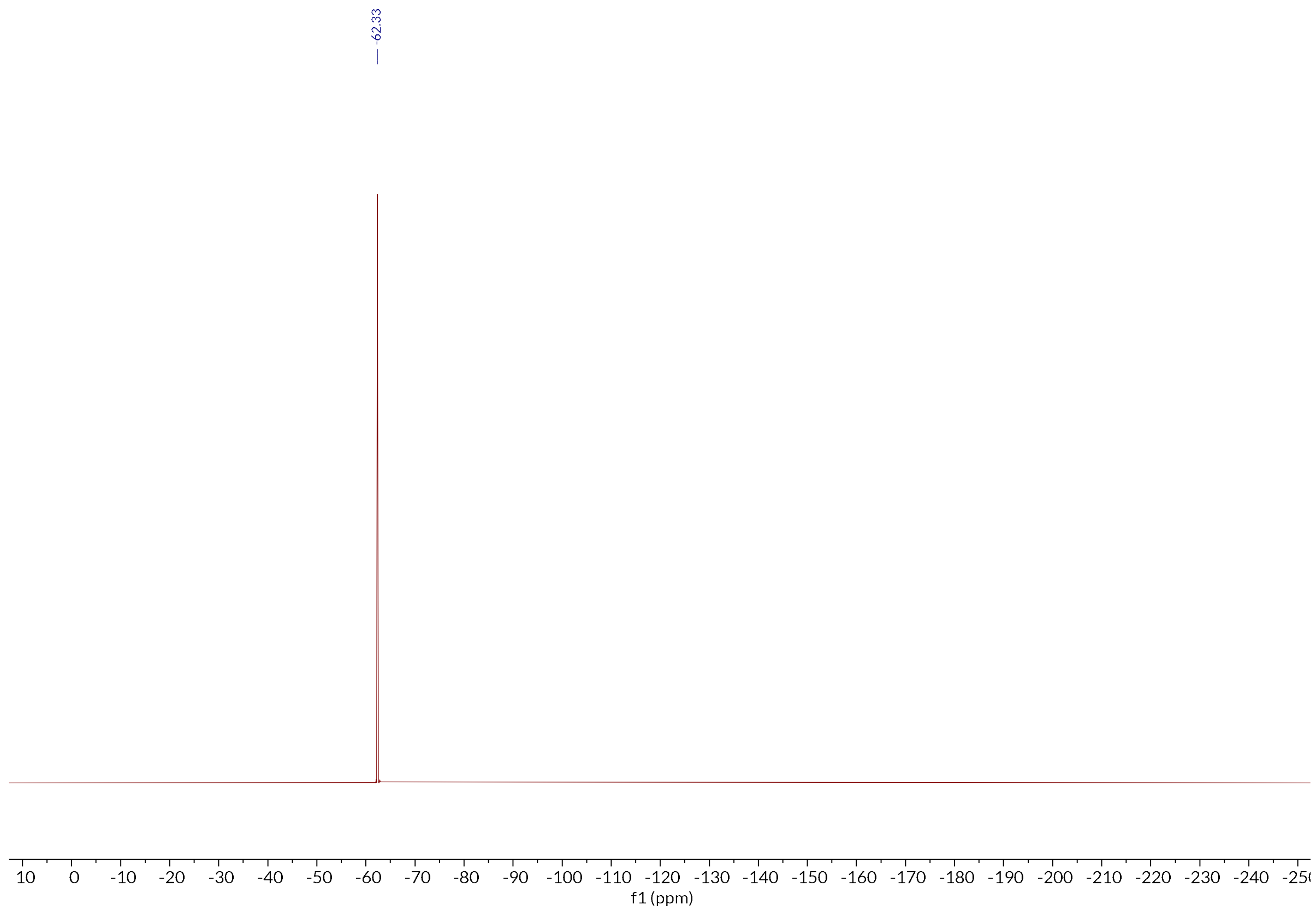
-62.33
10 0 -10 -20 -30 -40 -50 -60 -70 -80 -90 -100 -110 -120 -130 -140 -150 -160 -170 -180 -190 -200 -210 -220 -230 -240
f1 (ppm)

N-Cinnamyl-*N*-(naphthalen-1-ylmethyl)aniline (1ae)

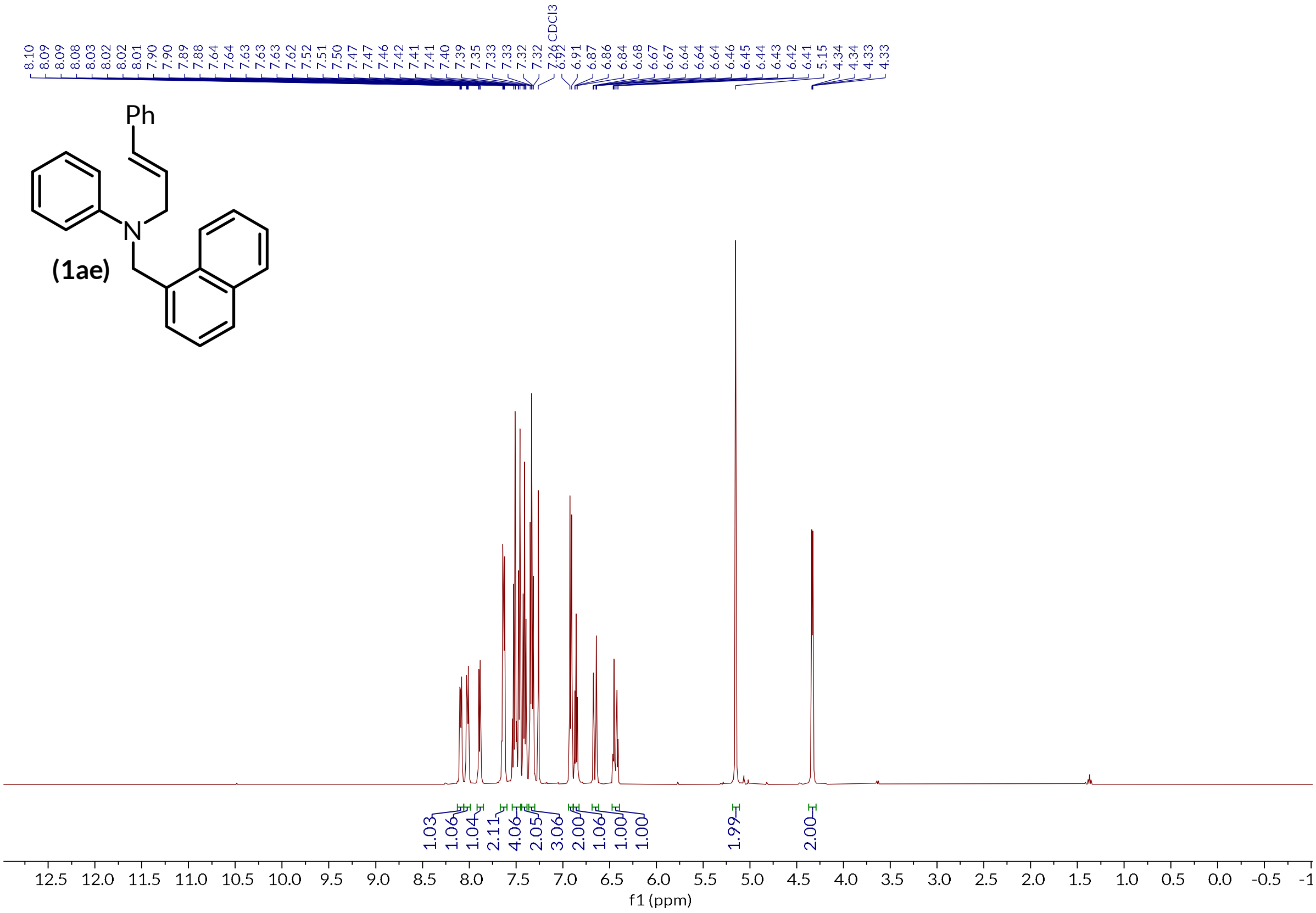

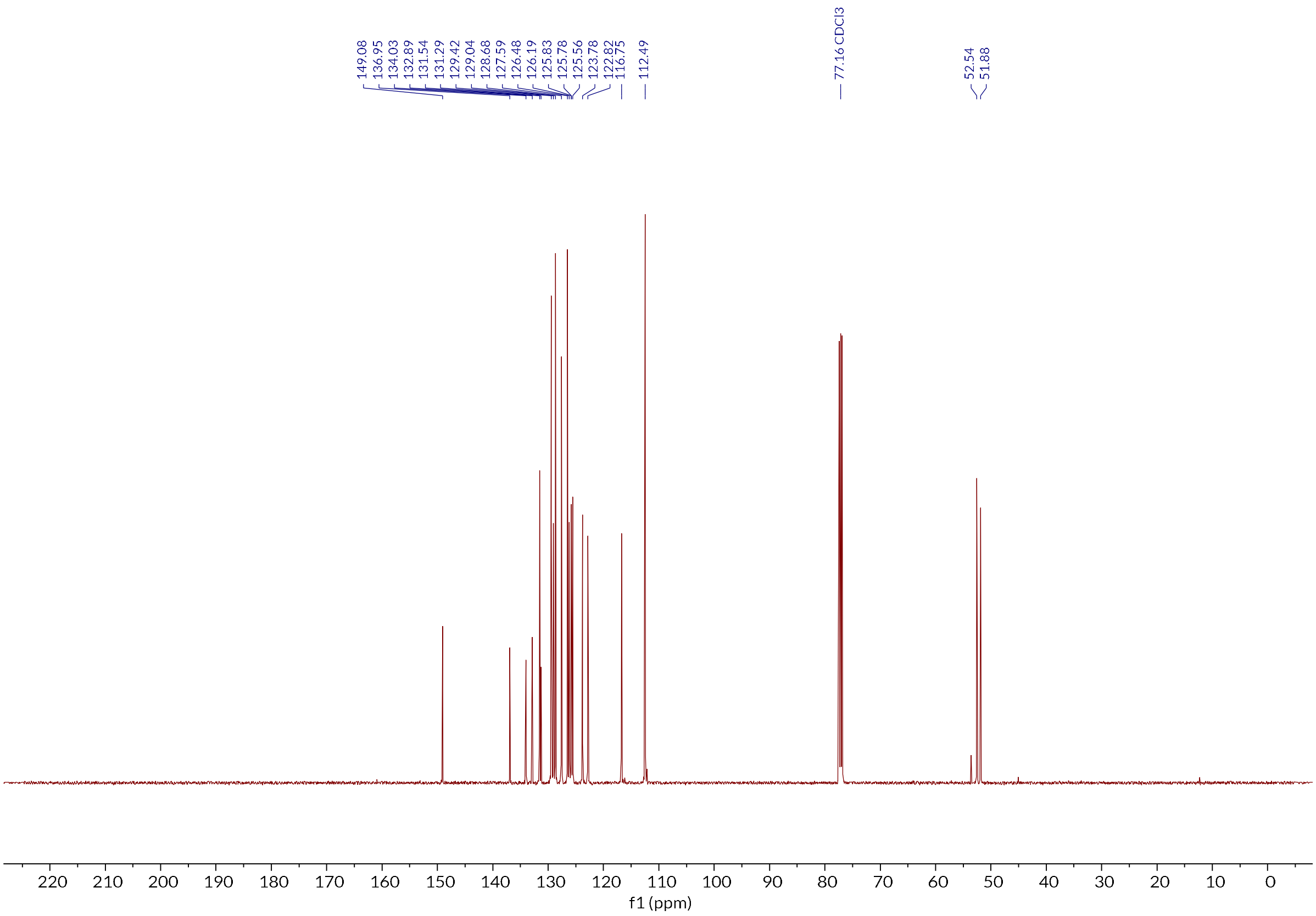

N-(Thiophen-2-ylmethyl)aniline (SI-04)

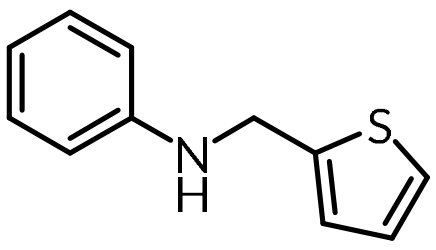

(SI-04)

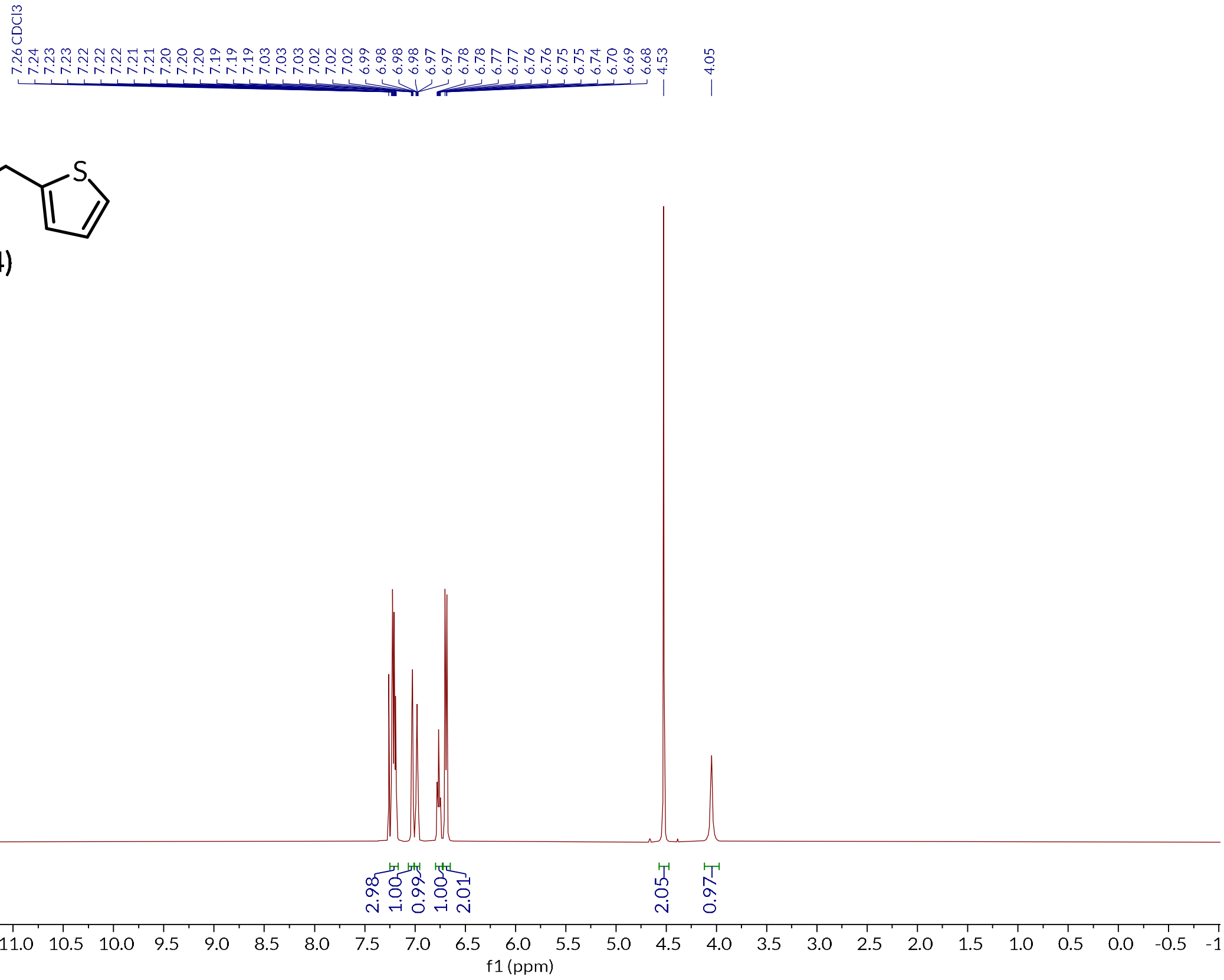

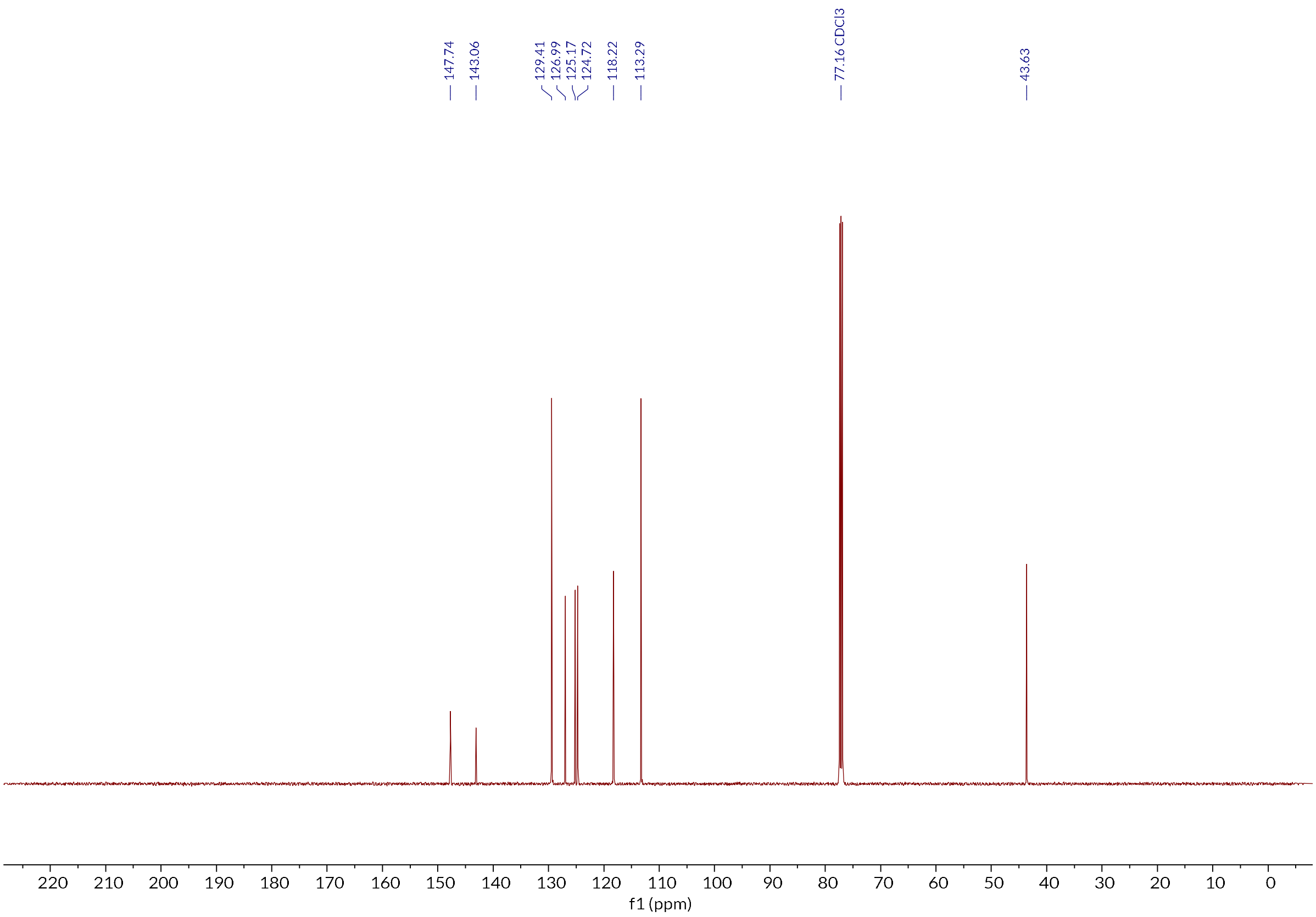

N-Cinnamyl-*N*-(thiophen-2-ylmethyl)aniline (1af)

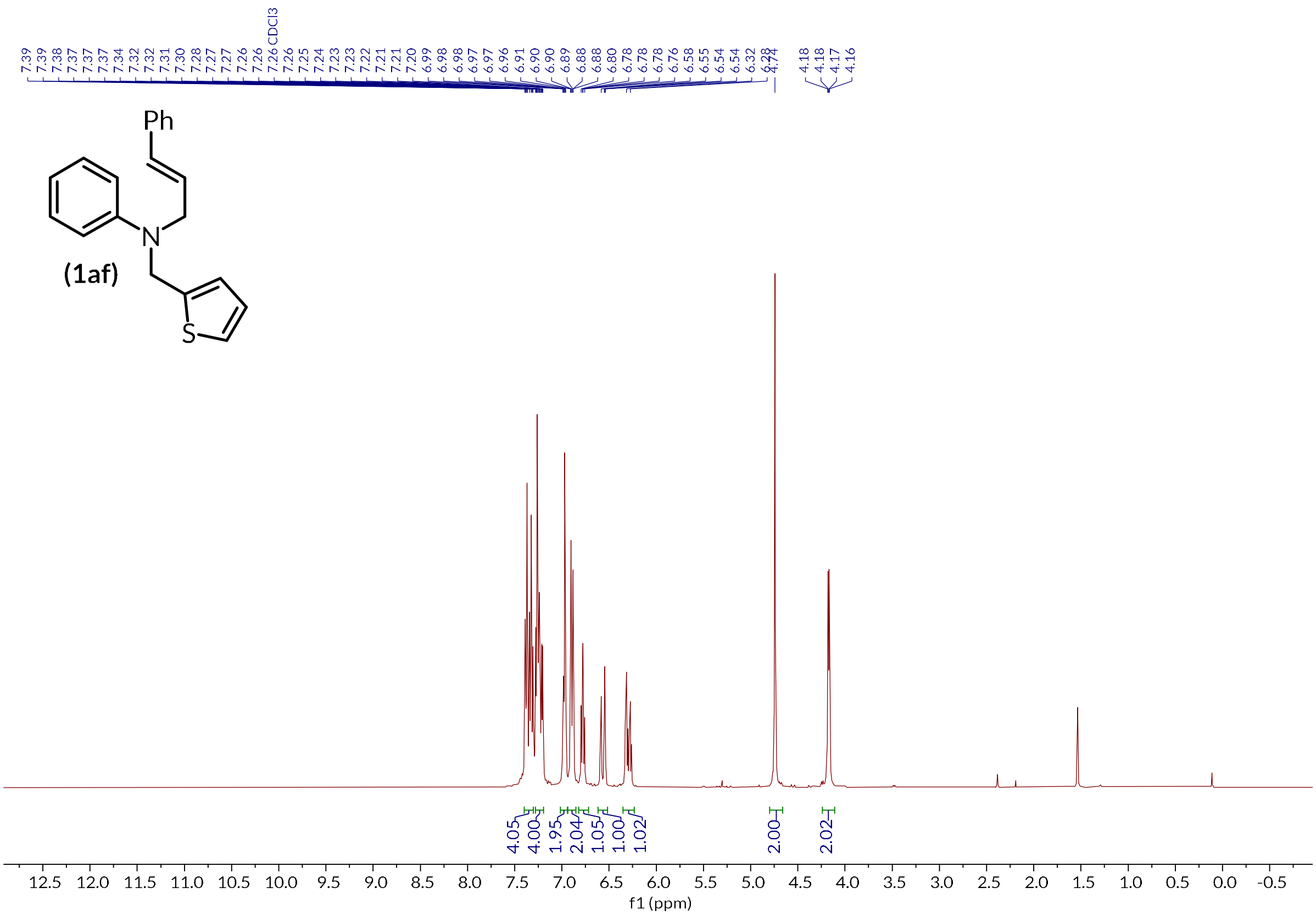

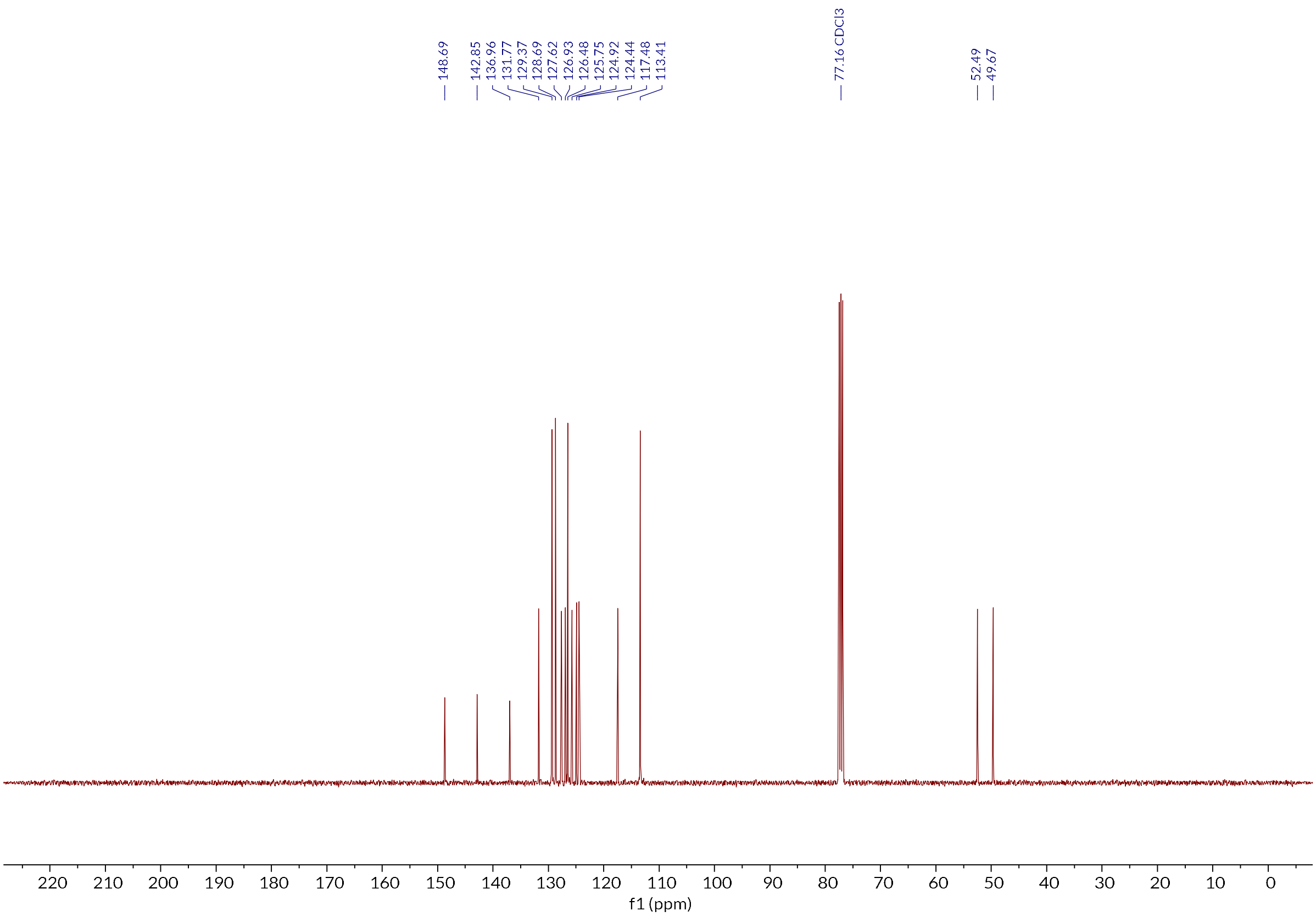

N-Cinnamyl-*N*-methylaniline (1ag)

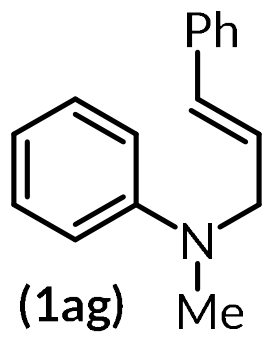

(1ag)

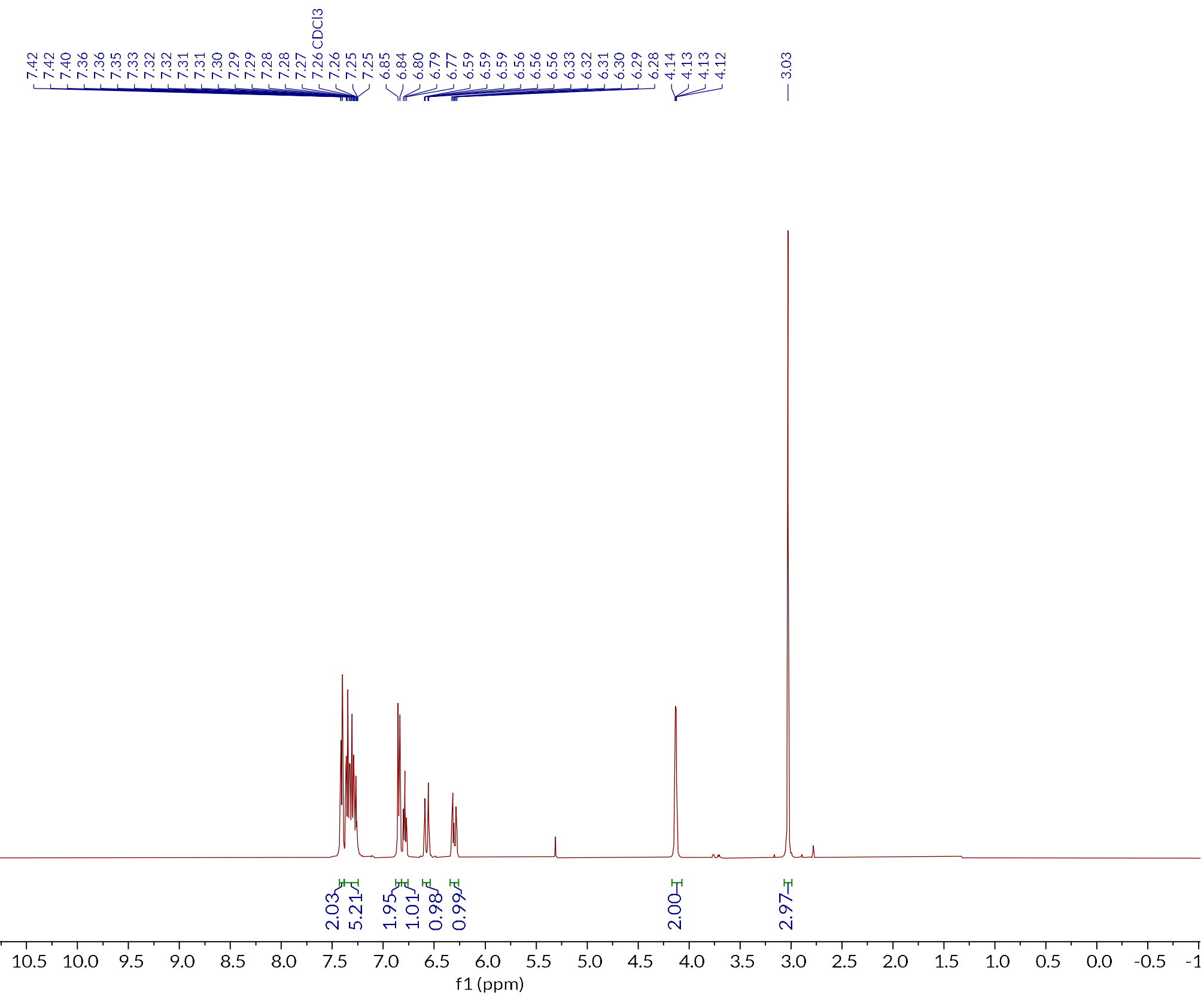

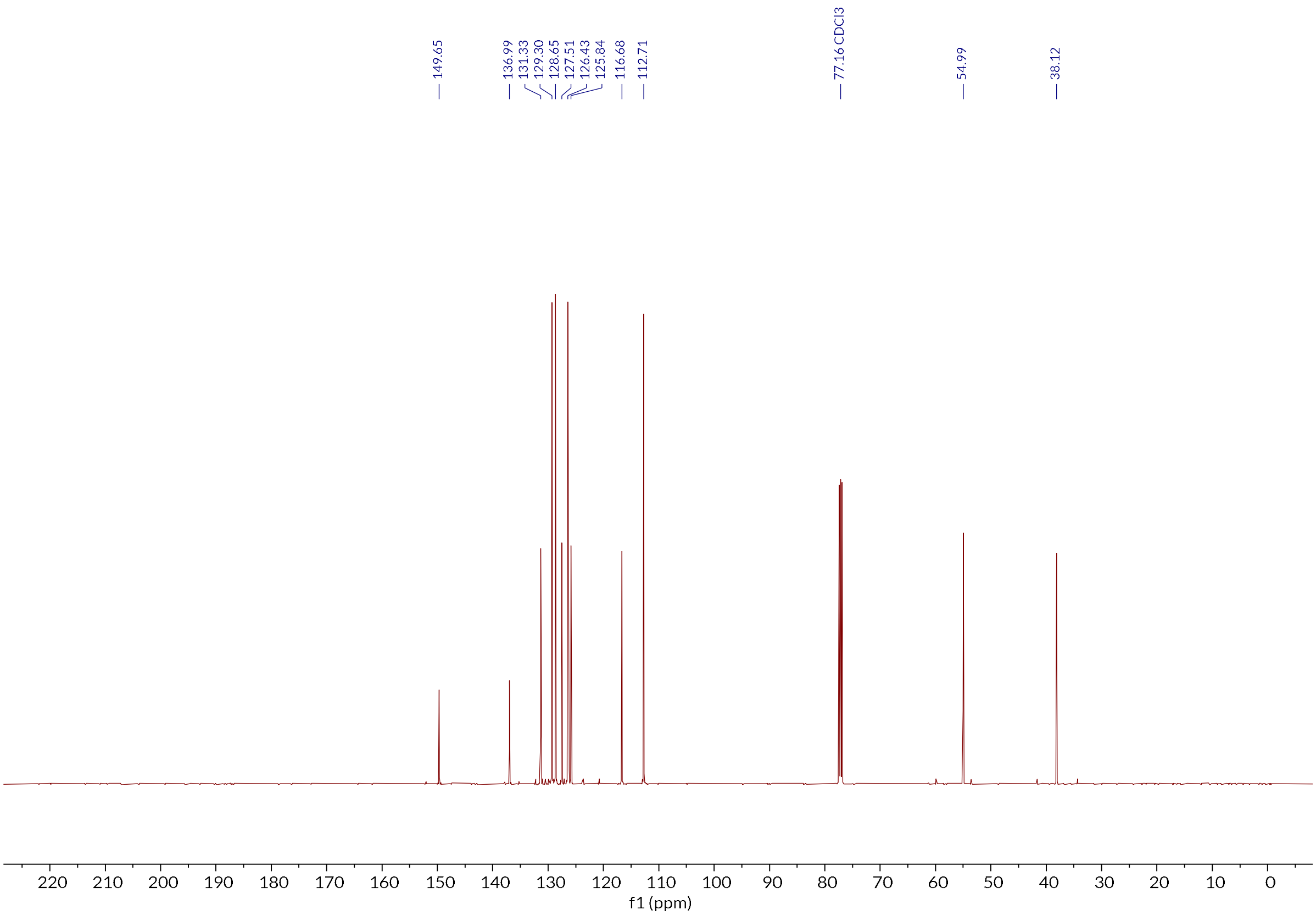

N-Cinnamyl-*N*-cyclohexylaniline (1ah)

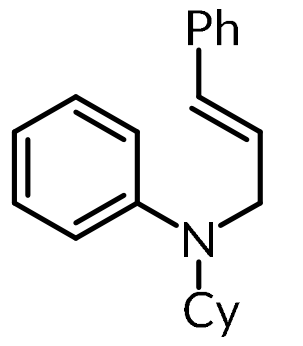

(1ah)

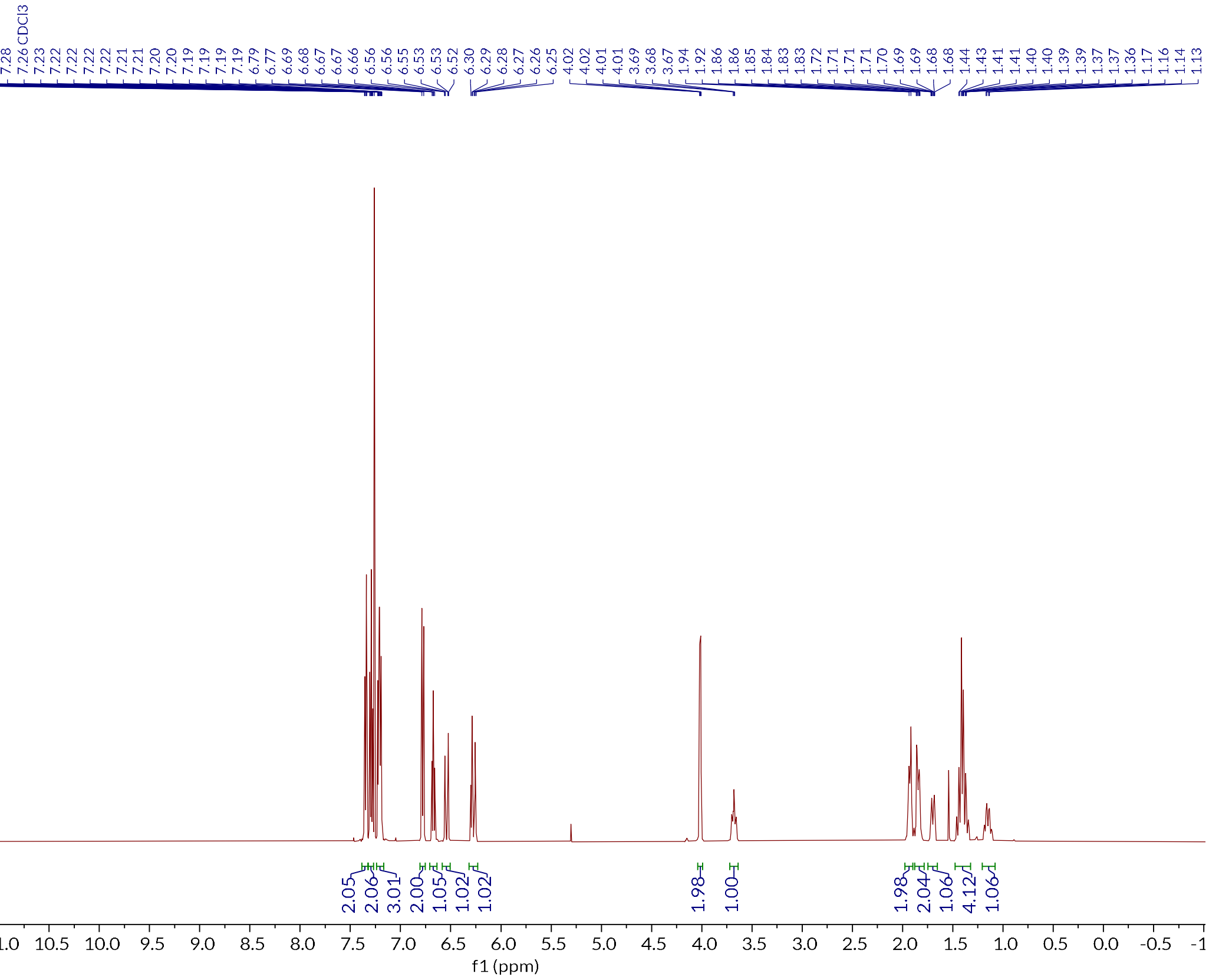

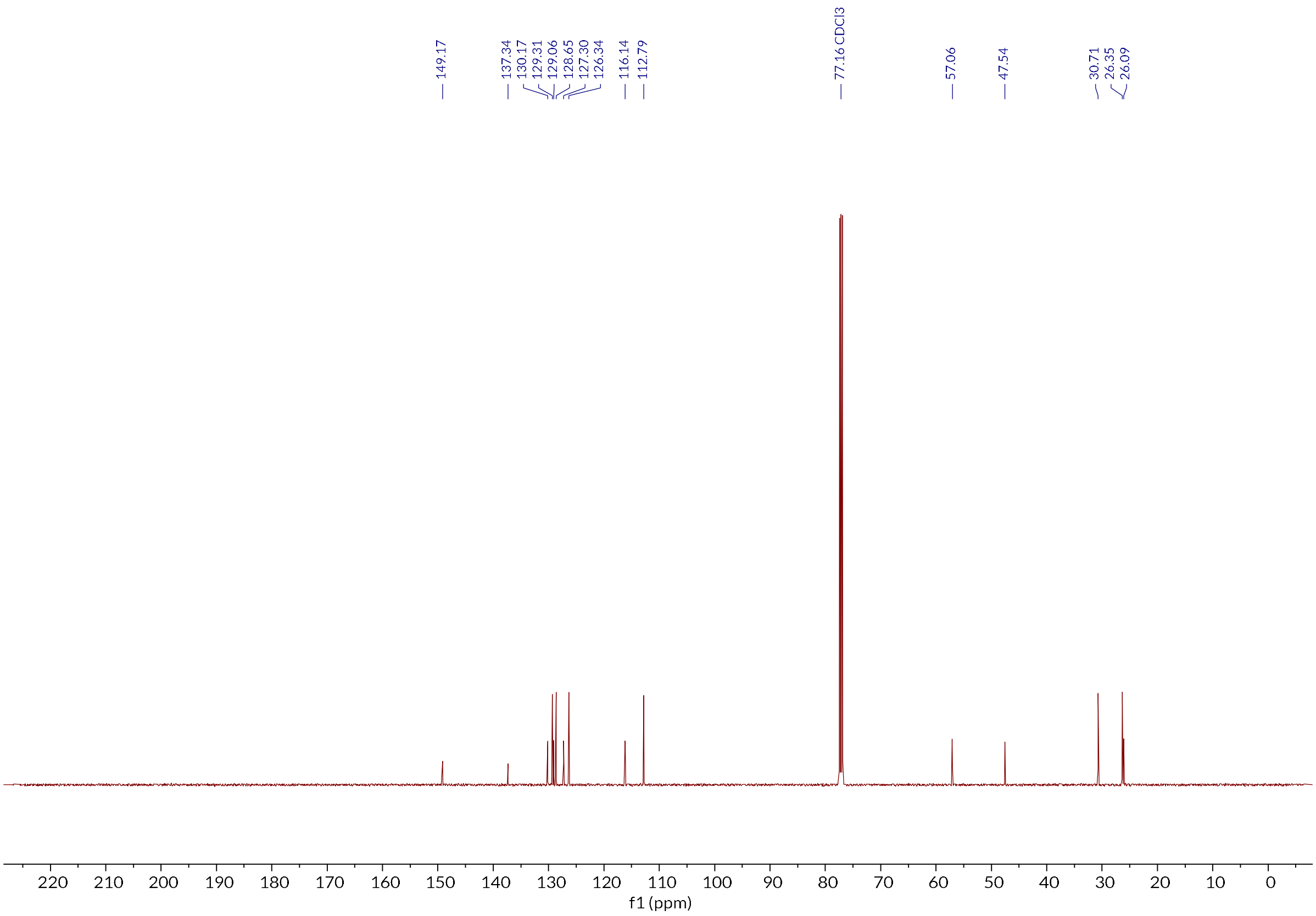

N-Cinnamyl-*N*-phenylaniline (1ai)

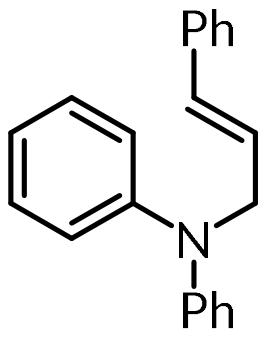

(1ai)

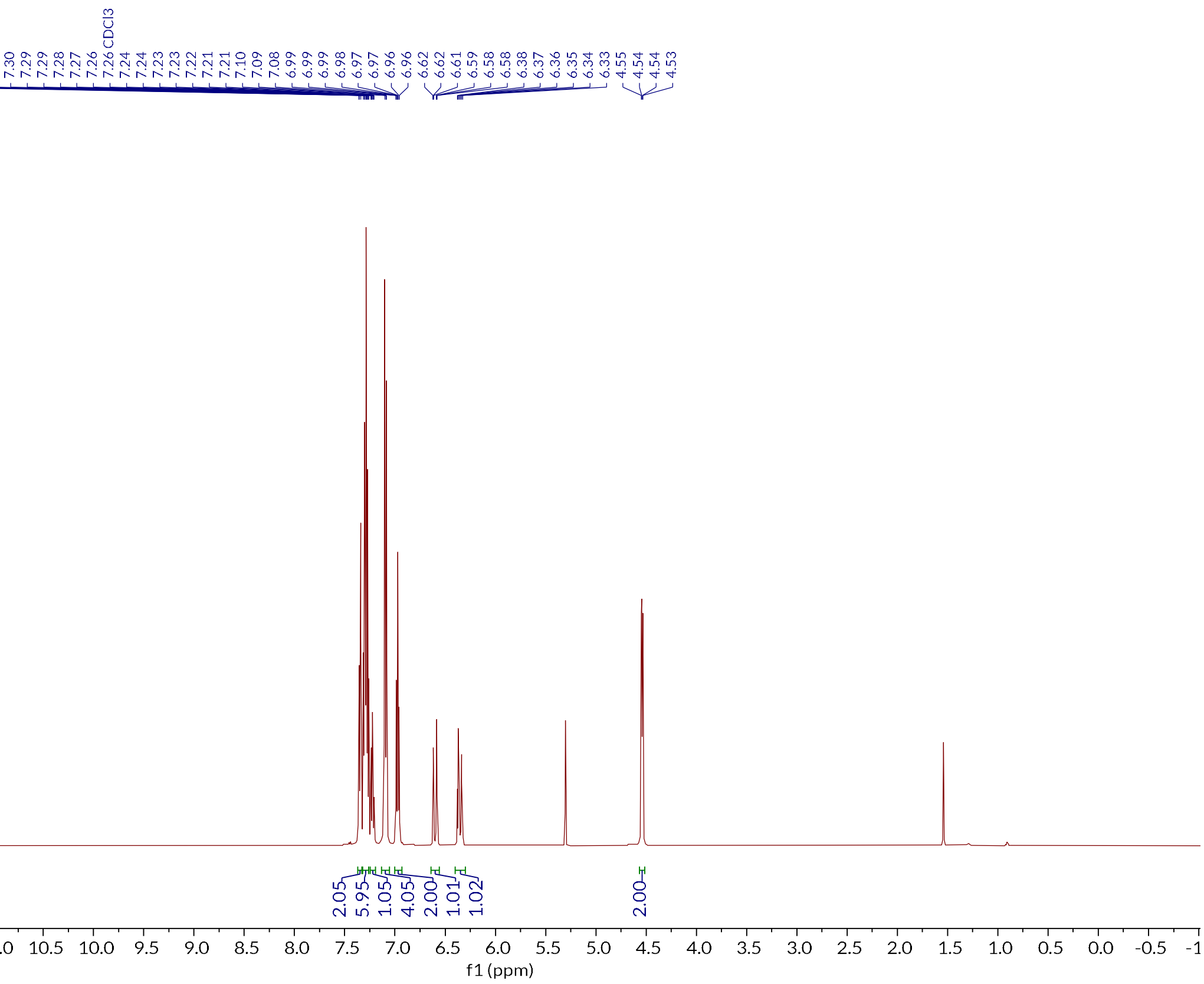

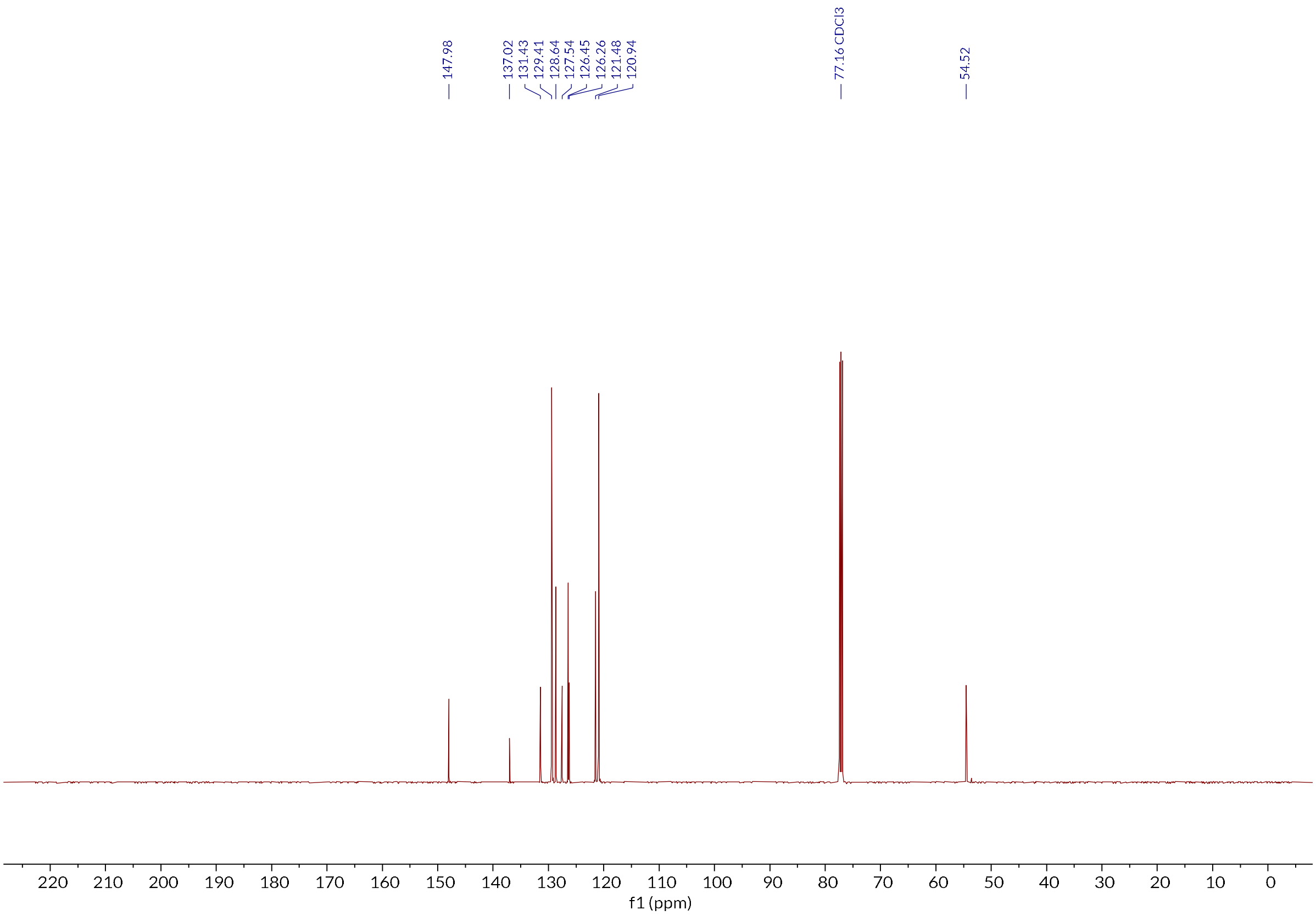

N-Allyl-*N*-cinnamylaniline (1aj)

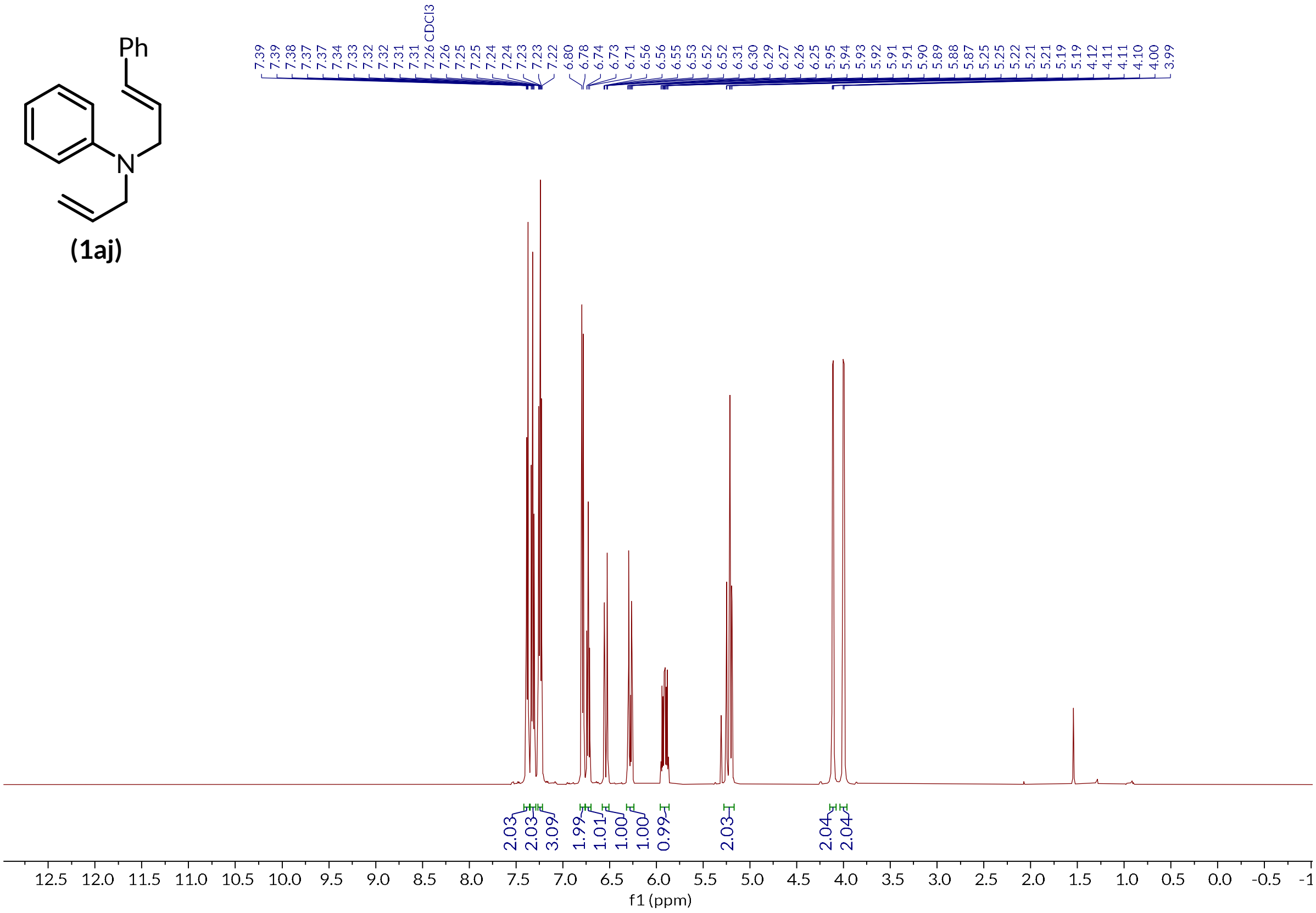

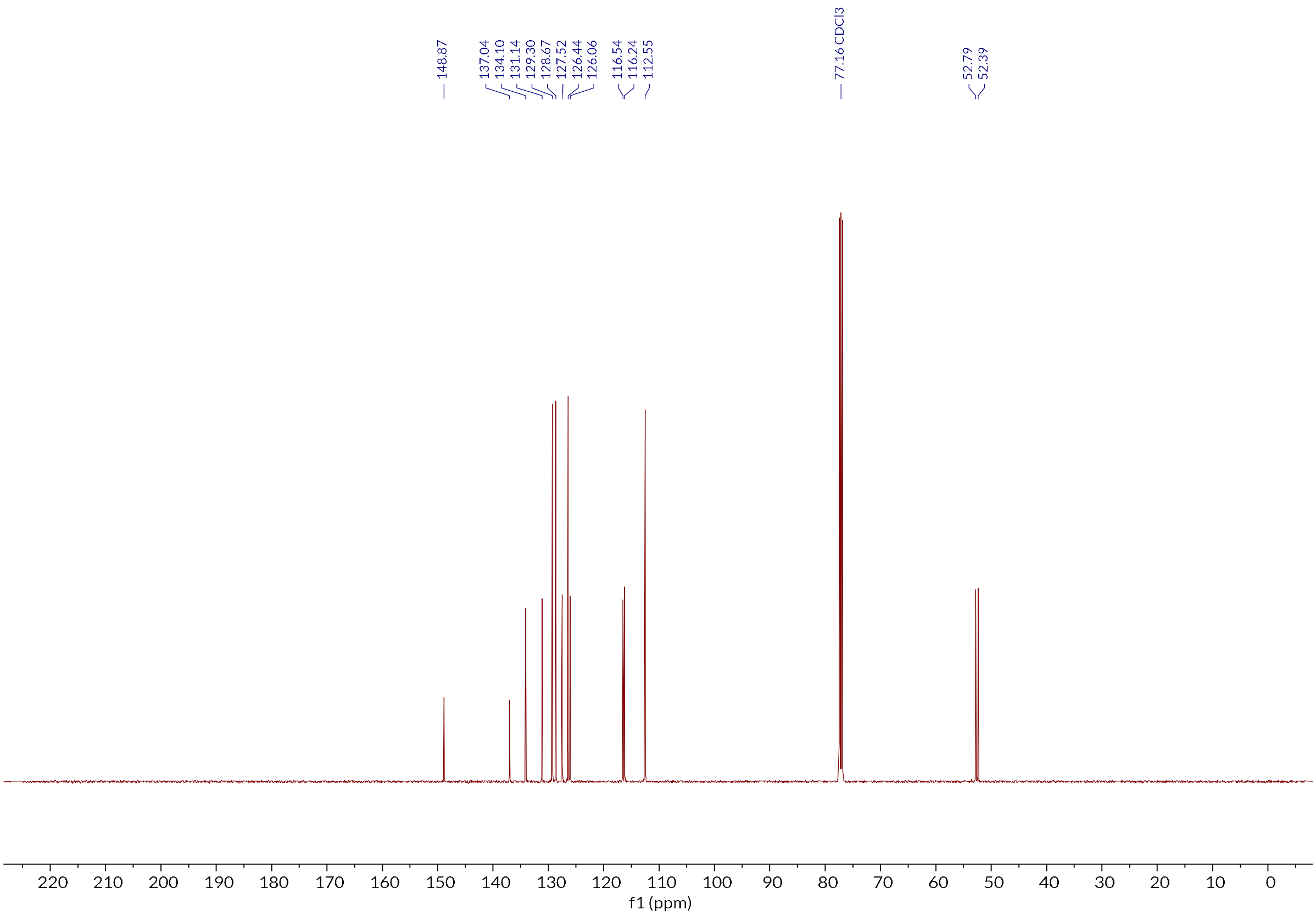

N,N-Dicinnamylaniline (1ak)

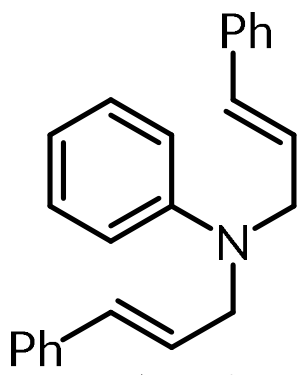

(1ak)

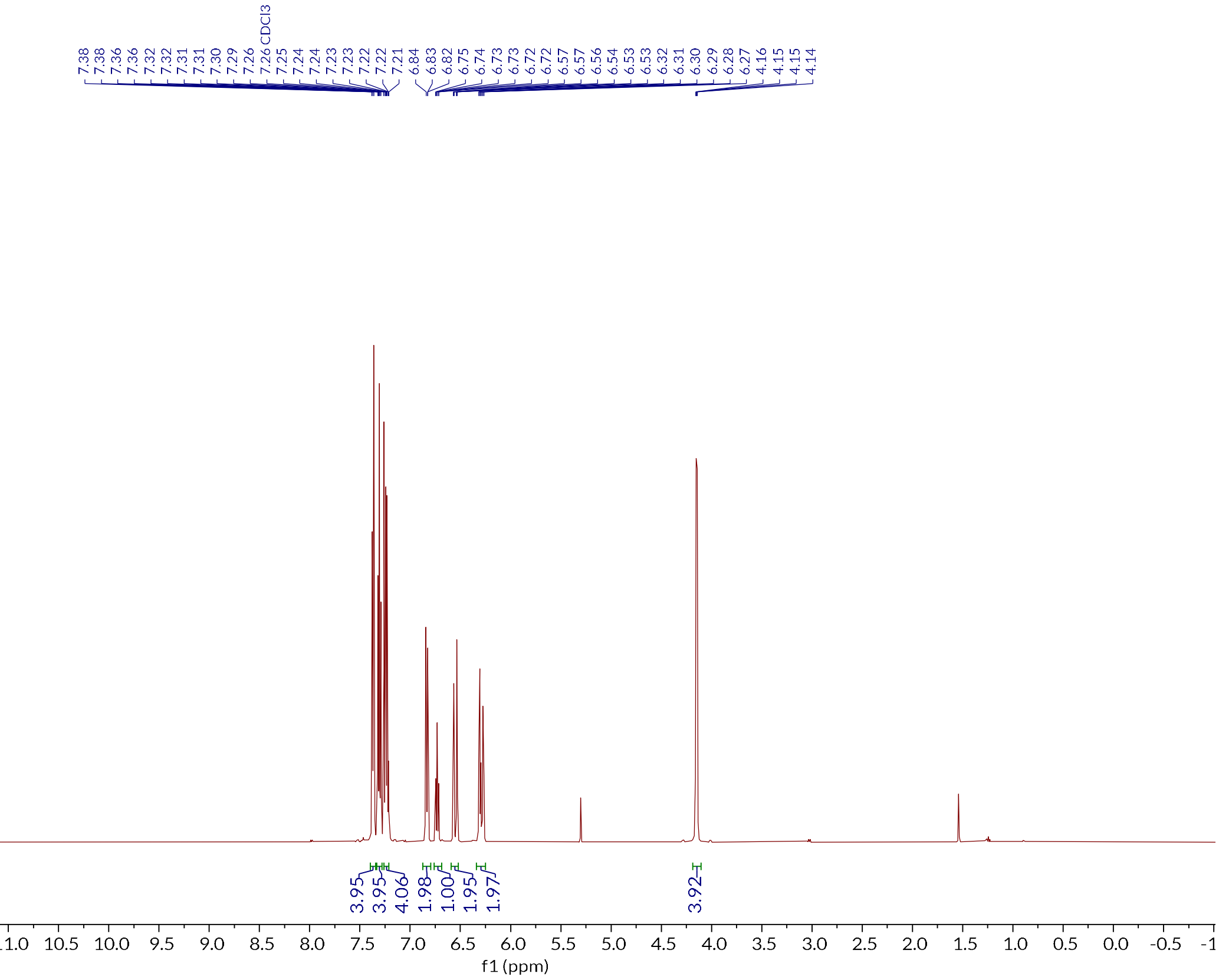

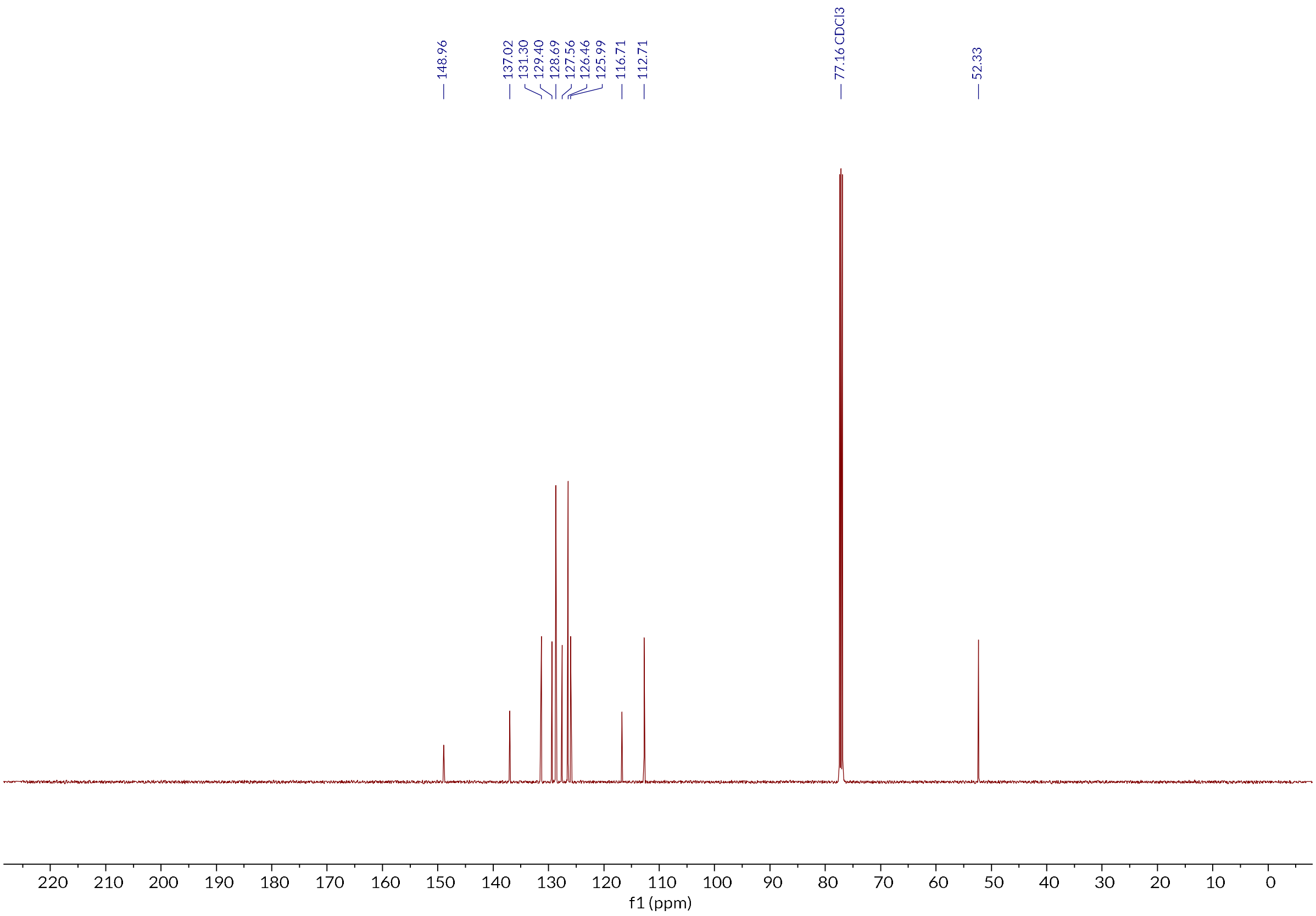

N-Cinnamyl-3,5-dimethoxyaniline (3g)

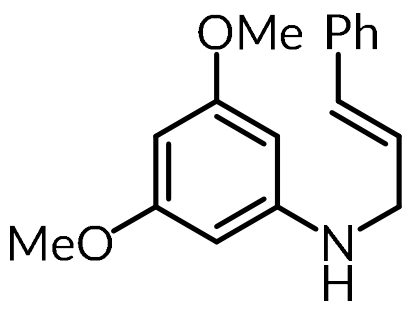

(3g)

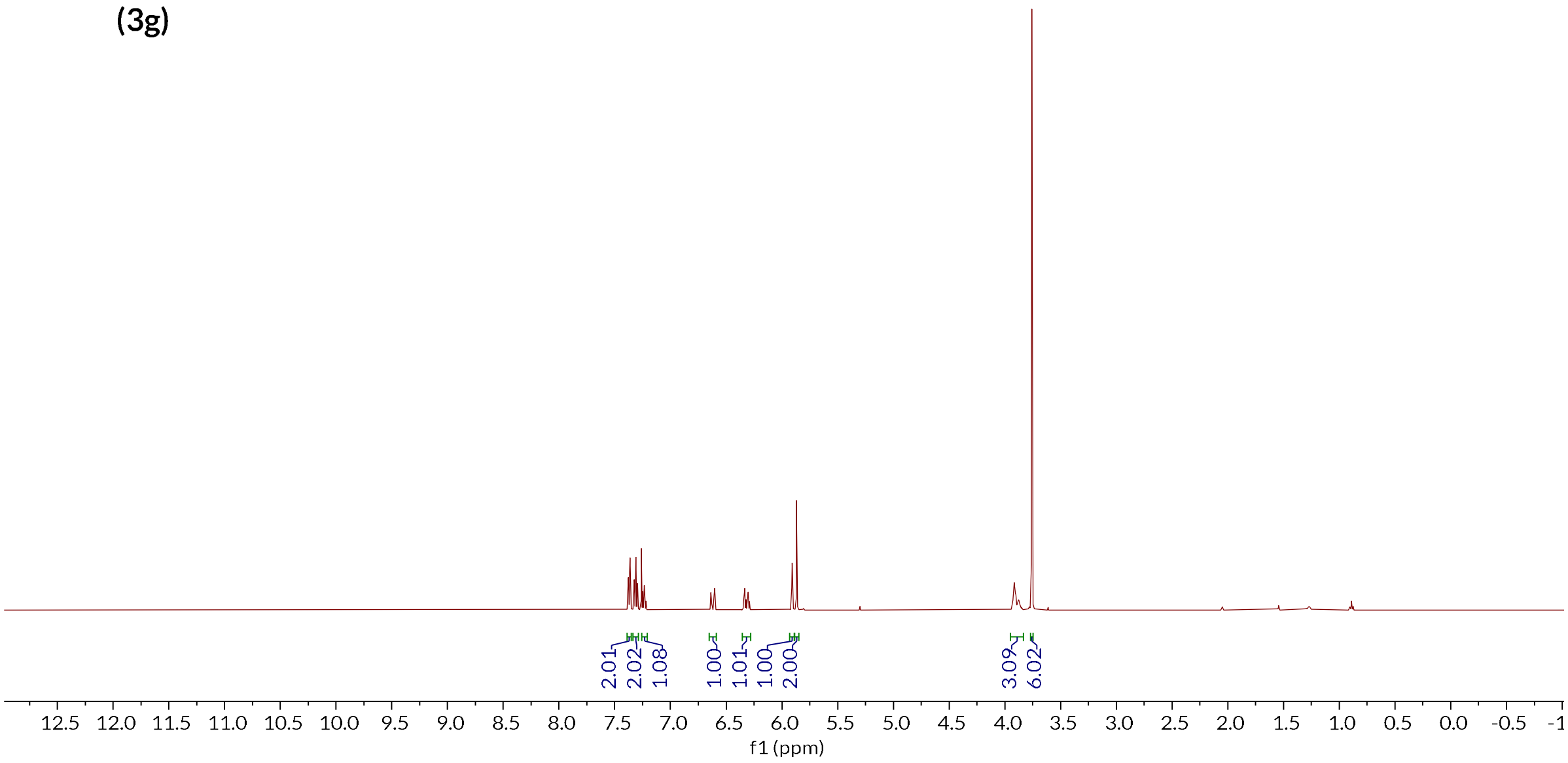

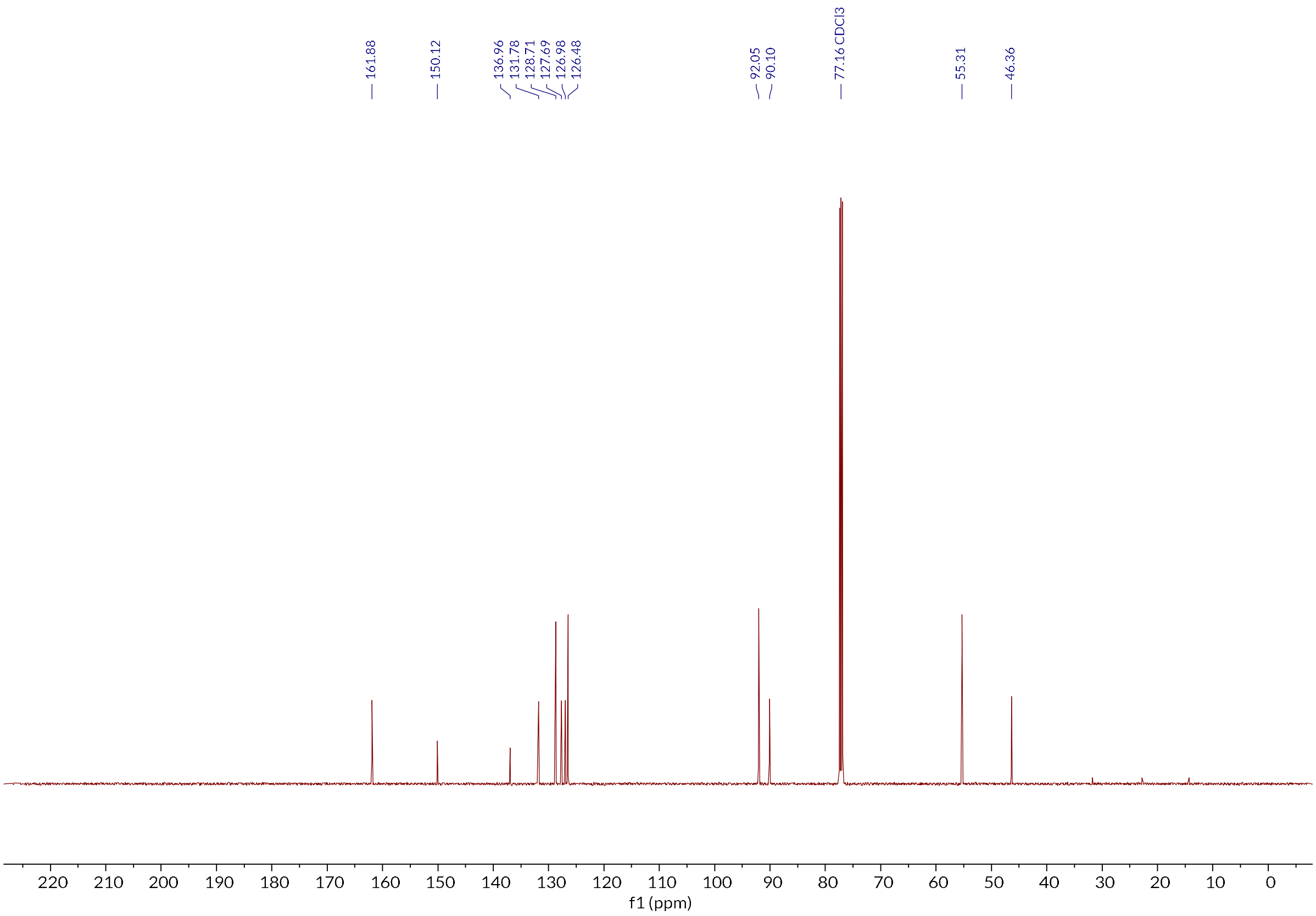

N-Cinnamyl-*N*-(3,5-dimethoxyphenyl)-4-methylbenzenesulfonamide (1al)

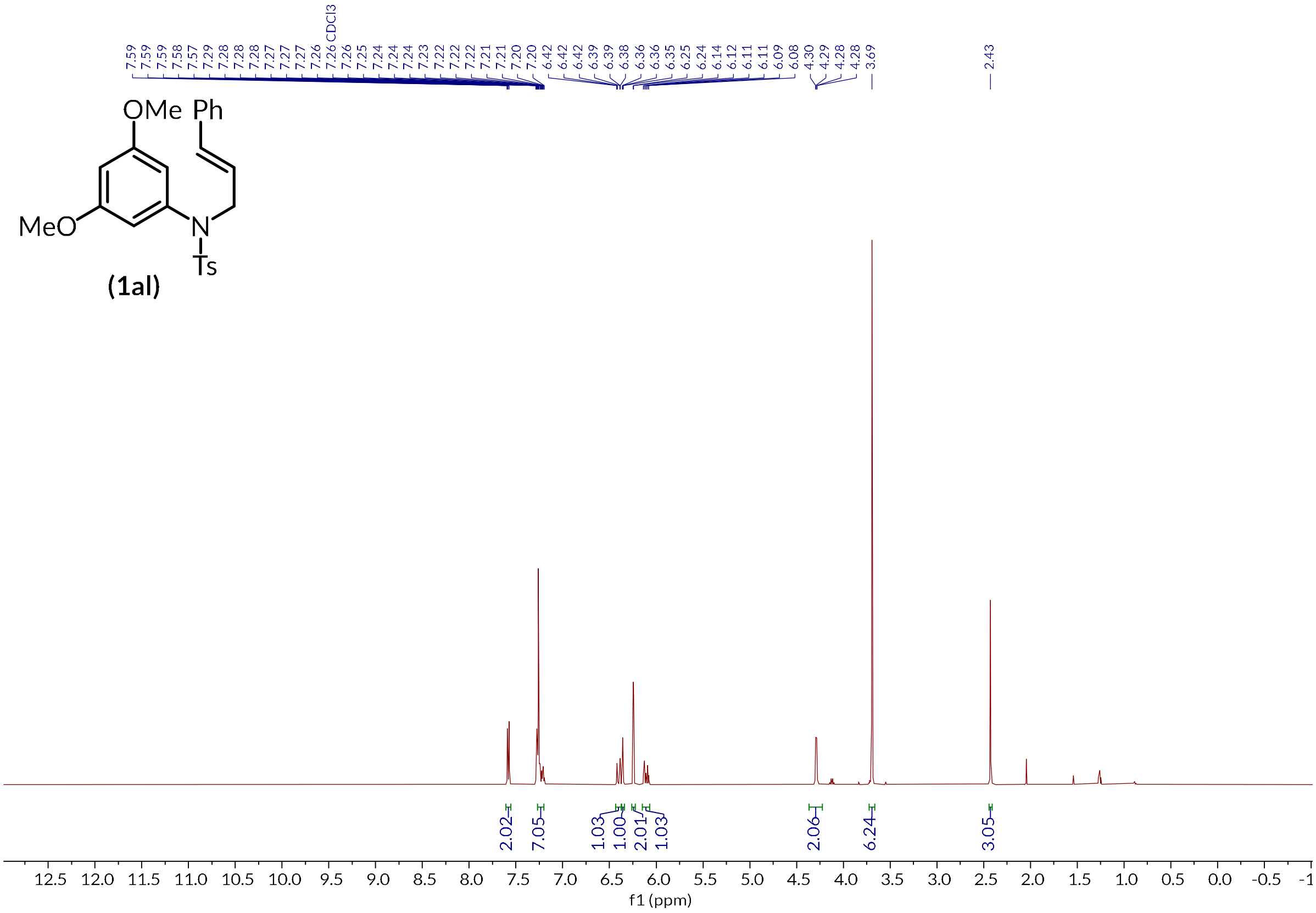

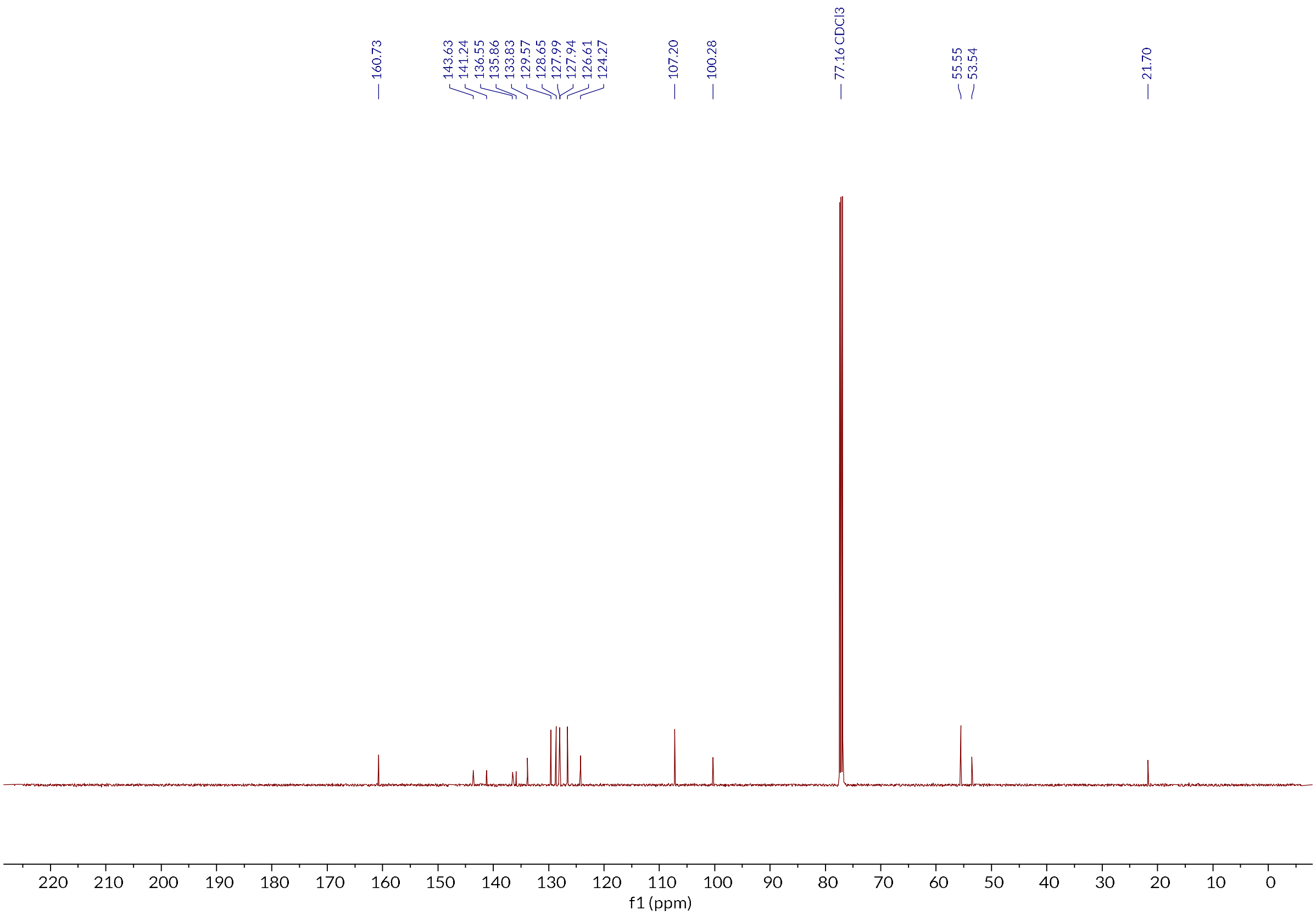

N-(3,5-Dimethoxyphenyl)cinnamamide (SI-05)

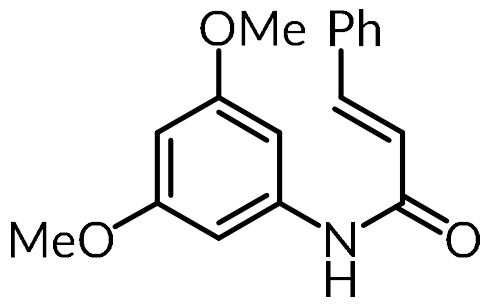

(SI-05)

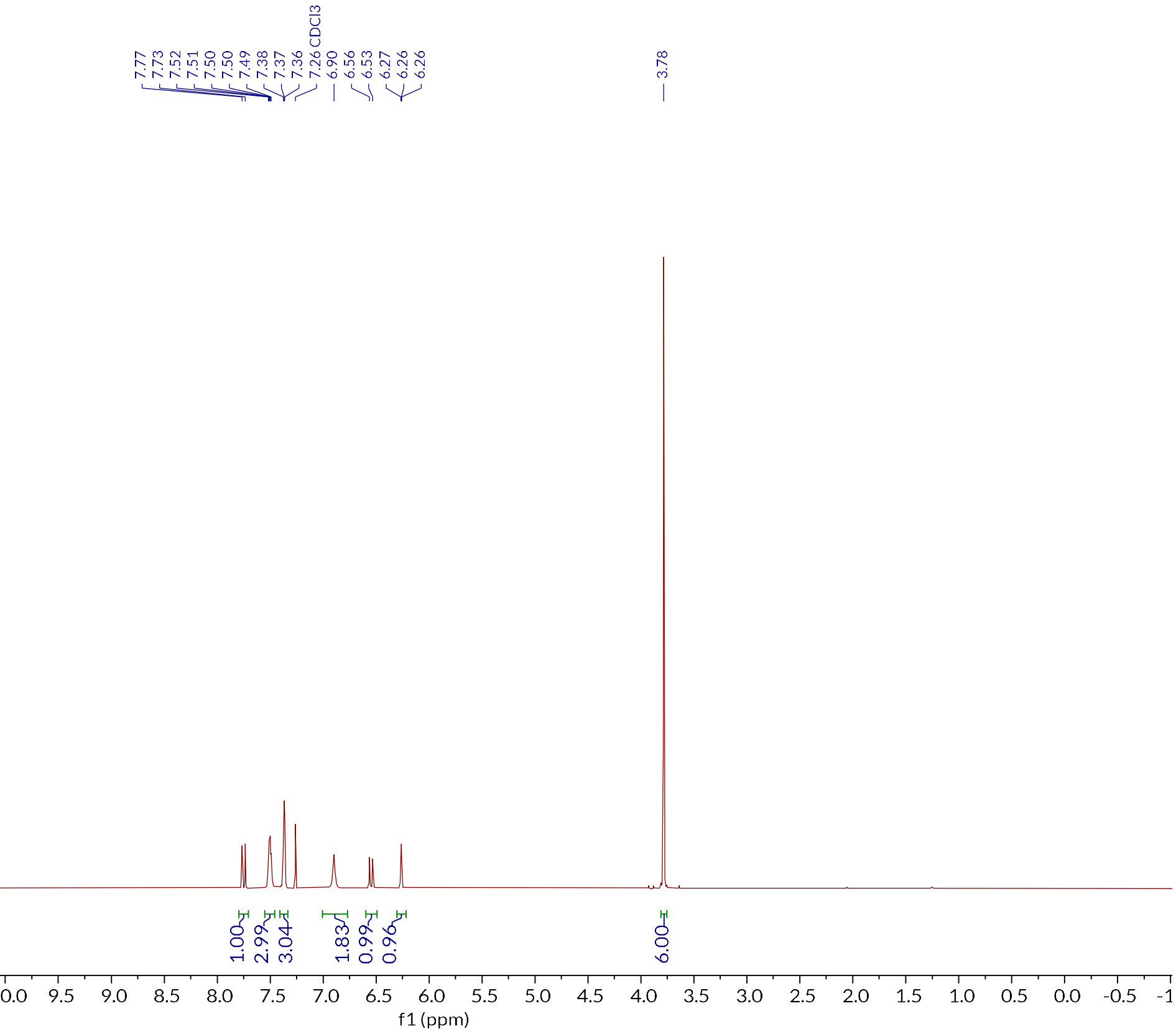

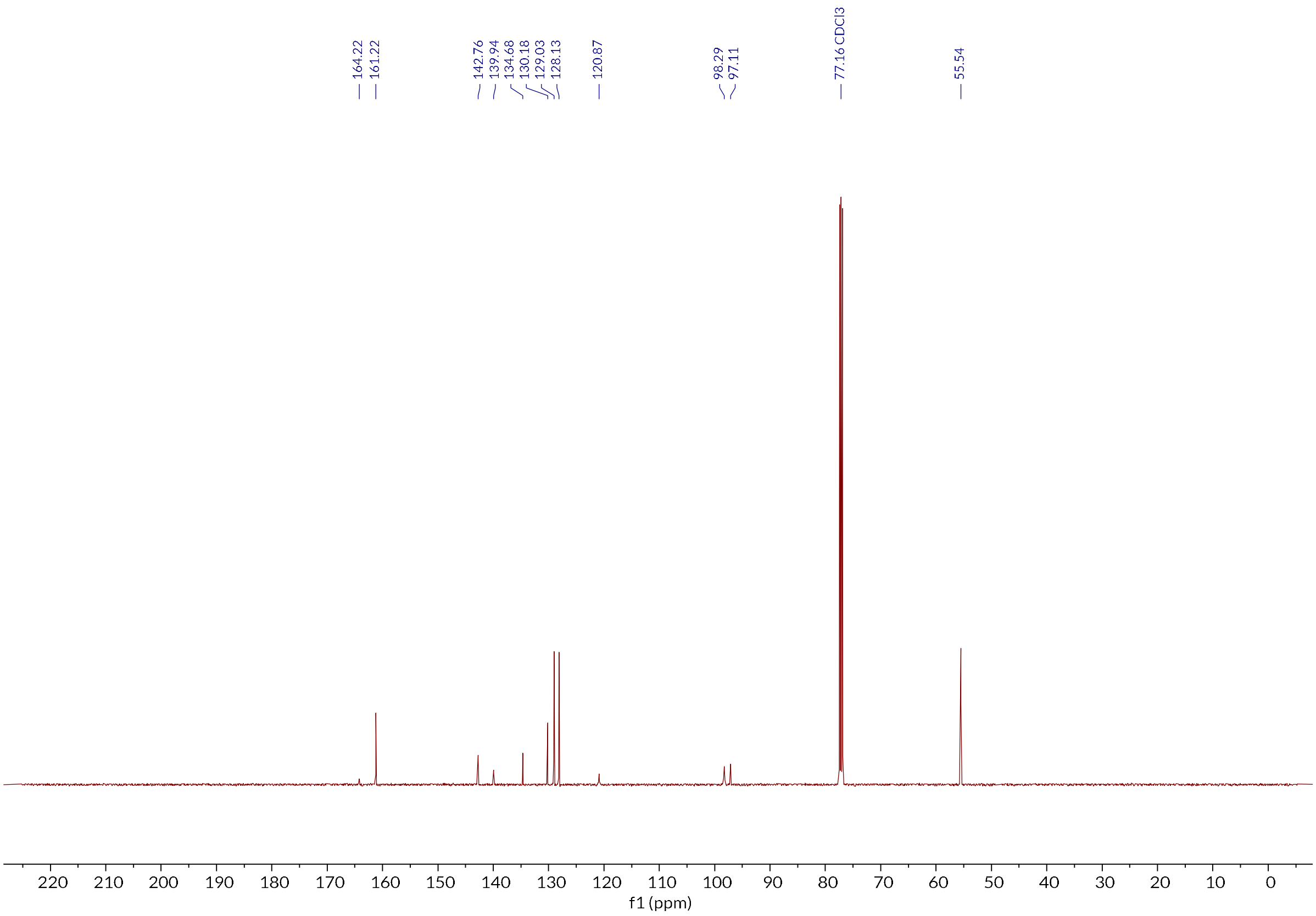

N-Benzyl-*N*-(3,5-dimethoxyphenyl)cinnamamide (1am)

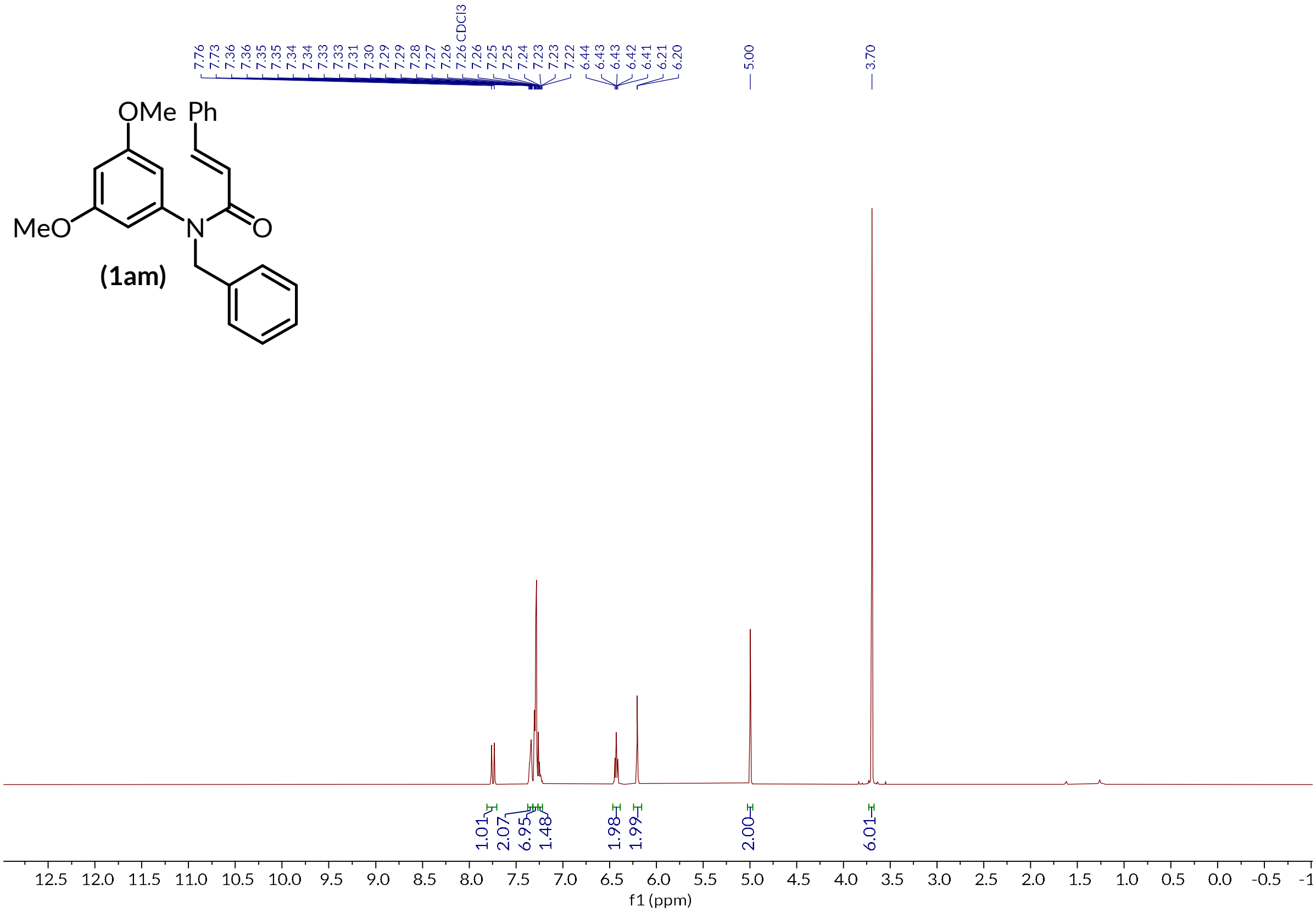

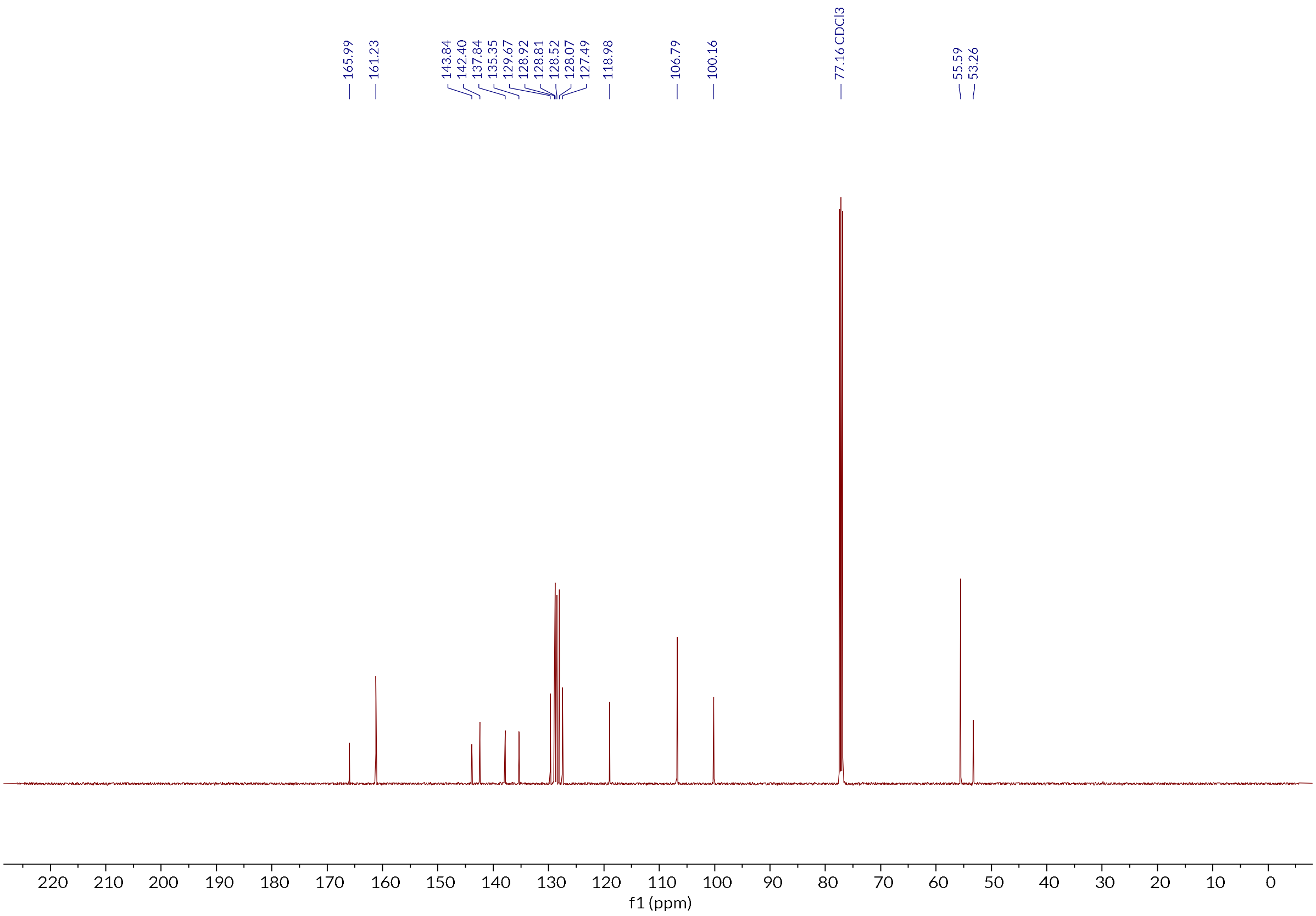

(*E*)-*N*-Benzyl-*N*-(3-(*p*-tolyl)allyl)aniline (1an)

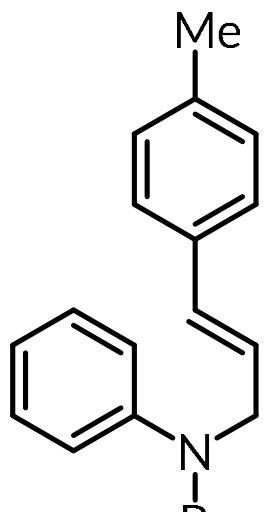

(1an)

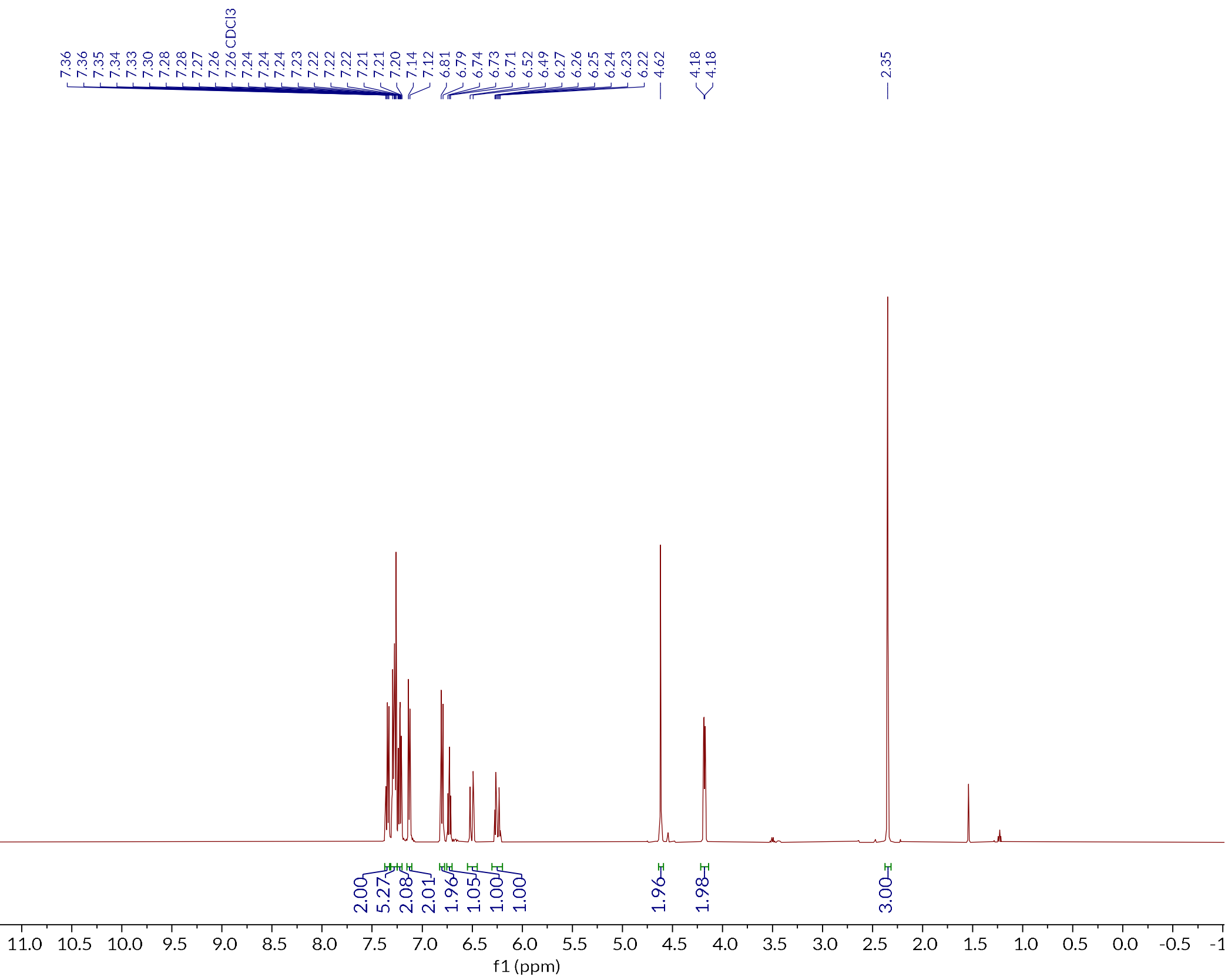

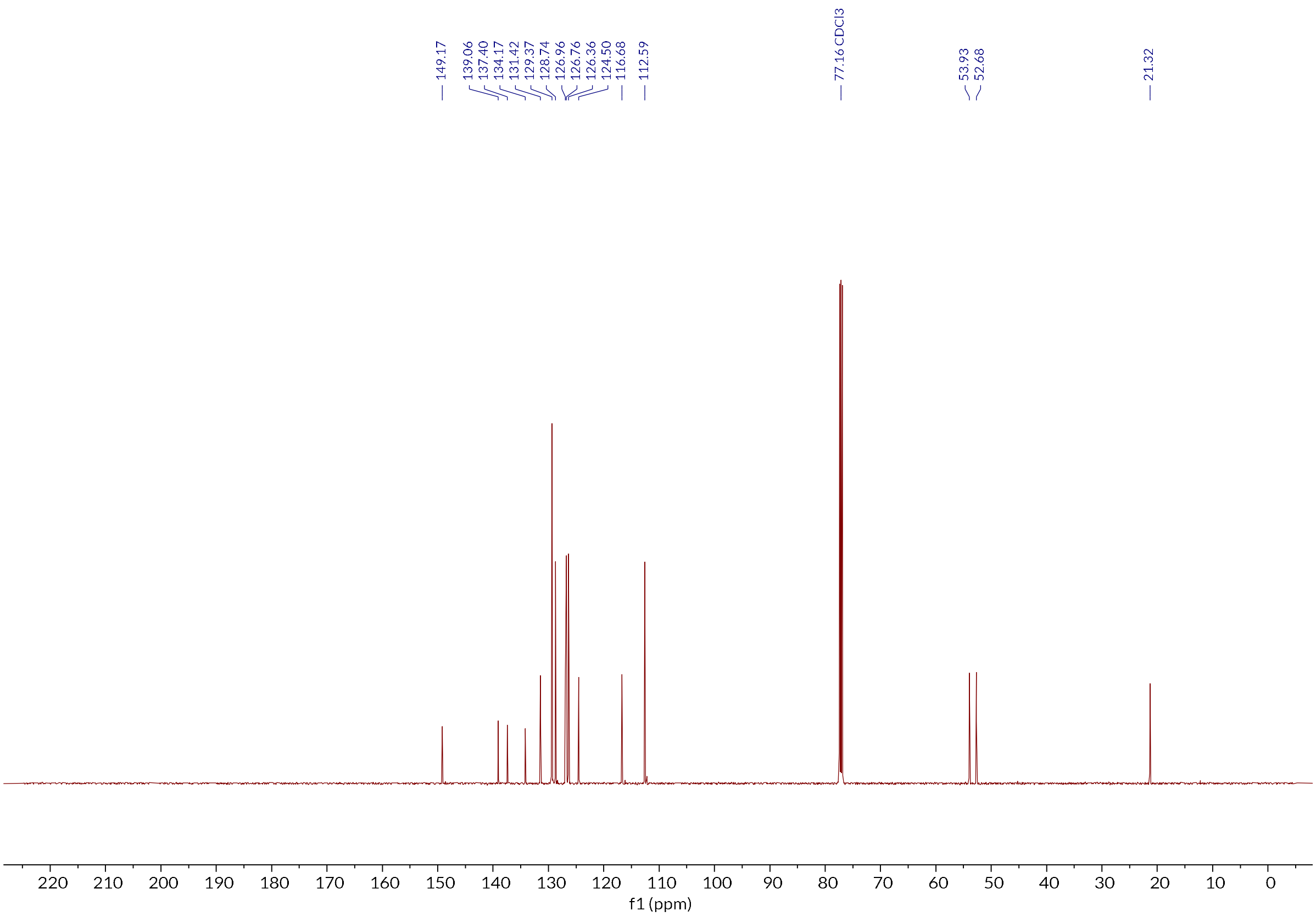

(*E*)-*N*-Benzyl-*N*-(3-(4-methoxyphenyl)allyl)aniline (1ao)

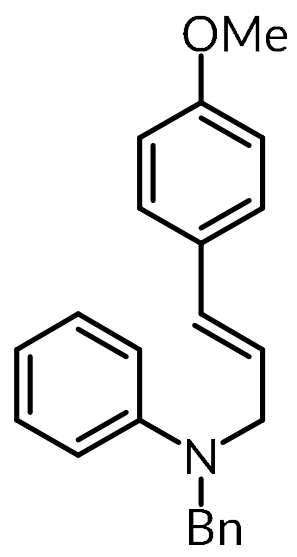

(1ao)

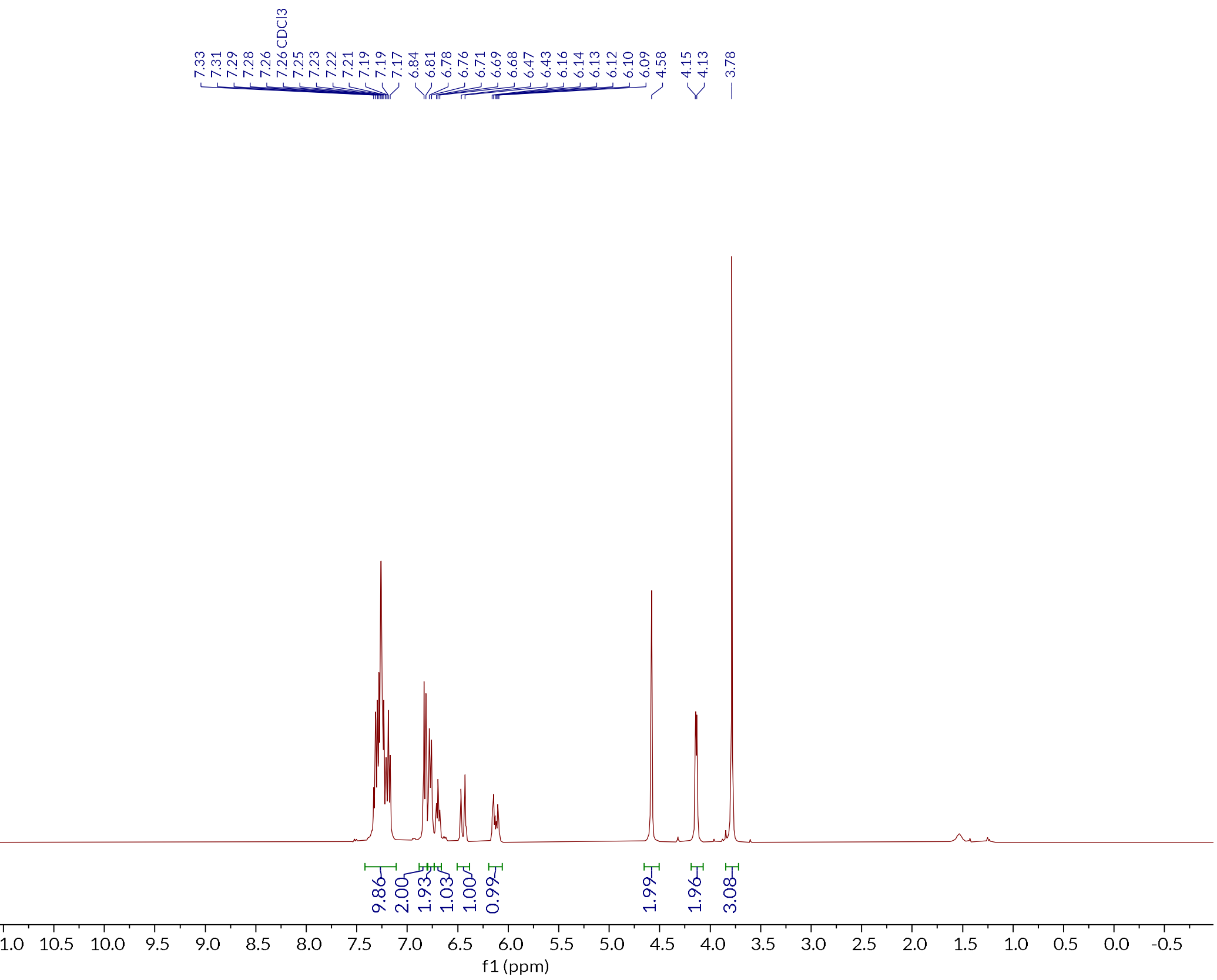

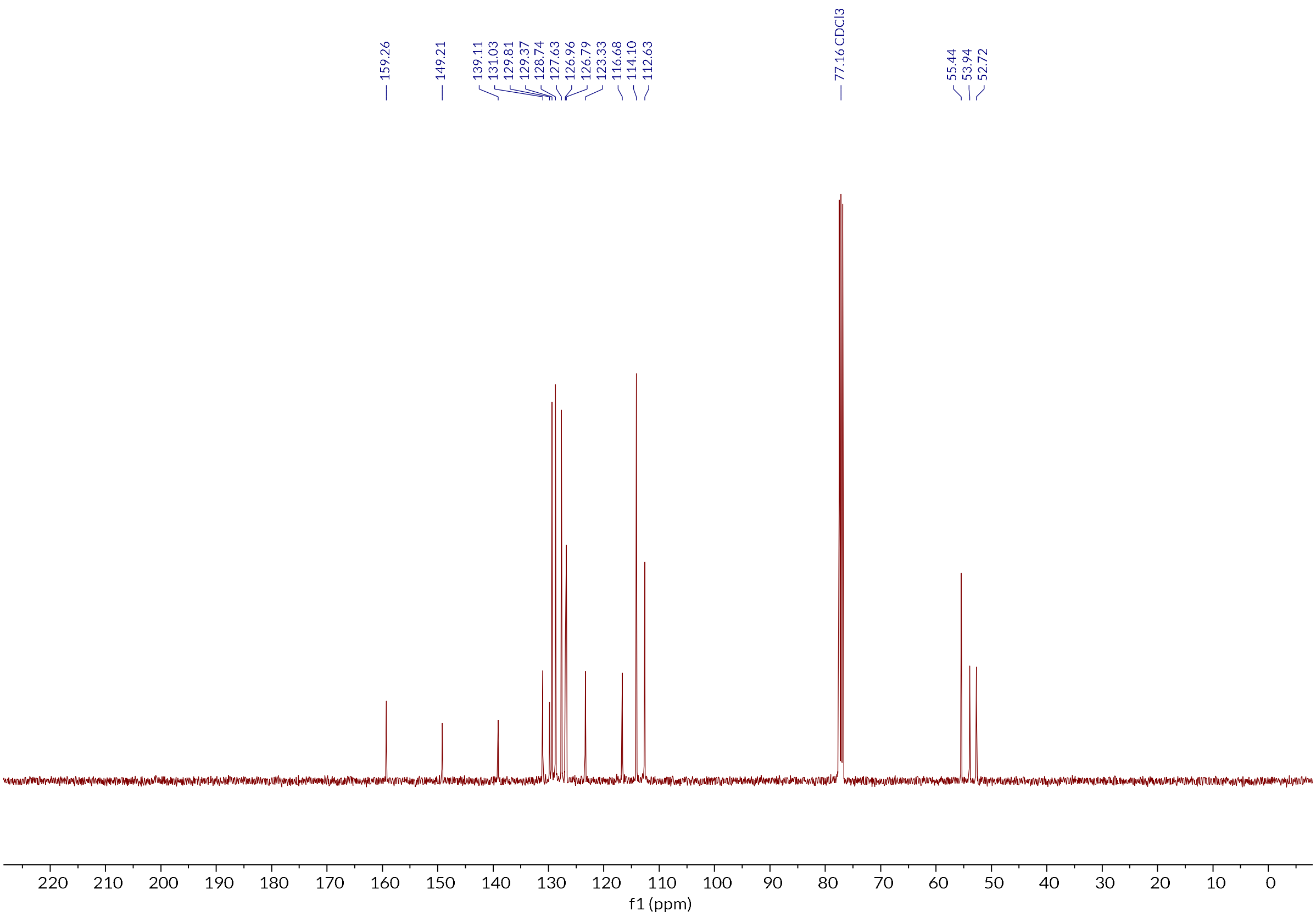

(*E*)-*N*-Benzyl-*N*-(3-(4-fluorophenyl)allyl)aniline (1ap)

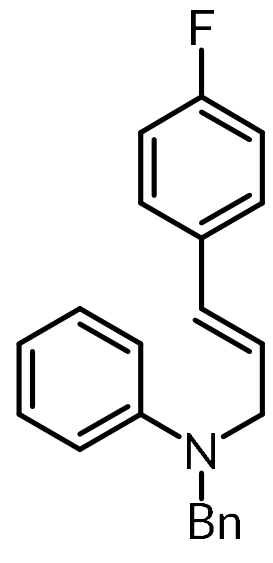

(1ap)

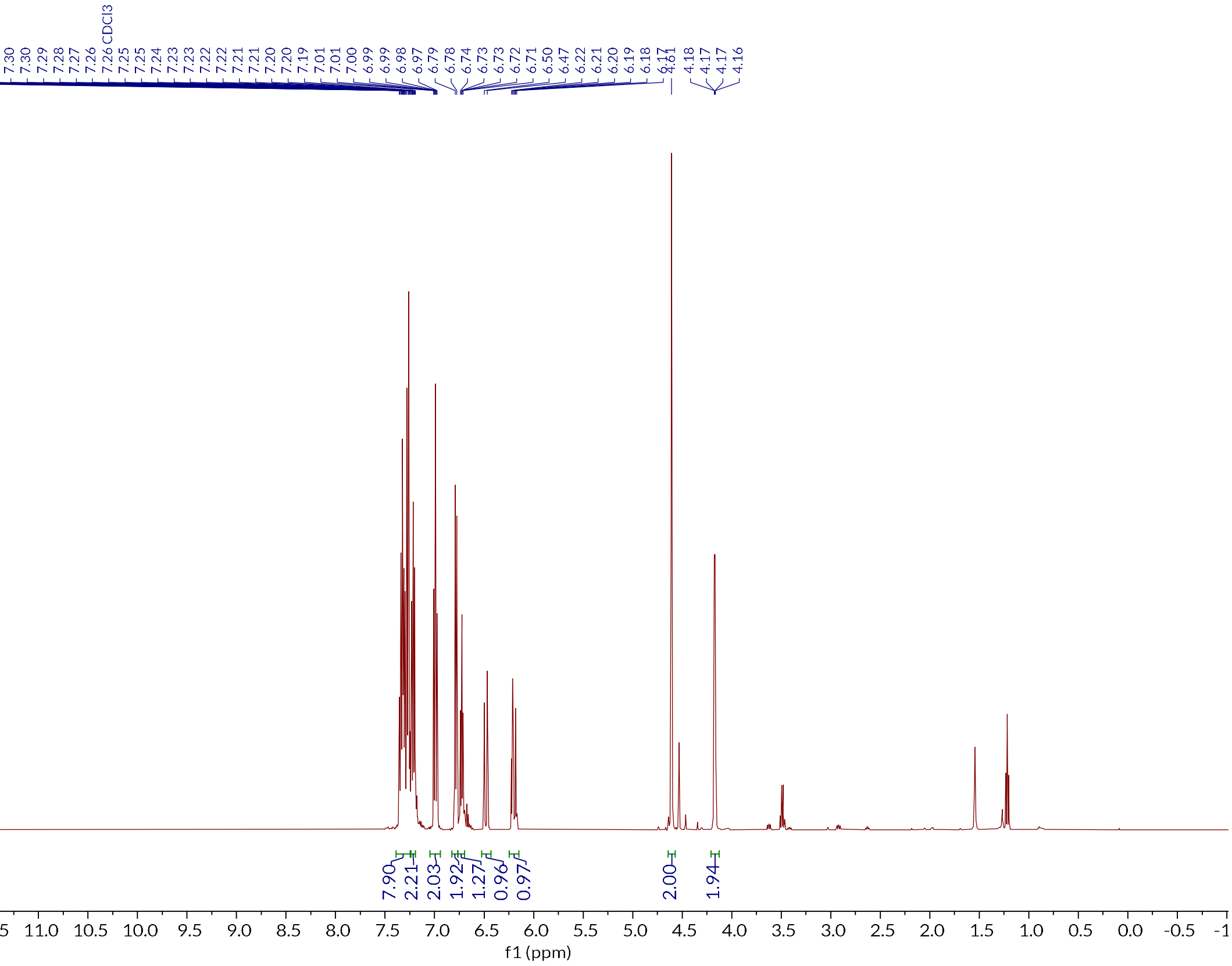

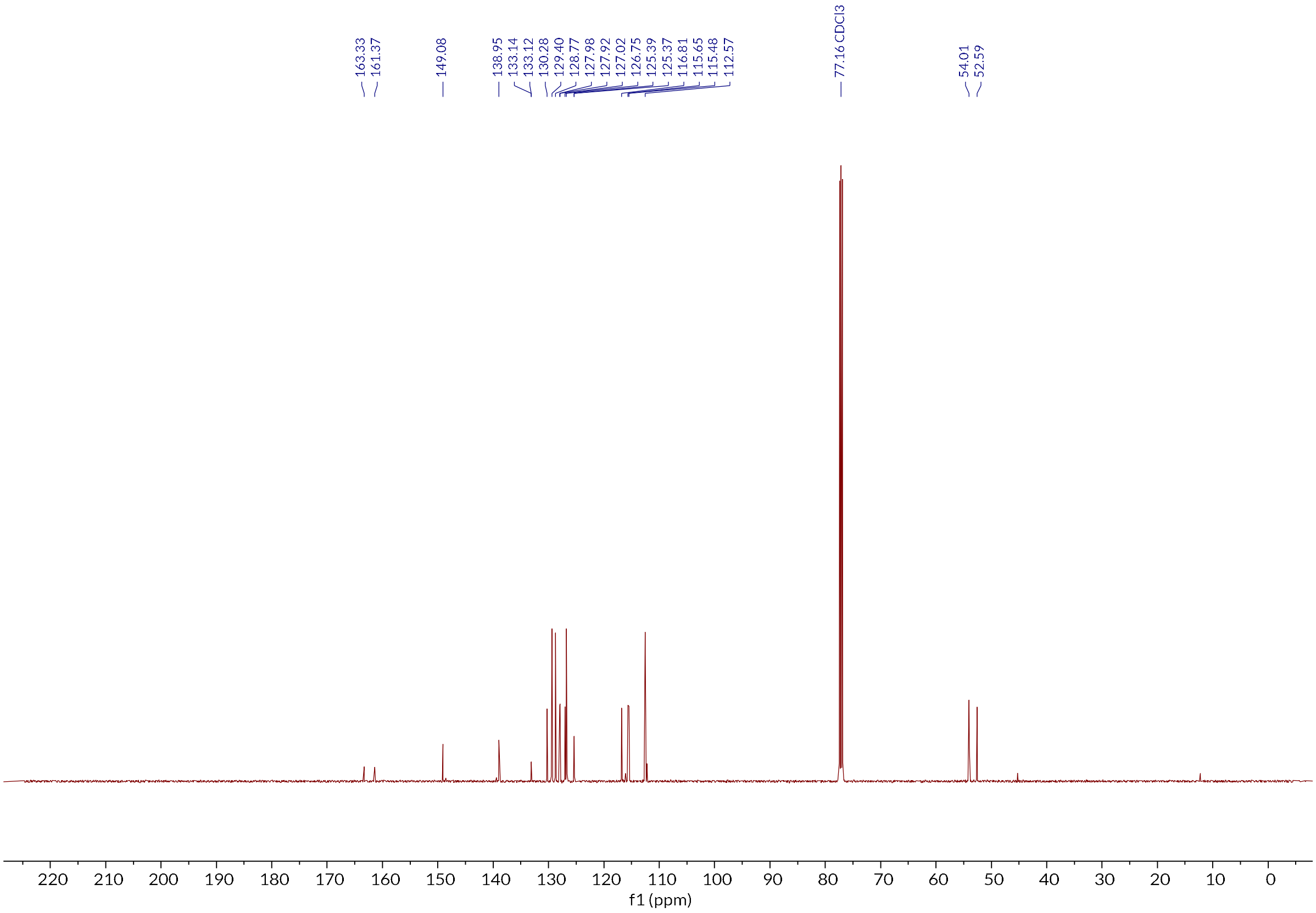

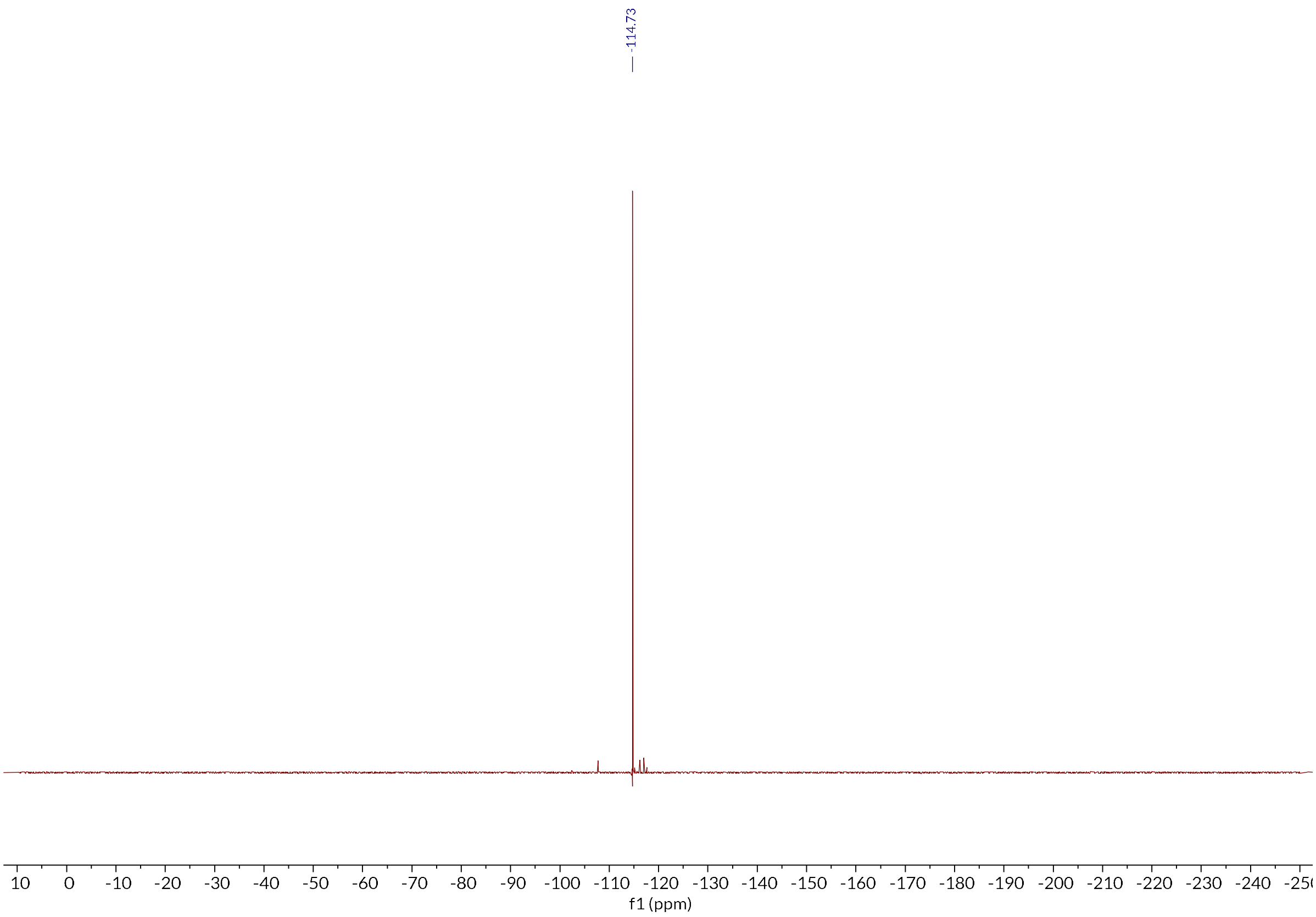
-114.73
10
0
-10
-20
-30
-40
-50
-60
-70
-80
-90
-100
-110
-120
-130
-140
-150
-160
-170
-180
-190
-200
-210
-220
-230
-240
f1 (ppm)

(*E*)-*N*-(3-(4-Bromophenyl)allyl)aniline (SI-06)

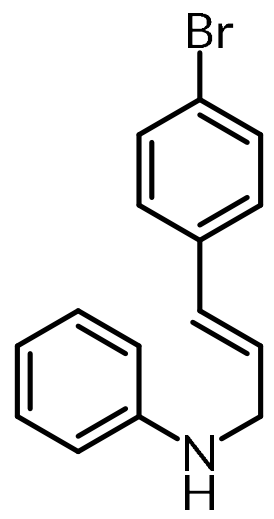

(SI-06)

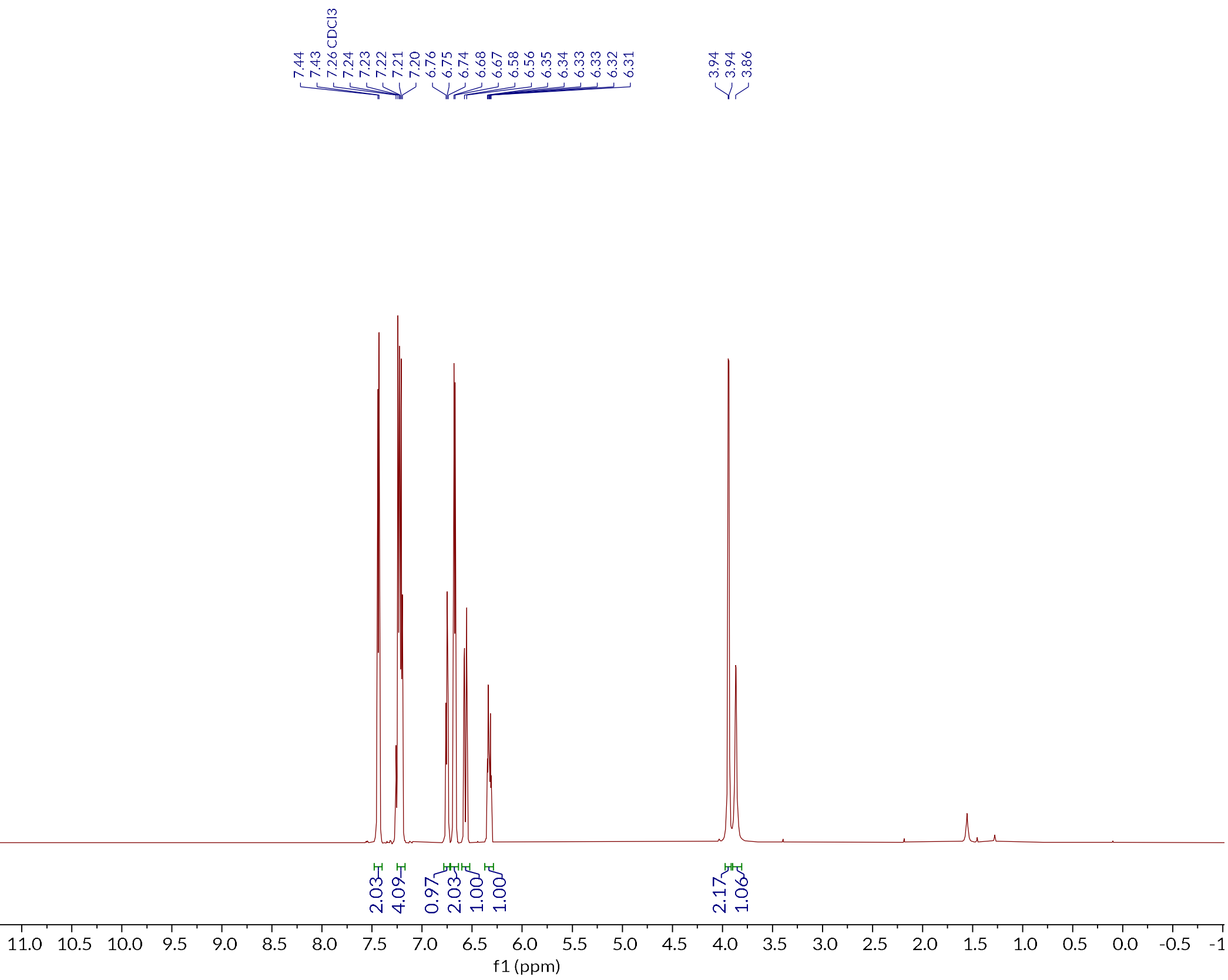

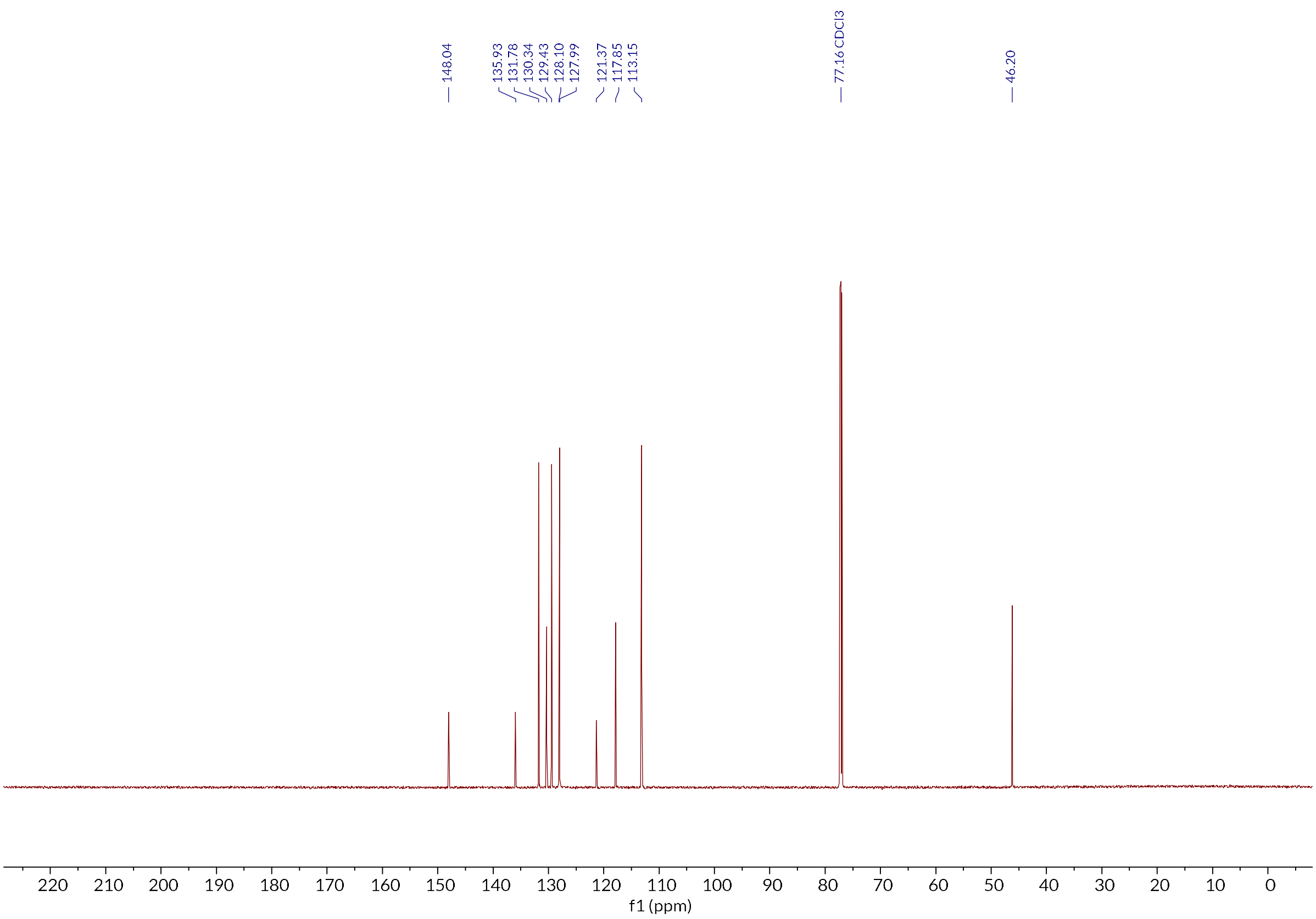

(*E*)-*N*-Benzyl-*N*-(3-(4-bromophenyl)allyl)aniline (1aq)

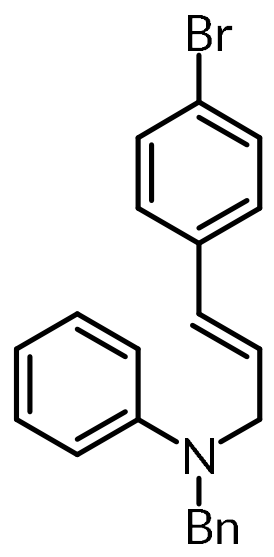

(1aq)

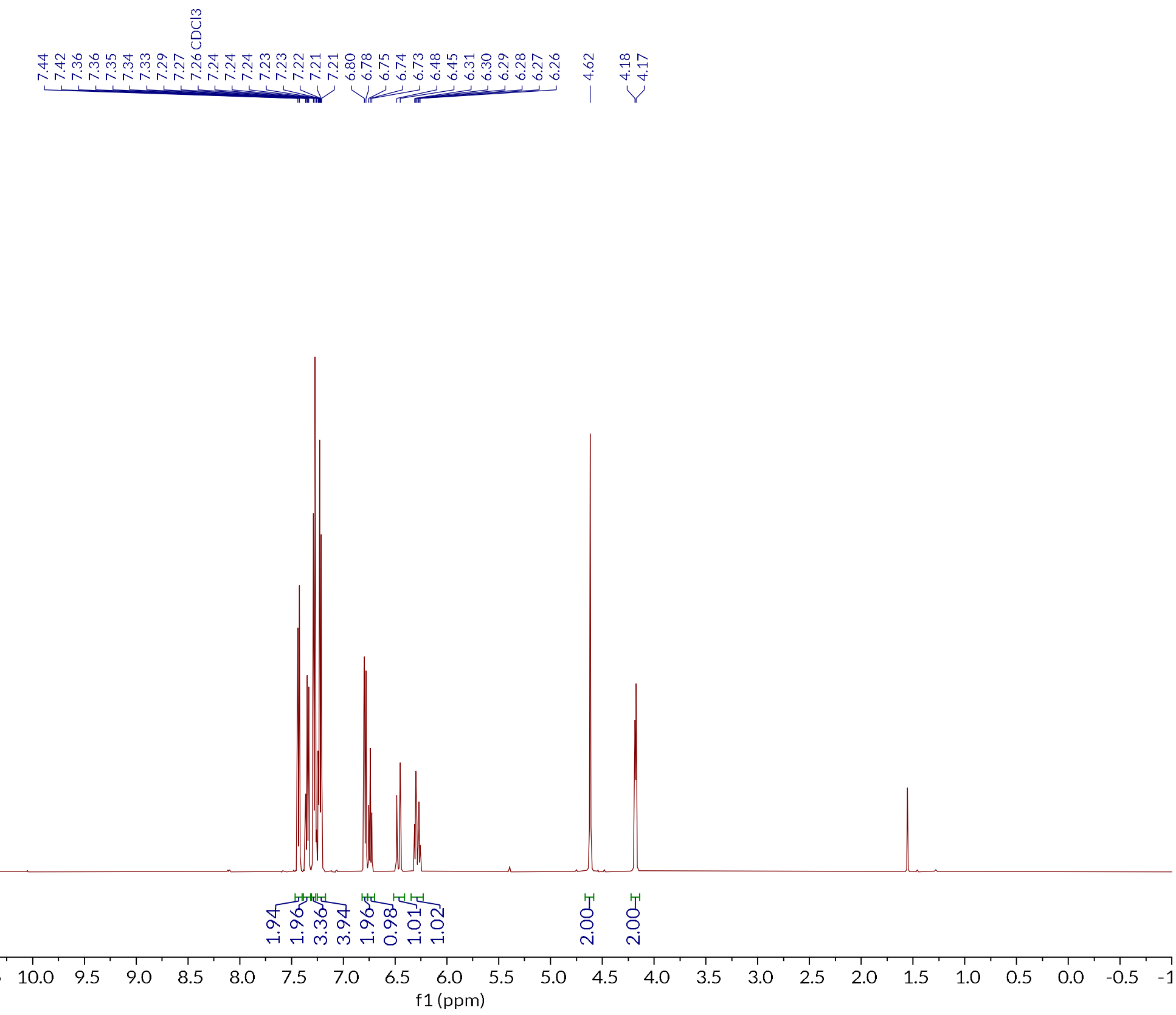

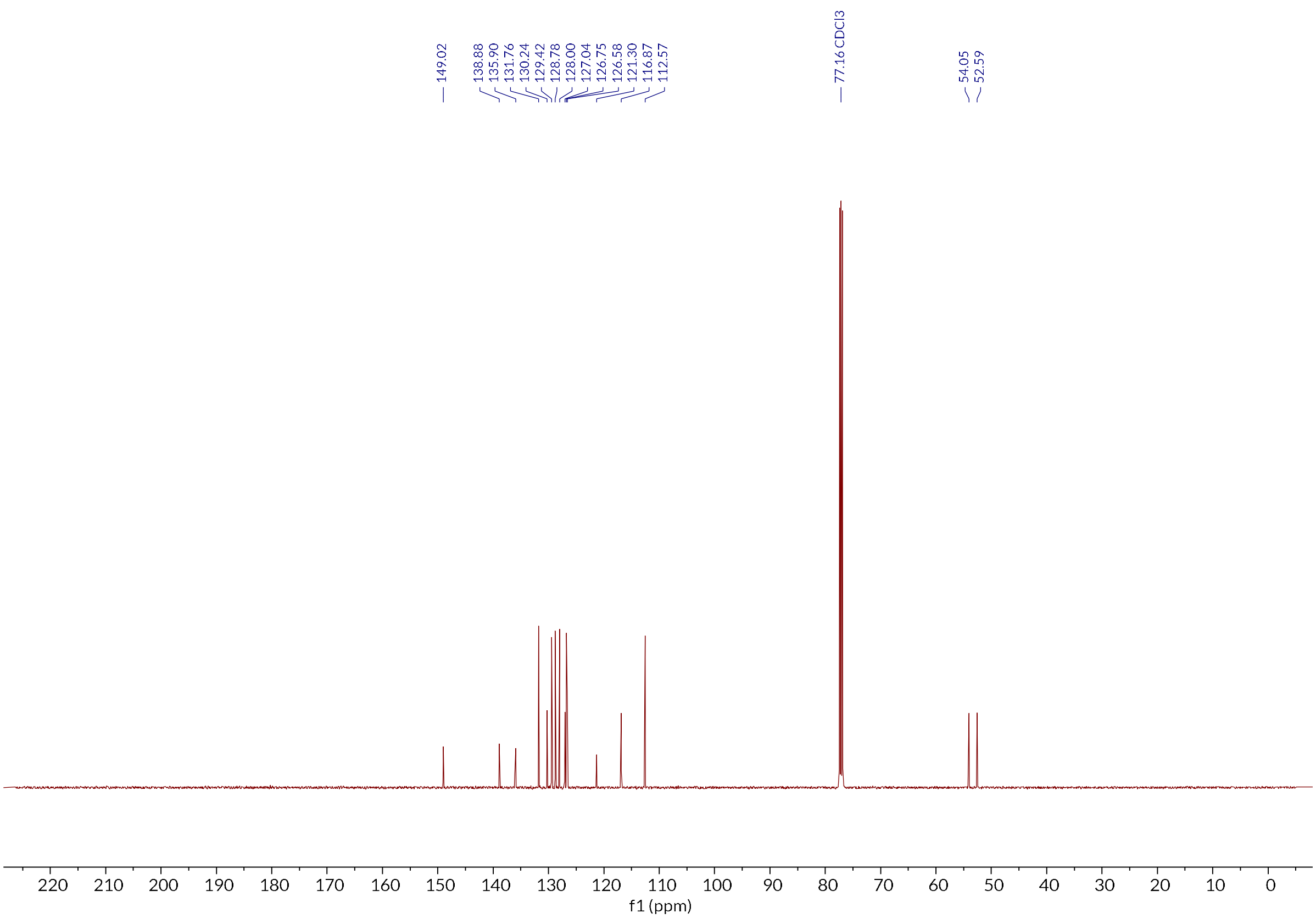

(*E*)-*N*-Benzyl-*N*- (3-(4-(trifluoromethyl)phenyl)allyl)aniline (1ar)

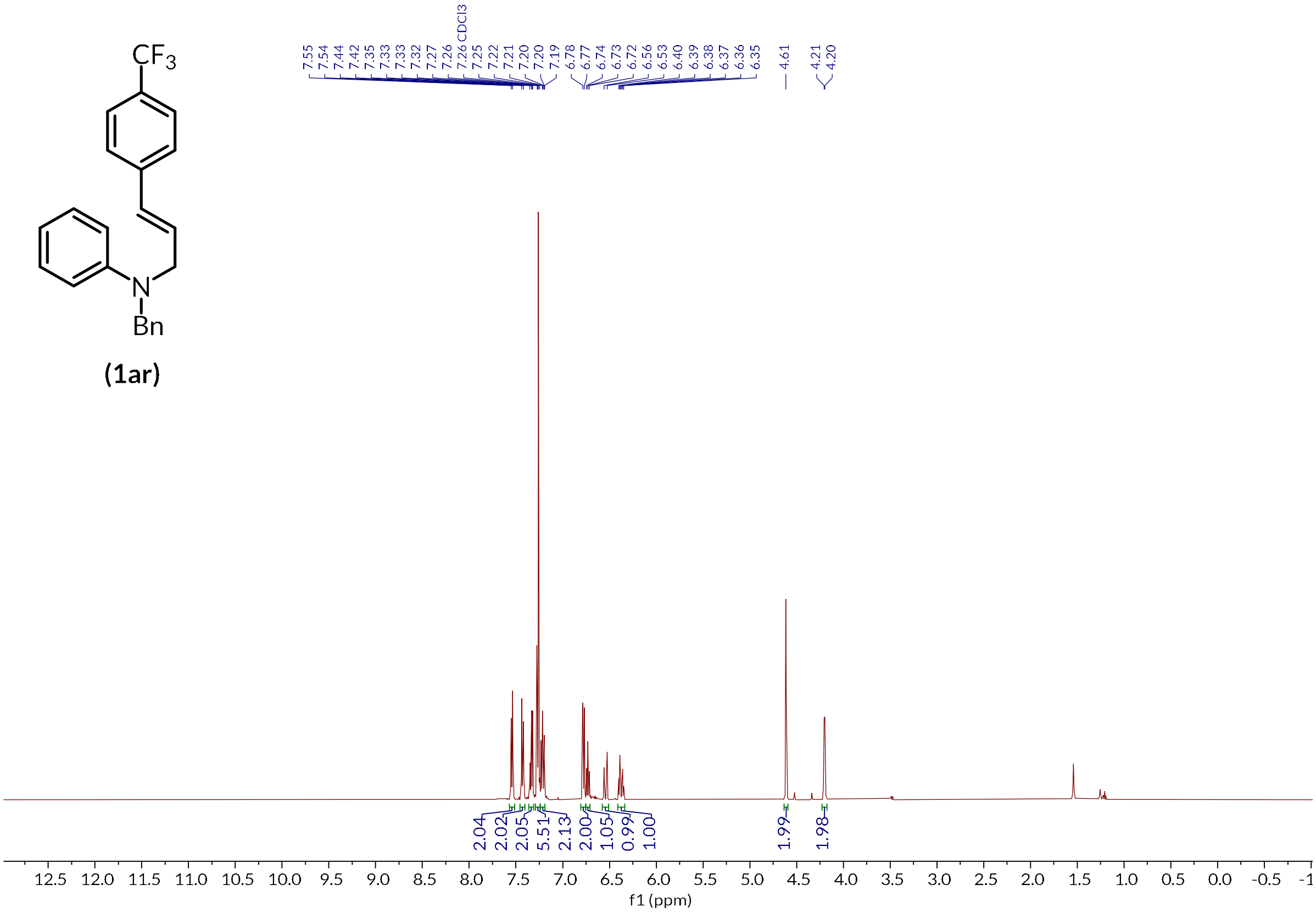

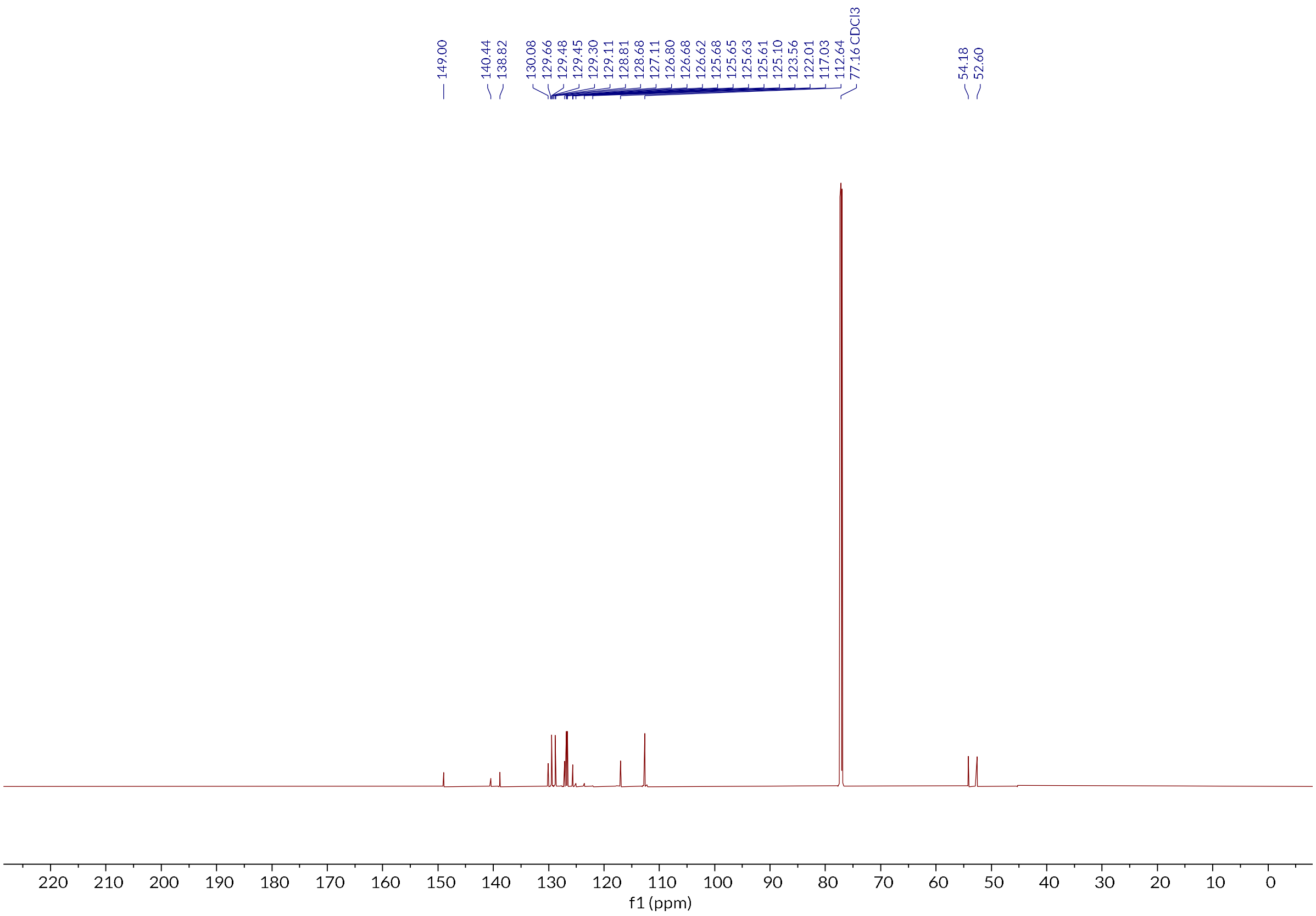

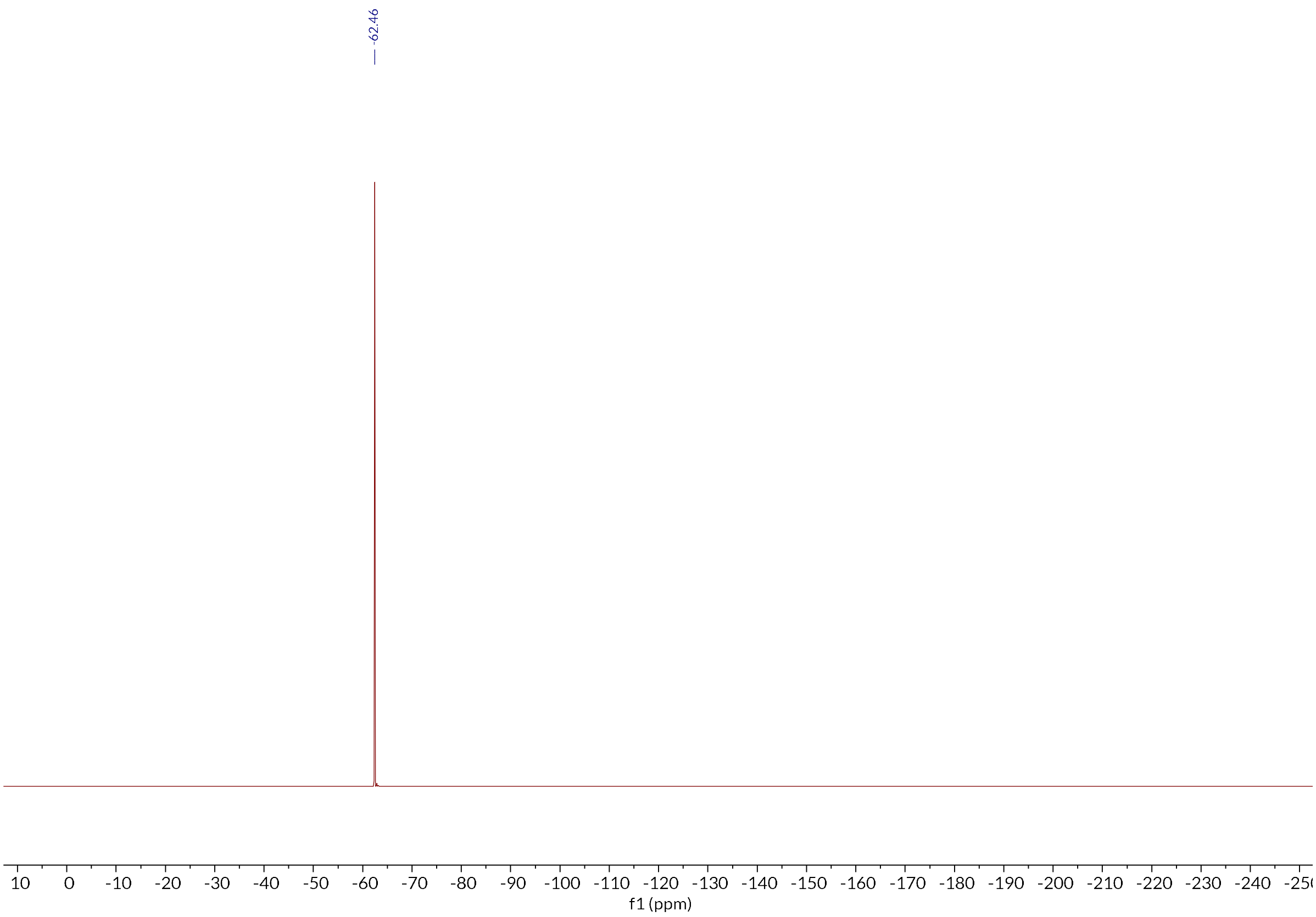
-62.46
10
0
-10
-20
-30
-40
-50
-60
-70
-80
-90
-100
-110
-120
-130
-140
-150
-160
-170
-180
-190
-200
-210
-220
-230
-240
f1 (ppm)

Methyl (*E*)-3-(3-oxoprop-1-en-1-yl)benzoate (SI-07)

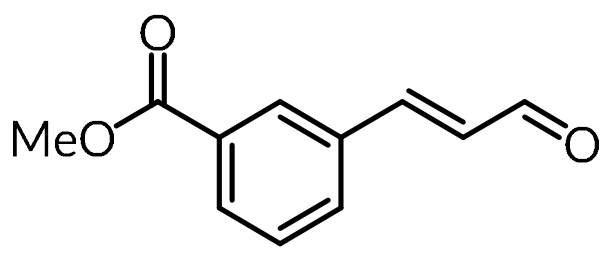

(SI-07)

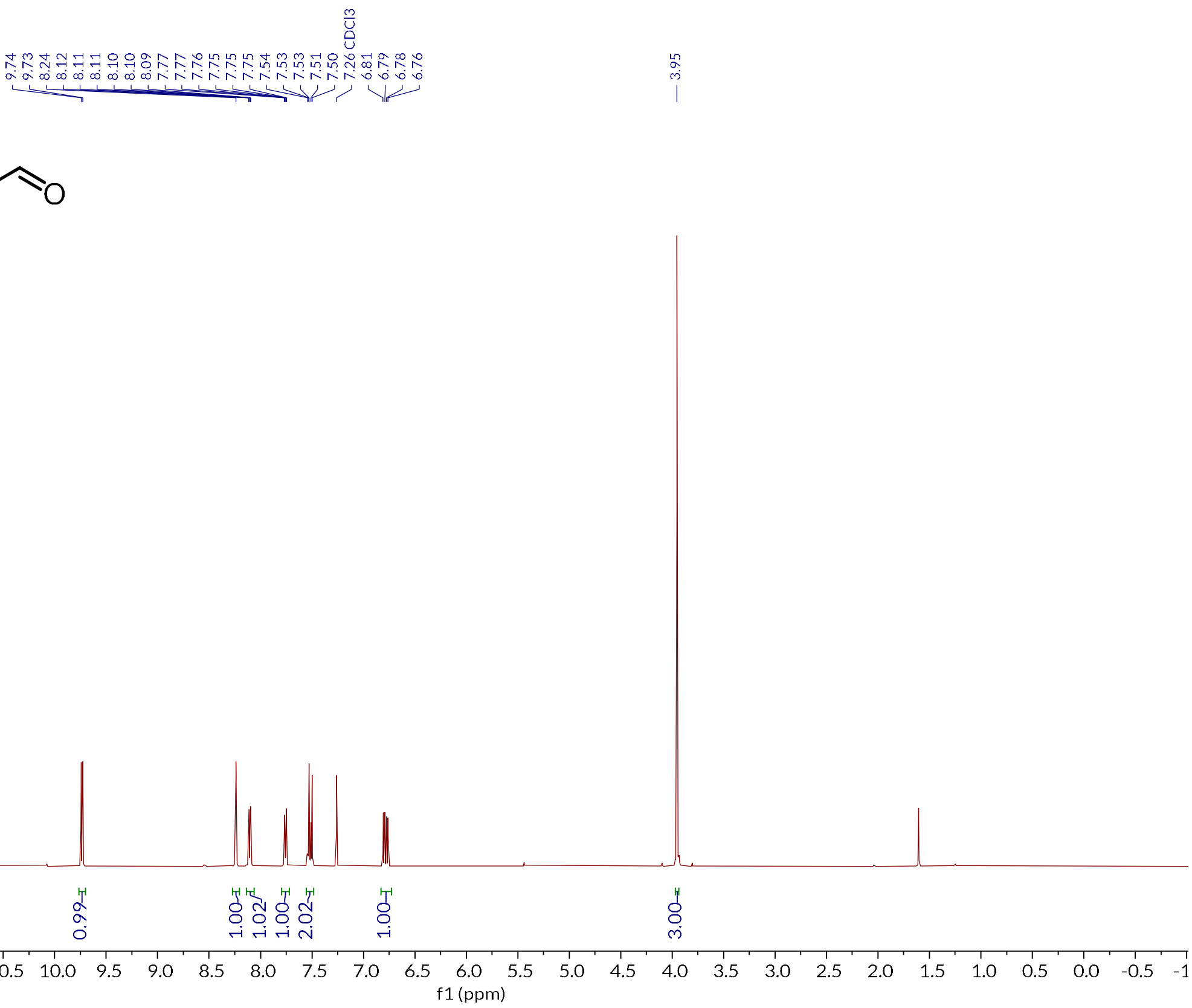

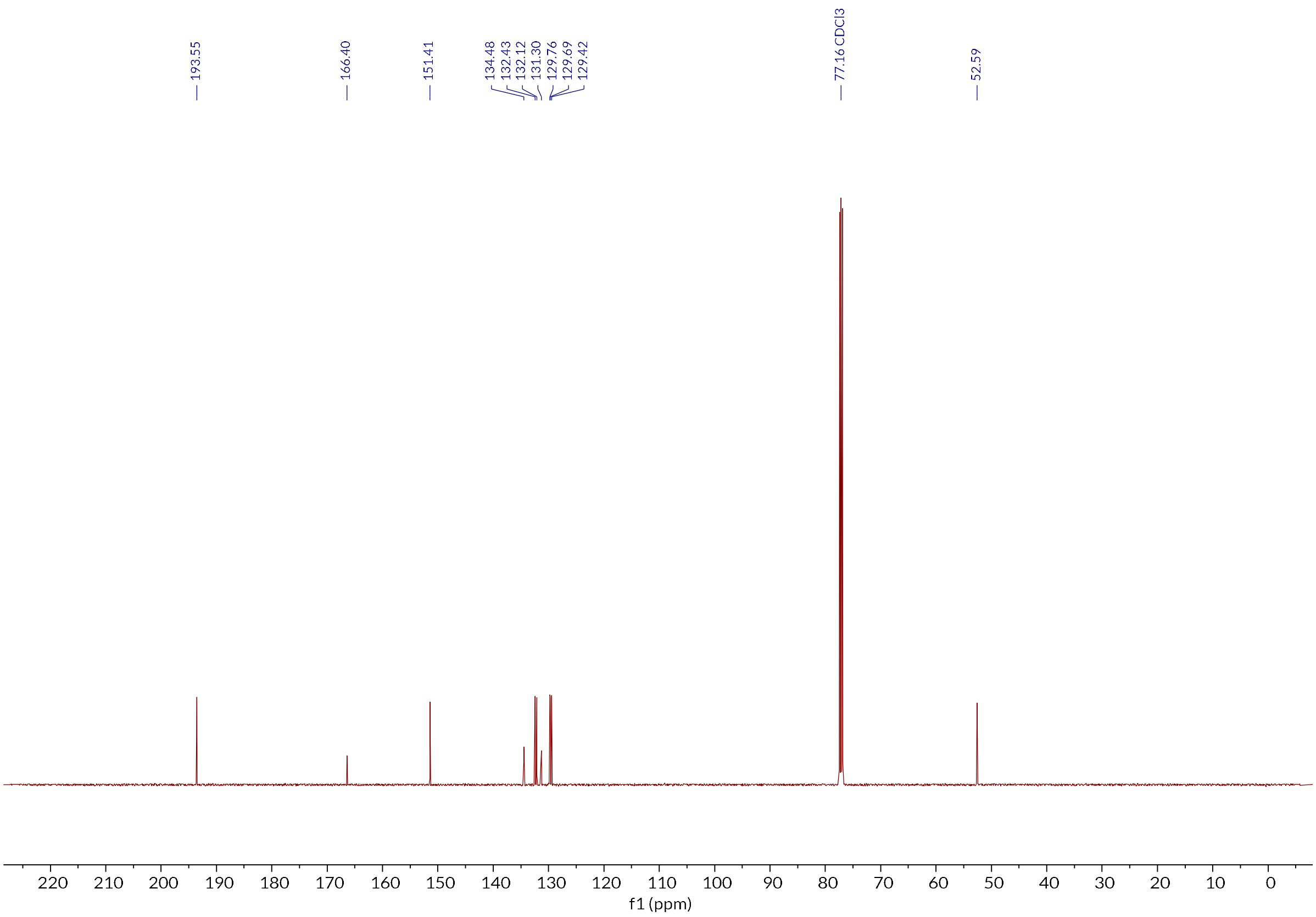

Methyl (*E*)-3-(3-(phenylamino)prop-1-en-1-yl)benzoate (SI-08)

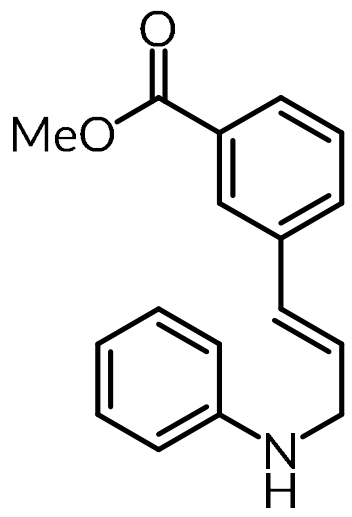

(SI-08)

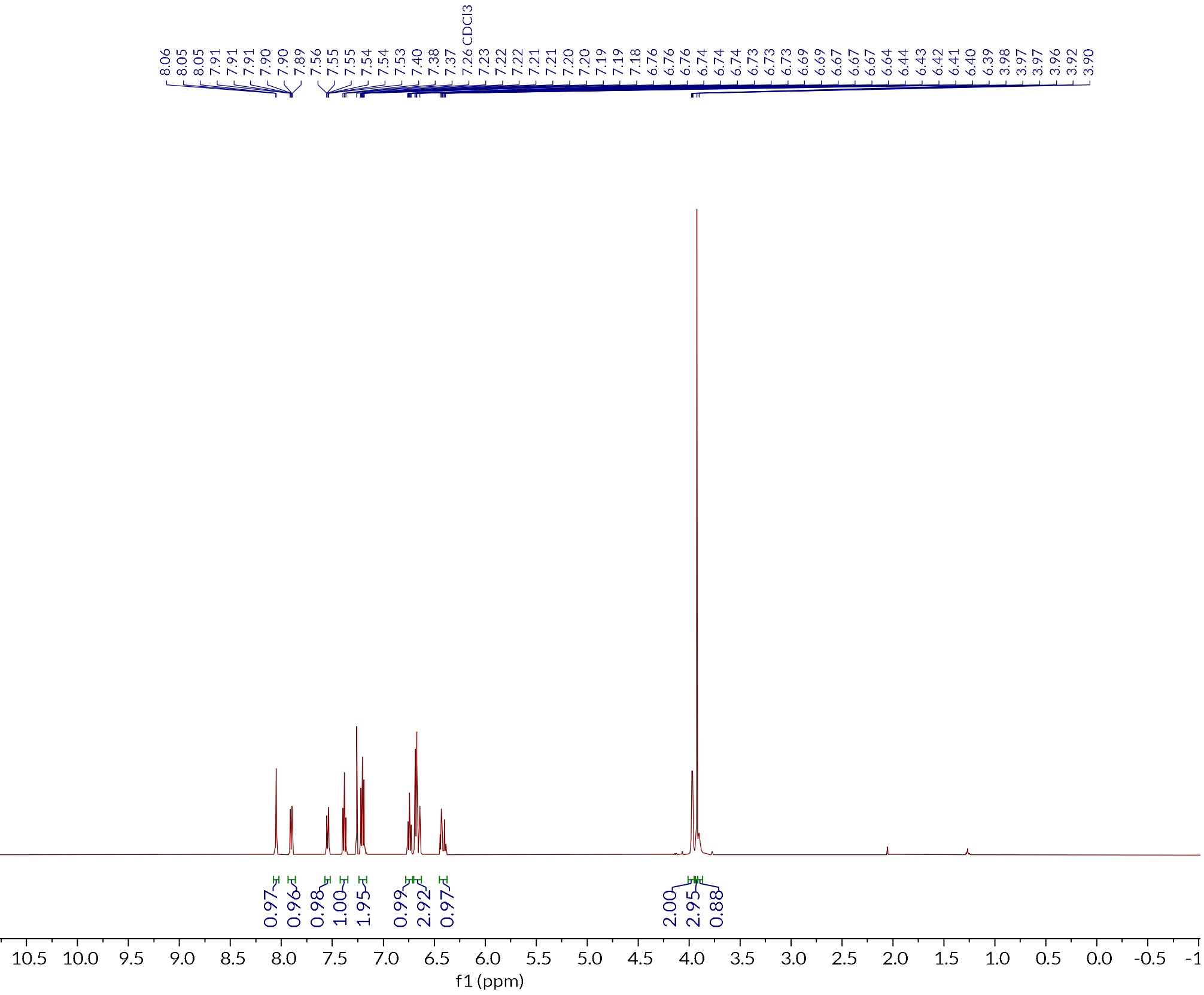

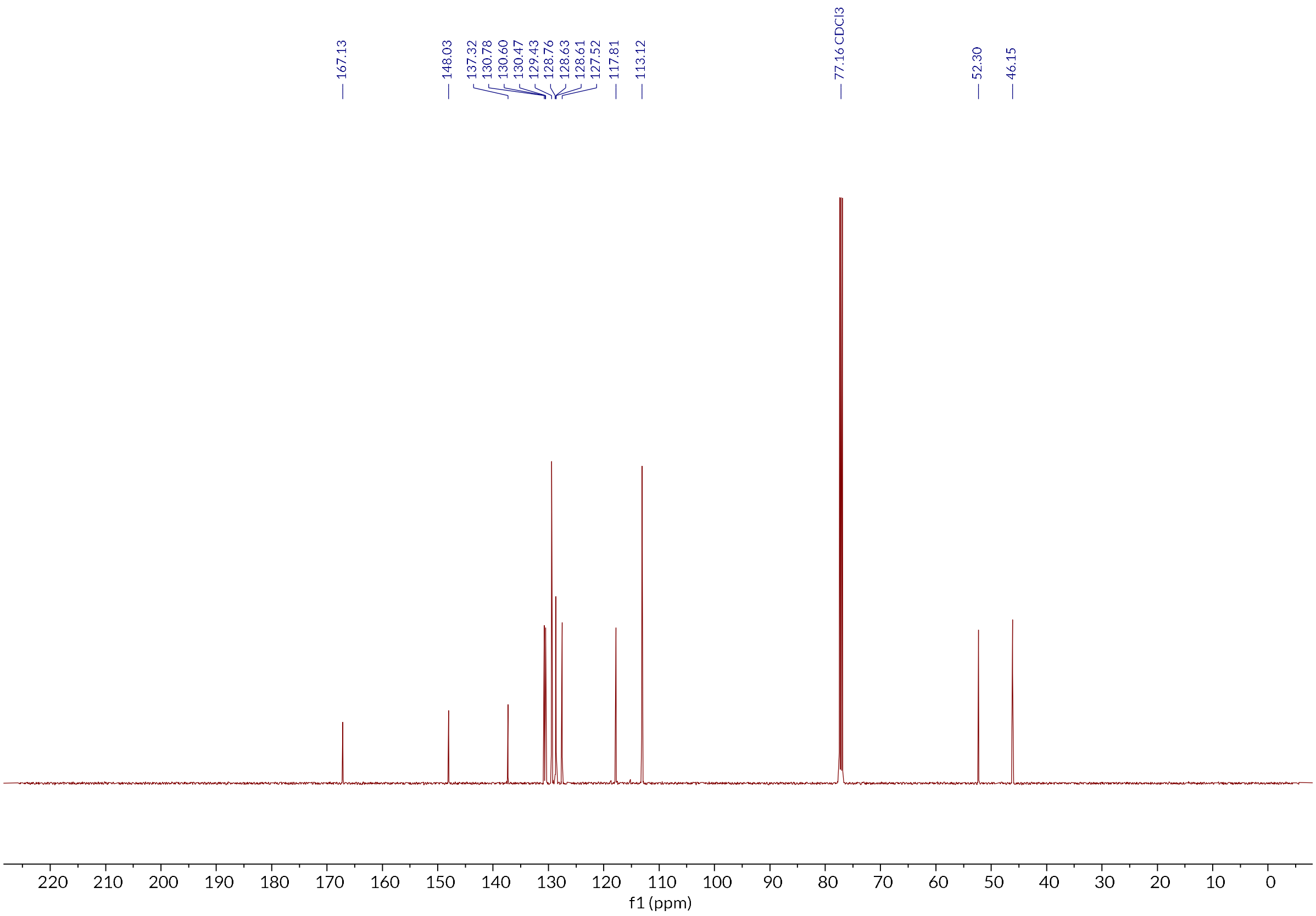

Methyl (*E*)-3-(3-(benzyl(phenyl)amino)prop-1-en-1-yl)benzoate (1as)

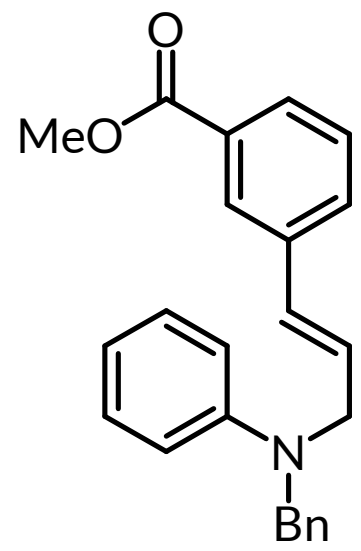

(1as)

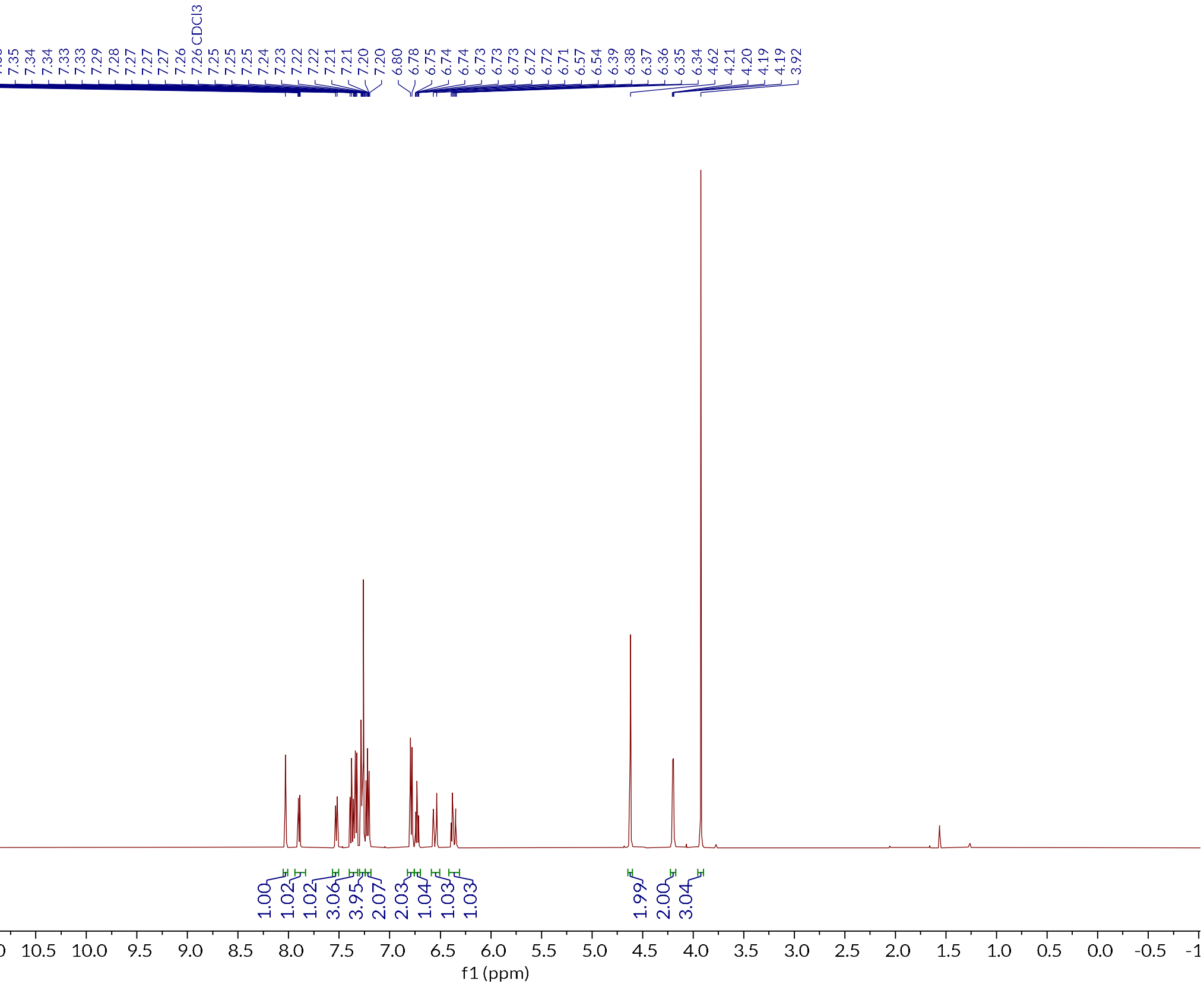

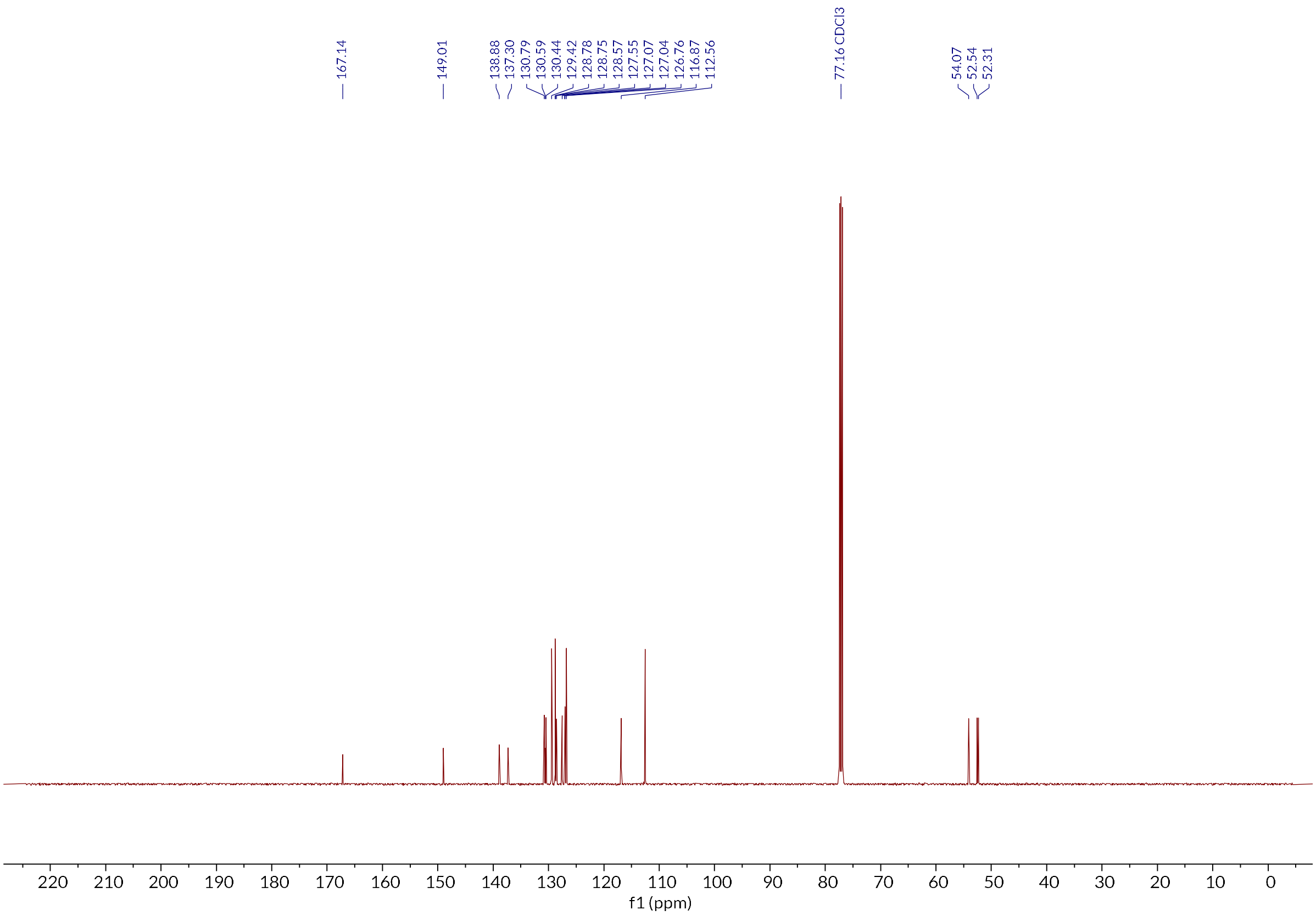

(*E*)-3-(3,5-Di-*tert*-butylphenyl)acrylaldehyde (SI-09)

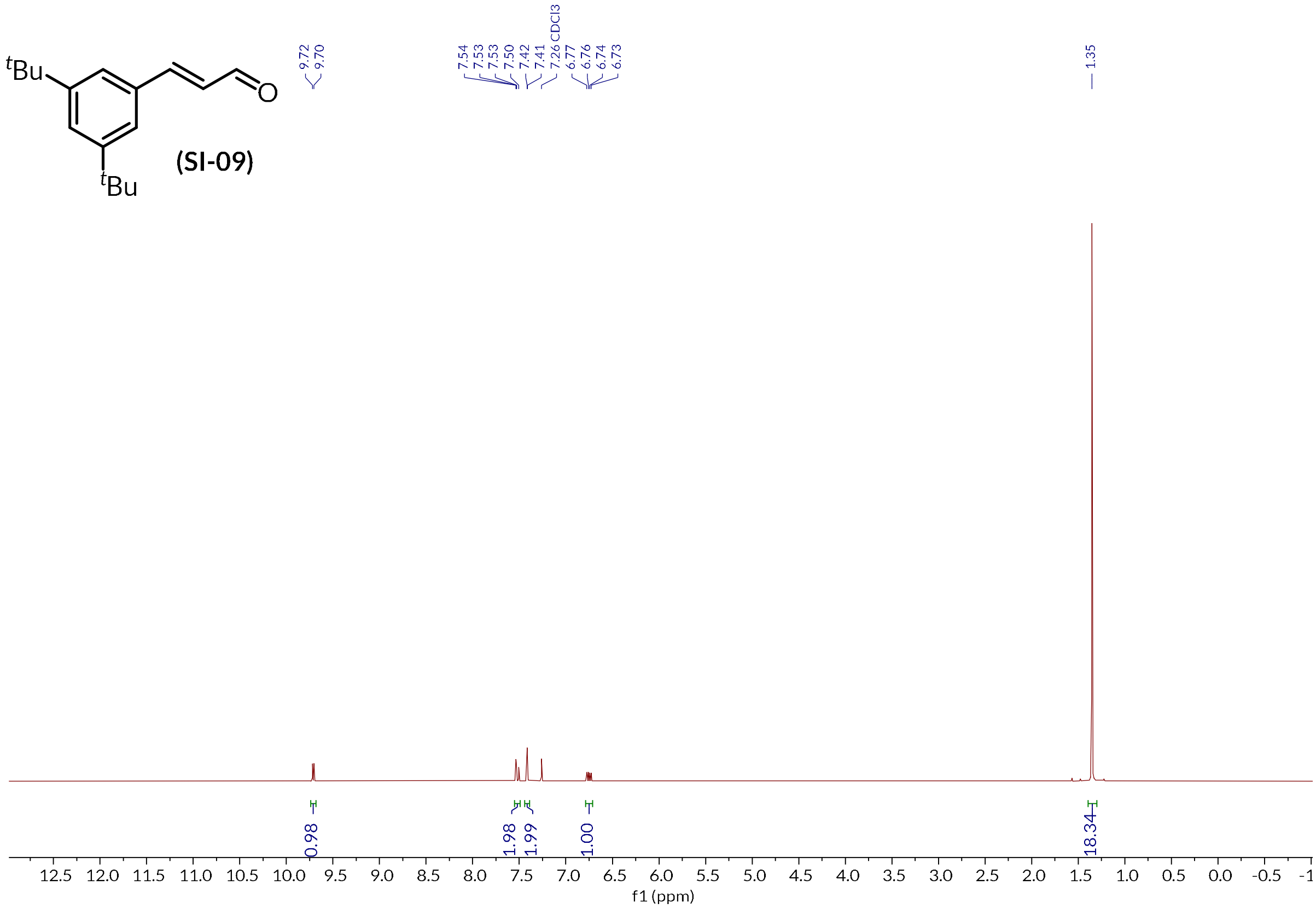

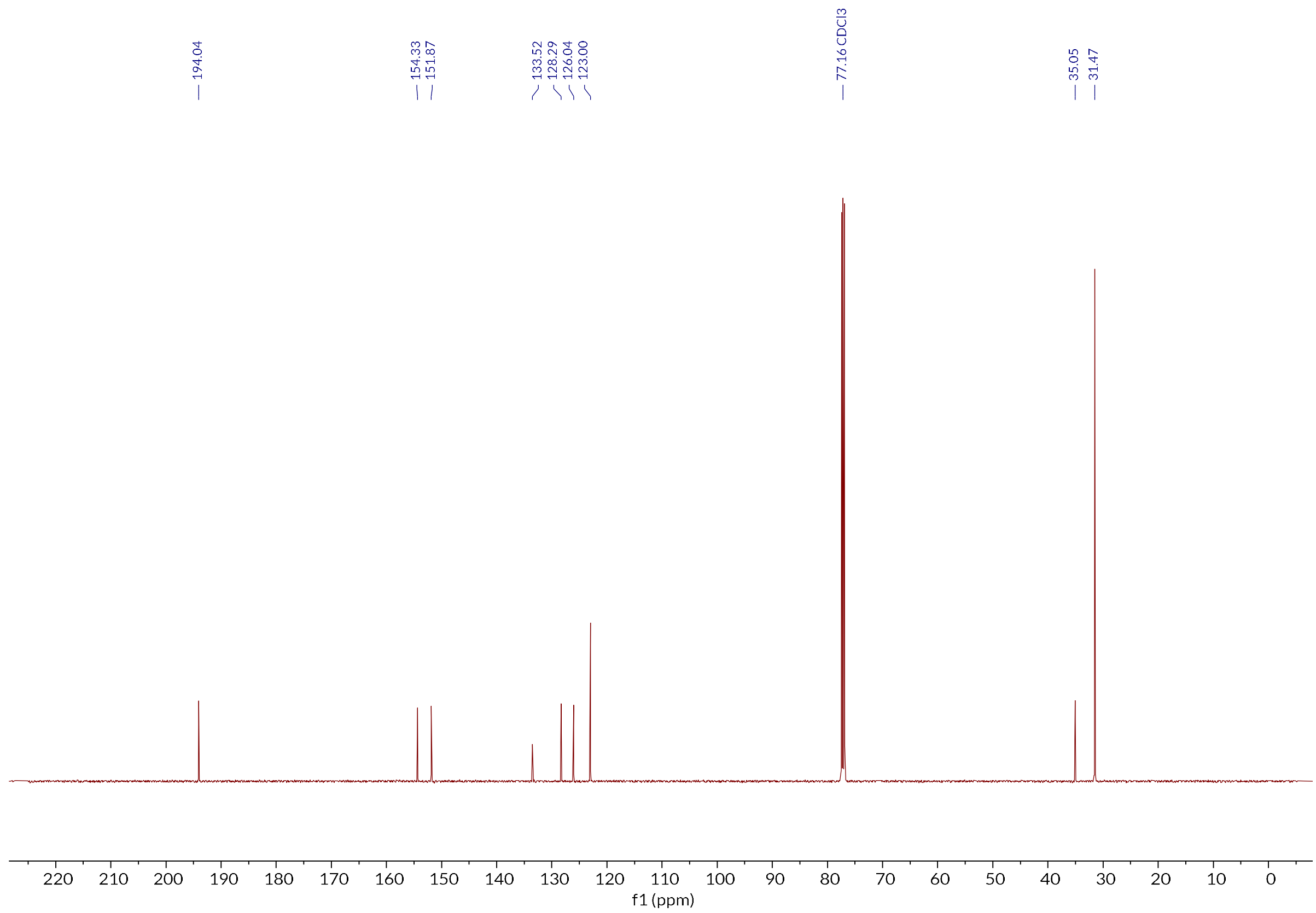

(*E*)-*N*-(3-(3,5-Di-*tert*-butylphenyl)allyl)aniline (SI-10)

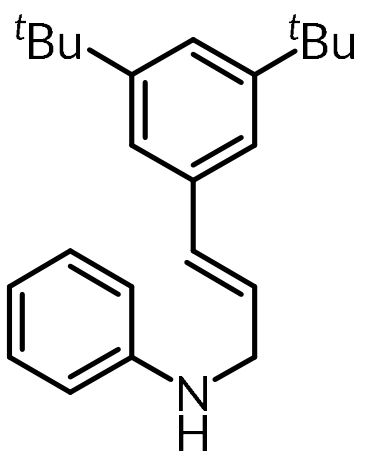

(SI-10)

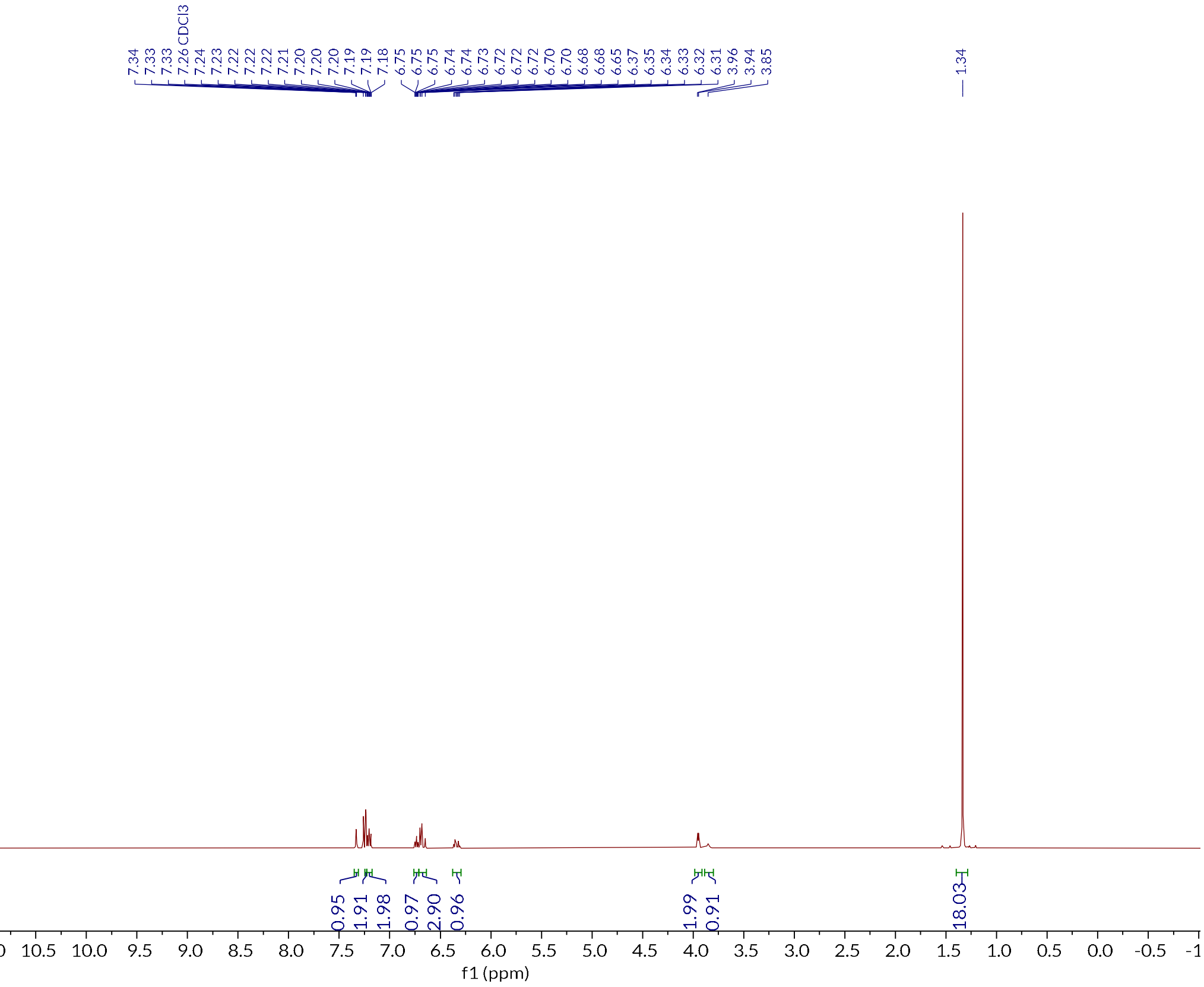

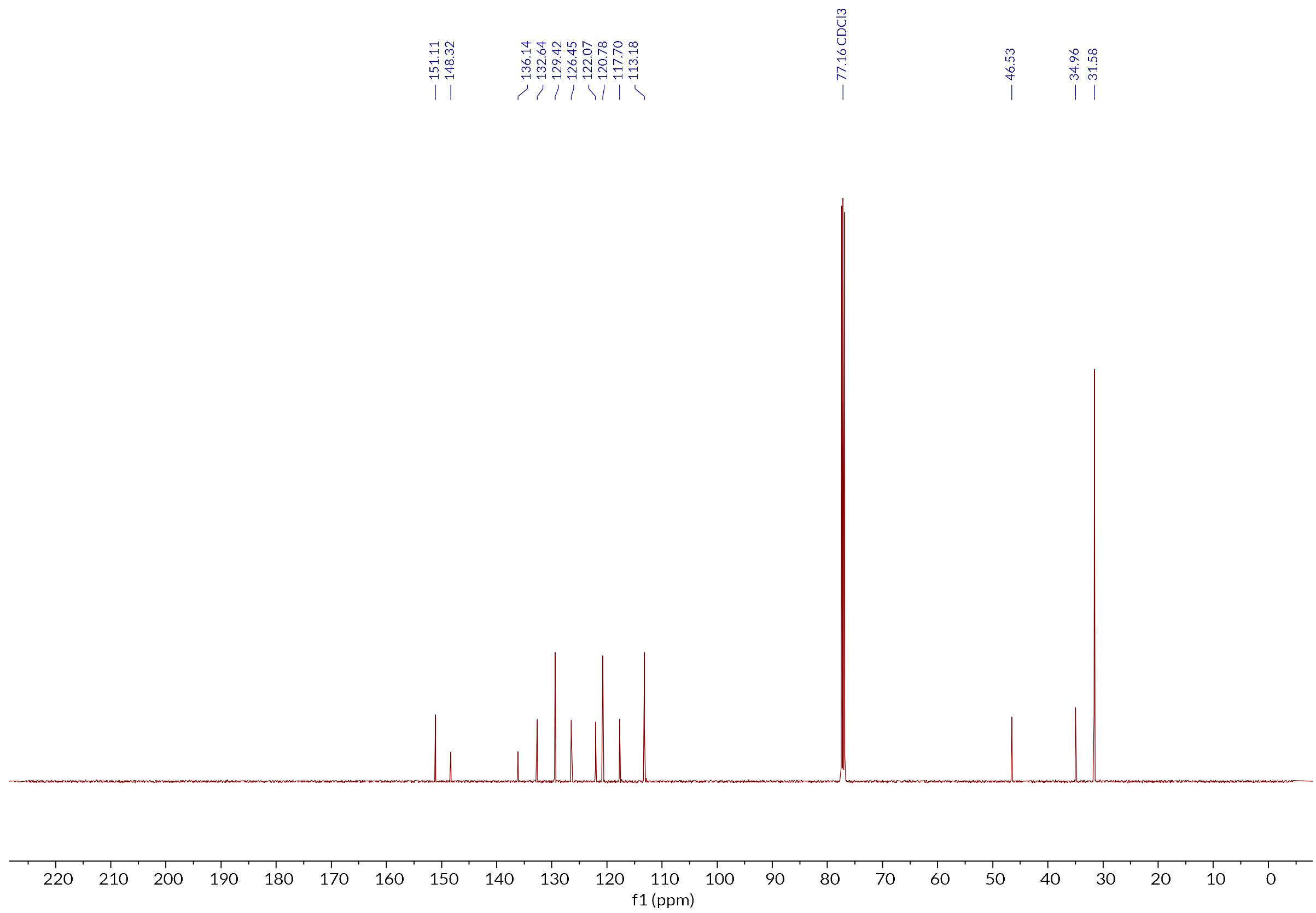

(*E*)-N-Benzyl-*N*-(3-(3,5-di-*tert*-butylphenyl)allyl)aniline (1at)

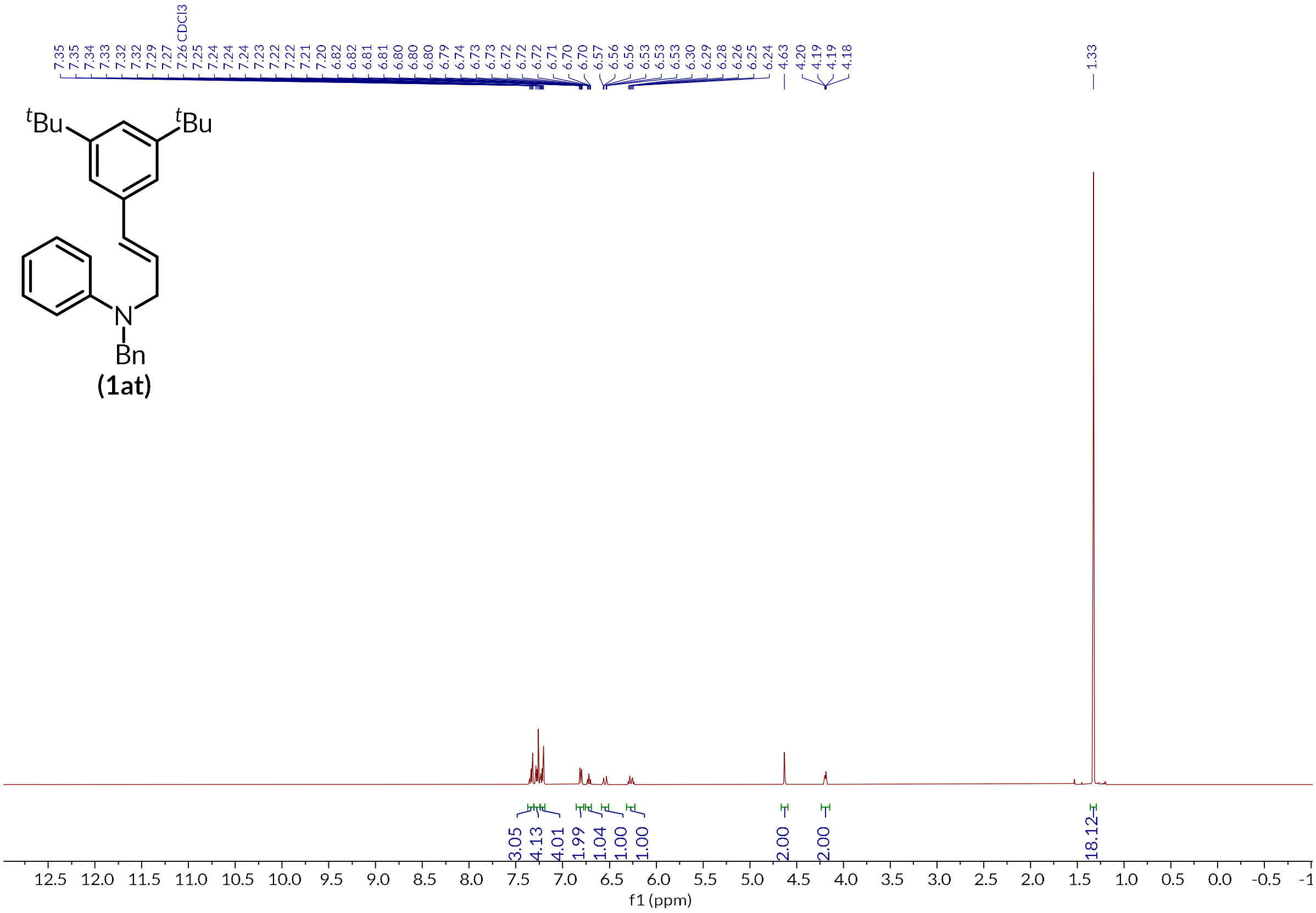

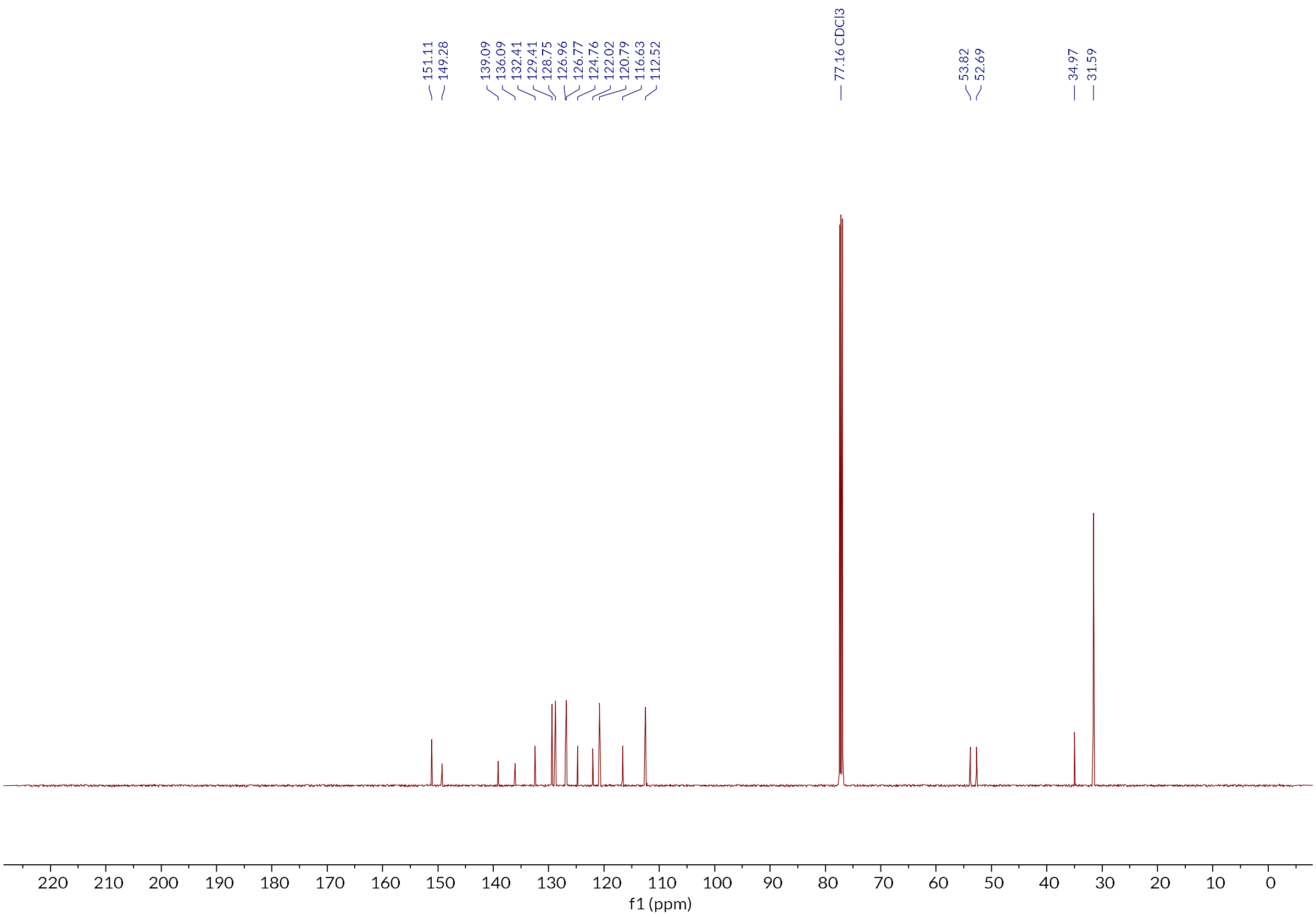

(*E*)-3-(2-Bromo-5-chlorophenyl)acrylaldehyde (SI-11)

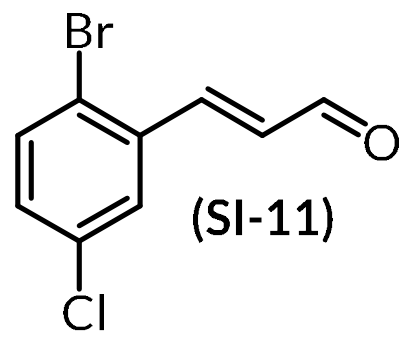

(SI-11)

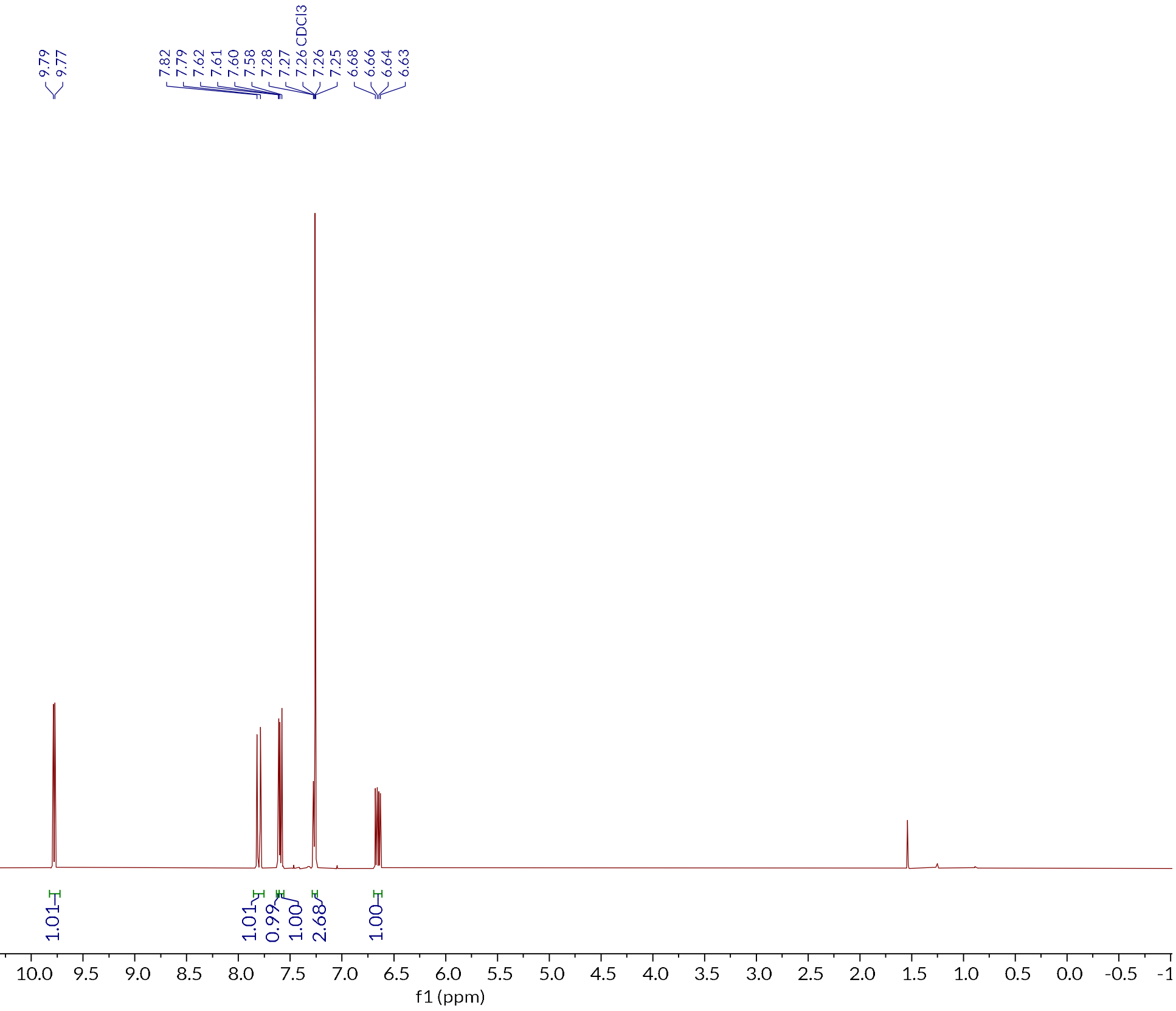

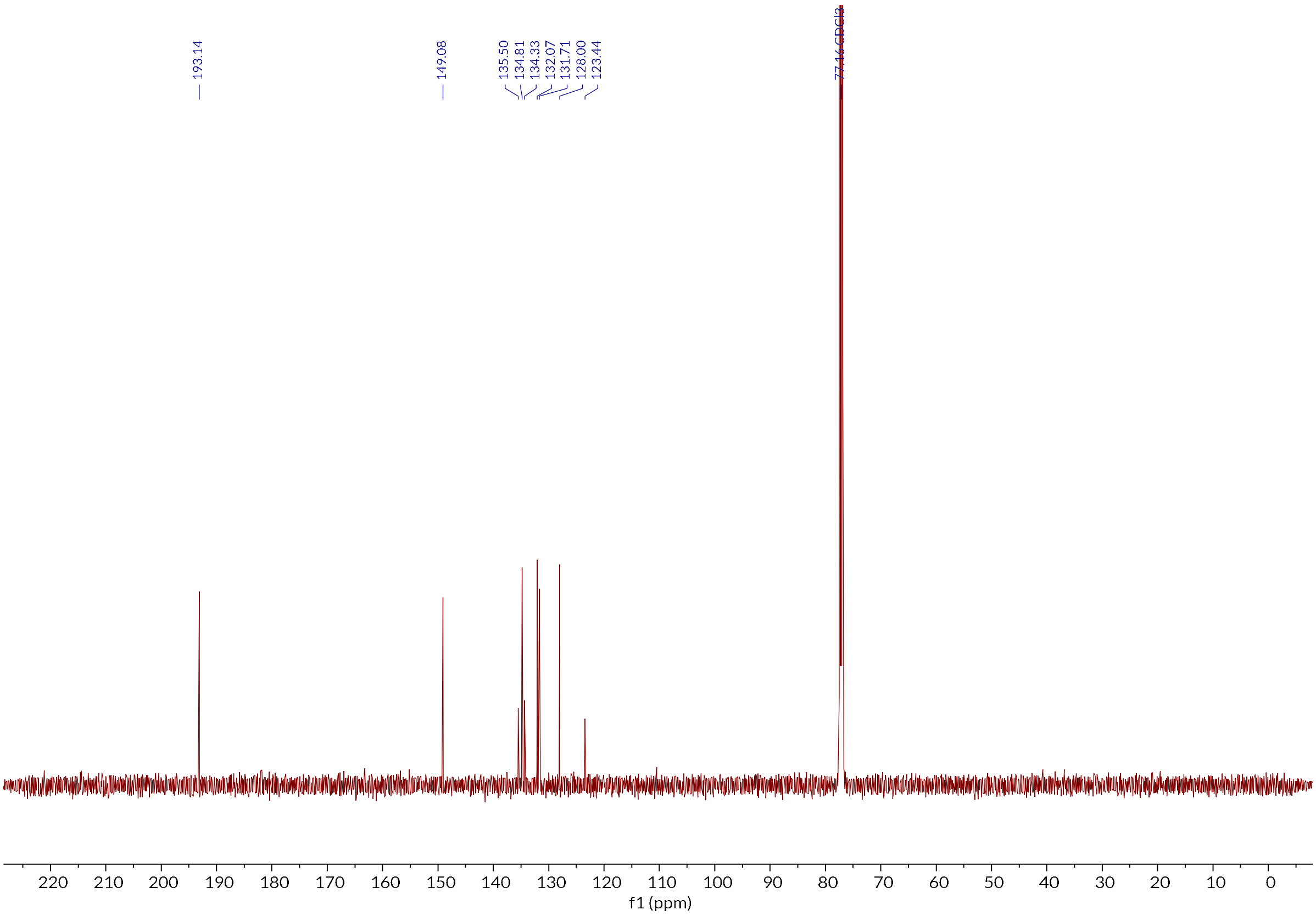

(*E*)-*N*-(3-(2-Bromo-5-chlorophenyl)allyl)aniline (SI-12)

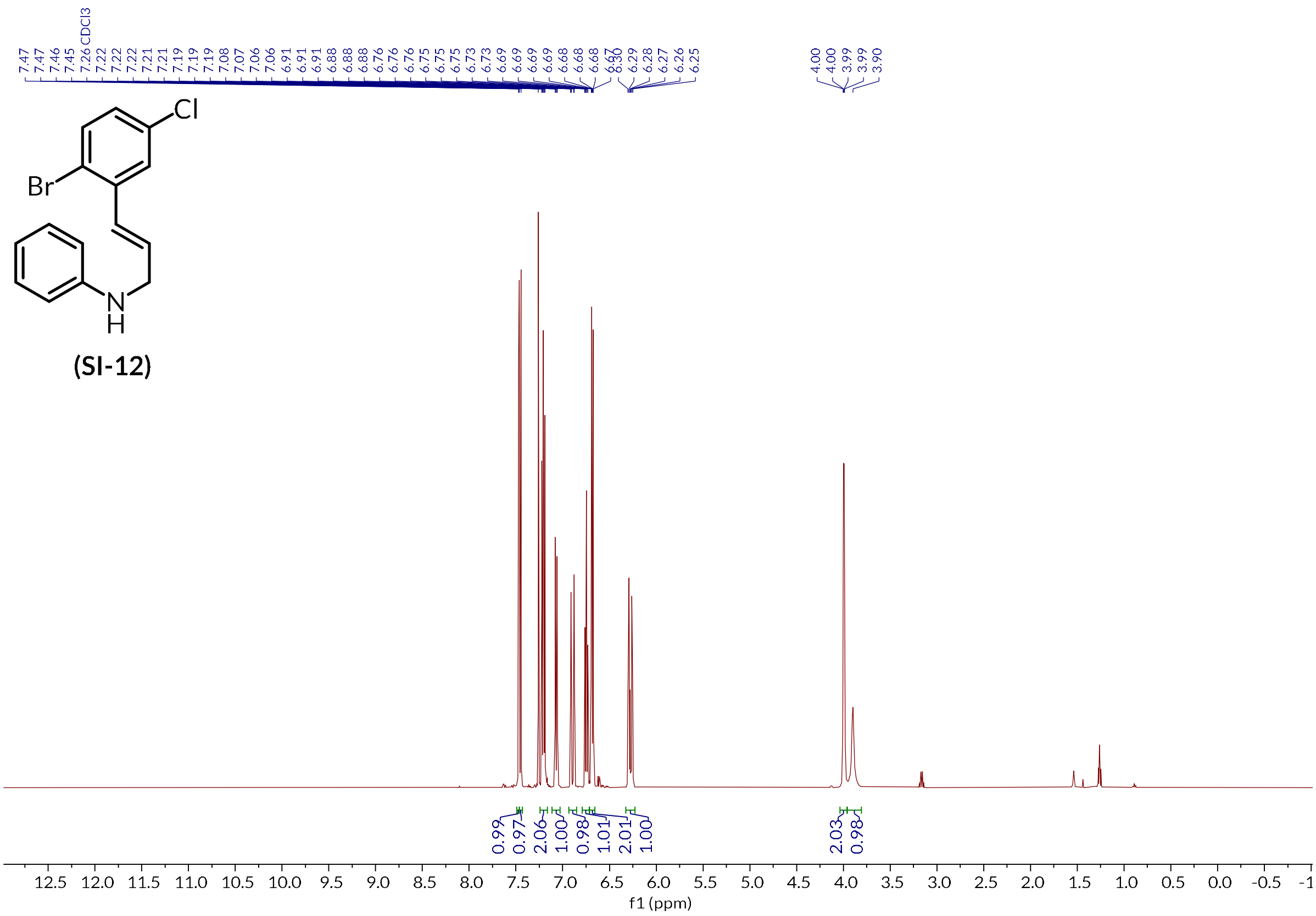

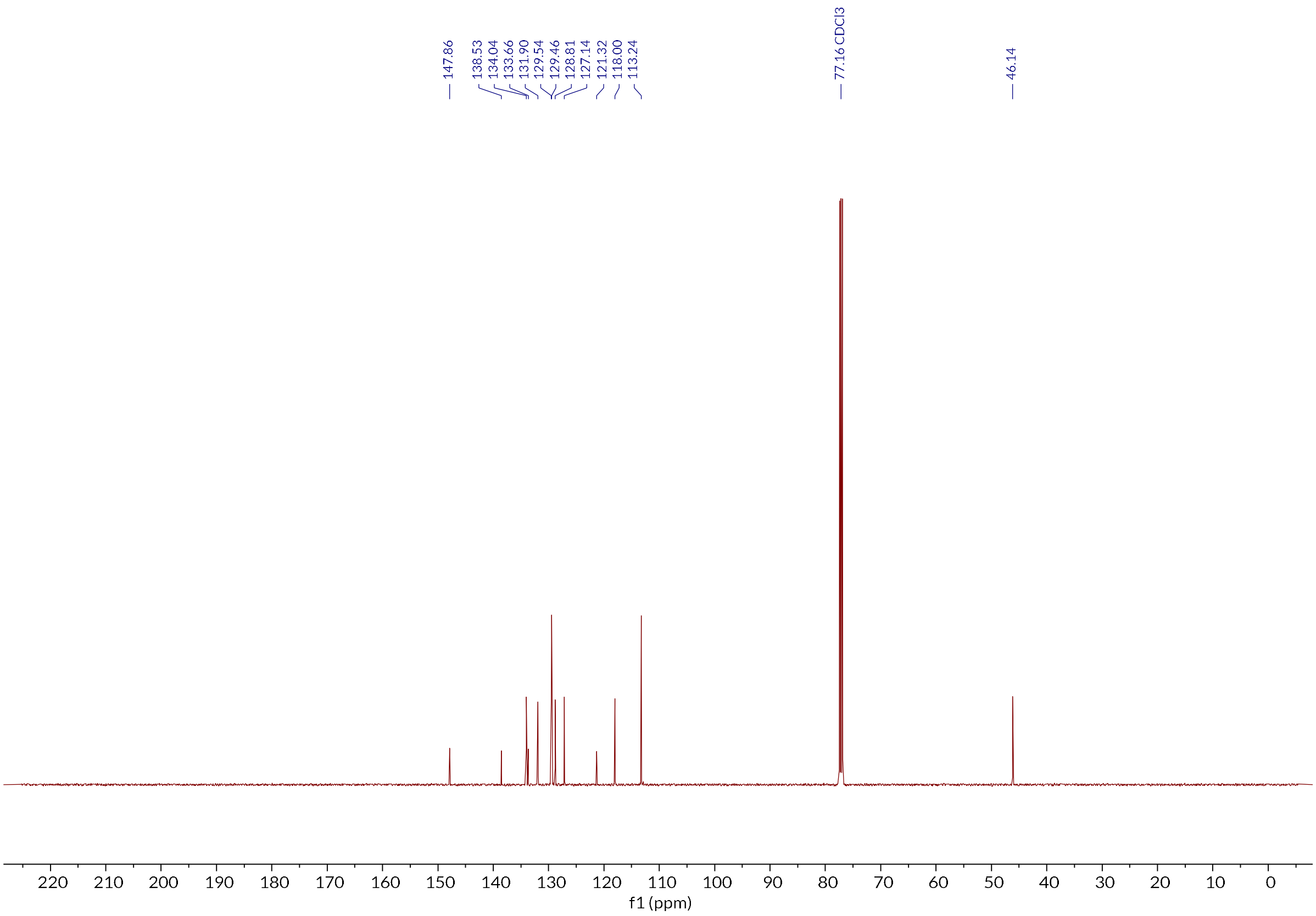

(*E*)-*N*-Benzyl-*N*-(3-(2-bromo-5-chlorophenyl)allyl)aniline (1au)

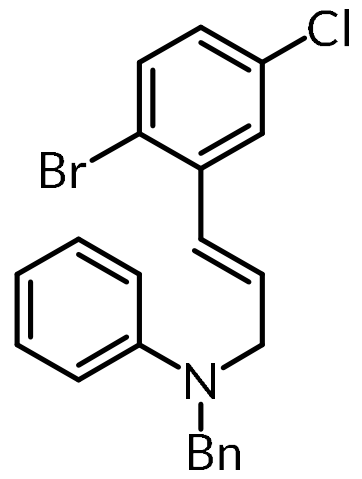

(1au)

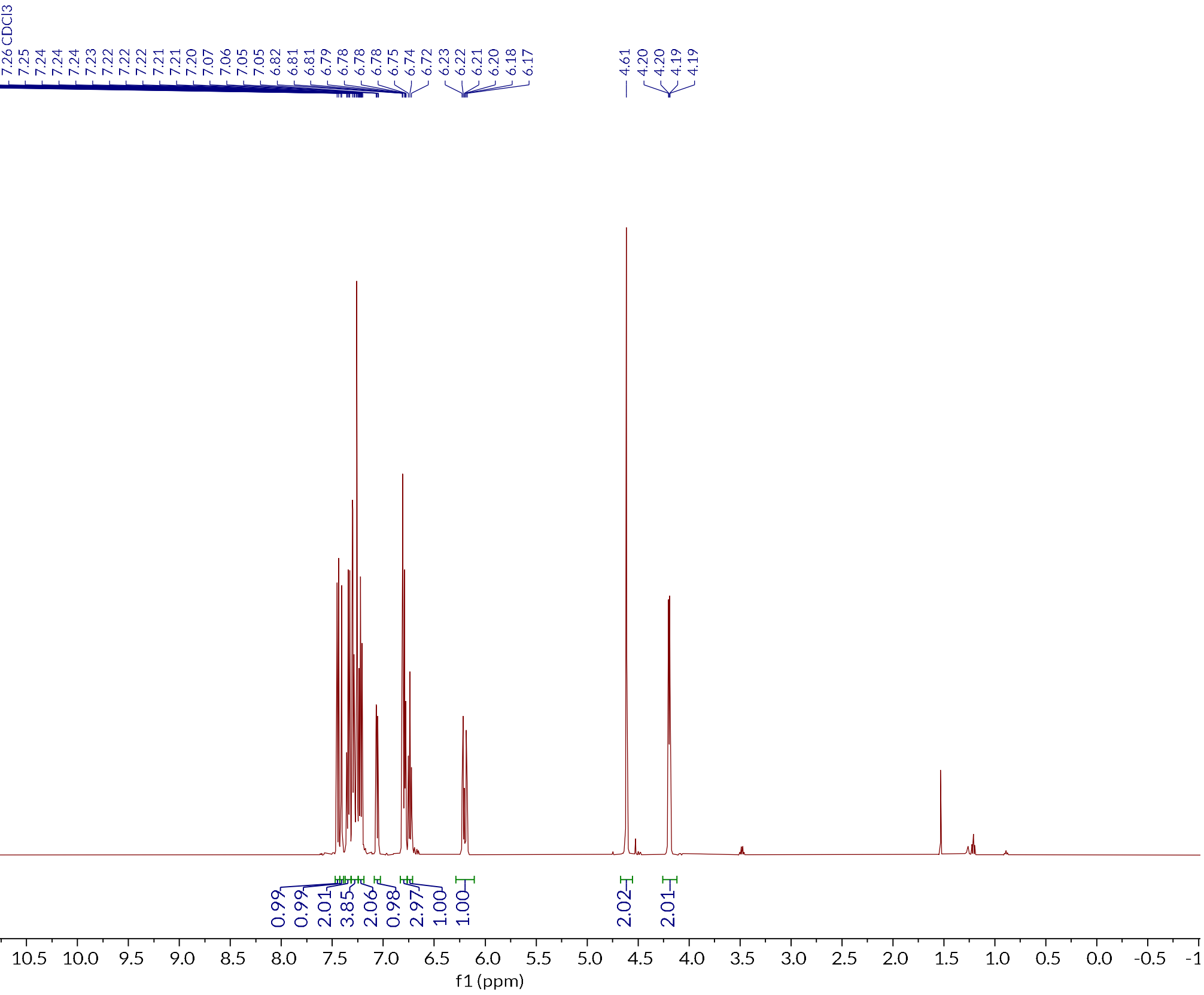

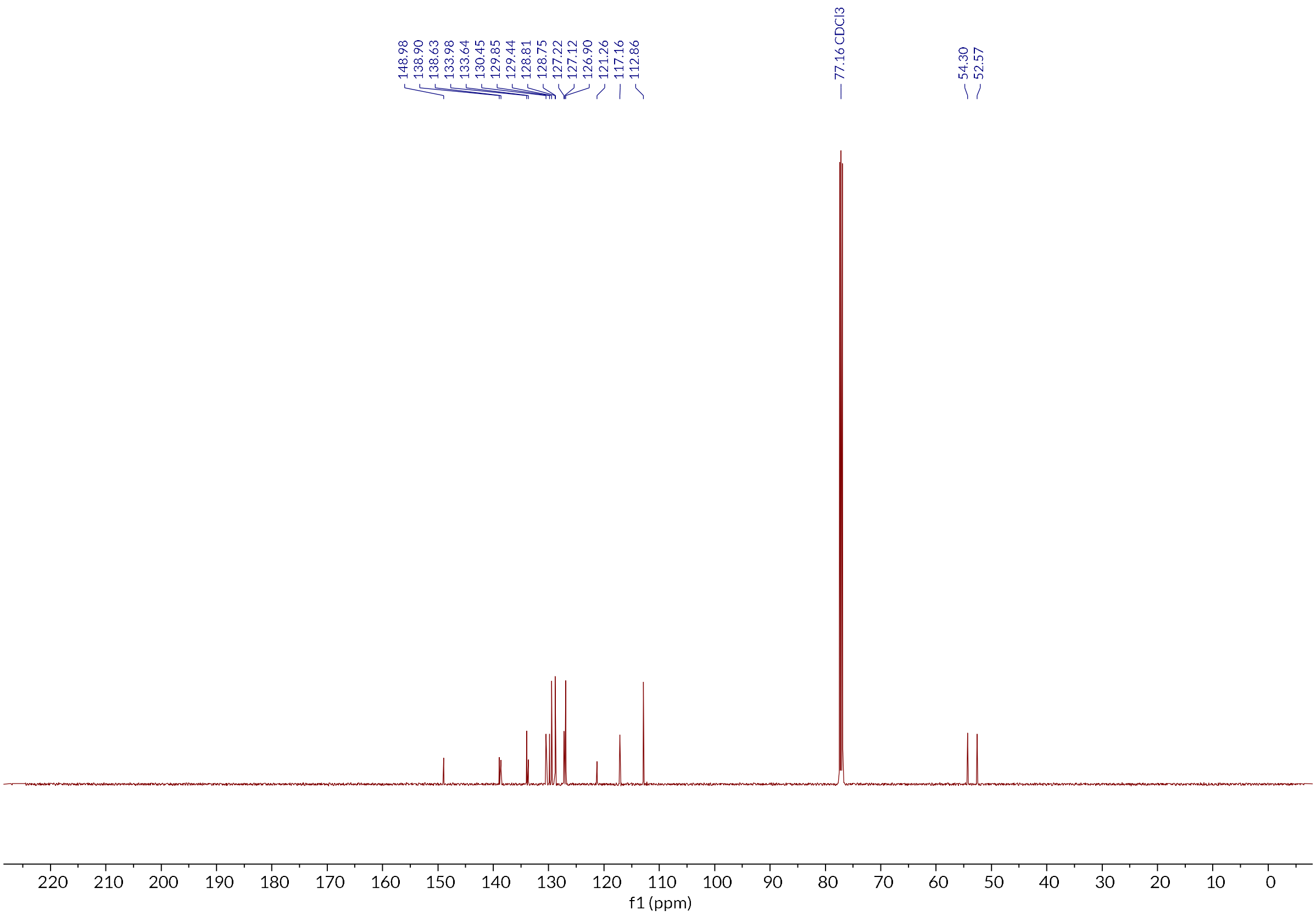

(*E*)-3-(2-(Trifluoromethoxy)phenyl)acrylaldehyde (SI-13)

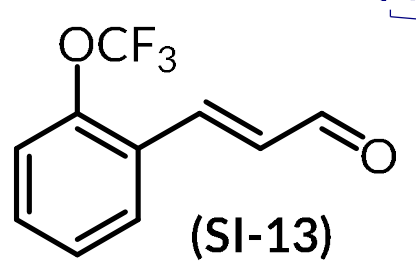

(SI-13)

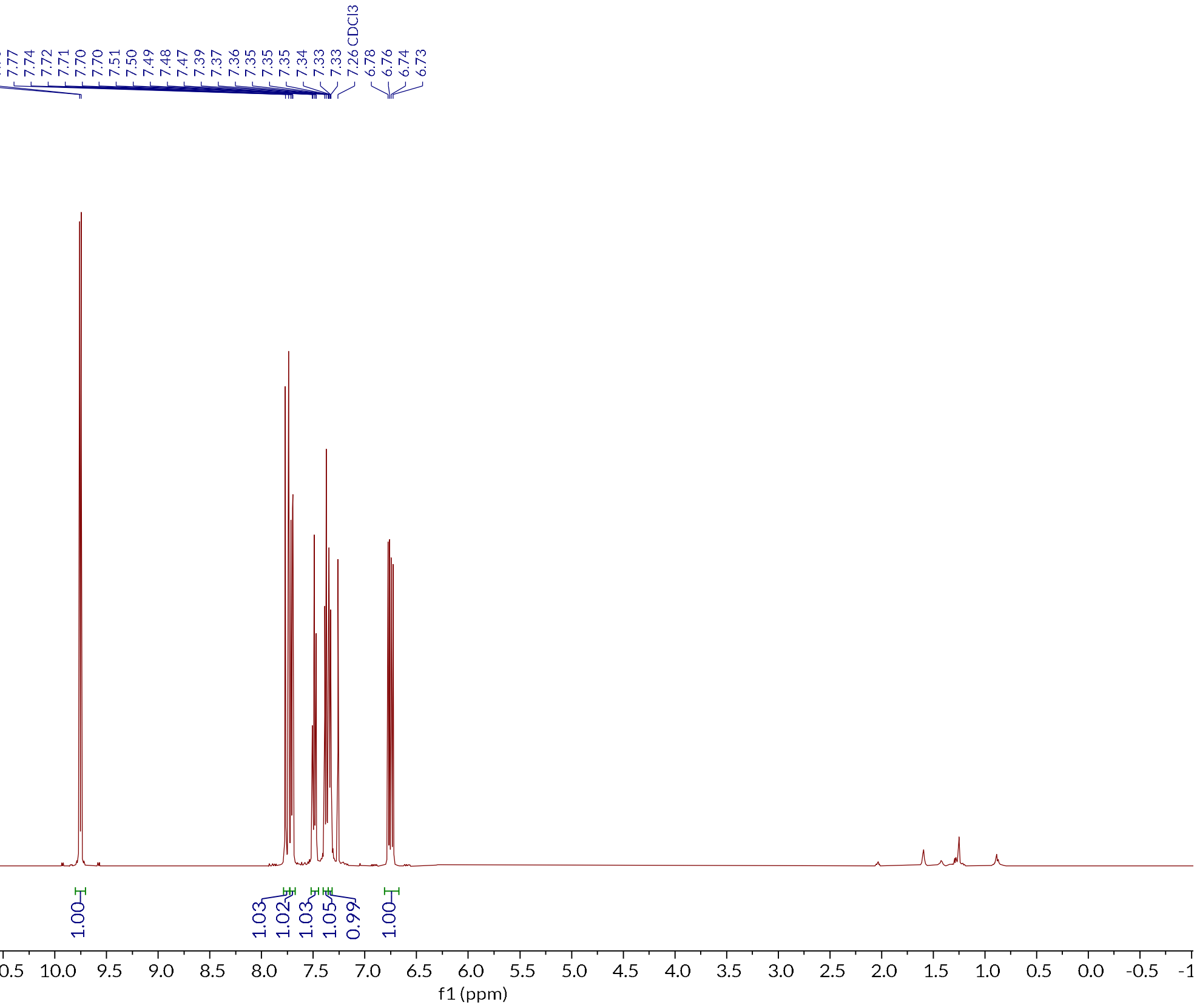

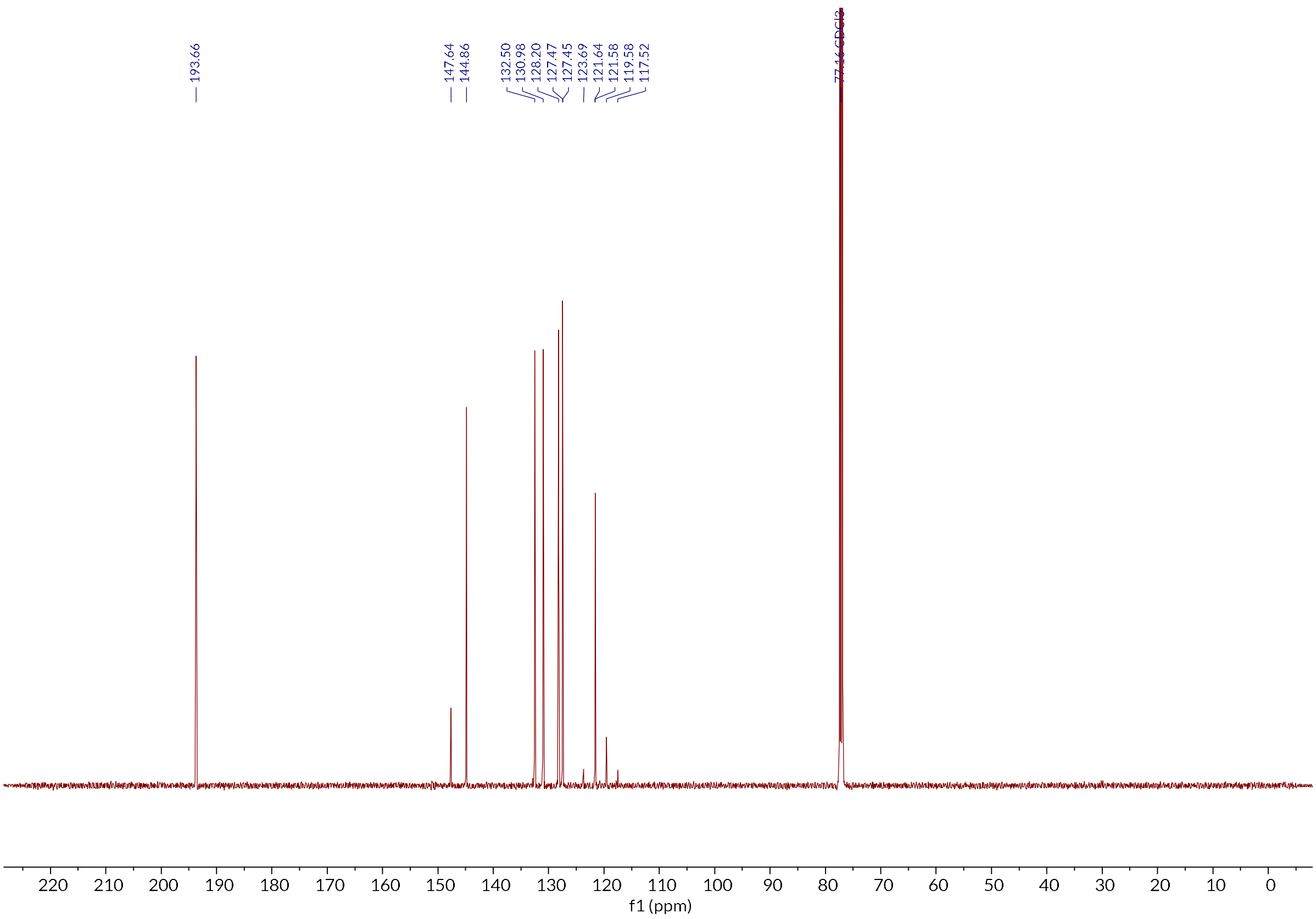

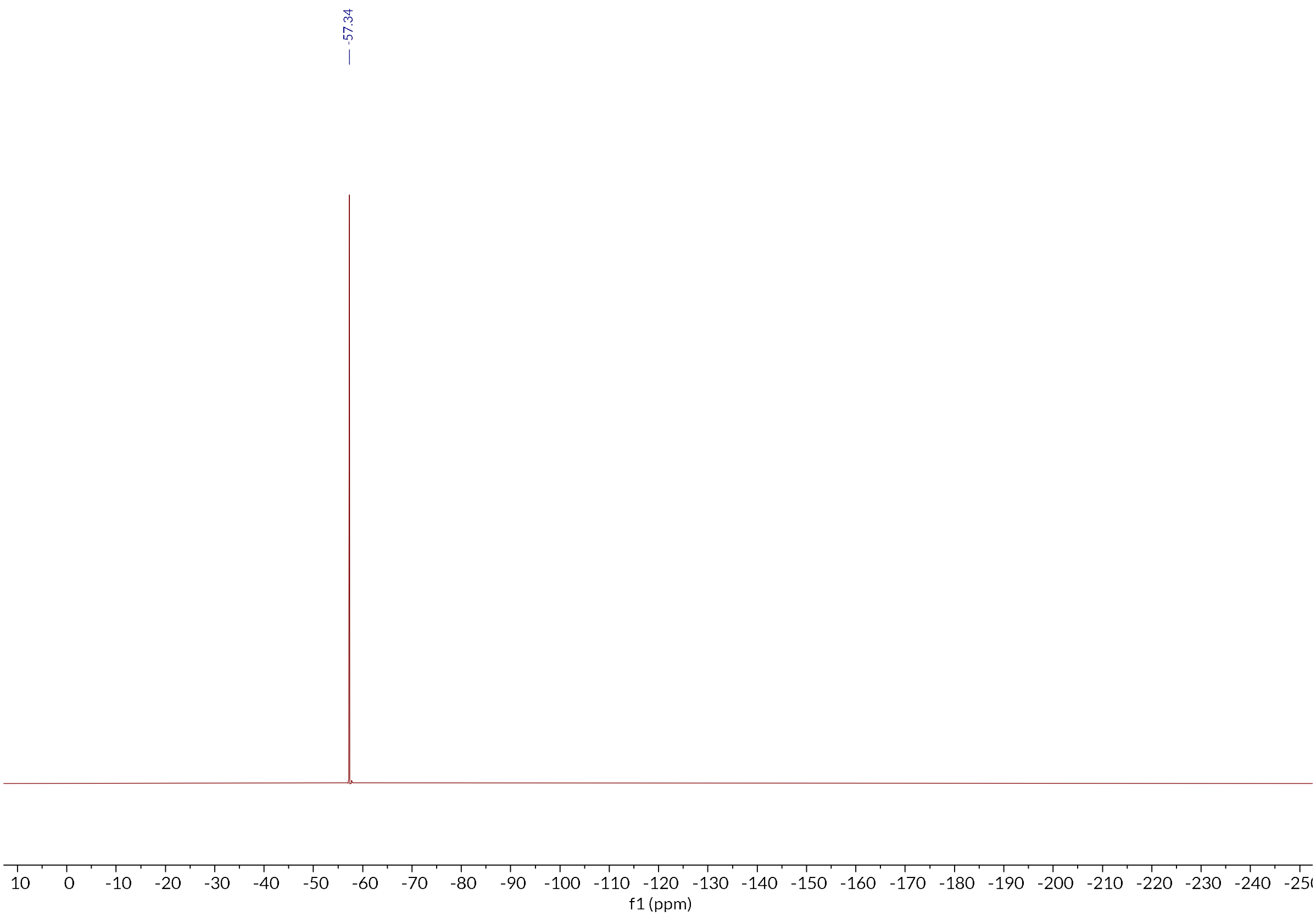
-57.34
10 0 -10 -20 -30 -40 -50 -60 -70 -80 -90 -100 -110 -120 -130 -140 -150 -160 -170 -180 -190 -200 -210 -220 -230 -240
f1 (ppm)

(*E*)-*N*-(3-(2-(trifluoromethoxy)phenyl)allyl)aniline (SI-14)

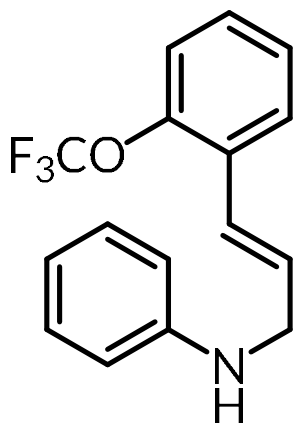

(SI-14)

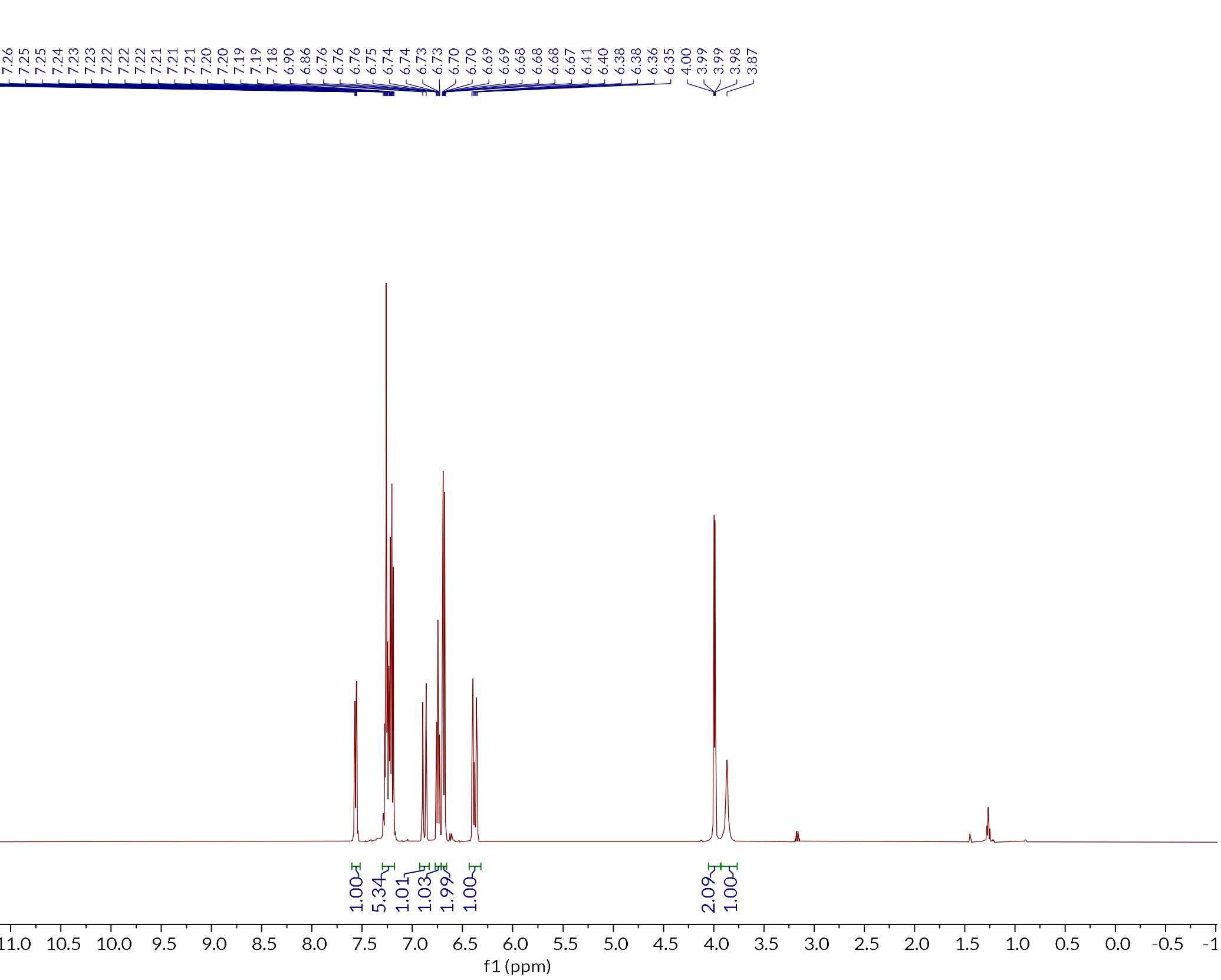

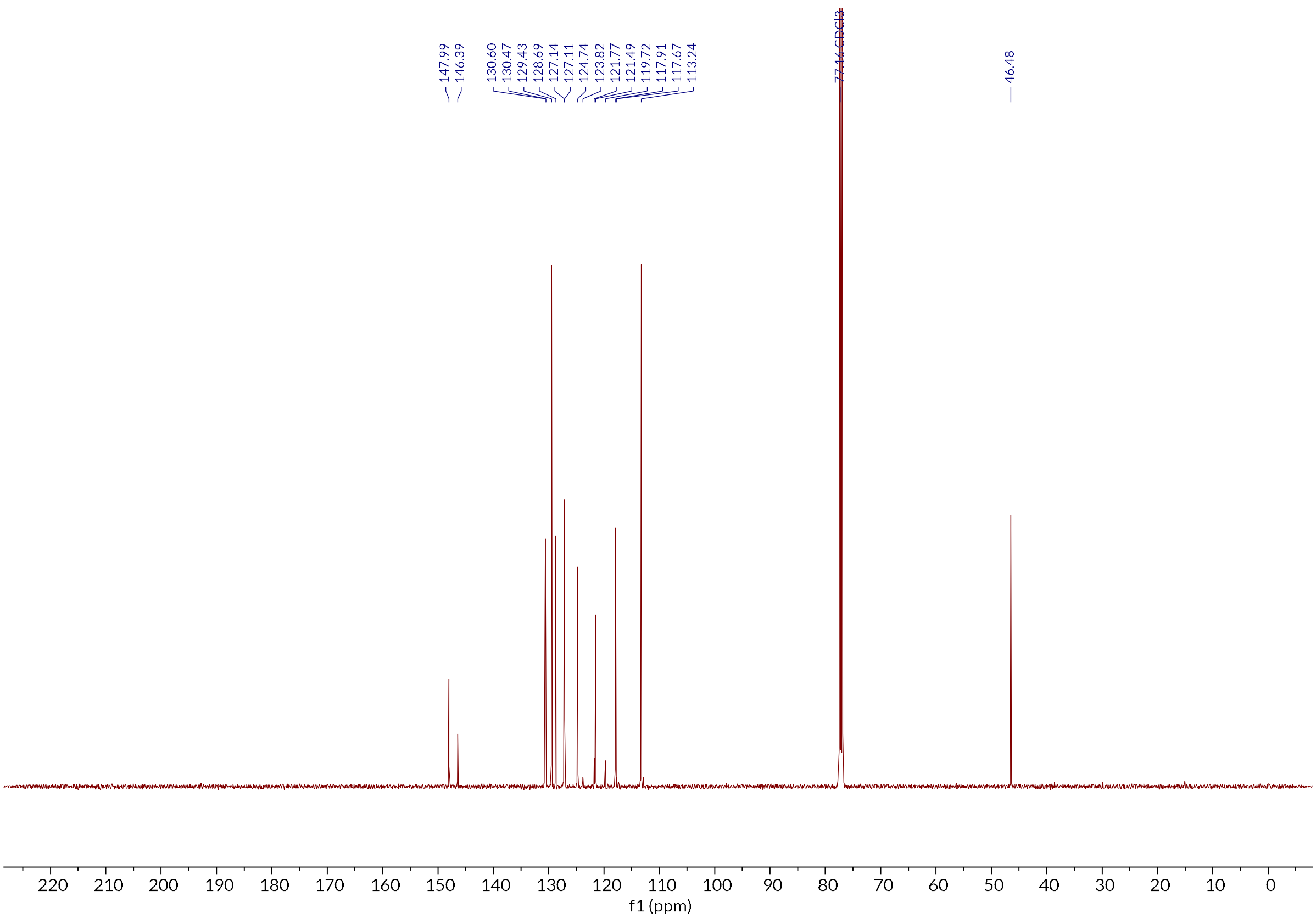

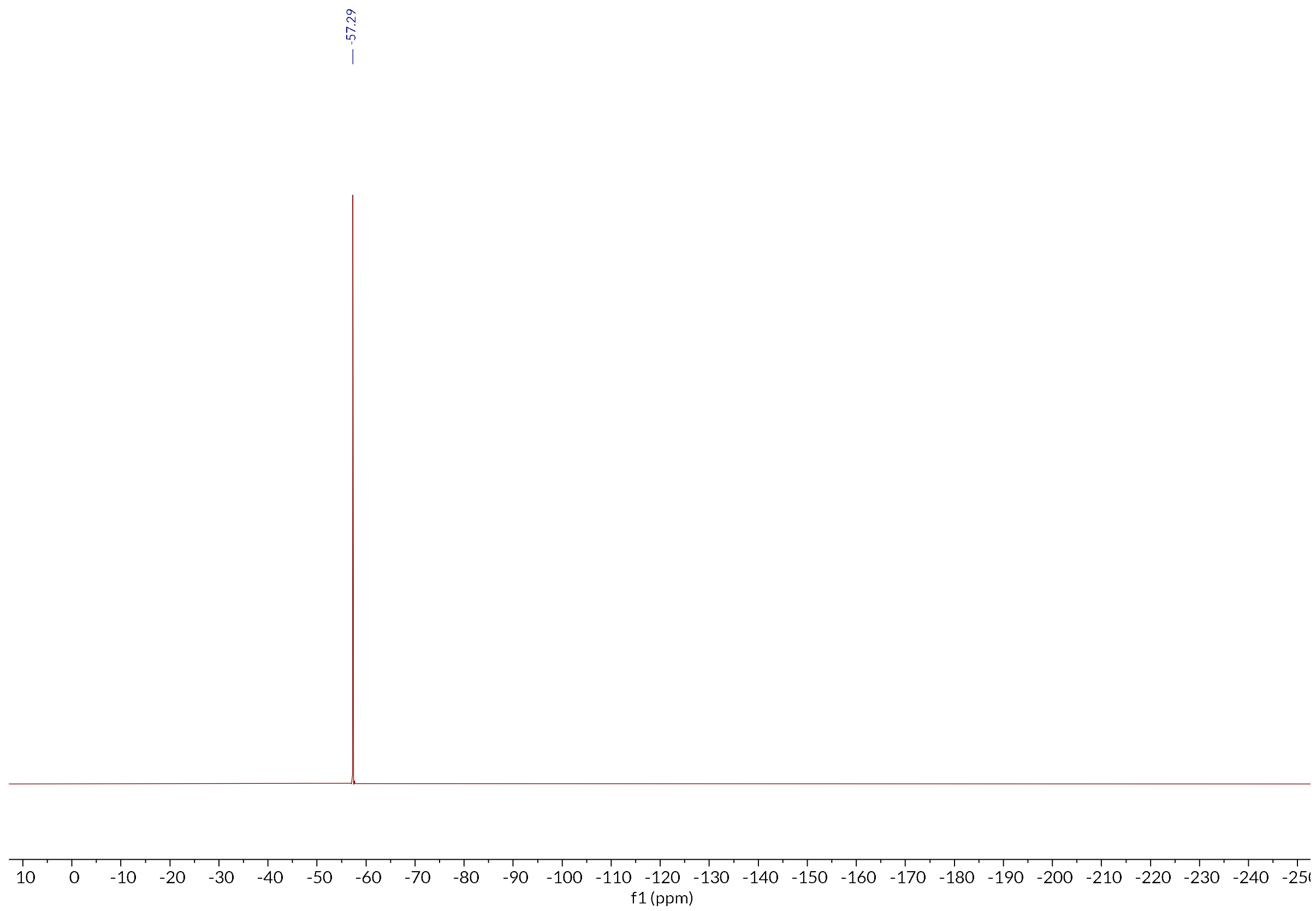
-57.29
10
0
-10
-20
-30
-40
-50
-60
-70
-80
-90
-100
-110
-120
-130
-140
-150
-160
-170
-180
-190
-200
-210
-220
-230
-240
f1 (ppm)

(*E*)-*N*-Benzyl-*N*-(3-(2-(trifluoromethoxy)phenyl)allyl)aniline (1av)

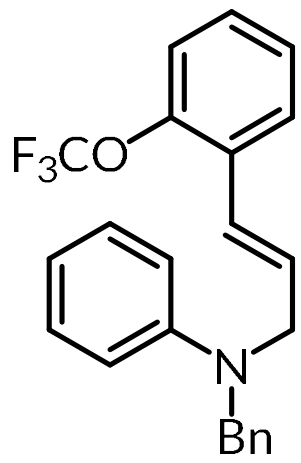

(1av)

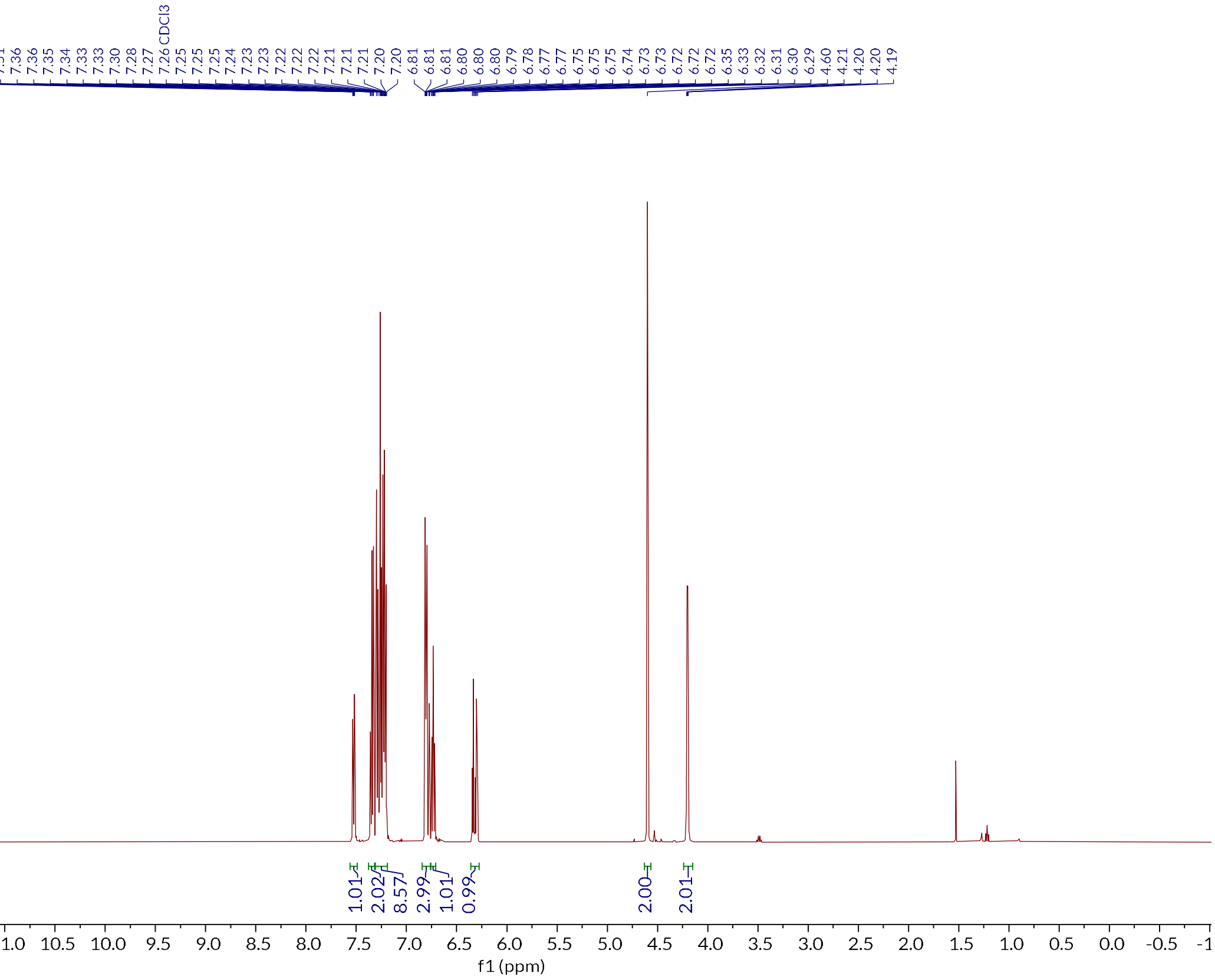

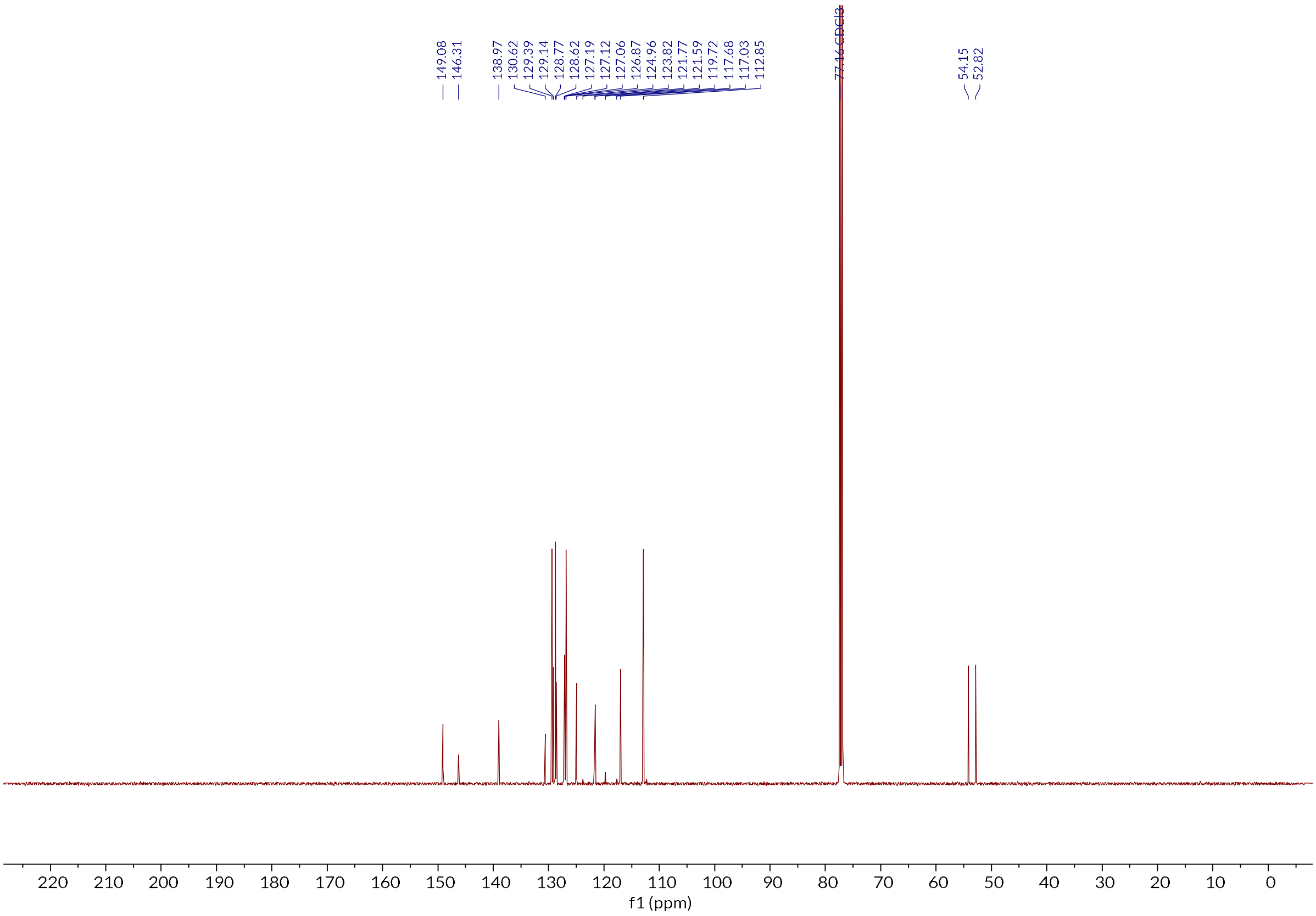

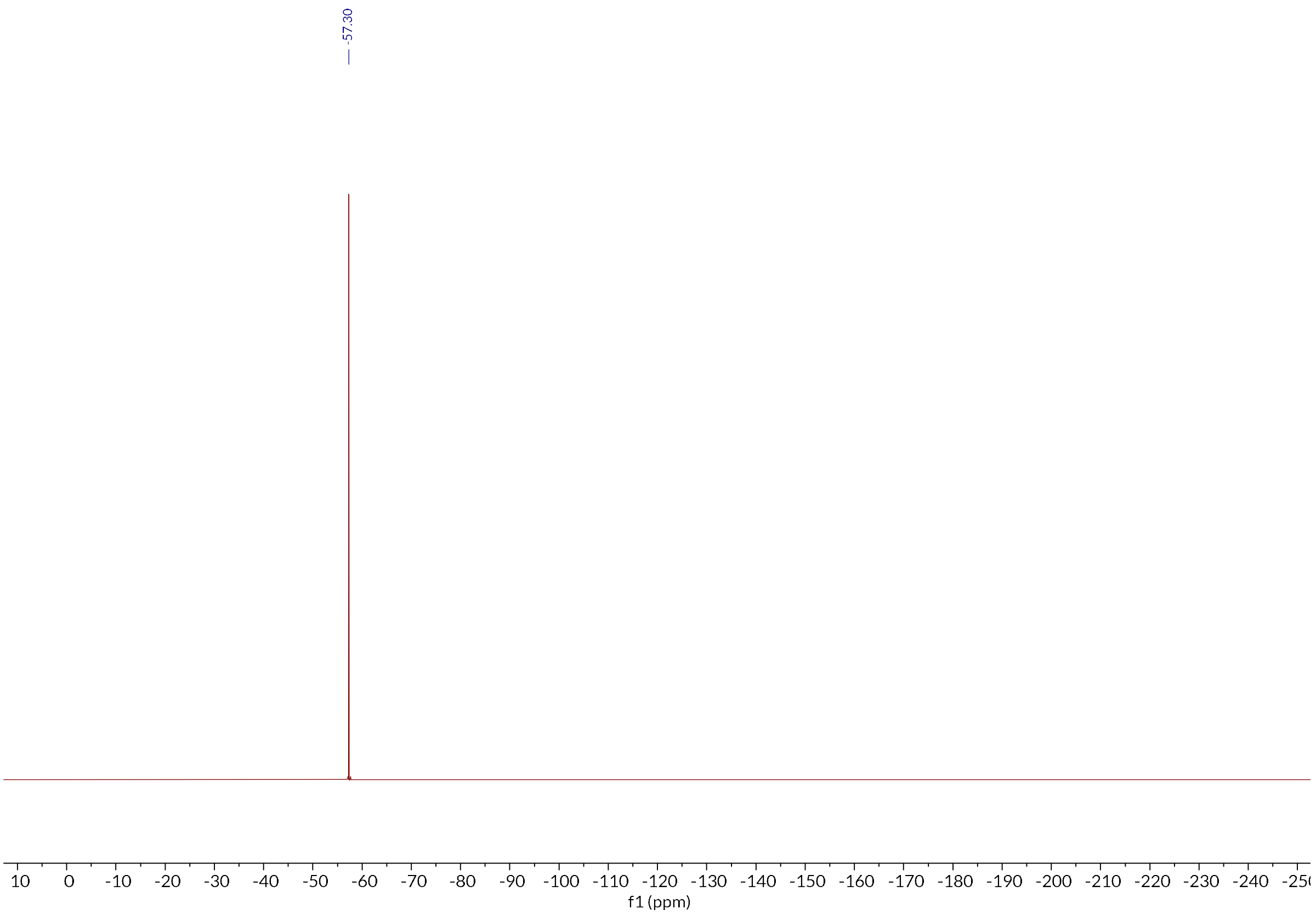
-57.30
10
0
-10
-20
-30
-40
-50
-60
-70
-80
-90
-100
-110
-120
-130
-140
-150
-160
-170
-180
-190
-200
-210
-220
-230
-240
f1 (ppm)

(*E*)-*N*-(3-(Furan-2-yl)allyl)aniline (3h)

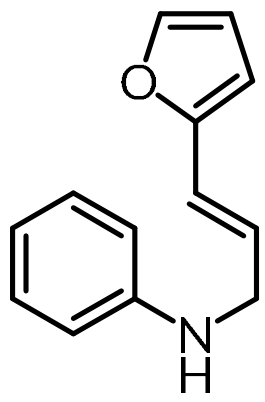

(3h)

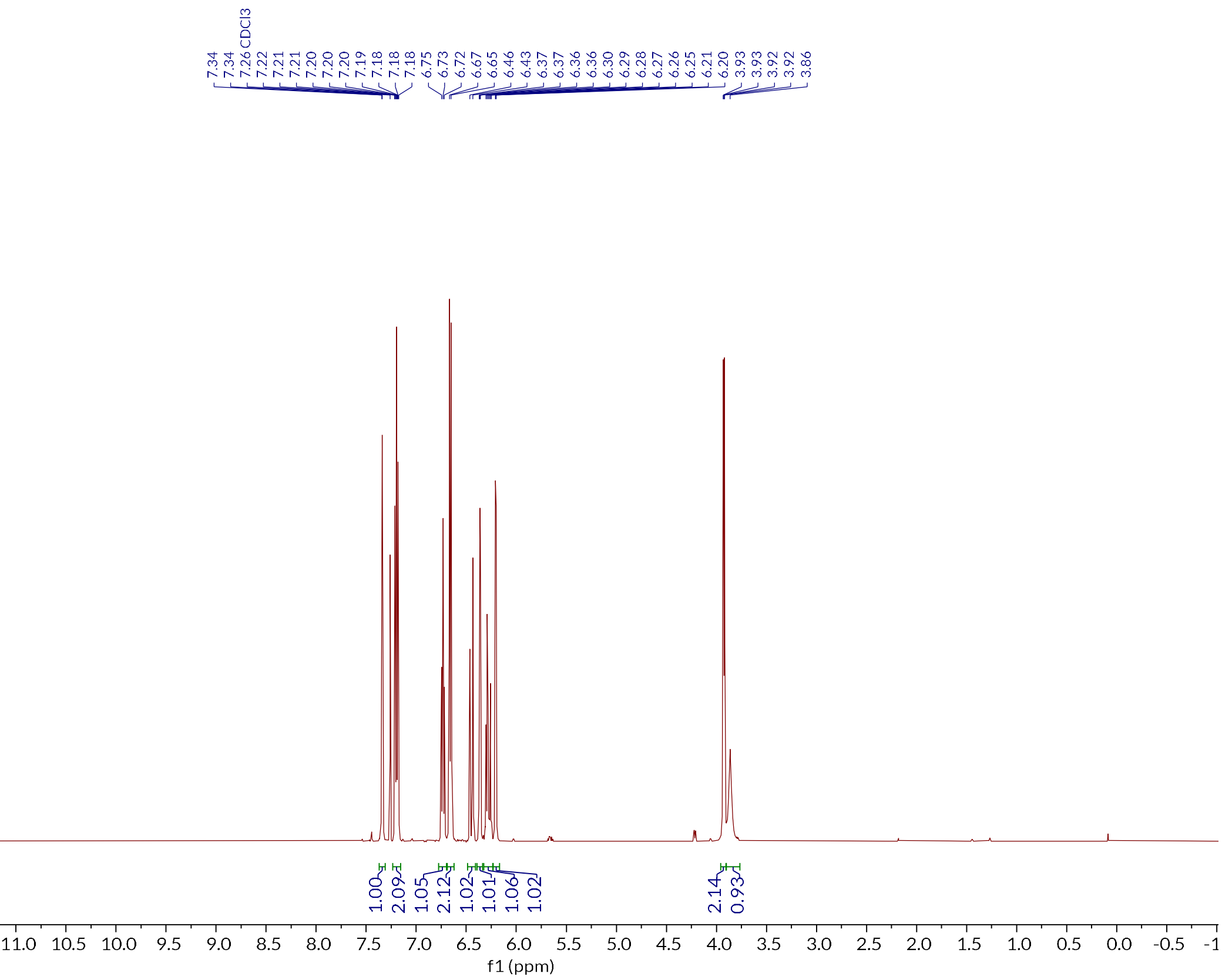

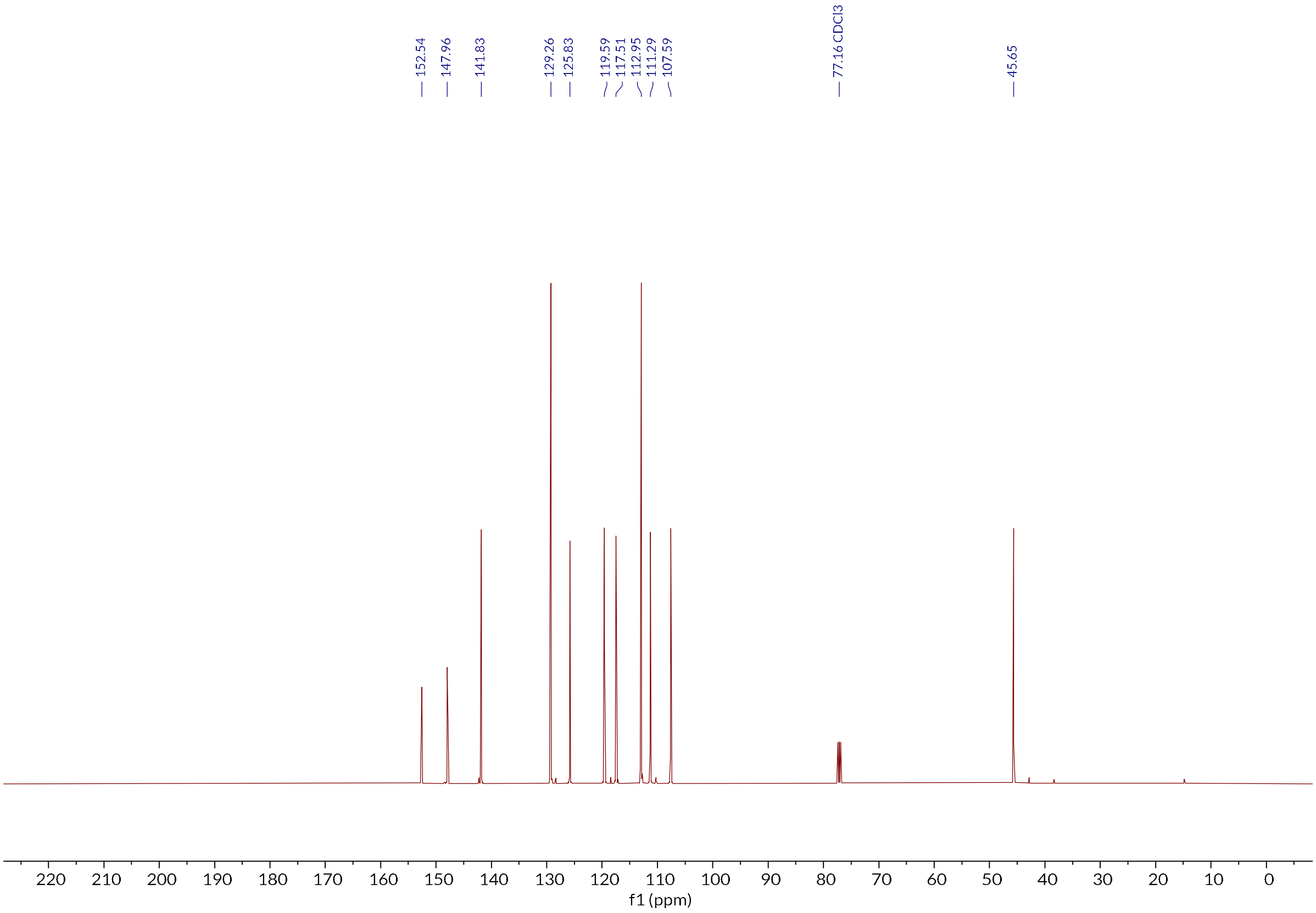

(*E*)-*N*-Benzyl-*N*-(3-(furan-2-yl)allyl)aniline (1aw)

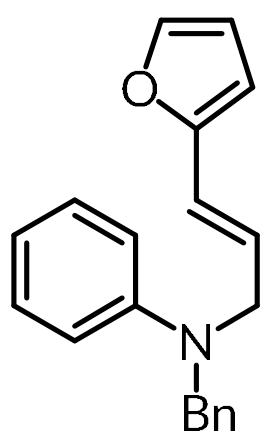

(1aw)

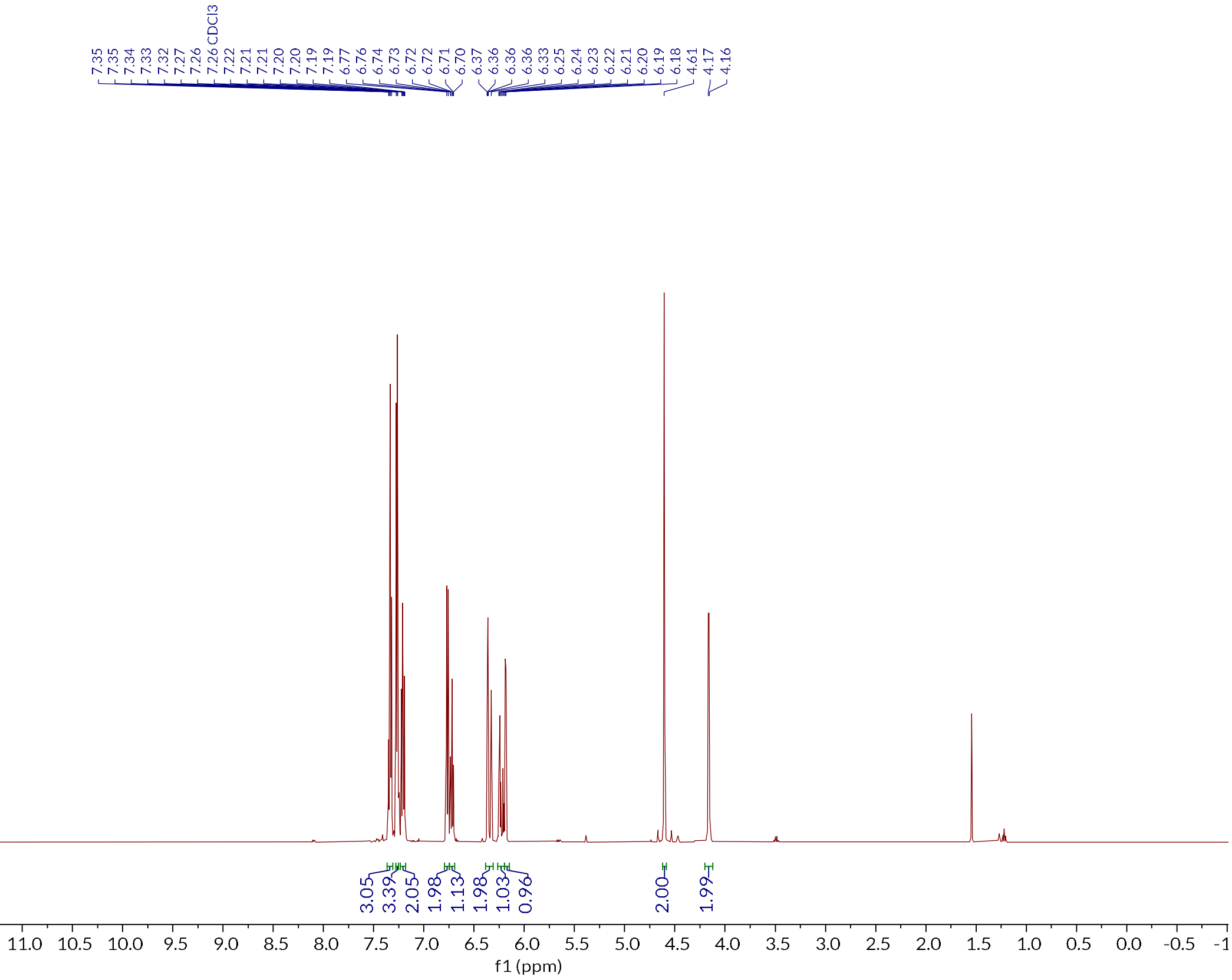

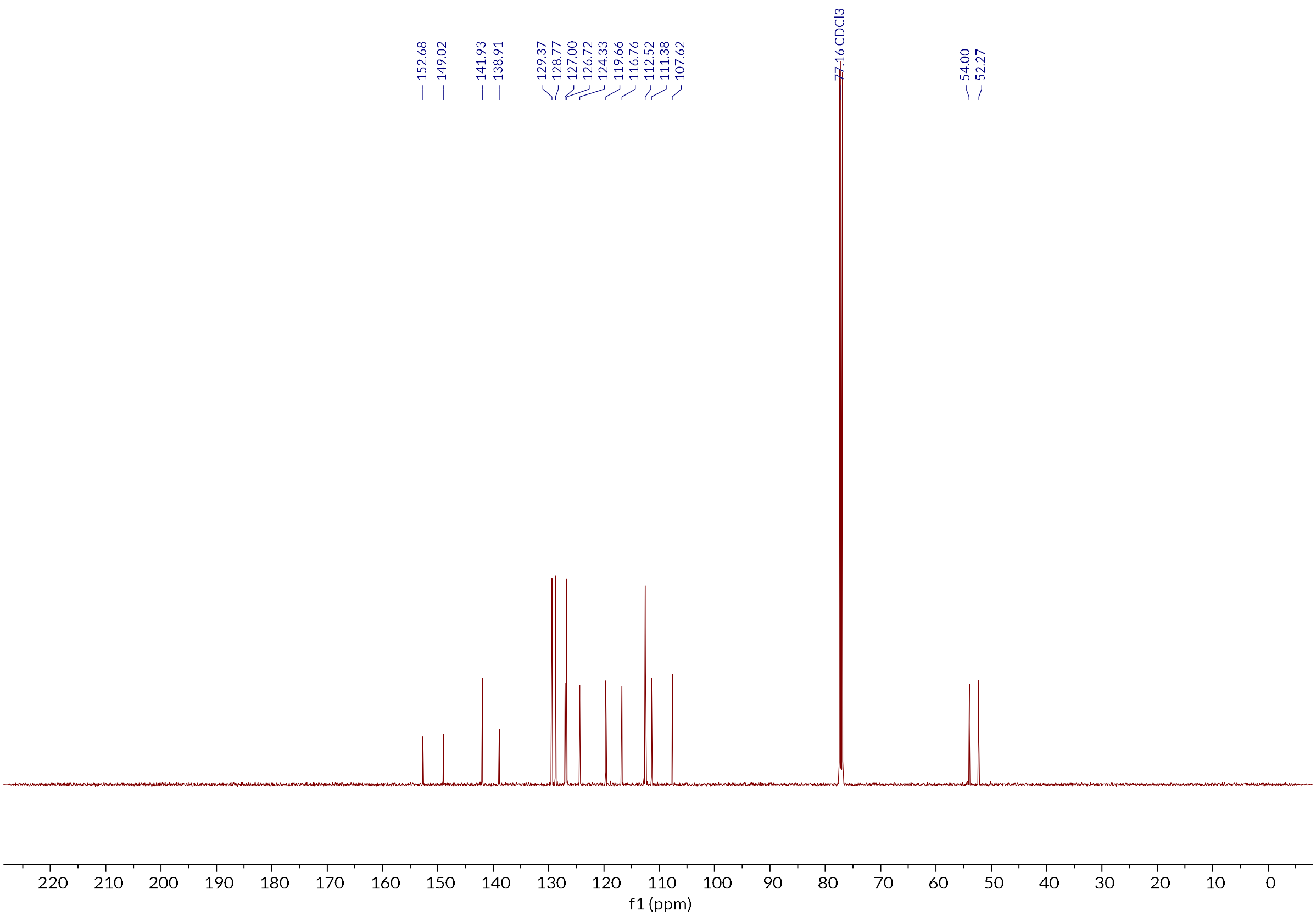

(*E*)-3-(Benzo[*b*]thiophen-3-yl)acrylaldehyde (SI-15)

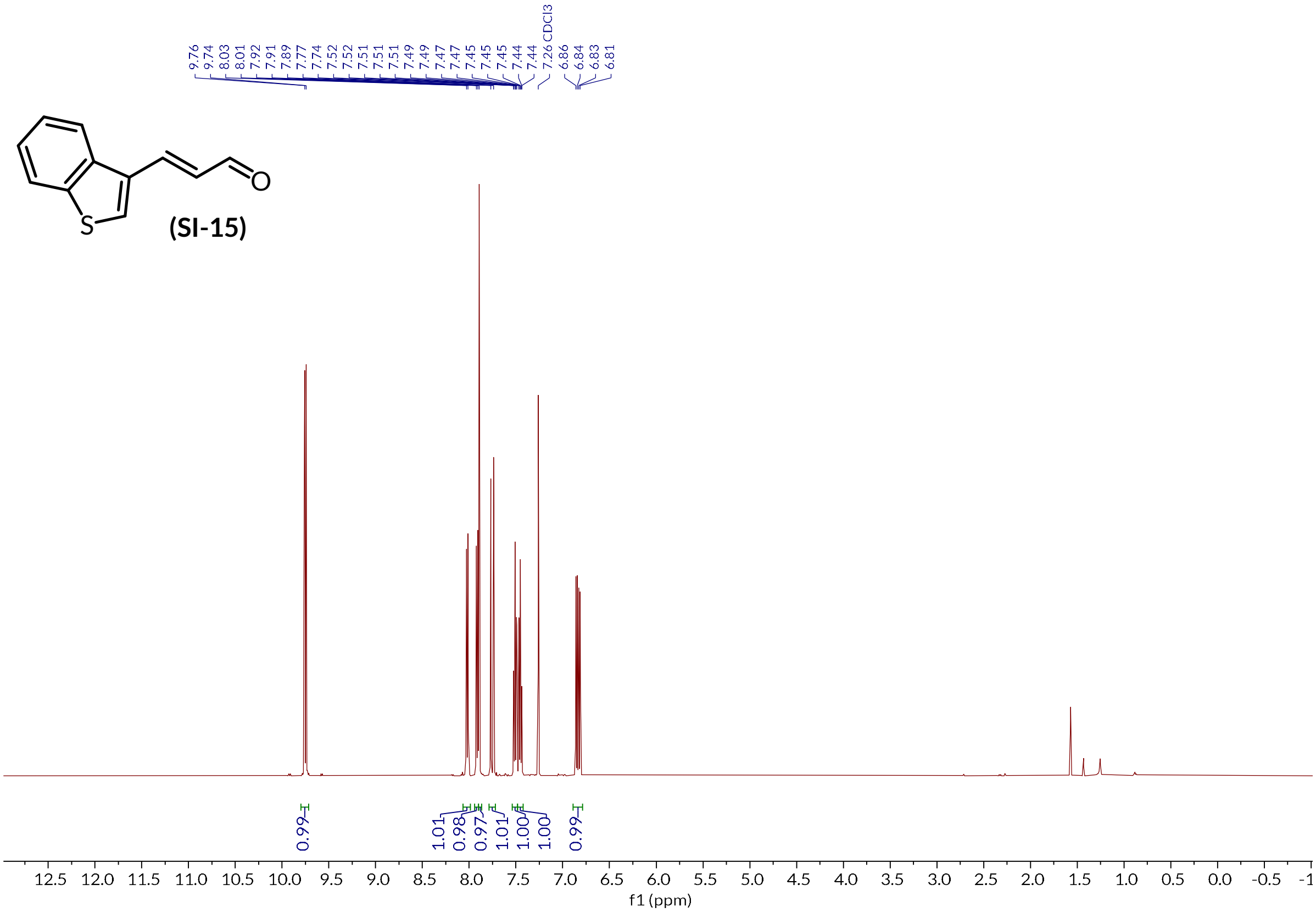

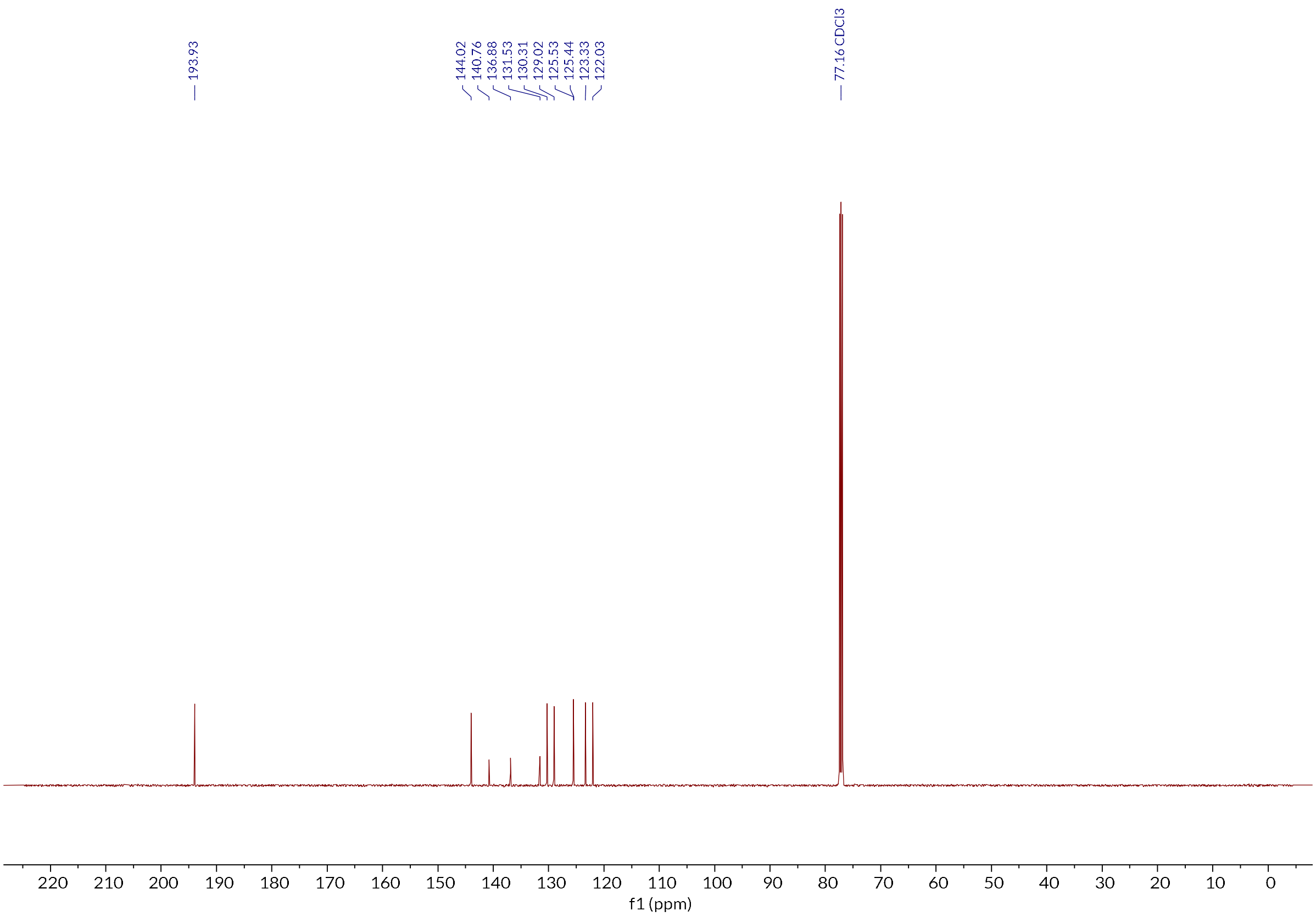

(*E*)-*N*-(3-(Benzo[*b*]thiophen-3-yl)allyl)aniline (SI-16)

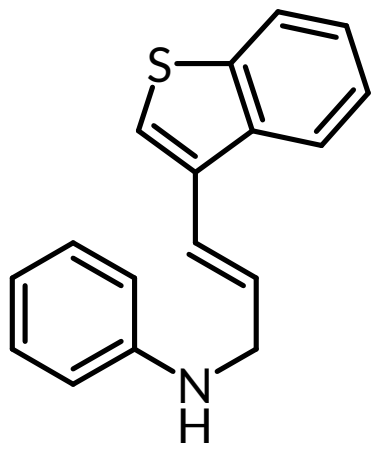

(SI-16)

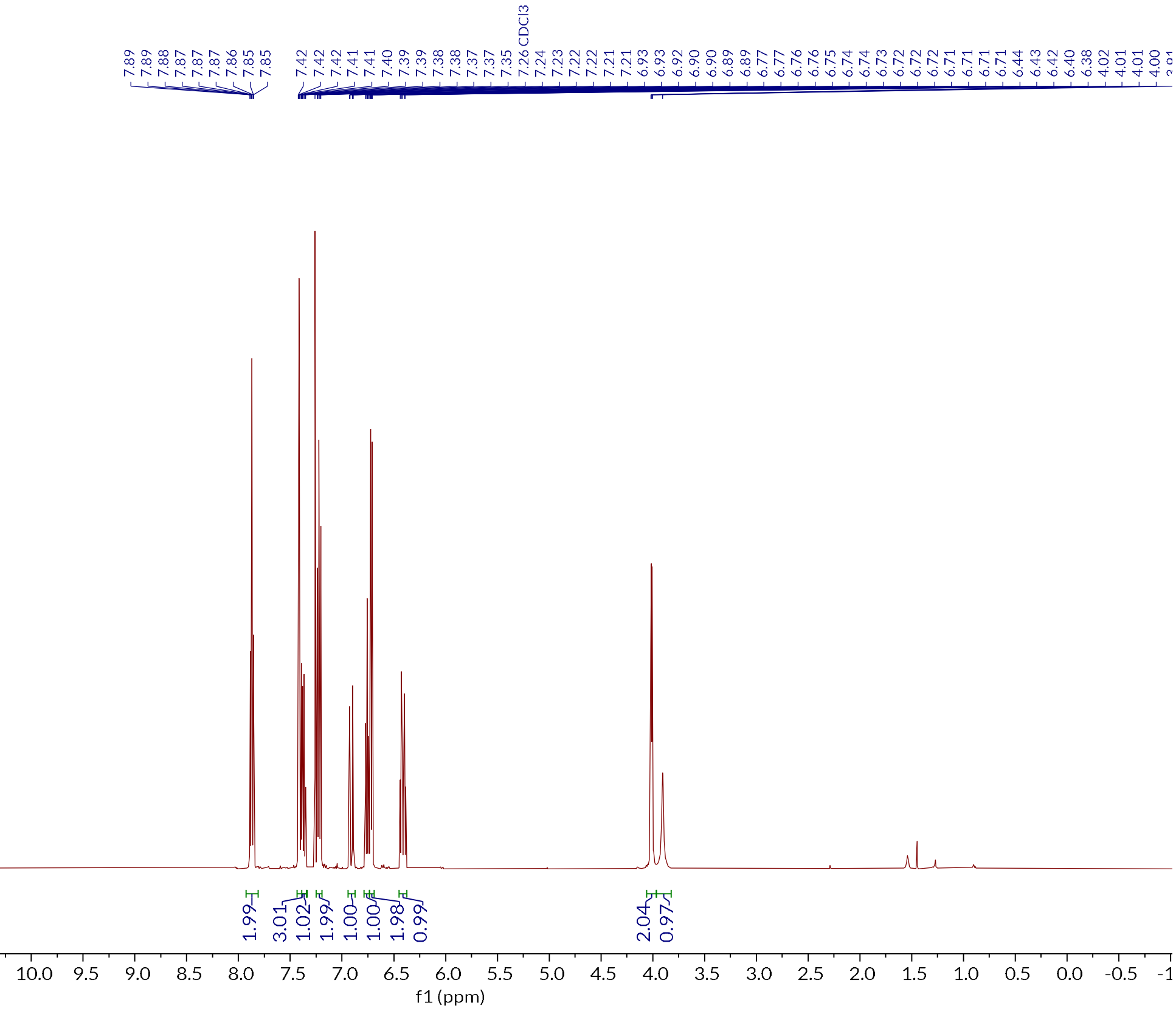

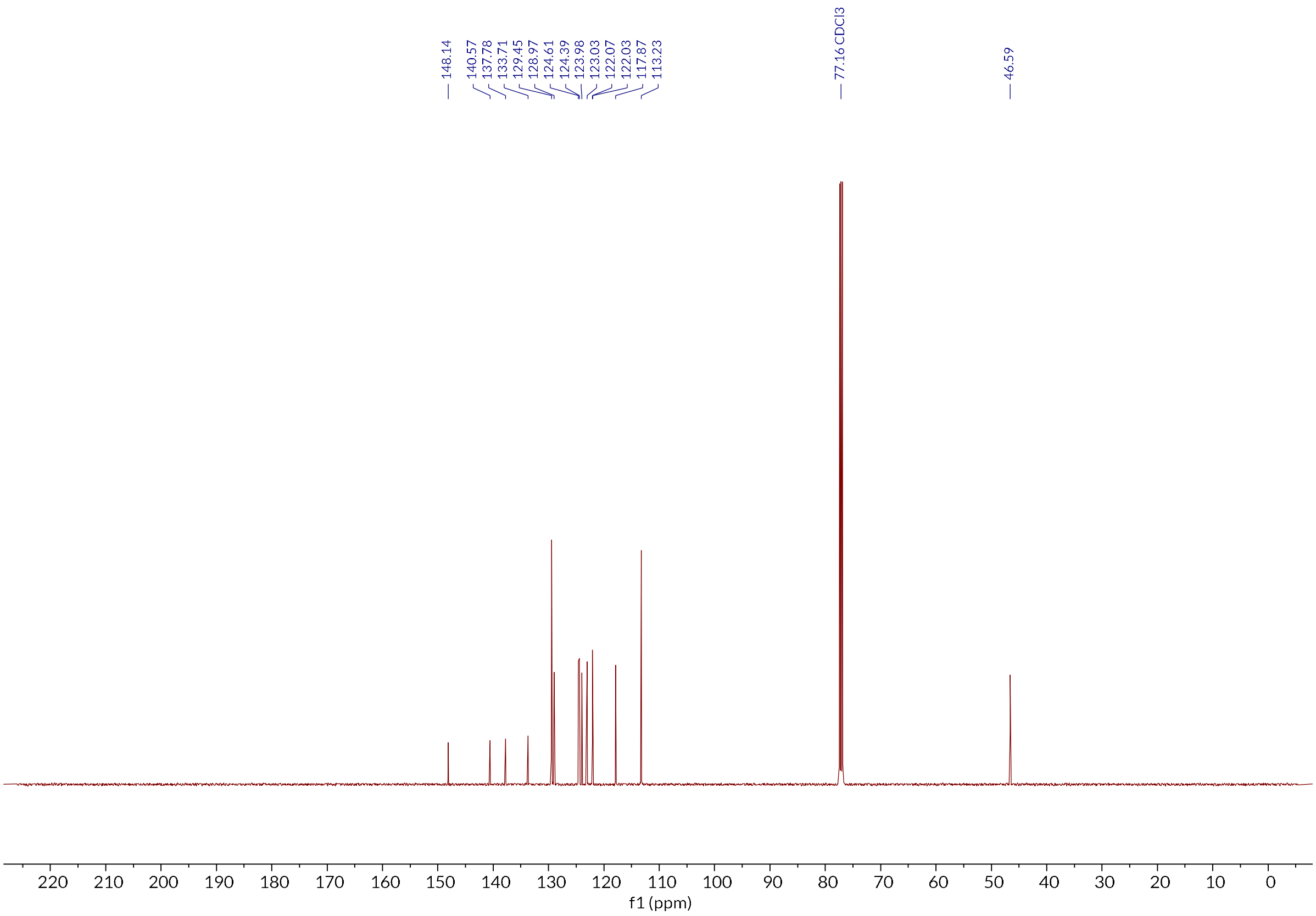

(*E*)-*N*-(3-(Benzo[*b*]thiophen-3-yl)allyl)-*N*-benzylaniline (1ax)

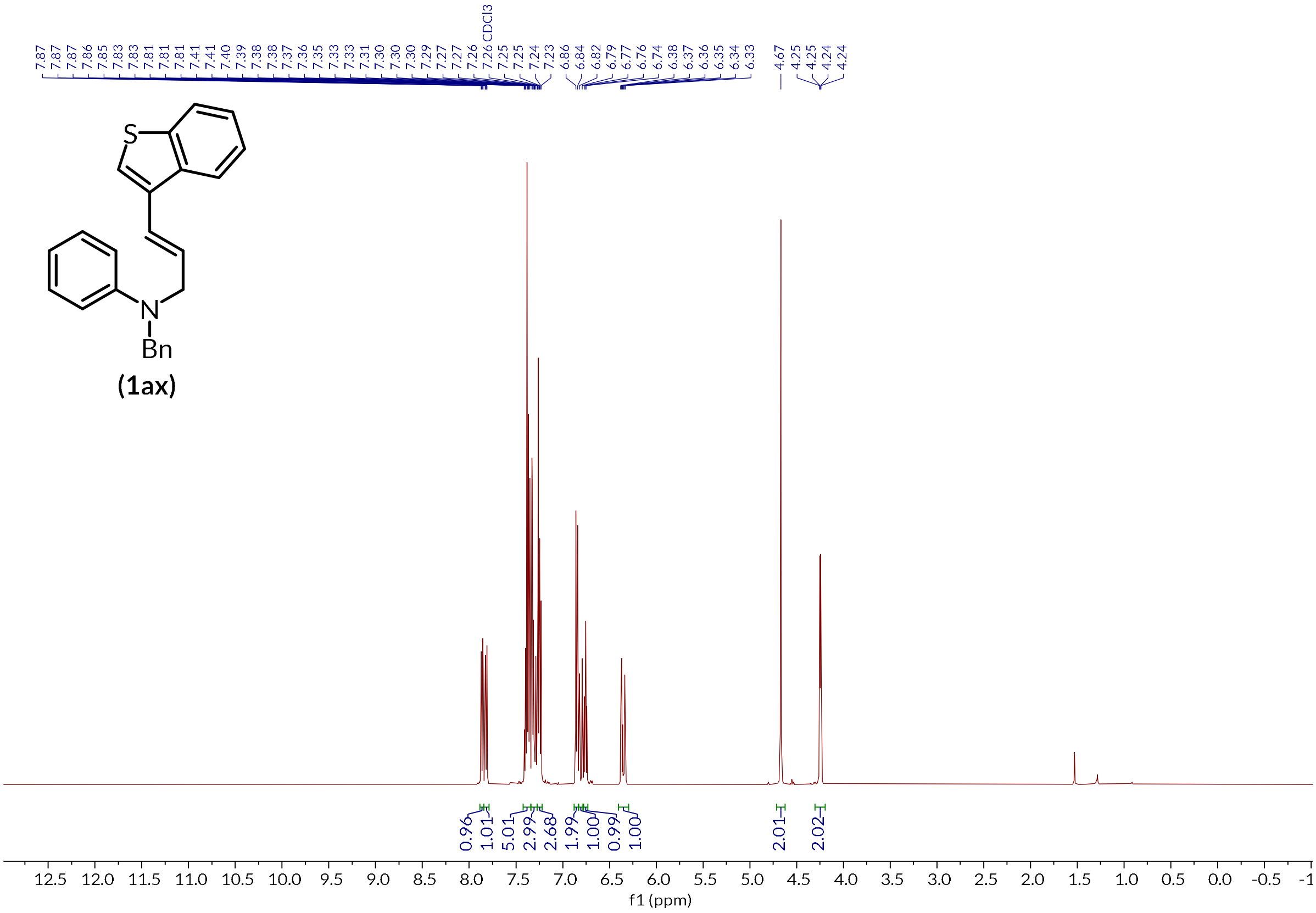

(1ax)

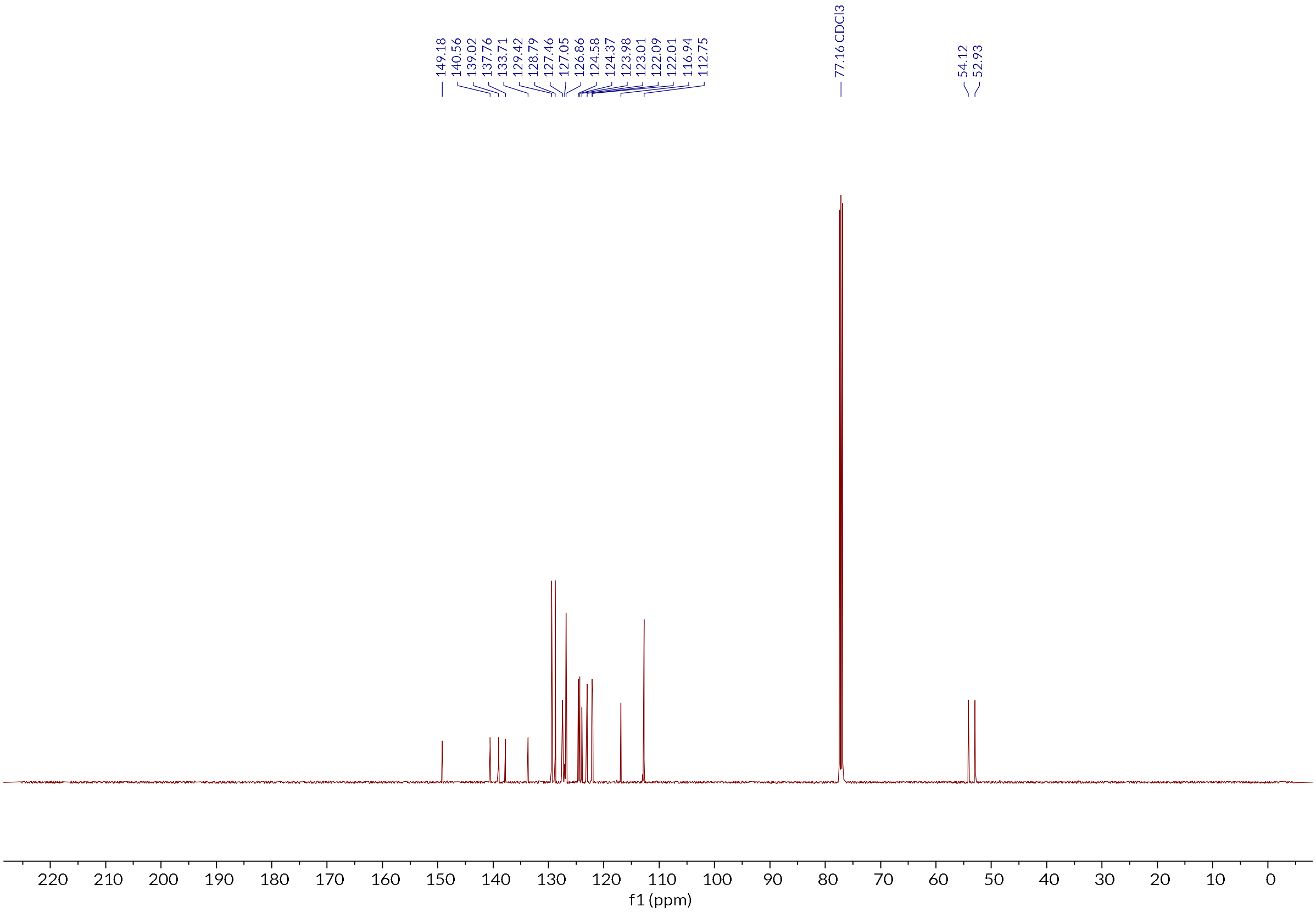

(*E*)-*N*-Benzyl-*N*-(3-(3-(4-fluorophenyl)-1-isopropyl-1*H*-indol-2-yl)allyl)aniline (1ay)

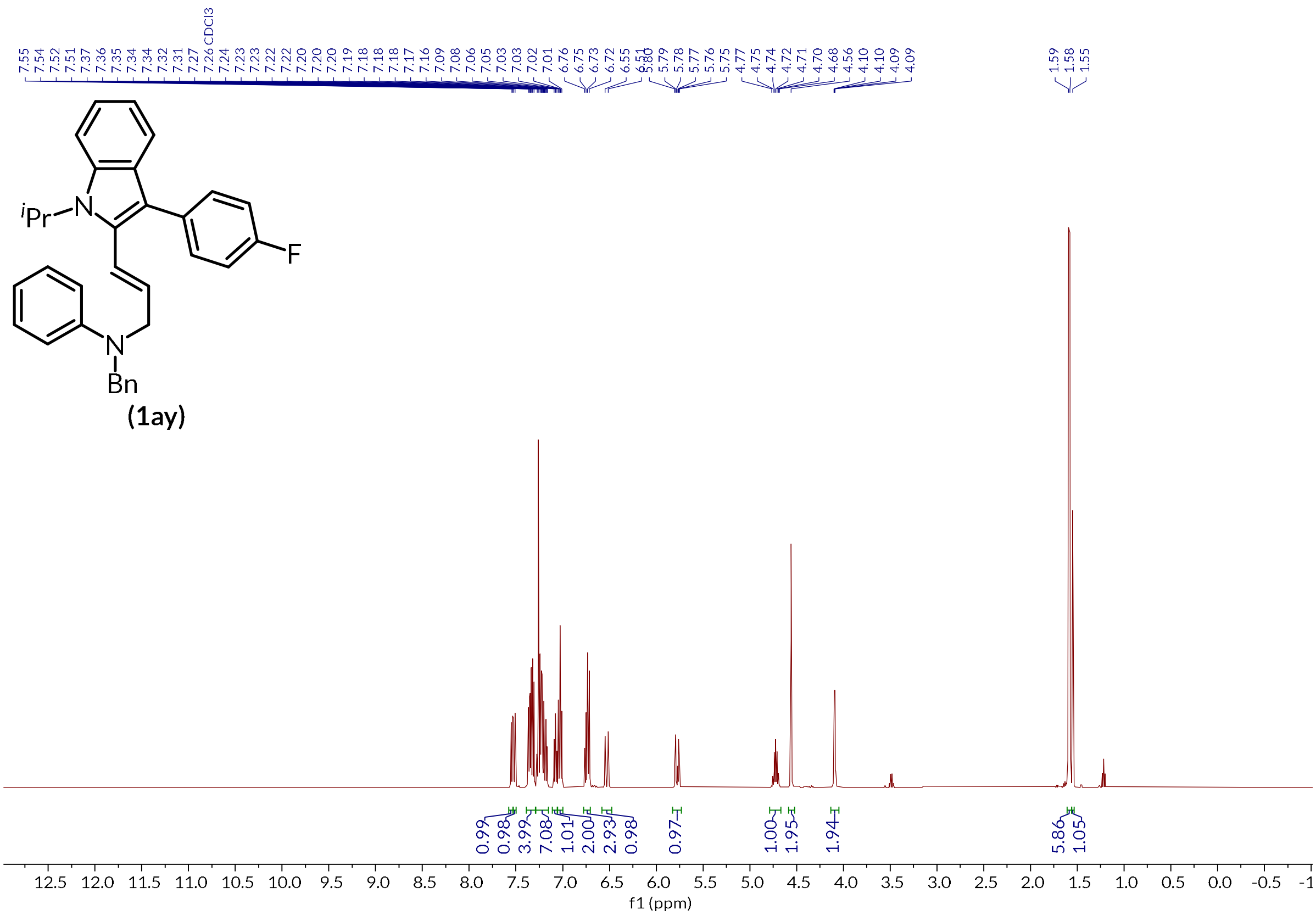

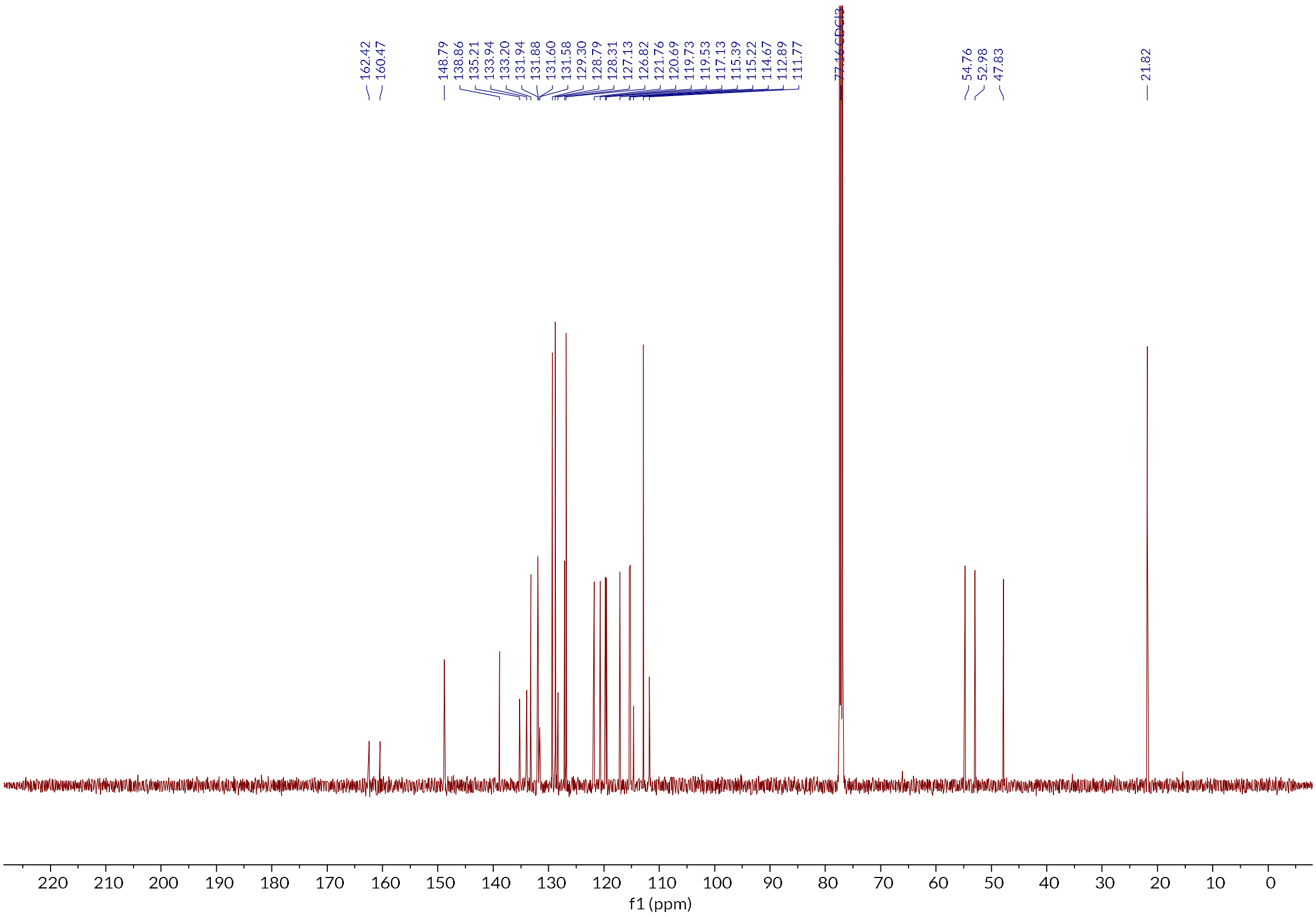

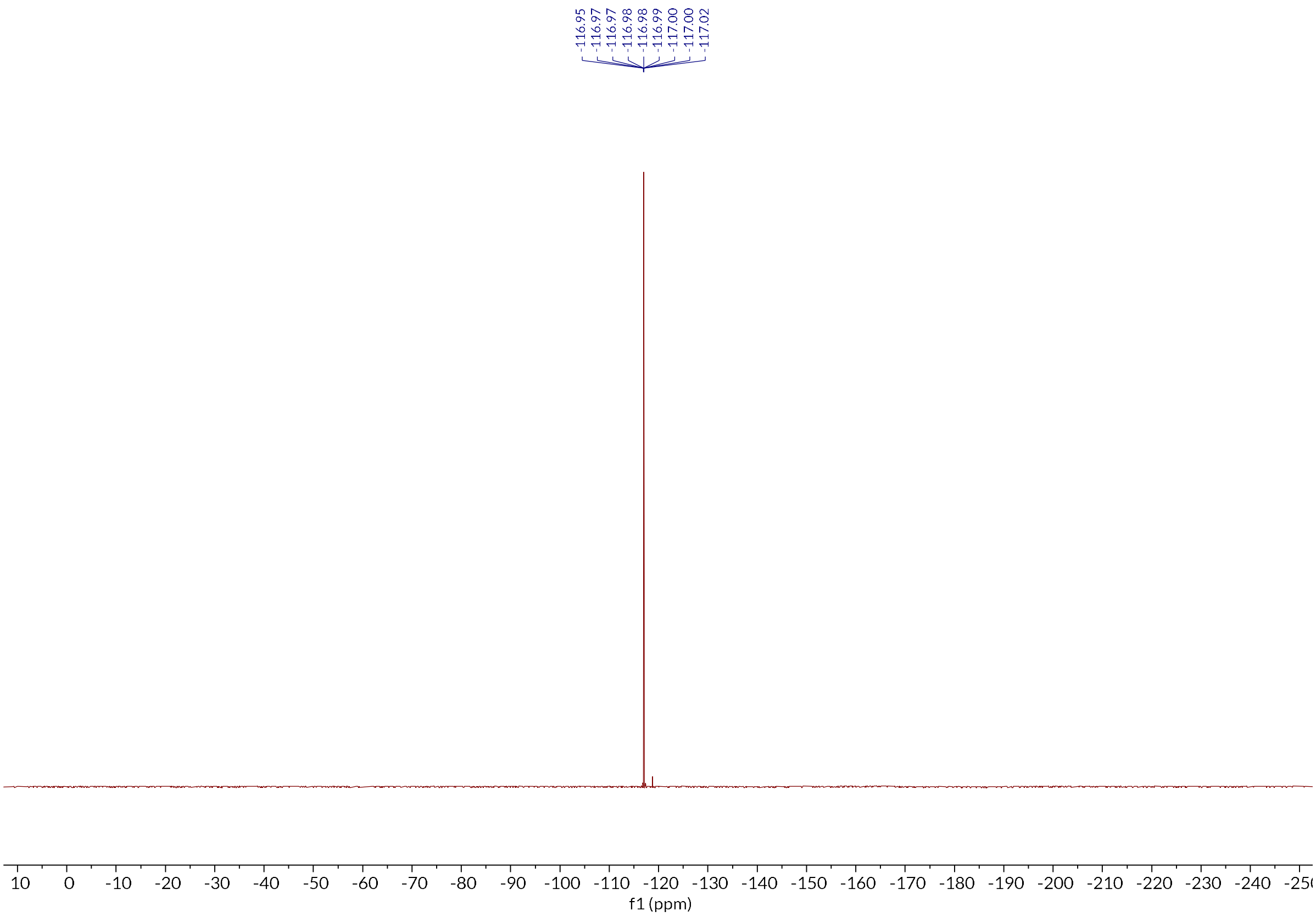
-116.95
-116.97
-116.97
-116.98
-116.98
-116.99
-117.00
-117.00
-117.02
10 0 -10 -20 -30 -40 -50 -60 -70 -80 -90 -100 -110 -120 -130 -140 -150 -160 -170 -180 -190 -200 -210 -220 -230 -240
f1 (ppm)

(2*E*,4*E*)-5-Phenylpenta-2,4-dienal (SI-17)

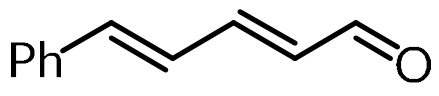

(SI-17)

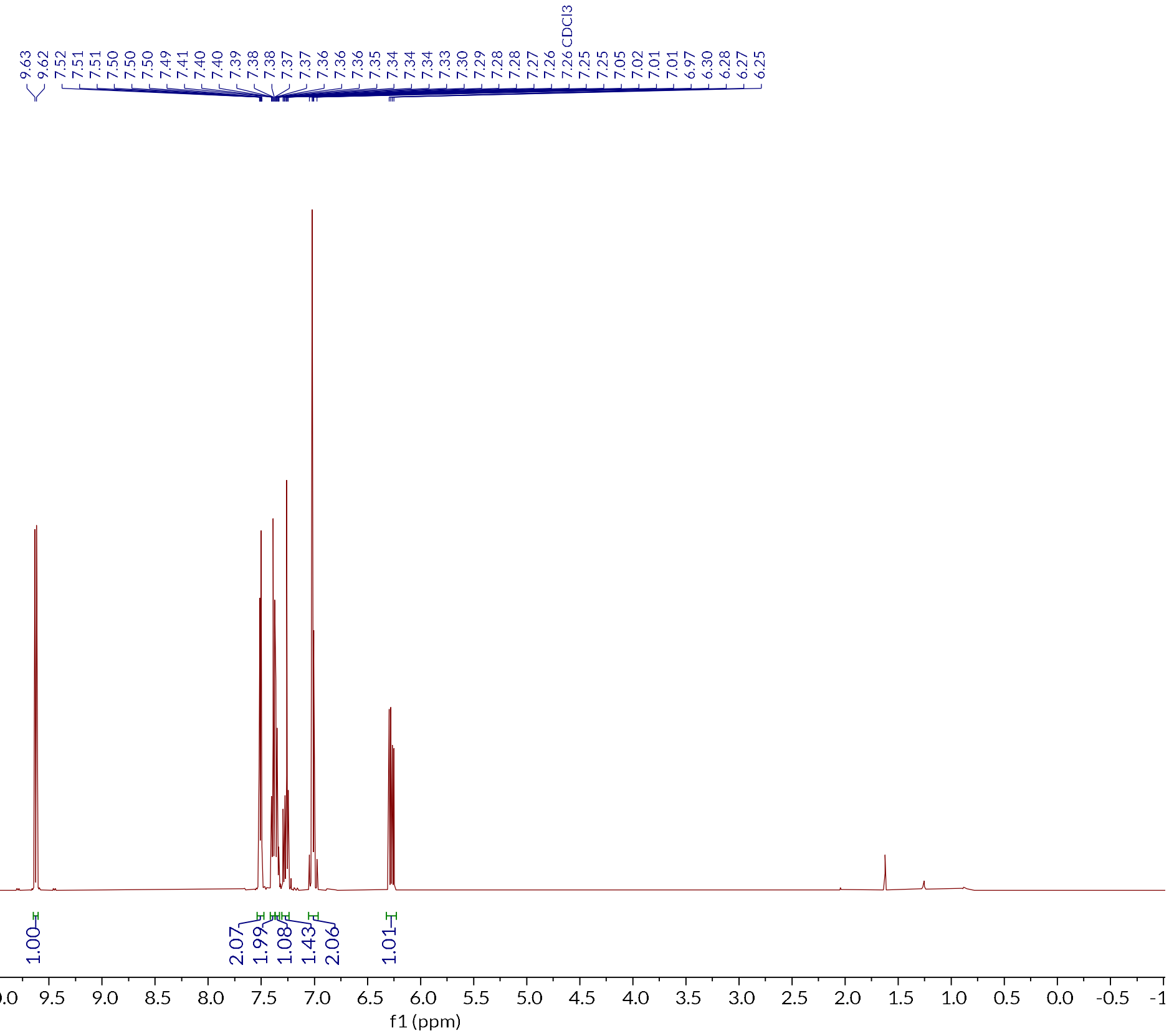

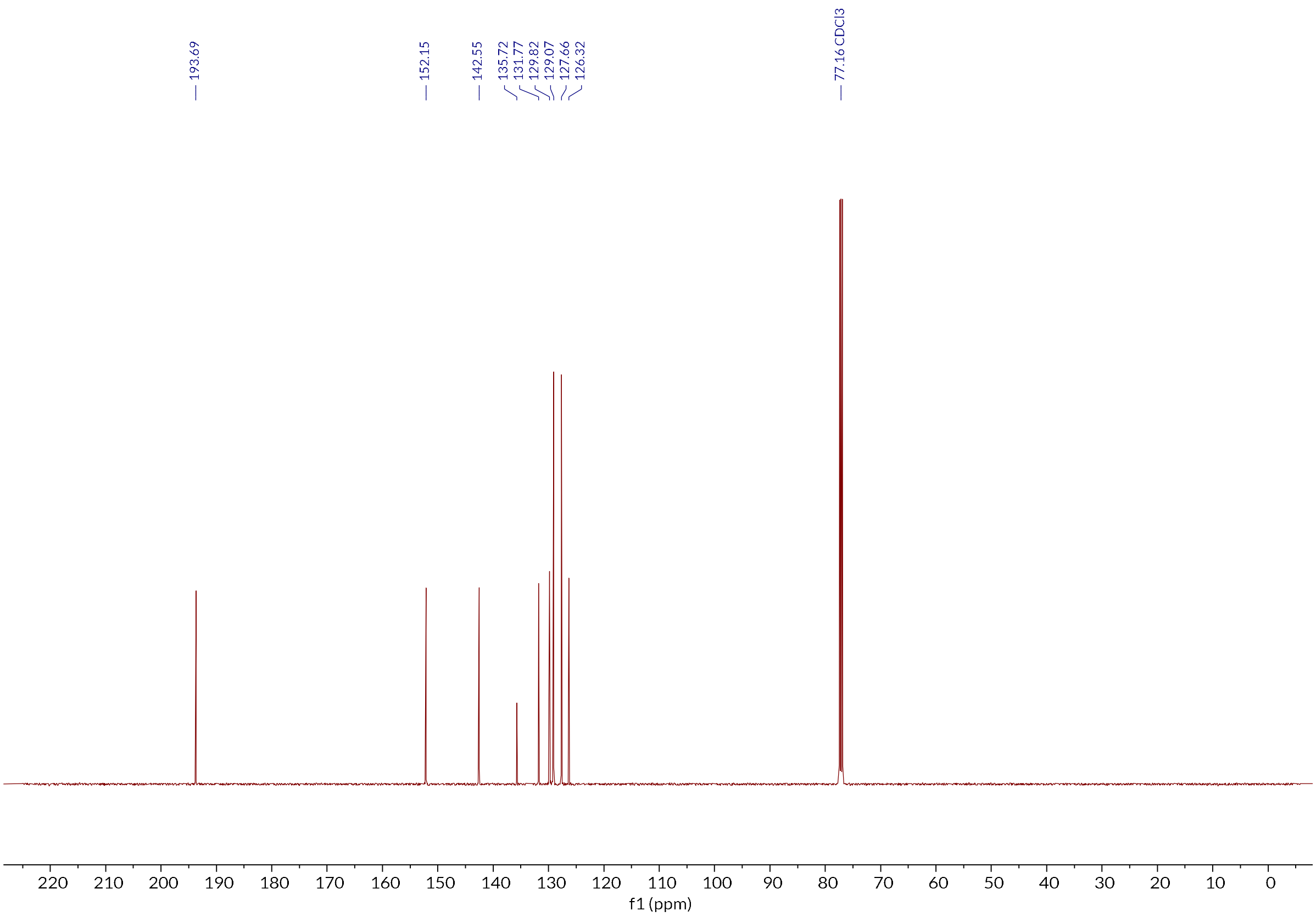

N-((2E,4E)-5-Phenylpenta-2,4-dien-1-yl)aniline (SI-18)

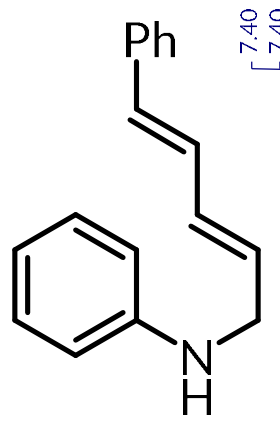

(SI-18)

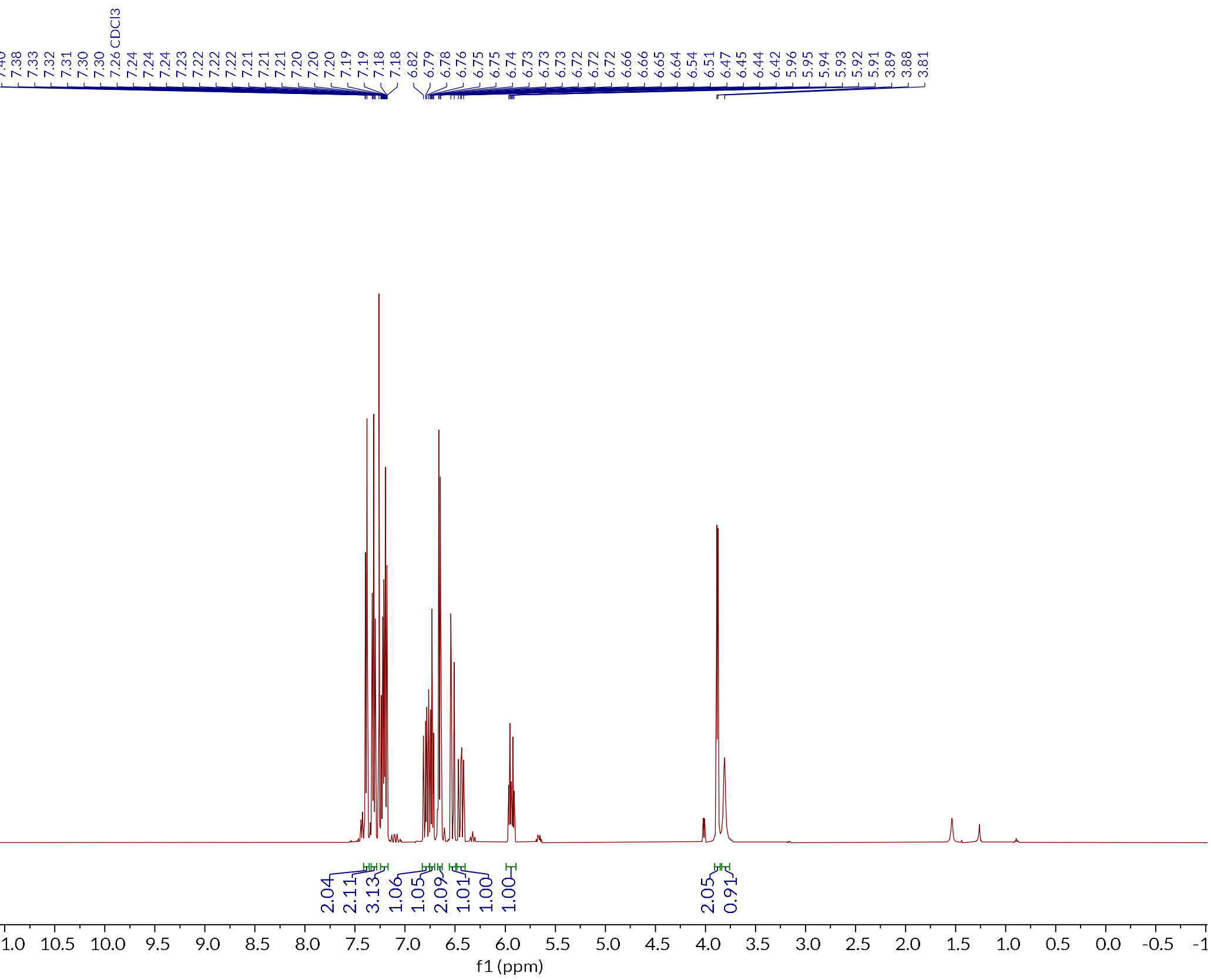

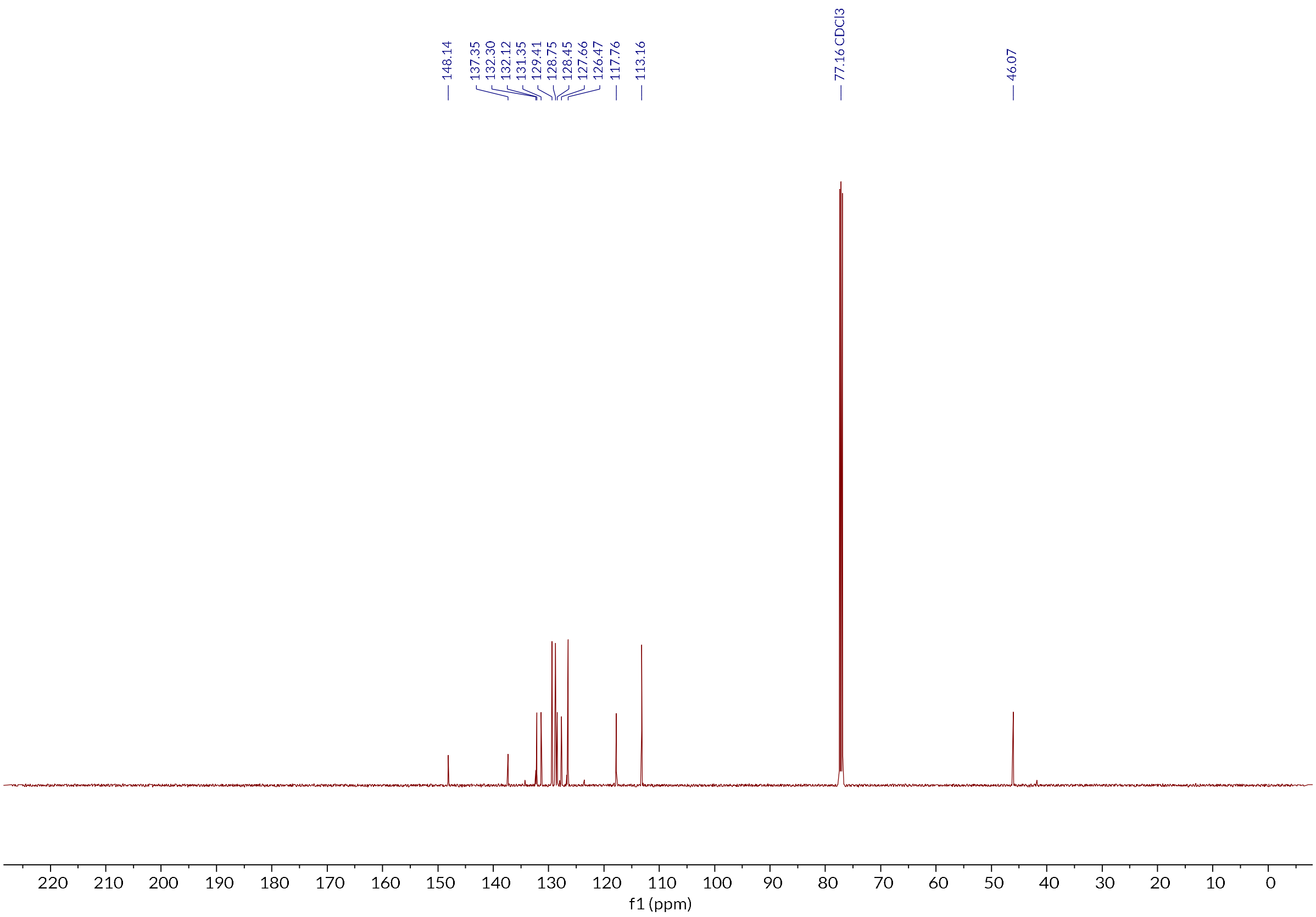

N-Benzyl-*N*-((2*E*,4*E*)-5-phenylpenta-2,4-dien-1-yl)aniline (1az)

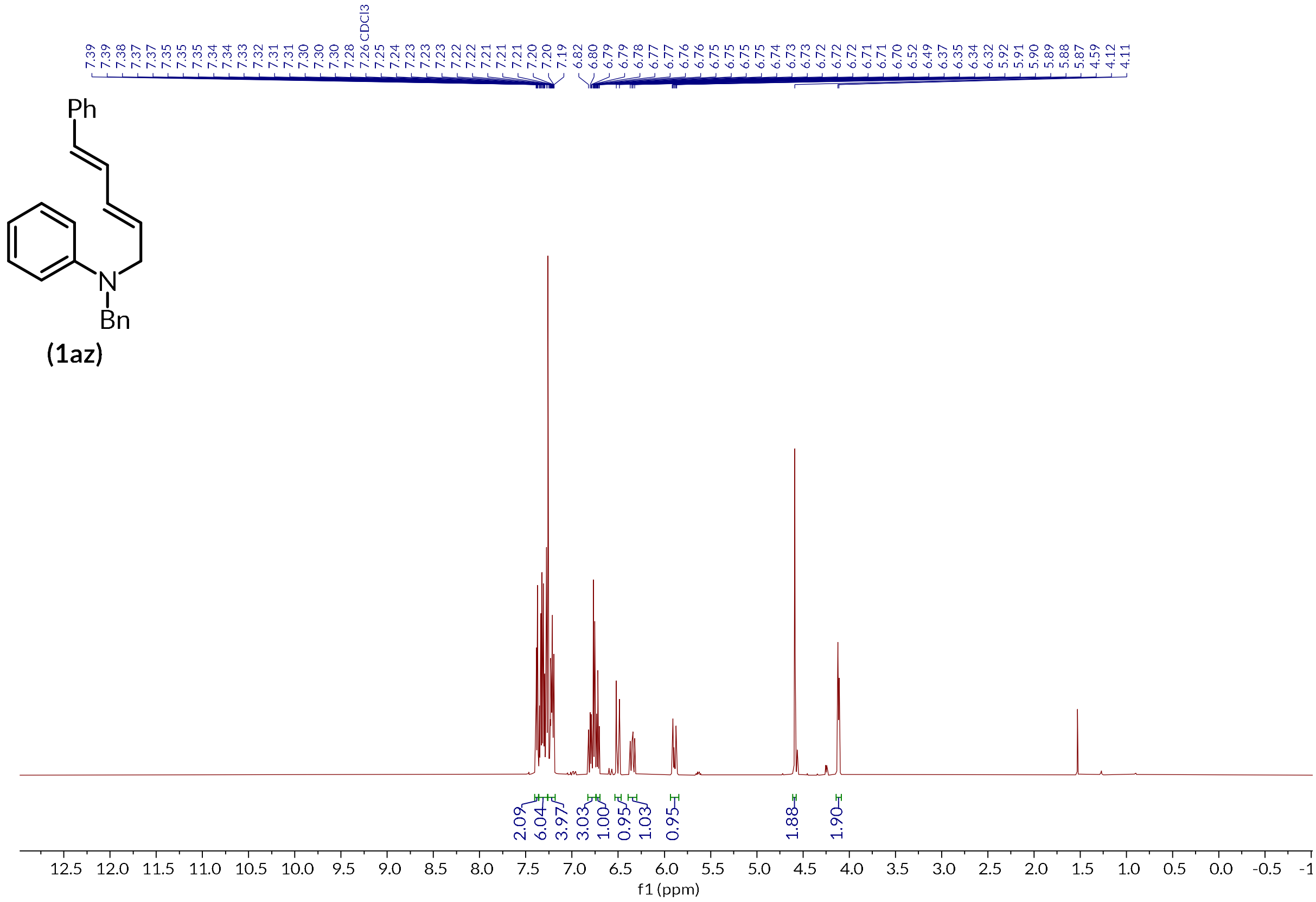

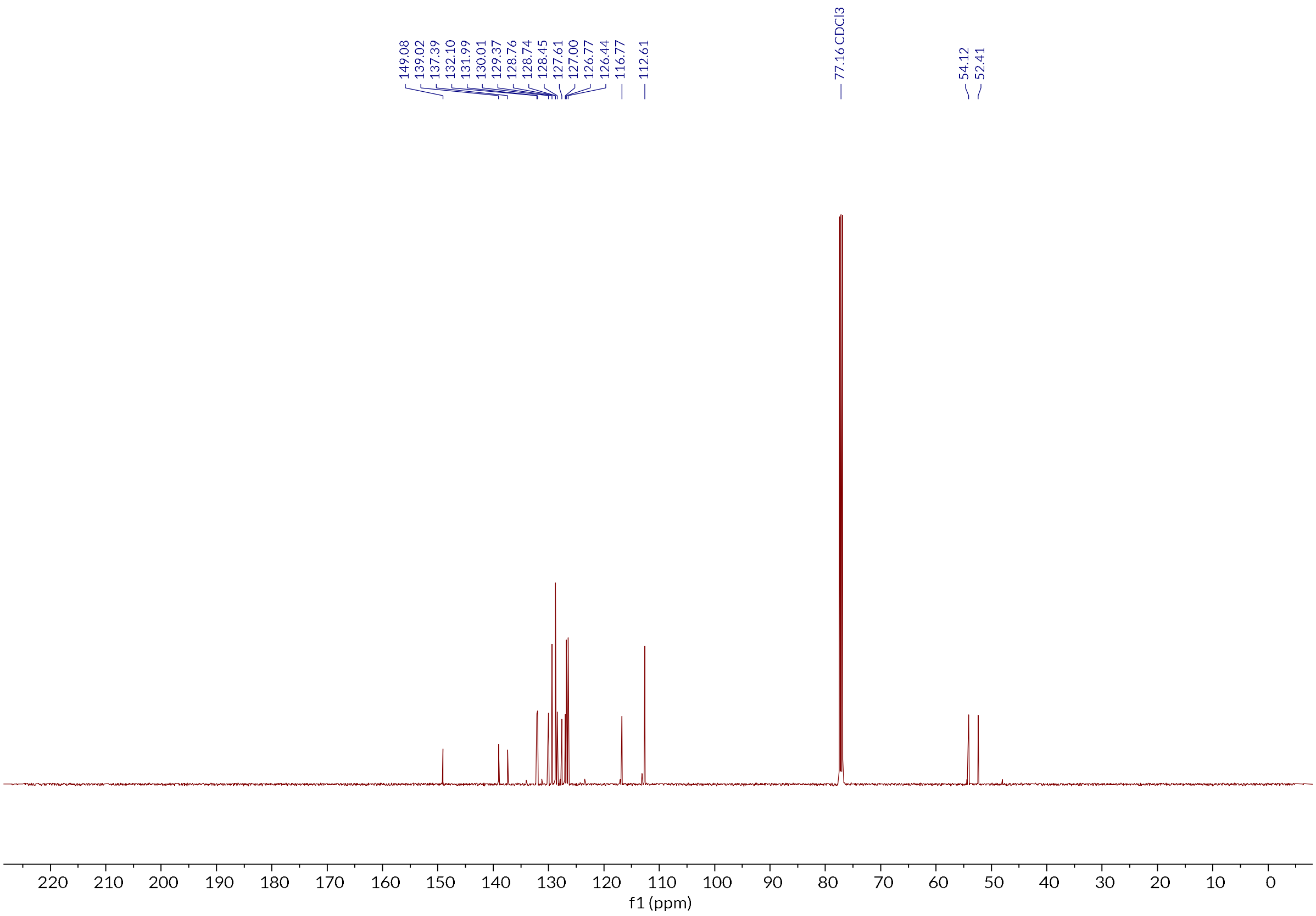

(*E*)-*N*-(4-Phenylbut-3-en-2-yl)aniline (SI-19)

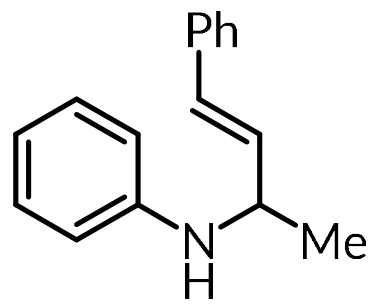

(SI-19)

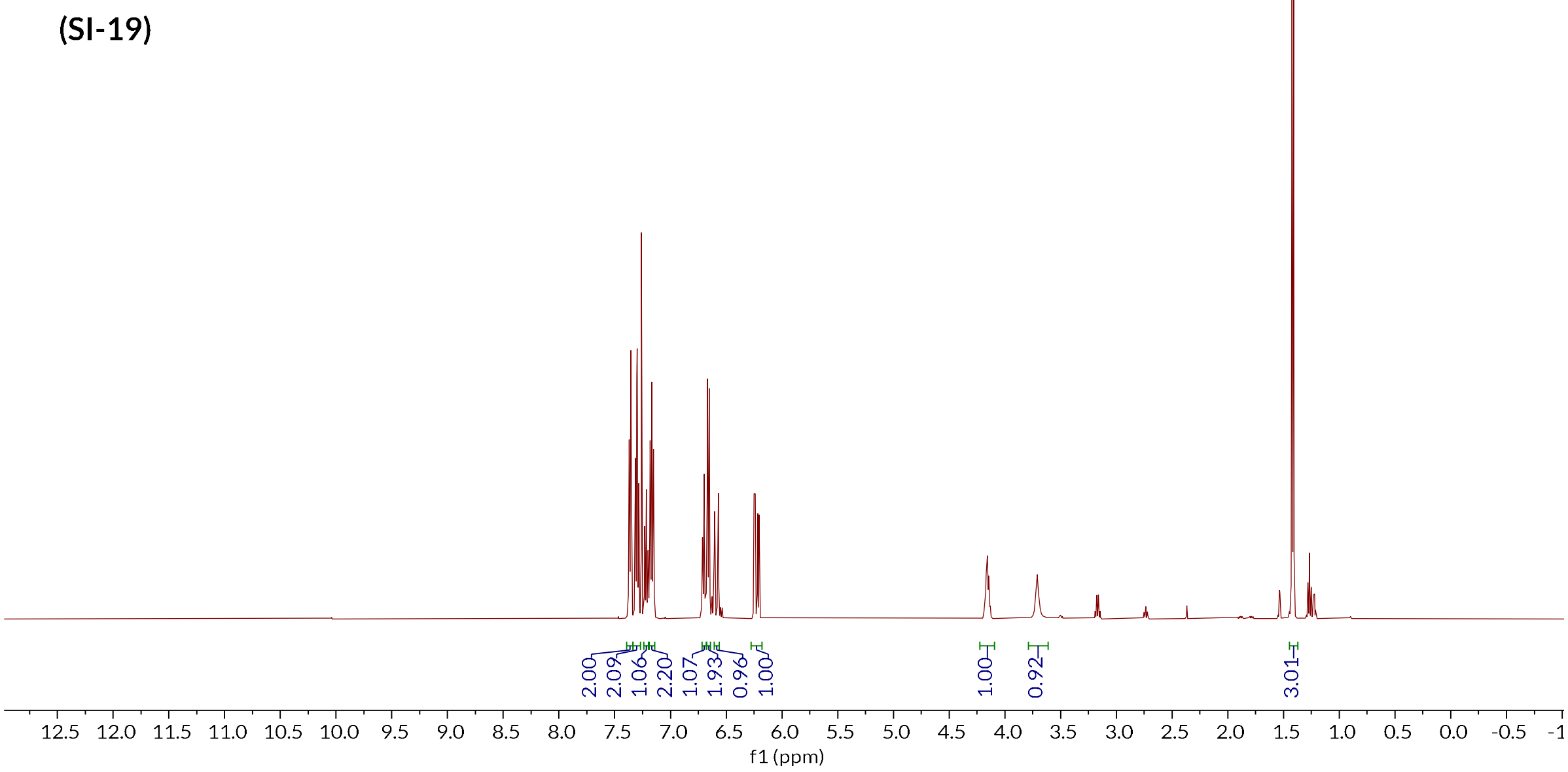

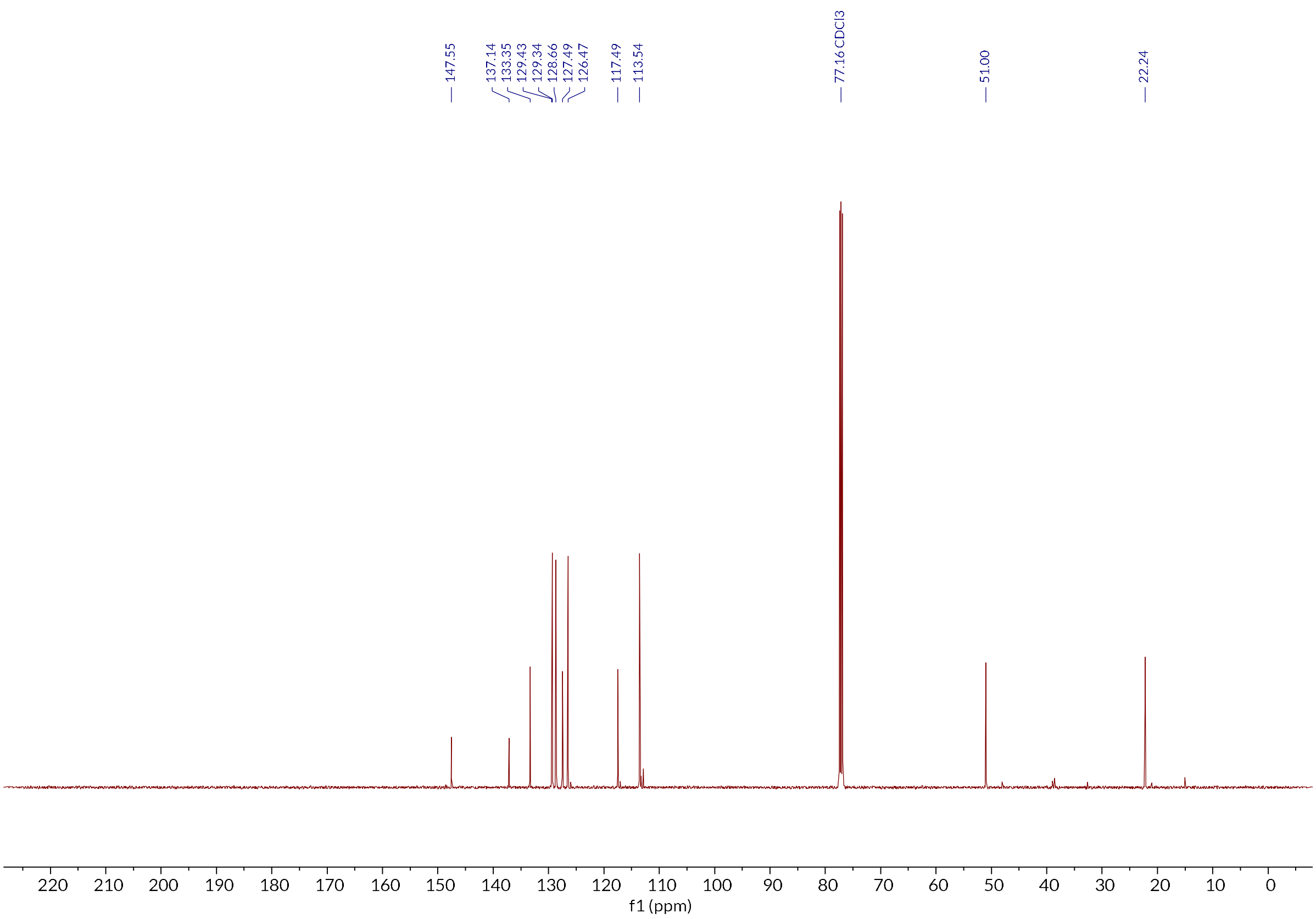

(*E*)-*N*-Benzyl-*N*-(4-phenylbut-3-en-2-yl)aniline (1ba)

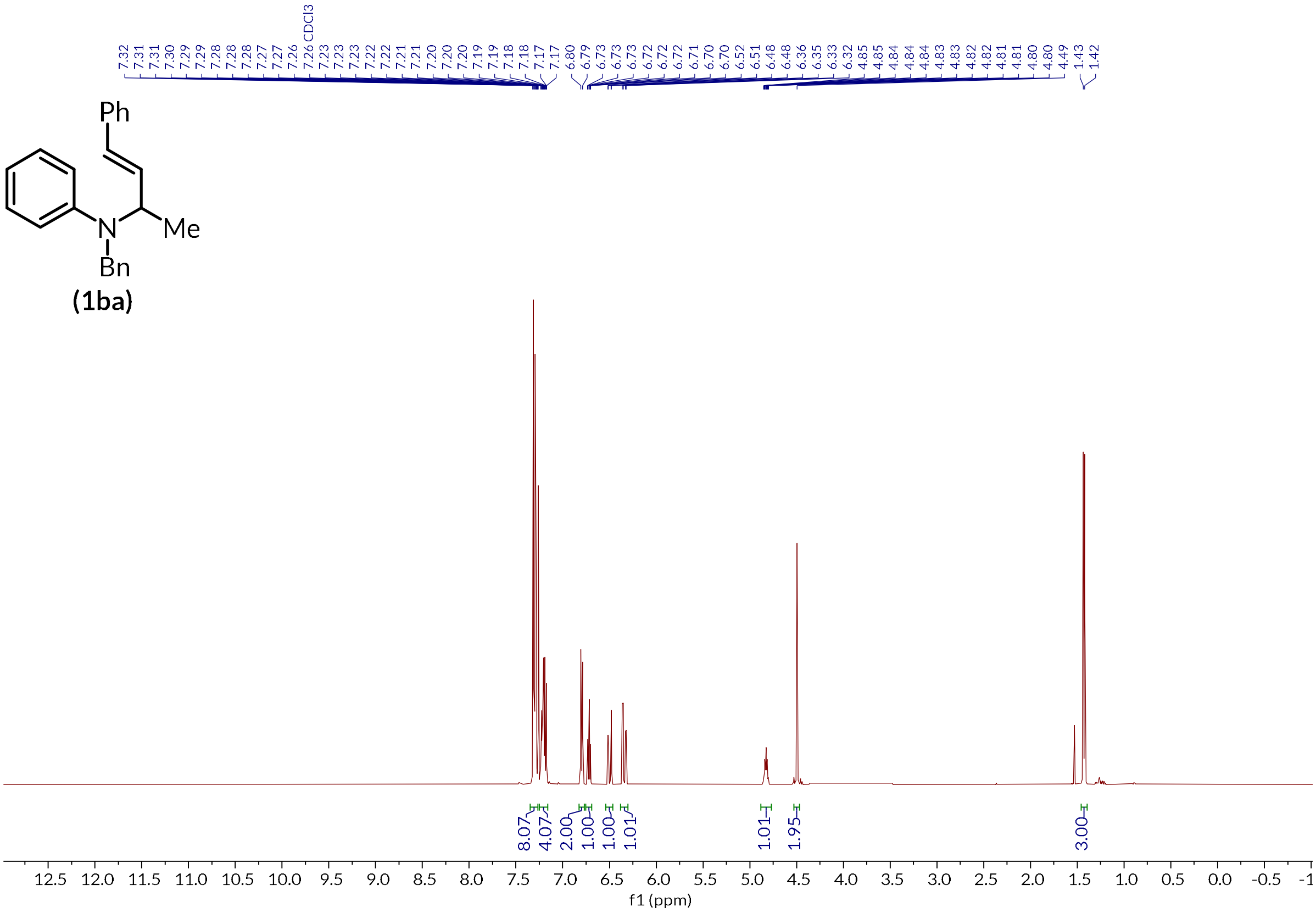

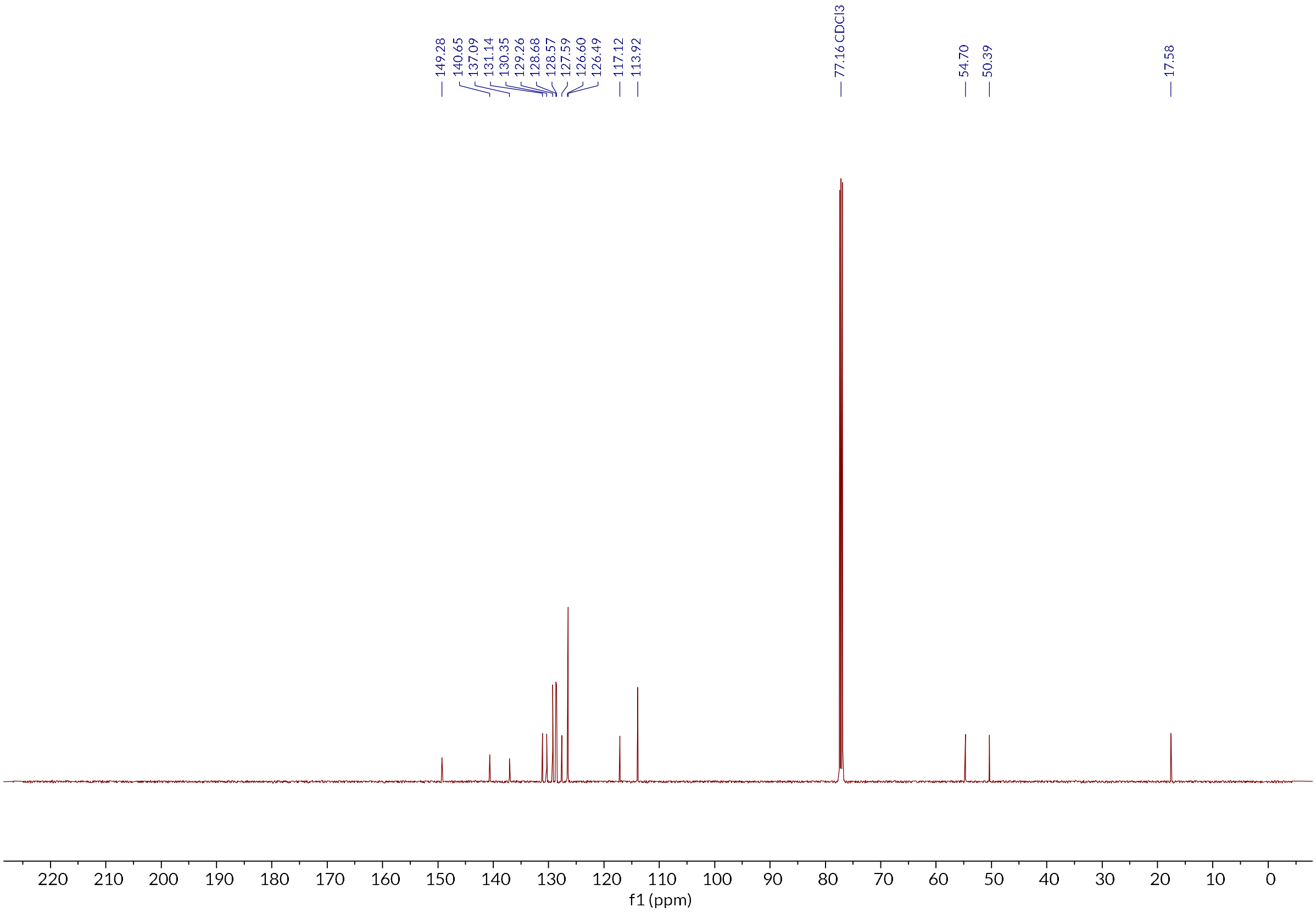

N-(But-3-en-1-yl)aniline (SI-20)

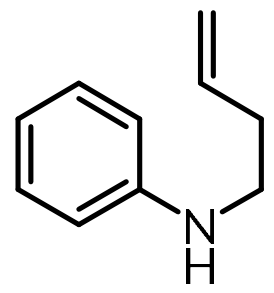

(SI-20)

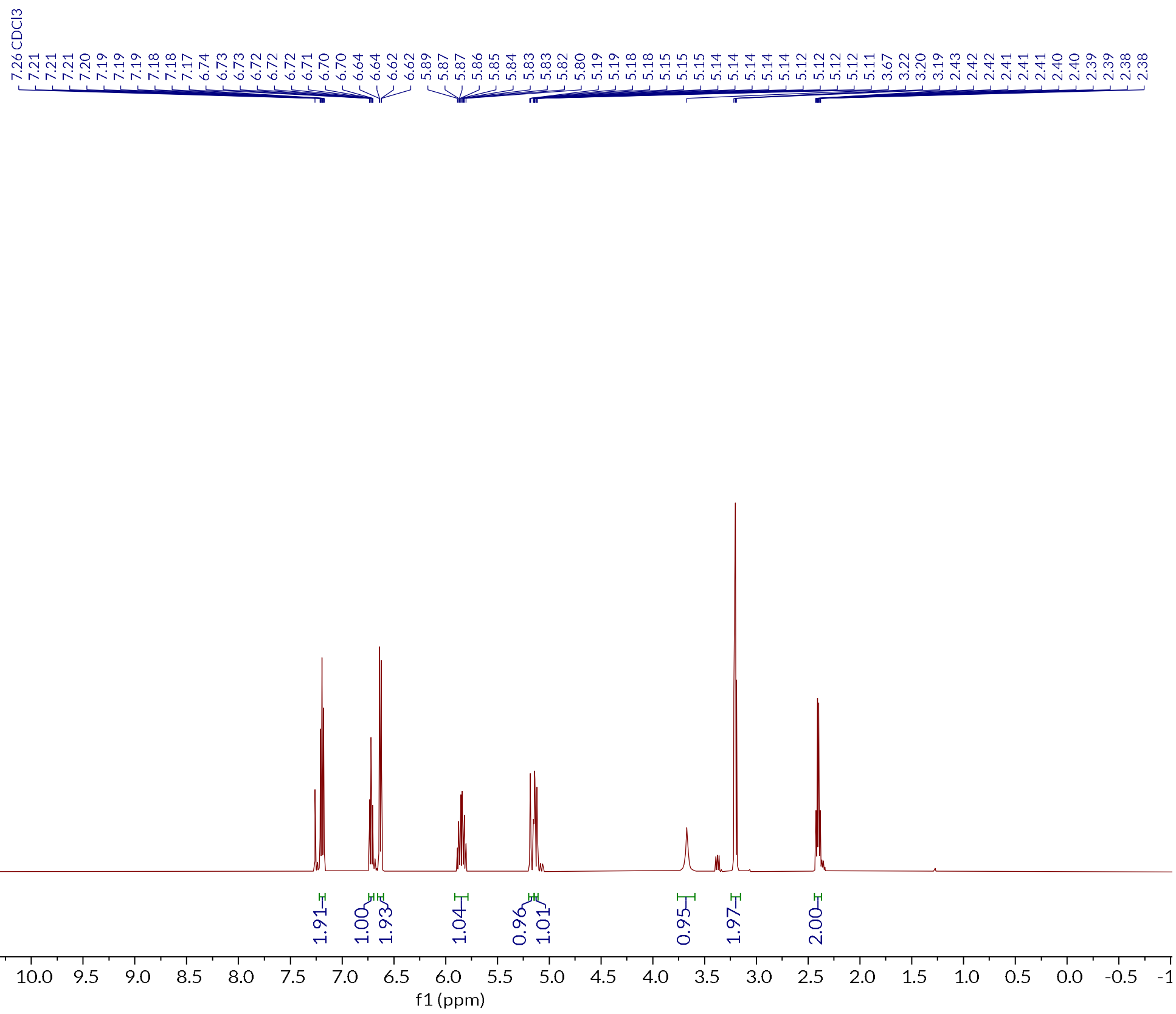

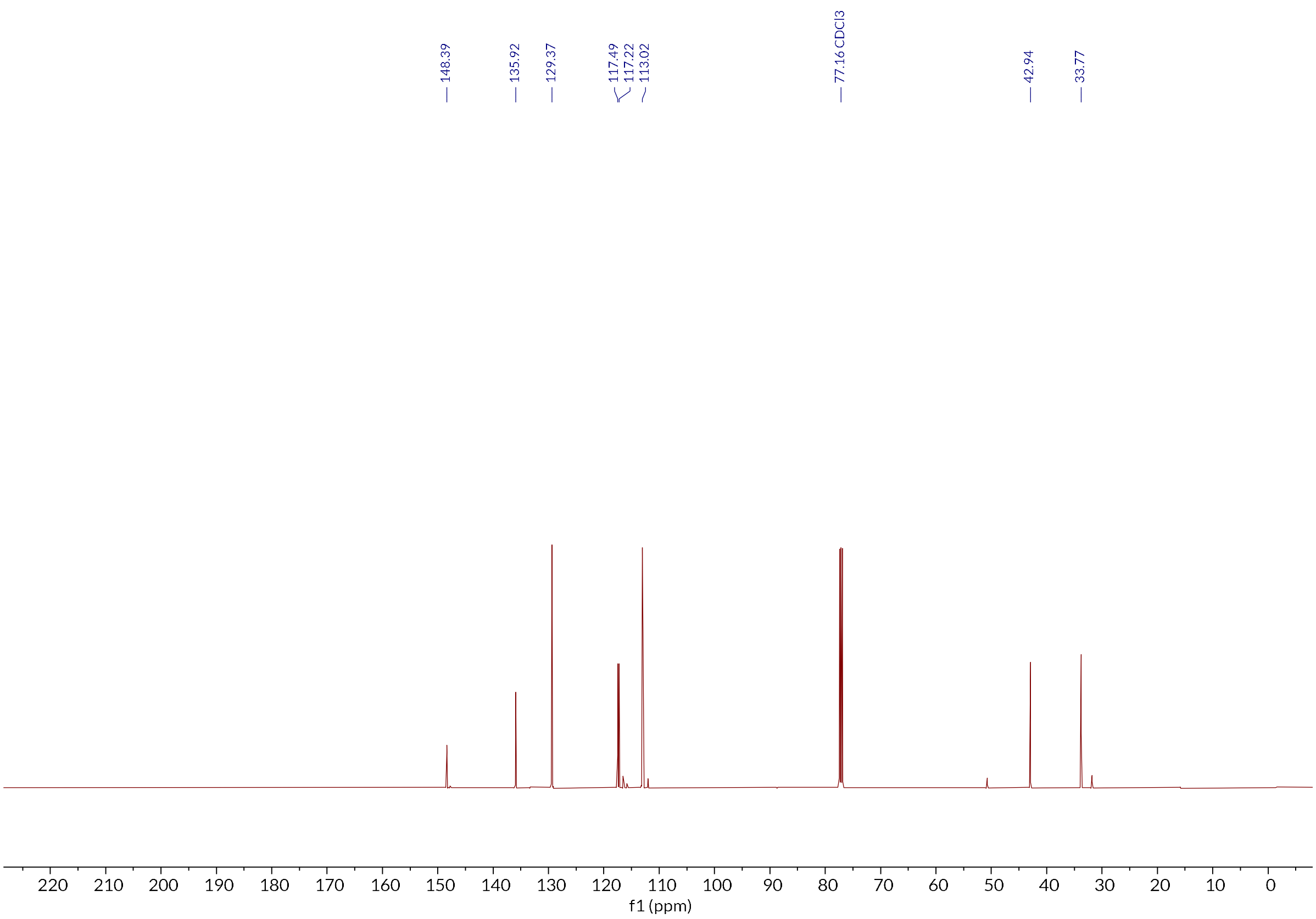

N-Benzyl-*N*-(but-3-en-1-yl)aniline (1bb)

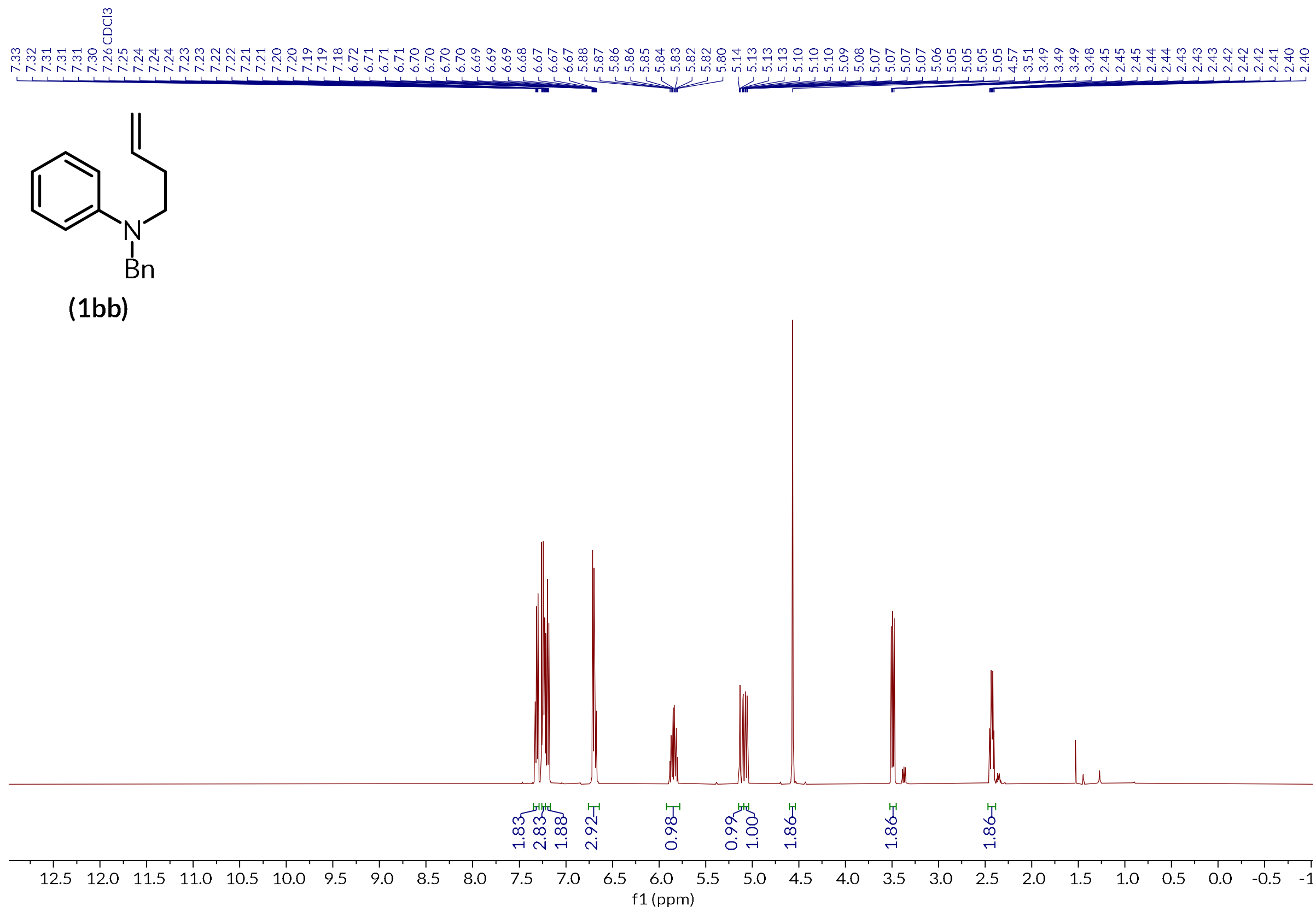

(1bb)

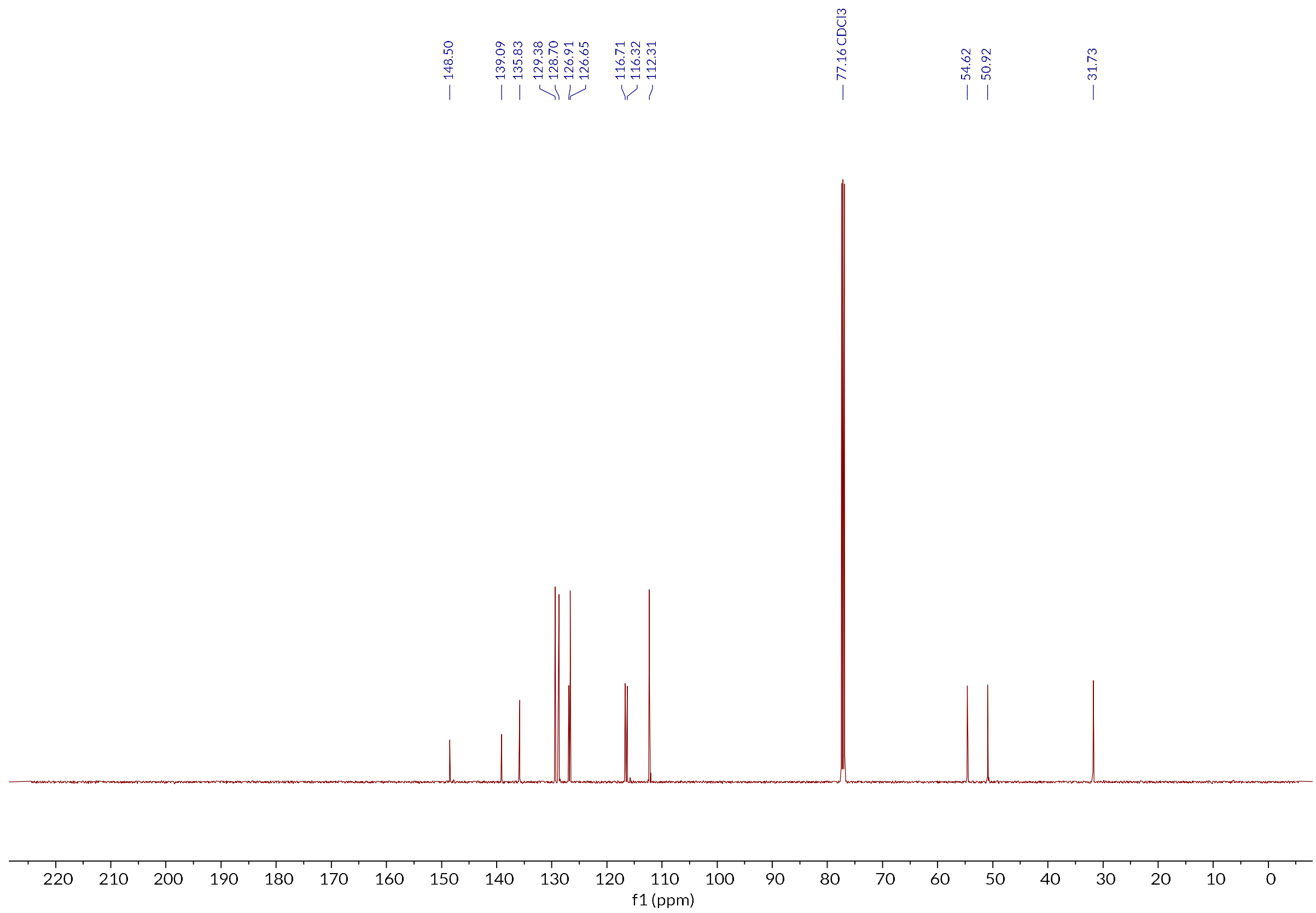

4-(*Tert*-butyl)-*N*-cinnamylaniline (SI-21)

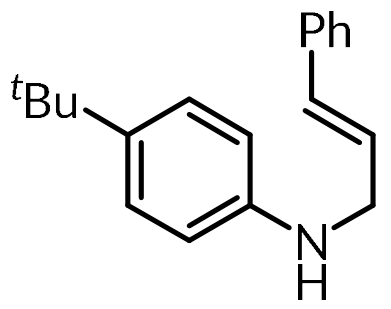

(SI-21)

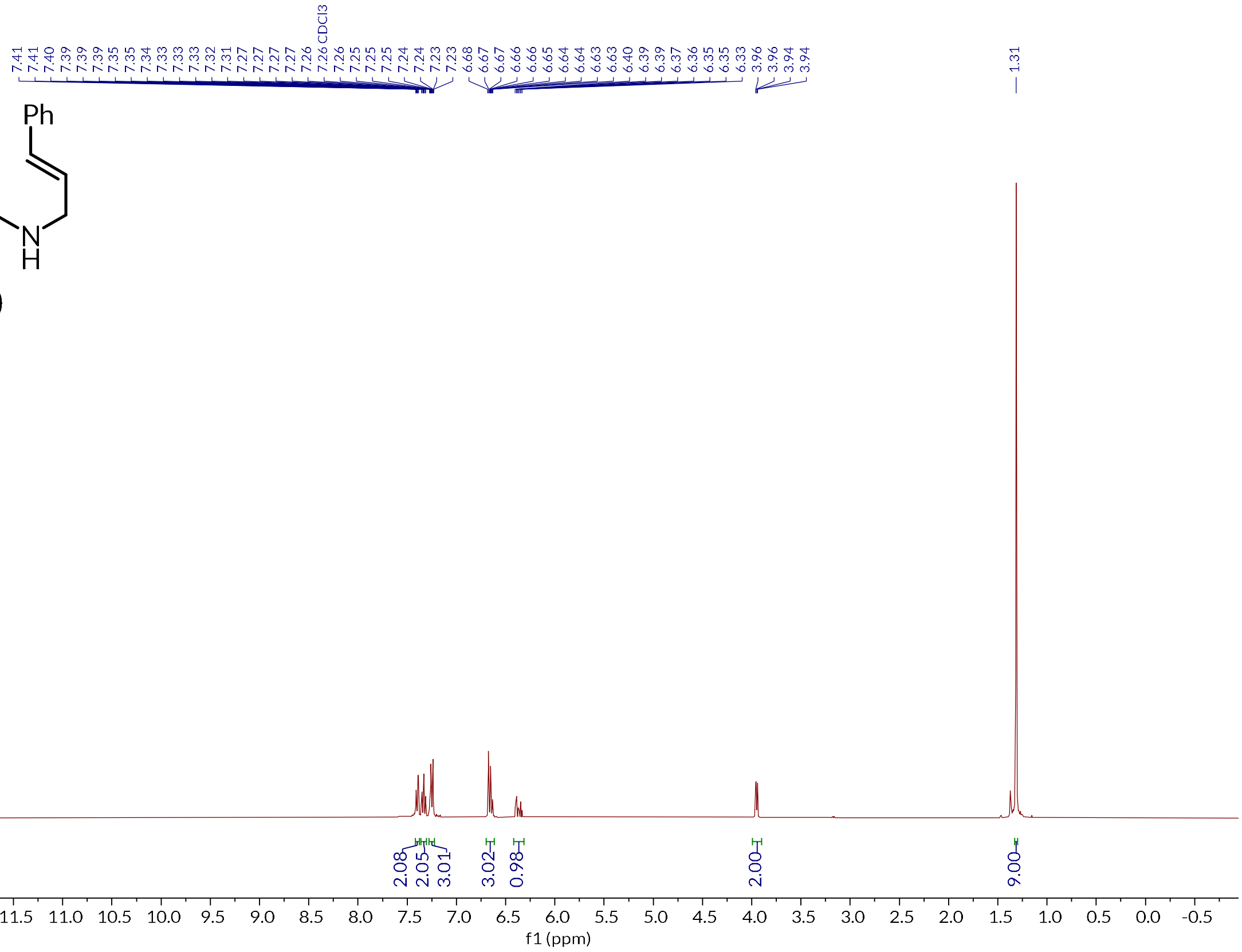

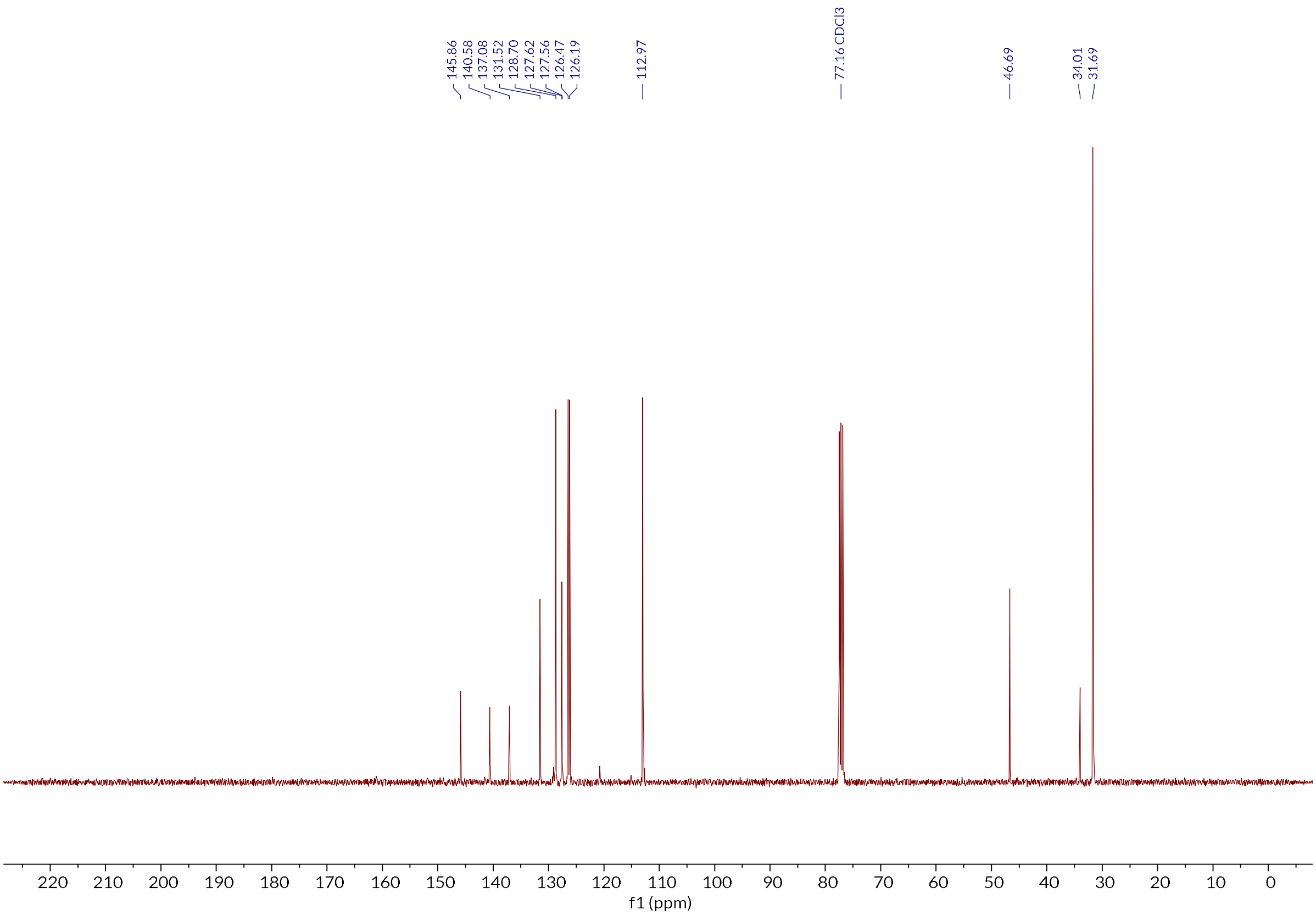

N-Benzyl-4-(*tert*-butyl)-N-cinnamylaniline (1bc)

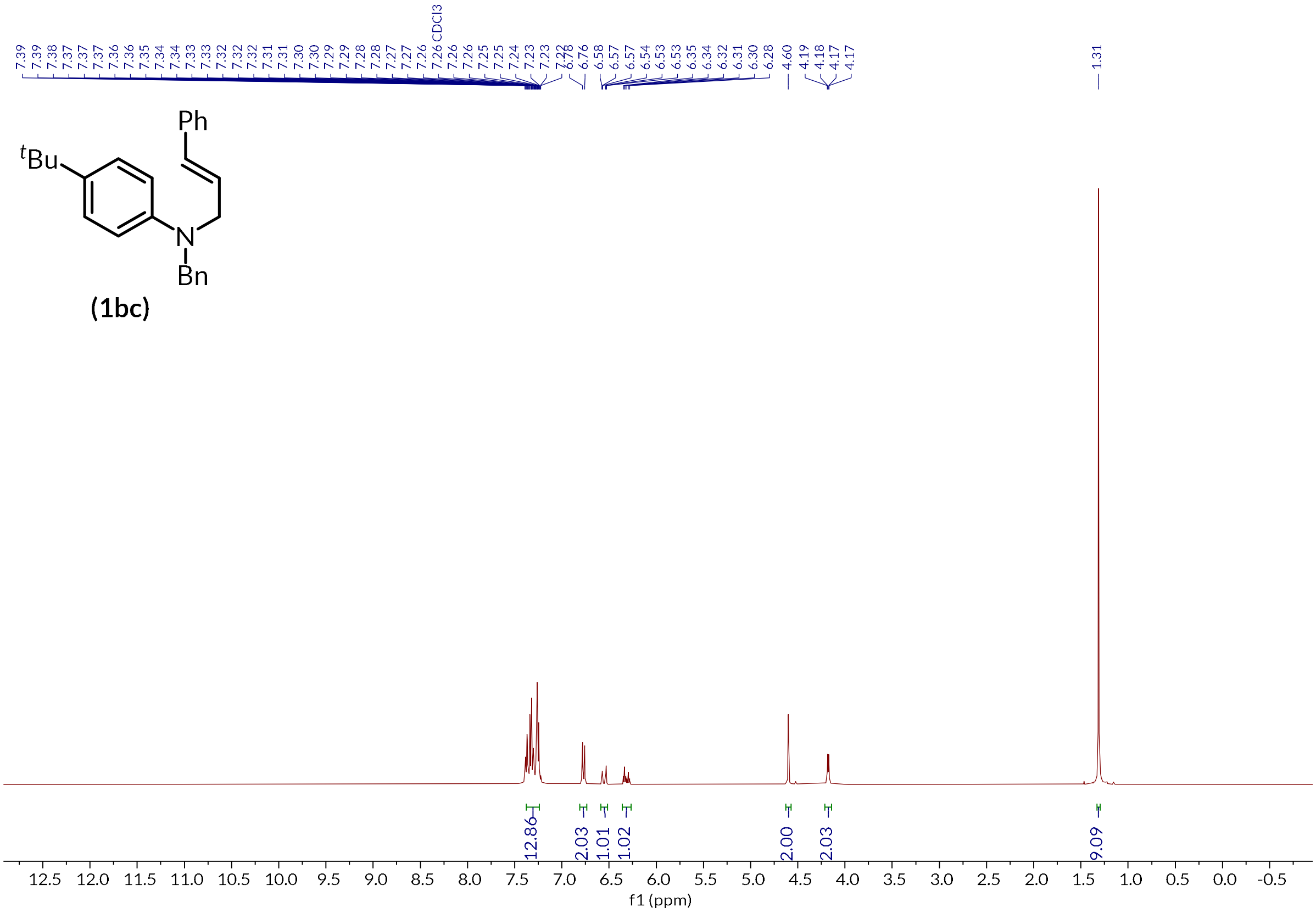

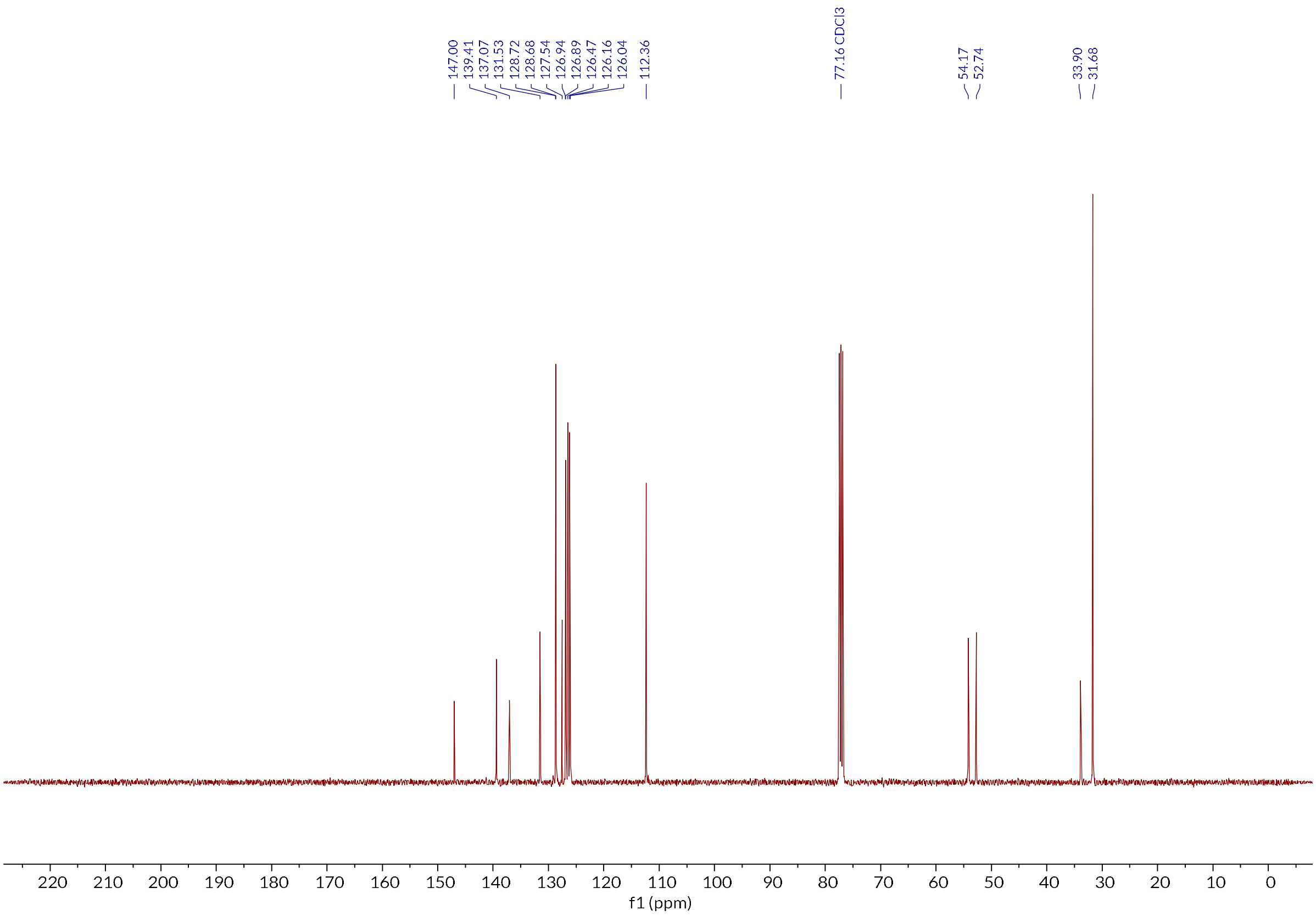

N-Benzyl-4-methoxyaniline (SI-22)

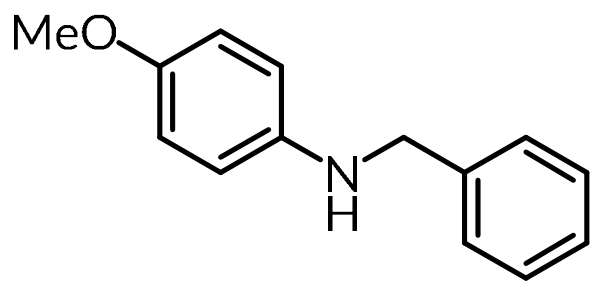

(SI-22)

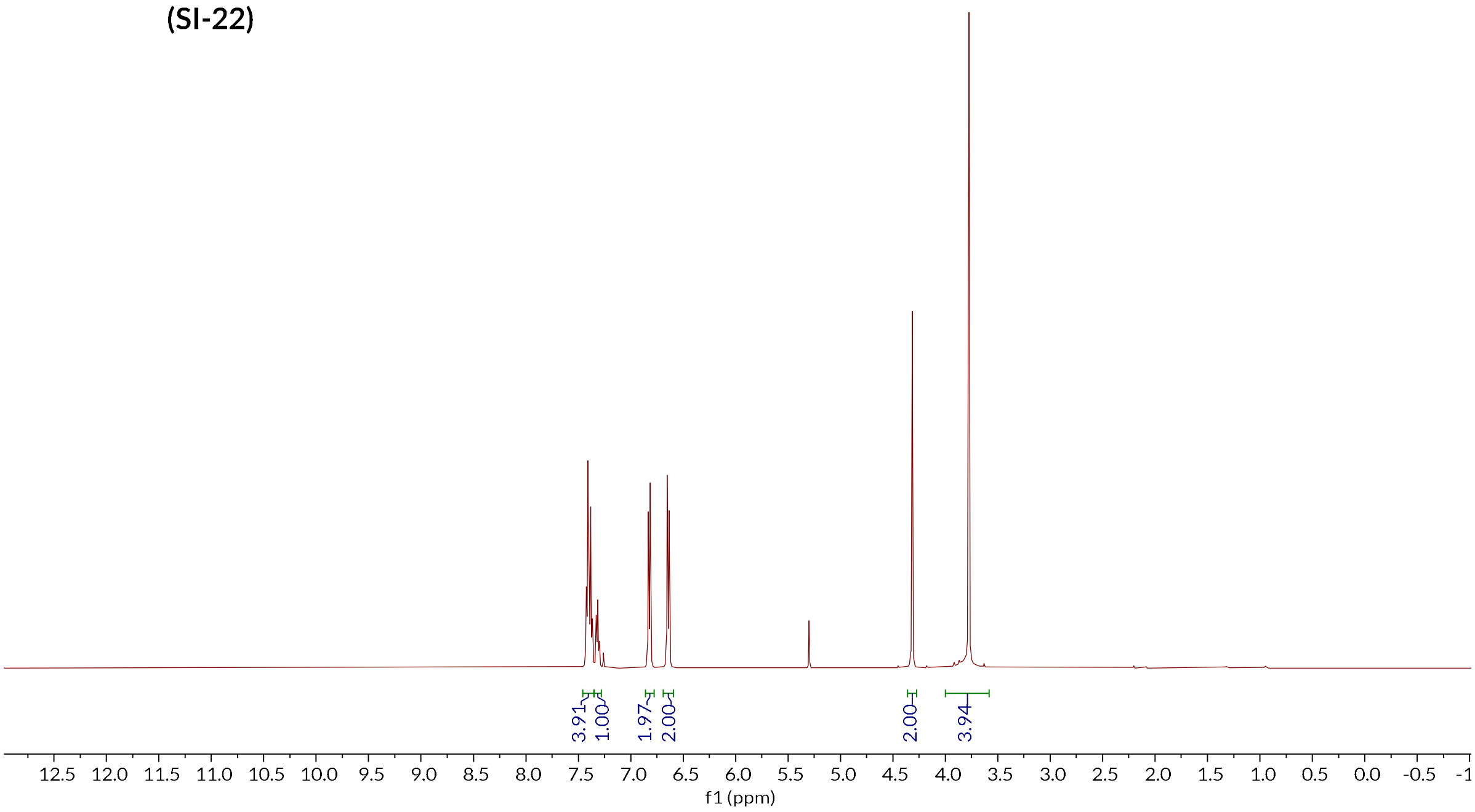

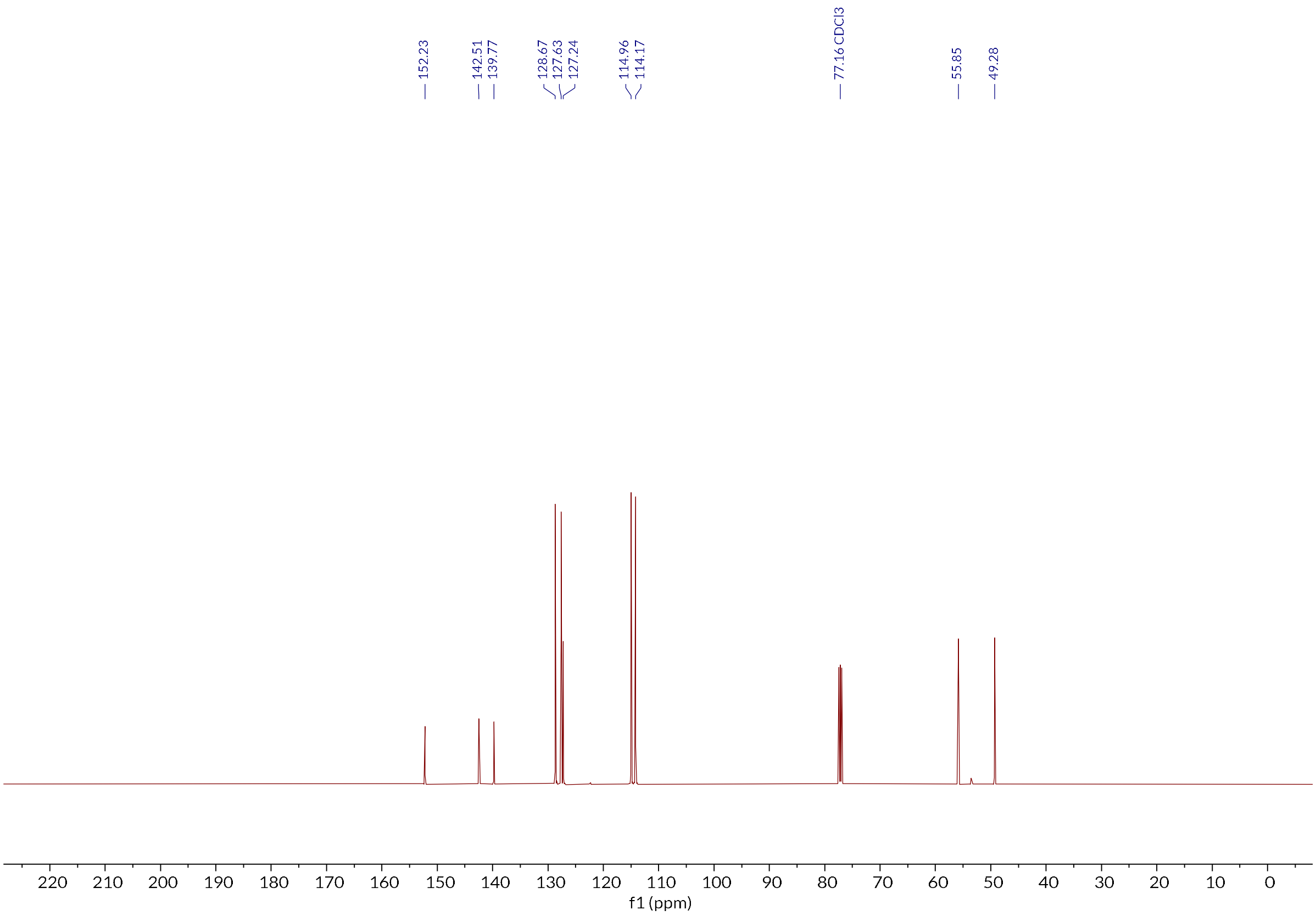

N-Benzyl-*N*-cinnamyl-4-methoxyaniline (1bd)

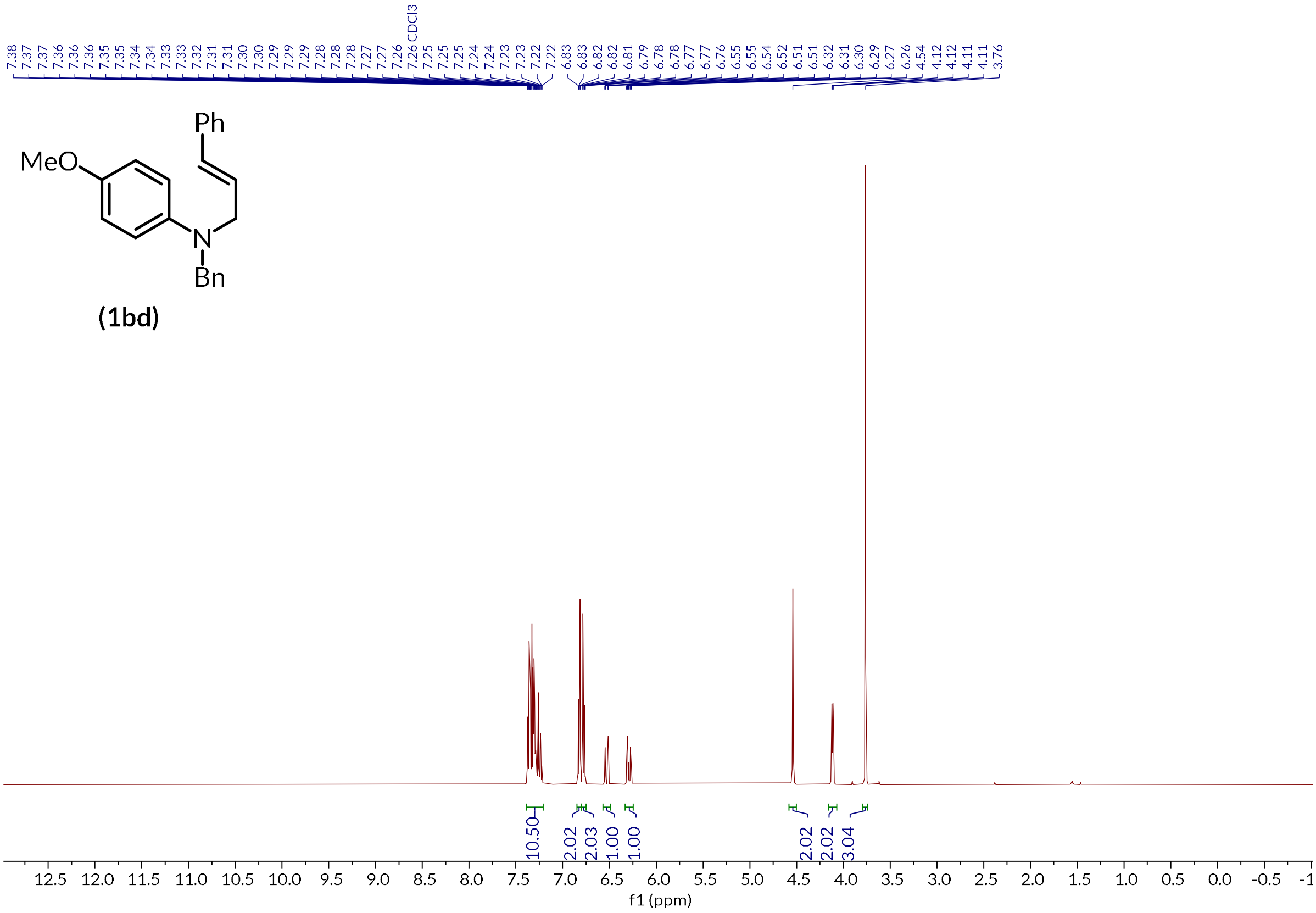

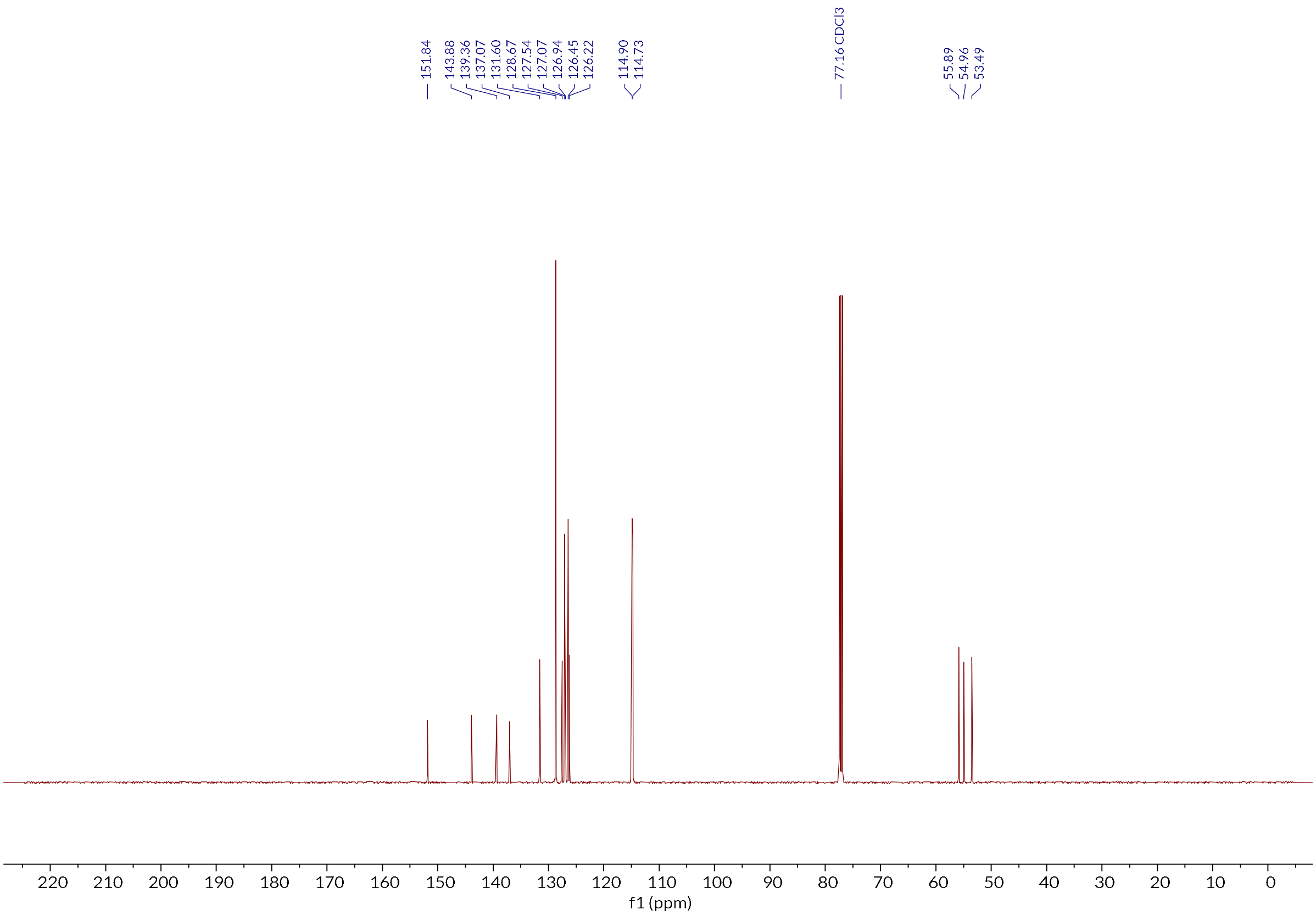

N-Cinnamyl-4-fluoroaniline (SI-23)

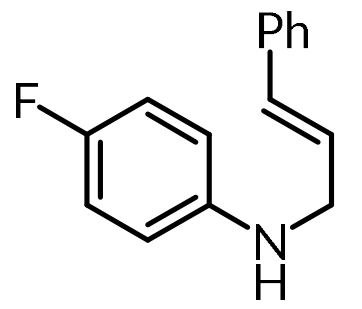

(SI-23)

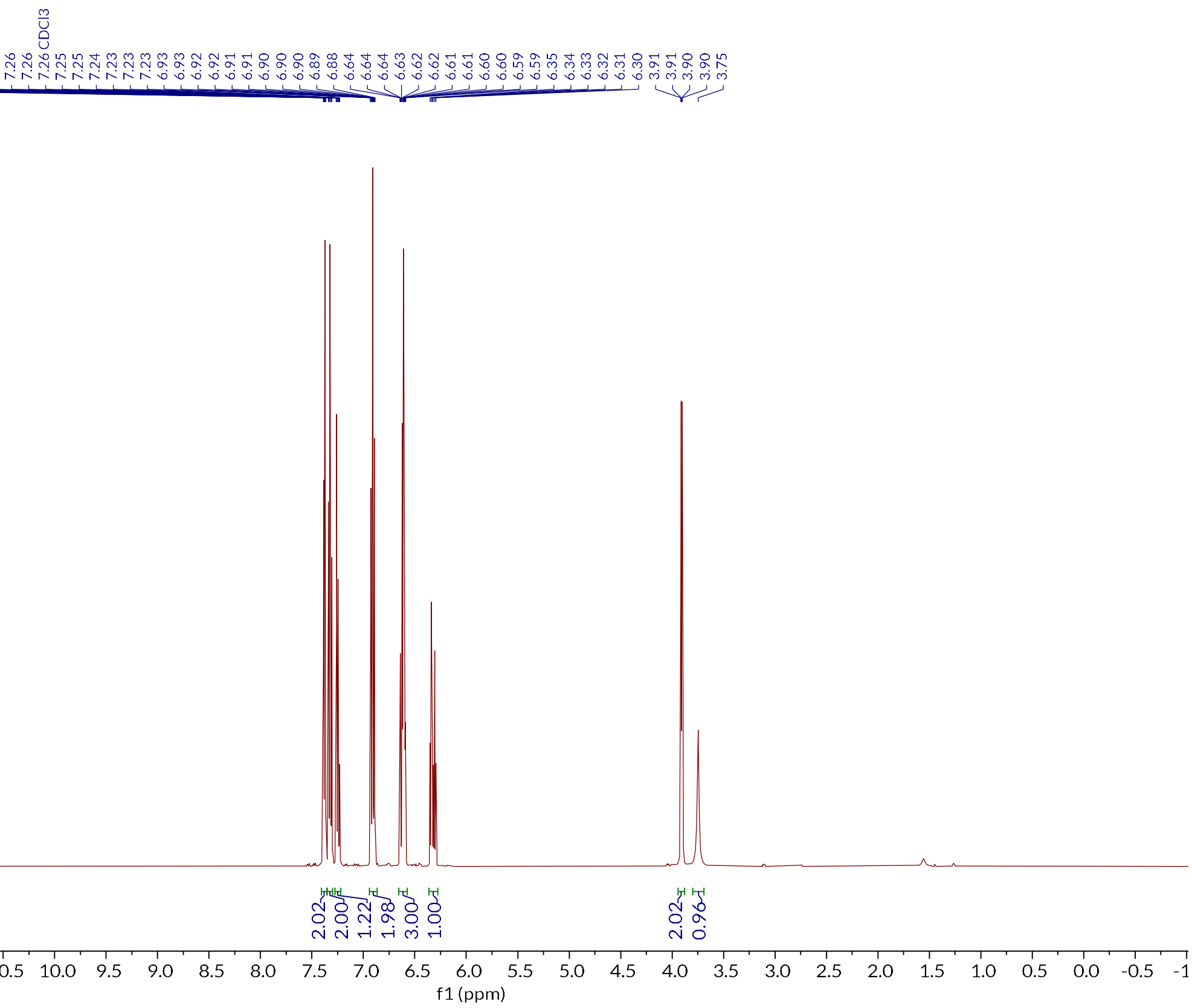

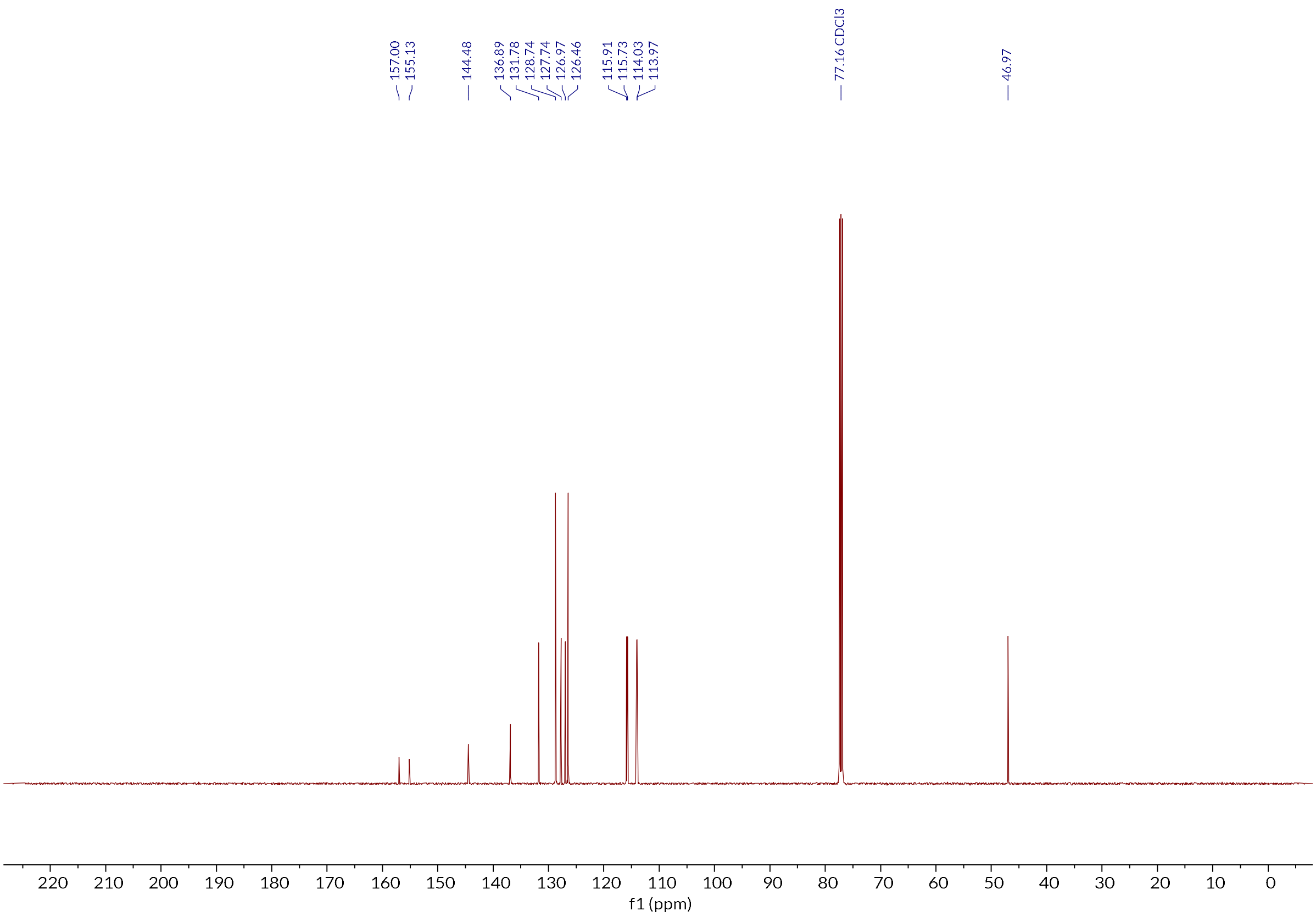

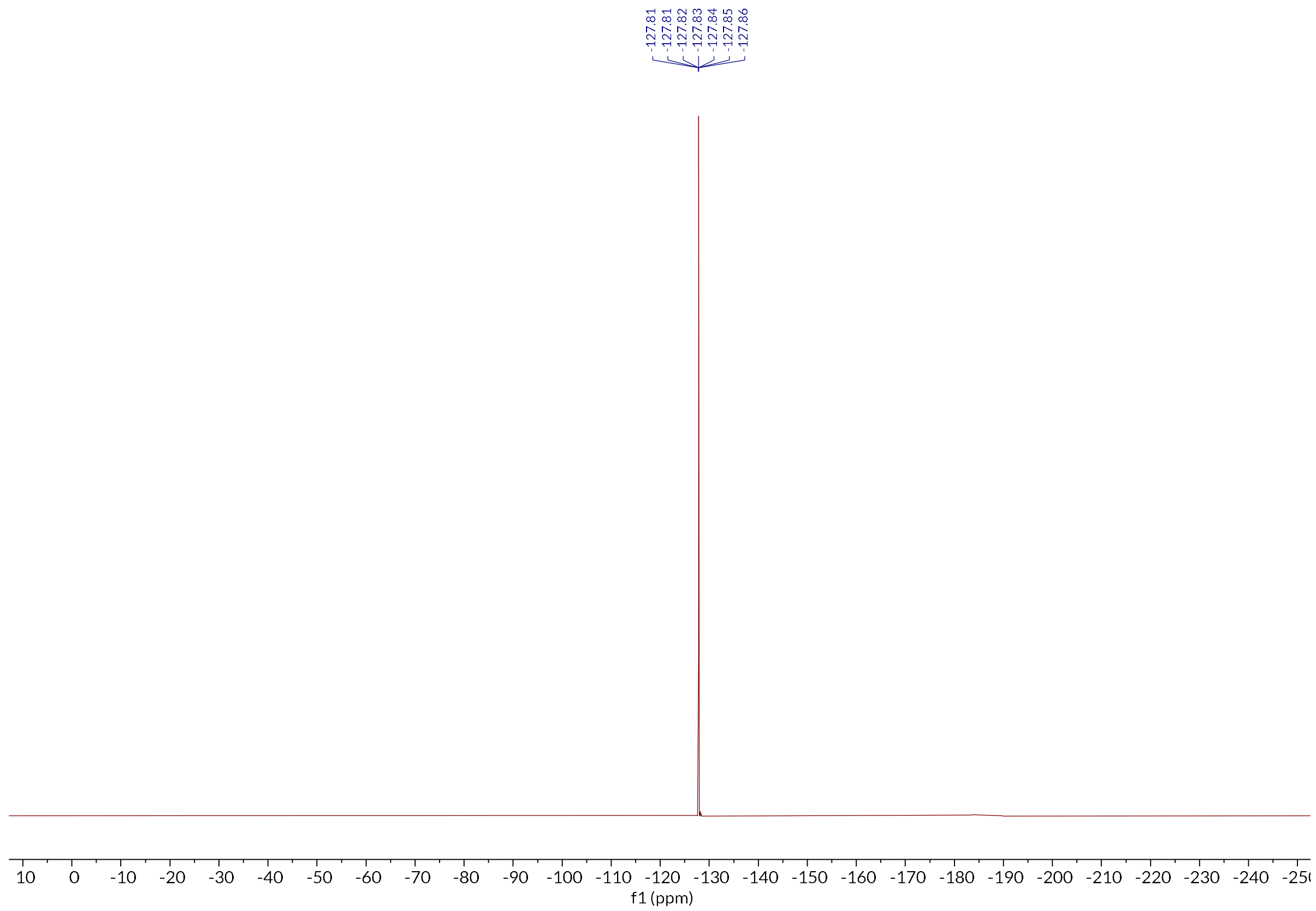
-127.81
-127.81
-127.82
-127.83
-127.84
-127.85
-127.86
10 0 -10 -20 -30 -40 -50 -60 -70 -80 -90 -100 -110 -120 -130 -140 -150 -160 -170 -180 -190 -200 -210 -220 -230 -240
f1 (ppm)

N-Benzyl-*N*-cinnamyl-4-fluoroaniline (1be)

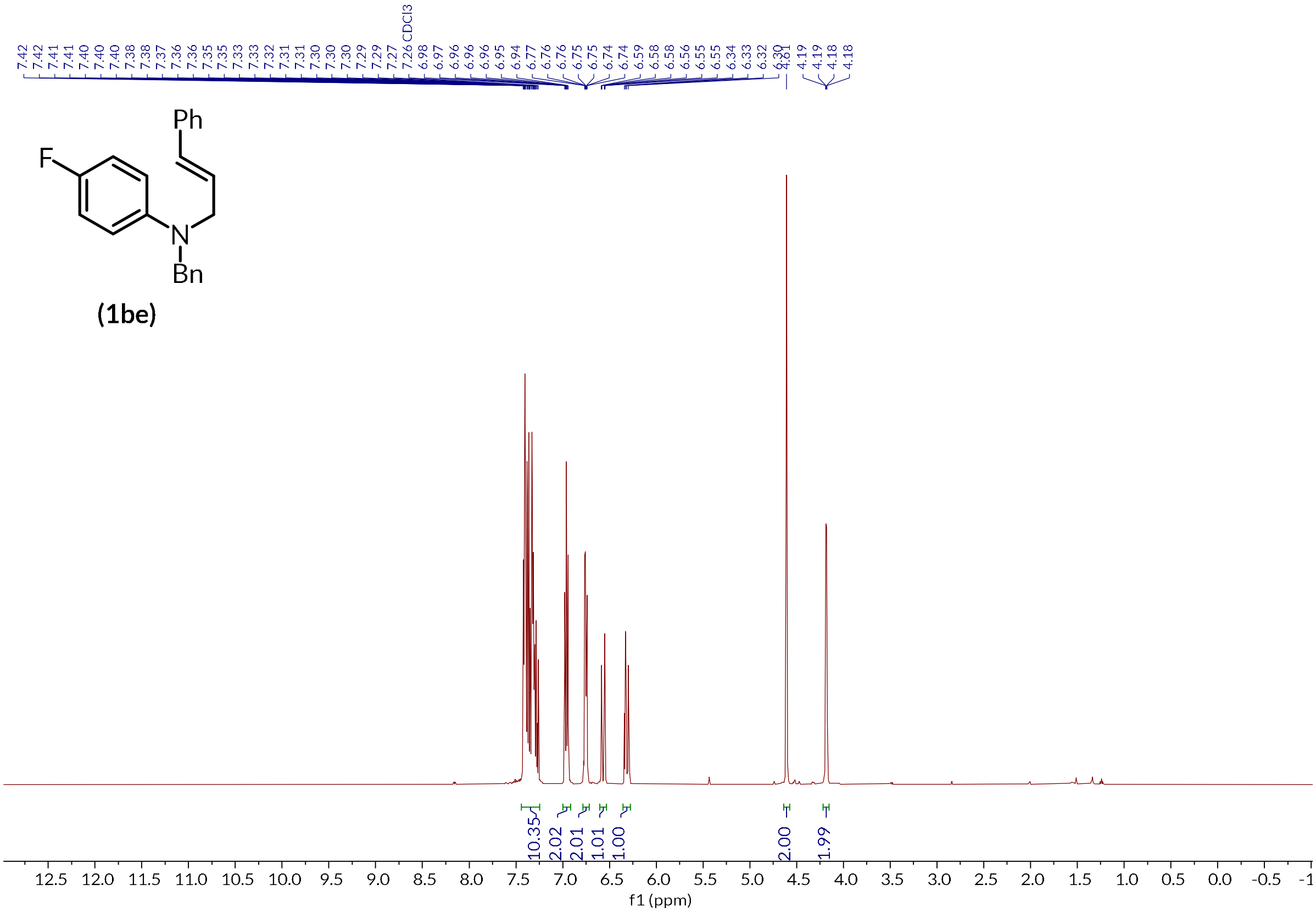

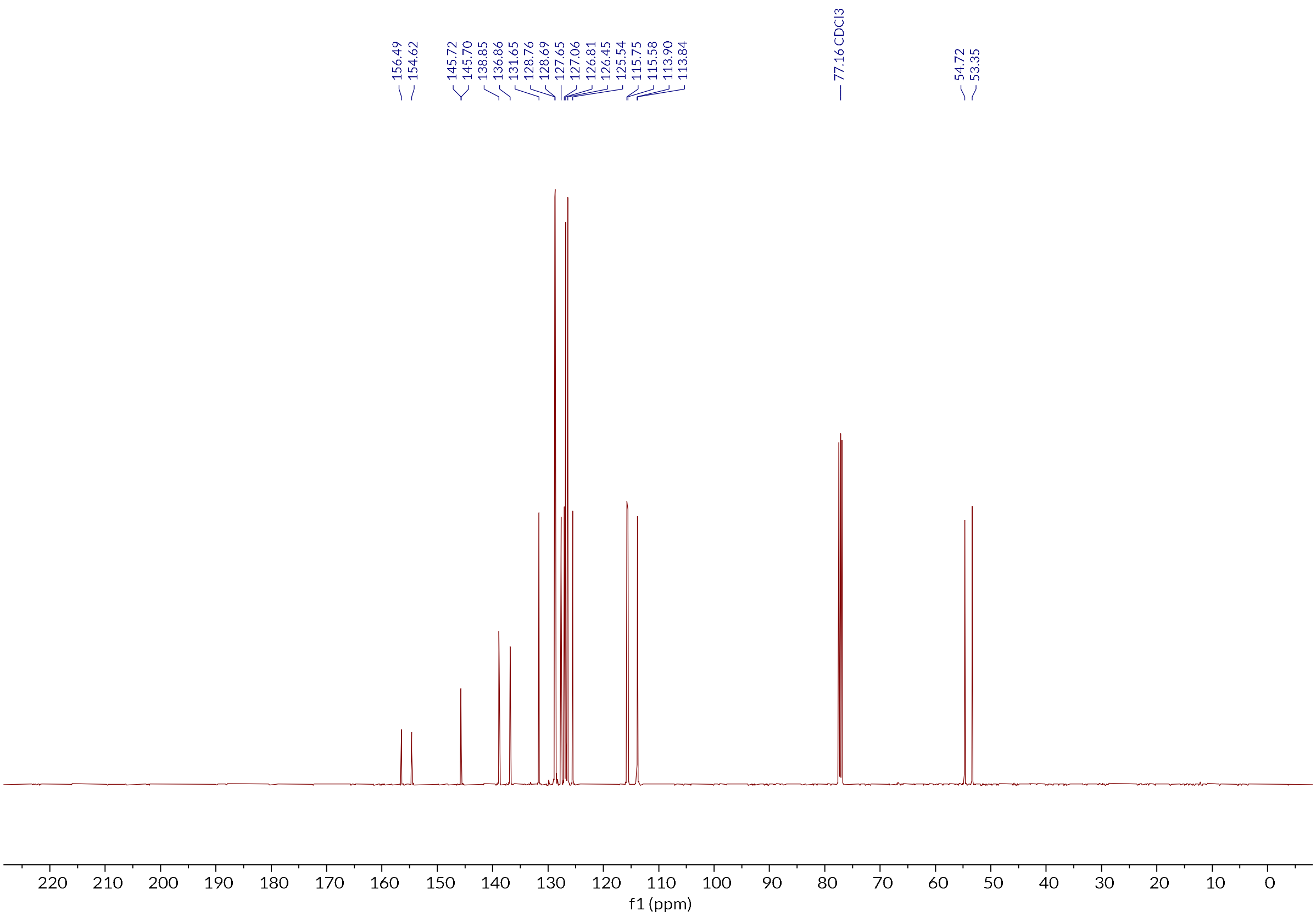

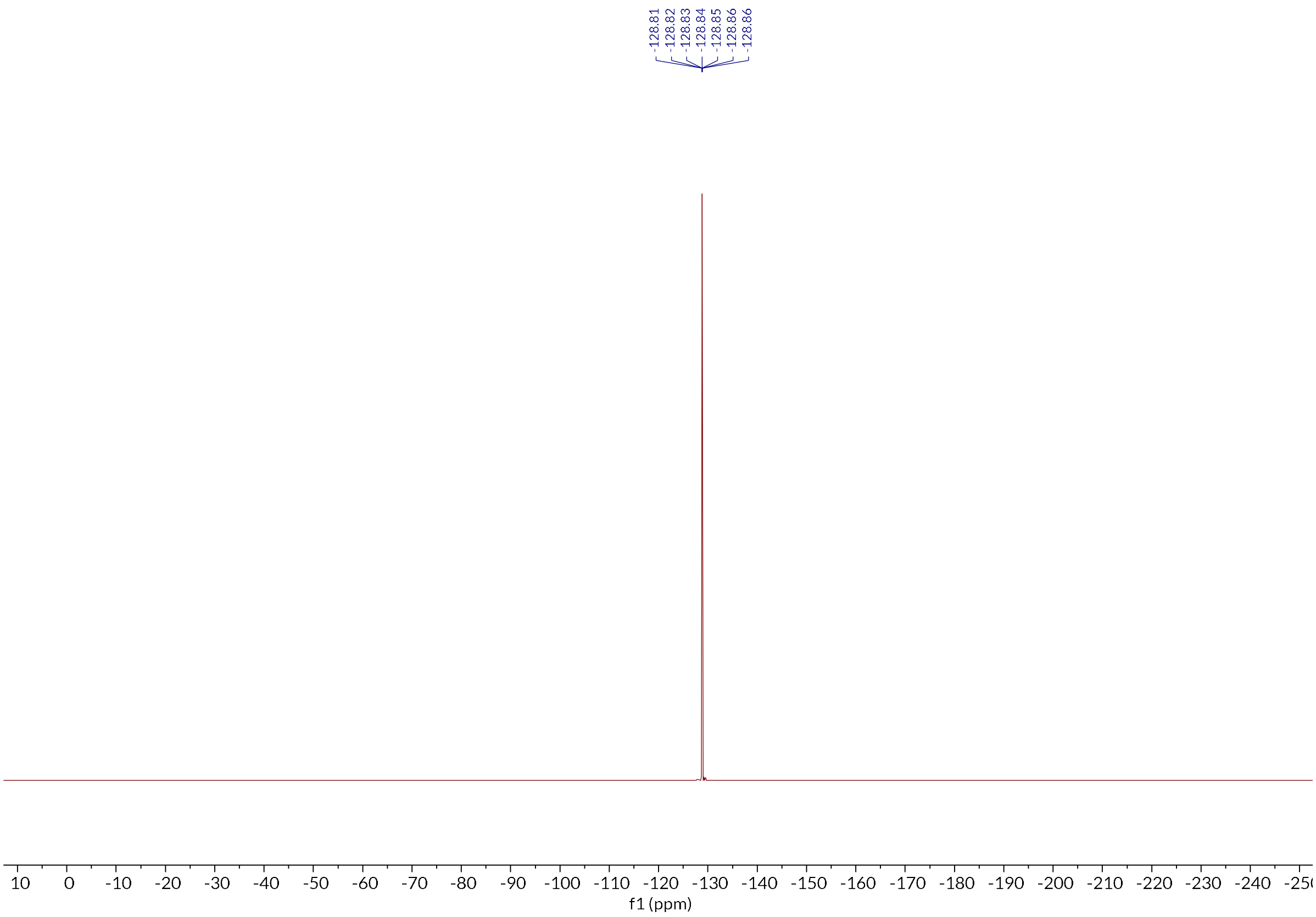
-128.81
-128.82
-128.83
-128.84
-128.85
-128.86
-128.86
10 0 -10 -20 -30 -40 -50 -60 -70 -80 -90 -100 -110 -120 -130 -140 -150 -160 -170 -180 -190 -200 -210 -220 -230 -240
f1 (ppm)

N-Cinnamyl-4-(trifluoromethyl)aniline (SI-24)

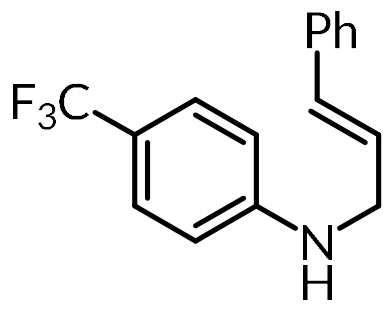

(SI-24)

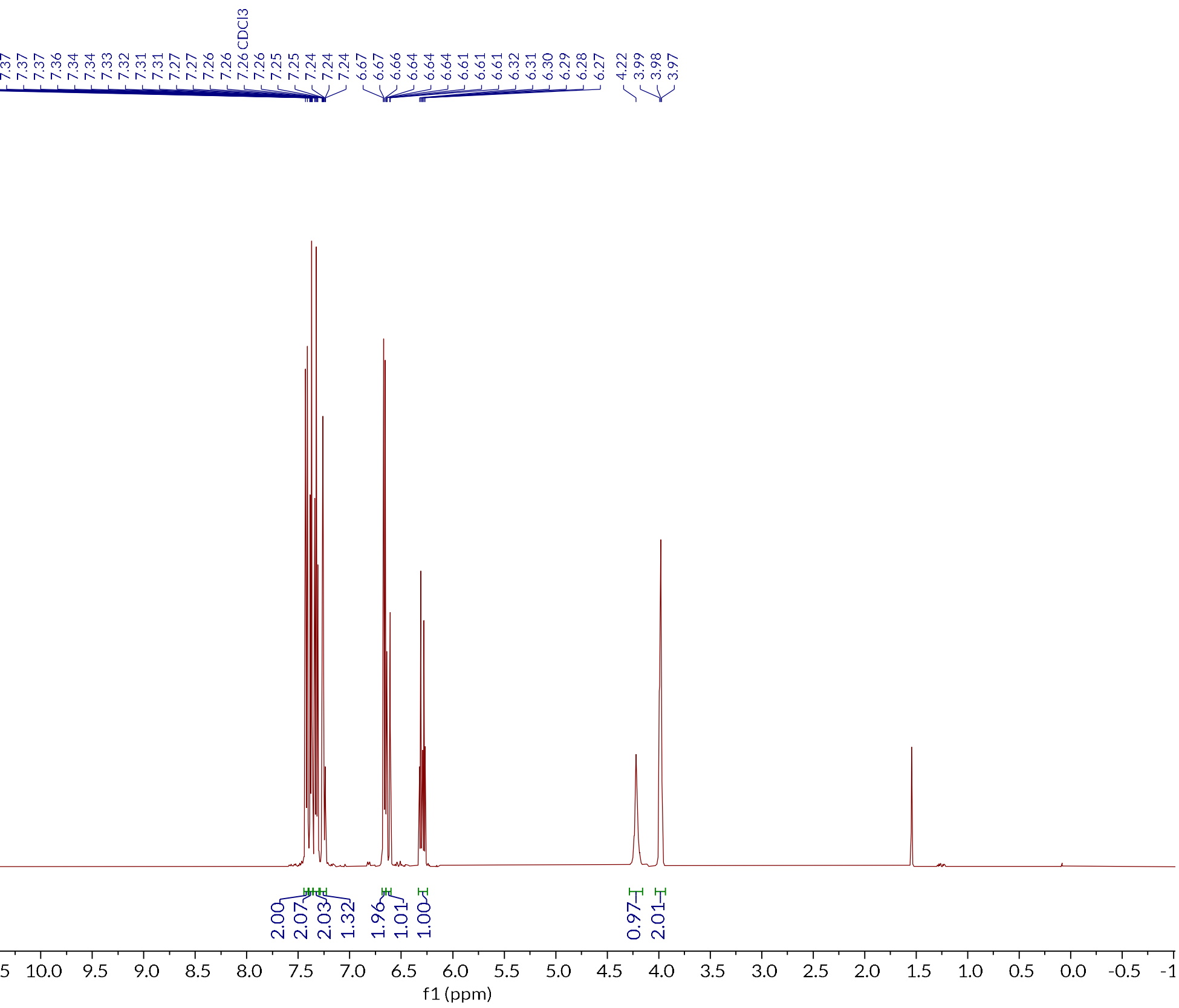

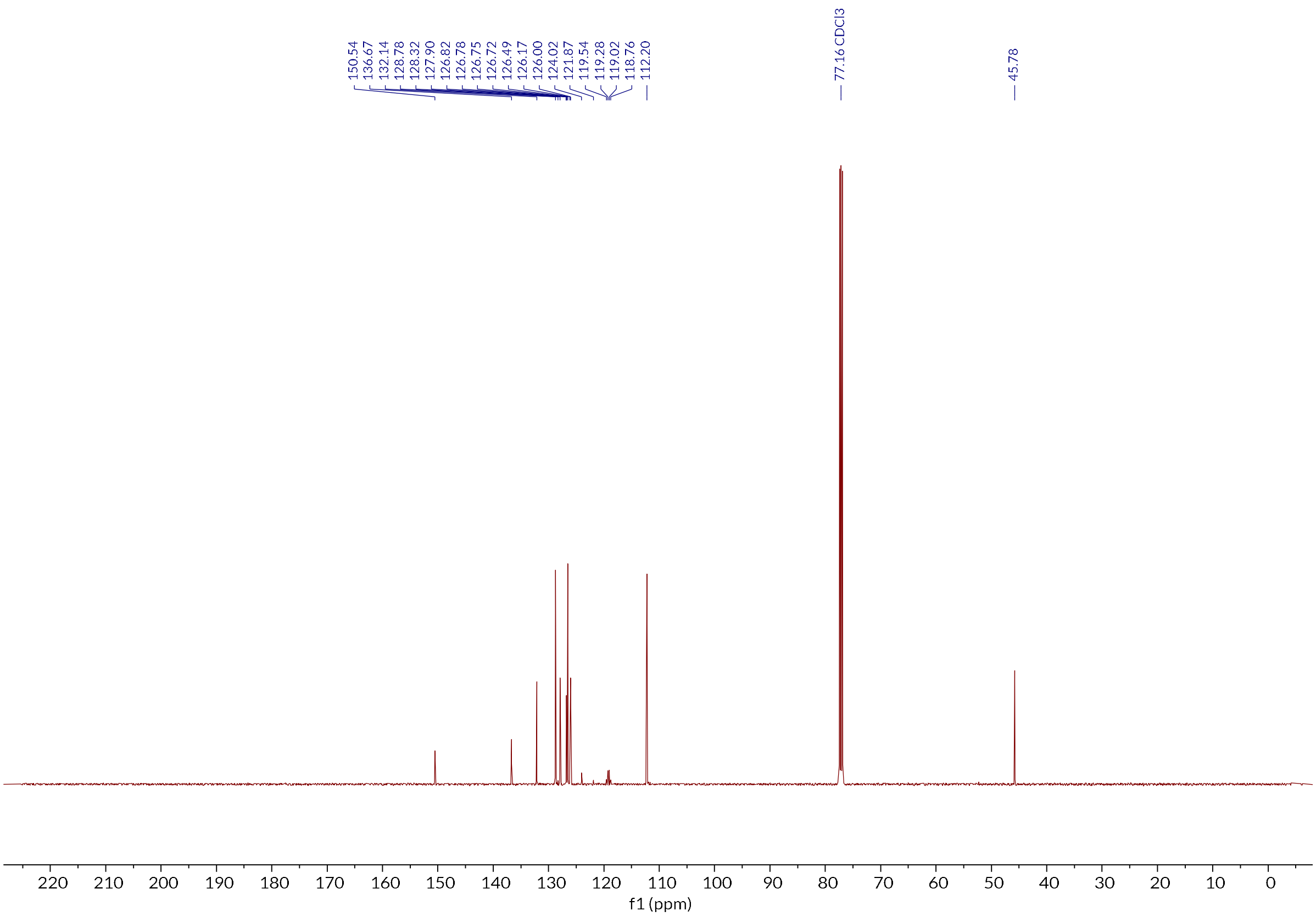

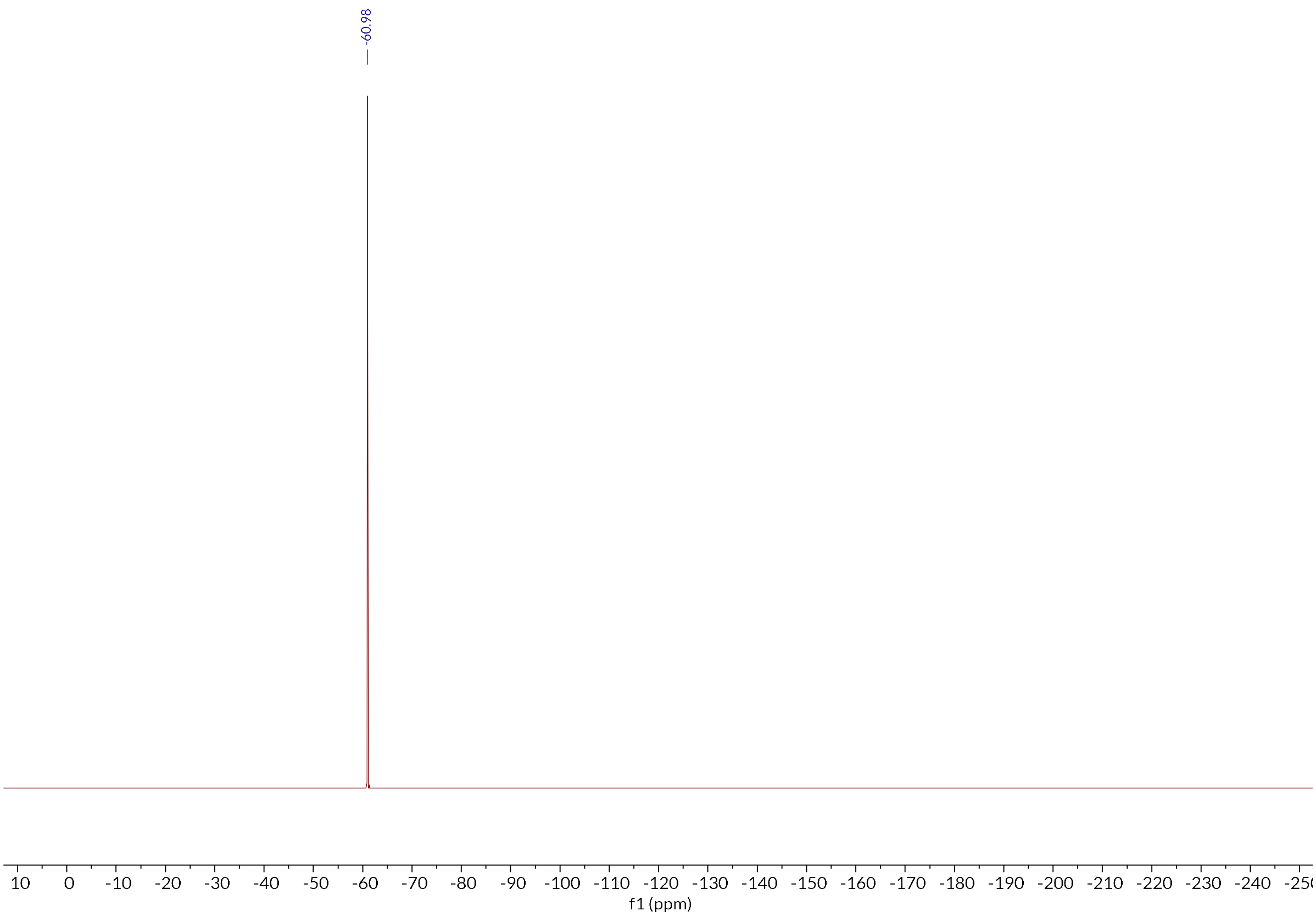
-60.98
10
0
-10
-20
-30
-40
-50
-60
-70
-80
-90
-100
-110
-120
-130
-140
-150
-160
-170
-180
-190
-200
-210
-220
-230
-240
f1 (ppm)

N-Benzyl-*N*-cinnamyl-4-(trifluoromethyl)aniline (1bf)

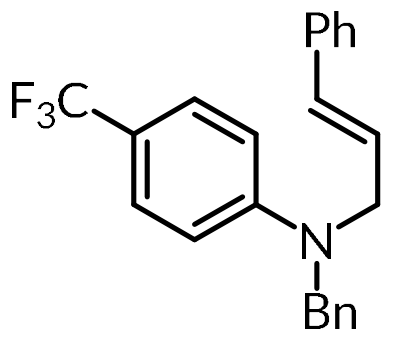

(1bf)

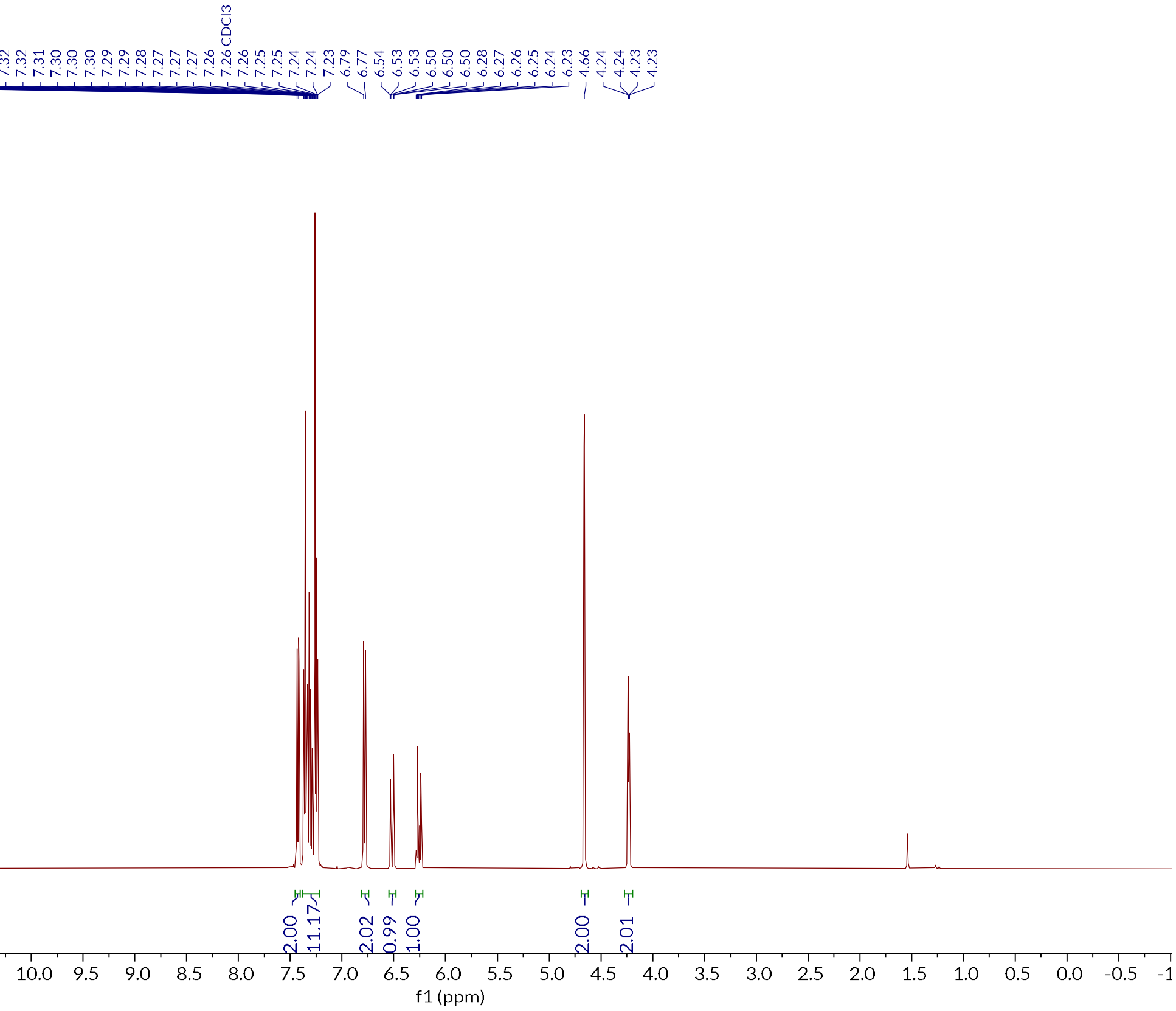

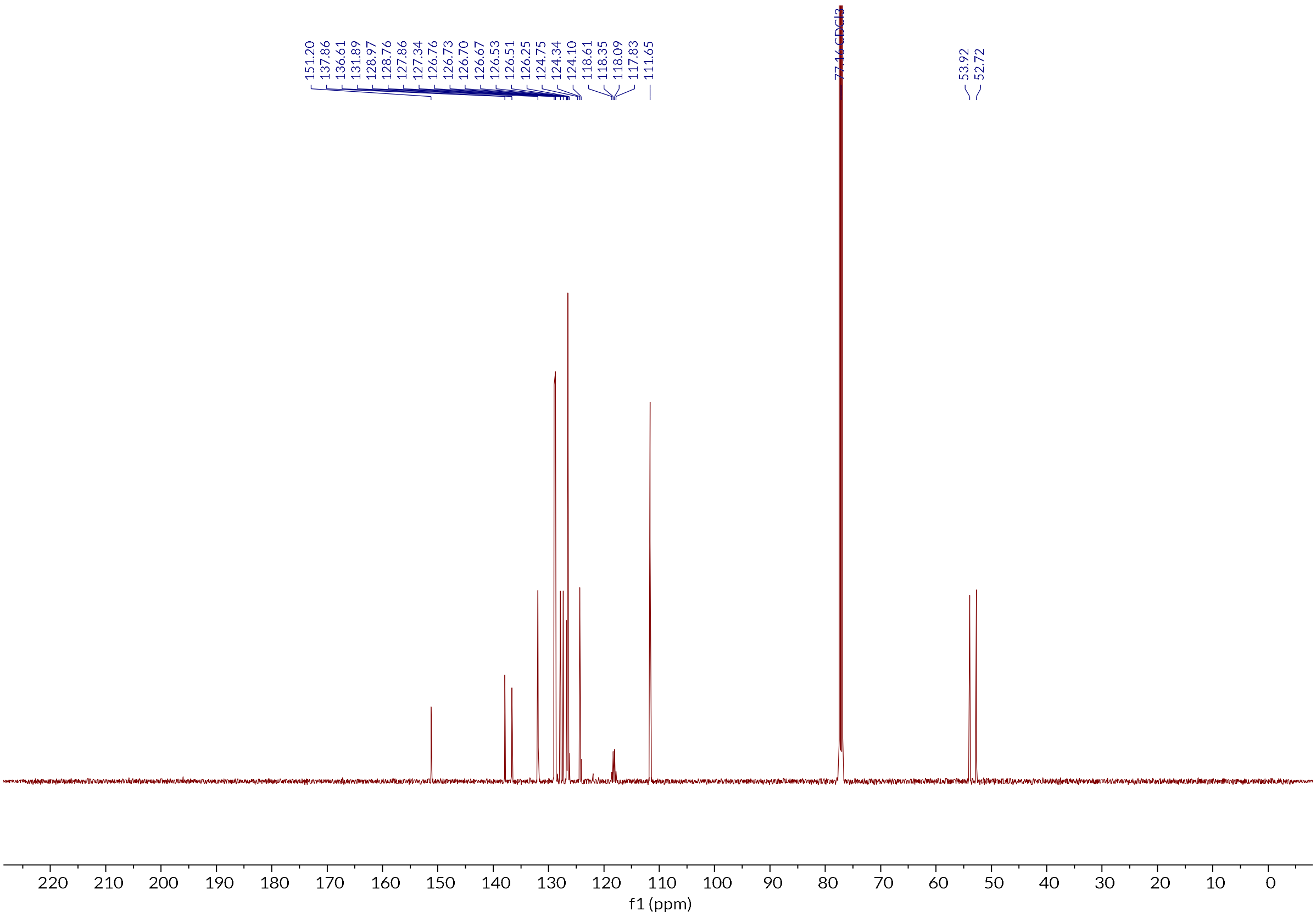

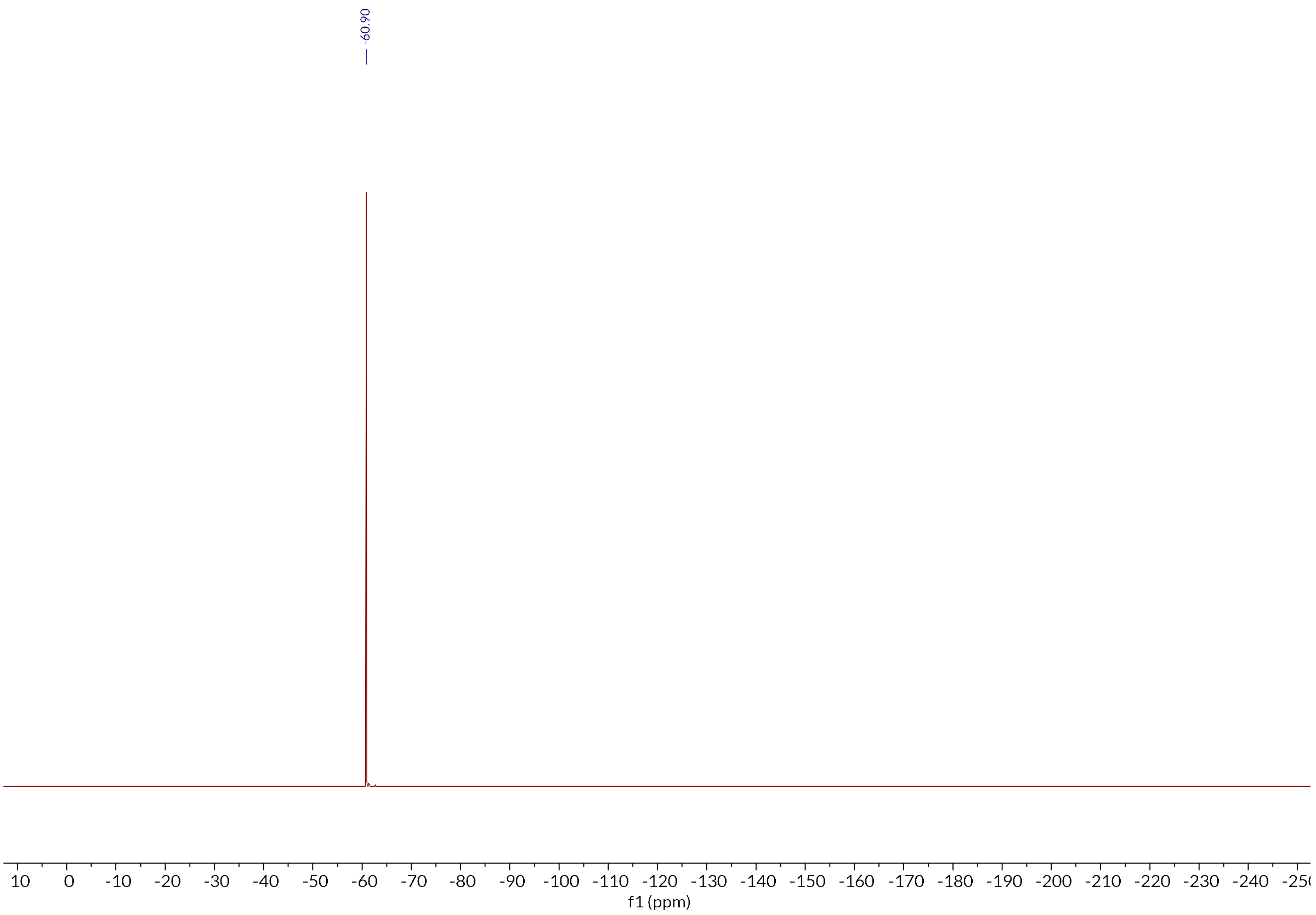
-60.90
10
0
-10
-20
-30
-40
-50
-60
-70
-80
-90
-100
-110
-120
-130
-140
-150
-160
-170
-180
-190
-200
-210
-220
-230
-240
f1 (ppm)

4-(Cinnamylamino)benzonitrile (SI-25)

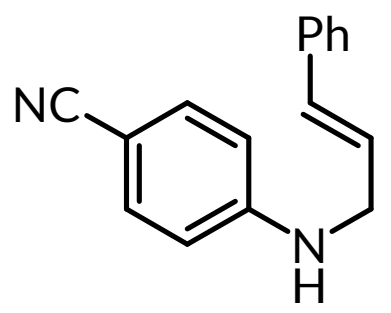

(SI-25)

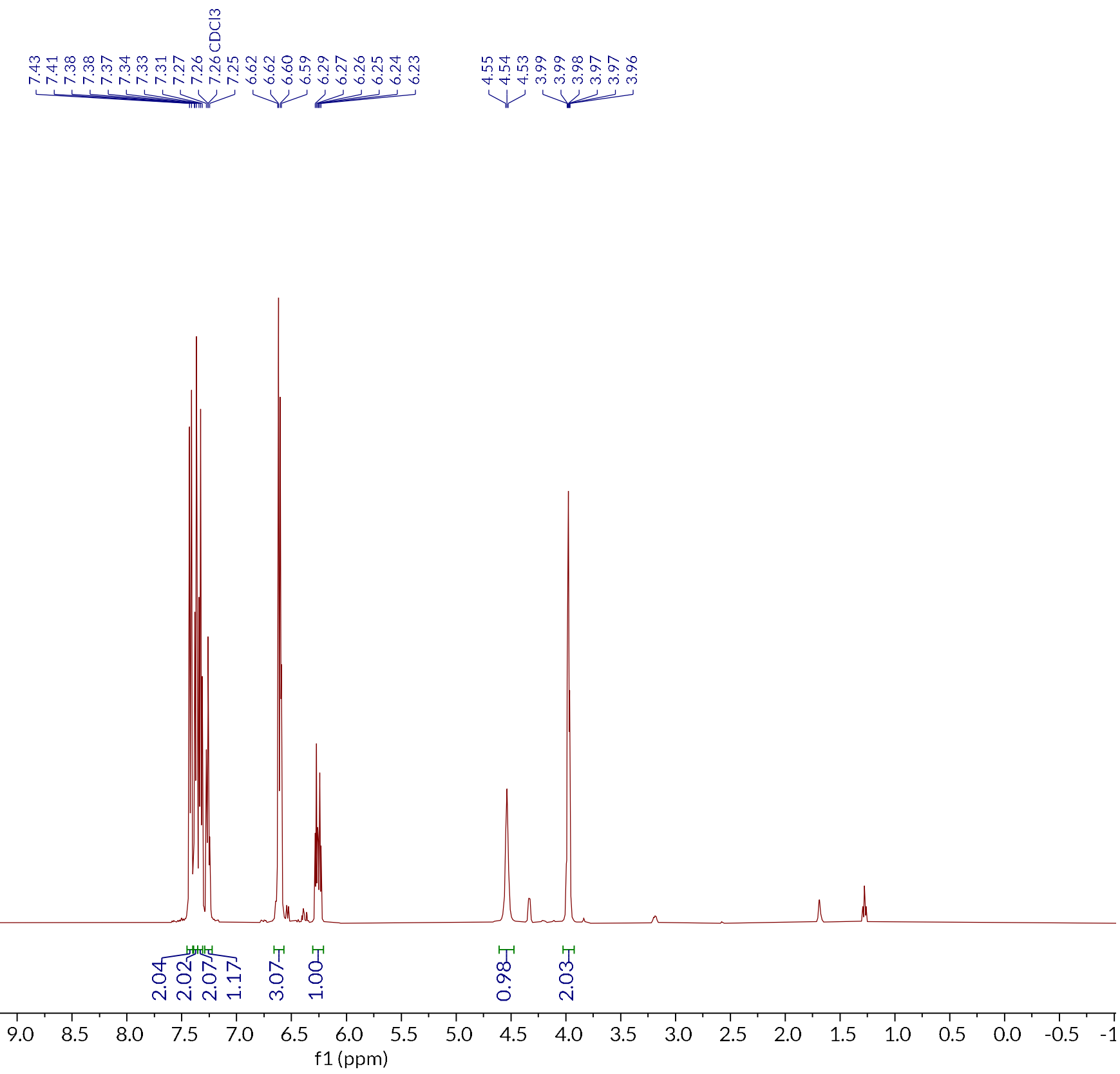

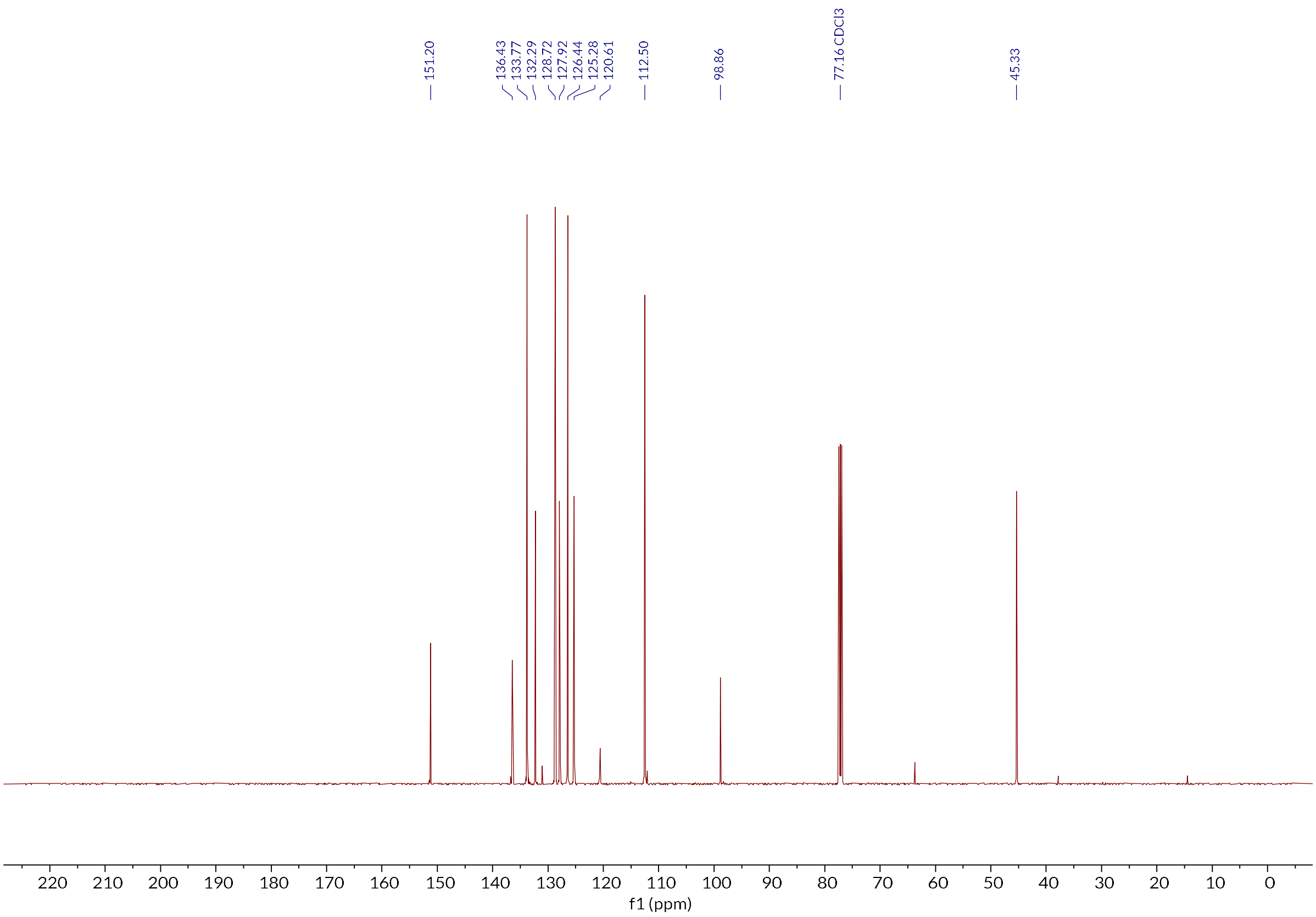

4-(Benzyl(cinnamyl)amino)benzonitrile (1bg)

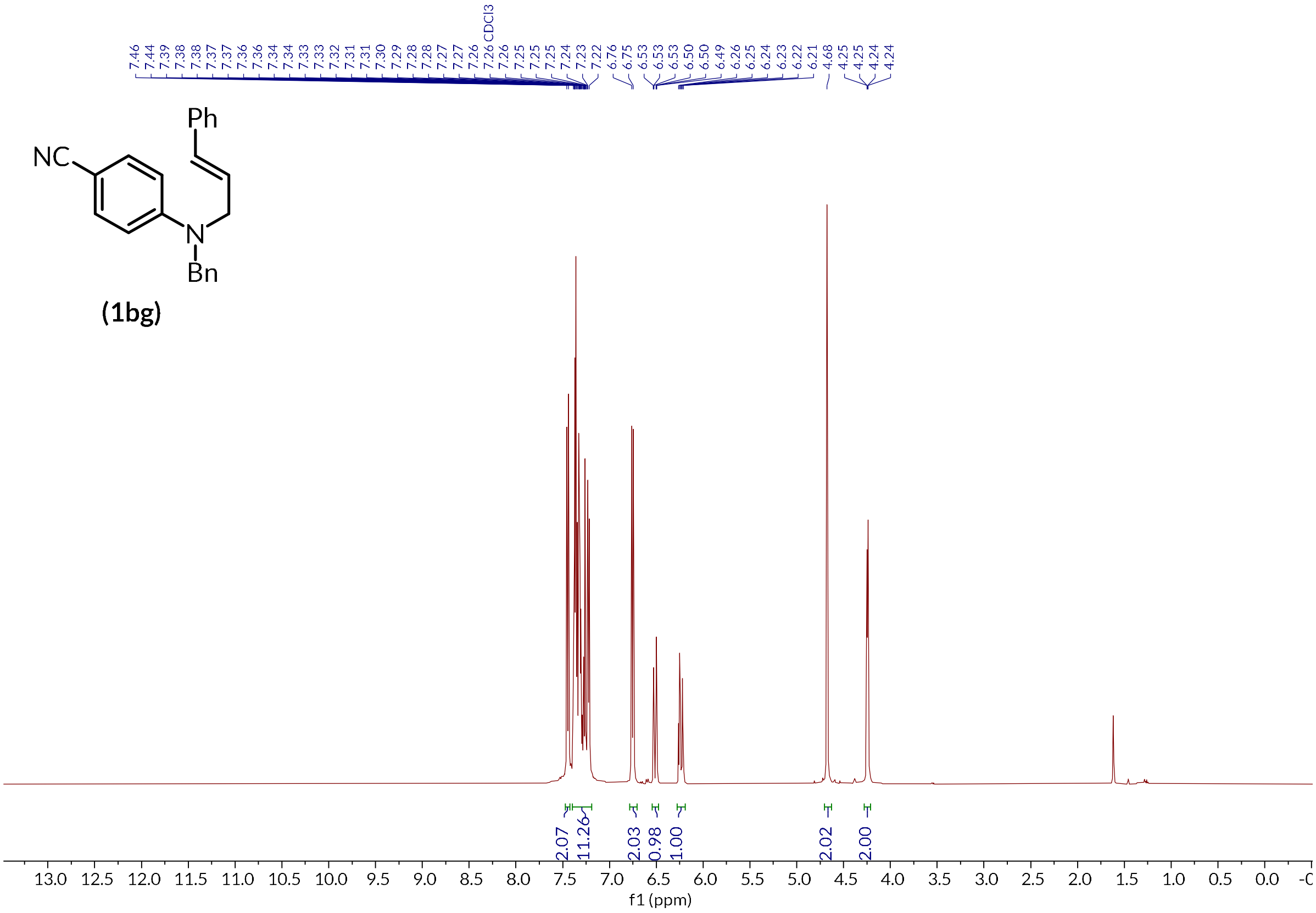

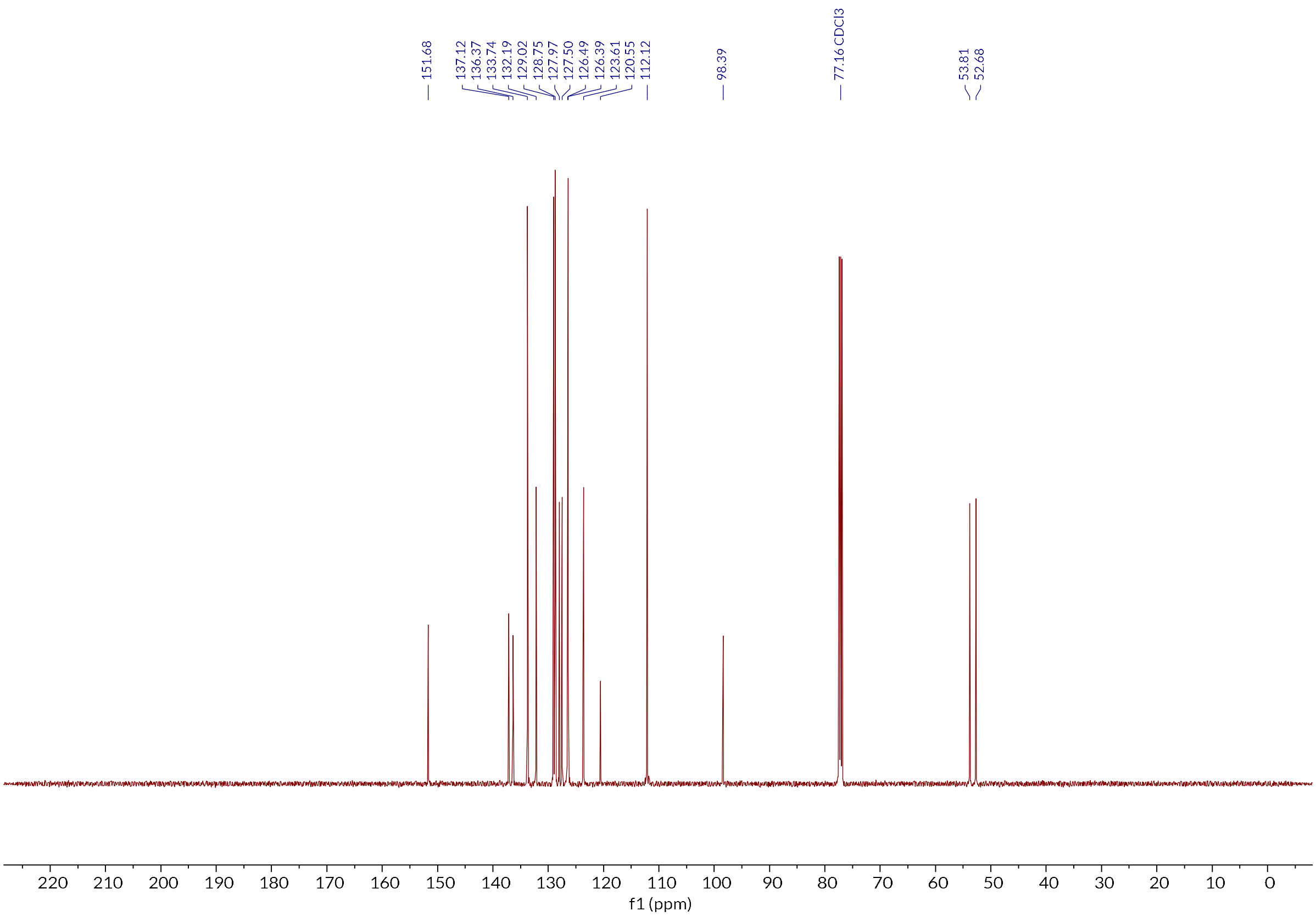

1-(4-(Cinnamylamino)phenyl)ethan-1-one (SI-26)

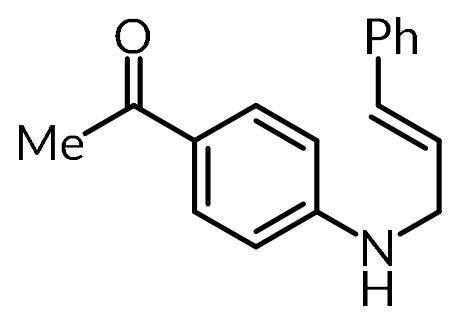

(SI-26)

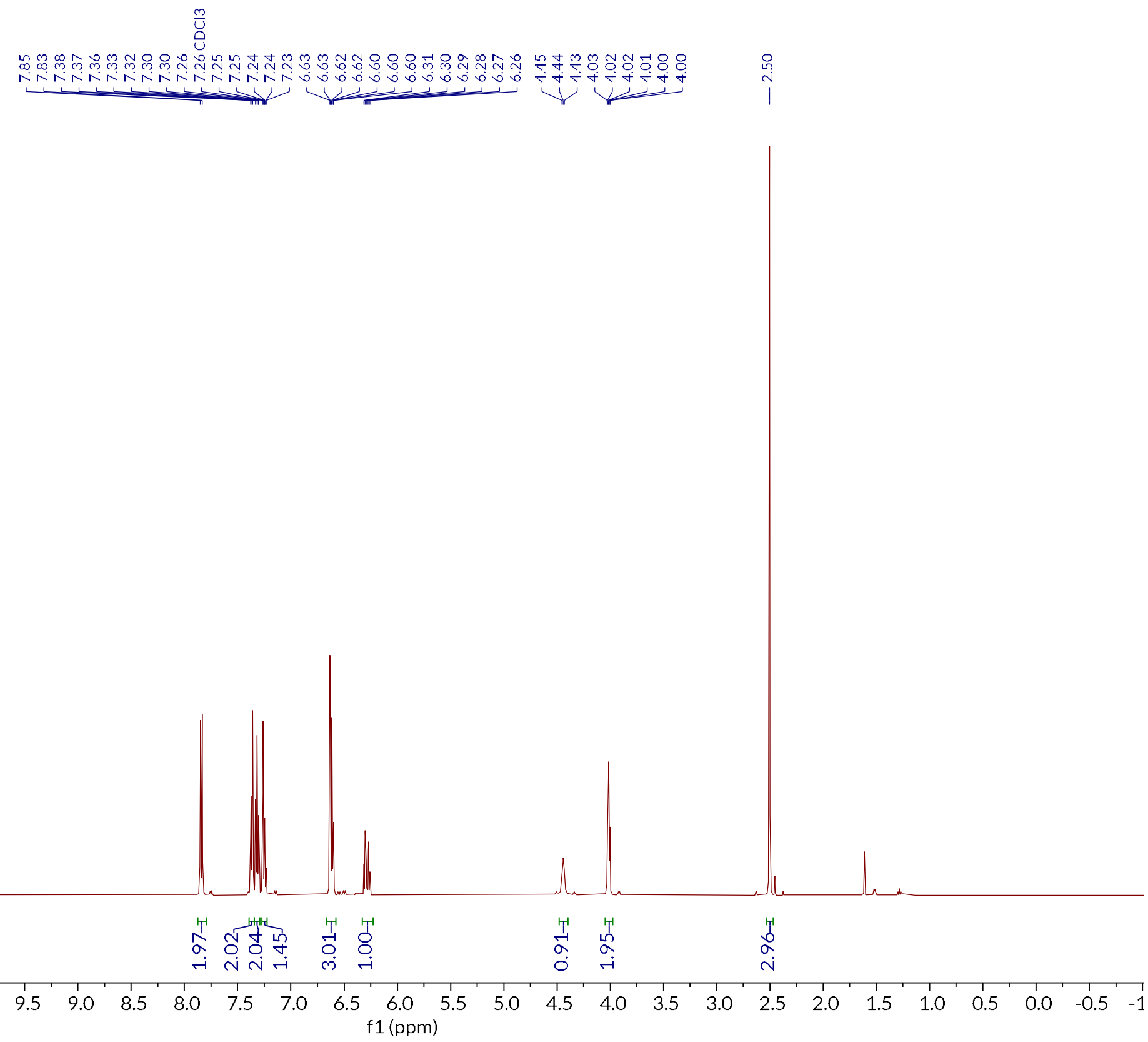

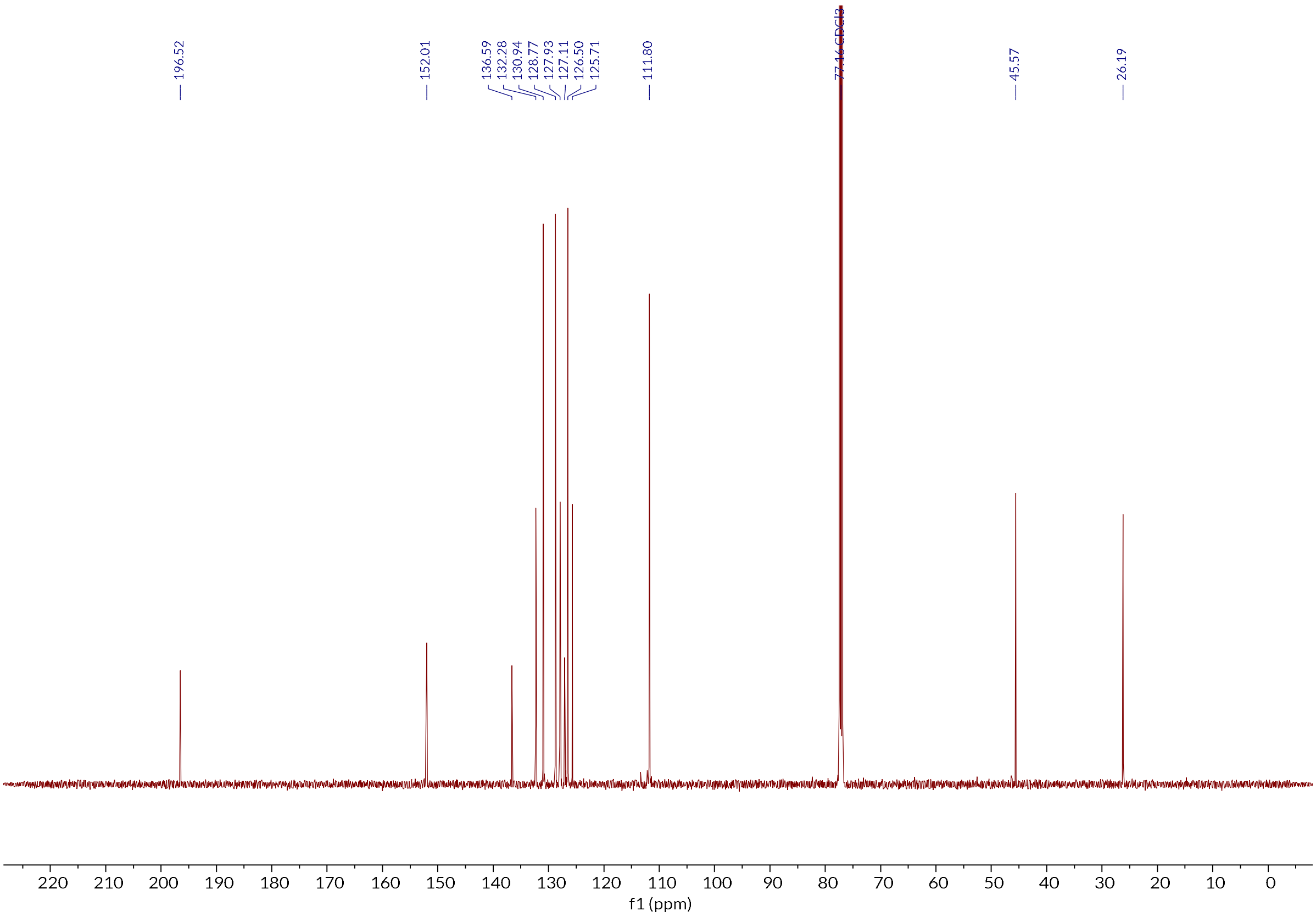

1-(4-(Benzyl(cinnamyl)amino)phenyl)ethan-1-one (1bh)

(1bh)

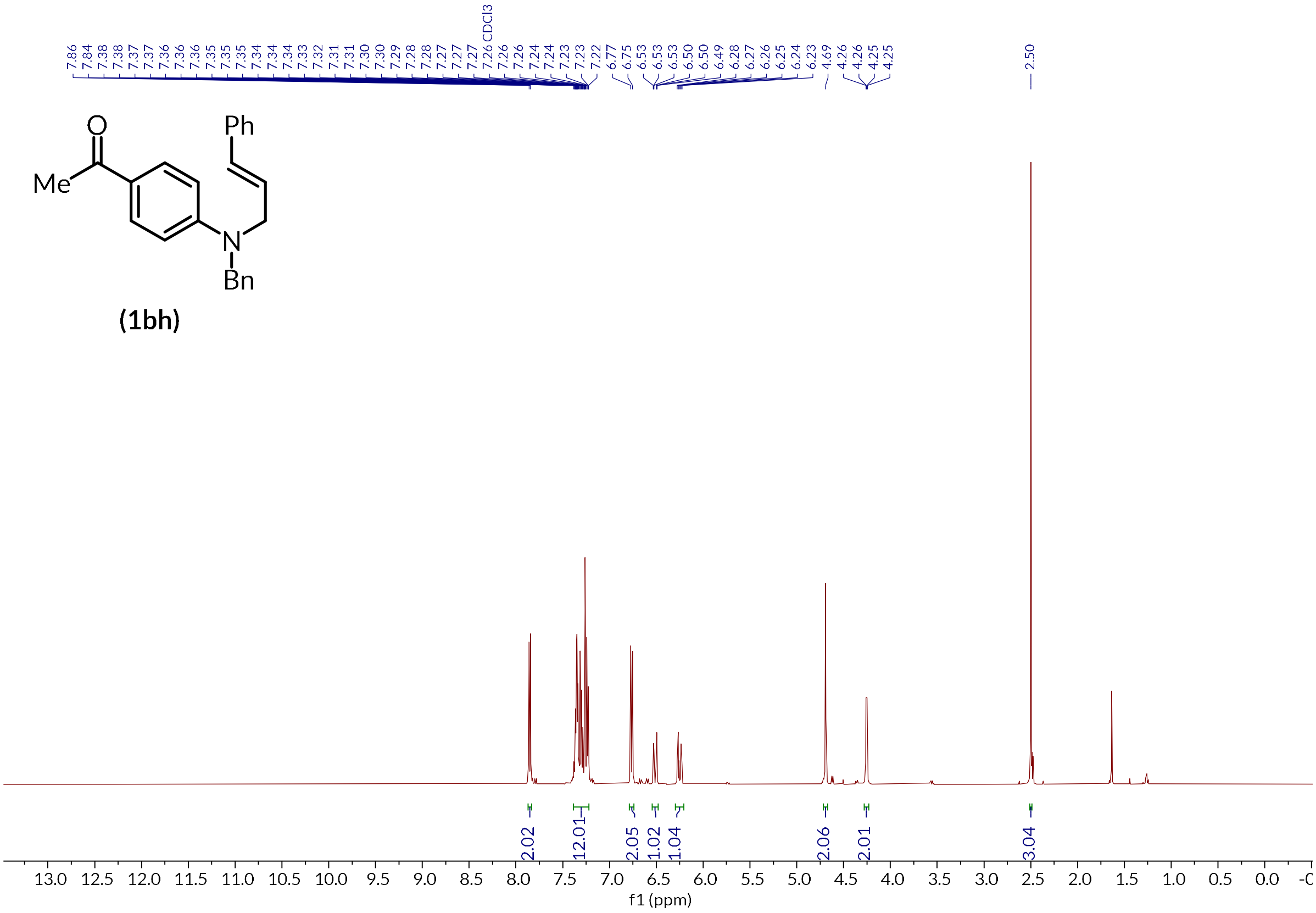

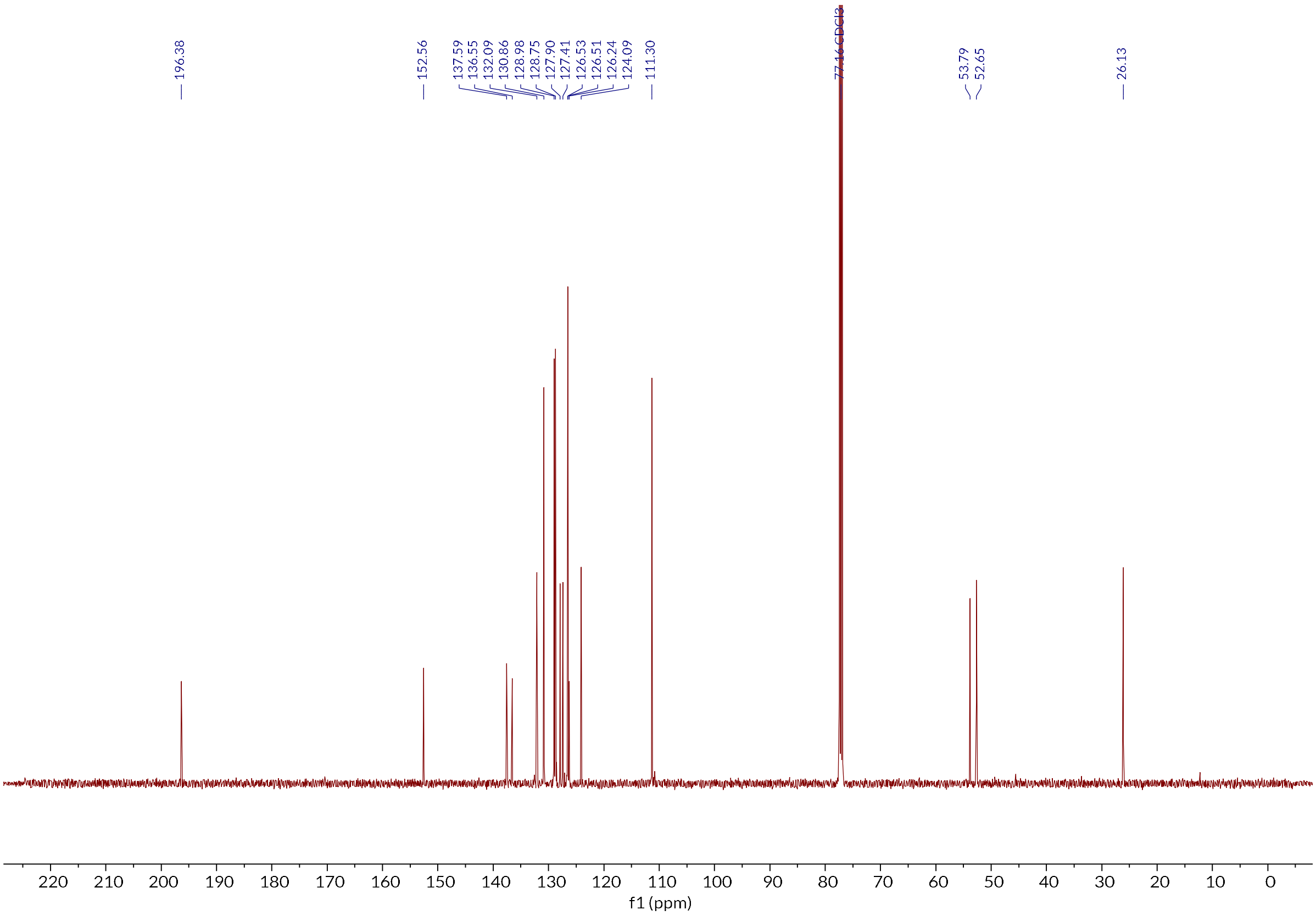

2-(4-(Cinnamylamino)phenyl)acetonitrile (SI-27)

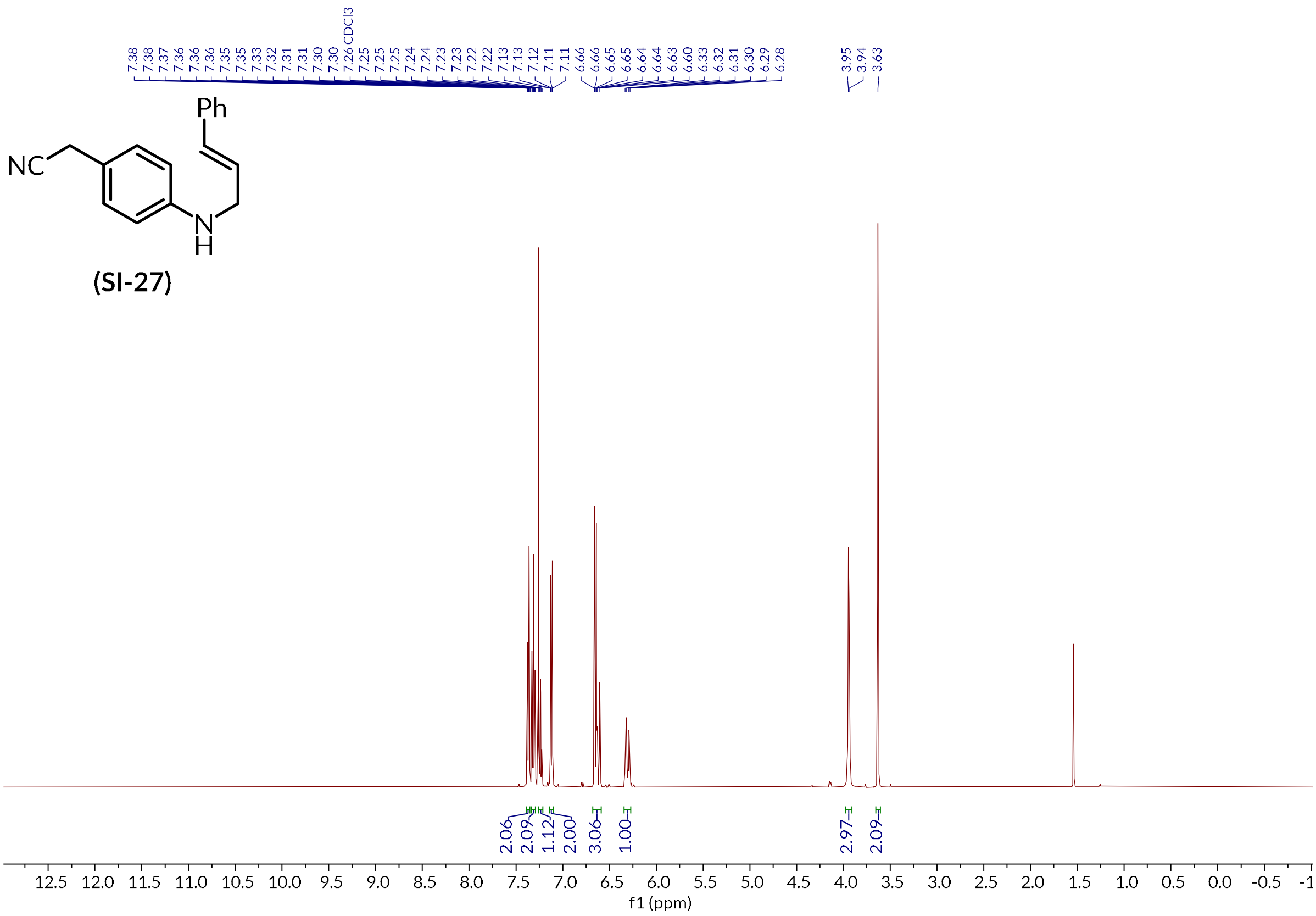

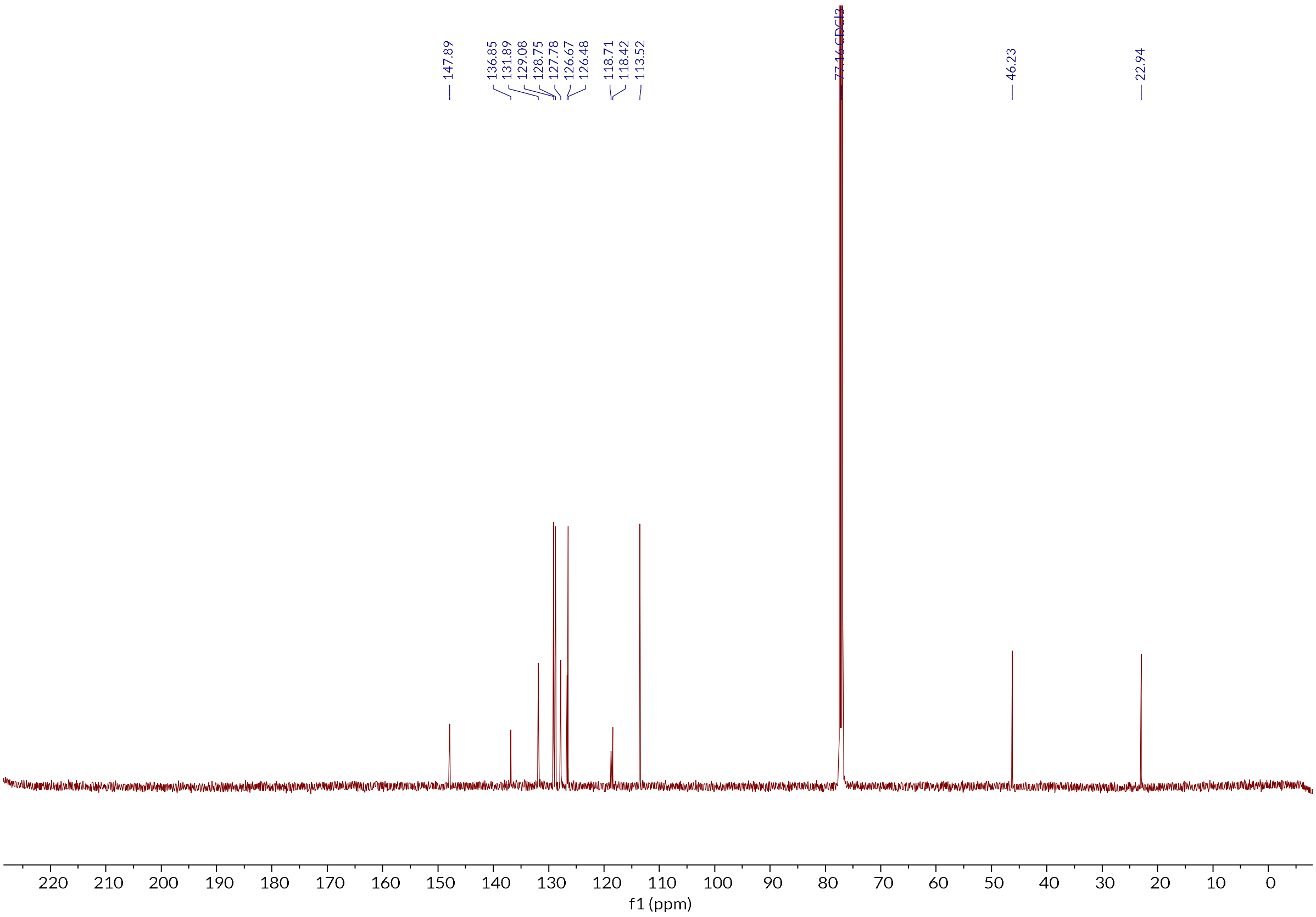

2-(4-(Benzyl(cinnamyl)amino)phenyl)acetonitrile (1bi)

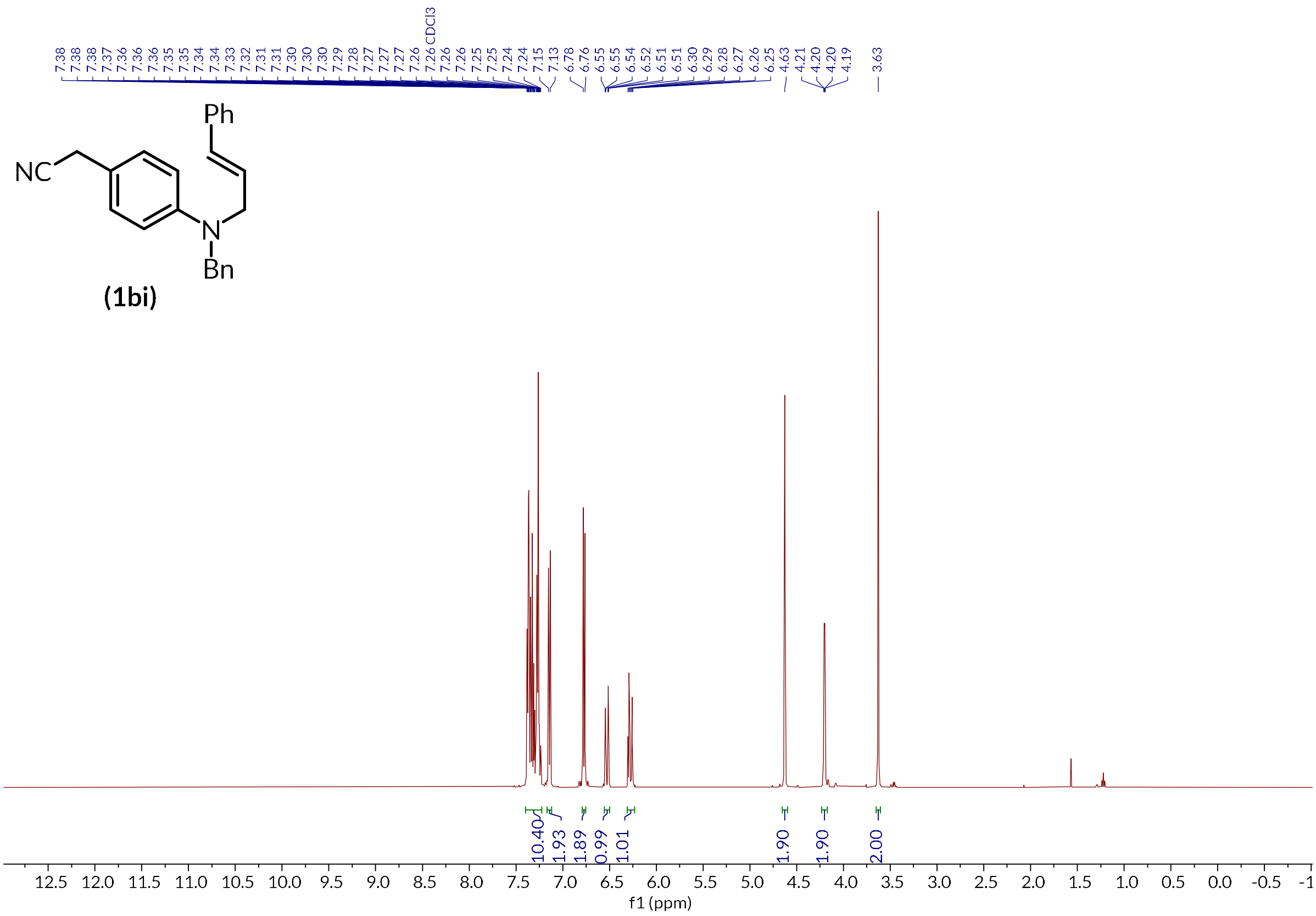

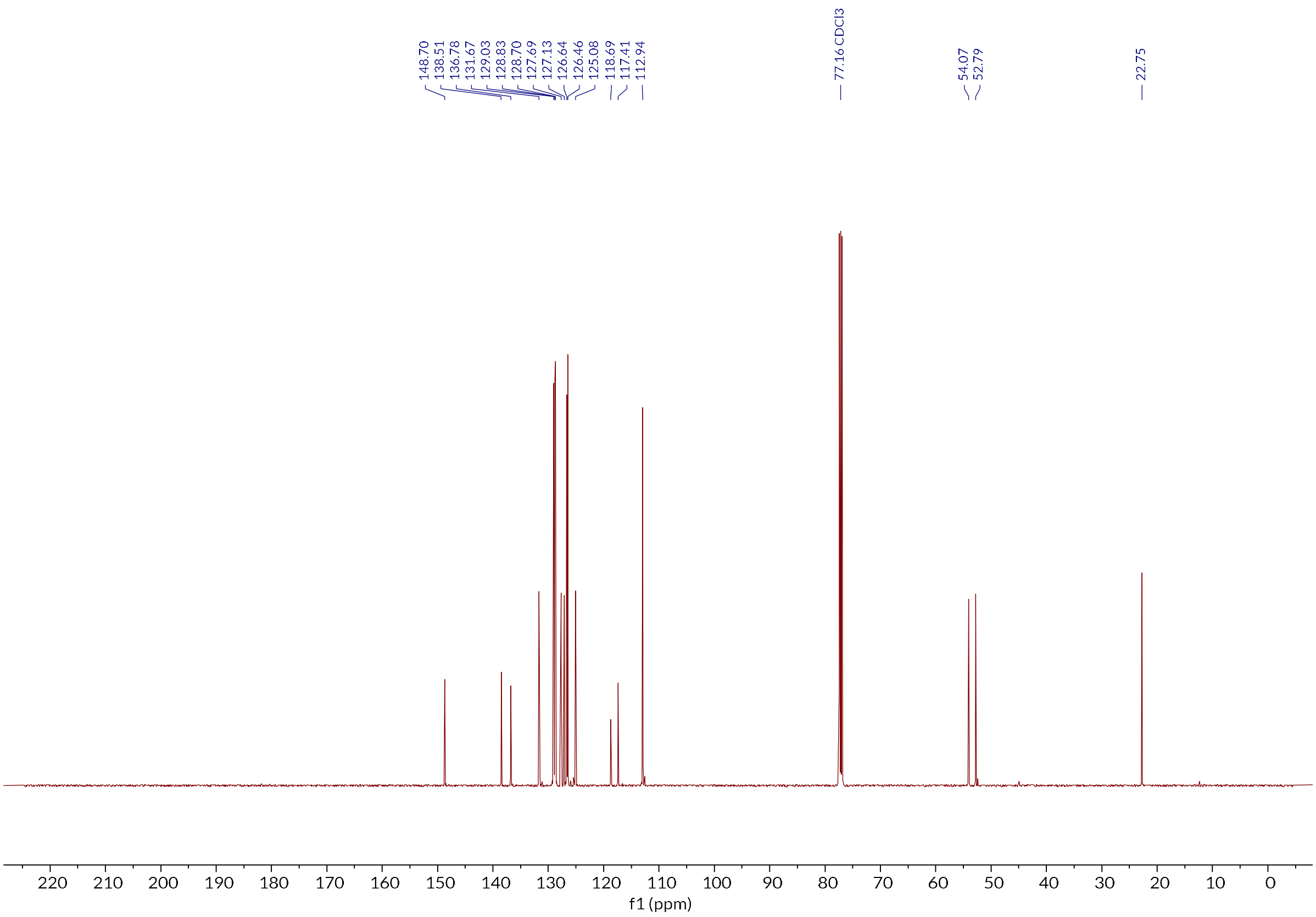

N-Cinnamyl-3,4,5-trifluoroaniline (SI-28)

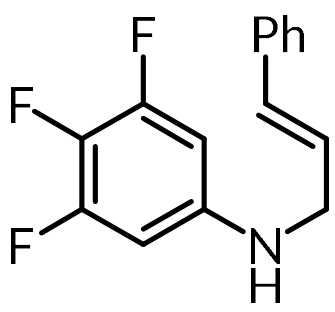

(SI-28)

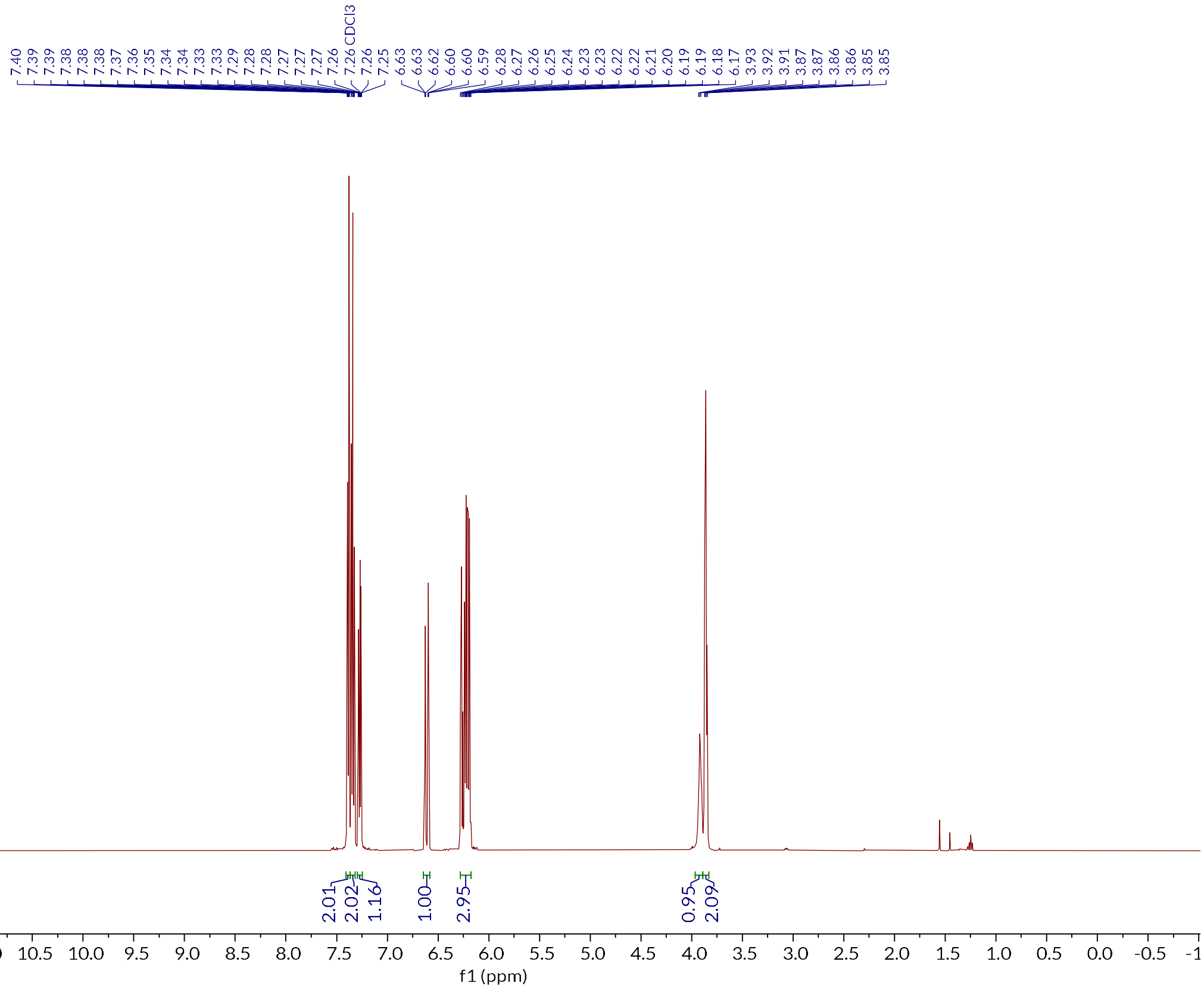

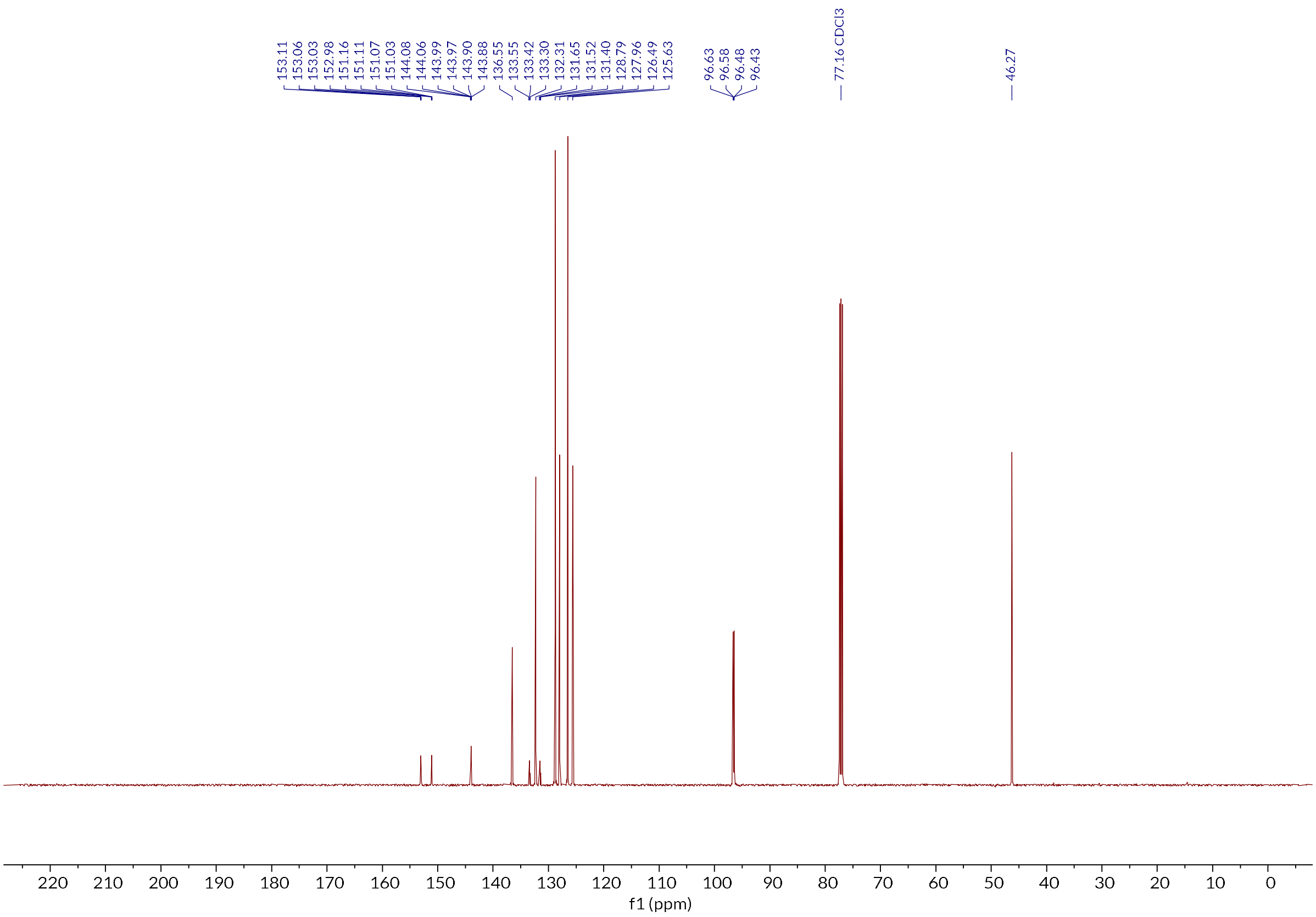

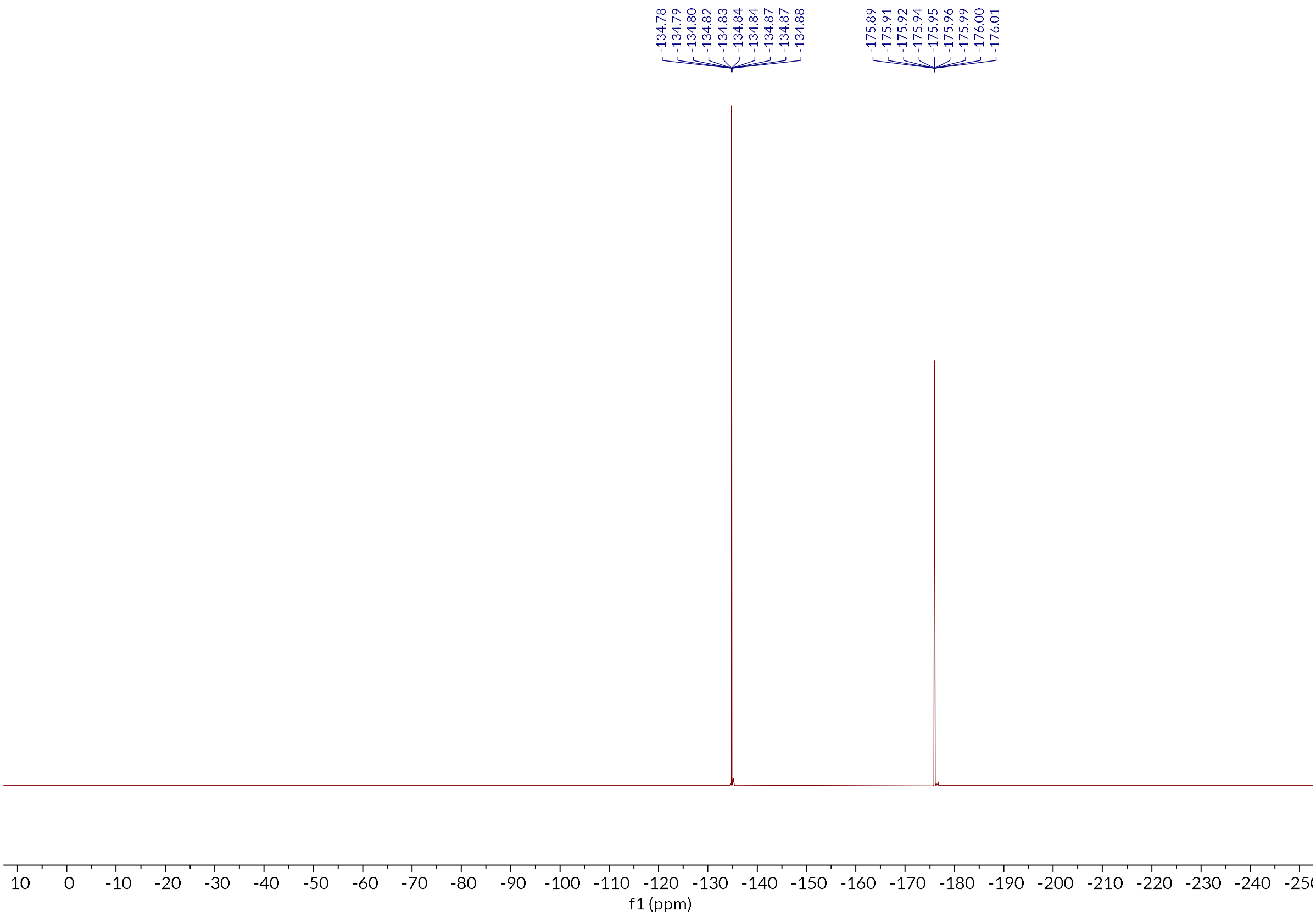
-134.78
-134.79
-134.80
-134.82
-134.83
-134.84
-134.84
-134.87
-134.87
-134.88
-175.89
-175.91
-175.92
-175.94
-175.95
-175.96
-175.99
-176.00
-176.01
10 0 -10 -20 -30 -40 -50 -60 -70 -80 -90 -100 -110 -120 -130 -140 -150 -160 -170 -180 -190 -200 -210 -220 -230 -240
f1 (ppm)

N-Benzyl-*N*-cinnamyl-3,4,5-trifluoroaniline (1bj)

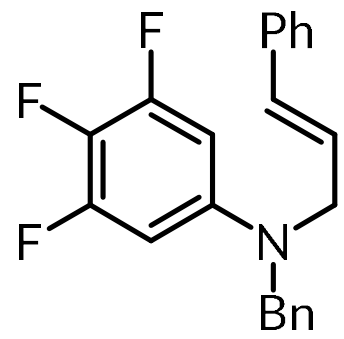

(1bj)

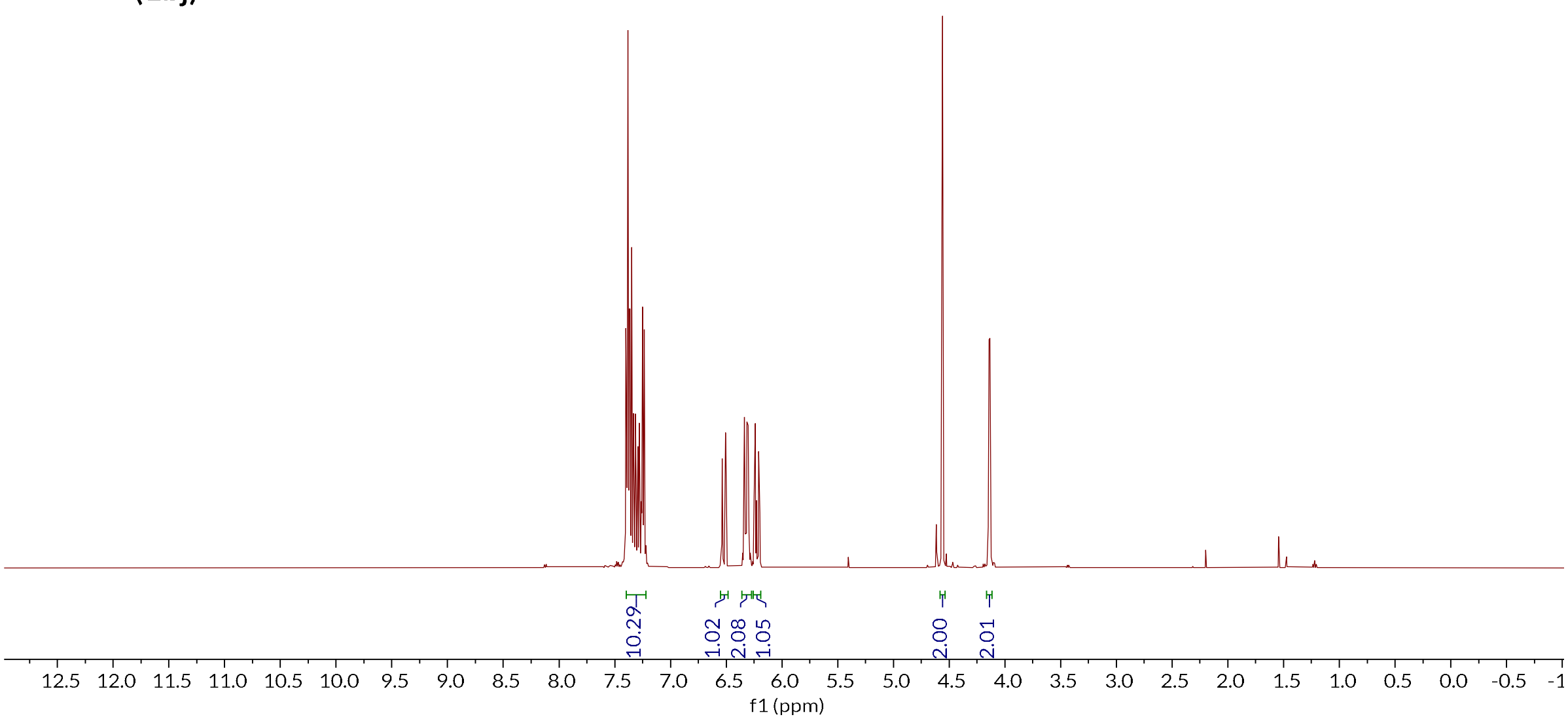

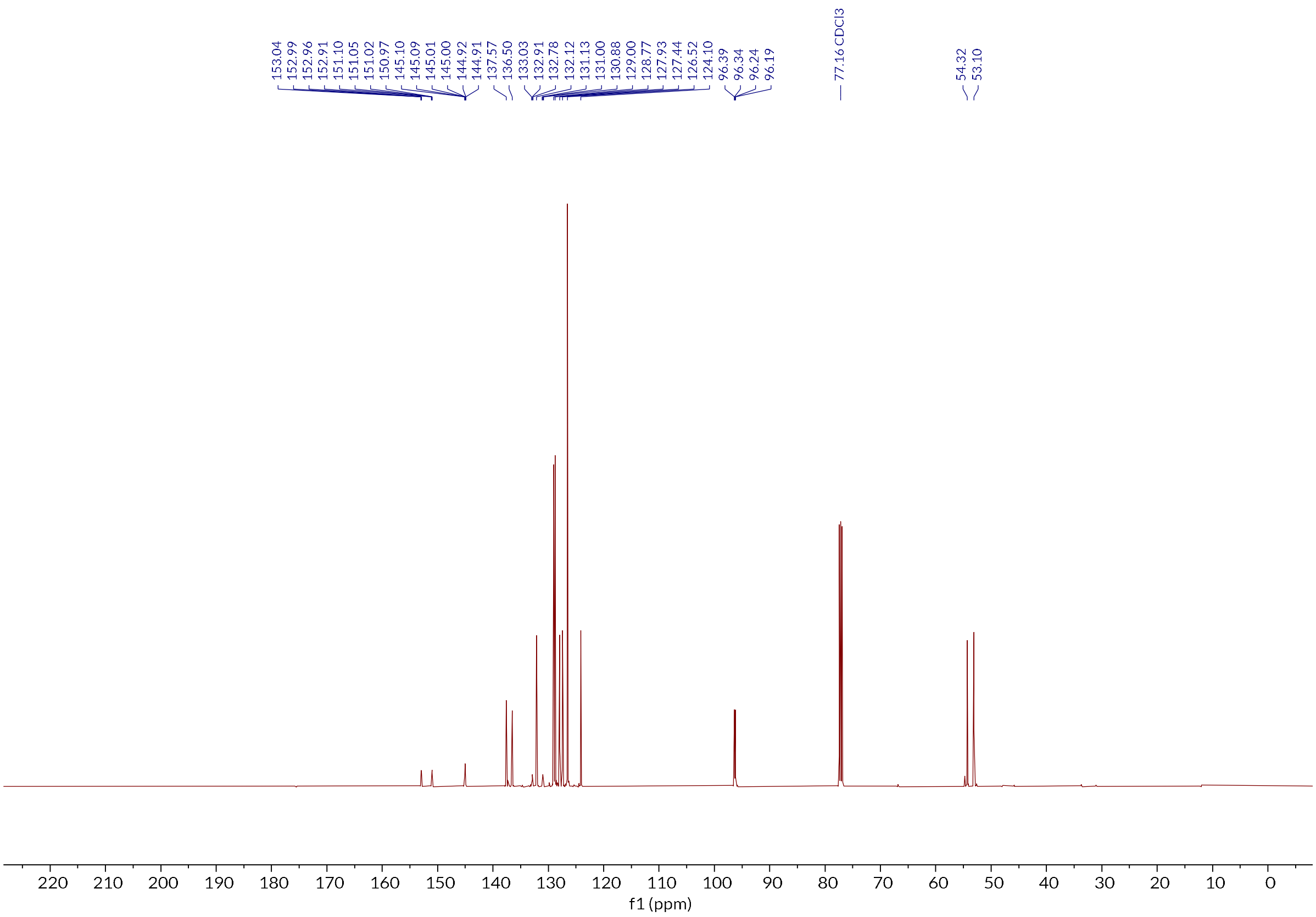

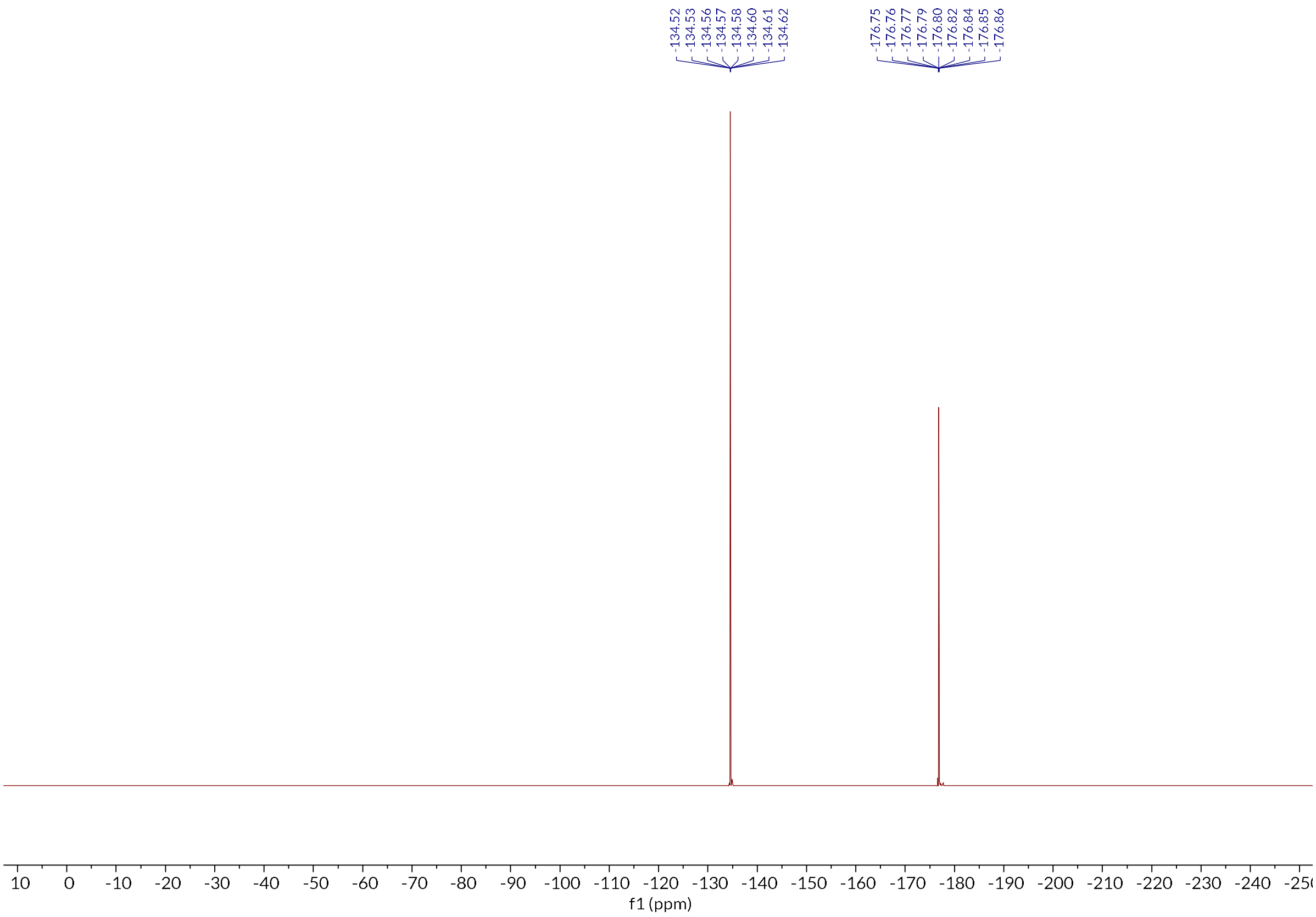
-134.52
-134.53
-134.56
-134.57
-134.58
-134.60
-134.61
-134.62
-176.75
-176.76
-176.77
-176.79
-176.80
-176.82
-176.84
-176.85
-176.86
10 0 -10 -20 -30 -40 -50 -60 -70 -80 -90 -100 -110 -120 -130 -140 -150 -160 -170 -180 -190 -200 -210 -220 -230 -240
f1 (ppm)

N-Cinnamyl-[1,1'-biphenyl]-3-amine (SI-29)

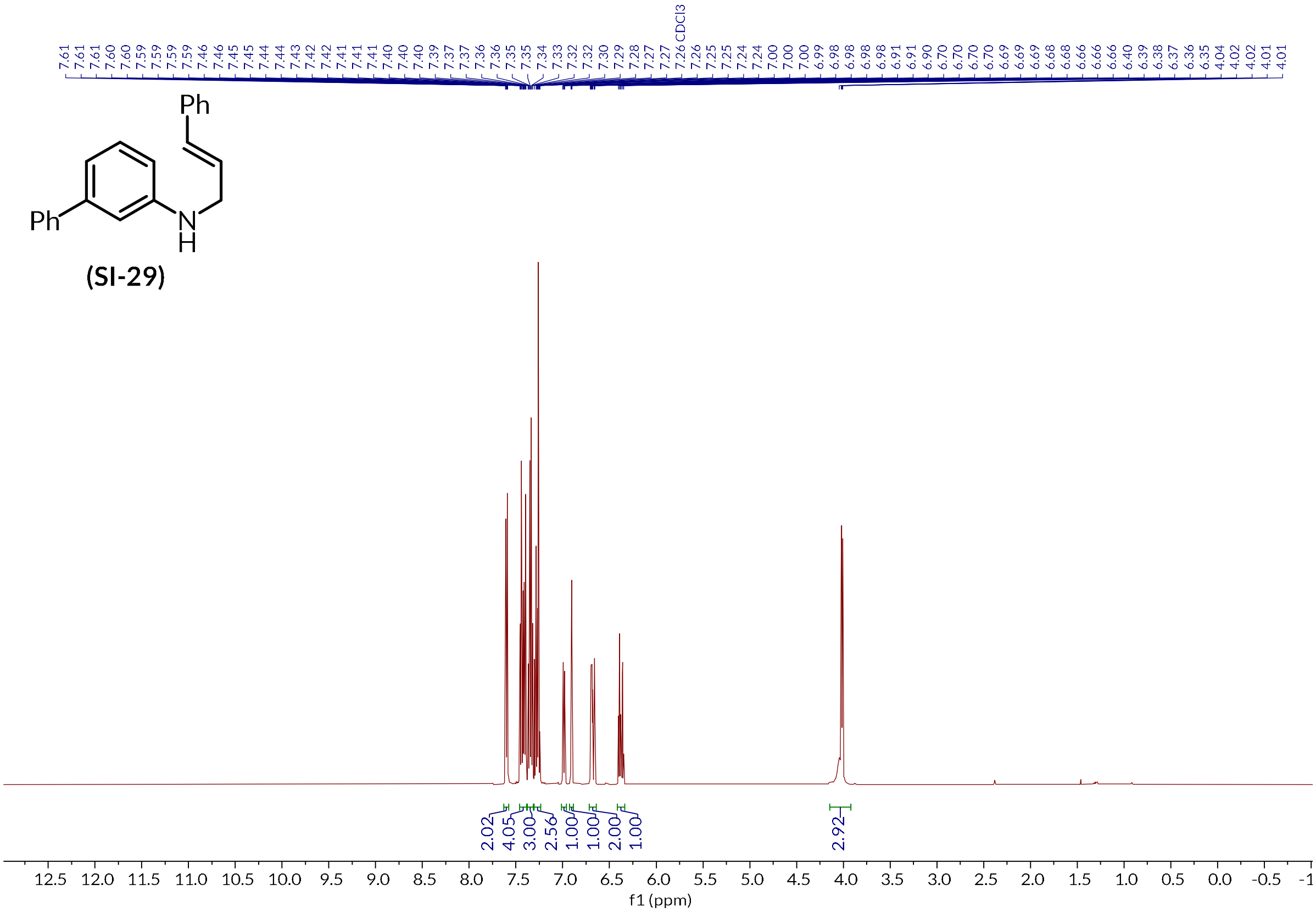

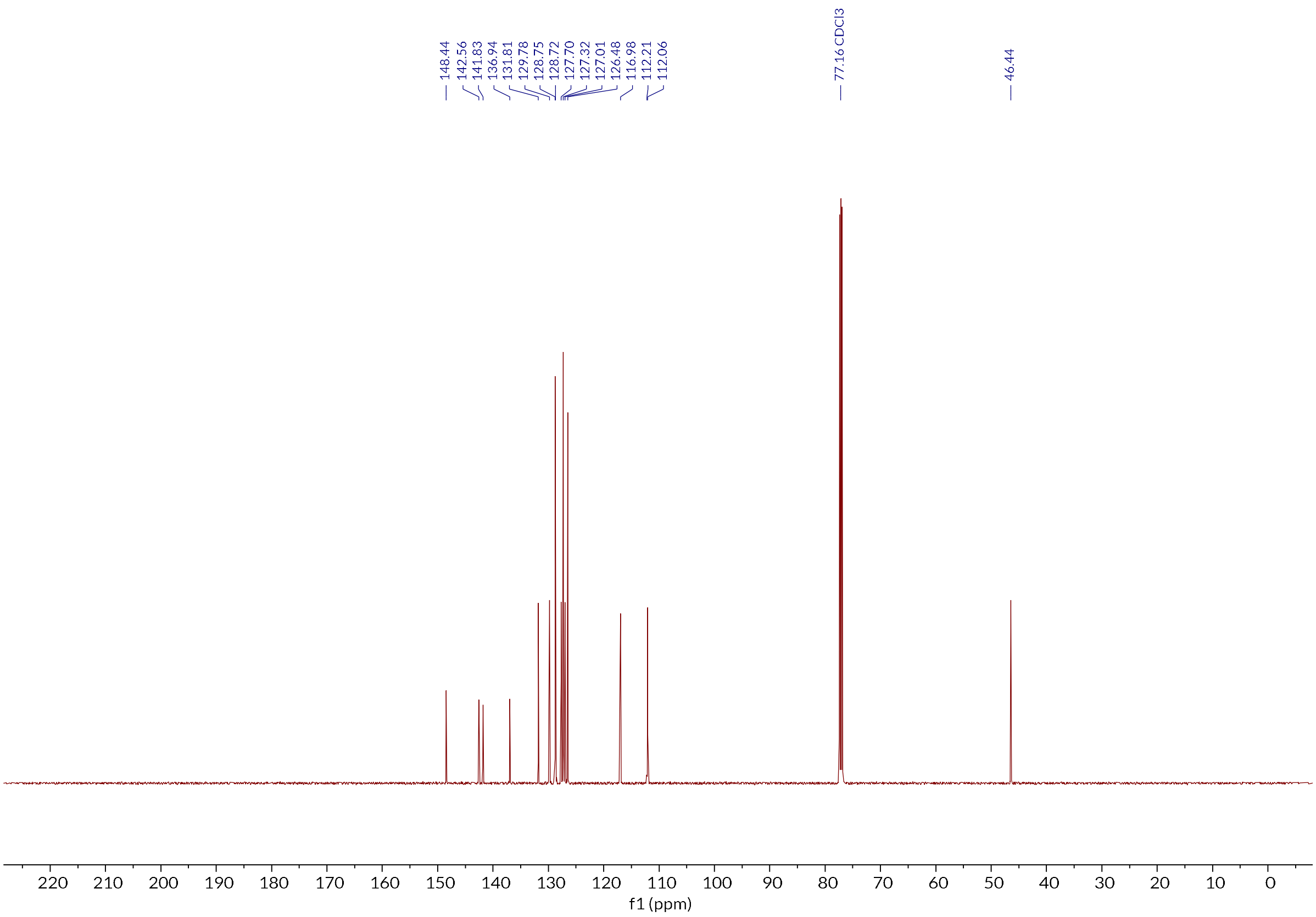

N-Benzyl-*N*-cinnamyl-[1,1'-biphenyl]-3-amine (1bk)

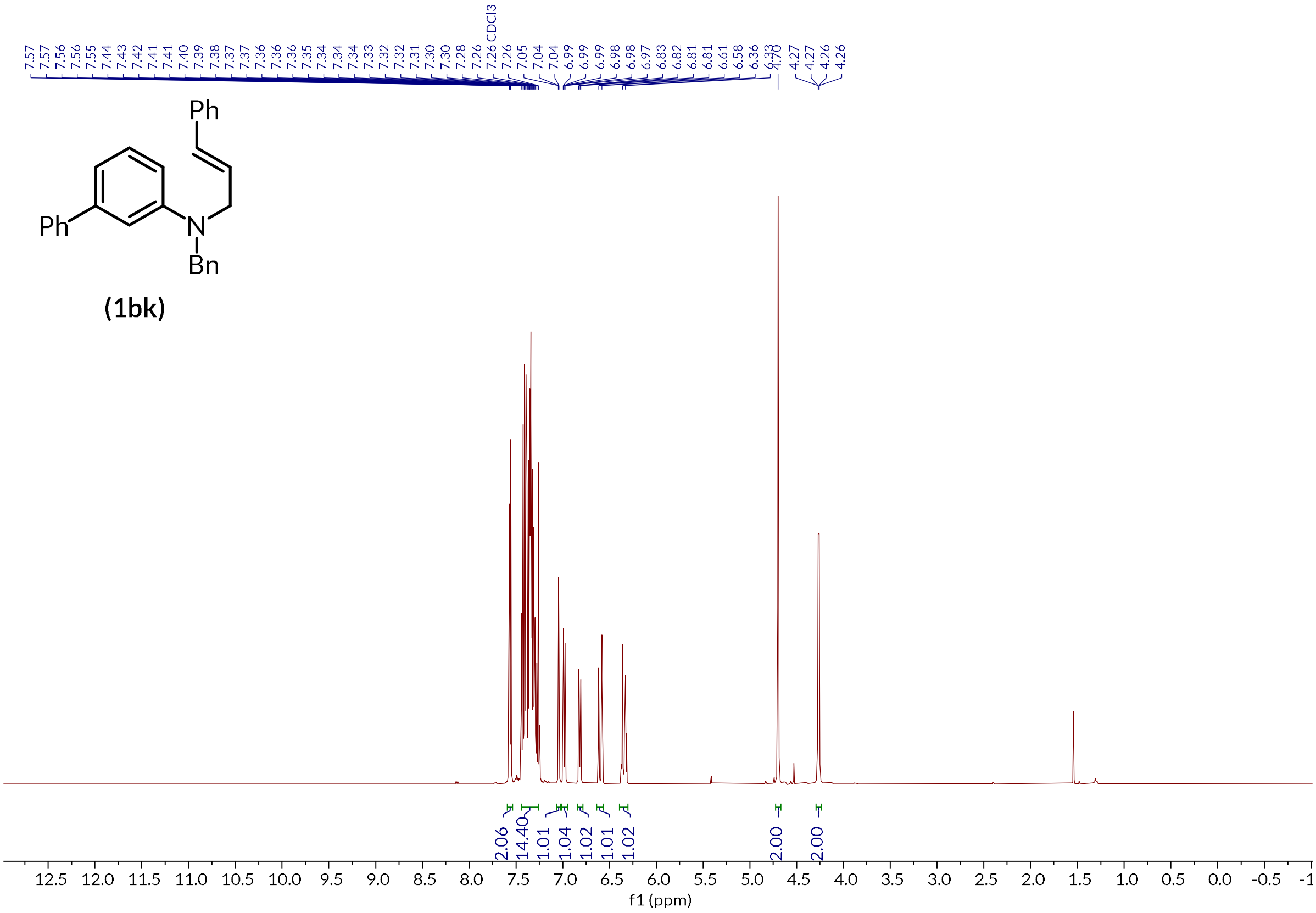

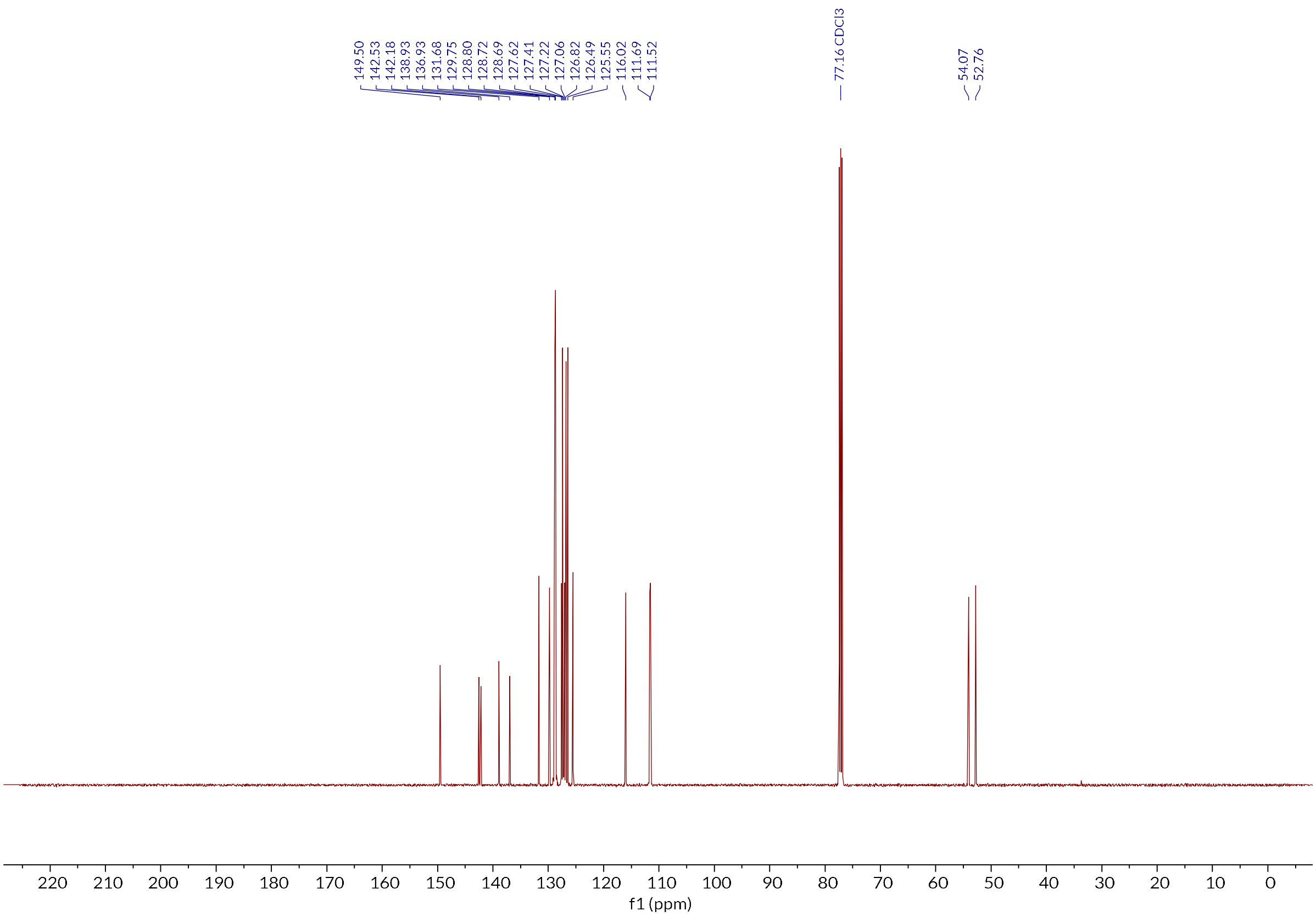

3-(*Tert*-butyl)-*N*-cinnamylaniline (SI-30)

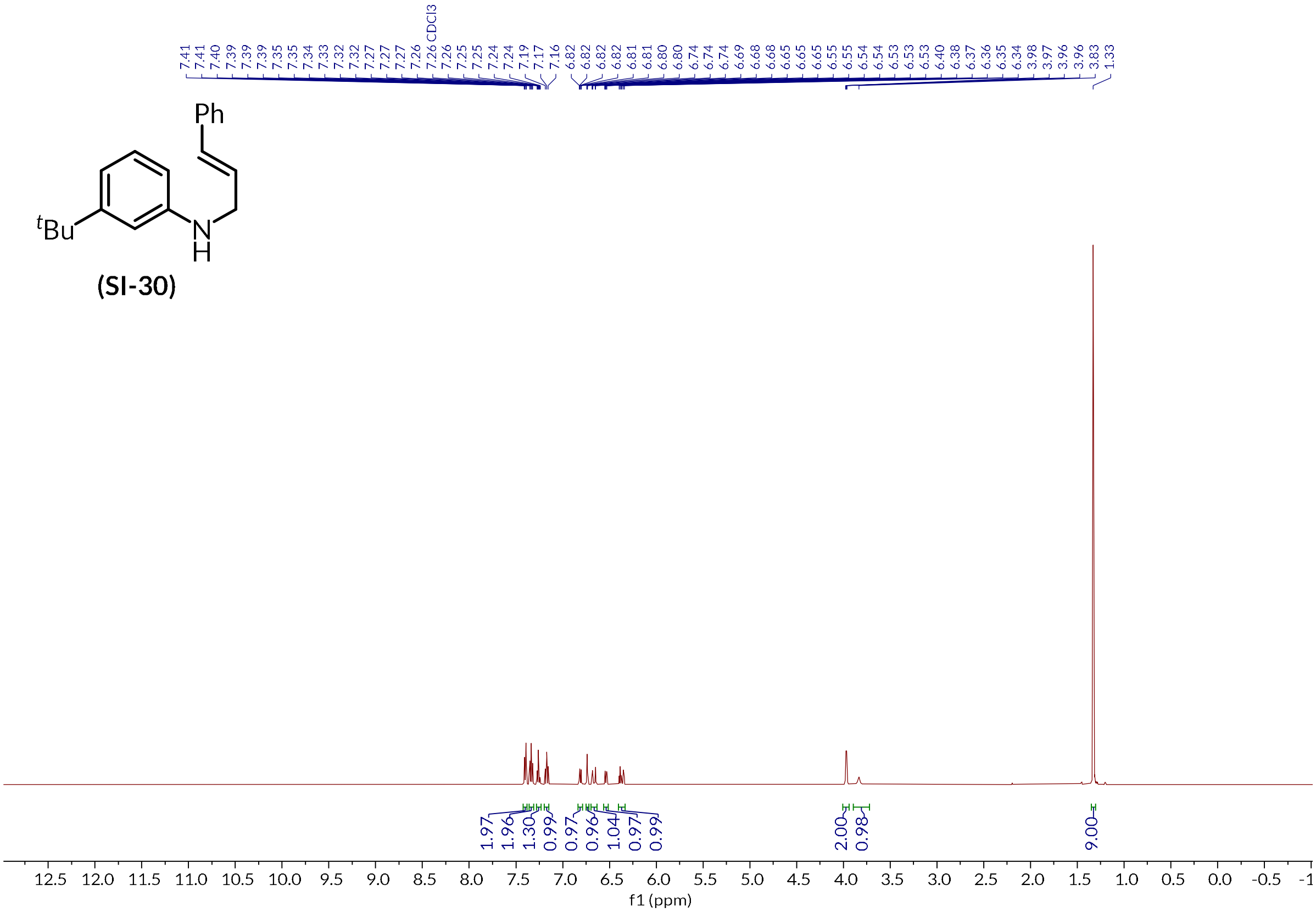

(SI-30)

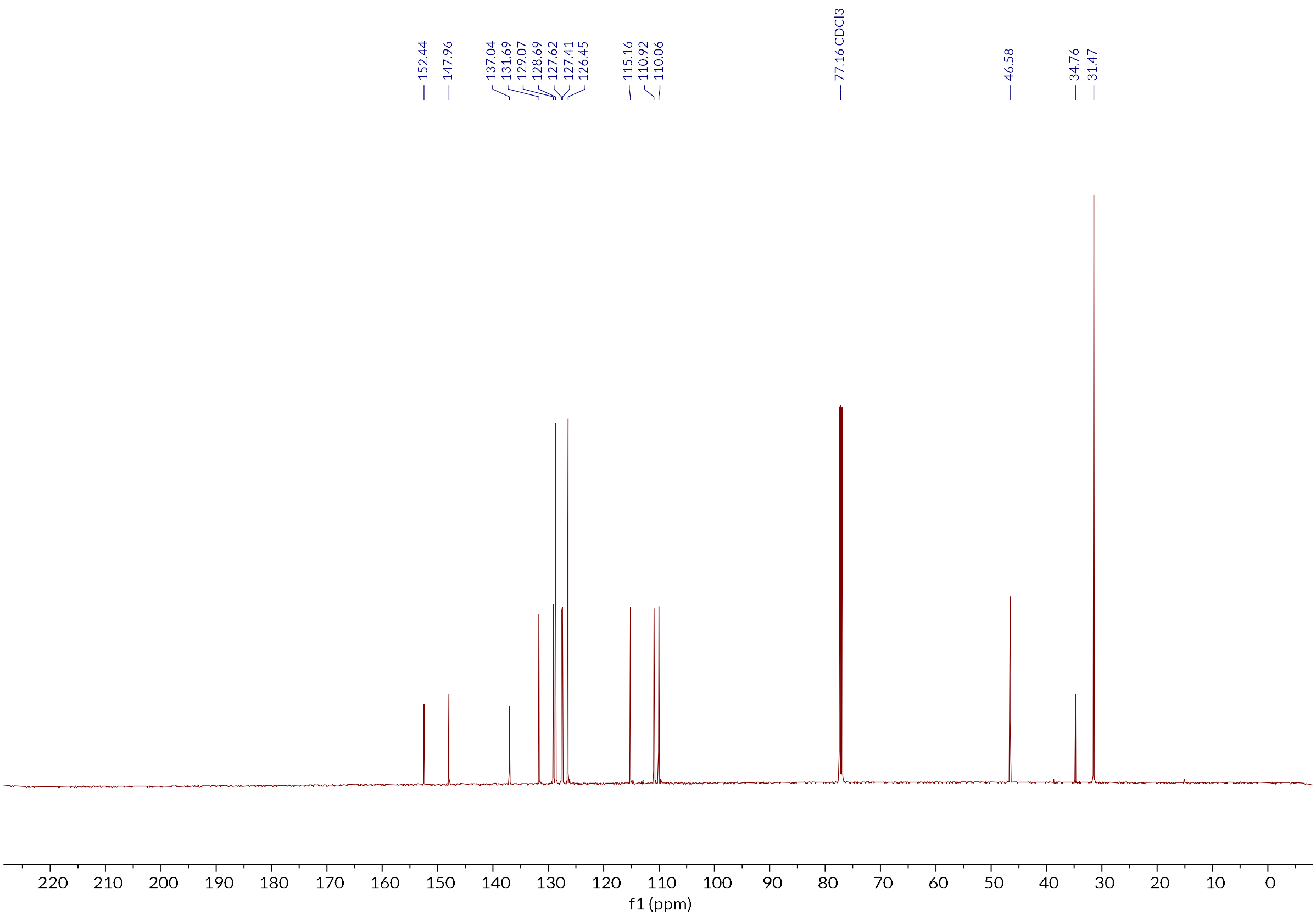

N-Benzyl-3-(*tert*-butyl)-N-cinnamylaniline (1bl)

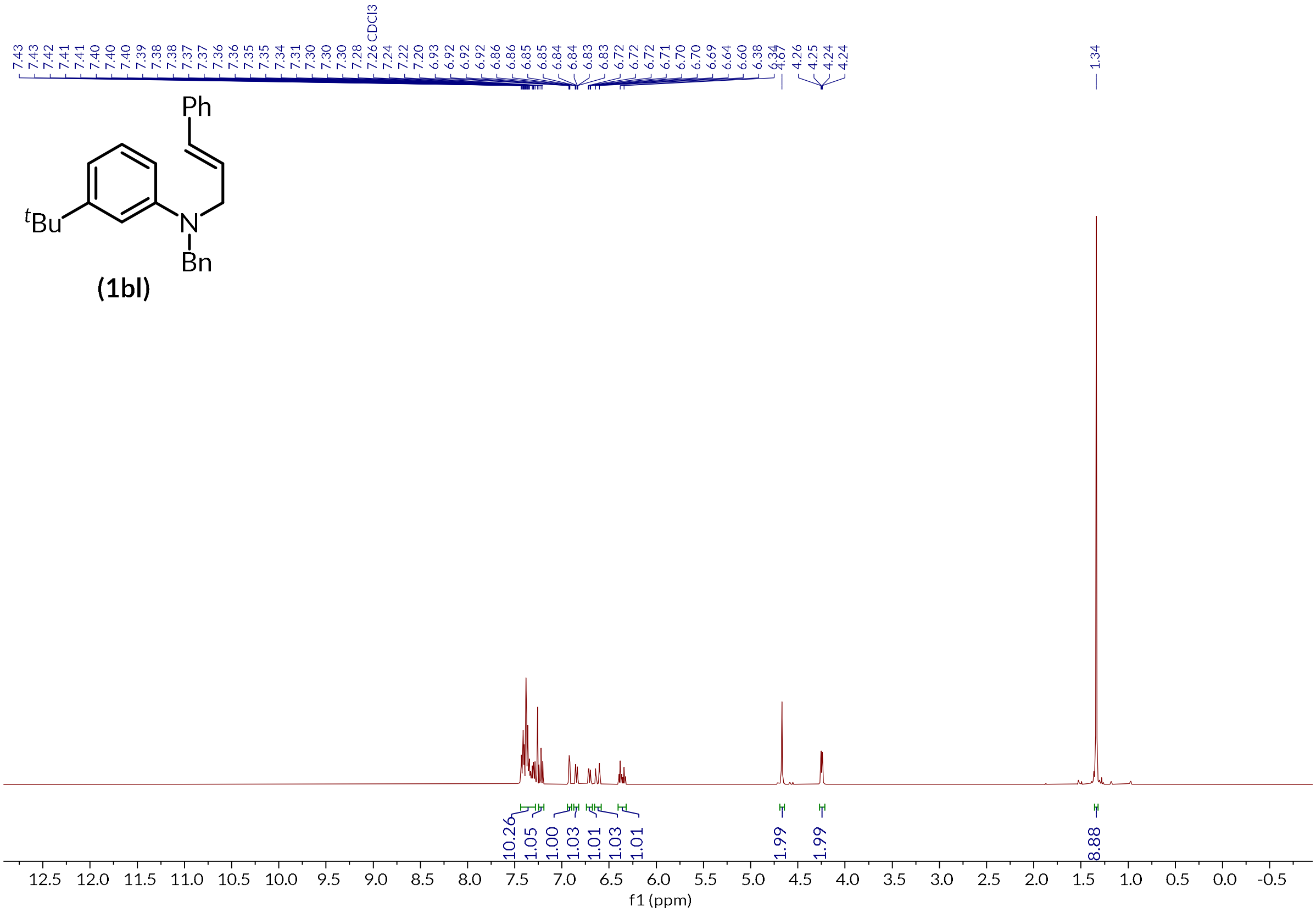

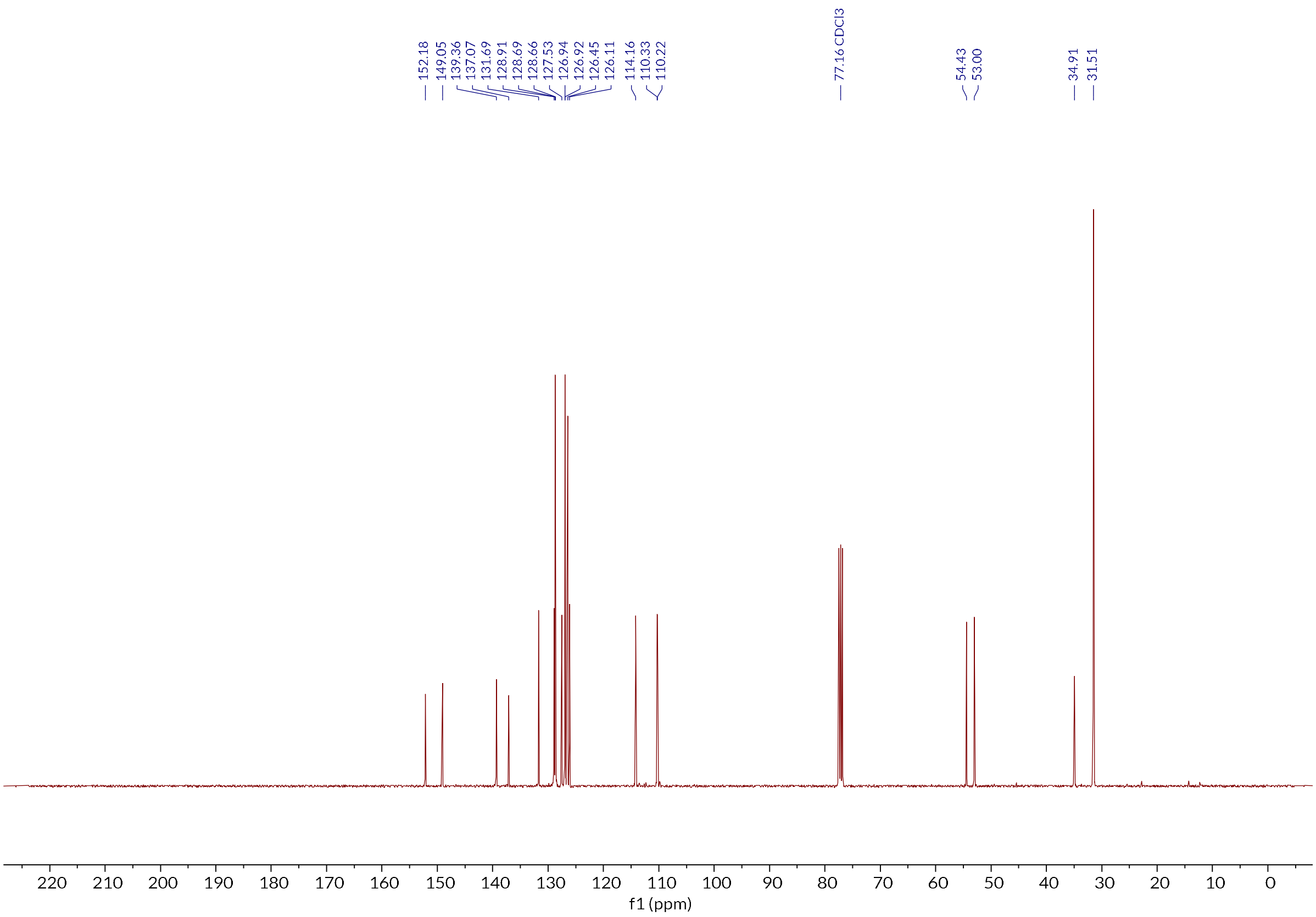

N-Cinnamyl-3-methoxyaniline (SI-31)

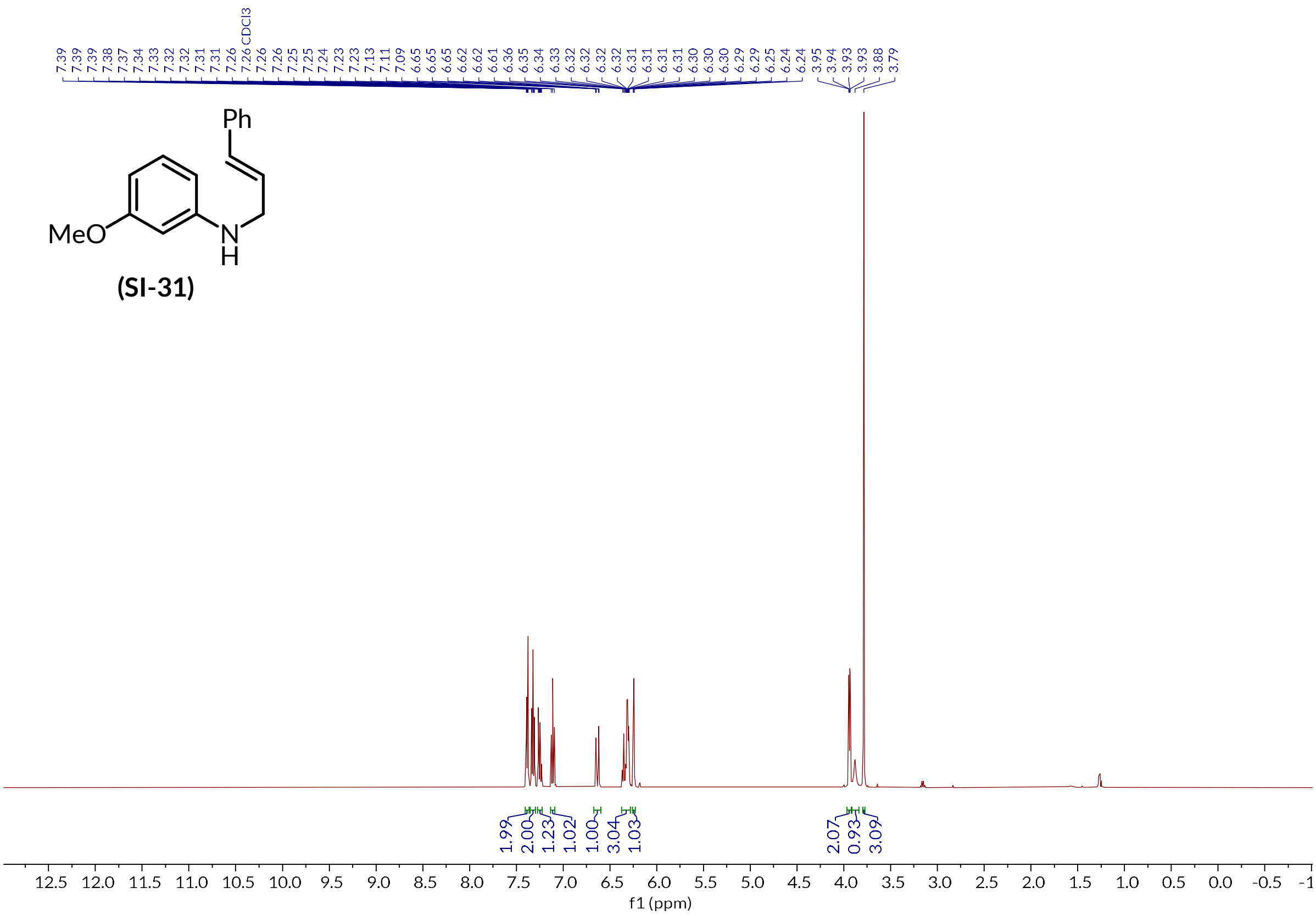

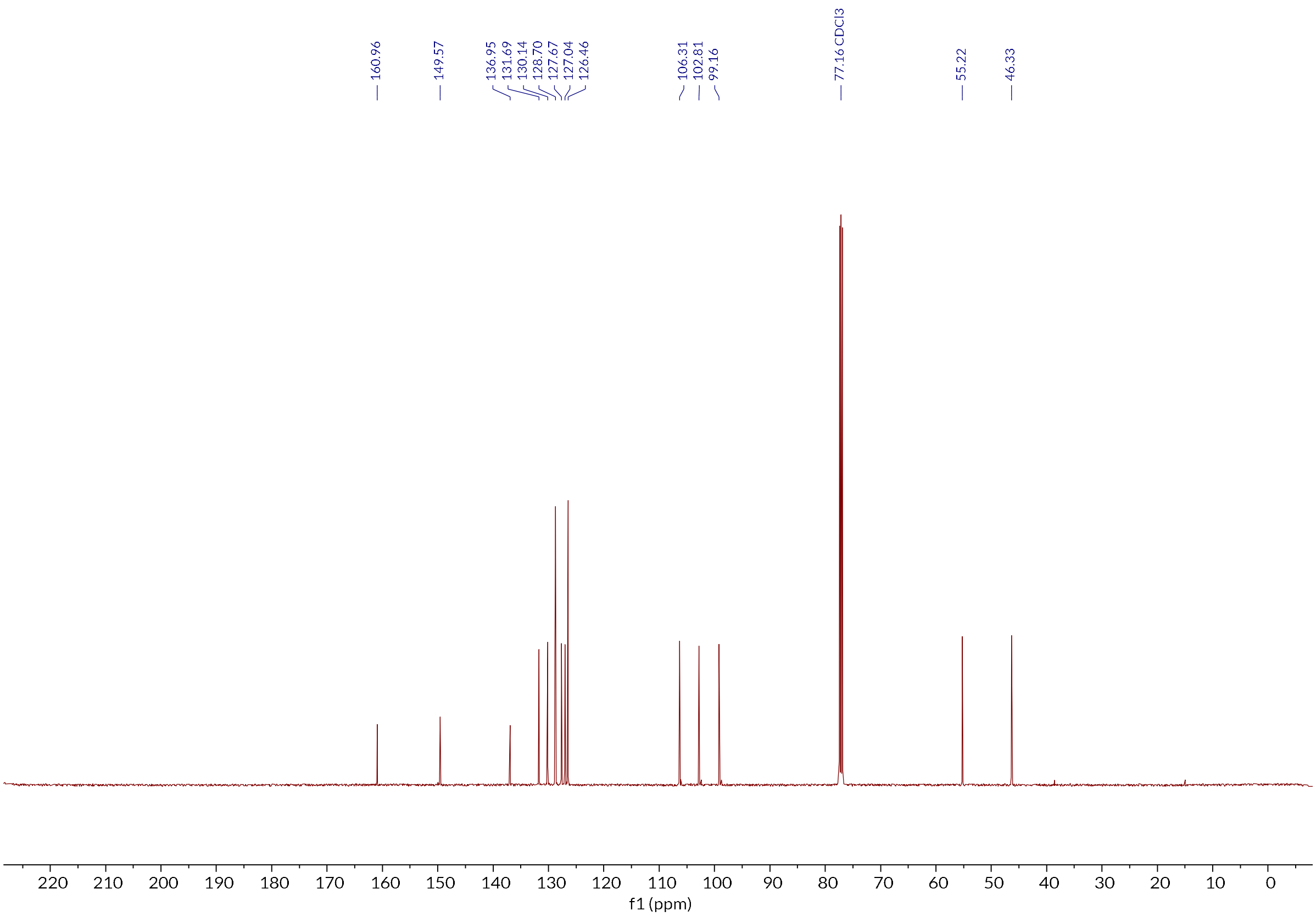

N-Benzyl-*N*-cinnamyl-3-methoxyaniline (1bm)

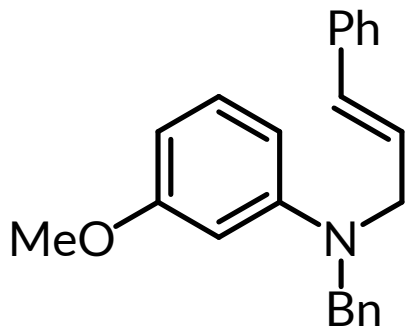

(1bm)

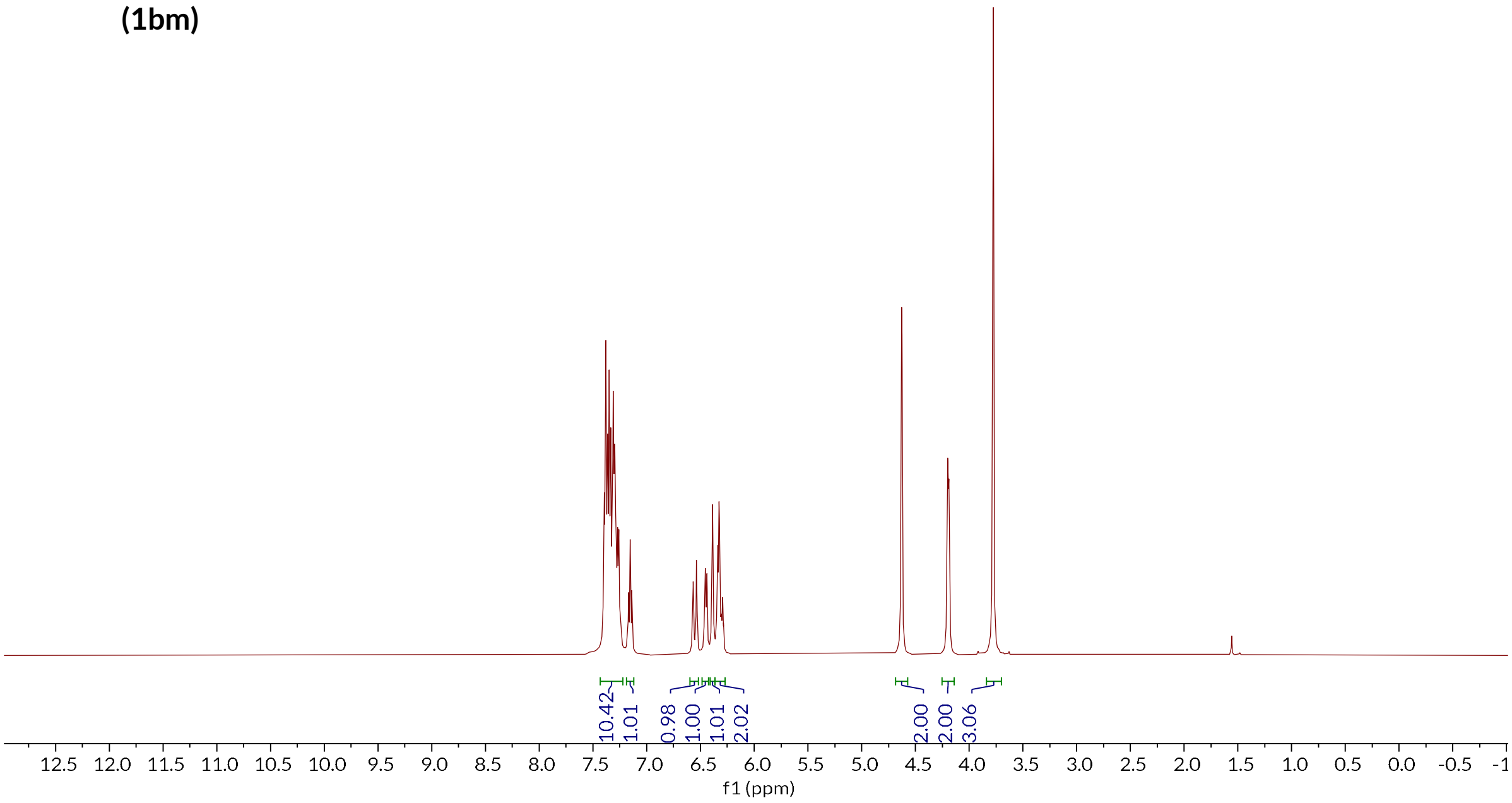

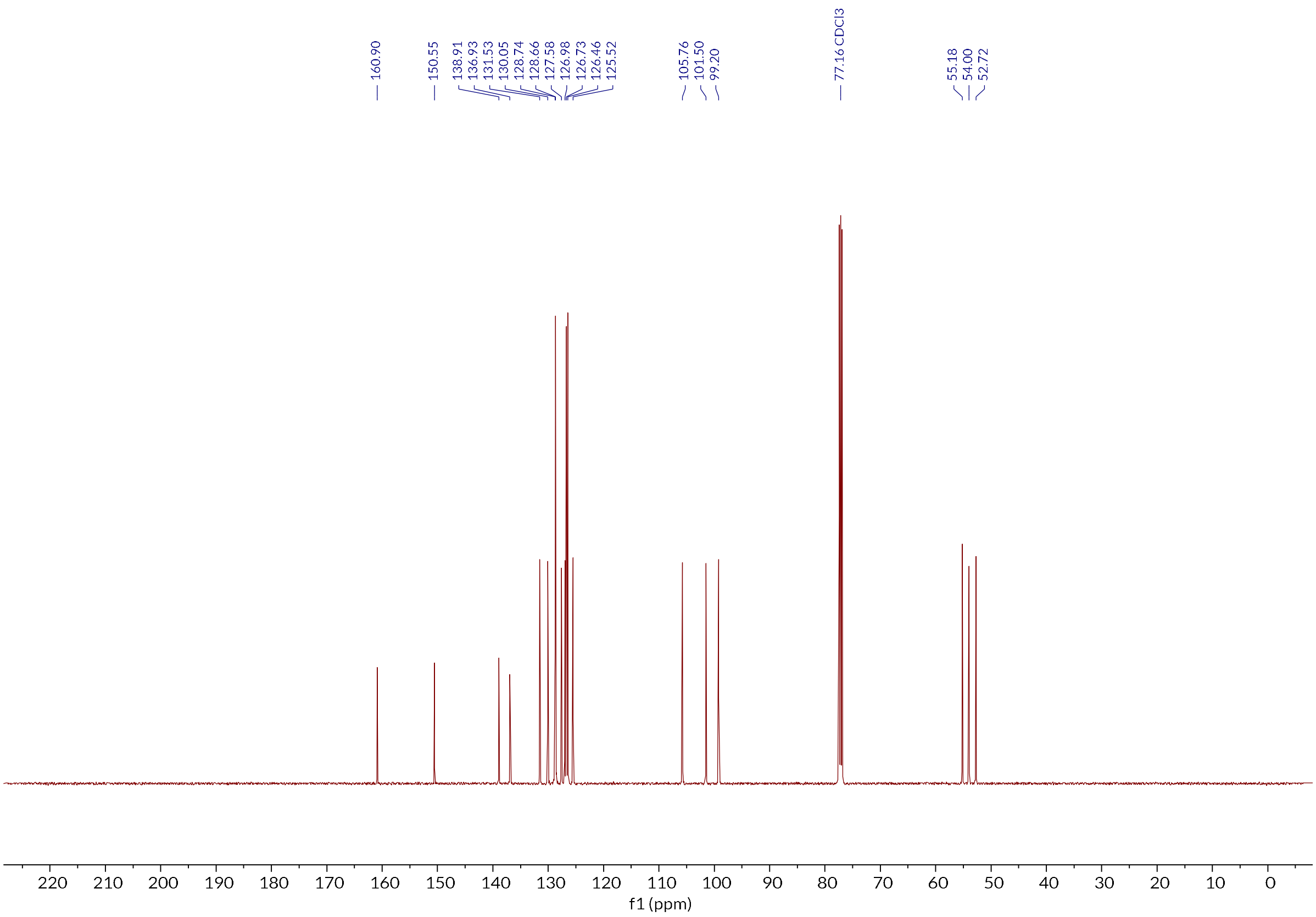

N-Cinnamyl-3-fluoroaniline (SI-32)

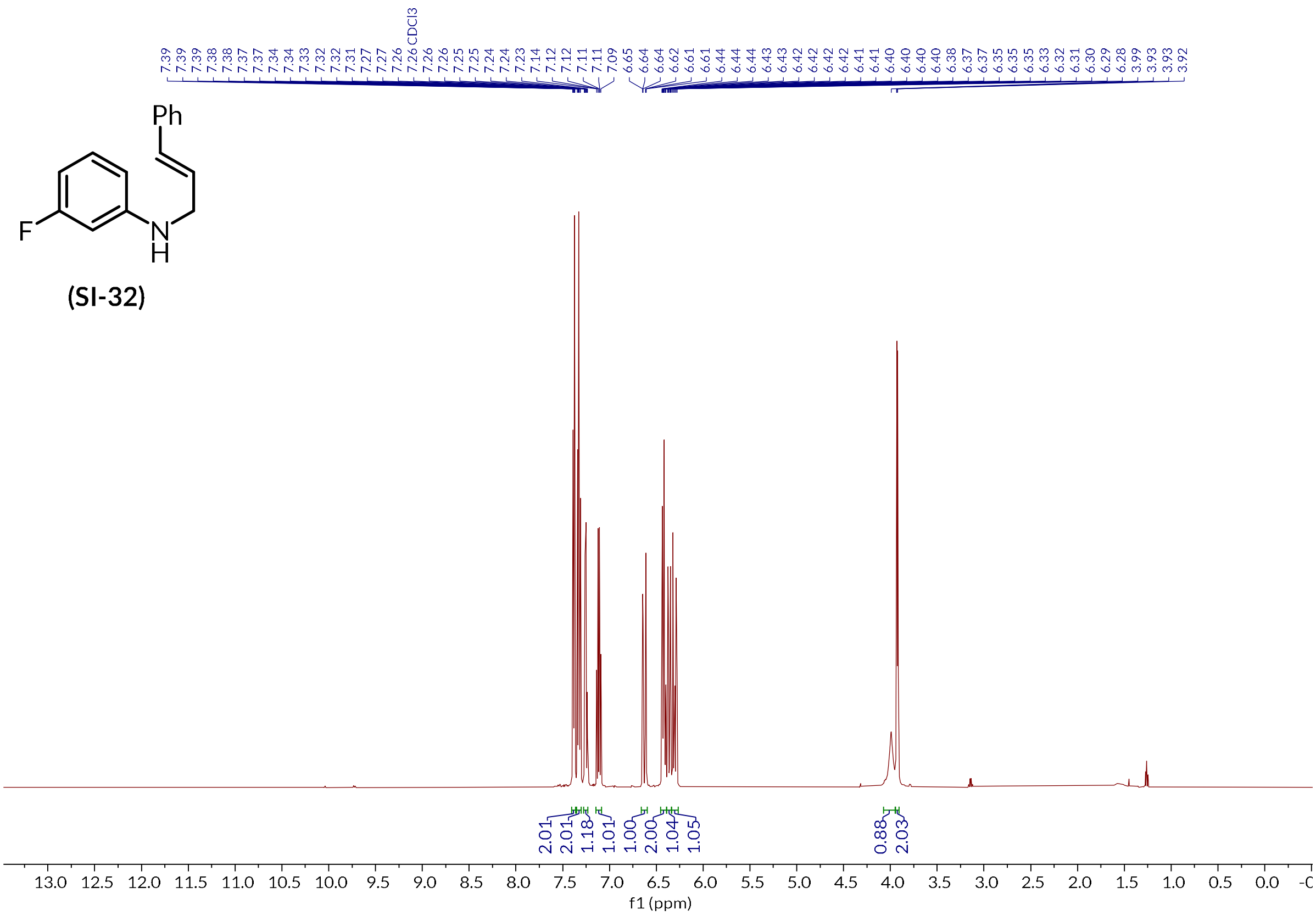

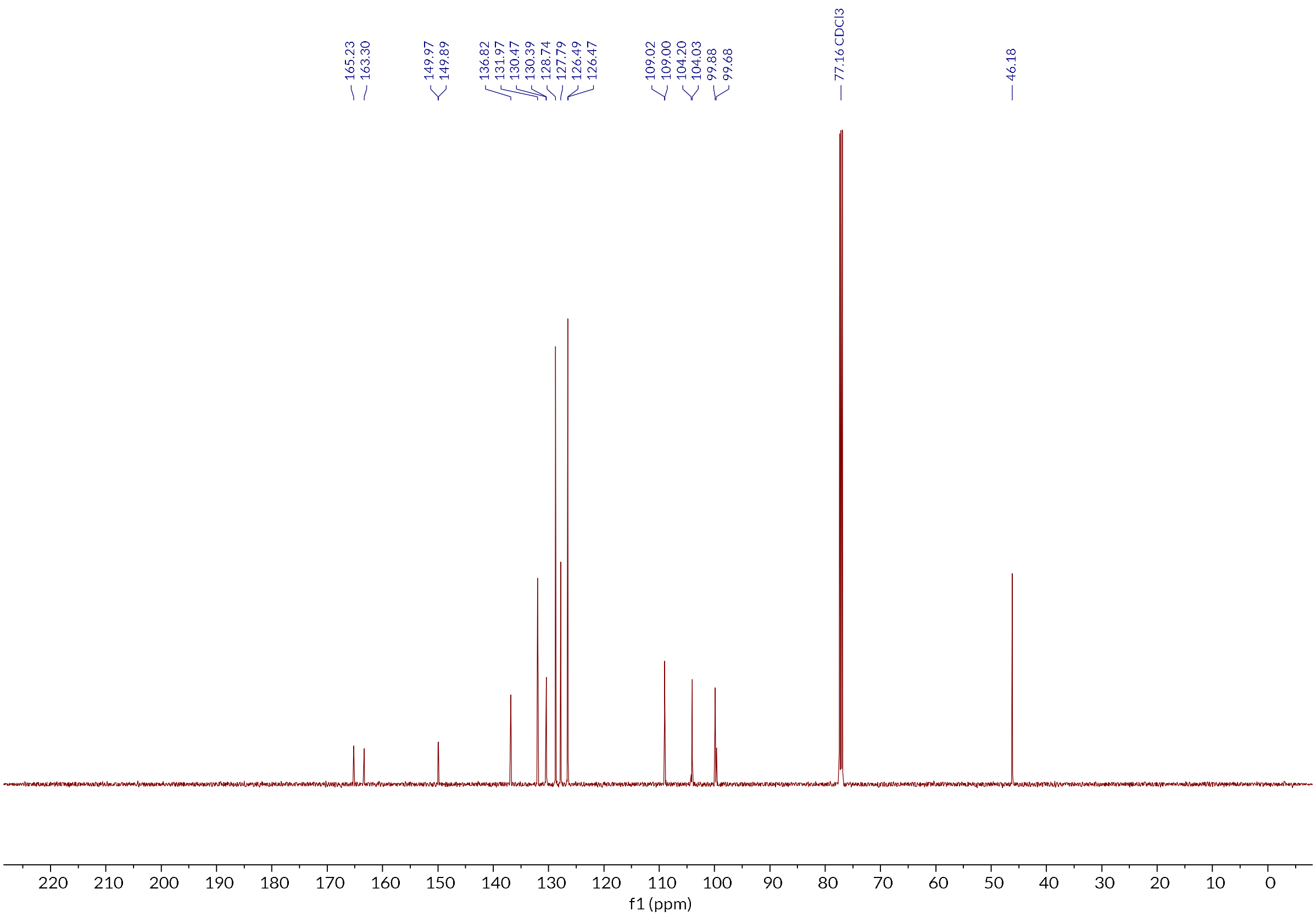

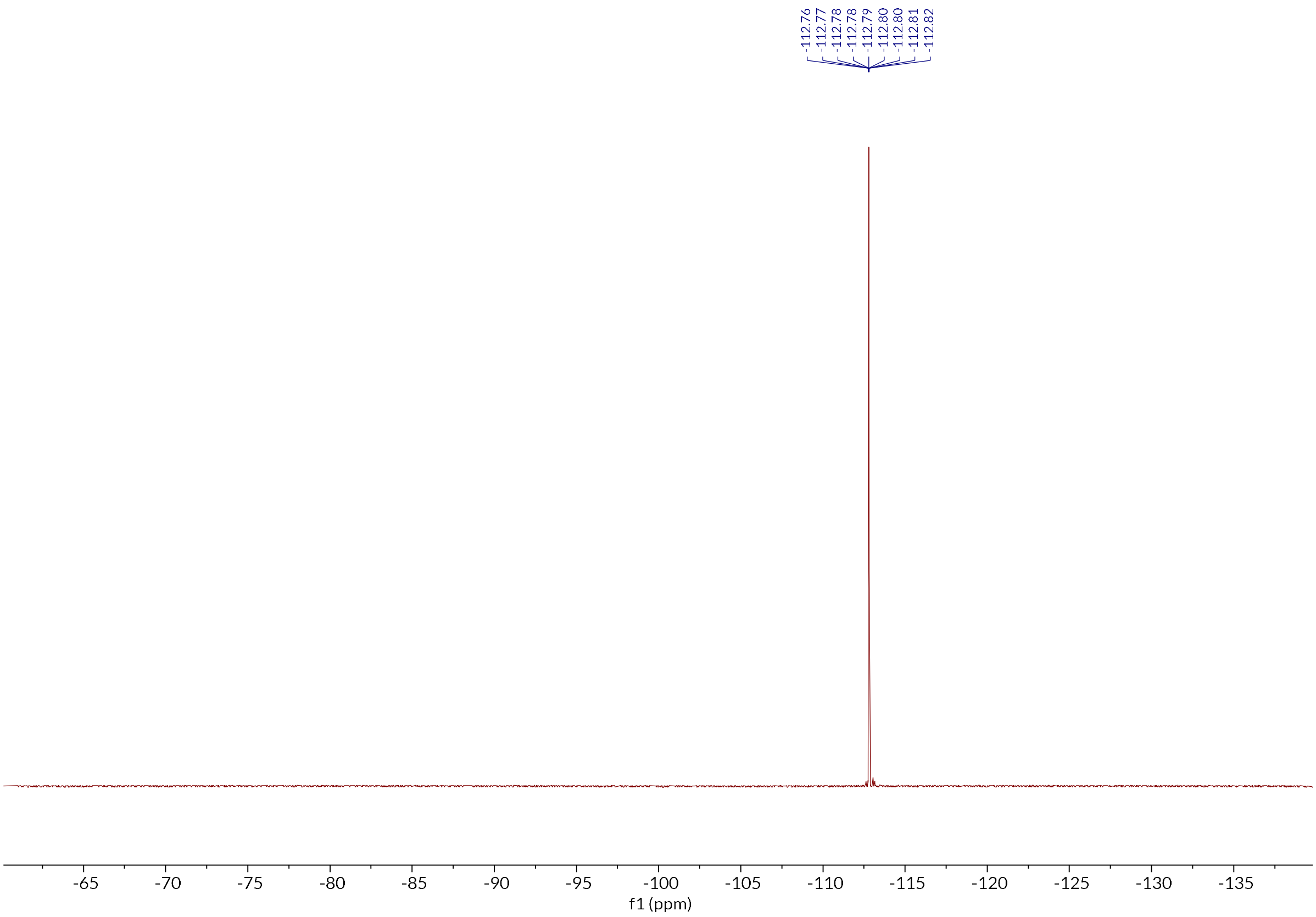
-112.76
-112.77
-112.78
-112.78
-112.79
-112.80
-112.80
-112.81
-112.82
-65
-70
-75
-80
-85
-90
-95
-100
-105
-110
-115
-120
-125
-130
-135
f1 (ppm)

N-Benzyl-*N*-cinnamyl-3-fluoroaniline (1bn)

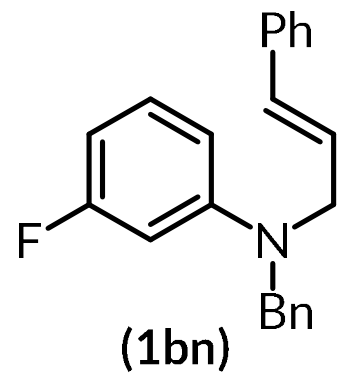

(1bn)

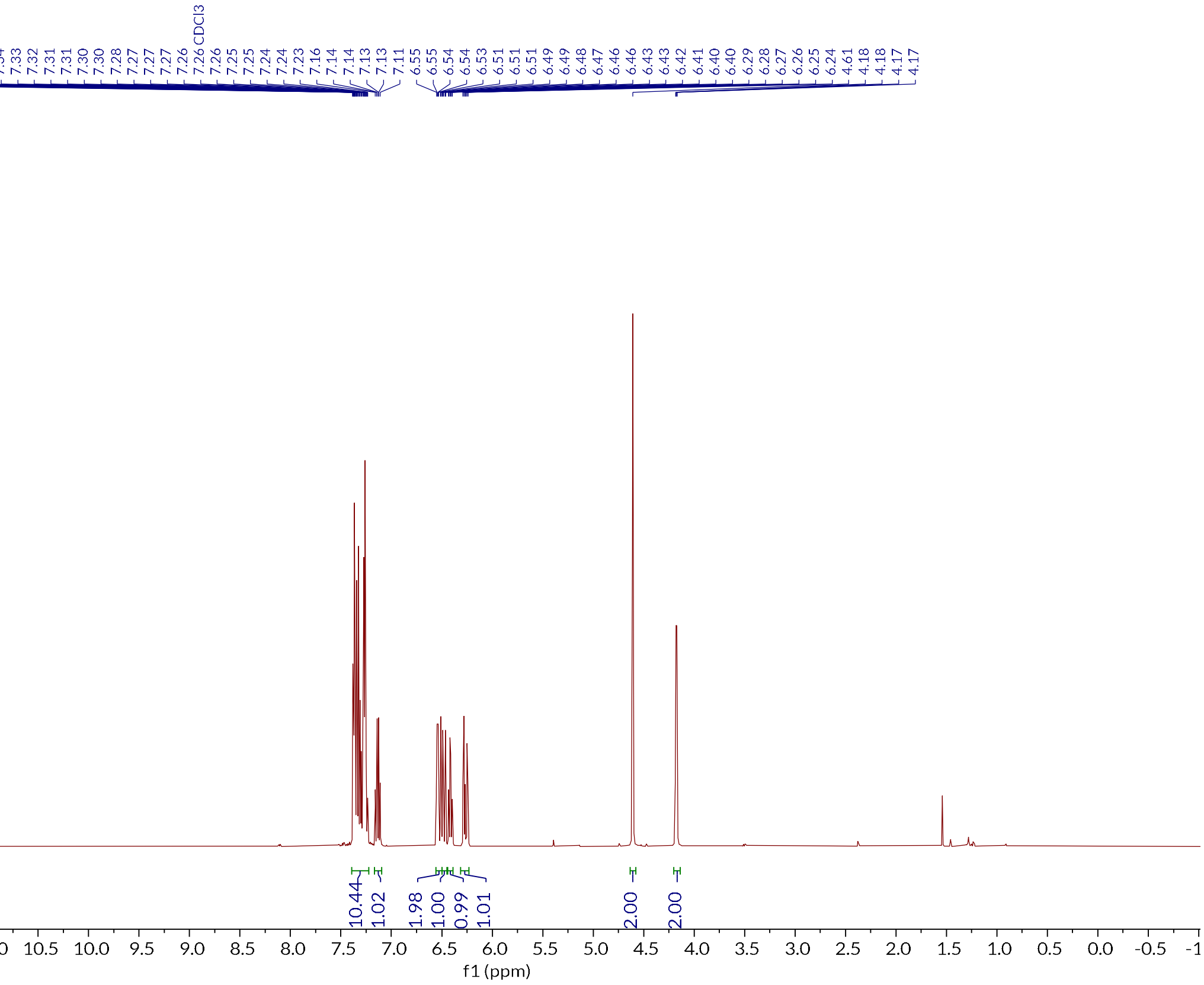

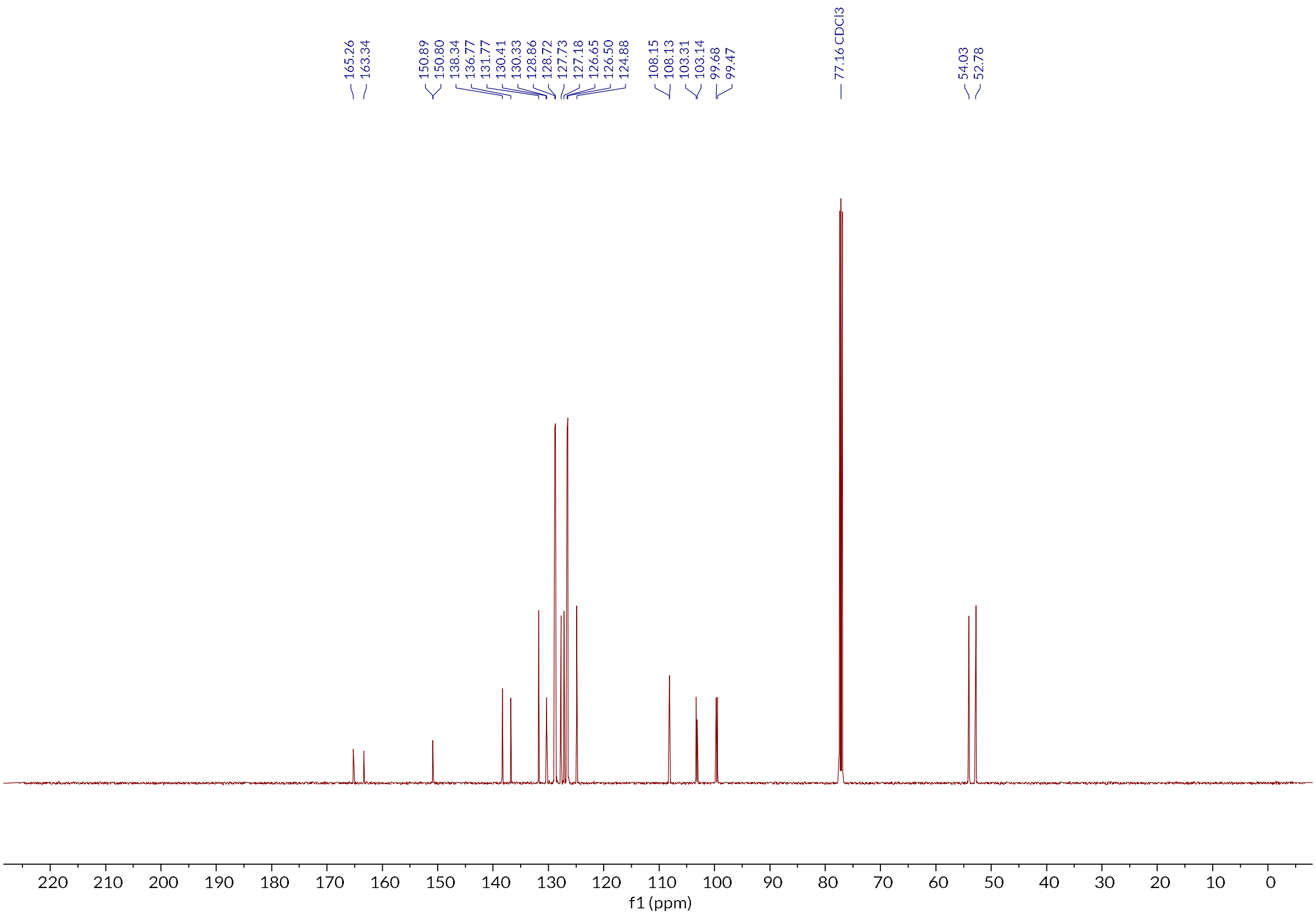

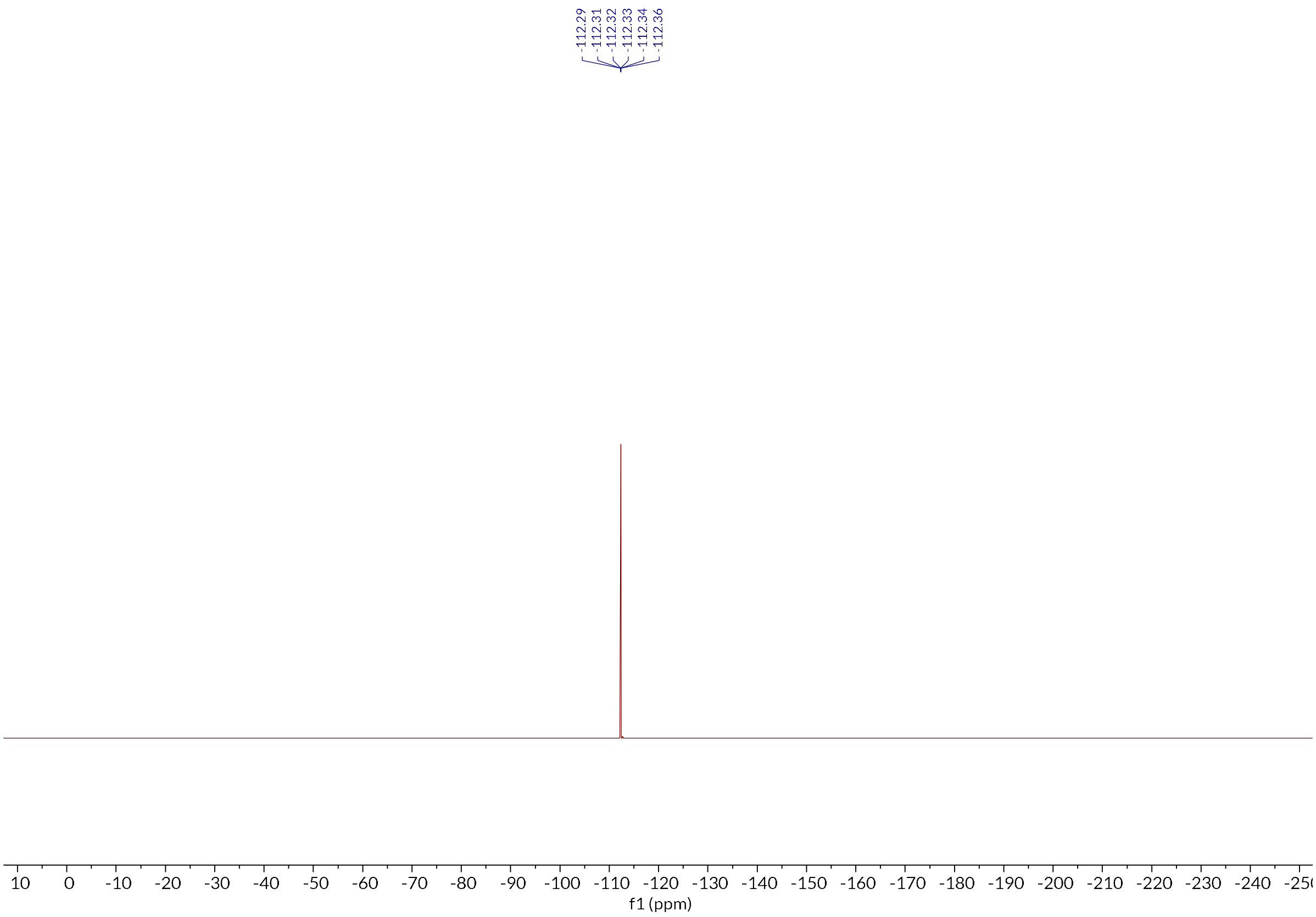
-112.29
-112.31
-112.32
-112.33
-112.34
-112.36
10 0 -10 -20 -30 -40 -50 -60 -70 -80 -90 -100 -110 -120 -130 -140 -150 -160 -170 -180 -190 -200 -210 -220 -230 -240
f1 (ppm)

N-Cinnamyl-3-(trifluoromethyl)aniline (SI-33)

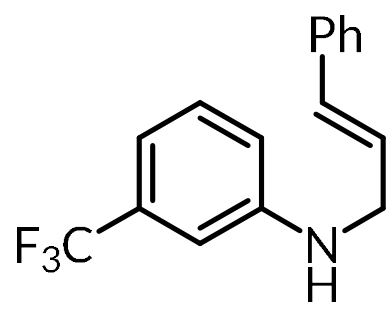

(SI-33)

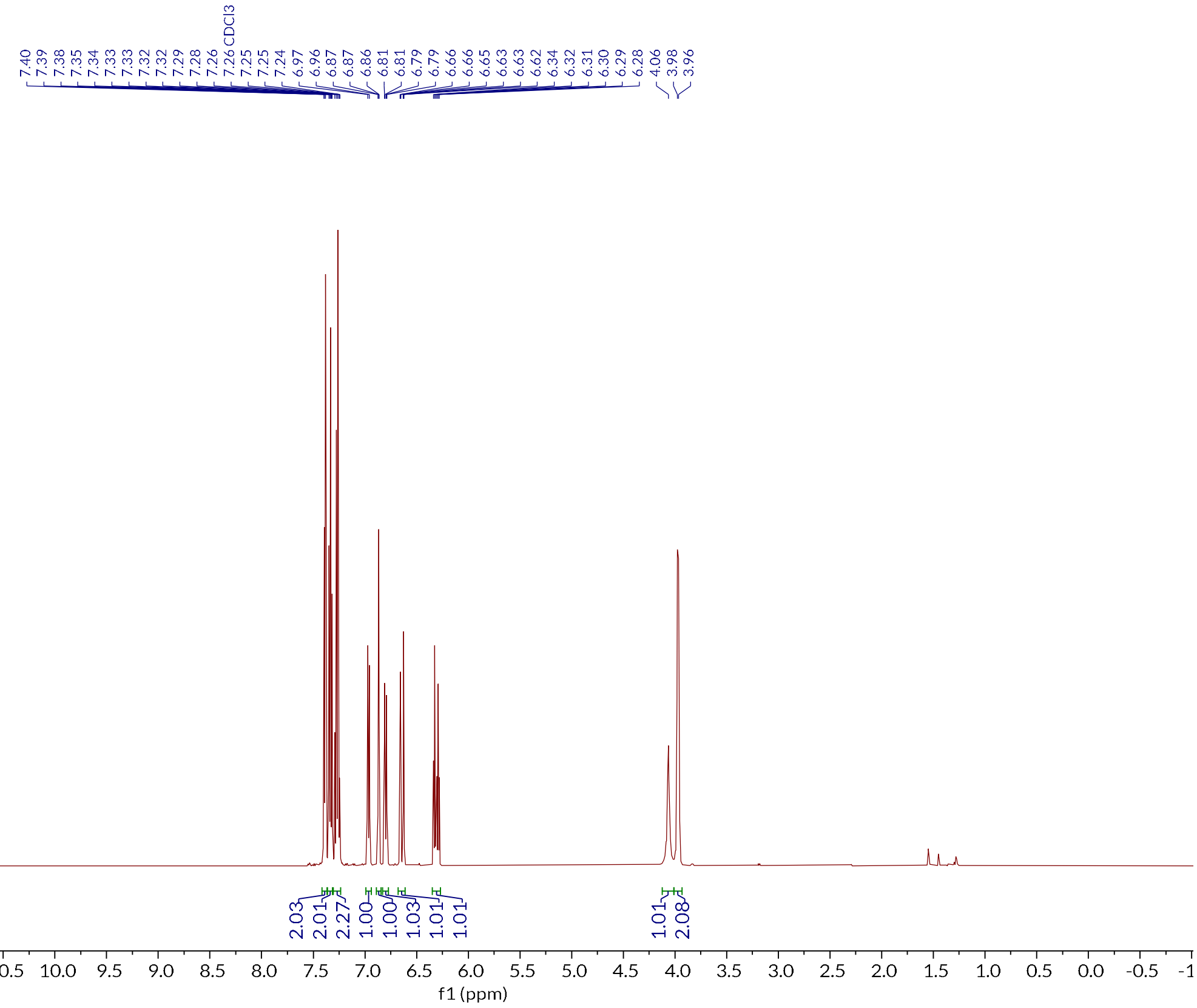

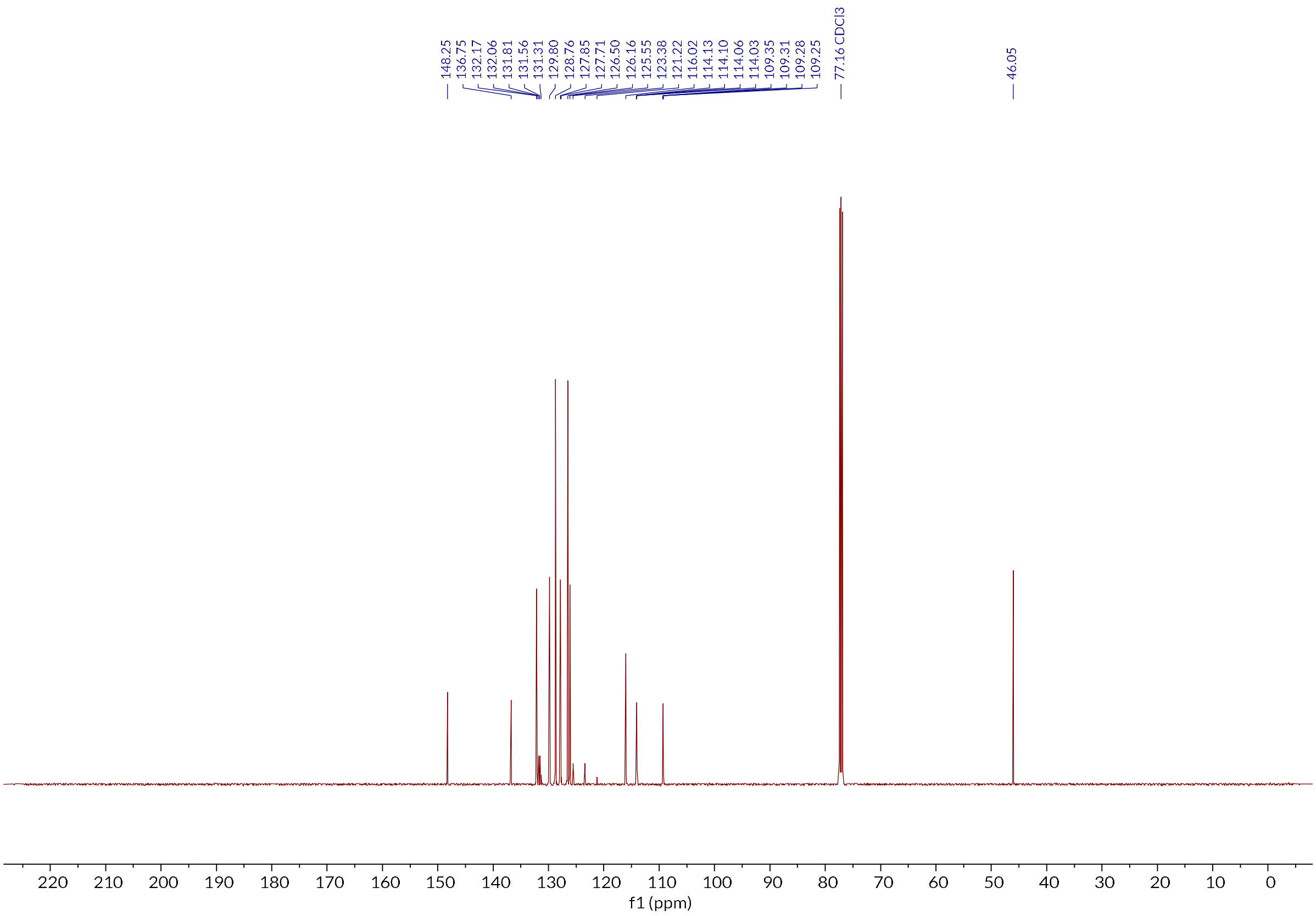

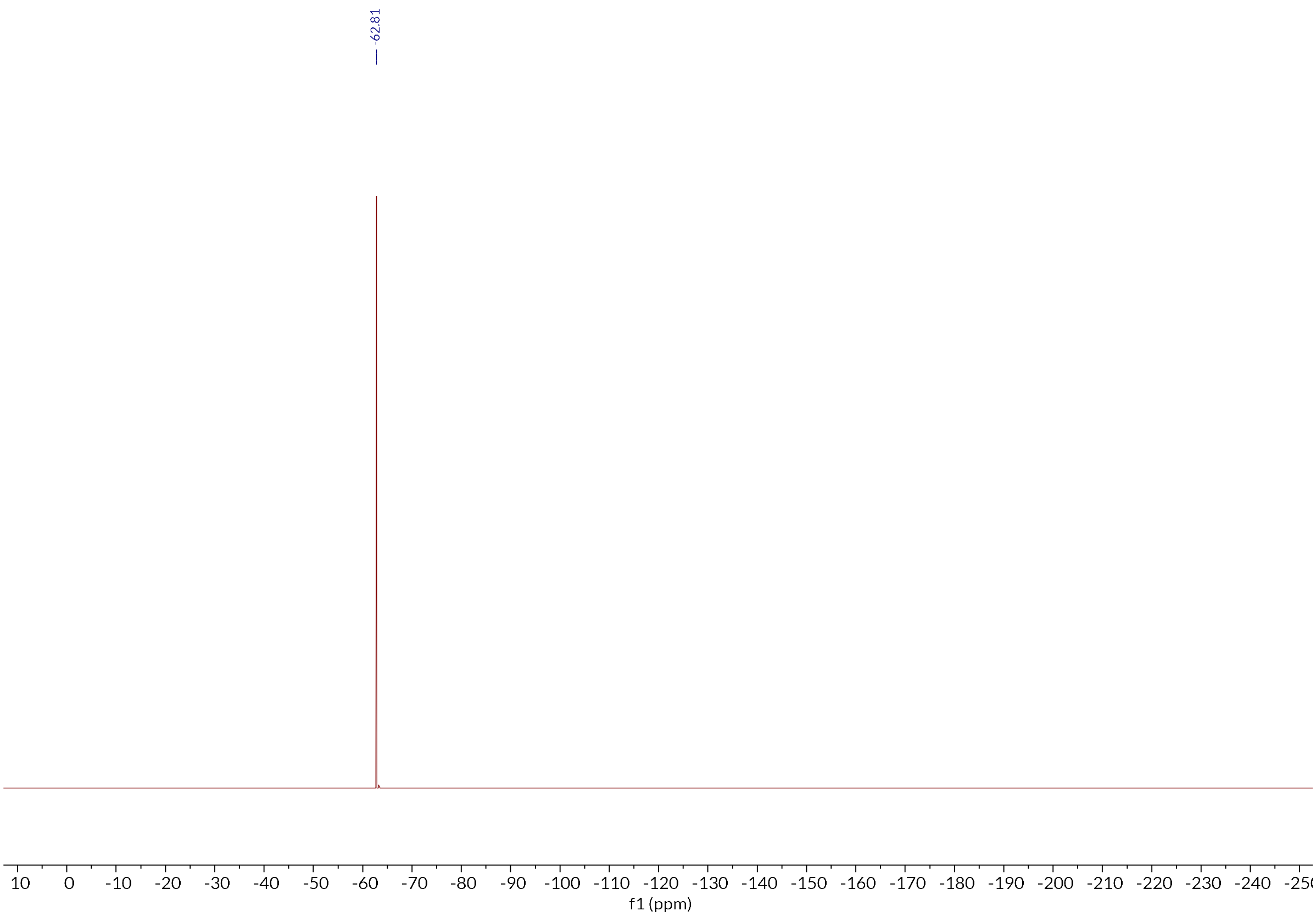
-62.81
10
0
-10
-20
-30
-40
-50
-60
-70
-80
-90
-100
-110
-120
-130
-140
-150
-160
-170
-180
-190
-200
-210
-220
-230
-240
f1 (ppm)

N-Benzyl-*N*-cinnamyl-3-(trifluoromethyl)aniline (1bo)

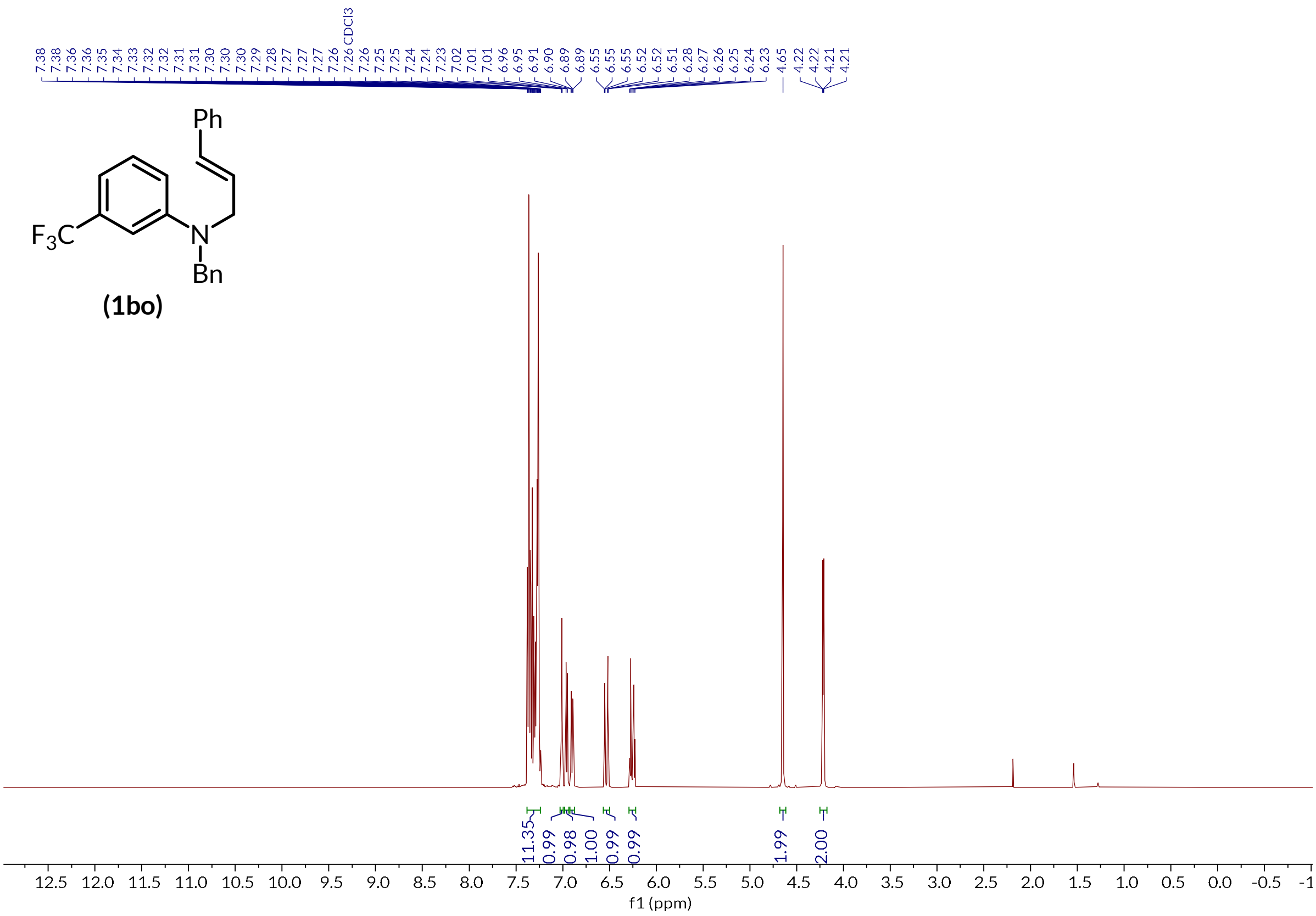

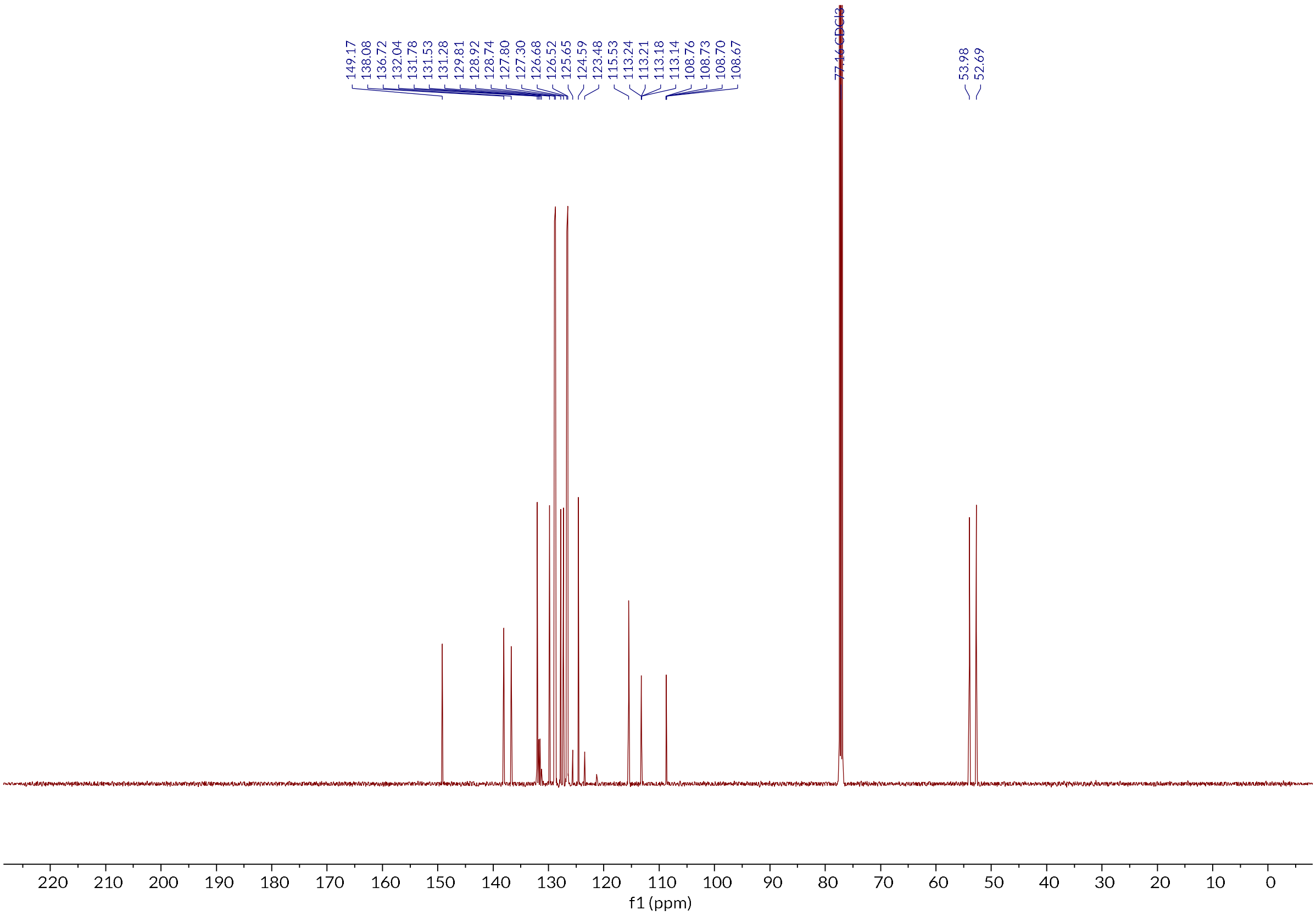

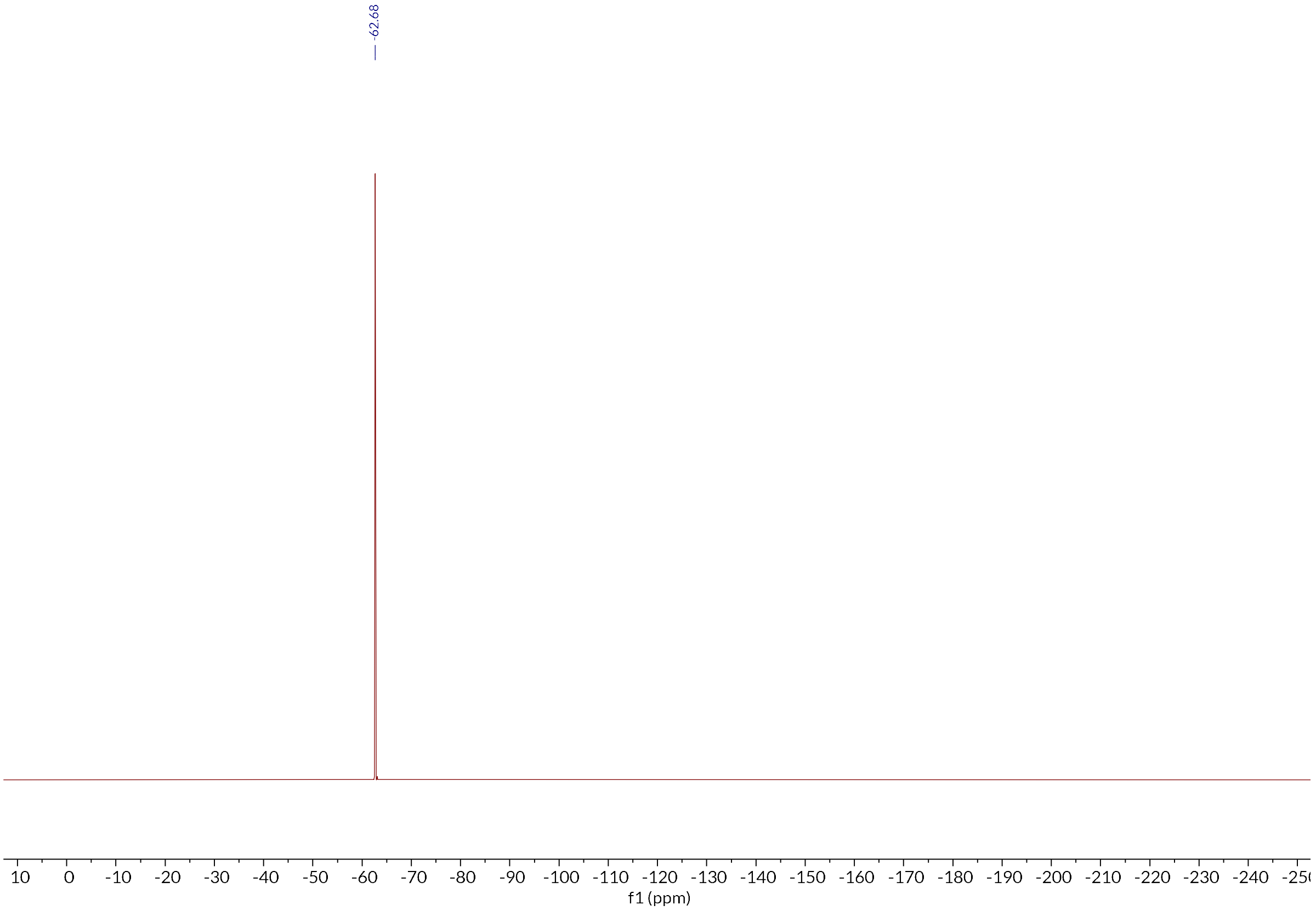
-62.68
10
0
-10
-20
-30
-40
-50
-60
-70
-80
-90
-100
-110
-120
-130
-140
-150
-160
-170
-180
-190
-200
-210
-220
-230
-240
f1 (ppm)

N-Cinnamylnaphthalen-1-amine (SI-34)

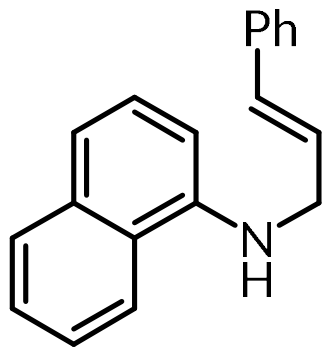

(SI-34)

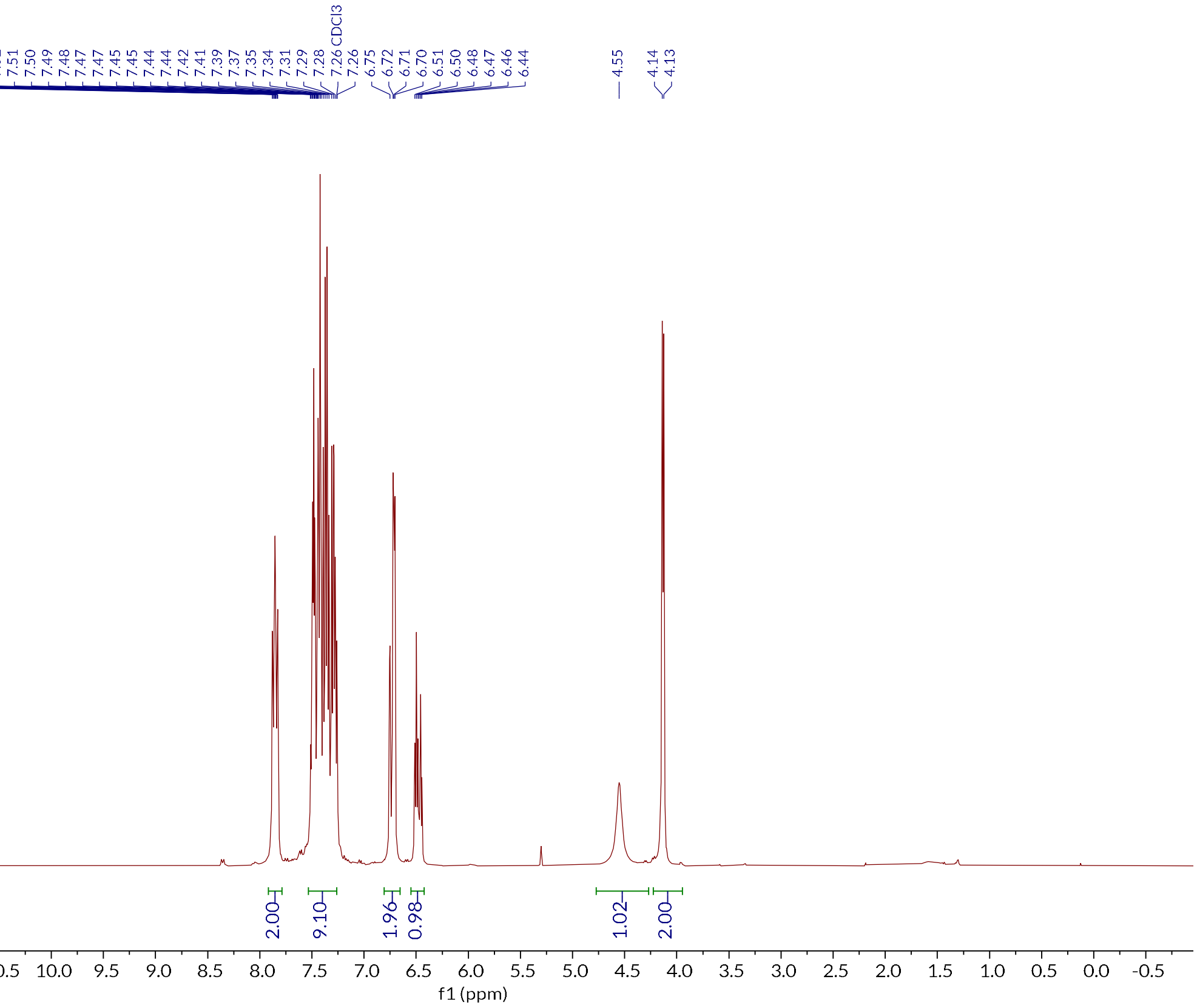

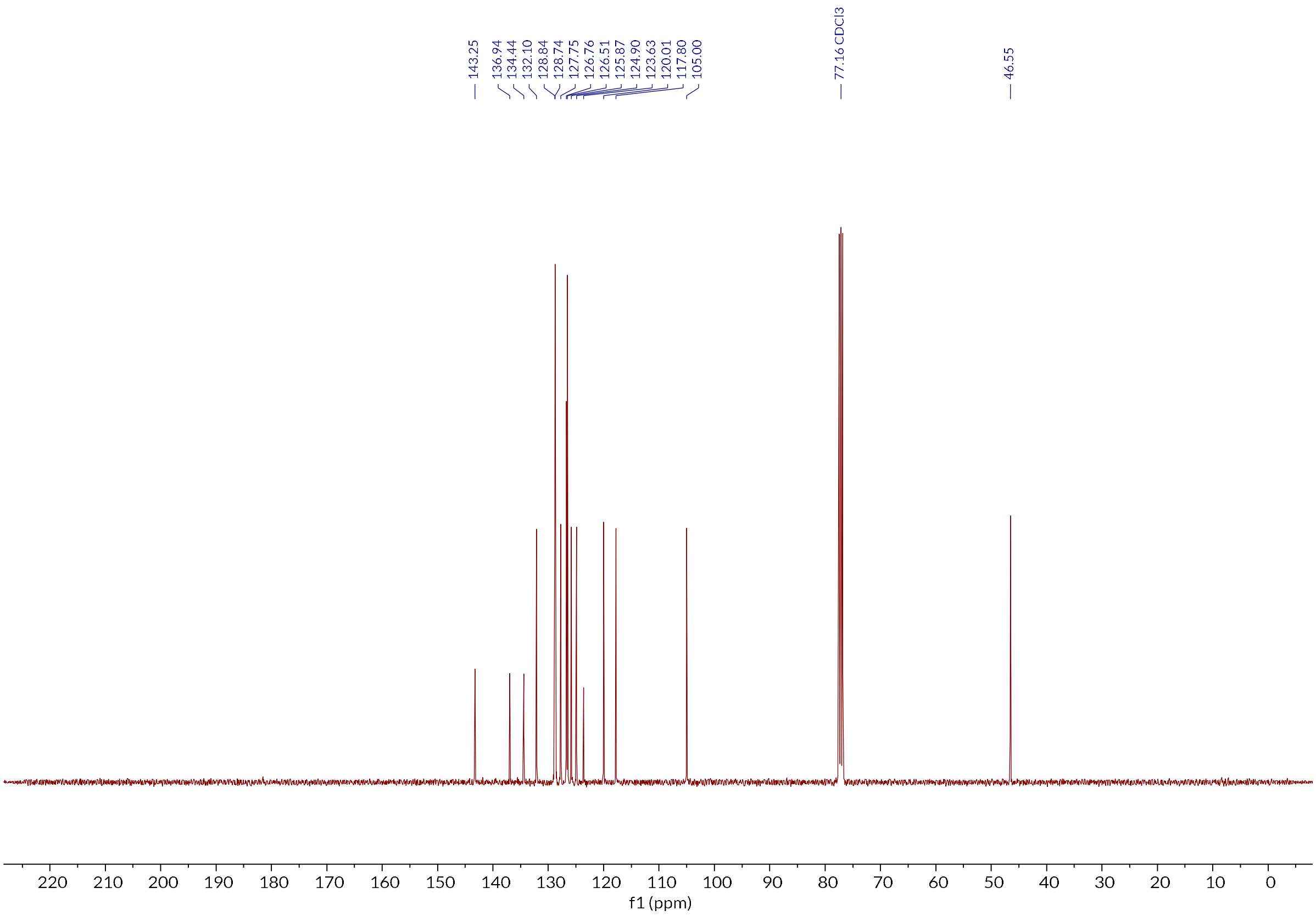

N-Benzyl-*N*-cinnamylnaphthalen-1-amine (1bp)

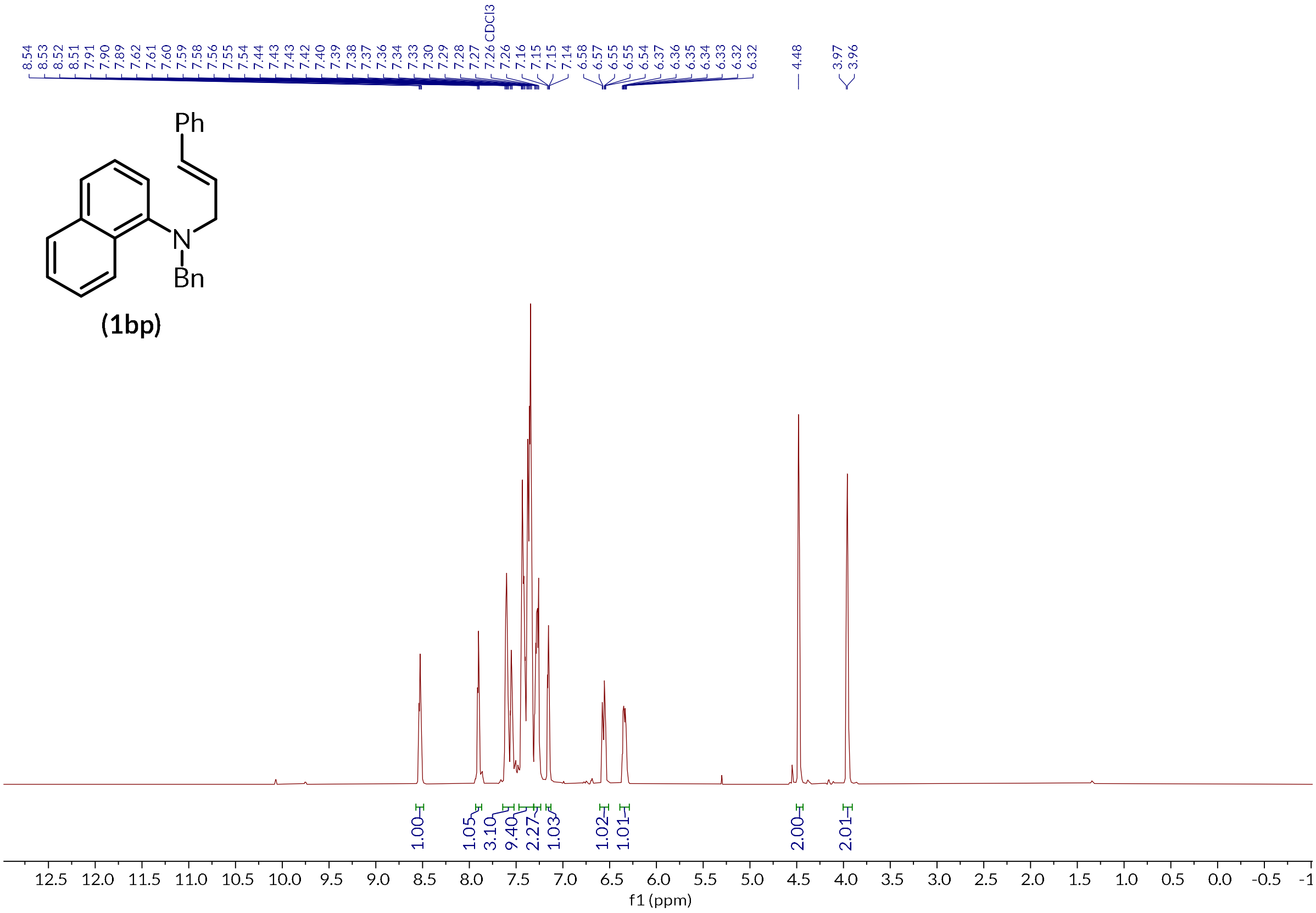

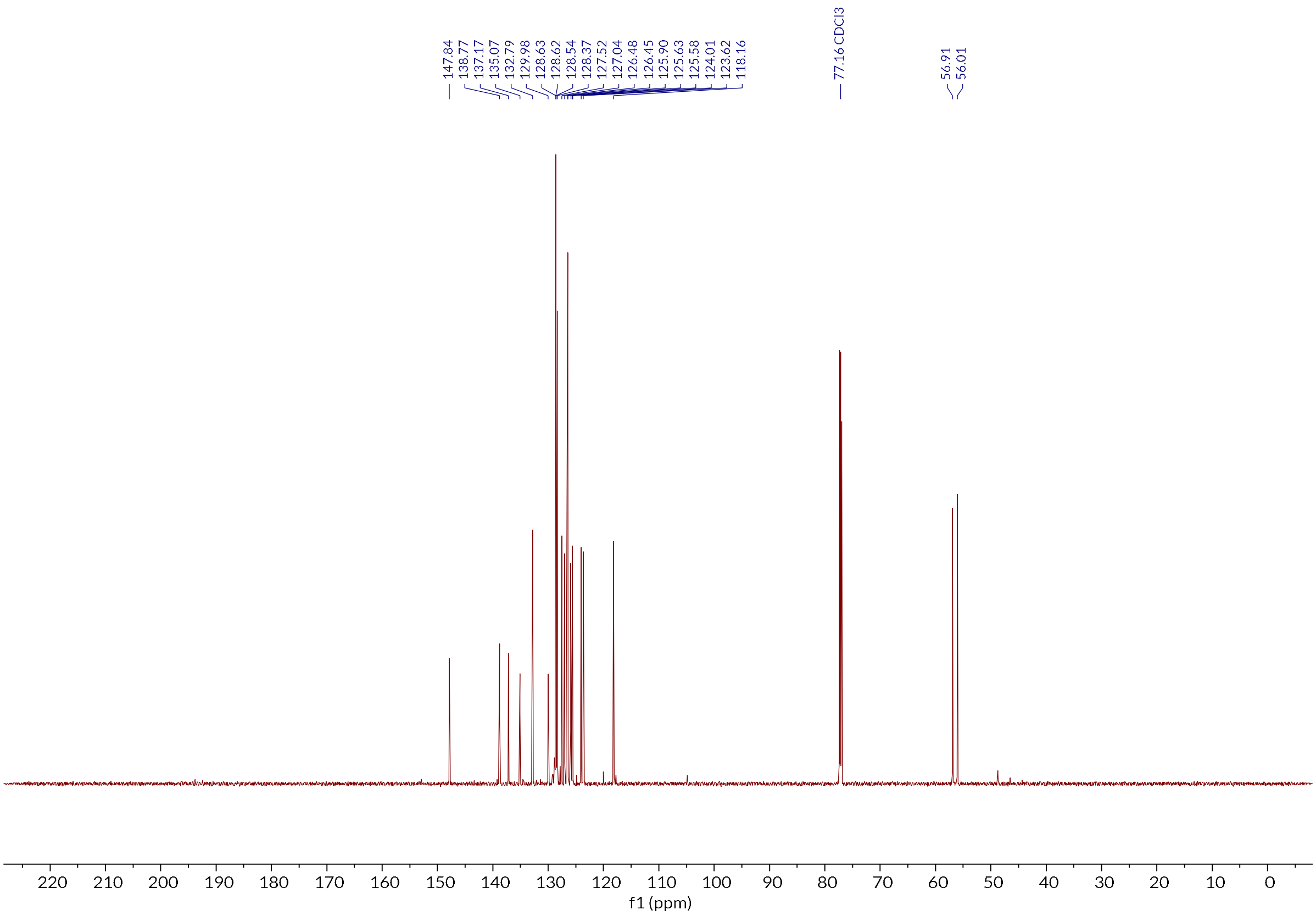

1-Cinnamylindoline (1bq)

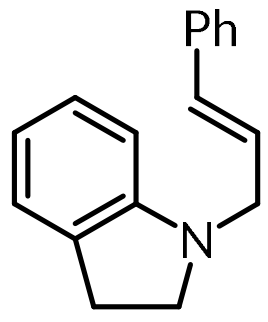

(1bq)

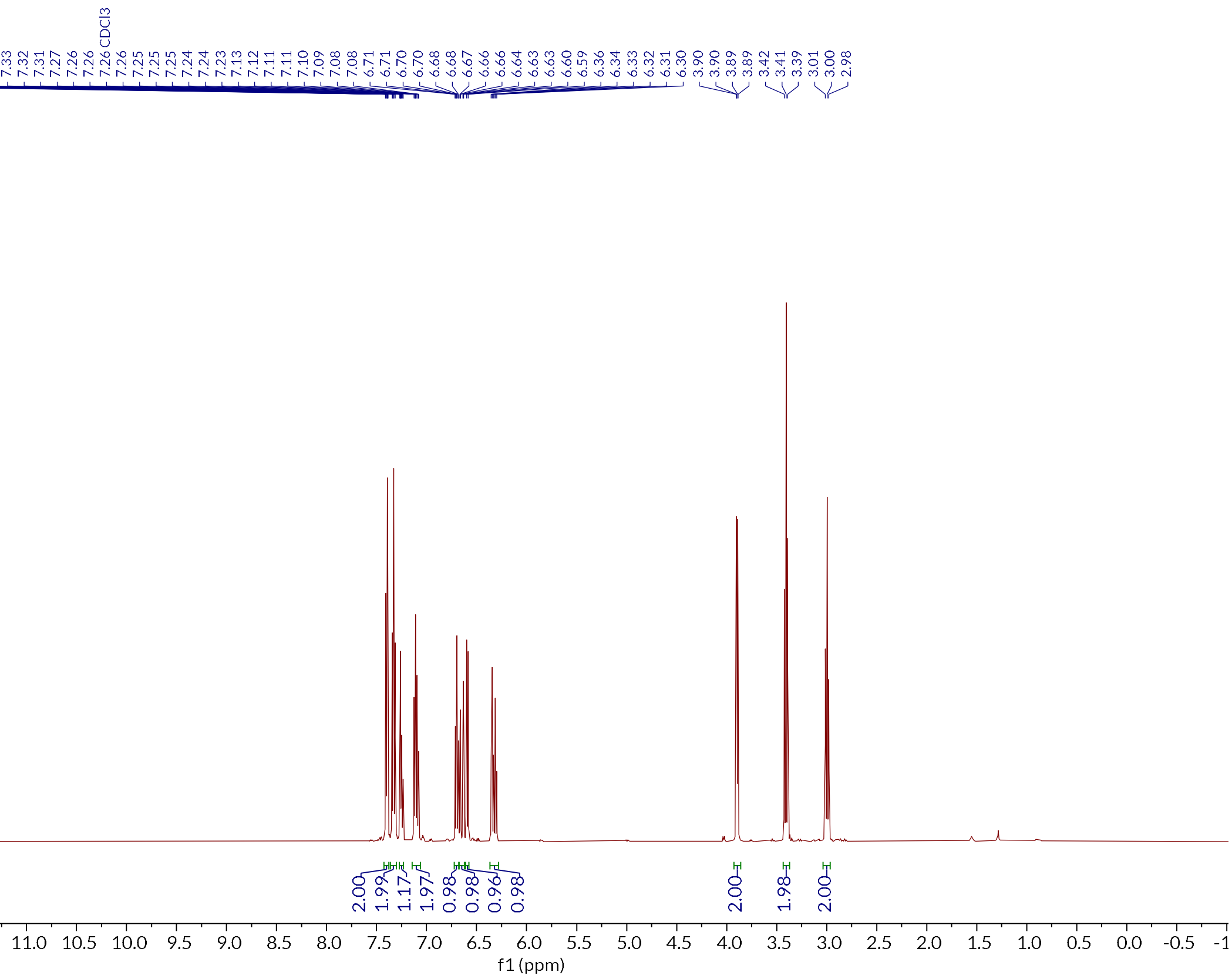

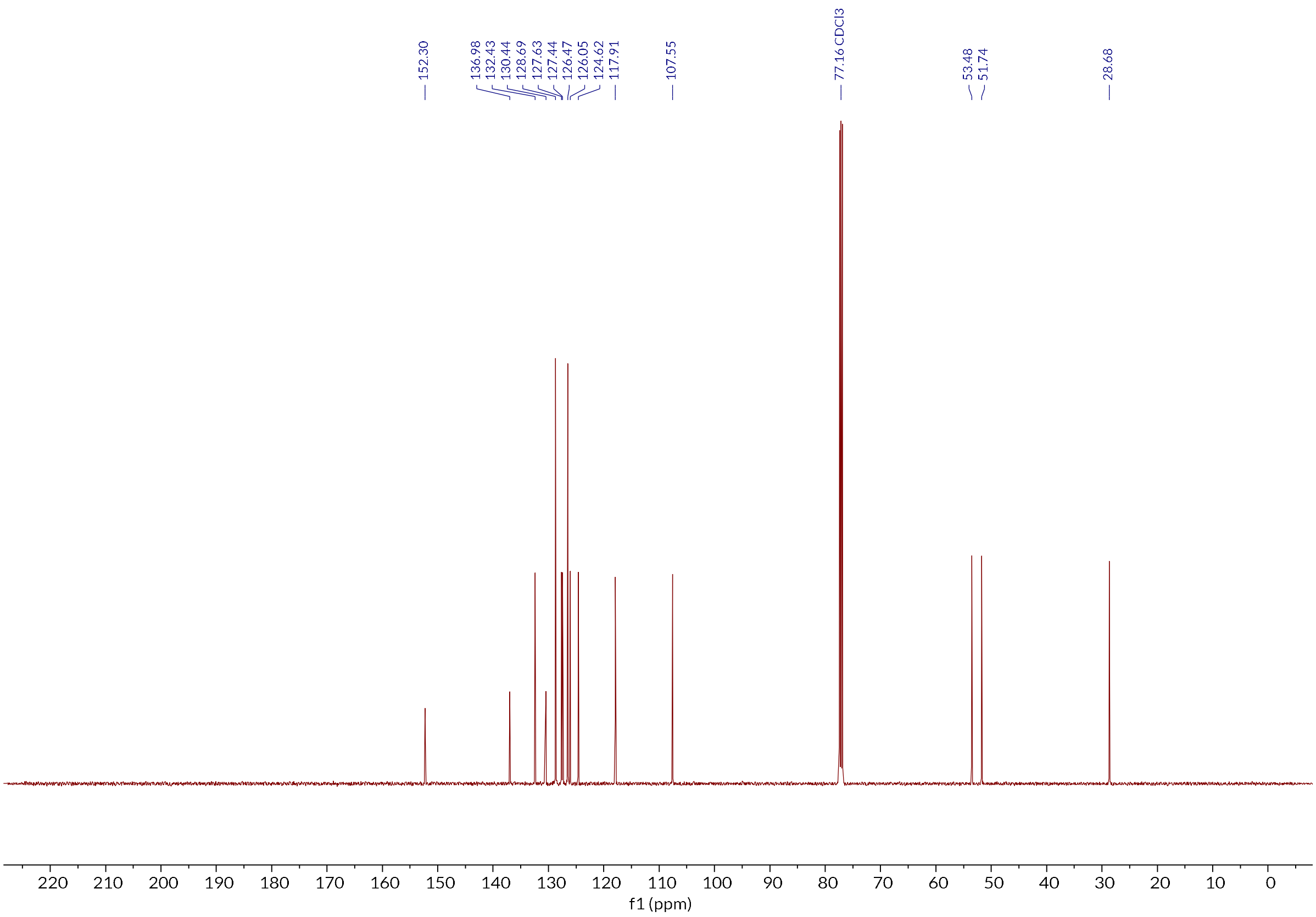

7-Chloro-1-cinnamyl-1,2,3,4-tetrahydro-5*H*-benzo[*b*]azepin-5-one (1br)

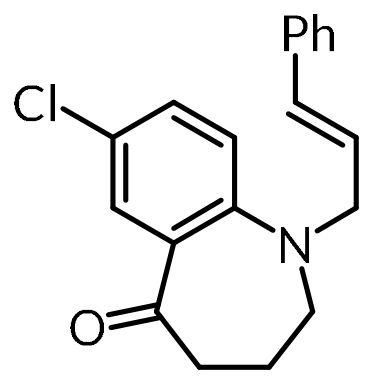

(1br)

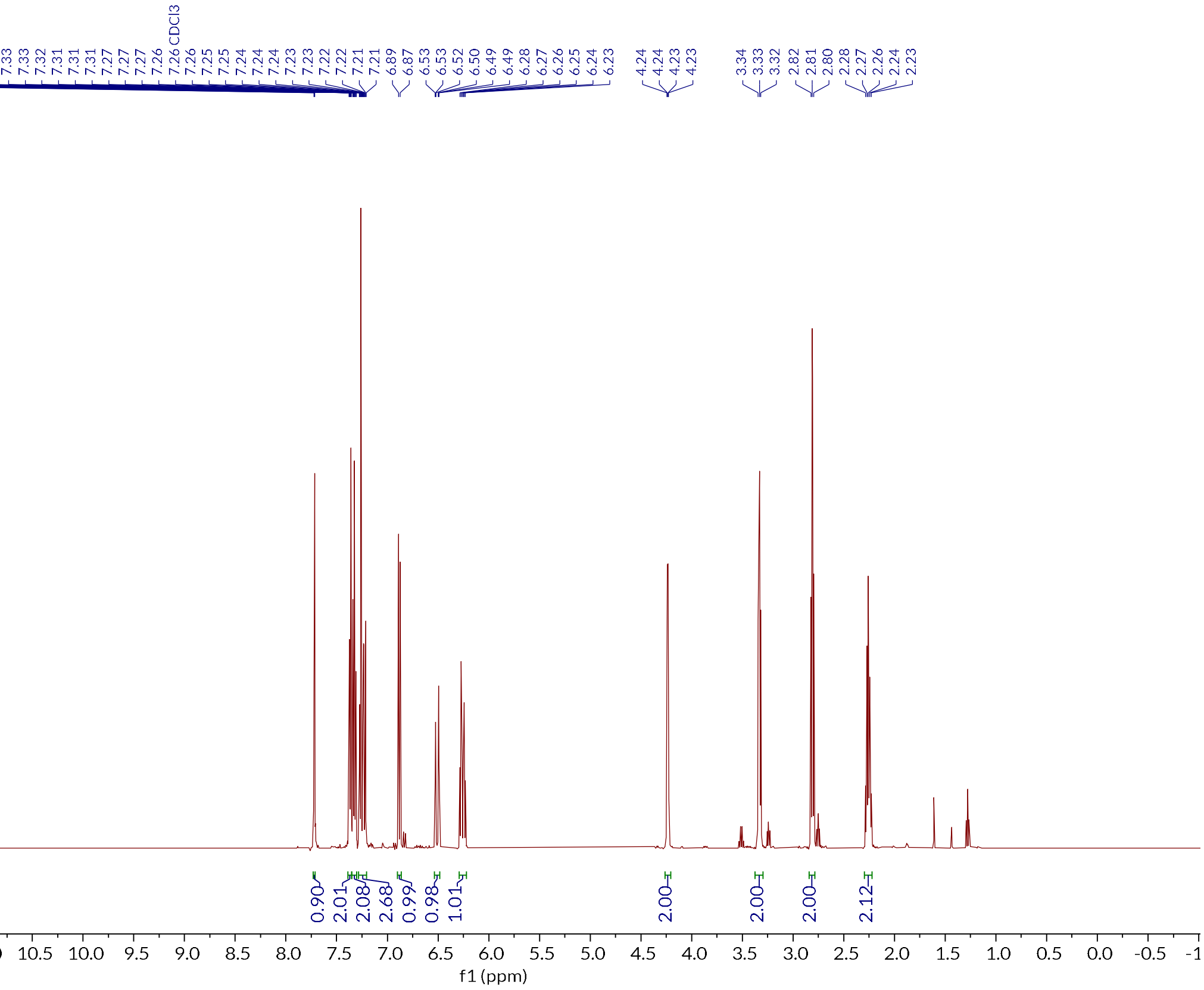

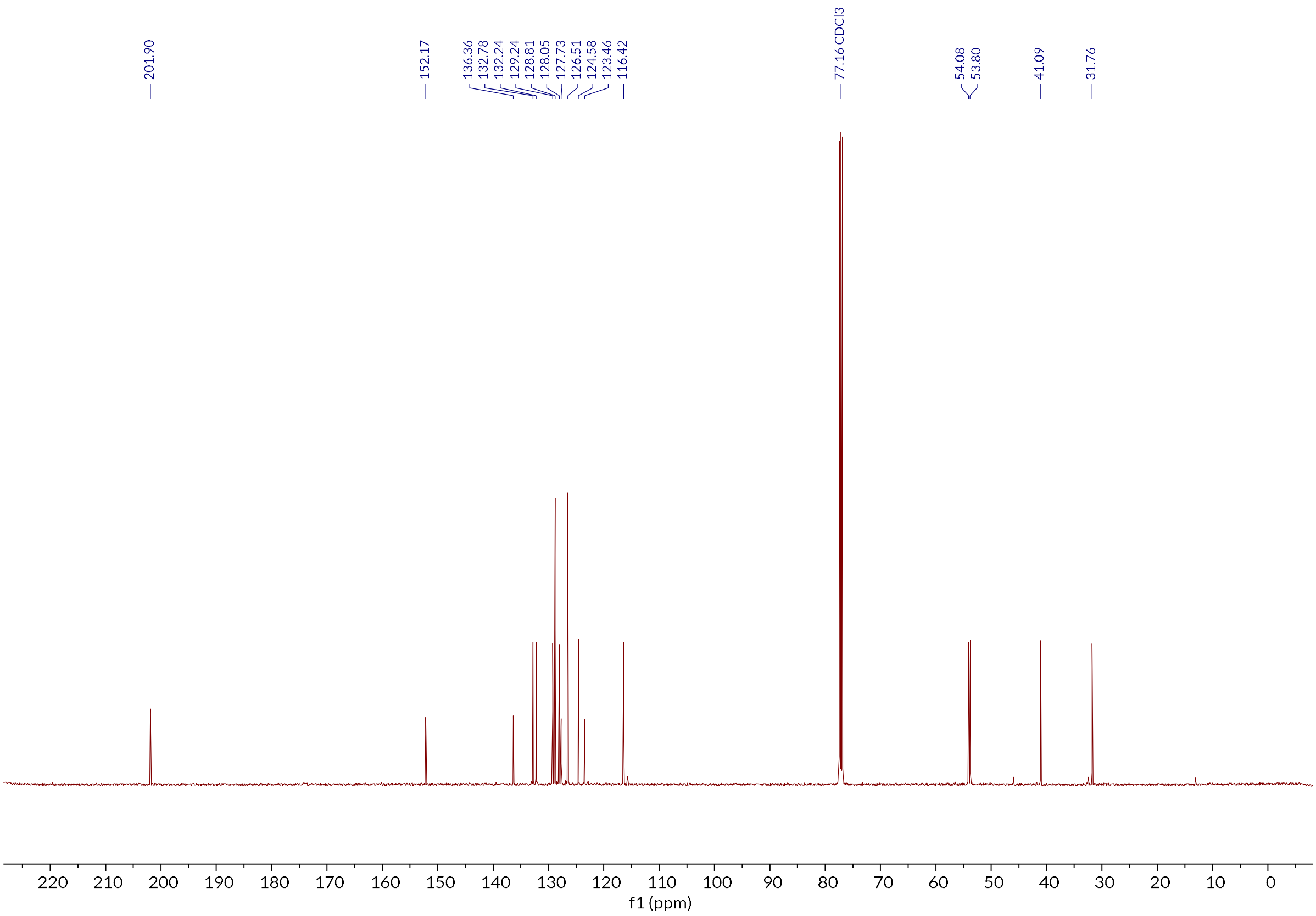

N-Cinnamyl-2-methylaniline (3b)

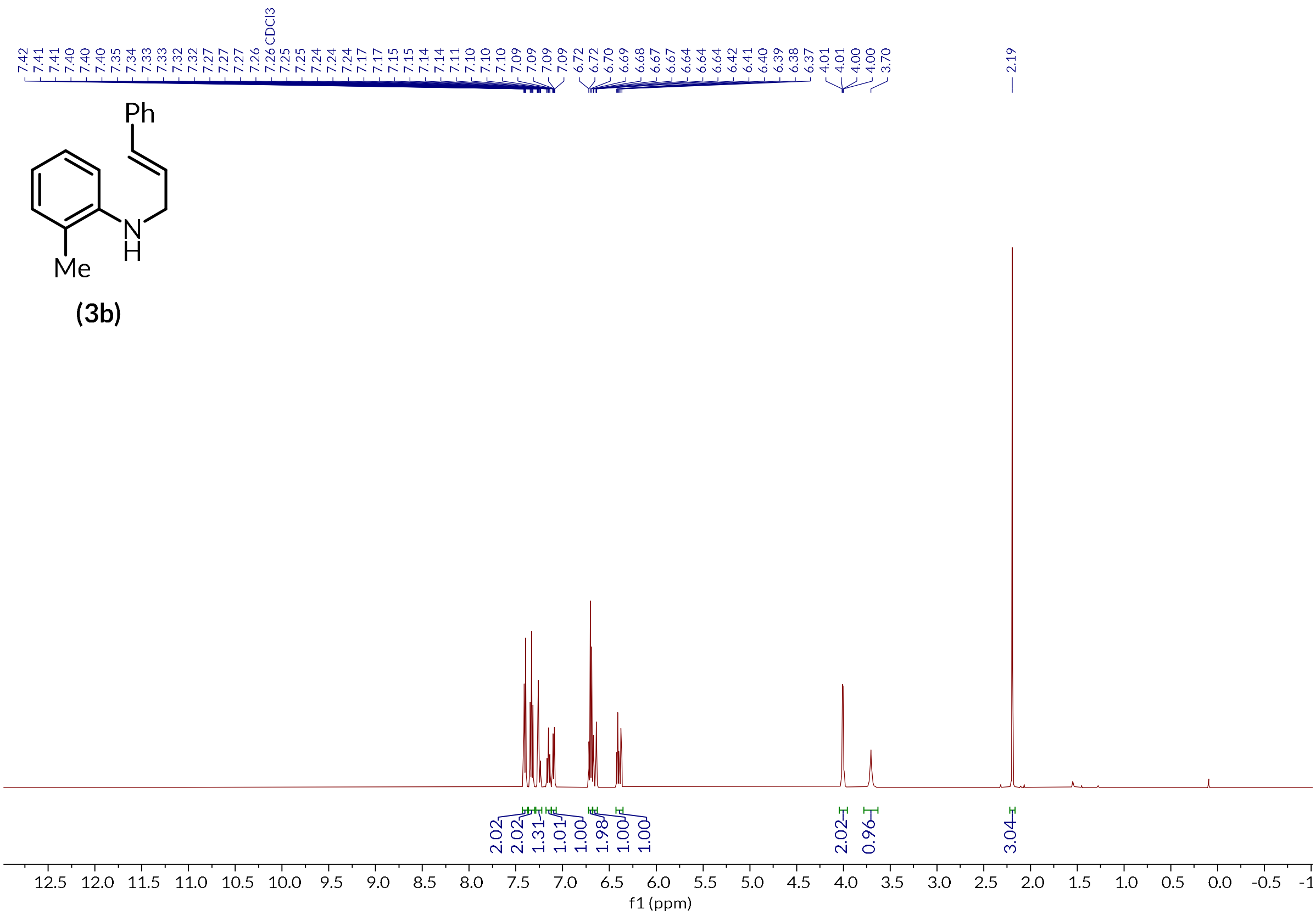

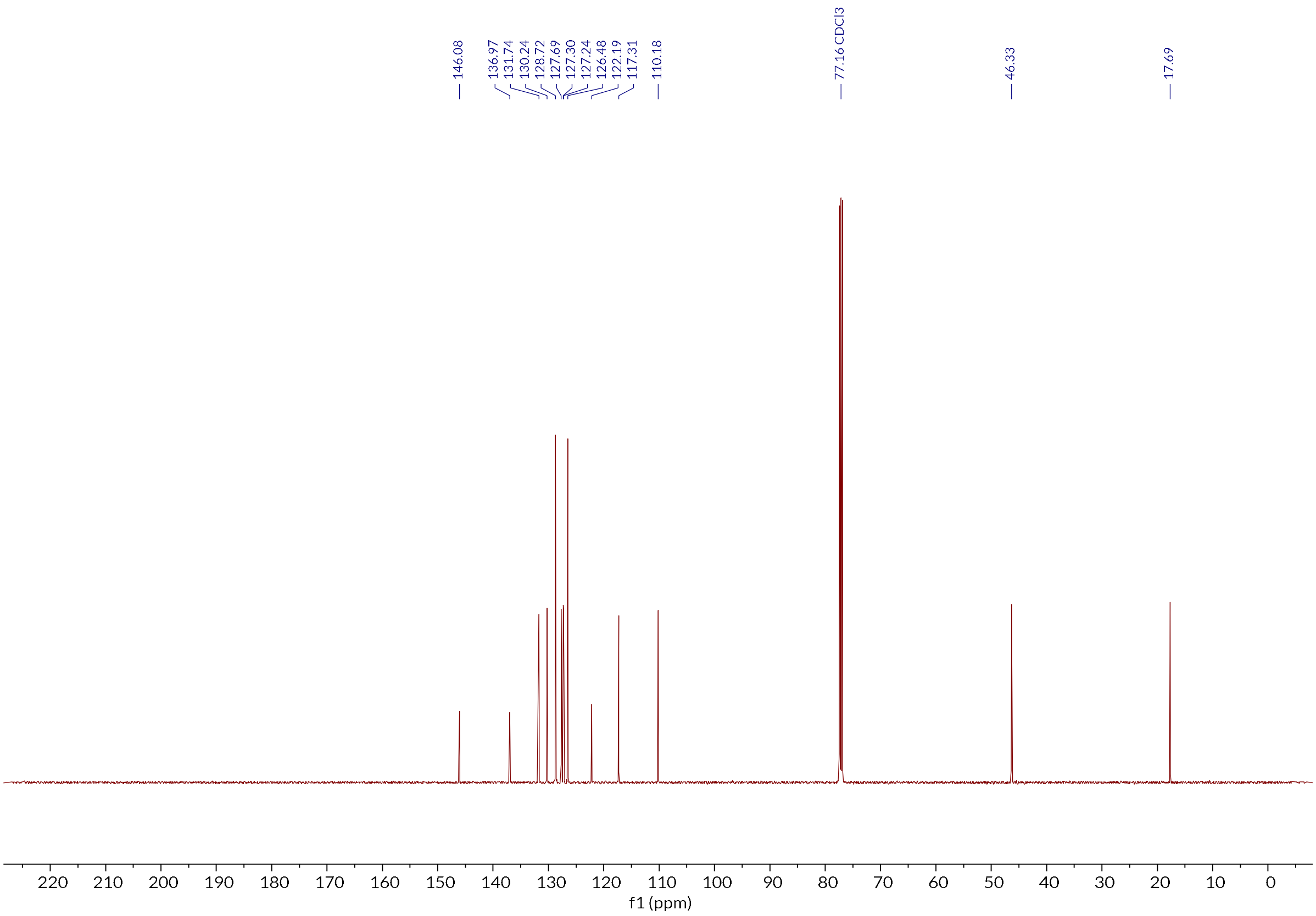

2-Bromo-*N*-cinnamylaniline (3c)

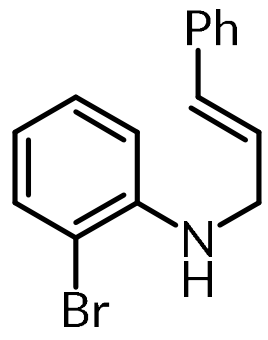

(3c)

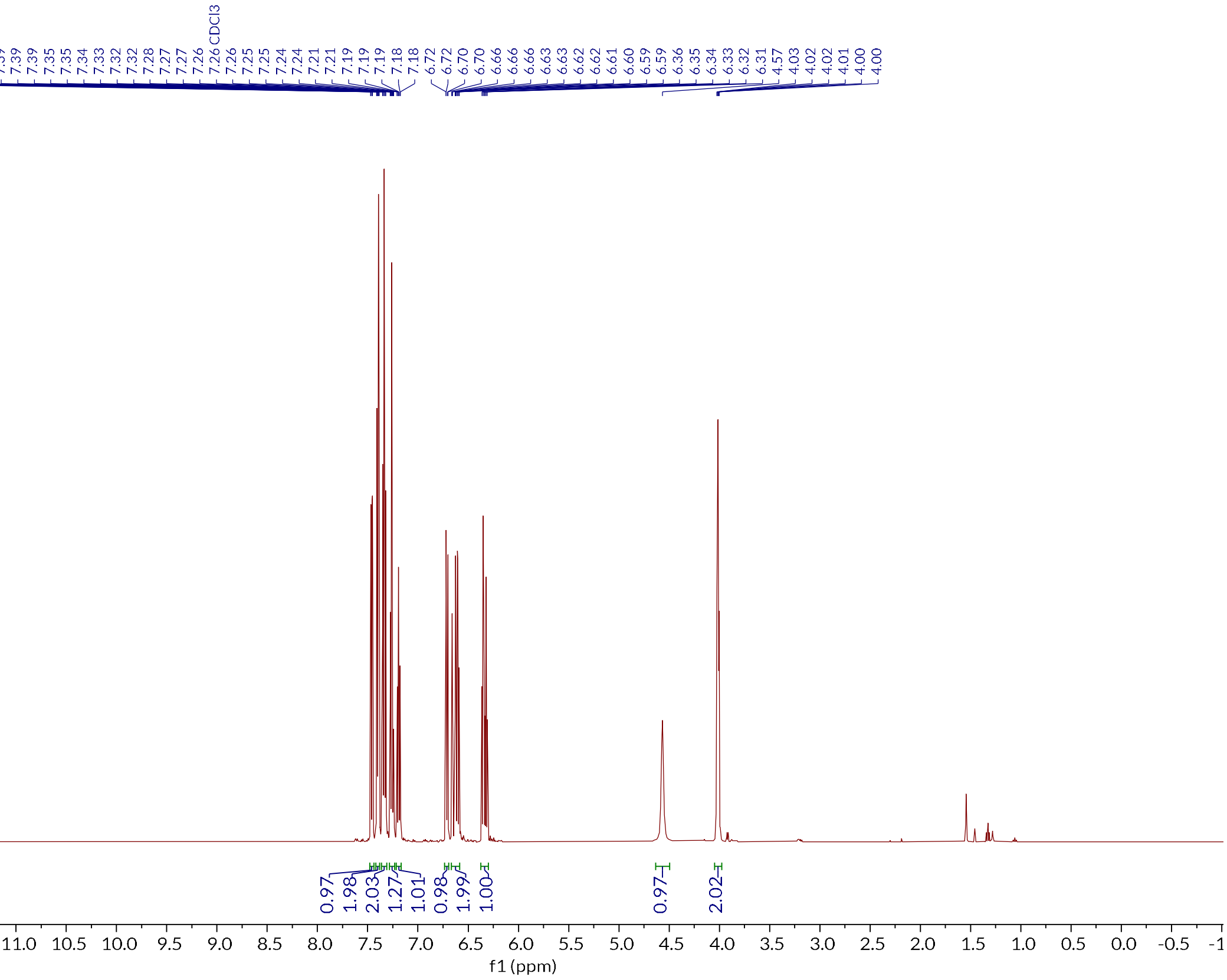

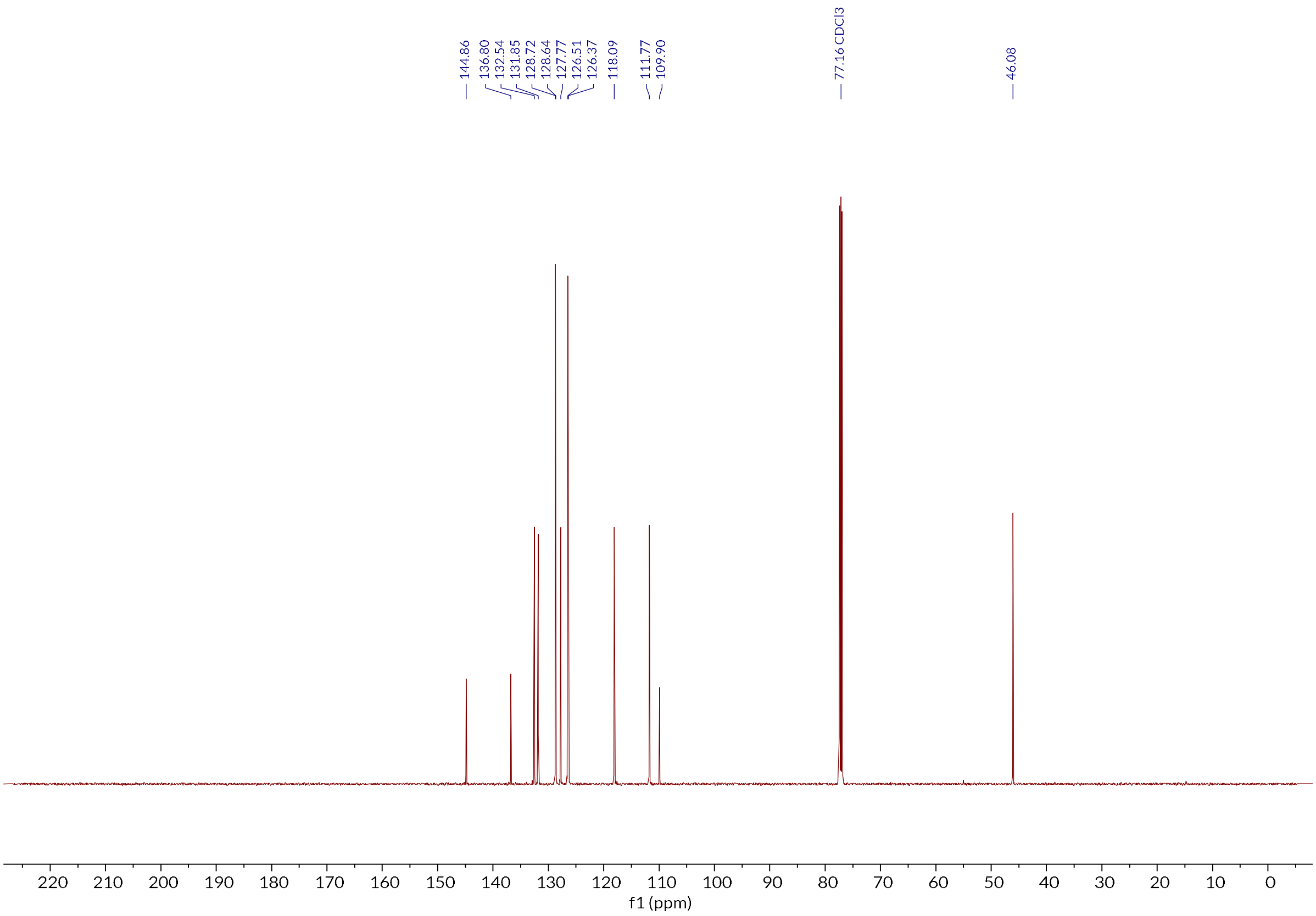

N-Cinnamyl-2-(methylthio)aniline (3d)

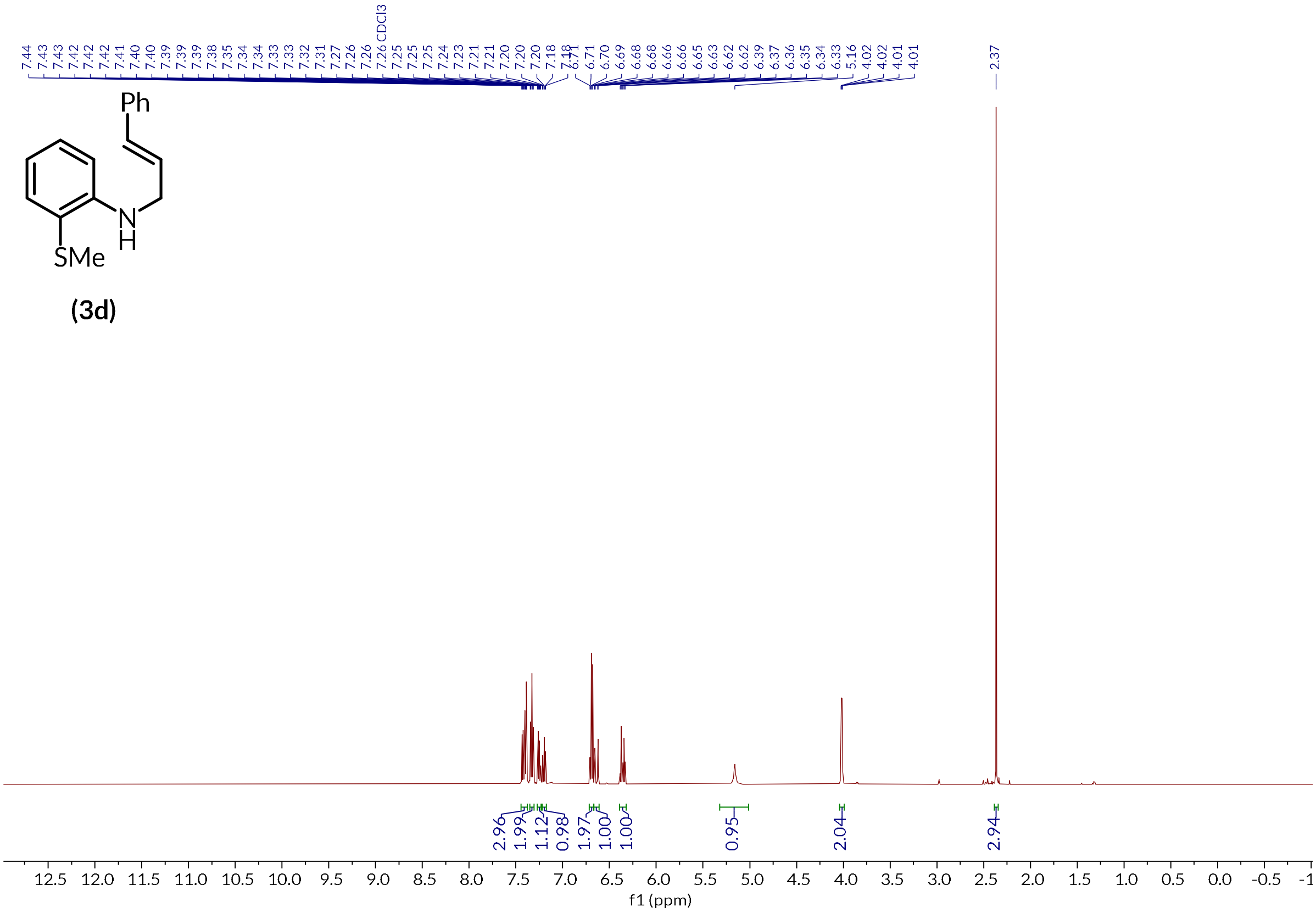

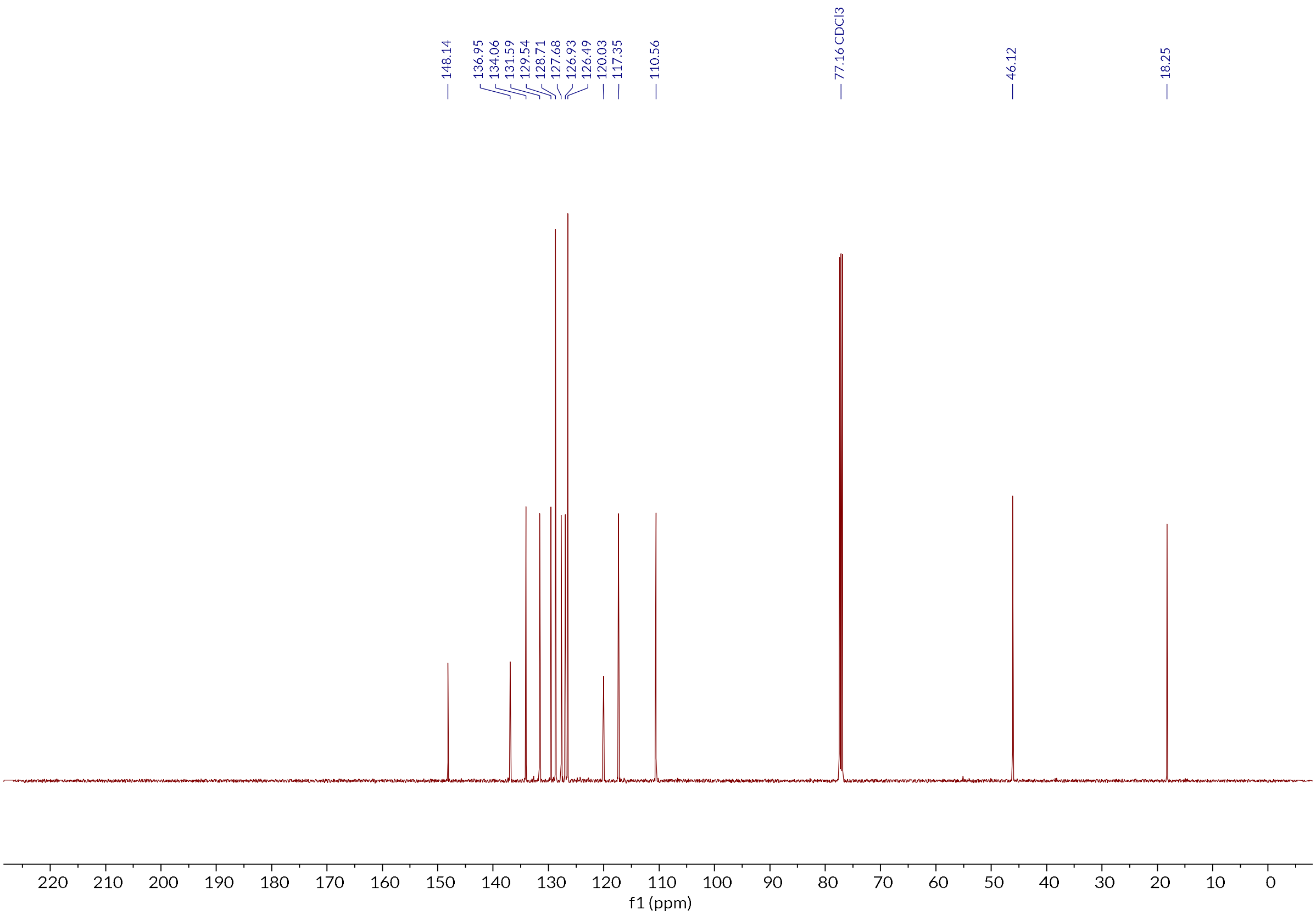

N-Cinnamyl-2-methoxyaniline (3e)

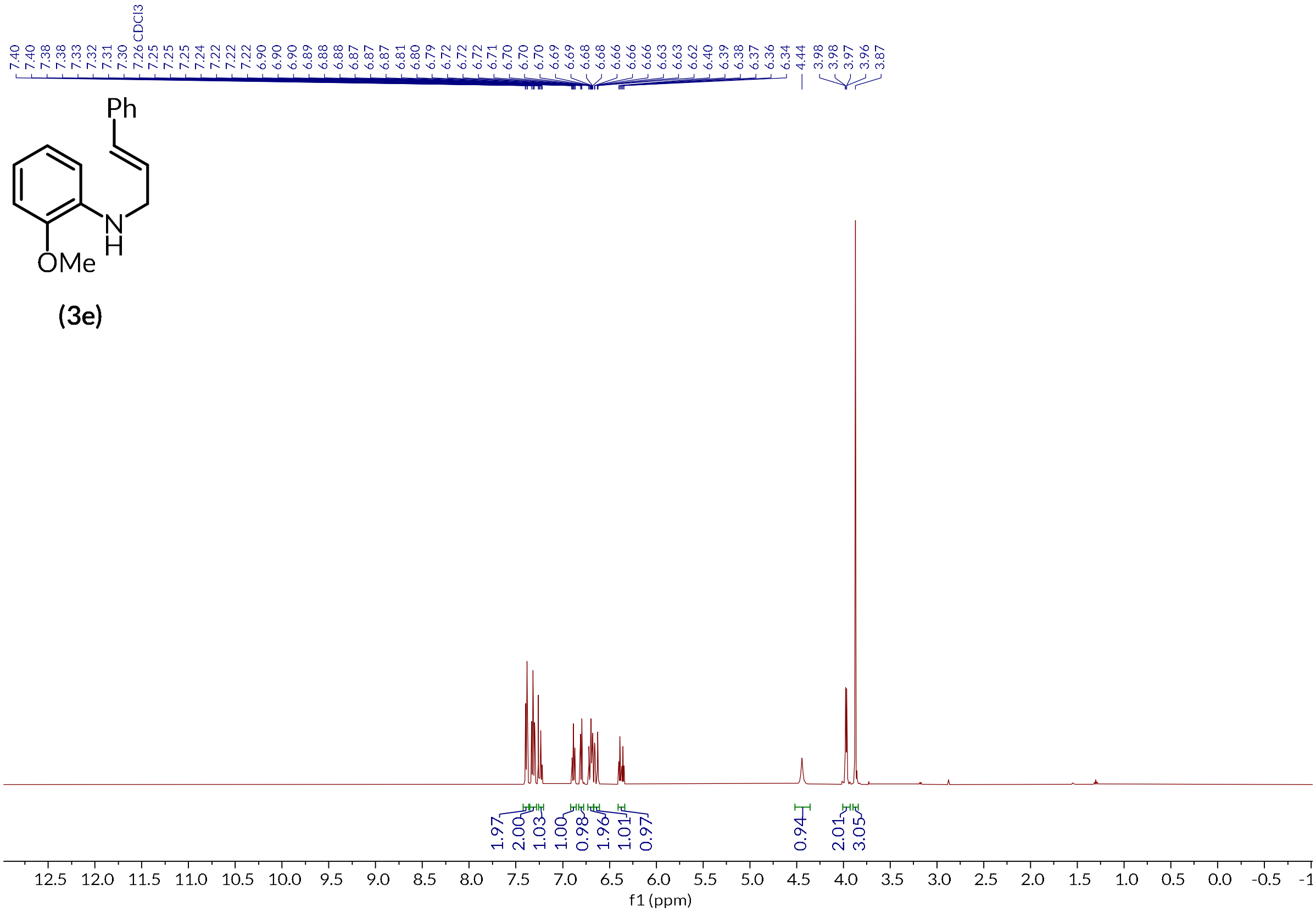

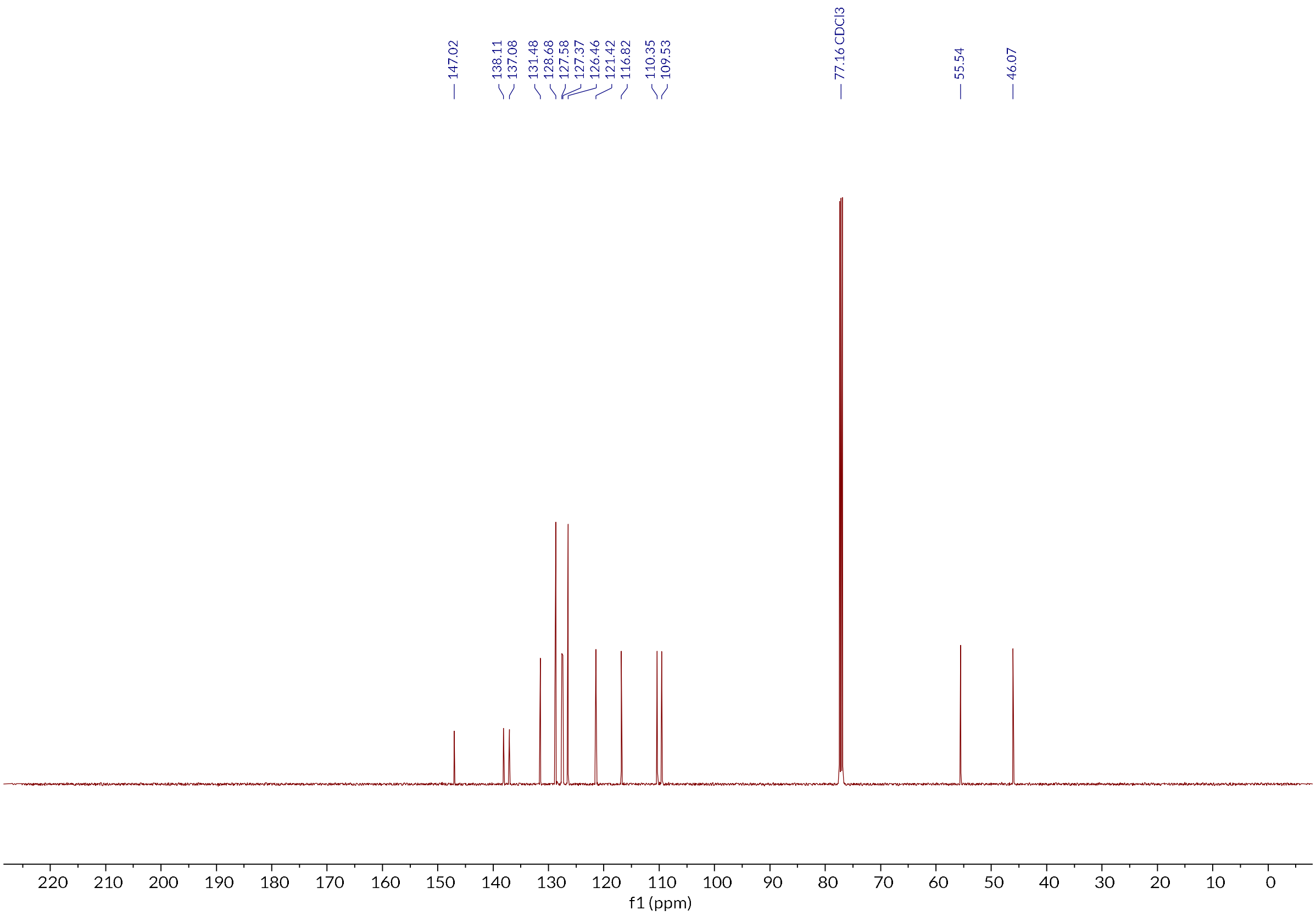

N-Cinnamyl-4-methoxy-2-methylaniline (3f)

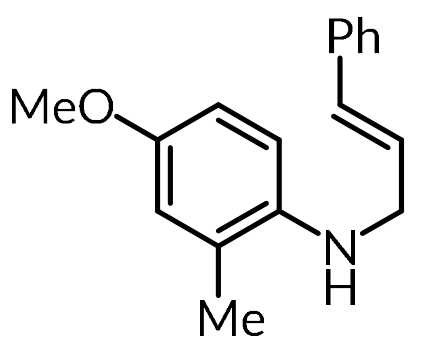

(3f)

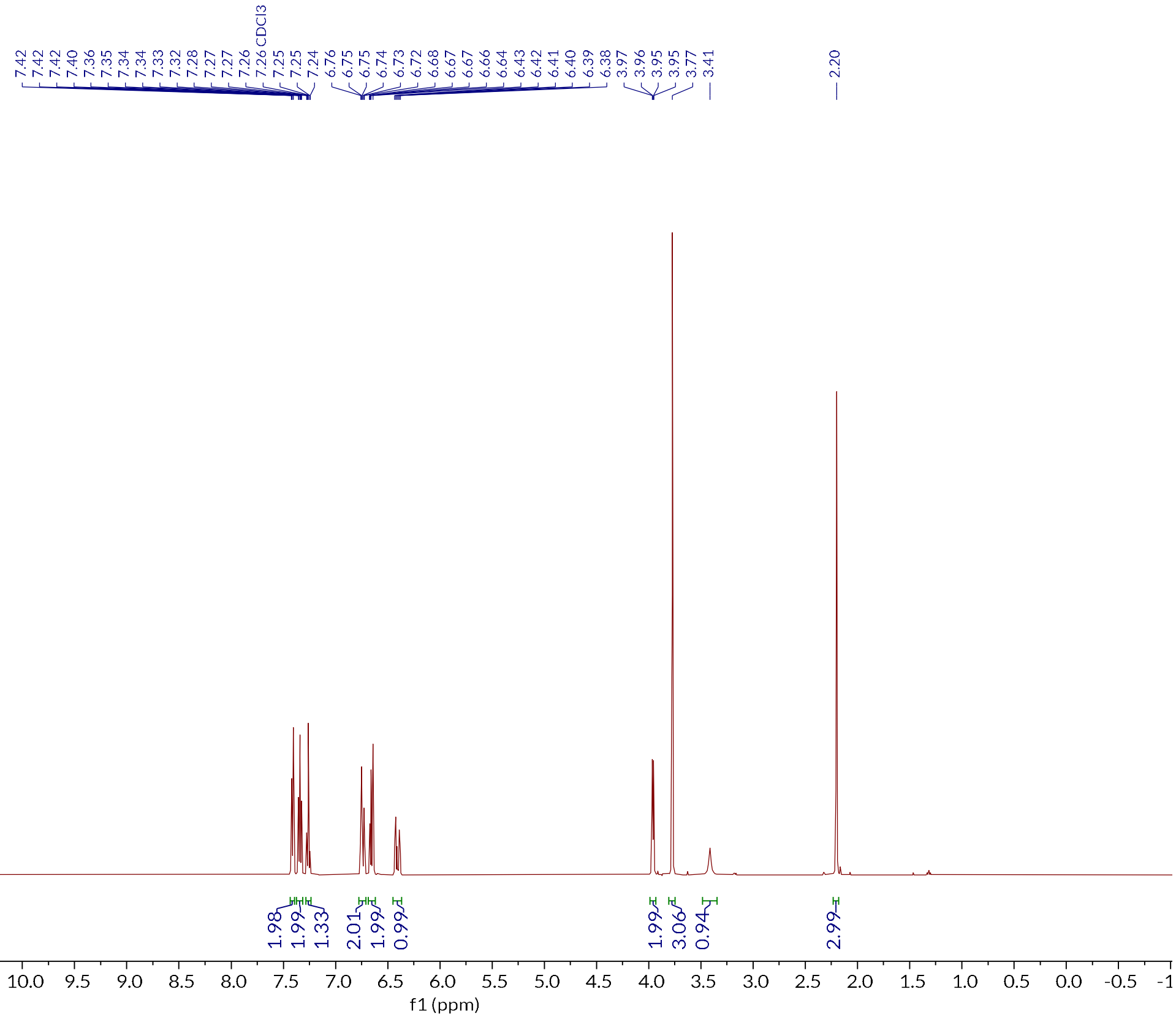

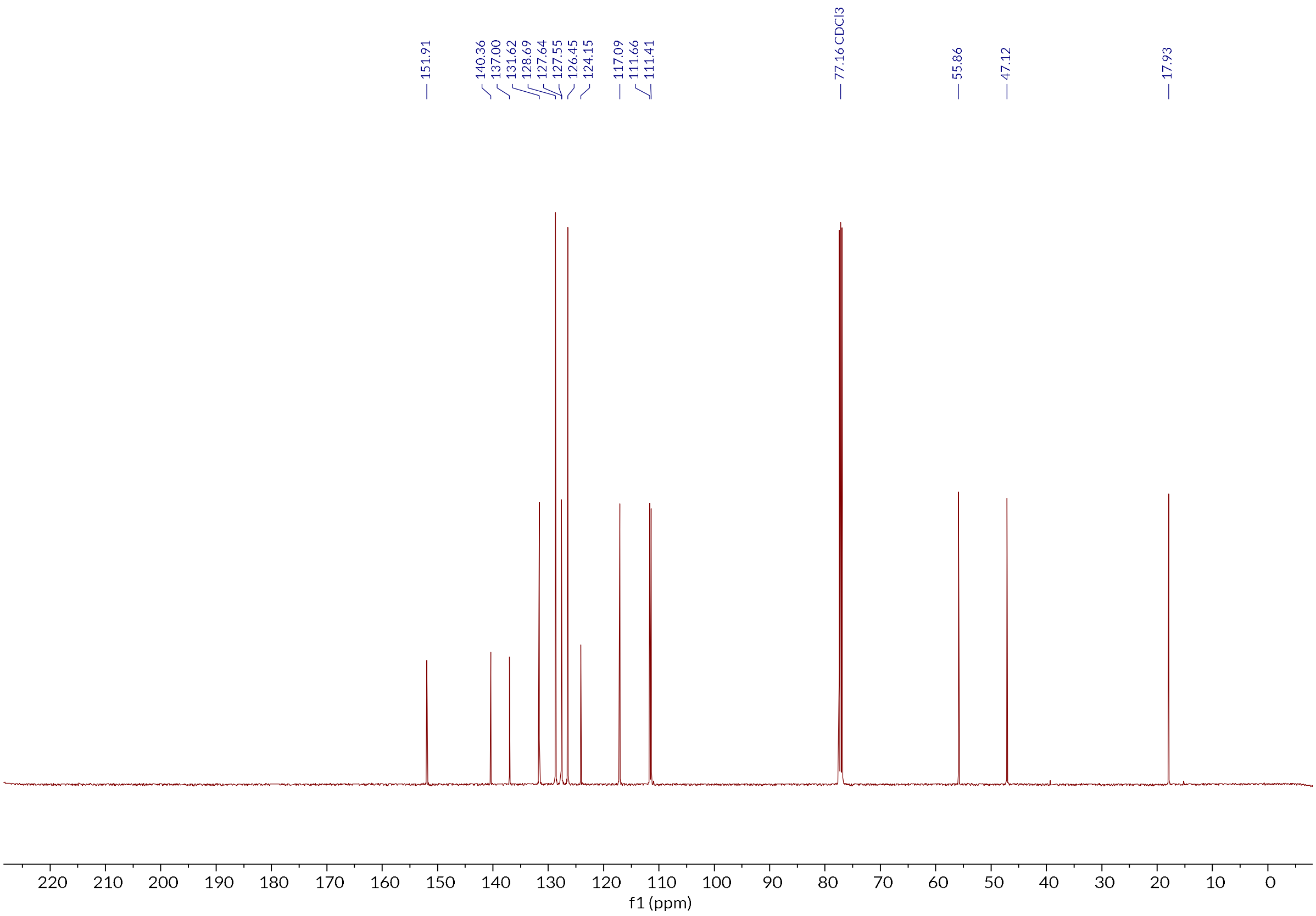

Ethyl (*E*)-4-(cinnamyl(phenyl)amino)but-2-enoate (5)

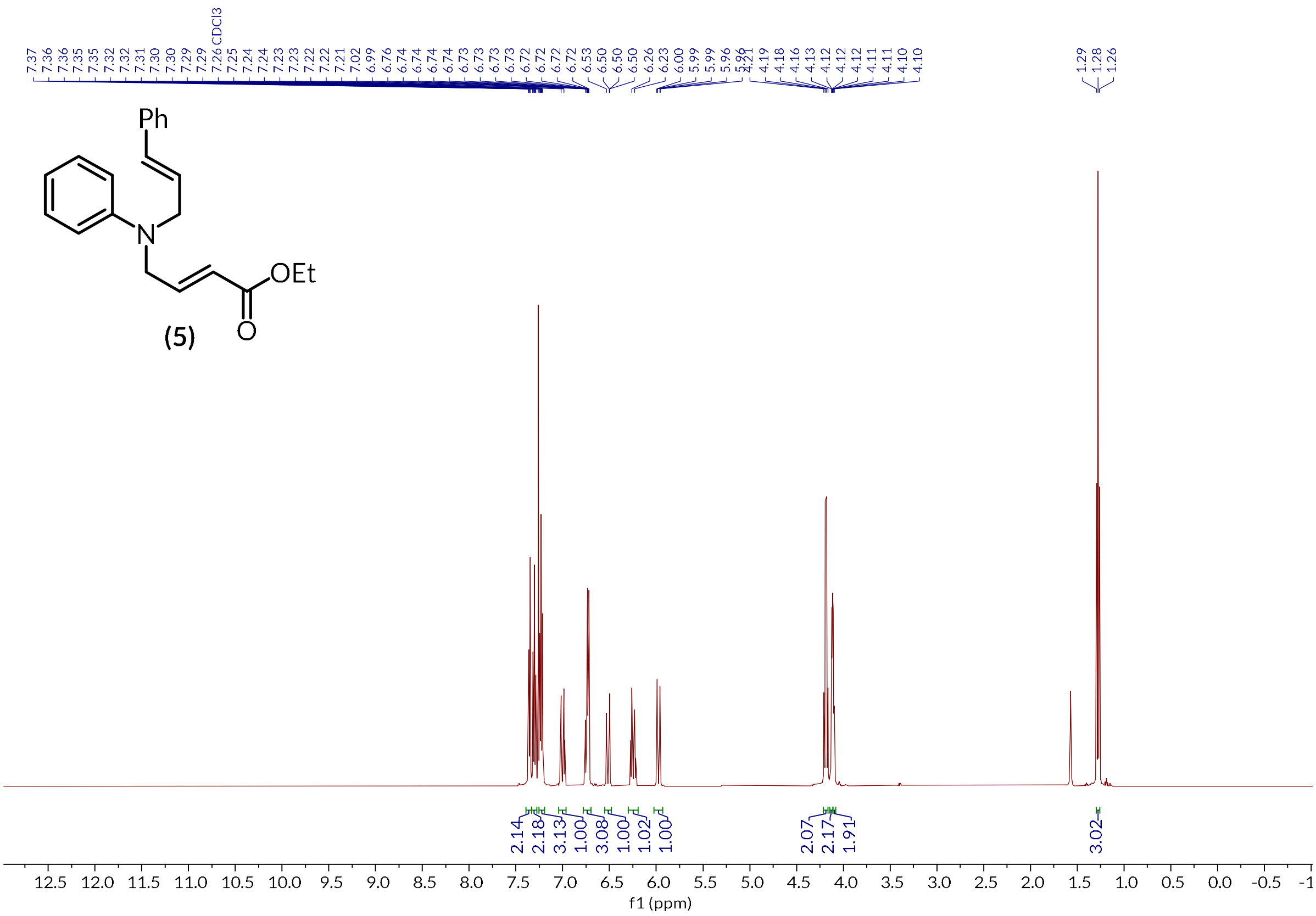

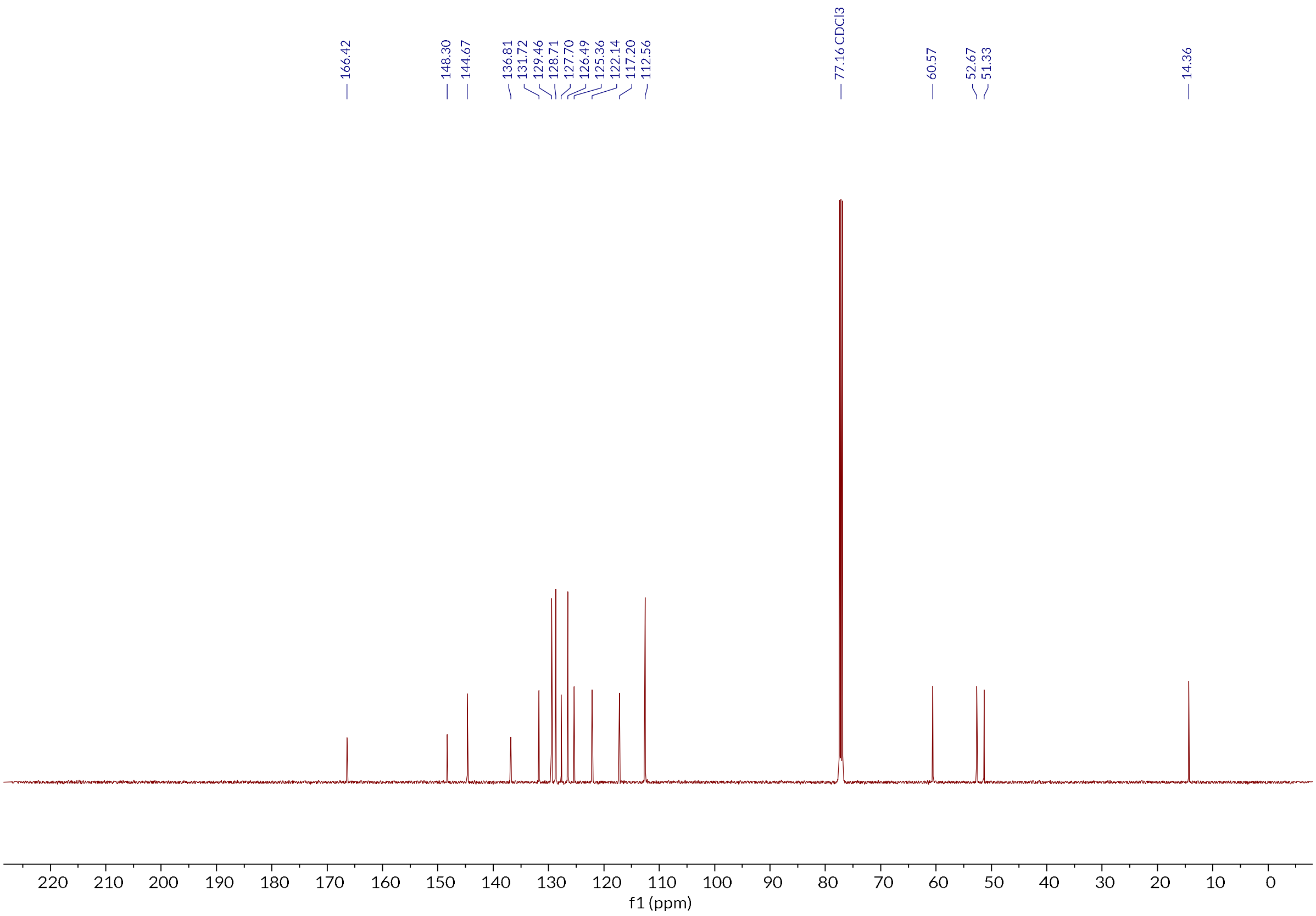

N-Cinnamyl-4-methoxy-*N*-(4-(trifluoromethyl)phenyl)aniline (7)

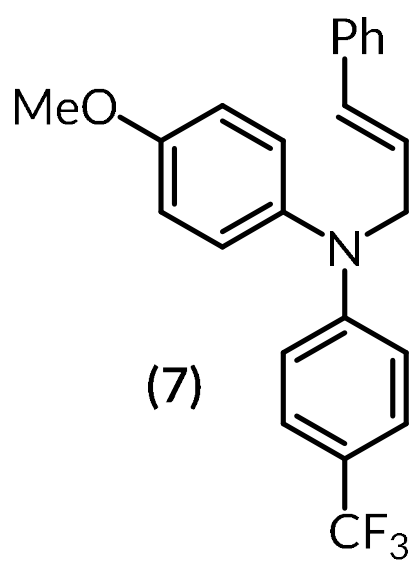

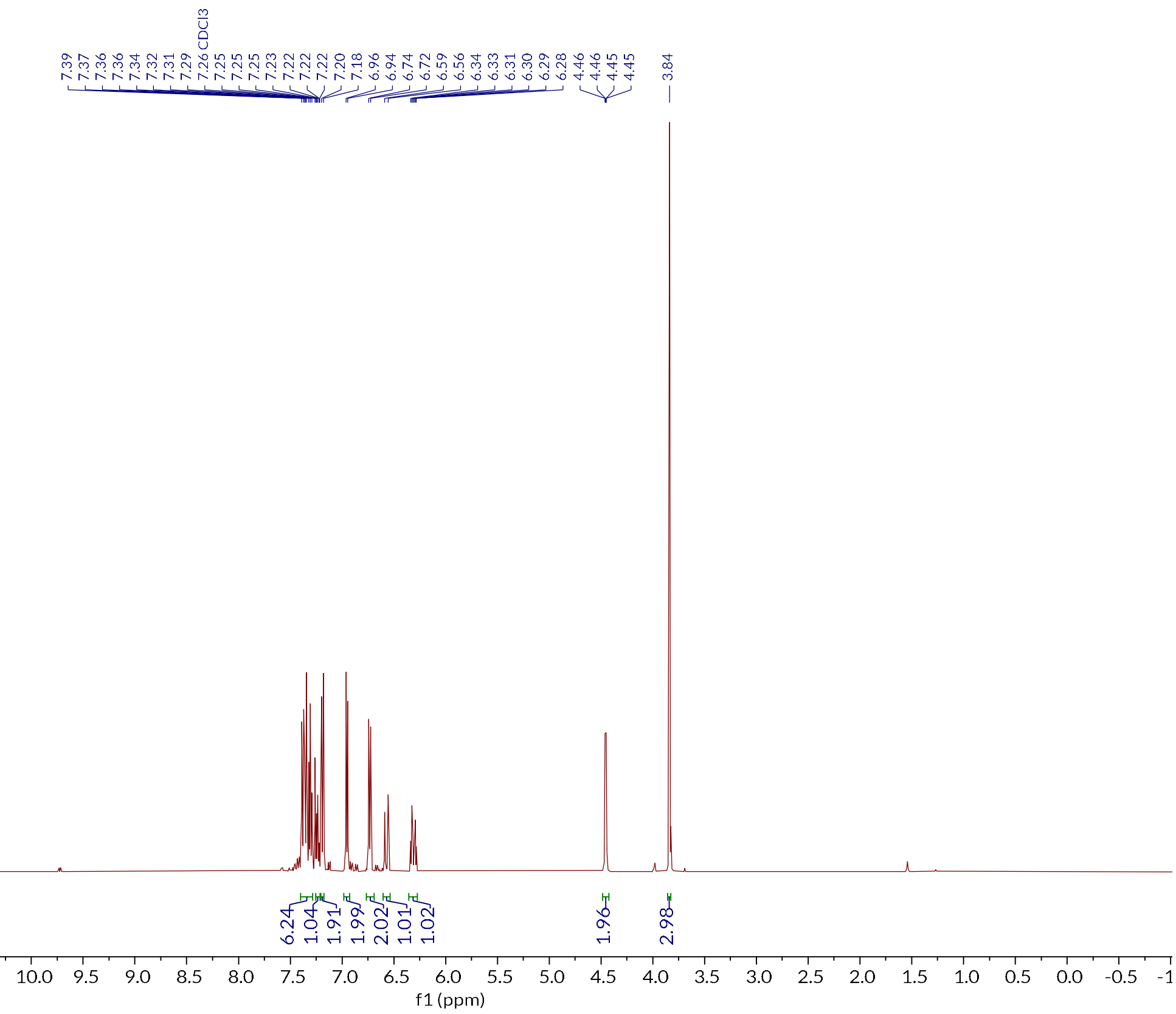

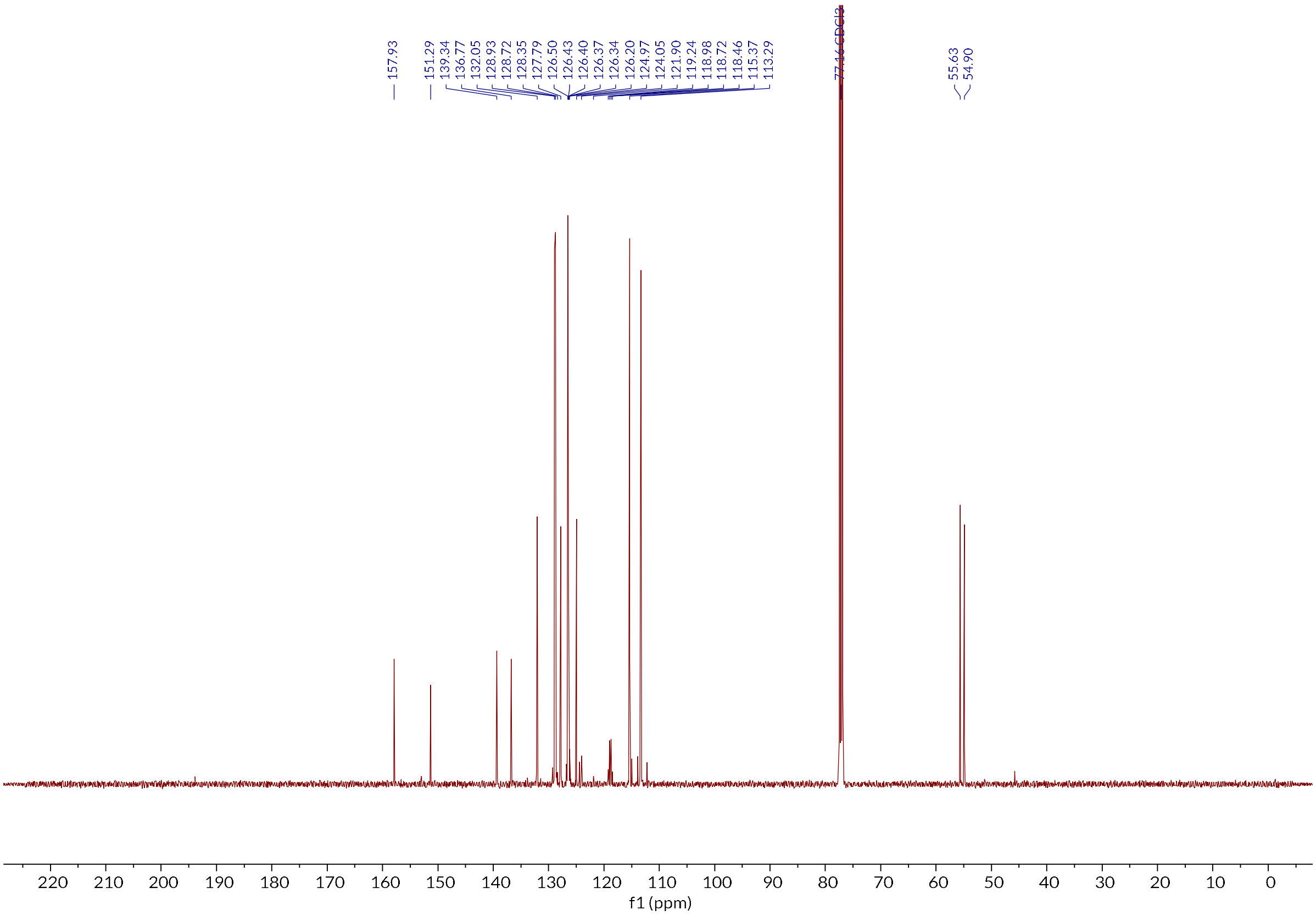

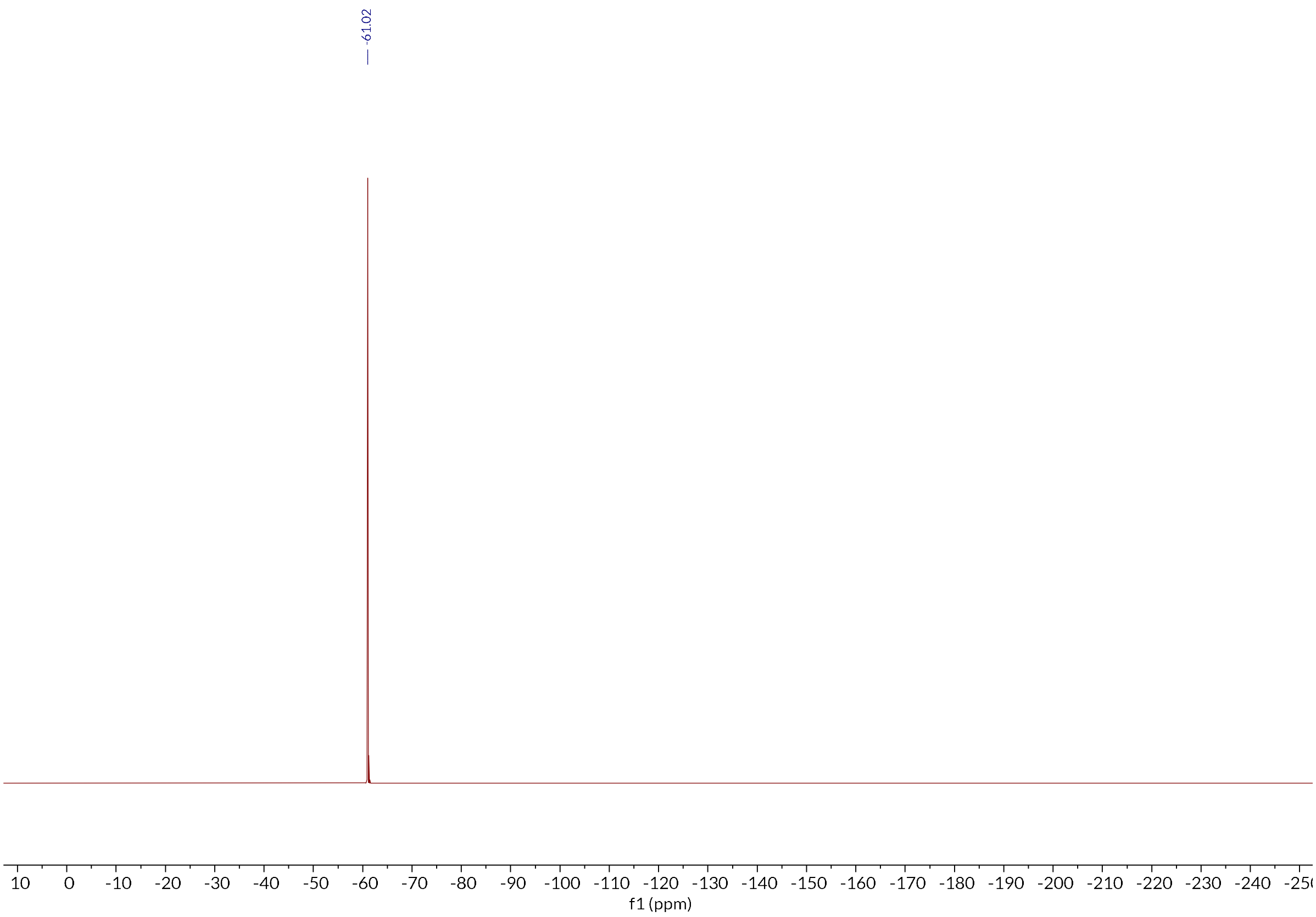
-61.02
10
0
-10
-20
-30
-40
-50
-60
-70
-80
-90
-100
-110
-120
-130
-140
-150
-160
-170
-180
-190
-200
-210
-220
-230
-240
f1 (ppm)

(±)-1-Benzyl-4-phenyl-1,2,3,4-tetrahydroquinoline (2aa)

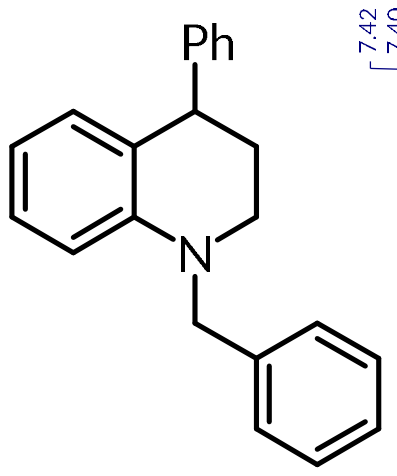

(2aa)

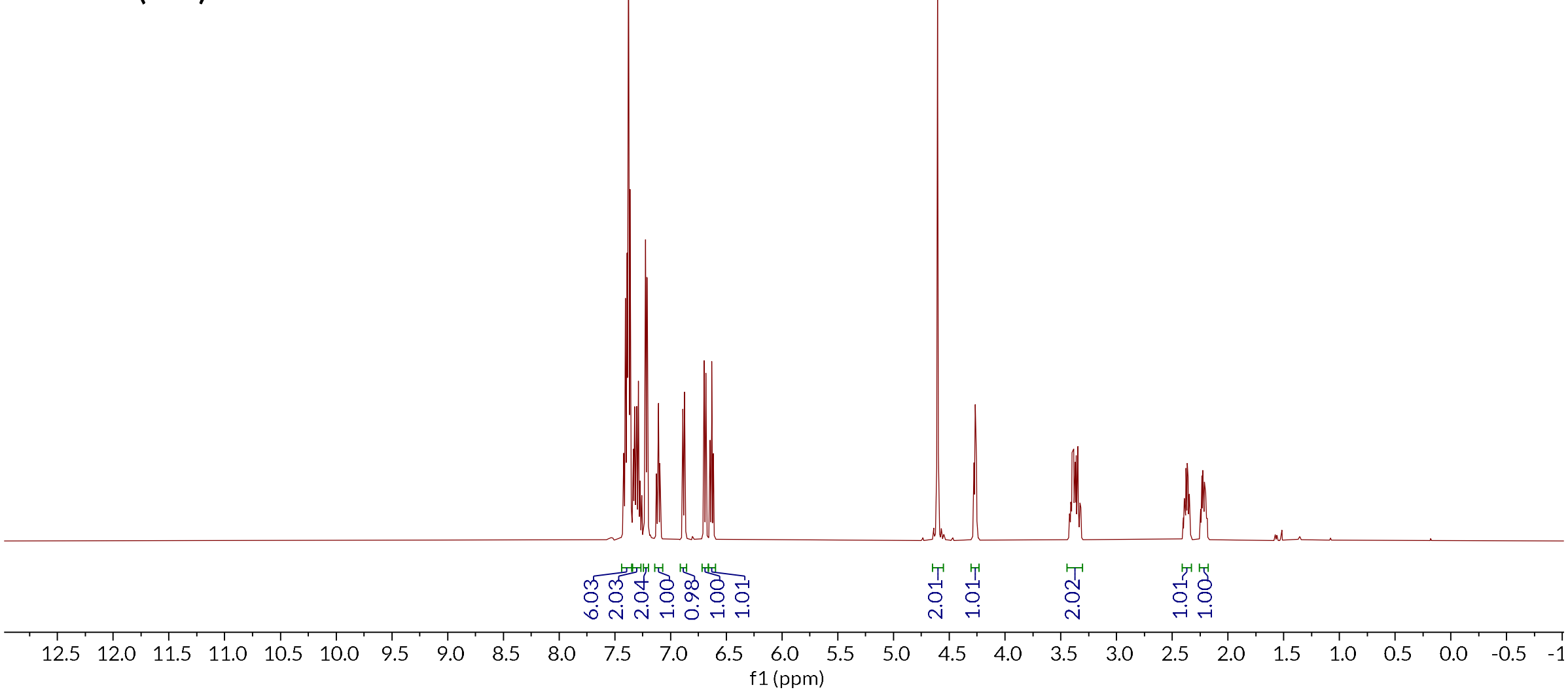

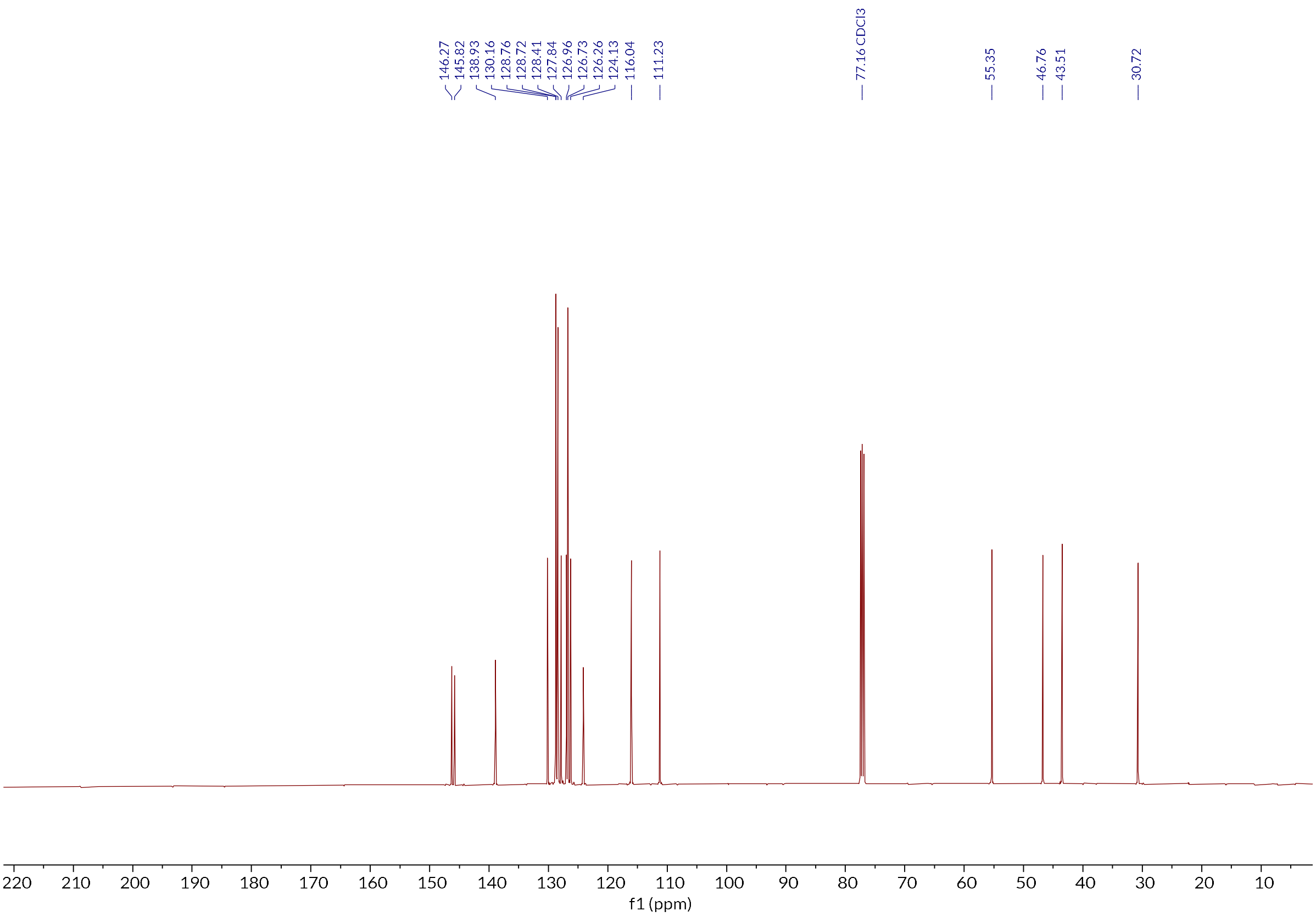

(±)-1-(4-Methoxybenzyl)-4-phenyl-1,2,3,4-tetrahydroquinoline (2ab)

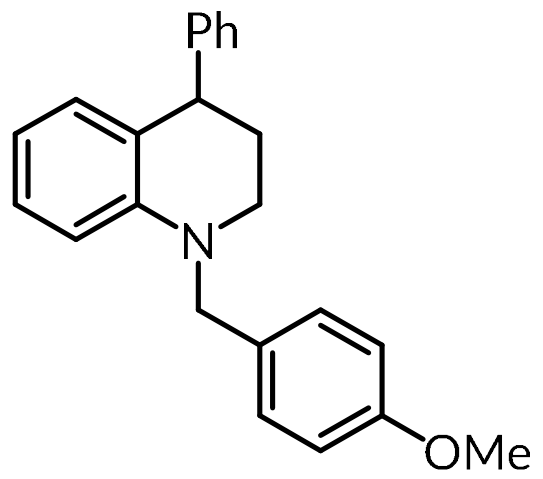

(2ab)

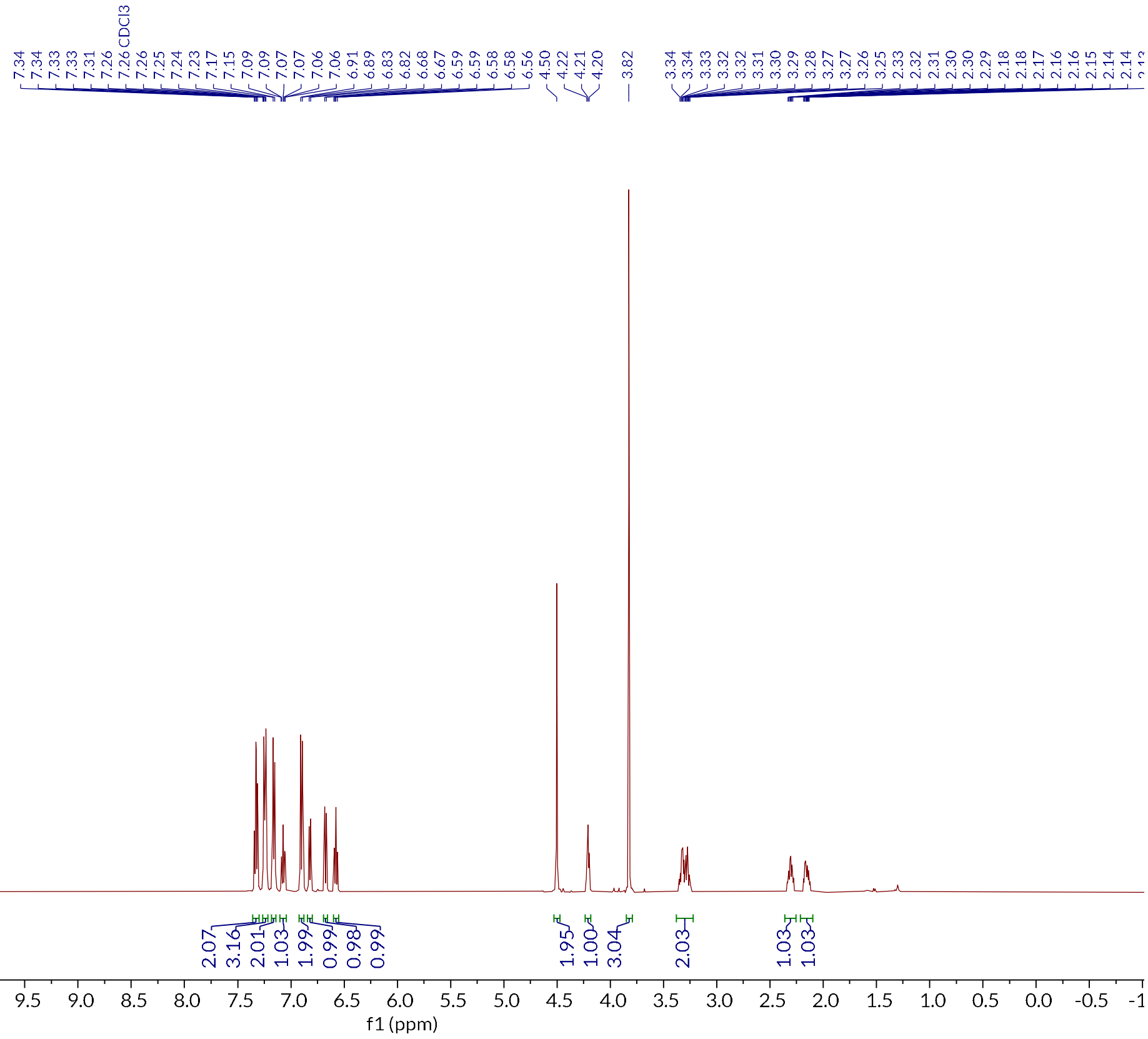

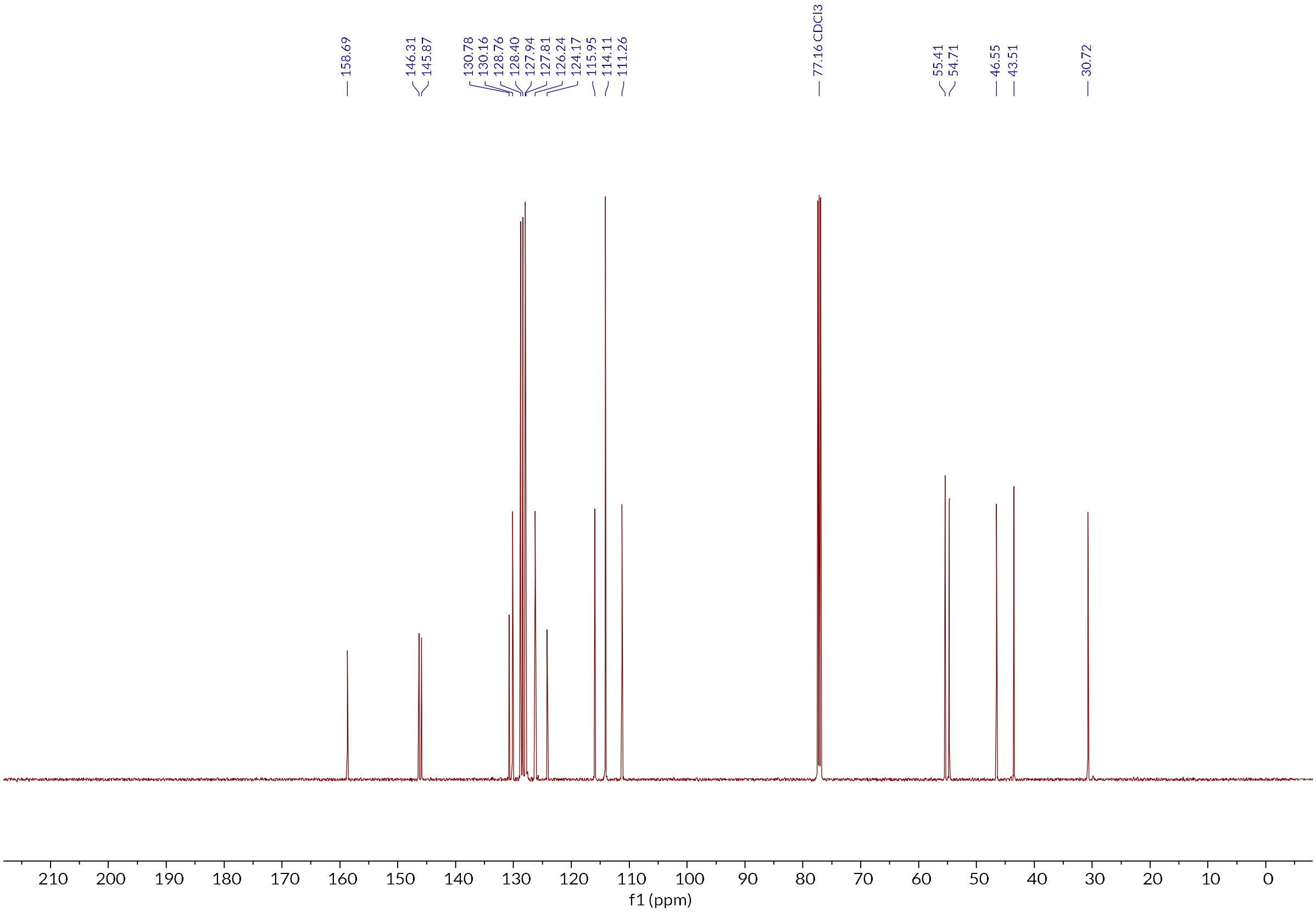

(±)-1-(4-Fluorobenzyl)-4-phenyl-1,2,3,4-tetrahydroquinoline (2ac)

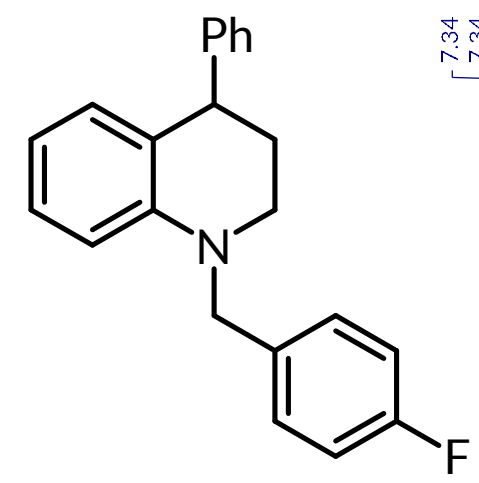

(2ac)

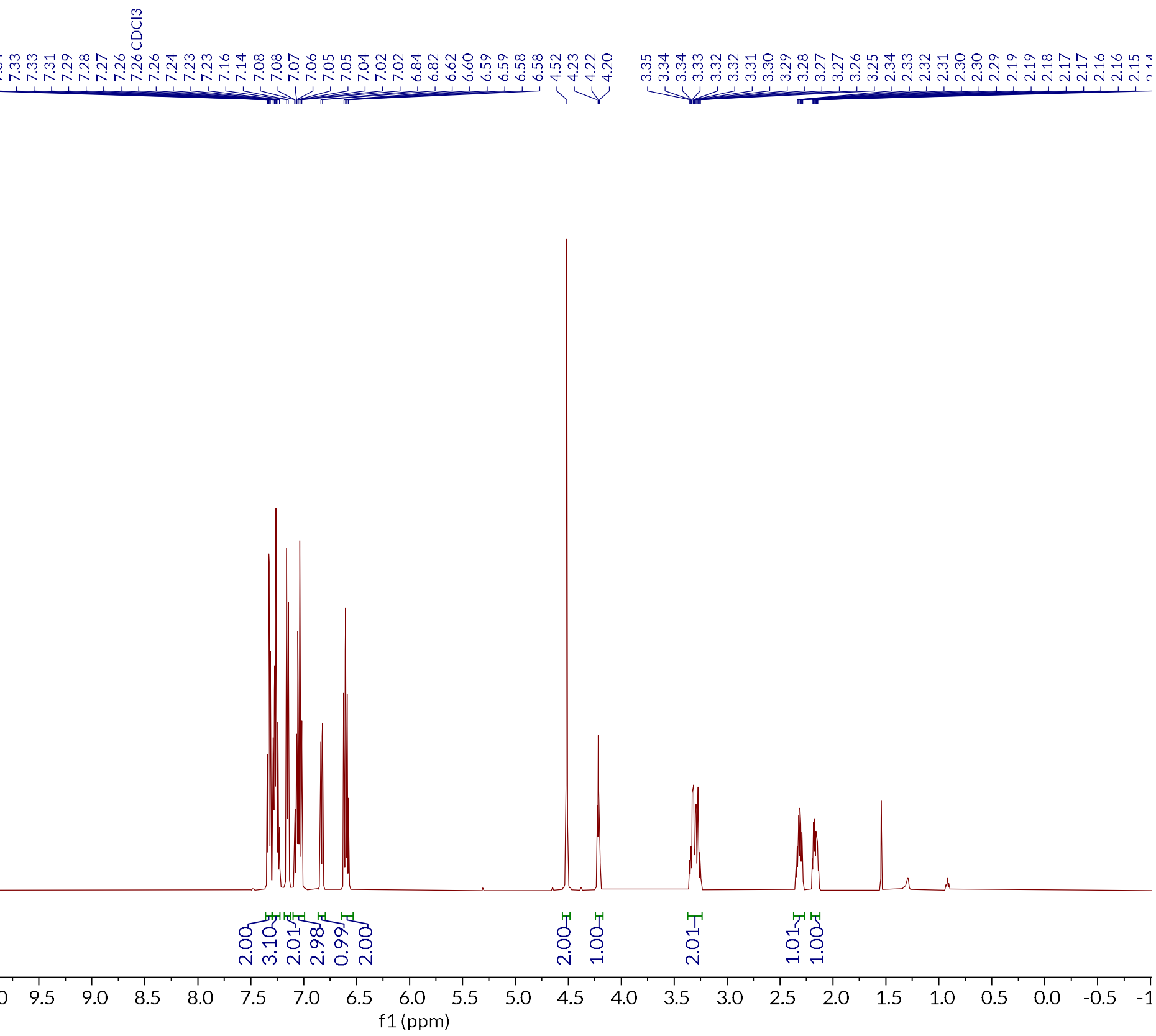

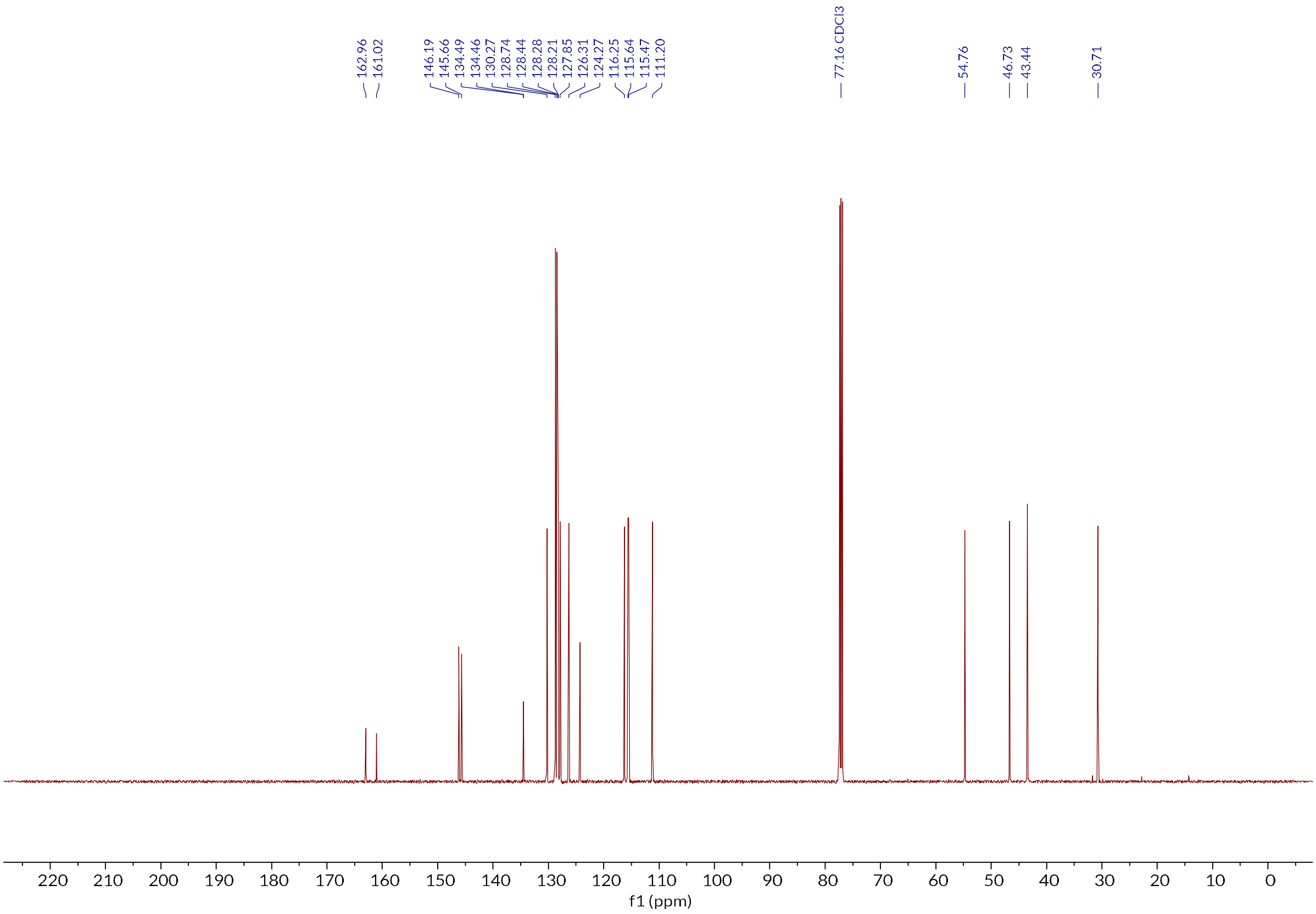

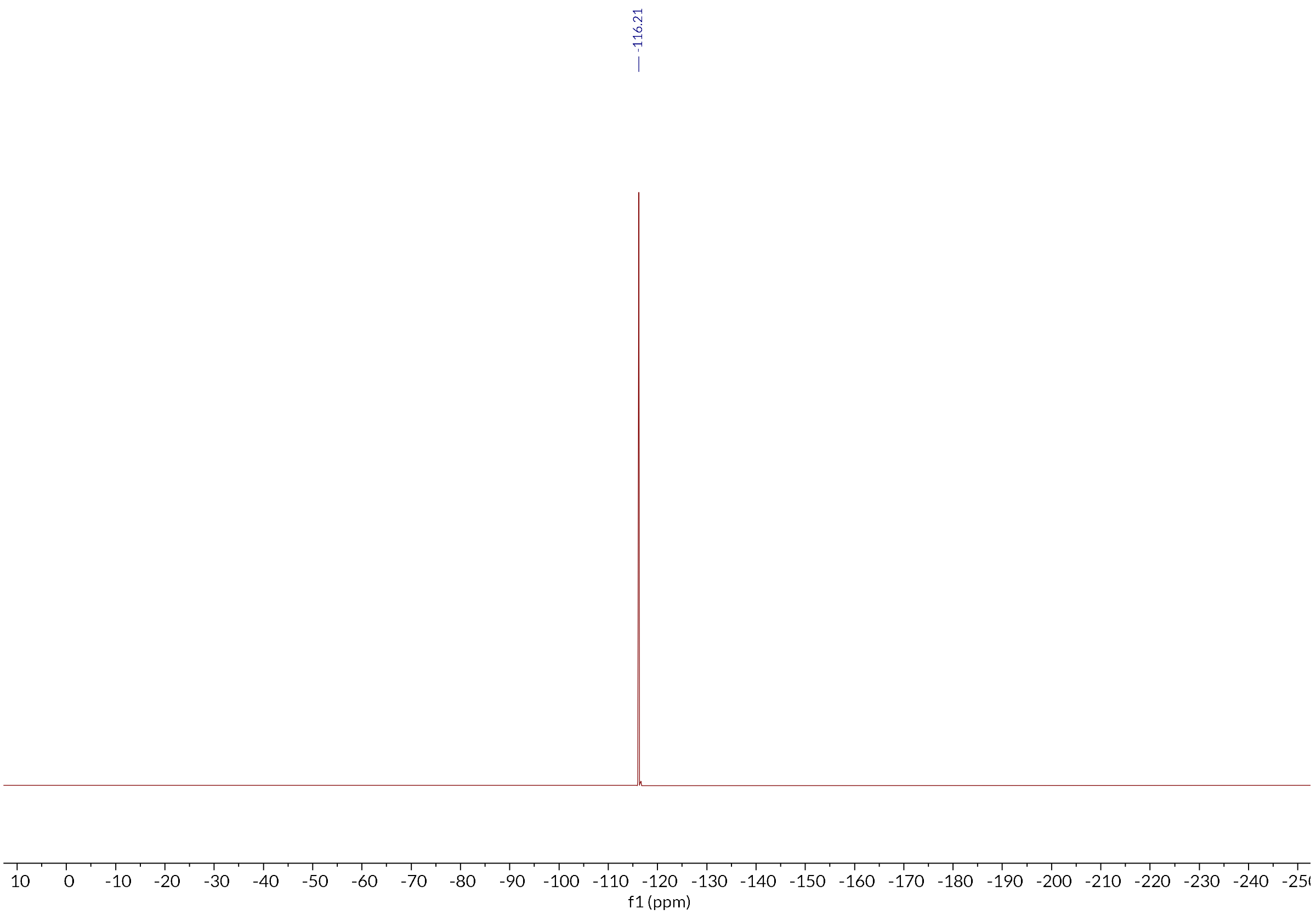
-116.21
10
0
-10
-20
-30
-40
-50
-60
-70
-80
-90
-100
-110
-120
-130
-140
-150
-160
-170
-180
-190
-200
-210
-220
-230
-240
f1 (ppm)

(±)-4-Phenyl-1-(4-(trifluoromethyl)benzyl)-1,2,3,4-tetrahydroquinoline (2ad)

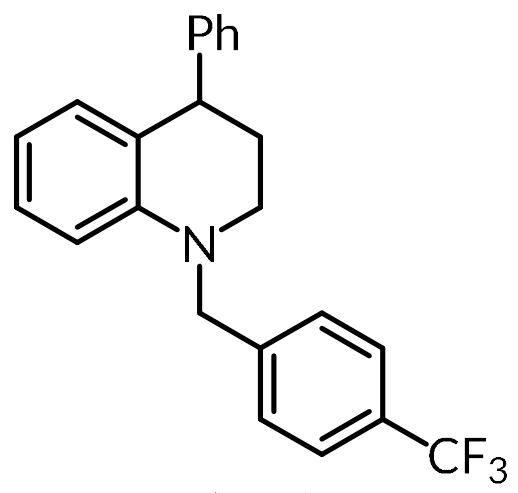

(2ad)

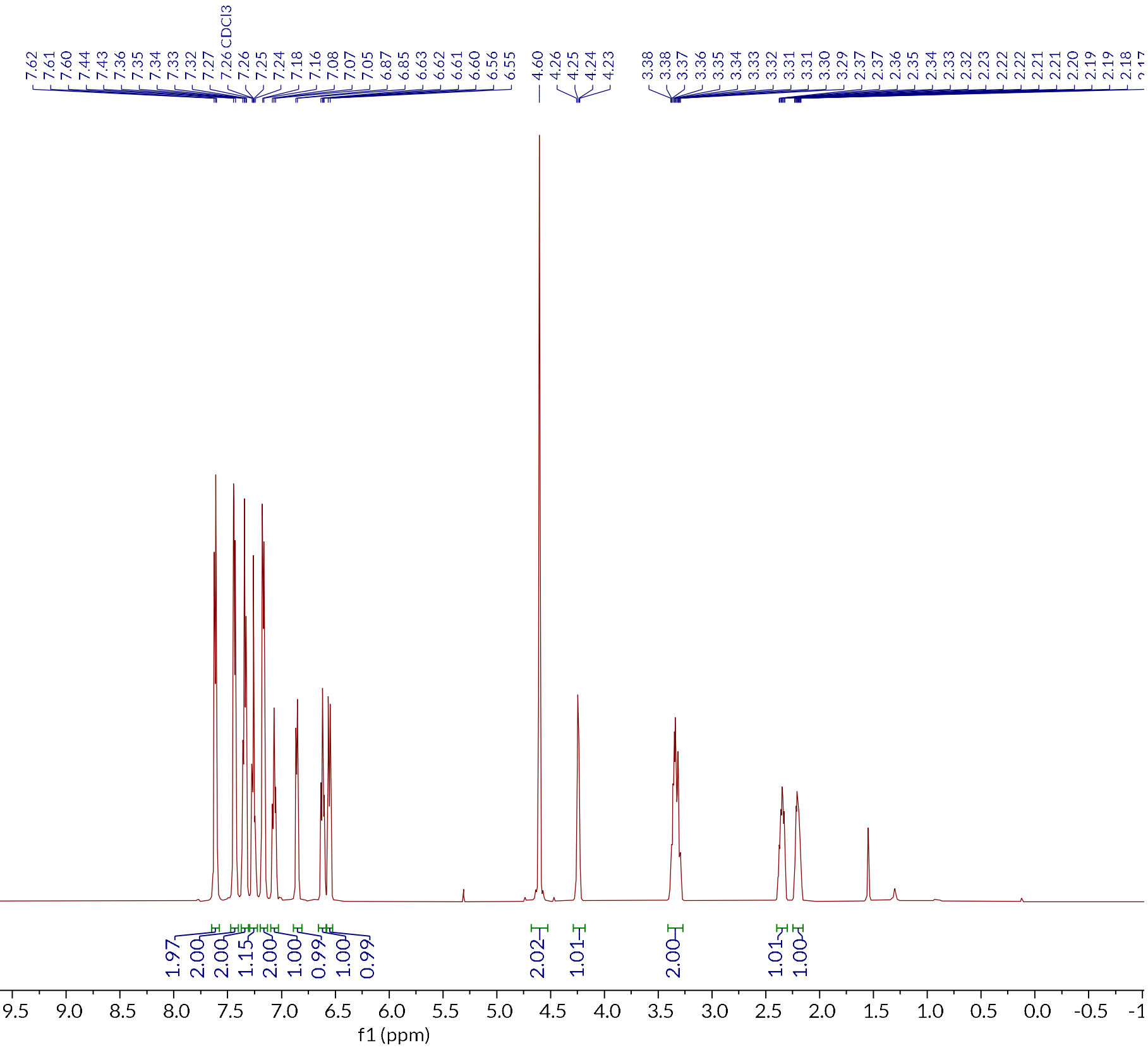

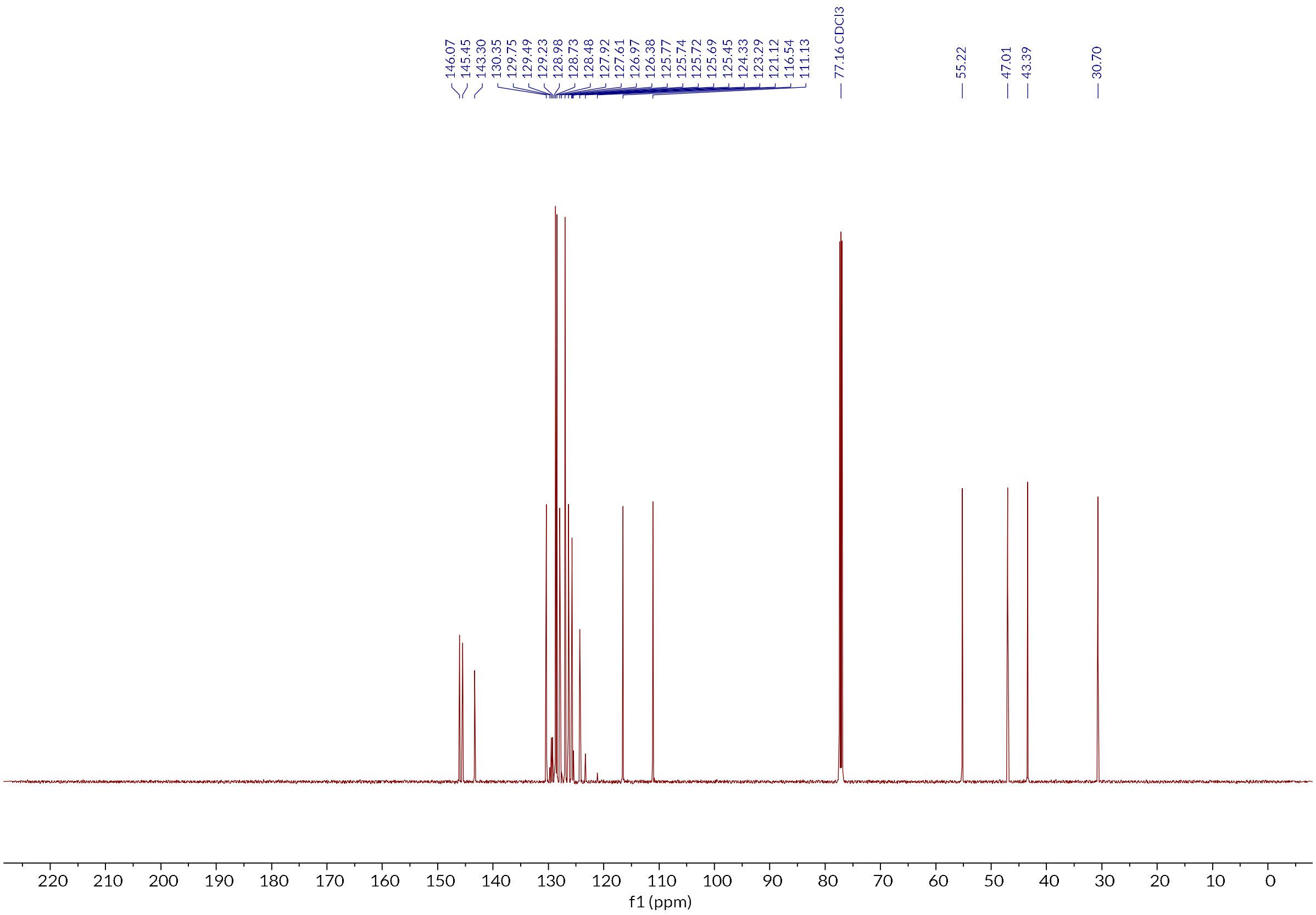

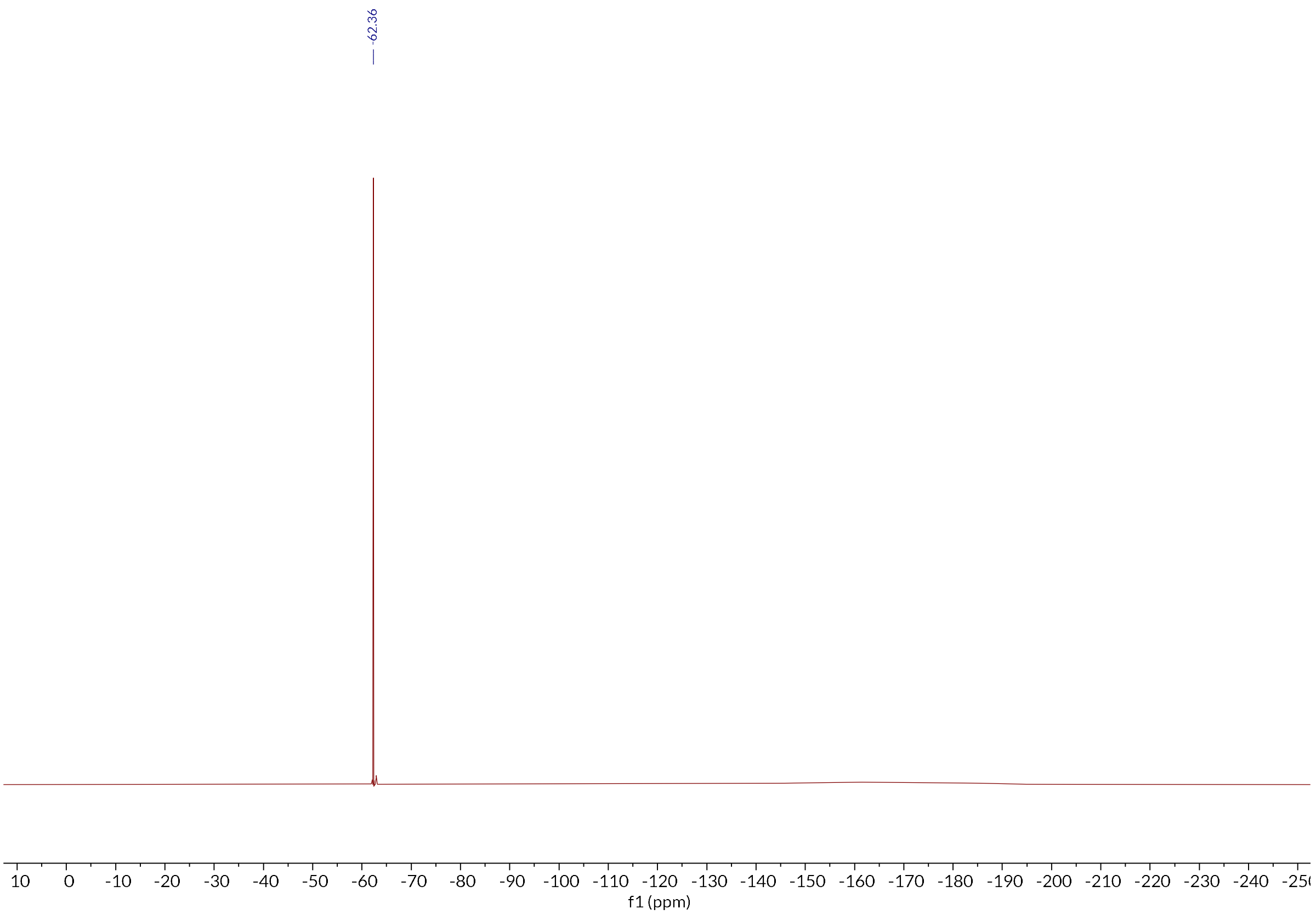
-62.36
10
0
-10
-20
-30
-40
-50
-60
-70
-80
-90
-100
-110
-120
-130
-140
-150
-160
-170
-180
-190
-200
-210
-220
-230
-240
f1 (ppm)

(±)-1-(Naphthalen-1-ylmethyl)-4-phenyl-1,2,3,4-tetrahydroquinoline (2ae)

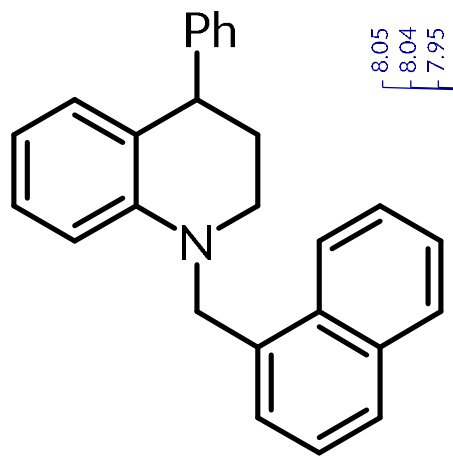

(2ae)

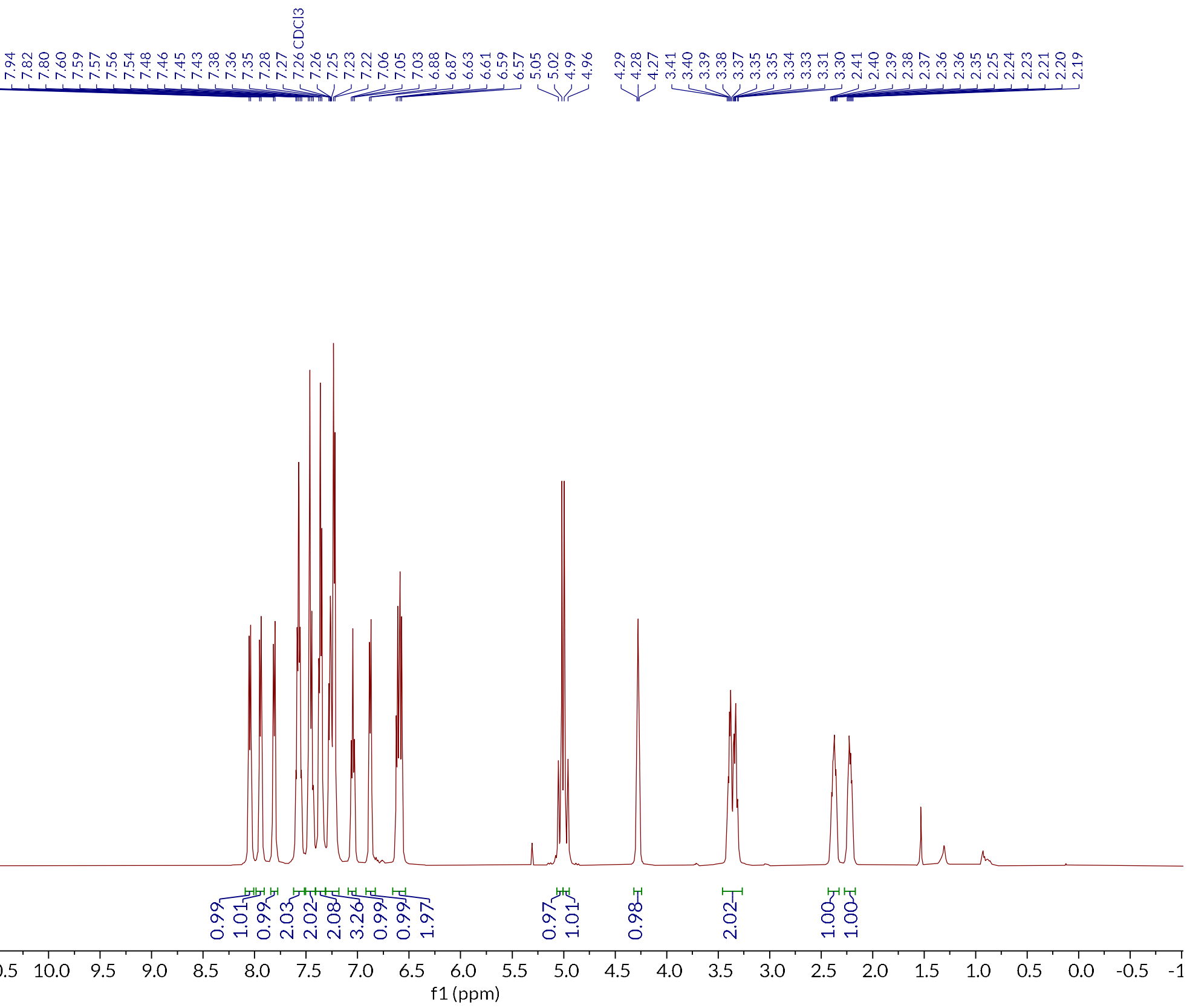

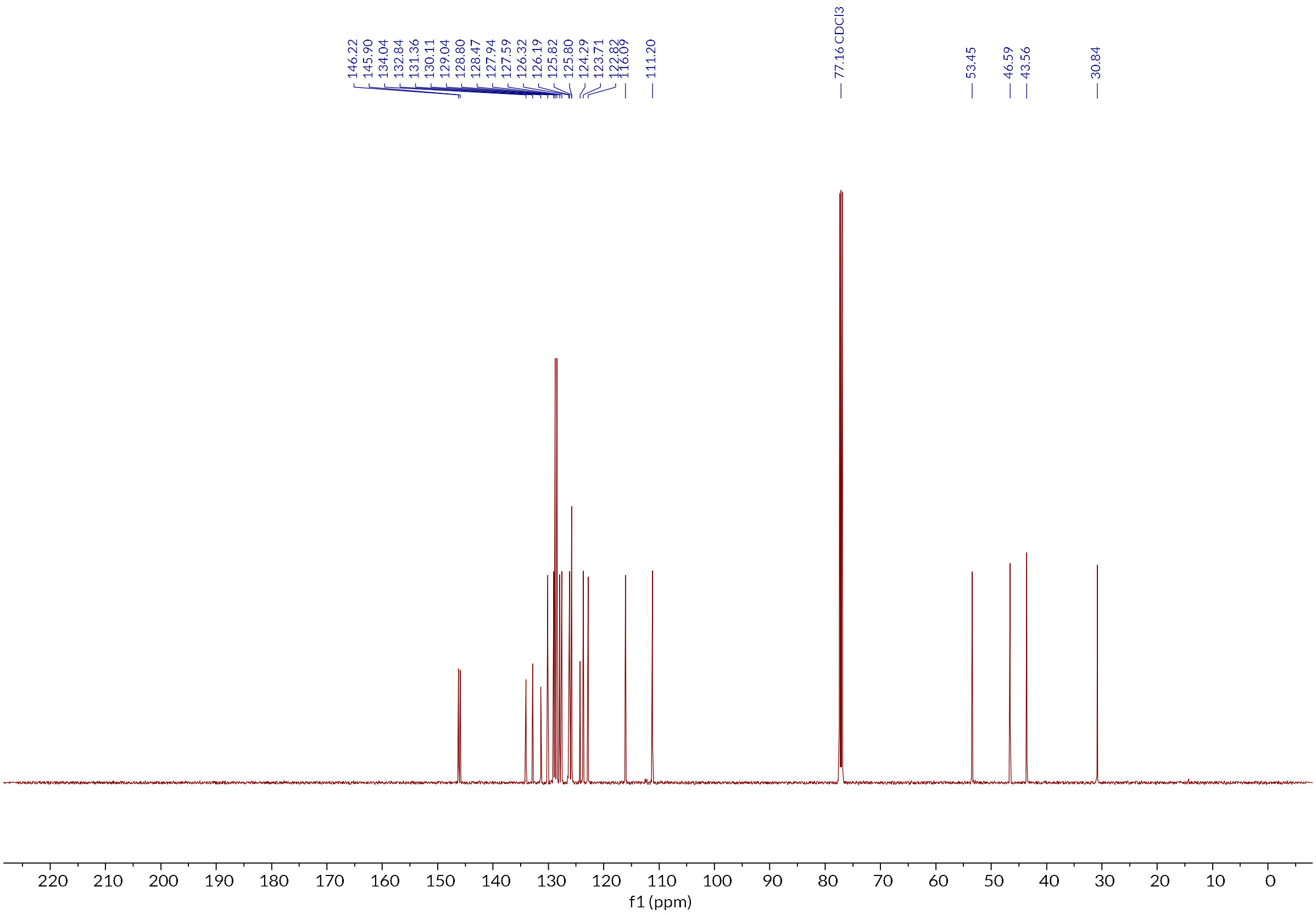

(±)-4-Phenyl-1-(thiophen-2-ylmethyl)-1,2,3,4-tetrahydroquinoline (2af)

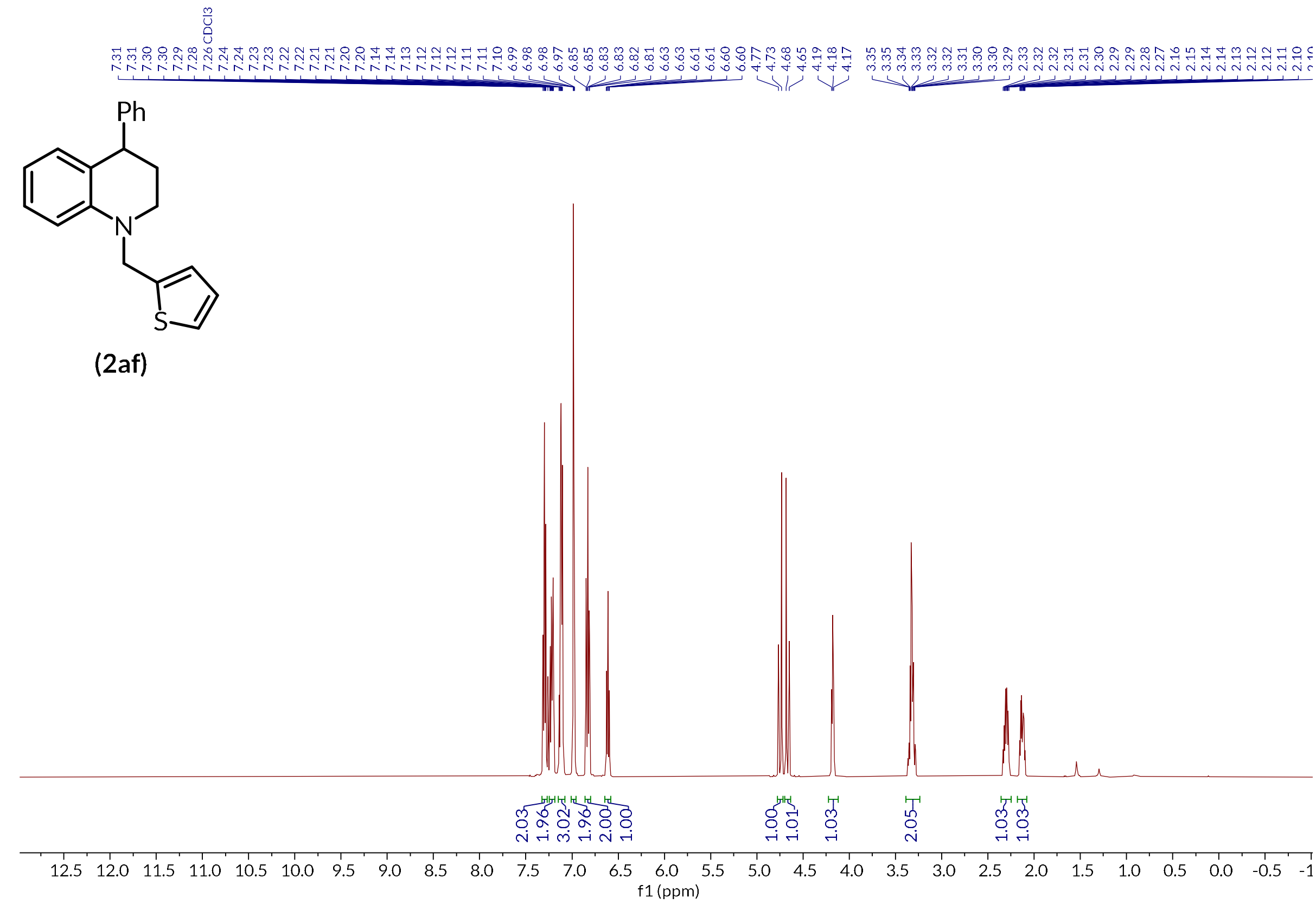

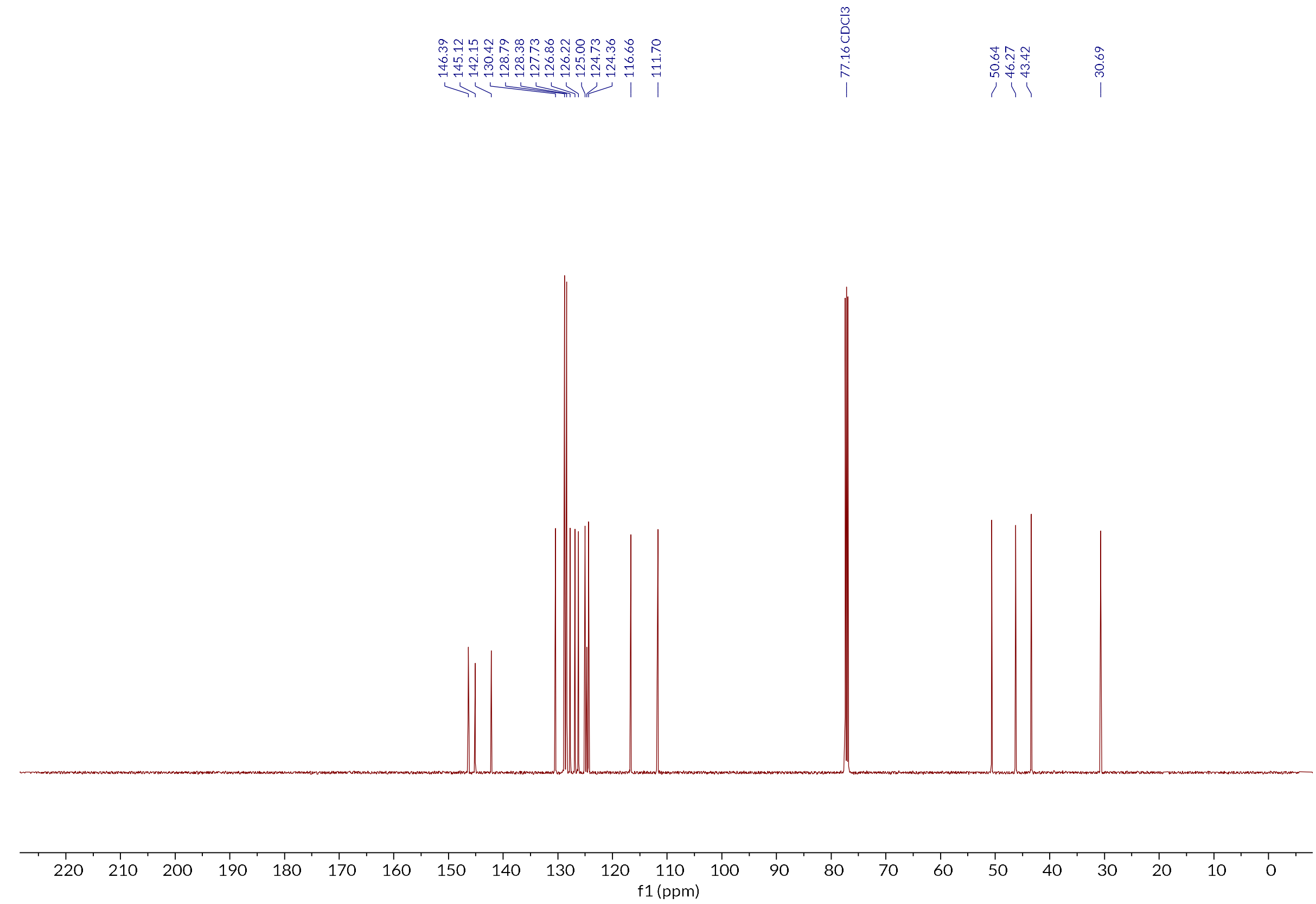

(±)-1-Methyl-4-phenyl-1,2,3,4-tetrahydroquinoline (2ag)

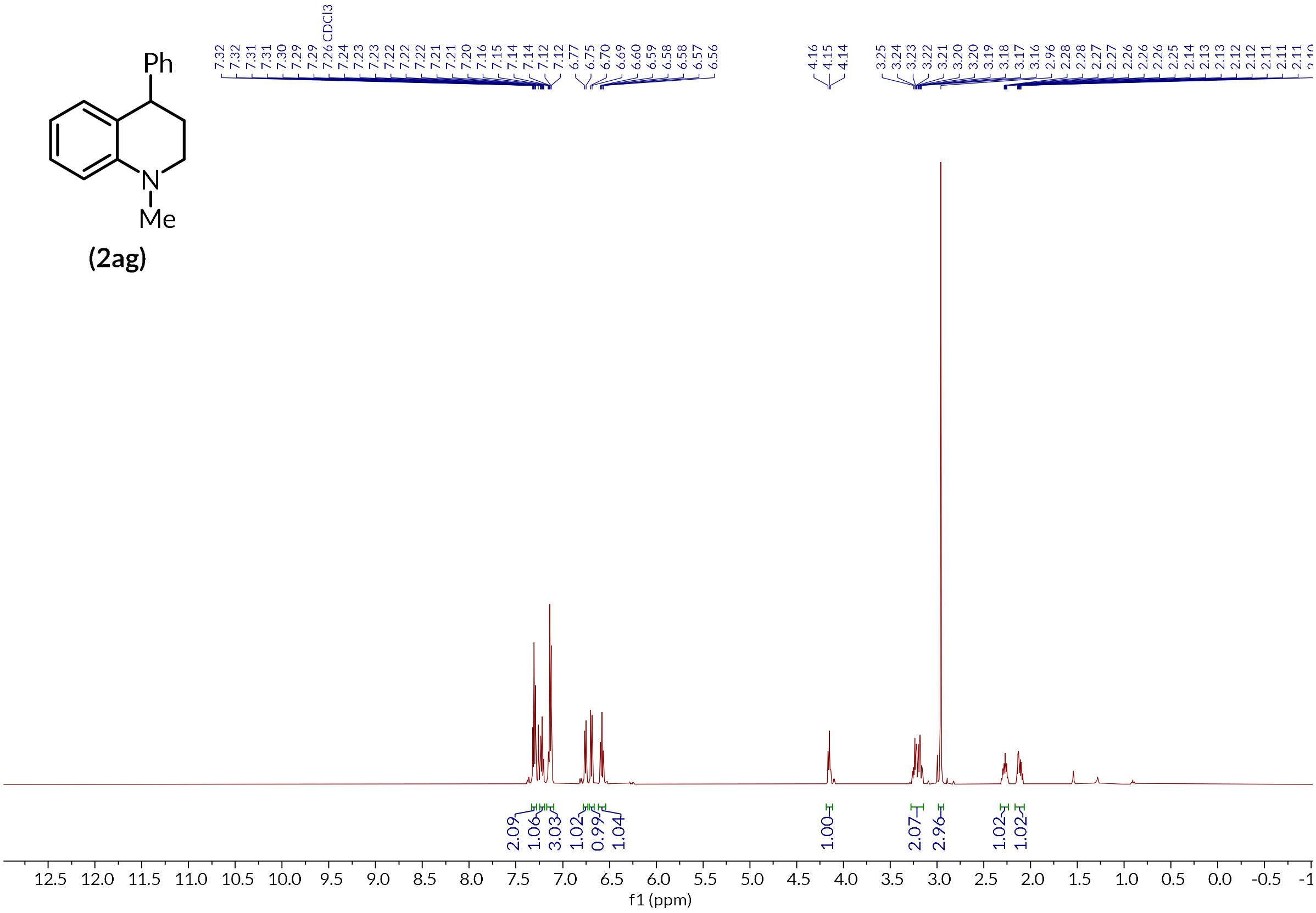

(2ag)

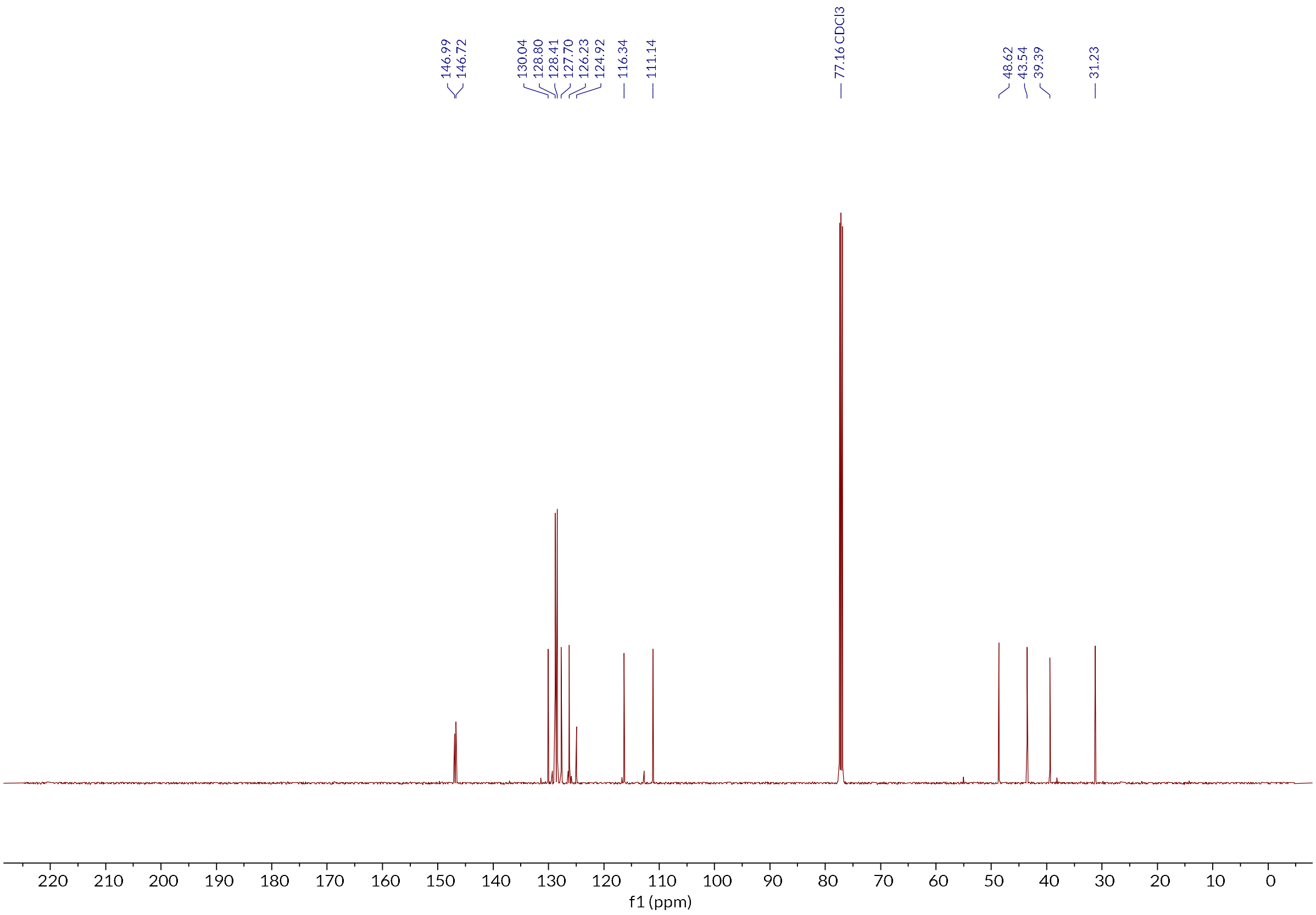

(±)-1-Cyclohexyl-4-phenyl-1,2,3,4-tetrahydroquinoline (2ah)

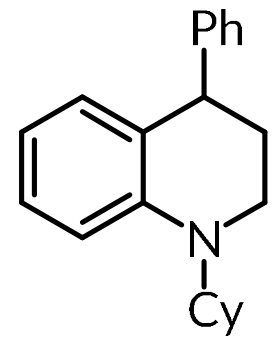

(2ah)

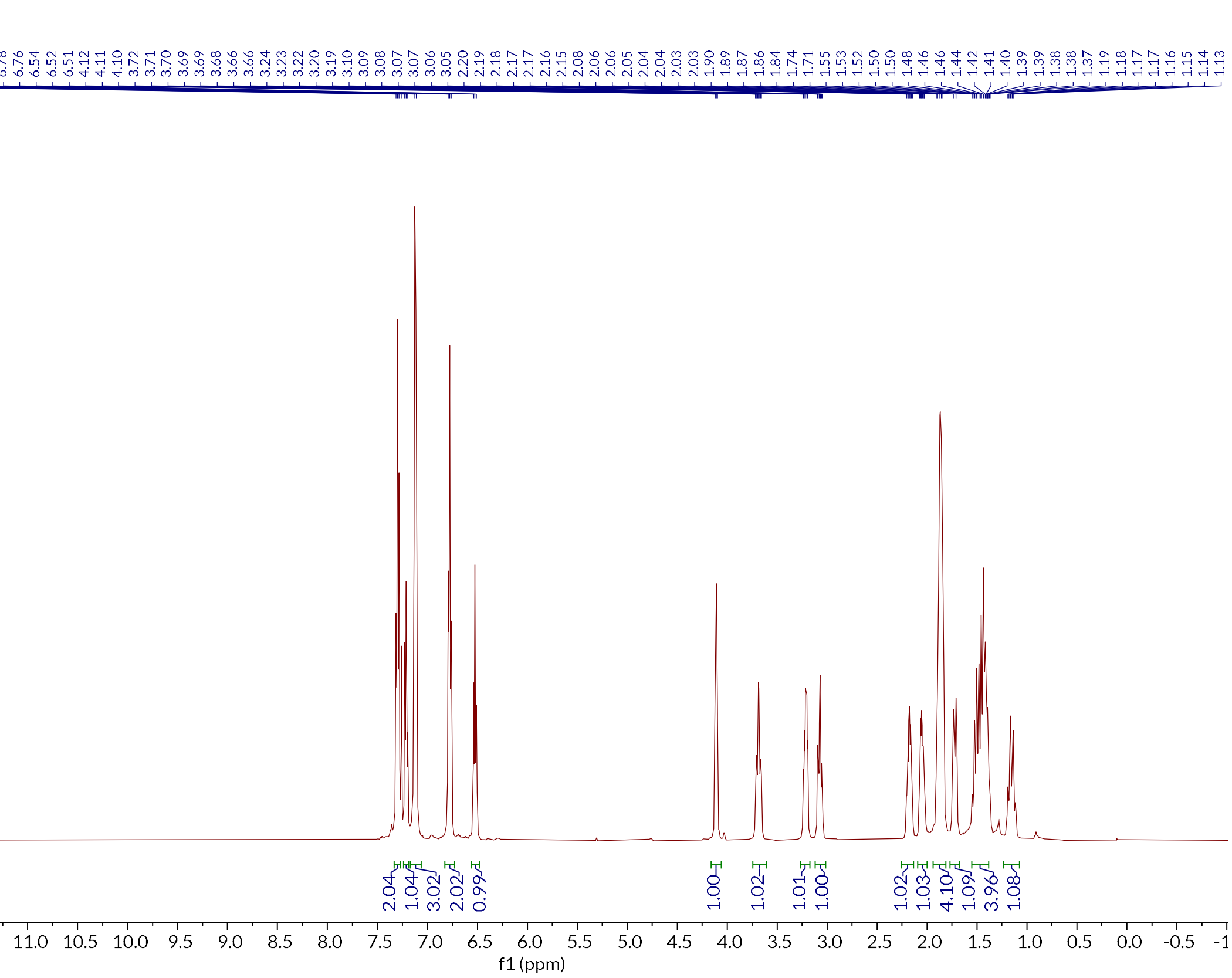

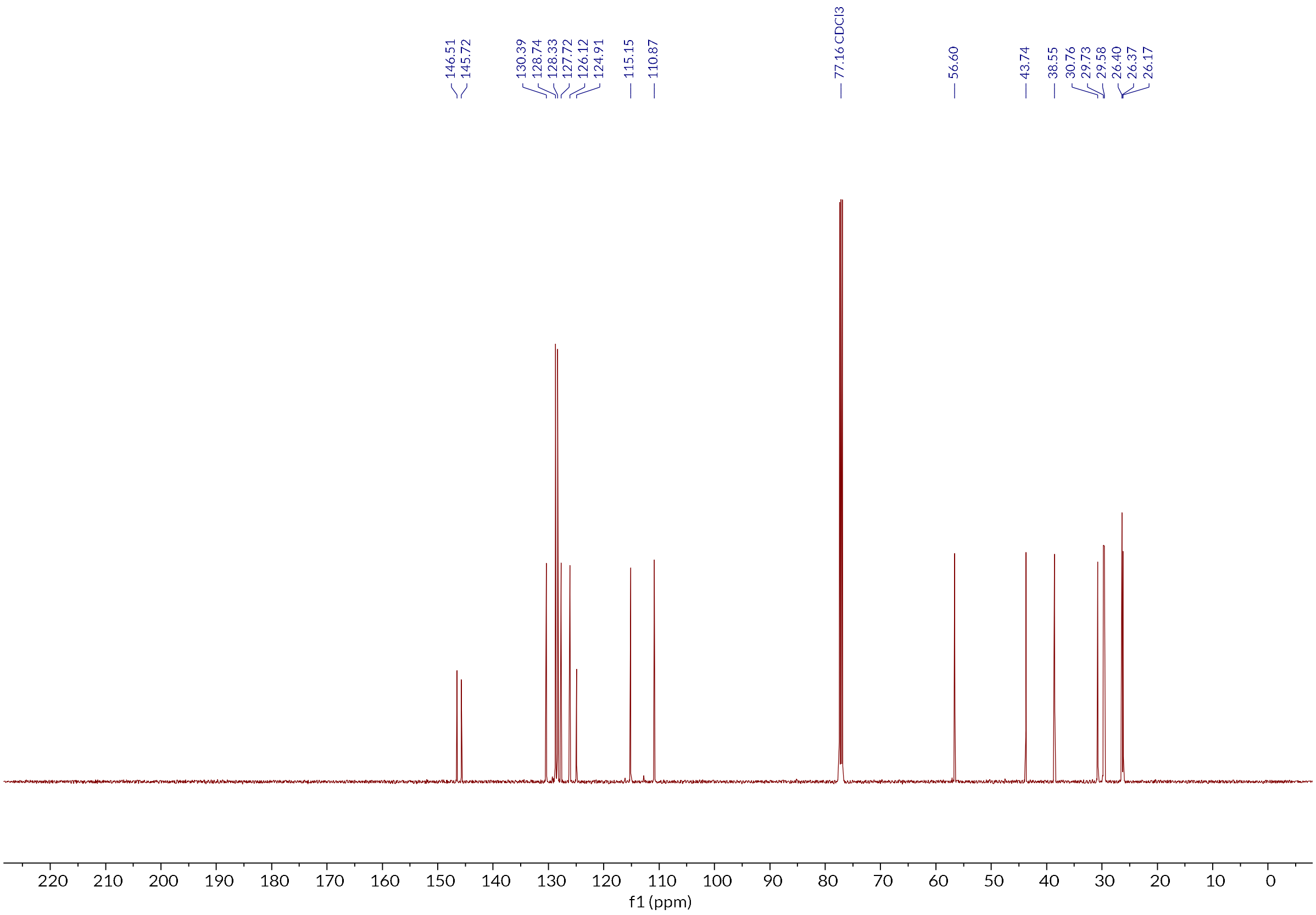

(±)-1,4-Diphenyl-1,2,3,4-tetrahydroquinoline (2ai)

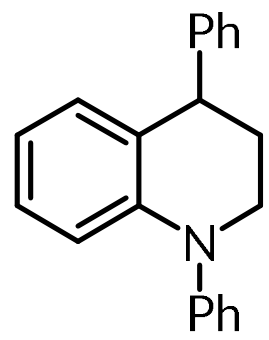

(2ai)

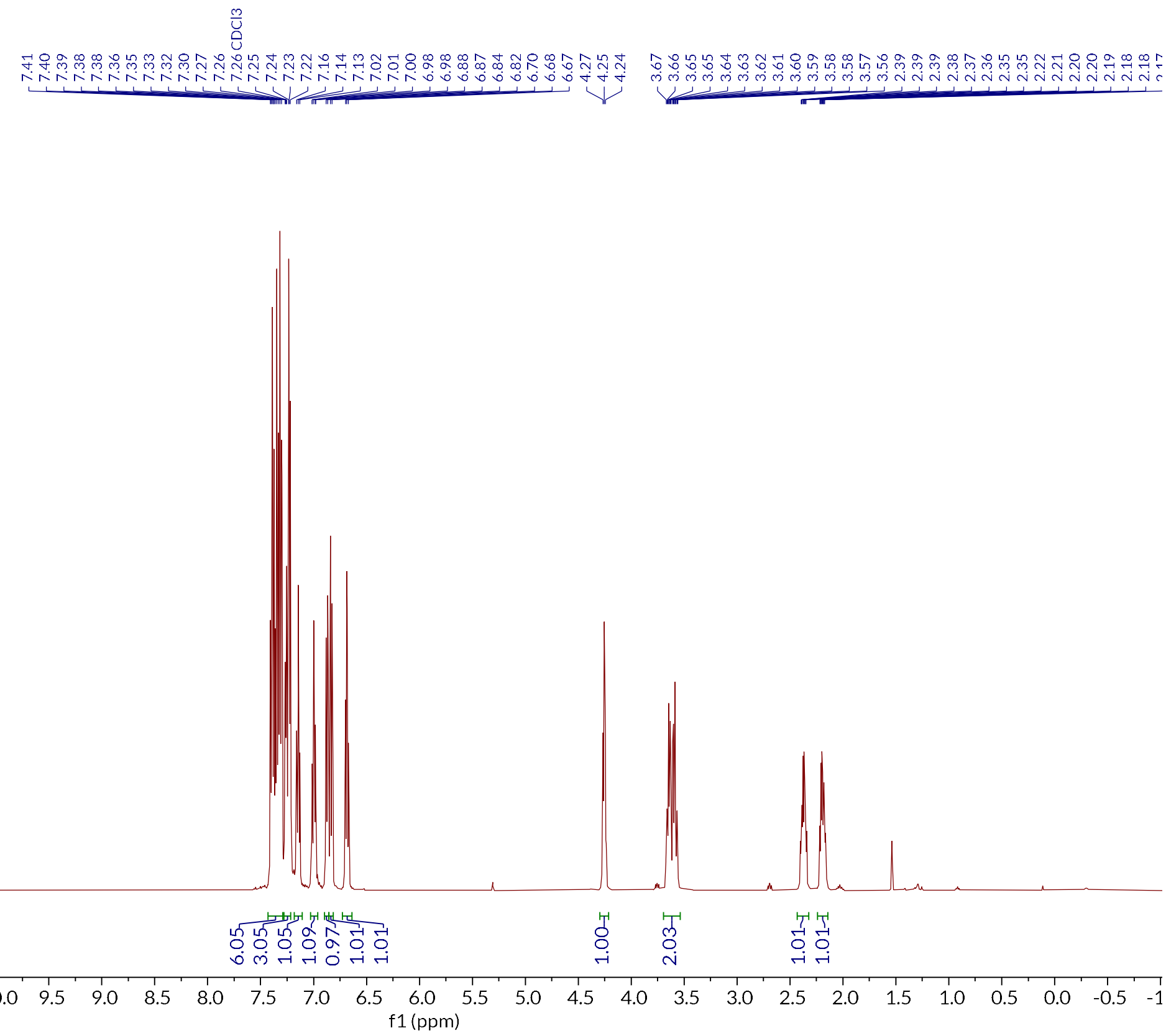

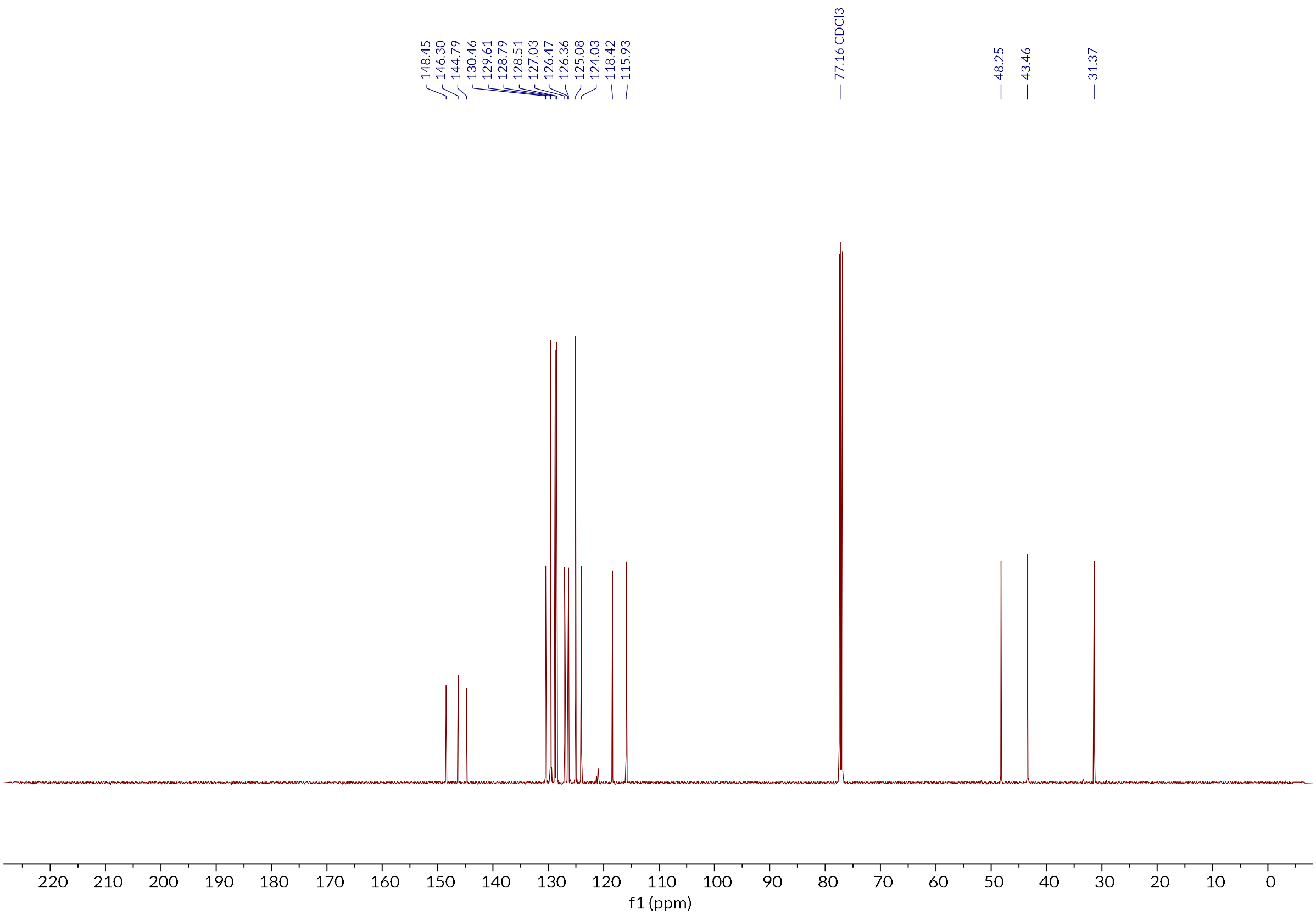

(±)-1-Allyl-4-phenyl-1,2,3,4-tetrahydroquinoline (2aj)

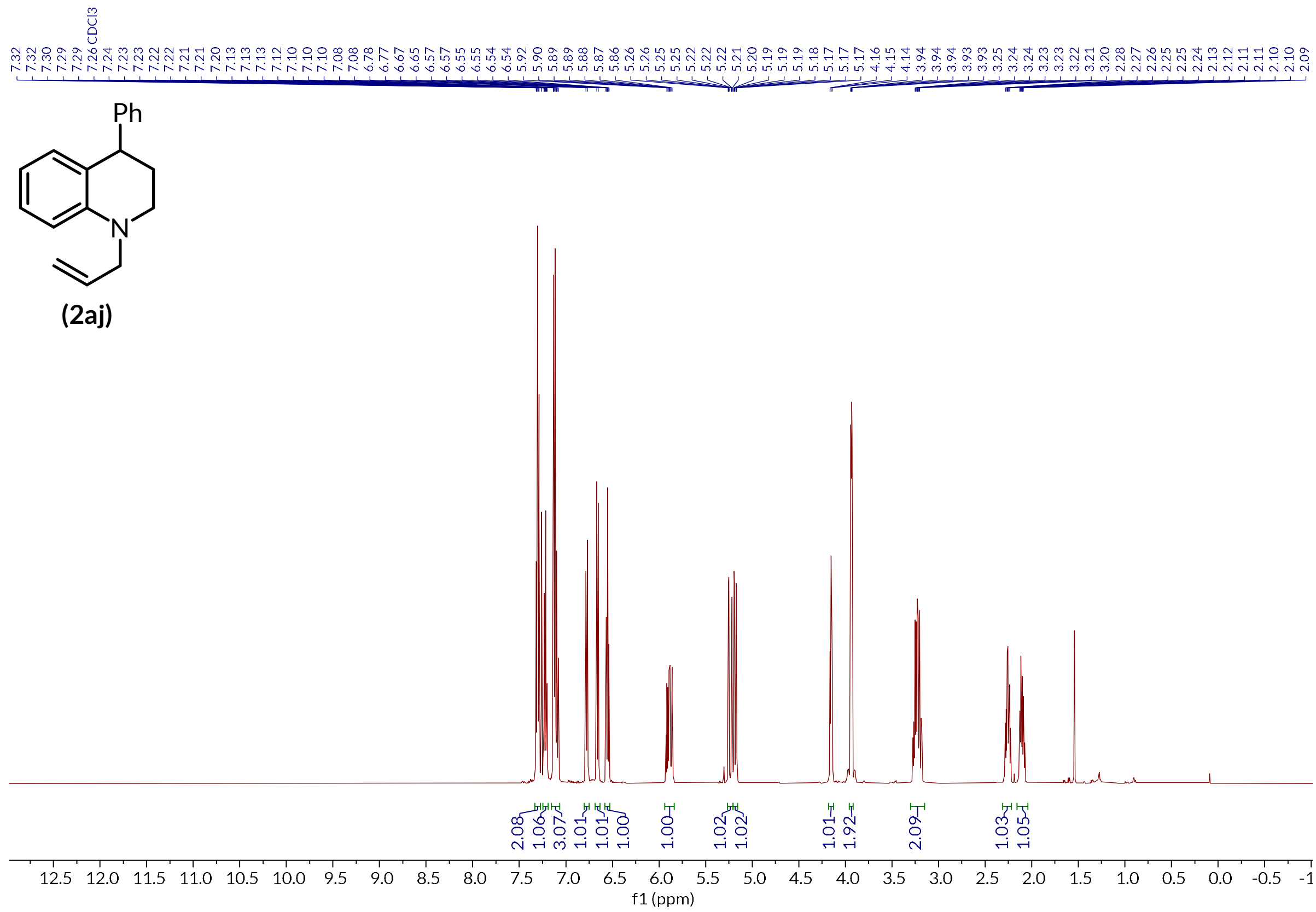

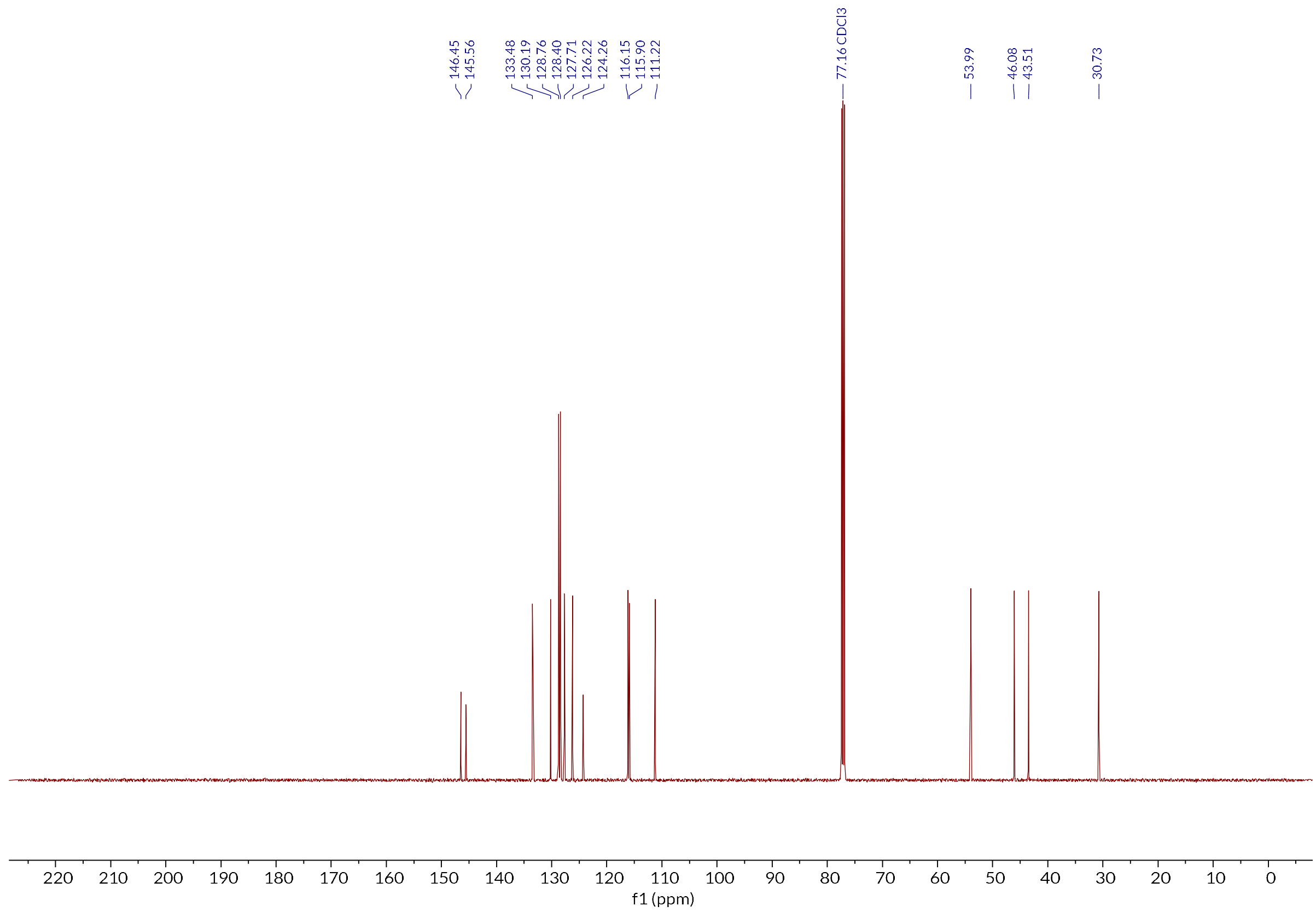

(±)-1,7-Diphenyl-2,3,6,7-tetrahydro-1*H*,5*H*-pyrido[3,2,1-*ij*]quinoline (2ak)

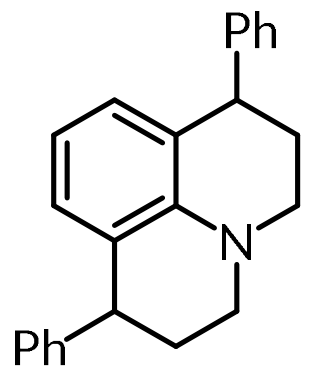

(2ak)

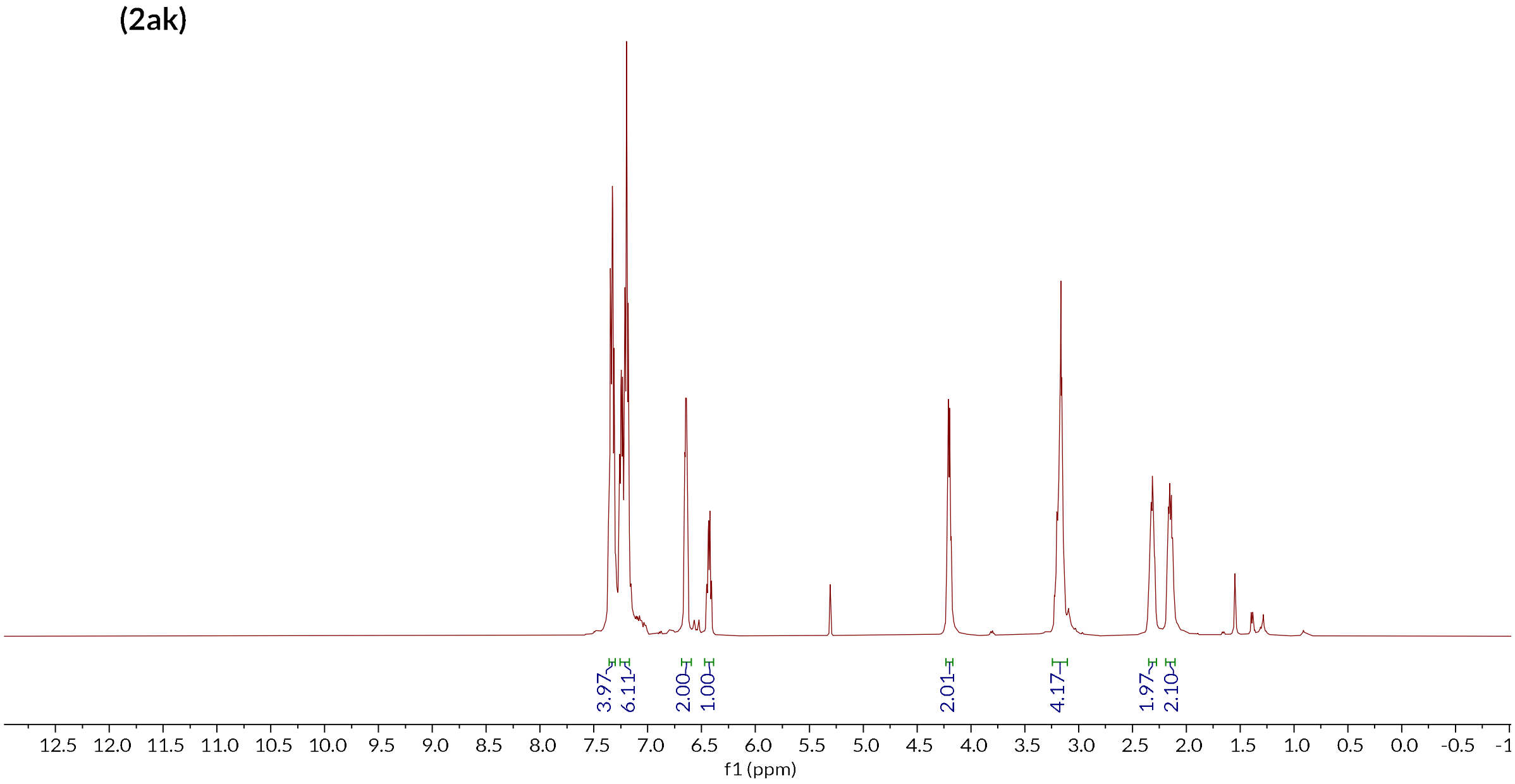

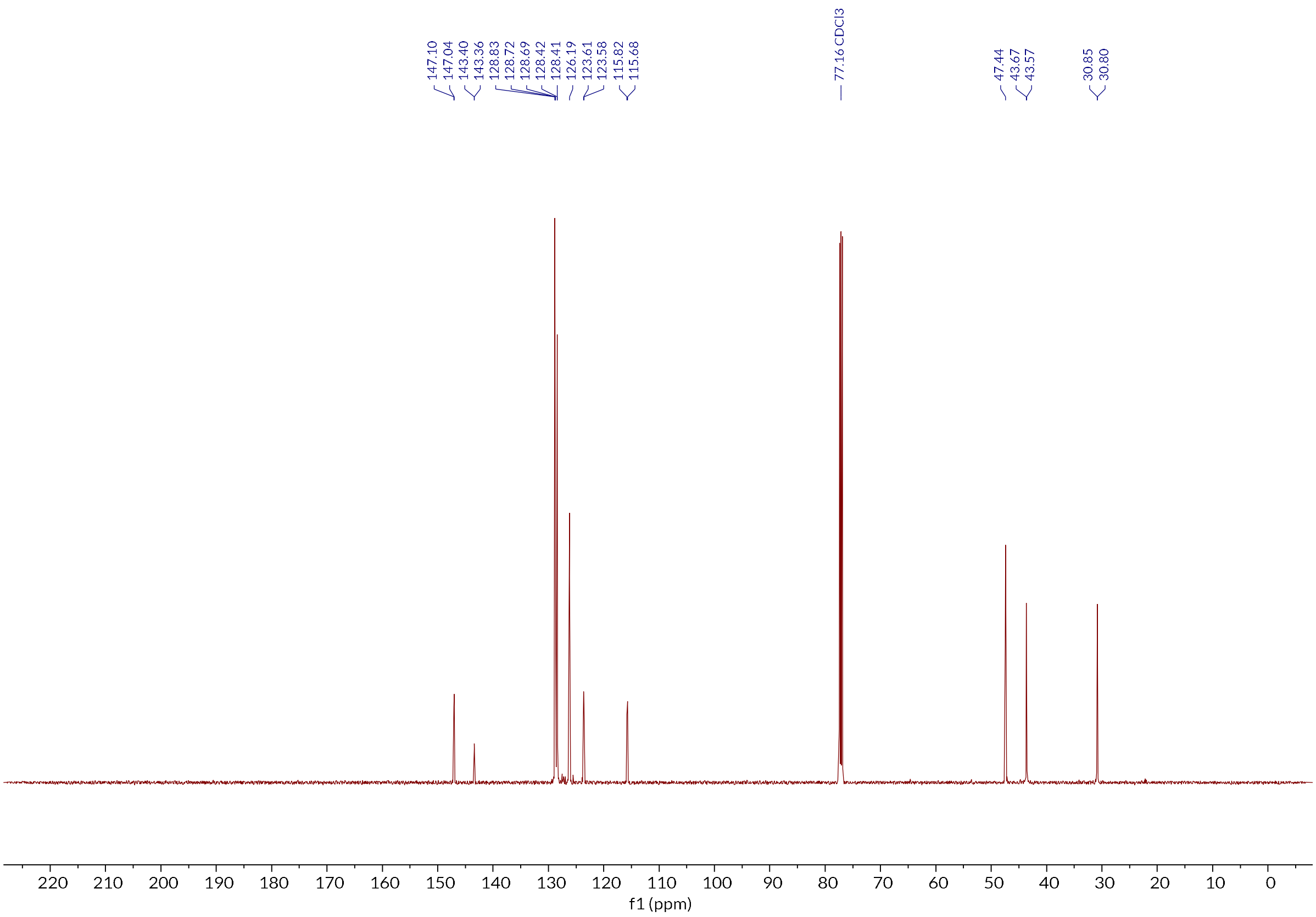

(±)-5,7-Dimethoxy-4-phenyl-1-tosyl-1,2,3,4-tetrahydroquinoline (2al)

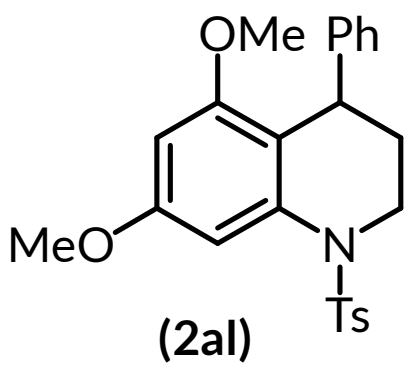

(2al)

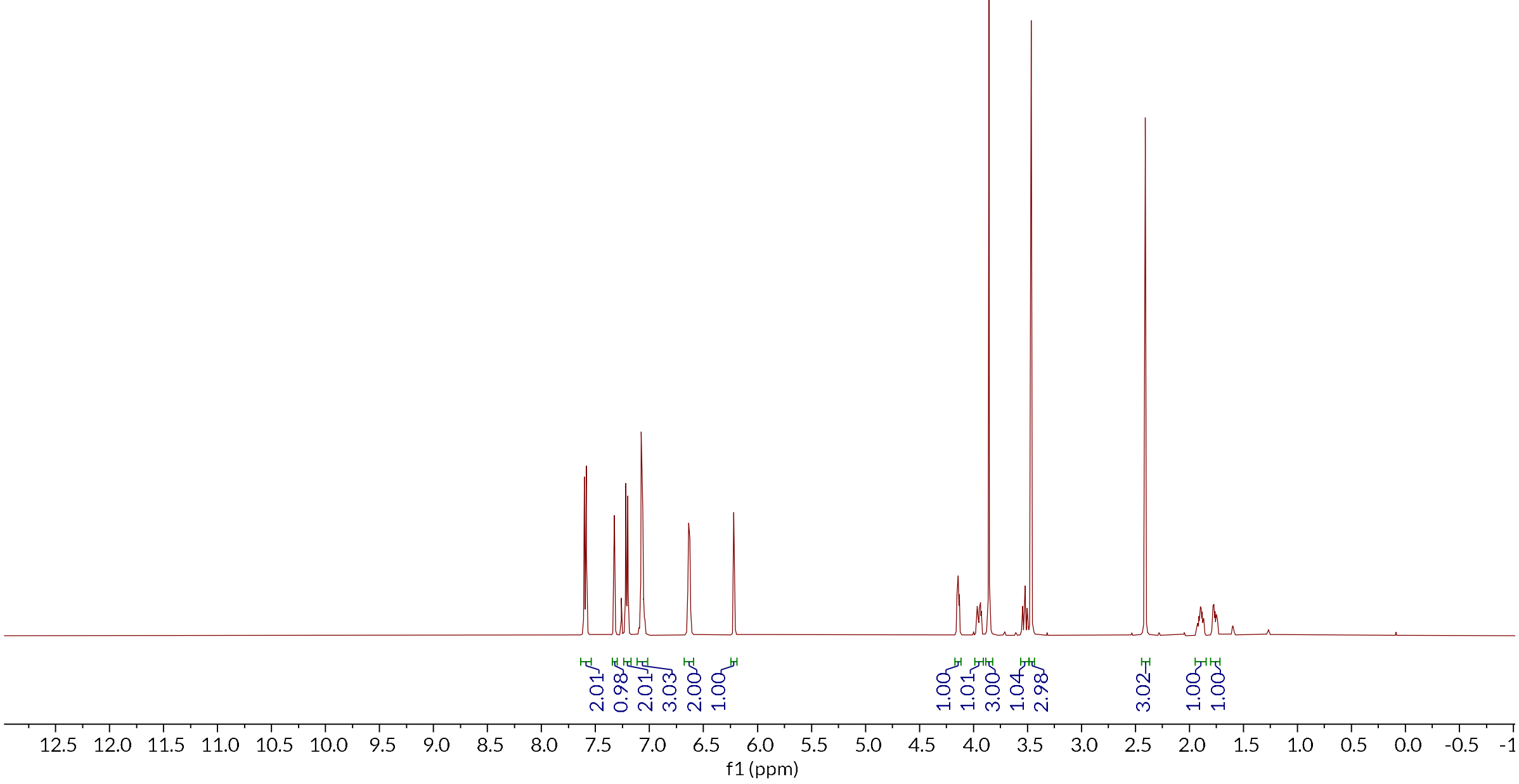

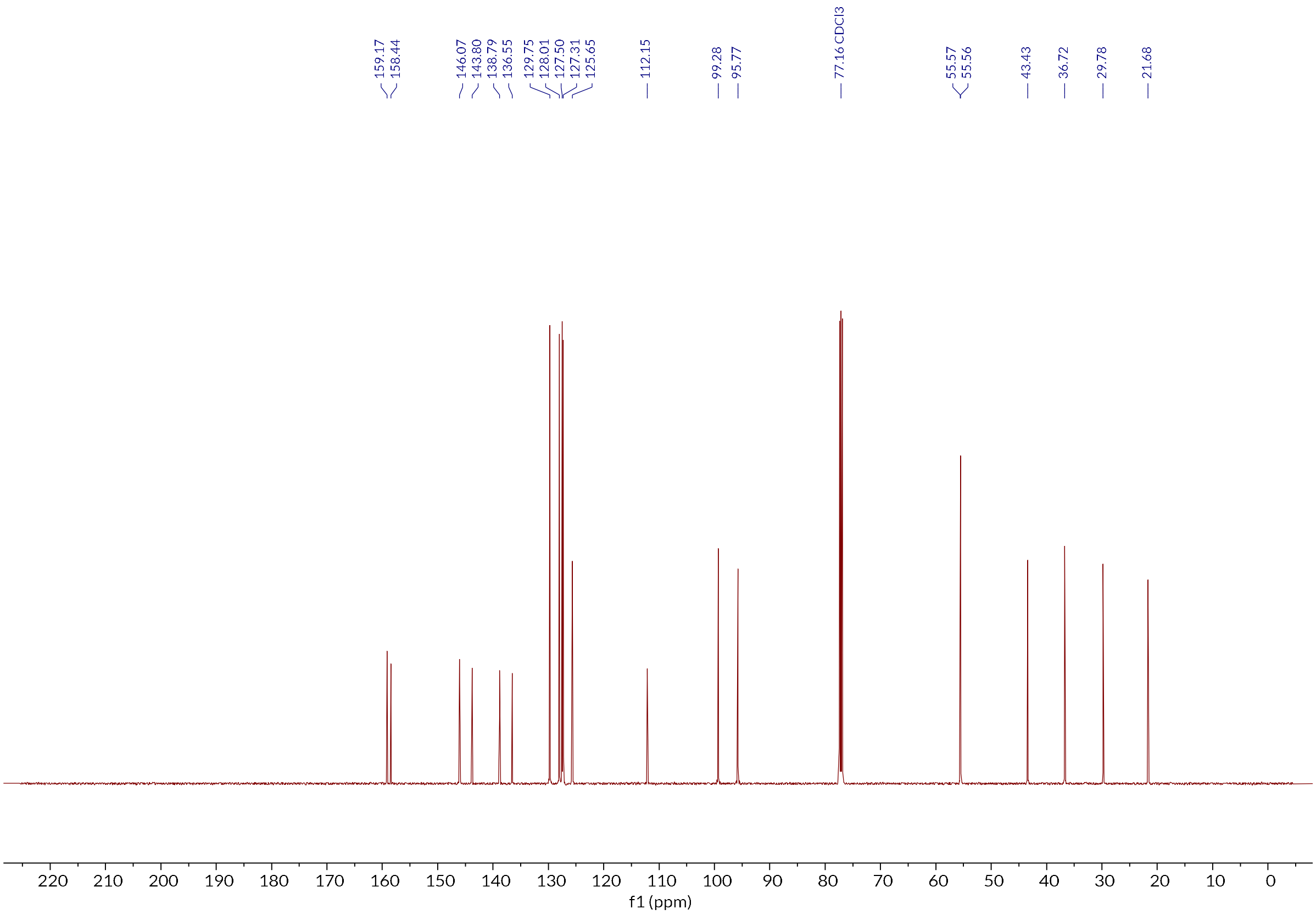

(±)-1-Benzyl-5,7-dimethoxy-4-phenyl-3,4-dihydroquinolin-2(1*H*)-one (2am)

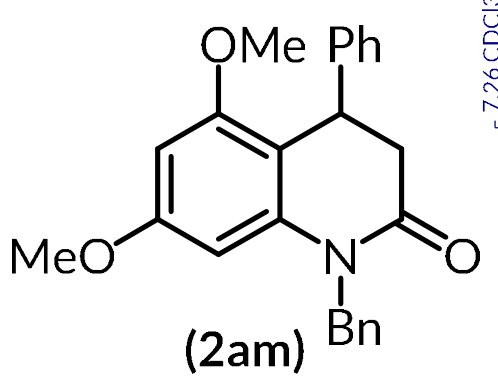

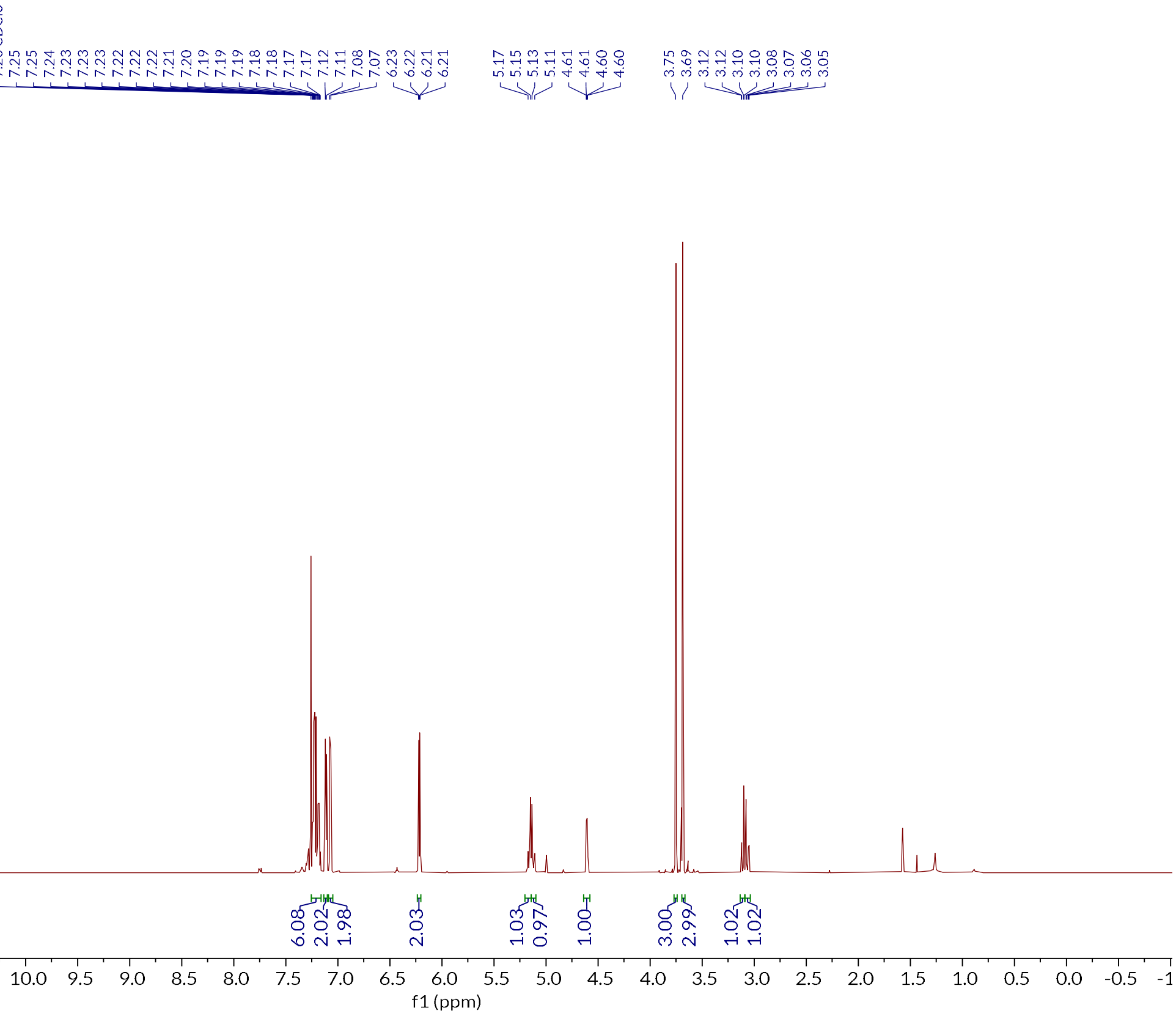

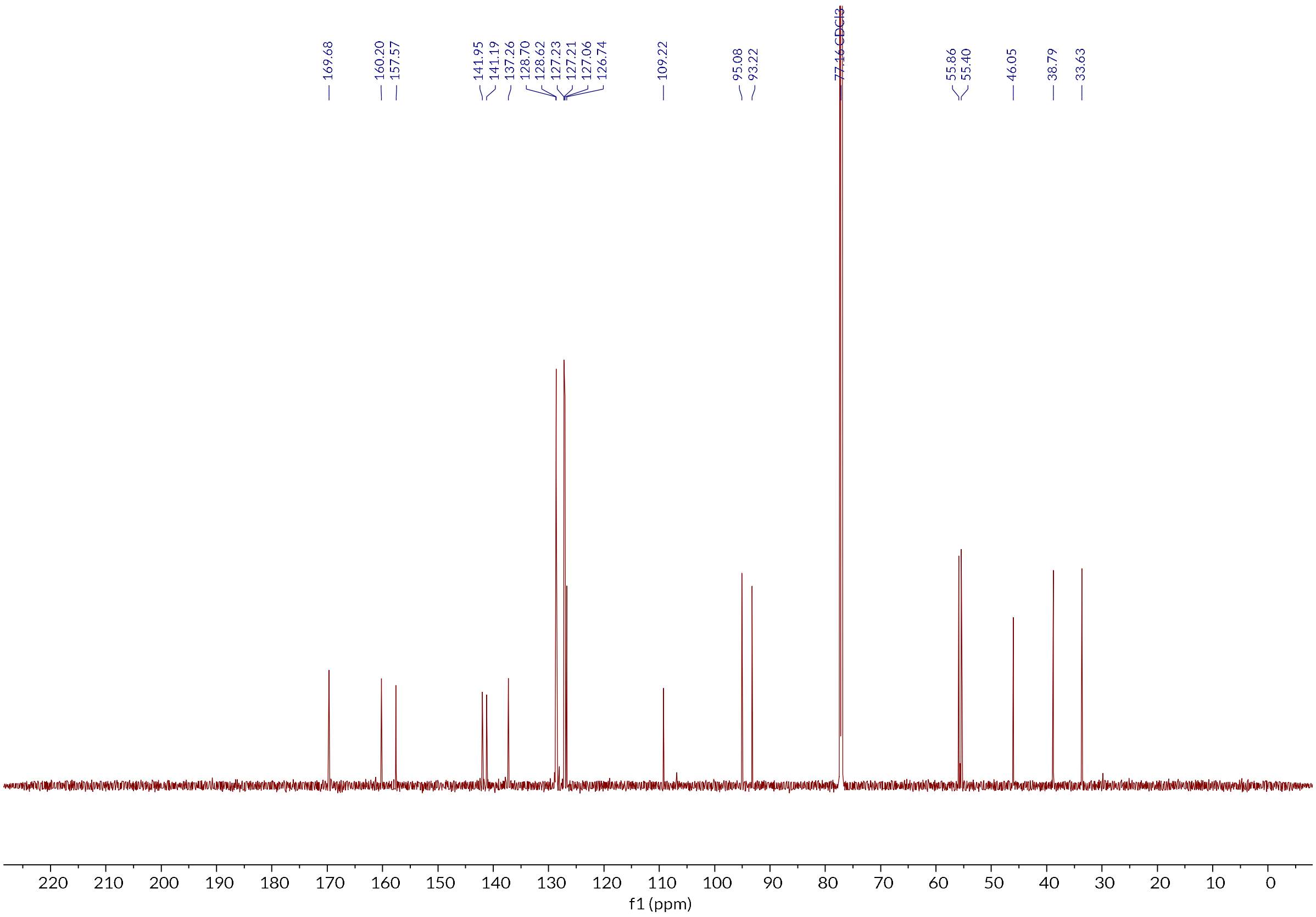

(±)-1-Benzyl-4-(*p*-tolyl)-1,2,3,4-tetrahydroquinoline (2an)

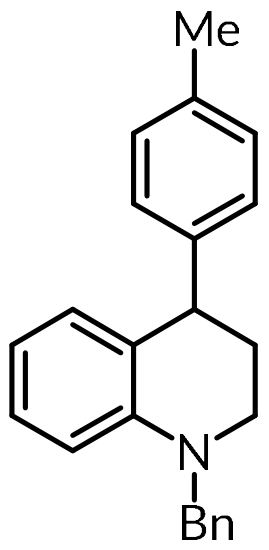

(2an)

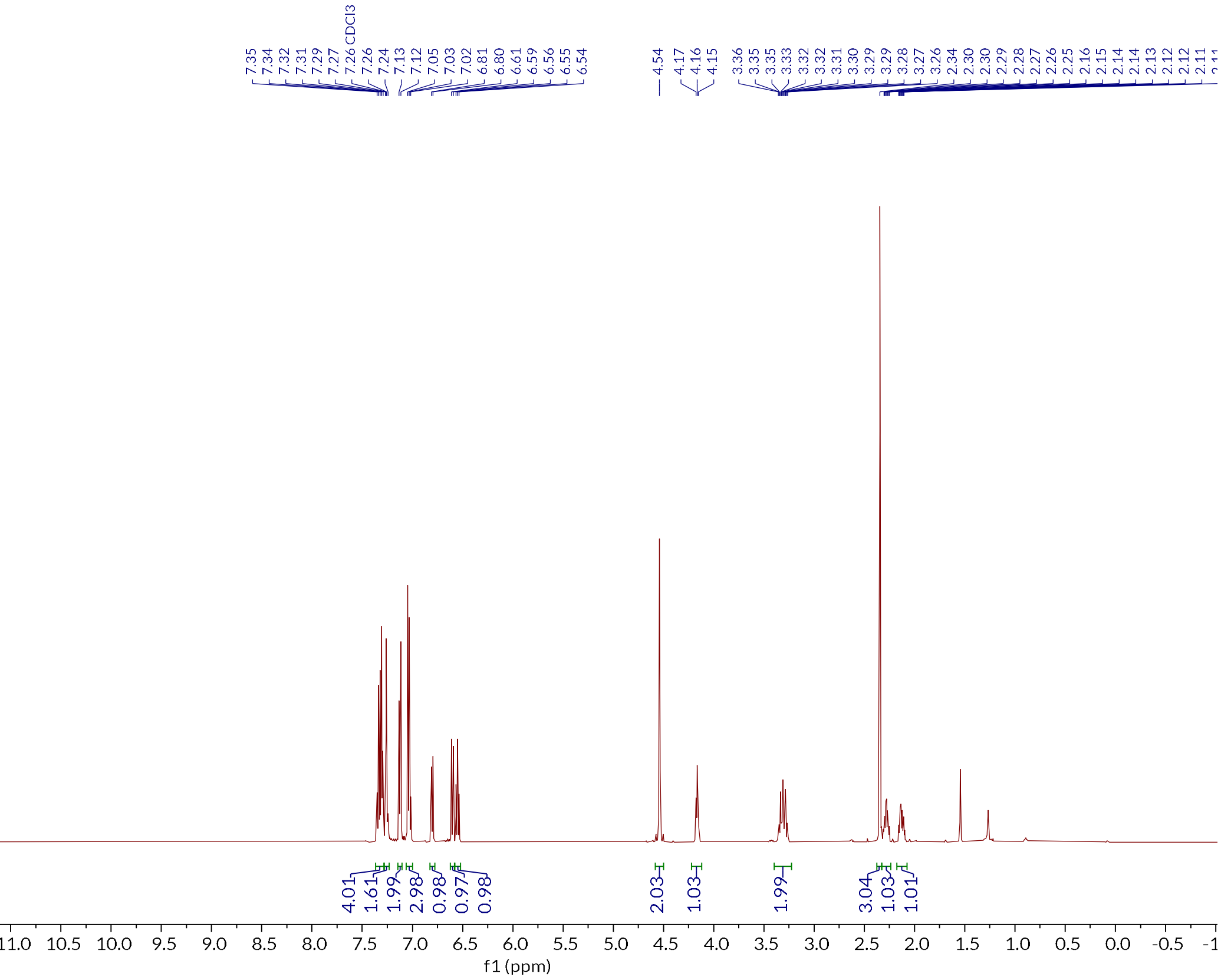

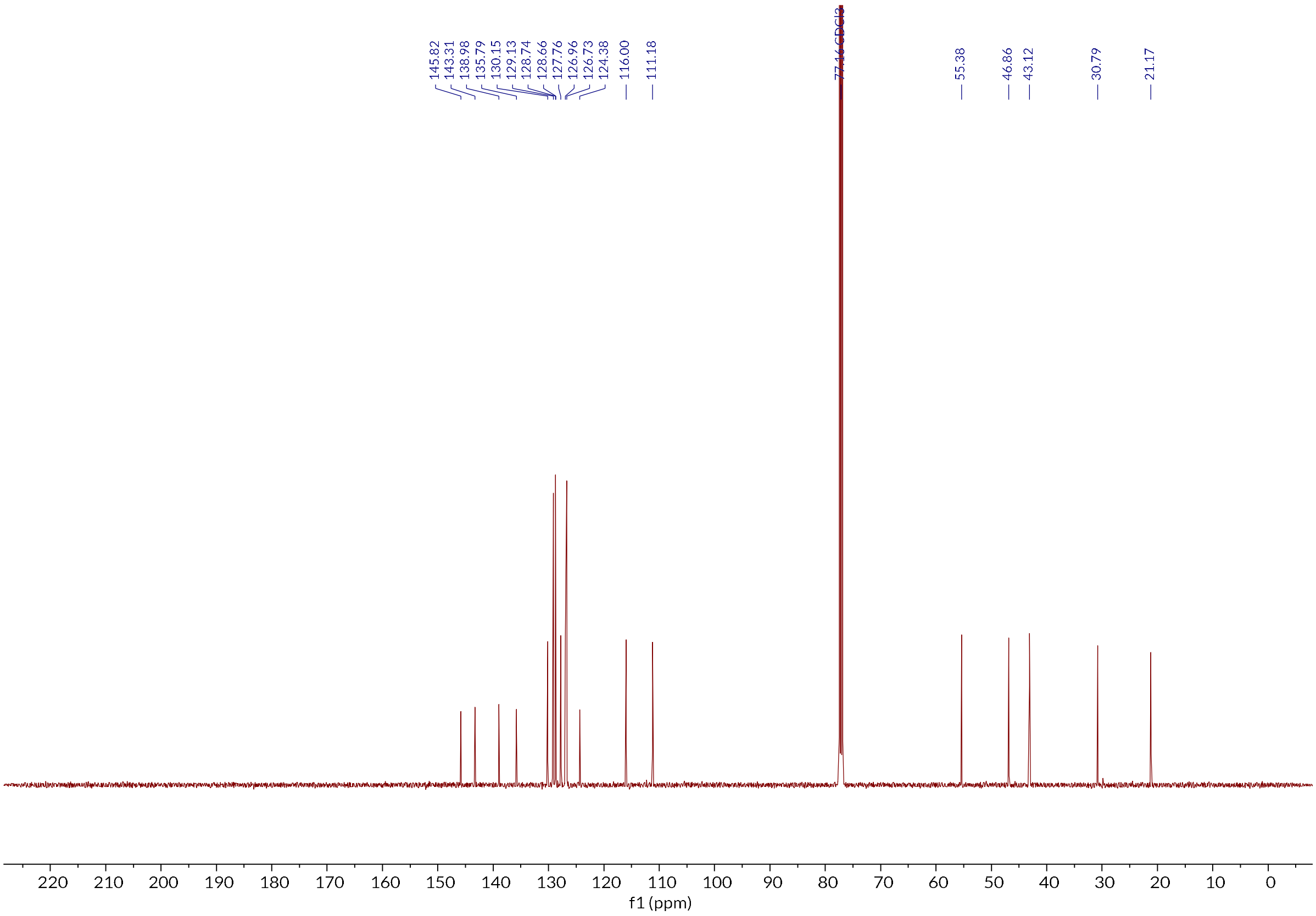

(±)-1-Benzyl-4-(4-methoxyphenyl)-1,2,3,4-tetrahydroquinoline (2ao)

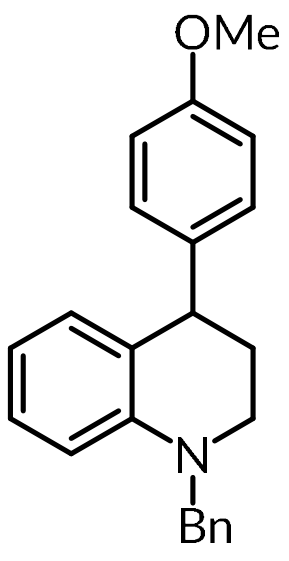

(2ao)

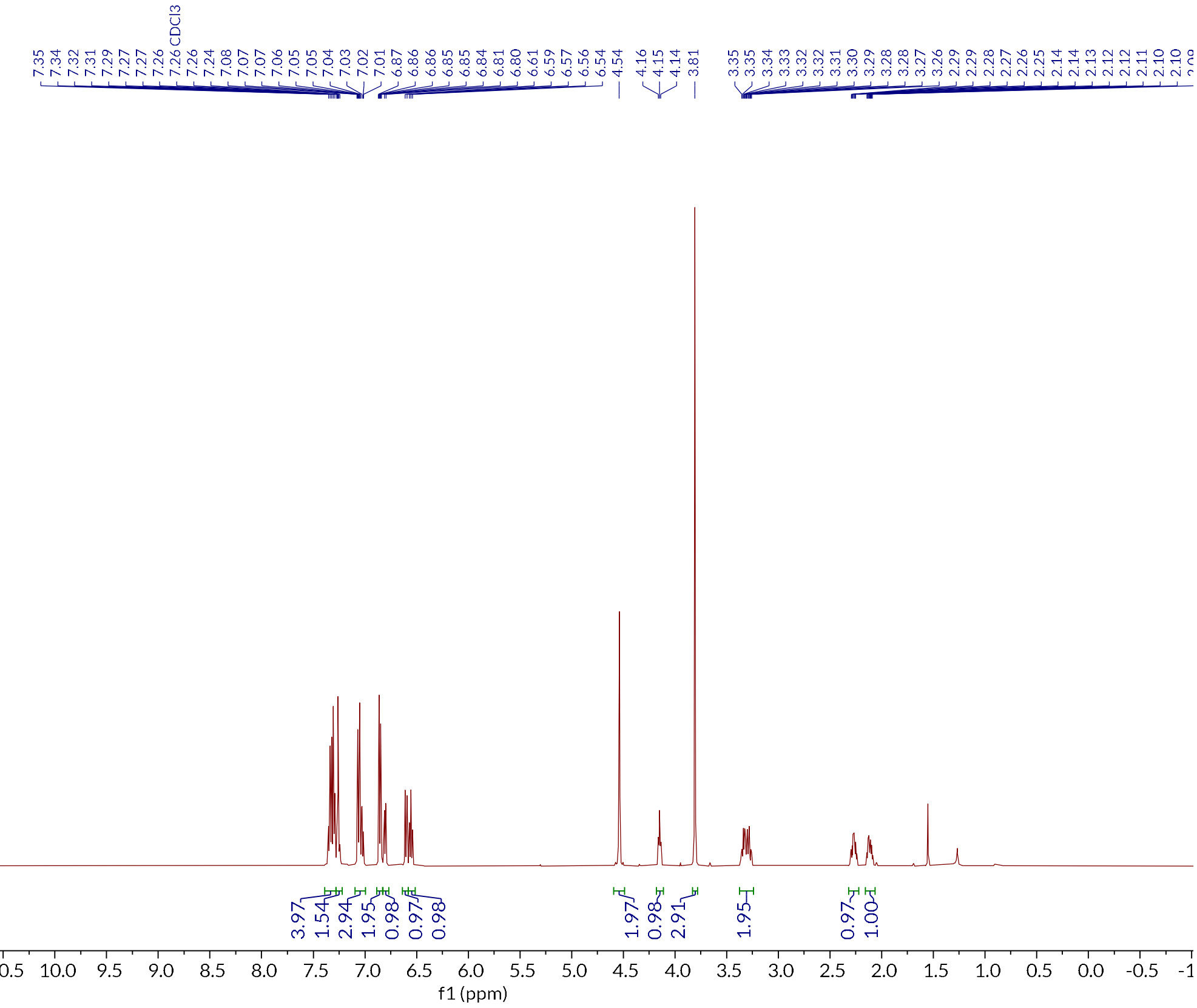

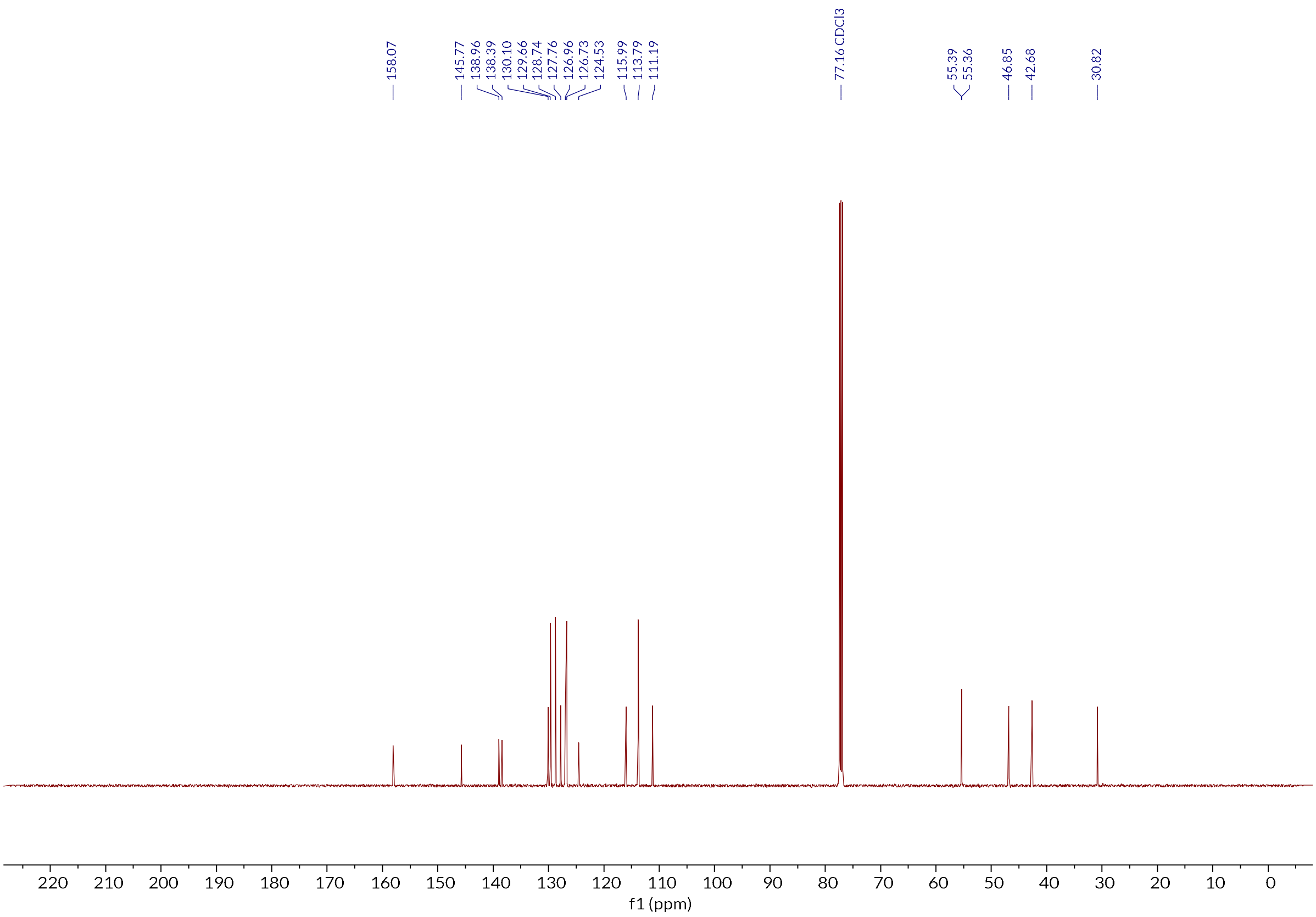

(±)-1-Benzyl-4-(4-fluorophenyl)-1,2,3,4-tetrahydroquinoline (2ap)

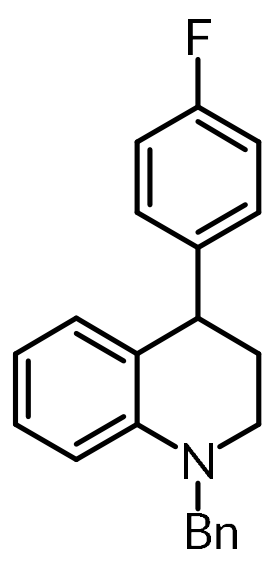

(2ap)

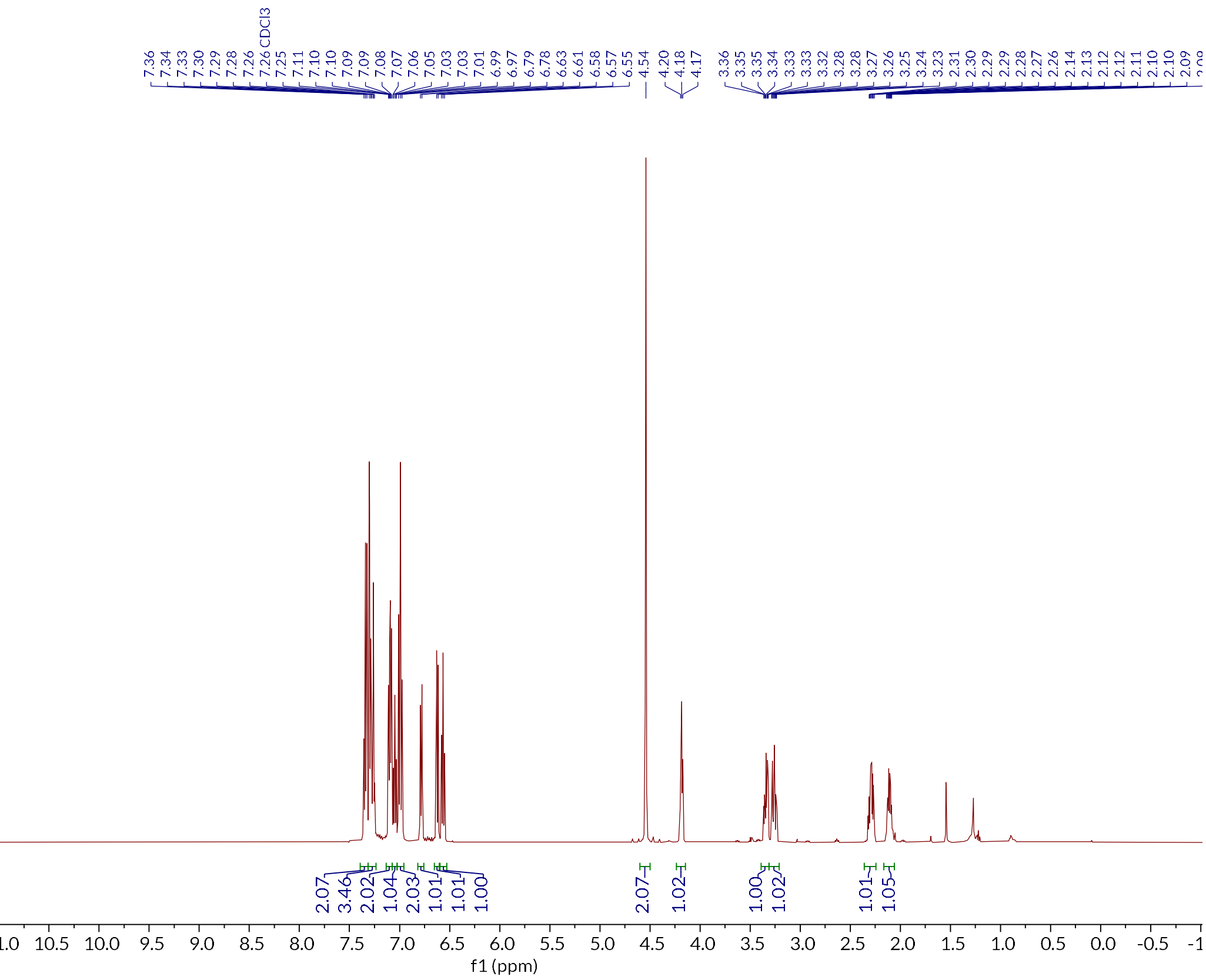

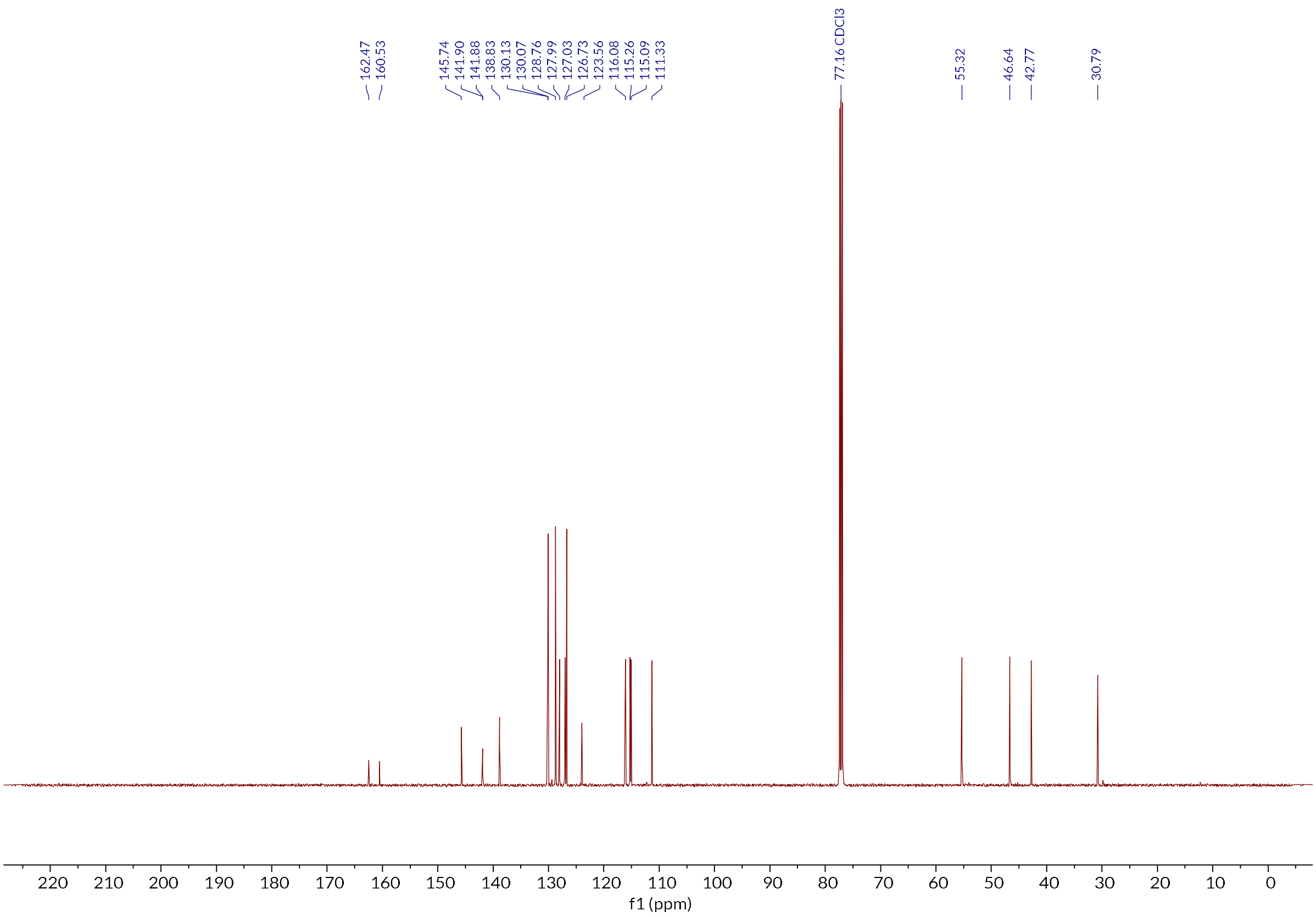

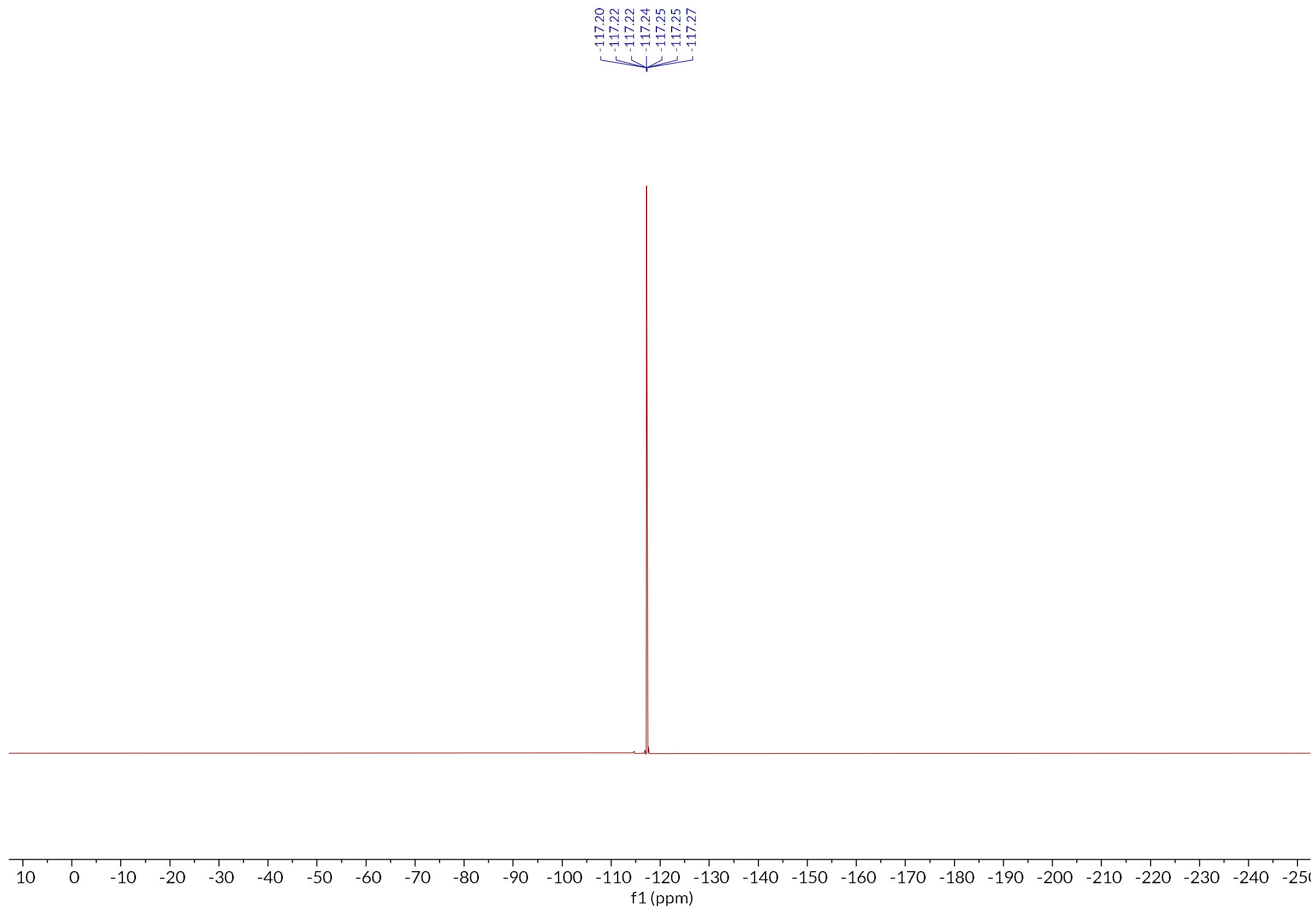
-117.20
-117.22
-117.22
-117.24
-117.25
-117.25
-117.27
10 0 -10 -20 -30 -40 -50 -60 -70 -80 -90 -100 -110 -120 -130 -140 -150 -160 -170 -180 -190 -200 -210 -220 -230 -240
f1 (ppm)

(±)-1-Benzyl-4-(4-bromophenyl)-1,2,3,4-tetrahydroquinoline (2aq)

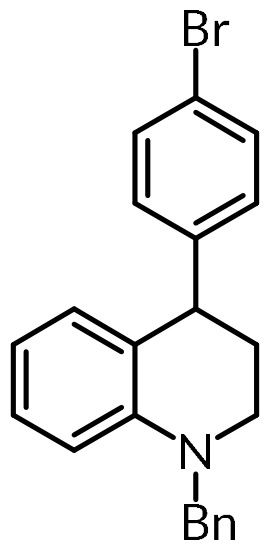

(2an)

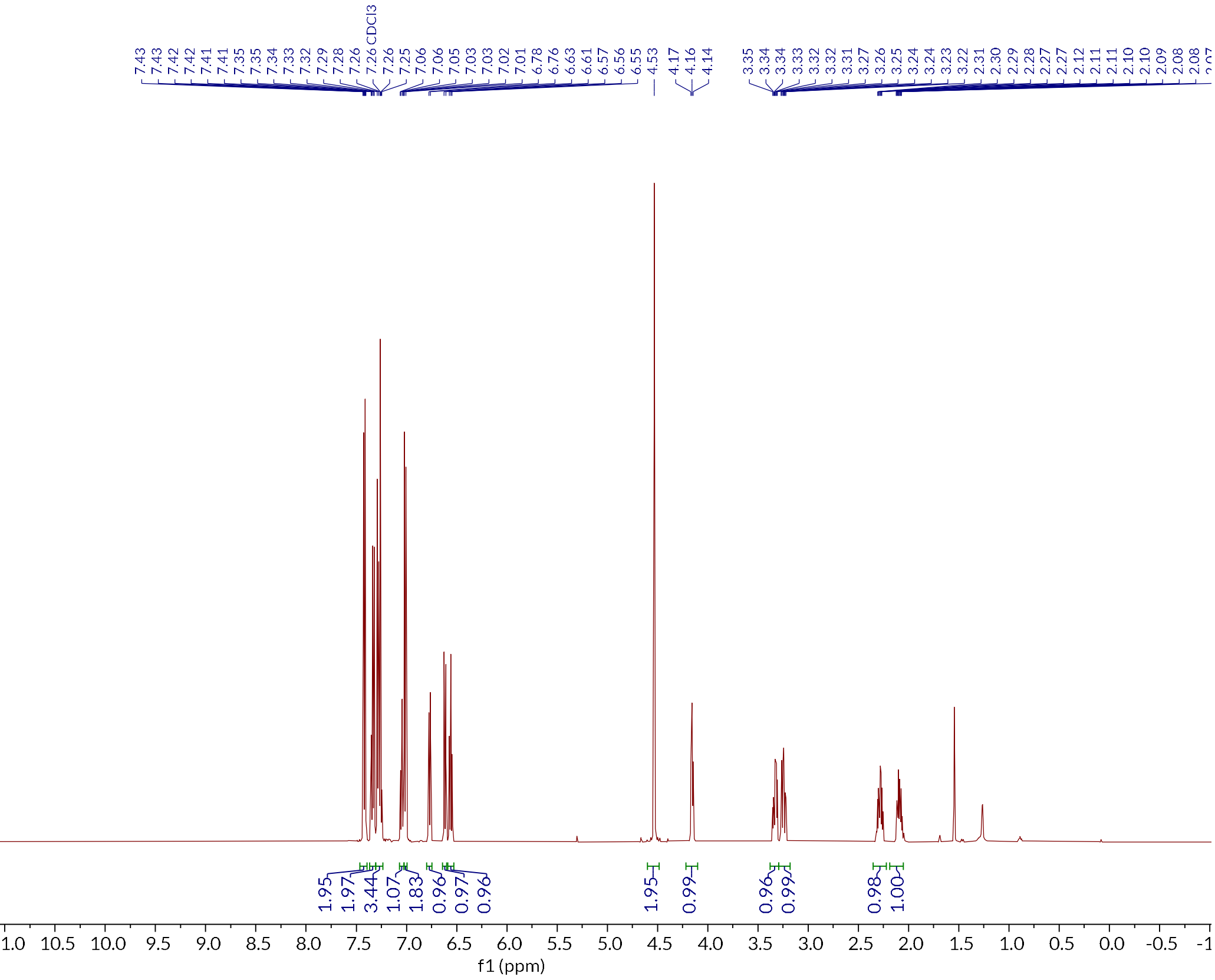

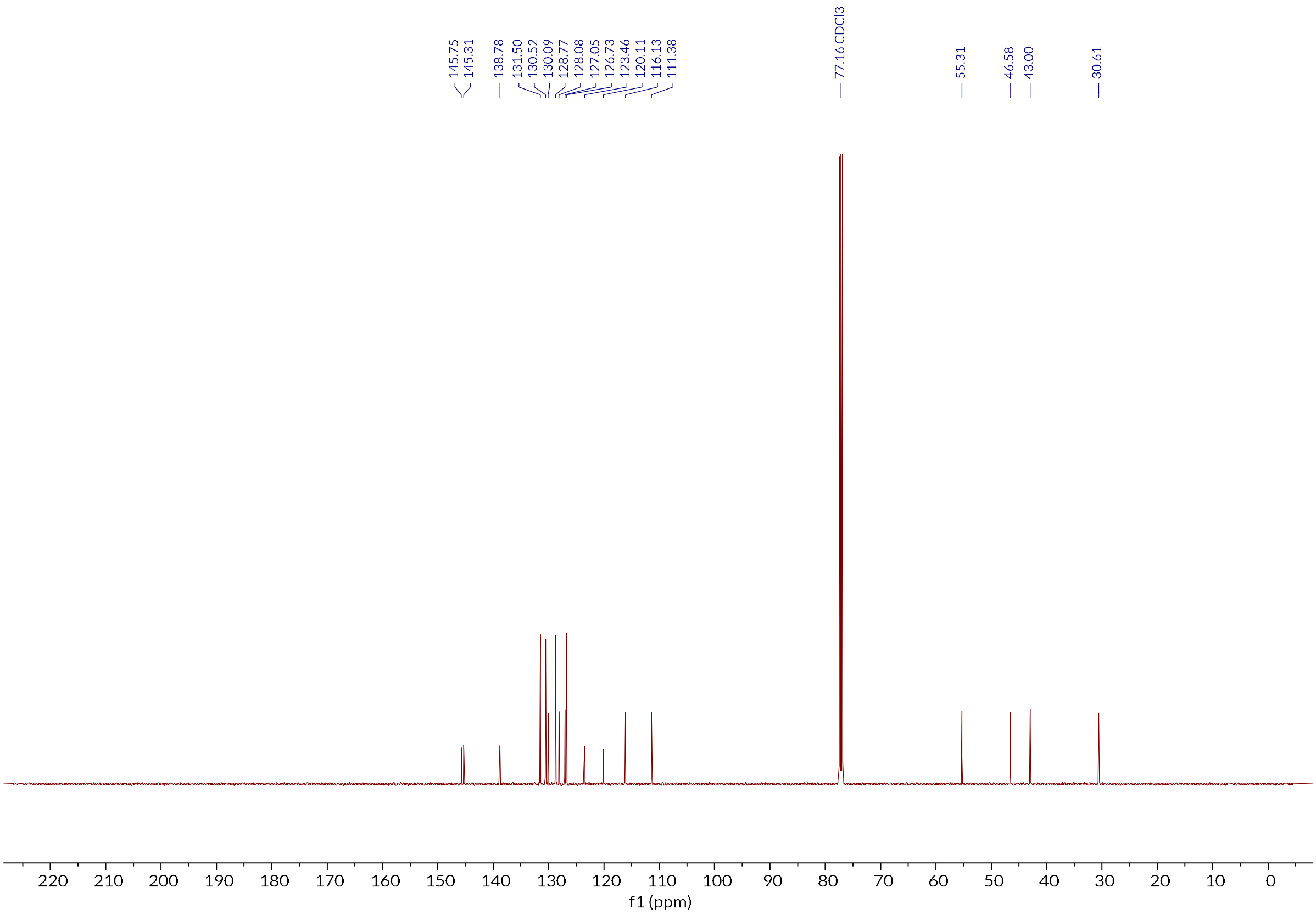

(±)-1-Benzyl-4-(4-trifluoromethylphenyl)-1,2,3,4-tetrahydroquinoline (2ar)

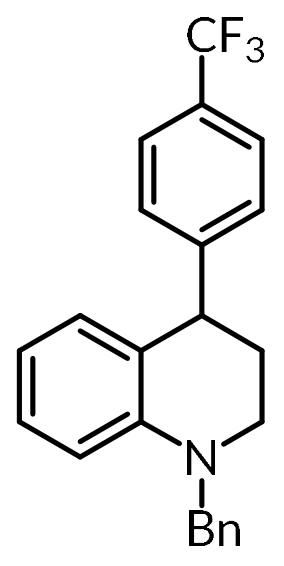

(2ar)

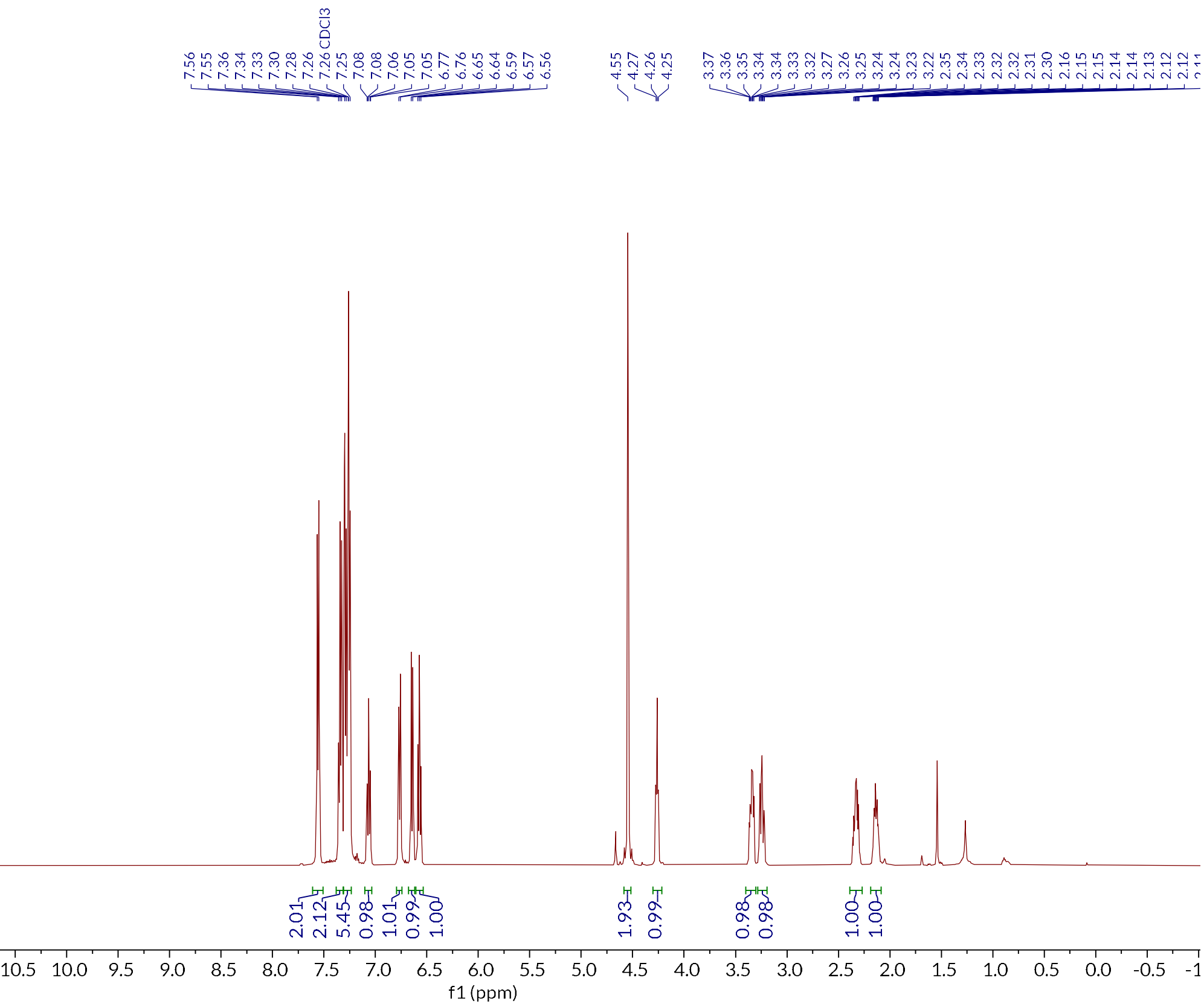

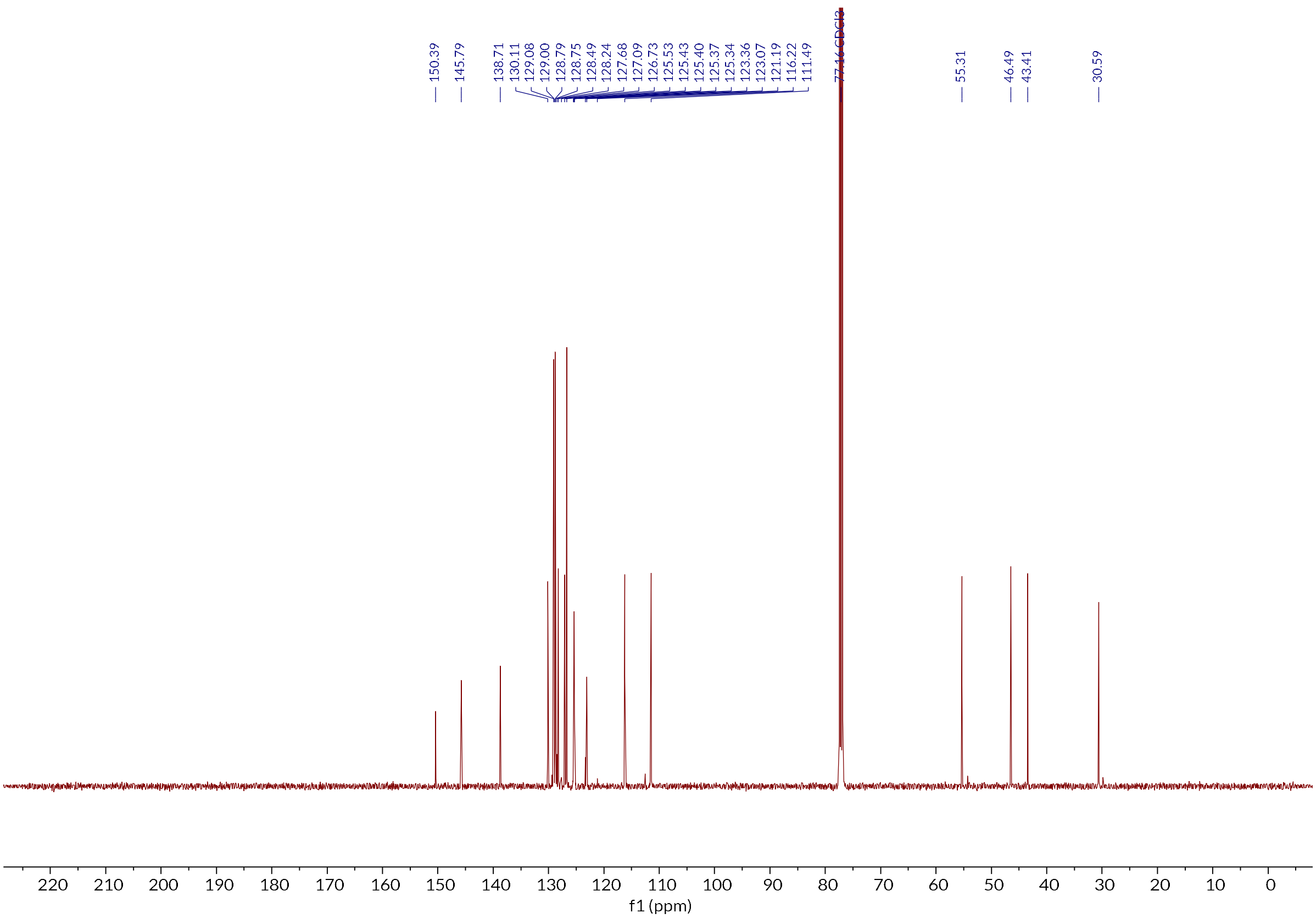

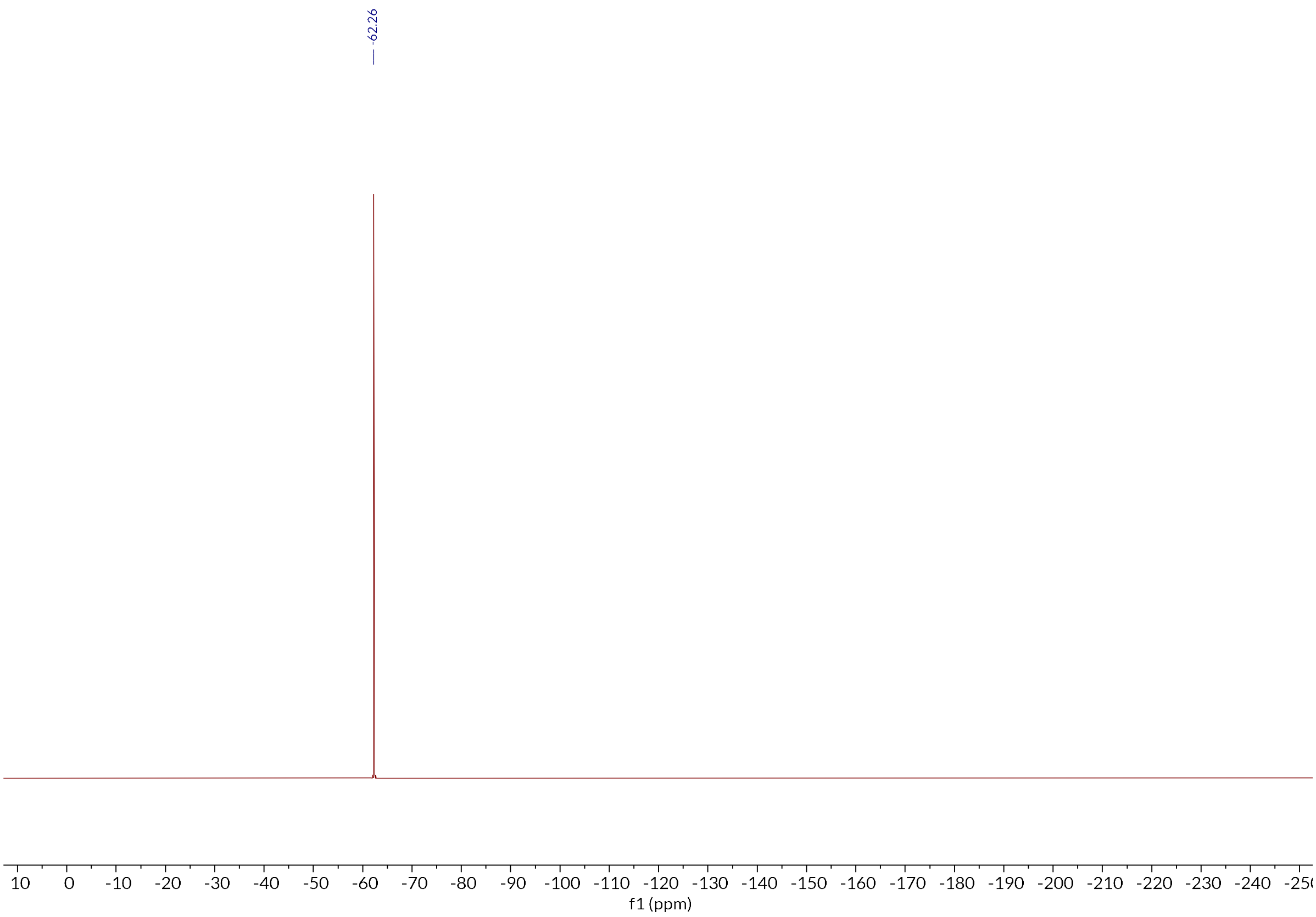
-62.26
10
0
-10
-20
-30
-40
-50
-60
-70
-80
-90
-100
-110
-120
-130
-140
-150
-160
-170
-180
-190
-200
-210
-220
-230
-240
f1 (ppm)

(±)-Methyl 3-(1-benzyl-1,2,3,4-tetrahydroquinolin-4-yl)benzoate (2as)

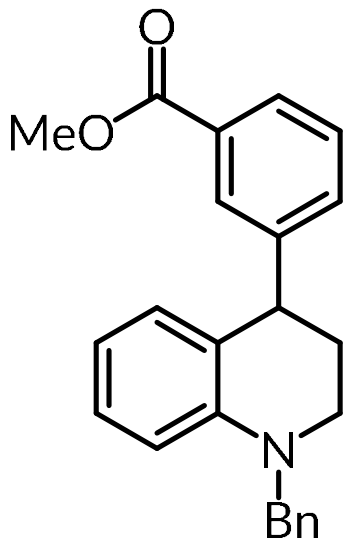

(2as)

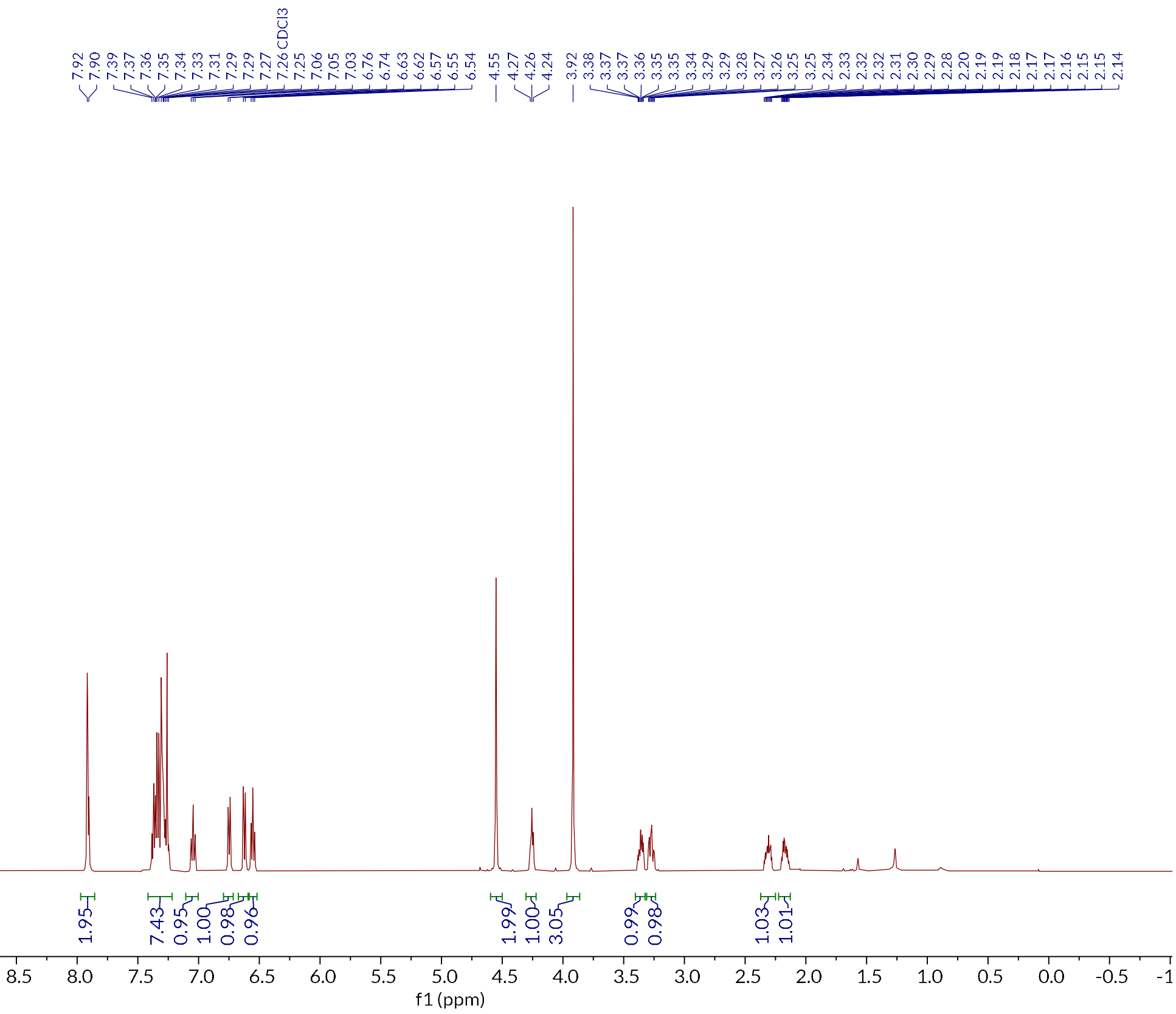

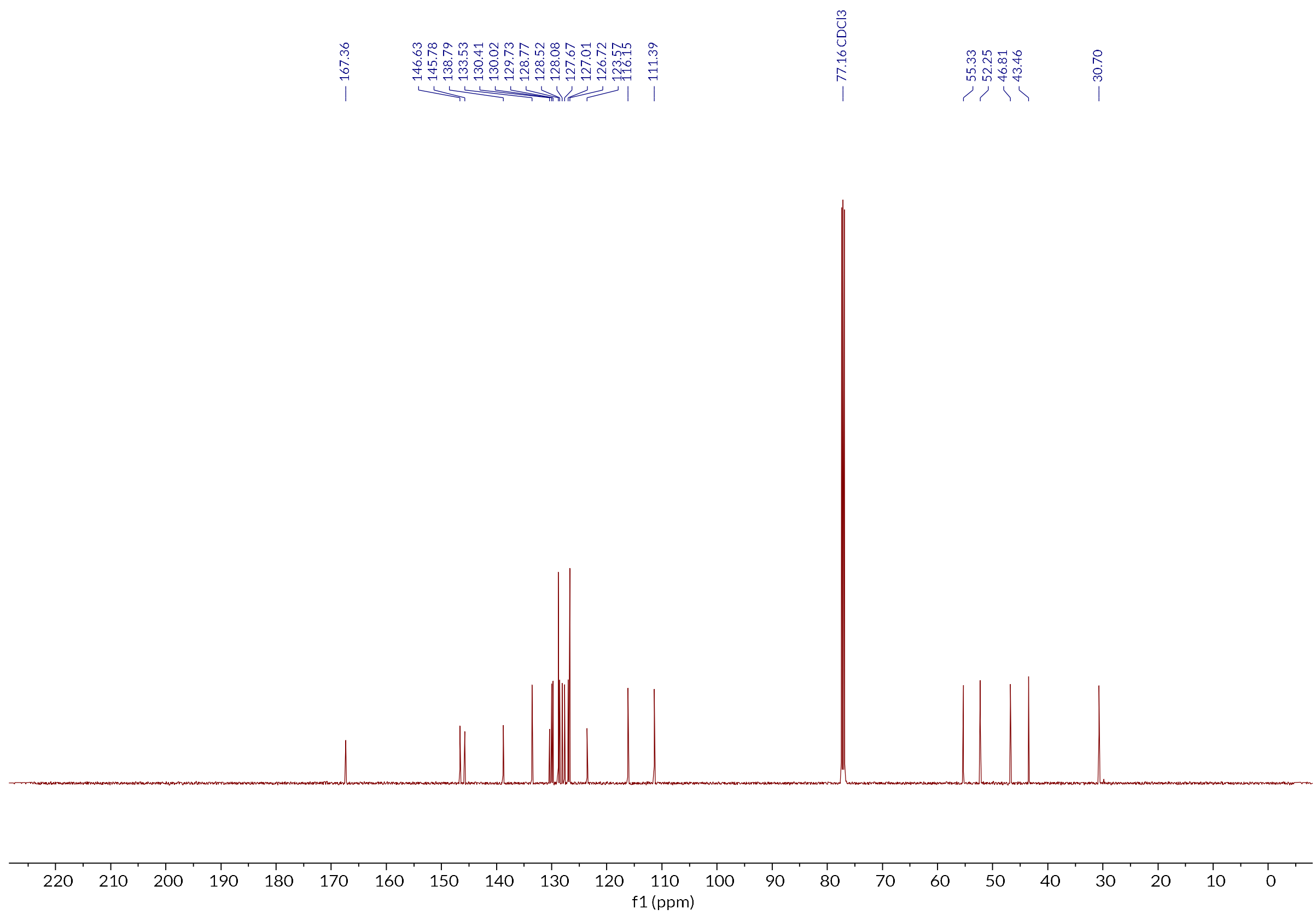

(±)-1-Benzyl-4-(3,5-di-*tert*-butylphenyl)-1,2,3,4-tetrahydroquinoline (2at)

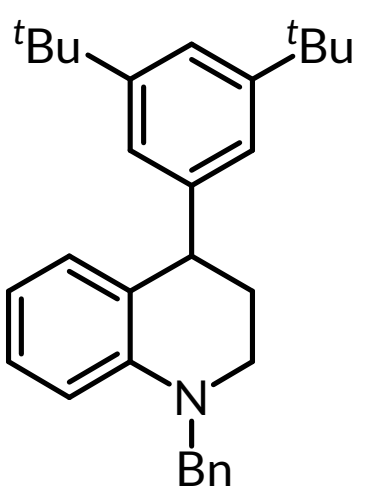

(2at)

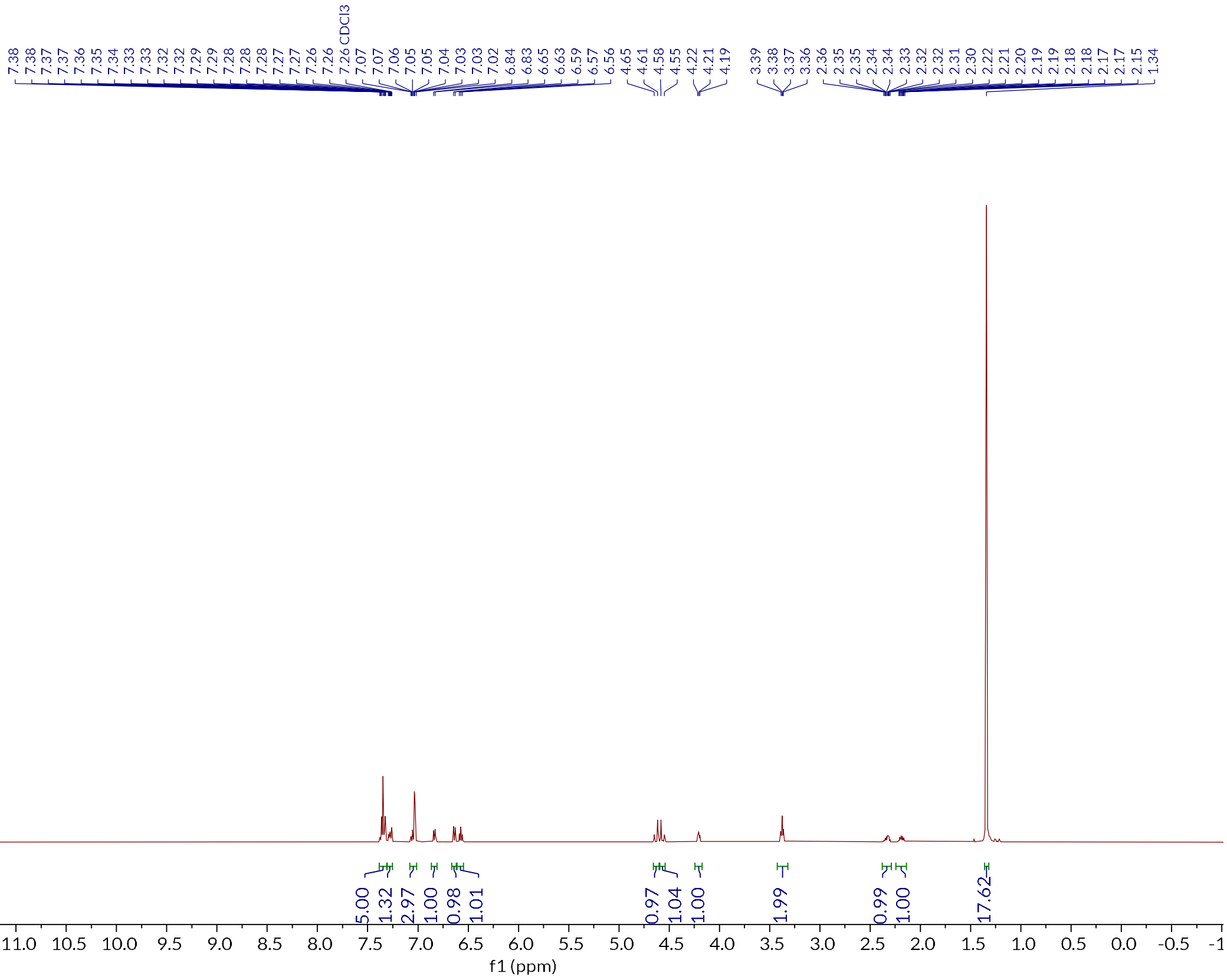

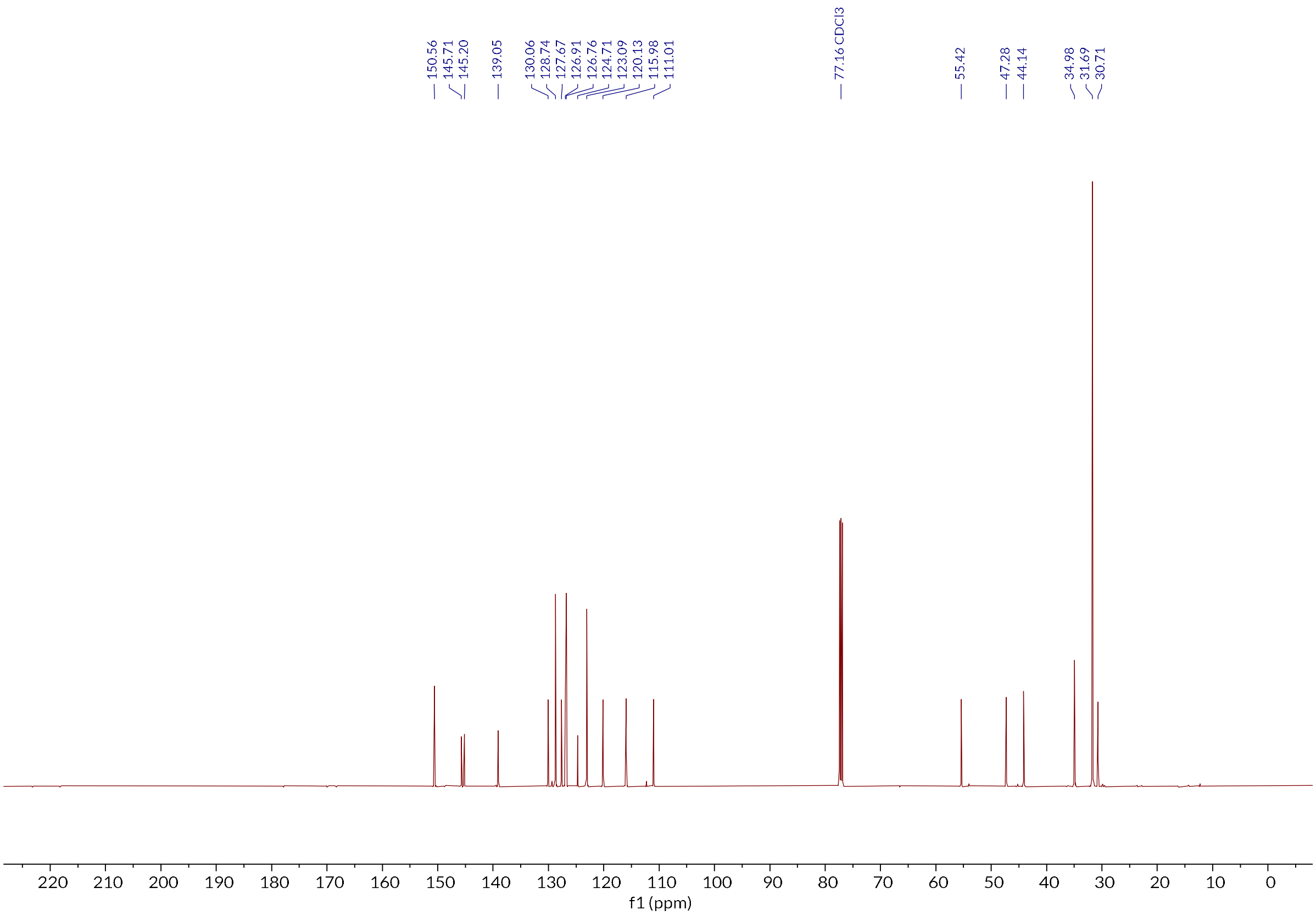

(±)-1-Benzyl-4-(2-bromo-5-chlorophenyl)-1,2,3,4-tetrahydroquinoline (2au)

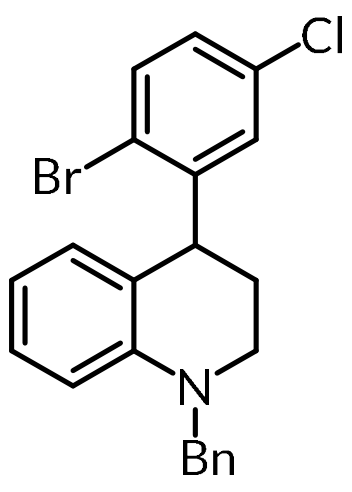

(2au)

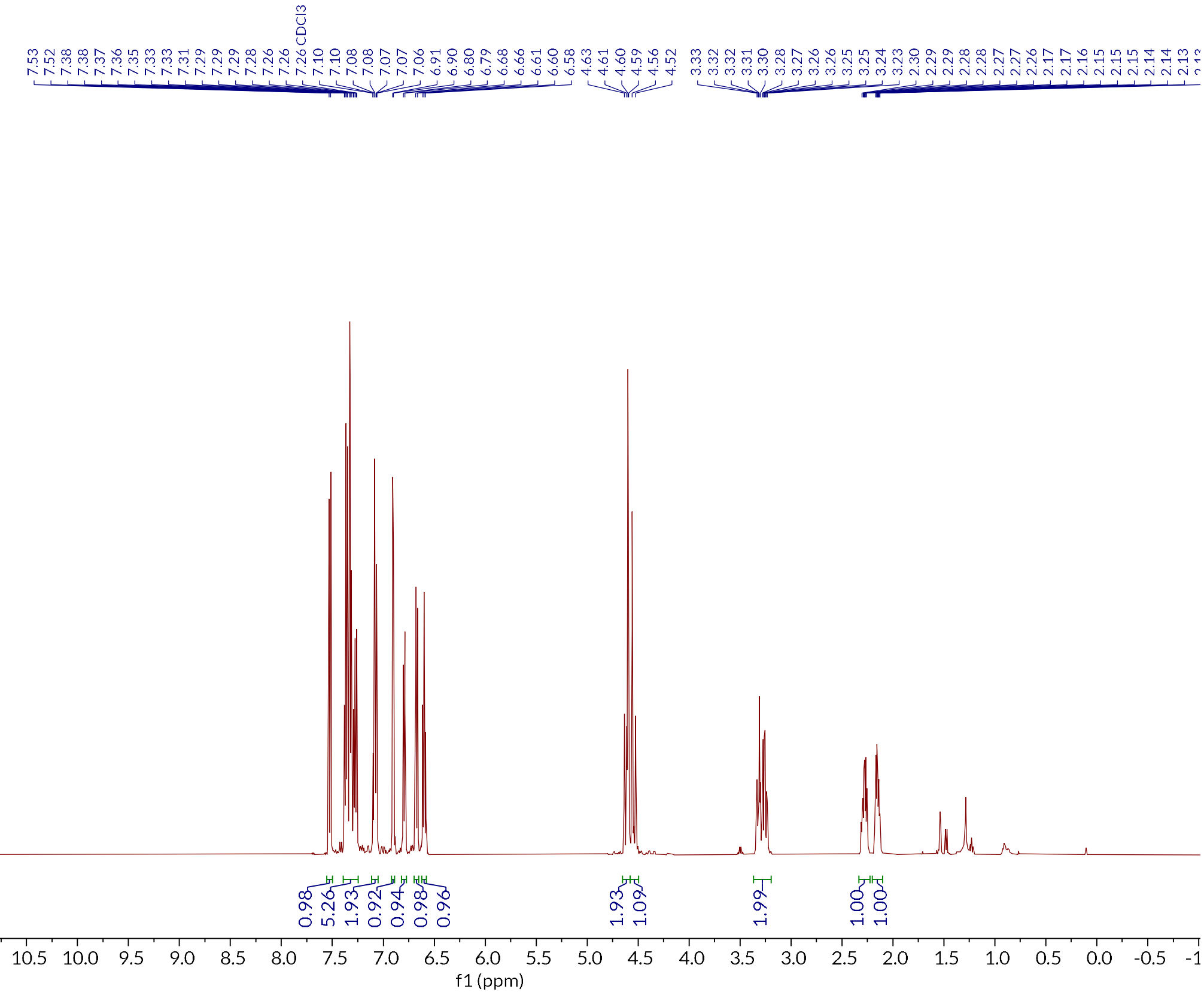

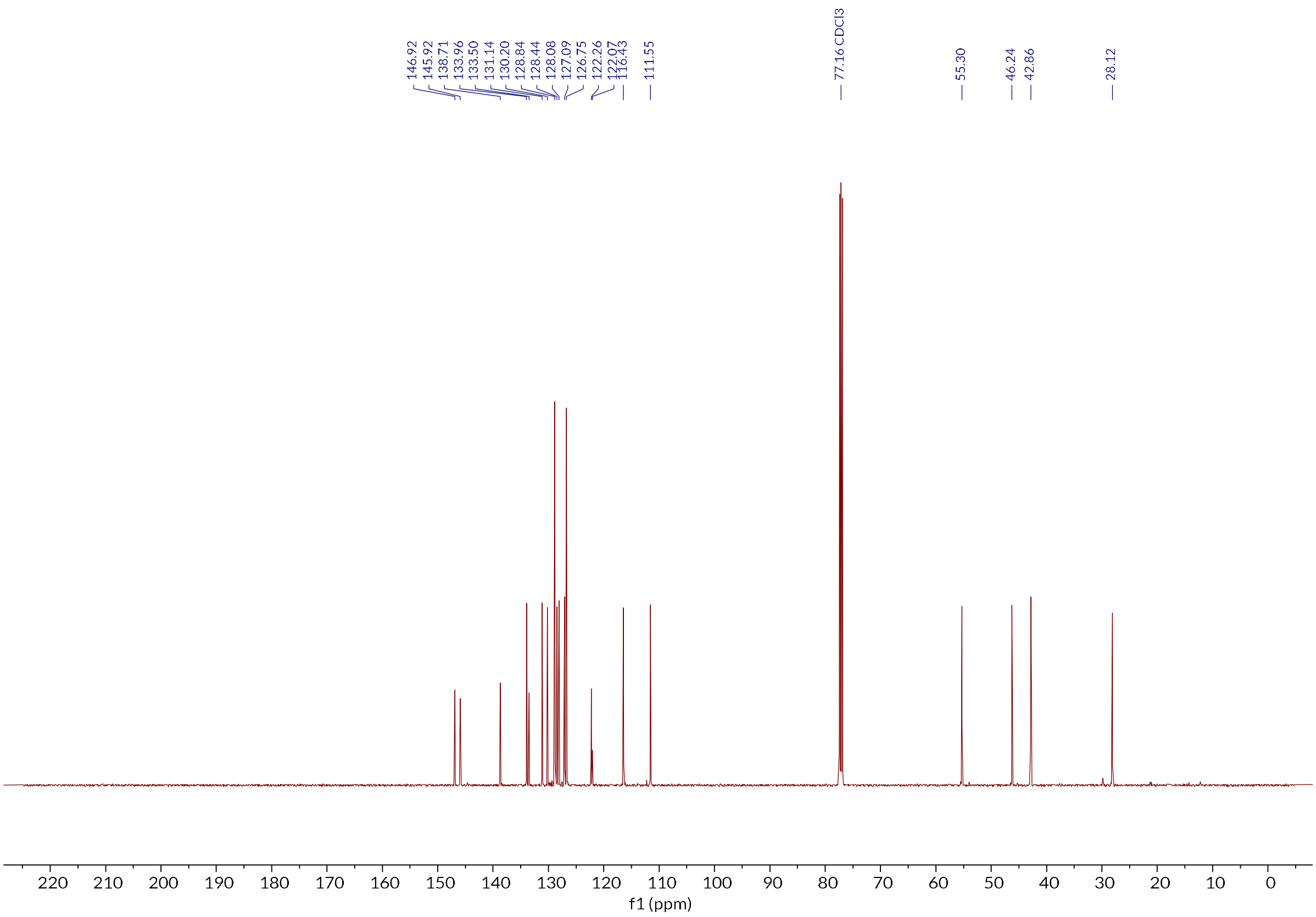

(±)-1-Benzyl-4-(2-(trifluoromethoxy)phenyl)-1,2,3,4-tetrahydroquinoline (2av)

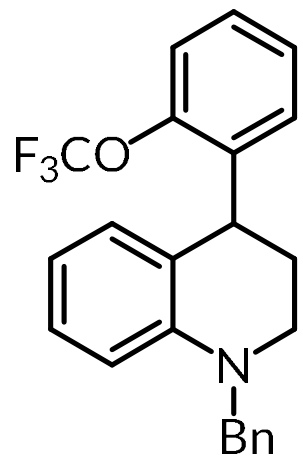

(2av)

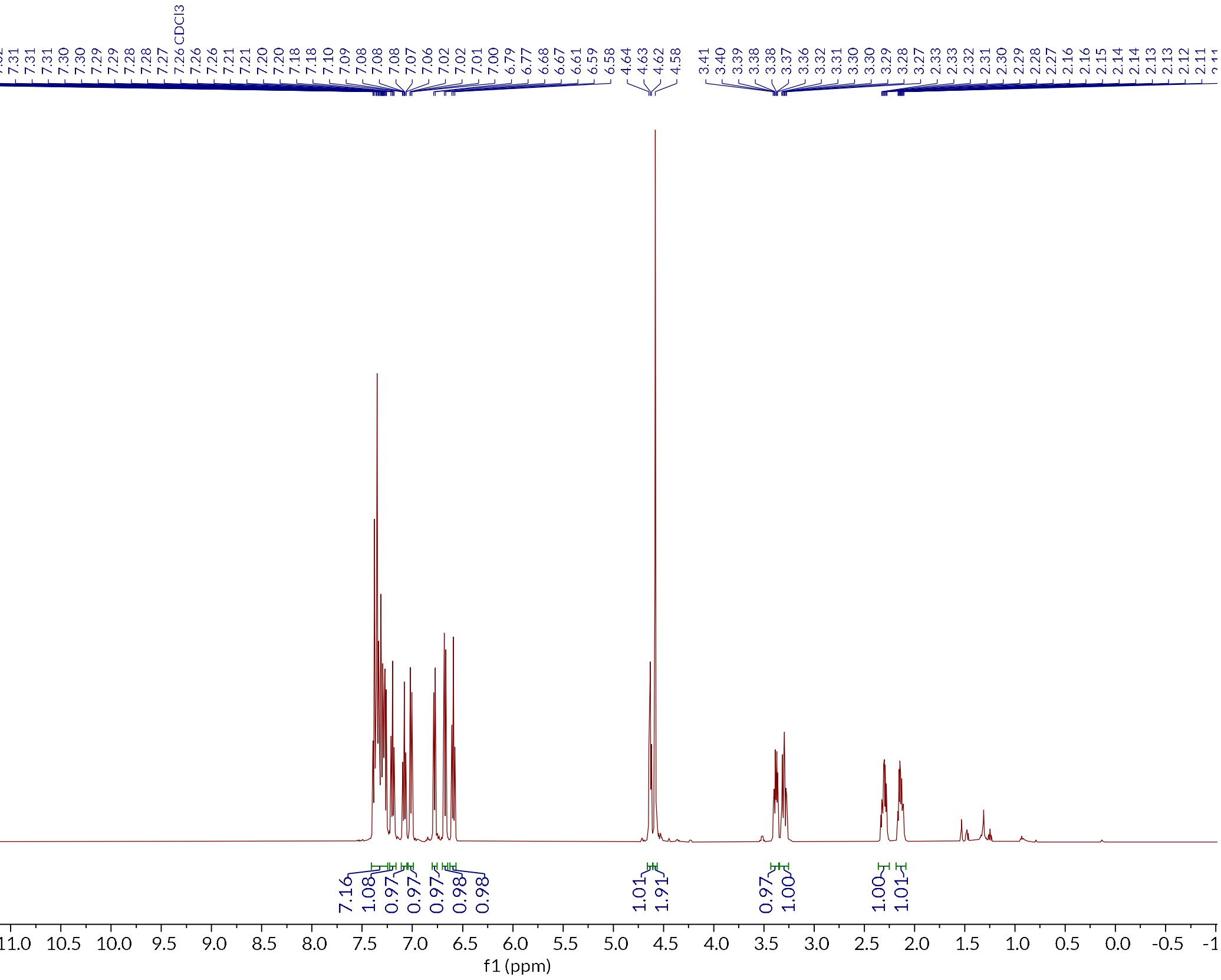

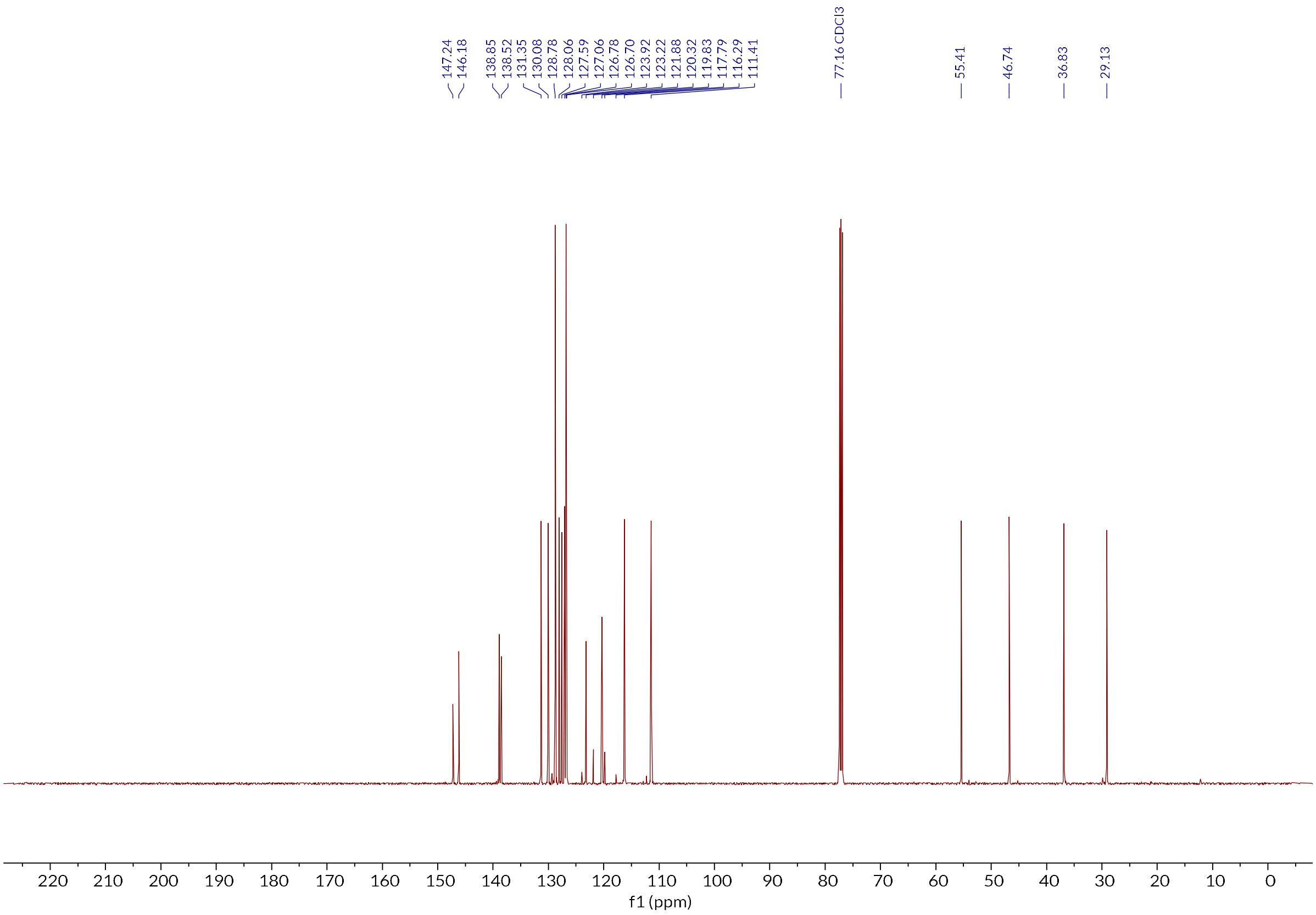

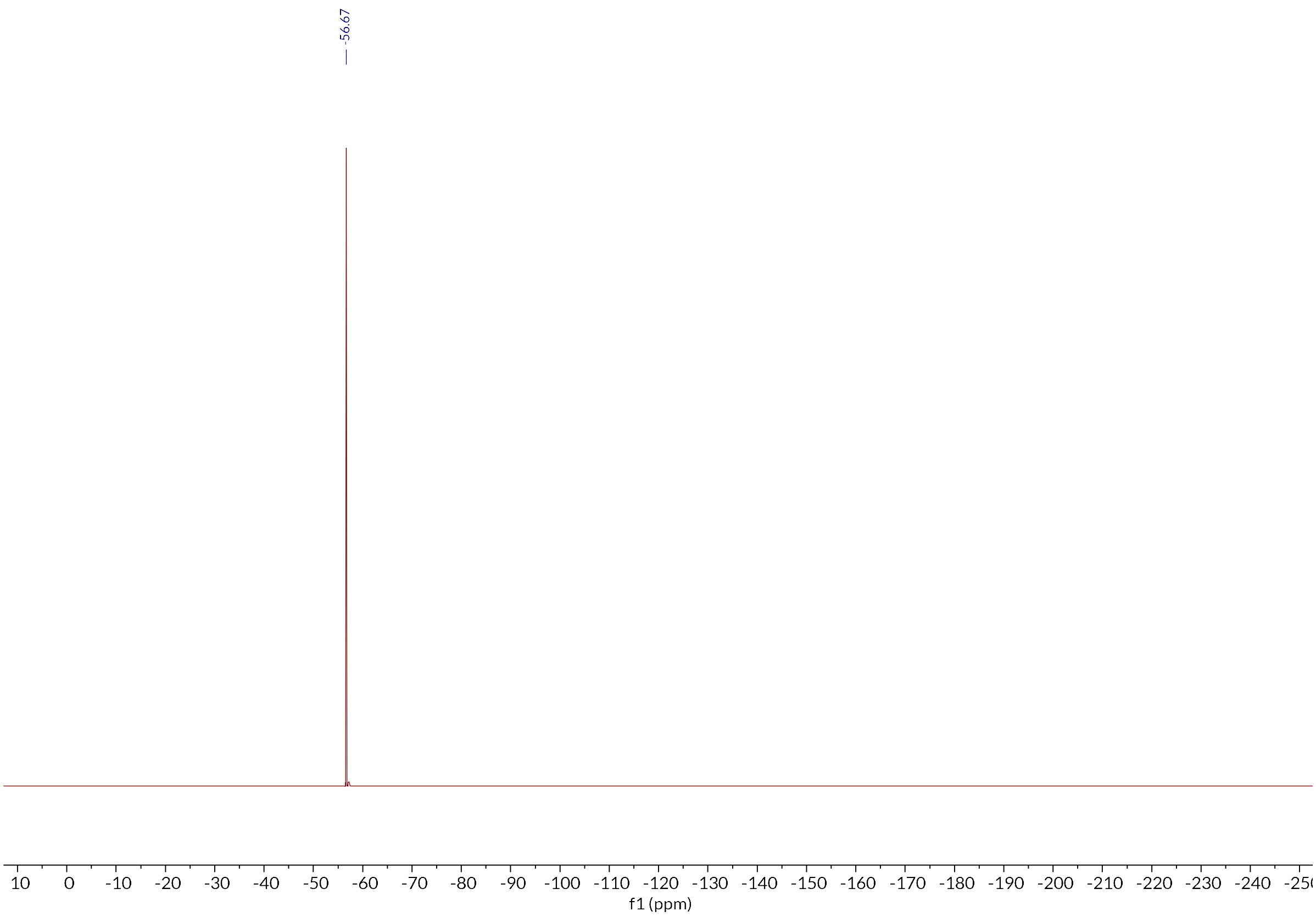
-56.67
10 0 -10 -20 -30 -40 -50 -60 -70 -80 -90 -100 -110 -120 -130 -140 -150 -160 -170 -180 -190 -200 -210 -220 -230 -240 -250
f1 (ppm)

(±)-1-Benzyl-4-(4-furan-2-yl)-1,2,3,4-tetrahydroquinoline (2aw)

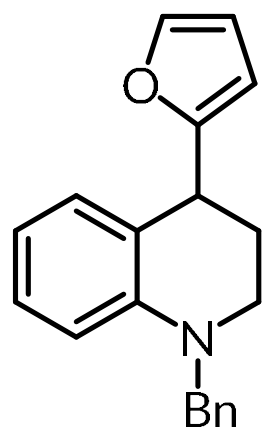

(2aw)

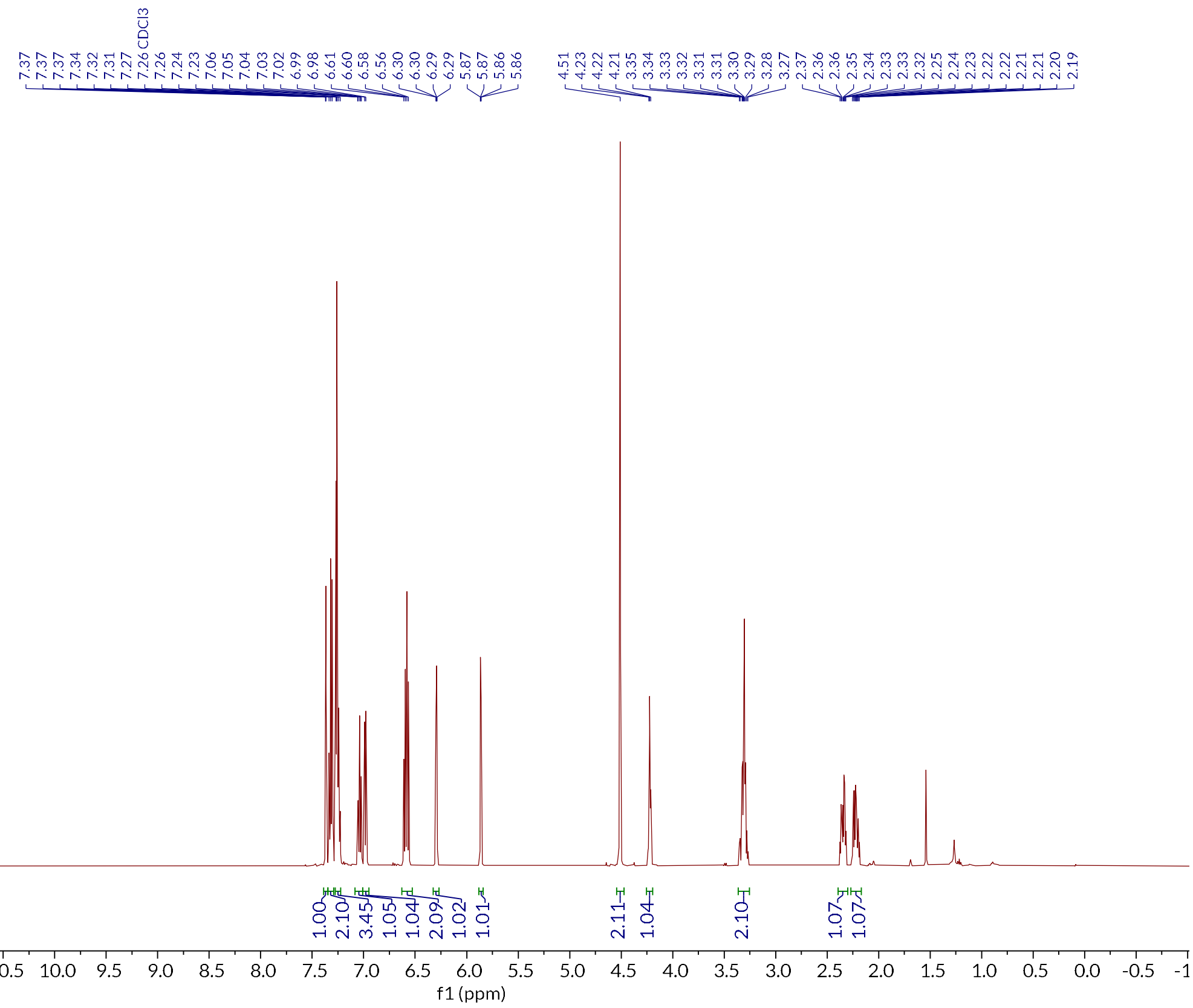

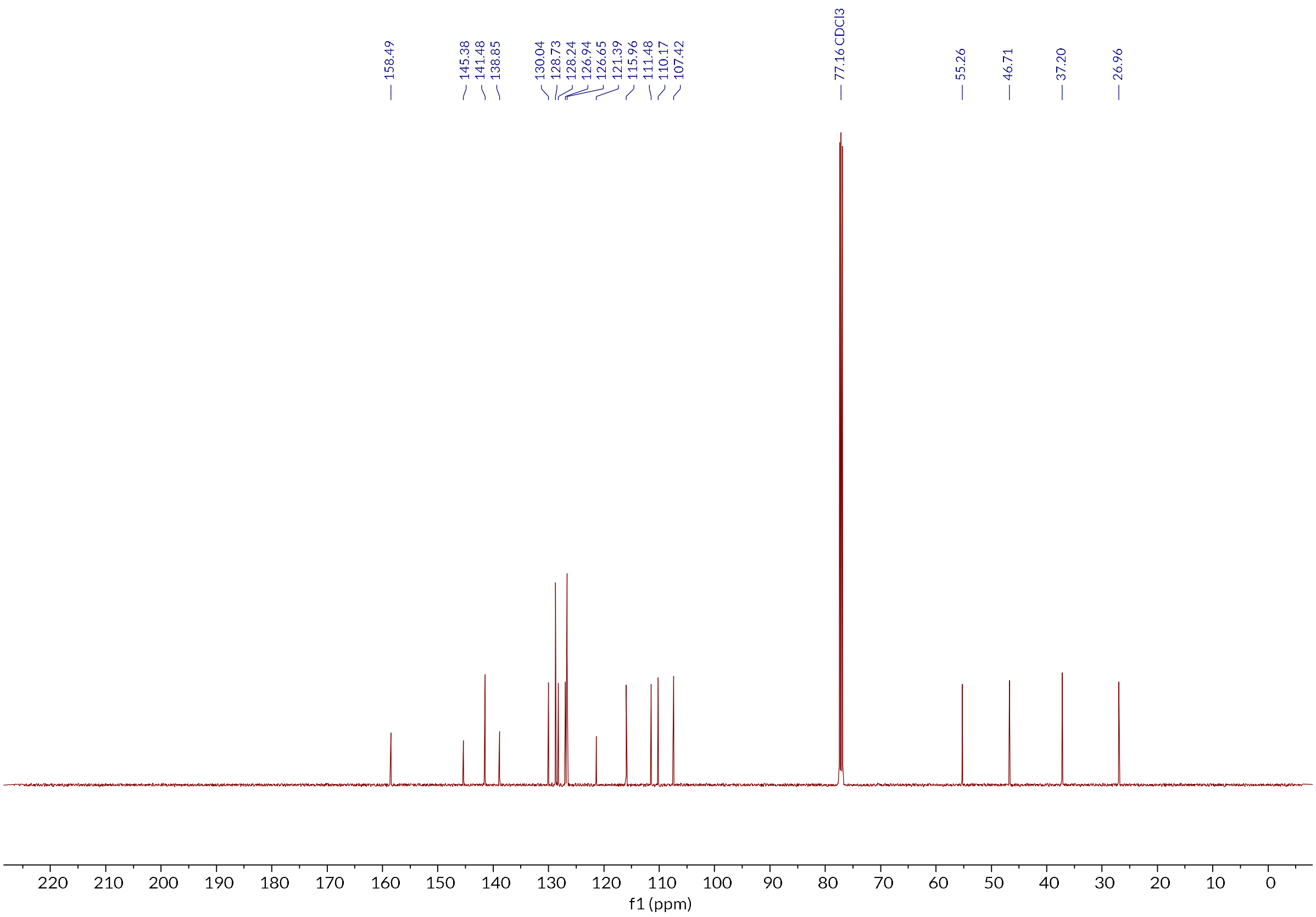

(±)-4-(Benzo[*b*]thiophen-3-yl)-1-benzyl-1,2,3,4-tetrahydroquinoline (2ax)

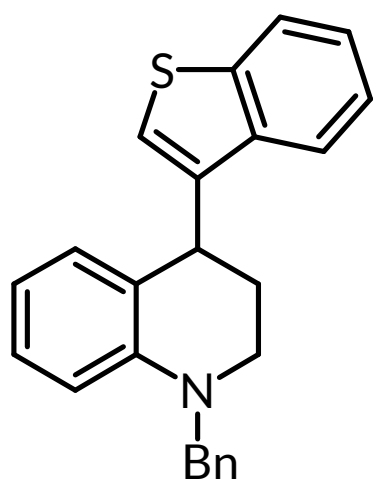

(2ax)

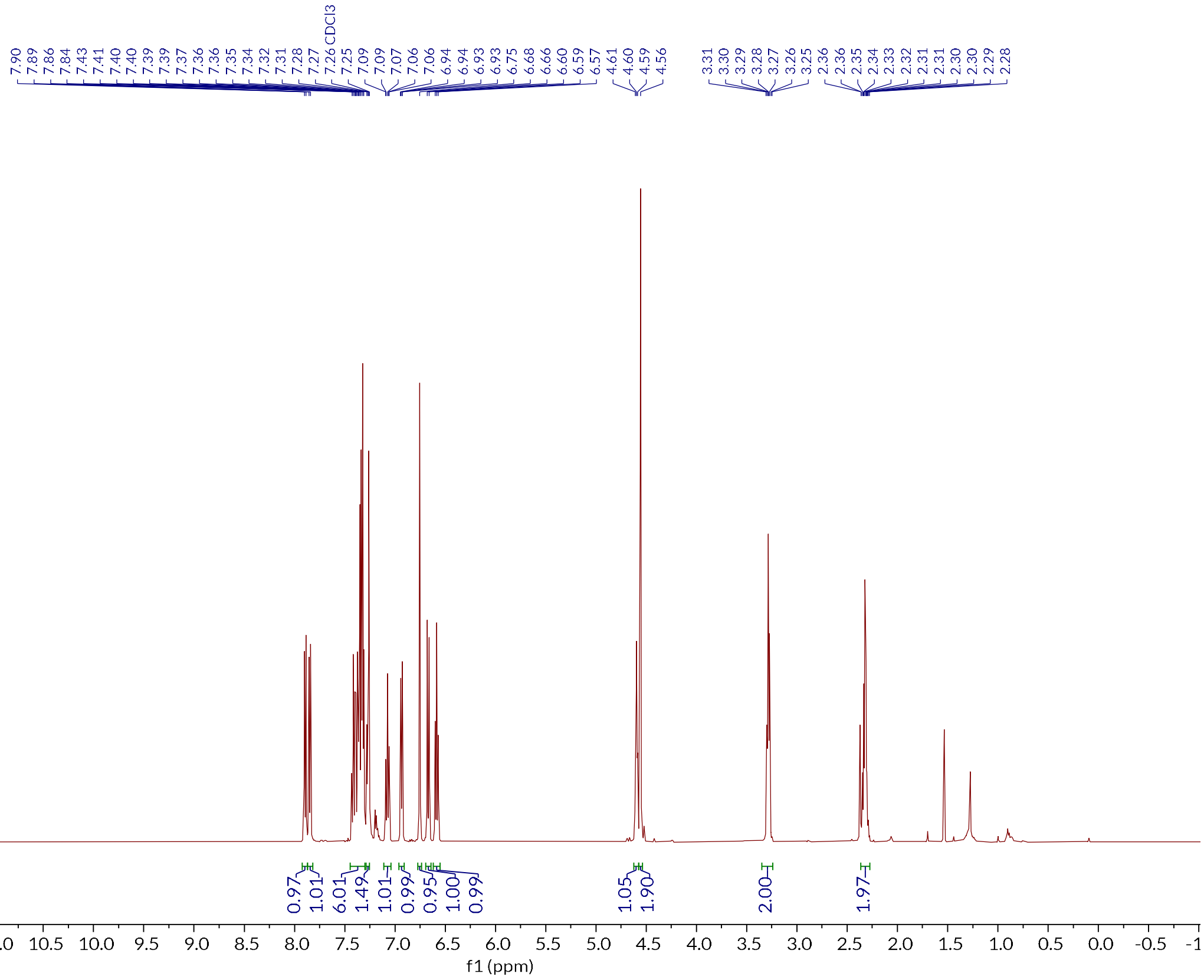

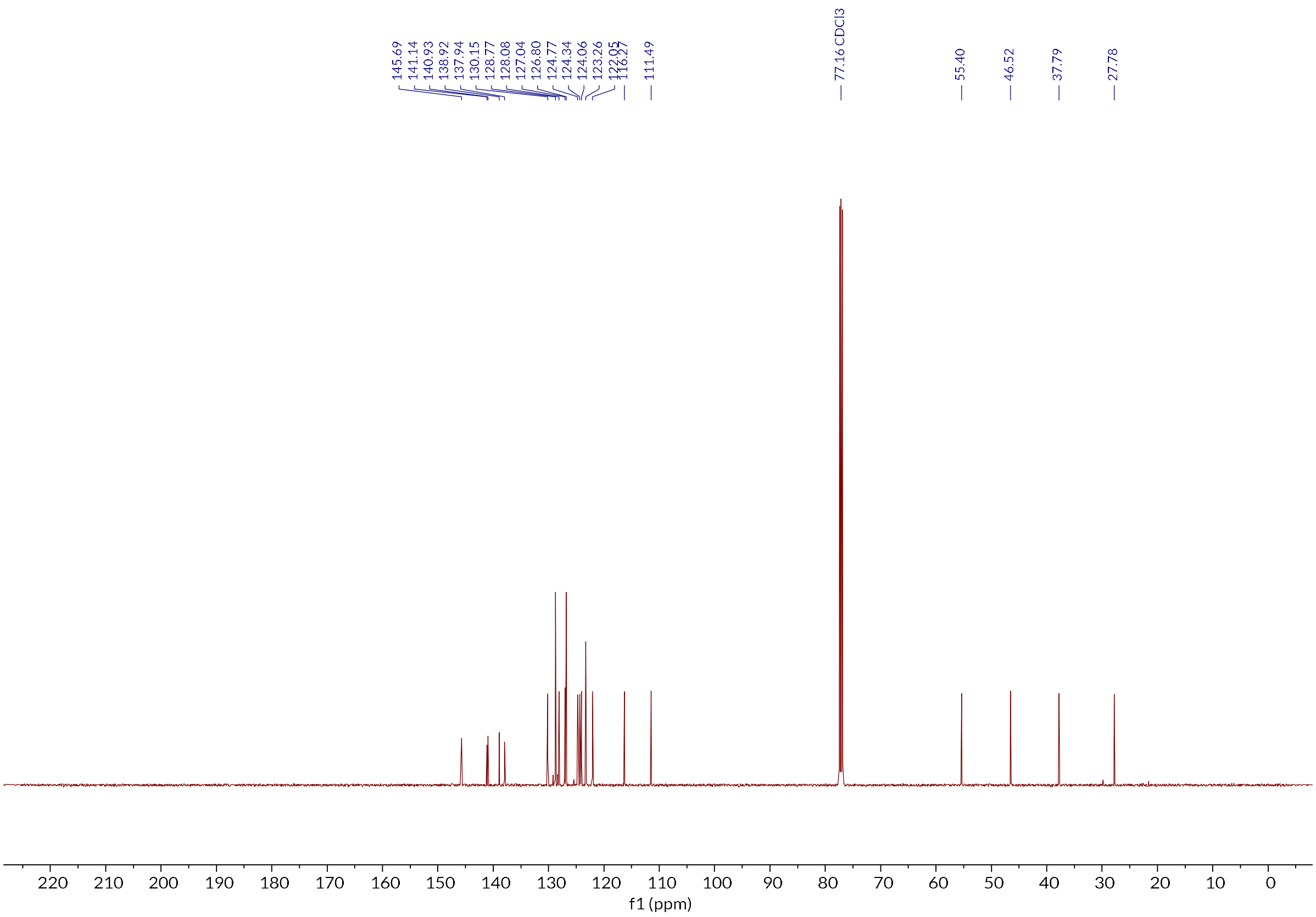

(±)-1-Benzyl-4-(3-(4-fluorophenyl)-1-isopropyl-1*H*-indol-2-yl)-1,2,3,4-tetrahydroquinoline (2ay)

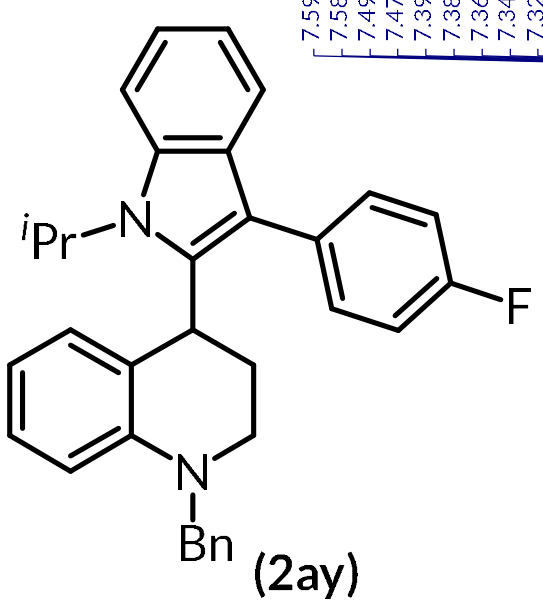

(2ay)

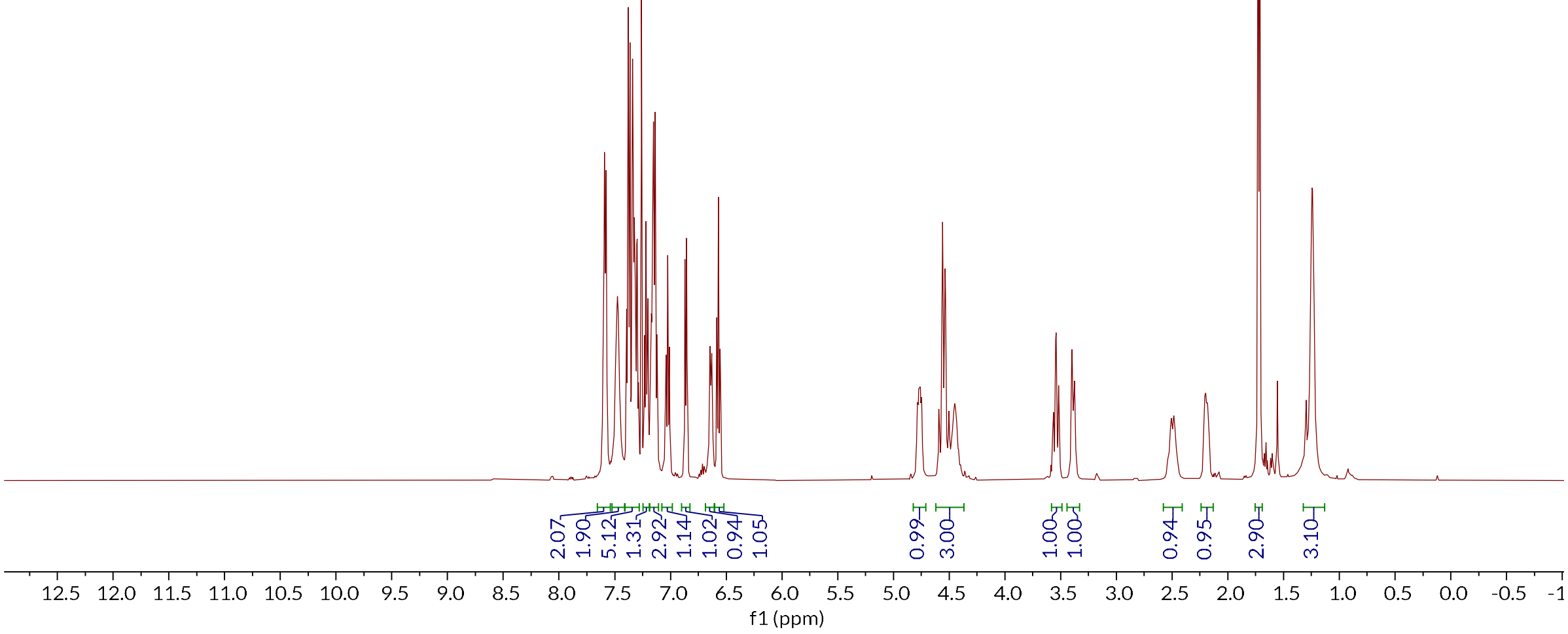

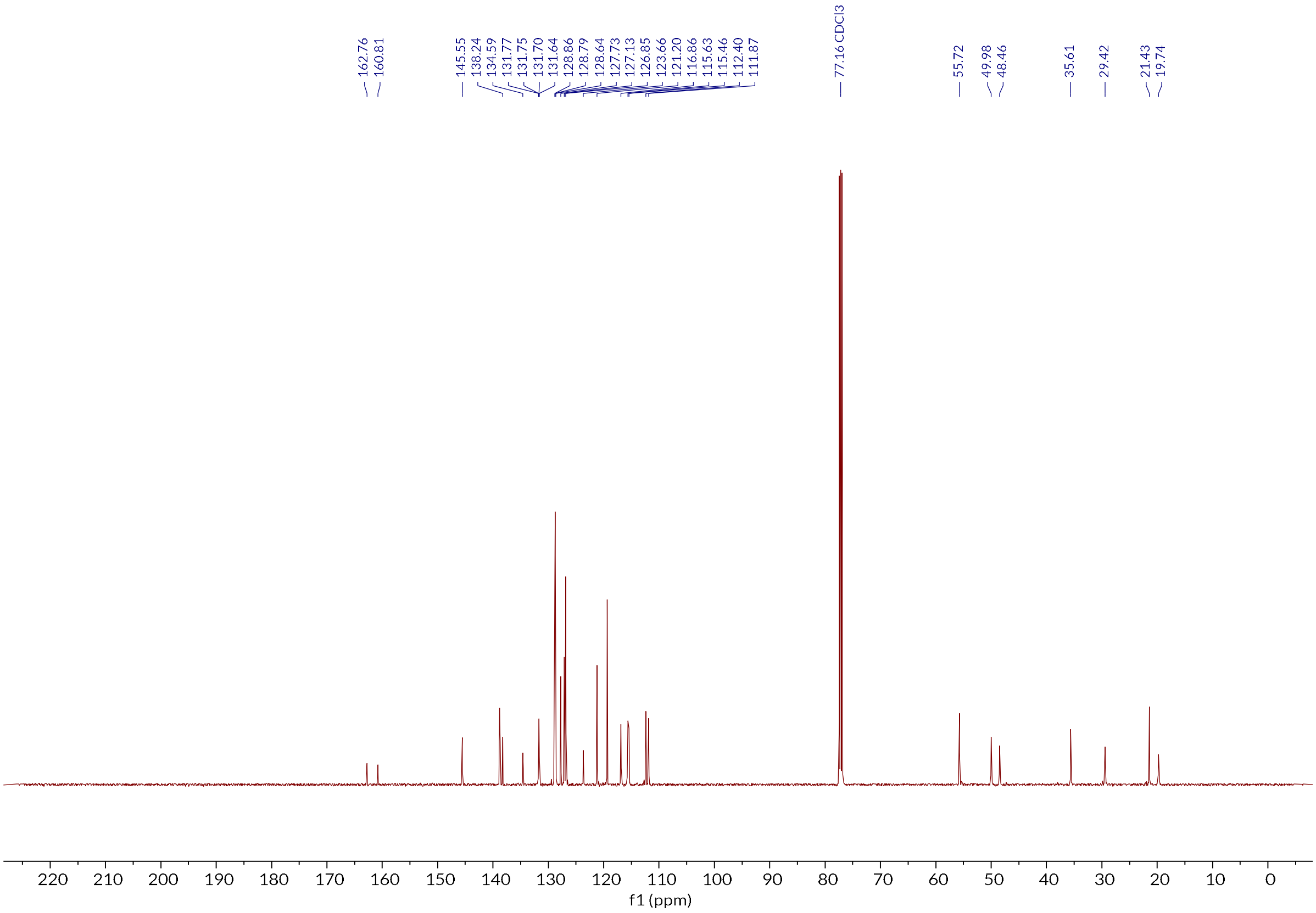

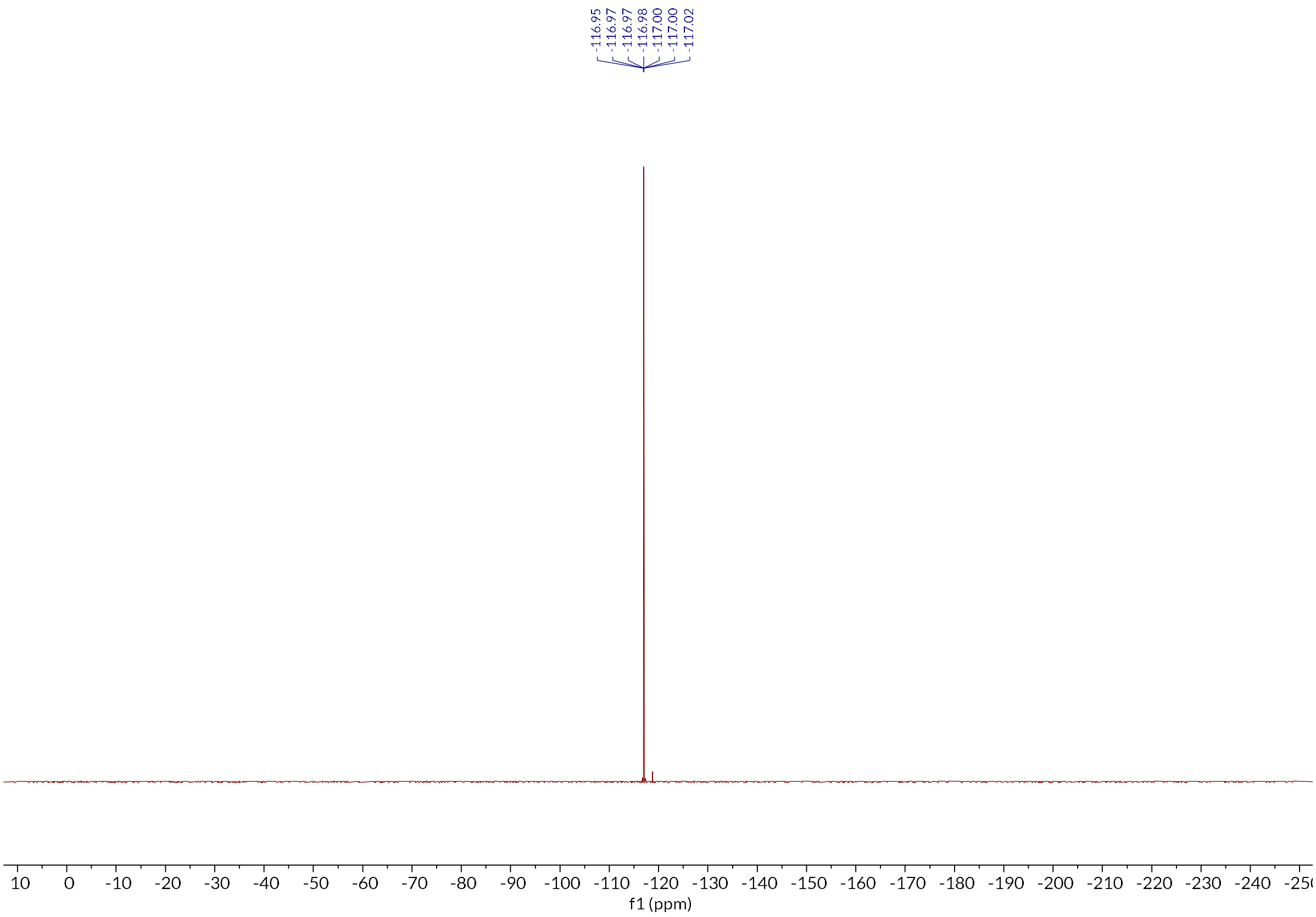
-116.95
-116.97
-116.97
-116.98
-117.00
-117.00
-117.02
10
0
-10
-20
-30
-40
-50
-60
-70
-80
-90
-100
-110
-120
-130
-140
-150
-160
-170
-180
-190
-200
-210
-220
-230
-240
f1 (ppm)

(±)-(*E*)-1-Benzyl-4-styryl-1,2,3,4-tetrahydroquinoline (2az)

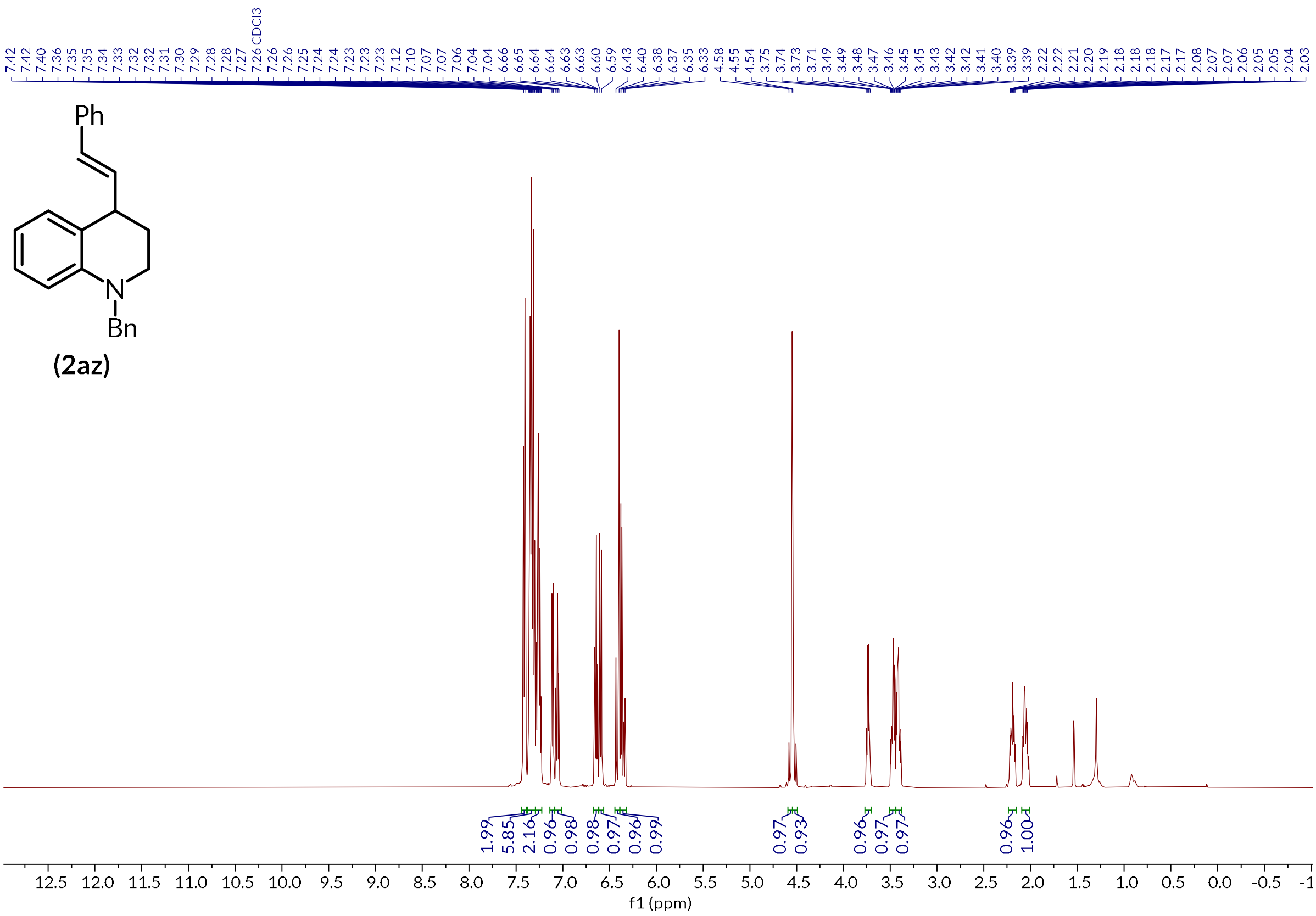

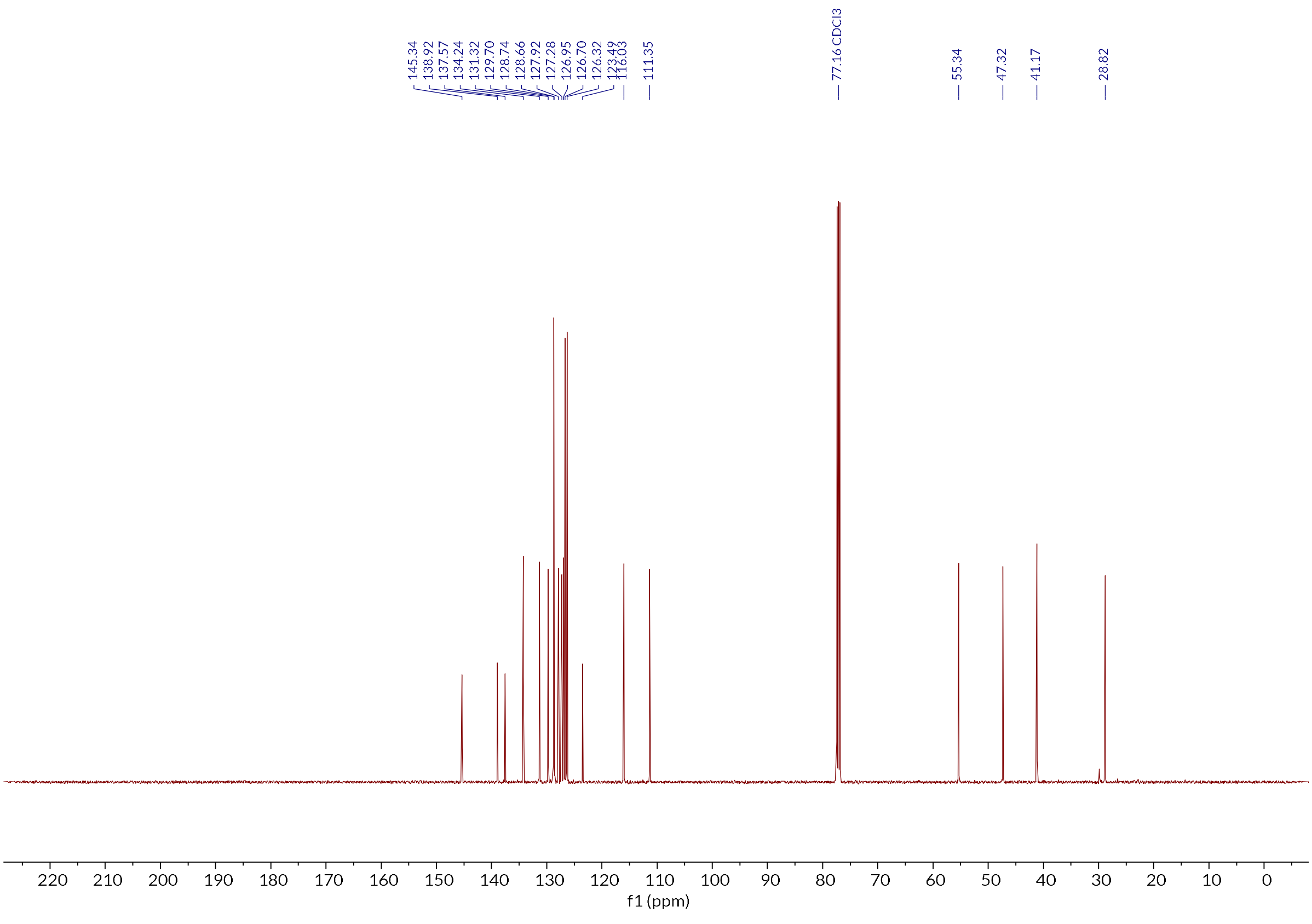

(±)-1-Benzyl-2-methyl-4-phenyl-1,2,3,4-tetrahydroquinoline (2ba)

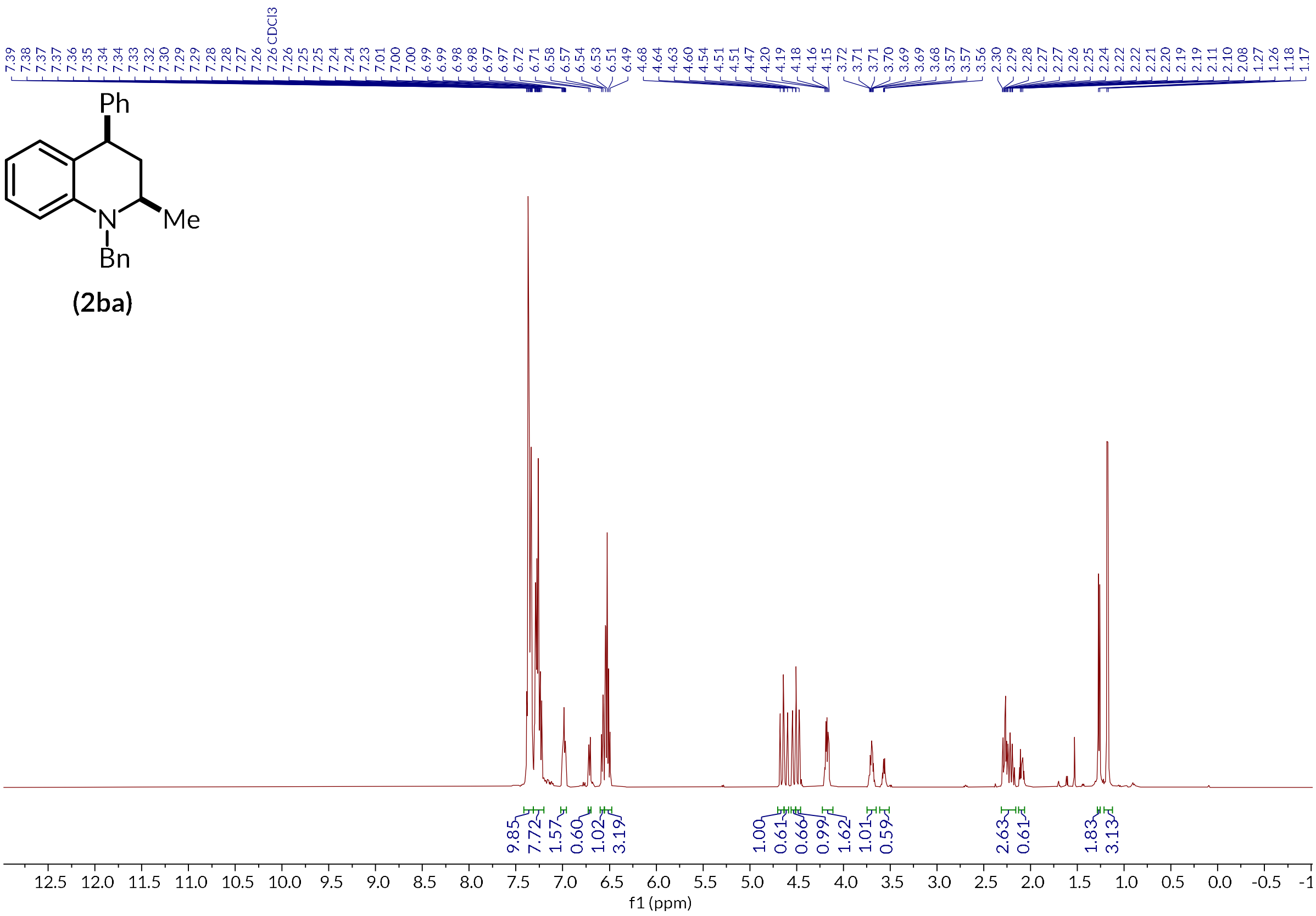

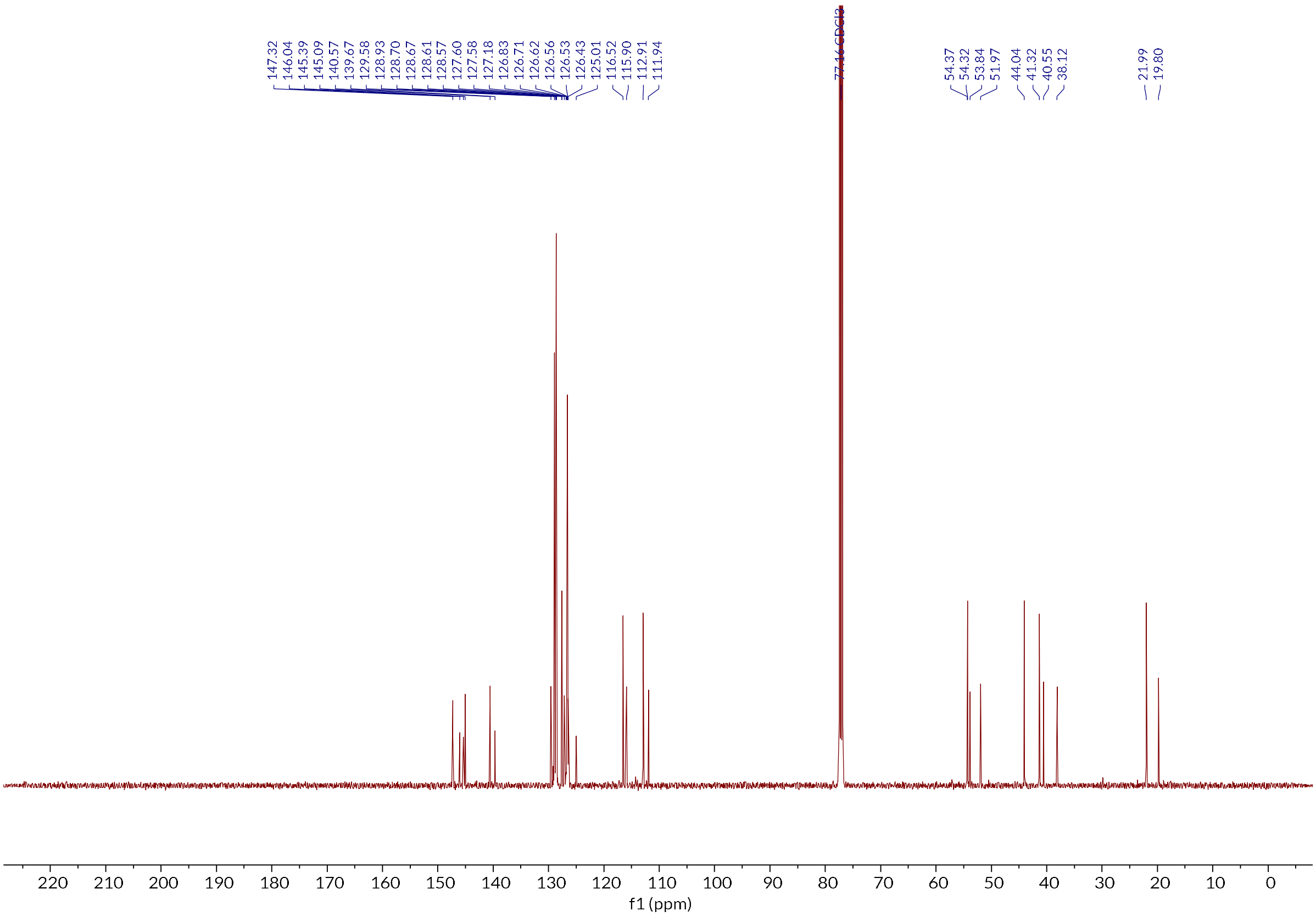

(±)-1-Benzyl-4-methyl-1,2,3,4-tetrahydroquinoline (2bb)

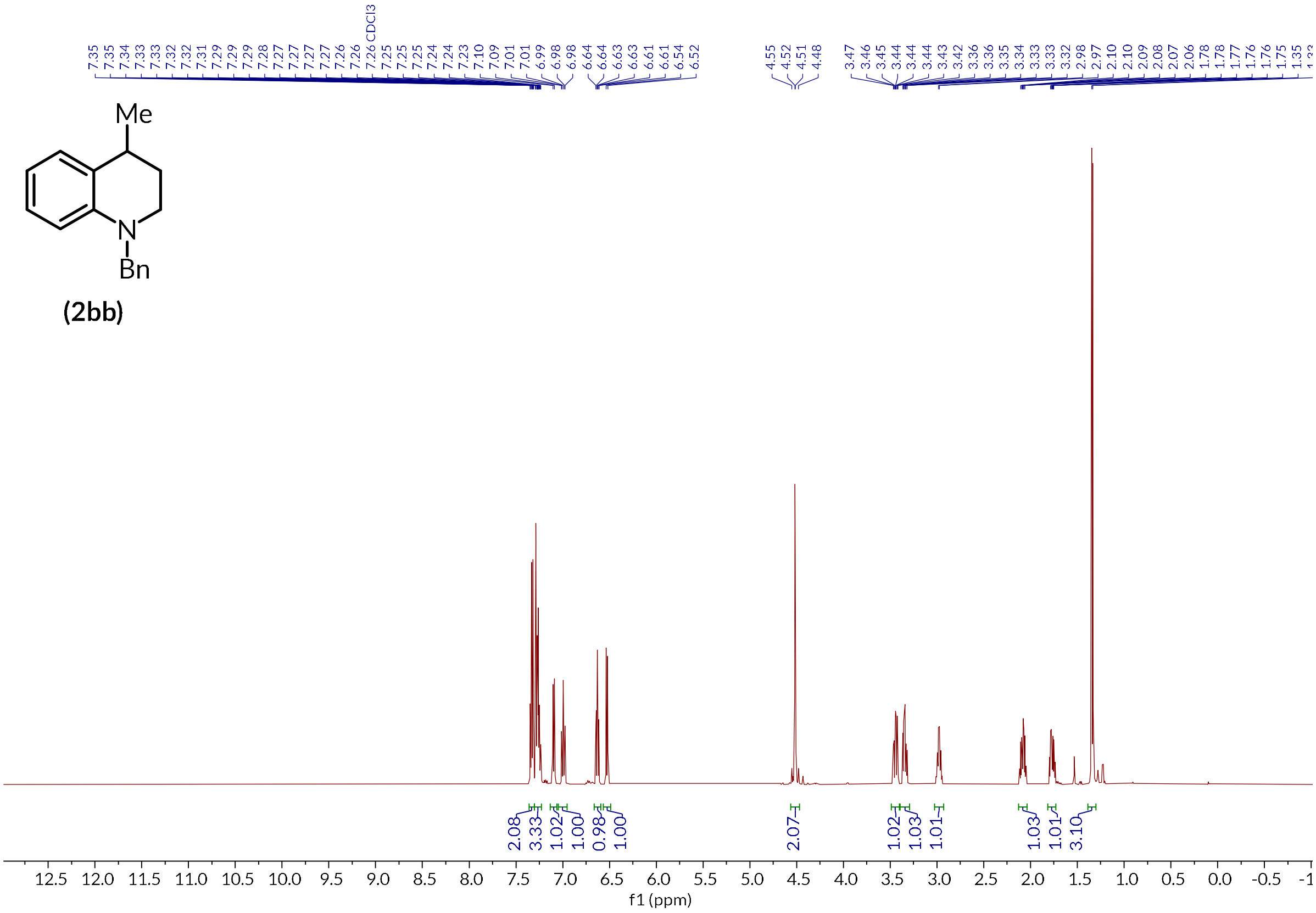

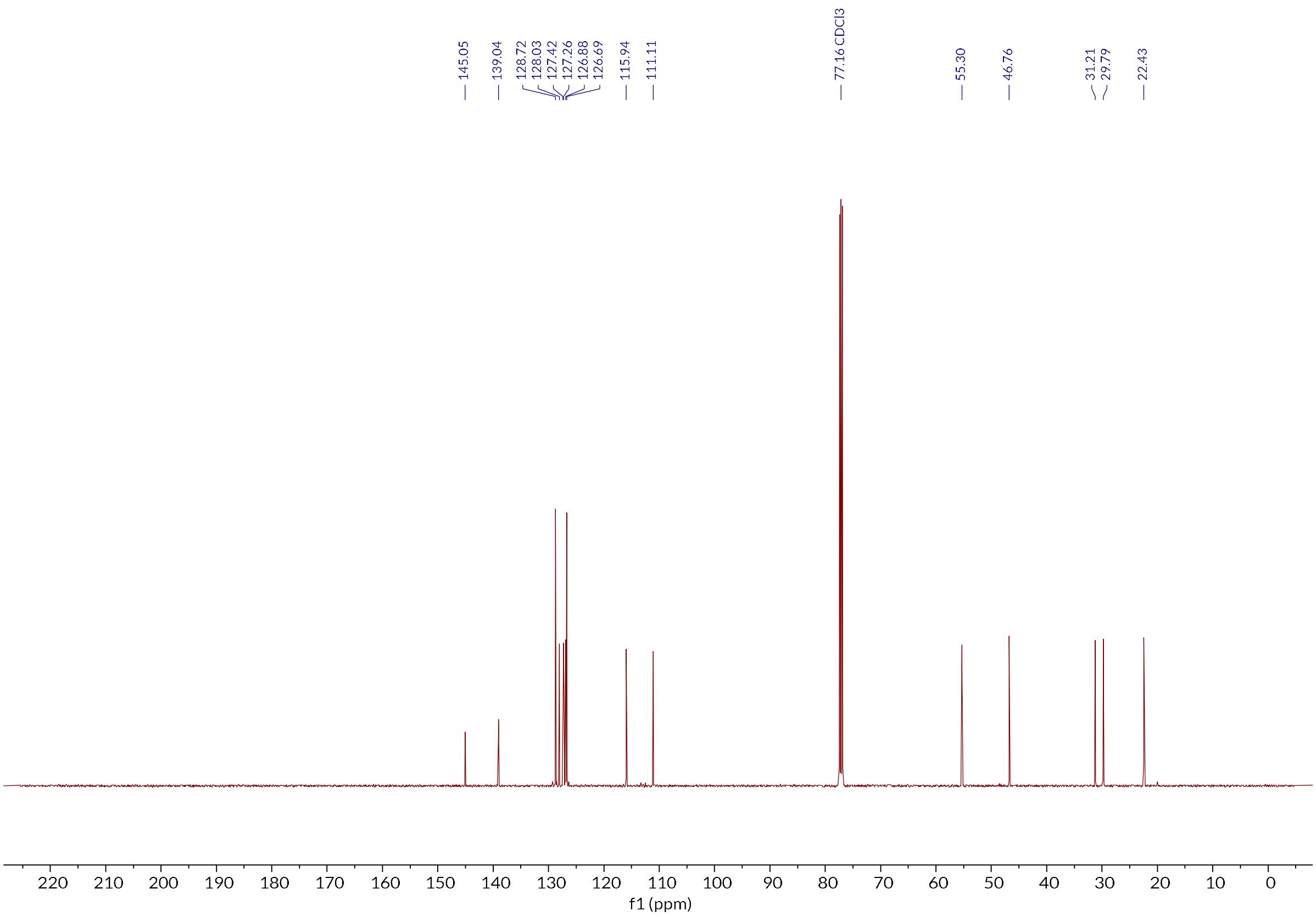

(±)-1-Benzyl-6-(*tert*-butyl)-4-phenyl-1,2,3,4-tetrahydroquinoline (2bc)

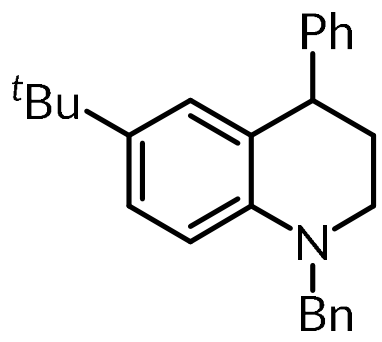

(2bc)

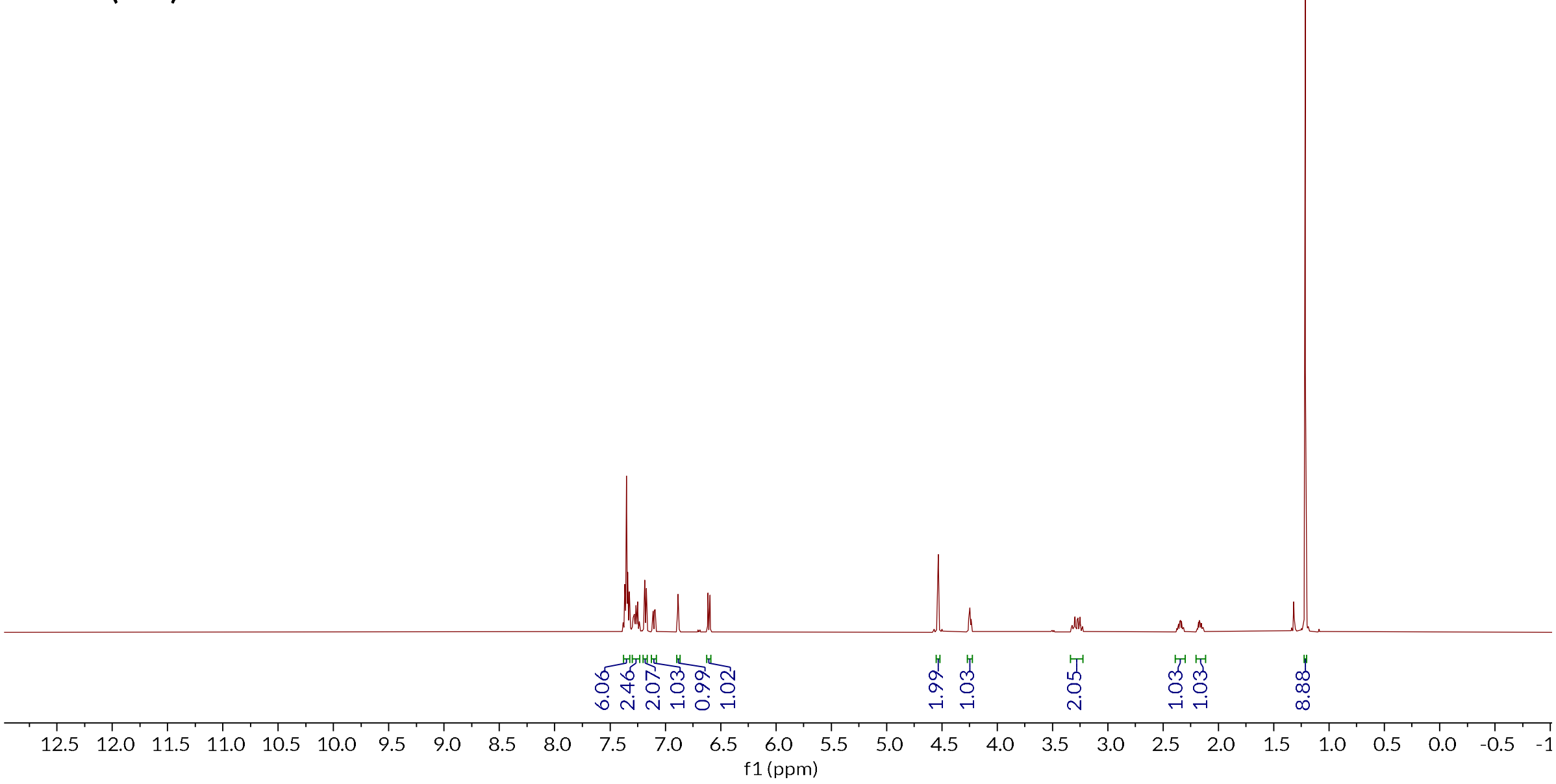

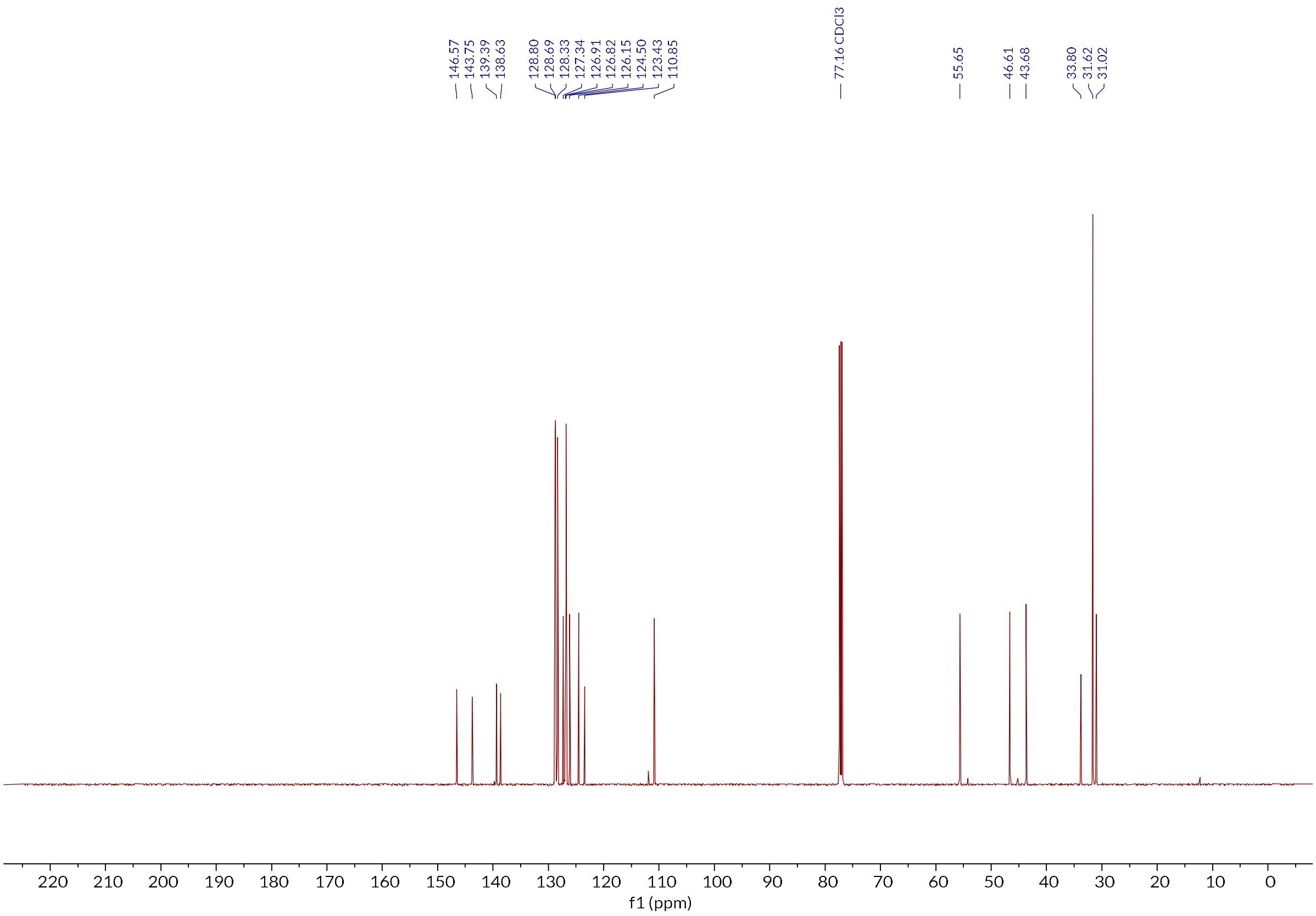

(±)-1-Benzyl-6-methoxy-4-phenyl-1,2,3,4-tetrahydroquinoline (2bd)

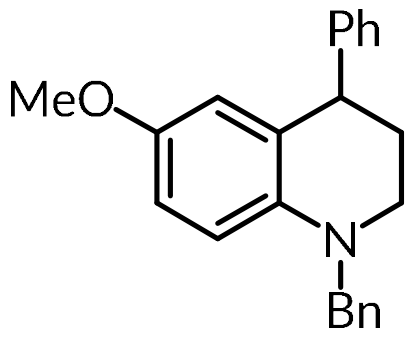

(2bd)

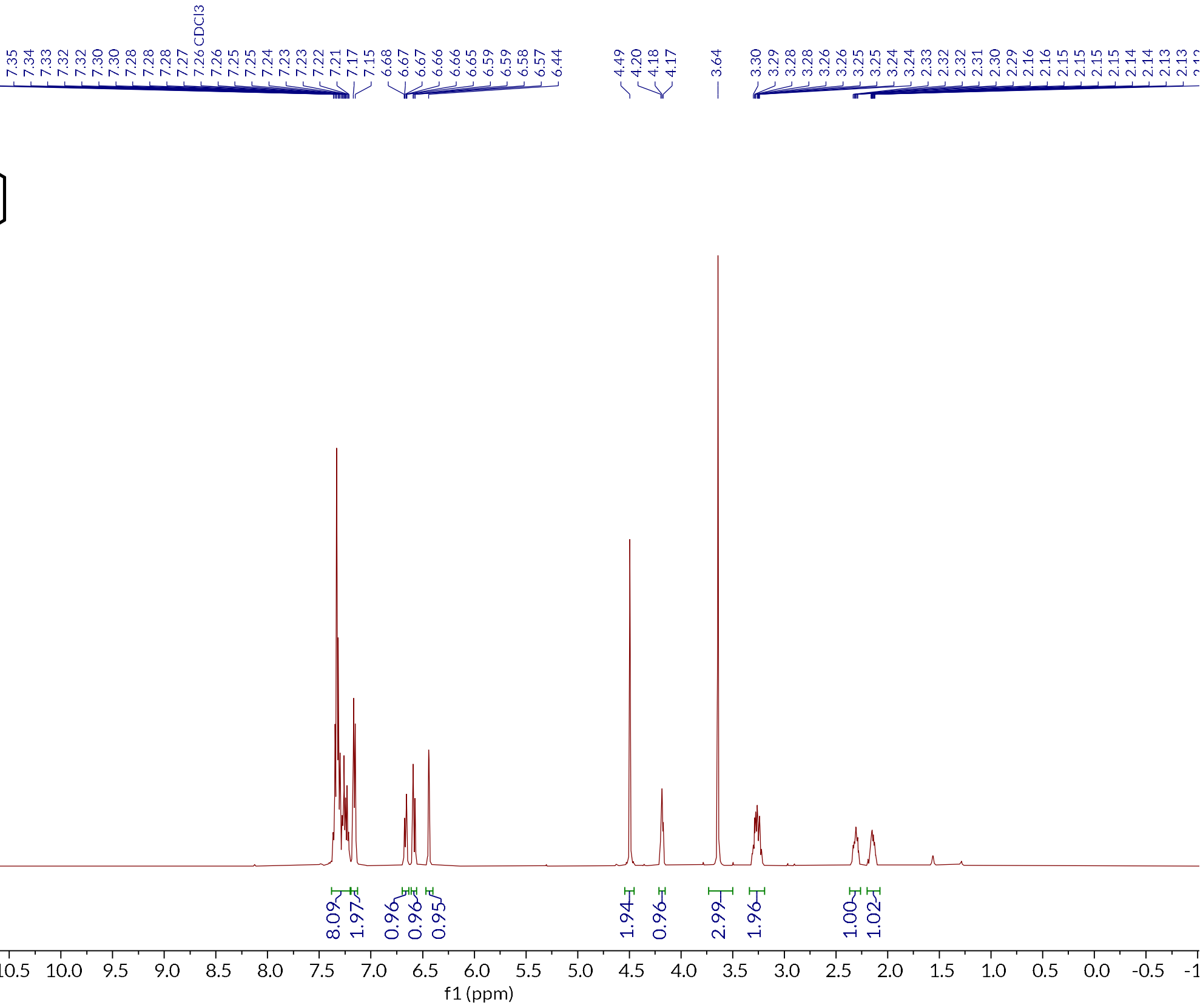

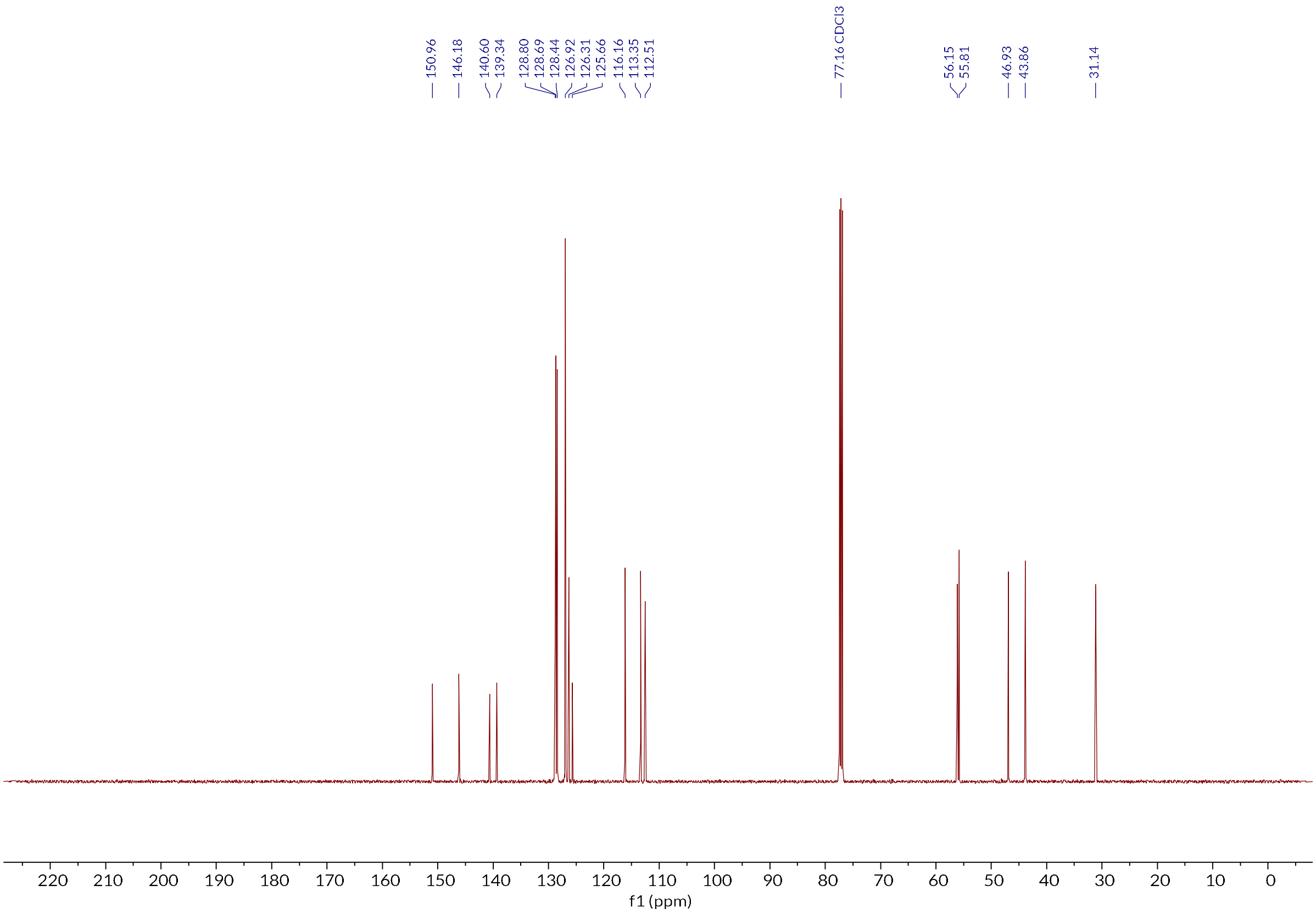

(±)-1-Benzyl-6-fluoro-4-phenyl-1,2,3,4-tetrahydroquinoline (2be)

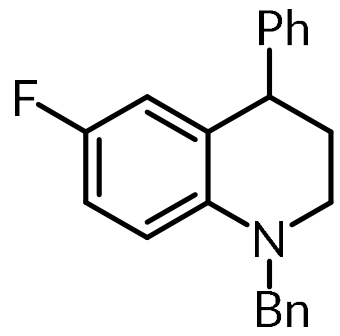

(2be)

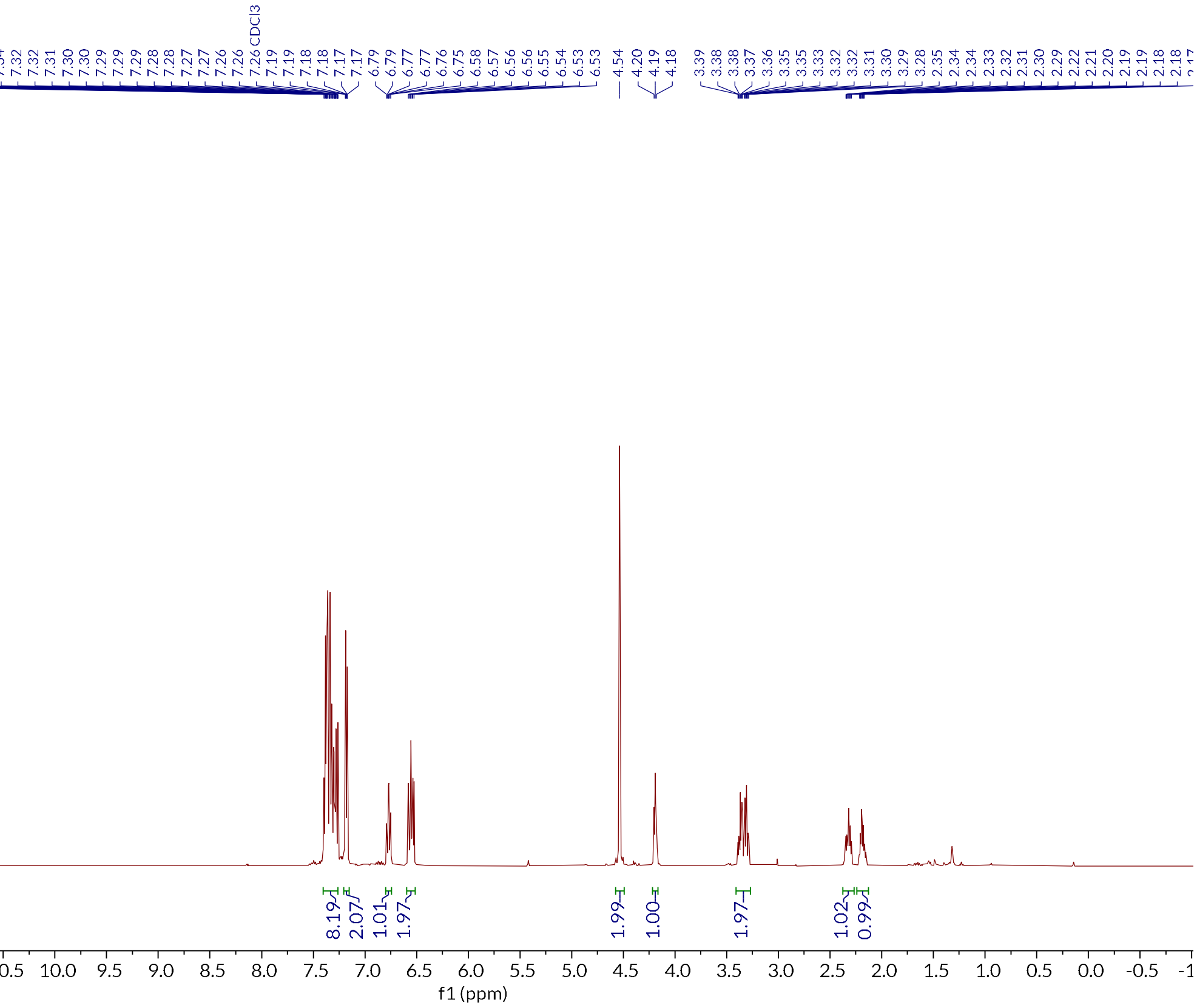

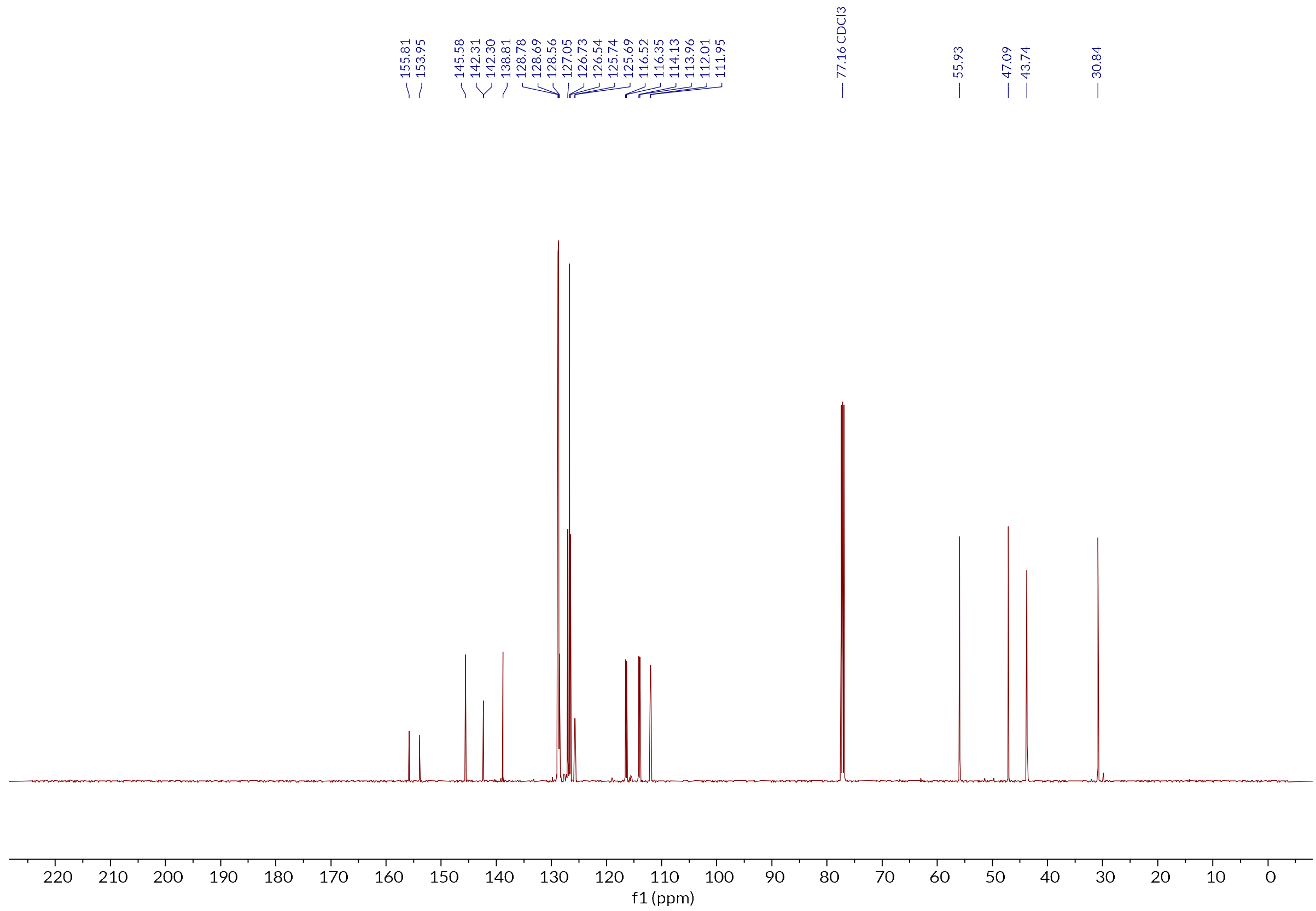

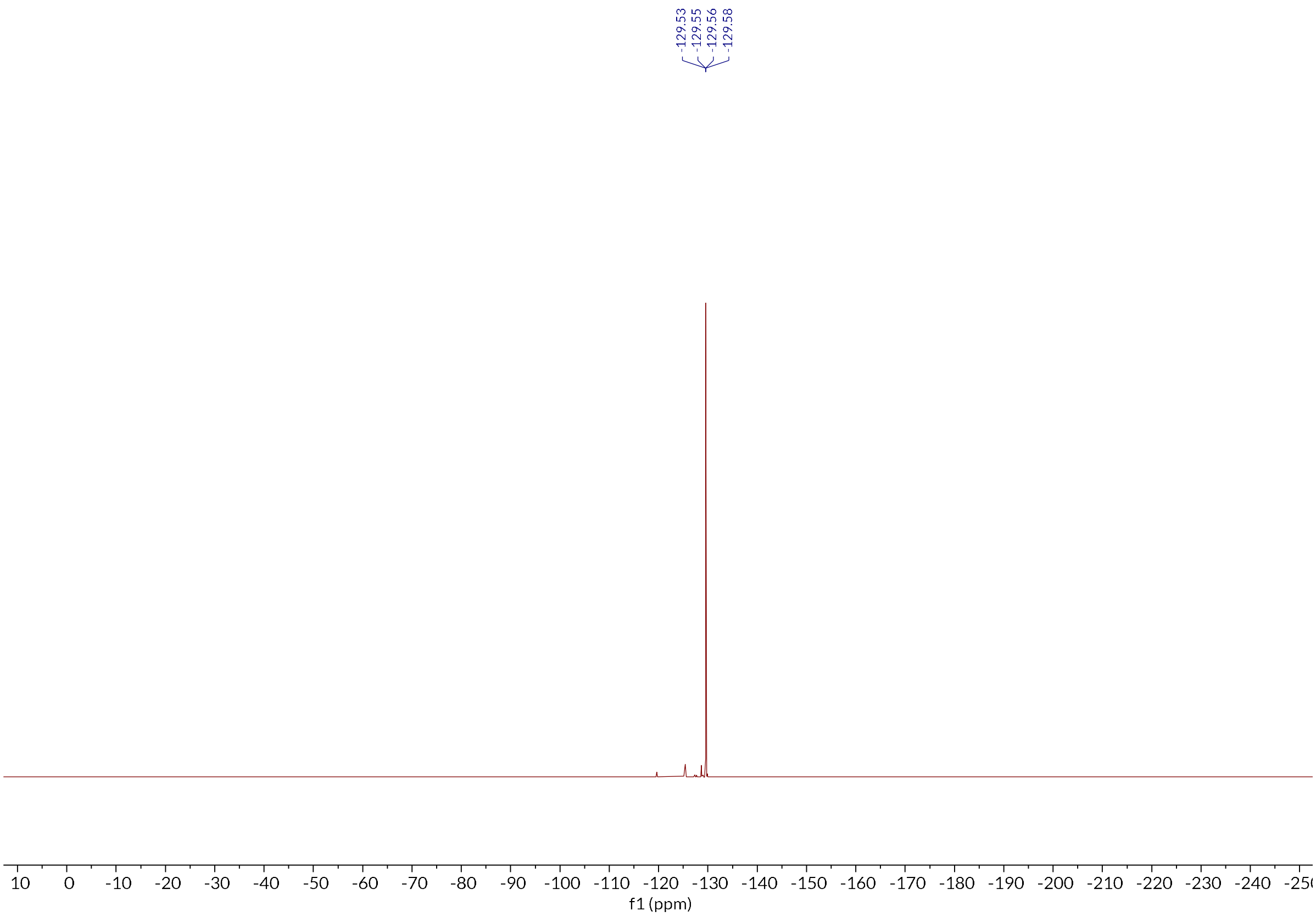
-129.53
-129.55
-129.56
-129.58
10
0
-10
-20
-30
-40
-50
-60
-70
-80
-90
-100
-110
-120
-130
-140
-150
-160
-170
-180
-190
-200
-210
-220
-230
-240
f1 (ppm)

(±)-1-Benzyl-4-phenyl-6-(trifluoromethyl)-1,2,3,4-tetrahydroquinoline (2bf)

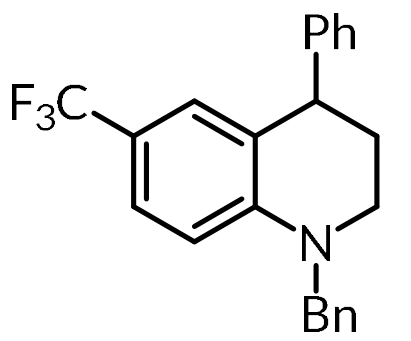

(2bf)

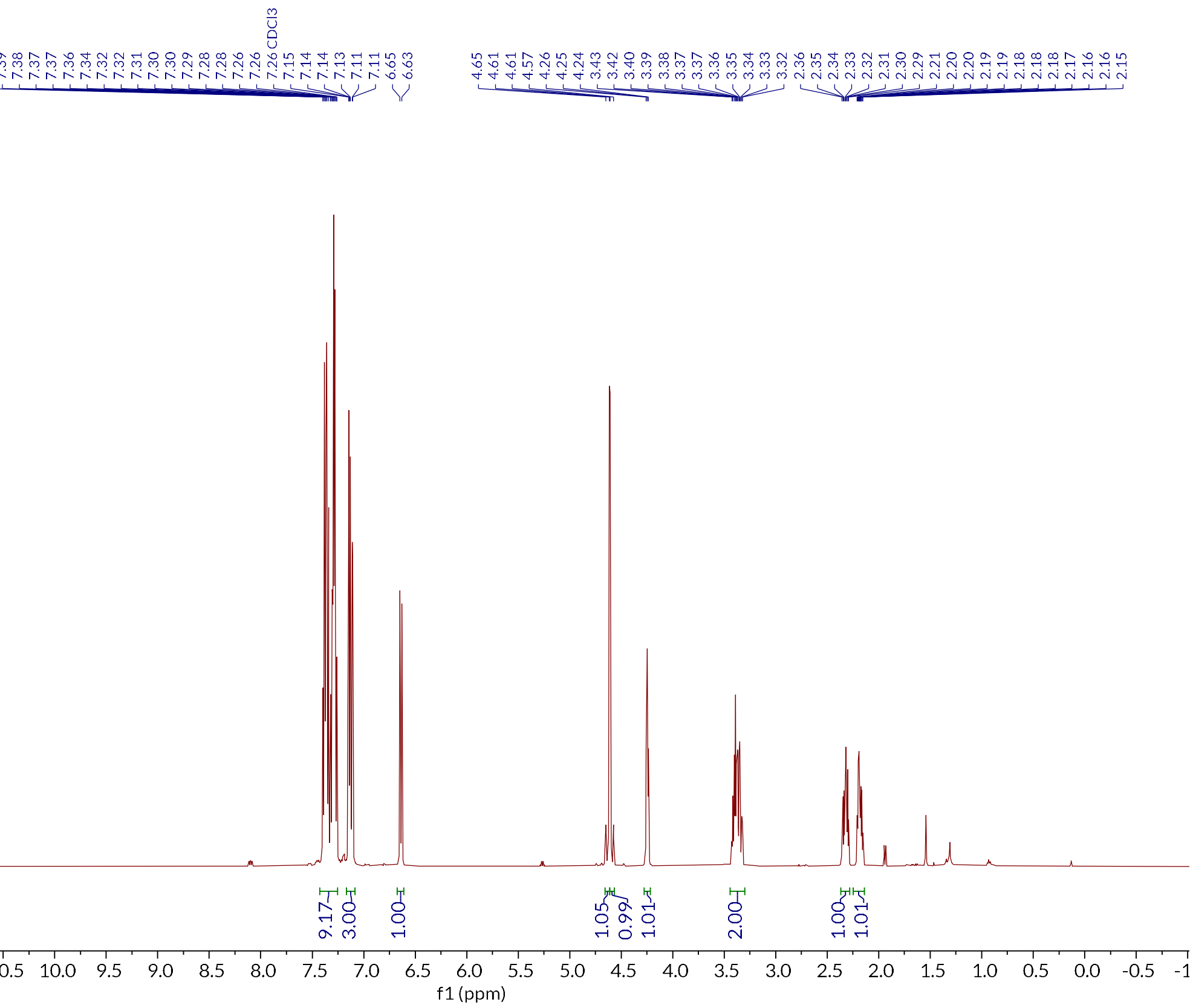

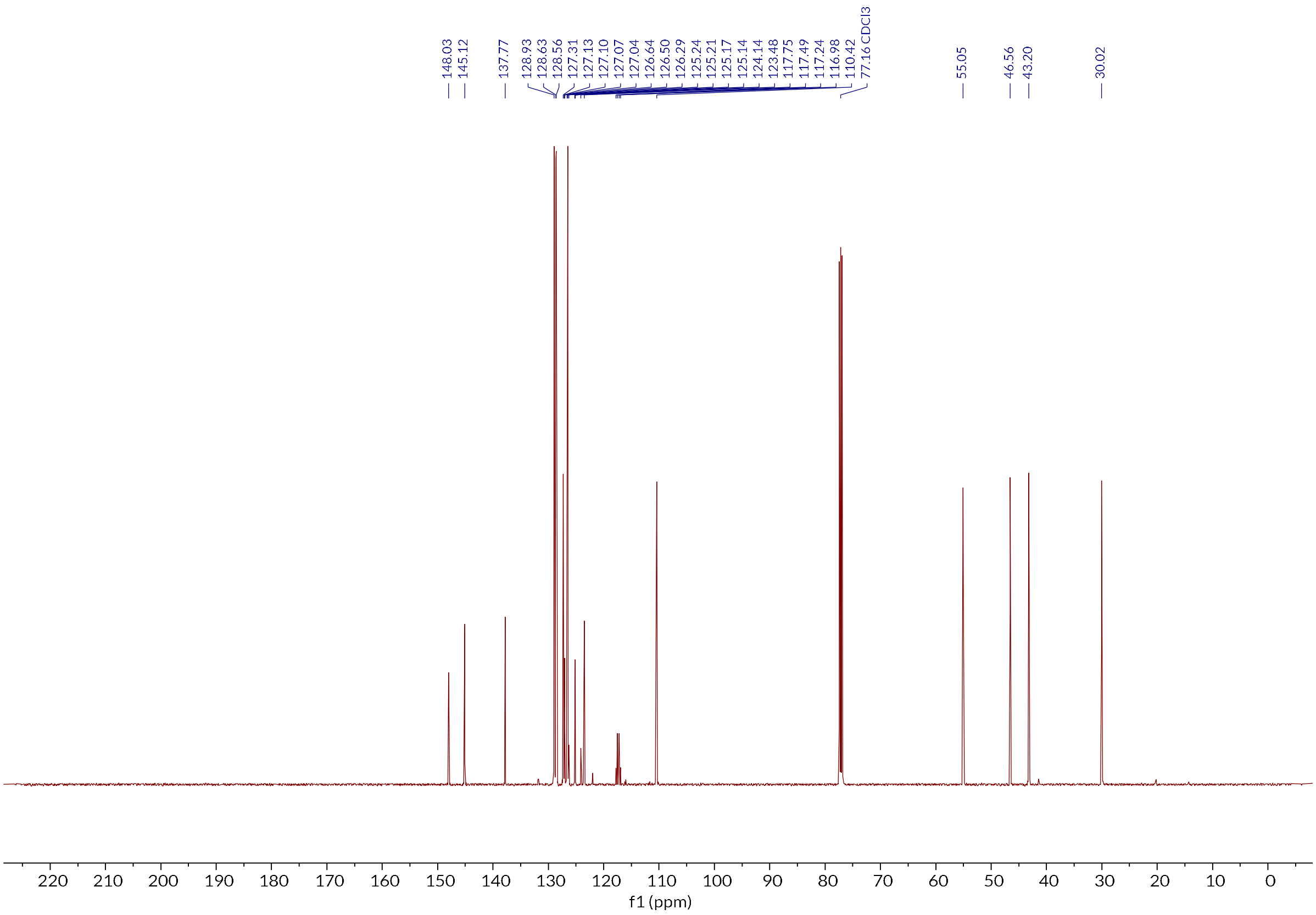

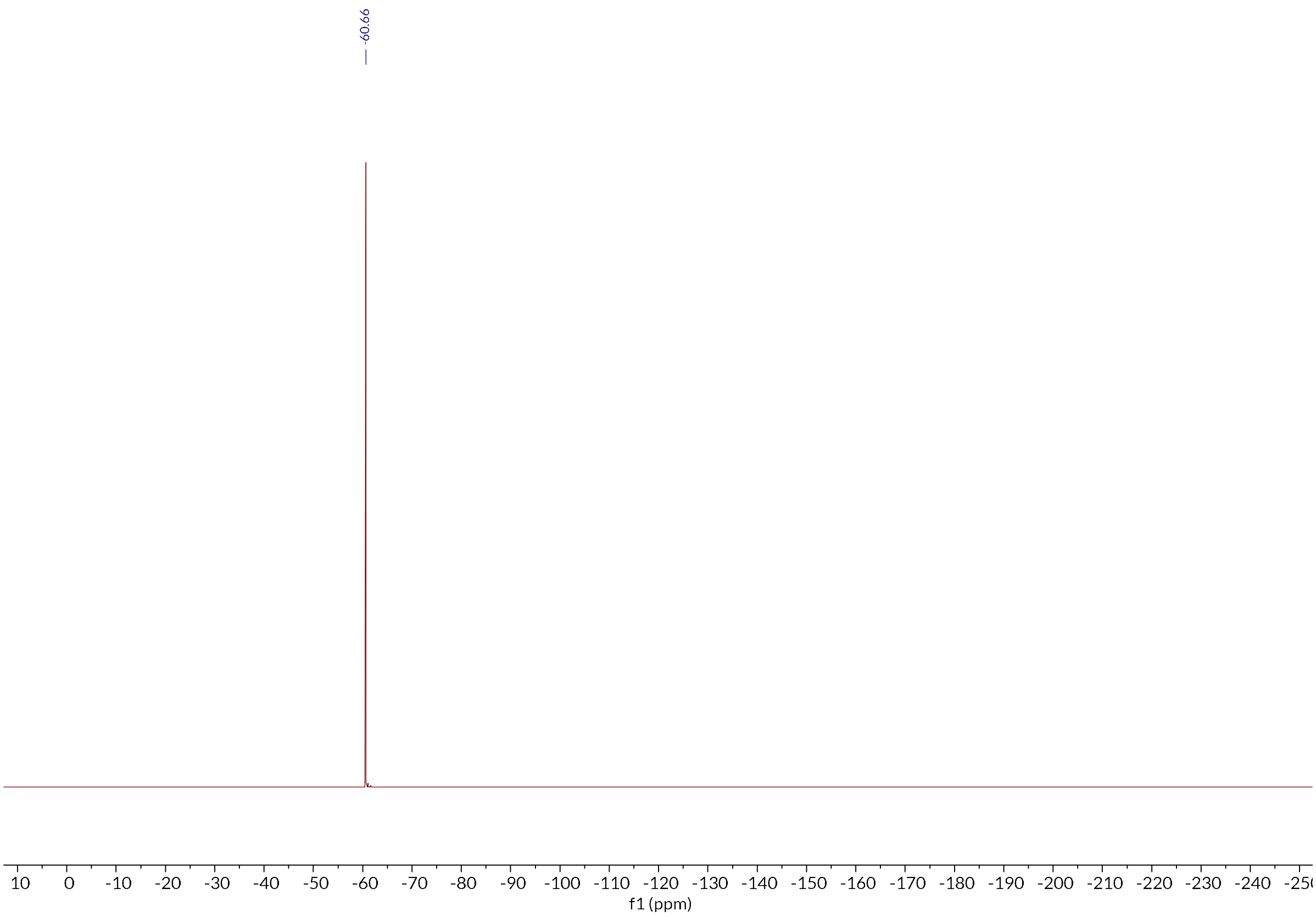

-60.66
10 0 -10 -20 -30 -40 -50 -60 -70 -80 -90 -100 -110 -120 -130 -140 -150 -160 -170 -180 -190 -200 -210 -220 -230 -240
f1 (ppm)

(±)-1-Benzyl-4-phenyl-1,2,3,4-tetrahydroquinoline-6-carbonitrile (2bg)

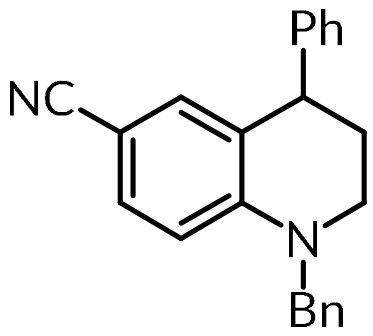

(2bg)

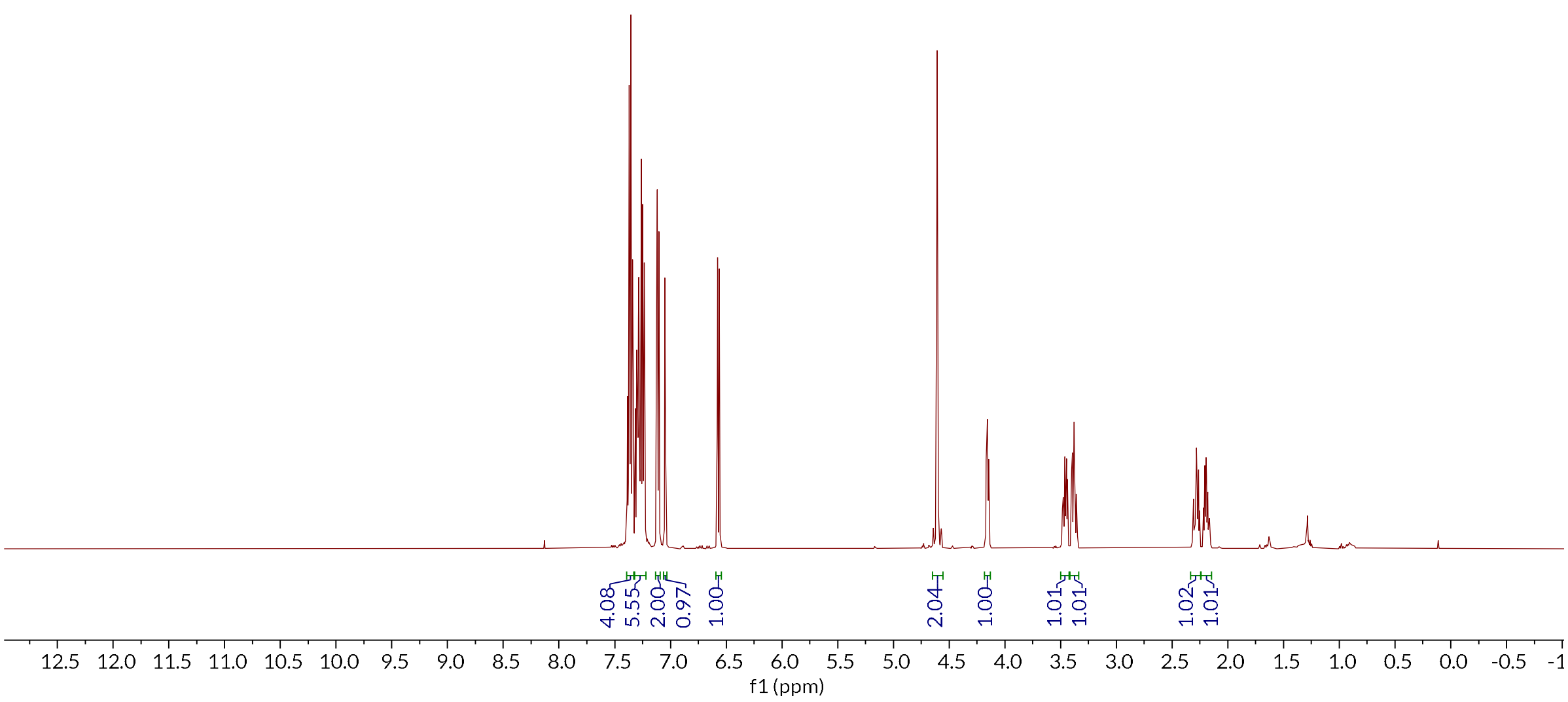

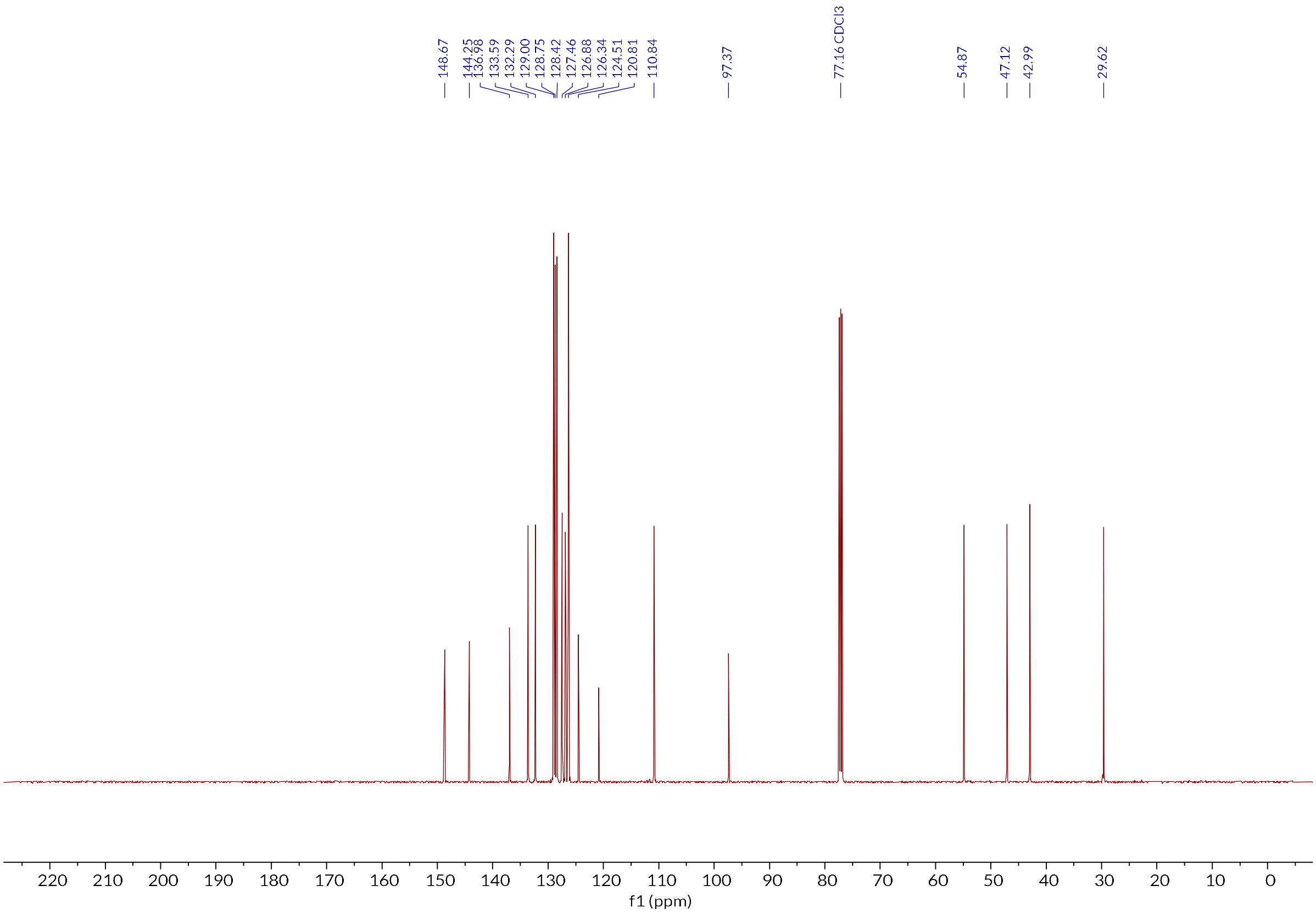

(±)-1-(1-Benzyl-4-phenyl-1,2,3,4-tetrahydroquinolin-6-yl)ethan-1-one (2bh)

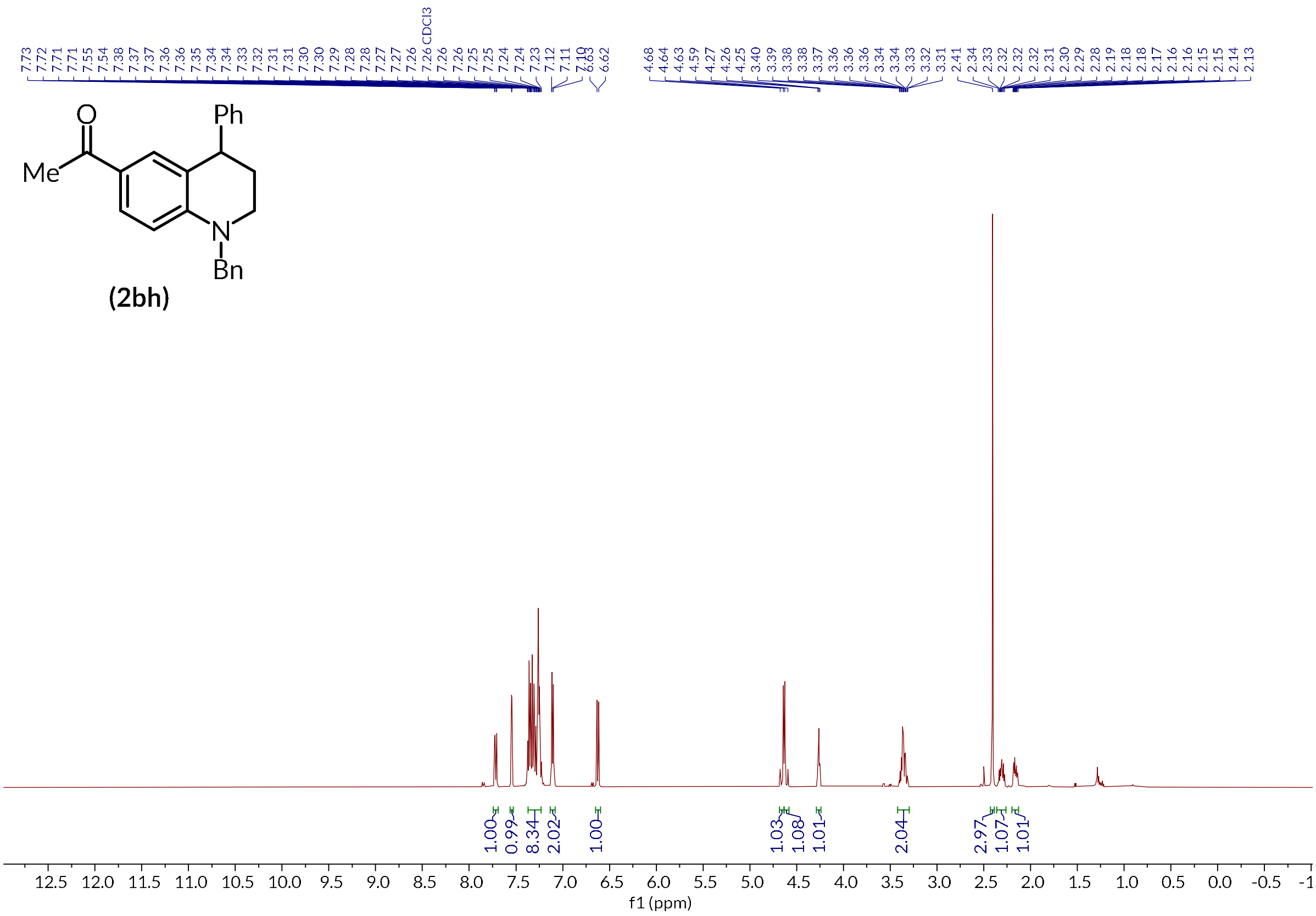

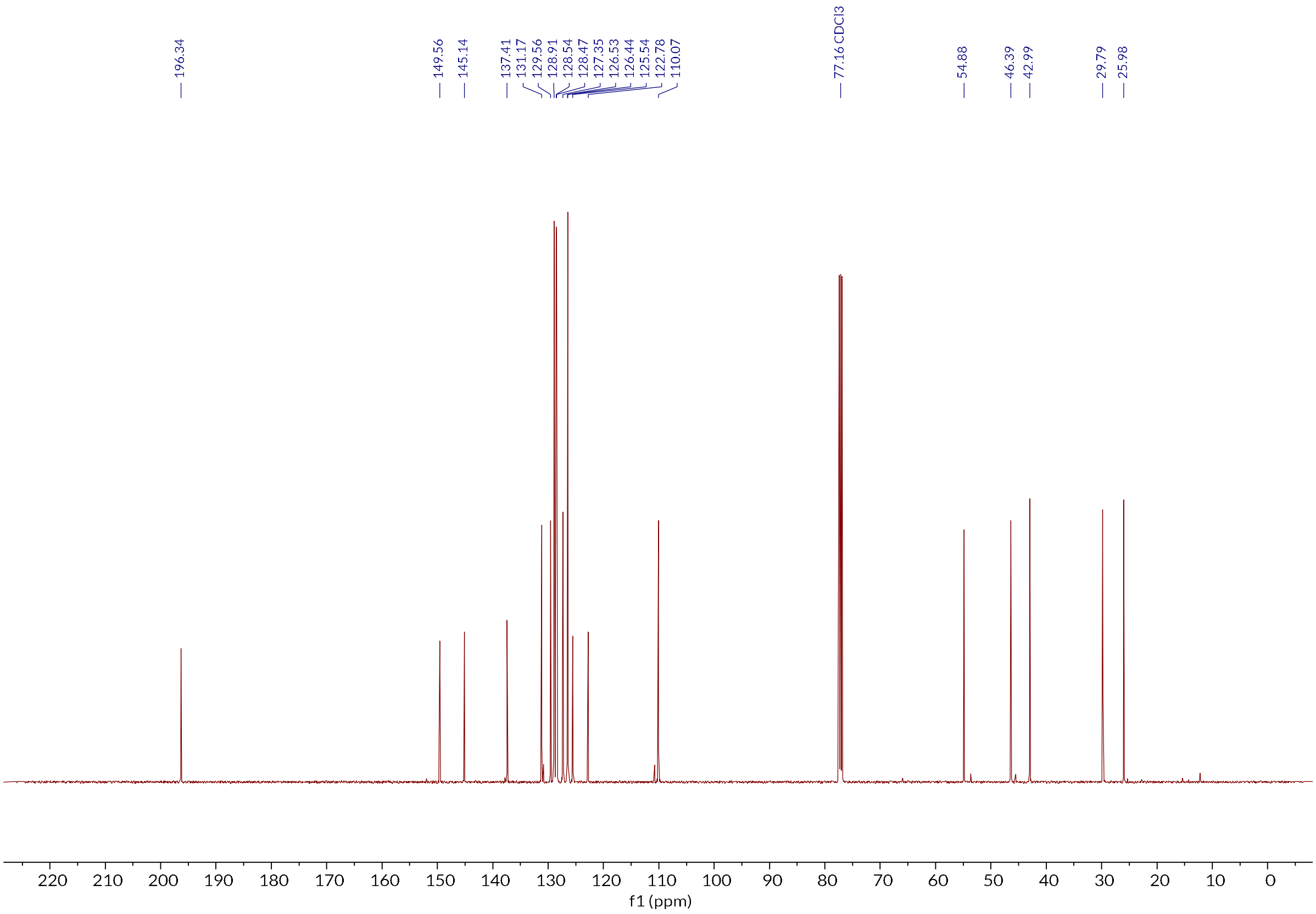

(±)-2-(1-Benzyl-4-phenyl-1,2,3,4-tetrahydroquinolin-6-yl)acetonitrile (2bi)

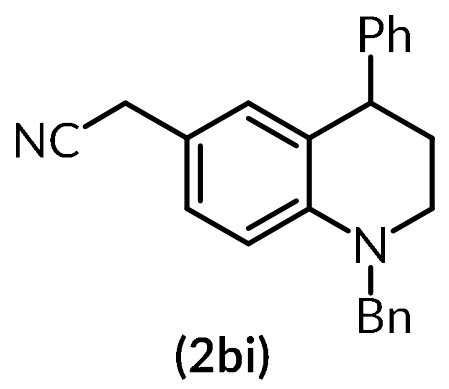

(2bi)

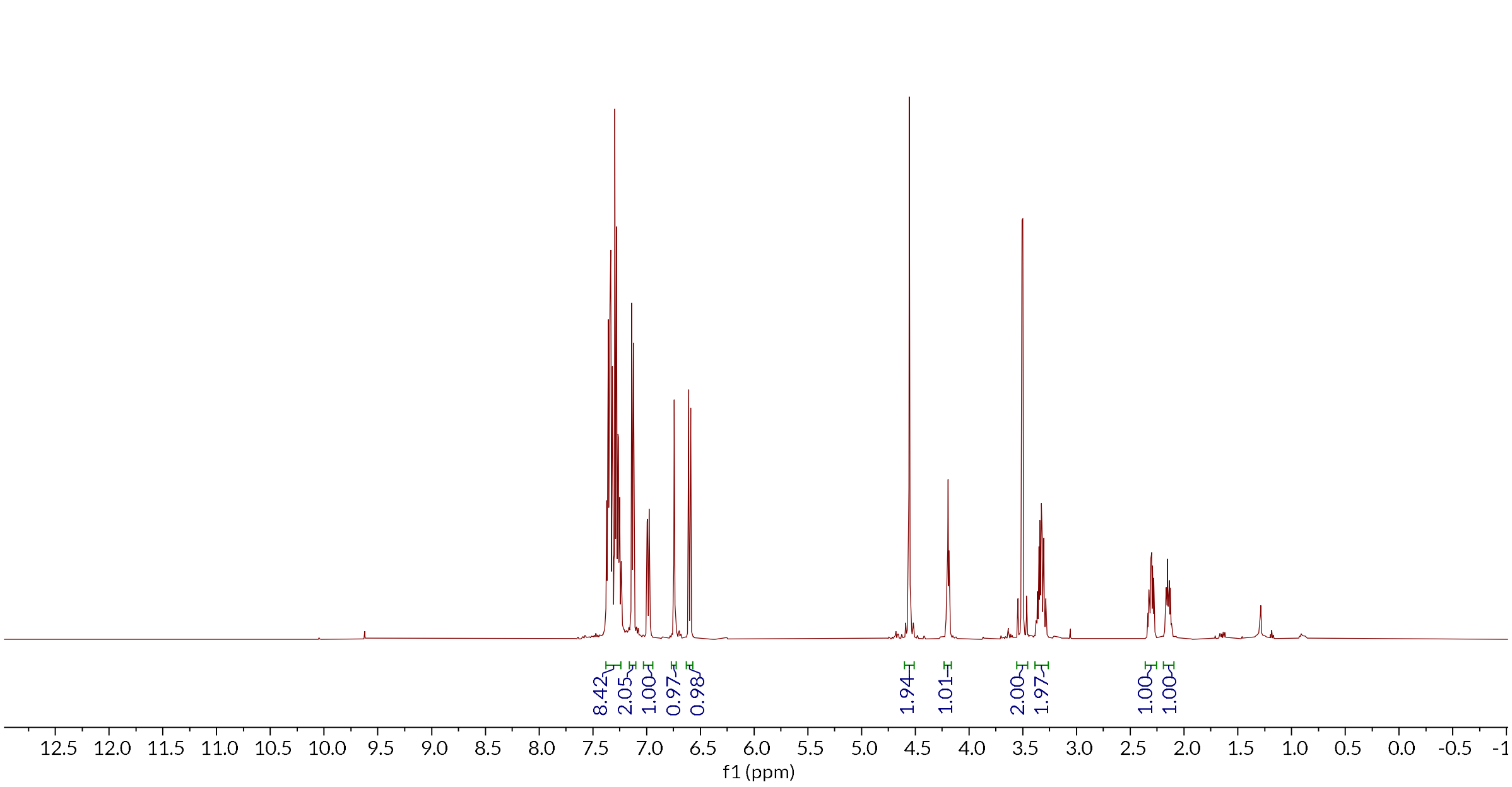

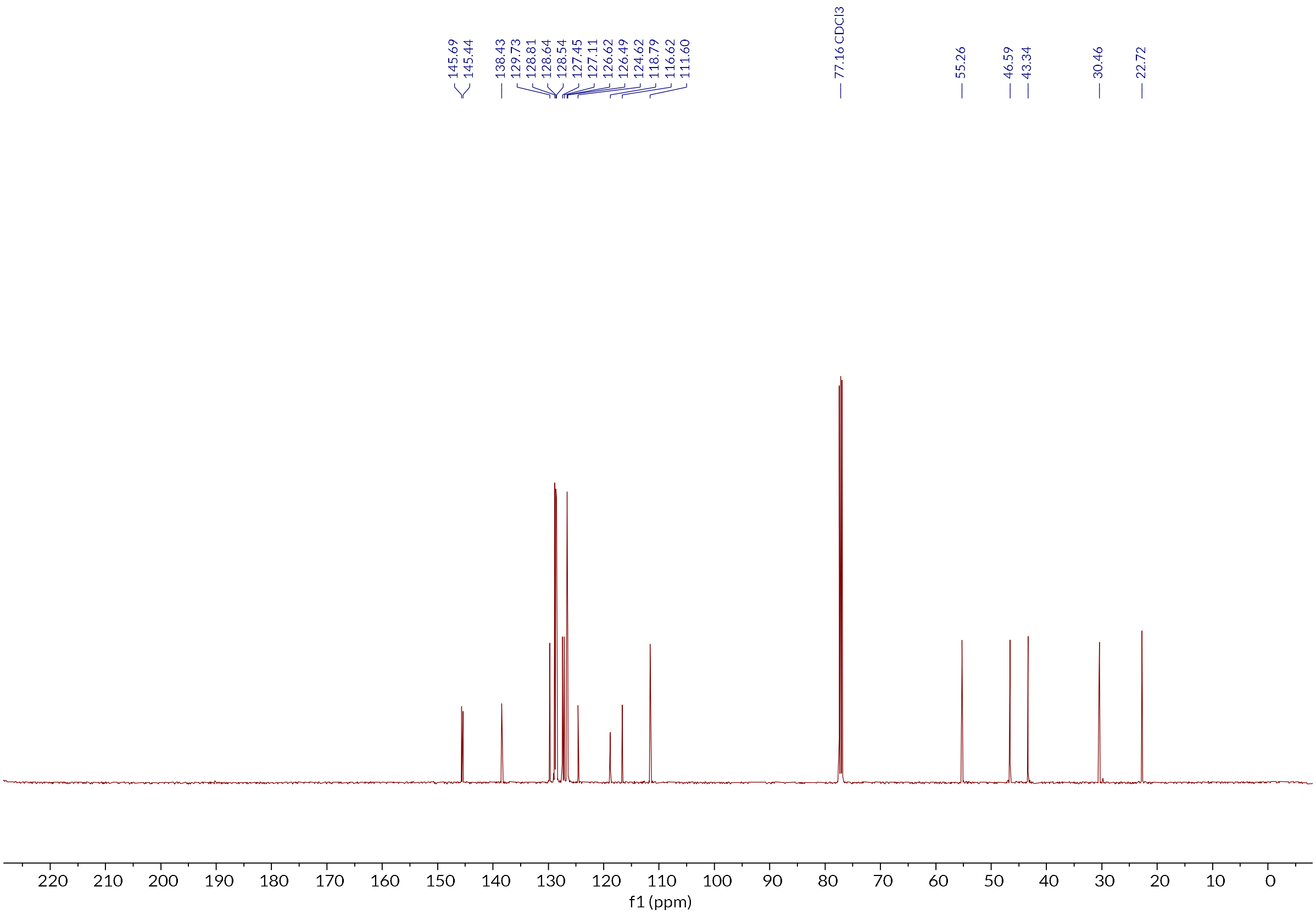

(±)-1-Benzyl-5,6,7-trifluoro-4-phenyl-1,2,3,4-tetrahydroquinoline (2bj)

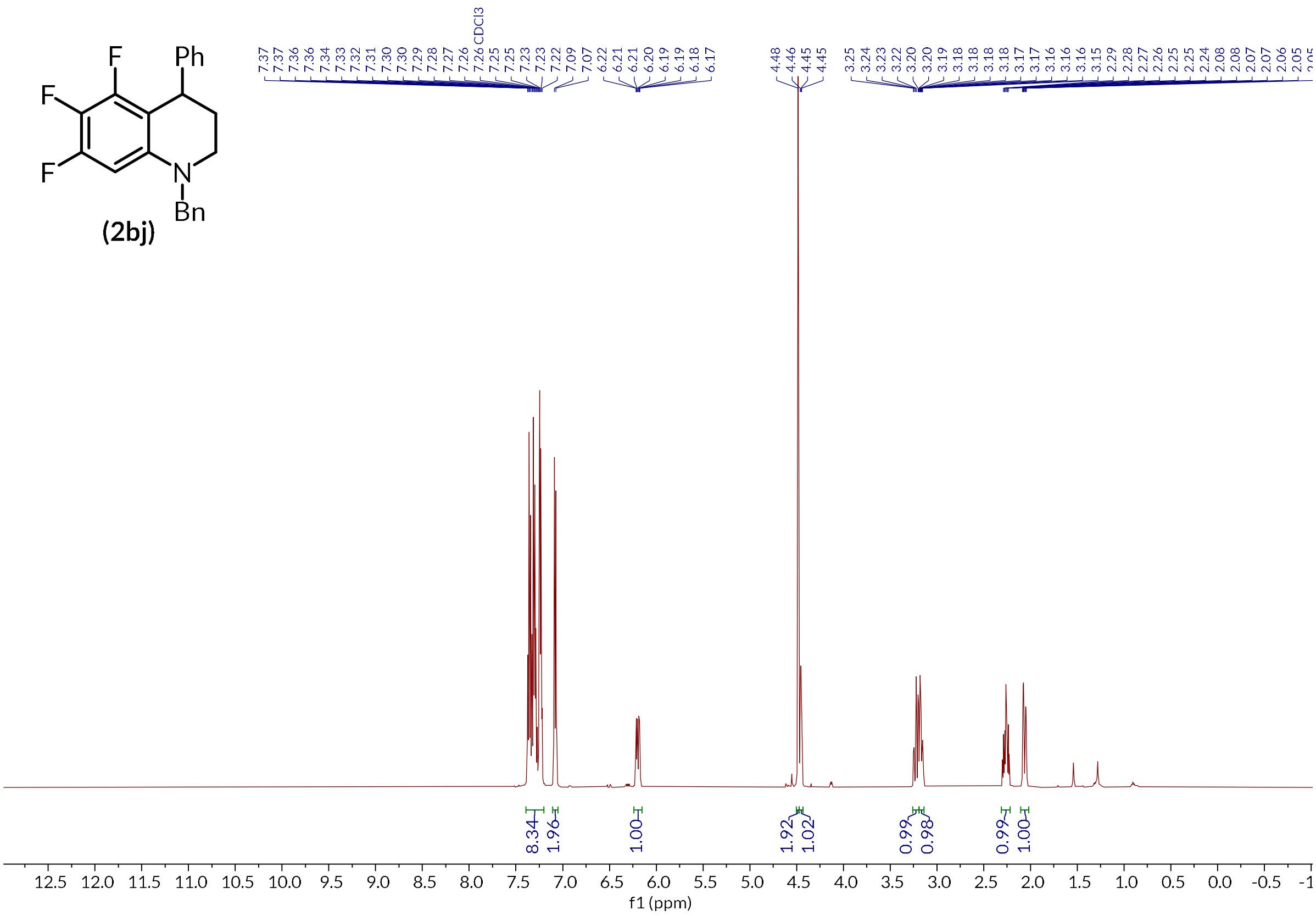

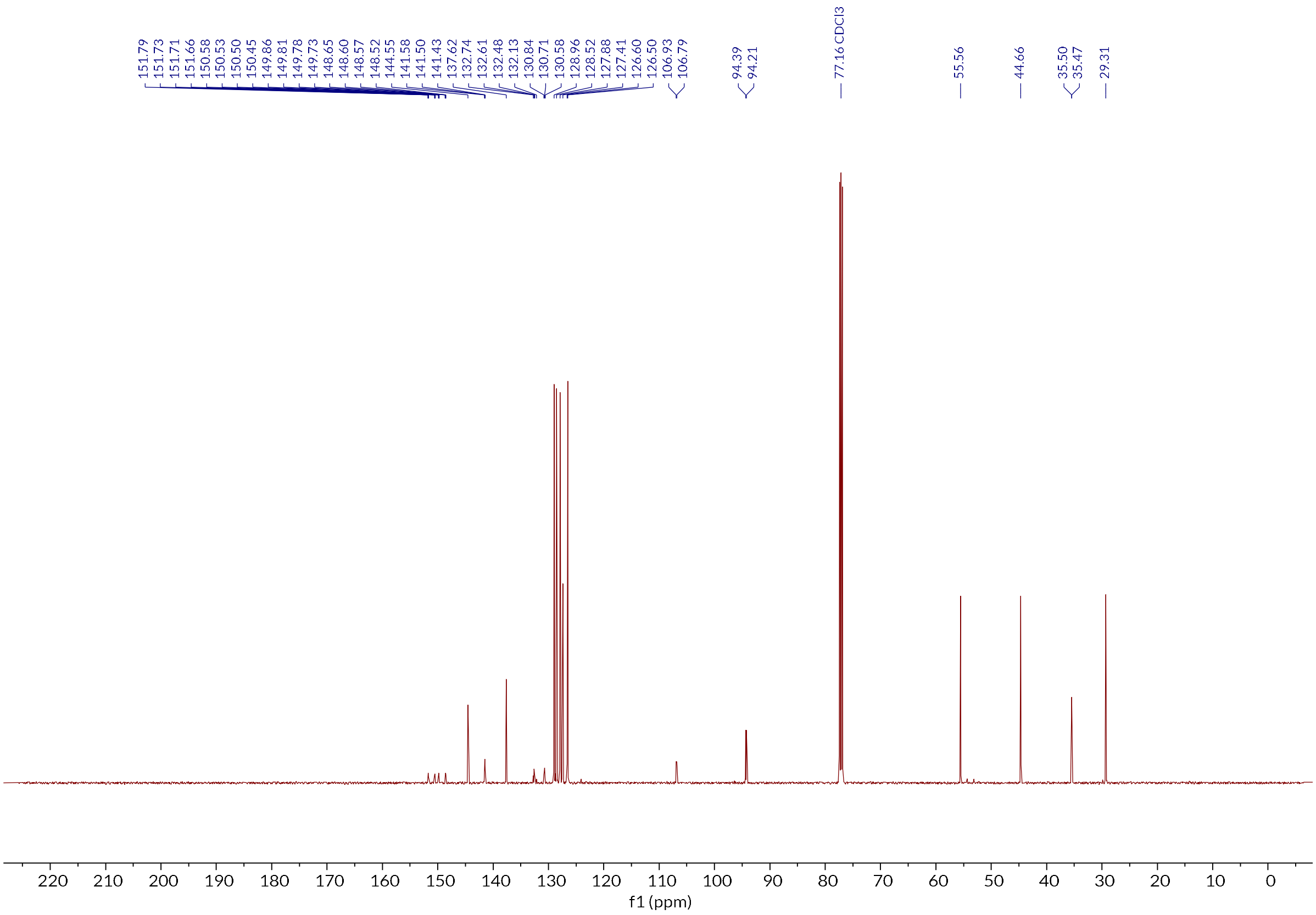

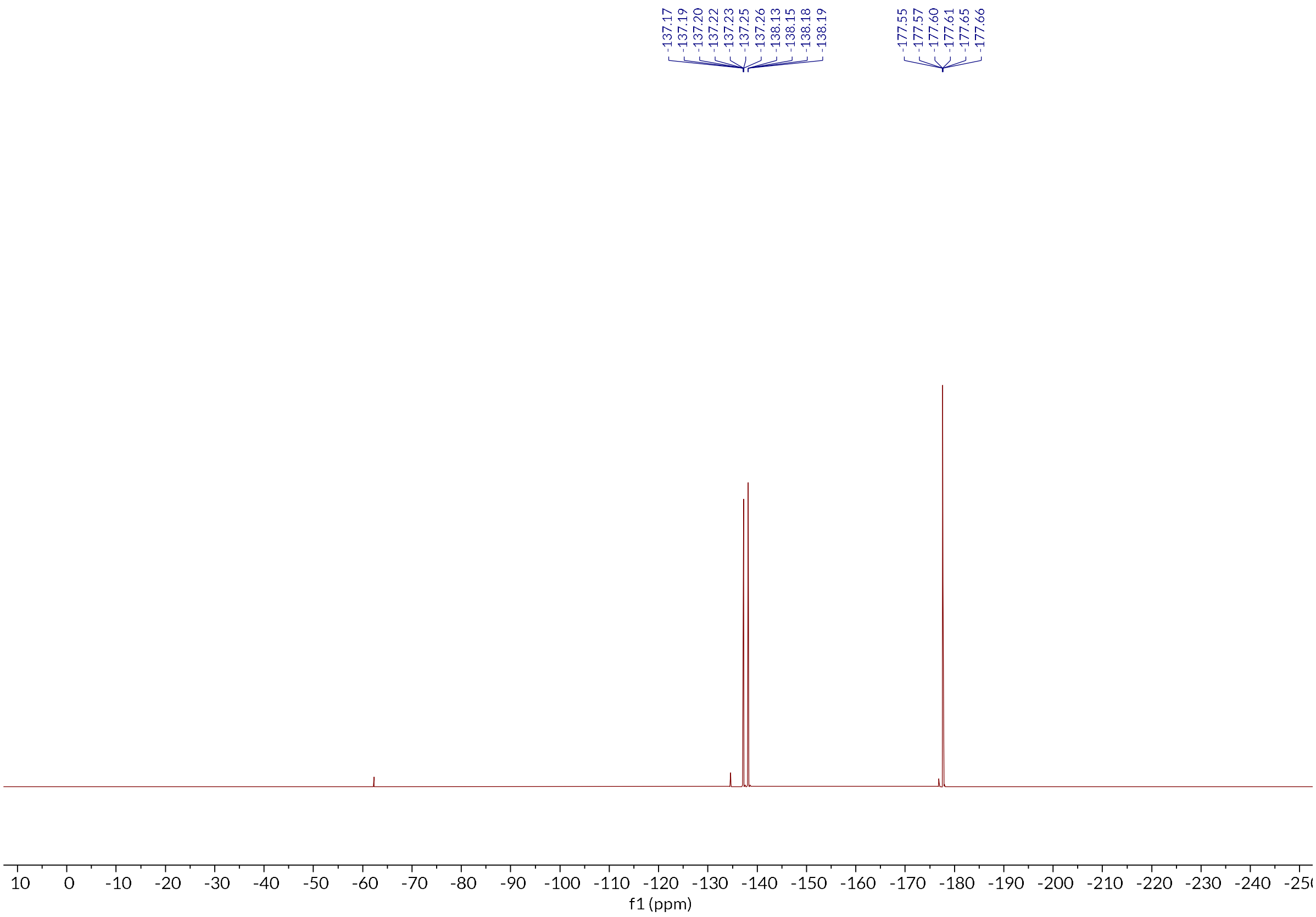
-137.17
-137.19
-137.20
-137.22
-137.23
-137.25
-137.26
-138.13
-138.15
-138.18
-138.19
-177.55
-177.57
-177.60
-177.61
-177.65
-177.66
10
0
-10
-20
-30
-40
-50
-60
-70
-80
-90
-100
-110
-120
-130
-140
-150
-160
-170
-180
-190
-200
-210
-220
-230
-240
f1 (ppm)

(±)-1-Benzyl-4,7-diphenyl-1,2,3,4-tetrahydroquinoline (2bk-a)

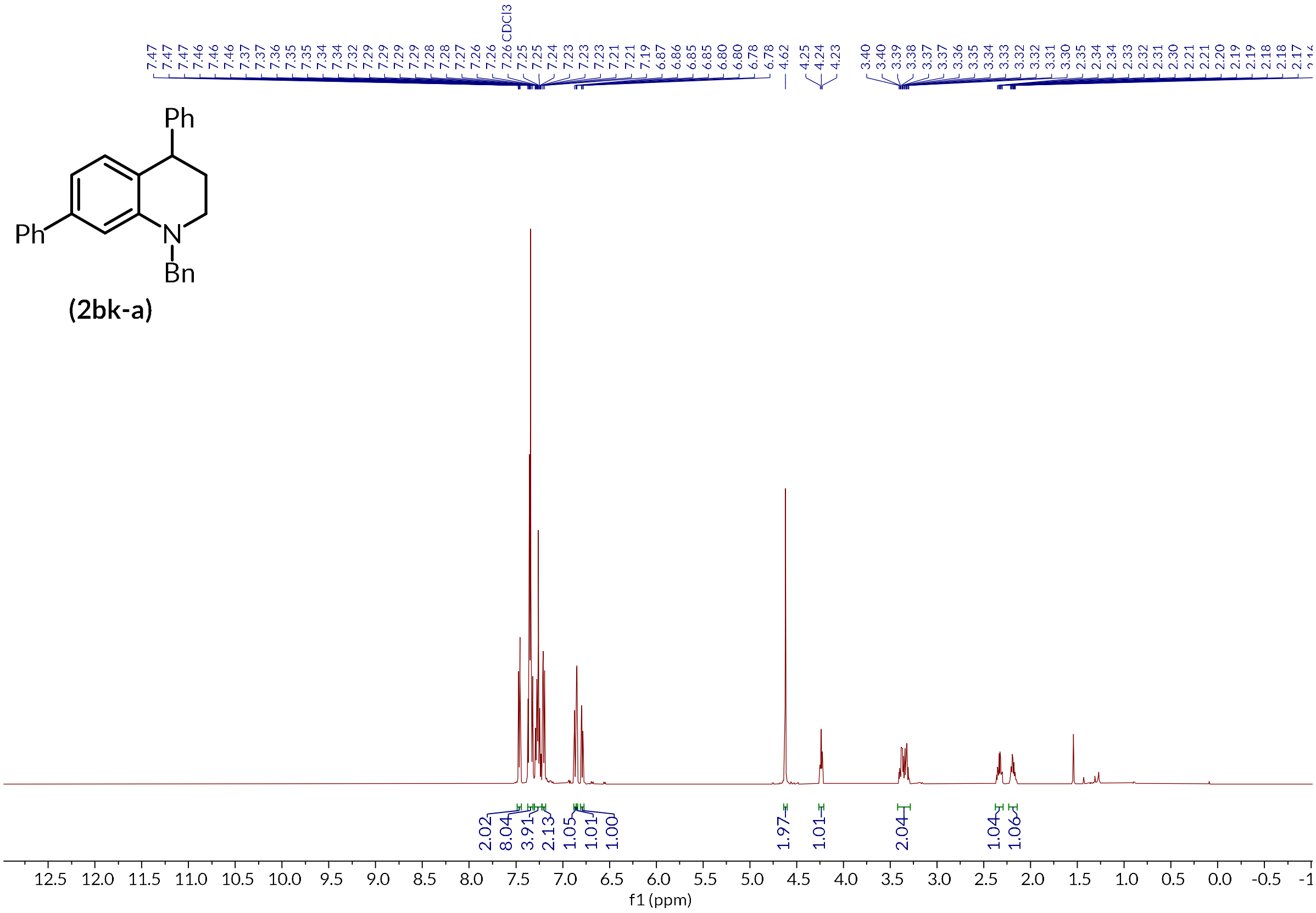

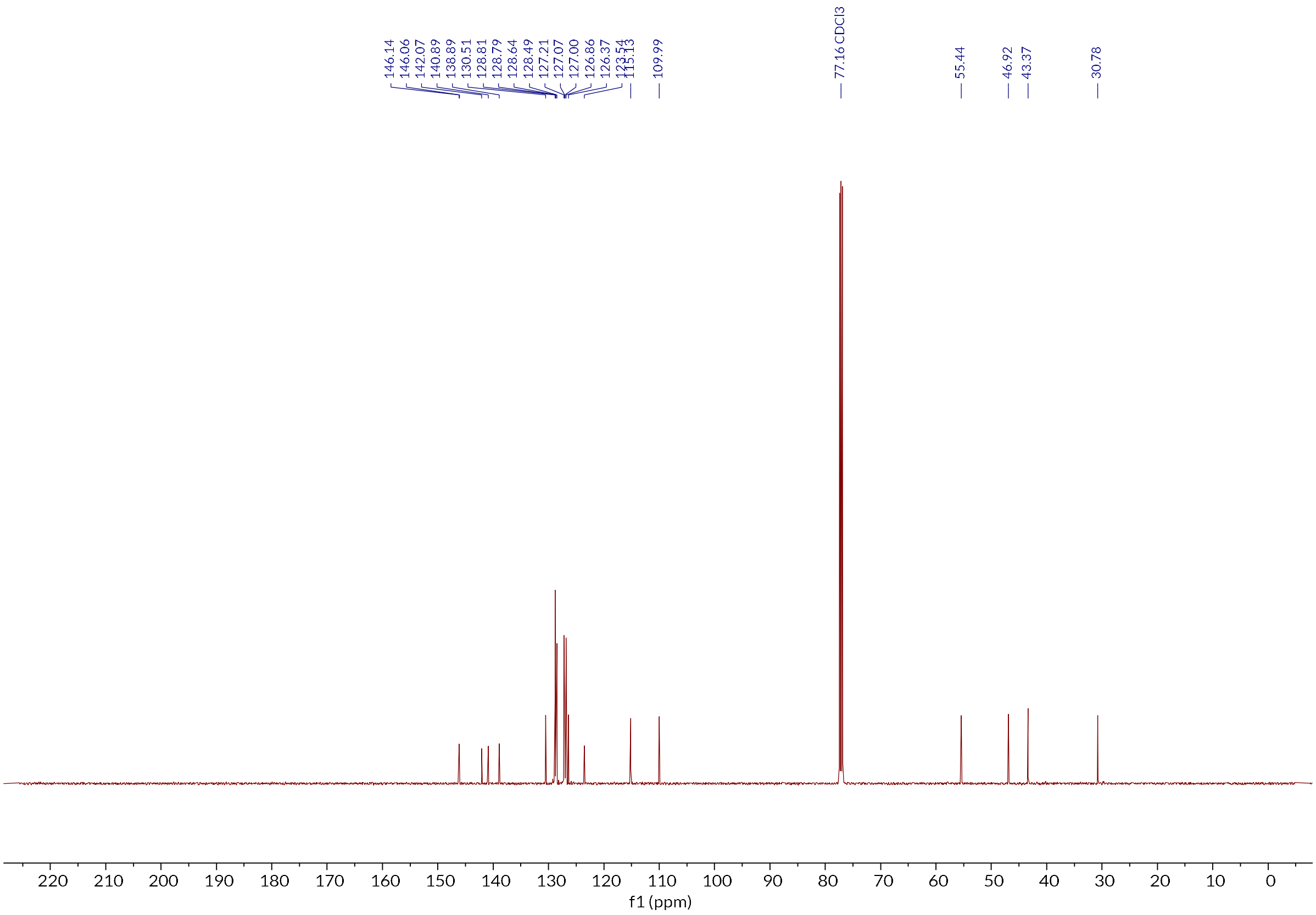

(±)-1-Benzyl-4,5-diphenyl-1,2,3,4-tetrahydroquinoline (2bk-b)

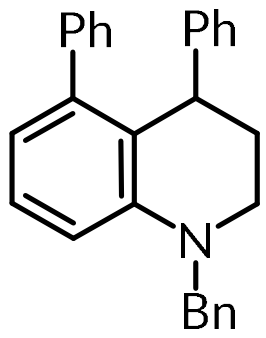

(2bk-b)

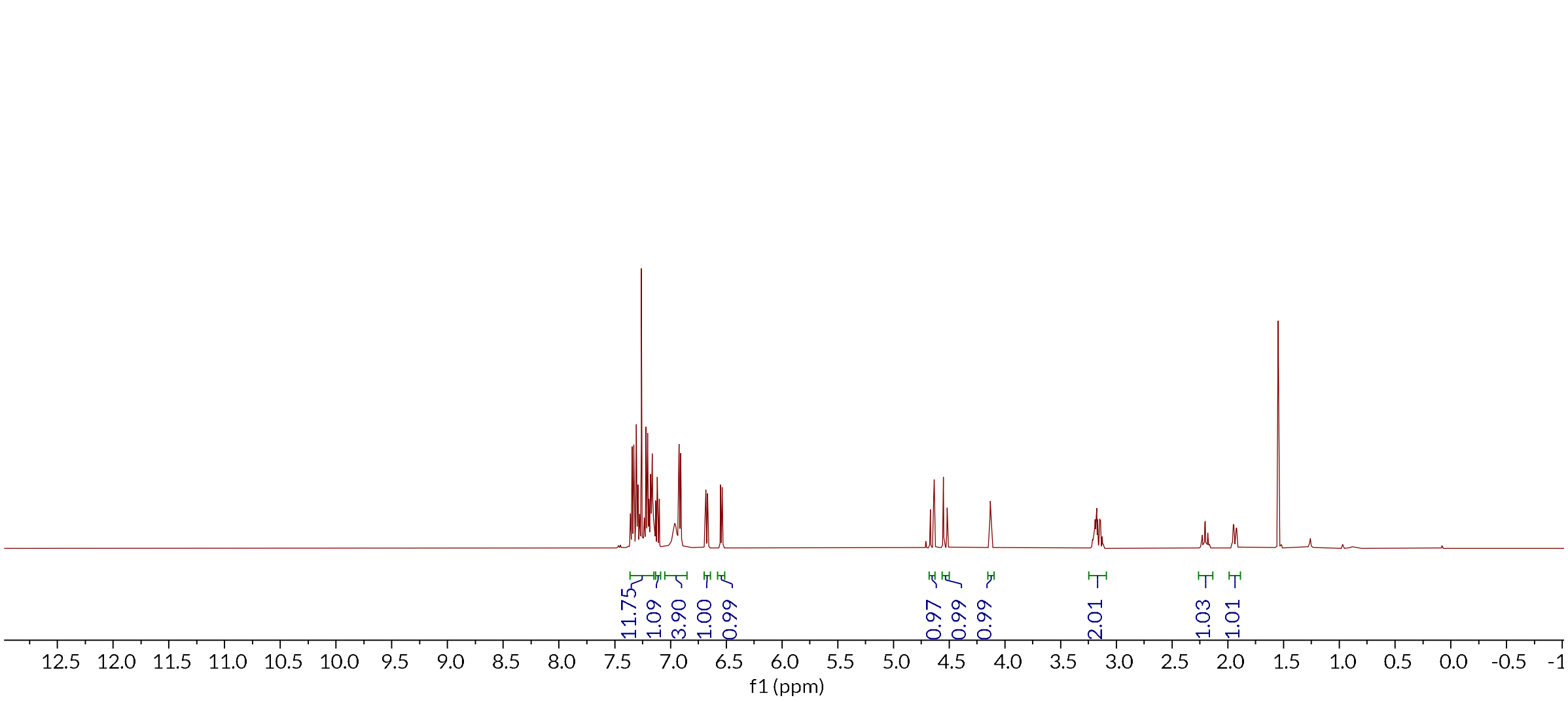

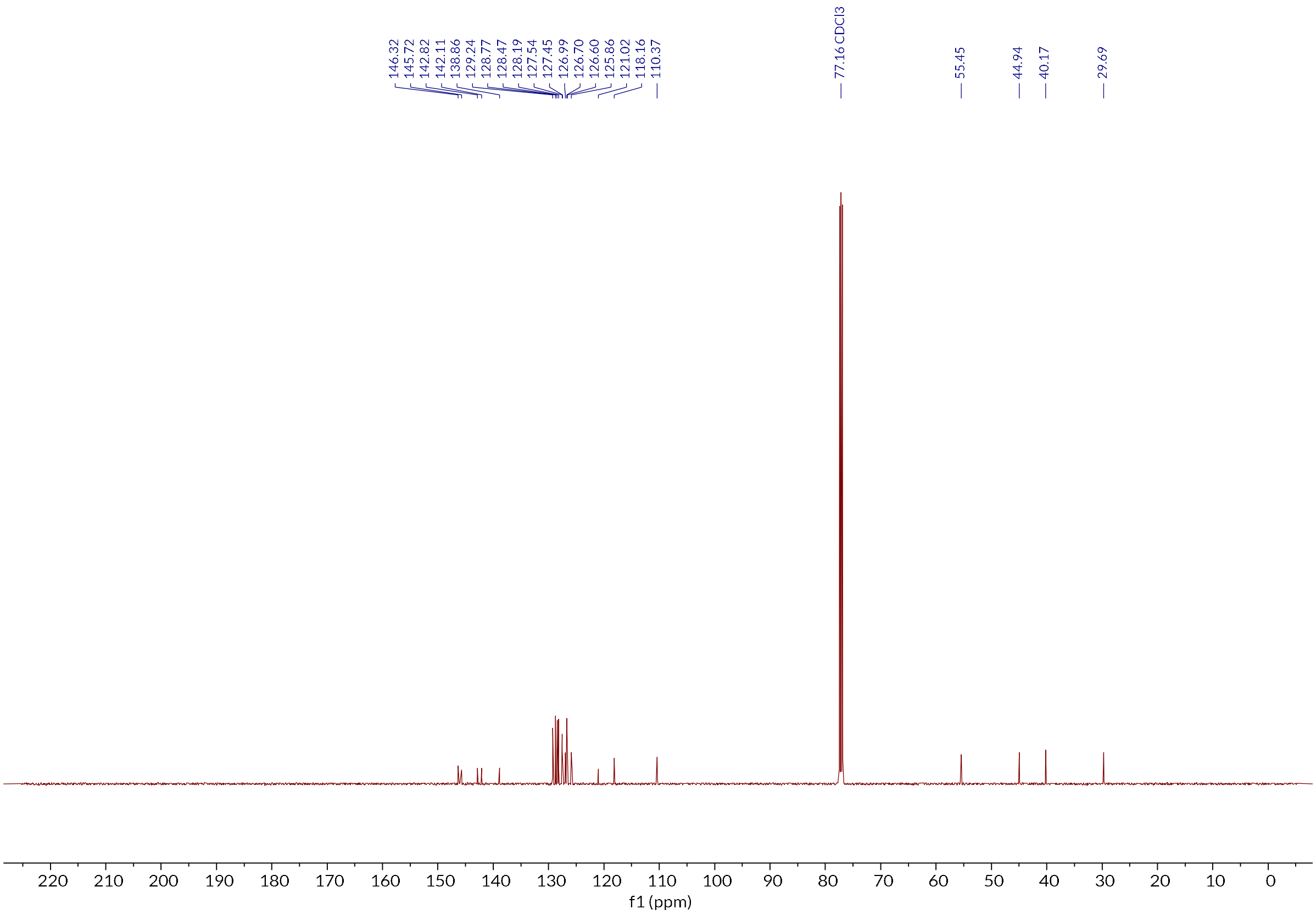

(±)-1-Benzyl-7-(tert-butyl)-4-phenyl-1,2,3,4-tetrahydroquinoline (2bl)

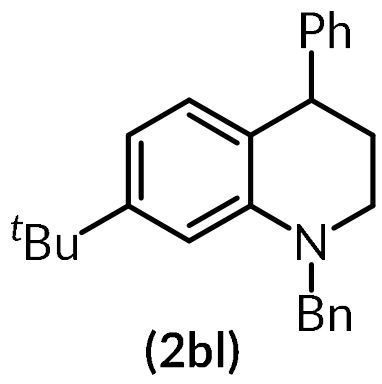

(2bl)

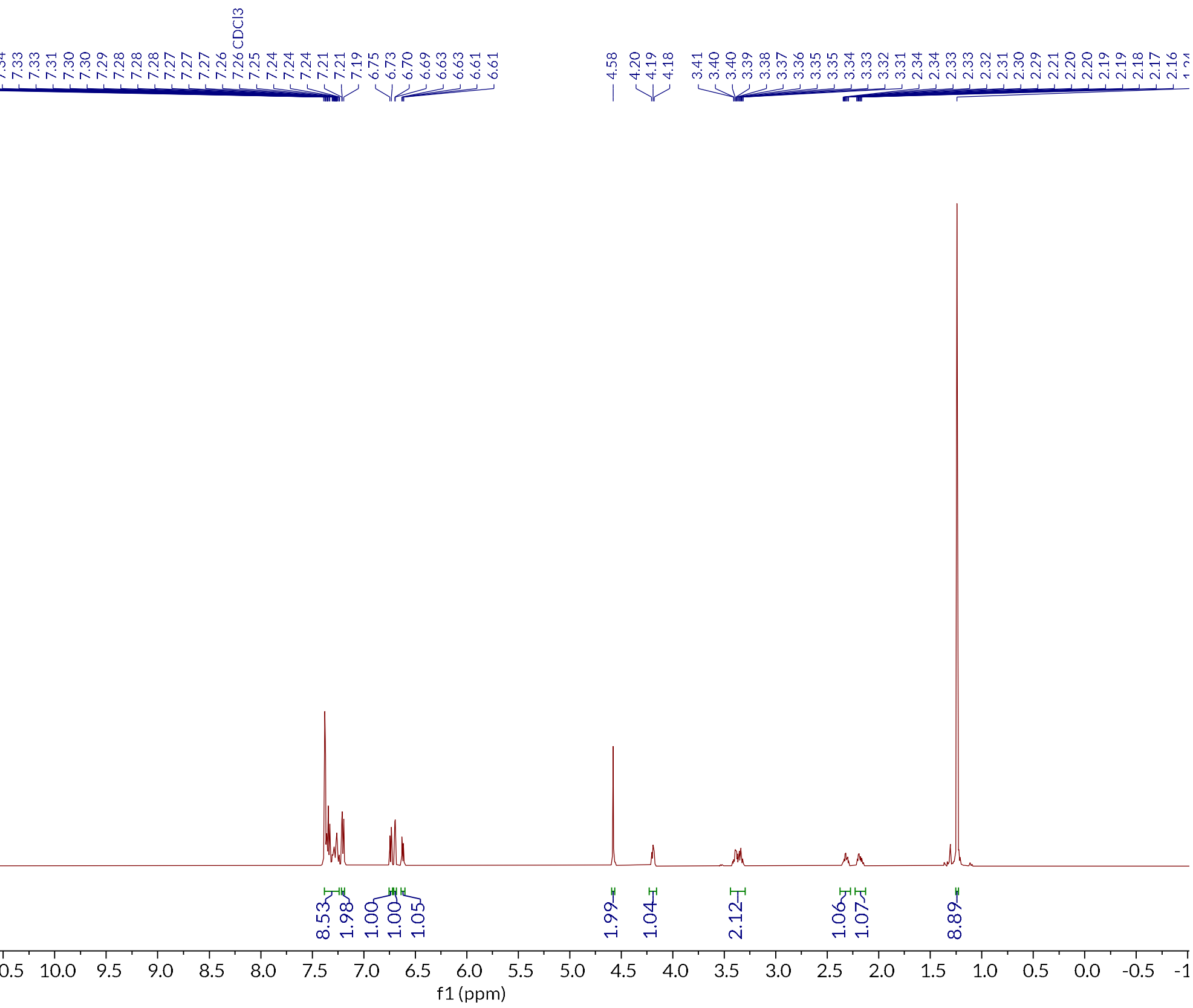

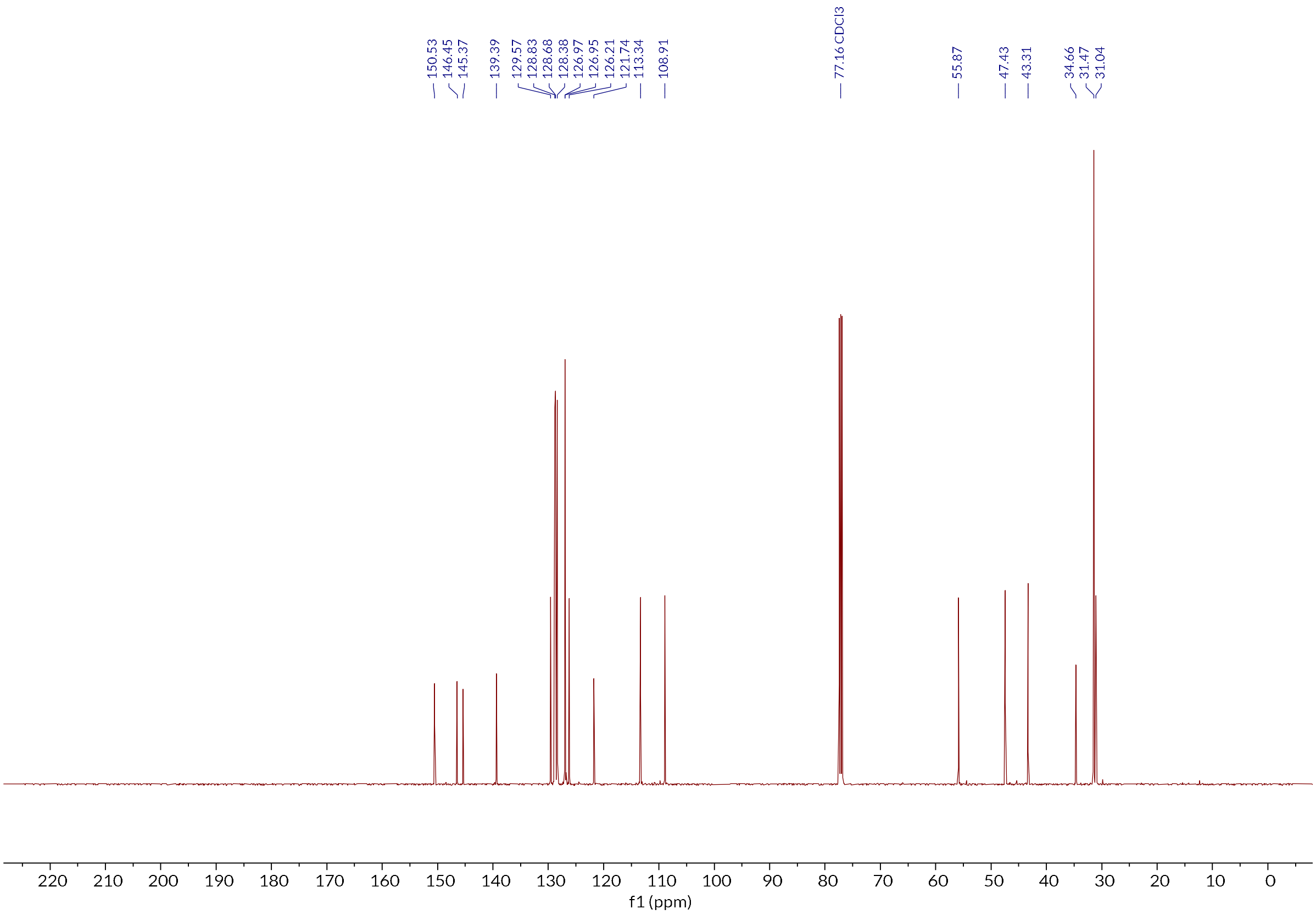

(±)-1-Benzyl-7-methoxy-4-phenyl-1,2,3,4-tetrahydroquinoline (2bm-a)

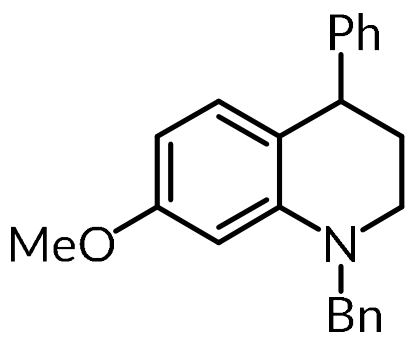

(2bm-a)

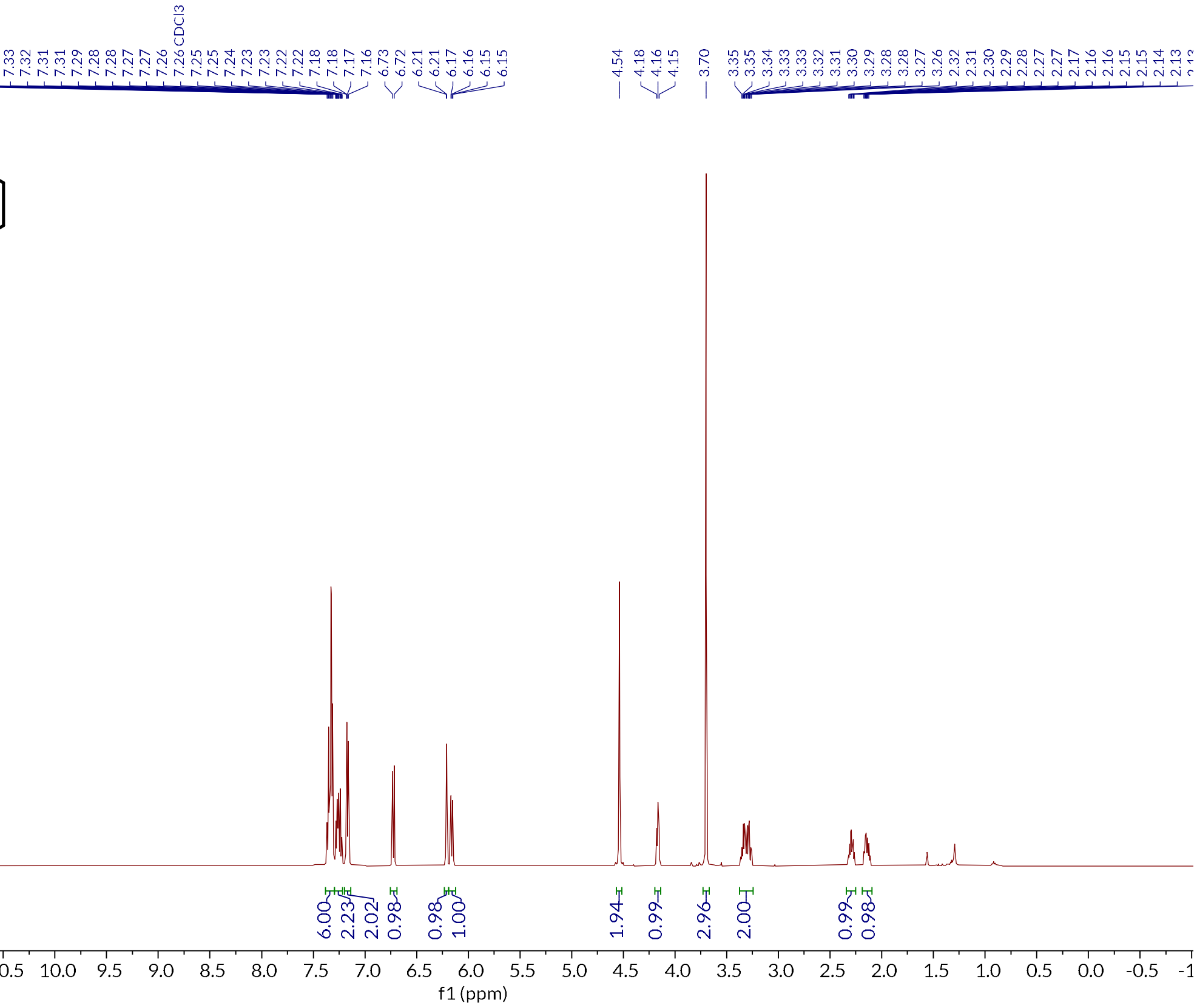

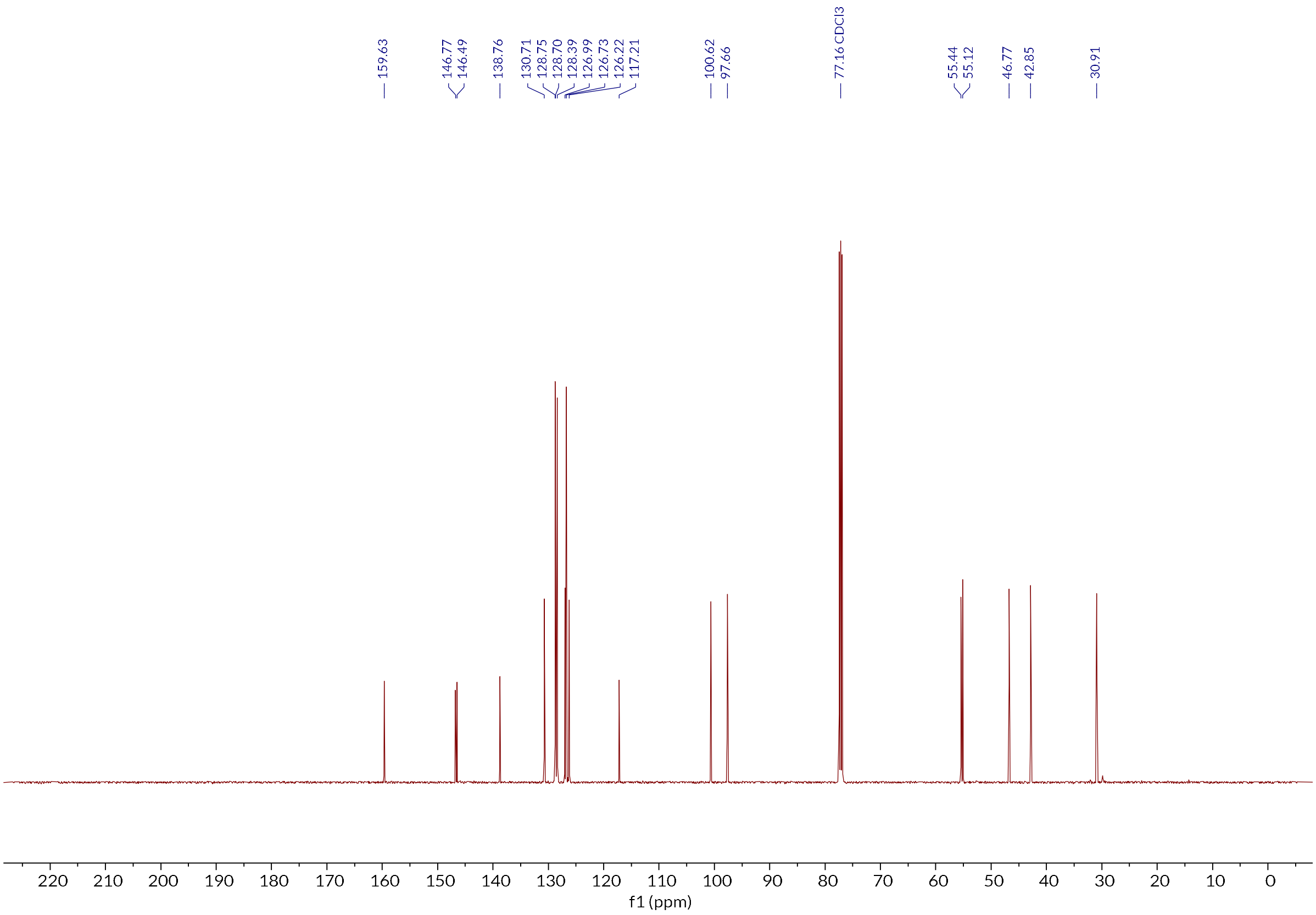

(±)-1-Benzyl-5-methoxy-4-phenyl-1,2,3,4-tetrahydroquinoline (2bm-b)

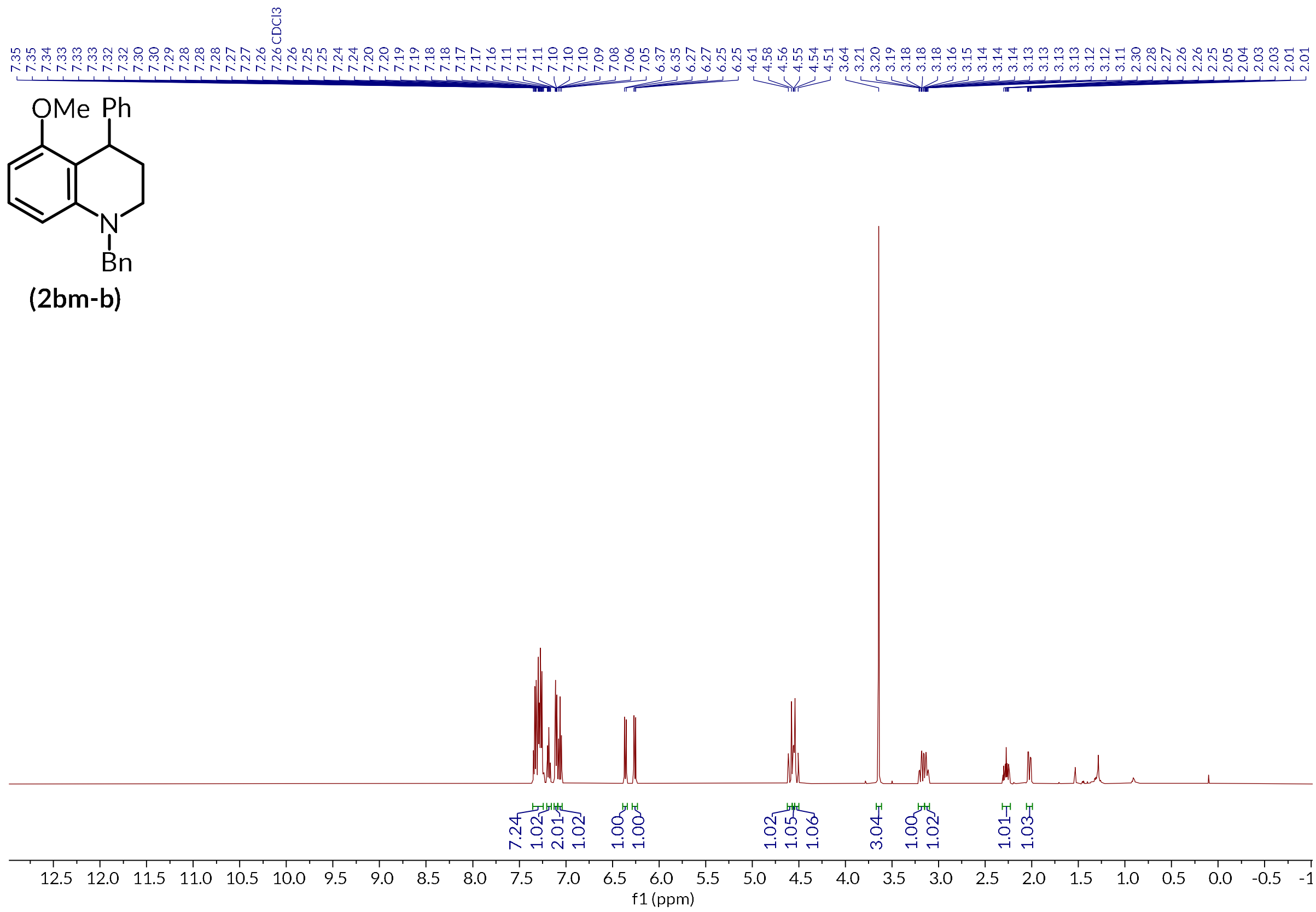

(2bm-b)

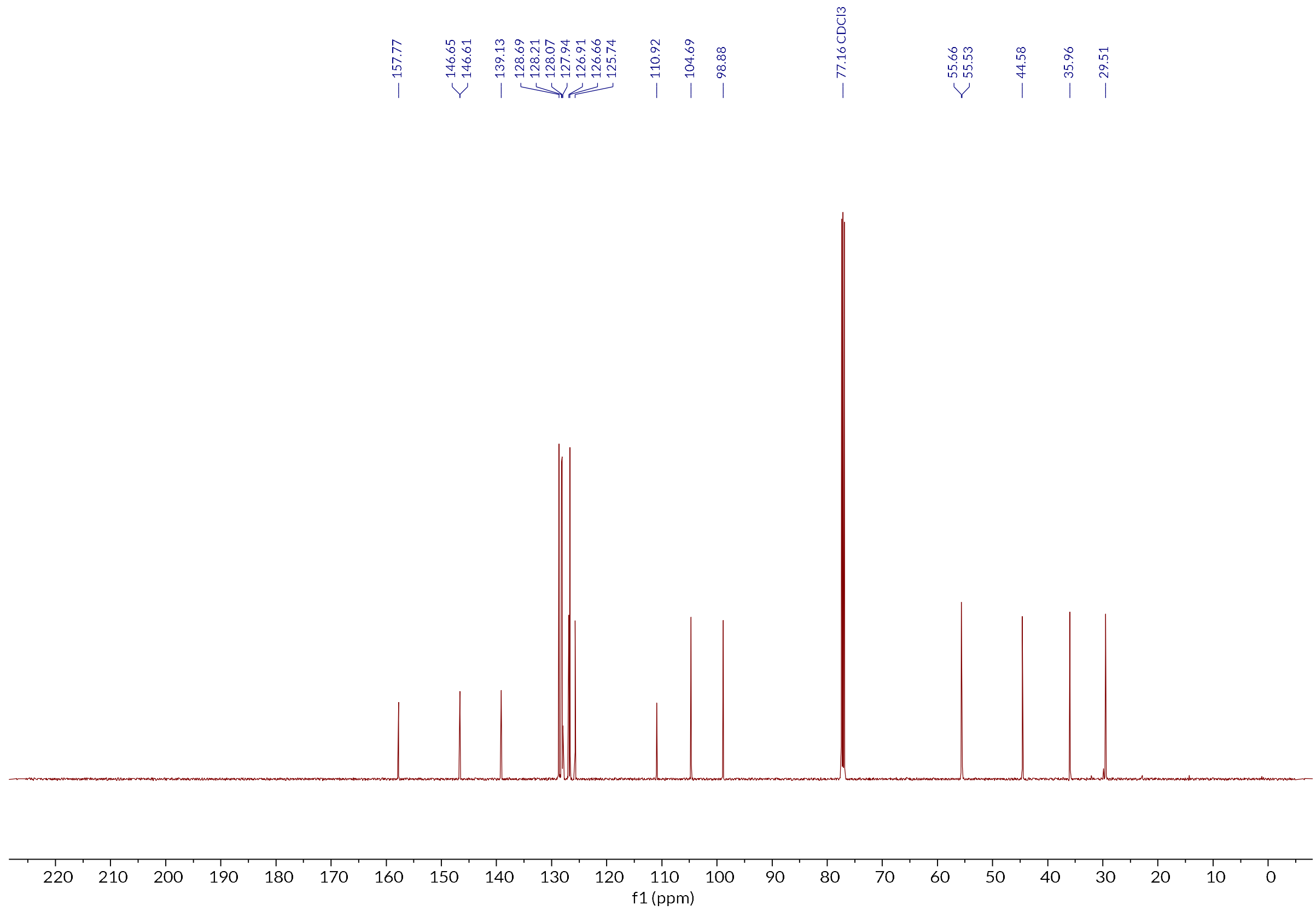

(±)-1-Benzyl-7-fluoro-4-phenyl-1,2,3,4-tetrahydroquinoline (2bn-a) and (±)-1-Benzyl-5-fluoro-4-phenyl-1,2,3,4-tetrahydroquinoline (2bn-b)

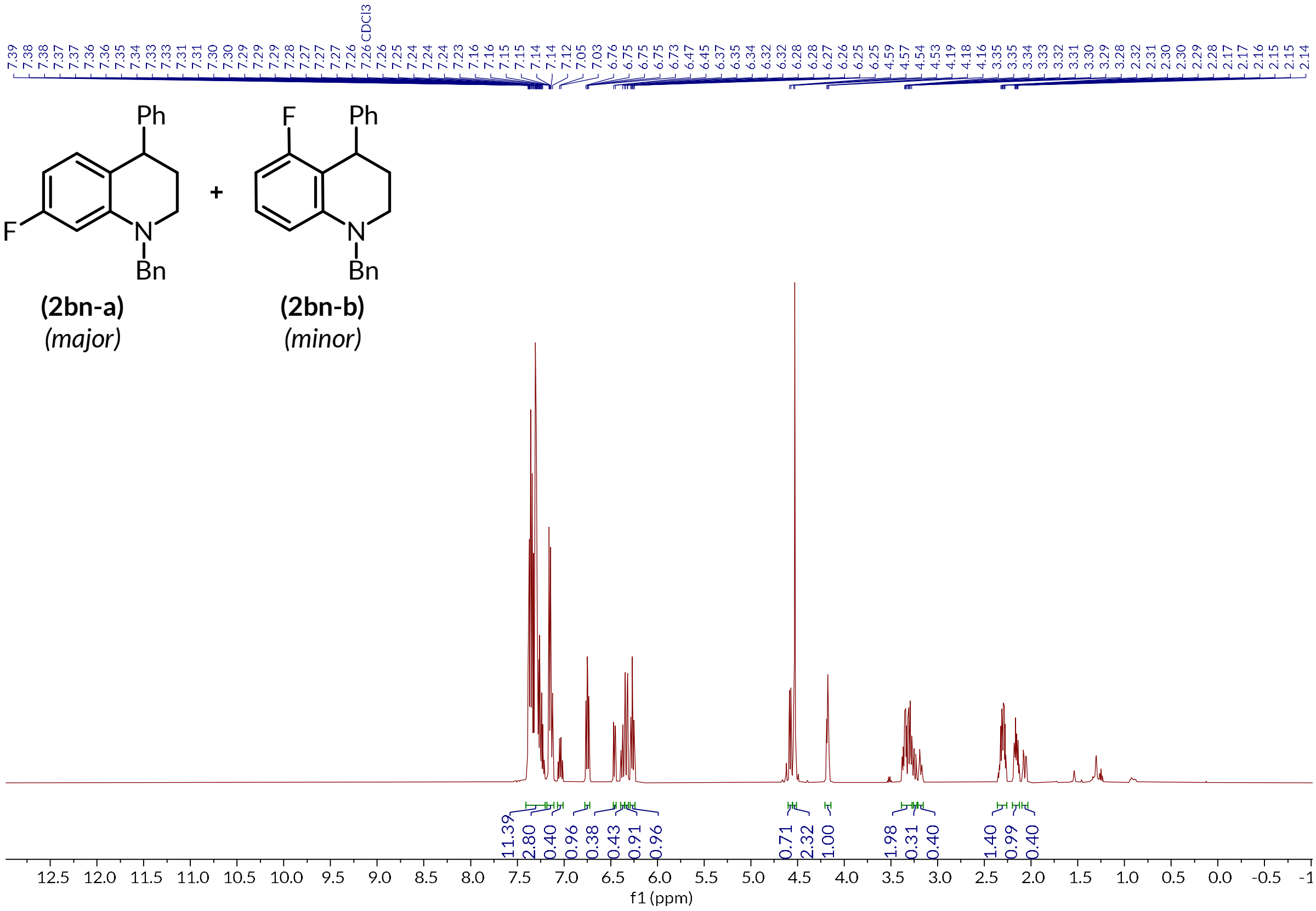

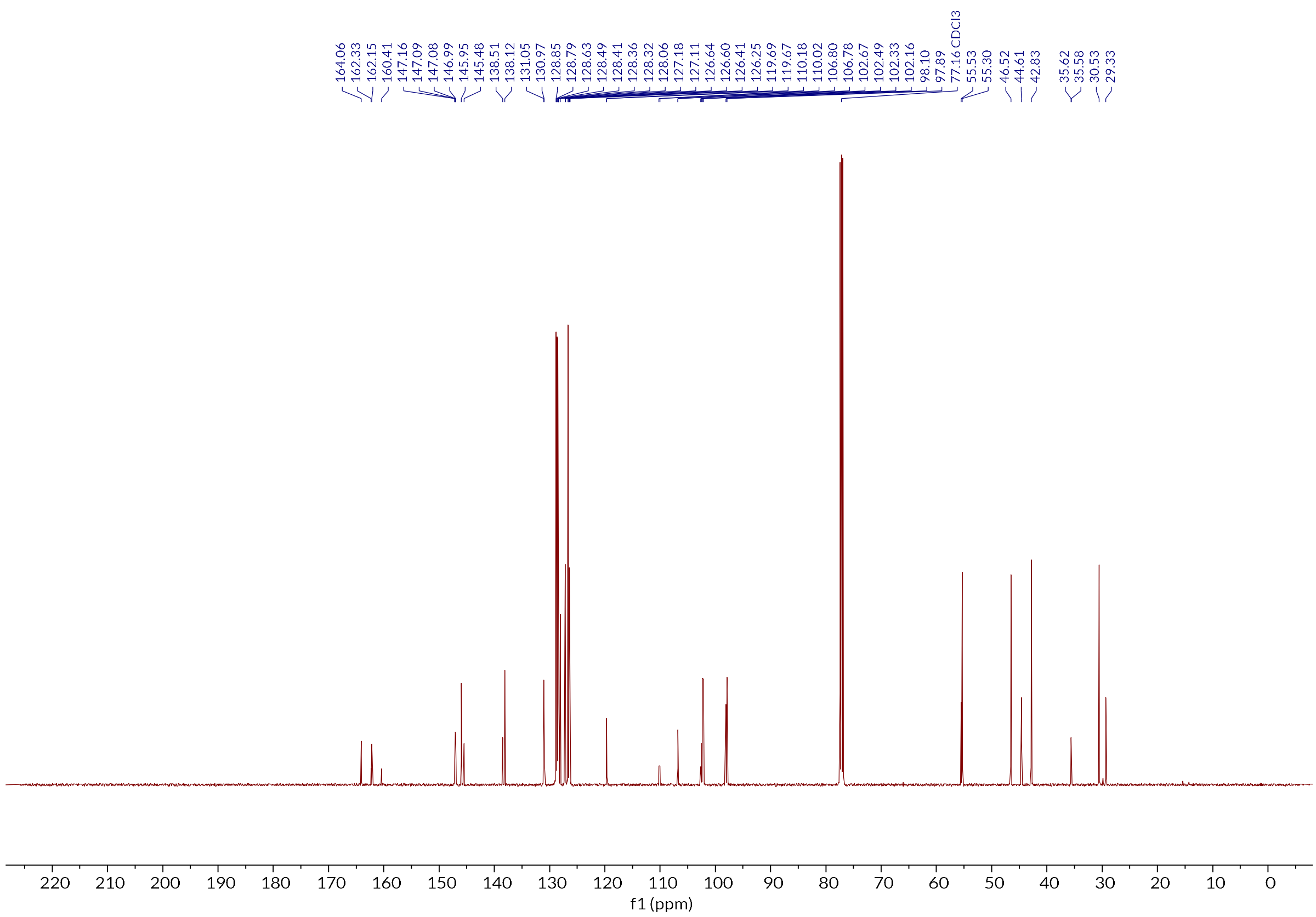

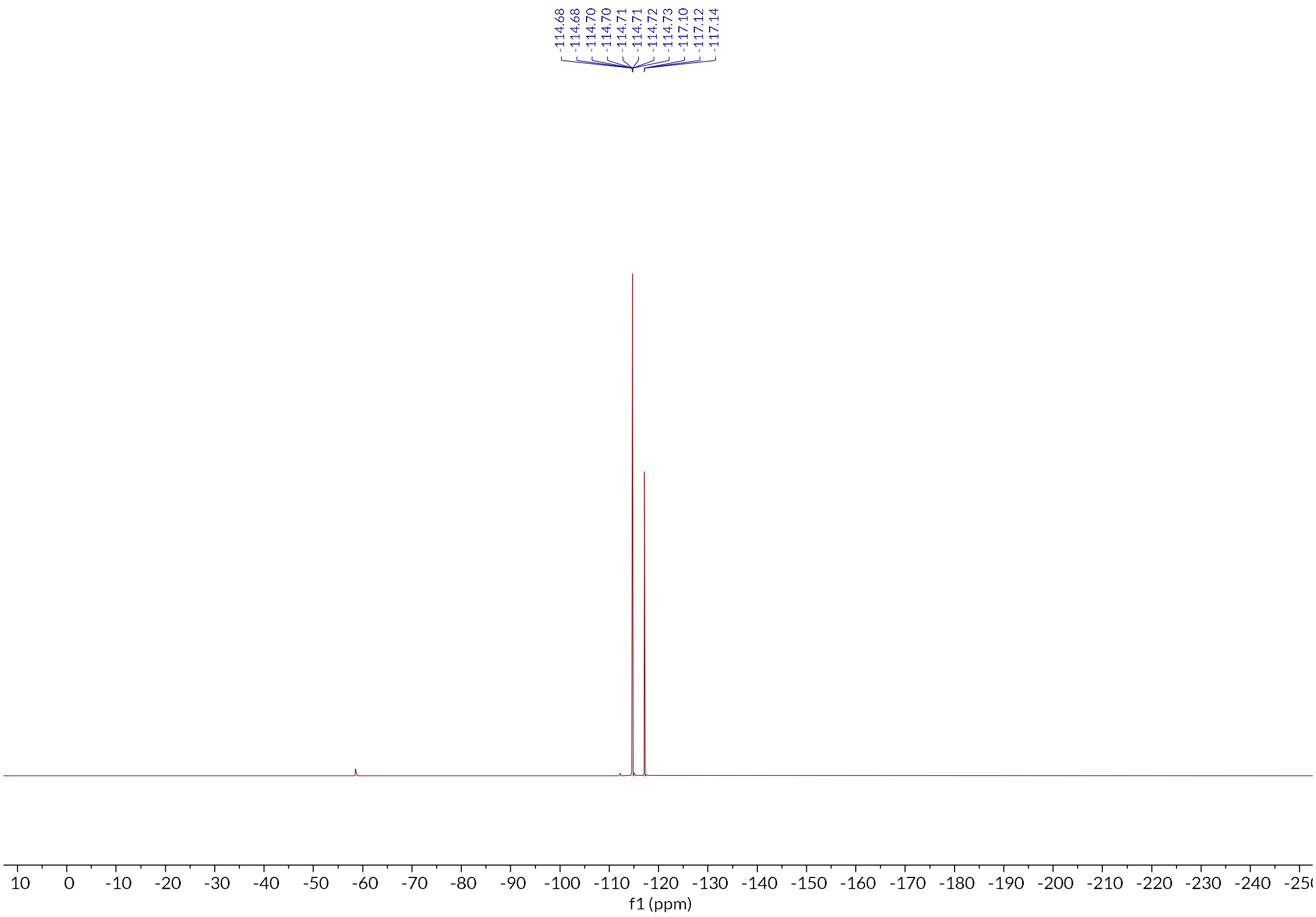
-114.68
-114.68
-114.70
-114.70
-114.71
-114.71
-114.72
-114.73
-117.10
-117.12
-117.14
10
0
-10
-20
-30
-40
-50
-60
-70
-80
-90
-100
-110
-120
-130
-140
-150
-160
-170
-180
-190
-200
-210
-220
-230
-240
f1 (ppm)

(±)-1-Benzyl-4-phenyl-7-(trifluoromethyl)-1,2,3,4-tetrahydroquinoline (2bo-a) and (±)-1-Benzyl-4-phenyl-5-(trifluoromethyl)-1,2,3,4-tetrahydroquinoline (2bo-b)

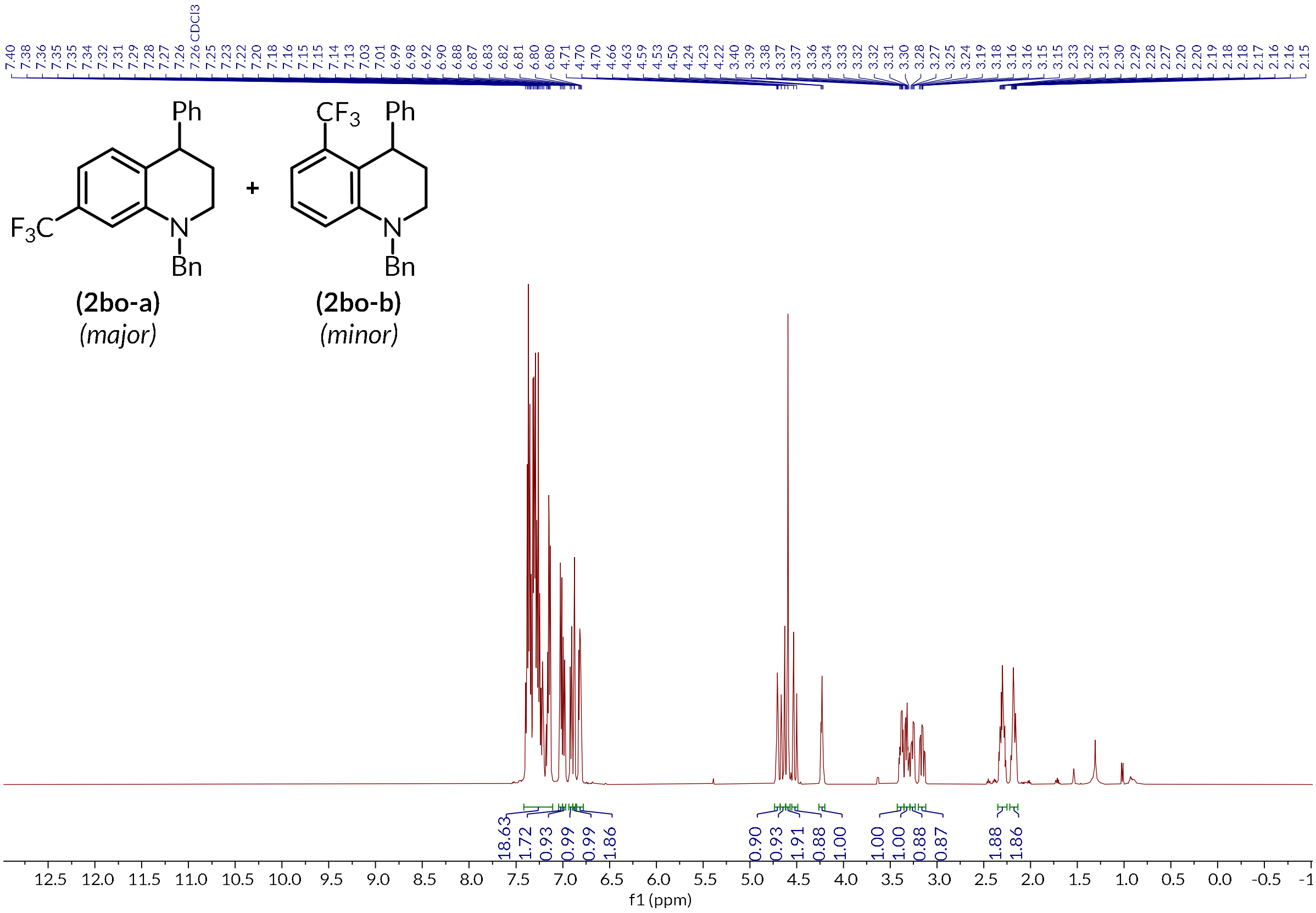

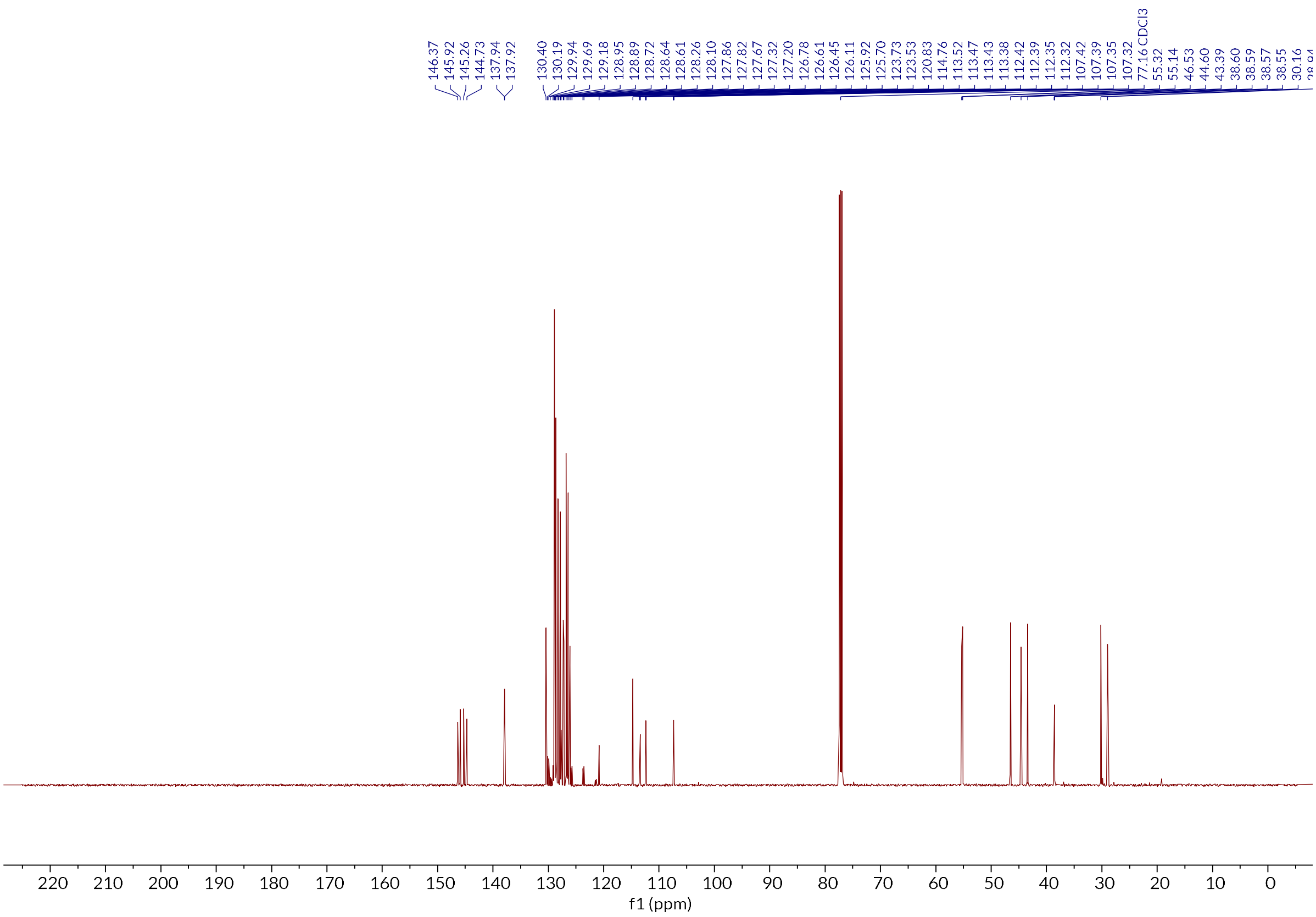

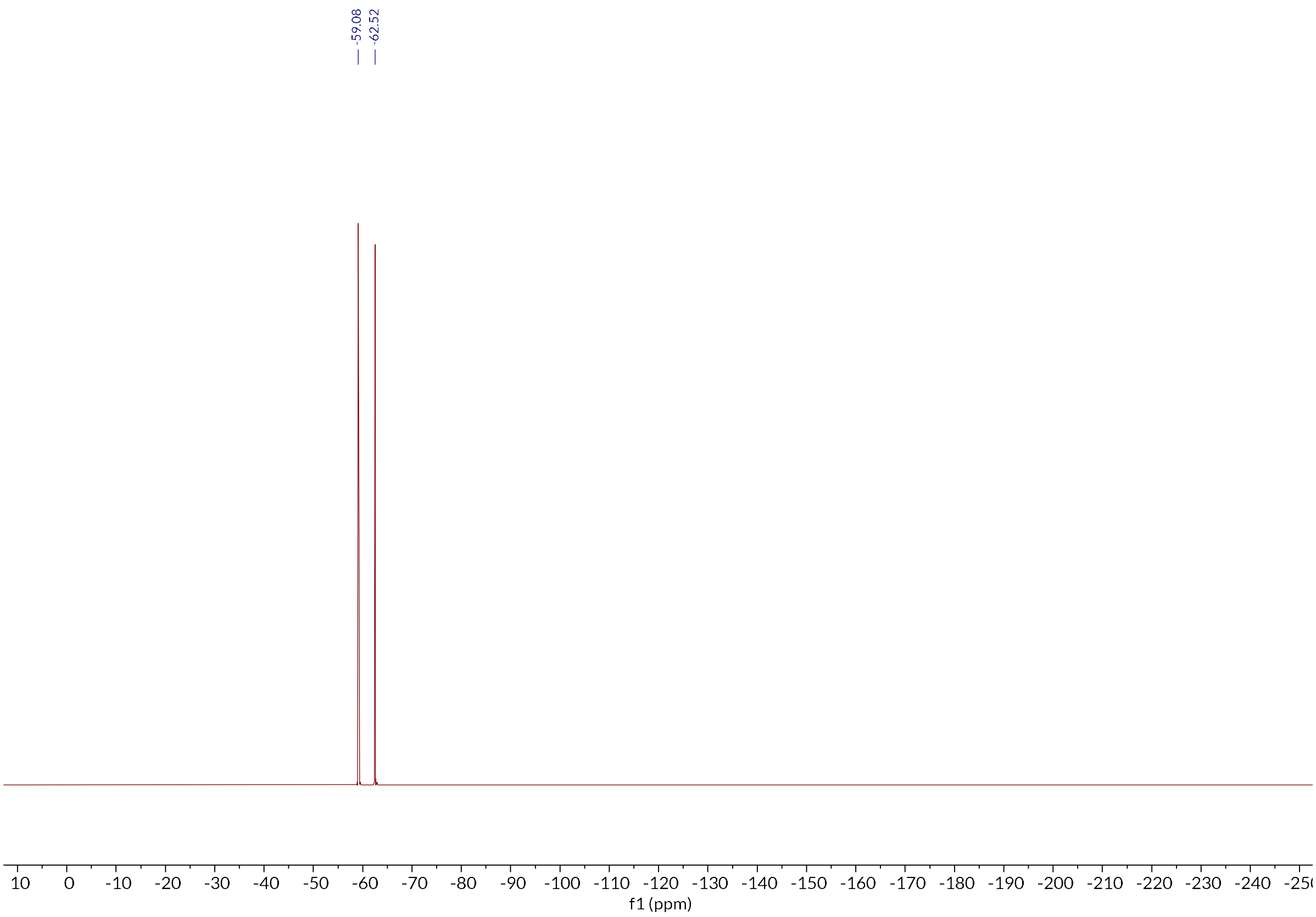
-59.08
-62.52
10 0 -10 -20 -30 -40 -50 -60 -70 -80 -90 -100 -110 -120 -130 -140 -150 -160 -170 -180 -190 -200 -210 -220 -230 -240
f1 (ppm)

(±)-1-Benzyl-4-phenyl-1,2,3,4-tetrahydrobenzo[*h*]quinoline (2bp)

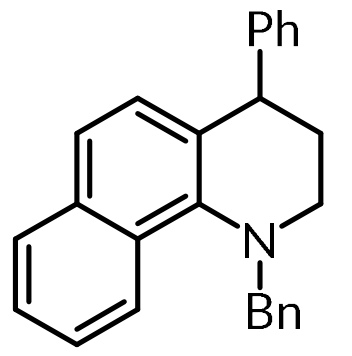

(2bp)

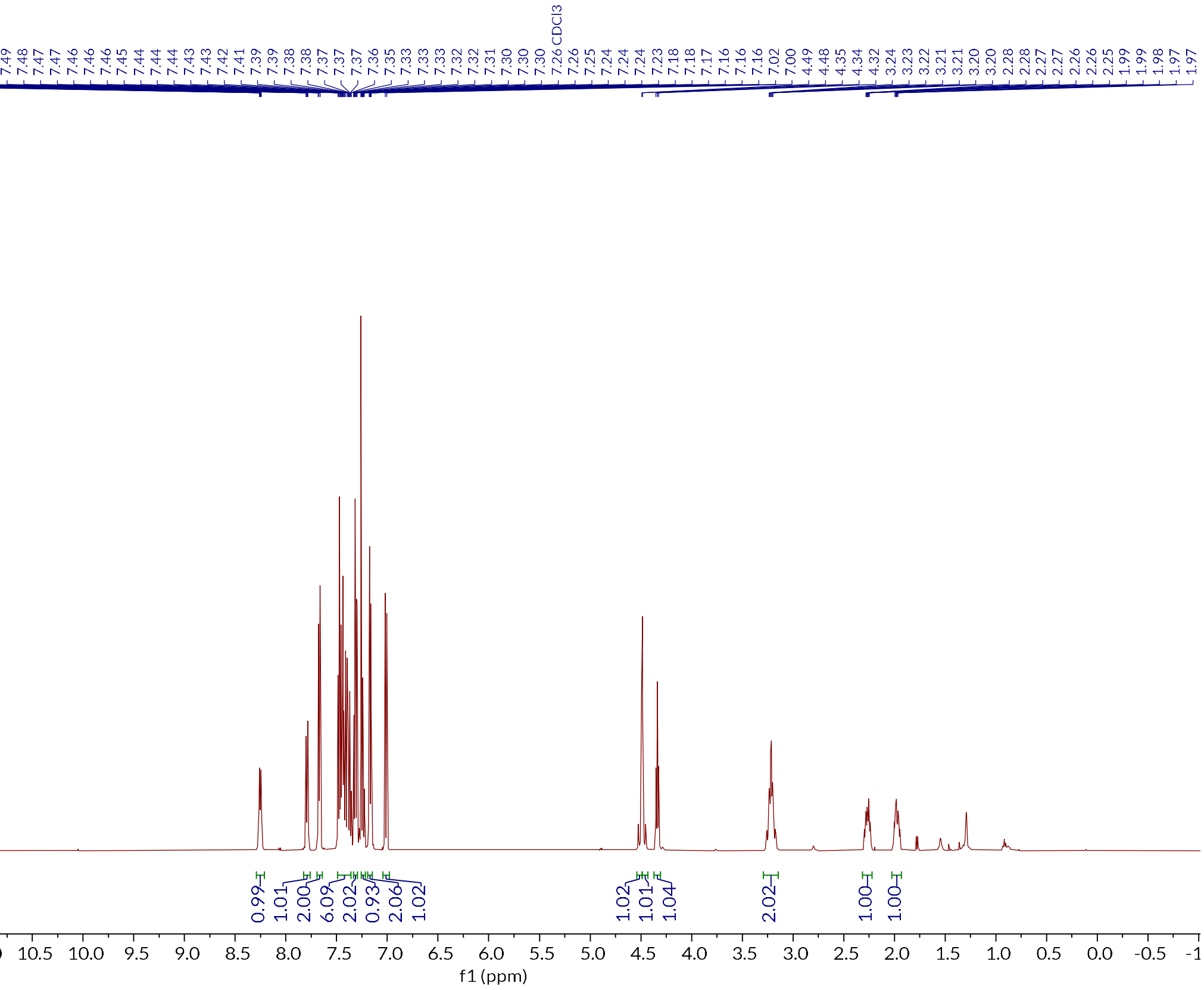

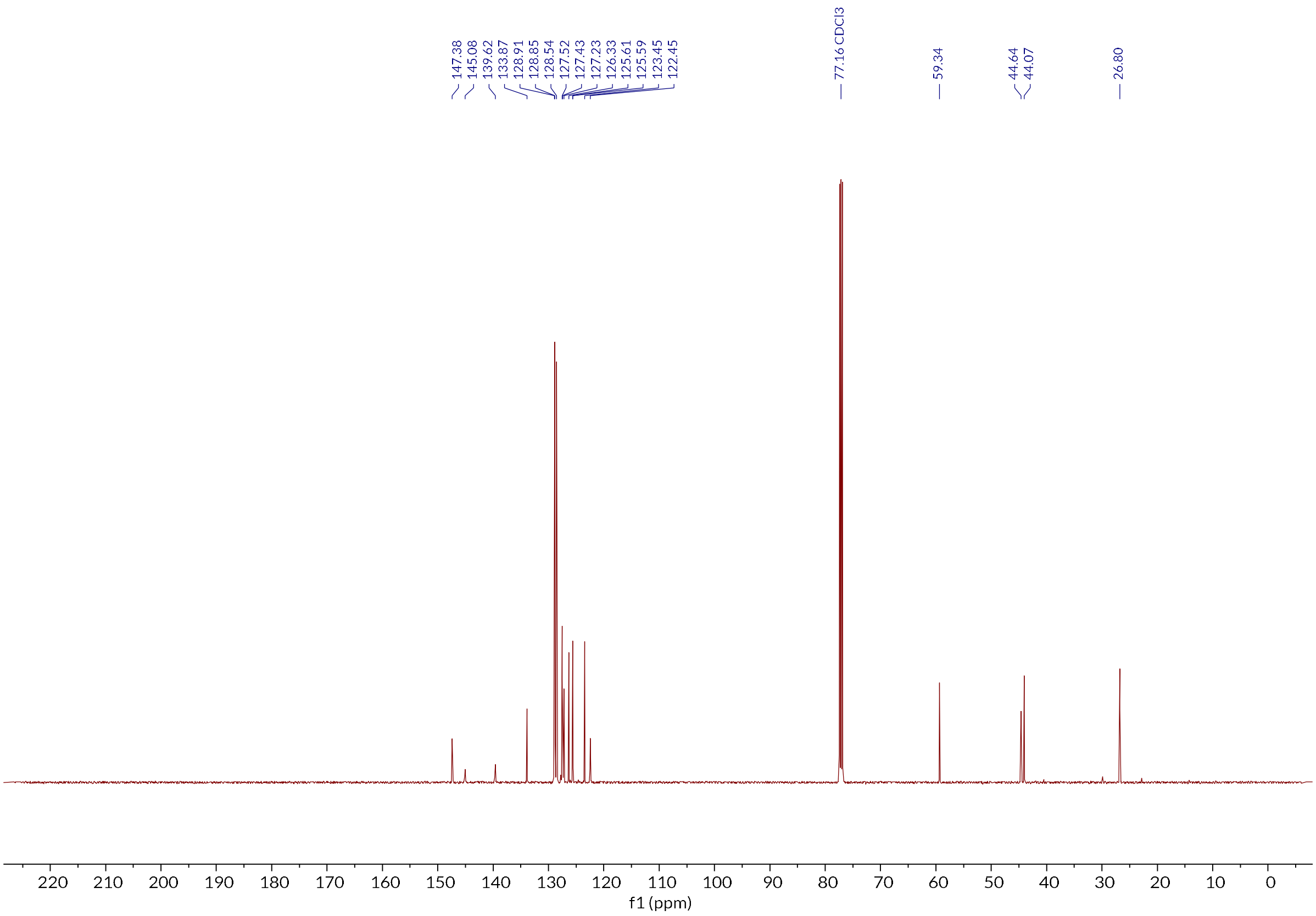

(±)-6-Phenyl-1,2,5,6-tetrahydro-4*H*-pyrrolo[3,2,1-*ij*]quinoline (2bq)

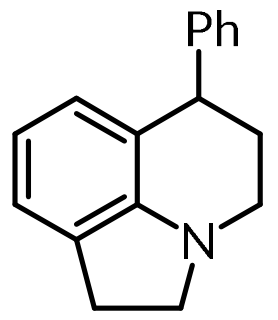

(2bq)

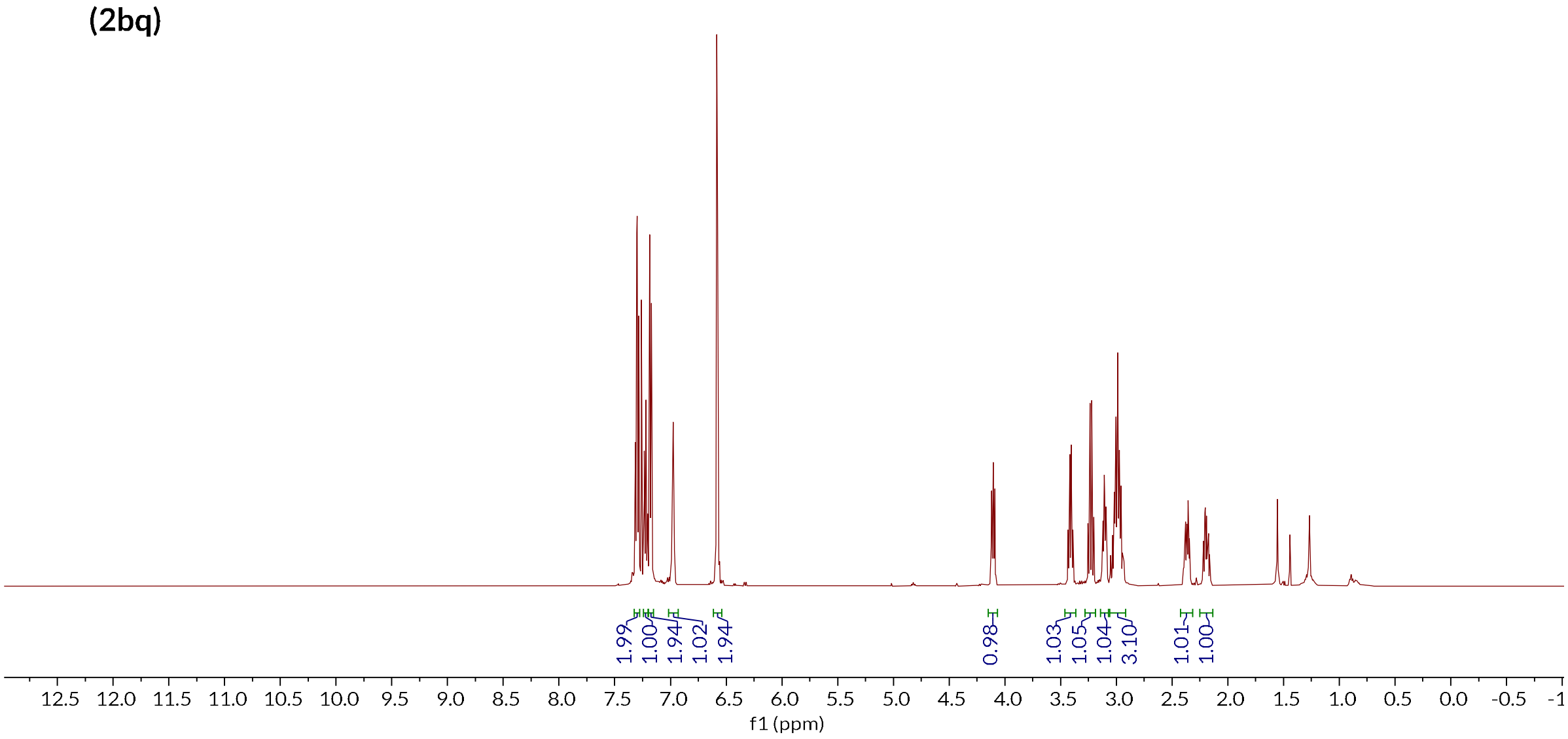

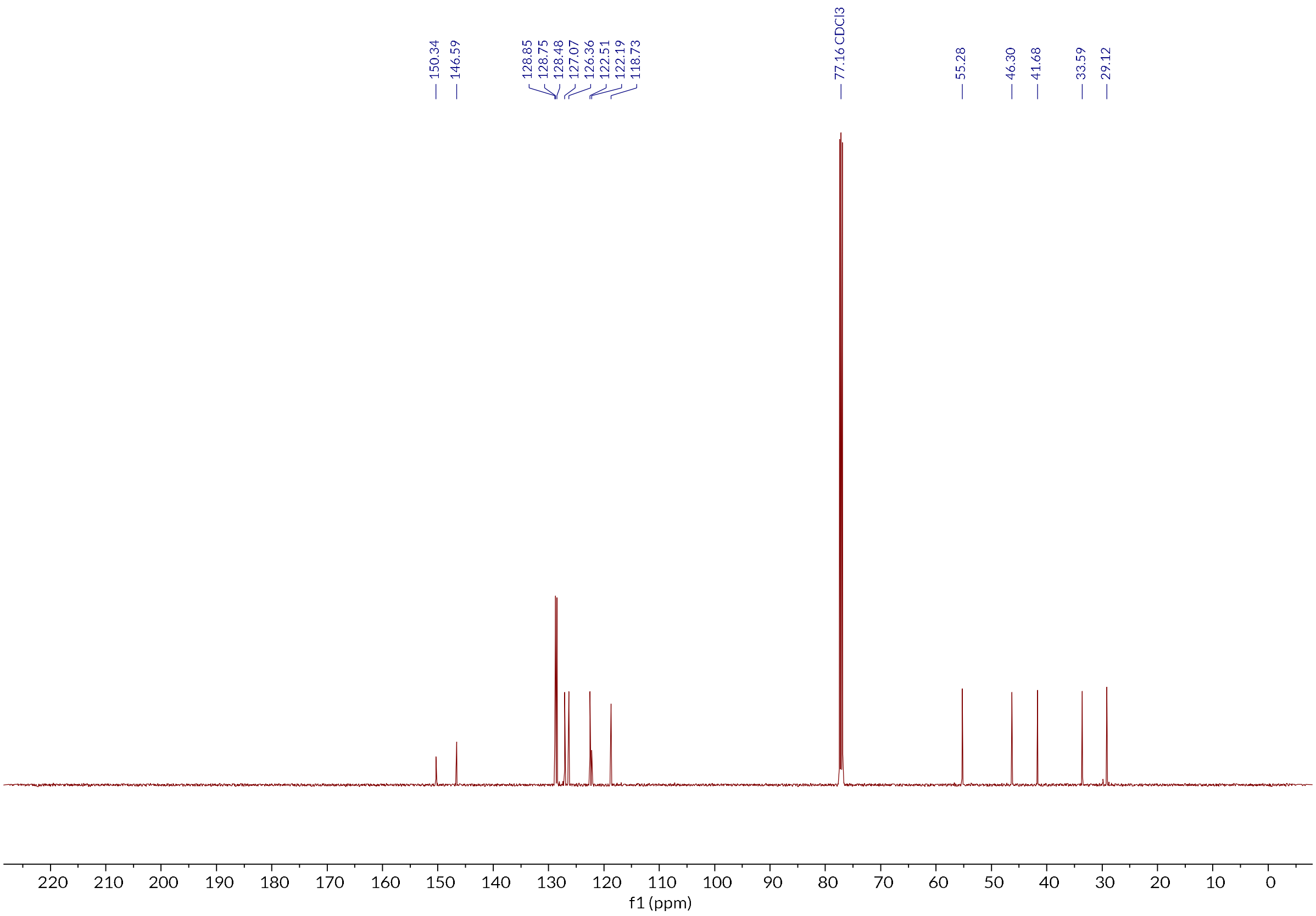

(±)-10-Chloro-1-phenyl-2,3,6,7-tetrahydro-1*H*-azepino[3,2,1-*ij*]quinolin-8(5*H*)-one (2br)

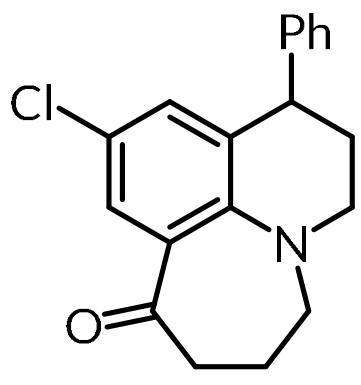

(2br)

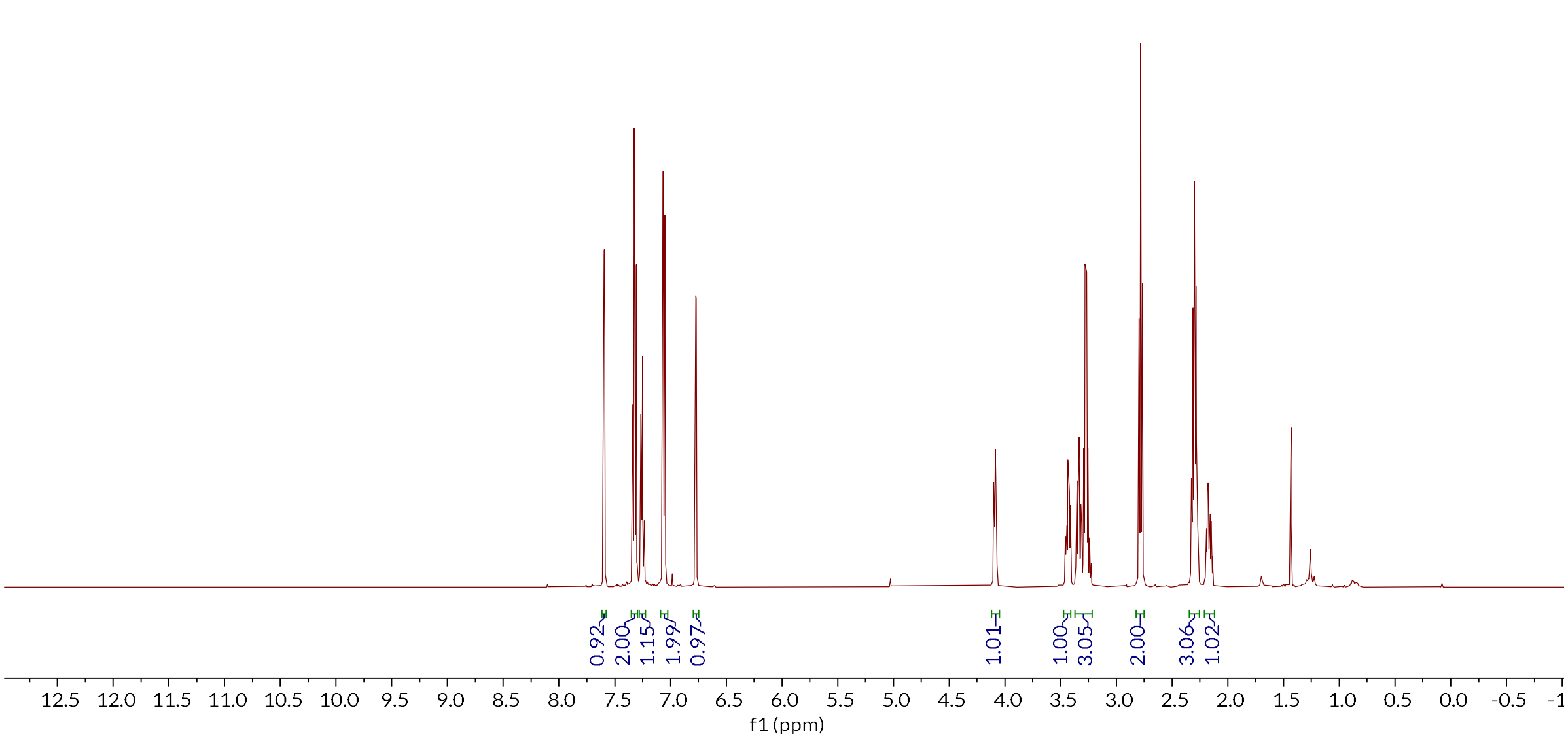

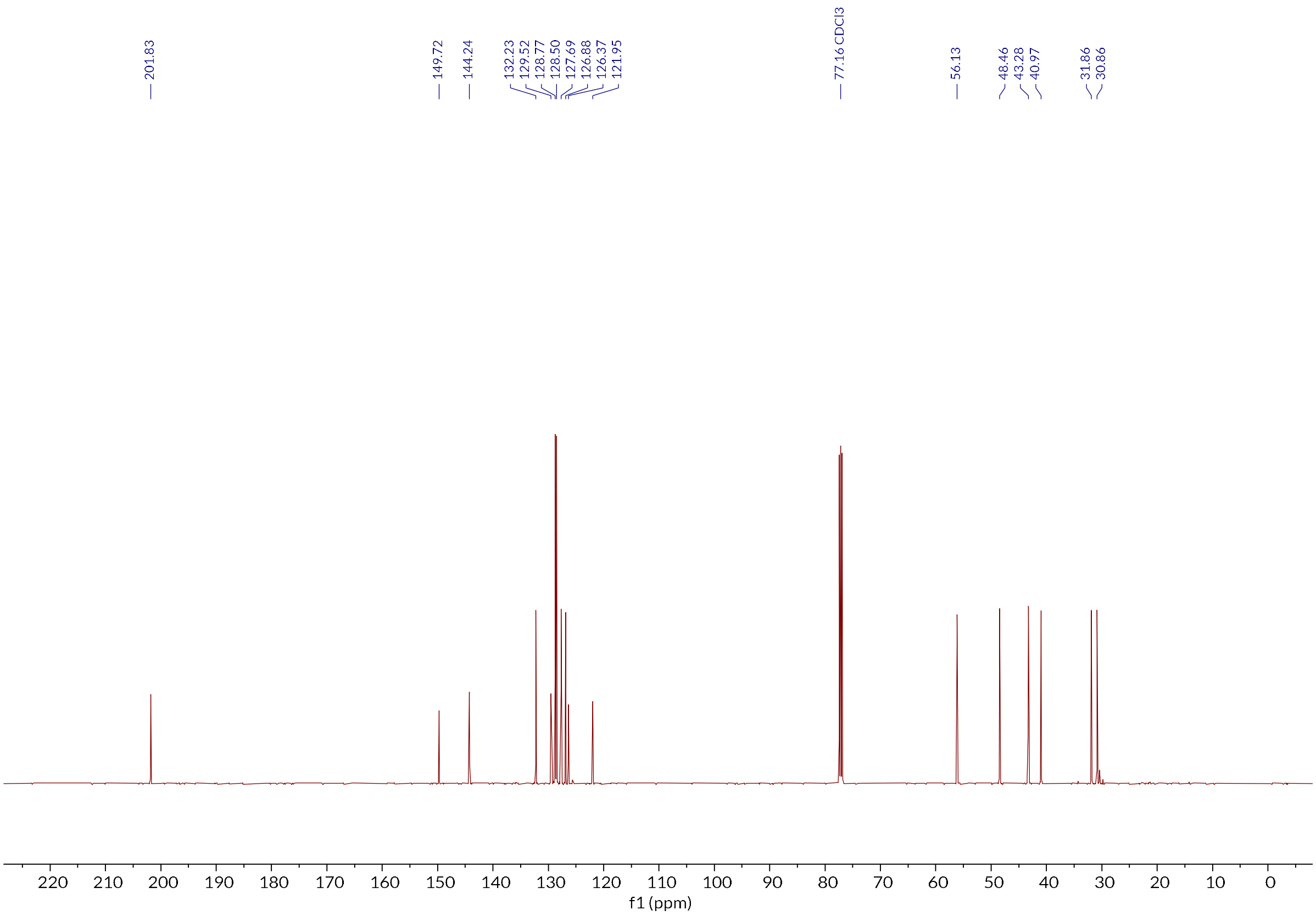

(±)-4-Phenyl-1,2,3,4-tetrahydroquinoline (4a)

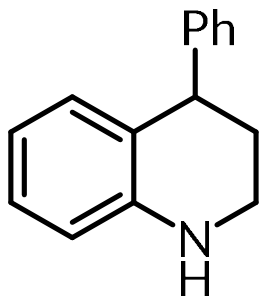

(4a)

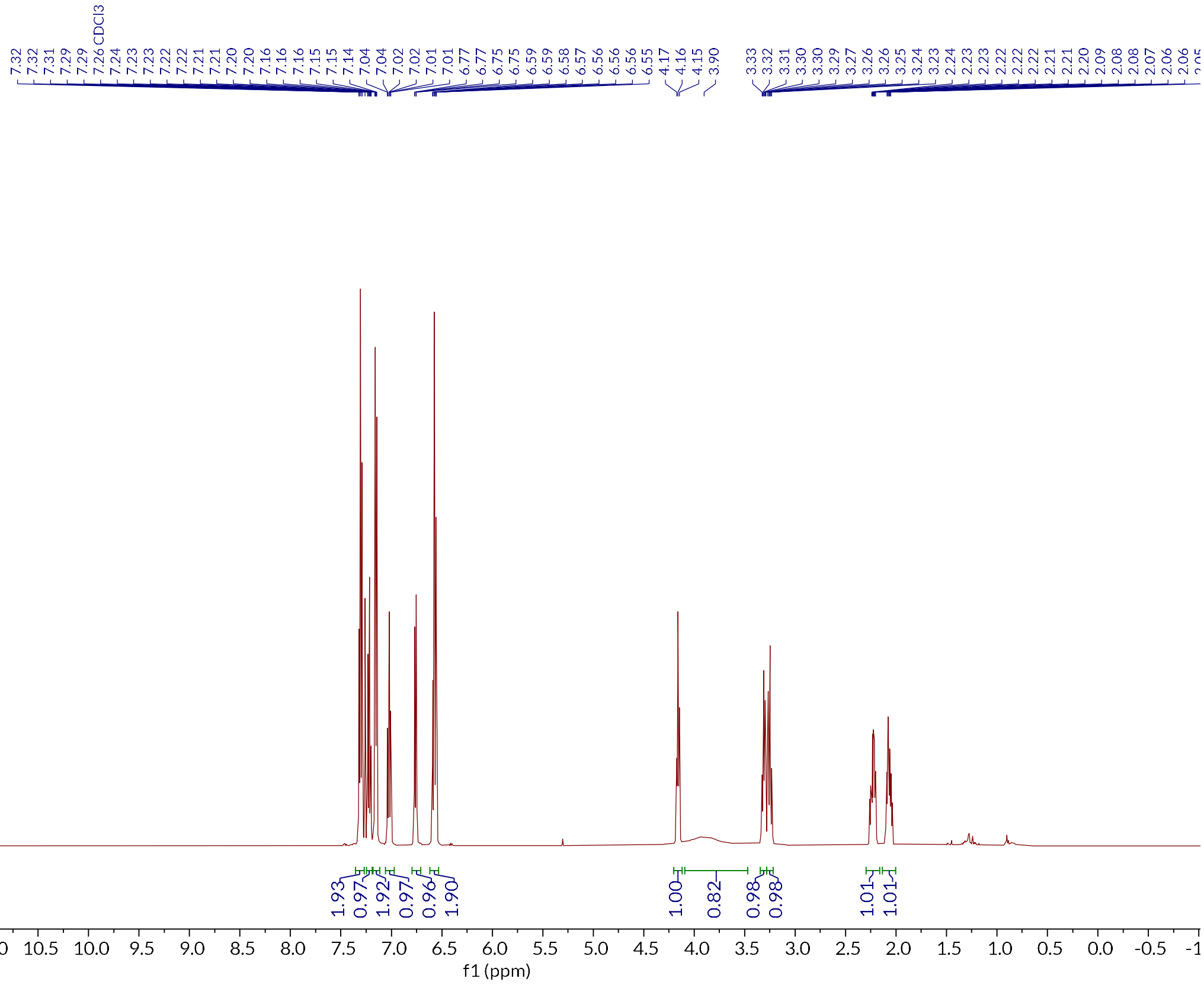

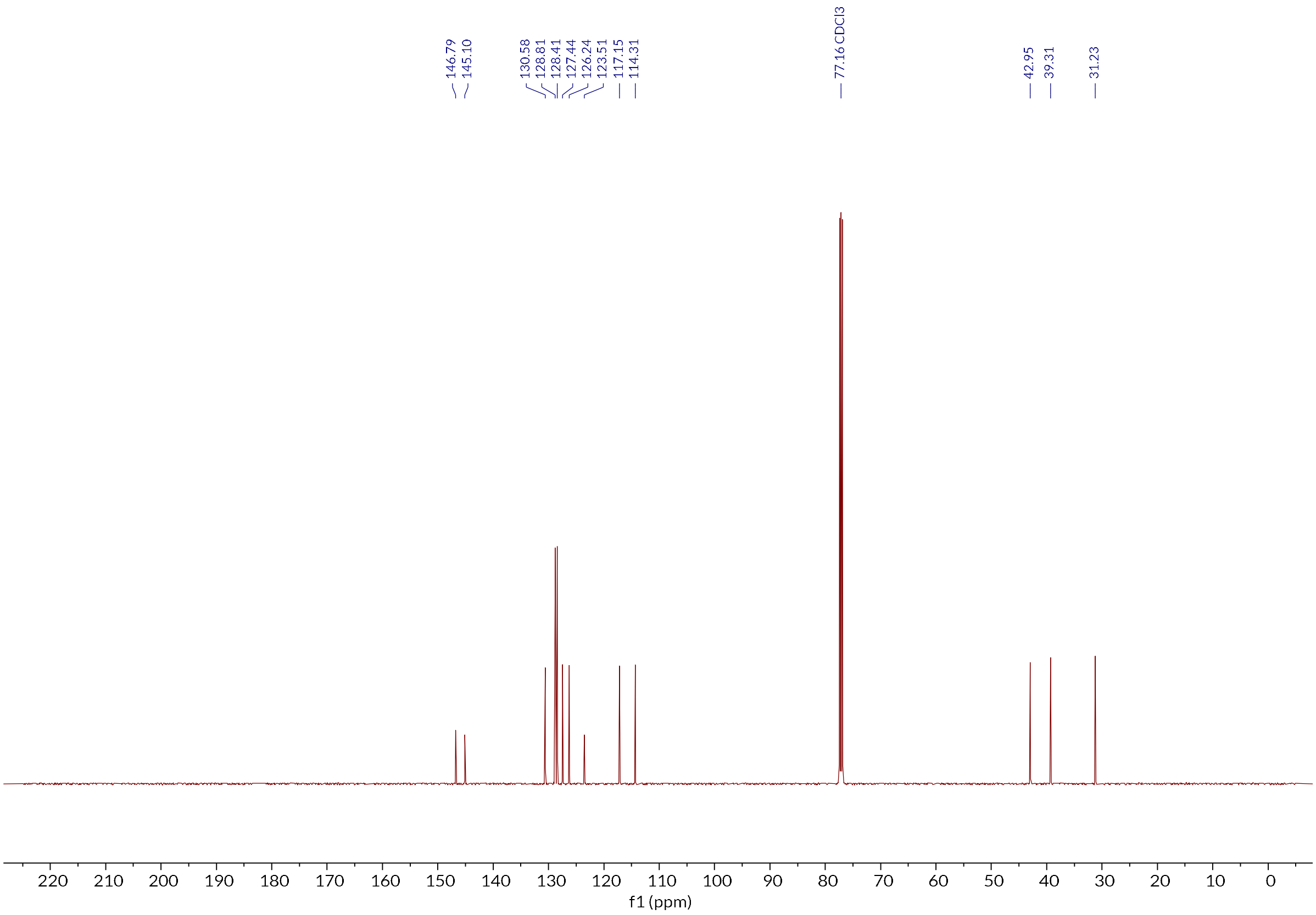

(±)-8-Methyl-4-phenyl-1,2,3,4-tetrahydroquinoline (4b)

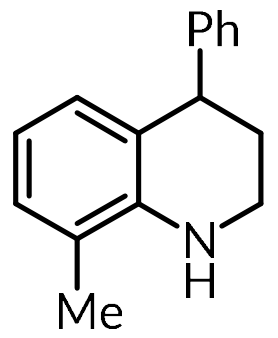

(4b)

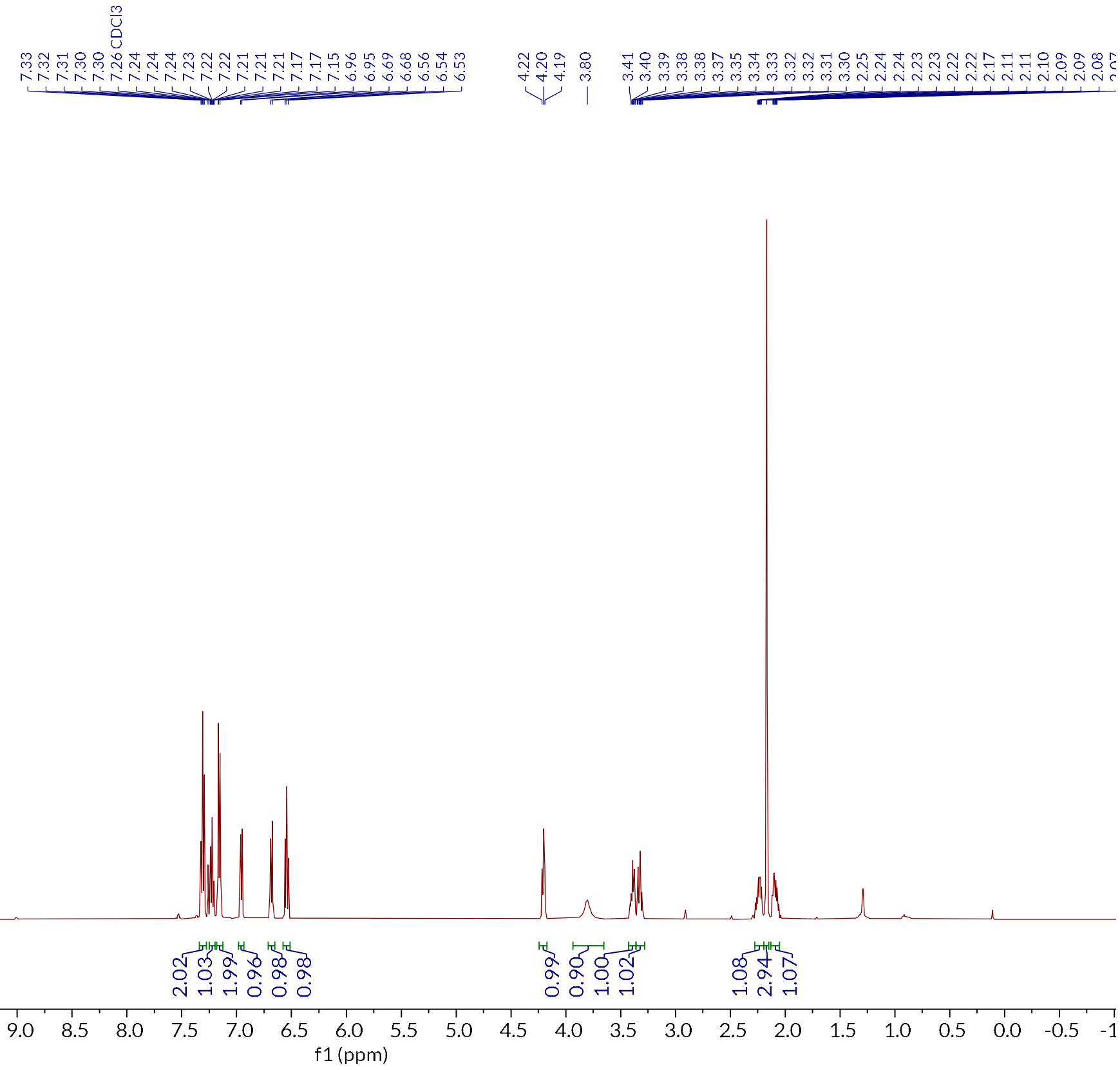

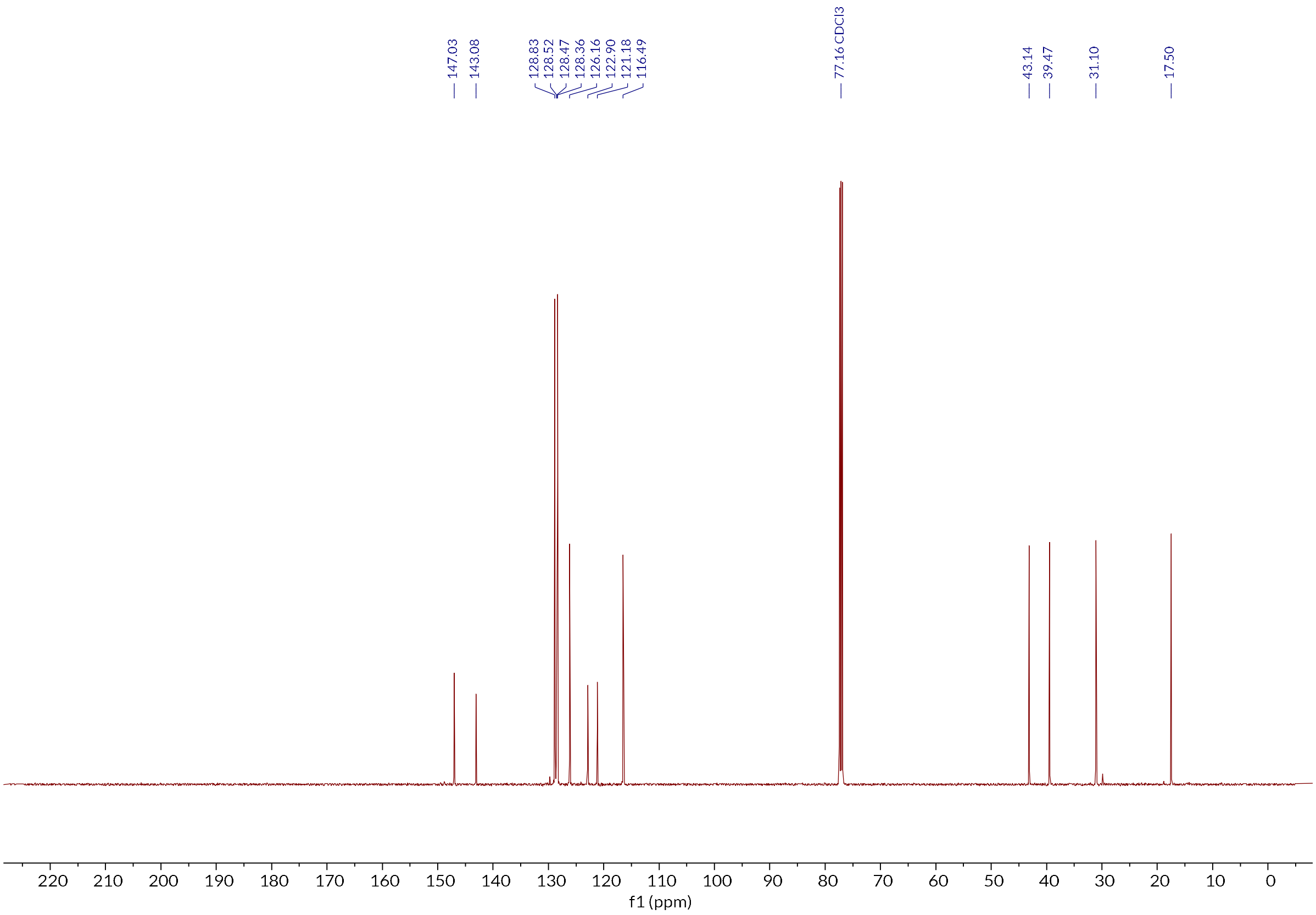

(±)-8-Bromo-4-phenyl-1,2,3,4-tetrahydroquinoline (4c)

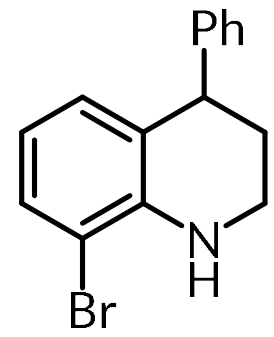

(4c)

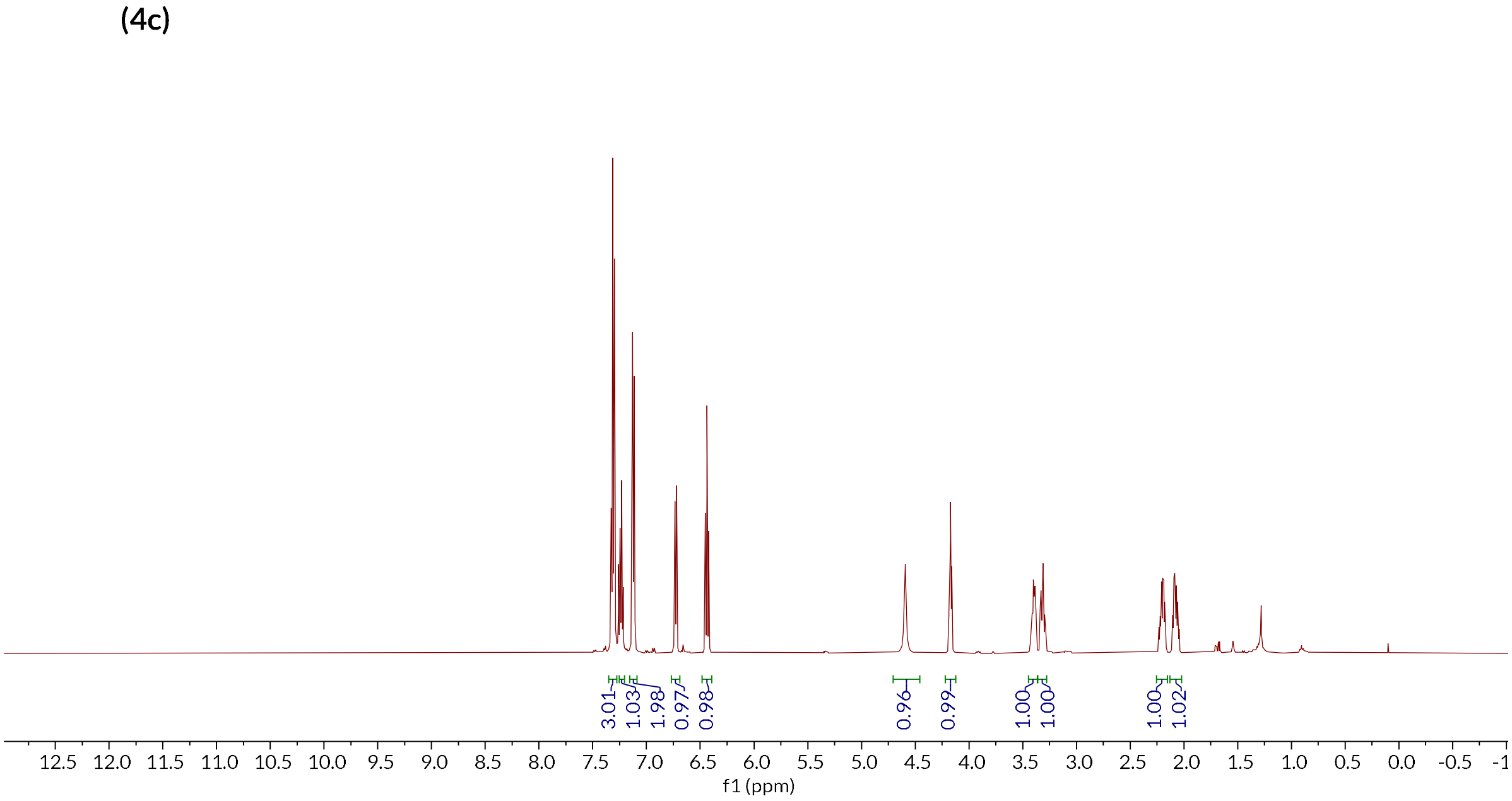

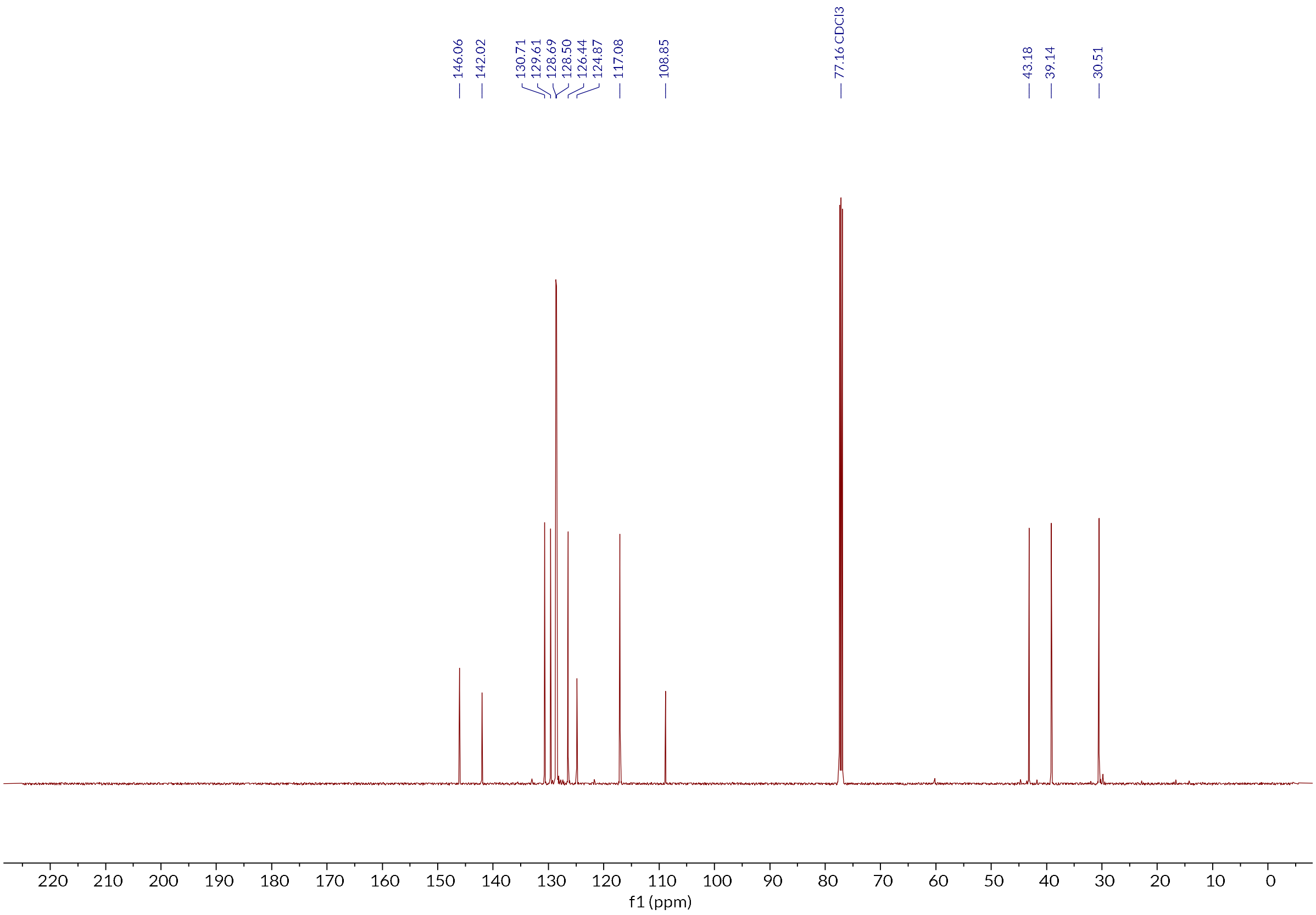

(±)-8-(Methylthio)-4-phenyl-1,2,3,4-tetrahydroquinoline (4d)

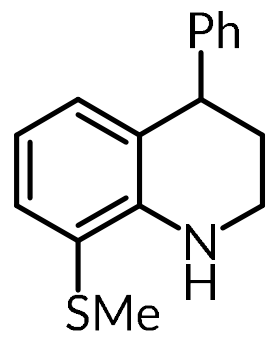

(4d)

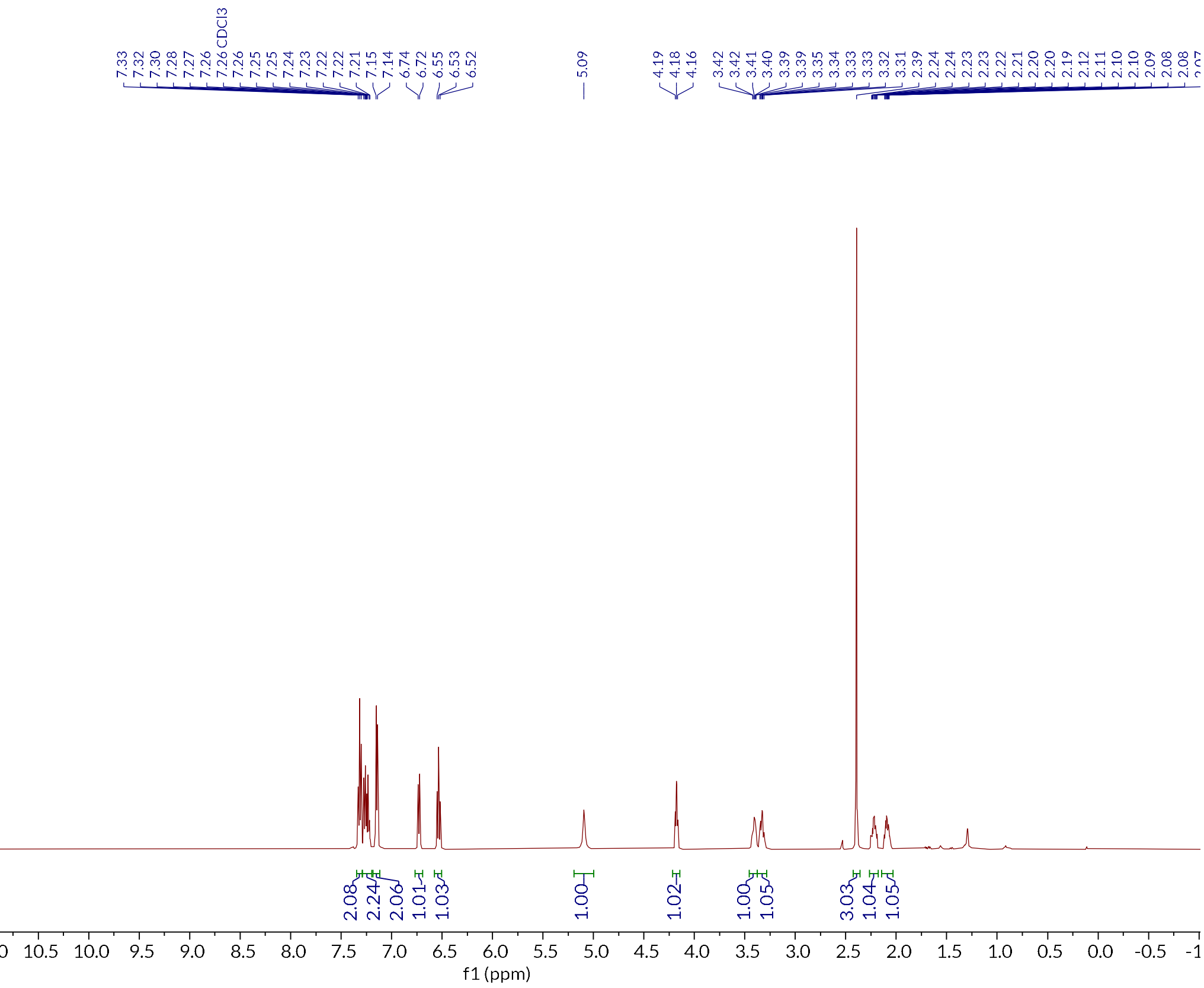

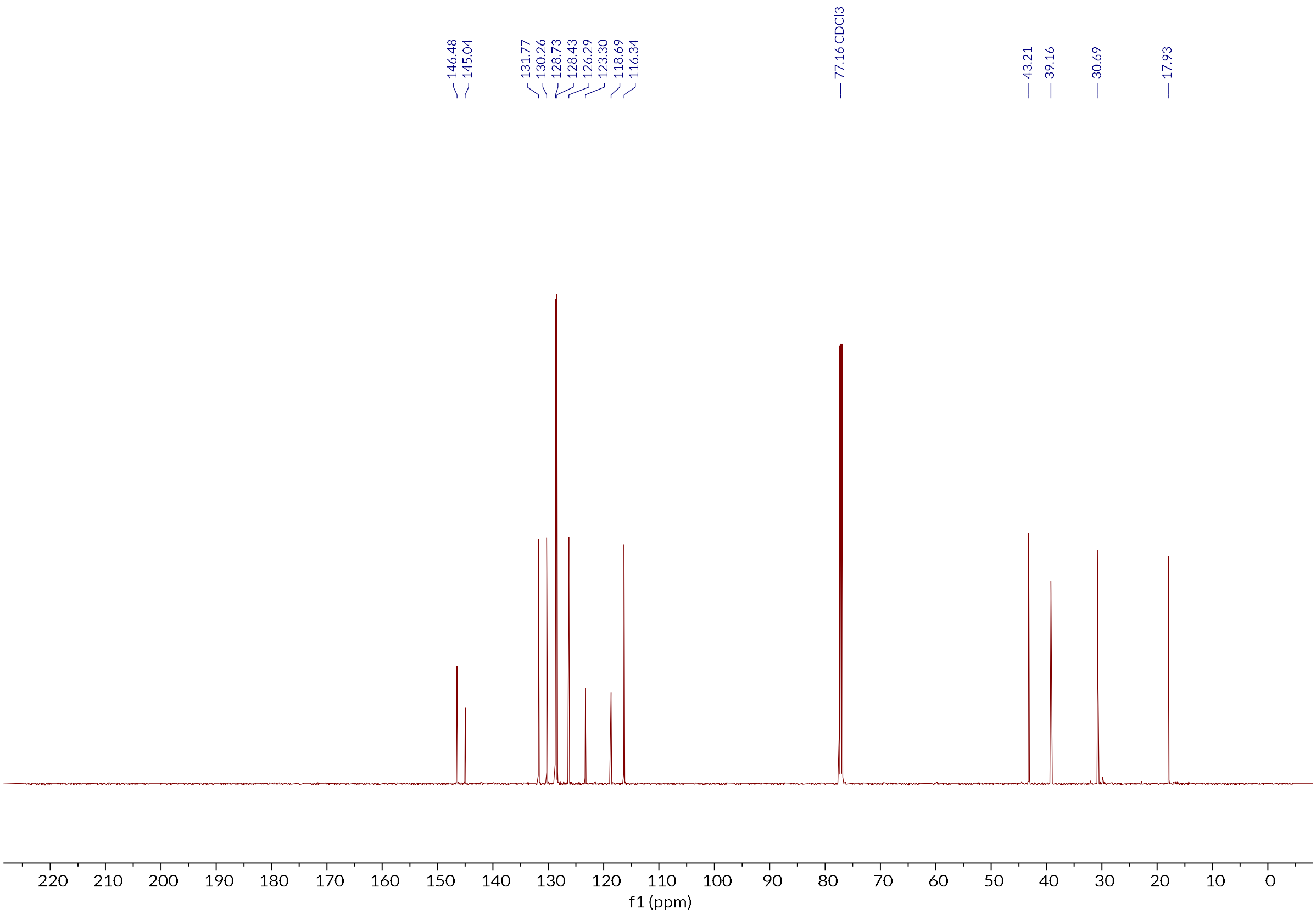

(±)-8-Methoxy-4-phenyl-1,2,3,4-tetrahydroquinoline (4e)

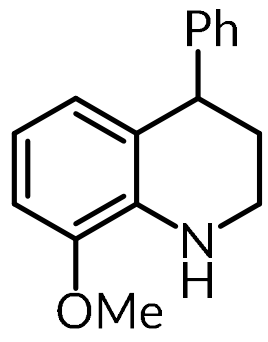

(4e)

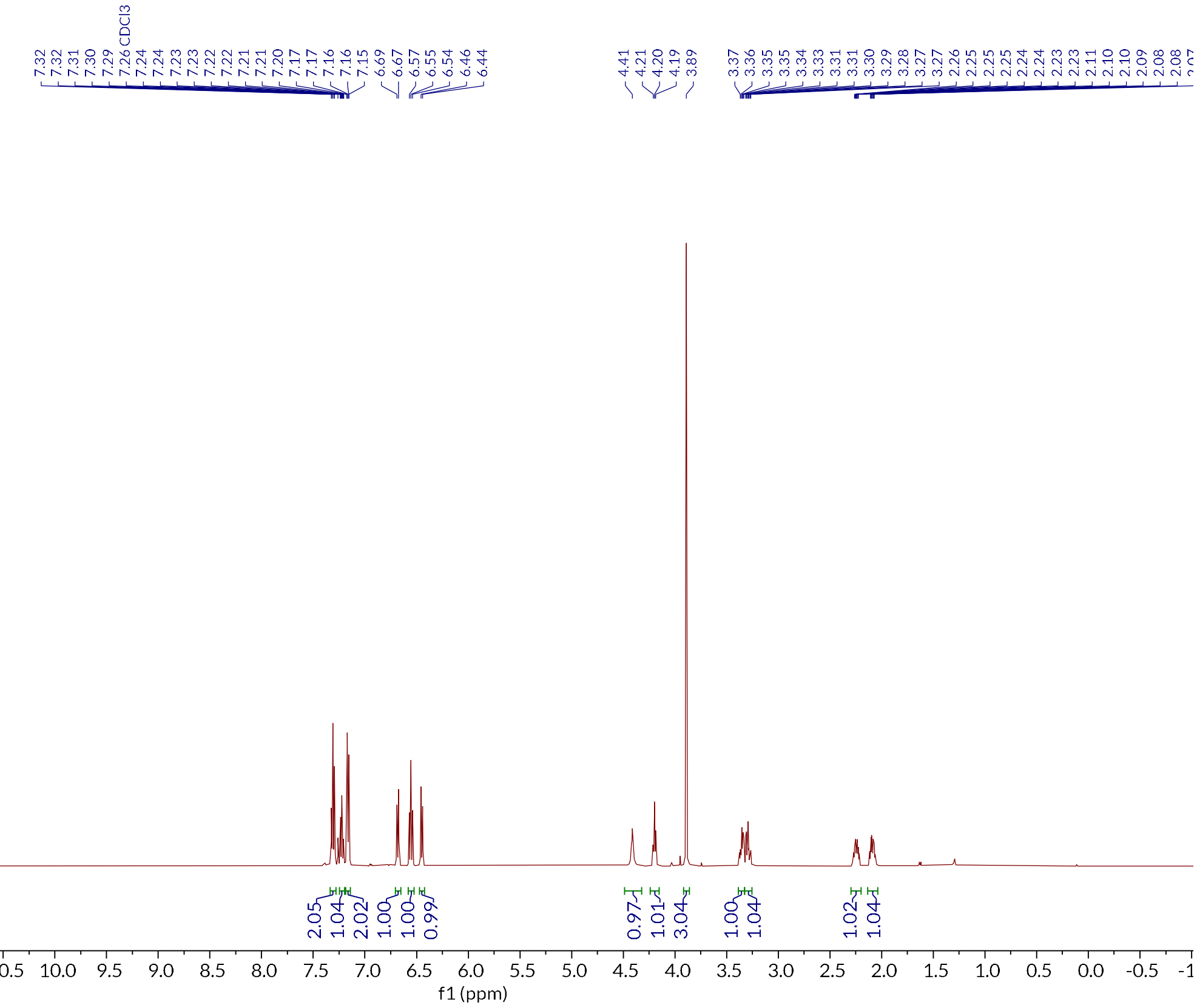

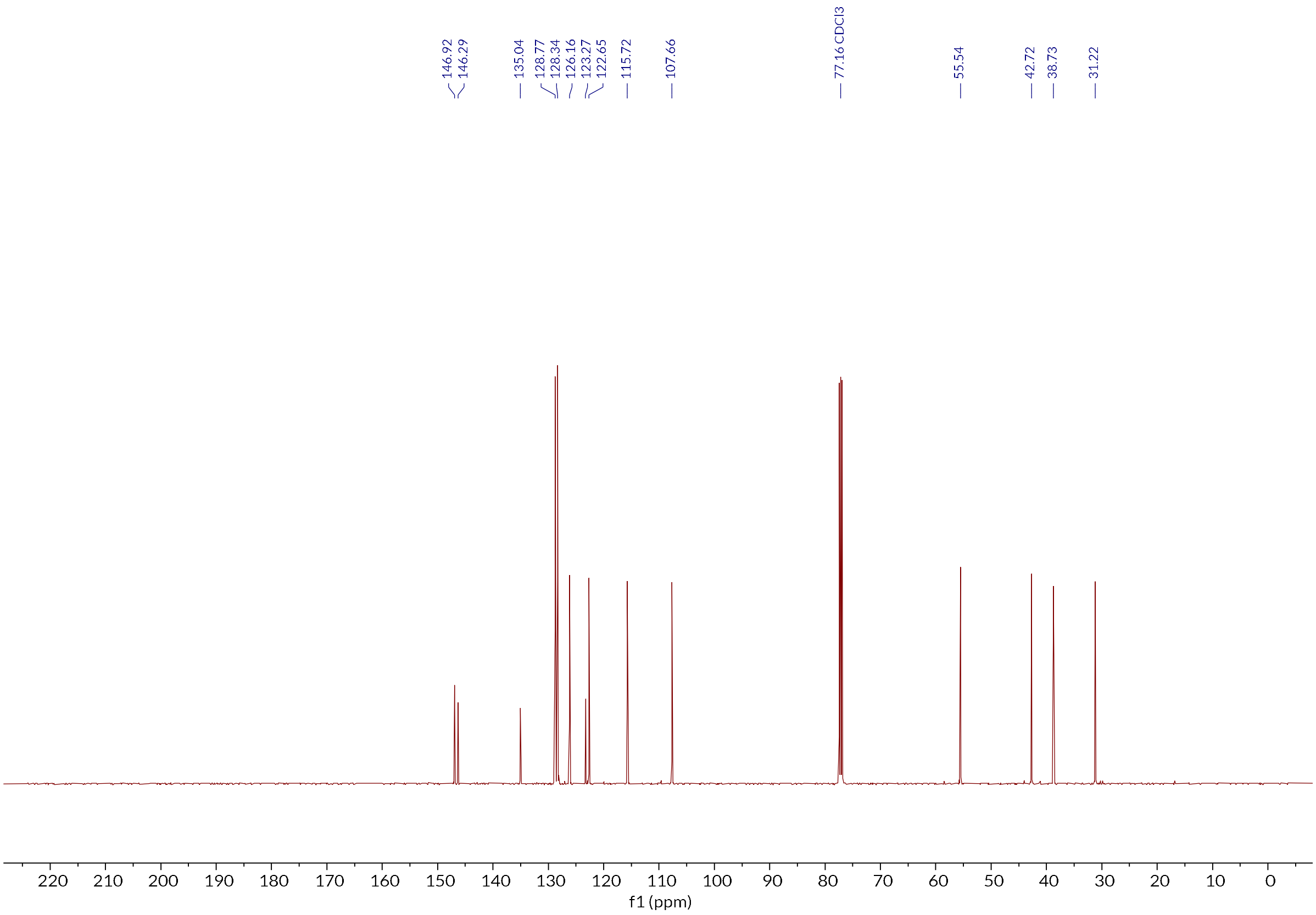

(±)-6-Methoxy-8-methyl-4-phenyl-1,2,3,4-tetrahydroquinoline (4f)

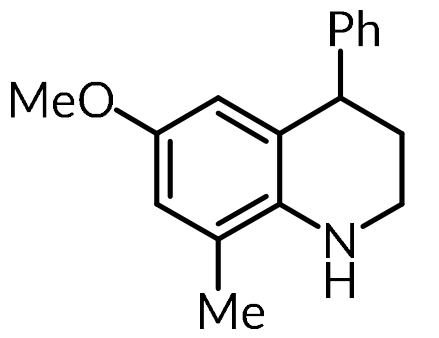

(4f)

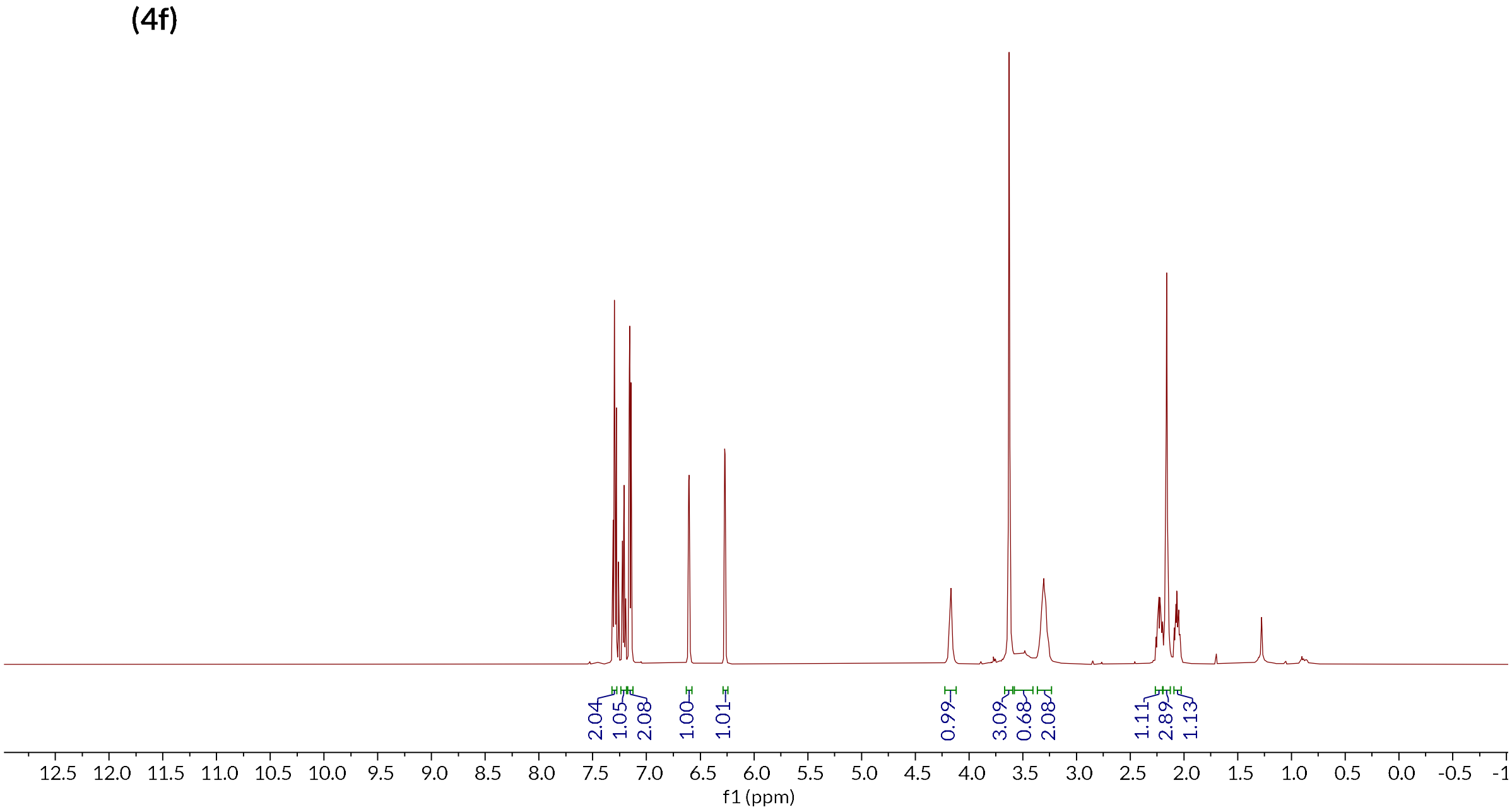

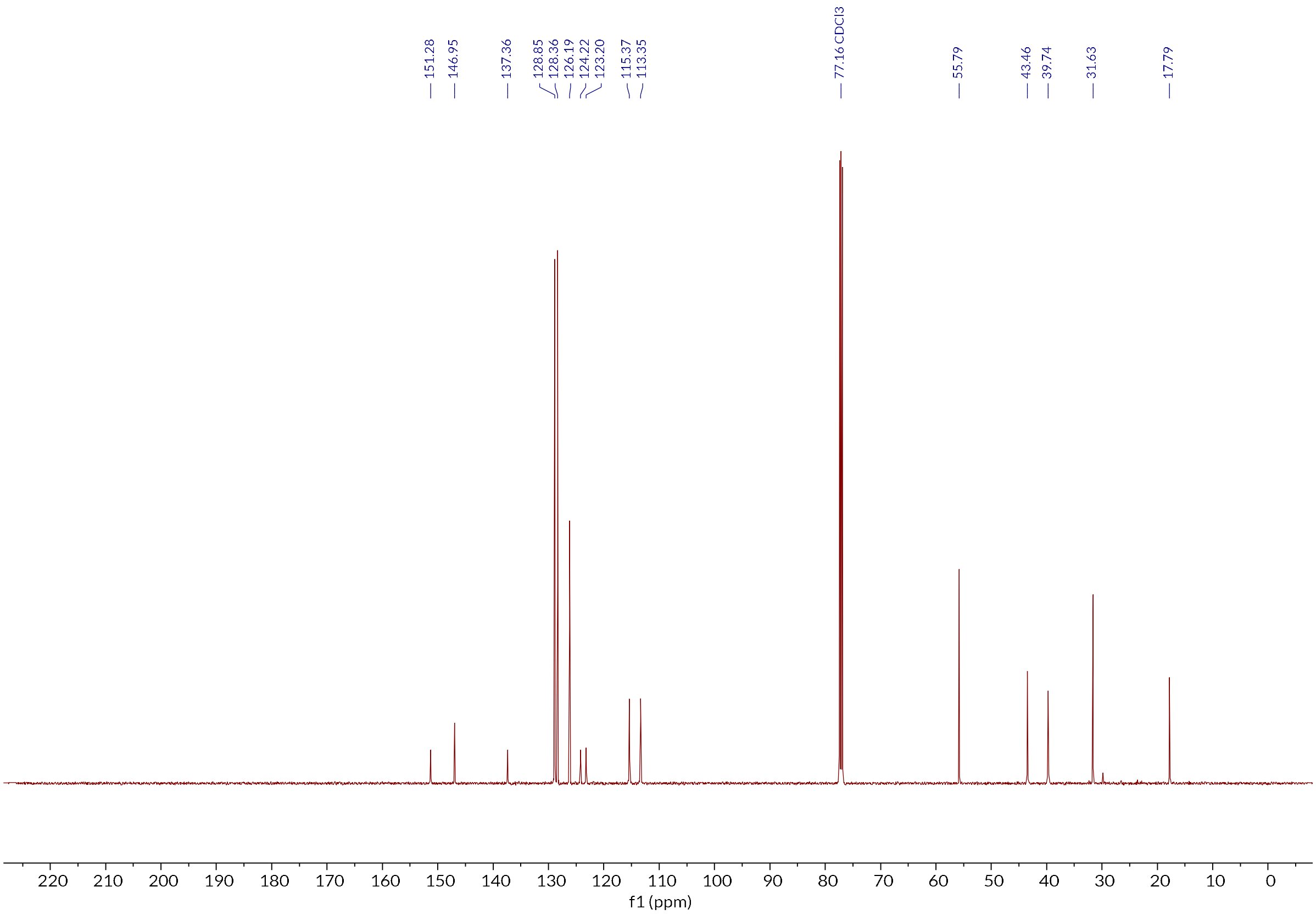

(±)-5,7-Dimethoxy-4-phenyl-1,2,3,4-tetrahydroquinoline (4g)

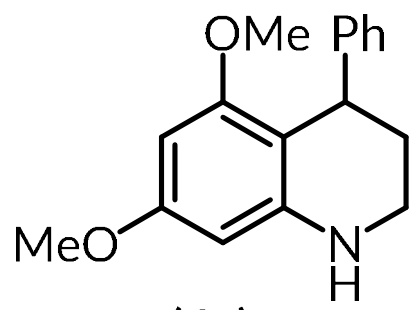

(4g)

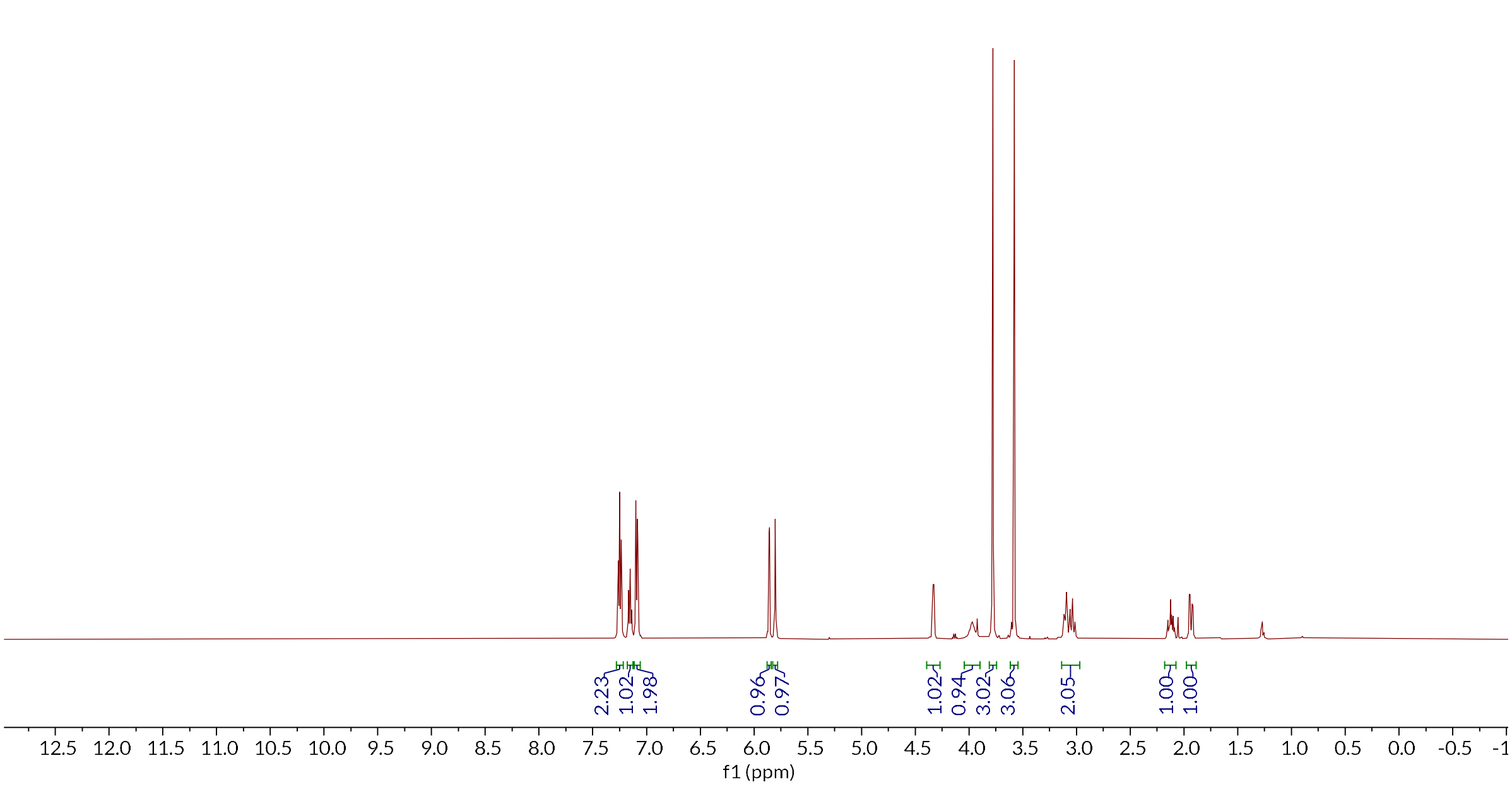

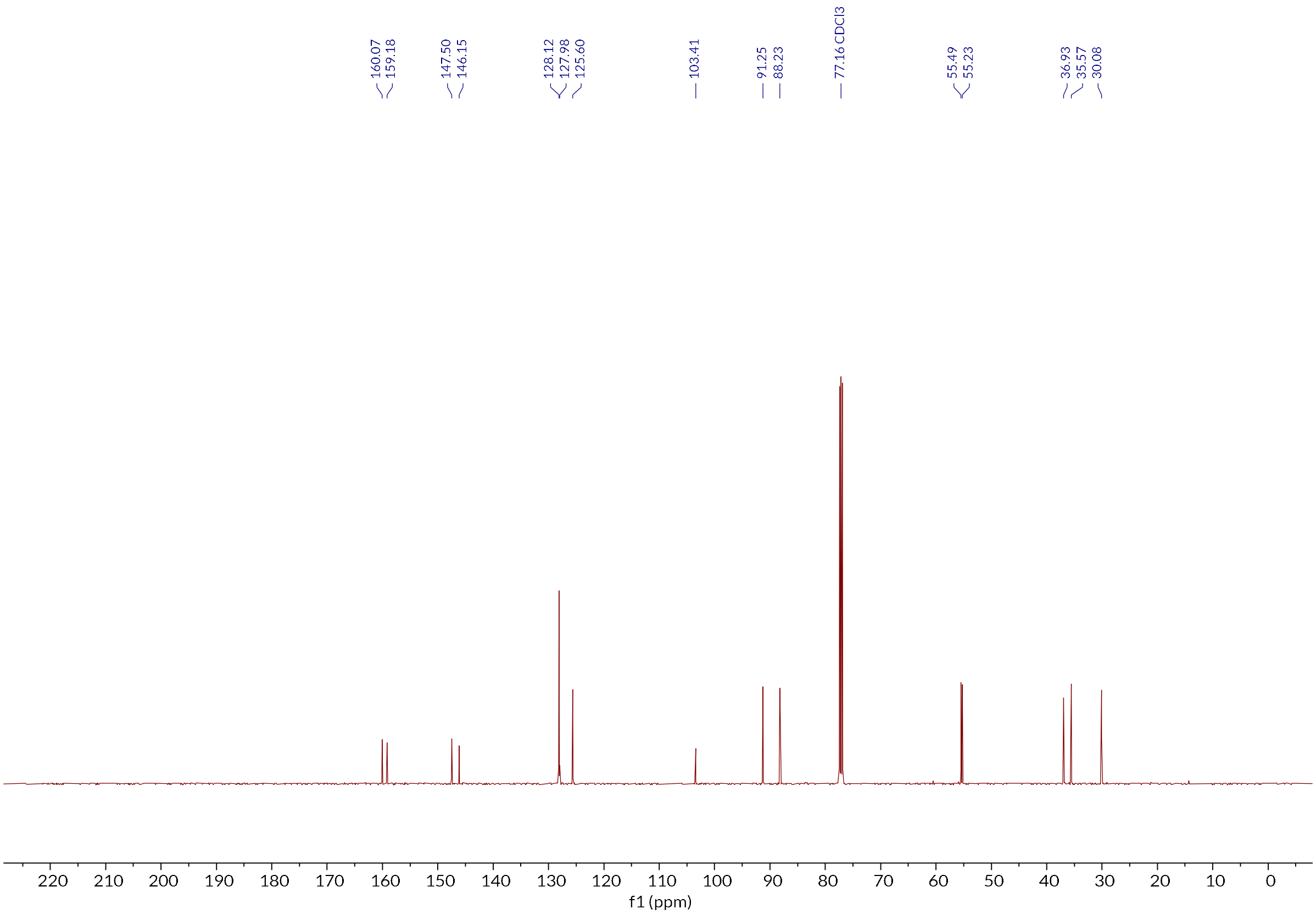

(±)-4-(Furan-2-yl)-1,2,3,4-tetrahydroquinoline (4h)

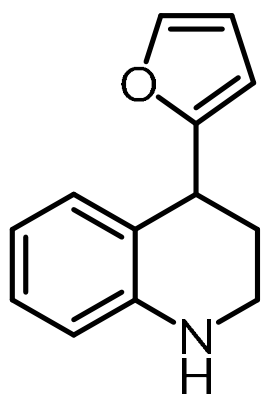

(4h)

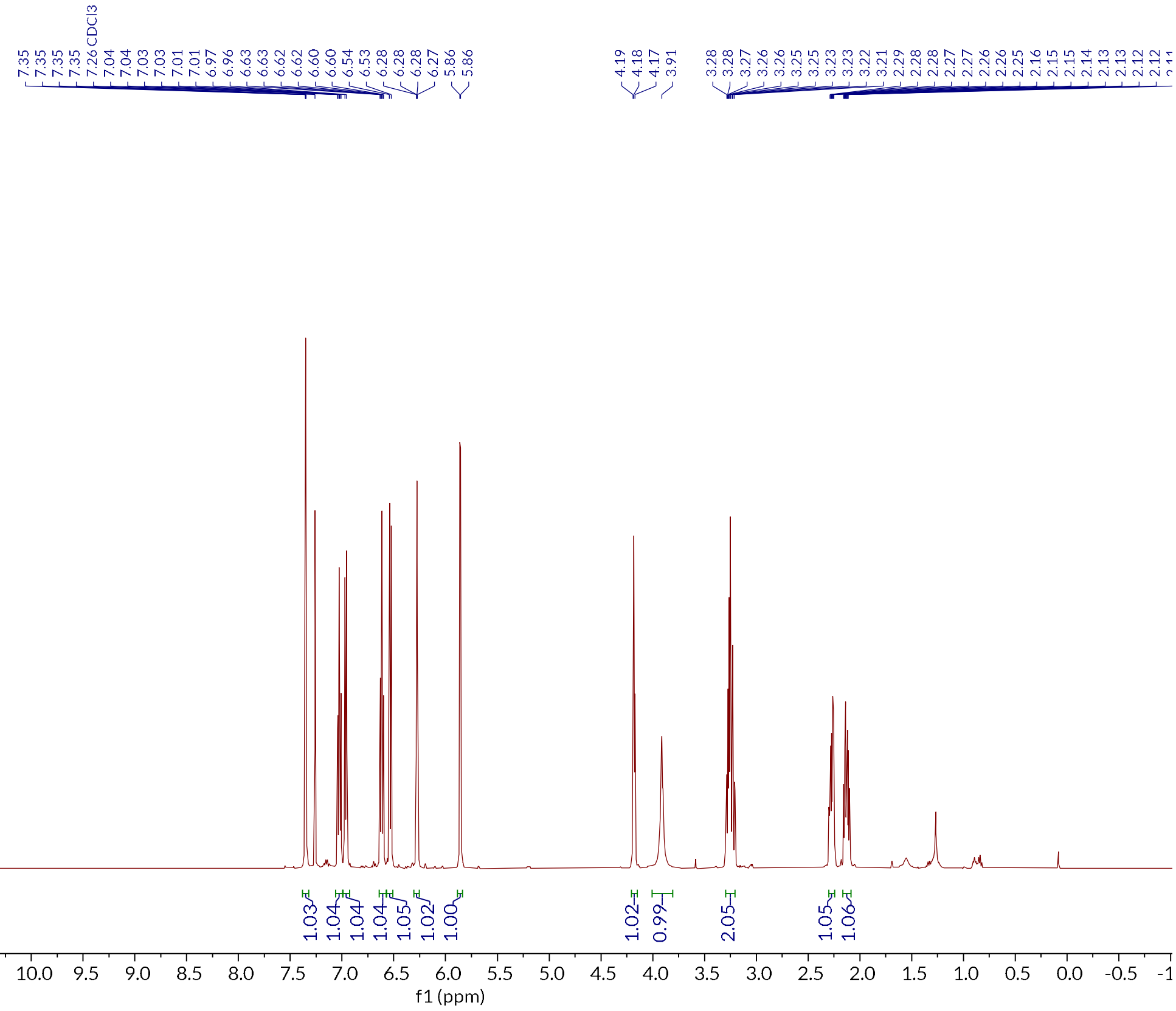

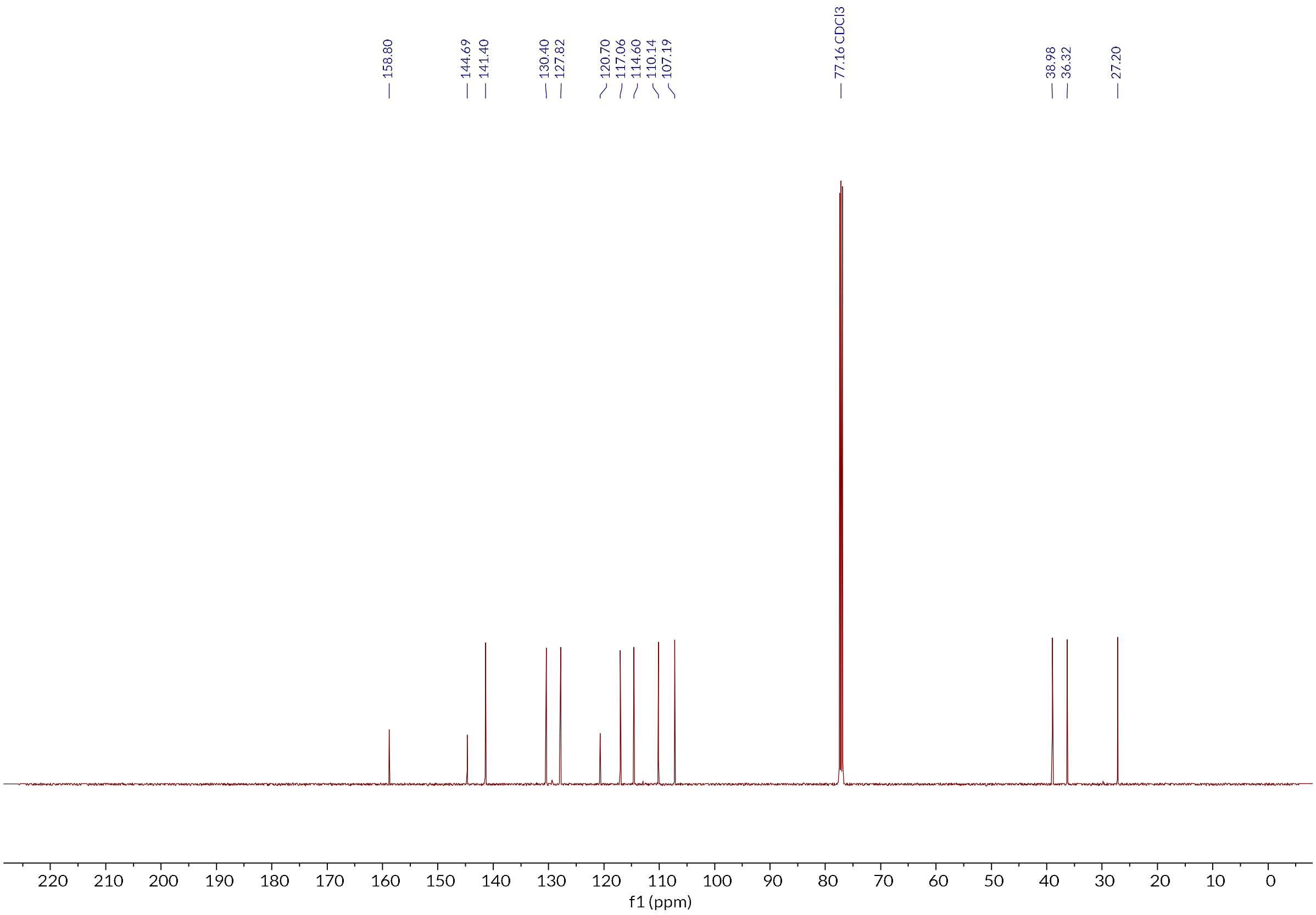

(±)-Ethyl-(*E*)-4-(4-phenyl-3,4-dihydroquinolin-1(2*H*)-yl)but-2-enoate (6a)

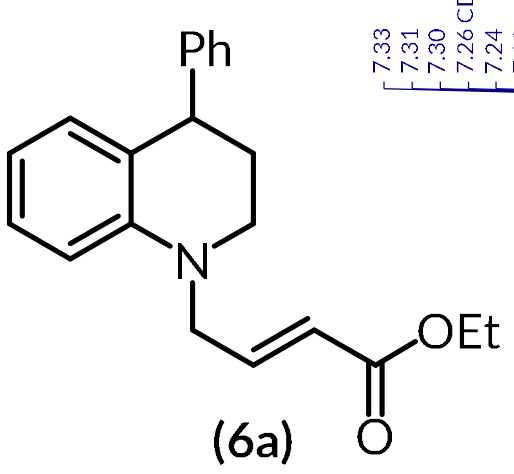

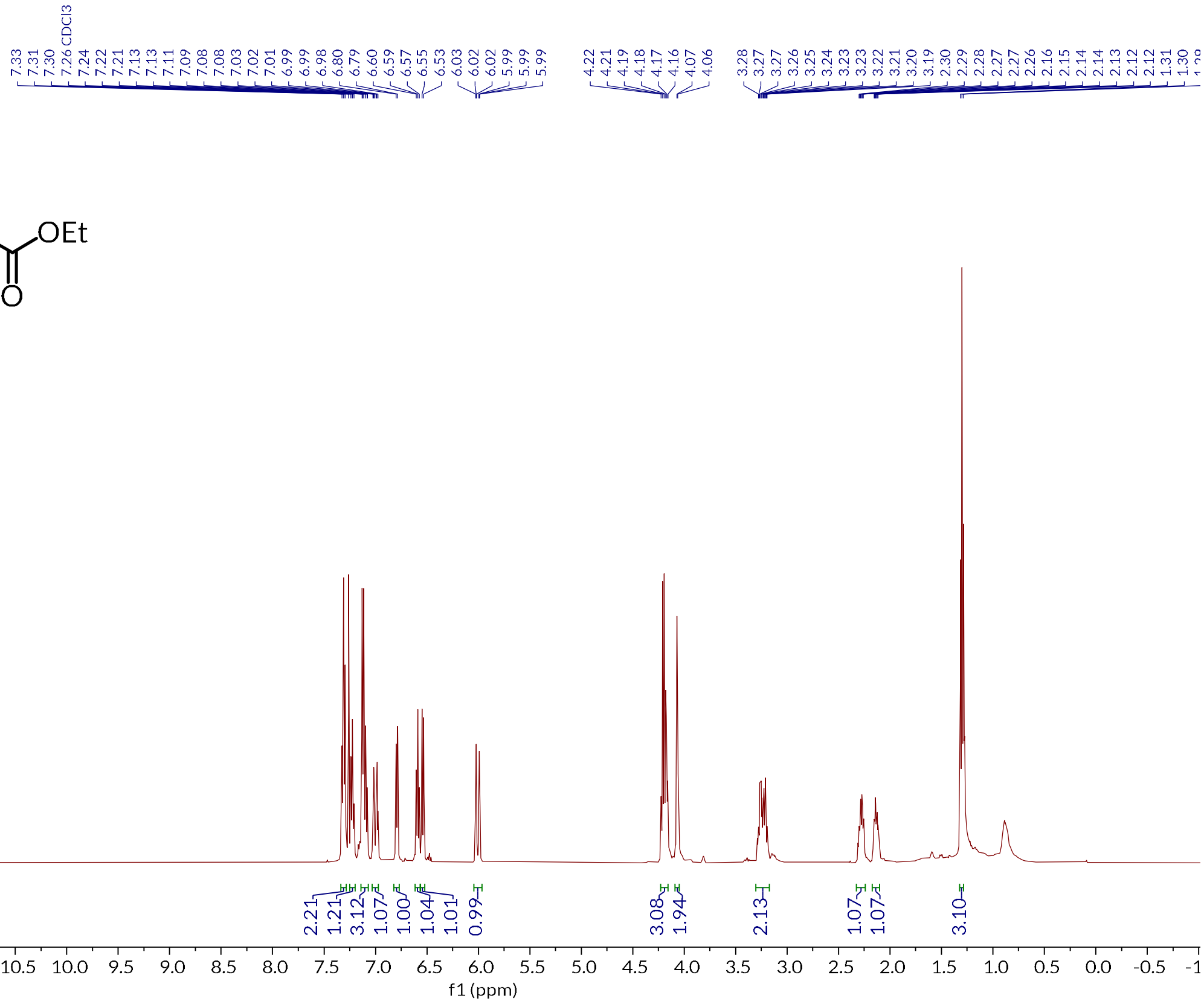

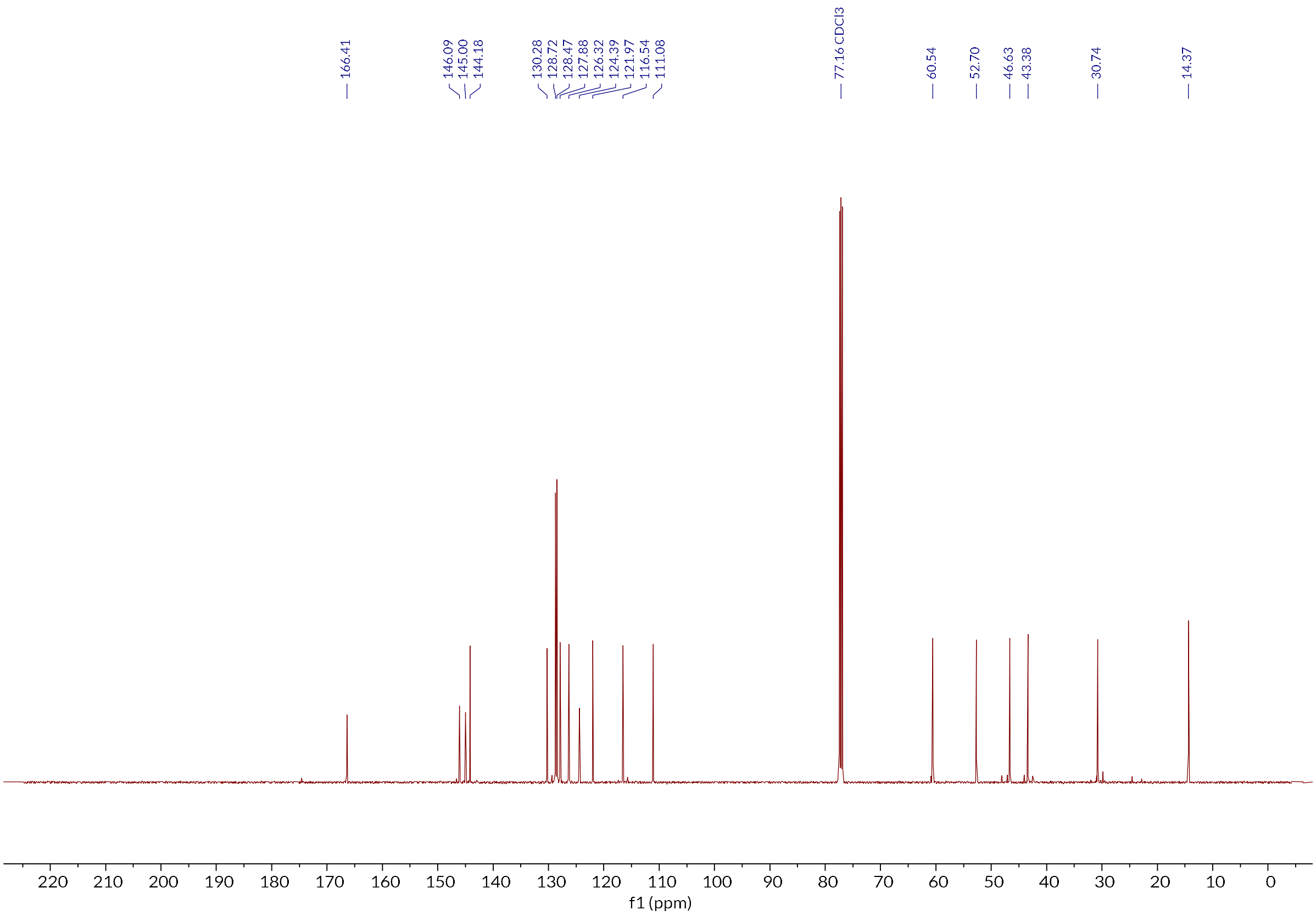

Syn-**Ethyl 2-(1,4-diphenylpiperidin-3-yl)acetate (6b)**

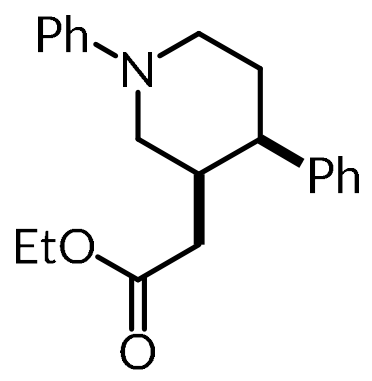

(*syn*-6b)

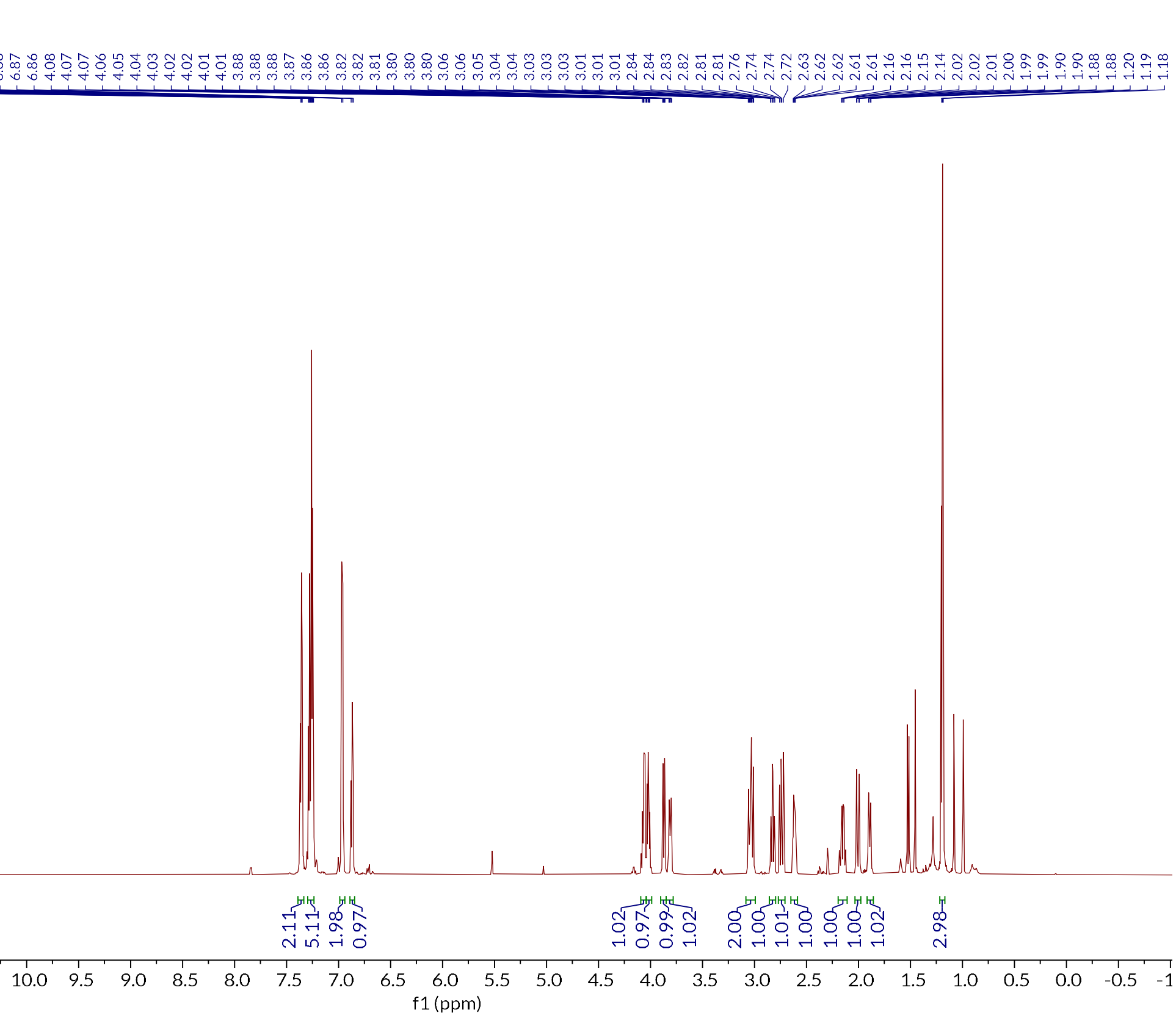

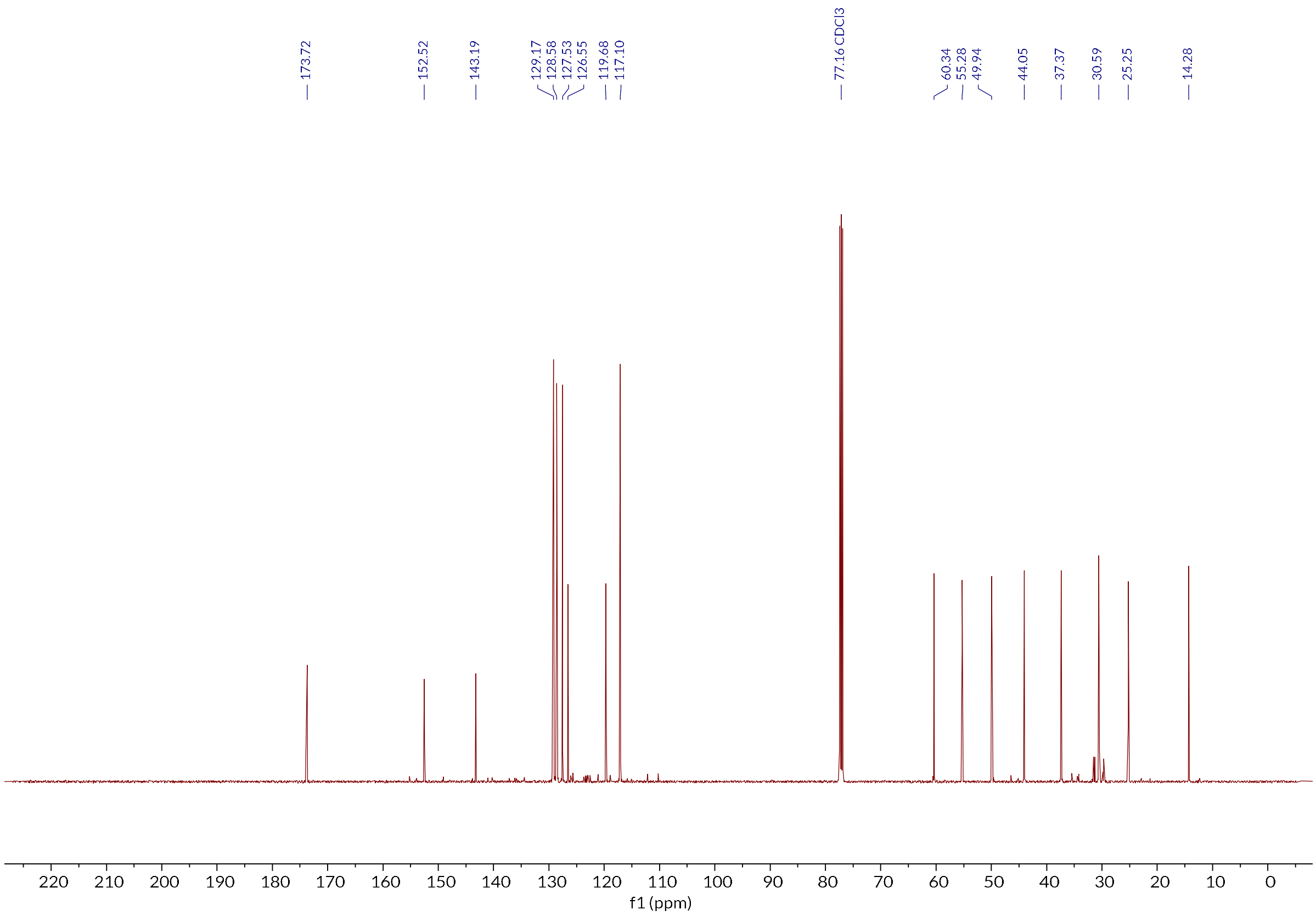

Anti-**Ethyl 2-(1,4-diphenylpiperidin-3-yl)acetate (6b)**

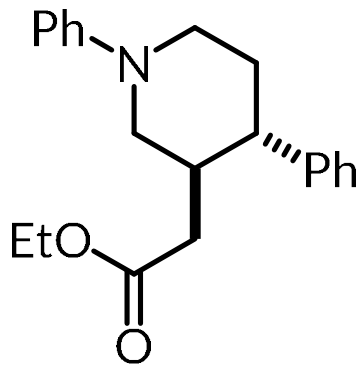

***(anti*-6b)**

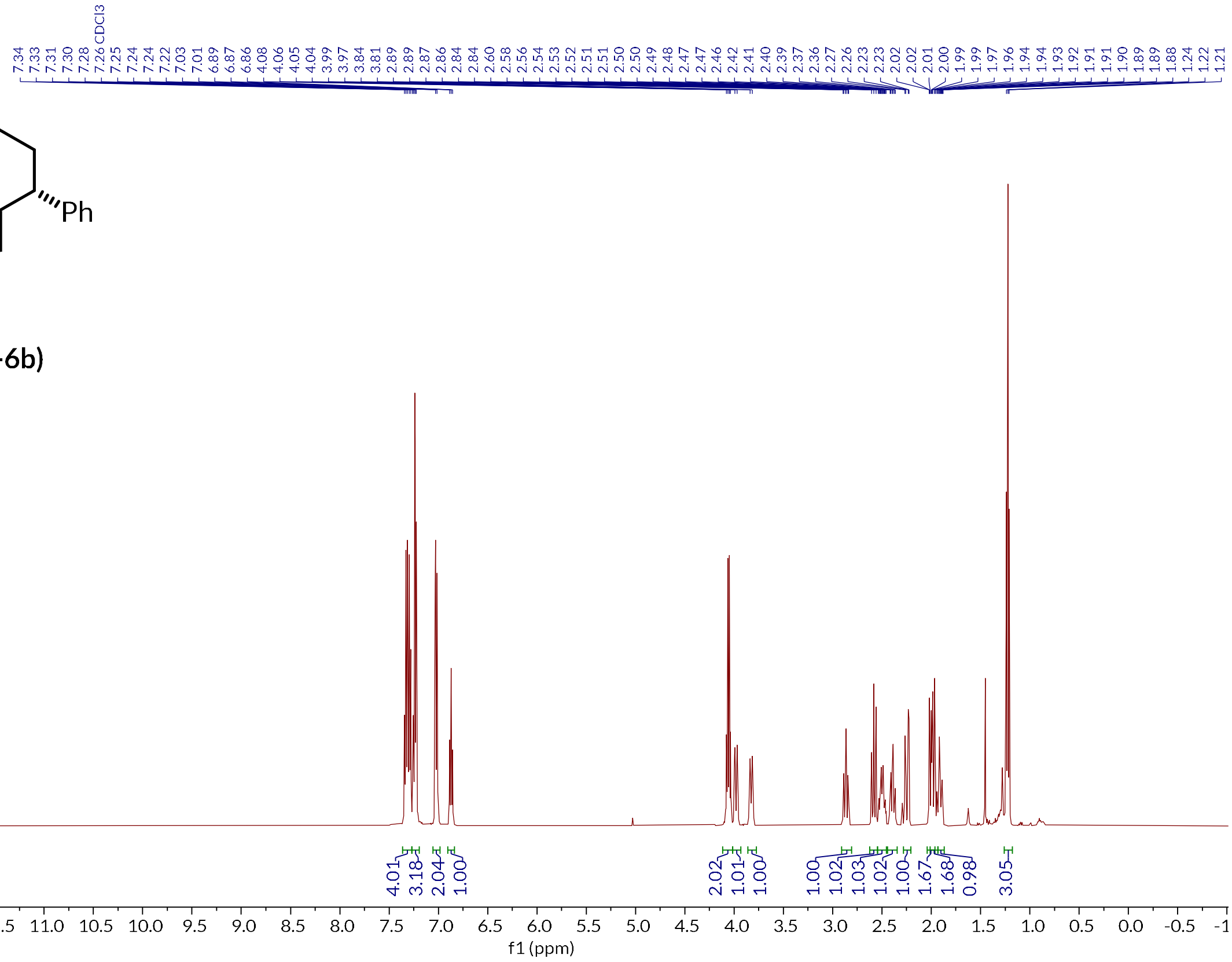

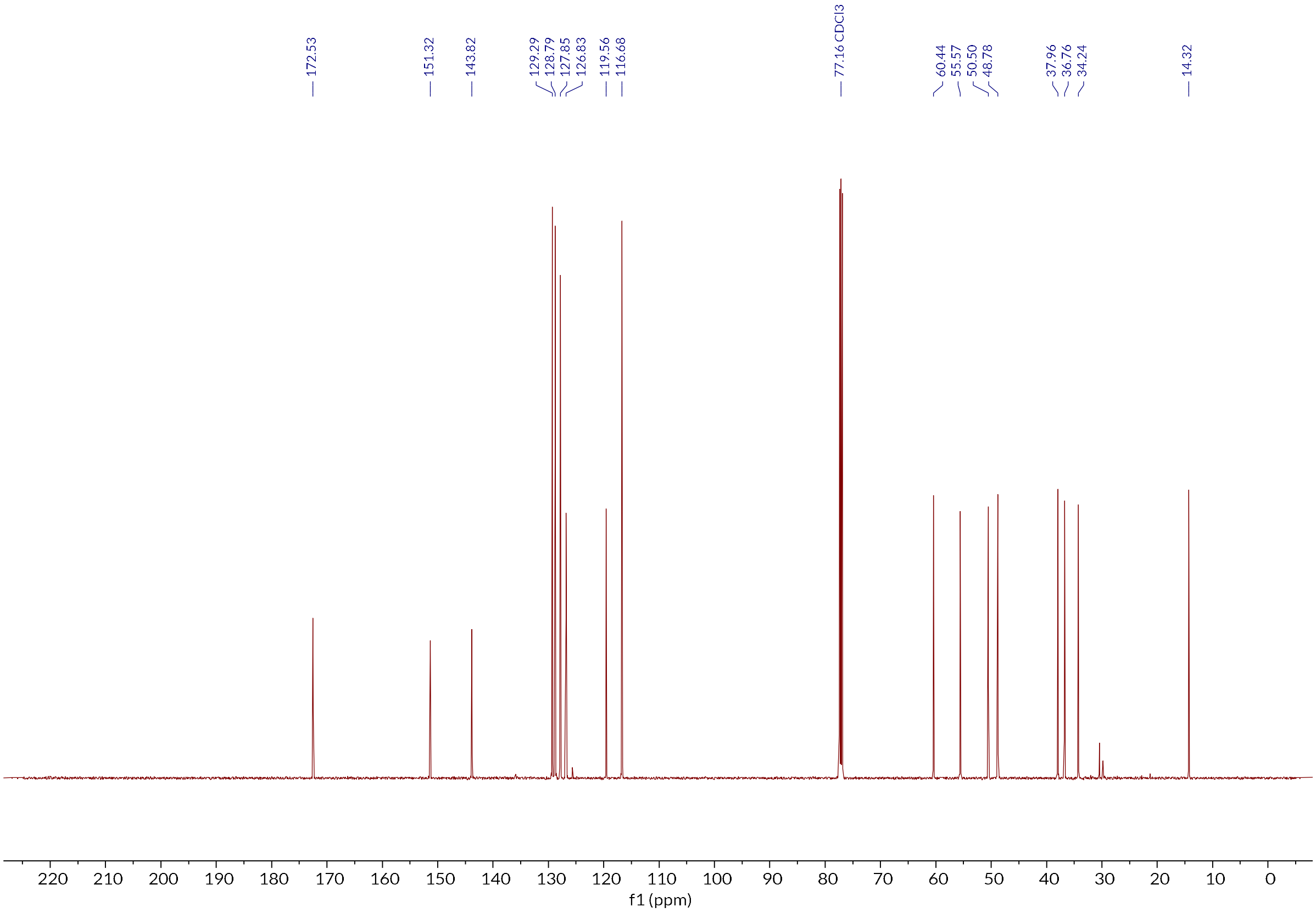

(±)-6-Methoxy-4-phenyl-1-(4-(trifluoromethyl)phenyl)-1,2,3,4-tetrahydroquinoline (8a)

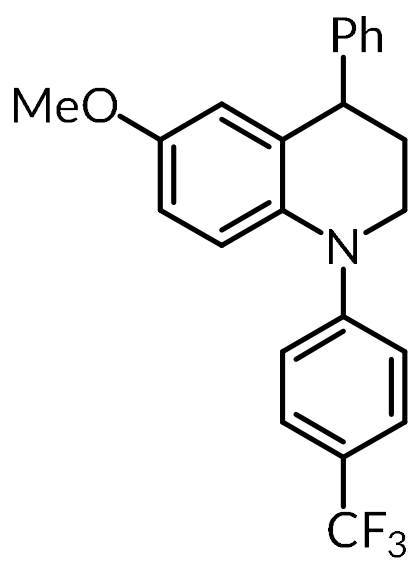

(8a)

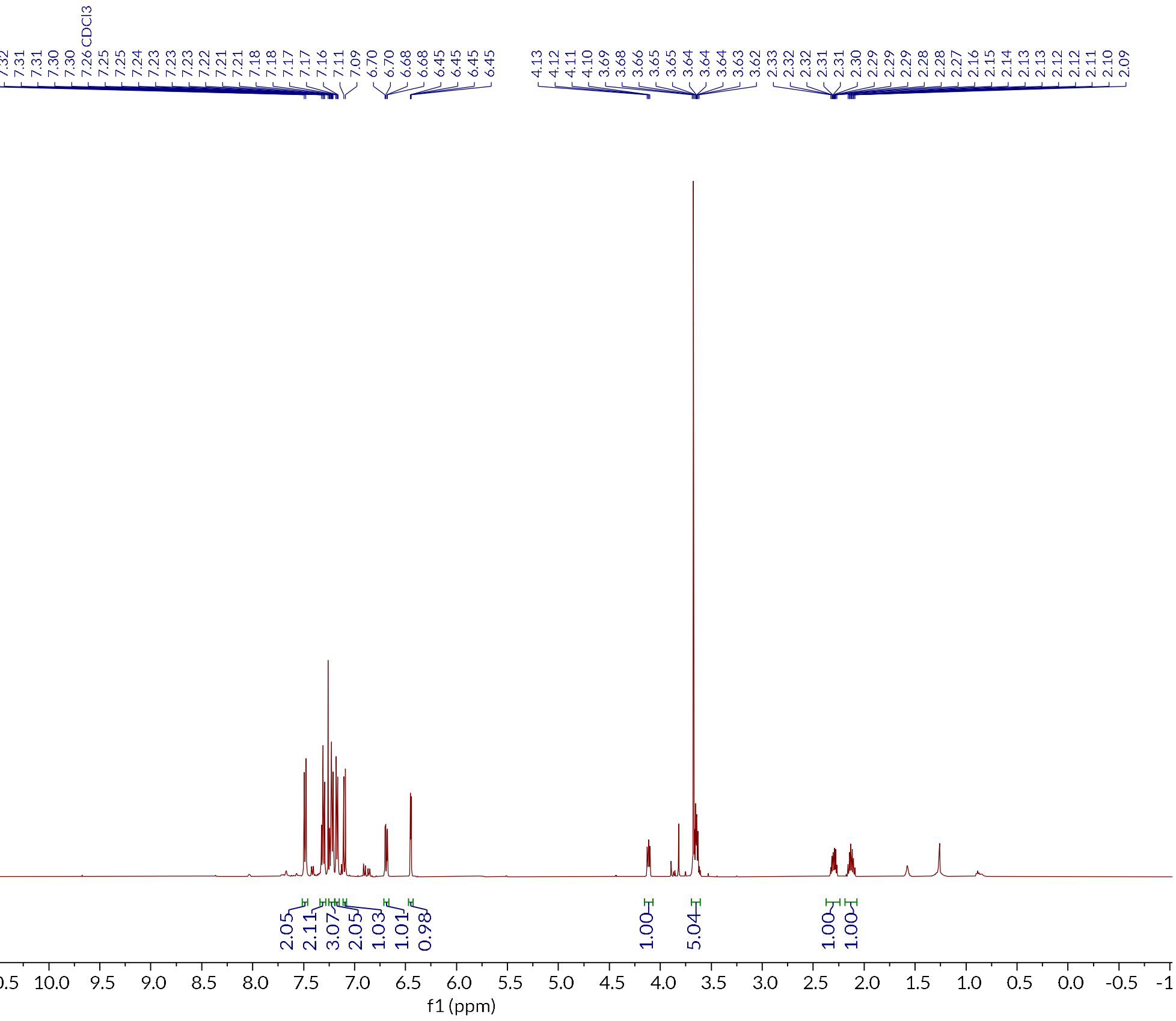

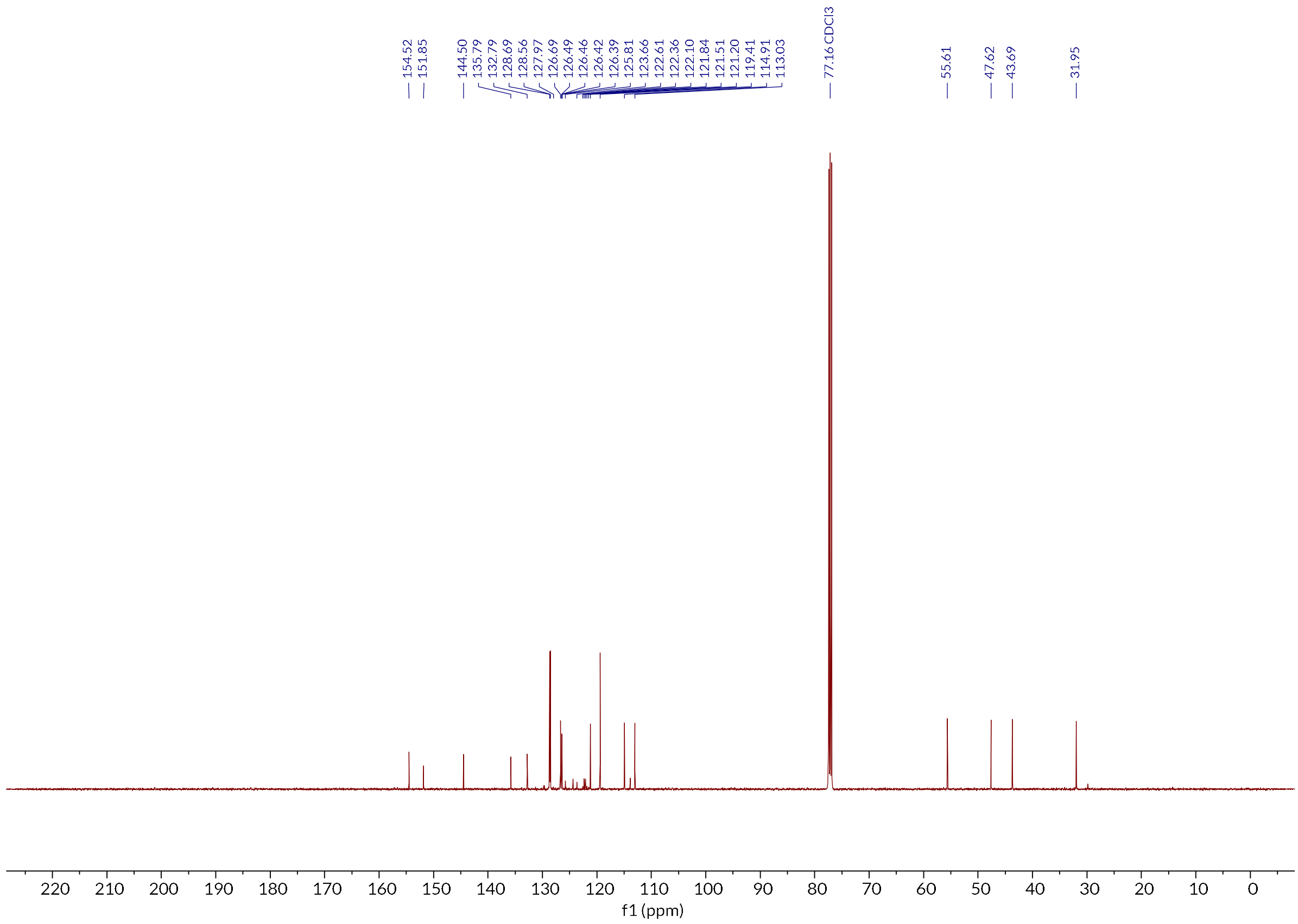

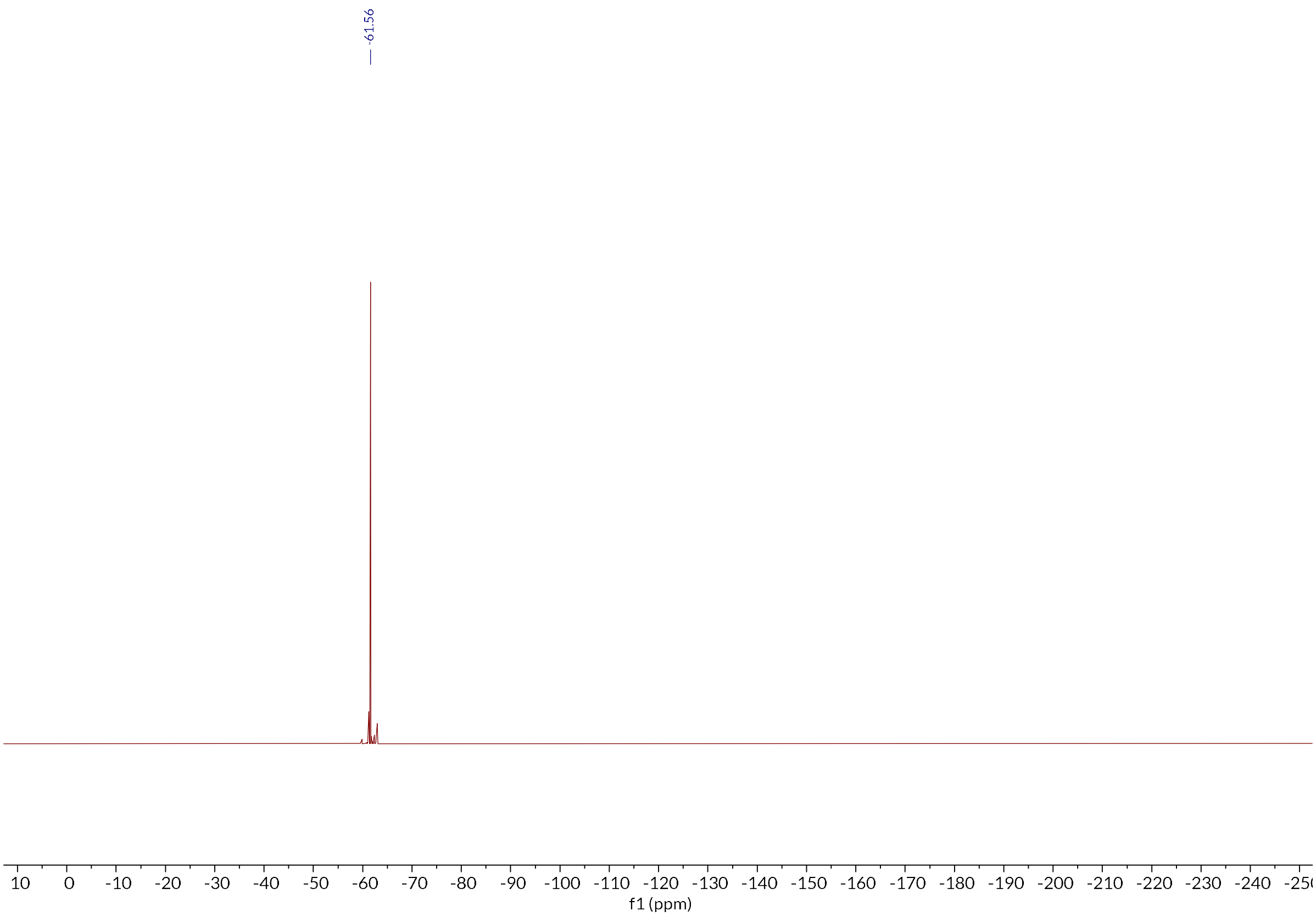
-61.56
10
0
-10
-20
-30
-40
-50
-60
-70
-80
-90
-100
-110
-120
-130
-140
-150
-160
-170
-180
-190
-200
-210
-220
-230
-240
f1 (ppm)

N-Cinnamylbenzen-d_5-amine (3a-d_5)

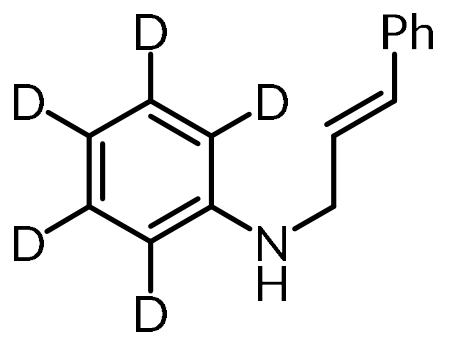

(3a-d$_5$)

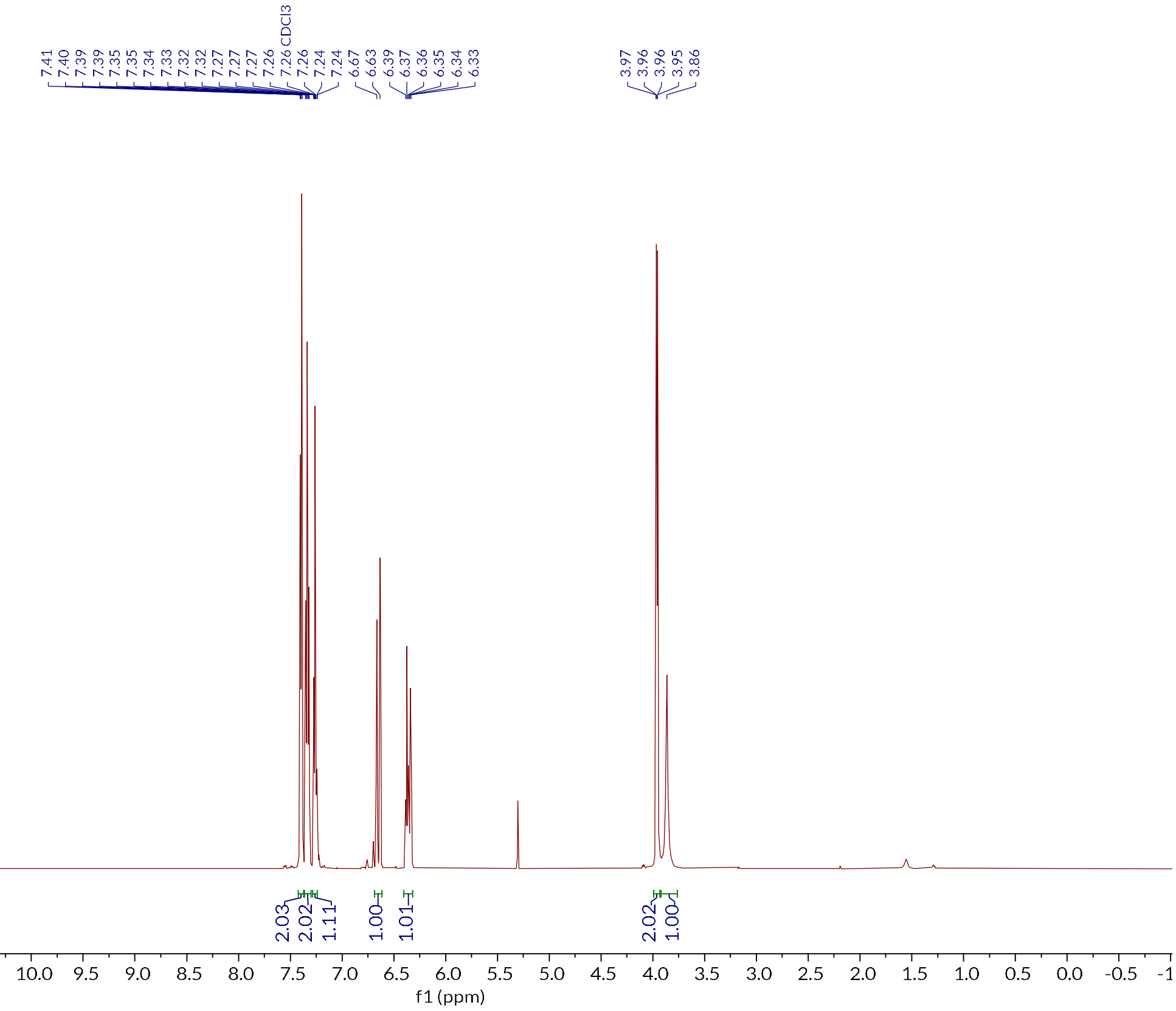

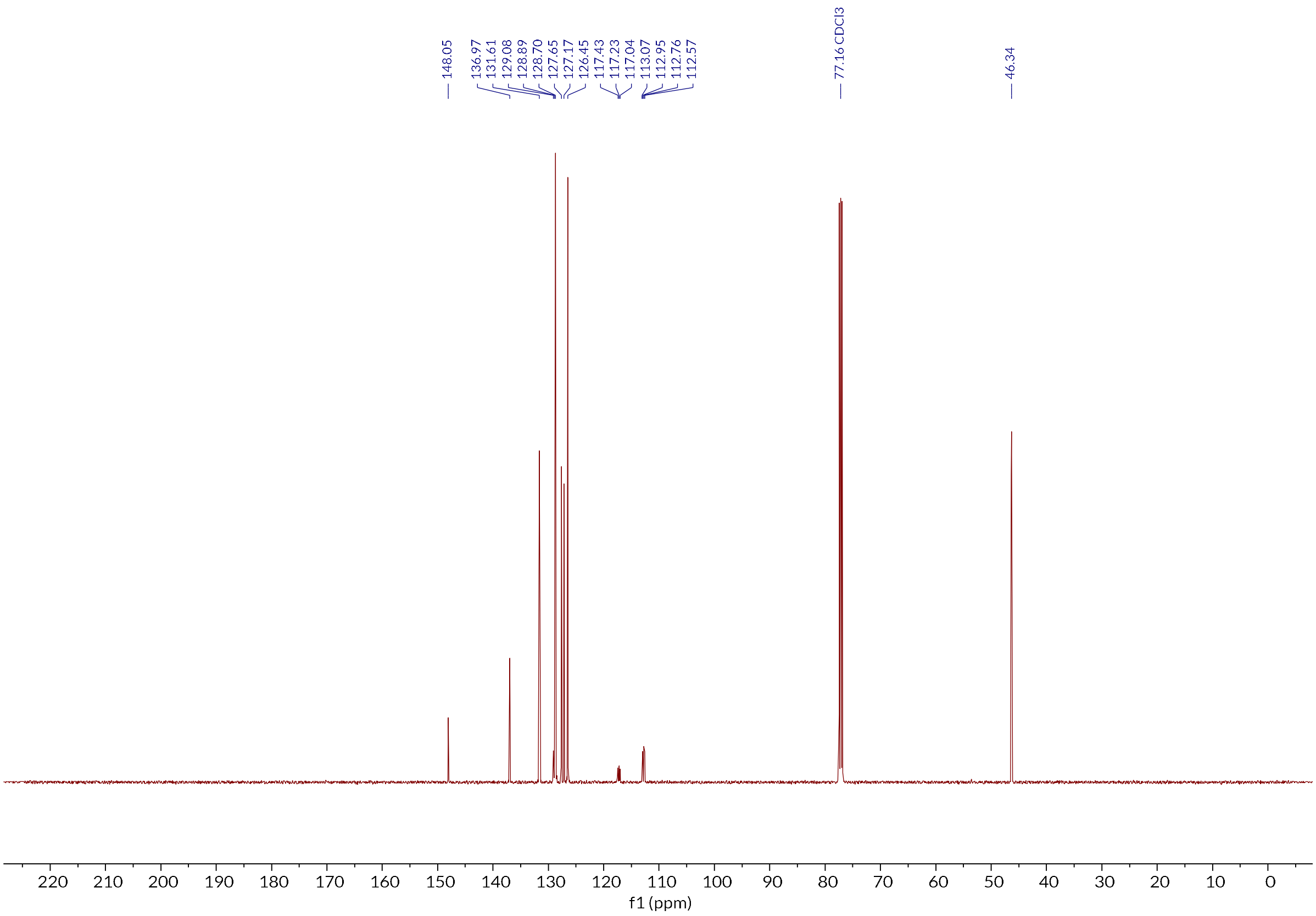

N-Benzyl-*N*-cinnamylaniline-d_5 (1aa-d_5)

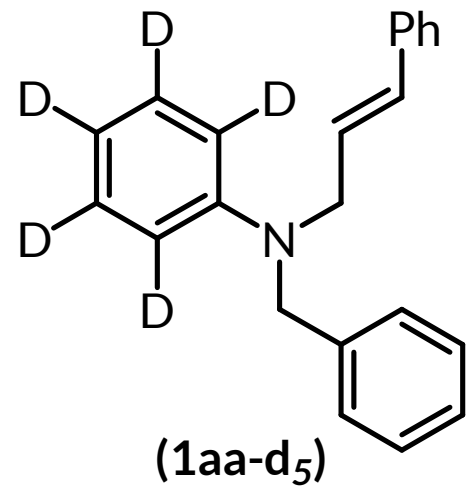

(1aa-d_5)

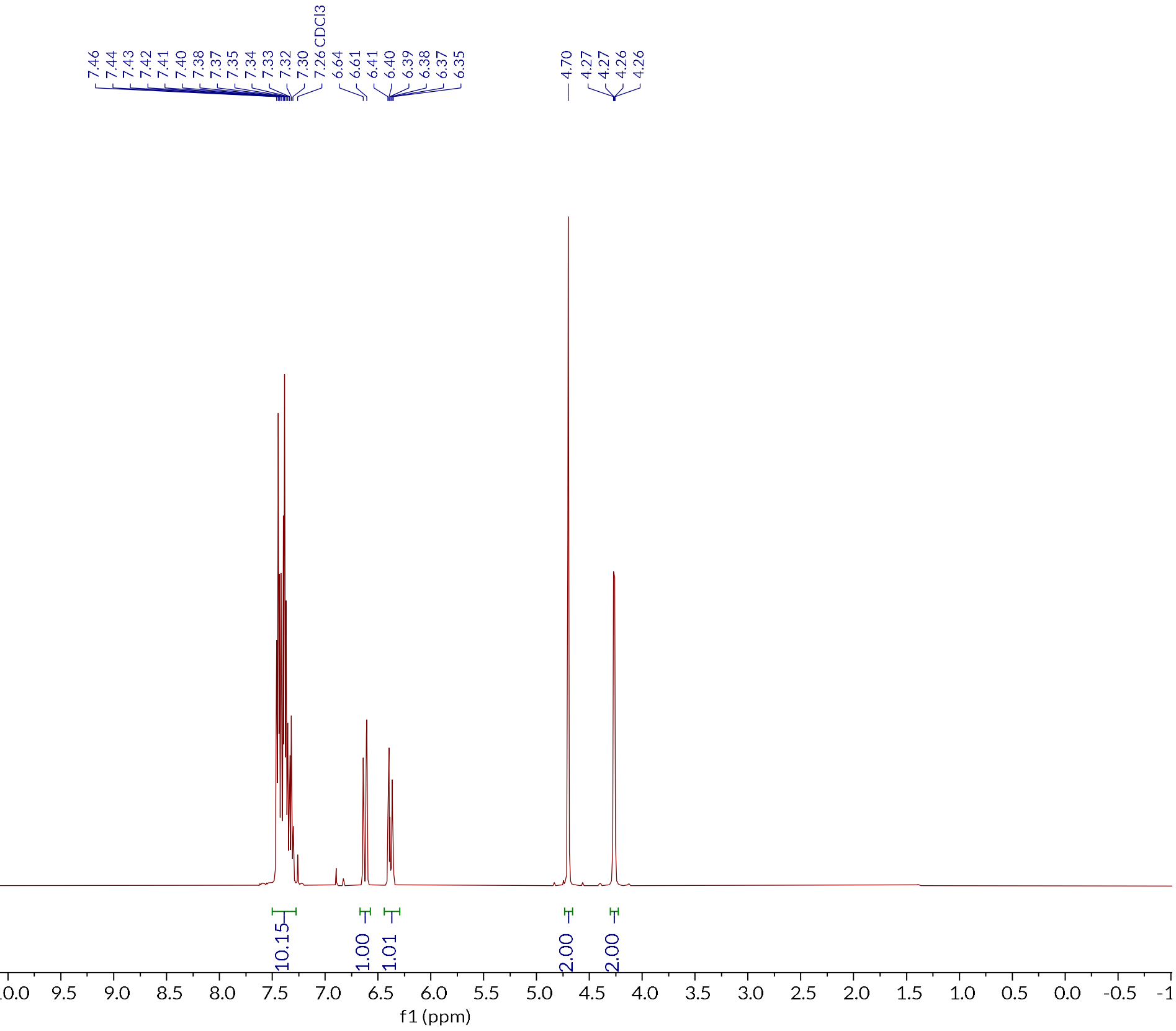

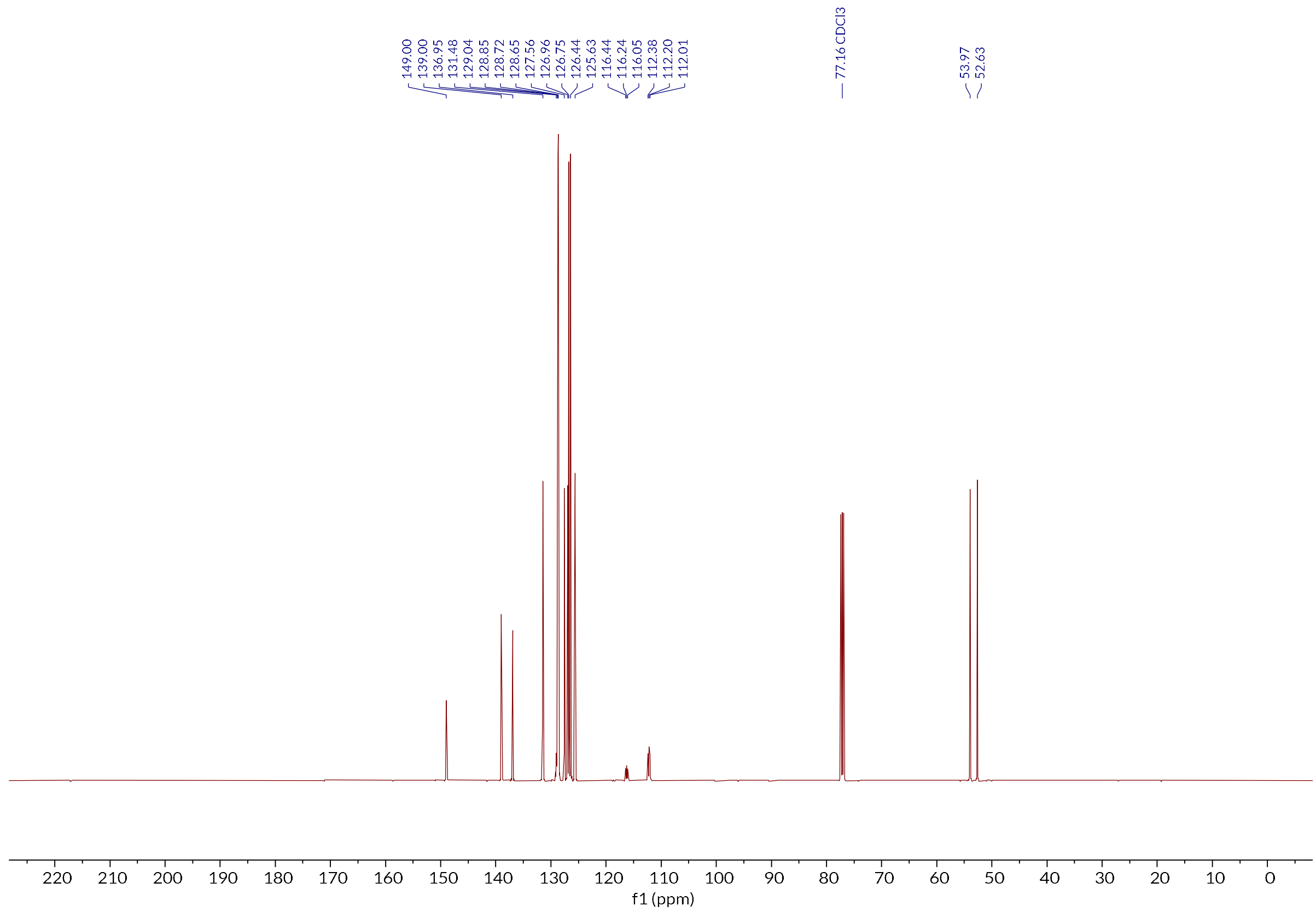

(±)-1-Benzyl-4-phenyl-1,2,3,4-tetrahydroquinoline (2aa-d_5)

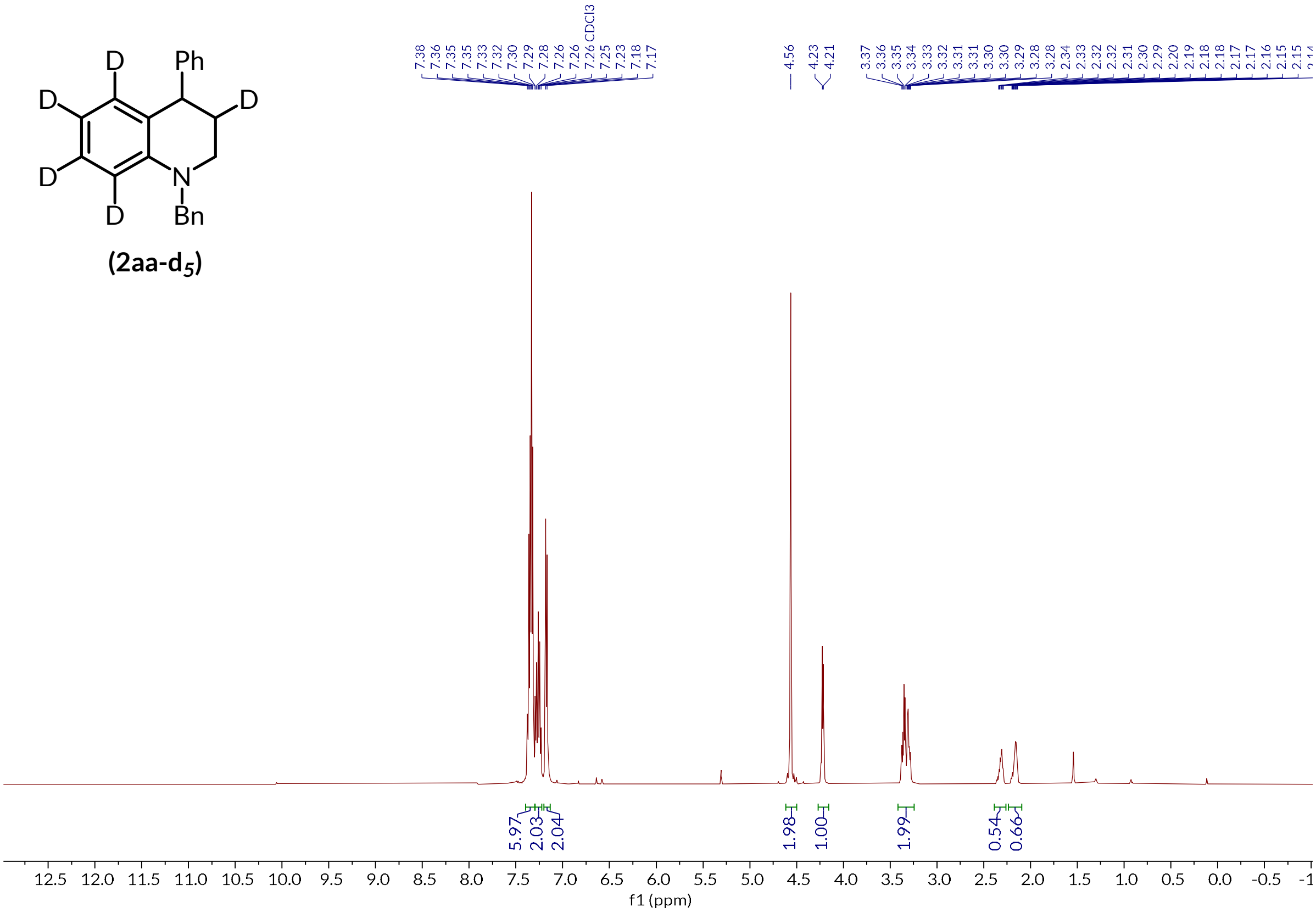

(2aa-d_5)

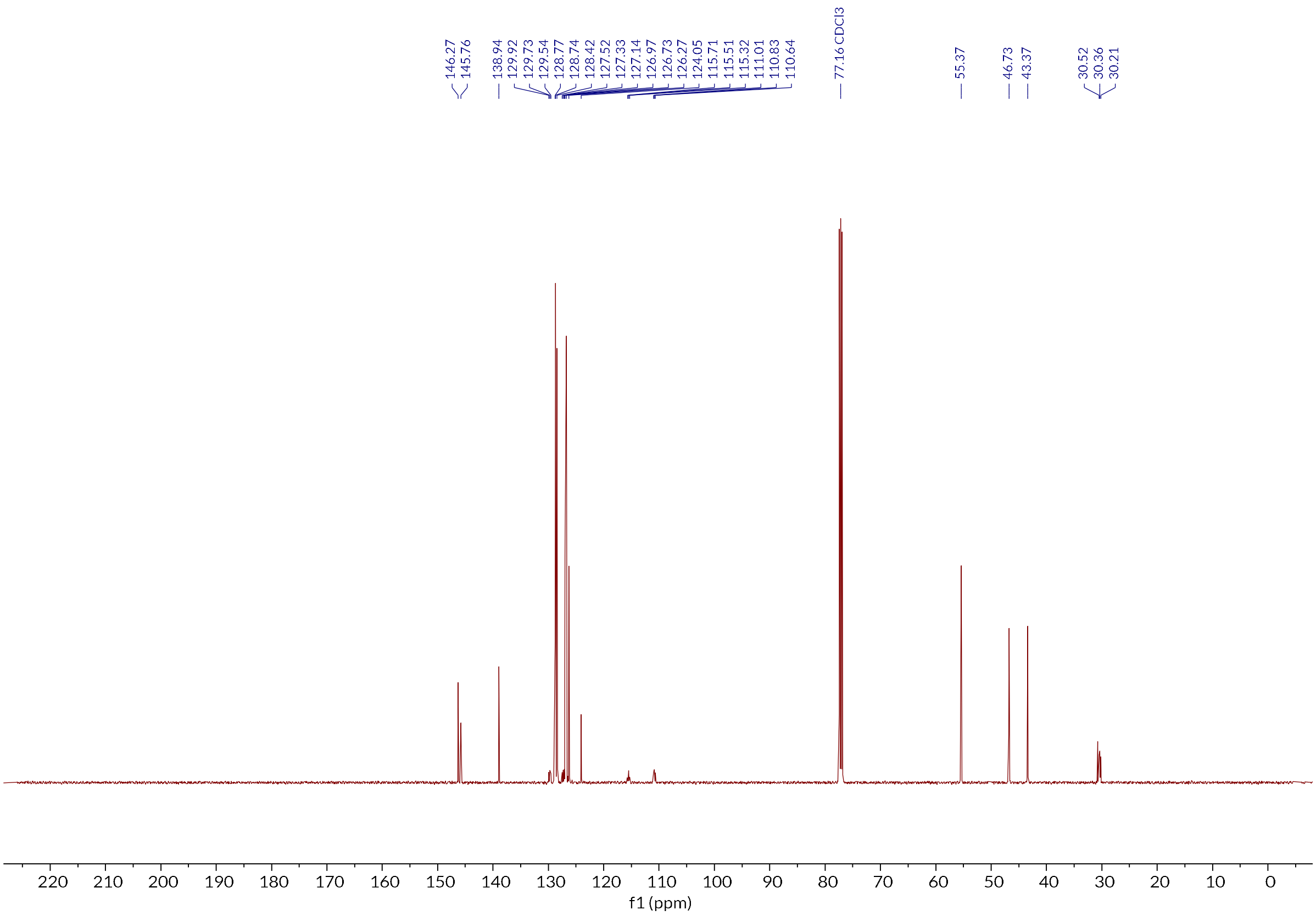

N-Cinnamyl-4-methoxy-*N*-phenylaniline (SI-35)

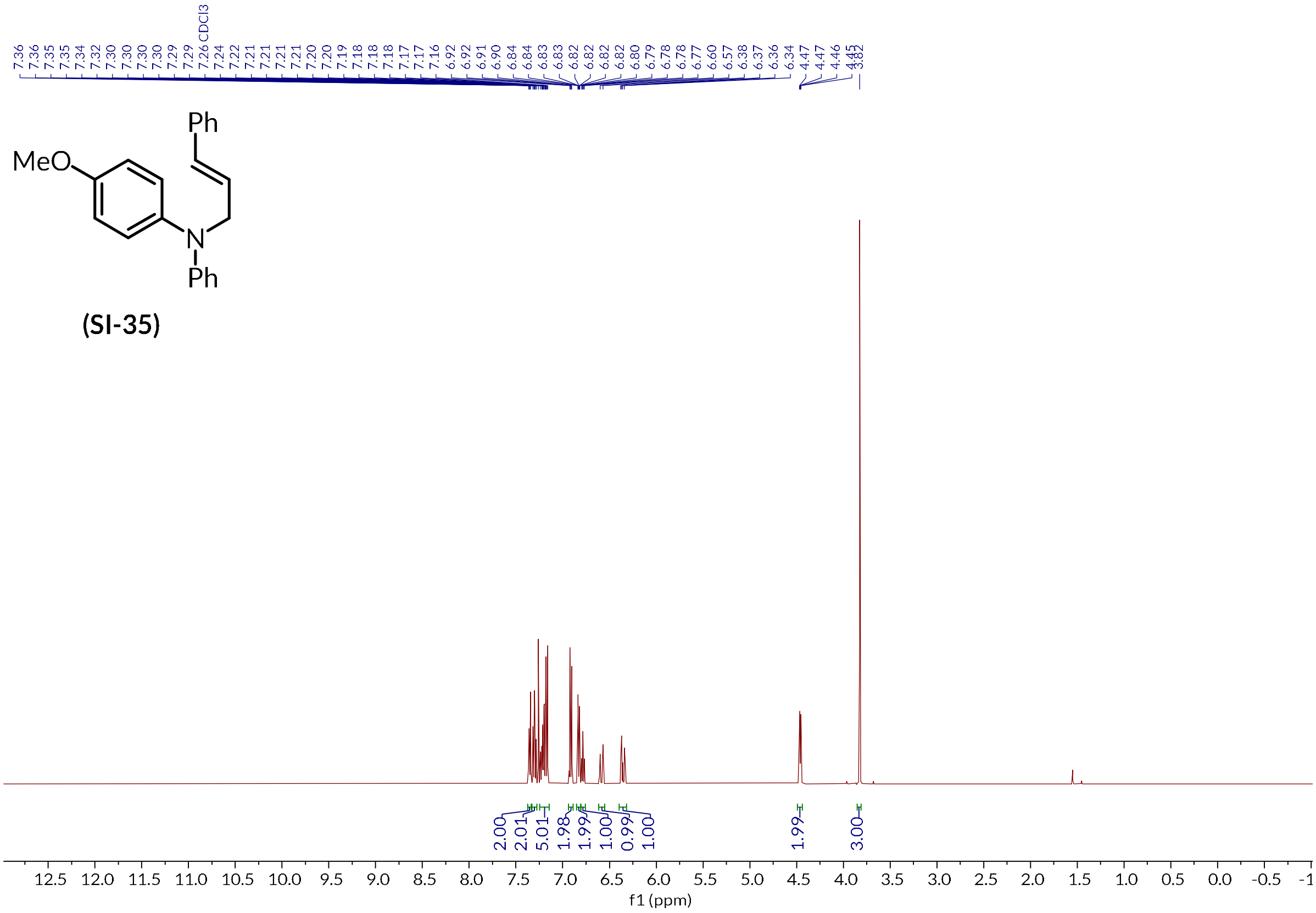

(SI-35)

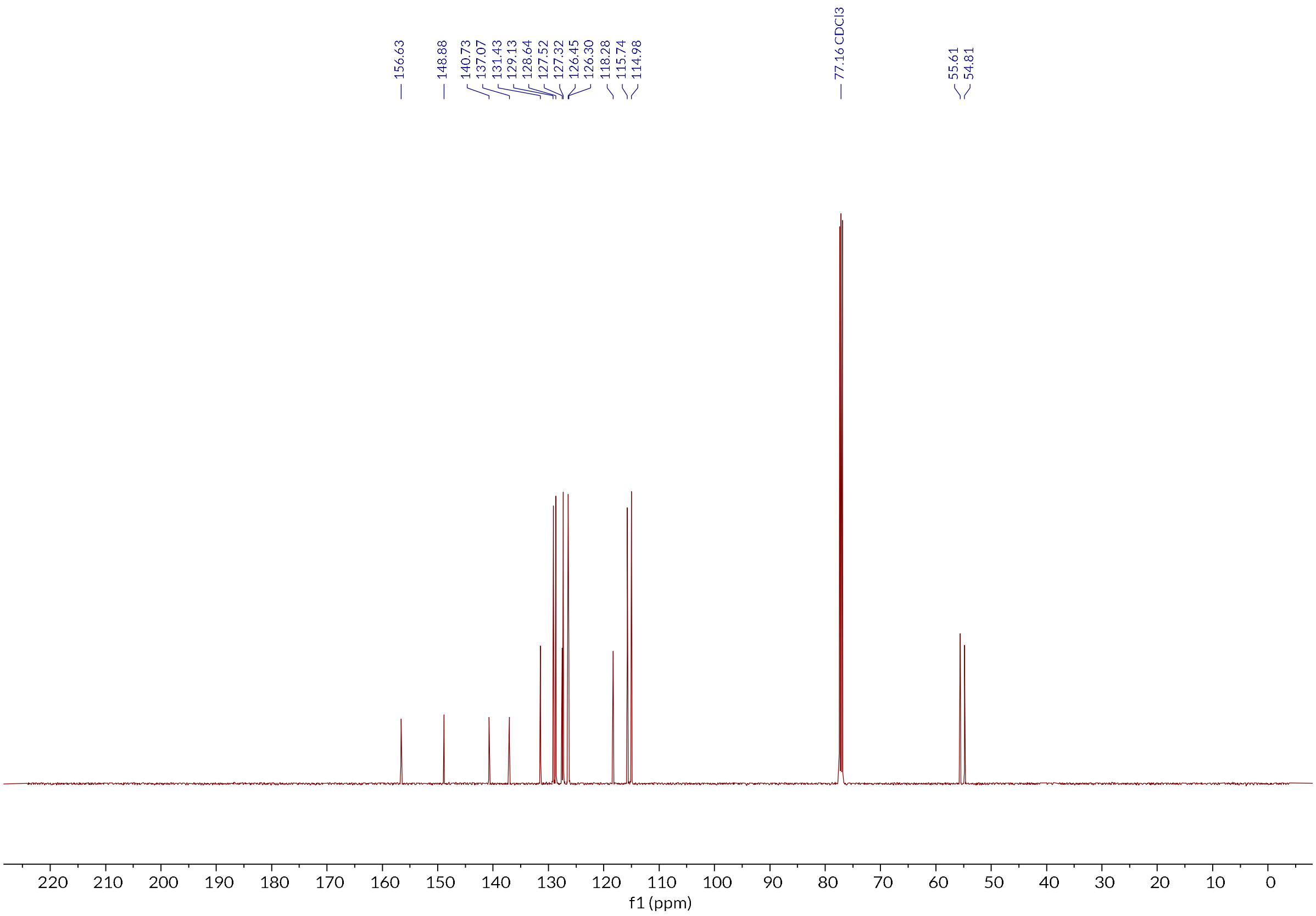

N-Cinnamyl-4-methyl-*N*-phenylaniline (SI-36)

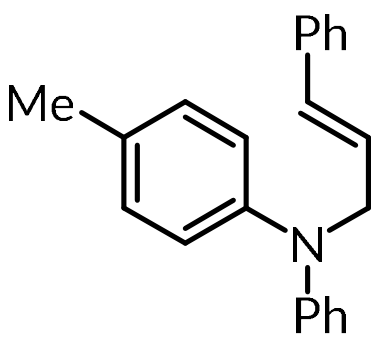

(SI-36)

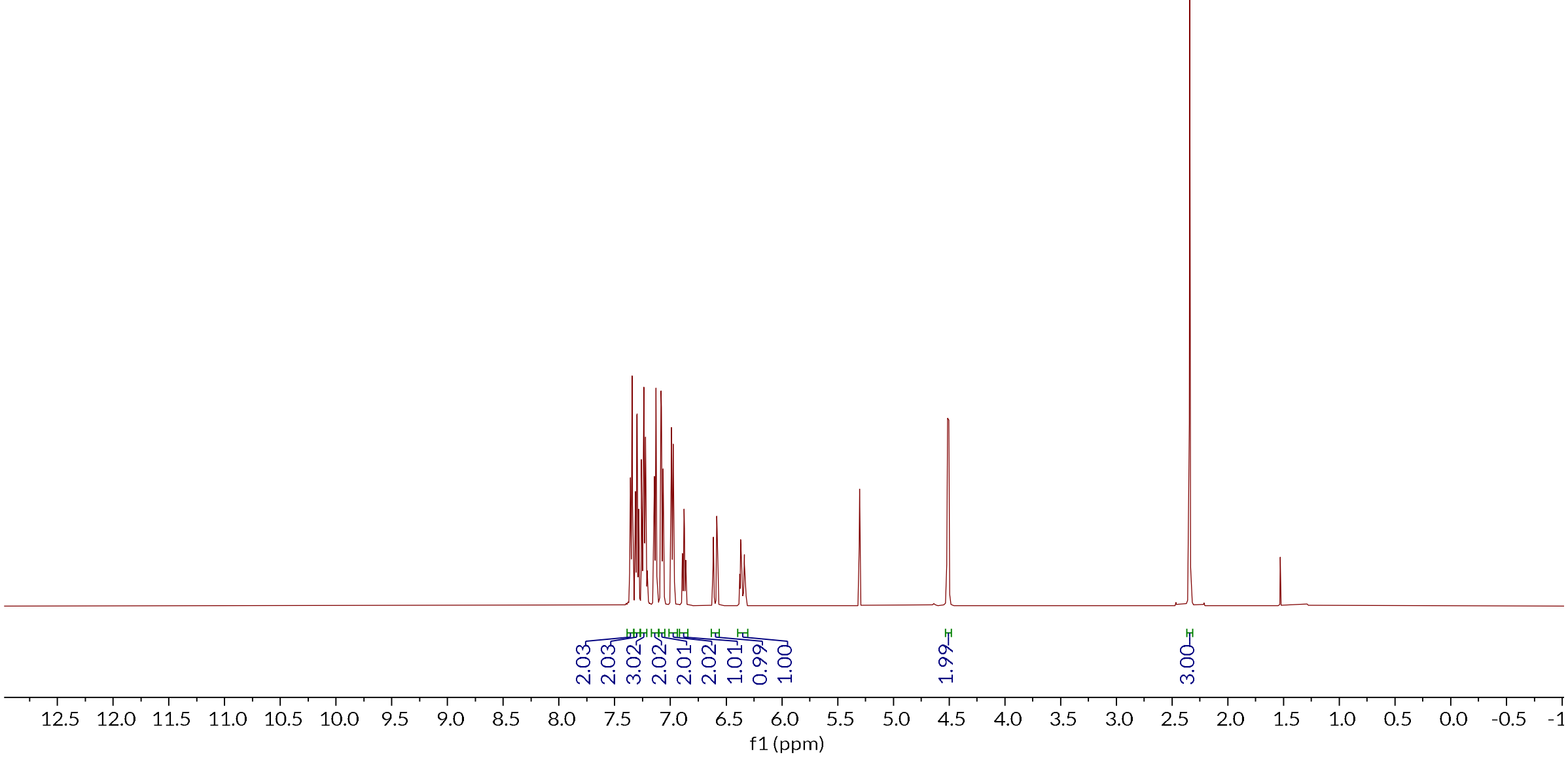

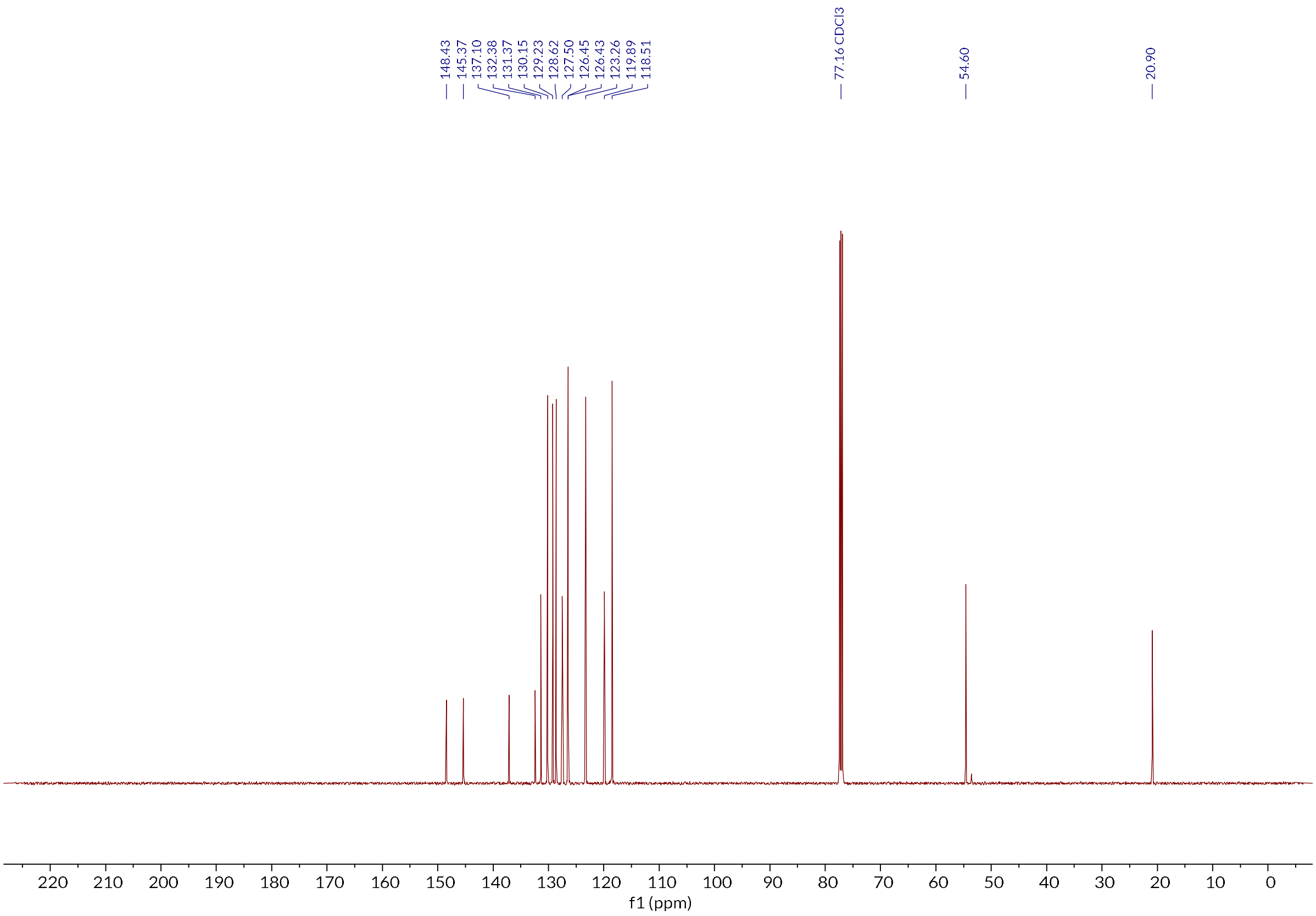

4-(*Tert*-butyl)-*N*-cinnamyl-*N*-phenylaniline (SI-37)

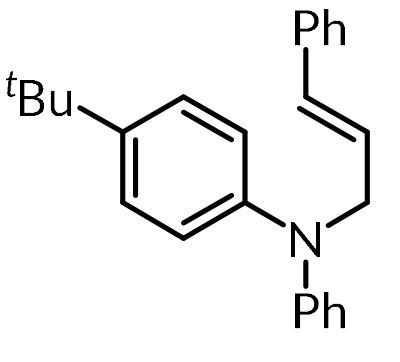

(SI-37)

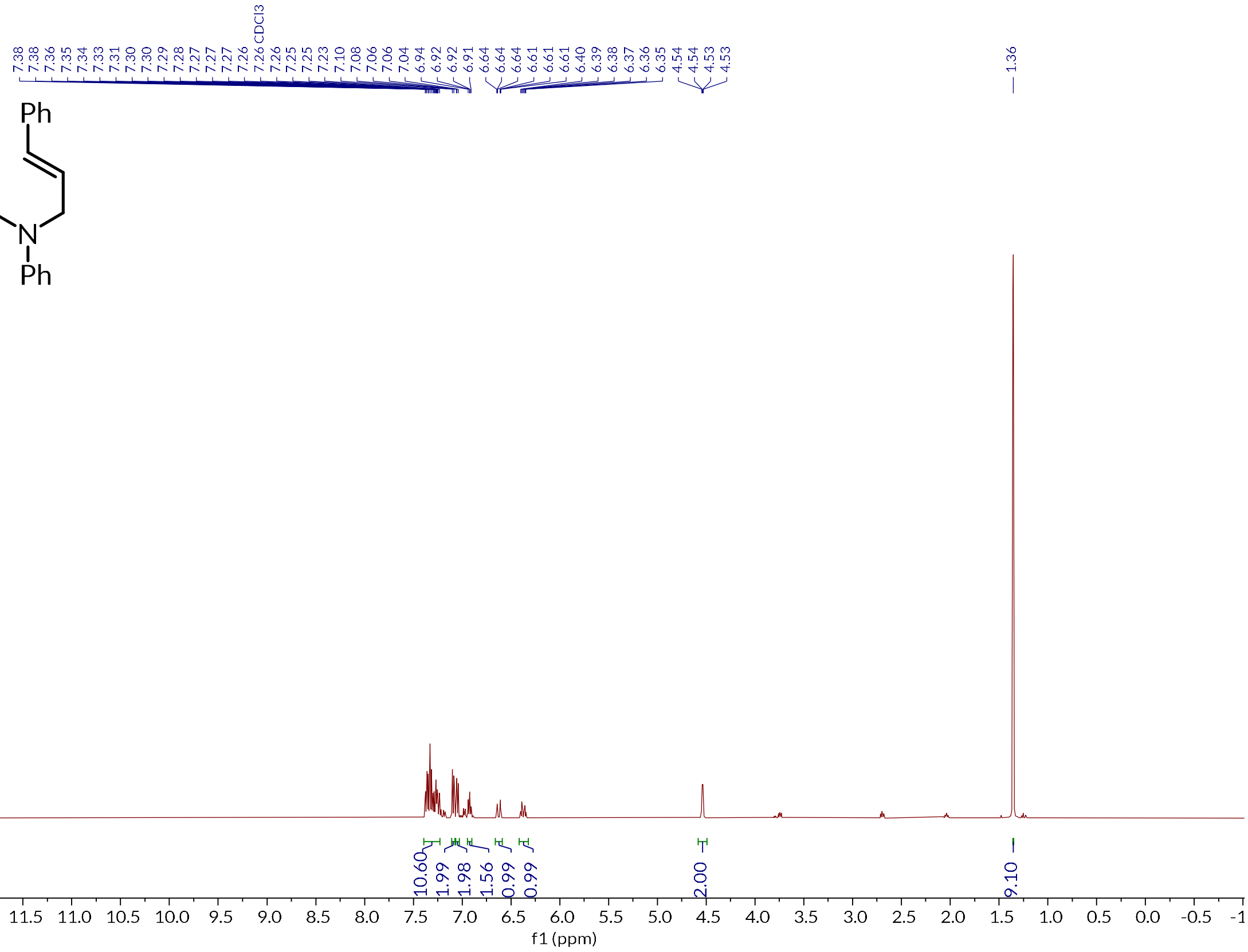

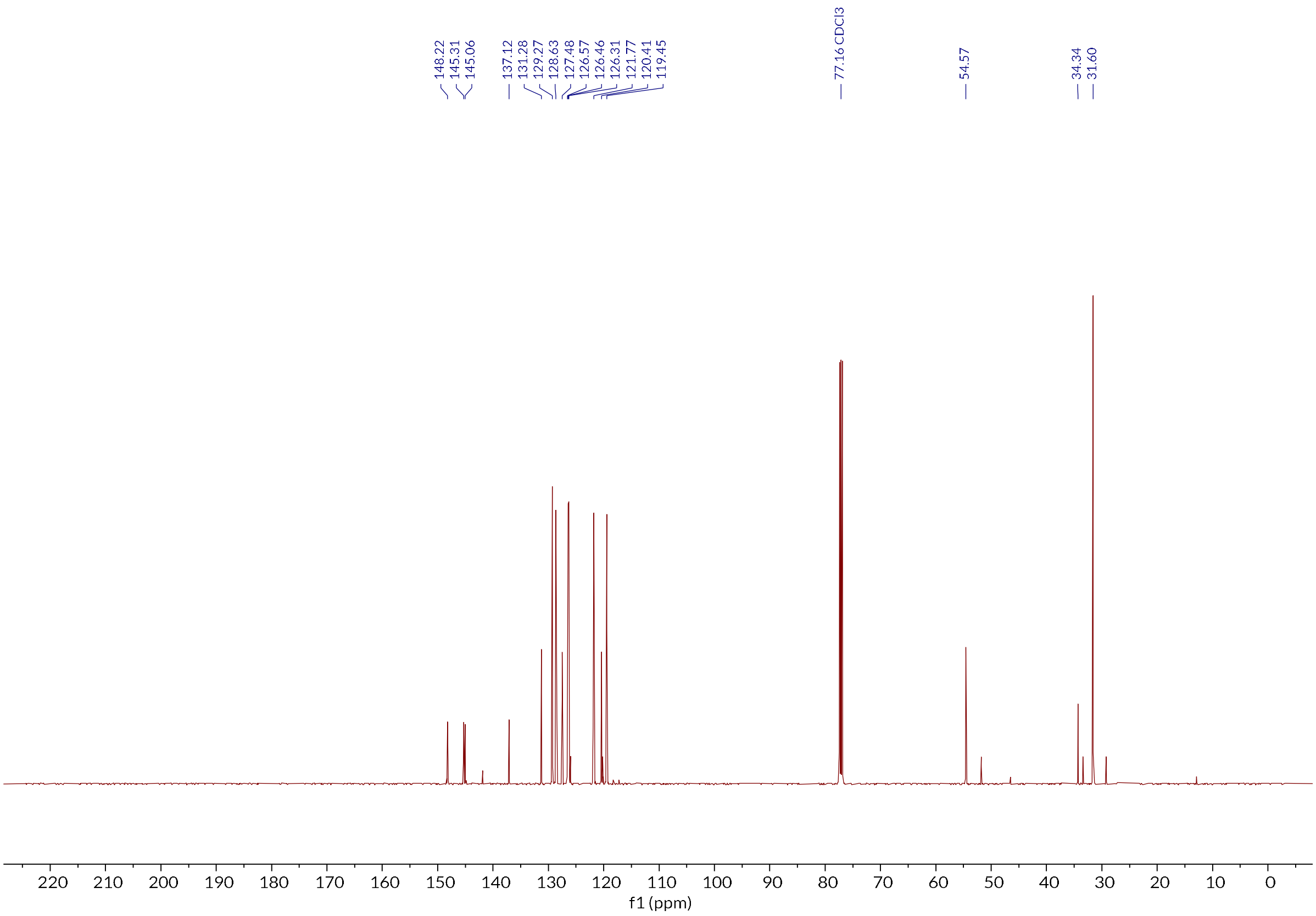

N-Cinnamyl-*N*-phenyl-[1,1'-biphenyl]-4-amine (SI-38)

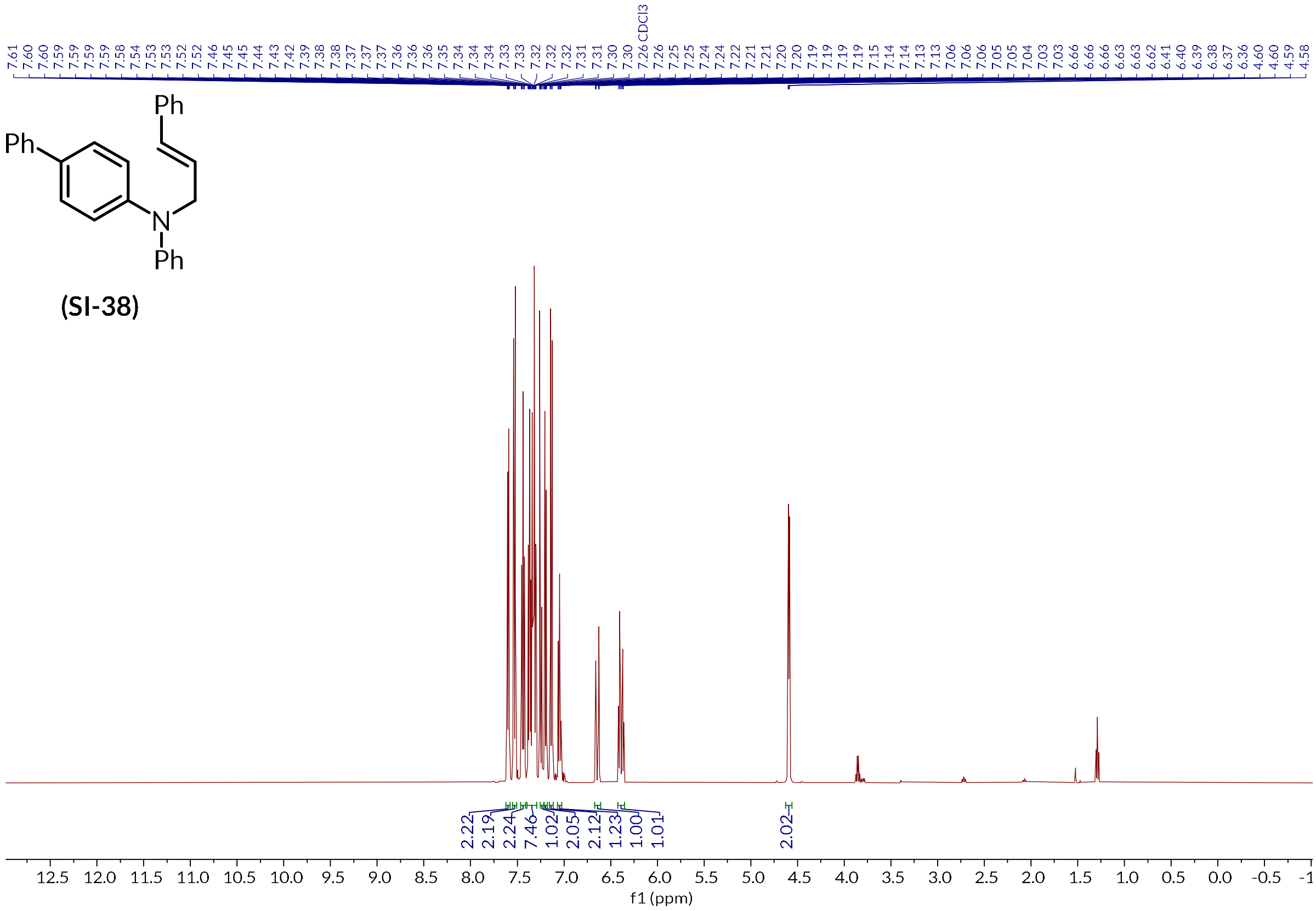

(SI-38)

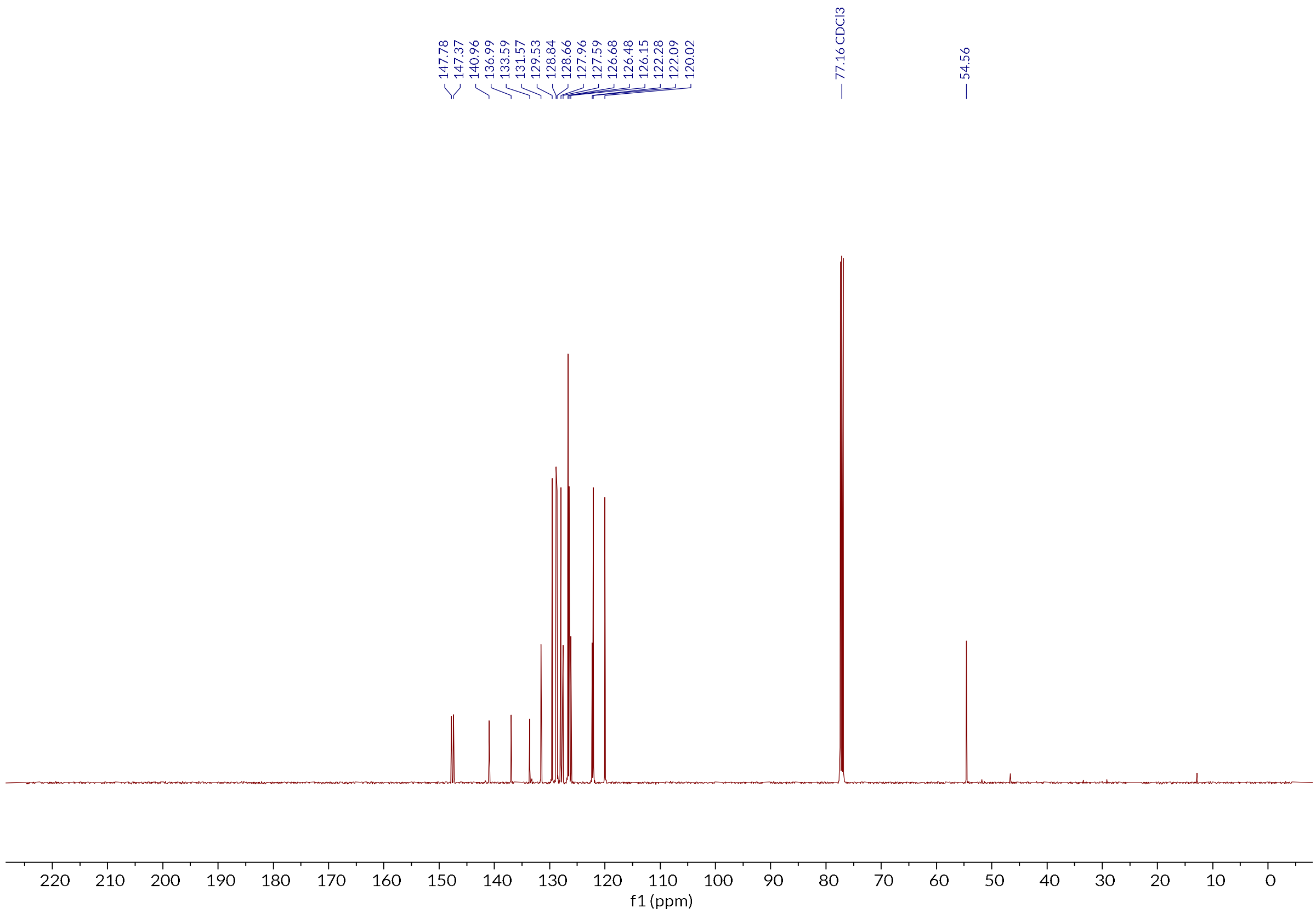

4-Chloro-*N*-cinnamyl-*N*-phenylaniline (SI-39)

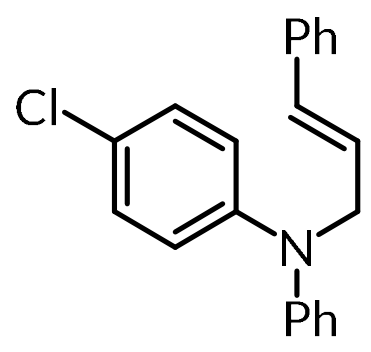

(SI-39)

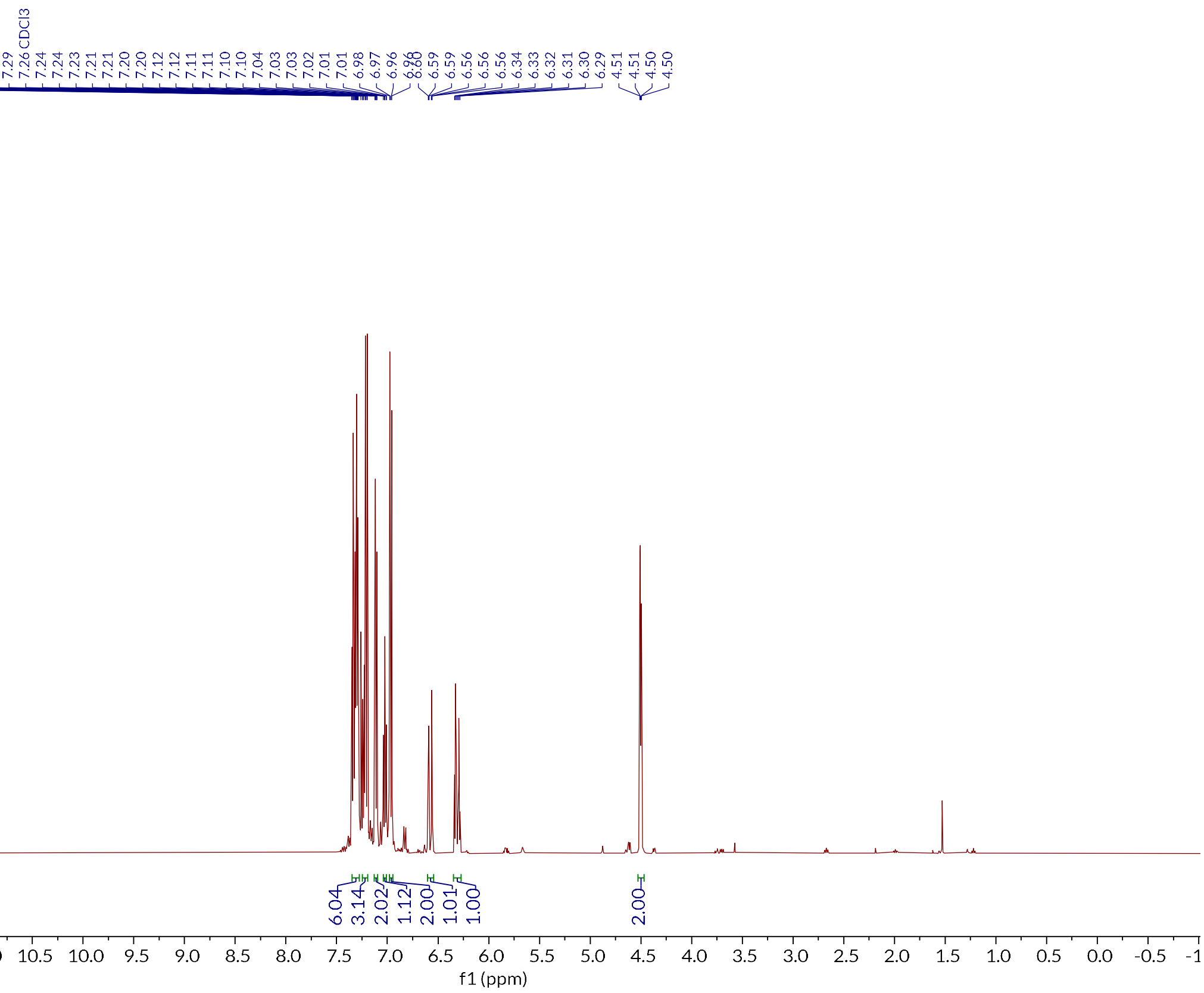

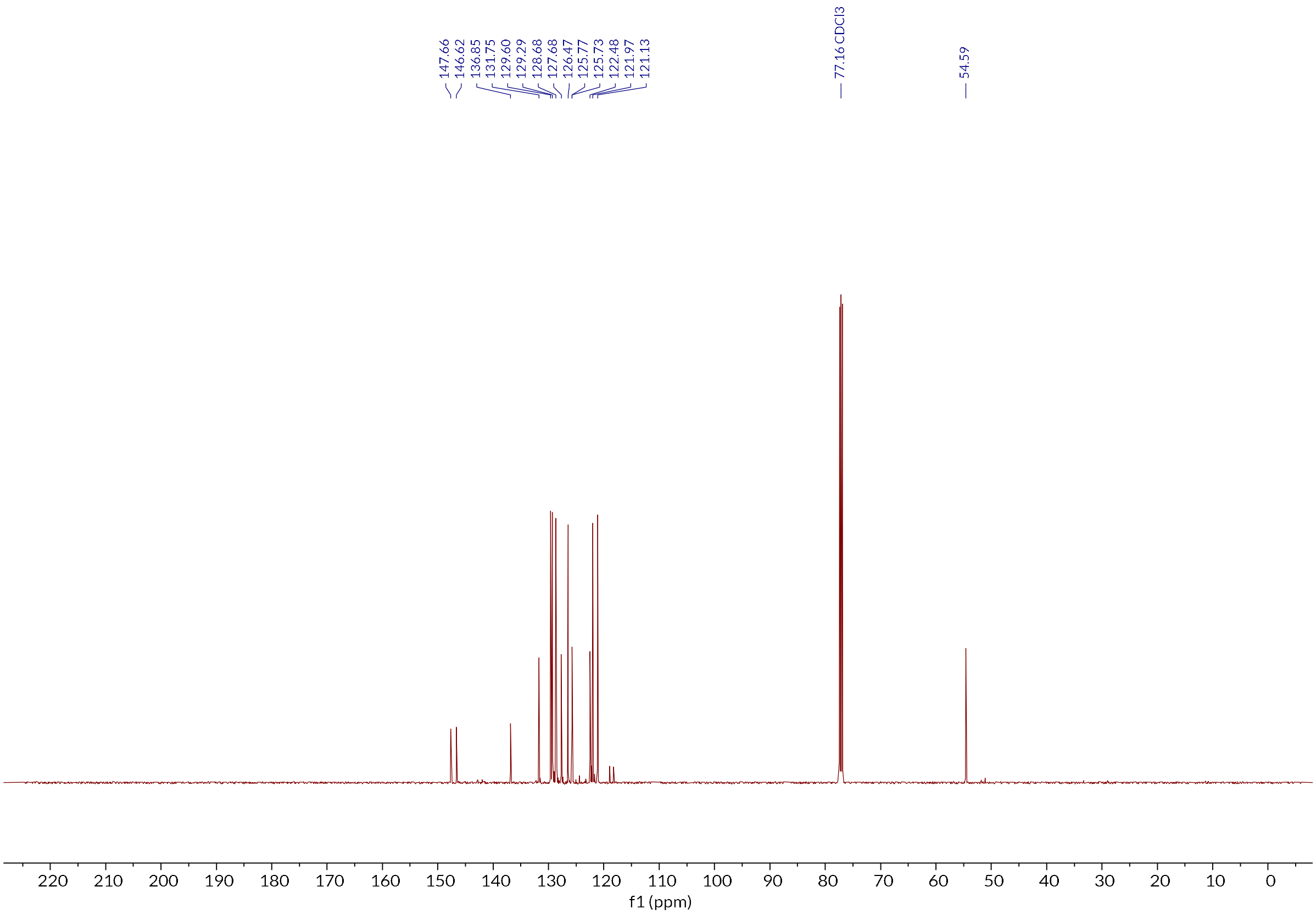

4-Bromo-*N*-cinnamyl-*N*-phenylaniline (SI-40)

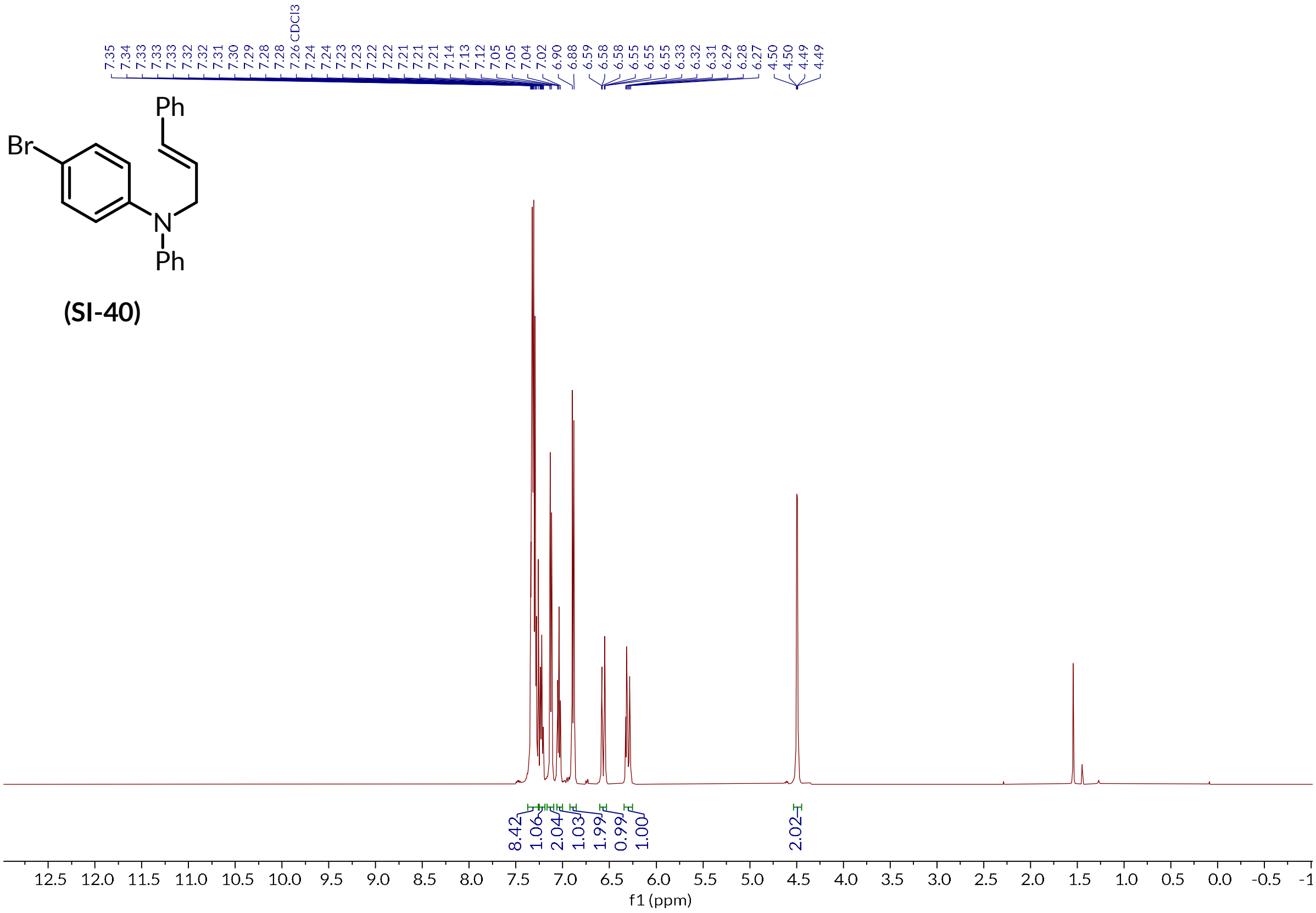

(SI-40)

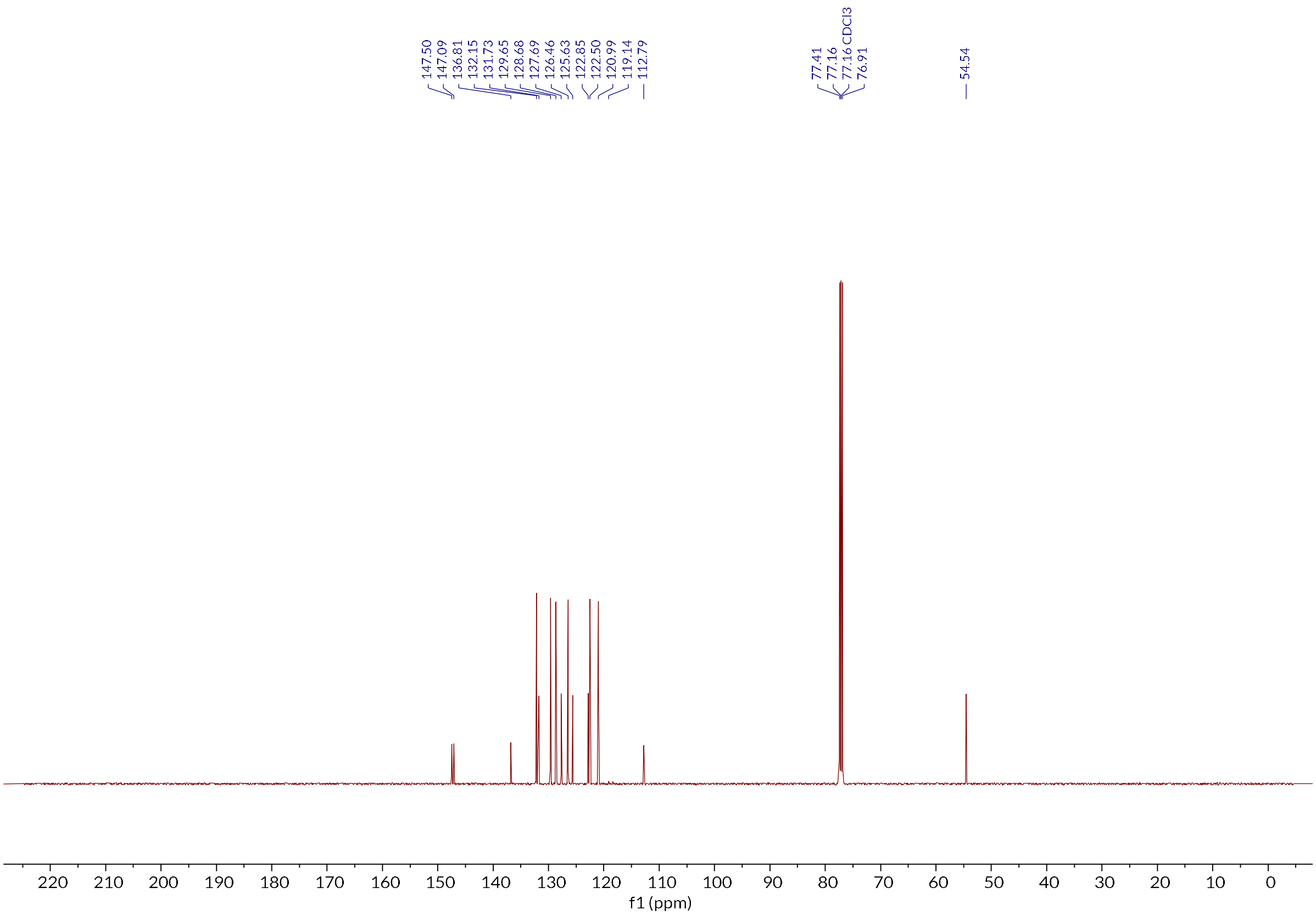

Methyl 4-(cinnamylamino)benzoate (SI-41)

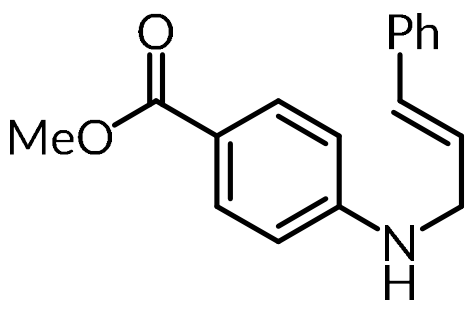

(SI-41)

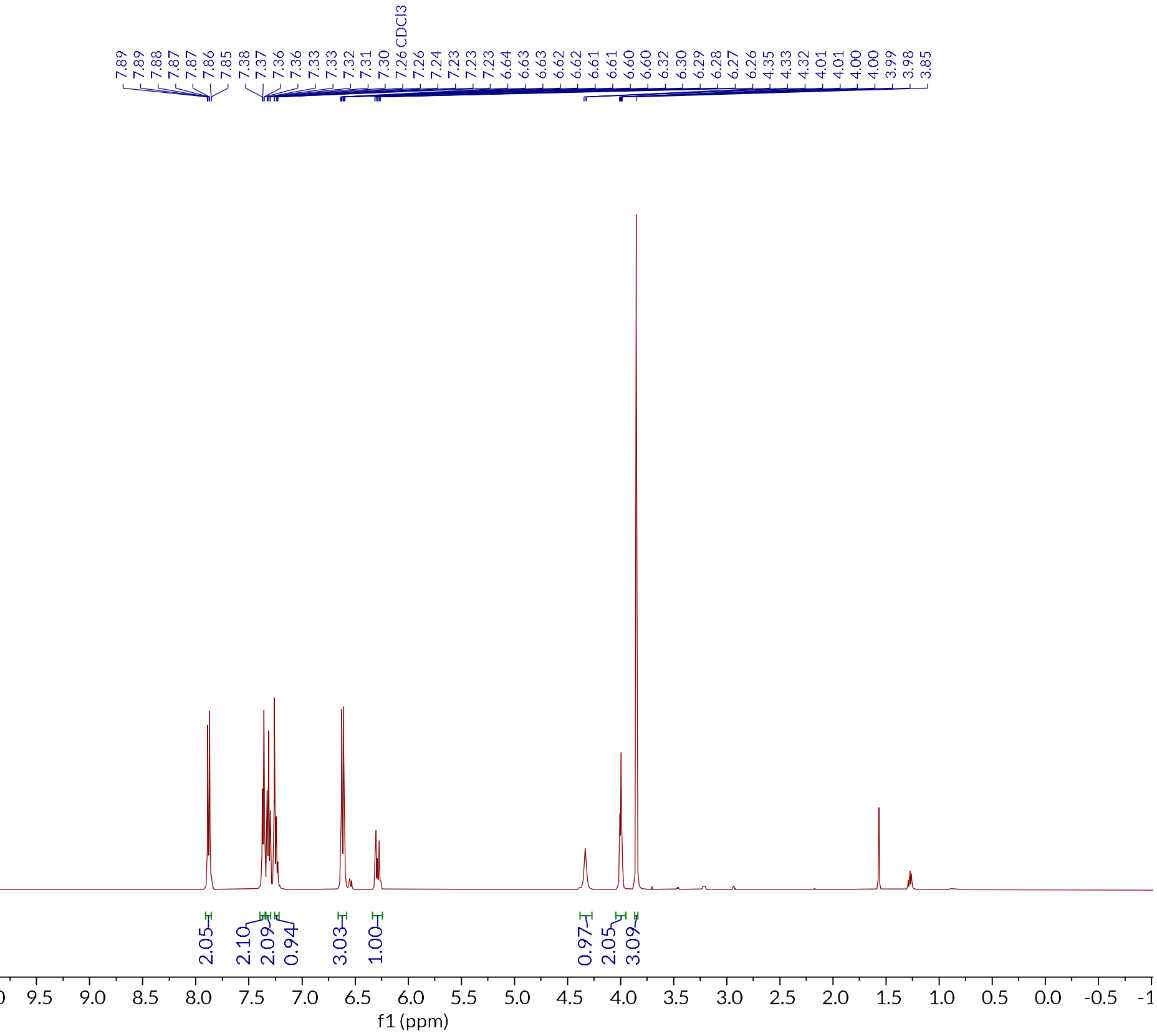

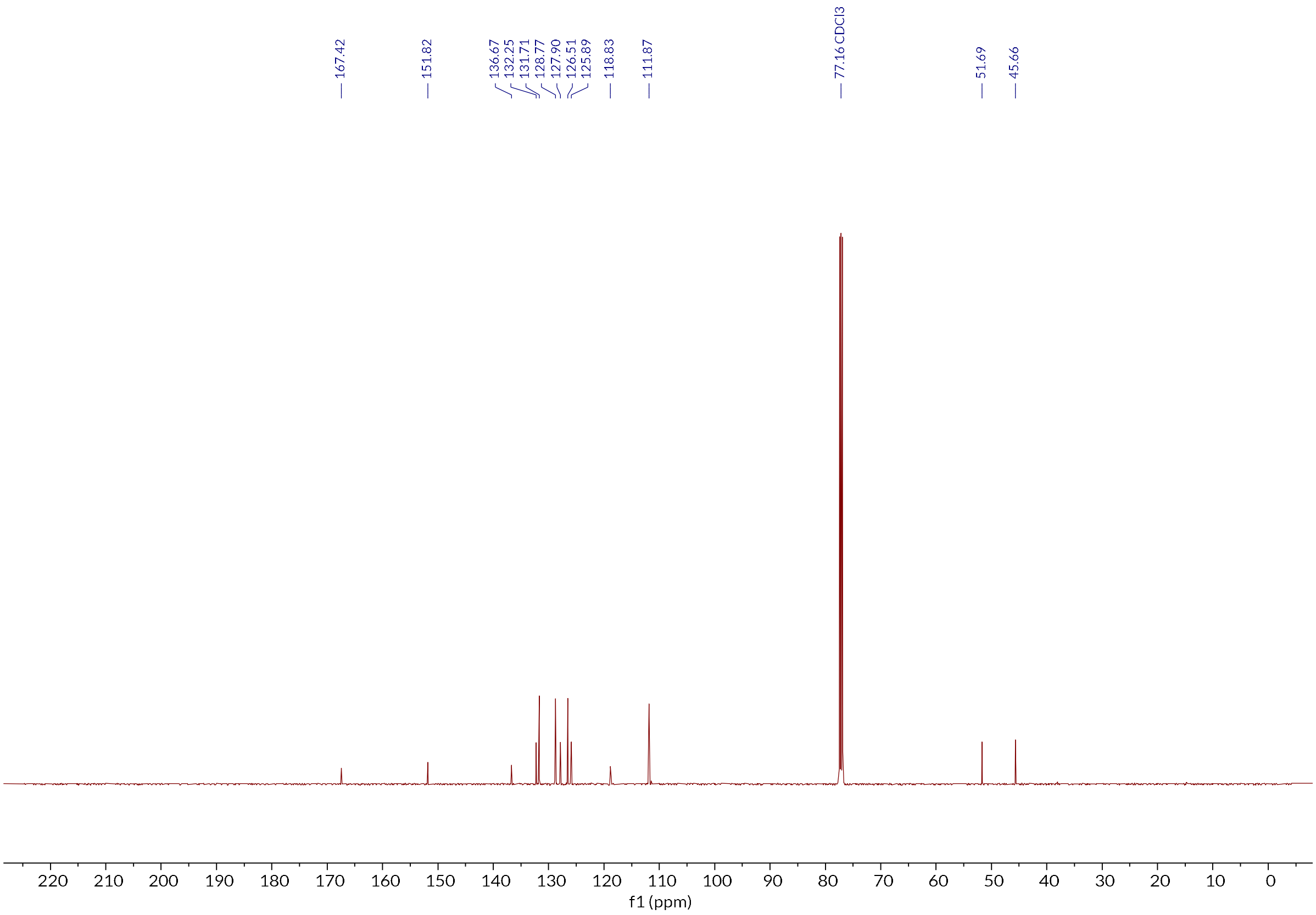

Methyl 4-(cinnamyl(phenyl)amino)benzoate (SI-42)

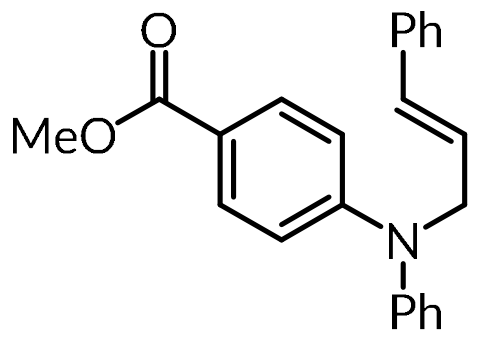

(SI-42)

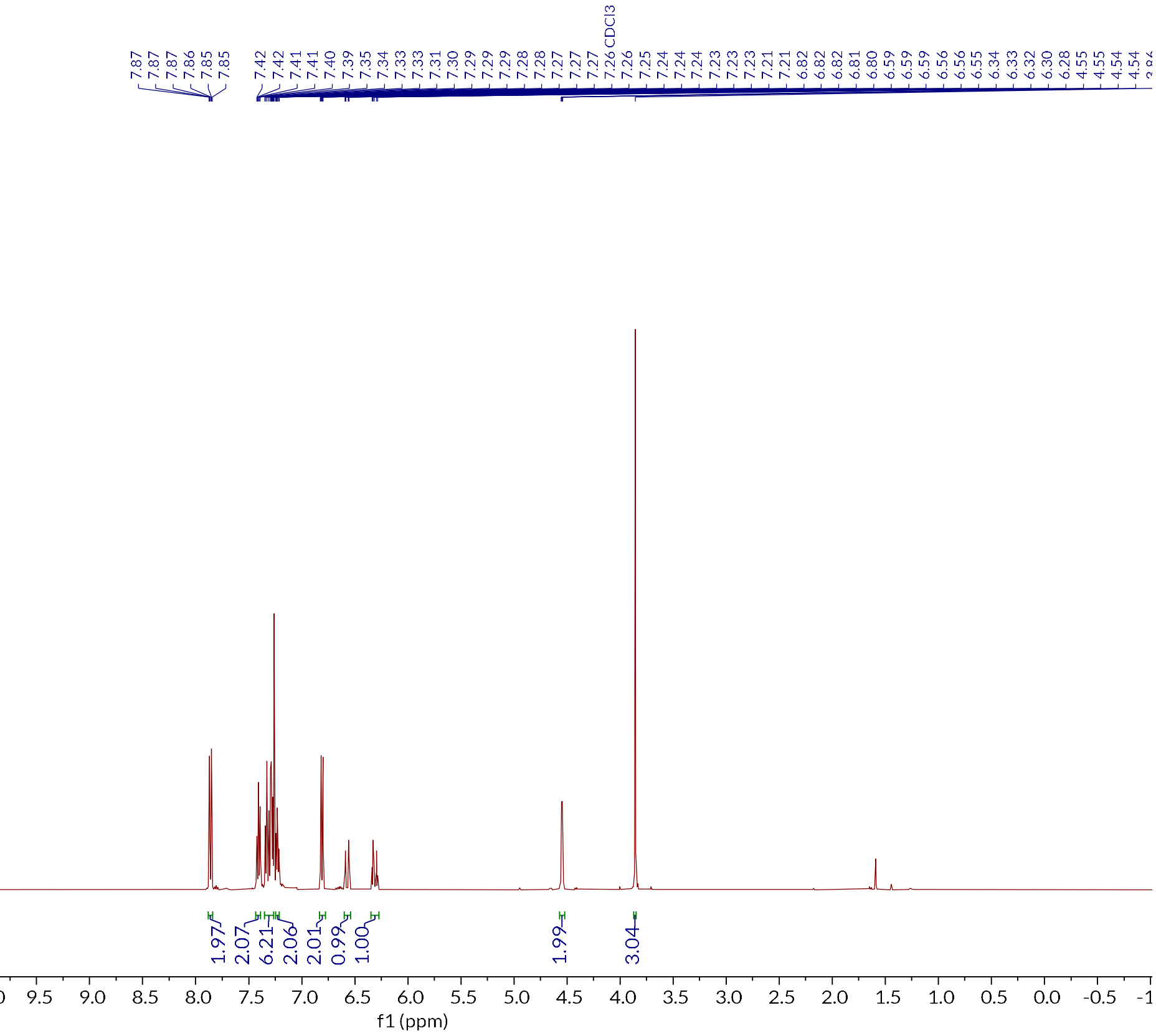

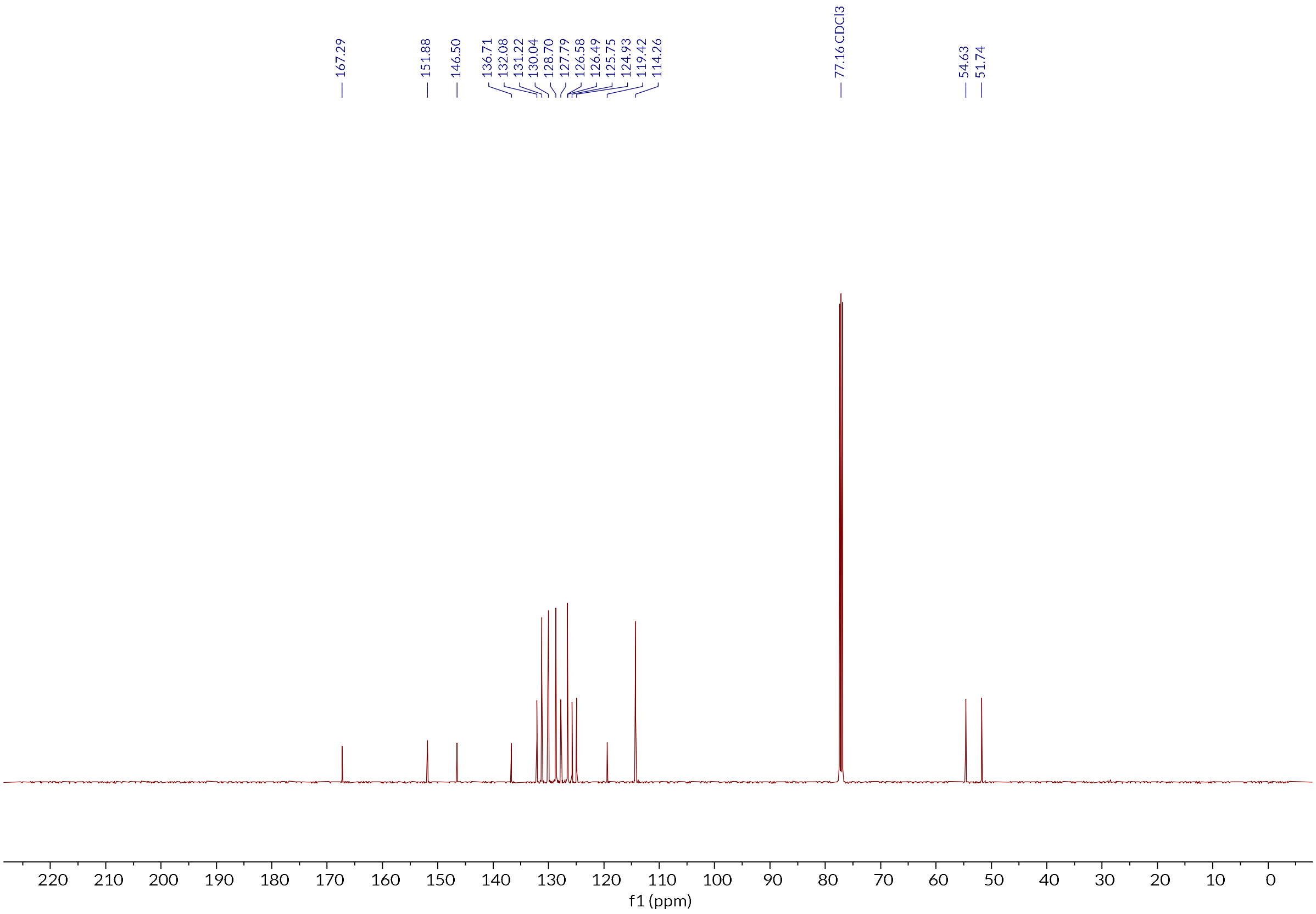

N-Cinnamyl-*N*-phenyl-4-(trifluoromethyl)aniline (SI-43)

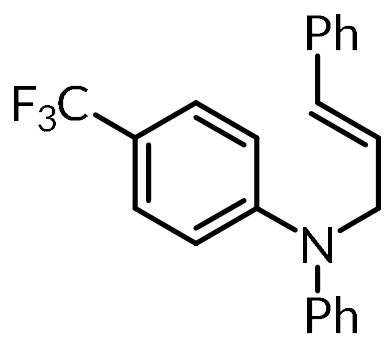

(SI-43)

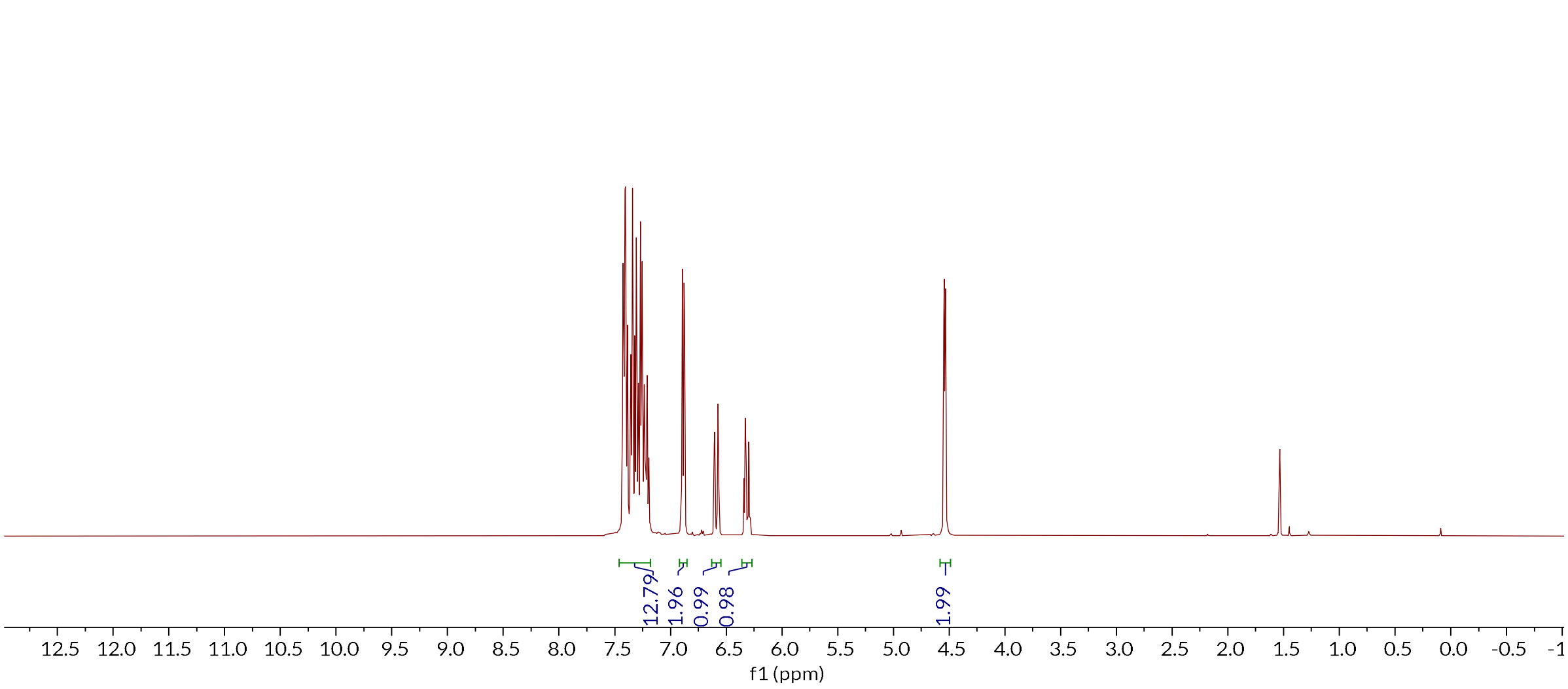

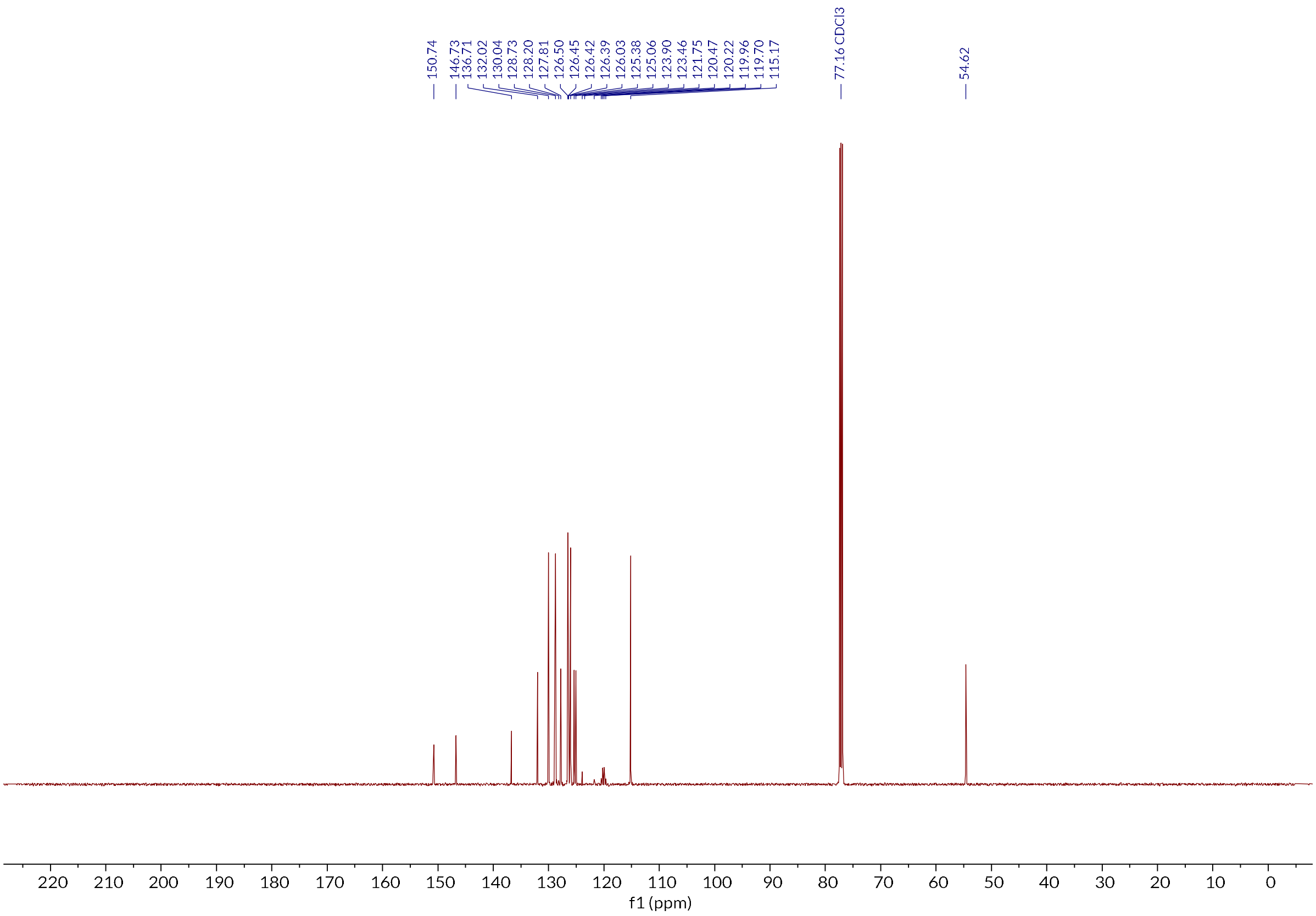

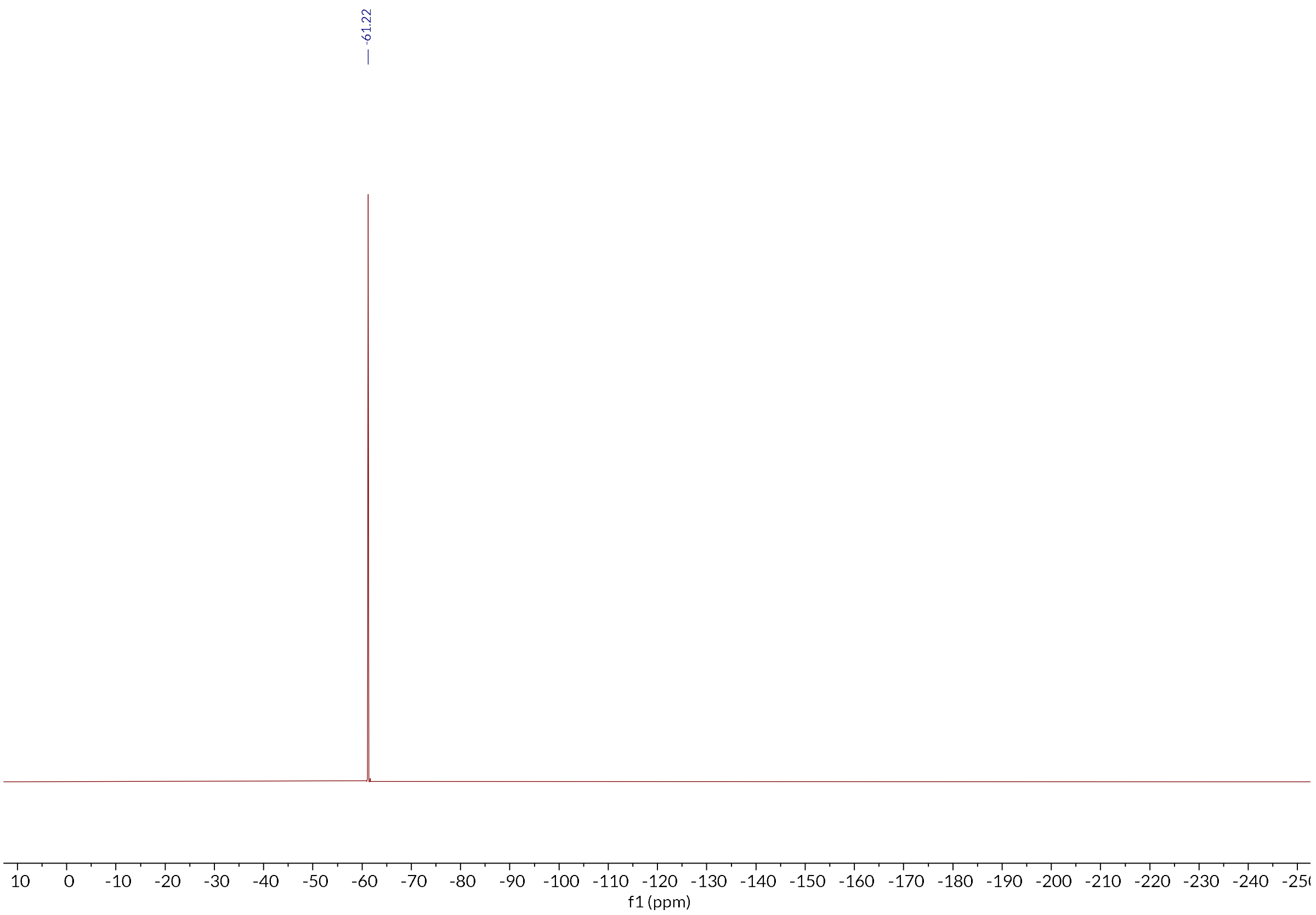
-61.22
10
0
-10
-20
-30
-40
-50
-60
-70
-80
-90
-100
-110
-120
-130
-140
-150
-160
-170
-180
-190
-200
-210
-220
-230
-240
f1 (ppm)

4-(Cinnamyl(phenyl)amino)benzonitrile (SI-44)

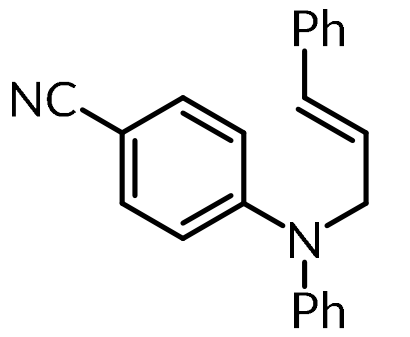

(SI-44)

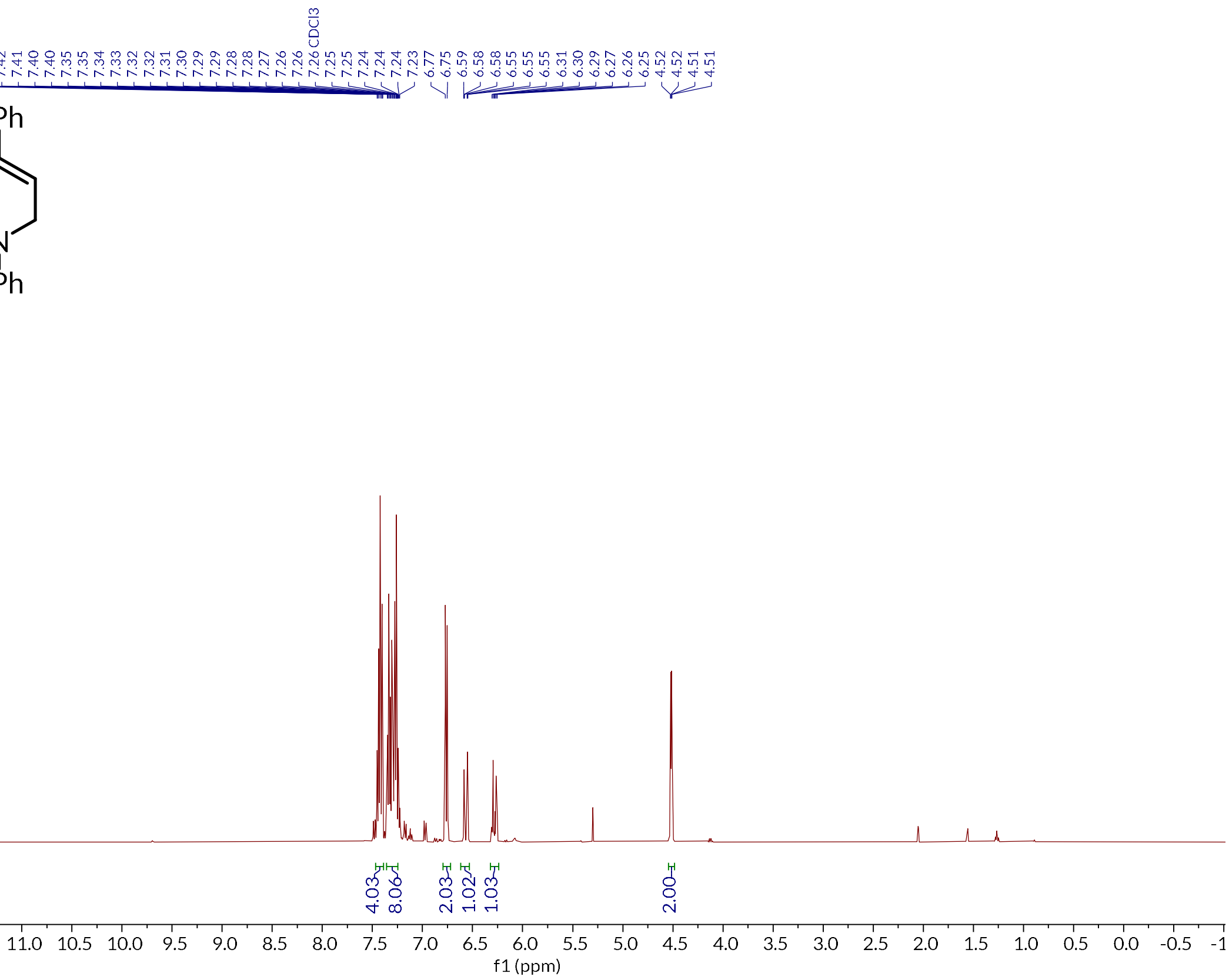

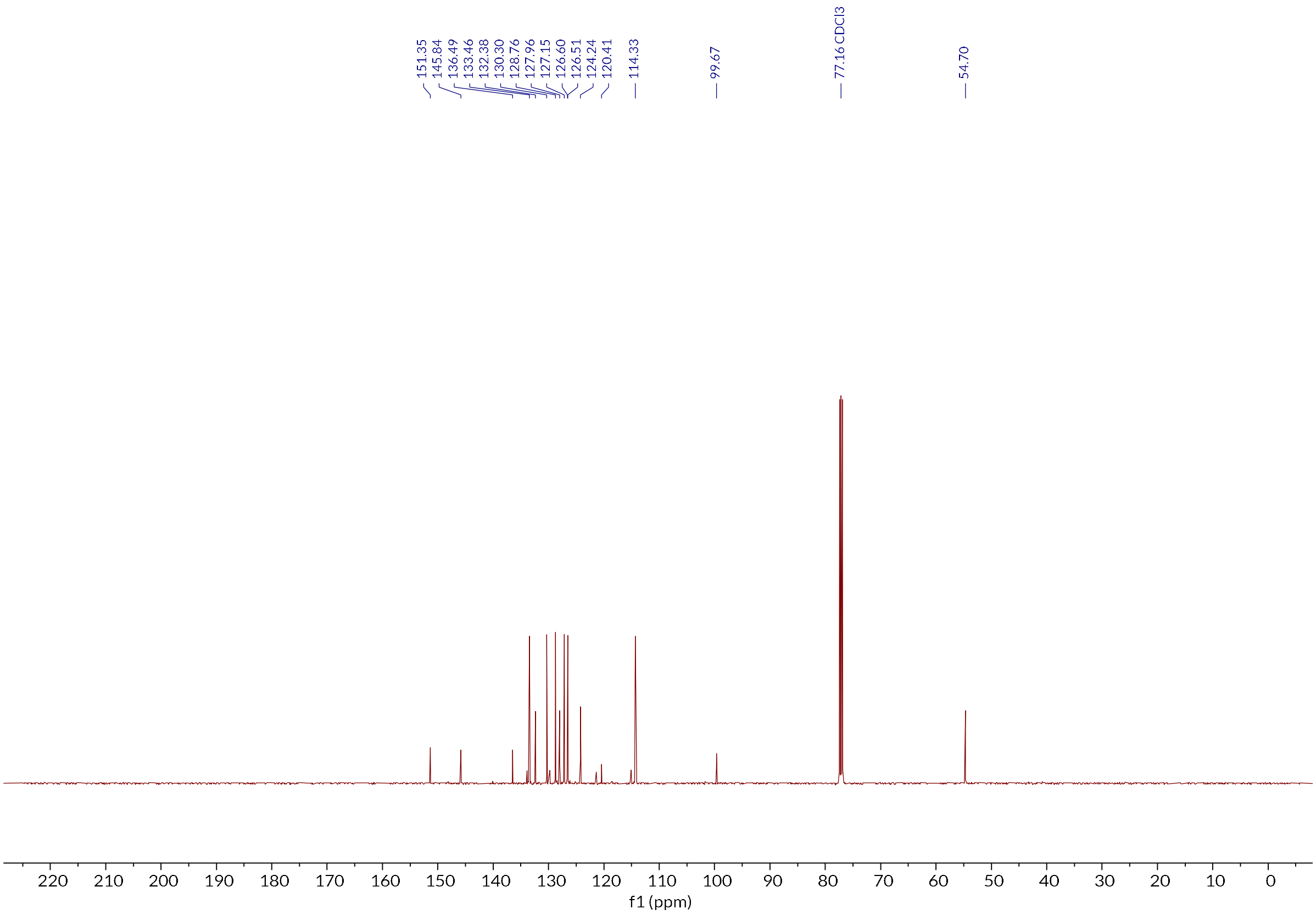

Supplement: Supplementary file 1 [file SC-017-D6SC05922H-s001.pdf]
